# Identification of Hammerhead Ribozymes in All Domains of Life Reveals Novel Structural Variations

**DOI:** 10.1371/journal.pcbi.1002031

**Published:** 2011-05-05

**Authors:** Jonathan Perreault, Zasha Weinberg, Adam Roth, Olivia Popescu, Pascal Chartrand, Gerardo Ferbeyre, Ronald R. Breaker

**Affiliations:** 1 Department of Molecular, Cellular and Developmental Biology, Yale University, New Haven, Connecticut, United States of America; 2 Howard Hughes Medical Institute, Yale University, New Haven, Connecticut, United States of America; 3 Department of Molecular Biophysics and Biochemistry, Yale University, New Haven, Connecticut, United States of America; 4 Department of Biochemistry, Université de Montréal, Montréal, Québec, Canada; University of British Columbia, Canada

## Abstract

Hammerhead ribozymes are small self-cleaving RNAs that promote strand scission by internal phosphoester transfer. Comparative sequence analysis was used to identify numerous additional representatives of this ribozyme class than were previously known, including the first representatives in fungi and archaea. Moreover, we have uncovered the first natural examples of “type II” hammerheads, and our findings reveal that this permuted form occurs in bacteria as frequently as type I and III architectures. We also identified a commonly occurring pseudoknot that forms a tertiary interaction critical for high-speed ribozyme activity. Genomic contexts of many hammerhead ribozymes indicate that they perform biological functions different from their known role in generating unit-length RNA transcripts of multimeric viroid and satellite virus genomes. In rare instances, nucleotide variation occurs at positions within the catalytic core that are otherwise strictly conserved, suggesting that core mutations are occasionally tolerated or preferred.

## Author Summary

The expanding diversity of noncoding RNA discoveries is revealing a broader spectrum of roles RNA plays in cellular signaling and in biochemical functions. These discoveries in part are being facilitated by the expanding collection of genomic sequence data and by computational methods used to search for novel RNAs. In addition to searching for new classes of structured RNAs, these methods can be used to reevaluate the distributions of long-known RNAs. We have used a bioinformatics search strategy to identify many novel variants of hammerhead self-cleaving ribozymes, including examples from species in all three domains of life. New architectural features and novel catalytic core variants were identified, and the genomic locations of some hammerhead ribozymes suggest important biological functions. This ribozyme class promotes RNA cleavage by an internal phosphoester transfer reaction by using a small catalytic core. The simple sequence and structural architecture coupled with the general utility of RNA strand scission may explain its great abundance in many organisms.

## Introduction

Hammerhead ribozymes [1] represent one of five distinct structural classes of natural self-cleaving RNAs identified to date [2]. The first hammerheads were discovered in viroids and plant satellite RNA viruses where they process RNA transcripts containing multimeric genomes to yield individual genomic RNAs [1], [3], [4]. Representatives of this ribozyme class have been studied extensively for the past 25 years because their small size and fundamental catalytic activity make them excellent models for RNA structure-function research [5].

**Figure 1:**
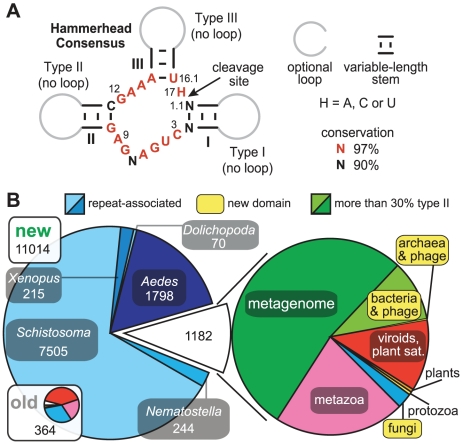
Consensus secondary structure model for hammerhead ribozymes and the expanded phylogenetic distribution of this self-cleaving ribozyme class. (A) Consensus sequence and secondary structure of the catalytic core of hammerhead ribozymes. Annotations are as described previously [61]. (B) Distribution of hammerhead sequences among all domains of life. The chart entitled “old” (inset) represents all previously known non-identical hammerhead ribozyme sequences [13]–[16], [24], [25], [62]–[64]. The “new” chart includes previously known examples as well as all additional non-identical hammerhead ribozymes found in this study. Chart sizes are scaled based on the number of unique sequences as indicated. The chart on the right reflects the distribution of a subset of hammerhead ribozymes (not to scale with charts to the left). Clades that for the first time have been found to carry hammerhead motifs are boxed in yellow. Note that a large number of the hammerheads that we consider new in this graphic have been recently published [15], [16], [43], [44] but the sequences of many were not available at the time of writing.

Although a minimal three-stem junction constitutes the catalytic core of the ribozyme (Figure 1A), additional sequence and structural elements form an extended hammerhead motif [6], [7] that yields robust RNA cleavage activity under physiological concentrations of Mg^2+^. Specifically, tertiary interactions form between the loop of stem II and either an internal or terminal loop in stem I that increase activity of the core by several orders of magnitude under low magnesium conditions. Identification of this tertiary substructure in high-speed hammerhead ribozymes [5], [8] resolved a long-standing paradox between biochemical data and atomic-resolution structures of minimal hammerhead ribozymes [5].

Several searches for new examples of hammerhead ribozymes have been performed previously [9]–[11] by taking advantage of the wealth of knowledge derived from mutational and biochemical analyses of various hammerhead ribozymes. By carefully establishing descriptors of the minimum functional consensus motif, dozens of new hammerhead representatives have been found in the parasitic worm *Schistosoma mansoni* [12], *Arabidopsis* thaliana [13], in mouse [14] and very recently in bacteria and human [15], [16]. A similar bioinformatics search for RNA structures homologous to hepatitis delta virus (HDV) ribozymes [17] revealed that representatives of this self-cleaving ribozyme class are far more widely distributed in many organisms. Moreover, among numerous noncoding RNA candidates revealed by our recent bioinformatics efforts was a distinct architectural variant of hammerhead ribozymes (see below). Given these observations, we speculated that far more hammerhead ribozymes may exist in the rapidly growing collection of genomic sequence data.

Using a combination of homology searches we found thousands of new hammerhead ribozyme sequences in all domains of life. These ribozymes are observed in the eubacterial and archaeal domains, as well as in fungi and humans. Moreover, many of the newfound hammerhead ribozymes exploit a pseudoknot interaction to form the tertiary structure necessary to stabilize the positioning of stems I and II. We also identified a number of active sequence variants that suggest the hammerhead consensus is more variable than previously thought.

Although the biological functions of these hammerhead ribozymes remain unproven, some could be involved in gene regulation based on their genomic contexts, similarly to what has been proposed for the mouse hammerhead and human HDV ribozymes [14], [17], [18]. Although *glmS* ribozymes [19] are known to control gene expression by using a metabolite as an active site cofactor to promote mRNA cleavage, gene regulation by other ribozymes such as the hammerhead might rely on protein- or small-molecule-mediated allosteric control of self-cleavage activity.

## Results

### Thousands of newfound hammerhead ribozymes

We used a comparative genomics pipeline [20] integrating homology searches [21] and the algorithms RNAMotif [22] and CMFinder [23] to identify structured RNAs in available sequences [20]. In addition to many novel motifs, we identified numerous examples of RNAs that conform to the well-established consensus sequence for hammerhead self-cleaving ribozymes (Figure 1A). We eventually conducted a comprehensive search of all available genomic DNA, which allowed us to expand the collection of hammerhead ribozymes from ∼360 previously known examples to more than 10,000 (Figure 1B; see sequence alignments in Dataset S1).

A large number of additional hammerhead ribozymes were identified in metazoans, including mosquitoes and sea anemones. While many hammerhead ribozymes associated with repeated elements were previously found in various species of *Dolichopoda* (cave crickets) [24], [25] and *Schistosoma mansoni* (parasitic worm) [12], they represent only a small fraction of all occurrences. *Aedes aegypti* (mosquito), *Nematostella vectensis* (sea anemone), *Xenopus tropicalis* (frog) and *Yarrowia lipolytica* (fungus) also appear to have hammerhead ribozymes associated with interspersed repeat elements, which are found in multiple copies in their genomes. Interestingly, we uncovered instances of this motif in humans, and the genetic contexts of two of these are conserved among many mammals.

Our search efforts also extended the range of known hammerhead ribozymes beyond the eukaryotic domain of life. At least three representatives are present in archaea and hundreds are present in bacteria (Figure 1B), where many are in proximity to integrase genes frequently grouped with prophages. Although the precise biological functions of these hammerheads remain unclear, the fact that nearly all carry conserved sequence and structural features (Figure 1A) previously proven to promote RNA cleavage by internal phosphoester transfer suggests that they also promote high-speed RNA cleavage. Almost without exception, the catalytic core of each representative matches the consensus hammerhead sequence. Also, the three base-paired stems enclosing the catalytic core typically show variability in sequence and length, with stem II commonly formed by as few as two base pairs.

**Figure 2:**
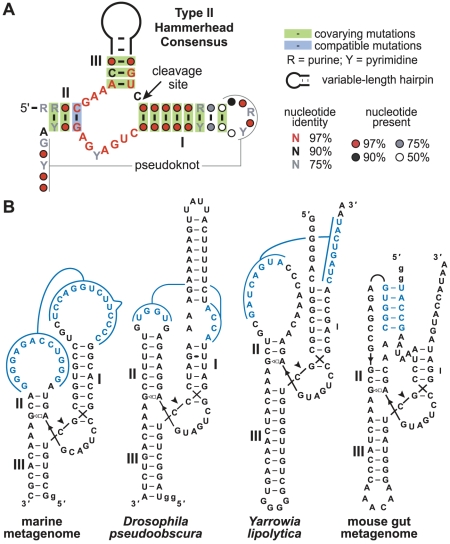
Type II hammerhead and representative pseudoknot substructures in type I, II and III ribozymes from diverse sources. (A) Consensus sequence and secondary structure of widespread type II hammerhead ribozymes identified in this study. A pseudoknot forms the tertiary contacts that are presumed to stabilize parallel orientation of stems I and II. (B) Sequences and secondary structures of four type II hammerhead ribozymes. Diagrams reflect the orientation of stems I and II in the catalytically active structure. Closed circles represent wobble base pairs and the open square and triangle represent a *trans* Hoogsteen/sugar-edge interaction [65]. Arrowhead indicates cleavage site.

However, several novel features for this ribozyme class were observed among the expanded list of representatives. Among the notable variants are the first natural examples of “type II” hammerhead architectures (Figure 2A), wherein stems I and III are closed by hairpin loops while stem II lacks a loop. Although type II hammerheads are functional [12], they were paradoxically thought to be absent in nature. Our findings reveal that all three circularly permuted architectures indeed are common in nature. Specifically, type II hammerhead ribozymes are very common in eubacteria and are also present in some archaeal species.

The type II hammerhead consensus identified in our bioinformatics search included a putative conserved pseudoknot linking the loop of stem I with the 3′ tail extending from the right shoulder of stem II (Figure 2A). Indeed, a majority of type II hammerhead motifs have potential pseudoknots of four or more base pairs between loop I and nucleotides immediately downstream of stem II. On further examination we found that pseudoknots can be formed by numerous representatives of all three hammerhead types (Figure 2B), suggesting that the tertiary structure required to stabilize the parallel assembly of stems I and II is commonly achieved by this base-paired substructure (see below).

**Figure 3:**
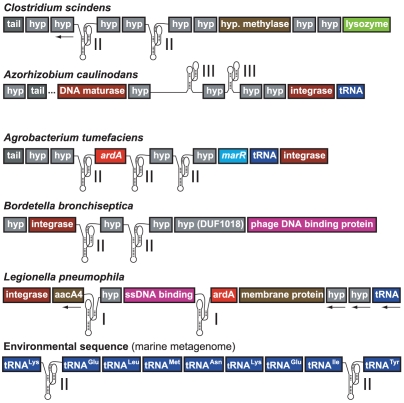
Examples of gene contexts of clustered hammerhead ribozymes. Hammerhead types (I, II or III) are indicated. Transcription from left to right is predicted for individual genes and operons, except in cases where arrows denote the opposite gene orientation. Genes, including those that encode hypothetical proteins (hyp), are labeled according to their respective genome annotations.

On many occasions, multiple hammerhead ribozymes are arranged in close proximity to flank individual genes or short blocks of genes in prophage genomes, although the identities of these genes are not constant (Figure 3). Such arrangements imply that long bacteriophage RNA transcripts may be processed into operon- or single-gene-length mRNAs, although other possibilities exist. For example, some hammerheads may not be functional, or successive ribozyme-mediated cleavage and ligation reactions could yield spliced or circular RNA products, although we were unable to detect either type of product in this study (data not shown).

Three tandem hammerhead arrangements from *Clostridium scindens*, *Azorhizobium caulinodans* (Figure S1) and *Agrobacterium tumefaciens* (Figure S2) were tested for cleavage activity during in vitro transcriptions of constructs corresponding to ∼2 kb fragments of the native polycistronic RNAs. In each case, cleavage products were observed that correspond to the sizes expected if all ribozymes were active and efficiently promoted self-cleavage reactions.

Ribozymes from the triple hammerhead arrangement of *A. tumefaciens* flank *ardA*, a gene involved in protecting phages from bacterial restriction enzymes, and another gene of unknown function (Figures 3 and S2). These ribozymes exhibit self-cleavage activity in vivo following cloning and transcription of the appropriate *A. tumefaciens* DNA fragment in *E. coli* (Figure S3). Although the biological purpose of this triple arrangement is unknown, the *ardA* gene is located immediately downstream of a hammerhead ribozyme in three strains of *Legionella*, suggesting that ribozyme action may be important for this gene.

### A pseudoknot commonly stabilizes the active hammerhead structure

**Figure 4:**
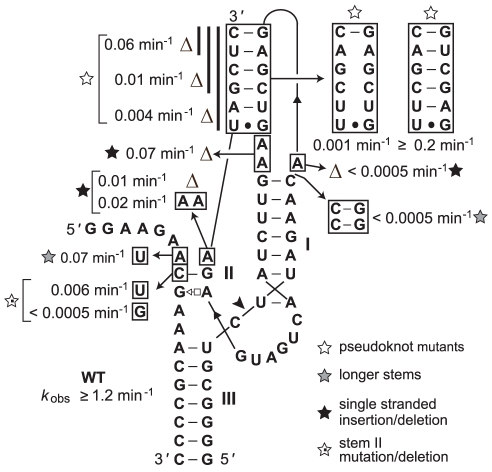
Mutational analysis of a metagenome-derived bimolecular hammerhead construct containing a one-base-pair stem II. The indicated *k*_obs_ values were established in ribozyme reaction buffer containing 0.5 mM Mg^2+^ with incubation at 23°C. Deletions are designated by a delta symbol. Other notations are as described in Figure 2.

Previous studies demonstrated that non-Watson/Crick contacts between the terminal or internal loops in stems I and II play a critical role in forming the tertiary structure necessary for high-speed hammerhead function [6], [7]. However, many newfound hammerhead representatives instead are predicted to use a pseudoknot interaction to stabilize the parallel alignment of stems I and II (Figure 2). This prediction was assessed by conducting a series of RNA cleavage assays using various mutants of the type II hammerhead ribozyme from a metagenome dataset (Figure 4) and from several other sources (see Figure 2).

A bimolecular construct based on the wild-type (WT) ribozyme sequence exhibits an observed rate constant (*k*_obs_) for RNA cleavage of greater than 1.2 min^−1^ under single-turnover conditions and simulated physiological conditions (23°C, 0.5 mM MgCl_2_, 100 mM NaCl, 50 mM Tris-HCl [pH 7.5 at 23°C]). All deletions or other mutations that are predicted to disrupt the pseudoknot substructure drastically reduce cleavage activity (Figure 4). For example, deleting two nucleotides from the 3′ terminus to reduce the pseudoknot from six to four base pairs caused the *k*_obs_ to decrease by a factor of ∼20, and deleting another two nucleotides from this terminus reduced activity by a factor of more than 100 compared to WT. Mutating the pseudoknot has a similar effect, while the compensatory mutation restores high activity (Figure 4).

Deletions or insertions of nucleotides surrounding the pseudoknot also reduced *k*_obs_ values by orders of magnitude. Moreover, stabilizing stem I by adding two base-pairs, or stabilizing stem II by adding one additional base-pair also decreased ribozyme activity substantially. All of these mutations are located outside of the highly conserved ribozyme core and are designed to promote local structure formation. However, these mutations change the relative positions of nucleotides that form the pseudoknot, which likely disrupts the proper orientation of this tertiary structure critical for high-speed activity.

We also assessed pseudoknot formation by subjecting the longer of the two strands that form the bimolecular construct to in-line probing [26], which is an assay that can be used to map structured versus unstructured portions of RNA molecules. The pattern of spontaneous RNA fragmentation is consistent with formation of the pseudoknot in the absence of the second strand (data not shown). Likewise, in-line probing of this portion of bimolecular constructs from two other hammerhead ribozymes indicates that pseudoknot formation occurs even in the absence of the remaining portion of the ribozyme (data not shown).

All hammerhead ribozymes representatives were examined for the presence of a pseudoknot contact between stems I and II, revealing that approximately 40% likely use this structural constraint (Figure S4). Stem lengths appear constrained by this base pairing (Figure 4), but the constraints do not follow a simple rule and seem to vary for different types of hammerheads. The identification of pseudoknot interactions between these two substructures expands the known tertiary interactions described previously [6], [7] that are essential for high activity. However, there are many hammerhead ribozyme examples that do not appear to use these contacts, suggesting other types of interactions exist or that none are used in some cases (Figure S5).

### Core nucleotide variation is present in some hammerhead ribozymes

**Figure 5:**
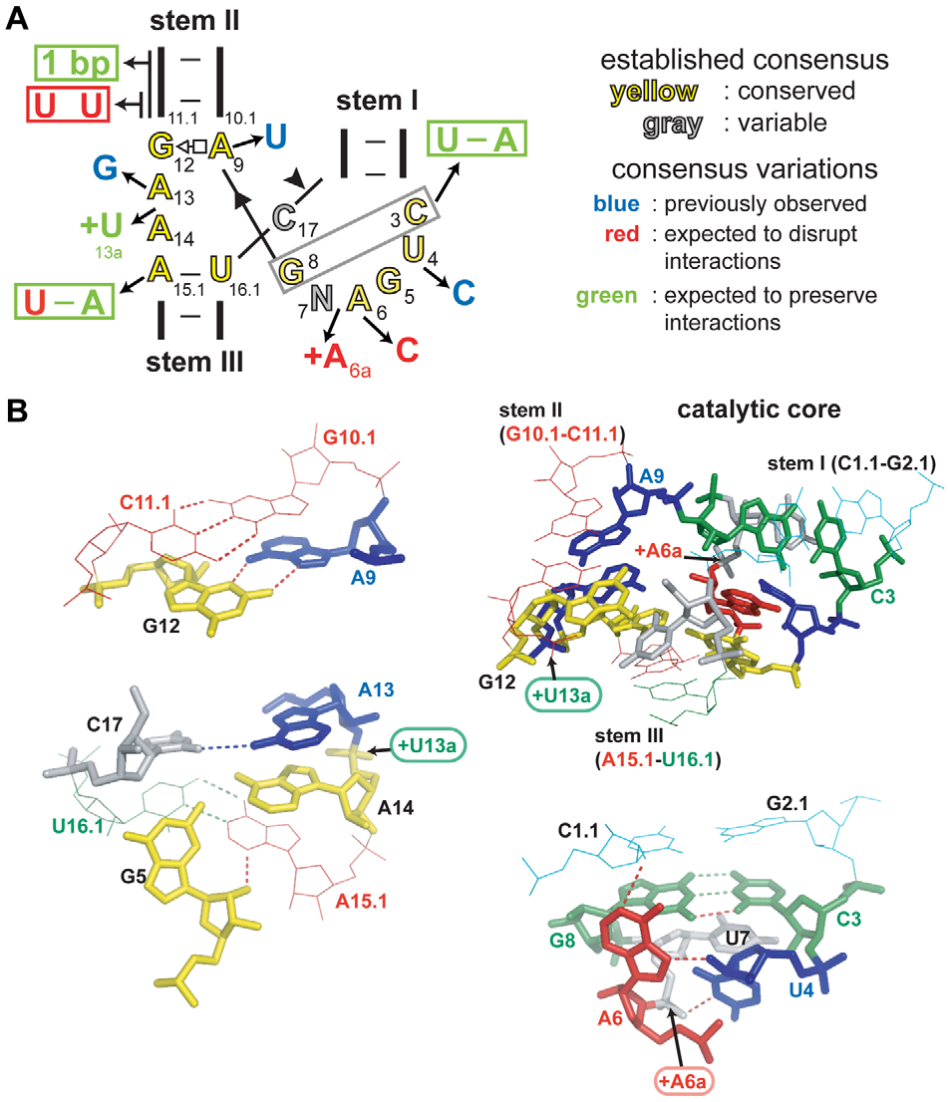
Rare nucleotide variations observed in the cores of some hammerhead ribozymes. (A) Consensus secondary structure of the hammerhead core with highly conserved residues in yellow and variable residues in gray. Blue letters designate active natural variants tested previously. Red and green letters designate natural variations tested in this study that are expected to have deleterious effects or neutral/compensatory effects, respectively, on ribozyme function. (B) Atomic-resolution structure of portions of the *Schistosoma mansoni* hammerhead core. Colors are as defined in (A), with the addition of yellow designating strictly conserved nucleotides (built from PDB accession 2GOZ with pymol [66]). Stem I is in cyan, stem II in red, and arrows indicate position of insertions. Dashed lines in red and green represent hydrogen bonds that are expected to be disrupted or maintained, respectively. Other notations are as described for Figure 2. For complete secondary structure and additional information on these variants see Figure S6 and Figure S7 for variants that were inactive.

The importance of conserved catalytic core nucleotides has been well established by numerous previous studies [5], including the use of systematic mutational analyses [27] and in vitro evolution [28], [29]. Some RNAs with core mutations do retain modest levels of cleavage activity, but the decreases are generally assumed to render the ribozyme biologically non-functional. Despite the fact that the core is exceptionally well conserved, three hammerhead ribozymes previously identified from viroids have core nucleotides that deviate from the consensus [30], [31], suggesting some changes do preserve biological function (shown in blue, Figure 5A and 5B).

Our expanded collection of hammerhead representatives revealed additional examples of core variation (Figure 5A). Most of the known interactions and important chemical groups within the core are minimally affected in these variants. However, some interactions predicted to be important based on atomic-resolution structural models are disrupted in some cases. Several ribozymes with variant core sequences were assayed to determine how these changes affect RNA cleavage activity.

Some of these variant cores carry compensatory changes that prevent severe alteration to the active structure (Figure 5). For example, core nucleotides C3 and G8 form a base pair, and these nucleotides covary to U3 and A8 in several hammerhead ribozyme examples. Ribozymes containing covarying nucleotides at these positions had already been proven to be active in vitro [32], but covariation at these positions had not previously been observed in nature.

A hammerhead sequence found in an intergenic region of bacteriophage Bcep176 (Figure S6) carries an A6C variation that is expected to disrupt at least one hydrogen bond and potentially two. Correspondingly, we observe a *k*_obs_ of less than 0.1 min^−1^, which is in agreement with the low activity that a previous mutational analysis of the core revealed for changes at this position [27]. Similarly, low activity of an insertion observed after A6 (called A6a in Figure 5B) is consistent with the fact it should disrupt a hydrogen bond observed in the crystal structure because the phosphate connecting A6 to N7 interacts with U4. Changing the backbone conformation at this position would be expected to be detrimental to an active core.

An insertion is likely to be easier to accommodate if the phosphate backbone is protruding out of the otherwise compact structure. Thus U13a, (Figure 5A and 5B) which is inserted in the “GAAA” region of the core, could point outside of the core, resulting in minimal structural change. A sequence with U15.1–A16.1 instead of A-U, usually considered essential, self-cleaves, albeit less efficiently than a typical hammerhead ribozyme. This is likely caused by the loss of an interaction observed between A15.1 and G5. WT ribozymes have been shown to exhibit at least 10-fold greater activity compared to mutants at nucleotides 15.1 and 16.1 examined in previous in vitro studies [27], [33].

The activities of these core variants are consistent with the findings of previous biochemical studies that assessed the importance of individual chemical groups for activity. For example, the U15.1–A16.1 and C6 mutations are expected to disrupt the core, and did result in low, but detectable, activity. Additionally, for some predicted ribozymes that have mutations expected to be highly disruptive, no activity was detected (Figure S7).

In addition to exhibiting variation of the core, some hammerhead ribozymes have very weak stems. In particular, stem II often consists of only two base-pairs and even a single base-pair in one case (Figure 4). It is even more surprising that stem II can start with a U10.1–U11.1 mismatch (Figure 5) since this is the most conserved base-pair of the hammerhead consensus, aside from A15.1–U16.1 (Figure 1A). However, this U-U mismatch had already been shown to support higher levels of cleavage activity than any other mispaired combination [27]. Weak stems III were also very common (Figure S8).

### Hammerhead ribozyme variants from high-salt environments

Several hammerhead ribozyme representatives were identified among sequences derived from viral fractions of solar salterns (see sequence alignments in Dataset S1). Solar salterns consist of a series of interconnected pools of increasing salinities, and culminate in crystallizer ponds from which various salts are precipitated and harvested. These saturating brines are inhabited predominantly by extreme halophiles of the archaeal domain, and these organisms contend with the acute hypersaline environment primarily by maintaining high intracellular concentrations of K^+^ ions [34]. Therefore, we speculated that hammerhead variants from this source might become active in high salt.

**Figure 6:**
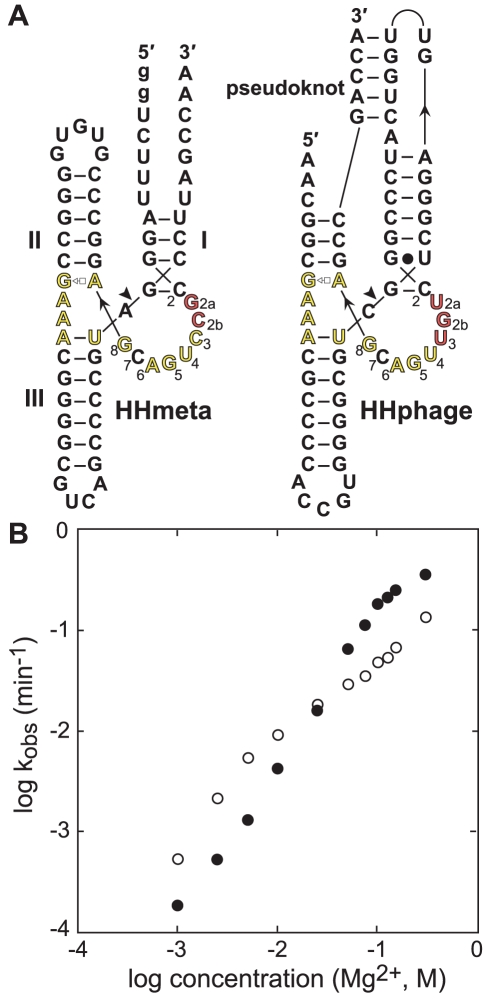
Variant hammerhead ribozymes encoded in saltern-derived DNA. (A) Secondary structure models of variants HHmeta and HHphage. Annotations are as described for Figures 2 and 5. Residues corresponding to the highly atypical insertions are numbered 2a and 2b. Guanosine residues depicted in lowercase were added to facilitate transcription in vitro. (B) Effect of MgCl_2_ concentration on the *k*_obs_ of HHmeta. *k*_obs_ values were determined in the absence of KCl (open circles) or in the presence of 3 M KCl (filled circles).

Three of the hammerhead examples from this environment carry short insertions in the catalytic core near the C3 nucleotide and P1 stem (Figure 6A). Such changes in this local region of the catalytic core are unprecedented among reported examples of hammerhead ribozymes. Furthermore, based on the atomic resolution structure of the hammerhead active site [8], insertions of this type are expected to destabilize the catalytic core. It is important to note that one of the sequences derived from saltern metagenomes had a typical consensus, so it appears that alteration of the catalytic core is not a requisite feature of hammerhead ribozymes from extremely halophilic environments. However, these three unusual variants were found only in the genomes and metagenomes of solar salterns.

To examine whether these alterations of the catalytic core reflect adaptations to hypersaline conditions, we prepared wild-type and mutant versions of HHmeta (Figure 6A) derived from saltern metagenomic data. Only very low levels of self-cleavage activity were detected for HHmeta during transcription in vitro (data not shown), despite the presence of 15 mM MgCl_2_. In contrast, an engineered mutant in which the two-nucleotide insertion (G2a and C2b) was removed to create a consensus catalytic core undergoes nearly quantitative self-cleavage during transcription (data not shown). Thus, the unusual insertion sequence in this saltern-derived hammerhead ribozyme impairs cleavage activity under standard assay conditions.

To test whether elevated salt concentrations can rescue this deficiency, we first determined the *k*_obs_ for self-cleavage of HHmeta in a high concentration of monovalent ions alone. HHmeta undergoes self-cleavage with a *k*_obs_ of 4×10^−4^ min^−1^ in 4 M LiCl (data not shown). For comparison, a consensus hammerhead ribozyme catalyzes strand scission with a *k*_obs_ of 0.17 min^−1^ under similar conditions [35], a *k*_obs_ that is 425-fold faster than that of the saltern-derived variant.

To assess whether more appreciable activity of HHmeta requires elevated levels of divalent metal ions, we measured *k*_obs_ values over a range of Mg^2+^ concentrations. The activity of the variant increases with increasing Mg^2+^ levels (Figure 6B), mirroring the behavior of consensus hammerhead ribozymes [36]. However, HHmeta requires substantially higher Mg^2+^ concentrations to achieve comparable *k*_obs_, such that a Mg^2+^ concentration of 300 mM is necessary to attain a *k*_obs_ of ∼0.13 min^−1^. Values for *k*_obs_ are slightly improved at higher Mg^2+^ concentrations when reactions are supplemented with 3 M KCl (Figure 6B), with the monovalent ions likely providing additional structure stabilization. Conversely, the added KCl results in slightly decreased *k*_obs_ values in the lower range of Mg^2+^ concentrations, due presumably to competition with Mg^2+^-binding sites [37]. Nonetheless, it is clear that the concentration of Mg^2+^, and not that of monovalent cations, has the most pronounced effect on the self-cleavage activity of HHmeta. Mg^2+^ ions are smaller and more densely charged than monovalent ions, and thus might more effectively stabilize the active structure of HHmeta through low-affinity, diffuse interactions [38]. Elevated Mg^2+^ concentrations might be important for global folding of HHmeta, or could be necessary to compensate for the putative destabilized active site of the variant. It is also possible that Mg^2+^ ions provide a larger direct contribution to catalysis in HHmeta than in consensus hammerhead ribozymes.

### Two conserved human hammerhead ribozymes are active

**Figure 7:**
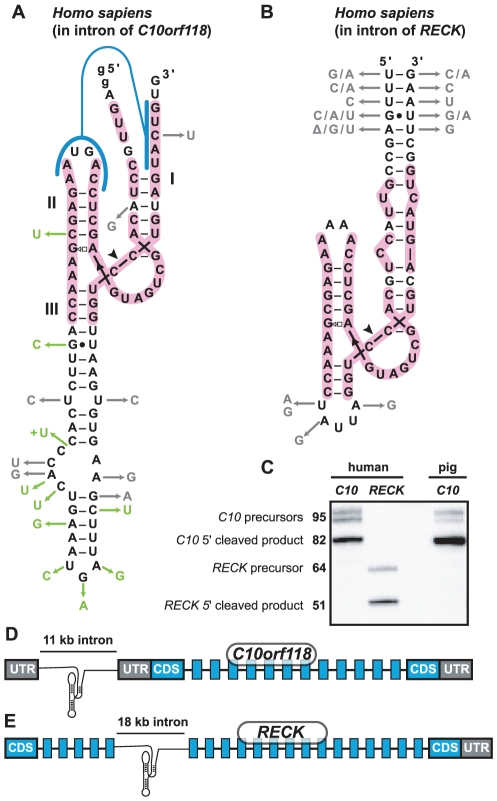
Two conserved human hammerhead ribozymes. (A) Hammerhead from human *C10orf118* intron with nucleotide substitutions and insertions occurring in pig shown in green. Variations observed in other mammals are in gray. Guanosine residues depicted in lowercase were added to facilitate transcription in vitro. (B) Hammerhead from human *RECK* intron with nucleotide variations observed in other mammals and birds in gray. Sequence with pink background highlights identical nucleotides between *C10* and *RECK* hammerhead sequences. Other notations are as in Figure 2. (C) Self-cleavage during transcription in vitro of *RECK* and *C10orf118* hammerhead ribozyme sequences from human and pig. The pig and human *RECK* hammerhead ribozymes are identical. Expected nucleotide lengths of RNA precursors and 5′ cleavage products are shown. First and last five nucleotides of *RECK* in (B) are depicted to illustrate boundaries of conservation, but are not part of the transcript. (D) and (E) Genetic contexts of the human hammerheads. Untranslated region (UTR) is colored in gray and coding sequence (CDS) in blue. Gene organization is not to scale, size of hammerhead-containing introns is according to NCBI annotation (build 37).

Our homology searches reveal the presence of nine regions in human genomic DNA that conform to the consensus for hammerhead ribozymes (see sequence alignments in Dataset S1). Two candidates (Figure 7A and 7B) appear to be conserved among some other vertebrates, and therefore were chosen for experimental validation. These two candidates are the same that have been reported very recently [15]. Robust self-cleaving activity of one representative, termed “*C10* hammerhead”, was observed during in vitro transcription for both human and pig sequences (Figure 7C). As do many new-found hammerhead ribozymes noted above, this RNA appears to use pseudoknot formation to stabilize the active structure. As expected, a truncated form of the ribozyme that lacks the five base-pair pseudoknot is inactive when assayed at 0.5 mM MgCl_2_ (data not shown).

The *C10* hammerhead is found within an intron in the 5′ untranslated region (UTR) of *C10orf118* (Figure 7D), which is a gene of unknown function that is conserved throughout mammals. The *C10* hammerhead is present in all examined sequenced mammalian species with the exception of mouse and rat, which do not carry an intron in the 5′ UTR of this gene. The biological significance of *C10* hammerhead self-cleavage is not clear. Genbank and GeneCards EST data indicate that the RNA is expressed in at least 18 tissues [39], [40] (Figure S9), and RT-PCR on the first exon of *C10orf118* yields product that demonstrate expression of the gene in four human cell lines (Figure S9). One possibility is that cells control 5′ UTR splicing by controlling hammerhead action.

The second human hammerhead we subjected to further analysis, termed “*RECK* hammerhead”, resides in an intron of the gene for *RECK* (reversion-inducing cysteine-rich protein with Kazal motifs), a negative regulator of certain metalloproteinases involved in tumor suppression [41]. This arrangement is conserved in all mammals and birds examined (Figure 7E). The ribozyme appears to lack a pseudoknot, but perhaps interactions between loop II and stem I substitute for this tertiary contact as is observed for many hammerhead representatives. The *RECK* hammerhead also tested positively for cleavage in vitro (Figure 7C). According to EST data (I.M.A.G.E. consortium) [42], the exons flanking the hammerhead-containing intron appear to be alternatively spliced, and are usually absent from *RECK* transcripts expressed in nervous system tissue, although they are present in the corresponding RNAs from most other tested tissues. Interestingly, two ESTs from *Bos taurus* have sequences corresponding exactly to the hammerhead's 3′ cleavage product fused with those matching RNA components of U snRNPs (U5 and U6, EST accession numbers are DV870859.1 and DV835419.1), suggesting that this ribozyme may be active in vivo.

## Discussion

The application of increasingly powerful bioinformatics algorithms to the expanding collection of DNA sequence data is facilitating the discovery of novel noncoding RNAs and revealing new locations for previously known examples. A recent report [17] revealed additional representatives of the HDV self-cleaving ribozyme class, which are widely distributed among many organisms. Previously, this ribozyme had been considered one of the least commonly occurring of the self-cleaving RNA classes. In the current study, we expand the number of reported hammerhead ribozymes by more than an order of magnitude compared to what was known previously, and we have identified members of this ribozyme class in all domains of life. Our findings strongly suggest that hammerhead ribozymes comprise the most abundant self-cleaving ribozyme class in nature. Almost simultaneously, three groups have recently used computational methods to discover additional hammerhead ribozymes. These efforts revealed hammerhead ribozymes in bacteria and various eukaryotes, although their methods differed from ours and were not used to identify variants from the consensus [15], [16], [43], [44].

Previous in vitro selection studies demonstrated that hammerhead ribozymes are among the first self-cleaving motifs to emerge from random-sequence populations [45], [46]. These findings suggest that this is one of the simplest ribozyme architectures that can cleave RNA efficiently and that this simplicity ensures multiple evolutionary origins. This latter conclusion also is supported by our observation that type I, II and III hammerhead motifs are very common, which would be unlikely if all hammerhead ribozymes descended from a single founding example of a given type.

Although the hammerhead consensus is highly conserved, there are rare instances in which the catalytic core is altered. Previous studies have established that mutations at most positions in the core resulted in drastic loss of activity [5], [27], and consequently such variants are not expected to be found in nature. Nevertheless, three divergent cores were previously shown to exhibit self-cleavage activity [30], [31], and we add eight additional variants to this collection (Figure 5A, 5B and 6). It is likely that any adverse effects resulting from the variant cores are offset by stabilizing influences from tertiary contacts outside the active site, which would permit physiologically relevant activities of these natural variants. Consistent with this hypothesis is the observation that the U4C variant that considerably decreases activity in vitro maintains sufficient activity in vivo to permit viroid infectivity [30].

The diversity of structural alternatives observed in our hammerhead collection hints at the inherent difficulty in any effort to comprehensively identify ribozyme representatives. Including more core variations or distal structure variations in search outputs will result in larger numbers of false positives. Given the simplicity of the motif, sequences that conform to the consensus are expected to occur by chance in large sequence databases, even if some of them might be incapable of folding into an active hammerhead.

Although numerous hammerhead examples can be discovered by comparative sequence analyses, the identification of those that are biologically relevant will ultimately require experimentation in vivo. For example, viroid hammerhead sequences can experience mutations at high frequency, and most of these mutations result in non-infectious phenotypes [47], but some are still infectious in spite of a less active ribozyme [30]. In this study, we tested 18 hammerhead ribozymes conforming to the consensus, with 14 exhibiting activity in vitro. No cleavage was detected for the remaining four examples under our assay conditions, although two of these inactive RNAs are derived from *Aedes* and *Nematostella*, organisms in which active hammerheads might require dimeric conformations (Figure S8). It is thus possible that these ribozymes follow a more complex folding pathway that is more difficult to reproduce experimentally. However, some other inactive candidates are more likely to be false positives, such as a putative type II hammerhead in humans, which lacks conservation of the hammerhead structure in closely related species (see sequence alignments in Dataset S1).

The previous absence of known natural examples of type II hammerheads suggested that this architecture might not be biologically useful. However, our findings demonstrate that all types of hammerhead ribozymes are exploited naturally. Nevertheless, the vast majority of hammerhead ribozymes associated with repeated genetic regions in eukaryotes are of type I. This is most likely due to the evolutionary origin of the repeats, wherein the initial sequence carried a type I hammerhead that was widely propagated. Alternatively, it is possible that repeat propagation may require a type I hammerhead architecture. For example, if the ribozyme was involved in *cis* cleavage and *trans* ligation reactions to DNA, then type I ribozymes are the only architecture that would provide a 2′,3′-cyclic phosphate terminus and the bulk of the catalytic architecture to ligate to a separate nucleic acid strand carrying a 5′ hydroxyl group. This ligation reaction between RNA and DNA with a type I hammerhead architecture has been previously demonstrated [48]. This is only an example of how type I hammerhead could have been favored.

Based upon the abundance of hammerhead motifs we find associated with DNA repeats, self-cleaving ribozymes appear to be especially common in selfish elements (Figure 1B). This trend is also evident for group I and group II self-splicing introns [49], which commonly are associated with selfish elements. Moreover, other self-cleaving ribozyme classes may have similar distributions, as is evident from the recent report of HDV ribozyme representatives associated with R2 retrotransposons [50]. A possible outcome of these arrangements is that some selfish element harboring a ribozyme will occasionally integrate at a site where the ribozyme provides a selective advantage to the host. Strongly suggestive of this scenario is the striking similarity between the two most conserved vertebrate hammerhead ribozymes (pink regions Figure 7A and 7B) and the repeat-associated hammerhead sequences found in *Xenopus* (see AAMC01XXXXXX accession numbers in sequence alignments of Dataset S1). Hence, a hammerhead-containing element in an ancestral amphibian, apparently still active in some contemporary frogs, might have been retained in *C10* and *RECK* introns because of advantages provided by self-cleavage at these sites, but would have been lost at most other positions.

The hammerheads in viroids process multimeric genomic RNAs, and in such cases constitutive RNA cleavage may be desirable. However, it is possible that some of the hammerheads of retroelements or bacteriophages will have more diverse functions, such as regulated RNA cleavage. This seems likely for the two validated hammerheads found in human introns, wherein the utility of constitutive cleavage activity would be difficult to rationalize. It is notable that the hammerhead ribozyme recently reported in mouse [14], [51] has a very large loop structure that could be naturally exploited for ribozyme control [18]. Similarly, the slower ribozyme variants in bacteriophages might become more active under the appropriate physiological conditions or upon interaction with molecular signals.

For some hammerhead variants such as HHmeta, activity may be facilitated by extreme salt concentrations. In vitro assays reveal that HHmeta requires at least 75 mM MgCl_2_ to attain biologically relevant *k*_obs_ values (greater than 0.1 min^−1^). For most organisms, this divalent magnesium concentration is not attained. However, for microbes inhabiting certain environments, such as the Dead Sea or high salinity zones of solar salterns, growth has been reported in extracellular MgCl_2_ concentrations ranging from 0.6 to >2 M [52], [53]. Importantly, for certain extreme halophiles grown in medium containing 0.75 M Mg^2+^, estimates of the intracellular Mg^2+^ concentrations range as high as 0.42 M [54]. Such a high-salt environment for HHmeta might relax the need for strict conservation of the catalytic core. The variant hammerhead may thus function constitutively in an extremely halophilic host, perhaps fulfilling an RNA processing role.

Alternatively, it is possible that HHmeta and related variants have been selected to function as gene control elements that modulate the expression of associated genes in response to fluctuating intracellular salt concentrations. HHmeta was identified in a metagenome survey as part of a short sequence fragment, and therefore its genomic context is unknown. However, the structurally analogous hammerhead ribozyme variant HHphage (Figure 6A), which resides within the completed genome sequences of haloviruses HF1 and HF2, is in each case positioned only 13 nucleotides upstream of the start codon corresponding to an ATP-dependent DNA helicase. HF1 and HF2 are highly related lytic bacteriophages targeting extreme halophiles of the archaeal domain, and possess linear double-stranded DNA genomes [55]. The HHphage-associated helicase gene is located in the section of the genome containing early genes, which are presumably involved in initiating virus replication, and corresponds to the first of several ORFs within a polycistronic transcript [56]. Intriguingly, the 5′ end of this major transcript was mapped using primer extension [56] to within three nucleotides of the HHphage cleavage site, suggesting that this hammerhead ribozyme variant is active in vivo.

Dilution of the environment is highly toxic for obligate extreme halophiles. Accordingly, for certain bacteriophages that infect these organisms, virulence is tightly controlled in response to salt concentrations [57]. This allows bacteriophage to proliferate more aggressively when dilution threatens the viability of their hosts. Conversely, when salt levels are saturating, a carrier state is established in which phage DNA is propagated with a minimal burden on the host organism [58]. It is conceivable, then, that an appropriately tuned hammerhead ribozyme variant could be utilized by a halovirus to modulate the stability of a key transcript in a salt-dependent manner, thereby acting as a component of this regulatory response.

The discovery of thousands of new hammerheads in all three domains of life provides many opportunities to examine the functions and biological utilities of these ribozymes in their natural contexts. The activities of some representatives may be regulated by RNA folding changes induced by changes in protein, metabolite, or metal ion concentrations, similar to the structure modulation observed with riboswitches. Previous engineering efforts produced numerous examples of allosteric hammerhead ribozymes or other RNAs, establishing a precedent for ligand-mediated regulation of ribozyme function [59], [60]. In this context, the pseudoknot interactions identified in our study could be more easily manipulated to create regulated allosteric ribozymes via rational design.

## Material and Methods

### Bioinformatics searches for hammerheads

Type II hammerheads were uncovered by a comparative genomics method described previously [21], [67]. Briefly, clusters of homologous non-coding sequences were analyzed with CMfinder to predict secondary structures and iterative homology searches conducted with RaveNnA [21]. A series of descriptors for RNAMotif were also used to find new hammerheads (descriptors in Text S1). All new hammerheads were combined with previously known examples and used as three updated alignments, type I-II-III, to perform homology searches on all RefSeq version 37 and available environmental sequences [21] using Infernal [68].

For final alignments, possible false positives were eliminated based on three criteria. First, any mutation in the core disqualified the hit. For this purpose, the consensus core was considered to be: C3, U4, G5, A6, N7, G8, A9, G12, A13, A14, A15, U16 and H17, where “N” means any nucleotide and “H” means A, C or U. Second, any mispairing directly adjacent to the core in stems I, II or III (*i.e.*, N10.1–N11.1, N1.1–N2.1, and A15.1–U16.1) also led us to reject the hit. Finally, multiple mispairs or bulges in short stems resulted in candidate disqualification. The list of rejected hits consisted mainly of cryptic mutant hammerheads that are part of repeated elements, but those occurring in typical gene contexts (*e.g.*, prophage) were often tested, as they were considered likely functional variants. Initially rejected hits were included in hammerhead alignments if activity could be measured.

### RNA synthesis and labeling

To produce in vitro transcription templates, PCR was performed using genomic DNA isolated from *Agrobacterium tumefaciens*, *Azorhizobium caulinodans* (ATCC), *Clostridium scindens* (ATCC), PaP3 bacteriophage (kind gift of Professor Fuquan Hu) [69], pork chops (Shaw's Supermarket) and human whole blood (Promega). In cases where genomic DNA was unavailable, templates were constructed from chemically synthesized oligodeoxynucleotides (see Table S1). Transcriptions were generally conducted in 80 mM HEPES–KOH (pH 7.5 at 23°C), 24 mM MgCl_2_, 2 mM spermidine, 40 mM DTT, 2 mM of each ribonucleotide and 40 U µl^−1^ of purified T7 RNA polymerase. For ribozyme assays in *trans*, RNA was purified using denaturing PAGE, visualized by UV shadowing, and eluted in 200 mM NaCl, 10 mM Tris-HCl (pH 7.5 at 23°C), and 1 mM EDTA. RNA was then precipitated in ethanol, and the resulting pellet was rinsed in 70% ethanol and resuspended in water. Concentration was measured by UV spectrophotometry with a Nanodrop ND8000 (ThermoScientific).

For 5′ labeling, RNA was dephosphorylated with calf intestinal phosphatase (NEB) according to the manufacturer's instructions. Following phosphatase inactivation at 94°C for 3 minutes, 1 pmole of dephosphorylated RNA was typically used for 5′-end-labeling with T4 polynucleotide kinase (NEB) and [γ-^32^P]ATP according to the manufacturer's instructions. Labeled RNA was gel-purified as described, but visualized by autoradiogram.

### Analysis of hammerhead kinetics in trans

To design bimolecular constructs, loop III was opened and base pairs were added to stabilize stem II by extending it to at least seven base pairs. Both RNA molecules were then transcribed from different synthetic DNA templates. RNA designated as the “ribozyme” (the strand not containing the cleavage site) was used in 200-fold excess for single-turnover kinetics. Typically, ∼5 nM radiolabeled substrate and 1 µM ribozyme were heated together at 65°C for two minutes in a 10 µl volume containing 100 mM Tris-HCl (pH 7.5 at 23°C) and 200 mM NaCl. After cooling to room temperature and removing time zero aliquots, 10 µl MgCl_2_ was added to a final concentration of 500 µM, unless otherwise stated. Reactions were stopped at various times with 5 volumes of stop buffer (80% formamide, 100 mM EDTA, 0.02% bromophenol blue and 0.02% xylene cyanol).

All time points for a given experiment were analyzed on the same denaturing gel, ranging from 6% to 20% polyacrylamide, depending on substrate and product sizes. After drying the gel, radiolabeled species were imaged using a Storm 820 PhosphorImager and analyzed with ImageQuant software (Molecular Dynamics). Values for *k*_obs_ were derived from the slope of the line obtained by plotting the natural logarithm of the fraction of precursor RNA remaining versus time. Calculations were performed assuming first order reaction kinetics using data points corresponding to the first 5% to 30% of the reaction. Many ribozymes exhibit biphasic reaction kinetics. For these, we used SigmaPlot (SYSTAT) to fit the curves to the equation F = a(1−e^−bt^)+c(1−e^−dt^) by non-linear regression, where “F” is the fraction cleaved, “t” is time, “a” is the fraction cleaved where RNA molecules are cleaved at *k*_obs_ “b” (the larger *k*_obs_) and “c” is the fraction cleaved at a *k*_obs_ “d” (the smaller *k*_obs_) [70]. *k*_obs_ values reported for rapidly cleaving ribozymes should be considered lower bounds due to the limitations of manual pipetting.

### Analysis of hammerhead kinetics in cis

To estimate *k*_obs_ values for reactions in *cis*, ribozyme cleavage time courses were performed during transcriptions in vitro. Transcriptions were assembled in either 80 mM HEPES–KOH (pH 7.5 at 23°C), 24 mM MgCl_2_, 2 mM spermidine, 40 mM DTT or in 10 µl volumes containing 50 mM Tris-HCl (pH 7.5 at 23°C), 100 mM NaCl, 10 mM MgCl_2_, 2 mM each rNTP, and 40 units µl^−1^ T7 RNA polymerase. Polymerization was allowed to proceed for 5 minutes at 37°C, at which point 5 µl of an equivalent mixture was added that also contained trace amounts of [α-^32^P]UTP and [α-^32^P]GTP. Incubations were continued at 37°C, and 1 µl aliquots were removed at various time points and added to 14 µl of stop buffer. Due to the initially low levels of incorporation of radiolabeled nucleotides, the earliest time point that can practically be assessed is 20 seconds. Note that, due to the requirements of T7 RNA polymerase, the Mg^2+^ concentrations used in these assays are considerably higher than those used for assays in *trans*. Note also that HHmeta, because of its requirement for particularly high Mg^2+^ concentrations, was able to be isolated in precursor form from standard in vitro transcriptions, and was subsequently assayed in cleavage assays *in cis*.

## Supporting Information

Figure S1 Activities of RNA transcripts carrying multiple hammerhead ribozymes. (A) Cleavage of internally radiolabeled RNA during in vitro transcription of PCR products from *Clostridium scindens*. Bands correspond to the expected sizes for hammerhead ribozyme cleavage (other size markers not shown also support indicated fragment sizes). (B) Cleavage of internally radiolabeled RNA during in vitro transcription of PCR products from *Azorhizobium caulinodans*. Full length *Azorhizobium caulinodans* RNA is not detectable, presumably because of efficient ribozyme cleavage.
(PDF)

Figure S2 *Agrobacterium tumefaciens* multiple hammerhead ribozyme arrangement. (A) Genome context of ribozymes with annotations as follows: “tail” is a structural protein of the phage tail, “ardA” is an Anti-Restriction Defense protein, “marR” is a transcriptional regulation protein and “integrase” is a protein with predicted DNA integration activity. (B) Ribozyme cleavage during in vitro transcription using T7 RNA polymerase to produce internally-radiolabeled RNAs. Bands corresponding to expected sizes and compositions for hammerhead cleavage products are annotated.
(PDF)

Figure S3 *Agrobacterium tumefaciens* multiple hammerhead ribozyme activity in vivo. (A) Various RNA constructs expressed from plasmids carrying portions of *A. tumefaciens* hammerheads and expressed in *E. coli* BL21 cells. Various deletion or mutant constructs are expressed and examined by Northern analysis in the indicated lanes on polyacrylamide gel electrophoresis in panels (B, C and D). (B, C, D) Northern analysis of RNA products express in *E. coli* BL21 cells from the plasmids (pUC19 with the lac promoter removed) depicted in A. Transcription by T7 RNA polymerase was induced by IPTG and *E. coli* RNA polymerase was inhibited by rifampicin where indicated to maximize the amount of T7 RNA polymerase transcripts. After 2 h IPTG induction, RNA was extracted with Trizol according to the manufacturer's instructions (in the presence of EDTA). The RNA products were separated by denaturing agarose gel electrophoresis and the resulting gel was used for blotting. Probes for ORF 3, 2 and 1 were successively used to generate the images presented in (B, C and D), respectively. Because probes could not be entirely washed off the membrane between different probing experiments, there is some carry over from the probing in B to C to D. This explains why some bands corresponding to “ORF 2” can be seen in panel D for example.
(PDF)

Figure S4 Pseudoknot interaction between stems I and II. (A) Proportion of each type of hammerhead ribozyme that is predicted to form a pseudoknot. (B) Proportion of hammerhead ribozymes with a pseudoknot versus the length of stem II. (C) Proportion of ribozymes with a pseudoknot versus the difference in length between stems I and II (stem II number of base pairs subtracted from stem I number of base pairs). The highly repetitive type I hammerhead representatives are excluded from the analyses in B and C. Notes: There is some bias in the distributions of pseudoknots with the various hammerhead types and stem lengths. For example, stem length difference disparities between different types of hammerheads is especially striking in type III hammerheads where a two base pair difference between stems I and II commonly are associated with a pseudoknot, while other length differences are not. However, our data indicate that the pseudoknot contact generally is a structurally versatile way to constrain the locations of stems I and II.
(PDF)

Figure S5 Tertiary interactions between stems I and II. (A) Proportion of each type of hammerhead ribozyme that has predicted tertiary interactions noted in the diagrams (terminal loops or internal bulges). Notations: U, uridine; Nm, m number of any nucleotides; Y, pyrimidine; N, any nucleotide; R, pyrimidine; A, adenosine. (B) Proportion of each type of hammerhead ribozyme that have the tertiary interactions depicted in A either with or without a pseudoknot. Note that even when no tertiary interactions are predicted, some unknown interaction or a variation of a known interaction could exist.
(PDF)

Figure S6 Complete sequences and secondary structure models of active hammerhead ribozymes that carry core variations. Positions diverging from the core consensus sequence are depicted in red. Variants were tested in trans or in cis as illustrated. Note that the RNA for the PaP3 bacteriophage and *Clostridium scindens* hammerhead ribozymes are part of larger RNAs transcribed from PCR products. Non-native guanosine residues (lowercase) were added to facilitate transcription in vitro. Gene context is as follows: PaP3 and Bcep176 (phage intergenic regions); *Clostridium scindens* (proximal to another hammerhead motif); *Solibacter usitatus* (region of potential “phage, plasmid or transposon”).
(PDF)

Figure S7 Secondary structures of examples of inactive hammerhead-like RNAs. Core nucleotides that differ from the consensus are depicted in red. Except for the *Yarrowia lipolytica* (a different representative than the one shown in Figure 2), *Xanthomonas* phage and eggplant viroid examples, these examples are not found in a genetic context expected for hammerhead ribozymes (based on previously known hammerhead ribozymes and those presented in this paper). Furthermore, some examples diverged from the consensus at more than one position (*Xanthomonas*, *Renibacterium salmonirum*, *Faecalibacterium prauznitzii*, *Monodelphis domestica*, *Burkholderia ambifaria* and *Aedes aegypti*).
(PDF)

Figure S8 Repeat-type putative hammerhead ribozyme arrangement and dimeric conformation. Secondary structures of a putative single hammerhead ribozyme of *Aedes aegypti* (A) and hypothetical dimeric conformation (B). Yellow box highlights loop III that can be completely base paired in a dimeric ribozyme (to form a 10 bp long stem). (B) Blue nucleotides correspond to one ribozyme and black to the other ribozyme. Green nucleotides correspond to one of the most common variants observed. (C) Palindromic character of stem III and loop III sequences for several ribozymes. The vast majority of these sequences would allow base-pairing in a dimeric hammerhead conformation like it has previously been described for ASBVd and newt hammerhead ribozymes [71]. Yellow shading as in (A).
(PDF)

Figure S9 *C10orf118* expression analysis in human cell lines. (A) The *C10* ribozyme is located within the first intron of the *C10orf118* gene. The putative start codon of the protein is present at the beginning of the second exon. Several EST sequences have been identified within the first intron of this gene, as shown in the figure (short lines with accession numbers). (B) Expression pattern of EST sequences that map to the *C10orf118* gene. %EST designates the proportion of *C10orf118* EST sequences that are found in a tissue in comparison to all *C10orf118* EST sequences. EST sequences were found in Genbank and GeneCards databases [46], [47]. (C) Proportions of embryo and adult EST sequences that map to the *C10orf118* gene. (D) RT-PCR results showing expression of the *C10orf118* mRNA in the different cell types indicated. The PCR primers were designed to anneal on the first exon of the gene.
(PDF)

Table S1 Primers used in this study.
(PDF)

Text S1 RNAMotif descriptors for hammerhead ribozymes.
(DOC)

Dataset S1 Multiple sequence alignments of all hammerhead sequences.
(PDF)
