# Supplementary material for: Identification of Hammerhead Ribozymes in All Domains of Life Reveals Novel Structural Variations
Source: PLoS Comput Biol. 2011 May 5;7(5):e1002031. doi: 10.1371/journal.pcbi.1002031 (PMC3088659; doi:10.1371/journal.pcbi.1002031)

A *Clostridium scindens* (two hammerhead ribozymes)

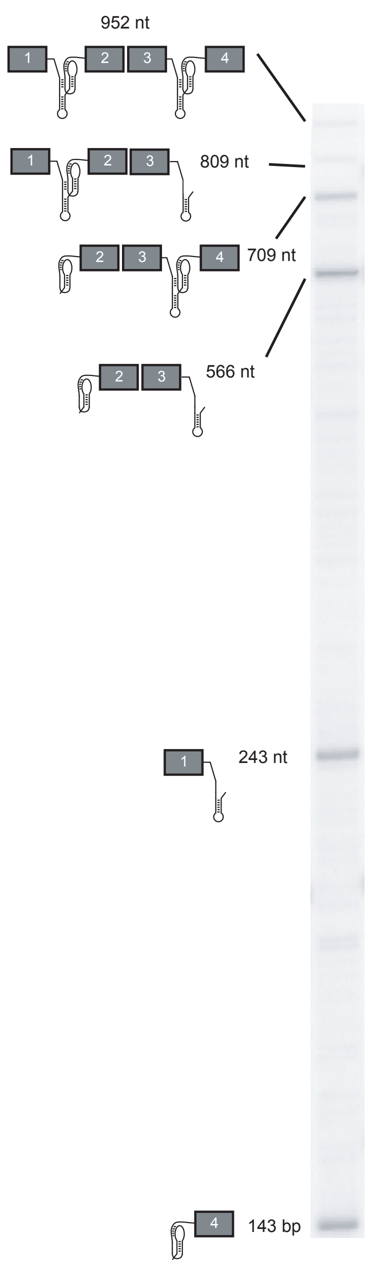

B *Azorhizobium caulinodans* (two hammerhead ribozymes)

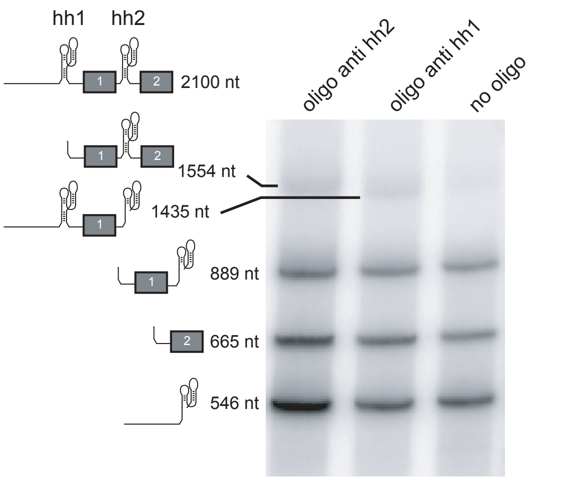

Supplement: Figure S1 — Activities of RNA transcripts carrying multiple hammerhead ribozymes. (A) Cleavage of internally radiolabeled RNA during in vitro transcription of PCR products from Clostridium scindens. Bands correspond to the expected sizes for hammerhead ribozyme cleavage (other size markers not shown also support indicated fragment sizes). (B) Cleavage of internally radiolabeled RNA during in vitro transcription of PCR products from Azorhizobium caulinodans. Full length Azorhizobium caulinodans RNA is not detectable, presumably because of efficient ribozyme cleavage. (PDF) [file pcbi.1002031.s001.pdf]
