# Supplementary figures and images for: Identification of Hammerhead Ribozymes in All Domains of Life Reveals Novel Structural Variations
Source: PLoS Comput Biol. 2011 May 5;7(5):e1002031. doi: 10.1371/journal.pcbi.1002031 (PMC3088659; doi:10.1371/journal.pcbi.1002031)

**A** *Agrobacterium tumefaciens*

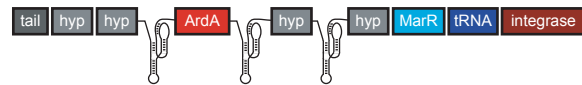

**B**

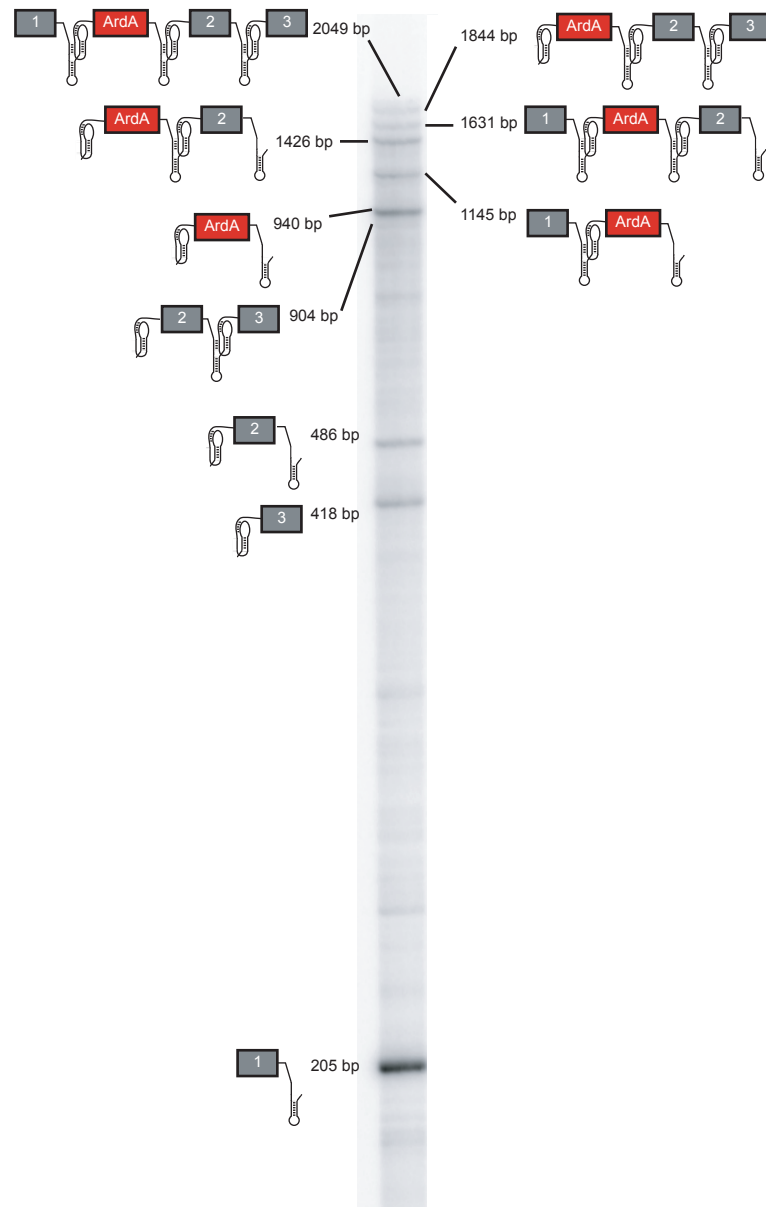

Supplement: Figure S2 — Agrobacterium tumefaciens multiple hammerhead ribozyme arrangement. (A) Genome context of ribozymes with annotations as follows: “tail” is a structural protein of the phage tail, “ardA” is an Anti-Restriction Defense protein, “marR” is a transcriptional regulation protein and “integrase” is a protein with predicted DNA integration activity. (B) Ribozyme cleavage during in vitro transcription using T7 RNA polymerase to produce internally-radiolabeled RNAs. Bands corresponding to expected sizes and compositions for hammerhead cleavage products are annotated. (PDF) [file pcbi.1002031.s002.pdf]

A Hammerhead mutant legend

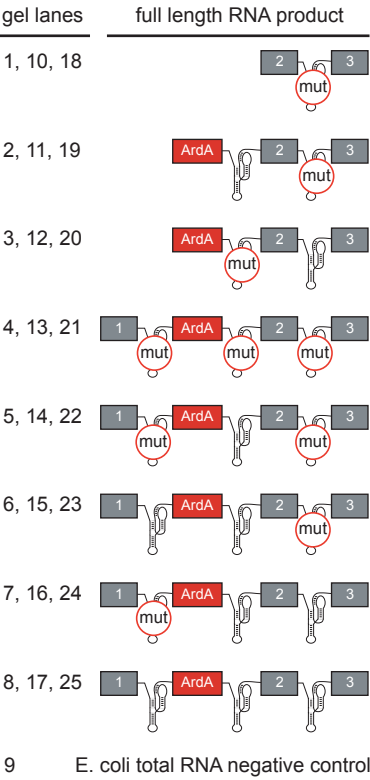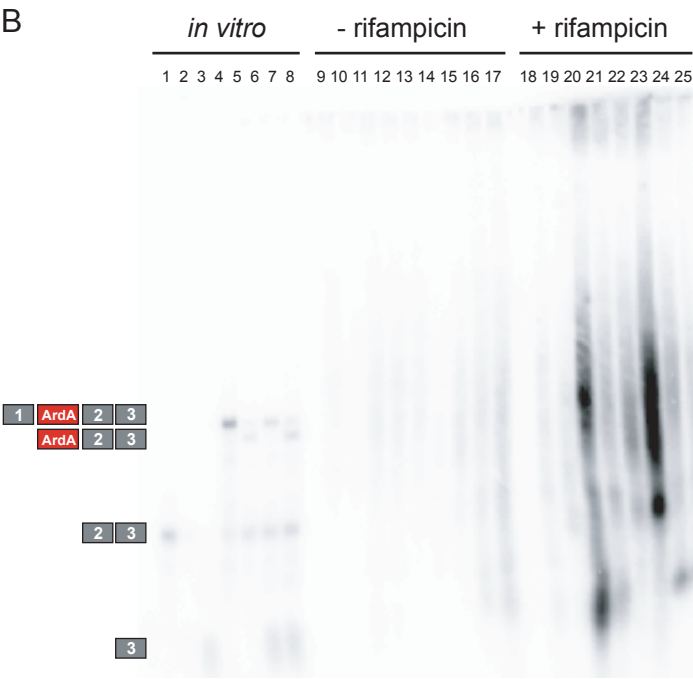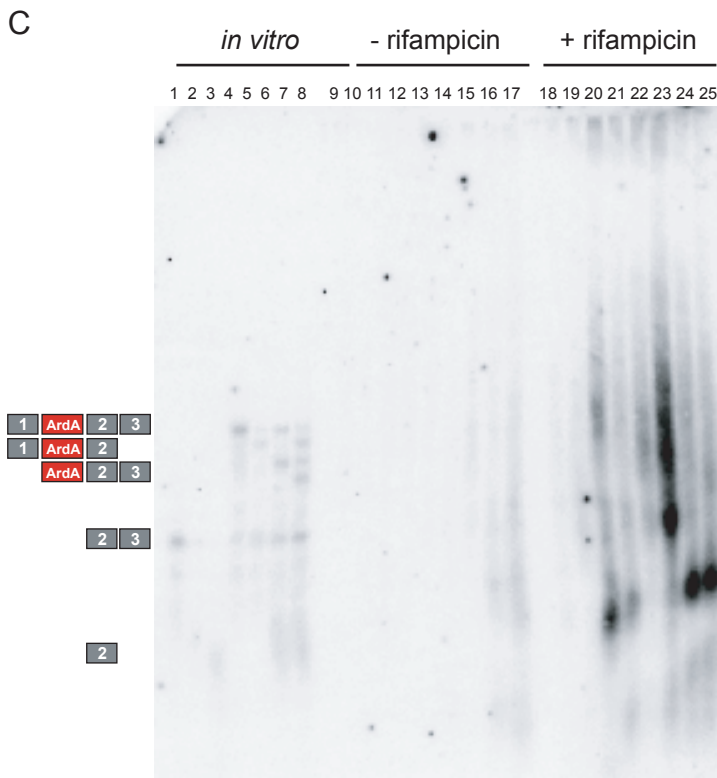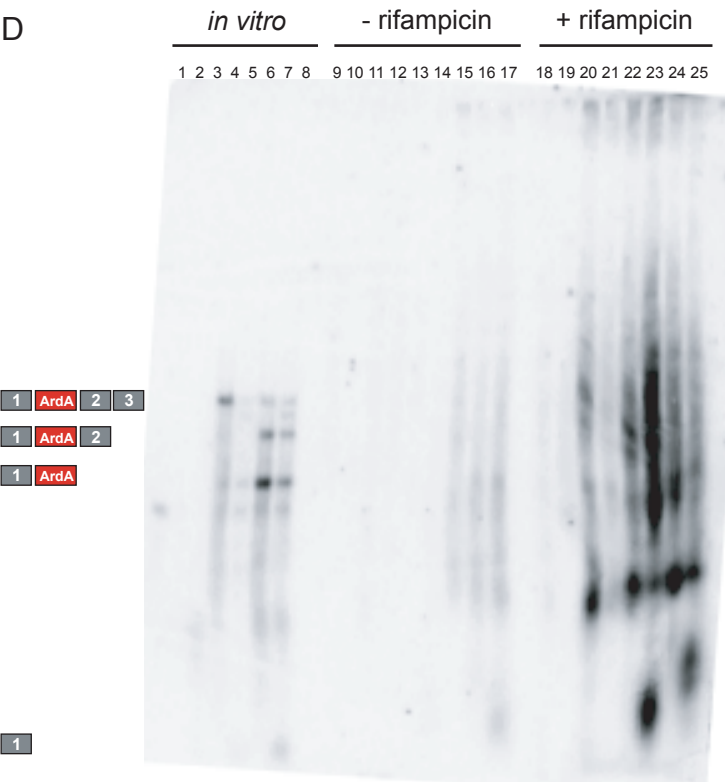

Supplement: Figure S3 — Agrobacterium tumefaciens multiple hammerhead ribozyme activity in vivo. (A) Various RNA constructs expressed from plasmids carrying portions of A. tumefaciens hammerheads and expressed in E. coli BL21 cells. Various deletion or mutant constructs are expressed and examined by Northern analysis in the indicated lanes on polyacrylamide gel electrophoresis in panels (B, C and D). (B, C, D) Northern analysis of RNA products express in E. coli BL21 cells from the plasmids (pUC19 with the lac promoter removed) depicted in A. Transcription by T7 RNA polymerase was induced by IPTG and E. coli RNA polymerase was inhibited by rifampicin where indicated to maximize the amount of T7 RNA polymerase transcripts. After 2 h IPTG induction, RNA was extracted with Trizol according to the manufacturer's instructions (in the presence of EDTA). The RNA products were separated by denaturing agarose gel electrophoresis and the resulting gel was used for blotting. Probes for ORF 3, 2 and 1 were successively used to generate the images presented in (B, C and D), respectively. Because probes could not be entirely washed off the membrane between different probing experiments, there is some carry over from the probing in B to C to D. This explains why some bands corresponding to “ORF 2” can be seen in panel D for example. (PDF) [file pcbi.1002031.s003.pdf]

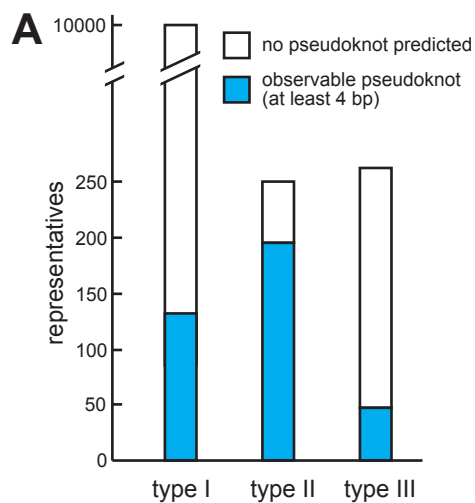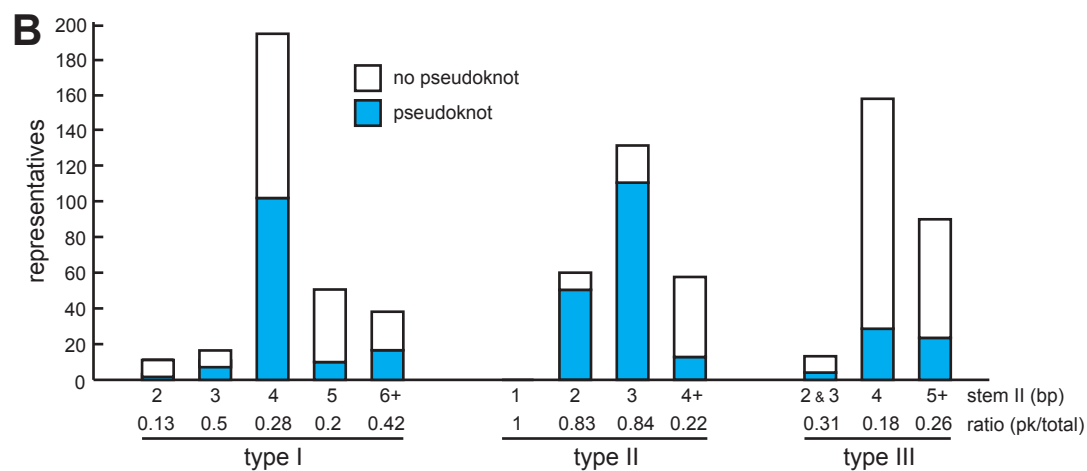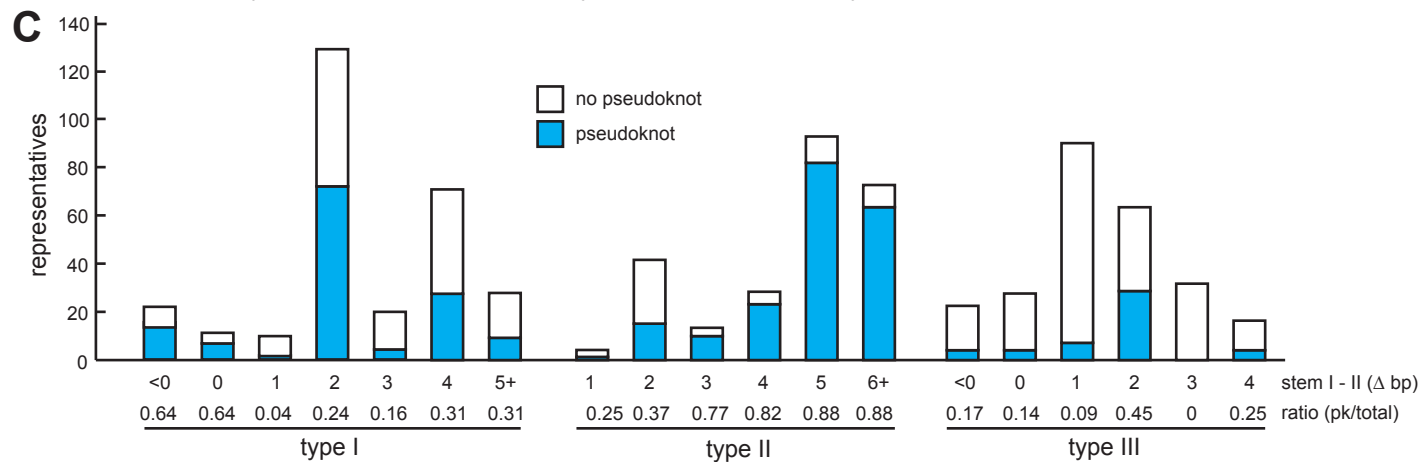

Supplement: Figure S4 — Pseudoknot interaction between stems I and II. (A) Proportion of each type of hammerhead ribozyme that is predicted to form a pseudoknot. (B) Proportion of hammerhead ribozymes with a pseudoknot versus the length of stem II. (C) Proportion of ribozymes with a pseudoknot versus the difference in length between stems I and II (stem II number of base pairs subtracted from stem I number of base pairs). The highly repetitive type I hammerhead representatives are excluded from the analyses in B and C. Notes: There is some bias in the distributions of pseudoknots with the various hammerhead types and stem lengths. For example, stem length difference disparities between different types of hammerheads is especially striking in type III hammerheads where a two base pair difference between stems I and II commonly are associated with a pseudoknot, while other length differences are not. However, our data indicate that the pseudoknot contact generally is a structurally versatile way to constrain the locations of stems I and II. (PDF) [file pcbi.1002031.s004.pdf]

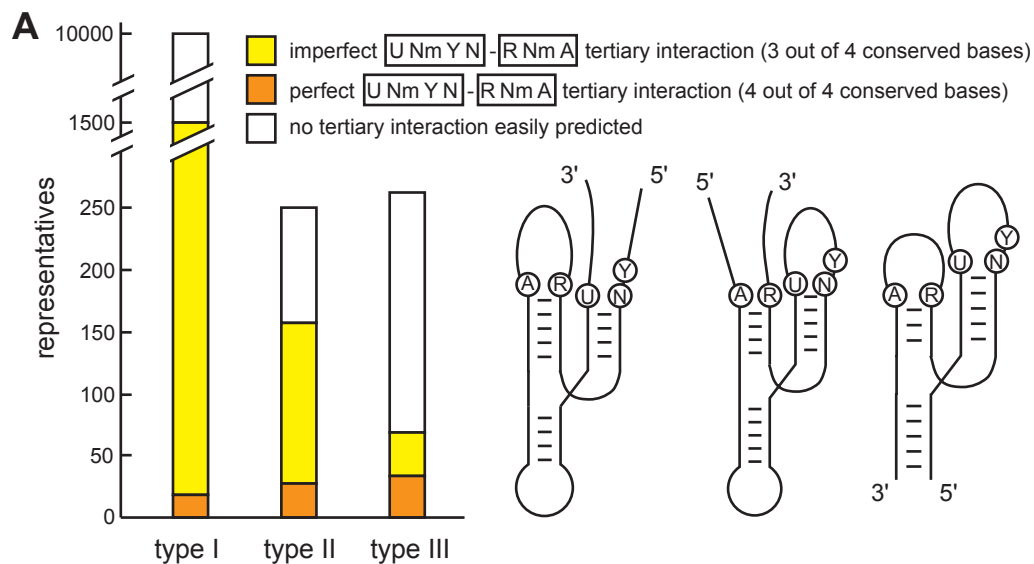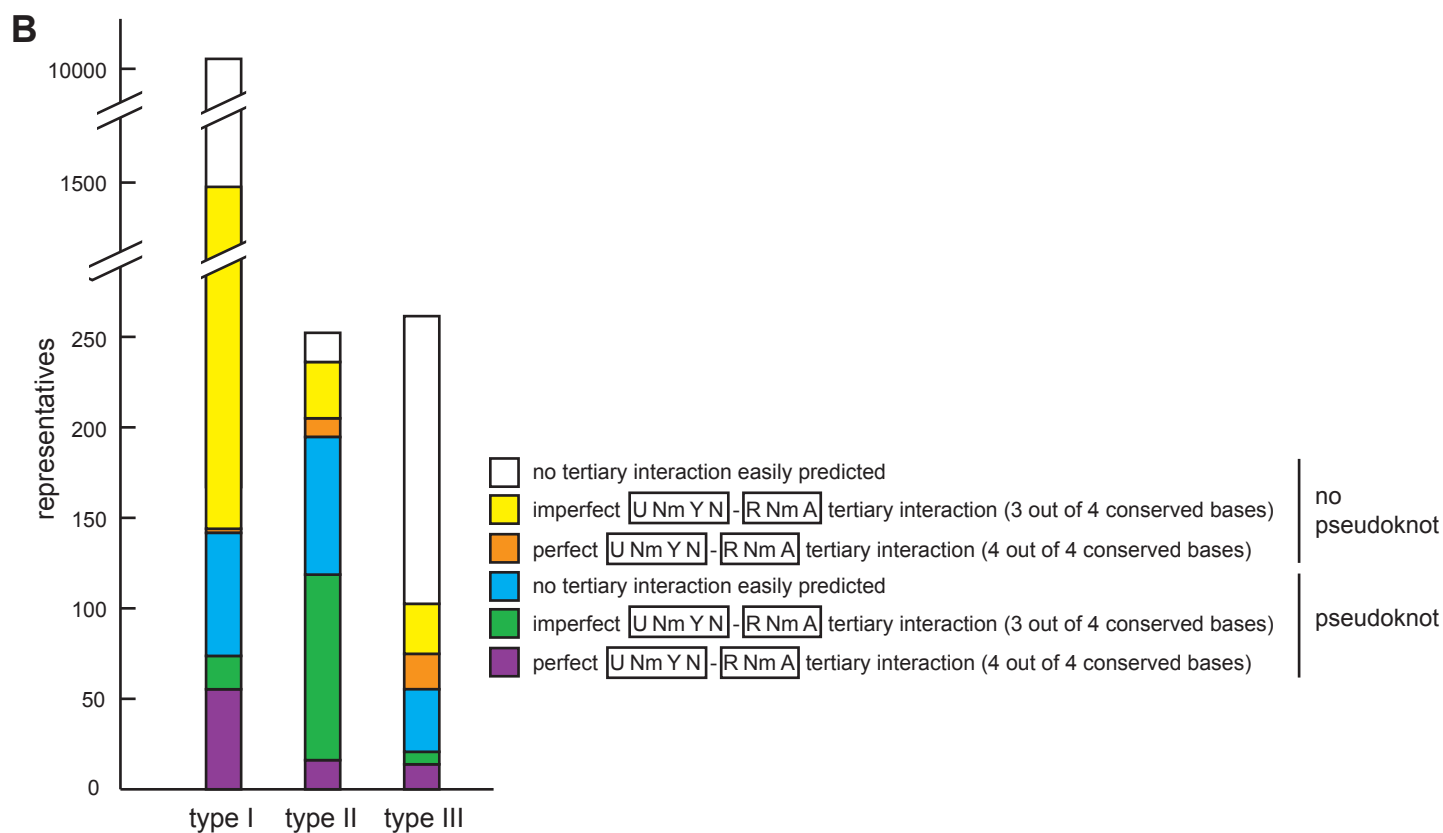

Supplement: Figure S5 — Tertiary interactions between stems I and II. (A) Proportion of each type of hammerhead ribozyme that has predicted tertiary interactions noted in the diagrams (terminal loops or internal bulges). Notations: U, uridine; Nm, m number of any nucleotides; Y, pyrimidine; N, any nucleotide; R, pyrimidine; A, adenosine. (B) Proportion of each type of hammerhead ribozyme that have the tertiary interactions depicted in A either with or without a pseudoknot. Note that even when no tertiary interactions are predicted, some unknown interaction or a variation of a known interaction could exist. (PDF) [file pcbi.1002031.s005.pdf]

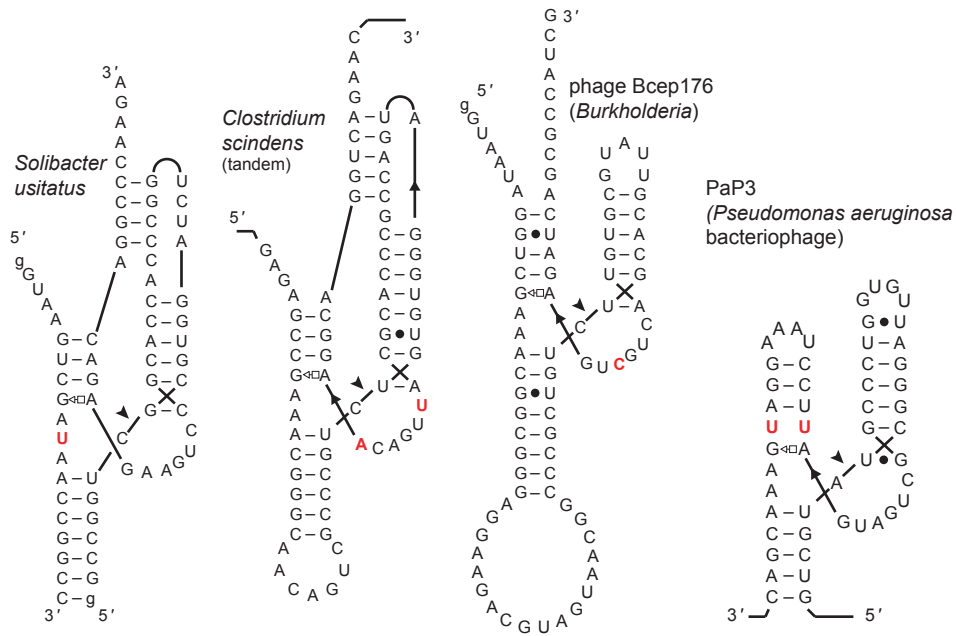

marine metagenome

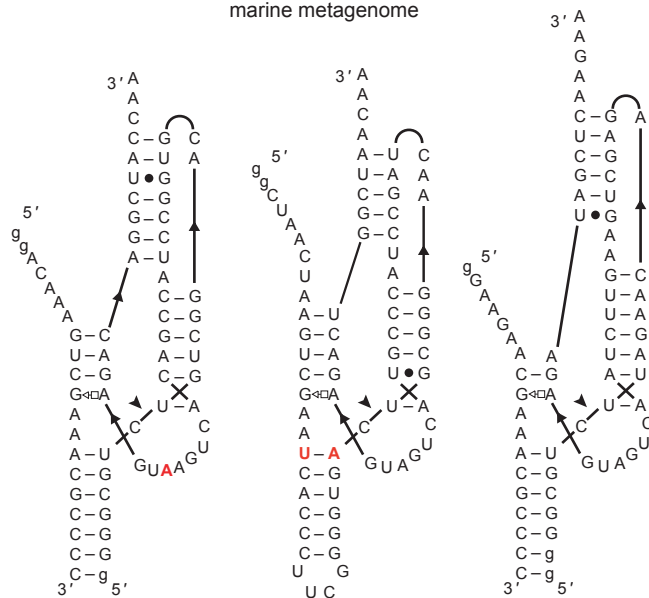

Supplement: Figure S6 — Complete sequences and secondary structure models of active hammerhead ribozymes that carry core variations. Positions diverging from the core consensus sequence are depicted in red. Variants were tested in trans or in cis as illustrated. Note that the RNA for the PaP3 bacteriophage and Clostridium scindens hammerhead ribozymes are part of larger RNAs transcribed from PCR products. Non-native guanosine residues (lowercase) were added to facilitate transcription in vitro. Gene context is as follows: PaP3 and Bcep176 (phage intergenic regions); Clostridium scindens (proximal to another hammerhead motif); Solibacter usitatus (region of potential “phage, plasmid or transposon”). (PDF) [file pcbi.1002031.s006.pdf]
