# Supplementary material for: Identification of Hammerhead Ribozymes in All Domains of Life Reveals Novel Structural Variations
Source: PLoS Comput Biol. 2011 May 5;7(5):e1002031. doi: 10.1371/journal.pcbi.1002031 (PMC3088659; doi:10.1371/journal.pcbi.1002031)
Supplement: Figure S7 — Secondary structures of examples of inactive hammerhead-like RNAs. Core nucleotides that differ from the consensus are depicted in red. Except for the Yarrowia lipolytica (a different representative than the one shown in Figure 2), Xanthomonas phage and eggplant viroid examples, these examples are not found in a genetic context expected for hammerhead ribozymes (based on previously known hammerhead ribozymes and those presented in this paper). Furthermore, some examples diverged from the consensus at more than one position (Xanthomonas, Renibacterium salmonirum, Faecalibacterium prauznitzii, Monodelphis domestica, Burkholderia ambifaria and Aedes aegypti). (PDF) [file pcbi.1002031.s007.pdf]

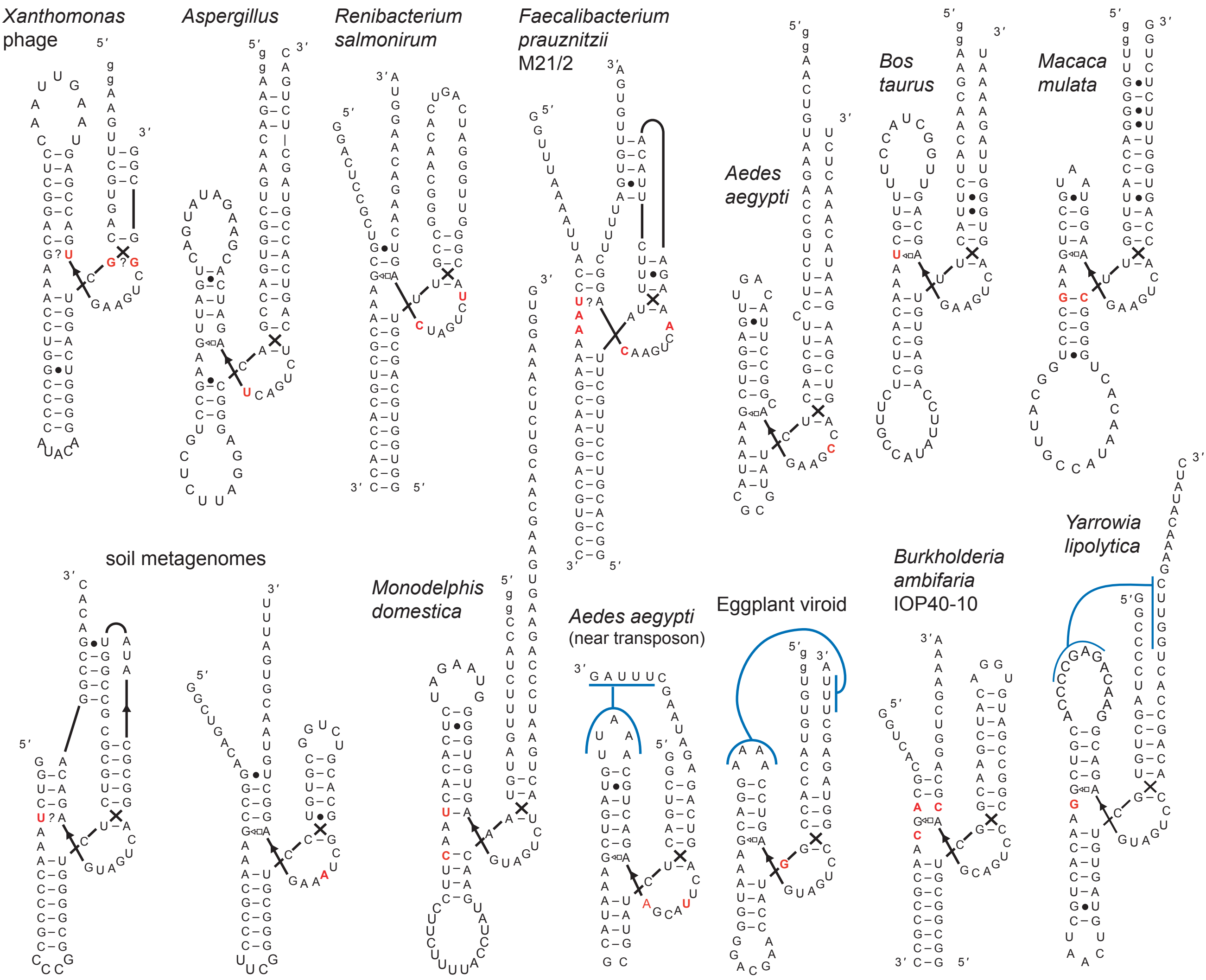
Xanthomonas phage
Aspergillus
Renibacterium salmonirum
Faecalibacterium prauznitzii M21/2
Aedes aegypti
Bos taurus
Macaca mulata
soil metagenomes
Monodelphis domestica
Aedes aegypti (near transposon)
Eggplant viroid
Burkholderia ambifaria IOP40-10
Yarrowia lipolytica
