# Supplementary material for: Identification of Hammerhead Ribozymes in All Domains of Life Reveals Novel Structural Variations
Source: PLoS Comput Biol. 2011 May 5;7(5):e1002031. doi: 10.1371/journal.pcbi.1002031 (PMC3088659; doi:10.1371/journal.pcbi.1002031)

A

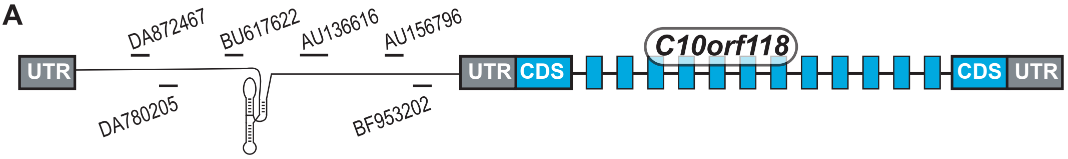


B

| TISSUE | % EST |
|---|---|
| Kidney | 14 |
| Lung | 14 |
| Placenta | 11 |
| Testis | 9 |
| Uterus | 8 |
| Brain | 6 |
| Liver | 6 |
| Muscle | 4 |
| Blood | 4 |
| Lymph Node | 3 |
| Mammary Gland | 3 |
| Bone Marrow | 3 |
| Prostate | 3 |
| Skin | 2 |
| Colon | 2 |
| Heart | 2 |
| Bladder | 1 |
| Eye | 1 |
| Pancreas/Stomach | 1 |

C

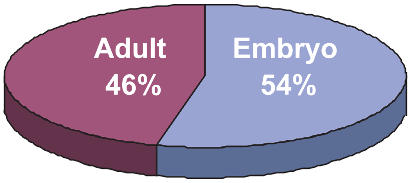


D

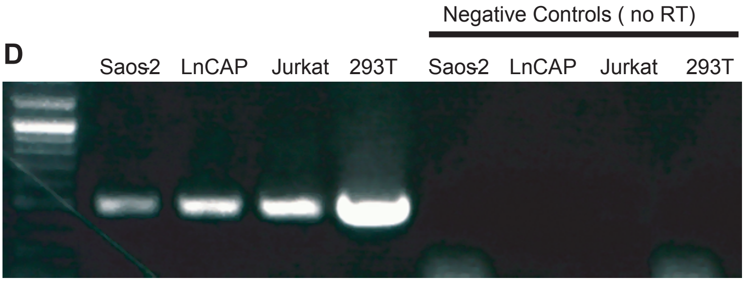

Supplement: Figure S9 — C10orf118 expression analysis in human cell lines. (A) The C10 ribozyme is located within the first intron of the C10orf118 gene. The putative start codon of the protein is present at the beginning of the second exon. Several EST sequences have been identified within the first intron of this gene, as shown in the figure (short lines with accession numbers). (B) Expression pattern of EST sequences that map to the C10orf118 gene. %EST designates the proportion of C10orf118 EST sequences that are found in a tissue in comparison to all C10orf118 EST sequences. EST sequences were found in Genbank and GeneCards databases [46], [47]. (C) Proportions of embryo and adult EST sequences that map to the C10orf118 gene. (D) RT-PCR results showing expression of the C10orf118 mRNA in the different cell types indicated. The PCR primers were designed to anneal on the first exon of the gene. (PDF) [file pcbi.1002031.s009.pdf]
