# Supplementary material for: Identification of Hammerhead Ribozymes in All Domains of Life Reveals Novel Structural Variations
Source: PLoS Comput Biol. 2011 May 5;7(5):e1002031. doi: 10.1371/journal.pcbi.1002031 (PMC3088659; doi:10.1371/journal.pcbi.1002031)
Supplement: Table S1 — Primers used in this study. (PDF) [file pcbi.1002031.s010.pdf]

Oligonucleotides

In the table below, each oligonucleotide is used, and is separated by a horizontal line. The sequence is listed on the last line of text for each oligonucleotide, and is right justified. “HH” is an abbreviation for “hammerhead”; A. tumefaciens refers to *Agrobacterium tumefaciens* str. C58 (also called 1303); A. caulinodans refers to *Azhorhizobium caulinodans* ORS571.

**name**	template	template (detail)	Forward/Rev	sequence
**JP_077_HH_Atu-a-F**	A. tumefaciens	tandem HH and flanking sequence in what is presumably a prophage	For	TAATACGACTCACTATAGGCCGCAGCTATCGGCACG
**JP_078_HH_Atu-b-F**	A. tumefaciens	tandem HH and flanking sequence in what is presumably a prophage	For	TAATACGACTCACTATAGGCAGTCACTCAACACATTC
**JP_079_HH_Atu-c-F**	A. tumefaciens	tandem HH and flanking sequence in what is presumably a prophage	For	TAATACGACTCACTATAGGAAGAGCTGGGAAACAAC
**JP_080_HH_Atu-a-R**	A. tumefaciens	tandem HH and flanking sequence in what is presumably a prophage	Rev	CCCATACCGGGCCAACATG
**JP_081_HH_Atu-b-R**	A. tumefaciens	tandem HH and flanking sequence in what is presumably a prophage	Rev	GACCGGAGGCCAGCCGAAG
**JP_082_HH_Atu-c-R**	A. tumefaciens	tandem HH and flanking sequence in what is presumably a prophage	Rev	ACTCGCATCGATCCACCGACC
**JP_085_HH_Atu-d-R**	A. tumefaciens	tandem HH and flanking sequence in what is presumably a prophage	Rev	AGGCCGTGTTGTCCAGCCC
**JP_088_antiHH1-1**	pairs with HH1 cugauga from A. tumefaciens		Rev	CTATTCCGCTAGCTCATCAGGCCCTAGGCGCGACCCTAGGAC
**JP_089_antiHH1-2**	pairs with HH2 cugauga from A. tumefaciens		Rev	TTTCGTGCGCTGCTCATCAGGCCCTAGGCGCGACCCTAGGAC
**JP_090_antiHH1-3**	pairs with HH3 cugauga from A. tumefaciens		Rev	GGGAATGGCAACCATCATCAGGCGGGGCATGCCAAGCCCCAC
**JP_091_HH1_mutant-R**	HH1 cugauga–>cuAauga from A. tumefaciens		Rev	CTATTCCGCTAGCTCATTAGGCCCTAGGCGCGACC
**JP_092_HH2_mutant-R**	HH2 cugauga–>cuAauga from A. tumefaciens		Rev	TTTCGTGCGCTGCTCATTAGGCCCTAGGCGCGACC
**JP_093_HH3_mutant-R**	HH3 cugauga–>cuAauga from A. tumefaciens		Rev	GGAATGGCAACCATCATTAGGCGGGGCATGCCAAG
**JP_094_HH1_mutant-F**	HH1 cugauga–>cuAauga from A. tumefaciens		For	GGTCGCGCCTAGGGCCTAATGAGCTAGCGGAATAG

**JP_095_HH2_mutant-F**	HH2 cugauga–>cuAauga from A. tumefaciens		For	GGTCGCGCCTAGGGCCTAATGAGCAGCGCACGAAA
**JP_096_HH3_mutant-F**	HH3 cugauga–>cuAauga from A. tumefaciens		For	CTTGGCATGCCCCGCCTAATGATGGTTGCCATTCC
**JP_097_HH1-2_small-F**	shorten 3HH from A. tumefaciens (940->384)		For	GAGAGATGGTCGACCTAGATCAATCCACATGTGGAAAG
**JP_098_HH1-2_small-R**	shorten 3HH from A. tumefaciens (940->384)		Rev	TTGATCTAGGTCGACCATCTCTCACTCCTTCGCTATCG
**JP_099_HH_Atu-C1-R**	A. tumefaciens	tandem HH and flanking sequence in what is presumably a prophage(to probe first fragment, before HH1)	Rev	CACCAGCCAGACCGTTGAACCGCTGGCC
**JP_109_HH_Aca-a-F**	A. caulinodans	prophage (near tandem HH)	For	taatacgactcactataggAACGGTGCAGTGACTGCAC
**JP_110_HH_Aca-a-R**	A. caulinodans	prophage (near tandem HH)	Rev	gcctcgggagagcgatcagcgttggcg
**JP_111_HH_Aca-b-R**	A. caulinodans	prophage (near tandem HH)	Rev	gggcctcgacttctccccggagacgttcg
**JP_112_HH_Aca-c-R**	A. caulinodans	prophage (near tandem HH)	Rev	caacctcttcctcgatcaccgccaccagatc
**JP_113_HH_Aca-c2-R**	A. caulinodans	prophage (near tandem HH)	Rev	caggggcgcggcgtctggcctcatcctcgg
**JP_123_HH_Aca-a2-F**	A. caulinodans	prophage (near tandem HH)	For	CGGTCACAGTCCCTCGCTA
**JP_124_HH_Aca-c3-R**	A. caulinodans	prophage (middle tandem HH)	Rev	GATACCCCTCGCGCCA
**JP_125_HH_Aca-b-F**	A. caulinodans	prophage (middle tandem HH)	For	GCTTGCGGCCAACTGGTCCGCG
**JP_126_HH_PaP3-R**		entire HH		CATGGTCGTTTCATCCTTTAGGAATCATCAGCCGGGACACACATCCCGATACGACAAccTATAGTGAGTCGTATTA
**JP_127_HH_Aca-a2-F-T7**	PCR from A. caulinodans prophage			TAATACGACTCACTATAggCGGTCACAGTCCCTCGCTA
**JP_128_HH_Atu_HH1mut-gaaa-F**	HH1 gaaa –> gCaa from A. caulinodans		For	CATCGCATAACTAAGCGcAACCGGGGGGCGTCCT
**JP_129_HH_Atu_HH2mut-gaaa-F**	HH2 gaaa –> gCaa from A. caulinodans		For	CATGTGGAAAGTAATGCGcAACCGGGTGTCGCTTC
**JP_130_HH_Atu_HH3mut-gaaa-F**	HH3 gaaa –> gCaa from A. caulinodans		For	CAATCCAACTGATACCGcAACCGGCTCCCTAGGG
**JP_131_HH_Atu_HH1mut-gaaa-R**	HH1 gaaa –> gCaa from A. caulinodans		Rev	AGGACGCCCCCCGGTTgCGCTTAGTTATGCGATG
**JP_132_HH_Atu_HH2mut-gaaa-R**	HH2 gaaa –> gCaa from A. caulinodans		Rev	GAAGCGACACCCGGTTgCGCATTACTTTCCACATG
**JP_133_HH_Atu_HH3mut-gaaa-R**	HH3 gaaa –> gCaa from A. caulinodans		Rev	CCCTAGGGAGCCGGTTgGGGTATCAGTTGGATTG

**JP_134_HH_Aca_HH1good-F**	A. caulinodans		For	GCCCGCGAGGGCCGAAACGATAGAGGAGCTAG
**JP_135_HH_Aca_HH1good-F**	A. caulinodans		Rev	CTAGCTCCTCTATCGTTTCGGCCCTCGCGGGC
**JP_136_HH_Aca_anti-HH1**	pairs with HH1 cugauga from A. tumefaciens	Rev		CTCGCGGGCCTCGTCAGGCCGGGACCCTCCCCG
**JP_137_HH_Aca_anti-HH2**	pairs with HH2 cugauga from A. tumefaciens	Rev		CTGCCATGGGCTCATCAGTGGACCGGCATCAGGTC
**JP_138_HH_Atu_HH1-and-2_3'fragment**	A. tumefaciens	Tandem HH and flanking sequence in what is presumably a prophage	For	TAATACGACTCACTATAgGTCCTAGGGTCGCGCC
**JP_139_HH_Atu_HH3_3'fragment**	A. tumefaciens		For	TAATACGACTCACTATAgGTGGGGCTTGGCATG
**JP_140_T7-term-F**	T7 terminator for annealing		For	GctagcataacccсttggggcctctaaacgggtcttgaggggttttttgA
**JP_141_T7-term-R**	T7 terminator for annealing		Rev	catgTCAAAAAACCCCTCAAGACCCGTTTAGAGGCCCCAAGGGGTTATGCTAGCtgca
**JP_142_T7-term-screen-F**	clones with T7-term		For	gctaGCATAACCCCTTGG
**JP_144_pUC19(836-821)**	pUC19(836-821)		Rev	CTGGCCTTTTGCTGGC
**JP_147_T7-term-screen-R**	clones with T7-term		Rev	AACCCCTCAAGACCCGTT
**JP_148_rz-JCVI_SCAF_1101668476676/866**	elongation		Rev	ggGGCGTTTCGACTTTGTCCTATAGTGAGTCGTATTA
**JP_149_rz-NC_008536.1/7550346**	elongation		Rev	gGCCGGTTATCGACTTACCTATAGTGAGTCGTATTA
**JP_150_rz-NC_010168.1/3000116**	elongation		Rev	gGTGGTGCAGCGTTTCGCAGCGGAGTCCTATAGTGAGTCGTATTA
**JP_151_rz-NZ_ABED02000017.1/64984**	elongation		Rev	gGCACGTCCTTGCTTTTTAGGTAATTTTAAACCTATAGTGAGTCGTATTA
**JP_152_rz-NZ_ABFY02000014.1/30371**	elongation		Rev	gGCCCGTTTCGGCTCTCCTATAGTGAGTCGTATTA
**JP_153_rz-NZ_ABLC01000053.1/26585**	elongation		Rev	gGCGGCGTTGCTGCGTGACCTATAGTGAGTCGTATTA
**JP_154_rz-NC_007497.1/12160**	elongation		Rev	ggCGCCGTTTCGACCTATTACCTATAGTGAGTCGTATTA
**JP_155_rz-JCVI_SCAF_1096626868593/854**	elongation		Rev	ggGGCGTTTCGTTCTTCCTATAGTGAGTCGTATTA
**JP_156_s-JCVI_SCAF_1101668476676/866**	elongation		Rev	TTGGTAGCCTGTCTCATTCAGTGTCGGTAGGCCACGTCCGACAGACGCCCCTATAGTGAGTCGTATTA
**JP_157_s-NC_008536.1/7550346**	elongation		Rev	TCTTGGGCCTGTCTCTTCAGGCGTGGTGGGCCAGATCCACGCGACCGGCCTATAGTGAGTCGTATTA
**JP_158_s-NC_010168.1/3000116**	elongation		Rev	TACCTTGTCTTGACTGATCAGATCGGCCCGTTGTGACTGATCCCAACCCGAAACGCTGCACCACcTATAGTGAGTCGTATTA
**JP_159_s-NZ_ABED02000017.1/64984**	elongation		Rev	

				TCACAACACTAAAAGCCTGTTCAGTTAAAGAATGTTCTATAAGCAAGGACGTGCcTATAGTGAGTCGTATTA
**JP_160_s-NZ_ABFY02000014.1/30371**	elongation		Rev	GTTCTGACCTGCCTTGTCAATGCGTGGGCGGTCATCCCACACAGACGGGCCTATAGTGAGTCGTATTA
**JP_161_s-NZ_ABLC01000053.1/26585**	elongation		Rev	TTTTCGACCTGCGTCGTCAGGCGTTCGATGTCCACATCGGCCGCGACGCCGCCTATAGTGAGTCGTATTA
**JP_162_s-NC_007497.1/12160**	elongation		Rev	CGATGGCGCTGATCTCAGCAGTACACGCATAACGTGCAGACAGCGCCTATAGTGAGTCGTATTA
**JP_163_s-JCVI_SCAF_1096626868593/854**	elongation		Rev	TTCTTGAGCTATCTCATCAGTTAGAACTTCAGCTCTGTTCTAAGACGCCCCTATAGTGAGTCGTATTA
**JP_164_Azo-other-phage-F**	Azorhizobium caulinodans	prophage head gene (2467000-2467500)	For	GCGCTCTACTGGAACCTCTG
**JP_165_Azo-other-phage-R**	Azorhizobium caulinodans	prophage head gene (2467000-2467500)	Rev	GCTGGTCTTGGCGTCCTC
**JP_166_Bce-HH-F**	Bacillus cereus ATCC 14579	>gi|30018278:2631880-2634325	For	TAAAACGATTAATTCCTTTGGTTC
**JP_167_Bce-HH-R**	Bacillus cereus ATCC 14579	>gi|30018278:2631880-2634325	Rev	ATCTTTCAGCCCCCCTAAA
**JP_168_Bce-HH-F**	Bacillus cereus ATCC 14579	>gi|30018278:2631880-2634325	For	TAATACGACTCACTATAggTAAAACGATTAATTCCTTTGG
**JP_178_rz8-mut_C->U**	use with substrate JP_163_s-JCVI_SCAF_1096626868593/854 (or other)		Rev	ggGGCGTTTCATTCTTCCTATAGTGAGTCGTATTA
**JP_179_rz8-mut_C->G**	use with substrate JP_163_s-JCVI_SCAF_1096626868593/854 (or other)		Rev	ggGGCGTTTCCTTCTTCCTATAGTGAGTCGTATTA
**JP_180_rz8-mut_+U-A_bp**	use with substrate JP_163_s-JCVI_SCAF_1096626868593/854 (or other)		Rev	ggGGCGTTTCGATCTTCCTATAGTGAGTCGTATTA
**JP_181_s8-mut__pk-4**	use with Rz JP_155_rz-JCVI_SCAF_1096626868593/854 (or other)		Rev	TATCTCATCAGTTAGAACTTCAGCTCTGTTCTAAGACGCCCCTATAGTGAGTCGTATTA
**JP_182_s8-mut_-extra**	use with Rz JP_155_rz-JCVI_SCAF_1096626868593/854 (or other)		Rev	GAGCTATCTCATCAGTTAGAACTTCAGCTCTGTTCTAAGACGCCCCTATAGTGAGTCGTATTA
**JP_183_s8-mut_pk-2**	use with Rz JP_155_rz-JCVI_SCAF_1096626868593/854 (or other)		Rev	GCTATCTCATCAGTTAGAACTTCAGCTCTGTTCTAAGACGCCCCTATAGTGAGTCGTATTA
**JP_184_Bce-HH-T7-R**	Bacillus cereus ATCC 14579	>gi|30018278:2631880-2634325	Rev	TAATACGACTCACTATAggATCTTTCAGCCCCCCTAAA
**JP_185_s8-mut_spacer+1**	use with Rz JP_155_rz-JCVI_SCAF_1096626868593/854 (or other, like JP_180 to see P2=2)		Rev	GAGCTATTCTCATCAGTTAGAACTTCAGCTCTGTTCTAAGACGCCCCTATAGTGAGTCGTATTA
**JP_186_s8-mut_P1+2bp**	use with Rz JP_155_rz-JCVI_SCAF_1096626868593/854 (or other, like JP_180 to see P2=2)		Rev	GAGCTATCTCATCAGTTAGAAC gg TT CAGCTC T cc GTTCTAAGACGCCCCTATAGTGAGTCGTATTA

**JP_187_s8-mut_L1-5'-1**	use with Rz JP_155_rz-JCVI_SCAF_1096626868593/854 (or other, like JP_180 to see P2=2)		Rev	GAGCTA TCTCATCAGTTAGAAC TT CAGCTC GTTCTAAGACGCCCCTATAGTGAGTCGTATTA
**JP_188_s8-mut_L1-3'-2**	use with Rz JP_155_rz-JCVI_SCAF_1096626868593/854 (or other, like JP_180 to see P2=2)		Rev	GAGCTA TCTCATCAGTTAGAAC CAGCTC T GTTCTAAGACGCCCCTATAGTGAGTCGTATTA
**JP_189_s8-mut_spacer-1**	use with Rz JP_155_rz-JCVI_SCAF_1096626868593/854 (or other)		Rev	GAGCTACTCATCAGTTAGAACTTCAGCTCTGTTCTAAGACGCCCCTATAGTGAGTCGTATTA
**JP_190_s8-mut_pk-6**	use with Rz JP_155_rz-JCVI_SCAF_1096626868593/854 (or other)		Rev	TCTCATCAGTTAGAACTTCAGCTCTGTTCTAAGACGCCCCTATAGTGAGTCGTATTA
**JP_191_HH_Bcl-F**	Bacillus clausii	CDS of spore germination protein KC (gerKC)	For	AGGAGGGAGATGATTATGTGTTG
**JP_192_HH_Bcl-R**	Bacillus clausii	CDS of spore germination protein KC (gerKC)	Rev	GGATCATTTTCCGTAACGATTC
**JP_193_HH_Bcl-T7-F**	Bacillus clausii	CDS of spore germination protein KC (gerKC)	For	TAATACGACTCACTATAGGAGGGAGATGATTATGTGTTG
**JP_194_PaP3-R**	PaP3 phage	potential type3 HH at end of genome	Rev	GACGCAGCTTTTTGTCAAGA
**JP_195_PaP3-F**	PaP3 phage	potential type3 HH at end of genome	For	TACGCCAATGCCAAGGTT
**JP_196_PaP3-T7-F**	PaP3 phage	potential type3 HH at end of genome	For	TAATACGACTCACTATAGGTACGCCAATGCCAAGGTT
**JP_199_Csc_2HH-F**	Clostridium scindens	tandem HH	For	GGTACGACGAGCATCTTGAA
**JP_200_Csc_2HH-T7-F**	Clostridium scindens	tandem HH	For	TAATACGACTCACTATAGGTACGACGAGCATCTTGAA
**JP_201_Csc_2HH-R**	Clostridium scindens	tandem HH	Rev	TGCCTGTATGTCTTTTCCGG
**JP_202_Bcep176-(7)cis-HH-R**	elongation		Rev	TGGCGCTGATCTCAGCAGTACACGCATAACGTGCAGACAGCGGGCCGTTACTACGTCTTCCTCCCGCCGTTTCGACCTATTCCTATAGTGAGTCGTATTA
**JP_209_AgroH1-Rz-R**	elongation		Rev	GGCCCGGTTTCGCTTAGTTATGCCTATAGTGAGTCGTATTA
**JP_210_AgroH1-subs-F**	elongation		For	TAATACGACTCACTATAGGCTCGGTCGTCCTAGGGTCGCGCCTAG
**JP_222_Rz?-F**	elongation		For	TAATACGACTCACTATAgGtCAtCtCtGAtGACCACCAAtGAtCtAtCAG
**JP_223_Rz?-R**	elongation		Rev	GATTCAGTCACCCGAAGGCGATCTTCACAGACTGATAGATCATTGGTGGT
**JP_233_Csc2_2HH-F**	Clostridium scindens	tandem HH	For	

				GGGAACGGGTTCTCC
**JP_234_Csc2_2HH-T7-F**	Clostridium scindens	tandem HH	For	TAATACGACTCACTATAGGGAACGGGTTCTCC
**JP_235_Csc2_2HH-R**	Clostridium scindens	tandem HH	Rev	GGAGTTTCCCTTGCTGC
**JP_236_16S(large)-F**	16S rRNA from bacteria		For	agagtttgatcctggctcag
**JP_237_16S(large)-R**	16S rRNA from bacteria		Rev	ggytaccttgttacgactt
**JP_238_Csc-16S-F**	Clostridium scindens	16S rRNA	For	gcaacctgccttgcact
**JP_239_Csc-16S-R**	Clostridium scindens	16S rRNA	Rev	accgaatggccttgcca
**JP_240_Atu-HH1-substr-R**	Agrobacterium	3HH region	Rev	GGCCACCCGCCTTA
**JP_245_Csc-between-2HH-R**	Clostridium scindens	sequence between both HH in phage	Rev	CGCCTCTGTTCCGGCTGAATTTACCG
**JP_246_Csc-before-2HH-R**	Clostridium scindens	sequence before first HH in phage	Rev	CTGCTGCCCCGTTTCGGCTTTTA
**JP_254_Atu-HH1-T7-F**	Agrobacterium	first HH of 3HHs	For	TAATACGACTCACTATAggCTCGGTCGTCCTAGGGTC
**JP_260_Rz_JCVI_scaf_1096626965523**	elongation		Rev	GGCAGTGAGTGAGTTTCGCCTATAGTGAGTCGTATTA
**JP_261_S_JCVI_scaf_1096626965523**	elongation		Rev	AATGACCTGGCTCTTCAGGGCAGGGAGGTCATCCCACTGCCGACTCACTCACTGCCTATAGTGAGTCGTATTA
**JP_266_t3-pk13**	elongation		Rev	GCGTGTTTCGTCCCCTCTGGACCCTACTCGTCAGGGCCACGGACGAGGTCCAGAAGGGGCCGTGGCCGACACGCCTATAGTGAGTCGTATTA
**JP_267_t3-weak**	elongation		Rev	TTCCCTTTCGTGGTAACTCATCAGGGCAGACTACCAATGCCTGCCGAGGGAAccTATAGTGAGTCGTATTA
**JP_268_t3-Droso**	elongation		Rev	TAGACTGTTTCGGAAGACACCACTCTTCCTCATCAGCGATCTTTTCTTTTTCAATAATGAAAAAGATGGTAAATCGGACAGCCTAccTATAGTGAGTCGTATTA
**JP_269_t1-mouse-gut-weak**	elongation		Rev	TTATGGTACTATCCCGACTACCCTTGTTTGGGTAGTTTCGCCGGCTCTCACCGTTGCTCATCAGGGGATTTATTCGGTAccTATAGTGAGTCGTATTA
**JP_270-t1-Cenarchaeum**	elongation		Rev	AGCAATAAATGCCACAGACACGGCACCGGTGGTCTGGCGCCGTGTTTCGTCCACATAGGACTCGTCAGTATGGCTGCCGGGATAccTATAGTGAGTCGTATTA
**JP_271_t1-Saltern**	elongation		Rev	TGCGGTGGCTACCGCGACGGGGCACGAGGCCCCGTTTCGCCCGCGGCCAAGCGGGCTCGTCAGGCGGCTATGAGGTGAccTATAGTGAGTCGTATTA
**JP_272_t1-Yarrowia**	elongation		Rev	TTATGACTAGTGGCTGCCGACACCAACAGCCCCCACTGTTGATGTTTCGACGCTAGTCATGGGTTTGTTCTCATCAGGGCAGCCAGTCCCCCTATAGTGAGTCGTATTA
**JP_273_t2-UAAA**	elongation		Rev	GTGTCGGCCTGTCTCATCAGTGACGCGCGGCCATATGCGCCAGACCCCGCCGGGGCGGGGTTTAGACcTATAGTGAGTCGTATTA
**JP_274_t2-CUAAAGA**	elongation		Rev	AAAATCACGTTACAGCCTCTTTAGCACACGCCAGACGTGCGGACGCCCCGAAGGGCGTTTCGGCCTGTCAGCcTATAGTGAGTCGTATTA
**JP_275_s8-mut_pk-swap**	use with Rz JP_155_rz-JCVI_SCAF_1096626868593/854 (or other)		Rev	GtcgaATCTCATCAGTTAGAACTTCAGCTCTGTTCTAAGACGCCCCTATAGTGAGTCGTATTA

**JP_276_s8-mut_pk-comp**	use with Rz JP_155_rz-JCVI_SCAF_1096626868593/854 (or other)		Rev	GtcgaATCTCATCAGTTAGAACTTCtcgaCTGTTCTAAGACGCCCCTATAGTGAGTCGTATTA
**JP_277_t1-X.phage(cugaagU)-R**	elongation	NC_007024.1/490-419	Rev	CCGCCGACCTGACCCCTGTATGGGGCCAGGTTTCGTCCGAGGTTAACTTACTCGGTCACTTCAGCGTCACGAACTTCCTATAGTGAGTCGTATTA
**JP_278_t1-Aedes(cuUacgaa)-R**	elongation	NW_001810328.1/53176-53230	Rev	CTAAATGCTTATCTCTGACAGATACGCGTATTTCGACTACAATTTGCAGTCTTCGTAAGTGTCAGCCTATAGTGAGTCGTATTA
**JP_279_t1-Aspergi(cugacUa-gaaG)-R**	elongation	NT_107005.1/1253669-1253736	Rev	GTCAGAGCTACGGTGACTGTGGCCCTCCTTAAGAGCAGGCTTCAATCAGTCATATCTTCGTGATCTAGTCAGACGGTCACCGACTTGTCTTCCTATAGTGAGTCGTATTA
**JP_280_t1-Ep.viroid(-G-)-R**	elongation	NC_004728.1/11-64	Rev	TAAAGCTCTACCCCATGGCAGAACCCCATTTCGTCCTTTTGGACTCATCAGGGGTGGTACACACCTATAGTGAGTCGTATTA
**JP_281_t1-Aedes(cCgauga)-R**	elongation	NZ_AAGE02023894.1/13988-14043	Rev	AGAGTTTGTATTCTTCGACAGATACGCGTATTTCGACCTCAACTGTAAGGCCGTCTTCGGTGTCGGAAGAAGACGGTCTTACAGTTCcTATAGTGAGTCGTATTA
**JP_282_t1-Bos(Uaaa)-R**	elongation	NW_001494989.2/1604723-1604611	Rev	ATTTTCTAACCCACAAACACTCTGGAATATGGCAAGAGAGTGTTTAGCAAAAGGTAGCCAACTGCTCTTCAGTGTAAGATGTTGCTTCcTATAGTGAGTCGTATTA
**JP_283_t1-Monodelphis(UaaC)-R**	elongation	NW_001581879.1/85484827-85484696	Rev	CACCTTTGAGACGTTGCTTCACTTCTGGGATTCAGTTTCTTCATAGG
**JP_284_t1-Macaca(gaaG)-R**	elongation	NW_001124105.1/14934-15038	Rev	CCAGAGAAACCACTGGAAGCCCCAGTGTTATGGCAATGCCAGGGCTTCAGGCATTACCTTCTTCAGTCCAATGGTCCCCAACCTATAGTGAGTCGTATTA
**JP_285_t1-Yarrowia(gGaa)-R**	elongation	NC_006068.1/1043901-1044007	Rev	GATATGTTTCGAACCAGTGGCTGTCTACACTACAGTTAGCAGTGTTCCGACGCTTGTCTCGGGGTCGTCTCATCAGGACAGCTAGGGGGCcTATAGTGAGTCGTATTA
**JP_286_t1-Monodelphis(UaaC)-F**	elongation	NW_001581879.1/85484827-85484696	For	TAATACGACTCACTATAGGCCATCTTTGATGTTCTGATGAGTGTGGGGTAAGATCTCACACTAACTTCCTTCTTTTACCTATGAAGAAACTGAATC
**JP_287_+5-Bcep176-(7)cis-HH-F**	Bcep176 previous PCR		Rev	TAATACGACTCACTATAGgcctcgtaataggtcgaaacggcgggagg
**JP_288_+5-Bcep176-(7)cis-HH-R**	Bcep176 previous PCR		Rev	GCGCTGATCTCAGCAGtac
**JP_289_manHH-t2-A-F**	human genome	Chr14-pos59056349(intron of neurexin2)	For	TATTTATAGAAGGACATTCATAG
**JP_290_manHH-t2-B-T7-F**	human genome	Chr14-pos78890872(intron of neurexin2)	For	TAATACGACTCACTATAGGACATTCATAGTGCTAGAGG
**JP_291_manHH-t2-A-R**	human genome	Chr14-pos78890872(intron of neurexin2)	Rev	CATCCTGCTTCCCTCCAAAGG
**JP_292_manHH-t2-B-R**	human genome	Chr14-pos78890872(intron of neurexin2)	Rev	GGTAATACCTGAATCATCAGC
**JP_293_manHH-t1-RECK-A-F**	human genome	Chr9-36072672(intron of RECK)	For	TCACAGTAGATTTGAGCCG
**JP_294_manHH-t1-RECK-B-T7-F**	human genome	Chr9-36072672(intron of RECK)	For	TAATACGACTCACTATAgGCCGTTACCTGCAGCTGATG
**JP_295_manHH-t1-RECK-A-R**	human genome	Chr9-36072672(intron of RECK)	Rev	TTAATACTATCTTAAGCCAGTAC
**JP_296_manHH-t1-RECK-B-R**	human genome	Chr9-36072672(intron of RECK)	Rev	GCCAGTACTGCAGGACCTAATAG
**JP_297_manHH-t1-c10-A-F**	human genome	Chr10-115928020(intron in 5'UTR of c10orf118)	For	AAATTGTTTCTTGACCAGTTGC

**JP_298_manHH-t1-c10-B-T7-F**	human genome	Chr10-115928020(intron in 5'UTR of c10orf118)	For TAATACGACTCACTATAggagttgcctacagctgatgagctc
**JP_299_manHH-t1-c10-A-R**	human genome	Chr10-115928020(intron in 5'UTR of c10orf118)	Rev TGTTTCTGTTTCTGACACAGTACTAC
**JP_300_manHH-t1-c10-B-R**	human genome	Chr10-115928020(intron in 5'UTR of c10orf118)	Rev CACAGTACTACAGGACCAATTC
**JP_301_Danio-no-P2-R**	elongation	Danio rerio	Rev ggTCCACCCATTTCCATCAGAGCAATGCTTCATTGCcGATGGGTGGAccTATAGTGAGTCGTATTA
**JP_302_chimp-no-P2-R**	elongation	Pan troglodytes	Rev ggATTCCATTTCTCTTCAGAGTGGAGATCCCCACTGATGGAATccTATAGTGAGTCGTATTA
**JP_303_Aedes-typical-R**	elongation	NW_0018112881/397739-397685	Rev CTGTGACAGATACACGTATTTCGACTACTACTTGTAGTCTTCTTCAGTGTCAGTTACTCGTATCCTATAGTGAGTCGTATTA
**JP_304_Nematostella-typical-R**	elongation	NZ_ABAV01012423.1/13414-13365	Rev aacTAACCATGGACAGCTGTTTCGTCCTTGTTAGGACTCGTCAGCATGGCATAccTATAGTGAGTCGTATTA
**JP_318_t2-env-(A-U)to(U-A)**	elongation	to obtain A15-U16 U16-A15 mutant	Rev TTGTTAGCCAGTCTCATCAGTACGGGTAGGCTAGTTCCCGCAGTCACCCCGAAGGGTGATTCGACTTAGTTAGccTATAGTGAGTCGTATTA
**ZW_093**	elongation		Rev TTACCTATTCCGCTAGCTCATCAGGCCCTAGGCGCGACCCT
**saltern 1 trunc B**	elongation	to obtain Hhmeta	For TAATACGACTCACTATAGGTCTTTAGGCGCCTGACGAGGCCCGTGTGGGGCCGAAAC
**saltern 2 trunc A**	elongation	to obtain Hhmeta	Rev TTGGCTAAGGCTACGGGGCTGACGCCCCGTTTCGGCCCCACACGGGCC
