# Supplementary material for: Identification of Hammerhead Ribozymes in All Domains of Life Reveals Novel Structural Variations
Source: PLoS Comput Biol. 2011 May 5;7(5):e1002031. doi: 10.1371/journal.pcbi.1002031 (PMC3088659; doi:10.1371/journal.pcbi.1002031)
Supplement: Text S1 — RNAMotif descriptors for hammerhead ribozymes. (DOC) [file pcbi.1002031.s011.doc]

# RNAMotif descriptors for hammerhead ribozymes

# Table of contents

[RNAMotif descriptors for hammerhead ribozymes 1](#__RefHeading___Toc252375710)

[Table of contents 1](#__RefHeading___Toc252375711)

[Example of RNAMotif descriptor annotated in detail 2](#__RefHeading___Toc252375712)

[Typical type I, II and III hammerhead ribozyme descriptors 6](#__RefHeading___Toc252375713)

[RNAMotif descriptor for type I hammerhead 6](#__RefHeading___Toc252375714)

[RNAMotif descriptor for type II hammerhead 10](#__RefHeading___Toc252375715)

[RNAMotif descriptor for type III hammerhead 14](#__RefHeading___Toc252375716)

[Descriptors for two hammerheads within 2kb of each other 18](#__RefHeading___Toc252375717)

[RNAMotif descriptor for type I+I hammerhead 18](#__RefHeading___Toc252375718)

[RNAMotif descriptor for type I+II hammerhead 24](#__RefHeading___Toc252375719)

[RNAMotif descriptor for type I+III hammerhead 30](#__RefHeading___Toc252375720)

[RNAMotif descriptor for type II+I hammerhead 36](#__RefHeading___Toc252375721)

[RNAMotif descriptor for type II+II hammerhead 42](#__RefHeading___Toc252375722)

[RNAMotif descriptor for type II+III hammerhead 48](#__RefHeading___Toc252375723)

[RNAMotif descriptor for type III+I hammerhead 54](#__RefHeading___Toc252375724)

[RNAMotif descriptor for type III+II hammerhead 60](#__RefHeading___Toc252375725)

[RNAMotif descriptor for type III+III hammerhead 66](#__RefHeading___Toc252375726)

[Variant hammerheads with NNNUGANGA core 72](#__RefHeading___Toc252375727)

[RNAMotif descriptor for typeI variant NNNUGANGA 72](#__RefHeading___Toc252375728)

[RNAMotif descriptor for typeII variant NNNUGANGA 76](#__RefHeading___Toc252375729)

[RNAMotif descriptor for typeII variant NNNUGANGA 80](#__RefHeading___Toc252375730)

Summary of content:

This file contains the RNAMotif descriptors used for the hammerhead ribozyme searches with this approach. They are all separated by subtitles. Any of these should work if they are copied in a text file and used as a descriptor with an installation of RNAMotif^1^. Note that most of the hammerhead ribozymes discovered, were actually found with the homology search RaveNna^2^ and Infernal^3^ although the RNAMotif searches did help building a more diversified alignment that was then used by RaveNna to find more hits. Note also that previously described descriptors^4^ have also been used for some searches.

1- Macke, T.J., Ecker, D.J., Gutell, R.R., Gautheret, D., Case D.A. & Sampath, R. RNAMotif, an RNA secondary structure definition and search algorithm. *Nucleic Acids Research* **29**, 4724-35 (2001).

2- Weinberg, Z. & Ruzzo, W.L. Sequence-based heuristics for faster annotation of non-coding RNA families. *Bioinformatics* **22**, 35-9 (2006).

3- Nawrocki, E.P., Kolbe, D.L. & Eddy, S.R. Infernal 1.0: inference of RNA alignments. *Bioinformatics* **25**, 1335-7 (2009).

4- Ferbeyre, G., Bourdeau, V., Pageau, M., Miramontes, P. & Cedergren, R. Distribution of Hammerhead and Hammerhead-like RNA Motifs Through the GenBank. *Genome Research* **10**, 1011-1019 (2000).

# Example of RNAMotif descriptor annotated in detail

######################################################

#rnamotif descriptor for typeI hammerhead

#######################################################

parms

wc += gu;

descr

#typeI

ss (len=10) # extra sequence for later manual analysis

h5 (minlen=4, maxlen=12) # stem I

ss (minlen=7, maxlen=8, seq="^cuganga") # one base allowed after core

h5 (minlen=2, maxlen=12) # stem II (it can be very short, only 2bp)

ss (minlen=3, maxlen=100) # loop II

h3 # stem II

ss (len=3, seq="gaa") # core (G12, A13, A14)

h5 (minlen=4, maxlen=12, seq="^a") # stem III (starts with A15)

ss (minlen=3, maxlen=100) # loop III

h3 (seq="u$") # stem III (ends with U16)

ss (len=1, seq="h") # cleavage site (anything but G)

h3 # stem I

ss (len=10) # extra sequence for later manual analysis

#################################################################################

# scoring rules #

# minimum (best) score is 3 worst accepted is 10, anything above 13 is rejected #

#################################################################################

score

{

SCORE=20; # start with 20 and substract bonuses

total_bonus=0;

lenCORE=length(ss[3]); # core (from C4 to A9)

if(lenCORE>7){ # if it has one extra base

total_bonus=total_bonus+3; # giva a +3 penalty

}

len3=length(h5[2]); # bonus if stem I is long

total_bonus=total_bonus-len3+4; # a length of 4 gives 0, 5 = -1, 6 = -2 ...

len1=length(h5[4]); # bonus if stem II is long

total_bonus=total_bonus-len1+2; # a length of 2 gives 0, 3 = -1, 4 = -2 ...

len2=length(h5[8]); # bonus if stem II is long

total_bonus=total_bonus-len2+4; # a length of 4 gives 0, 5 = -1, 6 = -2 ...

lenL1=length(ss[5]); # bonus if loop II is small

if(lenL1<40){ # if its smaller than 40 = -1

total_bonus--;

}

lenL2=length(ss[9]); # bonus if loop III is small

if(lenL2<40){ # if its smaller than 40 = -1

total_bonus--;

}

lenL1=length(ss[5]); # bonus if loop II is small

if(lenL1<30){ # if its smaller than 30, an additional -1

total_bonus--;

}

lenL2=length(ss[9]); # bonus if loop III is small

if(lenL2<30){ # if its smaller than 30, an additional -1

total_bonus--;

}

lenL1=length(ss[5]); # bonus if loop II is small

if(lenL1<20){ # if its smaller than 20, an additional -1

total_bonus--;

}

lenL2=length(ss[9]); # bonus if loop III is small

if(lenL2<20){ # if its smaller than 20, an additional -1

total_bonus--;

}

lenL1=length(ss[5]); # bonus if loop II is small

if(lenL1<10){ # if its smaller than 10, an additional -1

total_bonus--;

}

lenL2=length(ss[9]); # bonus if loop III is small

if(lenL2<10){ # if its smaller than 10, an additional -1

total_bonus--;

}

if(total_bonus>-10){ # at this point, reject hit if bonus

REJECT; # is higher (worst) than -10

}

# look if too much wobbles

# We have noticed that false positives are often rich in wobble base pairs.

# For that reason, all the following descriptions are meant to penalize excessive

# numbers of wobble base pairs in stems. Among other things, 2 consecutive wobbles

# would disqualify a hit (if the stem is too short without these wobbles)

i=1;

gu_ug=0; # variable to add wobbles

while(i<=(len3-1)){ # look at all bp in stem

if((h5[2,i,1]=="g")&&(h3[12,(len3-i+1),1]=="u")){ # determine if g-u

gu_ug++; # add to penalty

if((h5[2,(i+1),1]=="g")&&(h3[12,(len3-i+1-1),1]=="u")){ # reject if 2 g-u in a row

REJECT;

}

if((h5[2,(i+1),1]=="u")&&(h3[12,(len3-i+1-1),1]=="g")){ # same for u-g

REJECT;

}

}

if((h5[2,i,1]=="u")&&(h3[12,(len3-i+1),1]=="g")){ # same for u-g

gu_ug++;

if((h5[2,(i+1),1]=="g")&&(h3[12,(len3-i+1-1),1]=="u")){

REJECT;

}

if((h5[2,(i+1),1]=="u")&&(h3[12,(len3-i+1-1),1]=="g")){

REJECT;

}

}

i++;

}

if((h5[2,len3,1]=="g")&&(h3[12,1,1]=="u")){

gu_ug++;

}

if((h5[2,len3,1]=="u")&&(h3[12,1,1]=="g")){

gu_ug++;

}

i=1;

while(i<=(len1-1)){ # same for all stems

if((h5[4,i,1]=="g")&&(h3[6,(len1-i+1),1]=="u")){

gu_ug++;

if((h5[4,(i+1),1]=="g")&&(h3[6,(len1-i+1-1),1]=="u")){

REJECT;

}

if((h5[4,(i+1),1]=="u")&&(h3[6,(len1-i+1-1),1]=="g")){

REJECT;

}

}

if((h5[4,i,1]=="u")&&(h3[6,(len1-i+1),1]=="g")){

gu_ug++;

if((h5[4,(i+1),1]=="g")&&(h3[6,(len1-i+1-1),1]=="u")){

REJECT;

}

if((h5[4,(i+1),1]=="u")&&(h3[6,(len1-i+1-1),1]=="g")){

REJECT;

}

}

i++;

}

if((h5[4,len1,1]=="g")&&(h3[6,1,1]=="u")){

gu_ug++;

}

if((h5[4,len1,1]=="u")&&(h3[6,1,1]=="g")){

gu_ug++;

}

i=1;

while(i<=(len2-1)){

if((h5[8,i,1]=="g")&&(h3[10,(len2-i+1),1]=="u")){

gu_ug++;

if((h5[8,(i+1),1]=="g")&&(h3[10,(len2-i+1-1),1]=="u")){

REJECT;

}

if((h5[8,(i+1),1]=="u")&&(h3[10,(len2-i+1-1),1]=="g")){

REJECT;

}

}

if((h5[8,i,1]=="u")&&(h3[10,(len2-i+1),1]=="g")){

gu_ug++;

if((h5[8,(i+1),1]=="g")&&(h3[10,(len2-i+1-1),1]=="u")){

REJECT;

}

if((h5[8,(i+1),1]=="u")&&(h3[10,(len2-i+1-1),1]=="g")){

REJECT;

}

}

i++;

}

if((h5[8,len2,1]=="g")&&(h3[10,1,1]=="u")){

gu_ug++;

}

if((h5[8,len2,1]=="u")&&(h3[10,1,1]=="g")){

gu_ug++;

}

total_bonus=total_bonus+gu_ug;

if(total_bonus>-10){

REJECT;

}

SCORE=SCORE+total_bonus;

# Typical type I, II and III hammerhead ribozyme descriptors

## RNAMotif descriptor for type I hammerhead

#######################################################################

#rnamotif descriptor for typeI hammerhead

#######################################################################

parms

wc += gu;

descr

#typeI

h5 (minlen=3, maxlen=12)

ss (minlen=7, maxlen=8, seq="^cuganga")

h5 (minlen=2, maxlen=12)

ss (minlen=3, maxlen=100)

h3

ss (len=3, seq="gaa")

h5 (minlen=3, maxlen=12, seq="^a")

ss (minlen=3, maxlen=100)

h3 (seq="u$")

ss (len=1, seq="h")

h3

ss (len=10)

########################################################################################

# scoring rules, minimum (best) score is 3, worst is 20, anything above 13 is rejected

########################################################################################

score

{

SCORE = 20;

total_bonus = 0;

len=length( h5[1] );

if(len>3){

total_bonus--;

}

len=length( h5[3] );

if(len>3){

total_bonus--;

}

len=length( h5[7] );

if(len>3){

total_bonus--;

}

len=length( h5[1] );

if(len>5){

total_bonus--;

}

len=length( h5[3] );

if(len>5){

total_bonus--;

}

len=length( h5[7] );

if(len>5){

total_bonus--;

}

len=length(ss[4]);

if(len<7){

total_bonus--;

}

len=length(ss[8]);

if(len<7){

total_bonus--;

}

len=length(ss[2]);

if(len==7){

total_bonus--;

}

len=length(ss[4]);

if(len<20){

total_bonus--;

}

len=length(ss[8]);

if(len<20){

total_bonus--;

}

len=length(ss[4]);

if(len<10){

total_bonus--;

}

len=length(ss[8]);

if(len<10){

total_bonus--;

}

if(total_bonus > -7){

REJECT;

}

#look if too much wobbles

len=length( h5[1] );

i=0;

g=0;

c=0;

while(i<len){

if(h5[1,i,1]=="g"){

g++;

}

if(h5[1,i,1]=="c"){

c++;

}

if(h3[11,i,1]=="g"){

g++;

}

if(h3[11,i,1]=="c"){

c++;

}

if(g>0) {

if((c/g)<0.7){

total_bonus++;

}

}

i++;

}

len=length( h5[3] );

i=0;

g=0;

c=0;

while(i<len){

if(h5[3,i,1]=="g"){

g++;

}

if(h5[3,i,1]=="c"){

c++;

}

if(h3[5,i,1]=="g"){

g++;

}

if(h3[5,i,1]=="c"){

c++;

}

if(g>0) {

if((c/g)<0.7){

total_bonus++;

}

}

i++;

}

len=length( h5[7] );

i=0;

g=0;

c=0;

while(i<len){

if(h5[7,i,1]=="g"){

g++;

}

if(h5[7,i,1]=="c"){

c++;

}

if(h3[9,i,1]=="g"){

g++;

}

if(h3[9,i,1]=="c"){

c++;

}

if(g>0) {

if((c/g)<0.7){

total_bonus++;

}

}

i++;

}

if(total_bonus > -7){

REJECT;

}

SCORE = SCORE + total_bonus;

}

## RNAMotif descriptor for type II hammerhead

#######################################################################

#rnamotif descriptor for typeII hammerhead

#######################################################################

parms

wc += gu;

descr

h5 (minlen=2, maxlen=5)

ss (len=3, seq="gaa")

h5 (minlen=3, maxlen=12, seq="^a")

ss (minlen=3, maxlen=100)

h3 (seq="u$")

ss (len=1, seq="h")

h5 (minlen=3, maxlen=12)

ss (minlen=3, maxlen=100)

h3

ss (minlen=7, maxlen=8, seq="^cuganga")

h3

ss (len=10)

########################################################################################

# scoring rules, minimum (best) score is 3, worst is 20, anything above 13 is rejected

########################################################################################

score

{

SCORE = 20;

total_bonus = 0;

len=length( h5[1] );

if(len>3){

total_bonus--;

}

len=length( h5[3] );

if(len>3){

total_bonus--;

}

len=length( h5[7] );

if(len>3){

total_bonus--;

}

len=length( h5[1] );

if(len>5){

total_bonus--;

}

len=length( h5[3] );

if(len>5){

total_bonus--;

}

len=length( h5[7] );

if(len>5){

total_bonus--;

}

len=length(ss[4]);

if(len<7){

total_bonus--;

}

len=length(ss[8]);

if(len<7){

total_bonus--;

}

len=length(ss[10]);

if(len==7){

total_bonus--;

}

len=length(ss[4]);

if(len<20){

total_bonus--;

}

len=length(ss[8]);

if(len<20){

total_bonus--;

}

len=length(ss[4]);

if(len<10){

total_bonus--;

}

len=length(ss[8]);

if(len<10){

total_bonus--;

}

if(total_bonus > -7){

REJECT;

}

#look if too much wobbles

len=length( h5[1] );

i=0;

g=0;

c=0;

while(i<len){

if(h5[1,i,1]=="g"){

g++;

}

if(h5[1,i,1]=="c"){

c++;

}

if(h3[11,i,1]=="g"){

g++;

}

if(h3[11,i,1]=="c"){

c++;

}

if(g>0) {

if((c/g)<0.7){

total_bonus++;

}

}

i++;

}

len=length( h5[3] );

i=0;

g=0;

c=0;

while(i<len){

if(h5[3,i,1]=="g"){

g++;

}

if(h5[3,i,1]=="c"){

c++;

}

if(h3[5,i,1]=="g"){

g++;

}

if(h3[5,i,1]=="c"){

c++;

}

if(g>0) {

if((c/g)<0.7){

total_bonus++;

}

}

i++;

}

len=length( h5[7] );

i=0;

g=0;

c=0;

while(i<len){

if(h5[7,i,1]=="g"){

g++;

}

if(h5[7,i,1]=="c"){

c++;

}

if(h3[9,i,1]=="g"){

g++;

}

if(h3[9,i,1]=="c"){

c++;

}

if(g>0) {

if((c/g)<0.7){

total_bonus++;

}

}

i++;

}

if(total_bonus > -7){

REJECT;

}

SCORE = SCORE + total_bonus;

}

## RNAMotif descriptor for type III hammerhead

#####################################################################################

# rnamotif descriptor for type III hammerheads

#####################################################################################

parms

wc += gu;

descr

h5 (minlen=3, maxlen=12, seq="u$")

ss (len=1, seq="h")

h5 (minlen=3, maxlen=12)

ss (minlen=3, maxlen=100)

h3

ss (minlen=7, maxlen=8, seq="^cuganga")

h5 (minlen=2, maxlen=12)

ss (minlen=3, maxlen=100)

h3

ss (len=3, seq="gaa")

h3 (seq="^a")

ss (len=10)

########################################################################################

# scoring rules, minimum (best) score is 3, worst is 20, anything above 13 is rejected

########################################################################################

score

{

SCORE = 20;

total_bonus = 0;

len=length( h5[1] );

if(len>3){

total_bonus--;

}

len=length( h5[3] );

if(len>3){

total_bonus--;

}

len=length( h5[7] );

if(len>3){

total_bonus--;

}

len=length( h5[1] );

if(len>5){

total_bonus--;

}

len=length( h5[3] );

if(len>5){

total_bonus--;

}

len=length( h5[7] );

if(len>5){

total_bonus--;

}

len=length(ss[4]);

if(len<7){

total_bonus--;

}

len=length(ss[8]);

if(len<7){

total_bonus--;

}

len=length(ss[6]);

if(len==7){

total_bonus--;

}

len=length(ss[4]);

if(len<20){

total_bonus--;

}

len=length(ss[8]);

if(len<20){

total_bonus--;

}

len=length(ss[4]);

if(len<10){

total_bonus--;

}

len=length(ss[8]);

if(len<10){

total_bonus--;

}

if(total_bonus > -7){

REJECT;

}

#look if too much wobbles

len=length( h5[1] );

i=0;

g=0;

c=0;

while(i<len){

if(h5[1,i,1]=="g"){

g++;

}

if(h5[1,i,1]=="c"){

c++;

}

if(h3[11,i,1]=="g"){

g++;

}

if(h3[11,i,1]=="c"){

c++;

}

if(g>0) {

if((c/g)<0.7){

total_bonus++;

}

}

i++;

}

len=length( h5[3] );

i=0;

g=0;

c=0;

while(i<len){

if(h5[3,i,1]=="g"){

g++;

}

if(h5[3,i,1]=="c"){

c++;

}

if(h3[5,i,1]=="g"){

g++;

}

if(h3[5,i,1]=="c"){

c++;

}

if(g>0) {

if((c/g)<0.7){

total_bonus++;

}

}

i++;

}

len=length( h5[7] );

i=0;

g=0;

c=0;

while(i<len){

if(h5[7,i,1]=="g"){

g++;

}

if(h5[7,i,1]=="c"){

c++;

}

if(h3[9,i,1]=="g"){

g++;

}

if(h3[9,i,1]=="c"){

c++;

}

if(g>0) {

if((c/g)<0.7){

total_bonus++;

}

}

i++;

}

if(total_bonus > -7){

REJECT;

}

SCORE = SCORE + total_bonus;

}

# Descriptors for two hammerheads within 2kb of each other

## RNAMotif descriptor for type I+I hammerhead

######################################################

#rnamotif descriptor for typeI-typeI tandem hammerhead

#######################################################

parms

wc += gu;

descr

#typeI

h5 (minlen=3, maxlen=12)

ss (minlen=7, maxlen=8, seq="^cuganga")

h5 (minlen=2, maxlen=12)

ss (minlen=3, maxlen=100)

h3

ss (len=3, seq="gaa")

h5 (minlen=3, maxlen=12, seq="^a")

ss (minlen=3, maxlen=100)

h3 (seq="u$")

ss (len=1, seq="h")

h3

ss (minlen=0, maxlen=2000)

#typeI

h5 (minlen=3, maxlen=12)

ss (minlen=7, maxlen=8, seq="^cuganga")

h5 (minlen=2, maxlen=12)

ss (minlen=3, maxlen=100)

h3

ss (len=3, seq="gaa")

h5 (minlen=3, maxlen=12, seq="^a")

ss (minlen=3, maxlen=100)

h3 (seq="u$")

ss (len=1, seq="h")

h3

ss (len=10)

########################################################################################

# scoring rules, minimum (best) score is 3, worst is 20, anything above 13 is rejected

########################################################################################

score

{

SCORE = 20;

total_bonus = 0;

len=length( h5[1] );

if(len>3){

total_bonus--;

}

len=length( h5[3] );

if(len>3){

total_bonus--;

}

len=length( h5[7] );

if(len>3){

total_bonus--;

}

len=length( h5[13] );

if(len>3){

total_bonus--;

}

len=length( h5[15] );

if(len>3){

total_bonus--;

}

len=length( h5[19]);

if(len>3){

total_bonus--;

}

len=length( h5[1] );

if(len>5){

total_bonus--;

}

len=length( h5[3] );

if(len>5){

total_bonus--;

}

len=length( h5[7] );

if(len>5){

total_bonus--;

}

len=length( h5[13] );

if(len>5){

total_bonus--;

}

len=length( h5[15] );

if(len>5){

total_bonus--;

}

len=length( h5[19]);

if(len>5){

total_bonus--;

}

len=length(ss[4]);

if(len<8){

total_bonus--;

}

len=length(ss[8]);

if(len<8){

total_bonus--;

}

len=length(ss[16]);

if(len<8){

total_bonus--;

}

len=length(ss[20]);

if(len<8){

total_bonus--;

}

len=length(ss[2]);

if(len==7){

total_bonus--;

}

len=length(ss[14]);

if(len==7){

total_bonus--;

}

len=length(ss[4]);

if(len<20){

total_bonus--;

}

len=length(ss[8]);

if(len<20){

total_bonus--;

}

len=length(ss[16]);

if(len<20){

total_bonus--;

}

len=length(ss[20]);

if(len<20){

total_bonus--;

}

len=length(ss[4]);

if(len<10){

total_bonus--;

}

len=length(ss[8]);

if(len<10){

total_bonus--;

}

len=length(ss[16]);

if(len<10){

total_bonus--;

}

len=length(ss[20]);

if(len<10){

total_bonus--;

}

len=length(ss[12]);

if(len<1000){

total_bonus--;

}

if(total_bonus > -8){

REJECT;

}

#look if too much wobbles

len=length( h5[1] );

i=0;

g=0;

c=0;

while(i<len){

if(h5[1,i,1]=="g"){

g++;

}

if(h5[1,i,1]=="c"){

c++;

}

if(h3[11,i,1]=="g"){

g++;

}

if(h3[11,i,1]=="c"){

c++;

}

if(g>0) {

if((c/g)<0.7){

total_bonus++;

}

}

i++;

}

len=length( h5[3] );

i=0;

g=0;

c=0;

while(i<len){

if(h5[3,i,1]=="g"){

g++;

}

if(h5[3,i,1]=="c"){

c++;

}

if(h3[5,i,1]=="g"){

g++;

}

if(h3[5,i,1]=="c"){

c++;

}

if(g>0) {

if((c/g)<0.7){

total_bonus++;

}

}

i++;

}

len=length( h5[7] );

i=0;

g=0;

c=0;

while(i<len){

if(h5[7,i,1]=="g"){

g++;

}

if(h5[7,i,1]=="c"){

c++;

}

if(h3[9,i,1]=="g"){

g++;

}

if(h3[9,i,1]=="c"){

c++;

}

if(g>0) {

if((c/g)<0.7){

total_bonus++;

}

}

i++;

}

len=length( h5[13] );

i=0;

g=0;

c=0;

while(i<len){

if(h5[13,i,1]=="g"){

g++;

}

if(h5[13,i,1]=="c"){

c++;

}

if(h3[23,i,1]=="g"){

g++;

}

if(h3[23,i,1]=="c"){

c++;

}

if(g>0) {

if((c/g)<0.7){

total_bonus++;

}

}

i++;

}

len=length( h5[15] );

i=0;

g=0;

c=0;

while(i<len){

if(h5[15,i,1]=="g"){

g++;

}

if(h5[15,i,1]=="c"){

c++;

}

if(h3[17,i,1]=="g"){

g++;

}

if(h3[17,i,1]=="c"){

c++;

}

if(g>0) {

if((c/g)<0.7){

total_bonus++;

}

}

i++;

}

len=length( h5[19] );

i=0;

g=0;

c=0;

while(i<len){

if(h5[19,i,1]=="g"){

g++;

}

if(h5[19,i,1]=="c"){

c++;

}

if(h3[21,i,1]=="g"){

g++;

}

if(h3[21,i,1]=="c"){

c++;

}

if(g>0) {

if((c/g)<0.7){

total_bonus++;

}

}

i++;

}

if(total_bonus > -11){

REJECT;

}

SCORE = SCORE + total_bonus;

}

## RNAMotif descriptor for type I+II hammerhead

#rnamotif descriptor for typeI-typeII tandem hammerhead

parms

wc += gu;

descr

#typeI

h5 (minlen=3, maxlen=12)

ss (minlen=7, maxlen=8, seq="^cuganga")

h5 (minlen=2, maxlen=12)

ss (minlen=3, maxlen=100)

h3

ss (len=3, seq="gaa")

h5 (minlen=3, maxlen=12, seq="^a")

ss (minlen=3, maxlen=100)

h3 (seq="u$")

ss (len=1, seq="h")

h3

ss (minlen=0, maxlen=2000)

#typeII

h5 (minlen=2, maxlen=5)

ss (len=3, seq="gaa")

h5 (minlen=3, maxlen=12, seq="^a")

ss (minlen=3, maxlen=100)

h3 (seq="u$")

ss (len=1, seq="h")

h5 (minlen=3, maxlen=12)

ss (minlen=3, maxlen=100)

h3

ss (minlen=7, maxlen=8, seq="^cuganga")

h3

ss (len=10)

########################################################################################

# scoring rules, minimum (best) score is 3, worst is 20, anything above 13 is rejected

########################################################################################

score

{

SCORE = 20;

total_bonus = 0;

len=length( h5[1] );

if(len>3){

total_bonus--;

}

len=length( h5[3] );

if(len>3){

total_bonus--;

}

len=length( h5[7] );

if(len>3){

total_bonus--;

}

len=length( h5[13] );

if(len>3){

total_bonus--;

}

len=length( h5[15] );

if(len>3){

total_bonus--;

}

len=length( h5[19]);

if(len>3){

total_bonus--;

}

len=length( h5[1] );

if(len>5){

total_bonus--;

}

len=length( h5[3] );

if(len>5){

total_bonus--;

}

len=length( h5[7] );

if(len>5){

total_bonus--;

}

len=length( h5[13] );

if(len>5){

total_bonus--;

}

len=length( h5[15] );

if(len>5){

total_bonus--;

}

len=length( h5[19]);

if(len>5){

total_bonus--;

}

len=length(ss[4]);

if(len<8){

total_bonus--;

}

len=length(ss[8]);

if(len<8){

total_bonus--;

}

len=length(ss[16]);

if(len<8){

total_bonus--;

}

len=length(ss[20]);

if(len<8){

total_bonus--;

}

len=length(ss[2]);

if(len==7){

total_bonus--;

}

len=length(ss[22]);

if(len==7){

total_bonus--;

}

len=length(ss[4]);

if(len<20){

total_bonus--;

}

len=length(ss[8]);

if(len<20){

total_bonus--;

}

len=length(ss[16]);

if(len<20){

total_bonus--;

}

len=length(ss[20]);

if(len<20){

total_bonus--;

}

len=length(ss[4]);

if(len<10){

total_bonus--;

}

len=length(ss[8]);

if(len<10){

total_bonus--;

}

len=length(ss[16]);

if(len<10){

total_bonus--;

}

len=length(ss[20]);

if(len<10){

total_bonus--;

}

len=length(ss[12]);

if(len<1000){

total_bonus--;

}

if(total_bonus > -8){

REJECT;

}

#look if too much wobbles

len=length( h5[1] );

i=0;

g=0;

c=0;

while(i<len){

if(h5[1,i,1]=="g"){

g++;

}

if(h5[1,i,1]=="c"){

c++;

}

if(h3[11,i,1]=="g"){

g++;

}

if(h3[11,i,1]=="c"){

c++;

}

if(g>0) {

if((c/g)<0.7){

total_bonus++;

}

}

i++;

}

len=length( h5[3] );

i=0;

g=0;

c=0;

while(i<len){

if(h5[3,i,1]=="g"){

g++;

}

if(h5[3,i,1]=="c"){

c++;

}

if(h3[5,i,1]=="g"){

g++;

}

if(h3[5,i,1]=="c"){

c++;

}

if(g>0) {

if((c/g)<0.7){

total_bonus++;

}

}

i++;

}

len=length( h5[7] );

i=0;

g=0;

c=0;

while(i<len){

if(h5[7,i,1]=="g"){

g++;

}

if(h5[7,i,1]=="c"){

c++;

}

if(h3[9,i,1]=="g"){

g++;

}

if(h3[9,i,1]=="c"){

c++;

}

if(g>0) {

if((c/g)<0.7){

total_bonus++;

}

}

i++;

}

len=length( h5[13] );

i=0;

g=0;

c=0;

while(i<len){

if(h5[13,i,1]=="g"){

g++;

}

if(h5[13,i,1]=="c"){

c++;

}

if(h3[23,i,1]=="g"){

g++;

}

if(h3[23,i,1]=="c"){

c++;

}

if(g>0) {

if((c/g)<0.7){

total_bonus++;

}

}

i++;

}

len=length( h5[15] );

i=0;

g=0;

c=0;

while(i<len){

if(h5[15,i,1]=="g"){

g++;

}

if(h5[15,i,1]=="c"){

c++;

}

if(h3[17,i,1]=="g"){

g++;

}

if(h3[17,i,1]=="c"){

c++;

}

if(g>0) {

if((c/g)<0.7){

total_bonus++;

}

}

i++;

}

len=length( h5[19] );

i=0;

g=0;

c=0;

while(i<len){

if(h5[19,i,1]=="g"){

g++;

}

if(h5[19,i,1]=="c"){

c++;

}

if(h3[21,i,1]=="g"){

g++;

}

if(h3[21,i,1]=="c"){

c++;

}

if(g>0) {

if((c/g)<0.7){

total_bonus++;

}

}

i++;

}

if(total_bonus > -11){

REJECT;

}

SCORE = SCORE + total_bonus;

}

## RNAMotif descriptor for type I+III hammerhead

#rnamotif descriptor for typeI-typeIII tandem hammerhead

parms

wc += gu;

descr

#typeI

h5 (minlen=3, maxlen=12)

ss (minlen=7, maxlen=8, seq="^cuganga")

h5 (minlen=2, maxlen=12)

ss (minlen=3, maxlen=100)

h3

ss (len=3, seq="gaa")

h5 (minlen=3, maxlen=12, seq="^a")

ss (minlen=3, maxlen=100)

h3 (seq="u$")

ss (len=1, seq="h")

h3

ss (minlen=0, maxlen=2000)

#typeIII

h5 (minlen=3, maxlen=12, seq="u$")

ss (len=1, seq="h")

h5 (minlen=3, maxlen=12)

ss (minlen=3, maxlen=100)

h3

ss (minlen=7, maxlen=8, seq="^cuganga")

h5 (minlen=2, maxlen=12)

ss (minlen=3, maxlen=100)

h3

ss (len=3, seq="gaa")

h3 (seq="^a")

ss (len=10)

########################################################################################

# scoring rules, minimum (best) score is 3, worst is 20, anything above 13 is rejected

########################################################################################

score

{

SCORE = 20;

total_bonus = 0;

len=length( h5[1] );

if(len>3){

total_bonus--;

}

len=length( h5[3] );

if(len>3){

total_bonus--;

}

len=length( h5[7] );

if(len>3){

total_bonus--;

}

len=length( h5[13] );

if(len>3){

total_bonus--;

}

len=length( h5[15] );

if(len>3){

total_bonus--;

}

len=length( h5[19]);

if(len>3){

total_bonus--;

}

len=length( h5[1] );

if(len>5){

total_bonus--;

}

len=length( h5[3] );

if(len>5){

total_bonus--;

}

len=length( h5[7] );

if(len>5){

total_bonus--;

}

len=length( h5[13] );

if(len>5){

total_bonus--;

}

len=length( h5[15] );

if(len>5){

total_bonus--;

}

len=length( h5[19]);

if(len>5){

total_bonus--;

}

len=length(ss[4]);

if(len<8){

total_bonus--;

}

len=length(ss[8]);

if(len<8){

total_bonus--;

}

len=length(ss[16]);

if(len<8){

total_bonus--;

}

len=length(ss[20]);

if(len<8){

total_bonus--;

}

len=length(ss[2]);

if(len==7){

total_bonus--;

}

len=length(ss[18]);

if(len==7){

total_bonus--;

}

len=length(ss[4]);

if(len<20){

total_bonus--;

}

len=length(ss[8]);

if(len<20){

total_bonus--;

}

len=length(ss[16]);

if(len<20){

total_bonus--;

}

len=length(ss[20]);

if(len<20){

total_bonus--;

}

len=length(ss[4]);

if(len<10){

total_bonus--;

}

len=length(ss[8]);

if(len<10){

total_bonus--;

}

len=length(ss[16]);

if(len<10){

total_bonus--;

}

len=length(ss[20]);

if(len<10){

total_bonus--;

}

len=length(ss[12]);

if(len<1000){

total_bonus--;

}

if(total_bonus > -8){

REJECT;

}

#look if too much wobbles

len=length( h5[1] );

i=0;

g=0;

c=0;

while(i<len){

if(h5[1,i,1]=="g"){

g++;

}

if(h5[1,i,1]=="c"){

c++;

}

if(h3[11,i,1]=="g"){

g++;

}

if(h3[11,i,1]=="c"){

c++;

}

if(g>0) {

if((c/g)<0.7){

total_bonus++;

}

}

i++;

}

len=length( h5[3] );

i=0;

g=0;

c=0;

while(i<len){

if(h5[3,i,1]=="g"){

g++;

}

if(h5[3,i,1]=="c"){

c++;

}

if(h3[5,i,1]=="g"){

g++;

}

if(h3[5,i,1]=="c"){

c++;

}

if(g>0) {

if((c/g)<0.7){

total_bonus++;

}

}

i++;

}

len=length( h5[7] );

i=0;

g=0;

c=0;

while(i<len){

if(h5[7,i,1]=="g"){

g++;

}

if(h5[7,i,1]=="c"){

c++;

}

if(h3[9,i,1]=="g"){

g++;

}

if(h3[9,i,1]=="c"){

c++;

}

if(g>0) {

if((c/g)<0.7){

total_bonus++;

}

}

i++;

}

len=length( h5[13] );

i=0;

g=0;

c=0;

while(i<len){

if(h5[13,i,1]=="g"){

g++;

}

if(h5[13,i,1]=="c"){

c++;

}

if(h3[23,i,1]=="g"){

g++;

}

if(h3[23,i,1]=="c"){

c++;

}

if(g>0) {

if((c/g)<0.7){

total_bonus++;

}

}

i++;

}

len=length( h5[15] );

i=0;

g=0;

c=0;

while(i<len){

if(h5[15,i,1]=="g"){

g++;

}

if(h5[15,i,1]=="c"){

c++;

}

if(h3[17,i,1]=="g"){

g++;

}

if(h3[17,i,1]=="c"){

c++;

}

if(g>0) {

if((c/g)<0.7){

total_bonus++;

}

}

i++;

}

len=length( h5[19] );

i=0;

g=0;

c=0;

while(i<len){

if(h5[19,i,1]=="g"){

g++;

}

if(h5[19,i,1]=="c"){

c++;

}

if(h3[21,i,1]=="g"){

g++;

}

if(h3[21,i,1]=="c"){

c++;

}

if(g>0) {

if((c/g)<0.7){

total_bonus++;

}

}

i++;

}

if(total_bonus > -11){

REJECT;

}

SCORE = SCORE + total_bonus;

}

## RNAMotif descriptor for type II+I hammerhead

#rnamotif descriptor for typeII-typeI tandem hammerhead

parms

wc += gu;

descr

#typeII

h5 (minlen=2, maxlen=5)

ss (len=3, seq="gaa")

h5 (minlen=3, maxlen=12, seq="^a")

ss (minlen=3, maxlen=100)

h3 (seq="u$")

ss (len=1, seq="h")

h5 (minlen=3, maxlen=12)

ss (minlen=3, maxlen=100)

h3

ss (minlen=7, maxlen=8, seq="^cuganga")

h3

ss (minlen=0, maxlen=2000)

#typeI

h5 (minlen=3, maxlen=12)

ss (minlen=7, maxlen=8, seq="^cuganga")

h5 (minlen=2, maxlen=12)

ss (minlen=3, maxlen=100)

h3

ss (len=3, seq="gaa")

h5 (minlen=3, maxlen=12, seq="^a")

ss (minlen=3, maxlen=100)

h3 (seq="u$")

ss (len=1, seq="h")

h3

ss (len=10)

########################################################################################

# scoring rules, minimum (best) score is 3, worst is 20, anything above 13 is rejected

########################################################################################

score

{

SCORE = 20;

total_bonus = 0;

len=length( h5[1] );

if(len>3){

total_bonus--;

}

len=length( h5[3] );

if(len>3){

total_bonus--;

}

len=length( h5[7] );

if(len>3){

total_bonus--;

}

len=length( h5[13] );

if(len>3){

total_bonus--;

}

len=length( h5[15] );

if(len>3){

total_bonus--;

}

len=length( h5[19]);

if(len>3){

total_bonus--;

}

len=length( h5[1] );

if(len>5){

total_bonus--;

}

len=length( h5[3] );

if(len>5){

total_bonus--;

}

len=length( h5[7] );

if(len>5){

total_bonus--;

}

len=length( h5[13] );

if(len>5){

total_bonus--;

}

len=length( h5[15] );

if(len>5){

total_bonus--;

}

len=length( h5[19]);

if(len>5){

total_bonus--;

}

len=length(ss[4]);

if(len<8){

total_bonus--;

}

len=length(ss[8]);

if(len<8){

total_bonus--;

}

len=length(ss[16]);

if(len<8){

total_bonus--;

}

len=length(ss[20]);

if(len<8){

total_bonus--;

}

len=length(ss[10]);

if(len==7){

total_bonus--;

}

len=length(ss[14]);

if(len==7){

total_bonus--;

}

len=length(ss[4]);

if(len<20){

total_bonus--;

}

len=length(ss[8]);

if(len<20){

total_bonus--;

}

len=length(ss[16]);

if(len<20){

total_bonus--;

}

len=length(ss[20]);

if(len<20){

total_bonus--;

}

len=length(ss[4]);

if(len<10){

total_bonus--;

}

len=length(ss[8]);

if(len<10){

total_bonus--;

}

len=length(ss[16]);

if(len<10){

total_bonus--;

}

len=length(ss[20]);

if(len<10){

total_bonus--;

}

len=length(ss[12]);

if(len<1000){

total_bonus--;

}

if(total_bonus > -8){

REJECT;

}

#look if too much wobbles

len=length( h5[1] );

i=0;

g=0;

c=0;

while(i<len){

if(h5[1,i,1]=="g"){

g++;

}

if(h5[1,i,1]=="c"){

c++;

}

if(h3[11,i,1]=="g"){

g++;

}

if(h3[11,i,1]=="c"){

c++;

}

if(g>0) {

if((c/g)<0.7){

total_bonus++;

}

}

i++;

}

len=length( h5[3] );

i=0;

g=0;

c=0;

while(i<len){

if(h5[3,i,1]=="g"){

g++;

}

if(h5[3,i,1]=="c"){

c++;

}

if(h3[5,i,1]=="g"){

g++;

}

if(h3[5,i,1]=="c"){

c++;

}

if(g>0) {

if((c/g)<0.7){

total_bonus++;

}

}

i++;

}

len=length( h5[7] );

i=0;

g=0;

c=0;

while(i<len){

if(h5[7,i,1]=="g"){

g++;

}

if(h5[7,i,1]=="c"){

c++;

}

if(h3[9,i,1]=="g"){

g++;

}

if(h3[9,i,1]=="c"){

c++;

}

if(g>0) {

if((c/g)<0.7){

total_bonus++;

}

}

i++;

}

len=length( h5[13] );

i=0;

g=0;

c=0;

while(i<len){

if(h5[13,i,1]=="g"){

g++;

}

if(h5[13,i,1]=="c"){

c++;

}

if(h3[23,i,1]=="g"){

g++;

}

if(h3[23,i,1]=="c"){

c++;

}

if(g>0) {

if((c/g)<0.7){

total_bonus++;

}

}

i++;

}

len=length( h5[15] );

i=0;

g=0;

c=0;

while(i<len){

if(h5[15,i,1]=="g"){

g++;

}

if(h5[15,i,1]=="c"){

c++;

}

if(h3[17,i,1]=="g"){

g++;

}

if(h3[17,i,1]=="c"){

c++;

}

if(g>0) {

if((c/g)<0.7){

total_bonus++;

}

}

i++;

}

len=length( h5[19] );

i=0;

g=0;

c=0;

while(i<len){

if(h5[19,i,1]=="g"){

g++;

}

if(h5[19,i,1]=="c"){

c++;

}

if(h3[21,i,1]=="g"){

g++;

}

if(h3[21,i,1]=="c"){

c++;

}

if(g>0) {

if((c/g)<0.7){

total_bonus++;

}

}

i++;

}

if(total_bonus > -11){

REJECT;

}

SCORE = SCORE + total_bonus;

}

## RNAMotif descriptor for type II+II hammerhead

#rnamotif descriptor for typeII-typeII tandem hammerhead

parms

wc += gu;

descr

h5 (minlen=2, maxlen=5)

ss (len=3, seq="gaa")

h5 (minlen=3, maxlen=12, seq="^a")

ss (minlen=3, maxlen=100)

h3 (seq="u$")

ss (len=1, seq="h")

h5 (minlen=3, maxlen=12)

ss (minlen=3, maxlen=100)

h3

ss (minlen=7, maxlen=8, seq="^cuganga")

h3

ss (minlen=0, maxlen=2000)

h5 (minlen=2, maxlen=5)

ss (len=3, seq="gaa")

h5 (minlen=3, maxlen=12, seq="^a")

ss (minlen=3, maxlen=100)

h3 (seq="u$")

ss (len=1, seq="h")

h5 (minlen=3, maxlen=12)

ss (minlen=3, maxlen=100)

h3

ss (minlen=7, maxlen=8, seq="^cuganga")

h3

ss (len=10)

########################################################################################

# scoring rules, minimum (best) score is 3, worst is 20, anything above 13 is rejected

########################################################################################

score

{

SCORE = 20;

total_bonus = 0;

len=length( h5[1] );

if(len>3){

total_bonus--;

}

len=length( h5[3] );

if(len>3){

total_bonus--;

}

len=length( h5[7] );

if(len>3){

total_bonus--;

}

len=length( h5[13] );

if(len>3){

total_bonus--;

}

len=length( h5[15] );

if(len>3){

total_bonus--;

}

len=length( h5[19]);

if(len>3){

total_bonus--;

}

len=length(ss[10]);

if(len==7){

total_bonus--;

}

len=length(ss[22]);

if(len==7){

total_bonus--;

}

len=length( h5[1] );

if(len>5){

total_bonus--;

}

len=length( h5[3] );

if(len>5){

total_bonus--;

}

len=length( h5[7] );

if(len>5){

total_bonus--;

}

len=length( h5[13] );

if(len>5){

total_bonus--;

}

len=length( h5[15] );

if(len>5){

total_bonus--;

}

len=length( h5[19]);

if(len>5){

total_bonus--;

}

len=length(ss[4]);

if(len<8){

total_bonus--;

}

len=length(ss[8]);

if(len<8){

total_bonus--;

}

len=length(ss[16]);

if(len<8){

total_bonus--;

}

len=length(ss[20]);

if(len<8){

total_bonus--;

}

len=length(ss[4]);

if(len<20){

total_bonus--;

}

len=length(ss[8]);

if(len<20){

total_bonus--;

}

len=length(ss[16]);

if(len<20){

total_bonus--;

}

len=length(ss[20]);

if(len<20){

total_bonus--;

}

len=length(ss[4]);

if(len<10){

total_bonus--;

}

len=length(ss[8]);

if(len<10){

total_bonus--;

}

len=length(ss[16]);

if(len<10){

total_bonus--;

}

len=length(ss[20]);

if(len<10){

total_bonus--;

}

len=length(ss[12]);

if(len<1000){

total_bonus--;

}

if(total_bonus > -8){

REJECT;

}

#look if too much wobbles

len=length( h5[1] );

i=0;

g=0;

c=0;

while(i<len){

if(h5[1,i,1]=="g"){

g++;

}

if(h5[1,i,1]=="c"){

c++;

}

if(h3[11,i,1]=="g"){

g++;

}

if(h3[11,i,1]=="c"){

c++;

}

if(g>0) {

if((c/g)<0.7){

total_bonus++;

}

}

i++;

}

len=length( h5[3] );

i=0;

g=0;

c=0;

while(i<len){

if(h5[3,i,1]=="g"){

g++;

}

if(h5[3,i,1]=="c"){

c++;

}

if(h3[5,i,1]=="g"){

g++;

}

if(h3[5,i,1]=="c"){

c++;

}

if(g>0) {

if((c/g)<0.7){

total_bonus++;

}

}

i++;

}

len=length( h5[7] );

i=0;

g=0;

c=0;

while(i<len){

if(h5[7,i,1]=="g"){

g++;

}

if(h5[7,i,1]=="c"){

c++;

}

if(h3[9,i,1]=="g"){

g++;

}

if(h3[9,i,1]=="c"){

c++;

}

if(g>0) {

if((c/g)<0.7){

total_bonus++;

}

}

i++;

}

len=length( h5[13] );

i=0;

g=0;

c=0;

while(i<len){

if(h5[13,i,1]=="g"){

g++;

}

if(h5[13,i,1]=="c"){

c++;

}

if(h3[23,i,1]=="g"){

g++;

}

if(h3[23,i,1]=="c"){

c++;

}

if(g>0) {

if((c/g)<0.7){

total_bonus++;

}

}

i++;

}

len=length( h5[15] );

i=0;

g=0;

c=0;

while(i<len){

if(h5[15,i,1]=="g"){

g++;

}

if(h5[15,i,1]=="c"){

c++;

}

if(h3[17,i,1]=="g"){

g++;

}

if(h3[17,i,1]=="c"){

c++;

}

if(g>0) {

if((c/g)<0.7){

total_bonus++;

}

}

i++;

}

len=length( h5[19] );

i=0;

g=0;

c=0;

while(i<len){

if(h5[19,i,1]=="g"){

g++;

}

if(h5[19,i,1]=="c"){

c++;

}

if(h3[21,i,1]=="g"){

g++;

}

if(h3[21,i,1]=="c"){

c++;

}

if(g>0) {

if((c/g)<0.7){

total_bonus++;

}

}

i++;

}

if(total_bonus > -11){

REJECT;

}

SCORE = SCORE + total_bonus;

}

## RNAMotif descriptor for type II+III hammerhead

#rnamotif descriptor for typeII-typeIII tandem hammerhead

parms

wc += gu;

descr

#typeII

h5 (minlen=2, maxlen=5)

ss (len=3, seq="gaa")

h5 (minlen=3, maxlen=12, seq="^a")

ss (minlen=3, maxlen=100)

h3 (seq="u$")

ss (len=1, seq="h")

h5 (minlen=3, maxlen=12)

ss (minlen=3, maxlen=100)

h3

ss (minlen=7, maxlen=8, seq="^cuganga")

h3

ss (minlen=0, maxlen=2000)

#typeIII

h5 (minlen=3, maxlen=12, seq="u$")

ss (len=1, seq="h")

h5 (minlen=3, maxlen=12)

ss (minlen=3, maxlen=100)

h3

ss (minlen=7, maxlen=8, seq="^cuganga")

h5 (minlen=2, maxlen=12)

ss (minlen=3, maxlen=100)

h3

ss (len=3, seq="gaa")

h3 (seq="^a")

ss (len=10)

########################################################################################

# scoring rules, minimum (best) score is 3, worst is 20, anything above 13 is rejected

########################################################################################

score

{

SCORE = 20;

total_bonus = 0;

len=length( h5[1] );

if(len>3){

total_bonus--;

}

len=length( h5[3] );

if(len>3){

total_bonus--;

}

len=length( h5[7] );

if(len>3){

total_bonus--;

}

len=length( h5[13] );

if(len>3){

total_bonus--;

}

len=length( h5[15] );

if(len>3){

total_bonus--;

}

len=length( h5[19]);

if(len>3){

total_bonus--;

}

len=length( h5[1] );

if(len>5){

total_bonus--;

}

len=length( h5[3] );

if(len>5){

total_bonus--;

}

len=length( h5[7] );

if(len>5){

total_bonus--;

}

len=length( h5[13] );

if(len>5){

total_bonus--;

}

len=length( h5[15] );

if(len>5){

total_bonus--;

}

len=length( h5[19]);

if(len>5){

total_bonus--;

}

len=length(ss[4]);

if(len<8){

total_bonus--;

}

len=length(ss[8]);

if(len<8){

total_bonus--;

}

len=length(ss[16]);

if(len<8){

total_bonus--;

}

len=length(ss[20]);

if(len<8){

total_bonus--;

}

len=length(ss[10]);

if(len==7){

total_bonus--;

}

len=length(ss[18]);

if(len==7){

total_bonus--;

}

len=length(ss[4]);

if(len<20){

total_bonus--;

}

len=length(ss[8]);

if(len<20){

total_bonus--;

}

len=length(ss[16]);

if(len<20){

total_bonus--;

}

len=length(ss[20]);

if(len<20){

total_bonus--;

}

len=length(ss[4]);

if(len<10){

total_bonus--;

}

len=length(ss[8]);

if(len<10){

total_bonus--;

}

len=length(ss[16]);

if(len<10){

total_bonus--;

}

len=length(ss[20]);

if(len<10){

total_bonus--;

}

len=length(ss[12]);

if(len<1000){

total_bonus--;

}

if(total_bonus > -8){

REJECT;

}

#look if too much wobbles

len=length( h5[1] );

i=0;

g=0;

c=0;

while(i<len){

if(h5[1,i,1]=="g"){

g++;

}

if(h5[1,i,1]=="c"){

c++;

}

if(h3[11,i,1]=="g"){

g++;

}

if(h3[11,i,1]=="c"){

c++;

}

if(g>0) {

if((c/g)<0.7){

total_bonus++;

}

}

i++;

}

len=length( h5[3] );

i=0;

g=0;

c=0;

while(i<len){

if(h5[3,i,1]=="g"){

g++;

}

if(h5[3,i,1]=="c"){

c++;

}

if(h3[5,i,1]=="g"){

g++;

}

if(h3[5,i,1]=="c"){

c++;

}

if(g>0) {

if((c/g)<0.7){

total_bonus++;

}

}

i++;

}

len=length( h5[7] );

i=0;

g=0;

c=0;

while(i<len){

if(h5[7,i,1]=="g"){

g++;

}

if(h5[7,i,1]=="c"){

c++;

}

if(h3[9,i,1]=="g"){

g++;

}

if(h3[9,i,1]=="c"){

c++;

}

if(g>0) {

if((c/g)<0.7){

total_bonus++;

}

}

i++;

}

len=length( h5[13] );

i=0;

g=0;

c=0;

while(i<len){

if(h5[13,i,1]=="g"){

g++;

}

if(h5[13,i,1]=="c"){

c++;

}

if(h3[23,i,1]=="g"){

g++;

}

if(h3[23,i,1]=="c"){

c++;

}

if(g>0) {

if((c/g)<0.7){

total_bonus++;

}

}

i++;

}

len=length( h5[15] );

i=0;

g=0;

c=0;

while(i<len){

if(h5[15,i,1]=="g"){

g++;

}

if(h5[15,i,1]=="c"){

c++;

}

if(h3[17,i,1]=="g"){

g++;

}

if(h3[17,i,1]=="c"){

c++;

}

if(g>0) {

if((c/g)<0.7){

total_bonus++;

}

}

i++;

}

len=length( h5[19] );

i=0;

g=0;

c=0;

while(i<len){

if(h5[19,i,1]=="g"){

g++;

}

if(h5[19,i,1]=="c"){

c++;

}

if(h3[21,i,1]=="g"){

g++;

}

if(h3[21,i,1]=="c"){

c++;

}

if(g>0) {

if((c/g)<0.7){

total_bonus++;

}

}

i++;

}

if(total_bonus > -11){

REJECT;

}

SCORE = SCORE + total_bonus;

}

## RNAMotif descriptor for type III+I hammerhead

#rnamotif descriptor for typeIII-typeI tandem hammerhead

parms

wc += gu;

descr

#typeIII

h5 (minlen=3, maxlen=12, seq="u$")

ss (len=1, seq="h")

h5 (minlen=3, maxlen=12)

ss (minlen=3, maxlen=100)

h3

ss (minlen=7, maxlen=8, seq="^cuganga")

h5 (minlen=2, maxlen=12)

ss (minlen=3, maxlen=100)

h3

ss (len=3, seq="gaa")

h3 (seq="^a")

ss (minlen=0, maxlen=2000)

#typeI

h5 (minlen=3, maxlen=12)

ss (minlen=7, maxlen=8, seq="^cuganga")

h5 (minlen=2, maxlen=12)

ss (minlen=3, maxlen=100)

h3

ss (len=3, seq="gaa")

h5 (minlen=3, maxlen=12, seq="^a")

ss (minlen=3, maxlen=100)

h3 (seq="u$")

ss (len=1, seq="h")

h3

ss (len=10)

########################################################################################

# scoring rules, minimum (best) score is 3, worst is 20, anything above 13 is rejected

########################################################################################

score

{

SCORE = 20;

total_bonus = 0;

len=length( h5[1] );

if(len>3){

total_bonus--;

}

len=length( h5[3] );

if(len>3){

total_bonus--;

}

len=length( h5[7] );

if(len>3){

total_bonus--;

}

len=length( h5[13] );

if(len>3){

total_bonus--;

}

len=length( h5[15] );

if(len>3){

total_bonus--;

}

len=length( h5[19]);

if(len>3){

total_bonus--;

}

len=length( h5[1] );

if(len>5){

total_bonus--;

}

len=length( h5[3] );

if(len>5){

total_bonus--;

}

len=length( h5[7] );

if(len>5){

total_bonus--;

}

len=length( h5[13] );

if(len>5){

total_bonus--;

}

len=length( h5[15] );

if(len>5){

total_bonus--;

}

len=length( h5[19]);

if(len>5){

total_bonus--;

}

len=length(ss[4]);

if(len<8){

total_bonus--;

}

len=length(ss[8]);

if(len<8){

total_bonus--;

}

len=length(ss[16]);

if(len<8){

total_bonus--;

}

len=length(ss[20]);

if(len<8){

total_bonus--;

}

len=length(ss[6]);

if(len==7){

total_bonus--;

}

len=length(ss[14]);

if(len==7){

total_bonus--;

}

len=length(ss[4]);

if(len<20){

total_bonus--;

}

len=length(ss[8]);

if(len<20){

total_bonus--;

}

len=length(ss[16]);

if(len<20){

total_bonus--;

}

len=length(ss[20]);

if(len<20){

total_bonus--;

}

len=length(ss[4]);

if(len<10){

total_bonus--;

}

len=length(ss[8]);

if(len<10){

total_bonus--;

}

len=length(ss[16]);

if(len<10){

total_bonus--;

}

len=length(ss[20]);

if(len<10){

total_bonus--;

}

len=length(ss[12]);

if(len<1000){

total_bonus--;

}

if(total_bonus > -9){

REJECT;

}

#look if too much wobbles

len=length( h5[1] );

i=0;

g=0;

c=0;

while(i<len){

if(h5[1,i,1]=="g"){

g++;

}

if(h5[1,i,1]=="c"){

c++;

}

if(h3[11,i,1]=="g"){

g++;

}

if(h3[11,i,1]=="c"){

c++;

}

if(g>0) {

if((c/g)<0.7){

total_bonus++;

}

}

i++;

}

len=length( h5[3] );

i=0;

g=0;

c=0;

while(i<len){

if(h5[3,i,1]=="g"){

g++;

}

if(h5[3,i,1]=="c"){

c++;

}

if(h3[5,i,1]=="g"){

g++;

}

if(h3[5,i,1]=="c"){

c++;

}

if(g>0) {

if((c/g)<0.7){

total_bonus++;

}

}

i++;

}

len=length( h5[7] );

i=0;

g=0;

c=0;

while(i<len){

if(h5[7,i,1]=="g"){

g++;

}

if(h5[7,i,1]=="c"){

c++;

}

if(h3[9,i,1]=="g"){

g++;

}

if(h3[9,i,1]=="c"){

c++;

}

if(g>0) {

if((c/g)<0.7){

total_bonus++;

}

}

i++;

}

len=length( h5[13] );

i=0;

g=0;

c=0;

while(i<len){

if(h5[13,i,1]=="g"){

g++;

}

if(h5[13,i,1]=="c"){

c++;

}

if(h3[23,i,1]=="g"){

g++;

}

if(h3[23,i,1]=="c"){

c++;

}

if(g>0) {

if((c/g)<0.7){

total_bonus++;

}

}

i++;

}

len=length( h5[15] );

i=0;

g=0;

c=0;

while(i<len){

if(h5[15,i,1]=="g"){

g++;

}

if(h5[15,i,1]=="c"){

c++;

}

if(h3[17,i,1]=="g"){

g++;

}

if(h3[17,i,1]=="c"){

c++;

}

if(g>0) {

if((c/g)<0.7){

total_bonus++;

}

}

i++;

}

len=length( h5[19] );

i=0;

g=0;

c=0;

while(i<len){

if(h5[19,i,1]=="g"){

g++;

}

if(h5[19,i,1]=="c"){

c++;

}

if(h3[21,i,1]=="g"){

g++;

}

if(h3[21,i,1]=="c"){

c++;

}

if(g>0) {

if((c/g)<0.7){

total_bonus++;

}

}

i++;

}

if(total_bonus > -11){

REJECT;

}

SCORE = SCORE + total_bonus;

}

## RNAMotif descriptor for type III+II hammerhead

#rnamotif descriptor for typeIII-typeII tandem hammerhead

parms

wc += gu;

descr

h5 (minlen=3, maxlen=12, seq="u$")

ss (len=1, seq="h")

h5 (minlen=3, maxlen=12)

ss (minlen=3, maxlen=100)

h3

ss (minlen=7, maxlen=8, seq="^cuganga")

h5 (minlen=2, maxlen=12)

ss (minlen=3, maxlen=100)

h3

ss (len=3, seq="gaa")

h3 (seq="^a")

ss (minlen=0, maxlen=2000)

h5 (minlen=2, maxlen=5)

ss (len=3, seq="gaa")

h5 (minlen=3, maxlen=12, seq="^a")

ss (minlen=3, maxlen=100)

h3 (seq="u$")

ss (len=1, seq="h")

h5 (minlen=3, maxlen=12)

ss (minlen=3, maxlen=100)

h3

ss (minlen=7, maxlen=8, seq="^cuganga")

h3

ss (len=10)

########################################################################################

# scoring rules, minimum (best) score is 3, worst is 20, anything above 13 is rejected

########################################################################################

score

{

SCORE = 20;

total_bonus = 0;

len=length( h5[1] );

if(len>3){

total_bonus--;

}

len=length( h5[3] );

if(len>3){

total_bonus--;

}

len=length( h5[7] );

if(len>3){

total_bonus--;

}

len=length( h5[13] );

if(len>3){

total_bonus--;

}

len=length( h5[15] );

if(len>3){

total_bonus--;

}

len=length( h5[19]);

if(len>3){

total_bonus--;

}

len=length( h5[1] );

if(len>5){

total_bonus--;

}

len=length( h5[3] );

if(len>5){

total_bonus--;

}

len=length( h5[7] );

if(len>5){

total_bonus--;

}

len=length( h5[13] );

if(len>5){

total_bonus--;

}

len=length( h5[15] );

if(len>5){

total_bonus--;

}

len=length( h5[19]);

if(len>5){

total_bonus--;

}

len=length(ss[4]);

if(len<8){

total_bonus--;

}

len=length(ss[8]);

if(len<8){

total_bonus--;

}

len=length(ss[16]);

if(len<8){

total_bonus--;

}

len=length(ss[20]);

if(len<8){

total_bonus--;

}

len=length(ss[6]);

if(len==7){

total_bonus--;

}

len=length(ss[4]);

if(len<20){

total_bonus--;

}

len=length(ss[8]);

if(len<20){

total_bonus--;

}

len=length(ss[16]);

if(len<20){

total_bonus--;

}

len=length(ss[20]);

if(len<20){

total_bonus--;

}

len=length(ss[4]);

if(len<10){

total_bonus--;

}

len=length(ss[8]);

if(len<10){

total_bonus--;

}

len=length(ss[16]);

if(len<10){

total_bonus--;

}

len=length(ss[20]);

if(len<10){

total_bonus--;

}

len=length(ss[12]);

if(len<1000){

total_bonus--;

}

if(total_bonus > -9){

REJECT;

}

#look if too much wobbles

len=length( h5[1] );

i=0;

g=0;

c=0;

while(i<len){

if(h5[1,i,1]=="g"){

g++;

}

if(h5[1,i,1]=="c"){

c++;

}

if(h3[11,i,1]=="g"){

g++;

}

if(h3[11,i,1]=="c"){

c++;

}

if(g>0) {

if((c/g)<0.7){

total_bonus++;

}

}

i++;

}

len=length( h5[3] );

i=0;

g=0;

c=0;

while(i<len){

if(h5[3,i,1]=="g"){

g++;

}

if(h5[3,i,1]=="c"){

c++;

}

if(h3[5,i,1]=="g"){

g++;

}

if(h3[5,i,1]=="c"){

c++;

}

if(g>0) {

if((c/g)<0.7){

total_bonus++;

}

}

i++;

}

len=length( h5[7] );

i=0;

g=0;

c=0;

while(i<len){

if(h5[7,i,1]=="g"){

g++;

}

if(h5[7,i,1]=="c"){

c++;

}

if(h3[9,i,1]=="g"){

g++;

}

if(h3[9,i,1]=="c"){

c++;

}

if(g>0) {

if((c/g)<0.7){

total_bonus++;

}

}

i++;

}

len=length( h5[13] );

i=0;

g=0;

c=0;

while(i<len){

if(h5[13,i,1]=="g"){

g++;

}

if(h5[13,i,1]=="c"){

c++;

}

if(h3[23,i,1]=="g"){

g++;

}

if(h3[23,i,1]=="c"){

c++;

}

if(g>0) {

if((c/g)<0.7){

total_bonus++;

}

}

i++;

}

len=length( h5[15] );

i=0;

g=0;

c=0;

while(i<len){

if(h5[15,i,1]=="g"){

g++;

}

if(h5[15,i,1]=="c"){

c++;

}

if(h3[17,i,1]=="g"){

g++;

}

if(h3[17,i,1]=="c"){

c++;

}

if(g>0) {

if((c/g)<0.7){

total_bonus++;

}

}

i++;

}

len=length( h5[19] );

i=0;

g=0;

c=0;

while(i<len){

if(h5[19,i,1]=="g"){

g++;

}

if(h5[19,i,1]=="c"){

c++;

}

if(h3[21,i,1]=="g"){

g++;

}

if(h3[21,i,1]=="c"){

c++;

}

if(g>0) {

if((c/g)<0.7){

total_bonus++;

}

}

i++;

}

if(total_bonus > -11){

REJECT;

}

SCORE = SCORE + total_bonus;

}

## RNAMotif descriptor for type III+III hammerhead

#rnamotif descriptor for typeIII-typeI tandem hammerhead

parms

wc += gu;

descr

#typeIII

h5 (minlen=3, maxlen=12, seq="u$")

ss (len=1, seq="h")

h5 (minlen=3, maxlen=12)

ss (minlen=3, maxlen=100)

h3

ss (minlen=7, maxlen=8, seq="^cuganga")

h5 (minlen=2, maxlen=12)

ss (minlen=3, maxlen=100)

h3

ss (len=3, seq="gaa")

h3 (seq="^a")

ss (minlen=0, maxlen=2000)

#typeI

h5 (minlen=3, maxlen=12)

ss (minlen=7, maxlen=8, seq="^cuganga")

h5 (minlen=2, maxlen=12)

ss (minlen=3, maxlen=100)

h3

ss (len=3, seq="gaa")

h5 (minlen=3, maxlen=12, seq="^a")

ss (minlen=3, maxlen=100)

h3 (seq="u$")

ss (len=1, seq="h")

h3

ss (len=10)

########################################################################################

# scoring rules, minimum (best) score is 3, worst is 20, anything above 13 is rejected

########################################################################################

score

{

SCORE = 20;

total_bonus = 0;

len=length( h5[1] );

if(len>3){

total_bonus--;

}

len=length( h5[3] );

if(len>3){

total_bonus--;

}

len=length( h5[7] );

if(len>3){

total_bonus--;

}

len=length( h5[13] );

if(len>3){

total_bonus--;

}

len=length( h5[15] );

if(len>3){

total_bonus--;

}

len=length( h5[19]);

if(len>3){

total_bonus--;

}

len=length( h5[1] );

if(len>5){

total_bonus--;

}

len=length( h5[3] );

if(len>5){

total_bonus--;

}

len=length( h5[7] );

if(len>5){

total_bonus--;

}

len=length( h5[13] );

if(len>5){

total_bonus--;

}

len=length( h5[15] );

if(len>5){

total_bonus--;

}

len=length( h5[19]);

if(len>5){

total_bonus--;

}

len=length(ss[4]);

if(len<8){

total_bonus--;

}

len=length(ss[8]);

if(len<8){

total_bonus--;

}

len=length(ss[16]);

if(len<8){

total_bonus--;

}

len=length(ss[20]);

if(len<8){

total_bonus--;

}

len=length(ss[6]);

if(len==7){

total_bonus--;

}

len=length(ss[14]);

if(len==7){

total_bonus--;

}

len=length(ss[4]);

if(len<20){

total_bonus--;

}

len=length(ss[8]);

if(len<20){

total_bonus--;

}

len=length(ss[16]);

if(len<20){

total_bonus--;

}

len=length(ss[20]);

if(len<20){

total_bonus--;

}

len=length(ss[4]);

if(len<10){

total_bonus--;

}

len=length(ss[8]);

if(len<10){

total_bonus--;

}

len=length(ss[16]);

if(len<10){

total_bonus--;

}

len=length(ss[20]);

if(len<10){

total_bonus--;

}

len=length(ss[12]);

if(len<1000){

total_bonus--;

}

if(total_bonus > -9){

REJECT;

}

#look if too much wobbles

len=length( h5[1] );

i=0;

g=0;

c=0;

while(i<len){

if(h5[1,i,1]=="g"){

g++;

}

if(h5[1,i,1]=="c"){

c++;

}

if(h3[11,i,1]=="g"){

g++;

}

if(h3[11,i,1]=="c"){

c++;

}

if(g>0) {

if((c/g)<0.7){

total_bonus++;

}

}

i++;

}

len=length( h5[3] );

i=0;

g=0;

c=0;

while(i<len){

if(h5[3,i,1]=="g"){

g++;

}

if(h5[3,i,1]=="c"){

c++;

}

if(h3[5,i,1]=="g"){

g++;

}

if(h3[5,i,1]=="c"){

c++;

}

if(g>0) {

if((c/g)<0.7){

total_bonus++;

}

}

i++;

}

len=length( h5[7] );

i=0;

g=0;

c=0;

while(i<len){

if(h5[7,i,1]=="g"){

g++;

}

if(h5[7,i,1]=="c"){

c++;

}

if(h3[9,i,1]=="g"){

g++;

}

if(h3[9,i,1]=="c"){

c++;

}

if(g>0) {

if((c/g)<0.7){

total_bonus++;

}

}

i++;

}

len=length( h5[13] );

i=0;

g=0;

c=0;

while(i<len){

if(h5[13,i,1]=="g"){

g++;

}

if(h5[13,i,1]=="c"){

c++;

}

if(h3[23,i,1]=="g"){

g++;

}

if(h3[23,i,1]=="c"){

c++;

}

if(g>0) {

if((c/g)<0.7){

total_bonus++;

}

}

i++;

}

len=length( h5[15] );

i=0;

g=0;

c=0;

while(i<len){

if(h5[15,i,1]=="g"){

g++;

}

if(h5[15,i,1]=="c"){

c++;

}

if(h3[17,i,1]=="g"){

g++;

}

if(h3[17,i,1]=="c"){

c++;

}

if(g>0) {

if((c/g)<0.7){

total_bonus++;

}

}

i++;

}

len=length( h5[19] );

i=0;

g=0;

c=0;

while(i<len){

if(h5[19,i,1]=="g"){

g++;

}

if(h5[19,i,1]=="c"){

c++;

}

if(h3[21,i,1]=="g"){

g++;

}

if(h3[21,i,1]=="c"){

c++;

}

if(g>0) {

if((c/g)<0.7){

total_bonus++;

}

}

i++;

}

if(total_bonus > -11){

REJECT;

}

SCORE = SCORE + total_bonus;

}

# Variant hammerheads with NNNUGANGA core

## RNAMotif descriptor for typeI variant NNNUGANGA

#######################################################################

#rnamotif descriptor for typeI variant hammerhead with NNNUGANGA core

#######################################################################

parms

wc += gu;

descr

#typeI

ss (len=10)

h5 (minlen=4, maxlen=12)

ss (len=9, seq="^nnnuganga")

h5 (minlen=3, maxlen=12)

ss (minlen=3, maxlen=100)

h3

ss (len=3, seq="gaa")

h5 (minlen=4, maxlen=12, seq="^a")

ss (minlen=3, maxlen=100)

h3 (seq="u$")

ss (len=1, seq="h")

h3

ss (len=10)

########################################################################################

# scoring rules, minimum (best) score is 3, worst is 20, anything above 13 is rejected

########################################################################################

score

{

SCORE = 20;

total_bonus = 0;

len3=length( h5[2] );

total_bonus = total_bonus - len3 + 4;

len1=length( h5[4] );

total_bonus = total_bonus - len1 + 3;

len2=length( h5[8] );

total_bonus = total_bonus - len2 + 4;;

lenL1=length(ss[5]);

if(lenL1<40){

total_bonus--;

}

lenL2=length(ss[9]);

if(lenL2<40){

total_bonus--;

}

lenL1=length(ss[5]);

if(lenL1<30){

total_bonus--;

}

lenL2=length(ss[9]);

if(lenL2<30){

total_bonus--;

}

lenL1=length(ss[5]);

if(lenL1<20){

total_bonus--;

}

lenL2=length(ss[9]);

if(lenL2<20){

total_bonus--;

}

lenL1=length(ss[5]);

if(lenL1<10){

total_bonus--;

}

lenL2=length(ss[9]);

if(lenL2<10){

total_bonus--;

}

if(total_bonus > -7){

REJECT;

}

#look if too much wobbles

i=1;

gu_ug=0;

while(i<=(len3-1)){

if((h5[2,i,1]=="g") && (h3[12,(len3-i+1),1]=="u")){

gu_ug++;

if((h5[2,(i+1),1]=="g") && (h3[12,(len3-i+1-1),1]=="u")){

REJECT;

}

if((h5[2,(i+1),1]=="u") && (h3[12,(len3-i+1-1),1]=="g")){

REJECT;

}

}

if((h5[2,i,1]=="u") && (h3[12,(len3-i+1),1]=="g")){

gu_ug++;

if((h5[2,(i+1),1]=="g") && (h3[12,(len3-i+1-1),1]=="u")){

REJECT;

}

if((h5[2,(i+1),1]=="u") && (h3[12,(len3-i+1-1),1]=="g")){

REJECT;

}

}

i++;

}

if((h5[2,len3,1]=="g") && (h3[12,1,1]=="u")){

gu_ug++;

}

if((h5[2,len3,1]=="u") && (h3[12,1,1]=="g")){

gu_ug++;

}

i=1;

while(i<=(len1-1)){

if((h5[4,i,1]=="g") && (h3[6,(len1-i+1),1]=="u")){

gu_ug++;

if((h5[4,(i+1),1]=="g") && (h3[6,(len1-i+1-1),1]=="u")){

REJECT;

}

if((h5[4,(i+1),1]=="u") && (h3[6,(len1-i+1-1),1]=="g")){

REJECT;

}

}

if((h5[4,i,1]=="u") && (h3[6,(len1-i+1),1]=="g")){

gu_ug++;

if((h5[4,(i+1),1]=="g") && (h3[6,(len1-i+1-1),1]=="u")){

REJECT;

}

if((h5[4,(i+1),1]=="u") && (h3[6,(len1-i+1-1),1]=="g")){

REJECT;

}

}

i++;

}

if((h5[4,len1,1]=="g") && (h3[6,1,1]=="u")){

gu_ug++;

}

if((h5[4,len1,1]=="u") && (h3[6,1,1]=="g")){

gu_ug++;

}

i=1;

while(i<=(len2-1)){

if((h5[8,i,1]=="g") && (h3[10,(len2-i+1),1]=="u")){

gu_ug++;

if((h5[8,(i+1),1]=="g") && (h3[10,(len2-i+1-1),1]=="u")){

REJECT;

}

if((h5[8,(i+1),1]=="u") && (h3[10,(len2-i+1-1),1]=="g")){

REJECT;

}

}

if((h5[8,i,1]=="u") && (h3[10,(len2-i+1),1]=="g")){

gu_ug++;

if((h5[8,(i+1),1]=="g") && (h3[10,(len2-i+1-1),1]=="u")){

REJECT;

}

if((h5[8,(i+1),1]=="u") && (h3[10,(len2-i+1-1),1]=="g")){

REJECT;

}

}

i++;

}

if((h5[8,len2,1]=="g") && (h3[10,1,1]=="u")){

gu_ug++;

}

if((h5[8,len2,1]=="u") && (h3[10,1,1]=="g")){

gu_ug++;

}

total_bonus = total_bonus + gu_ug;

if(total_bonus > -7){

REJECT;

}

SCORE = SCORE + total_bonus;

}

## RNAMotif descriptor for typeII variant NNNUGANGA

#######################################################################

#rnamotif descriptor for typeII variant hammerhead with NNNUGANGA core

#######################################################################

parms

wc += gu;

descr

ss (len=10)

h5 (minlen=3, maxlen=10)

ss (len=3, seq="gaa")

h5 (minlen=4, maxlen=12, seq="^a")

ss (minlen=3, maxlen=100)

h3 (seq="u$")

ss (len=1, seq="h")

h5 (minlen=4, maxlen=12)

ss (minlen=3, maxlen=100)

h3

ss (len=9, seq="^nnnuganga")

h3

ss (len=10)

########################################################################################

# scoring rules, minimum (best) score is 3, worst is 20, anything above 13 is rejected

########################################################################################

score

{

SCORE = 20;

total_bonus = 0;

len3=length( h5[2] );

total_bonus = total_bonus - len3 + 3;

len1=length( h5[4] );

total_bonus = total_bonus - len1 + 4;

len2=length( h5[8] );

total_bonus = total_bonus - len2 + 4;;

lenL1=length(ss[5]);

if(lenL1<40){

total_bonus--;

}

lenL2=length(ss[9]);

if(lenL2<40){

total_bonus--;

}

lenL1=length(ss[5]);

if(lenL1<30){

total_bonus--;

}

lenL2=length(ss[9]);

if(lenL2<30){

total_bonus--;

}

lenL1=length(ss[5]);

if(lenL1<20){

total_bonus--;

}

lenL2=length(ss[9]);

if(lenL2<20){

total_bonus--;

}

lenL1=length(ss[5]);

if(lenL1<10){

total_bonus--;

}

lenL2=length(ss[9]);

if(lenL2<10){

total_bonus--;

}

if(total_bonus > -7){

REJECT;

}

#look if too much wobbles

i=1;

gu_ug=0;

while(i<=(len3-1)){

if((h5[2,i,1]=="g") && (h3[12,(len3-i+1),1]=="u")){

gu_ug++;

if((h5[2,(i+1),1]=="g") && (h3[12,(len3-i+1-1),1]=="u")){

REJECT;

}

if((h5[2,(i+1),1]=="u") && (h3[12,(len3-i+1-1),1]=="g")){

REJECT;

}

}

if((h5[2,i,1]=="u") && (h3[12,(len3-i+1),1]=="g")){

gu_ug++;

if((h5[2,(i+1),1]=="g") && (h3[12,(len3-i+1-1),1]=="u")){

REJECT;

}

if((h5[2,(i+1),1]=="u") && (h3[12,(len3-i+1-1),1]=="g")){

REJECT;

}

}

i++;

}

if((h5[2,len3,1]=="g") && (h3[12,1,1]=="u")){

gu_ug++;

}

if((h5[2,len3,1]=="u") && (h3[12,1,1]=="g")){

gu_ug++;

}

i=1;

while(i<=(len1-1)){

if((h5[4,i,1]=="g") && (h3[6,(len1-i+1),1]=="u")){

gu_ug++;

if((h5[4,(i+1),1]=="g") && (h3[6,(len1-i+1-1),1]=="u")){

REJECT;

}

if((h5[4,(i+1),1]=="u") && (h3[6,(len1-i+1-1),1]=="g")){

REJECT;

}

}

if((h5[4,i,1]=="u") && (h3[6,(len1-i+1),1]=="g")){

gu_ug++;

if((h5[4,(i+1),1]=="g") && (h3[6,(len1-i+1-1),1]=="u")){

REJECT;

}

if((h5[4,(i+1),1]=="u") && (h3[6,(len1-i+1-1),1]=="g")){

REJECT;

}

}

i++;

}

if((h5[4,len1,1]=="g") && (h3[6,1,1]=="u")){

gu_ug++;

}

if((h5[4,len1,1]=="u") && (h3[6,1,1]=="g")){

gu_ug++;

}

i=1;

while(i<=(len2-1)){

if((h5[8,i,1]=="g") && (h3[10,(len2-i+1),1]=="u")){

gu_ug++;

if((h5[8,(i+1),1]=="g") && (h3[10,(len2-i+1-1),1]=="u")){

REJECT;

}

if((h5[8,(i+1),1]=="u") && (h3[10,(len2-i+1-1),1]=="g")){

REJECT;

}

}

if((h5[8,i,1]=="u") && (h3[10,(len2-i+1),1]=="g")){

gu_ug++;

if((h5[8,(i+1),1]=="g") && (h3[10,(len2-i+1-1),1]=="u")){

REJECT;

}

if((h5[8,(i+1),1]=="u") && (h3[10,(len2-i+1-1),1]=="g")){

REJECT;

}

}

i++;

}

if((h5[8,len2,1]=="g") && (h3[10,1,1]=="u")){

gu_ug++;

}

if((h5[8,len2,1]=="u") && (h3[10,1,1]=="g")){

gu_ug++;

}

total_bonus = total_bonus + gu_ug;

if(total_bonus > -7){

REJECT;

}

SCORE = SCORE + total_bonus;

}

## RNAMotif descriptor for typeII variant NNNUGANGA

#######################################################################

#rnamotif descriptor for typeIII variant hammerhead with NNNUGANGA core

#######################################################################

parms

wc += gu;

descr

ss (len=10)

h5 (minlen=4, maxlen=12, seq="u$")

ss (len=1, seq="h")

h5 (minlen=4, maxlen=12)

ss (minlen=3, maxlen=100)

h3

ss (len=9, seq="^nnnuganga")

h5 (minlen=3, maxlen=12)

ss (minlen=3, maxlen=100)

h3

ss (len=3, seq="gaa")

h3 (seq="^a")

ss (len=10)

########################################################################################

# scoring rules, minimum (best) score is 3, worst is 20, anything above 13 is rejected

########################################################################################

score

{

SCORE = 20;

total_bonus = 0;

len3=length( h5[2] );

total_bonus = total_bonus - len3 + 4;

len1=length( h5[4] );

total_bonus = total_bonus - len1 + 4;

len2=length( h5[8] );

total_bonus = total_bonus - len2 + 3;;

lenL1=length(ss[5]);

if(lenL1<40){

total_bonus--;

}

lenL2=length(ss[9]);

if(lenL2<40){

total_bonus--;

}

lenL1=length(ss[5]);

if(lenL1<30){

total_bonus--;

}

lenL2=length(ss[9]);

if(lenL2<30){

total_bonus--;

}

lenL1=length(ss[5]);

if(lenL1<20){

total_bonus--;

}

lenL2=length(ss[9]);

if(lenL2<20){

total_bonus--;

}

lenL1=length(ss[5]);

if(lenL1<10){

total_bonus--;

}

lenL2=length(ss[9]);

if(lenL2<10){

total_bonus--;

}

if(total_bonus > -7){

REJECT;

}

#look if too much wobbles

i=1;

gu_ug=0;

while(i<=(len3-1)){

if((h5[2,i,1]=="g") && (h3[12,(len3-i+1),1]=="u")){

gu_ug++;

if((h5[2,(i+1),1]=="g") && (h3[12,(len3-i+1-1),1]=="u")){

REJECT;

}

if((h5[2,(i+1),1]=="u") && (h3[12,(len3-i+1-1),1]=="g")){

REJECT;

}

}

if((h5[2,i,1]=="u") && (h3[12,(len3-i+1),1]=="g")){

gu_ug++;

if((h5[2,(i+1),1]=="g") && (h3[12,(len3-i+1-1),1]=="u")){

REJECT;

}

if((h5[2,(i+1),1]=="u") && (h3[12,(len3-i+1-1),1]=="g")){

REJECT;

}

}

i++;

}

if((h5[2,len3,1]=="g") && (h3[12,1,1]=="u")){

gu_ug++;

}

if((h5[2,len3,1]=="u") && (h3[12,1,1]=="g")){

gu_ug++;

}

i=1;

while(i<=(len1-1)){

if((h5[4,i,1]=="g") && (h3[6,(len1-i+1),1]=="u")){

gu_ug++;

if((h5[4,(i+1),1]=="g") && (h3[6,(len1-i+1-1),1]=="u")){

REJECT;

}

if((h5[4,(i+1),1]=="u") && (h3[6,(len1-i+1-1),1]=="g")){

REJECT;

}

}

if((h5[4,i,1]=="u") && (h3[6,(len1-i+1),1]=="g")){

gu_ug++;

if((h5[4,(i+1),1]=="g") && (h3[6,(len1-i+1-1),1]=="u")){

REJECT;

}

if((h5[4,(i+1),1]=="u") && (h3[6,(len1-i+1-1),1]=="g")){

REJECT;

}

}

i++;

}

if((h5[4,len1,1]=="g") && (h3[6,1,1]=="u")){

gu_ug++;

}

if((h5[4,len1,1]=="u") && (h3[6,1,1]=="g")){

gu_ug++;

}

i=1;

while(i<=(len2-1)){

if((h5[8,i,1]=="g") && (h3[10,(len2-i+1),1]=="u")){

gu_ug++;

if((h5[8,(i+1),1]=="g") && (h3[10,(len2-i+1-1),1]=="u")){

REJECT;

}

if((h5[8,(i+1),1]=="u") && (h3[10,(len2-i+1-1),1]=="g")){

REJECT;

}

}

if((h5[8,i,1]=="u") && (h3[10,(len2-i+1),1]=="g")){

gu_ug++;

if((h5[8,(i+1),1]=="g") && (h3[10,(len2-i+1-1),1]=="u")){

REJECT;

}

if((h5[8,(i+1),1]=="u") && (h3[10,(len2-i+1-1),1]=="g")){

REJECT;

}

}

i++;

}

if((h5[8,len2,1]=="g") && (h3[10,1,1]=="u")){

gu_ug++;

}

if((h5[8,len2,1]=="u") && (h3[10,1,1]=="g")){

gu_ug++;

}

total_bonus = total_bonus + gu_ug;

if(total_bonus > -7){

REJECT;

}

SCORE = SCORE + total_bonus;

}
