# Supplementary material for: Identification of Hammerhead Ribozymes in All Domains of Life Reveals Novel Structural Variations
Source: PLoS Comput Biol. 2011 May 5;7(5):e1002031. doi: 10.1371/journal.pcbi.1002031 (PMC3088659; doi:10.1371/journal.pcbi.1002031)
Supplement: Figure S8 — Repeat-type putative hammerhead ribozyme arrangement and dimeric conformation. Secondary structures of a putative single hammerhead ribozyme of Aedes aegypti (A) and hypothetical dimeric conformation (B). Yellow box highlights loop III that can be completely base paired in a dimeric ribozyme (to form a 10 bp long stem). (B) Blue nucleotides correspond to one ribozyme and black to the other ribozyme. Green nucleotides correspond to one of the most common variants observed. (C) Palindromic character of stem III and loop III sequences for several ribozymes. The vast majority of these sequences would allow base-pairing in a dimeric hammerhead conformation like it has previously been described for ASBVd and newt hammerhead ribozymes [71]. Yellow shading as in (A). (PDF) [file pcbi.1002031.s008.pdf]

A

B

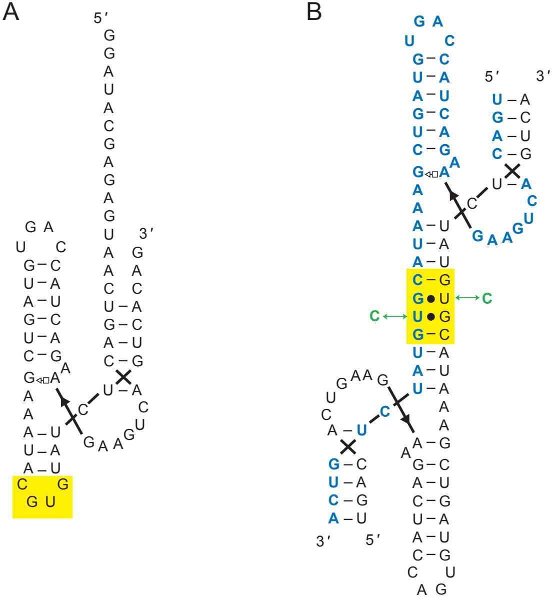


C

| | stem III | loop III | stem III |
|---|---|---|---|
| *Schistosoma mansoni* | A C<br>A C | C G C G<br>G G C C | G U<br>G U |
| *Aedes aegypti* | A U A<br>A U A | C G C G<br>G G C C | U A U<br>U A U |
| *Nematostella vectensis* | A C | A G C U | G U |
| *Xenopus tropicalis* | A | C C G G | U |
