# Supplementary material for: Identification of Hammerhead Ribozymes in All Domains of Life Reveals Novel Structural Variations
Source: PLoS Comput Biol. 2011 May 5;7(5):e1002031. doi: 10.1371/journal.pcbi.1002031 (PMC3088659; doi:10.1371/journal.pcbi.1002031)
Supplement: Dataset S1 — Multiple sequence alignments of all hammerhead sequences. (PDF) [file pcbi.1002031.s012.pdf]

Supplementary data for:
Identification of hammerhead ribozymes in all domains of life reveals novel structural variations

Jonathan Perreault[1], Zasha Weinberg[1,2], Adam Roth[1,2], Olivia Popescu[4], Pascal Chartrand[4], Gerardo Ferbeyre[4] & Ronald R. Breaker[1−3]

[1]Department of Molecular, Cellular and Developmental Biology, [2]Howard Hughes Medical Institute, [3]Department of Molecular Biophysics and Biochemistry, Yale University, Box 208103, New Haven, CT 06520-8103, USA. [4]Department of Biochemistry, Université de Montréal, 2900 Edouard-Montpetit, Montréal, Québec, Canada H3C 3J7.

Note: the presentation of hammerhead ribozyme alignments in this supplementary data follows the pattern of our presentation of previously found conserved RNA motifs [5].

Contents

1 Type I hammerhead ribozymes **2**
1.1 Taxa . . . 2
1.2 Nucleotide coordinates . . . 3
1.3 Multiple-sequence alignment . . . 651

2 Type I hammerhead ribozymes, *Dolichopoda* **975**
2.1 Taxa . . . 975
2.2 Nucleotide coordinates . . . 976
2.3 Multiple-sequence alignment . . . 980

3 Type II hammerhead ribozymes **982**
3.1 Taxa . . . 982
3.2 Nucleotide coordinates . . . 984
3.3 Multiple-sequence alignment . . . 990

4 Type III hammerhead ribozymes **999**
4.1 Taxa . . . 999
4.2 Nucleotide coordinates . . . 1001
4.3 Multiple-sequence alignment . . . 1009

1 Type I hammerhead ribozymes

1.1 Taxa

The taxonomy of each organism containing a putative type I hammerhead ribozyme is listed, with abbreviations identifying each hit (e.g., "Eco-1-1" and "Eco-1-2" might hypothetically represent two distinct RNAs in *E. coli*). The abbreviations will be used to identify each individual type I hammerhead ribozyme in Sections 1.2 and 1.3.

**abbrev. of hits**	**taxonomy of species**
Csy-1-1 to Csy-1-2	Archaea Thaumarchaeota Cenarchaeales Cenarchaeaceae *Cenarchaeum symbiosum* A unordered unplaced scaffolds
Csp-1-1 to Csp-1-2	Bacteria Cyanobacteria Chroococcales *Cyanothece sp.* PCC 8802
Ama-1-1 to Ama-1-2	Bacteria Cyanobacteria Oscillatoriales *Arthrospira maxima* CS-328
Mch-1-1 to Mch-1-2	Bacteria Cyanobacteria Oscillatoriales *Microcoleus chthonoplastes* PCC 7420
Cpa-1-1	Bacteria Firmicutes Clostridia Clostridiales Clostridiaceae *Clostridium papyrosolvens* DSM 2782
Cpe-1-1	Bacteria Firmicutes Clostridia Clostridiales Clostridiaceae *Clostridium perfringens* ATCC 13124
Cpe-2-1	Bacteria Firmicutes Clostridia Clostridiales Clostridiaceae *Clostridium perfringens* CPE str. F4969
Cpe-3-1	Bacteria Firmicutes Clostridia Clostridiales Clostridiaceae *Clostridium perfringens* D str. JGS1721
Cpe-4-1	Bacteria Firmicutes Clostridia Clostridiales Clostridiaceae *Clostridium perfringens* E str. JGS1987
Cpe-5-1	Bacteria Firmicutes Clostridia Clostridiales Clostridiaceae *Clostridium perfringens* NCTC 8239
Cpe-6-1	Bacteria Firmicutes Clostridia Clostridiales Clostridiaceae *Clostridium perfringens str.* 13
Dac-1-1 to Dac-1-2	Bacteria Firmicutes Clostridia Clostridiales Peptococcaceae *Desulfotomaculum acetoxidans* DSM 771
Dre-1-1 to Dre-1-3	Bacteria Firmicutes Clostridia Clostridiales Peptococcaceae *Desulfotomaculum reducens* MI-1
Fpr-1-1 to Fpr-1-2	Bacteria Firmicutes Clostridia Clostridiales Ruminococcaceae *Faecalibacterium prausnitzii* M21/2
Lva-1-1 to Lva-1-2	Bacteria Firmicutes Lactobacillales Lactobacillaceae *Lactobacillus vaginalis* ATCC 49540
Tsp-1-1	Bacteria Proteobacteria Alphaproteobacteria Rhodobacterales Rhodobacteraceae Thalassobium *Thalassiobium sp.* R2A62
Gox-1-1	Bacteria Proteobacteria Alphaproteobacteria Rhodospirillales Acetobacteraceae *Gluconobacter oxydans* 621H
Ldr-1-1 to Ldr-1-18	Bacteria Proteobacteria Gammaproteobacteria Legionellales Legionellaceae *Legionella drancourtii* LLAP12
Lpn-1-1 to Lpn-1-5	Bacteria Proteobacteria Gammaproteobacteria Legionellales Legionellaceae *Legionella pneumophila str.* Corby
Lpn-2-1 to Lpn-2-3	Bacteria Proteobacteria Gammaproteobacteria Legionellales Legionellaceae *Legionella pneumophila str.* Lens
Lpn-3-1 to Lpn-3-4	Bacteria Proteobacteria Gammaproteobacteria Legionellales Legionellaceae *Legionella pneumophila str.* Paris
Lpn-4-1 to Lpn-4-2	Bacteria Proteobacteria Gammaproteobacteria Legionellales Legionellaceae *Legionella pneumophila subsp. pneumophila str.* Philadelphia 1
Ehi-1-1 to Ehi-1-3	Eukaryota Amoebozoa Archamoebae Entamoebidae *Entamoeba histolytica* HM-1:IMSS
Tcr-1-1 to Tcr-1-4	Eukaryota Euglenozoa Kinetoplastida Trypanosomatidae Trypanosoma Schizotrypanum *Trypanosoma cruzi strain* CL Brener
Nfi-1-1	Eukaryota Fungi Dikarya Ascomycota Pezizomycotina Eurotiomycetes Eurotiomycetidae Eurotiales Trichocomaceae *Neosartorya fischeri* NRRL 181
Aca-1-1	Eukaryota Fungi Dikarya Ascomycota Pezizomycotina Eurotiomycetes Eurotiomycetidae Onygenales Ajellomycetaceae *Ajellomyces capsulatus* NAm1
Yli-1-1 to Yli-1-13	Eukaryota Fungi Dikarya Ascomycota Saccharomycotina Saccharomycetes Saccharomycetales Dipodascaceae *Yarrowia lipolytica* CLIB122
Lbi-1-1 to Lbi-1-3	Eukaryota Fungi Dikarya Basidiomycota Agaricomycotina Agaricomycetes Agaricomycetidae Agaricales Tricholomataceae *Laccaria bicolor* S238N-H82
Ppl-1-1 to Ppl-1-39	Eukaryota Fungi Dikarya Basidiomycota Agaricomycotina Agaricomycetes Polyporales *Postia placenta* Mad-698-R
Isc-1-1 to Isc-1-55	Eukaryota Metazoa Arthropoda Chelicerata Arachnida Acari Parasitiformes Ixodida Ixodoidea Ixodidae Ixodinae *Ixodes scapularis*
Tca-1-1 to Tca-1-14	Eukaryota Metazoa Arthropoda Hexapoda Insecta Pterygota Neoptera Endopterygota Coleoptera Polyphaga Cucujiformia Tenebrionidae *Tribolium castaneum*
Aae-1-1 to Aae-1-4346	Eukaryota Metazoa Arthropoda Hexapoda Insecta Pterygota Neoptera Endopterygota Diptera Nematocera Culicoidea Culicidae Culicinae Culicini Aedes Stegomyia *Aedes aegypti*

Cqu-1-1 to Cqu-1-18	Eukaryota Metazoa Arthropoda Hexapoda Insecta Pterygota Neoptera Endopterygota Diptera Nematocera Culicoidea Culicidae Culicinae Culicini Culex *Culex quinquefasciatus*
Bfl-1-1 to Bfl-1-141	Eukaryota Metazoa Chordata Cephalochordata Branchiostomidae *Branchiostoma floridae*
Gga-1-1 to Gga-1-4	Eukaryota Metazoa Chordata Craniata Vertebrata Euteleostomi Archosauria Dinosauria Saurischia Theropoda Coelurosauria Aves Neognathae Galliformes Phasianidae Phasianinae *Gallus gallus*
Tgu-1-1 to Tgu-1-3	Eukaryota Metazoa Chordata Craniata Vertebrata Euteleostomi Archosauria Dinosauria Saurischia Theropoda Coelurosauria Aves Neognathae Passeriformes Passeroidea Estrildidae Estrildinae *Taeniopygia guttata*
Mmu-1-1 to Mmu-1-34	Eukaryota Metazoa Chordata Craniata Vertebrata Euteleostomi Mammalia Eutheria Euarchontoglires Glires Rodentia Sciurognathi Muroidea Muridae Murinae *Mus musculus*
Rno-1-1 to Rno-1-24	Eukaryota Metazoa Chordata Craniata Vertebrata Euteleostomi Mammalia Eutheria Euarchontoglires Glires Rodentia Sciurognathi Muroidea Muridae Murinae *Rattus norvegicus*
Mmu-2-1 to Mmu-2-13	Eukaryota Metazoa Chordata Craniata Vertebrata Euteleostomi Mammalia Eutheria Euarchontoglires Primates Haplorrhini Catarrhini Cercopithecidae Cercopithecinae *Macaca mulatta*
Hsa-1-1 to Hsa-1-31	Eukaryota Metazoa Chordata Craniata Vertebrata Euteleostomi Mammalia Eutheria Euarchontoglires Primates Haplorrhini Catarrhini Hominidae *Homo sapiens*
Ptr-1-1 to Ptr-1-14	Eukaryota Metazoa Chordata Craniata Vertebrata Euteleostomi Mammalia Eutheria Euarchontoglires Primates Haplorrhini Catarrhini Hominidae *Pan troglodytes*
Mmu-3-1	Eukaryota Metazoa Chordata Craniata Vertebrata Euteleostomi Mammalia Eutheria Euarchontoglires Primates Strepsirrhini Lemuriformes Cheirogaleidae *Microcebus murinus*
Clu-1-1 to Clu-1-4	Eukaryota Metazoa Chordata Craniata Vertebrata Euteleostomi Mammalia Eutheria Laurasiatheria Carnivora Caniformia Canidae *Canis lupus familiaris*
Bta-1-1 to Bta-1-6	Eukaryota Metazoa Chordata Craniata Vertebrata Euteleostomi Mammalia Eutheria Laurasiatheria Cetartiodactyla Ruminantia Pecora Bovidae Bovinae *Bos taurus*
Ssc-1-1 to Ssc-1-4	Eukaryota Metazoa Chordata Craniata Vertebrata Euteleostomi Mammalia Eutheria Laurasiatheria Cetartiodactyla Suina Suidae *Sus scrofa*
Eca-1-1 to Eca-1-18	Eukaryota Metazoa Chordata Craniata Vertebrata Euteleostomi Mammalia Eutheria Laurasiatheria Perissodactyla Equidae *Equus caballus*
Mdo-1-1 to Mdo-1-26	Eukaryota Metazoa Chordata Craniata Vertebrata Euteleostomi Mammalia Metatheria Didelphimorphia Didelphidae *Monodelphis domestica*
Oan-1-1 to Oan-1-3	Eukaryota Metazoa Chordata Craniata Vertebrata Euteleostomi Mammalia Monotremata Ornithorhynchidae *Ornithorhynchus anatinus*
Nve-1-1 to Nve-1-472	Eukaryota Metazoa Cnidaria Anthozoa Hexacorallia Actiniaria Edwardsiidae *Nematostella vectensis*
Hma-1-1 to Hma-1-8	Eukaryota Metazoa Cnidaria Hydrozoa Hydroida Anthomedusae Hydridae *Hydra magnipapillata*
Spu-1-1 to Spu-1-2	Eukaryota Metazoa Echinodermata Eleutherozoa Echinozoa Echinoidea Euechinoidea Echinacea Echinoida Strongylocentrotidae *Strongylocentrotus purpuratus*
Cbr-1-1	Eukaryota Metazoa Nematoda Chromadorea Rhabditida Rhabditoidea Rhabditidae Peloderinae *Caenorhabditis briggsae*
Sma-1-1 to Sma-1-25348	Eukaryota Metazoa Platyhelminthes Trematoda Digenea Strigeidida Schistosomatoidea Schistosomatidae *Schistosoma mansoni*
Cre-1-1	Eukaryota Viridiplantae Chlorophyta Chlorophyceae Chlamydomonadales Chlamydomonadaceae *Chlamydomonas reinhardtii*
Pph-1-1	Viruses dsDNA viruses, no RNA stage Caudovirales Podoviridae *Pseudomonas phage* LUZ24
Pph-2-1	Viruses dsDNA viruses, no RNA stage Caudovirales Podoviridae *Pseudomonas phage* PaP3
env-1 to env-860	environmental samples

1.2 Nucleotide coordinates

Each type I hammerhead ribozyme (indicated by "RNA→") is listed.The accession number of the sequence containing each type I hammerhead ribozyme is given. Accessions beginning with "NC_" or "NZ_" are RefSeq, while other accession refer to environmental samples. Nucleotide coordinates are given for the 5′ and 3′ boundaries of each. If the 5′ coordinate is greater than the 3′ coordinate, the RNA is present on the reverse-complement strand of the containing genomic DNA sequence. Each hit is denoted by an abbreviation (like "Eco-1-1") that refers to a taxonomy given in Section 1.1.

abbrev.	Seq. accession		5′ at	3′ at
Cpe-6-1	NC_003366.1	-	1736982	1736923
Cpe-1-1	NC_008261.1	-	1954659	1954600
Cpe-4-1	NZ_ABDW01000059.1	-	340	281
Cpe-2-1	NZ_ABDX01000010.1	-	23618	23559
Cpe-5-1	NZ_ABDY01000005.1	-	88336	88277
Cpe-3-1	NZ_ABOO01000008.1	-	23430	23371
Rno-1-1	NW_047333.1	-	928105	928049
Rno-1-2	NW_001084654.1	-	7145102	7145046
Rno-1-3	AC_000078.1	-	7145102	7145046
Rno-1-4	NC_005109.2	-	8225637	8225581
Eca-1-1	NW_001867386.1	-	23487315	23487244
Eca-1-2	NC_009144.2	-	23487315	23487244
Eca-1-3	NW_001867430.1	-	34593780	34593721
Eca-1-4	NC_009151.2	-	64725704	64725645
env-1	JCVI_SCAF_1096626071977	-	1008	952
Aae-1-1	NW_001811181.1	+	54911	55032
Lbi-1-1	NW_001889892.1	+	660146	660216
Lbi-1-2	NW_001889913.1	-	377972	377902
Lbi-1-3	NW_001890030.1	-	4645	4575
Mdo-1-1	NW_001581960.1	-	1025514	1025454
Mdo-1-2	NC_008804.1	-	68092760	68092700
Mmu-1-1	NT_039455.7	-	10159831	10159779
Mmu-1-2	NW_001030882.1	-	10028774	10028722
Mmu-1-3	AC_000030.1	-	13328150	13328098
Mmu-1-4	NC_000074.5	-	13159831	13159779
Mmu-1-5	NT_039621.7	+	30126088	30126150
Mmu-1-6	NW_001030574.1	+	19245086	19245148
Mmu-1-7	AC_000037.1	+	70529623	70529685
Mmu-1-8	NC_000081.5	+	68840084	68840146
Ptr-1-1	NW_001234074.1	+	27790578	27790647
Ptr-1-2	NC_006471.2	+	110514165	110514234
Hsa-1-1	NT_016354.19	+	32585791	32585860
Hsa-1-2	NW_922162.1	+	55161944	55162013
Hsa-1-3	NW_001838915.1	+	32534370	32534439
Hsa-1-4	NC_000004.11	+	108038070	108038139
Hsa-1-5	AC_000047.1	+	105336970	105337039
Hsa-1-6	AC_000136.1	+	103772820	103772889
Tca-1-1	NC_007422.2	+	17720930	17720989
Tca-1-2	NW_001092856.1	+	919247	919306
Mmu-1-9	NT_096135.5	-	45986807	45986744
Mmu-1-10	NW_001030424.1	-	2351235	2351172
Mmu-1-11	AC_000033.1	-	90306699	90306636
Mmu-1-12	NC_000077.5	-	80481929	80481866
Rno-1-5	NW_047394.1	+	1959724	1959794
Rno-1-6	NW_001084685.1	+	1613621	1613691
Rno-1-7	AC_000081.1	+	40790678	40790748
Rno-1-8	NC_005112.2	+	41847501	41847571
Mmu-2-1	NW_001120965.1	-	6596314	6596239
Mmu-2-2	NC_007863.1	-	53882575	53882500
Eca-1-5	NW_001867402.1	-	33850523	33850466
Eca-1-6	NC_009145.2	-	33850523	33850466

Mdo-1-3	NW_001582019.1	-	3891955	3891896
Mdo-1-4	NC_008808.1	-	128975719	128975660
Mdo-1-5	NW_001581945.1	+	5882622	5882682
Mdo-1-6	NC_008804.1	+	395491444	395491504
Bfl-1-1	NW_002235018.1	+	1403922	1403984
Mmu-2-3	NW_001095188.1	+	354408	354475
Mmu-2-4	NC_007867.1	+	89580790	89580857
env-2	JCVI_SCAF_1096628065006	+	1603	1701
Eca-1-7	NW_001867435.1	-	11661067	11660990
Eca-1-8	NC_009152.2	-	52476376	52476299
Aca-1-1	NW_001813982.1	-	4396550	4396479
Yli-1-1	NC_006069.1	-	3040259	3040150
Aae-1-2	NW_001810458.1	+	771543	771630
env-3	JCVI_SCAF_1096627828063	+	566	624
Mmu-2-5	NW_001124215.1	+	4773788	4773853
Mmu-2-6	NC_007866.1	+	81403896	81403961
env-4	ABMG01116745	+	18	79
Hsa-1-7	NT_008413.18	-	27125614	27125555
Hsa-1-8	NW_924062.1	-	26948290	26948231
Hsa-1-9	NW_001839149.2	+	8780093	8780152
Hsa-1-10	NC_000009.11	-	27135614	27135555
Hsa-1-11	AC_000052.1	-	27063771	27063712
Hsa-1-12	AC_000141.1	-	27088128	27088069
Hsa-1-13	NG_011828.1	-	31468	31409
Ptr-1-3	NW_001240439.1	-	26940286	26940227
Ptr-1-4	NC_006476.2	-	27735512	27735453
Aae-1-3	NW_001811100.1	-	685711	685657
Aae-1-4	NW_001810556.1	-	164829	164761
Rno-1-9	NW_047491.1	-	14469586	14469502
Rno-1-10	NW_001084723.1	-	21650130	21650046
Rno-1-11	AC_000085.1	-	33246296	33246212
Rno-1-12	NC_005116.2	-	40153424	40153340
env-5	AATC01003456	+	575	633
env-6	AATF01005847	+	564	622
Mmu-2-7	NW_001120996.1	-	573348	573290
Mmu-2-8	NC_007863.1	-	167461387	167461329
Eca-1-9	NW_001867383.1	-	9179931	9179865
Eca-1-10	NC_009160.2	-	26931156	26931090
env-7	JCVI_SCAF_1096627611910	+	29	88
Nve-1-1	NW_001834101.1	+	37894	37950
env-8	JCVI_SCAF_1101667066268	-	105	44
Bfl-1-2	NW_002235192.1	-	920381	920330
Bfl-1-3	NW_002235002.1	-	1544210	1544159
Ptr-1-5	NW_001224596.1	+	2857779	2857830
Ptr-1-6	NC_006481.2	+	23923366	23923417
Hsa-1-14	NT_026437.12	+	6474153	6474204
Hsa-1-15	NW_925539.1	+	5280733	5280784
Hsa-1-16	NW_001838110.1	+	5277004	5277055
Hsa-1-17	NC_000014.8	+	25474153	25474204
Hsa-1-18	AC_000057.1	+	5338611	5338662
Hsa-1-19	AC_000146.1	+	5588180	5588231
Lva-1-1	NZ_GG693413.1	-	68804	68721

Lva-1-2	NZ_ACGV01000018.1	-	59000	58917
env-9	JCVI_SCAF_1096626877834	-	112	48
Oan-1-1	NW_001794261.1	-	5909438	5909386
Ptr-1-7	NW_001220645.1	+	4579043	4579123
Ptr-1-8	NC_006477.2	+	14362691	14362771
Nve-1-2	NW_001834151.1	+	29700	29773
env-10	JCVI_SCAF_1101668548346	-	514	449
Ssc-1-1	NW_001885478.1	-	560326	560267
Ssc-1-2	NC_010456.1	-	63994886	63994827
Nve-1-3	NW_001832879.1	-	12544	12493
env-11	JCVI_SCAF_1101667237532	-	103	43
Aae-1-5	NW_001809931.1	-	30773	30721
Mch-1-1	NZ_DS989859.1	-	143982	143921
Mch-1-2	NZ_DS989859.1	-	143751	143690
Mmu-1-13	NT_039169.7	+	6062852	6062898
Mmu-1-14	NC_000067.5	+	9062852	9062898
Mmu-1-15	NT_039606.7	-	54514931	54514885
Mmu-1-16	NW_001030560.1	-	27631387	27631341
Mmu-1-17	AC_000036.1	-	79172763	79172717
Mmu-1-18	NC_000080.5	-	82079887	82079841
Mmu-1-19	NW_001030678.1	-	15271253	15271207
Mmu-1-20	NT_039189.7	-	5959476	5959430
Mmu-1-21	AC_000023.1	-	196378552	196378506
Mmu-1-22	NC_000067.5	-	191287286	191287240
Rno-1-13	NW_047713.2	-	13152649	13152561
Rno-1-14	NW_001084839.1	-	5451615	5451527
Rno-1-15	AC_000073.1	-	71811940	71811852
Rno-1-16	NC_005104.2	-	76190015	76189927
Nve-1-4	NW_001833940.1	+	118684	118758
Nve-1-5	NW_001834402.1	-	1712580	1712531
Bfl-1-4	NW_002235303.1	-	278156	278107
Bfl-1-5	NW_002234937.1	-	1387222	1387150
Bfl-1-6	NW_002235698.1	-	50668	50619
Cbr-1-1	NZ_CAAC02000557.1	+	235864	235932
Bfl-1-7	NW_002235056.1	+	1513921	1513978
env-12	AF404073.1	+	70	162
Yli-1-2	NC_006067.1	-	904542	904463
Cre-1-1	NW_001843559.1	+	8061	8112
Mdo-1-7	NW_001581961.1	-	123960601	123960518
Mdo-1-8	NC_008804.1	-	235356879	235356796
Mdo-1-9	NW_001581837.1	-	102199877	102199829
Mdo-1-10	NC_008801.1	-	393908091	393908043
Mdo-1-11	NW_001581875.1	-	23404358	23404300
Mdo-1-12	NC_008802.1	-	215826348	215826290
Mdo-1-13	NW_001581837.1	+	54493717	54493817
Mdo-1-14	NC_008801.1	+	346201931	346202031
Mdo-1-15	NW_001581968.1	-	12002893	12002791
Mdo-1-16	NC_008805.1	-	74315481	74315379
Isc-1-1	NW_002824129.1	-	72721	72656
env-13	NCBI_READ_1113264282865	-	637	587
env-14	JCVI_SCAF_1101668047413	-	997	930
env-15	JCVI_SCAF_1096627788695	-	761	702

Nve-1-6	NW_001834389.1	+	687022	687076
env-16	JCVI_SCAF_1101667068912	+	589	645
Gox-1-1	NC_006672.1	-	30953	30888
Tca-1-3	NW_001094031.1	-	87520	87471
Tca-1-4	NW_001094218.1	-	5866	5817
Tca-1-5	NC_007423.2	+	15903578	15903627
Tca-1-6	NC_007423.2	-	15901649	15901600
Tca-1-7	NW_001092880.1	+	387573	387622
Tca-1-8	NW_001092880.1	-	385644	385595
env-17	JCVI_SCAF_1101668243010	-	104	44
Eca-1-11	NW_001867411.1	-	41716761	41716704
Eca-1-12	NC_009146.2	-	78308854	78308797
env-18	ABMJ01021607	+	18	83
Hma-1-1	NW_002158257.1	+	3410	3458
env-19	JCVI_SCAF_1096626865527	+	904	980
Dre-1-1	NC_009253.1	+	936444	936503
Dre-1-2	NC_009253.1	+	949822	949881
env-20	JCVI_SCAF_1096627143383	-	1314	1250
Tsp-1-1	NZ_GG697169.2	+	1013719	1013788
env-21	AATD01009237	-	532	476
env-22	ABOF01011406	+	59	114
env-23	ABOF01029488	-	72	17
env-24	ABOF01069025	+	11	68
env-25	ABOF01085622	+	11	70
Csp-1-1	NC_013161.1	+	4417484	4417544
Csp-1-2	NC_013161.1	-	2113048	2112988
env-26	JCVI_SCAF_1096627138737	+	1289	1347
Spu-1-1	NW_001336952.1	+	240937	240982
Spu-1-2	NW_001460378.1	-	448095	448050
Mmu-2-9	NW_001112573.1	+	8745039	8745101
Mmu-2-10	NC_007859.1	+	174924195	174924257
env-27	ABEF01004833.1	+	538	592
Nve-1-7	NW_001834191.1	-	53497	53451
Nve-1-8	NW_001834276.1	-	439310	439262
Nve-1-9	NW_001834169.1	-	281815	281767
Nve-1-10	NW_001834206.1	-	139606	139558
Nve-1-11	NW_001832211.1	+	5486	5534
Nve-1-12	NW_001834353.1	-	96753	96705
Nve-1-13	NW_001833676.1	-	40169	40117
Nve-1-14	NW_001833879.1	+	23149	23201
Nve-1-15	NW_001832688.1	-	6977	6929
Nve-1-16	NW_001834177.1	-	112975	112927
Nve-1-17	NW_001834177.1	-	110507	110459
Nve-1-18	NW_001834186.1	-	105909	105861
Nve-1-19	NW_001834203.1	-	147832	147784
Nve-1-20	NW_001834365.1	-	465701	465653
Nve-1-21	NW_001834411.1	-	523280	523232
Nve-1-22	NW_001834408.1	+	295362	295410
Nve-1-23	NW_001834346.1	+	314703	314751
Nve-1-24	NW_001834329.1	+	93558	93606
Nve-1-25	NW_001834297.1	-	446454	446406
Nve-1-26	NW_001834382.1	-	69837	69789

Nve-1-27	NW_001834038.1	+	27186	27234
Nve-1-28	NW_001834401.1	+	235899	235947
Nve-1-29	NW_001834260.1	-	327050	327002
Nve-1-30	NW_001834272.1	+	401494	401542
Nve-1-31	NW_001834033.1	+	34539	34587
Nve-1-32	NW_001834348.1	+	965007	965055
Nve-1-33	NW_001833924.1	-	27842	27794
Nve-1-34	NW_001834158.1	+	300618	300666
Nve-1-35	NW_001834207.1	+	57374	57422
Nve-1-36	NW_001834318.1	-	92780	92732
Nve-1-37	NW_001833878.1	-	50705	50657
Nve-1-38	NW_001834118.1	+	101421	101469
Nve-1-39	NW_001834118.1	+	107965	108013
Nve-1-40	NW_001827127.1	+	679	727
Nve-1-41	NW_001829216.1	-	342	294
Nve-1-42	NW_001832725.1	+	1884	1932
Nve-1-43	NW_001833119.1	+	25151	25199
Nve-1-44	NW_001833259.1	+	716	764
Nve-1-45	NW_001833893.1	-	58171	58123
Nve-1-46	NW_001833893.1	-	53819	53771
Nve-1-47	NW_001833934.1	+	73678	73726
Nve-1-48	NW_001833960.1	+	74840	74888
Nve-1-49	NW_001833965.1	-	101421	101373
Nve-1-50	NW_001833984.1	-	133825	133777
Nve-1-51	NW_001834019.1	-	136702	136654
Nve-1-52	NW_001834021.1	+	112378	112426
Nve-1-53	NW_001834021.1	+	119897	119945
Nve-1-54	NW_001834029.1	+	97437	97485
Nve-1-55	NW_001834029.1	-	97367	97319
Nve-1-56	NW_001834033.1	+	12618	12666
Nve-1-57	NW_001834043.1	-	66703	66655
Nve-1-58	NW_001834065.1	+	35426	35474
Nve-1-59	NW_001834067.1	+	8299	8347
Nve-1-60	NW_001834095.1	+	170931	170979
Nve-1-61	NW_001834107.1	+	221670	221718
Nve-1-62	NW_001834162.1	-	126344	126296
Nve-1-63	NW_001834164.1	+	109286	109334
Nve-1-64	NW_001834175.1	-	35664	35616
Nve-1-65	NW_001834180.1	+	146551	146599
Nve-1-66	NW_001834208.1	-	148078	148030
Nve-1-67	NW_001834225.1	-	355330	355282
Nve-1-68	NW_001834231.1	-	20406	20358
Nve-1-69	NW_001834245.1	-	264280	264232
Nve-1-70	NW_001834251.1	-	184251	184203
Nve-1-71	NW_001834260.1	+	313709	313757
Nve-1-72	NW_001834262.1	-	67149	67101
Nve-1-73	NW_001834288.1	-	105828	105780
Nve-1-74	NW_001834293.1	-	624154	624106
Nve-1-75	NW_001834299.1	+	70813	70861
Nve-1-76	NW_001834307.1	-	687855	687807
Nve-1-77	NW_001834315.1	-	63597	63549
Nve-1-78	NW_001834322.1	+	231546	231594

Nve-1-79	NW_001834330.1	-	819773	819725
Nve-1-80	NW_001834354.1	-	374285	374237
Nve-1-81	NW_001834365.1	+	466820	466868
Nve-1-82	NW_001834369.1	-	912884	912836
Nve-1-83	NW_001834371.1	-	1147366	1147318
Nve-1-84	NW_001834377.1	-	196301	196253
Nve-1-85	NW_001834381.1	+	1163611	1163659
Nve-1-86	NW_001834386.1	+	28169	28217
Nve-1-87	NW_001834387.1	-	610041	609993
Nve-1-88	NW_001834387.1	-	604699	604651
Nve-1-89	NW_001834389.1	+	68403	68451
Nve-1-90	NW_001834390.1	+	1425429	1425477
Nve-1-91	NW_001834391.1	+	1110891	1110939
Nve-1-92	NW_001834391.1	+	1568257	1568305
Nve-1-93	NW_001834391.1	-	329932	329884
Nve-1-94	NW_001834393.1	-	1246968	1246920
Nve-1-95	NW_001834400.1	-	442413	442365
Nve-1-96	NW_001834404.1	+	99281	99329
Nve-1-97	NW_001834405.1	+	320938	320986
Nve-1-98	NW_001834406.1	-	976622	976574
Nve-1-99	NW_001834411.1	-	2333090	2333042
Nve-1-100	NW_001834411.1	-	1606372	1606324
Nve-1-101	NW_001834413.1	+	2026980	2027028
Nve-1-102	NW_001834413.1	+	2627025	2627073
Nve-1-103	NW_001834359.1	+	816147	816195
Nve-1-104	NW_001834359.1	-	176822	176774
Nve-1-105	NW_001834407.1	+	845580	845637
Nve-1-106	NW_001834410.1	+	2240979	2241028
Nve-1-107	NW_001832034.1	+	2398	2446
Nve-1-108	NW_001834377.1	-	220011	219963
Nve-1-109	NW_001834049.1	+	143154	143202
Nve-1-110	NW_001834187.1	-	86617	86569
Nve-1-111	NW_001834411.1	-	1530887	1530839
Nve-1-112	NW_001833545.1	+	1388	1436
Nve-1-113	NW_001833861.1	+	98460	98508
Nve-1-114	NW_001834289.1	-	54828	54766
Nve-1-115	NW_001833955.1	-	9139	9077
Nve-1-116	NW_001834216.1	-	413097	413035
Nve-1-117	NW_001833993.1	-	30617	30556
Nve-1-118	NW_001834413.1	-	2219508	2219446
Nve-1-119	NW_001834107.1	+	221723	221769
Nve-1-120	NW_001831903.1	+	7624	7688
Nve-1-121	NW_001834340.1	+	351469	351531
Nve-1-122	NW_001834358.1	+	511716	511778
Nve-1-123	NW_001834129.1	+	1963	2025
Nve-1-124	NW_001833676.1	-	38534	38472
Nve-1-125	NW_001830649.1	+	8133	8195
Nve-1-126	NW_001834198.1	+	135861	135923
Nve-1-127	NW_001834311.1	+	634612	634674
Nve-1-128	NW_001834405.1	-	1735942	1735880
Nve-1-129	NW_001834393.1	-	1349745	1349683
Nve-1-130	NW_001834360.1	-	254958	254896

Nve-1-131	NW_001834395.1	+	1233585	1233647
Nve-1-132	NW_001834372.1	+	1088730	1088787
Nve-1-133	NW_001834393.1	+	6773	6832
Nve-1-134	NW_001834413.1	-	2221116	2221061
env-28	Z69686.1	+	342	390
env-29	Z69687.1	+	342	390
env-30	Z69696.1	+	341	389
env-31	Z69695.1	+	345	393
env-32	Z69690.1	+	342	390
env-33	Z69692.1	+	343	391
env-34	Z69702.1	+	341	389
env-35	Z69685.1	+	342	390
env-36	Z69698.1	+	341	389
env-37	Z69703.1	+	343	391
env-38	Z69691.1	+	342	390
Bfl-1-8	NW_002236005.1	+	44684	44733
Bfl-1-9	NW_002235311.1	-	594286	594233
Bfl-1-10	NW_002235437.1	+	478966	479019
Bfl-1-11	NW_002236089.1	+	23529	23582
Bfl-1-12	NW_002235437.1	-	478397	478344
Bfl-1-13	NW_002234988.1	+	1729540	1729593
Bfl-1-14	NW_002235496.1	+	3730	3783
Bfl-1-15	NW_002234960.1	+	55181	55234
Bfl-1-16	NW_002234936.1	+	5855582	5855635
Bfl-1-17	NW_002234936.1	-	5855262	5855209
Bfl-1-18	NW_002234941.1	+	2988548	2988601
Bfl-1-19	NW_002234943.1	+	2360458	2360511
Bfl-1-20	NW_002234943.1	-	2360111	2360058
Bfl-1-21	NW_002234950.1	+	3266742	3266795
Bfl-1-22	NW_002234951.1	+	858421	858474
Bfl-1-23	NW_002234951.1	-	855884	855831
Bfl-1-24	NW_002234962.1	+	134701	134754
Bfl-1-25	NW_002234966.1	+	3468489	3468542
Bfl-1-26	NW_002234966.1	+	3484538	3484591
Bfl-1-27	NW_002234966.1	-	3484261	3484208
Bfl-1-28	NW_002234966.1	-	3468057	3468004
Bfl-1-29	NW_002234990.1	+	1184892	1184945
Bfl-1-30	NW_002234990.1	-	1184339	1184286
Bfl-1-31	NW_002234996.1	-	803314	803261
Bfl-1-32	NW_002235000.1	-	2621412	2621359
Bfl-1-33	NW_002235005.1	+	2065994	2066047
Bfl-1-34	NW_002235005.1	-	2065518	2065465
Bfl-1-35	NW_002235062.1	-	345925	345872
Bfl-1-36	NW_002235062.1	-	139997	139944
Bfl-1-37	NW_002235069.1	-	814596	814543
Bfl-1-38	NW_002235091.1	+	538183	538236
Bfl-1-39	NW_002235103.1	+	1178773	1178826
Bfl-1-40	NW_002235103.1	-	1178299	1178246
Bfl-1-41	NW_002235162.1	-	1161142	1161089
Bfl-1-42	NW_002235184.1	+	317310	317363
Bfl-1-43	NW_002235184.1	-	316547	316494
Bfl-1-44	NW_002235197.1	+	585809	585862

Bfl-1-45	NW_002235284.1	-	820065	820012
Bfl-1-46	NW_002235300.1	+	231143	231196
Bfl-1-47	NW_002235300.1	+	625632	625685
Bfl-1-48	NW_002235300.1	-	625277	625224
Bfl-1-49	NW_002235300.1	-	230321	230268
Bfl-1-50	NW_002235328.1	+	629857	629910
Bfl-1-51	NW_002235328.1	-	628933	628880
Bfl-1-52	NW_002235400.1	-	436290	436237
Bfl-1-53	NW_002235422.1	+	320909	320962
Bfl-1-54	NW_002235422.1	-	320412	320359
Bfl-1-55	NW_002235433.1	-	231678	231625
Bfl-1-56	NW_002235462.1	+	488360	488413
Bfl-1-57	NW_002235496.1	+	939	992
Bfl-1-58	NW_002235527.1	-	140518	140465
Bfl-1-59	NW_002235663.1	+	110059	110112
Bfl-1-60	NW_002235679.1	+	165848	165901
Bfl-1-61	NW_002234942.1	-	4219484	4219431
Bfl-1-62	NW_002234949.1	-	2643199	2643146
Bfl-1-63	NW_002234950.1	-	3195161	3195108
Bfl-1-64	NW_002234961.1	-	1716607	1716554
Bfl-1-65	NW_002235012.1	+	2348094	2348147
Bfl-1-66	NW_002235037.1	+	664114	664167
Bfl-1-67	NW_002235047.1	-	664946	664893
Bfl-1-68	NW_002235183.1	-	379197	379144
Bfl-1-69	NW_002235299.1	-	251543	251490
Bfl-1-70	NW_002235377.1	+	114629	114682
Bfl-1-71	NW_002235417.1	-	512307	512254
Bfl-1-72	NW_002235584.1	+	85278	85331
Bfl-1-73	NW_002235822.1	+	9795	9856
Mdo-1-17	NW_001581877.1	-	1021230	1021175
Mdo-1-18	NC_008802.1	-	260720888	260720833
env-39	JCVI_SCAF_1096627765100	-	718	666
env-40	JCVI_SCAF_1096628308131	+	1352	1457
env-41	JCVI_SCAF_1101667233515	-	804	738
Aae-1-6	NW_001810728.1	+	662525	662577
Aae-1-7	NW_001809814.1	+	208806	208858
Aae-1-8	NW_001810000.1	-	1944759	1944707
Aae-1-9	NW_001810715.1	+	579652	579704
Aae-1-10	NW_001810715.1	-	579402	579350
Aae-1-11	NW_001810872.1	-	1727043	1726991
Aae-1-12	NW_001810913.1	-	272659	272607
Aae-1-13	NW_001811068.1	+	725511	725563
Aae-1-14	NW_001811124.1	+	322459	322511
Aae-1-15	NW_001811174.1	-	111733	111681
Aae-1-16	NW_001811240.1	+	375957	376009
Aae-1-17	NW_001811404.1	-	213265	213213
Aae-1-18	NW_001811518.1	+	377904	377956
Aae-1-19	NW_001811518.1	-	374223	374171
Aae-1-20	NW_001811696.1	+	1157007	1157059
Aae-1-21	NW_001810211.1	+	283549	283601
Aae-1-22	NW_001810211.1	-	283055	283003
Aae-1-23	NW_001811155.1	+	1123872	1123924

Aae-1-24	NW_001811376.1	-	98026	97974
Aae-1-25	NW_001811400.1	+	506291	506343
env-42	JCVI_SCAF_1096626888098	-	1301	1237
env-43	JCVI_SCAF_1096627423712	+	1356	1431
env-44	JCVI_SCAF_1096627423712	+	995	1060
env-45	NCBI_BBAY_READ_1106627149515	-	810	761
Pph-1-1	NC_010325.1	+	2407	2488
Pph-2-1	NC_004466.2	-	44111	44036
env-46	NCBI_READ_1113235566994	+	145	205
env-47	NCBI_READ_1113288819750	+	180	239
env-48	contig08285	+	1309	1371
env-49	JCVI_SCAF_1101668256158	+	54	113
env-50	ABDH01049587.1	+	351	407
Cpa-1-1	NZ_ACXX01000006.1	+	105453	105513
Ama-1-1	NZ_ABYK01000001.1	-	262331	262260
Ama-1-2	NZ_ABYK01000023.1	+	70304	70375
Mdo-1-19	NW_001581881.1	-	9305574	9305487
Mdo-1-20	NC_008802.1	-	501298565	501298478
env-51	ABLW01052804	+	33	102
env-52	soil_Contig11781	+	563	614
env-53	hgutS7_s7_171656	+	1196	1248
env-54	BAAZ01002540.1	-	1661	1595
Fpr-1-1	NZ_DS483500.1	+	205812	205885
Fpr-1-2	NZ_ABED02000025.1	+	205812	205885
env-55	JCVI_SCAF_1096627077510	-	1410	1348
env-56	JCVI_SCAF_1096627928495	+	259	320
env-57	hgutS8_s8_173201	-	2943	2878
env-58	hgutS7_s7_177175	-	368	291
env-59	hgutS7_s7_171656	+	416	469
env-60	hgutS7_s7_171656	+	78	131
Ptr-1-9	NW_001228236.1	+	3127644	3127702
Ptr-1-10	NC_006486.2	+	52179577	52179635
Mmu-1-23	NT_039240.7	+	19455284	19455364
Mmu-1-24	NW_001030721.1	+	5692847	5692927
Mmu-1-25	AC_000025.1	+	70376528	70376608
Mmu-1-26	NC_000069.5	+	70072931	70073011
Tca-1-9	NC_007418.2	+	11907167	11907226
Tca-1-10	NW_001092796.1	+	1376853	1376912
Rno-1-17	NW_047625.2	-	34595653	34595568
Rno-1-18	NW_001084804.1	-	9312707	9312622
Rno-1-19	AC_000070.1	-	137553720	137553635
Rno-1-20	NC_005101.2	-	148238483	148238398
Aae-1-26	NW_001810813.1	+	97811	97864
Aae-1-27	NW_001811223.1	-	83616	83563
Ppl-1-1	NW_002988900.1	+	28360	28432
Ppl-1-2	NW_002989076.1	-	64620	64548
Ppl-1-3	NW_002989222.1	+	2801	2873
Ppl-1-4	NW_002989069.1	+	62864	62936
Ppl-1-5	NW_002989957.1	+	255	327
Ppl-1-6	NW_002989048.1	-	156246	156174
Ppl-1-7	NW_002988888.1	-	613649	613577
Ppl-1-8	NW_002988940.1	+	38876	38948

Ppl-1-9	NW_002989037.1	-	165379	165307
Ppl-1-10	NW_002989042.1	+	824	896
Ppl-1-11	NW_002989096.1	-	126312	126240
Ppl-1-12	NW_002989855.1	+	453	525
Ppl-1-13	NW_002988904.1	-	507416	507344
Ppl-1-14	NW_002988919.1	+	1663	1735
Ppl-1-15	NW_002988940.1	+	43410	43482
Ppl-1-16	NW_002989037.1	-	164859	164787
Ppl-1-17	NW_002989058.1	-	167420	167348
Ppl-1-18	NW_002989099.1	+	4664	4736
Ppl-1-19	NW_002989702.1	-	8052	7980
Ppl-1-20	NW_002989814.1	-	1376	1304
Ppl-1-21	NW_002988969.1	+	376	448
Ppl-1-22	NW_002988987.1	-	243392	243320
Ppl-1-23	NW_002989096.1	-	131588	131516
Ppl-1-24	NW_002989165.1	+	620	692
Ppl-1-25	NW_002989165.1	+	29269	29341
Ppl-1-26	NW_002988984.1	-	271146	271074
Ppl-1-27	NW_002989069.1	+	57645	57718
Ppl-1-28	NW_002988903.1	+	284952	285024
Ppl-1-29	NW_002988958.1	+	24741	24813
Ppl-1-30	NW_002988969.1	+	65033	65105
Ppl-1-31	NW_002988992.1	-	168213	168141
Ppl-1-32	NW_002989102.1	+	837	909
Ppl-1-33	NW_002989222.1	+	992	1064
Ppl-1-34	NW_002989793.1	-	1003	931
Ppl-1-35	NW_002990076.1	-	452	380
Ppl-1-36	NW_002988952.1	+	15596	15669
Ppl-1-37	NW_002988958.1	+	25125	25198
Ppl-1-38	NW_002988969.1	+	65417	65490
Ppl-1-39	NW_002989069.1	+	63134	63207
Yli-1-3	NC_006069.1	-	1037930	1037865
Tca-1-11	NC_007418.2	+	16305889	16305936
Tca-1-12	NW_001092797.1	+	1703393	1703440
Tca-1-13	NW_001094240.1	-	115531	115486
Mmu-3-1	NT_166221.1	-	455569	455518
Dre-1-3	NC_009253.1	+	954995	955057
Dac-1-1	NZ_ABTQ01000041.1	-	18223	18159
Dac-1-2	NZ_ABTQ01000041.1	-	17708	17644
Bfl-1-74	NW_002235408.1	+	168220	168268
Bfl-1-75	NW_002234948.1	-	1888622	1888574
Bfl-1-76	NW_002235240.1	-	268407	268359
Bfl-1-77	NW_002235274.1	-	303106	303058
Bfl-1-78	NW_002234954.1	+	1024383	1024431
Bfl-1-79	NW_002234954.1	-	1023447	1023399
Bfl-1-80	NW_002234957.1	+	1549765	1549813
Bfl-1-81	NW_002234957.1	-	1548184	1548136
Bfl-1-82	NW_002234966.1	-	2948031	2947983
Bfl-1-83	NW_002234969.1	+	3372714	3372762
Bfl-1-84	NW_002234974.1	-	142437	142389
Bfl-1-85	NW_002234976.1	+	2767385	2767433
Bfl-1-86	NW_002234976.1	-	2765312	2765264

Bfl-1-87	NW_002234978.1	+	2346925	2346973
Bfl-1-88	NW_002234978.1	-	2346131	2346083
Bfl-1-89	NW_002234988.1	-	1686332	1686284
Bfl-1-90	NW_002234993.1	+	356498	356546
Bfl-1-91	NW_002234998.1	+	1990291	1990339
Bfl-1-92	NW_002234998.1	-	47961	47913
Bfl-1-93	NW_002235016.1	+	1809580	1809628
Bfl-1-94	NW_002235016.1	-	1809273	1809225
Bfl-1-95	NW_002235027.1	+	164410	164458
Bfl-1-96	NW_002235027.1	+	244546	244594
Bfl-1-97	NW_002235027.1	-	243862	243814
Bfl-1-98	NW_002235028.1	+	295808	295856
Bfl-1-99	NW_002235028.1	-	295346	295298
Bfl-1-100	NW_002235029.1	+	2177153	2177201
Bfl-1-101	NW_002235034.1	+	1059420	1059468
Bfl-1-102	NW_002235034.1	-	1059196	1059148
Bfl-1-103	NW_002235040.1	+	199392	199440
Bfl-1-104	NW_002235040.1	-	199180	199132
Bfl-1-105	NW_002235047.1	-	818829	818781
Bfl-1-106	NW_002235050.1	+	656291	656339
Bfl-1-107	NW_002235084.1	-	1507468	1507420
Bfl-1-108	NW_002235104.1	-	436665	436617
Bfl-1-109	NW_002235115.1	+	759795	759843
Bfl-1-110	NW_002235115.1	+	831181	831229
Bfl-1-111	NW_002235115.1	-	829585	829537
Bfl-1-112	NW_002235115.1	-	757428	757380
Bfl-1-113	NW_002235163.1	+	634872	634920
Bfl-1-114	NW_002235170.1	+	1245885	1245933
Bfl-1-115	NW_002235170.1	-	1232876	1232828
Bfl-1-116	NW_002235174.1	+	902572	902620
Bfl-1-117	NW_002235192.1	+	916638	916686
Bfl-1-118	NW_002235198.1	-	101596	101548
Bfl-1-119	NW_002235224.1	-	704438	704390
Bfl-1-120	NW_002235245.1	+	504776	504824
Bfl-1-121	NW_002235245.1	-	504592	504544
Bfl-1-122	NW_002235304.1	+	76957	77005
Bfl-1-123	NW_002235315.1	-	612534	612486
Bfl-1-124	NW_002235320.1	+	364117	364165
Bfl-1-125	NW_002235351.1	-	194204	194156
Bfl-1-126	NW_002235384.1	+	301388	301436
Bfl-1-127	NW_002235384.1	-	299914	299866
Bfl-1-128	NW_002235444.1	+	53319	53367
Bfl-1-129	NW_002235515.1	+	330775	330823
Bfl-1-130	NW_002235515.1	-	330078	330030
Bfl-1-131	NW_002235560.1	+	180197	180245
Bfl-1-132	NW_002235560.1	-	179651	179603
Bfl-1-133	NW_002235593.1	+	286916	286964
Bfl-1-134	NW_002235593.1	-	286434	286386
Bfl-1-135	NW_002235696.1	+	166394	166442
Bfl-1-136	NW_002235732.1	-	59193	59145
Bfl-1-137	NW_002234961.1	-	2131799	2131751
Bfl-1-138	NW_002235442.1	+	483375	483423

Bfl-1-139	NW_002235224.1	+	704653	704697
Bfl-1-140	NW_002235408.1	-	546165	546109
Bfl-1-141	NW_002235653.1	-	74749	74701
env-61	JCVI_SCAF_1096628253058	+	1366	1415
Nve-1-135	NW_001834409.1	-	2509834	2509770
Nve-1-136	NW_001834376.1	-	703060	702996
Nve-1-137	NW_001834383.1	+	1007324	1007388
Nve-1-138	NW_001833108.1	-	16848	16784
Nve-1-139	NW_001833119.1	+	28457	28521
Nve-1-140	NW_001833346.1	-	25996	25932
Nve-1-141	NW_001833742.1	-	20336	20272
Nve-1-142	NW_001833803.1	-	55969	55905
Nve-1-143	NW_001834001.1	-	48098	48034
Nve-1-144	NW_001834079.1	-	88132	88068
Nve-1-145	NW_001834162.1	-	264631	264567
Nve-1-146	NW_001834245.1	-	494410	494346
Nve-1-147	NW_001834327.1	-	503642	503578
Nve-1-148	NW_001834328.1	+	787322	787386
Nve-1-149	NW_001834383.1	+	1014169	1014233
Nve-1-150	NW_001833972.1	-	28515	28451
Nve-1-151	NW_001834300.1	-	268327	268263
Nve-1-152	NW_001834315.1	+	63215	63279
Isc-1-2	NW_002642081.1	+	13087	13133
Isc-1-3	NW_002769786.1	+	947	993
Isc-1-4	NW_002742091.1	+	480	526
Isc-1-5	NW_002679431.1	+	1134	1180
Isc-1-6	NW_002749100.1	+	1	47
Isc-1-7	NW_002680771.1	-	486	440
Isc-1-8	NW_002677169.1	-	293405	293359
Isc-1-9	NW_002517528.1	-	419	373
Isc-1-10	NW_002815200.1	-	769493	769447
Isc-1-11	NW_002828199.1	-	183653	183607
Isc-1-12	NW_002727546.1	-	862	816
Isc-1-13	NW_002862507.1	-	31622	31576
Isc-1-14	NW_002603068.1	-	19003	18957
Isc-1-15	NW_002605043.1	+	257	303
Isc-1-16	NW_002776155.1	-	53214	53168
Isc-1-17	NW_002676341.1	-	22256	22210
Isc-1-18	NW_002567796.1	+	383627	383673
Isc-1-19	NW_002562747.1	-	304	258
Isc-1-20	NW_002600415.1	-	910	864
Isc-1-21	NW_002855198.1	-	568	522
Isc-1-22	NW_002587241.1	+	184471	184517
Isc-1-23	NW_002560514.1	-	174	128
Isc-1-24	NW_002788893.1	-	181979	181933
Isc-1-25	NW_002663741.1	-	144	98
Isc-1-26	NW_002648027.1	+	485	531
Isc-1-27	NW_002796001.1	-	16434	16388
Isc-1-28	NW_002586008.1	+	247272	247318
Isc-1-29	NW_002524507.1	-	413797	413751
Isc-1-30	NW_002597356.1	+	309271	309317
Isc-1-31	NW_002866415.1	-	506	460

Isc-1-32	NW_002591206.1	+	25823	25869
Isc-1-33	NW_002597032.1	+	7422	7468
Isc-1-34	NW_002573293.1	-	490	444
Isc-1-35	NW_002634276.1	+	384	430
Isc-1-36	NW_002606474.1	-	21063	21017
Isc-1-37	NW_002708378.1	+	369	415
Isc-1-38	NW_002731203.1	+	41510	41556
Isc-1-39	NW_002697200.1	+	6997	7043
Isc-1-40	NW_002771710.1	-	693	647
Isc-1-41	NW_002810481.1	+	12784	12830
Isc-1-42	NW_002834404.1	-	163	117
Isc-1-43	NW_002840718.1	+	3168	3214
Isc-1-44	NW_002747002.1	+	353804	353850
Isc-1-45	NW_002820801.1	+	4665	4711
Isc-1-46	NW_002663060.1	+	238238	238284
Isc-1-47	NW_002603288.1	+	662683	662729
Isc-1-48	NW_002510003.1	-	129074	129028
Isc-1-49	NW_002583161.1	+	130350	130396
Isc-1-50	NW_002829613.1	-	1374	1328
Isc-1-51	NW_002822383.1	-	1202780	1202734
Isc-1-52	NW_002819664.1	+	951	997
Isc-1-53	NW_002598356.1	+	2483467	2483515
Aae-1-28	NW_001811605.1	-	24508	24462
Aae-1-29	NW_001811540.1	-	2404487	2404440
Aae-1-30	NW_001811351.1	+	362694	362741
Aae-1-31	NW_001810944.1	+	1166724	1166771
Aae-1-32	NW_001809911.1	+	233667	233714
Aae-1-33	NW_001810286.1	-	1702965	1702918
Aae-1-34	NW_001810318.1	-	939768	939721
Aae-1-35	NW_001810381.1	+	1574666	1574713
Aae-1-36	NW_001810599.1	+	1329326	1329373
Aae-1-37	NW_001810996.1	-	2113297	2113250
Aae-1-38	NW_001811551.1	-	123113	123066
Aae-1-39	NW_001809890.1	+	1737462	1737509
Aae-1-40	NW_001809890.1	-	1728926	1728879
Aae-1-41	NW_001810555.1	+	3795958	3796005
Aae-1-42	NW_001810555.1	-	3794126	3794079
Aae-1-43	NW_001810927.1	-	1016579	1016532
Aae-1-44	NW_001811007.1	+	1652020	1652067
Aae-1-45	NW_001811007.1	-	1643861	1643814
Aae-1-46	NW_001811051.1	+	545252	545299
Aae-1-47	NW_001811052.1	-	3007485	3007438
Aae-1-48	NW_001811454.1	+	54240	54287
Aae-1-49	NW_001811469.1	+	407372	407419
Aae-1-50	NW_001811529.1	+	995009	995056
Aae-1-51	NW_001811529.1	-	992088	992041
Aae-1-52	NW_001811529.1	-	988405	988358
Aae-1-53	NW_001811573.1	+	868494	868541
Aae-1-54	NW_001837414.1	-	24147	24100
Aae-1-55	NW_001810006.1	+	40367	40414
Aae-1-56	NW_001810728.1	+	161671	161718
Aae-1-57	NW_001810736.1	+	288960	289007

Aae-1-58	NW_001810744.1	+	1104698	1104745
Aae-1-59	NW_001810975.1	-	1522667	1522620
Aae-1-60	NW_001811051.1	+	790413	790460
Aae-1-61	NW_001811051.1	-	790016	789969
Aae-1-62	NW_001811422.1	-	383796	383749
Aae-1-63	NW_001811758.1	-	40175	40128
Aae-1-64	NW_001809890.1	+	715583	715630
Aae-1-65	NW_001811005.1	+	1486752	1486799
Aae-1-66	NW_001811012.1	+	1205652	1205699
Aae-1-67	NW_001811071.1	+	1175469	1175516
Aae-1-68	NW_001810371.1	+	187557	187605
Nve-1-153	NW_001834024.1	-	164335	164290
Nve-1-154	NW_001834114.1	-	7248	7203
Nve-1-155	NW_001833989.1	-	17869	17824
Nve-1-156	NW_001834135.1	-	289226	289181
Nve-1-157	NW_001834185.1	+	192241	192286
Nve-1-158	NW_001834225.1	+	168276	168321
Nve-1-159	NW_001834258.1	-	529512	529467
Nve-1-160	NW_001834277.1	-	444139	444094
Nve-1-161	NW_001834317.1	-	201595	201550
Nve-1-162	NW_001834338.1	+	365716	365761
Nve-1-163	NW_001834347.1	-	247931	247886
Nve-1-164	NW_001834350.1	-	989414	989369
Nve-1-165	NW_001834395.1	+	496948	496993
Nve-1-166	NW_001834401.1	+	1453253	1453298
Nve-1-167	NW_001834410.1	-	1845603	1845558
Nve-1-168	NW_001834309.1	-	483169	483122
Nve-1-169	NW_001834242.1	-	264570	264525
Nve-1-170	NW_001834256.1	-	416542	416496
Nve-1-171	NW_001834220.1	-	281922	281854
env-62	JCVI_SCAF_1101668298282	+	1243	1289
env-63	JCVI_SCAF_1096626313467	+	655	722
Yli-1-4	NC_006067.1	+	1267927	1268004
env-64	JCVI_SCAF_1101668082200	+	564	616
env-65	JCVI_SCAF_1101668159470	+	694	761
Bta-1-1	NW_001494423.2	+	527136	527199
Bta-1-2	NC_007328.3	+	35909687	35909750
Hma-1-2	NW_002077193.1	+	18279	18327
Hma-1-3	NW_002124763.1	+	4057	4105
Hma-1-4	NW_002106000.1	+	3510	3558
Hma-1-5	NW_002119109.1	-	833	785
Hma-1-6	NW_002164288.1	-	26210	26162
Yli-1-5	NC_006072.1	+	1873871	1873945
Yli-1-6	NC_006070.1	+	1831988	1832062
Yli-1-7	NC_006067.1	-	926838	926764
Yli-1-8	NC_006069.1	+	2240506	2240580
Yli-1-9	NC_006069.1	+	2071693	2071767
Yli-1-10	NC_006070.1	+	1990474	1990548
Yli-1-11	NC_006072.1	-	2111653	2111579
Yli-1-12	NC_006072.1	-	2055084	2055010
Yli-1-13	NC_006068.1	+	1042478	1042537
Sma-1-1	NW_003038086.1	+	176951	177000

Sma-1-2	NS_000200.1	+	272425544	272425593
env-66	ABMF01074935	-	79	11
Nfi-1-1	NW_001509761.1	-	12507	12424
Isc-1-54	NW_002721811.1	+	872	921
Isc-1-55	NW_002712427.1	+	309	358
env-67	ABLX01151415	+	14	63
Sma-1-3	NW_003038014.1	-	338331	338254
Sma-1-4	NS_000200.1	-	230079020	230078943
env-68	JCVI_SCAF_1096627819017	+	74	130
Mdo-1-21	NW_001581961.1	-	43237448	43237381
Mdo-1-22	NC_008804.1	-	154633726	154633659
Ldr-1-1	NZ_GG697213.1	+	55707	55766
Ldr-1-2	NZ_ACUL01000193.1	+	55707	55766
Lpn-4-1	NC_002942.5	-	1072076	1072023
Lpn-1-1	NC_009494.1	+	2514633	2514686
Lpn-2-1	NC_006369.1	-	1144756	1144703
Lpn-3-1	NC_006368.1	-	1171359	1171306
Ldr-1-3	NZ_GG697172.2	-	4062	4009
Ldr-1-4	NZ_ACUL01000006.1	-	4062	4009
Ldr-1-5	NZ_GG697225.1	-	31469	31411
Ldr-1-6	NZ_ACUL01000246.1	-	27663	27605
Ldr-1-7	NZ_GG697220.1	-	5568	5510
Ldr-1-8	NZ_ACUL01000219.1	-	3940	3882
Ldr-1-9	NZ_GG697210.1	-	187605	187554
Ldr-1-10	NZ_ACUL01000186.1	-	3471	3420
Lpn-3-2	NC_006368.1	+	2751461	2751515
Lpn-1-2	NC_009494.1	+	2095542	2095591
Ldr-1-11	NZ_GG697186.1	+	2211	2267
Ldr-1-12	NZ_ACUL01000097.1	+	2211	2267
Ldr-1-13	NZ_GG697205.1	+	194967	195020
Ldr-1-14	NZ_ACUL01000161.1	+	57418	57471
Lpn-2-2	NC_006369.1	+	2846397	2846450
Ldr-1-15	NZ_GG697176.2	-	2120	2064
Ldr-1-16	NZ_ACUL01000029.1	-	2120	2064
Lpn-1-3	NC_009494.1	+	2093328	2093384
Lpn-1-4	NC_009494.1	+	1515733	1515786
Lpn-3-3	NC_006368.1	+	2159430	2159483
Lpn-2-3	NC_006369.1	-	1207047	1206994
Lpn-3-4	NC_006368.1	-	1174061	1174006
Ldr-1-17	NZ_GG697215.1	-	2091	2034
Ldr-1-18	NZ_ACUL01000196.1	-	2091	2034
Lpn-4-2	NC_002942.5	-	1074855	1074800
Lpn-1-5	NC_009494.1	+	2511858	2511913
env-69	JCVI_SCAF_1096626609377	-	526	458
Sma-1-5	NW_003026465.1	+	429593	429666
Sma-1-6	NS_000200.1	+	207822187	207822260
Sma-1-7	NW_003038222.1	-	32110	32059
Sma-1-8	NS_000200.1	-	308819774	308819723
Sma-1-9	NW_003038118.1	+	181300	181364
Sma-1-10	NS_000200.1	+	277745427	277745491
env-70	NCBI_READ_1112079264739	-	309	249
env-71	JCVI_SCAF_1101668371663	-	785	728

Sma-1-11	NW_003038135.1	-	52950	52877
Sma-1-12	NS_000200.1	-	283925289	283925216
env-72	NCBI_BBAY_READ_1105897217131	-	305	190
Hma-1-7	NW_002160824.1	+	129091	129144
Hma-1-8	NW_002161880.1	+	6807	6860
Csy-1-1	NS_000189.1	+	691487	691550
Eca-1-13	NW_001867423.1	+	9494273	9494321
Eca-1-14	NC_009149.2	+	36560903	36560951
env-73	84502383	-	747	688
env-74	ABMB01022426	-	98	40
env-75	NCBI_READ_1112309042424	+	132	185
env-76	JCVI_SCAF_1101668035444	+	832	912
Sma-1-13	NW_003038022.1	+	622166	622226
Sma-1-14	NS_000200.1	+	236303008	236303068
Sma-1-15	NW_003037859.1	+	375455	375523
Sma-1-16	NS_000200.1	+	103054296	103054364
Aae-1-69	NW_001811129.1	+	4713376	4713459
Cqu-1-1	NW_001886869.1	-	185843	185775
Aae-1-70	NW_001811462.1	-	386848	386795
Aae-1-71	NW_001811485.1	-	2881342	2881283
Aae-1-72	NW_001811042.1	+	718517	718574
Aae-1-73	NW_001811498.1	+	206290	206342
Aae-1-74	NW_001811375.1	+	398697	398750
Aae-1-75	NW_001810156.1	+	1172558	1172612
Aae-1-76	NW_001809814.1	+	1082261	1082316
Aae-1-77	NW_001810974.1	+	176898	176951
Aae-1-78	NW_001810721.1	+	1251064	1251118
Aae-1-79	NW_001810307.1	+	183910	183965
Cqu-1-2	NW_001887254.1	+	133905	133957
Cqu-1-3	NW_001887222.1	+	277298	277350
Cqu-1-4	NW_001887310.1	+	35764	35816
Cqu-1-5	NW_001887310.1	-	5954	5902
Cqu-1-6	NW_001887352.1	+	121459	121511
Cqu-1-7	NW_001887553.1	+	178650	178702
Cqu-1-8	NW_001886739.1	-	933411	933359
Cqu-1-9	NW_001887091.1	+	202943	202995
Cqu-1-10	NW_001886715.1	+	1033335	1033387
Cqu-1-11	NW_001887996.1	+	33640	33692
Cqu-1-12	NW_001886868.1	-	37581	37529
Cqu-1-13	NW_001887301.1	-	26128	26076
Cqu-1-14	NW_001887547.1	+	74642	74694
Cqu-1-15	NW_001886914.1	-	587470	587418
Cqu-1-16	NW_001887097.1	+	231334	231386
Cqu-1-17	NW_001886773.1	-	954557	954505
Cqu-1-18	NW_001886707.1	-	1801093	1801041
Aae-1-80	NW_001837444.1	+	34233	34287
Aae-1-81	NW_001811618.1	+	1112562	1112618
Aae-1-82	NW_001811200.1	-	395926	395872
Aae-1-83	NW_001810783.1	+	2046658	2046712
Aae-1-84	NW_001810011.1	-	1600806	1600751
Aae-1-85	NW_001810381.1	-	1515683	1515628
Aae-1-86	NW_001811462.1	+	277499	277552

Aae-1-87	NW_001810575.1	-	1803735	1803683
Aae-1-88	NW_001811338.1	-	603919	603867
Aae-1-89	NW_001810844.1	-	1866218	1866166
Aae-1-90	NW_001811440.1	+	1129509	1129564
Aae-1-91	NW_001811494.1	-	357556	357502
Aae-1-92	NW_001811236.1	+	717688	717742
Aae-1-93	NW_001835485.1	+	5572	5626
Aae-1-94	NW_001811601.1	+	149125	149180
Aae-1-95	NW_001810750.1	-	1254768	1254709
Aae-1-96	NW_001809912.1	-	2022835	2022780
Aae-1-97	NW_001811329.1	+	1180491	1180549
Aae-1-98	NW_001811175.1	-	818395	818340
Aae-1-99	NW_001810156.1	-	155137	155085
Aae-1-100	NW_001811252.1	+	221529	221586
Aae-1-101	NW_001811007.1	-	1792937	1792878
Aae-1-102	NW_001811418.1	+	463492	463545
Aae-1-103	NW_001811772.1	-	2616497	2616443
Aae-1-104	NW_001810555.1	-	3146602	3146548
Aae-1-105	NW_001810392.1	-	891853	891801
Aae-1-106	NW_001811399.1	-	332791	332736
Aae-1-107	NW_001811005.1	+	1140215	1140268
Aae-1-108	NW_001810264.1	+	935504	935558
Aae-1-109	NW_001810681.1	-	1146275	1146216
Aae-1-110	NW_001811477.1	-	375152	375098
Aae-1-111	NW_001811772.1	+	2708171	2708225
Aae-1-112	NW_001810696.1	-	657068	657014
Aae-1-113	NW_001810623.1	-	77173	77119
Aae-1-114	NW_001811289.1	-	623431	623375
Aae-1-115	NW_001810901.1	-	1525595	1525540
Aae-1-116	NW_001811760.1	+	238373	238428
Aae-1-117	NW_001810592.1	+	1035417	1035472
Aae-1-118	NW_001811141.1	+	989017	989076
Aae-1-119	NW_001811362.1	-	24248	24193
Aae-1-120	NW_001811507.1	-	1051262	1051206
Aae-1-121	NW_001811522.1	+	456913	456971
Aae-1-122	NW_001811440.1	+	875418	875476
Aae-1-123	NW_001811085.1	-	2729178	2729127
Aae-1-124	NW_001811085.1	-	2724313	2724262
Aae-1-125	NW_001811462.1	+	4352948	4353003
Aae-1-126	NW_001811530.1	+	299548	299603
Aae-1-127	NW_001811059.1	-	233509	233456
Aae-1-128	NW_001811184.1	+	292994	293047
Aae-1-129	NW_001810714.1	+	3193937	3193990
Aae-1-130	NW_001811308.1	-	746786	746733
Aae-1-131	NW_001811523.1	-	460831	460776
Aae-1-132	NW_001810436.1	+	855780	855835
Aae-1-133	NW_001811748.1	-	316029	315974
Aae-1-134	NW_001810371.1	+	212466	212521
Aae-1-135	NW_001810328.1	-	1807523	1807468
Aae-1-136	NW_001811190.1	-	535249	535194
Aae-1-137	NW_001811194.1	+	598498	598553
Aae-1-138	NW_001811209.1	+	838147	838202

Aae-1-139	NW_001809979.1	-	2000699	2000644
Aae-1-140	NW_001810979.1	-	254586	254531
Aae-1-141	NW_001810988.1	-	771056	771001
Aae-1-142	NW_001811396.1	-	298932	298877
Aae-1-143	NW_001810791.1	-	765268	765213
Aae-1-144	NW_001811318.1	+	2757923	2757978
Aae-1-145	NW_001810715.1	+	304193	304248
Aae-1-146	NW_001811372.1	-	84199	84144
Aae-1-147	NW_001811340.1	+	2249625	2249680
Aae-1-148	NW_001811065.1	-	229088	229033
Aae-1-149	NW_001811467.1	-	276135	276080
Aae-1-150	NW_001809979.1	-	581355	581306
Aae-1-151	NW_001809982.1	-	129313	129257
Aae-1-152	NW_001809895.1	+	153761	153817
Aae-1-153	NW_001811158.1	-	784838	784782
Aae-1-154	NW_001811530.1	-	353688	353639
Aae-1-155	NW_001810813.1	+	1191633	1191688
Aae-1-156	NW_001810425.1	-	1911416	1911365
Aae-1-157	NW_001810938.1	-	149933	149881
Aae-1-158	NW_001811003.1	+	270588	270641
Aae-1-159	NW_001811060.1	-	474291	474238
Aae-1-160	NW_001811553.1	-	65850	65797
Aae-1-161	NW_001810339.1	-	956874	956819
Aae-1-162	NW_001810067.1	-	989870	989815
Aae-1-163	NW_001809951.1	+	61740	61793
Aae-1-164	NW_001809847.1	-	1599254	1599201
Aae-1-165	NW_001811069.1	-	1077057	1076996
Aae-1-166	NW_001811109.1	-	777969	777918
Aae-1-167	NW_001811451.1	-	2628028	2627973
Aae-1-168	NW_001810681.1	+	113736	113791
Aae-1-169	NW_001811429.1	+	1406106	1406161
Aae-1-170	NW_001811420.1	+	2275	2331
Aae-1-171	NW_001811662.1	+	18521	18576
Aae-1-172	NW_001811373.1	+	186502	186561
Aae-1-173	NW_001811366.1	-	387249	387189
Aae-1-174	NW_001811351.1	-	4872453	4872398
Aae-1-175	NW_001811351.1	-	337995	337940
Aae-1-176	NW_001811270.1	+	690035	690090
Aae-1-177	NW_001811262.1	+	423996	424051
Aae-1-178	NW_001811259.1	-	111264	111209
Aae-1-179	NW_001811254.1	+	810980	811035
Aae-1-180	NW_001811247.1	-	265113	265058
Aae-1-181	NW_001811207.1	-	2238412	2238357
Aae-1-182	NW_001811207.1	-	2154890	2154835
Aae-1-183	NW_001811200.1	-	681570	681515
Aae-1-184	NW_001811157.1	+	588287	588342
Aae-1-185	NW_001811138.1	-	758099	758044
Aae-1-186	NW_001810696.1	-	760281	760226
Aae-1-187	NW_001811130.1	-	2638197	2638142
Aae-1-188	NW_001811097.1	+	1107533	1107588
Aae-1-189	NW_001811090.1	+	178307	178362
Aae-1-190	NW_001810728.1	+	719101	719156

Aae-1-191	NW_001811085.1	-	1273777	1273722
Aae-1-192	NW_001810702.1	-	943087	943032
Aae-1-193	NW_001811484.1	-	607634	607579
Aae-1-194	NW_001811696.1	-	877158	877103
Aae-1-195	NW_001811078.1	-	556166	556111
Aae-1-196	NW_001811068.1	+	1040909	1040964
Aae-1-197	NW_001811043.1	+	1234666	1234721
Aae-1-198	NW_001811032.1	-	1168034	1167979
Aae-1-199	NW_001811007.1	-	3297936	3297881
Aae-1-200	NW_001810189.1	-	1450275	1450220
Aae-1-201	NW_001811005.1	+	949981	950036
Aae-1-202	NW_001811002.1	-	985199	985144
Aae-1-203	NW_001810997.1	+	707894	707949
Aae-1-204	NW_001810997.1	+	1457476	1457531
Aae-1-205	NW_001811507.1	-	330037	329982
Aae-1-206	NW_001810964.1	+	981334	981389
Aae-1-207	NW_001810490.1	+	1477177	1477236
Aae-1-208	NW_001810955.1	-	2882175	2882120
Aae-1-209	NW_001810944.1	+	260530	260585
Aae-1-210	NW_001810913.1	+	1072295	1072350
Aae-1-211	NW_001810818.1	-	1425308	1425253
Aae-1-212	NW_001810813.1	-	302019	301964
Aae-1-213	NW_001810805.1	-	1703619	1703564
Aae-1-214	NW_001810763.1	-	799328	799273
Aae-1-215	NW_001810425.1	+	1927610	1927665
Aae-1-216	NW_001810960.1	-	952063	952008
Aae-1-217	NW_001810681.1	-	113716	113661
Aae-1-218	NW_001810666.1	-	126943	126888
Aae-1-219	NW_001810637.1	+	903893	903948
Aae-1-220	NW_001810537.1	-	3257	3202
Aae-1-221	NW_001810447.1	+	1683838	1683893
Aae-1-222	NW_001810058.1	+	220403	220458
Aae-1-223	NW_001810034.1	+	1138599	1138654
Aae-1-224	NW_001810023.1	+	3677	3754
Aae-1-225	NW_001809878.1	-	34838	34783
Aae-1-226	NW_001811750.1	-	195764	195709
Aae-1-227	NW_001809827.1	-	175357	175302
Aae-1-228	NW_001809801.1	+	4908551	4908606
Aae-1-229	NW_001811577.1	+	240878	240933
Aae-1-230	NW_001811573.1	-	84929	84874
Aae-1-231	NW_001811354.1	+	325238	325293
Aae-1-232	NW_001811276.1	-	716580	716525
Aae-1-233	NW_001809935.1	-	1511089	1511034
Aae-1-234	NW_001811318.1	-	366033	365981
Aae-1-235	NW_001811142.1	-	520693	520640
Aae-1-236	NW_001811010.1	+	1002012	1002067
Aae-1-237	NW_001809847.1	+	130853	130908
Aae-1-238	NW_001809847.1	+	132604	132659
Aae-1-239	NW_001810750.1	+	462213	462268
Aae-1-240	NW_001810919.1	-	230695	230640
Aae-1-241	NW_001811089.1	-	432994	432939
Aae-1-242	NW_001811118.1	+	1546757	1546812

Aae-1-243	NW_001811142.1	+	793165	793220
Aae-1-244	NW_001811224.1	-	540515	540460
Aae-1-245	NW_001811562.1	+	2095623	2095678
Aae-1-246	NW_001809803.1	+	1639249	1639305
Aae-1-247	NW_001811039.1	-	322934	322878
Aae-1-248	NW_001811063.1	-	1186898	1186842
Aae-1-249	NW_001811410.1	+	354766	354822
Aae-1-250	NW_001810332.1	+	75469	75524
Aae-1-251	NW_001810715.1	-	1200721	1200666
Aae-1-252	NW_001810929.1	+	702226	702281
Aae-1-253	NW_001811063.1	+	1234919	1234974
Aae-1-254	NW_001811001.1	+	177980	178035
Aae-1-255	NW_001811429.1	+	724942	724997
Aae-1-256	NW_001811366.1	-	10139	10084
Aae-1-257	NW_001811362.1	-	278979	278913
Aae-1-258	NW_001811228.1	-	161702	161646
Aae-1-259	NW_001811028.1	+	784852	784909
Aae-1-260	NW_001809983.1	-	6141	6086
Aae-1-261	NW_001810011.1	-	1988789	1988737
Aae-1-262	NW_001809890.1	-	167600	167548
Aae-1-263	NW_001811780.1	+	236319	236378
Aae-1-264	NW_001811584.1	-	83101	83046
Aae-1-265	NW_001811207.1	-	1492251	1492198
Aae-1-266	NW_001811074.1	+	1501853	1501906
Aae-1-267	NW_001811047.1	+	747280	747335
Aae-1-268	NW_001811003.1	+	441253	441306
Aae-1-269	NW_001810955.1	+	711263	711318
Aae-1-270	NW_001810784.1	-	1237517	1237462
Aae-1-271	NW_001810307.1	+	754045	754098
Aae-1-272	NW_001810231.1	+	265335	265390
Aae-1-273	NW_001810200.1	-	524719	524666
Aae-1-274	NW_001810154.1	-	84180	84127
Aae-1-275	NW_001809811.1	-	202191	202136
Aae-1-276	NW_001810242.1	+	3331796	3331850
Aae-1-277	NW_001837144.1	-	10807	10752
Aae-1-278	NW_001811777.1	-	44309	44254
Aae-1-279	NW_001809901.1	+	542367	542422
Aae-1-280	NW_001811473.1	-	254615	254560
Aae-1-281	NW_001811742.1	+	332527	332582
Aae-1-282	NW_001811719.1	-	334906	334851
Aae-1-283	NW_001810999.1	+	1025780	1025835
Aae-1-284	NW_001811247.1	-	357168	357113
Aae-1-285	NW_001811680.1	-	312847	312792
Aae-1-286	NW_001811669.1	-	349823	349768
Aae-1-287	NW_001811660.1	+	265028	265083
Aae-1-288	NW_001811650.1	+	88233	88288
Aae-1-289	NW_001811628.1	-	252942	252887
Aae-1-290	NW_001811621.1	-	83021	82966
Aae-1-291	NW_001811618.1	-	595348	595293
Aae-1-292	NW_001811600.1	-	150303	150248
Aae-1-293	NW_001810905.1	-	1630830	1630775
Aae-1-294	NW_001811597.1	-	4847	4792

Aae-1-295	NW_001811596.1	-	1365805	1365750
Aae-1-296	NW_001811560.1	-	139398	139343
Aae-1-297	NW_001811524.1	-	523305	523250
Aae-1-298	NW_001811491.1	-	277930	277875
Aae-1-299	NW_001811486.1	-	283532	283477
Aae-1-300	NW_001810681.1	+	337652	337707
Aae-1-301	NW_001810945.1	-	948459	948404
Aae-1-302	NW_001811465.1	+	312561	312616
Aae-1-303	NW_001811107.1	-	1285625	1285570
Aae-1-304	NW_001810100.1	+	2018198	2018253
Aae-1-305	NW_001811463.1	+	2774368	2774423
Aae-1-306	NW_001810783.1	+	1665361	1665416
Aae-1-307	NW_001811462.1	+	2660709	2660764
Aae-1-308	NW_001811460.1	-	459664	459609
Aae-1-309	NW_001811448.1	+	31502	31557
Aae-1-310	NW_001811444.1	-	441601	441546
Aae-1-311	NW_001810305.1	-	172585	172530
Aae-1-312	NW_001810296.1	-	2172728	2172674
Aae-1-313	NW_001810178.1	-	712227	712172
Aae-1-314	NW_001810629.1	+	398240	398295
Aae-1-315	NW_001810214.1	+	22643	22699
Aae-1-316	NW_001810941.1	-	171933	171878
Aae-1-317	NW_001811772.1	+	1220977	1221046
Aae-1-318	NW_001811263.1	+	2948150	2948205
Aae-1-319	NW_001811385.1	-	1178664	1178607
Aae-1-320	NW_001810242.1	-	2632724	2632669
Aae-1-321	NW_001811083.1	+	939248	939306
Aae-1-322	NW_001811194.1	-	226379	226324
Aae-1-323	NW_001811429.1	+	2808809	2808864
Aae-1-324	NW_001811502.1	-	471794	471739
Aae-1-325	NW_001811418.1	+	2941289	2941344
Aae-1-326	NW_001811396.1	+	1954758	1954813
Aae-1-327	NW_001811385.1	-	428879	428824
Aae-1-328	NW_001811384.1	-	222374	222319
Aae-1-329	NW_001811346.1	-	475674	475619
Aae-1-330	NW_001811346.1	+	310558	310613
Aae-1-331	NW_001811339.1	-	505239	505184
Aae-1-332	NW_001811626.1	-	70200	70145
Aae-1-333	NW_001811207.1	-	2831382	2831327
Aae-1-334	NW_001811322.1	+	658790	658845
Aae-1-335	NW_001811312.1	+	517723	517778
Aae-1-336	NW_001810122.1	+	234215	234270
Aae-1-337	NW_001811281.1	-	155012	154957
Aae-1-338	NW_001811274.1	+	2149495	2149550
Aae-1-339	NW_001811272.1	+	575636	575691
Aae-1-340	NW_001811038.1	+	1184375	1184430
Aae-1-341	NW_001811354.1	-	535343	535288
Aae-1-342	NW_001811263.1	+	12428	12483
Aae-1-343	NW_001811252.1	+	247831	247886
Aae-1-344	NW_001811252.1	-	1579512	1579457
Aae-1-345	NW_001811237.1	+	561293	561348
Aae-1-346	NW_001811232.1	+	529670	529725

Aae-1-347	NW_001811208.1	+	809001	809056
Aae-1-348	NW_001811195.1	-	511947	511892
Aae-1-349	NW_001811499.1	-	452388	452333
Aae-1-350	NW_001811184.1	+	388211	388266
Aae-1-351	NW_001811149.1	-	554827	554772
Aae-1-352	NW_001811141.1	+	1953415	1953470
Aae-1-353	NW_001811133.1	+	223213	223268
Aae-1-354	NW_001811123.1	+	706551	706606
Aae-1-355	NW_001811119.1	-	965270	965215
Aae-1-356	NW_001811109.1	+	849782	849837
Aae-1-357	NW_001811097.1	-	30887	30832
Aae-1-358	NW_001811096.1	+	3121232	3121287
Aae-1-359	NW_001811067.1	-	694091	694036
Aae-1-360	NW_001811065.1	-	784067	784012
Aae-1-361	NW_001811052.1	+	428051	428106
Aae-1-362	NW_001811044.1	+	220141	220196
Aae-1-363	NW_001811043.1	-	795949	795894
Aae-1-364	NW_001811042.1	-	1256382	1256327
Aae-1-365	NW_001811032.1	+	1168042	1168097
Aae-1-366	NW_001811029.1	-	28642	28587
Aae-1-367	NW_001811016.1	-	8977	8922
Aae-1-368	NW_001811008.1	-	952082	952027
Aae-1-369	NW_001810996.1	-	3721244	3721189
Aae-1-370	NW_001810988.1	+	976513	976568
Aae-1-371	NW_001810984.1	+	2285400	2285455
Aae-1-372	NW_001810959.1	-	1022265	1022210
Aae-1-373	NW_001810954.1	-	428108	428053
Aae-1-374	NW_001810937.1	+	1011279	1011334
Aae-1-375	NW_001810912.1	+	2308353	2308408
Aae-1-376	NW_001810912.1	-	149422	149367
Aae-1-377	NW_001810864.1	-	814291	814236
Aae-1-378	NW_001810783.1	+	3745869	3745924
Aae-1-379	NW_001810782.1	-	3061385	3061330
Aae-1-380	NW_001810763.1	-	824502	824447
Aae-1-381	NW_001810736.1	+	1297755	1297810
Aae-1-382	NW_001810721.1	+	197857	197912
Aae-1-383	NW_001810715.1	-	1870244	1870189
Aae-1-384	NW_001810708.1	-	635213	635158
Aae-1-385	NW_001810637.1	+	1018466	1018521
Aae-1-386	NW_001811221.1	+	760682	760737
Aae-1-387	NW_001810519.1	-	153852	153797
Aae-1-388	NW_001810490.1	+	369105	369160
Aae-1-389	NW_001810469.1	+	276392	276448
Aae-1-390	NW_001810447.1	+	1573797	1573852
Aae-1-391	NW_001810361.1	-	1875347	1875292
Aae-1-392	NW_001810286.1	-	1158935	1158880
Aae-1-393	NW_001810275.1	-	1518754	1518699
Aae-1-394	NW_001810254.1	-	1693905	1693850
Aae-1-395	NW_001810241.1	-	67841	67786
Aae-1-396	NW_001810133.1	+	1157403	1157458
Aae-1-397	NW_001810111.1	+	299331	299386
Aae-1-398	NW_001810080.1	+	130741	130796

Aae-1-399	NW_001810034.1	-	1222479	1222424
Aae-1-400	NW_001809932.1	-	41826	41771
Aae-1-401	NW_001809912.1	-	4021674	4021619
Aae-1-402	NW_001809912.1	-	147212	147157
Aae-1-403	NW_001809869.1	-	206624	206569
Aae-1-404	NW_001809857.1	+	1470351	1470406
Aae-1-405	NW_001809822.1	+	228015	228071
Aae-1-406	NW_001809802.1	-	4454584	4454529
Aae-1-407	NW_001809802.1	+	4142354	4142409
Aae-1-408	NW_001809802.1	+	205357	205412
Aae-1-409	NW_001811121.1	-	968739	968684
Aae-1-410	NW_001811502.1	-	428420	428369
Aae-1-411	NW_001810197.1	+	24010	24065
Aae-1-412	NW_001810920.1	-	1664213	1664162
Aae-1-413	NW_001811052.1	-	999242	999187
Aae-1-414	NW_001811029.1	-	1073328	1073273
Aae-1-415	NW_001809869.1	-	60166	60111
Aae-1-416	NW_001811596.1	+	494526	494578
Aae-1-417	NW_001811369.1	+	108046	108098
Aae-1-418	NW_001811307.1	+	1132030	1132085
Aae-1-419	NW_001810307.1	+	1247710	1247762
Aae-1-420	NW_001811207.1	-	2686639	2686587
Aae-1-421	NW_001811551.1	+	2084526	2084578
Aae-1-422	NW_001811241.1	+	558021	558073
Aae-1-423	NW_001811218.1	+	2360523	2360578
Aae-1-424	NW_001811038.1	+	997641	997693
Aae-1-425	NW_001810895.1	-	836298	836246
Aae-1-426	NW_001811380.1	+	557860	557912
Aae-1-427	NW_001811763.1	-	221052	220999
Aae-1-428	NW_001811680.1	+	6587	6640
Aae-1-429	NW_001811279.1	-	625273	625220
Aae-1-430	NW_001811229.1	+	731658	731711
Aae-1-431	NW_001811140.1	-	137204	137151
Aae-1-432	NW_001810197.1	-	23990	23937
Aae-1-433	NW_001810242.1	-	2624487	2624432
Aae-1-434	NW_001810955.1	+	2513023	2513076
Aae-1-435	NW_001811026.1	+	1076831	1076884
Aae-1-436	NW_001811621.1	-	16493	16440
Aae-1-437	NW_001810935.1	-	44071	44018
Aae-1-438	NW_001811296.1	-	239941	239888
Aae-1-439	NW_001811220.1	-	319359	319304
Aae-1-440	NW_001811688.1	-	17933	17878
Aae-1-441	NW_001811688.1	-	3880	3825
Aae-1-442	NW_001811418.1	-	2461421	2461368
Aae-1-443	NW_001810715.1	+	202812	202867
Aae-1-444	NW_001811252.1	-	2812965	2812912
Aae-1-445	NW_001809834.1	-	198865	198810
Aae-1-446	NW_001811335.1	+	246204	246257
Aae-1-447	NW_001811274.1	+	1420205	1420258
Aae-1-448	NW_001810805.1	-	660973	660918
Aae-1-449	NW_001811417.1	+	381411	381464
Aae-1-450	NW_001811196.1	-	2945024	2944971

Aae-1-451	NW_001811018.1	-	4971342	4971289
Aae-1-452	NW_001810979.1	+	467282	467335
Aae-1-453	NW_001810972.1	+	94879	94932
Aae-1-454	NW_001810972.1	-	71229	71176
Aae-1-455	NW_001810962.1	+	930488	930541
Aae-1-456	NW_001810880.1	-	1911339	1911286
Aae-1-457	NW_001810830.1	-	1256826	1256773
Aae-1-458	NW_001810696.1	-	968526	968473
Aae-1-459	NW_001810458.1	+	3338261	3338314
Aae-1-460	NW_001810102.1	+	91822	91875
Aae-1-461	NW_001810133.1	+	1306467	1306520
Aae-1-462	NW_001810034.1	-	2068451	2068398
Aae-1-463	NW_001810023.1	+	2343083	2343136
Aae-1-464	NW_001809890.1	-	1338742	1338689
Aae-1-465	NW_001809819.1	+	143808	143861
Aae-1-466	NW_001810830.1	-	802137	802081
Aae-1-467	NW_001810728.1	+	814852	814906
Aae-1-468	NW_001810838.1	-	501539	501485
Aae-1-469	NW_001810599.1	+	330252	330306
Aae-1-470	NW_001810993.1	+	39726	39780
Aae-1-471	NW_001811218.1	-	455398	455343
Aae-1-472	NW_001811009.1	-	261513	261459
Aae-1-473	NW_001810912.1	-	768002	767948
Aae-1-474	NW_001811761.1	+	66882	66937
Aae-1-475	NW_001811761.1	-	66856	66801
Aae-1-476	NW_001811711.1	+	277761	277816
Aae-1-477	NW_001811669.1	+	349849	349904
Aae-1-478	NW_001810480.1	-	1209910	1209855
Aae-1-479	NW_001810825.1	-	174750	174695
Aae-1-480	NW_001810855.1	-	182726	182671
Aae-1-481	NW_001811253.1	+	648758	648813
Aae-1-482	NW_001811485.1	+	2988234	2988289
Aae-1-483	NW_001811225.1	+	447053	447108
Aae-1-484	NW_001811003.1	+	651605	651660
Aae-1-485	NW_001811428.1	-	546778	546723
Aae-1-486	NW_001811621.1	+	71186	71241
Aae-1-487	NW_001810508.1	+	148079	148134
Aae-1-488	NW_001810527.1	+	1540481	1540536
Aae-1-489	NW_001810231.1	+	594824	594880
Aae-1-490	NW_001809801.1	+	1512268	1512322
Aae-1-491	NW_001809801.1	+	254353	254408
Aae-1-492	NW_001809801.1	+	3712797	3712852
Aae-1-493	NW_001809801.1	+	5251690	5251745
Aae-1-494	NW_001809801.1	-	1507958	1507903
Aae-1-495	NW_001809802.1	+	70873	70928
Aae-1-496	NW_001809802.1	+	570865	570920
Aae-1-497	NW_001809802.1	+	573561	573616
Aae-1-498	NW_001809802.1	+	915399	915454
Aae-1-499	NW_001809802.1	+	2006804	2006859
Aae-1-500	NW_001809802.1	+	3161631	3161686
Aae-1-501	NW_001809802.1	+	3335012	3335067
Aae-1-502	NW_001809802.1	+	3474017	3474072

Aae-1-503	NW_001809802.1	-	3333759	3333704
Aae-1-504	NW_001809802.1	-	568162	568107
Aae-1-505	NW_001809802.1	-	565465	565410
Aae-1-506	NW_001809803.1	+	2496043	2496098
Aae-1-507	NW_001809806.1	-	12006	11951
Aae-1-508	NW_001809807.1	+	219215	219270
Aae-1-509	NW_001809807.1	-	106533	106478
Aae-1-510	NW_001809808.1	+	153507	153562
Aae-1-511	NW_001809809.1	-	148647	148592
Aae-1-512	NW_001809814.1	-	1579691	1579636
Aae-1-513	NW_001809820.1	-	284806	284751
Aae-1-514	NW_001809822.1	-	227981	227926
Aae-1-515	NW_001809825.1	-	830663	830608
Aae-1-516	NW_001809836.1	+	882742	882797
Aae-1-517	NW_001809836.1	-	900792	900737
Aae-1-518	NW_001809836.1	-	882305	882250
Aae-1-519	NW_001809838.1	+	106065	106120
Aae-1-520	NW_001809838.1	-	215466	215411
Aae-1-521	NW_001809841.1	+	296054	296109
Aae-1-522	NW_001809842.1	+	118174	118229
Aae-1-523	NW_001809842.1	+	144085	144140
Aae-1-524	NW_001809842.1	-	115283	115228
Aae-1-525	NW_001809843.1	+	136619	136674
Aae-1-526	NW_001809847.1	+	1304719	1304774
Aae-1-527	NW_001809847.1	-	2444655	2444600
Aae-1-528	NW_001809850.1	-	13223	13168
Aae-1-529	NW_001809857.1	-	1441693	1441638
Aae-1-530	NW_001809857.1	-	650923	650868
Aae-1-531	NW_001809863.1	-	28491	28436
Aae-1-532	NW_001809867.1	+	158738	158793
Aae-1-533	NW_001809877.1	-	206936	206881
Aae-1-534	NW_001809879.1	-	556446	556391
Aae-1-535	NW_001809880.1	+	236019	236074
Aae-1-536	NW_001809880.1	+	249911	249966
Aae-1-537	NW_001809890.1	+	1378667	1378722
Aae-1-538	NW_001809894.1	-	167195	167140
Aae-1-539	NW_001809901.1	+	66747	66802
Aae-1-540	NW_001809901.1	+	2176978	2177033
Aae-1-541	NW_001809901.1	-	251262	251207
Aae-1-542	NW_001809903.1	+	213493	213548
Aae-1-543	NW_001809912.1	+	1111054	1111109
Aae-1-544	NW_001809912.1	+	4084773	4084828
Aae-1-545	NW_001809912.1	+	4141024	4141079
Aae-1-546	NW_001809912.1	-	2568275	2568220
Aae-1-547	NW_001809913.1	+	360302	360357
Aae-1-548	NW_001809913.1	-	252137	252082
Aae-1-549	NW_001809921.1	-	86657	86602
Aae-1-550	NW_001809922.1	+	37930	37985
Aae-1-551	NW_001809924.1	-	436056	436001
Aae-1-552	NW_001809927.1	+	22018	22073
Aae-1-553	NW_001809930.1	-	270450	270395
Aae-1-554	NW_001809931.1	+	162563	162618

Aae-1-555	NW_001809935.1	+	2364775	2364830
Aae-1-556	NW_001809946.1	+	2065788	2065843
Aae-1-557	NW_001809946.1	-	2263408	2263353
Aae-1-558	NW_001809956.1	+	32233	32288
Aae-1-559	NW_001809956.1	-	30695	30640
Aae-1-560	NW_001809957.1	-	2240764	2240709
Aae-1-561	NW_001809958.1	-	122073	122018
Aae-1-562	NW_001809964.1	-	191465	191410
Aae-1-563	NW_001809967.1	+	14305	14360
Aae-1-564	NW_001809968.1	+	43126	43181
Aae-1-565	NW_001809968.1	+	2407901	2407956
Aae-1-566	NW_001809974.1	-	135356	135301
Aae-1-567	NW_001809982.1	-	209742	209687
Aae-1-568	NW_001809989.1	+	13	68
Aae-1-569	NW_001809989.1	-	1872121	1872066
Aae-1-570	NW_001809990.1	+	65606	65661
Aae-1-571	NW_001810000.1	+	453602	453657
Aae-1-572	NW_001810000.1	+	1723910	1723965
Aae-1-573	NW_001810000.1	+	2175704	2175759
Aae-1-574	NW_001810000.1	+	2270868	2270923
Aae-1-575	NW_001810000.1	-	472219	472164
Aae-1-576	NW_001810004.1	+	181545	181600
Aae-1-577	NW_001810011.1	-	1075932	1075877
Aae-1-578	NW_001810011.1	-	110931	110876
Aae-1-579	NW_001810019.1	+	91480	91535
Aae-1-580	NW_001810019.1	+	197578	197633
Aae-1-581	NW_001810022.1	+	352122	352177
Aae-1-582	NW_001810022.1	+	2937240	2937295
Aae-1-583	NW_001810022.1	-	4148424	4148369
Aae-1-584	NW_001810022.1	-	4099925	4099870
Aae-1-585	NW_001810022.1	-	3561520	3561465
Aae-1-586	NW_001810022.1	-	2545443	2545388
Aae-1-587	NW_001810022.1	-	2200447	2200392
Aae-1-588	NW_001810022.1	-	1480352	1480297
Aae-1-589	NW_001810022.1	-	852958	852903
Aae-1-590	NW_001810023.1	-	792969	792914
Aae-1-591	NW_001810027.1	-	145800	145745
Aae-1-592	NW_001810029.1	+	189151	189206
Aae-1-593	NW_001810034.1	+	613932	613987
Aae-1-594	NW_001810034.1	+	1522388	1522443
Aae-1-595	NW_001810034.1	-	1643757	1643702
Aae-1-596	NW_001810034.1	-	1457952	1457897
Aae-1-597	NW_001810034.1	-	340996	340941
Aae-1-598	NW_001810037.1	+	179044	179099
Aae-1-599	NW_001810038.1	+	176410	176465
Aae-1-600	NW_001810040.1	+	179373	179428
Aae-1-601	NW_001810050.1	+	156467	156522
Aae-1-602	NW_001810052.1	+	22746	22801
Aae-1-603	NW_001810052.1	-	19559	19504
Aae-1-604	NW_001810056.1	-	1481610	1481555
Aae-1-605	NW_001810056.1	-	763318	763263
Aae-1-606	NW_001810067.1	+	2338274	2338329

Aae-1-607	NW_001810067.1	-	1587133	1587078
Aae-1-608	NW_001810067.1	-	1545839	1545784
Aae-1-609	NW_001810067.1	-	1356368	1356313
Aae-1-610	NW_001810067.1	-	613208	613153
Aae-1-611	NW_001810070.1	-	134021	133966
Aae-1-612	NW_001810072.1	+	121289	121344
Aae-1-613	NW_001810089.1	+	1542204	1542259
Aae-1-614	NW_001810089.1	-	2235769	2235714
Aae-1-615	NW_001810089.1	-	2172314	2172259
Aae-1-616	NW_001810089.1	-	2076039	2075984
Aae-1-617	NW_001810089.1	-	98619	98564
Aae-1-618	NW_001810090.1	-	117855	117800
Aae-1-619	NW_001810091.1	-	45781	45726
Aae-1-620	NW_001810096.1	-	124362	124307
Aae-1-621	NW_001810100.1	+	1499153	1499208
Aae-1-622	NW_001810100.1	+	2068236	2068291
Aae-1-623	NW_001810102.1	-	4130	4075
Aae-1-624	NW_001810106.1	+	149716	149771
Aae-1-625	NW_001810111.1	+	1261469	1261524
Aae-1-626	NW_001810111.1	+	2143782	2143837
Aae-1-627	NW_001810111.1	+	2225535	2225590
Aae-1-628	NW_001810111.1	-	1022873	1022818
Aae-1-629	NW_001810114.1	-	122475	122420
Aae-1-630	NW_001810117.1	+	99875	99930
Aae-1-631	NW_001810118.1	-	108582	108527
Aae-1-632	NW_001810119.1	+	127832	127887
Aae-1-633	NW_001810119.1	-	125273	125218
Aae-1-634	NW_001810122.1	+	475439	475494
Aae-1-635	NW_001810122.1	+	1767554	1767609
Aae-1-636	NW_001810126.1	-	40710	40655
Aae-1-637	NW_001810130.1	+	54948	55003
Aae-1-638	NW_001810133.1	-	3725703	3725648
Aae-1-639	NW_001810133.1	-	3368378	3368323
Aae-1-640	NW_001810133.1	-	1950672	1950617
Aae-1-641	NW_001810133.1	-	981440	981385
Aae-1-642	NW_001810133.1	-	781047	780992
Aae-1-643	NW_001810133.1	-	594057	594002
Aae-1-644	NW_001810134.1	-	962905	962850
Aae-1-645	NW_001810134.1	-	548030	547975
Aae-1-646	NW_001810134.1	-	169380	169325
Aae-1-647	NW_001810145.1	+	182829	182884
Aae-1-648	NW_001810145.1	+	185192	185247
Aae-1-649	NW_001810156.1	+	1152147	1152202
Aae-1-650	NW_001810156.1	-	1072542	1072487
Aae-1-651	NW_001810156.1	-	331744	331689
Aae-1-652	NW_001810166.1	+	21293	21348
Aae-1-653	NW_001810167.1	+	460592	460647
Aae-1-654	NW_001810167.1	+	746436	746491
Aae-1-655	NW_001810167.1	+	2233276	2233331
Aae-1-656	NW_001810167.1	-	1885087	1885032
Aae-1-657	NW_001810167.1	-	1618341	1618286
Aae-1-658	NW_001810167.1	-	643674	643619

Aae-1-659	NW_001810170.1	+	117035	117090
Aae-1-660	NW_001810174.1	-	25950	25895
Aae-1-661	NW_001810178.1	+	174934	174989
Aae-1-662	NW_001810178.1	+	949955	950010
Aae-1-663	NW_001810189.1	+	767692	767747
Aae-1-664	NW_001810189.1	-	164673	164618
Aae-1-665	NW_001810196.1	-	42585	42530
Aae-1-666	NW_001810200.1	+	47146	47201
Aae-1-667	NW_001810200.1	+	718006	718061
Aae-1-668	NW_001810200.1	-	1860138	1860083
Aae-1-669	NW_001810200.1	-	78565	78510
Aae-1-670	NW_001810200.1	-	46533	46478
Aae-1-671	NW_001810200.1	-	43830	43775
Aae-1-672	NW_001810221.1	-	1680689	1680634
Aae-1-673	NW_001810221.1	-	1197982	1197927
Aae-1-674	NW_001810225.1	+	25522	25577
Aae-1-675	NW_001810231.1	+	262382	262437
Aae-1-676	NW_001810231.1	-	2113705	2113650
Aae-1-677	NW_001810241.1	-	97637	97582
Aae-1-678	NW_001810242.1	+	907560	907615
Aae-1-679	NW_001810242.1	+	1501020	1501075
Aae-1-680	NW_001810242.1	+	2195403	2195458
Aae-1-681	NW_001810242.1	+	2754740	2754795
Aae-1-682	NW_001810242.1	+	3752030	3752085
Aae-1-683	NW_001810242.1	-	4324380	4324325
Aae-1-684	NW_001810242.1	-	3057645	3057590
Aae-1-685	NW_001810248.1	-	55134	55079
Aae-1-686	NW_001810254.1	+	743949	744004
Aae-1-687	NW_001810254.1	-	409022	408967
Aae-1-688	NW_001810264.1	+	152333	152388
Aae-1-689	NW_001810264.1	+	847155	847210
Aae-1-690	NW_001810264.1	-	150496	150441
Aae-1-691	NW_001810266.1	-	4130	4075
Aae-1-692	NW_001810275.1	+	635251	635306
Aae-1-693	NW_001810275.1	-	1021276	1021221
Aae-1-694	NW_001810285.1	+	12718	12773
Aae-1-695	NW_001810286.1	-	2039412	2039357
Aae-1-696	NW_001810286.1	-	1731365	1731310
Aae-1-697	NW_001810286.1	-	191012	190957
Aae-1-698	NW_001810287.1	+	100824	100879
Aae-1-699	NW_001810288.1	-	77581	77526
Aae-1-700	NW_001810296.1	+	846661	846716
Aae-1-701	NW_001810298.1	+	82836	82891
Aae-1-702	NW_001810299.1	-	22191	22136
Aae-1-703	NW_001810307.1	-	582608	582553
Aae-1-704	NW_001810307.1	-	197807	197752
Aae-1-705	NW_001810310.1	+	53422	53477
Aae-1-706	NW_001810318.1	-	1550330	1550275
Aae-1-707	NW_001810318.1	-	411067	411012
Aae-1-708	NW_001810328.1	-	902201	902146
Aae-1-709	NW_001810328.1	-	371912	371857
Aae-1-710	NW_001810339.1	-	1708810	1708755

Aae-1-711	NW_001810350.1	+	556700	556755
Aae-1-712	NW_001810350.1	+	559397	559452
Aae-1-713	NW_001810350.1	+	758308	758363
Aae-1-714	NW_001810350.1	+	1656978	1657033
Aae-1-715	NW_001810350.1	-	3194480	3194425
Aae-1-716	NW_001810350.1	-	2763797	2763742
Aae-1-717	NW_001810350.1	-	555487	555432
Aae-1-718	NW_001810351.1	-	351	296
Aae-1-719	NW_001810361.1	+	988820	988875
Aae-1-720	NW_001810361.1	-	1985898	1985843
Aae-1-721	NW_001810361.1	-	986644	986589
Aae-1-722	NW_001810371.1	+	853522	853577
Aae-1-723	NW_001810371.1	+	1554104	1554159
Aae-1-724	NW_001810371.1	-	842581	842526
Aae-1-725	NW_001810371.1	-	551800	551745
Aae-1-726	NW_001810371.1	-	84510	84455
Aae-1-727	NW_001810381.1	+	898460	898515
Aae-1-728	NW_001810381.1	+	1763792	1763847
Aae-1-729	NW_001810381.1	-	1843872	1843817
Aae-1-730	NW_001810381.1	-	1532404	1532349
Aae-1-731	NW_001810392.1	-	1545120	1545065
Aae-1-732	NW_001810403.1	-	1644421	1644366
Aae-1-733	NW_001810403.1	-	912629	912574
Aae-1-734	NW_001810408.1	-	32849	32794
Aae-1-735	NW_001810414.1	+	328998	329053
Aae-1-736	NW_001810414.1	+	930442	930497
Aae-1-737	NW_001810414.1	+	1676171	1676226
Aae-1-738	NW_001810414.1	-	1674903	1674848
Aae-1-739	NW_001810414.1	-	1052887	1052832
Aae-1-740	NW_001810414.1	-	669608	669553
Aae-1-741	NW_001810425.1	+	376317	376372
Aae-1-742	NW_001810425.1	+	542865	542920
Aae-1-743	NW_001810425.1	+	1154410	1154465
Aae-1-744	NW_001810425.1	+	1911441	1911496
Aae-1-745	NW_001810436.1	+	885267	885322
Aae-1-746	NW_001810436.1	+	887977	888032
Aae-1-747	NW_001810447.1	+	45465	45520
Aae-1-748	NW_001810447.1	+	1013760	1013815
Aae-1-749	NW_001810447.1	+	1016457	1016512
Aae-1-750	NW_001810447.1	+	1445894	1445949
Aae-1-751	NW_001810447.1	-	1010527	1010472
Aae-1-752	NW_001810447.1	-	1007829	1007774
Aae-1-753	NW_001810447.1	-	587764	587709
Aae-1-754	NW_001810458.1	+	3261005	3261060
Aae-1-755	NW_001810458.1	+	3501354	3501409
Aae-1-756	NW_001810458.1	-	314748	314693
Aae-1-757	NW_001810459.1	+	340318	340373
Aae-1-758	NW_001810459.1	+	1385245	1385300
Aae-1-759	NW_001810459.1	-	1637955	1637900
Aae-1-760	NW_001810469.1	+	1232489	1232544
Aae-1-761	NW_001810469.1	-	1030105	1030050
Aae-1-762	NW_001810475.1	-	11898	11843

Aae-1-763	NW_001810483.1	-	27462	27407
Aae-1-764	NW_001810490.1	-	1987256	1987201
Aae-1-765	NW_001810493.1	-	20671	20616
Aae-1-766	NW_001810508.1	+	1295551	1295606
Aae-1-767	NW_001810508.1	-	695694	695639
Aae-1-768	NW_001810519.1	+	1300407	1300462
Aae-1-769	NW_001810519.1	-	233582	233527
Aae-1-770	NW_001810526.1	-	82128	82073
Aae-1-771	NW_001810537.1	+	1077575	1077630
Aae-1-772	NW_001810537.1	+	1454604	1454659
Aae-1-773	NW_001810537.1	+	1482946	1483001
Aae-1-774	NW_001810537.1	-	1644801	1644746
Aae-1-775	NW_001810555.1	+	745408	745463
Aae-1-776	NW_001810555.1	+	2663452	2663507
Aae-1-777	NW_001810555.1	+	2666150	2666205
Aae-1-778	NW_001810555.1	+	3191713	3191768
Aae-1-779	NW_001810555.1	+	3625107	3625162
Aae-1-780	NW_001810555.1	+	3629395	3629450
Aae-1-781	NW_001810555.1	-	3010675	3010620
Aae-1-782	NW_001810555.1	-	2638182	2638127
Aae-1-783	NW_001810555.1	-	2349410	2349355
Aae-1-784	NW_001810556.1	-	430604	430549
Aae-1-785	NW_001810556.1	-	349735	349680
Aae-1-786	NW_001810556.1	-	145400	145345
Aae-1-787	NW_001810558.1	+	61	116
Aae-1-788	NW_001810565.1	-	138510	138455
Aae-1-789	NW_001810575.1	+	799550	799605
Aae-1-790	NW_001810575.1	+	1768865	1768920
Aae-1-791	NW_001810575.1	-	1861632	1861577
Aae-1-792	NW_001810575.1	-	1429659	1429604
Aae-1-793	NW_001810575.1	-	575288	575233
Aae-1-794	NW_001810584.1	+	620620	620675
Aae-1-795	NW_001810584.1	+	1920390	1920445
Aae-1-796	NW_001810592.1	-	810909	810854
Aae-1-797	NW_001810592.1	-	23022	22967
Aae-1-798	NW_001810593.1	-	612	557
Aae-1-799	NW_001810599.1	-	1517722	1517667
Aae-1-800	NW_001810607.1	-	375621	375566
Aae-1-801	NW_001810616.1	-	514323	514268
Aae-1-802	NW_001810616.1	-	150903	150848
Aae-1-803	NW_001810623.1	+	1036463	1036518
Aae-1-804	NW_001810623.1	+	1044350	1044405
Aae-1-805	NW_001810623.1	+	1059054	1059109
Aae-1-806	NW_001810623.1	+	1082779	1082834
Aae-1-807	NW_001810623.1	+	1099923	1099978
Aae-1-808	NW_001810629.1	+	896164	896219
Aae-1-809	NW_001810637.1	+	2891782	2891837
Aae-1-810	NW_001810637.1	-	2670960	2670905
Aae-1-811	NW_001810637.1	-	2471579	2471524
Aae-1-812	NW_001810648.1	+	917013	917068
Aae-1-813	NW_001810648.1	+	1097056	1097111
Aae-1-814	NW_001810648.1	-	1722107	1722052

Aae-1-815	NW_001810656.1	+	1521014	1521069
Aae-1-816	NW_001810666.1	+	1463984	1464039
Aae-1-817	NW_001810666.1	+	1807904	1807959
Aae-1-818	NW_001810666.1	-	1660415	1660360
Aae-1-819	NW_001810666.1	-	1335246	1335191
Aae-1-820	NW_001810666.1	-	1329509	1329454
Aae-1-821	NW_001810666.1	-	856547	856492
Aae-1-822	NW_001810675.1	+	1178466	1178521
Aae-1-823	NW_001810681.1	+	1321843	1321898
Aae-1-824	NW_001810681.1	-	818184	818129
Aae-1-825	NW_001810688.1	+	1238654	1238709
Aae-1-826	NW_001810688.1	-	387906	387851
Aae-1-827	NW_001810696.1	+	940280	940335
Aae-1-828	NW_001810702.1	-	517049	516994
Aae-1-829	NW_001810708.1	+	1046253	1046308
Aae-1-830	NW_001810714.1	+	1048101	1048156
Aae-1-831	NW_001810714.1	+	1260351	1260406
Aae-1-832	NW_001810714.1	+	1672978	1673033
Aae-1-833	NW_001810714.1	+	2768294	2768349
Aae-1-834	NW_001810714.1	+	2892043	2892098
Aae-1-835	NW_001810714.1	+	3283798	3283853
Aae-1-836	NW_001810714.1	+	3943693	3943748
Aae-1-837	NW_001810714.1	-	1672959	1672904
Aae-1-838	NW_001810715.1	+	93195	93250
Aae-1-839	NW_001810715.1	+	459541	459596
Aae-1-840	NW_001810715.1	+	919406	919461
Aae-1-841	NW_001810715.1	-	979416	979361
Aae-1-842	NW_001810721.1	-	1368497	1368442
Aae-1-843	NW_001810721.1	-	559978	559923
Aae-1-844	NW_001810736.1	+	1157924	1157979
Aae-1-845	NW_001810736.1	-	255316	255261
Aae-1-846	NW_001810744.1	+	1805957	1806012
Aae-1-847	NW_001810744.1	-	1481025	1480970
Aae-1-848	NW_001810750.1	+	200157	200212
Aae-1-849	NW_001810750.1	+	342270	342325
Aae-1-850	NW_001810750.1	-	347487	347432
Aae-1-851	NW_001810756.1	-	868350	868295
Aae-1-852	NW_001810756.1	-	341011	340956
Aae-1-853	NW_001810756.1	-	99349	99294
Aae-1-854	NW_001810763.1	+	71920	71975
Aae-1-855	NW_001810763.1	+	413682	413737
Aae-1-856	NW_001810763.1	+	824520	824575
Aae-1-857	NW_001810763.1	+	875237	875292
Aae-1-858	NW_001810769.1	+	1400005	1400060
Aae-1-859	NW_001810769.1	-	750193	750138
Aae-1-860	NW_001810778.1	+	1697455	1697510
Aae-1-861	NW_001810778.1	-	206579	206524
Aae-1-862	NW_001810782.1	-	963092	963037
Aae-1-863	NW_001810782.1	-	799970	799915
Aae-1-864	NW_001810783.1	+	628534	628589
Aae-1-865	NW_001810784.1	+	928075	928130
Aae-1-866	NW_001810784.1	+	1237532	1237587

Aae-1-867	NW_001810784.1	-	103414	103359
Aae-1-868	NW_001810787.1	-	6850	6795
Aae-1-869	NW_001810791.1	+	242779	242834
Aae-1-870	NW_001810791.1	+	1660639	1660694
Aae-1-871	NW_001810791.1	-	1610948	1610893
Aae-1-872	NW_001810791.1	-	1599223	1599168
Aae-1-873	NW_001810791.1	-	1171699	1171644
Aae-1-874	NW_001810791.1	-	667099	667044
Aae-1-875	NW_001810795.1	-	463102	463047
Aae-1-876	NW_001810800.1	+	1788764	1788819
Aae-1-877	NW_001810800.1	-	703548	703493
Aae-1-878	NW_001810813.1	+	532527	532582
Aae-1-879	NW_001810813.1	-	876444	876389
Aae-1-880	NW_001810813.1	-	504636	504581
Aae-1-881	NW_001810816.1	+	206967	207022
Aae-1-882	NW_001810816.1	-	176490	176435
Aae-1-883	NW_001810821.1	+	30055	30110
Aae-1-884	NW_001810825.1	-	1255633	1255578
Aae-1-885	NW_001810830.1	+	647769	647824
Aae-1-886	NW_001810830.1	+	1117648	1117703
Aae-1-887	NW_001810830.1	-	1276018	1275963
Aae-1-888	NW_001810830.1	-	1056969	1056914
Aae-1-889	NW_001810830.1	-	829299	829244
Aae-1-890	NW_001810830.1	-	408110	408055
Aae-1-891	NW_001810837.1	+	3330944	3330999
Aae-1-892	NW_001810837.1	+	3900684	3900739
Aae-1-893	NW_001810844.1	-	475469	475414
Aae-1-894	NW_001810852.1	+	15236	15291
Aae-1-895	NW_001810852.1	+	183099	183154
Aae-1-896	NW_001810852.1	+	359980	360035
Aae-1-897	NW_001810852.1	+	1527242	1527297
Aae-1-898	NW_001810852.1	-	1657919	1657864
Aae-1-899	NW_001810852.1	-	952509	952454
Aae-1-900	NW_001810852.1	-	608498	608443
Aae-1-901	NW_001810852.1	-	10359	10304
Aae-1-902	NW_001810852.1	-	499	444
Aae-1-903	NW_001810857.1	-	35908	35853
Aae-1-904	NW_001810858.1	-	1761688	1761633
Aae-1-905	NW_001810868.1	+	1377243	1377298
Aae-1-906	NW_001810868.1	-	1736295	1736240
Aae-1-907	NW_001810868.1	-	149661	149606
Aae-1-908	NW_001810876.1	+	233019	233074
Aae-1-909	NW_001810876.1	+	465026	465081
Aae-1-910	NW_001810876.1	+	1003576	1003631
Aae-1-911	NW_001810876.1	+	1699965	1700020
Aae-1-912	NW_001810876.1	-	355979	355924
Aae-1-913	NW_001810876.1	-	231617	231562
Aae-1-914	NW_001810880.1	-	1590918	1590863
Aae-1-915	NW_001810881.1	+	1372838	1372893
Aae-1-916	NW_001810881.1	-	372090	372035
Aae-1-917	NW_001810884.1	+	728001	728056
Aae-1-918	NW_001810891.1	+	224704	224759

Aae-1-919	NW_001810891.1	-	1072512	1072457
Aae-1-920	NW_001810895.1	+	689957	690012
Aae-1-921	NW_001810895.1	+	1046015	1046070
Aae-1-922	NW_001810895.1	+	1048713	1048768
Aae-1-923	NW_001810895.1	-	807051	806996
Aae-1-924	NW_001810901.1	+	306426	306481
Aae-1-925	NW_001810901.1	-	1194701	1194646
Aae-1-926	NW_001810901.1	-	773749	773694
Aae-1-927	NW_001810901.1	-	470676	470621
Aae-1-928	NW_001810904.1	+	13258	13313
Aae-1-929	NW_001810905.1	+	87126	87181
Aae-1-930	NW_001810905.1	-	1589882	1589827
Aae-1-931	NW_001810905.1	-	782724	782669
Aae-1-932	NW_001810905.1	-	443875	443820
Aae-1-933	NW_001810909.1	+	335073	335128
Aae-1-934	NW_001810912.1	+	1433536	1433591
Aae-1-935	NW_001810912.1	+	1436231	1436286
Aae-1-936	NW_001810912.1	+	1438928	1438983
Aae-1-937	NW_001810912.1	+	1719984	1720039
Aae-1-938	NW_001810912.1	+	3628949	3629004
Aae-1-939	NW_001810912.1	-	2037479	2037424
Aae-1-940	NW_001810912.1	-	1431910	1431855
Aae-1-941	NW_001810912.1	-	1386281	1386226
Aae-1-942	NW_001810913.1	+	897214	897269
Aae-1-943	NW_001810913.1	-	879407	879352
Aae-1-944	NW_001810916.1	-	225524	225469
Aae-1-945	NW_001810919.1	+	485522	485577
Aae-1-946	NW_001810919.1	-	364413	364358
Aae-1-947	NW_001810920.1	+	855310	855365
Aae-1-948	NW_001810920.1	-	1715071	1715016
Aae-1-949	NW_001810923.1	+	1621422	1621477
Aae-1-950	NW_001810929.1	+	1432891	1432946
Aae-1-951	NW_001810929.1	-	826326	826271
Aae-1-952	NW_001810937.1	+	1035194	1035249
Aae-1-953	NW_001810937.1	+	2318162	2318217
Aae-1-954	NW_001810938.1	+	559100	559155
Aae-1-955	NW_001810938.1	+	1332925	1332980
Aae-1-956	NW_001810938.1	-	1530039	1529984
Aae-1-957	NW_001810938.1	-	1331000	1330945
Aae-1-958	NW_001810939.1	+	517213	517268
Aae-1-959	NW_001810939.1	-	1444829	1444774
Aae-1-960	NW_001810944.1	+	619916	619971
Aae-1-961	NW_001810944.1	+	952470	952525
Aae-1-962	NW_001810944.1	-	342518	342463
Aae-1-963	NW_001810944.1	-	313736	313681
Aae-1-964	NW_001810945.1	+	903023	903078
Aae-1-965	NW_001810945.1	+	1310631	1310686
Aae-1-966	NW_001810945.1	-	684478	684423
Aae-1-967	NW_001810945.1	-	278695	278640
Aae-1-968	NW_001810952.1	+	519409	519464
Aae-1-969	NW_001810953.1	+	414661	414716
Aae-1-970	NW_001810955.1	+	438014	438069

Aae-1-971	NW_001810955.1	+	706815	706870
Aae-1-972	NW_001810955.1	+	1381035	1381090
Aae-1-973	NW_001810955.1	+	1417856	1417911
Aae-1-974	NW_001810955.1	+	3271691	3271746
Aae-1-975	NW_001810955.1	+	3726454	3726509
Aae-1-976	NW_001810955.1	-	3738193	3738138
Aae-1-977	NW_001810955.1	-	2414182	2414127
Aae-1-978	NW_001810955.1	-	2112262	2112207
Aae-1-979	NW_001810955.1	-	1414626	1414571
Aae-1-980	NW_001810956.1	+	1092969	1093024
Aae-1-981	NW_001810956.1	-	1584101	1584046
Aae-1-982	NW_001810958.1	+	855248	855303
Aae-1-983	NW_001810959.1	+	415531	415586
Aae-1-984	NW_001810960.1	+	573565	573620
Aae-1-985	NW_001810960.1	-	1156244	1156189
Aae-1-986	NW_001810960.1	-	1152927	1152872
Aae-1-987	NW_001810962.1	+	260146	260201
Aae-1-988	NW_001810963.1	+	1058838	1058893
Aae-1-989	NW_001810963.1	-	986042	985987
Aae-1-990	NW_001810964.1	+	849329	849384
Aae-1-991	NW_001810964.1	-	1410653	1410598
Aae-1-992	NW_001810964.1	-	413689	413634
Aae-1-993	NW_001810966.1	-	631187	631132
Aae-1-994	NW_001810967.1	+	666022	666077
Aae-1-995	NW_001810967.1	+	876311	876366
Aae-1-996	NW_001810970.1	+	726910	726965
Aae-1-997	NW_001810970.1	+	3407437	3407492
Aae-1-998	NW_001810970.1	+	3675619	3675674
Aae-1-999	NW_001810972.1	+	597407	597462
Aae-1-1000	NW_001810975.1	-	883258	883203
Aae-1-1001	NW_001810977.1	+	903758	903813
Aae-1-1002	NW_001810977.1	+	1533627	1533682
Aae-1-1003	NW_001810979.1	-	1481761	1481706
Aae-1-1004	NW_001810979.1	-	1328855	1328800
Aae-1-1005	NW_001810981.1	+	377239	377294
Aae-1-1006	NW_001810981.1	+	481930	481985
Aae-1-1007	NW_001810981.1	+	670379	670434
Aae-1-1008	NW_001810981.1	+	937263	937318
Aae-1-1009	NW_001810982.1	+	585060	585115
Aae-1-1010	NW_001810984.1	+	1723274	1723329
Aae-1-1011	NW_001810984.1	+	2727340	2727395
Aae-1-1012	NW_001810984.1	-	1125962	1125907
Aae-1-1013	NW_001810985.1	+	990804	990859
Aae-1-1014	NW_001810986.1	+	994812	994867
Aae-1-1015	NW_001810986.1	-	1094582	1094527
Aae-1-1016	NW_001810986.1	-	992198	992143
Aae-1-1017	NW_001810988.1	+	1226889	1226944
Aae-1-1018	NW_001810990.1	+	694052	694107
Aae-1-1019	NW_001810990.1	-	1057393	1057338
Aae-1-1020	NW_001810990.1	-	287169	287114
Aae-1-1021	NW_001810991.1	+	950808	950863
Aae-1-1022	NW_001810991.1	-	274031	273976

Aae-1-1023	NW_001810993.1	-	991966	991911
Aae-1-1024	NW_001810993.1	-	871145	871090
Aae-1-1025	NW_001810994.1	-	1174703	1174648
Aae-1-1026	NW_001810994.1	-	659887	659832
Aae-1-1027	NW_001810994.1	-	467024	466969
Aae-1-1028	NW_001810995.1	+	1154550	1154605
Aae-1-1029	NW_001810996.1	+	2363818	2363873
Aae-1-1030	NW_001810996.1	-	1918385	1918330
Aae-1-1031	NW_001810996.1	-	1393362	1393307
Aae-1-1032	NW_001810996.1	-	99018	98963
Aae-1-1033	NW_001810997.1	+	129121	129176
Aae-1-1034	NW_001810997.1	+	890766	890821
Aae-1-1035	NW_001810997.1	-	1489167	1489112
Aae-1-1036	NW_001810999.1	+	128449	128504
Aae-1-1037	NW_001810999.1	+	640603	640658
Aae-1-1038	NW_001810999.1	-	1197248	1197193
Aae-1-1039	NW_001810999.1	-	126769	126714
Aae-1-1040	NW_001811000.1	-	645801	645746
Aae-1-1041	NW_001811001.1	+	81515	81570
Aae-1-1042	NW_001811001.1	-	413212	413157
Aae-1-1043	NW_001811002.1	-	1186948	1186893
Aae-1-1044	NW_001811003.1	-	1394726	1394671
Aae-1-1045	NW_001811003.1	-	621252	621197
Aae-1-1046	NW_001811004.1	+	52862	52917
Aae-1-1047	NW_001811004.1	+	1000089	1000144
Aae-1-1048	NW_001811004.1	+	1006340	1006395
Aae-1-1049	NW_001811004.1	+	1140805	1140860
Aae-1-1050	NW_001811004.1	-	355513	355458
Aae-1-1051	NW_001811005.1	+	134285	134340
Aae-1-1052	NW_001811005.1	-	1179589	1179534
Aae-1-1053	NW_001811006.1	+	1316871	1316926
Aae-1-1054	NW_001811006.1	-	690136	690081
Aae-1-1055	NW_001811006.1	-	569408	569353
Aae-1-1056	NW_001811006.1	-	10404	10349
Aae-1-1057	NW_001811007.1	+	2061621	2061676
Aae-1-1058	NW_001811007.1	+	3044252	3044307
Aae-1-1059	NW_001811007.1	+	3126313	3126368
Aae-1-1060	NW_001811007.1	+	3454192	3454247
Aae-1-1061	NW_001811007.1	-	3229023	3228968
Aae-1-1062	NW_001811007.1	-	3126273	3126218
Aae-1-1063	NW_001811008.1	+	23075	23130
Aae-1-1064	NW_001811008.1	-	1157698	1157643
Aae-1-1065	NW_001811009.1	+	760155	760210
Aae-1-1066	NW_001811009.1	-	29060	29005
Aae-1-1067	NW_001811010.1	-	515239	515184
Aae-1-1068	NW_001811012.1	+	581055	581110
Aae-1-1069	NW_001811013.1	+	1004711	1004766
Aae-1-1070	NW_001811015.1	+	1218944	1218999
Aae-1-1071	NW_001811016.1	-	204242	204187
Aae-1-1072	NW_001811017.1	+	100721	100776
Aae-1-1073	NW_001811018.1	+	3097546	3097601
Aae-1-1074	NW_001811018.1	+	3869614	3869669

Aae-1-1075	NW_001811019.1	+	2028300	2028355
Aae-1-1076	NW_001811019.1	-	2999100	2999045
Aae-1-1077	NW_001811019.1	-	2778947	2778892
Aae-1-1078	NW_001811020.1	+	558139	558194
Aae-1-1079	NW_001811020.1	-	557323	557268
Aae-1-1080	NW_001811021.1	+	1326751	1326806
Aae-1-1081	NW_001811021.1	-	1070326	1070271
Aae-1-1082	NW_001811022.1	+	957534	957589
Aae-1-1083	NW_001811022.1	+	1014944	1014999
Aae-1-1084	NW_001811022.1	-	632806	632751
Aae-1-1085	NW_001811024.1	-	854270	854215
Aae-1-1086	NW_001811025.1	+	158908	158963
Aae-1-1087	NW_001811026.1	+	292473	292528
Aae-1-1088	NW_001811026.1	-	831171	831116
Aae-1-1089	NW_001811026.1	-	173078	173023
Aae-1-1090	NW_001811027.1	+	617253	617308
Aae-1-1091	NW_001811027.1	+	1051549	1051604
Aae-1-1092	NW_001811029.1	+	228176	228231
Aae-1-1093	NW_001811029.1	+	724900	724955
Aae-1-1094	NW_001811029.1	-	861388	861333
Aae-1-1095	NW_001811029.1	-	825848	825793
Aae-1-1096	NW_001811030.1	+	1974029	1974084
Aae-1-1097	NW_001811030.1	+	2000867	2000922
Aae-1-1098	NW_001811030.1	-	3080948	3080893
Aae-1-1099	NW_001811030.1	-	1816013	1815958
Aae-1-1100	NW_001811031.1	+	709744	709799
Aae-1-1101	NW_001811031.1	+	1092450	1092505
Aae-1-1102	NW_001811031.1	-	280743	280688
Aae-1-1103	NW_001811032.1	+	133538	133593
Aae-1-1104	NW_001811032.1	-	130661	130606
Aae-1-1105	NW_001811036.1	+	11	66
Aae-1-1106	NW_001811036.1	+	732585	732640
Aae-1-1107	NW_001811036.1	-	61862	61807
Aae-1-1108	NW_001811038.1	+	407493	407548
Aae-1-1109	NW_001811038.1	-	404345	404290
Aae-1-1110	NW_001811040.1	+	1065605	1065660
Aae-1-1111	NW_001811040.1	-	761116	761061
Aae-1-1112	NW_001811041.1	+	1611004	1611059
Aae-1-1113	NW_001811041.1	+	2539589	2539644
Aae-1-1114	NW_001811041.1	-	165635	165580
Aae-1-1115	NW_001811042.1	-	833886	833831
Aae-1-1116	NW_001811043.1	+	769555	769610
Aae-1-1117	NW_001811043.1	-	1052247	1052192
Aae-1-1118	NW_001811043.1	-	182177	182122
Aae-1-1119	NW_001811044.1	-	1148917	1148862
Aae-1-1120	NW_001811045.1	-	377668	377613
Aae-1-1121	NW_001811046.1	+	1081314	1081369
Aae-1-1122	NW_001811047.1	-	1028821	1028766
Aae-1-1123	NW_001811048.1	-	172943	172888
Aae-1-1124	NW_001811049.1	+	934979	935034
Aae-1-1125	NW_001811049.1	-	930966	930911
Aae-1-1126	NW_001811050.1	+	198839	198894

Aae-1-1127	NW_001811050.1	-	197359	197304
Aae-1-1128	NW_001811052.1	+	3394557	3394612
Aae-1-1129	NW_001811052.1	-	1604978	1604923
Aae-1-1130	NW_001811053.1	-	994194	994139
Aae-1-1131	NW_001811054.1	+	592952	593007
Aae-1-1132	NW_001811055.1	+	1248758	1248813
Aae-1-1133	NW_001811055.1	-	1247658	1247603
Aae-1-1134	NW_001811056.1	+	703338	703393
Aae-1-1135	NW_001811056.1	+	706025	706080
Aae-1-1136	NW_001811056.1	-	702964	702909
Aae-1-1137	NW_001811057.1	+	704415	704470
Aae-1-1138	NW_001811058.1	+	993372	993427
Aae-1-1139	NW_001811062.1	+	967522	967577
Aae-1-1140	NW_001811063.1	+	458235	458290
Aae-1-1141	NW_001811063.1	+	1605062	1605117
Aae-1-1142	NW_001811063.1	-	2982437	2982382
Aae-1-1143	NW_001811063.1	-	454482	454427
Aae-1-1144	NW_001811065.1	+	798276	798331
Aae-1-1145	NW_001811065.1	-	698994	698939
Aae-1-1146	NW_001811066.1	-	1295978	1295923
Aae-1-1147	NW_001811067.1	-	1197294	1197239
Aae-1-1148	NW_001811068.1	+	399651	399706
Aae-1-1149	NW_001811068.1	-	815933	815878
Aae-1-1150	NW_001811068.1	-	6584	6529
Aae-1-1151	NW_001811069.1	+	989783	989838
Aae-1-1152	NW_001811069.1	+	1146459	1146514
Aae-1-1153	NW_001811071.1	-	880647	880592
Aae-1-1154	NW_001811072.1	+	418388	418443
Aae-1-1155	NW_001811072.1	+	563432	563487
Aae-1-1156	NW_001811074.1	+	2714862	2714917
Aae-1-1157	NW_001811074.1	-	3217908	3217853
Aae-1-1158	NW_001811076.1	+	47533	47588
Aae-1-1159	NW_001811076.1	+	464734	464789
Aae-1-1160	NW_001811076.1	-	1135827	1135772
Aae-1-1161	NW_001811076.1	-	806869	806814
Aae-1-1162	NW_001811077.1	+	262618	262673
Aae-1-1163	NW_001811077.1	-	461993	461938
Aae-1-1164	NW_001811078.1	-	248654	248599
Aae-1-1165	NW_001811079.1	-	605253	605198
Aae-1-1166	NW_001811079.1	-	308851	308796
Aae-1-1167	NW_001811082.1	+	1190141	1190196
Aae-1-1168	NW_001811083.1	-	622509	622454
Aae-1-1169	NW_001811085.1	+	1744589	1744644
Aae-1-1170	NW_001811085.1	-	2979851	2979796
Aae-1-1171	NW_001811085.1	-	1209273	1209218
Aae-1-1172	NW_001811089.1	-	525290	525235
Aae-1-1173	NW_001811090.1	+	22126	22181
Aae-1-1174	NW_001811091.1	-	1161169	1161114
Aae-1-1175	NW_001811091.1	-	664604	664549
Aae-1-1176	NW_001811093.1	+	94469	94524
Aae-1-1177	NW_001811094.1	+	991328	991383
Aae-1-1178	NW_001811094.1	-	797356	797301

Aae-1-1179	NW_001811095.1	+	957912	957967
Aae-1-1180	NW_001811096.1	+	946038	946093
Aae-1-1181	NW_001811096.1	-	1590825	1590770
Aae-1-1182	NW_001811096.1	-	766466	766411
Aae-1-1183	NW_001811097.1	+	44980	45035
Aae-1-1184	NW_001811097.1	+	590632	590687
Aae-1-1185	NW_001811098.1	-	551414	551359
Aae-1-1186	NW_001811101.1	-	329023	328968
Aae-1-1187	NW_001811102.1	-	817555	817500
Aae-1-1188	NW_001811107.1	+	241995	242050
Aae-1-1189	NW_001811107.1	+	695032	695087
Aae-1-1190	NW_001811107.1	+	766080	766135
Aae-1-1191	NW_001811107.1	+	1319426	1319481
Aae-1-1192	NW_001811107.1	+	1572425	1572480
Aae-1-1193	NW_001811107.1	+	1747781	1747836
Aae-1-1194	NW_001811107.1	-	2494394	2494339
Aae-1-1195	NW_001811107.1	-	2093901	2093846
Aae-1-1196	NW_001811107.1	-	1571119	1571064
Aae-1-1197	NW_001811107.1	-	1171402	1171347
Aae-1-1198	NW_001811108.1	+	968004	968059
Aae-1-1199	NW_001811108.1	-	283195	283140
Aae-1-1200	NW_001811109.1	-	402882	402827
Aae-1-1201	NW_001811110.1	+	386200	386255
Aae-1-1202	NW_001811110.1	+	905166	905221
Aae-1-1203	NW_001811110.1	+	993593	993648
Aae-1-1204	NW_001811110.1	-	380702	380647
Aae-1-1205	NW_001811111.1	+	1182130	1182185
Aae-1-1206	NW_001811115.1	-	730453	730398
Aae-1-1207	NW_001811115.1	-	378536	378481
Aae-1-1208	NW_001811115.1	-	160613	160558
Aae-1-1209	NW_001811116.1	+	1020082	1020137
Aae-1-1210	NW_001811116.1	-	1104652	1104597
Aae-1-1211	NW_001811116.1	-	181388	181333
Aae-1-1212	NW_001811118.1	+	287887	287942
Aae-1-1213	NW_001811118.1	+	1668050	1668105
Aae-1-1214	NW_001811118.1	+	3101689	3101744
Aae-1-1215	NW_001811118.1	-	255990	255935
Aae-1-1216	NW_001811119.1	+	901962	902017
Aae-1-1217	NW_001811119.1	+	1022982	1023037
Aae-1-1218	NW_001811119.1	-	360748	360693
Aae-1-1219	NW_001811120.1	+	571678	571733
Aae-1-1220	NW_001811120.1	-	414452	414397
Aae-1-1221	NW_001811121.1	-	854341	854286
Aae-1-1222	NW_001811122.1	+	397662	397717
Aae-1-1223	NW_001811124.1	-	766457	766402
Aae-1-1224	NW_001811124.1	-	281798	281743
Aae-1-1225	NW_001811125.1	+	173586	173641
Aae-1-1226	NW_001811126.1	+	450113	450168
Aae-1-1227	NW_001811126.1	+	624843	624898
Aae-1-1228	NW_001811126.1	-	617765	617710
Aae-1-1229	NW_001811126.1	-	449777	449722
Aae-1-1230	NW_001811126.1	-	240751	240696

Aae-1-1231	NW_001811128.1	+	146547	146602
Aae-1-1232	NW_001811129.1	+	1662264	1662319
Aae-1-1233	NW_001811129.1	+	1677784	1677839
Aae-1-1234	NW_001811129.1	+	4508883	4508938
Aae-1-1235	NW_001811129.1	-	3960901	3960846
Aae-1-1236	NW_001811129.1	-	3351022	3350967
Aae-1-1237	NW_001811129.1	-	2585093	2585038
Aae-1-1238	NW_001811130.1	+	141951	142006
Aae-1-1239	NW_001811130.1	+	1669538	1669593
Aae-1-1240	NW_001811130.1	+	2442111	2442166
Aae-1-1241	NW_001811130.1	+	3128509	3128564
Aae-1-1242	NW_001811130.1	-	2143751	2143696
Aae-1-1243	NW_001811130.1	-	1646702	1646647
Aae-1-1244	NW_001811131.1	+	695760	695815
Aae-1-1245	NW_001811133.1	-	525286	525231
Aae-1-1246	NW_001811134.1	-	570917	570862
Aae-1-1247	NW_001811138.1	+	225	280
Aae-1-1248	NW_001811139.1	-	152023	151968
Aae-1-1249	NW_001811141.1	+	1829772	1829827
Aae-1-1250	NW_001811141.1	+	2922794	2922849
Aae-1-1251	NW_001811141.1	-	2842721	2842666
Aae-1-1252	NW_001811141.1	-	1431423	1431368
Aae-1-1253	NW_001811143.1	+	247925	247980
Aae-1-1254	NW_001811143.1	+	844473	844528
Aae-1-1255	NW_001811144.1	+	965657	965712
Aae-1-1256	NW_001811145.1	-	1039009	1038954
Aae-1-1257	NW_001811146.1	-	635577	635522
Aae-1-1258	NW_001811147.1	+	713425	713480
Aae-1-1259	NW_001811147.1	+	824934	824989
Aae-1-1260	NW_001811149.1	+	832123	832178
Aae-1-1261	NW_001811149.1	-	164597	164542
Aae-1-1262	NW_001811151.1	-	188595	188540
Aae-1-1263	NW_001811152.1	-	823771	823716
Aae-1-1264	NW_001811153.1	+	593309	593364
Aae-1-1265	NW_001811156.1	-	360994	360939
Aae-1-1266	NW_001811158.1	+	735333	735388
Aae-1-1267	NW_001811158.1	+	787674	787729
Aae-1-1268	NW_001811158.1	-	782140	782085
Aae-1-1269	NW_001811158.1	-	733653	733598
Aae-1-1270	NW_001811158.1	-	610873	610818
Aae-1-1271	NW_001811159.1	+	88374	88429
Aae-1-1272	NW_001811159.1	+	489536	489591
Aae-1-1273	NW_001811161.1	-	306444	306389
Aae-1-1274	NW_001811161.1	-	196254	196199
Aae-1-1275	NW_001811161.1	-	181033	180978
Aae-1-1276	NW_001811163.1	-	2698983	2698928
Aae-1-1277	NW_001811163.1	-	542100	542045
Aae-1-1278	NW_001811164.1	+	99707	99762
Aae-1-1279	NW_001811164.1	+	561452	561507
Aae-1-1280	NW_001811166.1	+	644119	644174
Aae-1-1281	NW_001811168.1	-	901990	901935
Aae-1-1282	NW_001811169.1	+	853599	853654

Aae-1-1283	NW_001811170.1	+	115370	115425
Aae-1-1284	NW_001811170.1	+	796409	796464
Aae-1-1285	NW_001811171.1	-	774381	774326
Aae-1-1286	NW_001811174.1	+	422245	422300
Aae-1-1287	NW_001811174.1	-	3231435	3231380
Aae-1-1288	NW_001811174.1	-	2080197	2080142
Aae-1-1289	NW_001811174.1	-	421187	421132
Aae-1-1290	NW_001811174.1	-	51370	51315
Aae-1-1291	NW_001811176.1	-	131371	131316
Aae-1-1292	NW_001811179.1	+	430125	430180
Aae-1-1293	NW_001811179.1	+	903287	903342
Aae-1-1294	NW_001811179.1	-	320368	320313
Aae-1-1295	NW_001811180.1	+	744278	744333
Aae-1-1296	NW_001811180.1	-	935518	935463
Aae-1-1297	NW_001811180.1	-	743322	743267
Aae-1-1298	NW_001811182.1	+	667303	667358
Aae-1-1299	NW_001811182.1	-	372156	372101
Aae-1-1300	NW_001811183.1	-	528769	528714
Aae-1-1301	NW_001811183.1	-	77397	77342
Aae-1-1302	NW_001811185.1	+	80941	80996
Aae-1-1303	NW_001811185.1	+	326344	326399
Aae-1-1304	NW_001811185.1	+	1777641	1777696
Aae-1-1305	NW_001811185.1	-	79712	79657
Aae-1-1306	NW_001811186.1	+	620204	620259
Aae-1-1307	NW_001811186.1	-	749590	749535
Aae-1-1308	NW_001811186.1	-	500790	500735
Aae-1-1309	NW_001811190.1	-	590271	590216
Aae-1-1310	NW_001811191.1	+	188777	188832
Aae-1-1311	NW_001811191.1	-	635969	635914
Aae-1-1312	NW_001811192.1	-	320274	320219
Aae-1-1313	NW_001811193.1	+	505674	505729
Aae-1-1314	NW_001811193.1	+	694509	694564
Aae-1-1315	NW_001811194.1	+	58480	58535
Aae-1-1316	NW_001811194.1	+	221574	221629
Aae-1-1317	NW_001811194.1	+	691744	691799
Aae-1-1318	NW_001811196.1	+	2880944	2880999
Aae-1-1319	NW_001811196.1	+	3156462	3156517
Aae-1-1320	NW_001811197.1	+	893031	893086
Aae-1-1321	NW_001811198.1	+	755135	755190
Aae-1-1322	NW_001811201.1	+	446488	446543
Aae-1-1323	NW_001811201.1	-	91384	91329
Aae-1-1324	NW_001811201.1	-	52684	52629
Aae-1-1325	NW_001811202.1	+	627061	627116
Aae-1-1326	NW_001811203.1	-	167041	166986
Aae-1-1327	NW_001811204.1	+	2824	2879
Aae-1-1328	NW_001811204.1	-	537631	537576
Aae-1-1329	NW_001811204.1	-	360069	360014
Aae-1-1330	NW_001811205.1	-	921347	921292
Aae-1-1331	NW_001811205.1	-	920182	920127
Aae-1-1332	NW_001811207.1	+	1413009	1413064
Aae-1-1333	NW_001811207.1	-	3076955	3076900
Aae-1-1334	NW_001811207.1	-	1229507	1229452

Aae-1-1335	NW_001811208.1	+	590622	590677
Aae-1-1336	NW_001811208.1	+	731899	731954
Aae-1-1337	NW_001811211.1	-	324779	324724
Aae-1-1338	NW_001811214.1	+	201165	201220
Aae-1-1339	NW_001811214.1	-	576032	575977
Aae-1-1340	NW_001811216.1	+	937902	937957
Aae-1-1341	NW_001811217.1	-	250158	250103
Aae-1-1342	NW_001811218.1	-	151084	151029
Aae-1-1343	NW_001811219.1	+	453003	453058
Aae-1-1344	NW_001811219.1	+	806916	806971
Aae-1-1345	NW_001811219.1	-	194698	194643
Aae-1-1346	NW_001811221.1	+	293288	293343
Aae-1-1347	NW_001811221.1	+	381856	381911
Aae-1-1348	NW_001811221.1	+	801787	801842
Aae-1-1349	NW_001811221.1	+	872950	873005
Aae-1-1350	NW_001811222.1	-	749825	749770
Aae-1-1351	NW_001811222.1	-	530609	530554
Aae-1-1352	NW_001811225.1	+	382895	382950
Aae-1-1353	NW_001811226.1	-	937204	937149
Aae-1-1354	NW_001811227.1	-	580079	580024
Aae-1-1355	NW_001811227.1	-	342852	342797
Aae-1-1356	NW_001811228.1	+	161708	161763
Aae-1-1357	NW_001811229.1	+	305057	305112
Aae-1-1358	NW_001811230.1	+	750538	750593
Aae-1-1359	NW_001811230.1	-	202749	202694
Aae-1-1360	NW_001811232.1	+	686391	686446
Aae-1-1361	NW_001811232.1	-	709942	709887
Aae-1-1362	NW_001811235.1	+	357395	357450
Aae-1-1363	NW_001811235.1	+	885565	885620
Aae-1-1364	NW_001811235.1	-	438711	438656
Aae-1-1365	NW_001811240.1	-	3359601	3359546
Aae-1-1366	NW_001811240.1	-	28993	28938
Aae-1-1367	NW_001811241.1	-	608137	608082
Aae-1-1368	NW_001811243.1	+	780687	780742
Aae-1-1369	NW_001811244.1	+	41772	41827
Aae-1-1370	NW_001811244.1	+	437711	437766
Aae-1-1371	NW_001811245.1	+	610567	610622
Aae-1-1372	NW_001811245.1	-	607124	607069
Aae-1-1373	NW_001811248.1	-	444406	444351
Aae-1-1374	NW_001811252.1	+	1624133	1624188
Aae-1-1375	NW_001811252.1	+	2637725	2637780
Aae-1-1376	NW_001811252.1	-	1896349	1896294
Aae-1-1377	NW_001811254.1	+	754057	754112
Aae-1-1378	NW_001811255.1	+	184571	184626
Aae-1-1379	NW_001811255.1	-	852606	852551
Aae-1-1380	NW_001811255.1	-	183007	182952
Aae-1-1381	NW_001811257.1	-	506576	506521
Aae-1-1382	NW_001811259.1	+	242417	242472
Aae-1-1383	NW_001811259.1	-	157666	157611
Aae-1-1384	NW_001811260.1	+	279146	279201
Aae-1-1385	NW_001811260.1	-	295898	295843
Aae-1-1386	NW_001811260.1	-	284418	284363

Aae-1-1387	NW_001811262.1	+	308771	308826
Aae-1-1388	NW_001811263.1	+	851335	851390
Aae-1-1389	NW_001811263.1	-	2172182	2172127
Aae-1-1390	NW_001811264.1	+	360319	360374
Aae-1-1391	NW_001811266.1	+	293575	293630
Aae-1-1392	NW_001811266.1	+	567036	567091
Aae-1-1393	NW_001811267.1	+	667235	667290
Aae-1-1394	NW_001811268.1	-	802187	802132
Aae-1-1395	NW_001811272.1	-	656586	656531
Aae-1-1396	NW_001811273.1	-	652327	652272
Aae-1-1397	NW_001811274.1	+	2106991	2107046
Aae-1-1398	NW_001811274.1	+	2459085	2459140
Aae-1-1399	NW_001811274.1	-	660668	660613
Aae-1-1400	NW_001811276.1	-	124498	124443
Aae-1-1401	NW_001811277.1	+	662355	662410
Aae-1-1402	NW_001811277.1	-	658786	658731
Aae-1-1403	NW_001811279.1	+	802169	802224
Aae-1-1404	NW_001811280.1	-	552806	552751
Aae-1-1405	NW_001811283.1	-	156344	156289
Aae-1-1406	NW_001811284.1	-	114386	114331
Aae-1-1407	NW_001811284.1	-	75794	75739
Aae-1-1408	NW_001811285.1	+	352730	352785
Aae-1-1409	NW_001811285.1	+	2879605	2879660
Aae-1-1410	NW_001811285.1	-	2878710	2878655
Aae-1-1411	NW_001811285.1	-	2318339	2318284
Aae-1-1412	NW_001811285.1	-	1346879	1346824
Aae-1-1413	NW_001811285.1	-	1019970	1019915
Aae-1-1414	NW_001811287.1	-	535766	535711
Aae-1-1415	NW_001811288.1	+	1003	1058
Aae-1-1416	NW_001811288.1	-	521643	521588
Aae-1-1417	NW_001811288.1	-	476741	476686
Aae-1-1418	NW_001811296.1	+	2713349	2713404
Aae-1-1419	NW_001811296.1	-	463229	463174
Aae-1-1420	NW_001811297.1	-	630618	630563
Aae-1-1421	NW_001811297.1	-	628706	628651
Aae-1-1422	NW_001811298.1	+	267622	267677
Aae-1-1423	NW_001811298.1	-	490505	490450
Aae-1-1424	NW_001811299.1	-	407795	407740
Aae-1-1425	NW_001811299.1	-	405097	405042
Aae-1-1426	NW_001811300.1	+	773624	773679
Aae-1-1427	NW_001811300.1	-	773135	773080
Aae-1-1428	NW_001811300.1	-	308762	308707
Aae-1-1429	NW_001811301.1	+	327197	327252
Aae-1-1430	NW_001811301.1	-	730450	730395
Aae-1-1431	NW_001811302.1	-	362182	362127
Aae-1-1432	NW_001811304.1	+	343528	343583
Aae-1-1433	NW_001811304.1	+	548707	548762
Aae-1-1434	NW_001811307.1	+	1103891	1103946
Aae-1-1435	NW_001811307.1	+	2377214	2377269
Aae-1-1436	NW_001811307.1	-	2210720	2210665
Aae-1-1437	NW_001811308.1	-	529408	529353
Aae-1-1438	NW_001811309.1	+	608837	608892

Aae-1-1439	NW_001811309.1	-	607493	607438
Aae-1-1440	NW_001811310.1	+	662486	662541
Aae-1-1441	NW_001811312.1	+	231532	231587
Aae-1-1442	NW_001811313.1	+	80031	80086
Aae-1-1443	NW_001811313.1	+	82743	82798
Aae-1-1444	NW_001811313.1	-	518267	518212
Aae-1-1445	NW_001811313.1	-	77031	76976
Aae-1-1446	NW_001811315.1	+	241210	241265
Aae-1-1447	NW_001811315.1	-	670242	670187
Aae-1-1448	NW_001811317.1	+	262657	262712
Aae-1-1449	NW_001811317.1	+	499723	499778
Aae-1-1450	NW_001811322.1	+	690391	690446
Aae-1-1451	NW_001811323.1	-	254790	254735
Aae-1-1452	NW_001811329.1	+	405791	405846
Aae-1-1453	NW_001811329.1	+	1874274	1874329
Aae-1-1454	NW_001811329.1	+	1929849	1929904
Aae-1-1455	NW_001811329.1	+	2006024	2006079
Aae-1-1456	NW_001811329.1	-	2241258	2241203
Aae-1-1457	NW_001811329.1	-	1808507	1808452
Aae-1-1458	NW_001811331.1	-	727849	727794
Aae-1-1459	NW_001811332.1	+	612611	612666
Aae-1-1460	NW_001811334.1	-	99446	99391
Aae-1-1461	NW_001811334.1	-	9715	9660
Aae-1-1462	NW_001811335.1	-	572528	572473
Aae-1-1463	NW_001811336.1	+	457628	457683
Aae-1-1464	NW_001811337.1	+	365238	365293
Aae-1-1465	NW_001811339.1	+	648178	648233
Aae-1-1466	NW_001811340.1	-	2841166	2841111
Aae-1-1467	NW_001811345.1	-	375920	375865
Aae-1-1468	NW_001811345.1	-	267972	267917
Aae-1-1469	NW_001811350.1	+	651287	651342
Aae-1-1470	NW_001811350.1	-	688764	688709
Aae-1-1471	NW_001811351.1	+	11987	12042
Aae-1-1472	NW_001811351.1	+	179853	179908
Aae-1-1473	NW_001811351.1	-	3326764	3326709
Aae-1-1474	NW_001811351.1	-	2648595	2648540
Aae-1-1475	NW_001811351.1	-	1588606	1588551
Aae-1-1476	NW_001811352.1	+	515812	515867
Aae-1-1477	NW_001811352.1	+	655237	655292
Aae-1-1478	NW_001811352.1	+	2529569	2529624
Aae-1-1479	NW_001811352.1	-	448051	447996
Aae-1-1480	NW_001811354.1	+	303325	303380
Aae-1-1481	NW_001811354.1	-	301576	301521
Aae-1-1482	NW_001811356.1	-	426022	425967
Aae-1-1483	NW_001811357.1	-	149303	149248
Aae-1-1484	NW_001811360.1	-	469263	469208
Aae-1-1485	NW_001811362.1	-	684688	684633
Aae-1-1486	NW_001811363.1	+	15157	15212
Aae-1-1487	NW_001811363.1	+	1587475	1587530
Aae-1-1488	NW_001811363.1	-	2168220	2168165
Aae-1-1489	NW_001811364.1	+	142320	142375
Aae-1-1490	NW_001811366.1	+	2544	2599

Aae-1-1491	NW_001811366.1	+	153381	153436
Aae-1-1492	NW_001811366.1	-	491339	491284
Aae-1-1493	NW_001811367.1	-	431098	431043
Aae-1-1494	NW_001811367.1	-	123844	123789
Aae-1-1495	NW_001811368.1	+	649991	650046
Aae-1-1496	NW_001811371.1	+	464784	464839
Aae-1-1497	NW_001811371.1	+	566496	566551
Aae-1-1498	NW_001811372.1	+	411520	411575
Aae-1-1499	NW_001811372.1	-	648874	648819
Aae-1-1500	NW_001811373.1	-	202663	202608
Aae-1-1501	NW_001811374.1	+	49936	49991
Aae-1-1502	NW_001811375.1	-	529825	529770
Aae-1-1503	NW_001811375.1	-	29892	29837
Aae-1-1504	NW_001811377.1	+	466635	466690
Aae-1-1505	NW_001811377.1	-	453492	453437
Aae-1-1506	NW_001811379.1	-	218952	218897
Aae-1-1507	NW_001811382.1	-	715556	715501
Aae-1-1508	NW_001811383.1	+	179396	179451
Aae-1-1509	NW_001811383.1	+	186566	186621
Aae-1-1510	NW_001811385.1	+	515862	515917
Aae-1-1511	NW_001811385.1	+	1055382	1055437
Aae-1-1512	NW_001811385.1	-	1300754	1300699
Aae-1-1513	NW_001811385.1	-	277122	277067
Aae-1-1514	NW_001811392.1	-	552194	552139
Aae-1-1515	NW_001811396.1	-	847878	847823
Aae-1-1516	NW_001811398.1	+	222871	222926
Aae-1-1517	NW_001811403.1	-	633548	633493
Aae-1-1518	NW_001811403.1	-	571048	570993
Aae-1-1519	NW_001811406.1	+	254020	254075
Aae-1-1520	NW_001811406.1	+	466889	466944
Aae-1-1521	NW_001811407.1	+	3097	3152
Aae-1-1522	NW_001811407.1	+	96184	96239
Aae-1-1523	NW_001811407.1	+	1798052	1798107
Aae-1-1524	NW_001811407.1	+	2655734	2655789
Aae-1-1525	NW_001811407.1	+	2871653	2871708
Aae-1-1526	NW_001811407.1	-	2952592	2952537
Aae-1-1527	NW_001811409.1	-	635486	635431
Aae-1-1528	NW_001811412.1	+	479319	479374
Aae-1-1529	NW_001811413.1	+	605977	606032
Aae-1-1530	NW_001811415.1	-	423148	423093
Aae-1-1531	NW_001811416.1	-	623820	623765
Aae-1-1532	NW_001811417.1	-	537220	537165
Aae-1-1533	NW_001811417.1	-	107618	107563
Aae-1-1534	NW_001811418.1	+	1534073	1534128
Aae-1-1535	NW_001811418.1	-	2169205	2169150
Aae-1-1536	NW_001811419.1	-	453338	453283
Aae-1-1537	NW_001811426.1	-	699999	699944
Aae-1-1538	NW_001811429.1	-	1848225	1848170
Aae-1-1539	NW_001811437.1	+	199414	199469
Aae-1-1540	NW_001811437.1	-	194703	194648
Aae-1-1541	NW_001811440.1	+	893809	893864
Aae-1-1542	NW_001811441.1	+	390304	390359

Aae-1-1543	NW_001811441.1	+	565672	565727
Aae-1-1544	NW_001811445.1	+	7637	7692
Aae-1-1545	NW_001811445.1	-	100707	100652
Aae-1-1546	NW_001811445.1	-	7066	7011
Aae-1-1547	NW_001811449.1	+	124714	124769
Aae-1-1548	NW_001811450.1	+	327429	327484
Aae-1-1549	NW_001811451.1	+	1345703	1345758
Aae-1-1550	NW_001811451.1	-	2119456	2119401
Aae-1-1551	NW_001811454.1	+	395	450
Aae-1-1552	NW_001811457.1	-	410443	410388
Aae-1-1553	NW_001811458.1	-	325801	325746
Aae-1-1554	NW_001811462.1	+	1060870	1060925
Aae-1-1555	NW_001811462.1	+	1309701	1309756
Aae-1-1556	NW_001811462.1	+	2824134	2824189
Aae-1-1557	NW_001811462.1	+	3173779	3173834
Aae-1-1558	NW_001811462.1	-	3417954	3417899
Aae-1-1559	NW_001811462.1	-	1307460	1307405
Aae-1-1560	NW_001811466.1	+	336100	336155
Aae-1-1561	NW_001811473.1	+	324744	324799
Aae-1-1562	NW_001811474.1	+	1806624	1806679
Aae-1-1563	NW_001811474.1	-	2692960	2692905
Aae-1-1564	NW_001811474.1	-	2207113	2207058
Aae-1-1565	NW_001811474.1	-	1857426	1857371
Aae-1-1566	NW_001811476.1	-	420437	420382
Aae-1-1567	NW_001811476.1	-	298887	298832
Aae-1-1568	NW_001811477.1	+	117975	118030
Aae-1-1569	NW_001811477.1	+	225256	225311
Aae-1-1570	NW_001811477.1	+	397986	398041
Aae-1-1571	NW_001811484.1	-	519838	519783
Aae-1-1572	NW_001811492.1	-	108639	108584
Aae-1-1573	NW_001811493.1	+	93670	93725
Aae-1-1574	NW_001811495.1	+	452671	452726
Aae-1-1575	NW_001811495.1	+	476171	476226
Aae-1-1576	NW_001811496.1	+	1870000	1870055
Aae-1-1577	NW_001811496.1	+	2014695	2014750
Aae-1-1578	NW_001811501.1	+	53248	53303
Aae-1-1579	NW_001811502.1	-	69937	69882
Aae-1-1580	NW_001811504.1	-	50599	50544
Aae-1-1581	NW_001811506.1	+	278108	278163
Aae-1-1582	NW_001811512.1	+	13100	13155
Aae-1-1583	NW_001811514.1	+	60021	60076
Aae-1-1584	NW_001811514.1	+	95710	95765
Aae-1-1585	NW_001811514.1	+	290737	290792
Aae-1-1586	NW_001811514.1	+	600234	600289
Aae-1-1587	NW_001811514.1	-	447376	447321
Aae-1-1588	NW_001811514.1	-	287398	287343
Aae-1-1589	NW_001811514.1	-	284696	284641
Aae-1-1590	NW_001811517.1	+	107569	107624
Aae-1-1591	NW_001811519.1	-	135294	135239
Aae-1-1592	NW_001811521.1	-	369012	368957
Aae-1-1593	NW_001811527.1	-	306976	306921
Aae-1-1594	NW_001811528.1	-	515581	515526

Aae-1-1595	NW_001811528.1	-	152460	152405
Aae-1-1596	NW_001811529.1	+	1541871	1541926
Aae-1-1597	NW_001811529.1	+	2631610	2631665
Aae-1-1598	NW_001811529.1	-	2456583	2456528
Aae-1-1599	NW_001811529.1	-	1540375	1540320
Aae-1-1600	NW_001811533.1	-	359998	359943
Aae-1-1601	NW_001811534.1	+	96204	96259
Aae-1-1602	NW_001811534.1	-	418610	418555
Aae-1-1603	NW_001811535.1	-	344781	344726
Aae-1-1604	NW_001811540.1	+	2652824	2652879
Aae-1-1605	NW_001811540.1	-	2586657	2586602
Aae-1-1606	NW_001811544.1	-	55644	55589
Aae-1-1607	NW_001811548.1	+	218373	218428
Aae-1-1608	NW_001811549.1	+	34825	34880
Aae-1-1609	NW_001811550.1	-	413372	413317
Aae-1-1610	NW_001811551.1	+	376196	376251
Aae-1-1611	NW_001811551.1	+	518405	518460
Aae-1-1612	NW_001811551.1	+	1817314	1817369
Aae-1-1613	NW_001811555.1	+	469456	469511
Aae-1-1614	NW_001811562.1	+	183769	183824
Aae-1-1615	NW_001811562.1	+	581048	581103
Aae-1-1616	NW_001811562.1	+	808168	808223
Aae-1-1617	NW_001811562.1	+	2484478	2484533
Aae-1-1618	NW_001811563.1	-	123376	123321
Aae-1-1619	NW_001811565.1	-	53657	53602
Aae-1-1620	NW_001811567.1	-	300043	299988
Aae-1-1621	NW_001811571.1	+	426245	426300
Aae-1-1622	NW_001811573.1	+	118844	118899
Aae-1-1623	NW_001811573.1	-	4897822	4897767
Aae-1-1624	NW_001811573.1	-	4791962	4791907
Aae-1-1625	NW_001811573.1	-	52538	52483
Aae-1-1626	NW_001811574.1	-	2208461	2208406
Aae-1-1627	NW_001811574.1	-	2205763	2205708
Aae-1-1628	NW_001811574.1	-	1550457	1550402
Aae-1-1629	NW_001811575.1	-	254817	254762
Aae-1-1630	NW_001811580.1	+	518	573
Aae-1-1631	NW_001811584.1	+	58436	58491
Aae-1-1632	NW_001811590.1	-	286729	286674
Aae-1-1633	NW_001811591.1	+	252434	252489
Aae-1-1634	NW_001811596.1	-	728778	728723
Aae-1-1635	NW_001811596.1	-	516121	516066
Aae-1-1636	NW_001811599.1	+	169378	169433
Aae-1-1637	NW_001811599.1	-	231051	230996
Aae-1-1638	NW_001811602.1	-	135837	135782
Aae-1-1639	NW_001811603.1	+	153028	153083
Aae-1-1640	NW_001811606.1	-	198728	198673
Aae-1-1641	NW_001811607.1	+	542953	543008
Aae-1-1642	NW_001811607.1	+	1601107	1601162
Aae-1-1643	NW_001811607.1	+	1742536	1742591
Aae-1-1644	NW_001811607.1	+	2783872	2783927
Aae-1-1645	NW_001811608.1	+	119011	119066
Aae-1-1646	NW_001811609.1	+	7069	7124

Aae-1-1647	NW_001811609.1	-	164441	164386
Aae-1-1648	NW_001811610.1	+	164818	164873
Aae-1-1649	NW_001811612.1	-	184378	184323
Aae-1-1650	NW_001811612.1	-	168521	168466
Aae-1-1651	NW_001811614.1	-	402533	402478
Aae-1-1652	NW_001811618.1	+	761260	761315
Aae-1-1653	NW_001811618.1	-	1116512	1116457
Aae-1-1654	NW_001811618.1	-	886296	886241
Aae-1-1655	NW_001811620.1	+	248992	249047
Aae-1-1656	NW_001811621.1	-	353145	353090
Aae-1-1657	NW_001811623.1	-	427452	427397
Aae-1-1658	NW_001811623.1	-	424865	424810
Aae-1-1659	NW_001811623.1	-	71971	71916
Aae-1-1660	NW_001811627.1	+	177838	177893
Aae-1-1661	NW_001811627.1	+	374211	374266
Aae-1-1662	NW_001811631.1	+	44387	44442
Aae-1-1663	NW_001811635.1	+	326426	326481
Aae-1-1664	NW_001811635.1	-	56533	56478
Aae-1-1665	NW_001811639.1	-	421858	421803
Aae-1-1666	NW_001811640.1	+	1295357	1295412
Aae-1-1667	NW_001811641.1	+	258946	259001
Aae-1-1668	NW_001811645.1	+	156090	156145
Aae-1-1669	NW_001811648.1	+	84539	84594
Aae-1-1670	NW_001811650.1	-	188180	188125
Aae-1-1671	NW_001811651.1	+	132447	132502
Aae-1-1672	NW_001811651.1	+	223125	223180
Aae-1-1673	NW_001811651.1	+	2669699	2669754
Aae-1-1674	NW_001811651.1	-	1653622	1653567
Aae-1-1675	NW_001811651.1	-	111766	111711
Aae-1-1676	NW_001811652.1	-	35696	35641
Aae-1-1677	NW_001811661.1	+	363931	363986
Aae-1-1678	NW_001811662.1	-	18510	18455
Aae-1-1679	NW_001811665.1	+	143486	143541
Aae-1-1680	NW_001811666.1	-	276802	276747
Aae-1-1681	NW_001811671.1	+	352480	352535
Aae-1-1682	NW_001811672.1	-	95421	95366
Aae-1-1683	NW_001811672.1	-	17055	17000
Aae-1-1684	NW_001811673.1	+	144176	144231
Aae-1-1685	NW_001811673.1	+	926486	926541
Aae-1-1686	NW_001811677.1	+	279024	279079
Aae-1-1687	NW_001811684.1	+	4310742	4310797
Aae-1-1688	NW_001811692.1	-	124763	124708
Aae-1-1689	NW_001811692.1	-	92279	92224
Aae-1-1690	NW_001811695.1	-	120472	120417
Aae-1-1691	NW_001811702.1	+	253824	253879
Aae-1-1692	NW_001811703.1	+	376130	376185
Aae-1-1693	NW_001811705.1	+	151255	151310
Aae-1-1694	NW_001811705.1	+	215778	215833
Aae-1-1695	NW_001811707.1	+	1837305	1837360
Aae-1-1696	NW_001811708.1	-	303644	303589
Aae-1-1697	NW_001811711.1	+	210465	210520
Aae-1-1698	NW_001811711.1	-	184211	184156

Aae-1-1699	NW_001811717.1	-	81994	81939
Aae-1-1700	NW_001811718.1	+	1039034	1039089
Aae-1-1701	NW_001811718.1	-	51747	51692
Aae-1-1702	NW_001811725.1	+	241695	241750
Aae-1-1703	NW_001811727.1	+	247922	247977
Aae-1-1704	NW_001811727.1	-	342820	342765
Aae-1-1705	NW_001811729.1	-	2511832	2511777
Aae-1-1706	NW_001811729.1	-	1491583	1491528
Aae-1-1707	NW_001811732.1	-	280985	280930
Aae-1-1708	NW_001811739.1	-	1866506	1866451
Aae-1-1709	NW_001811740.1	+	285803	285858
Aae-1-1710	NW_001811748.1	+	10926	10981
Aae-1-1711	NW_001811749.1	-	146301	146246
Aae-1-1712	NW_001811750.1	+	1668879	1668934
Aae-1-1713	NW_001811752.1	-	105881	105826
Aae-1-1714	NW_001811758.1	-	35731	35676
Aae-1-1715	NW_001811761.1	-	1737208	1737153
Aae-1-1716	NW_001811762.1	+	312132	312187
Aae-1-1717	NW_001811766.1	+	22910	22965
Aae-1-1718	NW_001811772.1	+	1536584	1536639
Aae-1-1719	NW_001811776.1	+	75400	75455
Aae-1-1720	NW_001811781.1	+	28	83
Aae-1-1721	NW_001811783.1	+	1939693	1939748
Aae-1-1722	NW_001811783.1	-	2034606	2034551
Aae-1-1723	NW_001811783.1	-	1938195	1938140
Aae-1-1724	NW_001811783.1	-	1935497	1935442
Aae-1-1725	NW_001811786.1	-	51686	51631
Aae-1-1726	NW_001811789.1	+	175668	175723
Aae-1-1727	NW_001835323.1	+	4843	4898
Aae-1-1728	NW_001835328.1	-	4212	4157
Aae-1-1729	NW_001835585.1	+	2067	2122
Aae-1-1730	NW_001835623.1	-	4236	4181
Aae-1-1731	NW_001835701.1	+	5440	5495
Aae-1-1732	NW_001835894.1	-	5438	5383
Aae-1-1733	NW_001835948.1	-	5982	5927
Aae-1-1734	NW_001836081.1	-	1732	1677
Aae-1-1735	NW_001836302.1	-	1196	1141
Aae-1-1736	NW_001836630.1	-	2016	1961
Aae-1-1737	NW_001836900.1	+	610	665
Aae-1-1738	NW_001837216.1	-	9348	9293
Aae-1-1739	NW_001837245.1	-	4567	4512
Aae-1-1740	NW_001837408.1	+	249	304
Aae-1-1741	NW_001837454.1	+	10149	10204
Aae-1-1742	NW_001837458.1	+	86960	87015
Aae-1-1743	NW_001811163.1	+	2712162	2712217
Aae-1-1744	NW_001835570.1	+	852	907
Aae-1-1745	NW_001811291.1	-	556922	556867
Aae-1-1746	NW_001811196.1	+	1996954	1997009
Aae-1-1747	NW_001811007.1	+	3712768	3712823
Aae-1-1748	NW_001811109.1	-	1090239	1090184
Aae-1-1749	NW_001811479.1	-	189402	189347
Aae-1-1750	NW_001810955.1	-	3582266	3582211

Aae-1-1751	NW_001810721.1	-	406691	406636
Aae-1-1752	NW_001811385.1	+	1172634	1172689
Aae-1-1753	NW_001811707.1	+	530006	530061
Aae-1-1754	NW_001810825.1	+	21180	21235
Aae-1-1755	NW_001811027.1	-	406252	406197
Aae-1-1756	NW_001811072.1	-	480385	480330
Aae-1-1757	NW_001811147.1	+	206073	206128
Aae-1-1758	NW_001811089.1	+	1004079	1004134
Aae-1-1759	NW_001810837.1	-	1804641	1804586
Aae-1-1760	NW_001810637.1	-	4045985	4045930
Aae-1-1761	NW_001810575.1	+	1758766	1758821
Aae-1-1762	NW_001811006.1	+	388550	388605
Aae-1-1763	NW_001810239.1	-	38461	38406
Aae-1-1764	NW_001810328.1	-	111804	111749
Aae-1-1765	NW_001810350.1	-	981366	981311
Aae-1-1766	NW_001810351.1	-	33293	33238
Aae-1-1767	NW_001810513.1	-	46230	46175
Aae-1-1768	NW_001810555.1	+	46039	46094
Aae-1-1769	NW_001810805.1	+	645511	645566
Aae-1-1770	NW_001810816.1	-	1466363	1466308
Aae-1-1771	NW_001810895.1	-	541099	541044
Aae-1-1772	NW_001810935.1	-	311723	311668
Aae-1-1773	NW_001810962.1	+	1544277	1544332
Aae-1-1774	NW_001810971.1	+	769965	770020
Aae-1-1775	NW_001810988.1	-	757065	757010
Aae-1-1776	NW_001810990.1	-	1029779	1029724
Aae-1-1777	NW_001811005.1	-	1307950	1307895
Aae-1-1778	NW_001811030.1	-	1973962	1973907
Aae-1-1779	NW_001811085.1	+	2774702	2774757
Aae-1-1780	NW_001811152.1	-	74535	74480
Aae-1-1781	NW_001811183.1	+	543144	543199
Aae-1-1782	NW_001811271.1	-	739372	739317
Aae-1-1783	NW_001811285.1	-	2151537	2151482
Aae-1-1784	NW_001811327.1	+	685756	685811
Aae-1-1785	NW_001811363.1	+	1697692	1697747
Aae-1-1786	NW_001811385.1	-	904665	904610
Aae-1-1787	NW_001811396.1	+	1488693	1488748
Aae-1-1788	NW_001811435.1	-	81224	81169
Aae-1-1789	NW_001811462.1	+	2938967	2939022
Aae-1-1790	NW_001811564.1	-	138518	138463
Aae-1-1791	NW_001811761.1	-	1173932	1173877
Aae-1-1792	NW_001811777.1	-	52563	52508
Aae-1-1793	NW_001810783.1	+	1158382	1158437
Aae-1-1794	NW_001810023.1	-	2352312	2352257
Aae-1-1795	NW_001810156.1	-	241648	241593
Aae-1-1796	NW_001810714.1	+	877637	877692
Aae-1-1797	NW_001810944.1	+	876472	876527
Aae-1-1798	NW_001810955.1	+	435123	435178
Aae-1-1799	NW_001810976.1	+	741594	741649
Aae-1-1800	NW_001810997.1	-	1457440	1457385
Aae-1-1801	NW_001811031.1	-	159731	159676
Aae-1-1802	NW_001811103.1	+	299708	299763

Aae-1-1803	NW_001811252.1	+	3074220	3074275
Aae-1-1804	NW_001811298.1	+	285540	285595
Aae-1-1805	NW_001811683.1	+	153395	153450
Aae-1-1806	NW_001811758.1	+	272563	272618
Aae-1-1807	NW_001811596.1	-	376774	376719
Aae-1-1808	NW_001811665.1	+	105006	105061
Aae-1-1809	NW_001811618.1	+	1241715	1241770
Aae-1-1810	NW_001811312.1	+	270790	270845
Aae-1-1811	NW_001811340.1	-	1681742	1681687
Aae-1-1812	NW_001811585.1	-	2636378	2636323
Aae-1-1813	NW_001811562.1	-	2129374	2129319
Aae-1-1814	NW_001811502.1	-	252496	252441
Aae-1-1815	NW_001811317.1	+	432166	432221
Aae-1-1816	NW_001811240.1	+	1708541	1708596
Aae-1-1817	NW_001810555.1	-	4193697	4193642
Aae-1-1818	NW_001811026.1	+	1262788	1262843
Aae-1-1819	NW_001811247.1	+	578609	578664
Aae-1-1820	NW_001811063.1	+	1590831	1590886
Aae-1-1821	NW_001811462.1	-	485228	485173
Aae-1-1822	NW_001811139.1	+	220985	221040
Aae-1-1823	NW_001811307.1	+	3057487	3057542
Aae-1-1824	NW_001811640.1	-	1918441	1918386
Aae-1-1825	NW_001810813.1	-	242692	242637
Aae-1-1826	NW_001811067.1	+	616243	616298
Aae-1-1827	NW_001811129.1	-	1847839	1847784
Aae-1-1828	NW_001811107.1	-	497555	497500
Aae-1-1829	NW_001810982.1	-	1042264	1042209
Aae-1-1830	NW_001809979.1	+	1099354	1099409
Aae-1-1831	NW_001809879.1	+	1734031	1734086
Aae-1-1832	NW_001811122.1	+	587754	587809
Aae-1-1833	NW_001811271.1	+	382256	382311
Aae-1-1834	NW_001811329.1	+	999454	999509
Aae-1-1835	NW_001811590.1	-	156389	156334
Aae-1-1836	NW_001809801.1	+	4368640	4368695
Aae-1-1837	NW_001809843.1	+	34583	34638
Aae-1-1838	NW_001810056.1	-	2204300	2204245
Aae-1-1839	NW_001810200.1	+	374094	374149
Aae-1-1840	NW_001810436.1	+	1288503	1288558
Aae-1-1841	NW_001810467.1	+	36856	36911
Aae-1-1842	NW_001811058.1	-	434569	434514
Aae-1-1843	NW_001811185.1	-	2362940	2362885
Aae-1-1844	NW_001811381.1	-	491105	491050
Aae-1-1845	NW_001809877.1	+	77948	78003
Aae-1-1846	NW_001810361.1	+	102847	102902
Aae-1-1847	NW_001810547.1	-	2218	2163
Aae-1-1848	NW_001810644.1	+	8089	8144
Aae-1-1849	NW_001810714.1	-	958060	958005
Aae-1-1850	NW_001810855.1	-	1447992	1447937
Aae-1-1851	NW_001810891.1	-	34249	34194
Aae-1-1852	NW_001810895.1	-	1320692	1320637
Aae-1-1853	NW_001810917.1	+	185592	185647
Aae-1-1854	NW_001811122.1	-	783884	783829

Aae-1-1855	NW_001811186.1	-	595597	595542
Aae-1-1856	NW_001811410.1	-	597310	597255
Aae-1-1857	NW_001811252.1	-	266355	266300
Aae-1-1858	NW_001811185.1	-	1777627	1777572
Aae-1-1859	NW_001811168.1	+	48856	48911
Aae-1-1860	NW_001811130.1	-	899292	899237
Aae-1-1861	NW_001810872.1	+	1688948	1689003
Aae-1-1862	NW_001810880.1	-	3809433	3809378
Aae-1-1863	NW_001810340.1	-	47064	47009
Aae-1-1864	NW_001810855.1	+	1427638	1427693
Aae-1-1865	NW_001811041.1	-	3502308	3502253
Aae-1-1866	NW_001810328.1	-	1524365	1524310
Aae-1-1867	NW_001810371.1	-	1902053	1901998
Aae-1-1868	NW_001810425.1	-	1045651	1045596
Aae-1-1869	NW_001810696.1	-	328788	328733
Aae-1-1870	NW_001810830.1	+	1256847	1256902
Aae-1-1871	NW_001810837.1	+	1568851	1568906
Aae-1-1872	NW_001810901.1	-	961996	961941
Aae-1-1873	NW_001810947.1	-	1487927	1487872
Aae-1-1874	NW_001810985.1	-	426035	425980
Aae-1-1875	NW_001810994.1	+	264860	264915
Aae-1-1876	NW_001810999.1	-	1104273	1104218
Aae-1-1877	NW_001811007.1	-	3365715	3365660
Aae-1-1878	NW_001811021.1	-	866714	866659
Aae-1-1879	NW_001811031.1	+	305946	306001
Aae-1-1880	NW_001811104.1	+	707406	707461
Aae-1-1881	NW_001811163.1	-	2065692	2065637
Aae-1-1882	NW_001811185.1	-	2394162	2394107
Aae-1-1883	NW_001811218.1	+	2318606	2318661
Aae-1-1884	NW_001811243.1	+	599854	599909
Aae-1-1885	NW_001811302.1	-	542842	542787
Aae-1-1886	NW_001811358.1	-	649852	649797
Aae-1-1887	NW_001811396.1	-	2631248	2631193
Aae-1-1888	NW_001811453.1	-	172438	172383
Aae-1-1889	NW_001811478.1	+	204025	204080
Aae-1-1890	NW_001811496.1	+	2720573	2720628
Aae-1-1891	NW_001811654.1	-	194484	194429
Aae-1-1892	NW_001811718.1	-	2516108	2516053
Aae-1-1893	NW_001811092.1	-	228814	228762
Aae-1-1894	NW_001811560.1	+	139404	139459
Aae-1-1895	NW_001810984.1	-	659257	659202
Aae-1-1896	NW_001810884.1	-	1581705	1581650
Aae-1-1897	NW_001811398.1	+	372521	372576
Aae-1-1898	NW_001811666.1	-	17560	17505
Aae-1-1899	NW_001810371.1	+	917698	917753
Aae-1-1900	NW_001810045.1	-	1803566	1803511
Aae-1-1901	NW_001809989.1	-	2114036	2113981
Aae-1-1902	NW_001810556.1	-	957197	957142
Aae-1-1903	NW_001810666.1	+	1804130	1804185
Aae-1-1904	NW_001811046.1	+	482630	482685
Aae-1-1905	NW_001811067.1	-	704484	704429
Aae-1-1906	NW_001811208.1	+	604848	604903

Aae-1-1907	NW_001811362.1	-	281058	281003
Aae-1-1908	NW_001811490.1	+	700	755
Aae-1-1909	NW_001811574.1	+	651262	651317
Aae-1-1910	NW_001811607.1	+	884626	884681
Aae-1-1911	NW_001810436.1	+	716839	716894
Aae-1-1912	NW_001811165.1	+	964573	964628
Aae-1-1913	NW_001810984.1	+	1433824	1433879
Aae-1-1914	NW_001810436.1	+	404058	404113
Aae-1-1915	NW_001810040.1	-	19536	19481
Aae-1-1916	NW_001809987.1	+	227491	227546
Aae-1-1917	NW_001810178.1	+	253187	253242
Aae-1-1918	NW_001810402.1	+	4018	4073
Aae-1-1919	NW_001810784.1	-	1635542	1635487
Aae-1-1920	NW_001810805.1	+	131624	131679
Aae-1-1921	NW_001810976.1	+	876539	876594
Aae-1-1922	NW_001811040.1	-	1065581	1065526
Aae-1-1923	NW_001811150.1	-	830445	830390
Aae-1-1924	NW_001811263.1	-	2603088	2603033
Aae-1-1925	NW_001811274.1	+	473424	473479
Aae-1-1926	NW_001811521.1	-	136542	136487
Aae-1-1927	NW_001811695.1	-	79735	79680
Aae-1-1928	NW_001811786.1	-	69307	69252
Aae-1-1929	NW_001809905.1	+	182228	182283
Aae-1-1930	NW_001811696.1	+	1475337	1475392
Aae-1-1931	NW_001809802.1	-	184177	184122
Aae-1-1932	NW_001810782.1	+	522327	522382
Aae-1-1933	NW_001811023.1	-	672258	672203
Aae-1-1934	NW_001811111.1	-	978946	978891
Aae-1-1935	NW_001811174.1	-	2067712	2067657
Aae-1-1936	NW_001811307.1	+	1275306	1275361
Aae-1-1937	NW_001809836.1	-	2120783	2120728
Aae-1-1938	NW_001811191.1	-	767630	767575
Aae-1-1939	NW_001810339.1	+	2017175	2017230
Aae-1-1940	NW_001810200.1	-	513135	513080
Aae-1-1941	NW_001810381.1	-	980112	980057
Aae-1-1942	NW_001810813.1	+	1375093	1375148
Aae-1-1943	NW_001810830.1	+	679904	679959
Aae-1-1944	NW_001810994.1	-	1512832	1512777
Aae-1-1945	NW_001811362.1	+	332577	332632
Aae-1-1946	NW_001811367.1	+	90204	90259
Aae-1-1947	NW_001811295.1	+	675765	675820
Aae-1-1948	NW_001811529.1	+	691368	691423
Aae-1-1949	NW_001811546.1	+	216153	216208
Aae-1-1950	NW_001811629.1	+	967524	967579
Aae-1-1951	NW_001811668.1	+	383926	383981
Aae-1-1952	NW_001811634.1	-	300809	300754
Aae-1-1953	NW_001811351.1	+	1597518	1597573
Aae-1-1954	NW_001811396.1	+	1232355	1232410
Aae-1-1955	NW_001811562.1	+	2290408	2290463
Aae-1-1956	NW_001811501.1	+	69303	69358
Aae-1-1957	NW_001811487.1	+	19819	19874
Aae-1-1958	NW_001811530.1	-	247402	247347

Aae-1-1959	NW_001811482.1	-	502624	502569
Aae-1-1960	NW_001811789.1	+	162542	162597
Aae-1-1961	NW_001811479.1	-	72866	72811
Aae-1-1962	NW_001811477.1	-	388231	388176
Aae-1-1963	NW_001811448.1	+	233527	233582
Aae-1-1964	NW_001811409.1	-	100078	100023
Aae-1-1965	NW_001811396.1	+	2218183	2218238
Aae-1-1966	NW_001811363.1	+	890484	890539
Aae-1-1967	NW_001811361.1	+	530263	530318
Aae-1-1968	NW_001811341.1	-	198770	198715
Aae-1-1969	NW_001811307.1	-	2348603	2348548
Aae-1-1970	NW_001811288.1	+	145838	145893
Aae-1-1971	NW_001811274.1	+	2205677	2205732
Aae-1-1972	NW_001811263.1	+	1388564	1388619
Aae-1-1973	NW_001811183.1	-	740434	740379
Aae-1-1974	NW_001811095.1	-	409054	408999
Aae-1-1975	NW_001811159.1	+	370432	370487
Aae-1-1976	NW_001809858.1	-	132618	132563
Aae-1-1977	NW_001810566.1	+	49749	49804
Aae-1-1978	NW_001811151.1	-	949192	949137
Aae-1-1979	NW_001837393.1	+	14151	14206
Aae-1-1980	NW_001810034.1	+	2277849	2277900
Aae-1-1981	NW_001811027.1	-	480351	480296
Aae-1-1982	NW_001811062.1	-	517920	517865
Aae-1-1983	NW_001810214.1	+	45309	45364
Aae-1-1984	NW_001810919.1	-	1684625	1684570
Aae-1-1985	NW_001811204.1	+	88451	88506
Aae-1-1986	NW_001810102.1	-	162546	162491
Aae-1-1987	NW_001809946.1	+	98371	98426
Aae-1-1988	NW_001809957.1	-	1368092	1368037
Aae-1-1989	NW_001810938.1	+	1173034	1173089
Aae-1-1990	NW_001810996.1	+	2509484	2509539
Aae-1-1991	NW_001811072.1	+	982793	982848
Aae-1-1992	NW_001811076.1	-	627958	627903
Aae-1-1993	NW_001810022.1	-	2913496	2913441
Aae-1-1994	NW_001811238.1	+	576079	576134
Aae-1-1995	NW_001811024.1	+	168770	168825
Aae-1-1996	NW_001811123.1	-	155484	155429
Aae-1-1997	NW_001811196.1	+	137462	137517
Aae-1-1998	NW_001811329.1	+	1174905	1174960
Aae-1-1999	NW_001811026.1	+	1102497	1102552
Aae-1-2000	NW_001811007.1	+	862993	863048
Aae-1-2001	NW_001810955.1	+	1012664	1012719
Aae-1-2002	NW_001810167.1	+	1563757	1563812
Aae-1-2003	NW_001810681.1	+	495353	495408
Aae-1-2004	NW_001810912.1	+	3558702	3558757
Aae-1-2005	NW_001810963.1	+	243452	243507
Aae-1-2006	NW_001811067.1	+	497467	497522
Aae-1-2007	NW_001811142.1	-	948192	948137
Aae-1-2008	NW_001811166.1	-	564924	564869
Aae-1-2009	NW_001811229.1	-	1243869	1243814
Aae-1-2010	NW_001809957.1	-	913178	913123

Aae-1-2011	NW_001811450.1	-	303981	303926
Aae-1-2012	NW_001810156.1	-	111182	111127
Aae-1-2013	NW_001810537.1	-	1368290	1368235
Aae-1-2014	NW_001811104.1	-	421083	421028
Aae-1-2015	NW_001810584.1	+	1708726	1708781
Aae-1-2016	NW_001810825.1	-	586970	586915
Aae-1-2017	NW_001810133.1	+	910964	911019
Aae-1-2018	NW_001810178.1	-	1785272	1785217
Aae-1-2019	NW_001810599.1	+	925950	926005
Aae-1-2020	NW_001811043.1	+	852104	852159
Aae-1-2021	NW_001811054.1	-	212515	212460
Aae-1-2022	NW_001811140.1	-	323172	323117
Aae-1-2023	NW_001811141.1	-	292596	292541
Aae-1-2024	NW_001811183.1	-	543074	543019
Aae-1-2025	NW_001811368.1	+	359864	359919
Aae-1-2026	NW_001811426.1	+	139630	139685
Aae-1-2027	NW_001811507.1	-	1078853	1078798
Aae-1-2028	NW_001810242.1	-	2251301	2251246
Aae-1-2029	NW_001810575.1	+	1588515	1588570
Aae-1-2030	NW_001810750.1	-	1588941	1588886
Aae-1-2031	NW_001810805.1	-	746993	746938
Aae-1-2032	NW_001811144.1	-	463467	463412
Aae-1-2033	NW_001811119.1	+	437813	437868
Aae-1-2034	NW_001811118.1	-	1463218	1463163
Aae-1-2035	NW_001811105.1	+	900621	900676
Aae-1-2036	NW_001811063.1	-	374506	374451
Aae-1-2037	NW_001811052.1	-	756185	756130
Aae-1-2038	NW_001810909.1	-	1725499	1725444
Aae-1-2039	NW_001811229.1	+	2437061	2437116
Aae-1-2040	NW_001810592.1	-	27708	27653
Aae-1-2041	NW_001811183.1	-	447491	447436
Aae-1-2042	NW_001811021.1	+	982873	982930
Aae-1-2043	NW_001810214.1	-	75110	75055
Aae-1-2044	NW_001811252.1	+	981811	981866
Aae-1-2045	NW_001810982.1	+	1282757	1282812
Aae-1-2046	NW_001810275.1	+	853377	853432
Aae-1-2047	NW_001810480.1	-	1274283	1274228
Aae-1-2048	NW_001811007.1	-	2151805	2151750
Aae-1-2049	NW_001811138.1	+	932495	932550
Aae-1-2050	NW_001811155.1	+	280687	280742
Aae-1-2051	NW_001811192.1	-	783280	783225
Aae-1-2052	NW_001811195.1	-	833378	833323
Aae-1-2053	NW_001811267.1	+	138045	138100
Aae-1-2054	NW_001836061.1	-	5756	5705
Aae-1-2055	NW_001811382.1	-	495041	494986
Aae-1-2056	NW_001810923.1	-	523855	523800
Aae-1-2057	NW_001811207.1	+	2621864	2621919
Aae-1-2058	NW_001811634.1	+	56690	56745
Aae-1-2059	NW_001809876.1	-	15432	15377
Aae-1-2060	NW_001810637.1	-	2434298	2434243
Aae-1-2061	NW_001810728.1	+	382051	382106
Aae-1-2062	NW_001810954.1	+	611363	611418

Aae-1-2063	NW_001810955.1	+	3682695	3682750
Aae-1-2064	NW_001810955.1	-	1803910	1803855
Aae-1-2065	NW_001811018.1	-	1853195	1853140
Aae-1-2066	NW_001811065.1	-	34873	34818
Aae-1-2067	NW_001810555.1	+	3747770	3747825
Aae-1-2068	NW_001810629.1	-	128578	128523
Aae-1-2069	NW_001810637.1	-	3464414	3464359
Aae-1-2070	NW_001810763.1	-	802634	802579
Aae-1-2071	NW_001810935.1	-	1479382	1479327
Aae-1-2072	NW_001811293.1	-	271524	271469
Aae-1-2073	NW_001811640.1	+	800524	800579
Aae-1-2074	NW_001811767.1	+	362988	363043
Aae-1-2075	NW_001810555.1	+	3598767	3598822
Aae-1-2076	NW_001811130.1	-	2756910	2756855
Aae-1-2077	NW_001810858.1	+	1484100	1484155
Aae-1-2078	NW_001810681.1	-	1170845	1170790
Aae-1-2079	NW_001811013.1	+	1061721	1061776
Aae-1-2080	NW_001810791.1	-	1200234	1200179
Aae-1-2081	NW_001810328.1	+	1486333	1486388
Aae-1-2082	NW_001810296.1	+	553826	553881
Aae-1-2083	NW_001810200.1	-	1274693	1274638
Aae-1-2084	NW_001809802.1	+	302067	302122
Aae-1-2085	NW_001809879.1	+	1916137	1916192
Aae-1-2086	NW_001810458.1	-	2652134	2652079
Aae-1-2087	NW_001810913.1	-	1543873	1543818
Aae-1-2088	NW_001810945.1	+	1399019	1399074
Aae-1-2089	NW_001810959.1	+	846064	846119
Aae-1-2090	NW_001811007.1	+	3497819	3497874
Aae-1-2091	NW_001811011.1	-	1196001	1195946
Aae-1-2092	NW_001811130.1	-	2063451	2063396
Aae-1-2093	NW_001811174.1	+	2280956	2281011
Aae-1-2094	NW_001811189.1	-	446095	446040
Aae-1-2095	NW_001811340.1	+	962898	962953
Aae-1-2096	NW_001811363.1	+	149063	149118
Aae-1-2097	NW_001811363.1	+	1165664	1165719
Aae-1-2098	NW_001811693.1	-	247848	247793
Aae-1-2099	NW_001810027.1	-	99396	99341
Aae-1-2100	NW_001809845.1	+	255183	255238
Aae-1-2101	NW_001810181.1	-	53566	53511
Aae-1-2102	NW_001810993.1	+	1255006	1255061
Aae-1-2103	NW_001809956.1	+	23212	23267
Aae-1-2104	NW_001809956.1	+	27312	27367
Aae-1-2105	NW_001810022.1	-	411622	411567
Aae-1-2106	NW_001810081.1	-	138692	138637
Aae-1-2107	NW_001810167.1	-	1563743	1563688
Aae-1-2108	NW_001810784.1	+	170625	170680
Aae-1-2109	NW_001810868.1	+	358093	358148
Aae-1-2110	NW_001810880.1	+	1560296	1560351
Aae-1-2111	NW_001811057.1	+	116931	116986
Aae-1-2112	NW_001811060.1	-	375986	375931
Aae-1-2113	NW_001811066.1	+	51181	51236
Aae-1-2114	NW_001811081.1	-	1028337	1028282

Aae-1-2115	NW_001811085.1	+	1315490	1315545
Aae-1-2116	NW_001811087.1	+	699530	699585
Aae-1-2117	NW_001811139.1	+	943795	943850
Aae-1-2118	NW_001811141.1	+	630350	630405
Aae-1-2119	NW_001811178.1	+	539017	539072
Aae-1-2120	NW_001811219.1	-	64031	63976
Aae-1-2121	NW_001811220.1	+	319374	319429
Aae-1-2122	NW_001811223.1	+	419198	419253
Aae-1-2123	NW_001811244.1	+	688683	688738
Aae-1-2124	NW_001811354.1	+	270277	270332
Aae-1-2125	NW_001811600.1	+	85346	85401
Aae-1-2126	NW_001811611.1	-	264396	264341
Aae-1-2127	NW_001811793.1	+	290836	290891
Aae-1-2128	NW_001810034.1	+	1601118	1601173
Aae-1-2129	NW_001810681.1	+	1865417	1865472
Aae-1-2130	NW_001810805.1	+	286646	286701
Aae-1-2131	NW_001810868.1	-	1488019	1487964
Aae-1-2132	NW_001811005.1	-	1115980	1115925
Aae-1-2133	NW_001811131.1	+	7	62
Aae-1-2134	NW_001811301.1	-	6196	6141
Aae-1-2135	NW_001811462.1	+	3233418	3233473
Aae-1-2136	NW_001811626.1	-	134135	134080
Aae-1-2137	NW_001809946.1	+	2305144	2305199
Aae-1-2138	NW_001809902.1	-	43097	43042
Aae-1-2139	NW_001810151.1	-	53735	53680
Aae-1-2140	NW_001811136.1	+	1009907	1009962
Aae-1-2141	NW_001810342.1	-	53419	53364
Aae-1-2142	NW_001811412.1	-	177359	177304
Aae-1-2143	NW_001810637.1	-	208566	208511
Aae-1-2144	NW_001811252.1	+	2878279	2878334
Aae-1-2145	NW_001810913.1	+	1542531	1542586
Aae-1-2146	NW_001810584.1	+	168363	168418
Aae-1-2147	NW_001811098.1	+	541248	541303
Aae-1-2148	NW_001810805.1	-	771924	771869
Aae-1-2149	NW_001810970.1	-	3812047	3811992
Aae-1-2150	NW_001811480.1	+	367097	367152
Aae-1-2151	NW_001811540.1	+	2249591	2249646
Aae-1-2152	NW_001810929.1	-	570532	570477
Aae-1-2153	NW_001810045.1	+	1349631	1349686
Aae-1-2154	NW_001810350.1	-	810416	810361
Aae-1-2155	NW_001811048.1	-	399946	399891
Aae-1-2156	NW_001811277.1	-	163591	163536
Aae-1-2157	NW_001811333.1	+	100255	100310
Aae-1-2158	NW_001810231.1	-	970875	970820
Aae-1-2159	NW_001810986.1	+	1018748	1018803
Aae-1-2160	NW_001811024.1	+	1045829	1045884
Aae-1-2161	NW_001811211.1	+	861268	861323
Aae-1-2162	NW_001811739.1	-	1454636	1454581
Aae-1-2163	NW_001810328.1	+	1046341	1046396
Aae-1-2164	NW_001810508.1	+	81527	81582
Aae-1-2165	NW_001810715.1	+	979436	979491
Aae-1-2166	NW_001810721.1	-	191160	191105

Aae-1-2167	NW_001811031.1	-	1300569	1300514
Aae-1-2168	NW_001811086.1	+	865083	865138
Aae-1-2169	NW_001811224.1	+	515664	515719
Aae-1-2170	NW_001810555.1	+	4152317	4152372
Aae-1-2171	NW_001810167.1	+	2276779	2276834
Aae-1-2172	NW_001811761.1	-	916575	916520
Aae-1-2173	NW_001810490.1	+	933773	933828
Aae-1-2174	NW_001810744.1	-	836195	836140
Aae-1-2175	NW_001810837.1	-	646702	646647
Aae-1-2176	NW_001811043.1	+	795961	796016
Aae-1-2177	NW_001811119.1	+	761133	761188
Aae-1-2178	NW_001811187.1	-	518133	518078
Aae-1-2179	NW_001811673.1	+	1250653	1250708
Aae-1-2180	NW_001810242.1	+	1116001	1116056
Aae-1-2181	NW_001810575.1	-	1758747	1758692
Aae-1-2182	NW_001811635.1	-	234998	234943
Aae-1-2183	NW_001810947.1	-	267946	267891
Aae-1-2184	NW_001810769.1	-	652618	652563
Aae-1-2185	NW_001811351.1	-	1924354	1924299
Aae-1-2186	NW_001811188.1	-	106872	106817
Aae-1-2187	NW_001811284.1	-	452717	452662
Aae-1-2188	NW_001811071.1	-	496880	496825
Aae-1-2189	NW_001811233.1	-	834205	834150
Aae-1-2190	NW_001811252.1	+	1662467	1662522
Aae-1-2191	NW_001811141.1	-	774700	774645
Aae-1-2192	NW_001811020.1	-	1165765	1165710
Aae-1-2193	NW_001811010.1	+	736904	736959
Aae-1-2194	NW_001810964.1	-	765848	765793
Aae-1-2195	NW_001810955.1	+	1556727	1556782
Aae-1-2196	NW_001810035.1	+	47491	47546
Aae-1-2197	NW_001810167.1	+	1404192	1404247
Aae-1-2198	NW_001810439.1	-	29739	29684
Aae-1-2199	NW_001811074.1	-	305384	305329
Aae-1-2200	NW_001811293.1	+	178531	178586
Aae-1-2201	NW_001811376.1	-	608072	608017
Aae-1-2202	NW_001835922.1	+	3867	3922
Aae-1-2203	NW_001810979.1	+	224373	224428
Aae-1-2204	NW_001810756.1	-	936662	936607
Aae-1-2205	NW_001810221.1	-	1392863	1392808
Aae-1-2206	NW_001811010.1	+	945890	945945
Aae-1-2207	NW_001811296.1	+	2023293	2023348
Aae-1-2208	NW_001811724.1	+	156558	156613
Aae-1-2209	NW_001811046.1	+	1248195	1248250
Aae-1-2210	NW_001810250.1	-	52317	52262
Aae-1-2211	NW_001810067.1	-	1000152	1000097
Aae-1-2212	NW_001810469.1	+	995910	995965
Aae-1-2213	NW_001811052.1	-	1017965	1017910
Aae-1-2214	NW_001810023.1	+	1844097	1844152
Aae-1-2215	NW_001809924.1	-	2221116	2221061
Aae-1-2216	NW_001811050.1	-	1028008	1027953
Aae-1-2217	NW_001811242.1	+	633779	633834
Aae-1-2218	NW_001810898.1	-	253043	252988

Aae-1-2219	NW_001811081.1	-	102382	102327
Aae-1-2220	NW_001811215.1	-	298319	298264
Aae-1-2221	NW_001811364.1	-	548222	548167
Aae-1-2222	NW_001810425.1	-	1196755	1196700
Aae-1-2223	NW_001811044.1	-	184481	184426
Aae-1-2224	NW_001811264.1	+	202720	202775
Aae-1-2225	NW_001811342.1	+	661314	661369
Aae-1-2226	NW_001810880.1	-	3009044	3008989
Aae-1-2227	NW_001811068.1	-	597752	597697
Aae-1-2228	NW_001811085.1	-	91290	91239
Aae-1-2229	NW_001811462.1	+	277503	277554
Aae-1-2230	NW_001811141.1	+	1407254	1407309
Aae-1-2231	NW_001811252.1	+	2114136	2114191
Aae-1-2232	NW_001811399.1	-	62720	62665
Aae-1-2233	NW_001811329.1	-	1691238	1691183
Aae-1-2234	NW_001811235.1	-	193712	193657
Aae-1-2235	NW_001811233.1	-	679412	679357
Aae-1-2236	NW_001811218.1	-	2585698	2585643
Aae-1-2237	NW_001811109.1	+	689808	689863
Aae-1-2238	NW_001811096.1	+	1875745	1875800
Aae-1-2239	NW_001811010.1	-	1213198	1213143
Aae-1-2240	NW_001811191.1	-	659146	659091
Aae-1-2241	NW_001810984.1	+	1274466	1274521
Aae-1-2242	NW_001810955.1	+	2573524	2573579
Aae-1-2243	NW_001810805.1	-	302429	302374
Aae-1-2244	NW_001810118.1	+	130278	130333
Aae-1-2245	NW_001810375.1	-	44037	43982
Aae-1-2246	NW_001810947.1	+	279841	279896
Aae-1-2247	NW_001811022.1	+	16058	16113
Aae-1-2248	NW_001811085.1	-	1312774	1312719
Aae-1-2249	NW_001811248.1	+	205664	205719
Aae-1-2250	NW_001811252.1	+	144532	144587
Aae-1-2251	NW_001811369.1	+	373992	374047
Aae-1-2252	NW_001809830.1	+	116865	116920
Aae-1-2253	NW_001809913.1	-	229865	229810
Aae-1-2254	NW_001809935.1	+	2078364	2078419
Aae-1-2255	NW_001809955.1	-	219932	219877
Aae-1-2256	NW_001810022.1	-	3764864	3764809
Aae-1-2257	NW_001810339.1	+	1394204	1394259
Aae-1-2258	NW_001810637.1	-	2245566	2245511
Aae-1-2259	NW_001810791.1	+	878840	878895
Aae-1-2260	NW_001810830.1	-	62070	62015
Aae-1-2261	NW_001811063.1	-	3570115	3570060
Aae-1-2262	NW_001811114.1	-	27760	27705
Aae-1-2263	NW_001811196.1	+	1302552	1302607
Aae-1-2264	NW_001811209.1	+	428332	428387
Aae-1-2265	NW_001811226.1	-	495979	495924
Aae-1-2266	NW_001811327.1	+	291934	291989
Aae-1-2267	NW_001811607.1	+	1270293	1270348
Aae-1-2268	NW_001811772.1	+	2237735	2237790
Aae-1-2269	NW_001836155.1	-	3805	3750
Aae-1-2270	NW_001809968.1	-	2339913	2339858

Aae-1-2271	NW_001811385.1	-	1101709	1101654
Aae-1-2272	NW_001810133.1	-	1191725	1191670
Aae-1-2273	NW_001810556.1	-	1529321	1529266
Aae-1-2274	NW_001810975.1	+	883283	883338
Aae-1-2275	NW_001811363.1	-	1165395	1165340
Aae-1-2276	NW_001811385.1	+	1534876	1534931
Aae-1-2277	NW_001811351.1	-	1913607	1913552
Aae-1-2278	NW_001811341.1	-	419308	419253
Aae-1-2279	NW_001811326.1	+	171232	171287
Aae-1-2280	NW_001811105.1	-	96822	96767
Aae-1-2281	NW_001811037.1	-	73702	73647
Aae-1-2282	NW_001811032.1	+	920940	920995
Aae-1-2283	NW_001811618.1	-	856996	856941
Aae-1-2284	NW_001810939.1	+	769960	770015
Aae-1-2285	NW_001811007.1	-	2797670	2797615
Aae-1-2286	NW_001811016.1	+	35998	36053
Aae-1-2287	NW_001811003.1	+	137105	137160
Aae-1-2288	NW_001810962.1	-	975359	975304
Aae-1-2289	NW_001811050.1	+	1110733	1110788
Aae-1-2290	NW_001811585.1	-	2641622	2641567
Aae-1-2291	NW_001811718.1	+	2147201	2147256
Aae-1-2292	NW_001811061.1	+	1008190	1008245
Aae-1-2293	NW_001810133.1	-	291300	291245
Aae-1-2294	NW_001810984.1	-	3466609	3466554
Aae-1-2295	NW_001810045.1	-	2032008	2031953
Aae-1-2296	NW_001810458.1	+	3029584	3029639
Aae-1-2297	NW_001810696.1	+	949258	949313
Aae-1-2298	NW_001810852.1	-	1325276	1325221
Aae-1-2299	NW_001811047.1	+	1052901	1052956
Aae-1-2300	NW_001810575.1	-	1361234	1361179
Aae-1-2301	NW_001810783.1	-	591999	591944
Aae-1-2302	NW_001810962.1	+	635976	636031
Aae-1-2303	NW_001811215.1	+	749711	749766
Aae-1-2304	NW_001811369.1	+	178365	178420
Aae-1-2305	NW_001810666.1	+	1447961	1448016
Aae-1-2306	NW_001809957.1	-	910361	910306
Aae-1-2307	NW_001810937.1	+	3702332	3702387
Aae-1-2308	NW_001811163.1	+	214112	214167
Aae-1-2309	NW_001809893.1	-	148866	148811
Aae-1-2310	NW_001810763.1	-	849863	849808
Aae-1-2311	NW_001810955.1	-	2374111	2374056
Aae-1-2312	NW_001809823.1	+	172749	172804
Aae-1-2313	NW_001810169.1	-	36051	35996
Aae-1-2314	NW_001811318.1	-	1551507	1551452
Aae-1-2315	NW_001811528.1	+	319544	319599
Aae-1-2316	NW_001809802.1	+	1078139	1078194
Aae-1-2317	NW_001809805.1	-	188567	188512
Aae-1-2318	NW_001809836.1	+	1420798	1420853
Aae-1-2319	NW_001809847.1	-	5191	5136
Aae-1-2320	NW_001809873.1	-	255784	255729
Aae-1-2321	NW_001809968.1	-	908220	908165
Aae-1-2322	NW_001810005.1	-	137870	137815

Aae-1-2323	NW_001810022.1	-	2479749	2479694
Aae-1-2324	NW_001810111.1	+	1567606	1567661
Aae-1-2325	NW_001810133.1	-	1292256	1292201
Aae-1-2326	NW_001810189.1	-	2230992	2230937
Aae-1-2327	NW_001810207.1	-	96091	96036
Aae-1-2328	NW_001810231.1	-	798997	798942
Aae-1-2329	NW_001810351.1	+	1563356	1563411
Aae-1-2330	NW_001810351.1	+	2020170	2020225
Aae-1-2331	NW_001810356.1	+	31298	31353
Aae-1-2332	NW_001810371.1	+	535946	536001
Aae-1-2333	NW_001810371.1	+	1672347	1672402
Aae-1-2334	NW_001810371.1	-	535287	535232
Aae-1-2335	NW_001810403.1	-	743762	743707
Aae-1-2336	NW_001810556.1	-	38945	38890
Aae-1-2337	NW_001810686.1	+	9064	9119
Aae-1-2338	NW_001810728.1	+	644615	644670
Aae-1-2339	NW_001810728.1	+	1679998	1680053
Aae-1-2340	NW_001810782.1	+	3552808	3552863
Aae-1-2341	NW_001810791.1	+	804279	804334
Aae-1-2342	NW_001810868.1	-	564928	564873
Aae-1-2343	NW_001810912.1	+	3044508	3044563
Aae-1-2344	NW_001810916.1	+	977904	977959
Aae-1-2345	NW_001810935.1	+	225923	225978
Aae-1-2346	NW_001810955.1	+	2362763	2362818
Aae-1-2347	NW_001810966.1	+	1205420	1205475
Aae-1-2348	NW_001810975.1	+	529540	529595
Aae-1-2349	NW_001810996.1	-	1411846	1411791
Aae-1-2350	NW_001811003.1	+	1214658	1214713
Aae-1-2351	NW_001811005.1	-	129414	129359
Aae-1-2352	NW_001811012.1	-	1388544	1388489
Aae-1-2353	NW_001811031.1	+	216975	217030
Aae-1-2354	NW_001811031.1	+	868924	868979
Aae-1-2355	NW_001811049.1	+	956455	956510
Aae-1-2356	NW_001811061.1	+	582599	582654
Aae-1-2357	NW_001811086.1	+	234929	234984
Aae-1-2358	NW_001811086.1	+	947654	947709
Aae-1-2359	NW_001811089.1	+	969892	969947
Aae-1-2360	NW_001811099.1	-	115098	115043
Aae-1-2361	NW_001811119.1	-	142597	142542
Aae-1-2362	NW_001811136.1	+	234103	234158
Aae-1-2363	NW_001811141.1	-	2195648	2195593
Aae-1-2364	NW_001811152.1	-	3084168	3084113
Aae-1-2365	NW_001811157.1	-	572375	572320
Aae-1-2366	NW_001811162.1	+	324751	324806
Aae-1-2367	NW_001811184.1	-	554635	554580
Aae-1-2368	NW_001811185.1	+	1844414	1844469
Aae-1-2369	NW_001811185.1	-	1843085	1843030
Aae-1-2370	NW_001811187.1	+	660707	660762
Aae-1-2371	NW_001811196.1	+	1883407	1883462
Aae-1-2372	NW_001811218.1	+	661114	661169
Aae-1-2373	NW_001811238.1	-	549945	549890
Aae-1-2374	NW_001811252.1	+	2934896	2934951

Aae-1-2375	NW_001811272.1	+	731384	731439
Aae-1-2376	NW_001811326.1	-	18645	18590
Aae-1-2377	NW_001811351.1	+	1913637	1913692
Aae-1-2378	NW_001811379.1	-	217028	216973
Aae-1-2379	NW_001811385.1	+	928875	928930
Aae-1-2380	NW_001811418.1	+	2909266	2909321
Aae-1-2381	NW_001811443.1	-	248464	248409
Aae-1-2382	NW_001811462.1	-	596685	596630
Aae-1-2383	NW_001811463.1	-	610484	610429
Aae-1-2384	NW_001811488.1	+	378420	378475
Aae-1-2385	NW_001811546.1	+	357305	357360
Aae-1-2386	NW_001811579.1	+	347336	347391
Aae-1-2387	NW_001811598.1	-	185808	185753
Aae-1-2388	NW_001811609.1	+	252695	252750
Aae-1-2389	NW_001811629.1	+	1883695	1883750
Aae-1-2390	NW_001811647.1	-	416643	416588
Aae-1-2391	NW_001811686.1	+	287582	287637
Aae-1-2392	NW_001811692.1	+	137151	137206
Aae-1-2393	NW_001811743.1	-	261104	261049
Aae-1-2394	NW_001836439.1	+	2190	2245
Aae-1-2395	NW_001837394.1	-	742	687
Aae-1-2396	NW_001809803.1	-	1639233	1639178
Aae-1-2397	NW_001811311.1	-	762672	762617
Aae-1-2398	NW_001811406.1	-	368039	367984
Aae-1-2399	NW_001811483.1	+	337265	337320
Aae-1-2400	NW_001811130.1	-	578537	578482
Aae-1-2401	NW_001811618.1	-	1351207	1351152
Aae-1-2402	NW_001811662.1	-	1629973	1629918
Aae-1-2403	NW_001811714.1	+	302365	302420
Aae-1-2404	NW_001811789.1	-	257040	256985
Aae-1-2405	NW_001811757.1	+	129715	129770
Aae-1-2406	NW_001811684.1	+	4225219	4225274
Aae-1-2407	NW_001811662.1	-	2444405	2444350
Aae-1-2408	NW_001811640.1	+	1241412	1241467
Aae-1-2409	NW_001811618.1	+	780452	780507
Aae-1-2410	NW_001811562.1	-	2244471	2244416
Aae-1-2411	NW_001811513.1	-	4114	4059
Aae-1-2412	NW_001811462.1	-	2659242	2659187
Aae-1-2413	NW_001811418.1	-	2570172	2570117
Aae-1-2414	NW_001811507.1	-	1280595	1280540
Aae-1-2415	NW_001811454.1	+	595403	595458
Aae-1-2416	NW_001811398.1	-	353101	353046
Aae-1-2417	NW_001811209.1	+	377187	377242
Aae-1-2418	NW_001811238.1	-	876160	876105
Aae-1-2419	NW_001811344.1	+	359220	359275
Aae-1-2420	NW_001811314.1	-	537241	537186
Aae-1-2421	NW_001811307.1	+	3045855	3045910
Aae-1-2422	NW_001811274.1	+	595852	595907
Aae-1-2423	NW_001811263.1	+	2519555	2519610
Aae-1-2424	NW_001811433.1	+	54667	54722
Aae-1-2425	NW_001811258.1	+	275744	275799
Aae-1-2426	NW_001811258.1	+	285980	286035

Aae-1-2427	NW_001811003.1	-	1239908	1239853
Aae-1-2428	NW_001811191.1	-	637850	637795
Aae-1-2429	NW_001811225.1	+	364131	364186
Aae-1-2430	NW_001811246.1	-	181604	181549
Aae-1-2431	NW_001811242.1	+	308600	308655
Aae-1-2432	NW_001811269.1	+	478560	478615
Aae-1-2433	NW_001811690.1	-	204511	204456
Aae-1-2434	NW_001811207.1	-	2865612	2865557
Aae-1-2435	NW_001811393.1	+	76045	76100
Aae-1-2436	NW_001811236.1	+	193602	193657
Aae-1-2437	NW_001811225.1	+	436003	436058
Aae-1-2438	NW_001811189.1	-	821966	821911
Aae-1-2439	NW_001811185.1	-	1761388	1761333
Aae-1-2440	NW_001811142.1	-	958012	957957
Aae-1-2441	NW_001811067.1	-	123636	123581
Aae-1-2442	NW_001811032.1	-	126068	126013
Aae-1-2443	NW_001810221.1	+	1431823	1431878
Aae-1-2444	NW_001810556.1	+	452091	452146
Aae-1-2445	NW_001810929.1	-	1391284	1391229
Aae-1-2446	NW_001811118.1	+	164977	165032
Aae-1-2447	NW_001811351.1	-	2171380	2171325
Aae-1-2448	NW_001810242.1	-	1151540	1151485
Aae-1-2449	NW_001811233.1	-	110097	110042
Aae-1-2450	NW_001811240.1	+	465589	465644
Aae-1-2451	NW_001811060.1	+	828114	828169
Aae-1-2452	NW_001811050.1	+	732564	732619
Aae-1-2453	NW_001810000.1	-	1017071	1017016
Aae-1-2454	NW_001811626.1	-	467121	467066
Aae-1-2455	NW_001810133.1	+	3647222	3647277
Aae-1-2456	NW_001810584.1	+	966538	966593
Aae-1-2457	NW_001810490.1	+	193075	193130
Aae-1-2458	NW_001811023.1	-	616102	616047
Aae-1-2459	NW_001811041.1	-	1814759	1814704
Aae-1-2460	NW_001811551.1	+	671613	671668
Aae-1-2461	NW_001809879.1	+	1866311	1866366
Aae-1-2462	NW_001809879.1	-	2315615	2315560
Aae-1-2463	NW_001809979.1	-	2433635	2433580
Aae-1-2464	NW_001810264.1	+	1226349	1226404
Aae-1-2465	NW_001810480.1	+	242679	242734
Aae-1-2466	NW_001810566.1	+	42	97
Aae-1-2467	NW_001810959.1	+	655565	655620
Aae-1-2468	NW_001811014.1	+	324285	324340
Aae-1-2469	NW_001811059.1	+	641916	641971
Aae-1-2470	NW_001811071.1	-	985439	985384
Aae-1-2471	NW_001811089.1	+	681818	681873
Aae-1-2472	NW_001811127.1	+	313591	313646
Aae-1-2473	NW_001811218.1	-	1238340	1238285
Aae-1-2474	NW_001811366.1	+	10897	10952
Aae-1-2475	NW_001811518.1	-	1736023	1735968
Aae-1-2476	NW_001811551.1	+	2758887	2758942
Aae-1-2477	NW_001810111.1	-	1196543	1196484
Aae-1-2478	NW_001810022.1	+	3500167	3500222

Aae-1-2479	NW_001810962.1	+	870173	870228
Aae-1-2480	NW_001811290.1	+	750577	750632
Aae-1-2481	NW_001810458.1	-	4190054	4189999
Aae-1-2482	NW_001810714.1	-	2180329	2180274
Aae-1-2483	NW_001810989.1	+	206472	206527
Aae-1-2484	NW_001810998.1	+	60315	60370
Aae-1-2485	NW_001811201.1	-	639023	638968
Aae-1-2486	NW_001811242.1	+	467263	467318
Aae-1-2487	NW_001811282.1	+	495578	495633
Aae-1-2488	NW_001811316.1	+	258150	258205
Aae-1-2489	NW_001811336.1	+	772665	772720
Aae-1-2490	NW_001811351.1	-	2734175	2734120
Aae-1-2491	NW_001811352.1	+	1994875	1994930
Aae-1-2492	NW_001811377.1	-	518708	518653
Aae-1-2493	NW_001811462.1	-	3158218	3158163
Aae-1-2494	NW_001811684.1	+	2357828	2357883
Aae-1-2495	NW_001836144.1	-	2211	2156
Aae-1-2496	NW_001810629.1	+	1782510	1782565
Aae-1-2497	NW_001810744.1	-	772582	772527
Aae-1-2498	NW_001810888.1	-	1445392	1445337
Aae-1-2499	NW_001811091.1	-	342786	342731
Aae-1-2500	NW_001810913.1	-	310433	310378
Aae-1-2501	NW_001811169.1	-	448395	448340
Aae-1-2502	NW_001810888.1	+	839581	839636
Aae-1-2503	NW_001810838.1	-	779648	779593
Aae-1-2504	NW_001810838.1	+	1218489	1218544
Aae-1-2505	NW_001811237.1	+	350683	350738
Aae-1-2506	NW_001810519.1	-	1726080	1726025
Aae-1-2507	NW_001810847.1	-	1208837	1208782
Aae-1-2508	NW_001810547.1	-	1816728	1816673
Aae-1-2509	NW_001809957.1	+	1098051	1098106
Aae-1-2510	NW_001810211.1	+	2144399	2144454
Aae-1-2511	NW_001809968.1	+	1597433	1597488
Aae-1-2512	NW_001810145.1	-	491528	491473
Aae-1-2513	NW_001810782.1	-	3192216	3192161
Aae-1-2514	NW_001811375.1	+	387639	387694
Aae-1-2515	NW_001811375.1	+	393812	393867
Aae-1-2516	NW_001835343.1	+	5104	5159
Aae-1-2517	NW_001809890.1	-	952006	951951
Aae-1-2518	NW_001809814.1	-	2756370	2756315
Aae-1-2519	NW_001811107.1	+	1581867	1581922
Aae-1-2520	NW_001809935.1	+	2518528	2518583
Aae-1-2521	NW_001810111.1	+	2048930	2048985
Aae-1-2522	NW_001811696.1	-	2539452	2539397
Aae-1-2523	NW_001810034.1	+	6953	7008
Aae-1-2524	NW_001836748.1	-	2133	2078
Aae-1-2525	NW_001809890.1	+	38140	38195
Aae-1-2526	NW_001810178.1	-	975248	975193
Aae-1-2527	NW_001810133.1	-	3374353	3374298
Aae-1-2528	NW_001809979.1	+	417423	417478
Aae-1-2529	NW_001811363.1	-	1435967	1435912
Aae-1-2530	NW_001809862.1	+	49333	49388

Aae-1-2531	NW_001810459.1	+	1464582	1464637
Aae-1-2532	NW_001810555.1	-	602184	602129
Aae-1-2533	NW_001810637.1	-	2504654	2504599
Aae-1-2534	NW_001810666.1	+	468631	468686
Aae-1-2535	NW_001810681.1	+	584831	584886
Aae-1-2536	NW_001810714.1	+	1224921	1224976
Aae-1-2537	NW_001810872.1	-	926057	926002
Aae-1-2538	NW_001810881.1	+	234702	234757
Aae-1-2539	NW_001810966.1	+	688846	688901
Aae-1-2540	NW_001810977.1	+	833354	833409
Aae-1-2541	NW_001811020.1	-	317182	317127
Aae-1-2542	NW_001811055.1	+	599986	600041
Aae-1-2543	NW_001811072.1	+	1276205	1276260
Aae-1-2544	NW_001811092.1	-	251234	251179
Aae-1-2545	NW_001811221.1	-	703777	703722
Aae-1-2546	NW_001811351.1	-	5030968	5030913
Aae-1-2547	NW_001811354.1	-	714819	714764
Aae-1-2548	NW_001811450.1	-	480449	480394
Aae-1-2549	NW_001811529.1	-	1840762	1840707
Aae-1-2550	NW_001811565.1	-	177963	177908
Aae-1-2551	NW_001811698.1	-	119331	119276
Aae-1-2552	NW_001809879.1	+	857417	857472
Aae-1-2553	NW_001810022.1	-	2990012	2989957
Aae-1-2554	NW_001810414.1	+	353690	353745
Aae-1-2555	NW_001810011.1	-	1444610	1444555
Aae-1-2556	NW_001810156.1	-	1843759	1843704
Aae-1-2557	NW_001810219.1	+	110553	110608
Aae-1-2558	NW_001810623.1	-	847636	847581
Aae-1-2559	NW_001810638.1	-	356606	356551
Aae-1-2560	NW_001810837.1	-	2529788	2529733
Aae-1-2561	NW_001810905.1	+	775823	775878
Aae-1-2562	NW_001810929.1	+	150978	151033
Aae-1-2563	NW_001810955.1	+	845230	845285
Aae-1-2564	NW_001810994.1	-	532841	532786
Aae-1-2565	NW_001811009.1	+	775743	775798
Aae-1-2566	NW_001811019.1	-	2874175	2874120
Aae-1-2567	NW_001811033.1	-	40489	40434
Aae-1-2568	NW_001811093.1	+	628933	628988
Aae-1-2569	NW_001811121.1	-	161198	161143
Aae-1-2570	NW_001811130.1	+	2638209	2638264
Aae-1-2571	NW_001811130.1	+	3134582	3134637
Aae-1-2572	NW_001811204.1	-	464324	464269
Aae-1-2573	NW_001811252.1	+	2167813	2167868
Aae-1-2574	NW_001811307.1	+	2117711	2117766
Aae-1-2575	NW_001811407.1	-	2331745	2331690
Aae-1-2576	NW_001836765.1	-	5407	5352
Aae-1-2577	NW_001810283.1	-	118607	118552
Aae-1-2578	NW_001810637.1	+	4046007	4046062
Aae-1-2579	NW_001811038.1	+	65230	65285
Aae-1-2580	NW_001811207.1	+	2166822	2166877
Aae-1-2581	NW_001810458.1	+	662943	662998
Aae-1-2582	NW_001811118.1	-	2955412	2955357

Aae-1-2583	NW_001811618.1	-	119058	119003
Aae-1-2584	NW_001811409.1	+	611558	611613
Aae-1-2585	NW_001811151.1	+	499528	499583
Aae-1-2586	NW_001811151.1	-	499372	499317
Aae-1-2587	NW_001811131.1	-	1097948	1097893
Aae-1-2588	NW_001811089.1	-	471140	471085
Aae-1-2589	NW_001811749.1	+	7921	7976
Aae-1-2590	NW_001811060.1	-	287513	287458
Aae-1-2591	NW_001810231.1	-	1071913	1071858
Aae-1-2592	NW_001811094.1	+	954500	954555
Aae-1-2593	NW_001810111.1	+	832966	833021
Aae-1-2594	NW_001810286.1	+	275647	275702
Aae-1-2595	NW_001810508.1	+	577791	577846
Aae-1-2596	NW_001810791.1	-	802182	802127
Aae-1-2597	NW_001810825.1	-	536507	536452
Aae-1-2598	NW_001810990.1	+	849808	849863
Aae-1-2599	NW_001811023.1	-	431262	431207
Aae-1-2600	NW_001811123.1	-	1129653	1129598
Aae-1-2601	NW_001811163.1	-	331552	331497
Aae-1-2602	NW_001811193.1	+	826893	826948
Aae-1-2603	NW_001811228.1	+	575092	575147
Aae-1-2604	NW_001811258.1	+	553397	553452
Aae-1-2605	NW_001811307.1	+	3014510	3014565
Aae-1-2606	NW_001811349.1	-	92315	92260
Aae-1-2607	NW_001811409.1	+	513689	513744
Aae-1-2608	NW_001811415.1	-	136750	136695
Aae-1-2609	NW_001811462.1	+	3138205	3138260
Aae-1-2610	NW_001811475.1	+	76979	77034
Aae-1-2611	NW_001811183.1	-	693148	693093
Aae-1-2612	NW_001811493.1	-	417174	417119
Aae-1-2613	NW_001811507.1	+	1016314	1016369
Aae-1-2614	NW_001811540.1	+	1019650	1019705
Aae-1-2615	NW_001811534.1	+	110929	110984
Aae-1-2616	NW_001811495.1	-	134170	134115
Aae-1-2617	NW_001811456.1	+	382002	382057
Aae-1-2618	NW_001811351.1	+	1846207	1846262
Aae-1-2619	NW_001811317.1	-	617230	617175
Aae-1-2620	NW_001810955.1	+	2956260	2956315
Aae-1-2621	NW_001810853.1	+	36790	36845
Aae-1-2622	NW_001811234.1	-	279454	279399
Aae-1-2623	NW_001810987.1	-	877854	877799
Aae-1-2624	NW_001811118.1	-	1572539	1572484
Aae-1-2625	NW_001811296.1	-	878607	878552
Aae-1-2626	NW_001810912.1	+	2111586	2111641
Aae-1-2627	NW_001811049.1	+	704262	704317
Aae-1-2628	NW_001811048.1	+	1075796	1075851
Aae-1-2629	NW_001811037.1	-	141667	141612
Aae-1-2630	NW_001811030.1	+	1590087	1590142
Aae-1-2631	NW_001811083.1	-	135116	135061
Aae-1-2632	NW_001810962.1	-	645406	645351
Aae-1-2633	NW_001811071.1	-	556438	556383
Aae-1-2634	NW_001811230.1	+	481379	481434

Aae-1-2635	NW_001810022.1	-	3929345	3929290
Aae-1-2636	NW_001810868.1	-	259652	259597
Aae-1-2637	NW_001809912.1	+	663486	663541
Aae-1-2638	NW_001810011.1	-	1563953	1563898
Aae-1-2639	NW_001810022.1	-	3967314	3967259
Aae-1-2640	NW_001810782.1	+	4848375	4848430
Aae-1-2641	NW_001811131.1	-	833515	833460
Aae-1-2642	NW_001811240.1	+	4950596	4950651
Aae-1-2643	NW_001811263.1	+	668227	668282
Aae-1-2644	NW_001809820.1	-	207019	206964
Aae-1-2645	NW_001809887.1	+	50114	50169
Aae-1-2646	NW_001810339.1	+	1196409	1196464
Aae-1-2647	NW_001810791.1	+	1105373	1105428
Aae-1-2648	NW_001811017.1	+	179203	179258
Aae-1-2649	NW_001809822.1	+	81763	81818
Aae-1-2650	NW_001809879.1	+	618259	618314
Aae-1-2651	NW_001809935.1	+	194147	194202
Aae-1-2652	NW_001810067.1	-	1580594	1580539
Aae-1-2653	NW_001810436.1	+	1634838	1634893
Aae-1-2654	NW_001810974.1	-	1327527	1327472
Aae-1-2655	NW_001810984.1	-	1752379	1752324
Aae-1-2656	NW_001810985.1	+	1308237	1308292
Aae-1-2657	NW_001811055.1	-	47241	47186
Aae-1-2658	NW_001811129.1	-	2200695	2200640
Aae-1-2659	NW_001811166.1	+	351057	351112
Aae-1-2660	NW_001811218.1	+	1328314	1328369
Aae-1-2661	NW_001811317.1	+	387970	388025
Aae-1-2662	NW_001811363.1	+	1051417	1051472
Aae-1-2663	NW_001811374.1	+	2633793	2633848
Aae-1-2664	NW_001811420.1	-	242523	242468
Aae-1-2665	NW_001811440.1	+	2619003	2619058
Aae-1-2666	NW_001837424.1	+	31	86
Aae-1-2667	NW_001810813.1	-	1009461	1009406
Aae-1-2668	NW_001810881.1	-	280666	280611
Aae-1-2669	NW_001810508.1	-	1313174	1313119
Aae-1-2670	NW_001811650.1	-	358111	358056
Aae-1-2671	NW_001810242.1	-	3549700	3549645
Aae-1-2672	NW_001809801.1	+	2779367	2779422
Aae-1-2673	NW_001811227.1	+	754464	754519
Aae-1-2674	NW_001809950.1	-	54446	54391
Aae-1-2675	NW_001810708.1	-	1095176	1095121
Aae-1-2676	NW_001810997.1	+	28129	28184
Aae-1-2677	NW_001811027.1	-	643580	643525
Aae-1-2678	NW_001811240.1	-	4594018	4593963
Aae-1-2679	NW_001811274.1	+	2015231	2015286
Aae-1-2680	NW_001811492.1	+	303137	303192
Aae-1-2681	NW_001811711.1	-	149580	149525
Aae-1-2682	NW_001809968.1	+	616426	616481
Aae-1-2683	NW_001810089.1	-	1987769	1987714
Aae-1-2684	NW_001810145.1	+	1141230	1141285
Aae-1-2685	NW_001811039.1	+	845046	845101
Aae-1-2686	NW_001811506.1	-	200327	200272

Aae-1-2687	NW_001811596.1	-	2841473	2841418
Aae-1-2688	NW_001810133.1	-	3749112	3749057
Aae-1-2689	NW_001811459.1	-	199792	199737
Aae-1-2690	NW_001810148.1	+	46457	46512
Aae-1-2691	NW_001810783.1	+	2238170	2238225
Aae-1-2692	NW_001810837.1	+	802355	802410
Aae-1-2693	NW_001809912.1	+	1865999	1866054
Aae-1-2694	NW_001810189.1	+	2100678	2100733
Aae-1-2695	NW_001810350.1	+	2913878	2913933
Aae-1-2696	NW_001810555.1	-	2244950	2244895
Aae-1-2697	NW_001810714.1	+	958087	958142
Aae-1-2698	NW_001810917.1	+	1468913	1468968
Aae-1-2699	NW_001810950.1	-	470036	469981
Aae-1-2700	NW_001810994.1	-	568476	568421
Aae-1-2701	NW_001811007.1	-	2539358	2539303
Aae-1-2702	NW_001811179.1	+	868874	868929
Aae-1-2703	NW_001811183.1	-	641145	641090
Aae-1-2704	NW_001811354.1	+	303624	303679
Aae-1-2705	NW_001811425.1	+	283090	283145
Aae-1-2706	NW_001811562.1	-	448989	448934
Aae-1-2707	NW_001811619.1	+	22421	22476
Aae-1-2708	NW_001810852.1	-	1059261	1059209
Aae-1-2709	NW_001811312.1	+	304238	304293
env-77	JCVI_SCAF_1096627510720	-	1705	1650
Aae-1-2710	NW_001811274.1	+	1479355	1479410
Aae-1-2711	NW_001811164.1	-	263092	263037
Aae-1-2712	NW_001837449.1	+	39752	39807
Aae-1-2713	NW_001811130.1	+	2508520	2508575
Aae-1-2714	NW_001811632.1	+	243305	243360
Aae-1-2715	NW_001811072.1	-	804784	804729
Aae-1-2716	NW_001811047.1	-	674654	674599
Aae-1-2717	NW_001810955.1	-	1835303	1835248
Aae-1-2718	NW_001809936.1	+	30506	30561
Aae-1-2719	NW_001811141.1	-	1540738	1540683
Aae-1-2720	NW_001810935.1	-	213833	213778
Aae-1-2721	NW_001810221.1	-	1399304	1399249
Aae-1-2722	NW_001810044.1	+	69199	69254
Aae-1-2723	NW_001809801.1	+	1785513	1785568
Aae-1-2724	NW_001810242.1	-	1030269	1030214
Aae-1-2725	NW_001811060.1	-	455083	455028
Aae-1-2726	NW_001811349.1	-	506391	506336
Aae-1-2727	NW_001811618.1	-	492258	492203
Aae-1-2728	NW_001809888.1	-	198697	198642
Aae-1-2729	NW_001810318.1	-	1118059	1118004
Aae-1-2730	NW_001810556.1	-	1065284	1065229
Aae-1-2731	NW_001810688.1	+	626229	626284
Aae-1-2732	NW_001810830.1	+	1675080	1675135
Aae-1-2733	NW_001810958.1	-	908554	908499
Aae-1-2734	NW_001811155.1	-	1140040	1139985
Aae-1-2735	NW_001811240.1	+	4931475	4931530
Aae-1-2736	NW_001811279.1	+	409285	409340
Aae-1-2737	NW_001811349.1	-	674745	674690

Aae-1-2738	NW_001811512.1	-	462061	462006
Aae-1-2739	NW_001811774.1	-	284669	284614
Aae-1-2740	NW_001810912.1	+	2538998	2539053
Aae-1-2741	NW_001810880.1	+	870844	870899
Aae-1-2742	NW_001810852.1	-	1568163	1568108
Aae-1-2743	NW_001810610.1	-	3260	3205
Aae-1-2744	NW_001810537.1	+	1960885	1960940
Aae-1-2745	NW_001810527.1	+	520554	520609
Aae-1-2746	NW_001811044.1	-	825310	825255
Aae-1-2747	NW_001810436.1	+	1080577	1080632
Aae-1-2748	NW_001810681.1	-	1378924	1378869
Aae-1-2749	NW_001810440.1	+	7427	7482
Aae-1-2750	NW_001810242.1	+	1049047	1049102
Aae-1-2751	NW_001810499.1	+	612537	612592
Aae-1-2752	NW_001811073.1	+	1163893	1163948
Aae-1-2753	NW_001809802.1	-	38621	38566
Aae-1-2754	NW_001809847.1	+	1794238	1794293
Aae-1-2755	NW_001810133.1	-	2860806	2860751
Aae-1-2756	NW_001810242.1	+	2288265	2288320
Aae-1-2757	NW_001810813.1	+	330797	330852
Aae-1-2758	NW_001810844.1	+	1132600	1132655
Aae-1-2759	NW_001810955.1	-	250738	250683
Aae-1-2760	NW_001810980.1	-	1226938	1226883
Aae-1-2761	NW_001810984.1	-	2591356	2591301
Aae-1-2762	NW_001811051.1	-	646350	646295
Aae-1-2763	NW_001811065.1	+	362623	362678
Aae-1-2764	NW_001811115.1	+	1063411	1063466
Aae-1-2765	NW_001811130.1	+	1646720	1646775
Aae-1-2766	NW_001811174.1	-	2810953	2810898
Aae-1-2767	NW_001811185.1	+	1913693	1913748
Aae-1-2768	NW_001811189.1	+	739341	739396
Aae-1-2769	NW_001811215.1	+	722983	723038
Aae-1-2770	NW_001811248.1	-	205645	205590
Aae-1-2771	NW_001811351.1	+	3010390	3010445
Aae-1-2772	NW_001811540.1	-	1992829	1992774
Aae-1-2773	NW_001811618.1	-	389503	389448
Aae-1-2774	NW_001837205.1	-	4202	4147
Aae-1-2775	NW_001809836.1	+	1449073	1449128
Aae-1-2776	NW_001810893.1	-	1321655	1321600
Aae-1-2777	NW_001809847.1	-	1794232	1794177
Aae-1-2778	NW_001810178.1	+	1385388	1385443
Aae-1-2779	NW_001811240.1	+	117258	117313
Aae-1-2780	NW_001811315.1	-	368904	368849
Aae-1-2781	NW_001811498.1	+	216257	216312
Aae-1-2782	NW_001811686.1	-	183943	183888
Aae-1-2783	NW_001810203.1	-	11198	11143
Aae-1-2784	NW_001809838.1	-	210456	210401
Aae-1-2785	NW_001809913.1	+	1330150	1330205
Aae-1-2786	NW_001810072.1	+	54597	54652
Aae-1-2787	NW_001810078.1	+	2217943	2217998
Aae-1-2788	NW_001810095.1	-	133718	133663
Aae-1-2789	NW_001810122.1	+	2284962	2285017

Aae-1-2790	NW_001810147.1	+	17587	17642
Aae-1-2791	NW_001810198.1	+	45228	45283
Aae-1-2792	NW_001810242.1	+	3918031	3918086
Aae-1-2793	NW_001810273.1	+	36115	36170
Aae-1-2794	NW_001810458.1	+	1095235	1095290
Aae-1-2795	NW_001810555.1	-	2979245	2979190
Aae-1-2796	NW_001810556.1	+	2042532	2042587
Aae-1-2797	NW_001810592.1	+	937995	938050
Aae-1-2798	NW_001810648.1	+	1221844	1221899
Aae-1-2799	NW_001810655.1	-	14950	14895
Aae-1-2800	NW_001810744.1	+	421505	421560
Aae-1-2801	NW_001810783.1	+	2921331	2921386
Aae-1-2802	NW_001810955.1	-	1754451	1754396
Aae-1-2803	NW_001810975.1	+	457087	457142
Aae-1-2804	NW_001811016.1	+	789927	789982
Aae-1-2805	NW_001811037.1	+	1078309	1078364
Aae-1-2806	NW_001811041.1	+	771866	771921
Aae-1-2807	NW_001811054.1	+	221852	221907
Aae-1-2808	NW_001811058.1	-	608759	608704
Aae-1-2809	NW_001811060.1	-	338359	338304
Aae-1-2810	NW_001811176.1	+	142001	142056
Aae-1-2811	NW_001811200.1	+	516476	516531
Aae-1-2812	NW_001811224.1	-	587163	587108
Aae-1-2813	NW_001811235.1	+	714115	714170
Aae-1-2814	NW_001811239.1	+	443375	443430
Aae-1-2815	NW_001811239.1	+	445065	445120
Aae-1-2816	NW_001811396.1	+	2535530	2535585
Aae-1-2817	NW_001811462.1	+	1286180	1286235
Aae-1-2818	NW_001811623.1	+	73328	73383
Aae-1-2819	NW_001811664.1	-	240734	240679
Aae-1-2820	NW_001811791.1	+	188532	188587
Aae-1-2821	NW_001837152.1	-	1297	1242
Aae-1-2822	NW_001809901.1	+	1893702	1893757
Aae-1-2823	NW_001810056.1	-	332245	332190
Aae-1-2824	NW_001810350.1	+	3541351	3541406
Aae-1-2825	NW_001810969.1	-	296696	296641
Aae-1-2826	NW_001835519.1	+	5437	5492
Aae-1-2827	NW_001809889.1	+	64752	64807
Aae-1-2828	NW_001809881.1	+	115483	115538
Aae-1-2829	NW_001810791.1	-	1462853	1462798
Aae-1-2830	NW_001810974.1	-	505718	505663
Aae-1-2831	NW_001837295.1	+	6433	6488
Aae-1-2832	NW_001811486.1	+	229220	229275
Aae-1-2833	NW_001811525.1	+	213205	213260
Aae-1-2834	NW_001811573.1	+	4345571	4345626
Aae-1-2835	NW_001811510.1	-	208547	208492
Aae-1-2836	NW_001811032.1	+	1160044	1160099
Aae-1-2837	NW_001811241.1	+	2307922	2307977
Aae-1-2838	NW_001811710.1	-	160937	160882
Aae-1-2839	NW_001811618.1	+	2370298	2370353
Aae-1-2840	NW_001811509.1	-	334074	334019
Aae-1-2841	NW_001811673.1	-	76847	76792

Aae-1-2842	NW_001811718.1	-	222230	222175
Aae-1-2843	NW_001811067.1	-	497428	497373
Aae-1-2844	NW_001811066.1	+	419859	419914
Aae-1-2845	NW_001810954.1	-	1711170	1711115
Aae-1-2846	NW_001810984.1	-	31028	30973
Aae-1-2847	NW_001810783.1	+	796113	796168
Aae-1-2848	NW_001811018.1	-	4843932	4843877
Aae-1-2849	NW_001810508.1	-	869122	869067
Aae-1-2850	NW_001810783.1	-	1055347	1055292
Aae-1-2851	NW_001809802.1	-	35994	35939
Aae-1-2852	NW_001810022.1	+	299105	299160
Aae-1-2853	NW_001810318.1	-	623620	623565
Aae-1-2854	NW_001810437.1	+	21588	21643
Aae-1-2855	NW_001810681.1	-	1873604	1873549
Aae-1-2856	NW_001810756.1	-	651065	651010
Aae-1-2857	NW_001810880.1	+	3009171	3009226
Aae-1-2858	NW_001810905.1	+	994653	994708
Aae-1-2859	NW_001810984.1	-	2587195	2587140
Aae-1-2860	NW_001811034.1	+	1073747	1073802
Aae-1-2861	NW_001811115.1	+	920151	920206
Aae-1-2862	NW_001811129.1	+	312029	312084
Aae-1-2863	NW_001811131.1	-	803323	803268
Aae-1-2864	NW_001811221.1	-	525301	525246
Aae-1-2865	NW_001811409.1	+	164492	164547
Aae-1-2866	NW_001811523.1	+	404547	404602
Aae-1-2867	NW_001811629.1	-	618269	618214
Aae-1-2868	NW_001809868.1	+	2331267	2331322
Aae-1-2869	NW_001811474.1	+	2197080	2197135
Aae-1-2870	NW_001811596.1	-	725625	725570
Aae-1-2871	NW_001811629.1	-	1883398	1883343
Aae-1-2872	NW_001809852.1	+	121830	121885
Aae-1-2873	NW_001809875.1	-	11818	11763
Aae-1-2874	NW_001811791.1	-	188492	188433
Aae-1-2875	NW_001810981.1	+	518832	518886
Aae-1-2876	NW_001809912.1	+	3497815	3497868
Aae-1-2877	NW_001810133.1	+	158903	158958
Aae-1-2878	NW_001810403.1	+	271701	271754
Aae-1-2879	NW_001811227.1	-	323169	323116
Aae-1-2880	NW_001811133.1	-	824095	824040
Aae-1-2881	NW_001811499.1	+	438444	438496
Aae-1-2882	NW_001811671.1	-	352122	352067
Aae-1-2883	NW_001811007.1	+	3764396	3764452
Aae-1-2884	NW_001811385.1	+	302046	302100
Aae-1-2885	NW_001811384.1	-	140846	140792
Aae-1-2886	NW_001811498.1	+	171742	171796
Aae-1-2887	NW_001810350.1	-	1336879	1336827
Aae-1-2888	NW_001810955.1	-	2556042	2555990
Aae-1-2889	NW_001811095.1	-	50472	50420
Aae-1-2890	NW_001811647.1	-	34183	34128
Aae-1-2891	NW_001811252.1	+	1712116	1712166
Aae-1-2892	NW_001811621.1	-	428464	428409
Aae-1-2893	NW_001811496.1	-	660627	660577

Aae-1-2894	NW_001810929.1	-	1335898	1335843
Aae-1-2895	NW_001811729.1	+	1923063	1923118
Aae-1-2896	NW_001811163.1	+	425582	425637
Aae-1-2897	NW_001811094.1	+	242391	242446
Aae-1-2898	NW_001811065.1	-	516973	516918
Aae-1-2899	NW_001809847.1	-	2503204	2503149
Aae-1-2900	NW_001811048.1	+	873709	873764
Aae-1-2901	NW_001810034.1	+	1142049	1142104
Aae-1-2902	NW_001810935.1	-	234395	234342
Aae-1-2903	NW_001811212.1	-	56703	56650
Aae-1-2904	NW_001810000.1	+	1943128	1943183
Aae-1-2905	NW_001810556.1	+	1891071	1891126
Aae-1-2906	NW_001811141.1	+	3028884	3028939
Aae-1-2907	NW_001811004.1	+	784053	784108
Aae-1-2908	NW_001811540.1	-	1893430	1893375
Aae-1-2909	NW_001810243.1	-	1118996	1118941
Aae-1-2910	NW_001811161.1	-	219827	219772
Aae-1-2911	NW_001811771.1	-	98257	98202
Aae-1-2912	NW_001811210.1	-	877500	877445
Aae-1-2913	NW_001811127.1	+	638802	638857
Aae-1-2914	NW_001810490.1	+	305335	305390
Aae-1-2915	NW_001811003.1	+	818604	818659
Aae-1-2916	NW_001811366.1	-	231824	231769
Aae-1-2917	NW_001811534.1	-	78370	78315
Aae-1-2918	NW_001810666.1	-	1142207	1142152
Aae-1-2919	NW_001811243.1	+	788372	788427
Aae-1-2920	NW_001810838.1	-	1115670	1115615
Aae-1-2921	NW_001810941.1	-	1612502	1612447
Aae-1-2922	NW_001810963.1	+	870335	870390
Aae-1-2923	NW_001810067.1	+	1667077	1667132
Aae-1-2924	NW_001810816.1	-	682619	682564
Aae-1-2925	NW_001811579.1	+	172692	172742
Aae-1-2926	NW_001811118.1	+	2442988	2443043
Aae-1-2927	NW_001811680.1	-	318026	317971
Aae-1-2928	NW_001809942.1	+	190166	190221
Aae-1-2929	NW_001810935.1	+	1135248	1135303
Aae-1-2930	NW_001811540.1	-	2566806	2566756
Aae-1-2931	NW_001811229.1	-	117801	117746
Aae-1-2932	NW_001811252.1	-	513041	512986
Aae-1-2933	NW_001811266.1	+	694821	694876
Aae-1-2934	NW_001811350.1	-	431418	431363
Aae-1-2935	NW_001811391.1	-	635023	634968
Aae-1-2936	NW_001811252.1	+	1901527	1901576
Aae-1-2937	NW_001811377.1	-	570769	570721
Aae-1-2938	NW_001837352.1	-	15192	15138
Aae-1-2939	NW_001811373.1	-	186496	186442
Aae-1-2940	NW_001811507.1	+	2314797	2314850
Aae-1-2941	NW_001811351.1	+	2734187	2734241
Aae-1-2942	NW_001811185.1	+	754903	754958
Aae-1-2943	NW_001811041.1	+	1137677	1137728
Aae-1-2944	NW_001811108.1	+	907001	907056
Aae-1-2945	NW_001811210.1	+	780977	781032

Aae-1-2946	NW_001811660.1	-	218140	218085
Aae-1-2947	NW_001811084.1	-	366364	366310
Aae-1-2948	NW_001811446.1	-	186163	186109
Aae-1-2949	NW_001810592.1	+	1239632	1239685
Aae-1-2950	NW_001810947.1	-	900819	900767
Aae-1-2951	NW_001810935.1	-	197967	197915
Aae-1-2952	NW_001811384.1	-	9109	9057
Aae-1-2953	NW_001811010.1	-	1296818	1296769
Aae-1-2954	NW_001810235.1	-	99541	99490
Aae-1-2955	NW_001810078.1	+	954732	954784
Aae-1-2956	NW_001811105.1	+	1024292	1024346
Aae-1-2957	NW_001809879.1	+	549481	549535
Aae-1-2958	NW_001811084.1	-	937657	937605
Aae-1-2959	NW_001811593.1	-	402762	402708
Aae-1-2960	NW_001811037.1	+	458748	458800
Aae-1-2961	NW_001810715.1	+	732304	732358
Aae-1-2962	NW_001811065.1	+	784698	784752
Aae-1-2963	NW_001810565.1	+	90184	90238
Aae-1-2964	NW_001811759.1	+	6644	6698
Aae-1-2965	NW_001811609.1	+	319953	320007
Aae-1-2966	NW_001810912.1	+	717227	717281
Aae-1-2967	NW_001810231.1	-	1137779	1137725
Aae-1-2968	NW_001810960.1	+	239365	239419
Aae-1-2969	NW_001811174.1	+	2591619	2591673
Aae-1-2970	NW_001810945.1	-	121440	121386
Aae-1-2971	NW_001811432.1	-	237820	237766
Aae-1-2972	NW_001809927.1	-	141987	141933
Aae-1-2973	NW_001809924.1	+	368637	368691
Aae-1-2974	NW_001810986.1	-	1140211	1140157
Aae-1-2975	NW_001811670.1	+	490914	490976
Aae-1-2976	NW_001809816.1	-	42672	42618
Aae-1-2977	NW_001811300.1	+	281405	281459
Aae-1-2978	NW_001810681.1	-	822849	822795
Aae-1-2979	NW_001810750.1	-	308856	308802
Aae-1-2980	NW_001811237.1	+	835415	835469
Aae-1-2981	NW_001811281.1	+	88568	88622
Aae-1-2982	NW_001811597.1	-	174641	174587
Aae-1-2983	NW_001811440.1	-	702546	702494
Aae-1-2984	NW_001811296.1	-	356421	356369
Aae-1-2985	NW_001811263.1	+	2933054	2933106
Aae-1-2986	NW_001811207.1	+	2984203	2984257
Aae-1-2987	NW_001811393.1	+	518408	518461
Aae-1-2988	NW_001811196.1	-	2884408	2884356
Aae-1-2989	NW_001811193.1	-	756433	756379
Aae-1-2990	NW_001811140.1	-	381800	381748
Aae-1-2991	NW_001811137.1	-	771436	771384
Aae-1-2992	NW_001811496.1	-	2464639	2464585
Aae-1-2993	NW_001811120.1	-	406102	406048
Aae-1-2994	NW_001811109.1	-	458824	458772
Aae-1-2995	NW_001811085.1	+	1635116	1635168
Aae-1-2996	NW_001811059.1	+	854808	854860
Aae-1-2997	NW_001811056.1	+	13812	13866

Aae-1-2998	NW_001811035.1	-	1106277	1106225
Aae-1-2999	NW_001834983.1	+	2506	2558
Aae-1-3000	NW_001811539.1	-	508542	508475
Aae-1-3001	NW_001811360.1	+	538592	538645
Aae-1-3002	NW_001810178.1	+	1462583	1462635
Aae-1-3003	NW_001811389.1	-	607628	607576
Aae-1-3004	NW_001811327.1	-	143442	143390
Aae-1-3005	NW_001836490.1	-	7767	7715
Aae-1-3006	NW_001811381.1	-	113190	113138
Aae-1-3007	NW_001811573.1	-	1625306	1625254
Aae-1-3008	NW_001811673.1	-	124838	124786
Aae-1-3009	NW_001810656.1	+	1186416	1186471
Aae-1-3010	NW_001811684.1	-	375235	375183
Aae-1-3011	NW_001810852.1	+	1702854	1702908
Aae-1-3012	NW_001810763.1	+	656902	656954
Aae-1-3013	NW_001810616.1	-	755258	755204
Aae-1-3014	NW_001811463.1	-	937880	937828
Aae-1-3015	NW_001810034.1	-	1348797	1348745
Aae-1-3016	NW_001809957.1	-	1959918	1959866
Aae-1-3017	NW_001811432.1	-	335473	335419
Aae-1-3018	NW_001811252.1	-	381481	381428
Aae-1-3019	NW_001811710.1	-	251980	251926
Aae-1-3020	NW_001811702.1	+	55249	55303
Aae-1-3021	NW_001811206.1	+	83159	83213
Aae-1-3022	NW_001811193.1	+	740375	740429
Aae-1-3023	NW_001811077.1	+	836895	836949
Aae-1-3024	NW_001811050.1	+	1027431	1027485
Aae-1-3025	NW_001811024.1	-	533069	533015
Aae-1-3026	NW_001810403.1	-	177954	177900
Aae-1-3027	NW_001811011.1	-	799208	799152
Aae-1-3028	NW_001811007.1	-	2876952	2876898
Aae-1-3029	NW_001811462.1	-	2322218	2322164
Aae-1-3030	NW_001810980.1	+	1180780	1180834
Aae-1-3031	NW_001810966.1	-	1566586	1566532
Aae-1-3032	NW_001810838.1	-	1446876	1446822
Aae-1-3033	NW_001811520.1	+	170742	170796
Aae-1-3034	NW_001810178.1	-	47667	47613
Aae-1-3035	NW_001810056.1	+	1095863	1095917
Aae-1-3036	NW_001811469.1	+	83928	83982
Aae-1-3037	NW_001809935.1	+	2050936	2050990
Aae-1-3038	NW_001811596.1	+	1090289	1090343
Aae-1-3039	NW_001811599.1	+	233174	233228
Aae-1-3040	NW_001811744.1	+	243060	243113
Aae-1-3041	NW_001810969.1	-	240541	240489
Aae-1-3042	NW_001811718.1	+	566996	567048
Aae-1-3043	NW_001811707.1	-	2230545	2230496
Aae-1-3044	NW_001810159.1	+	75690	75742
Aae-1-3045	NW_001810638.1	+	320037	320089
Aae-1-3046	NW_001811079.1	-	670358	670305
Aae-1-3047	NW_001810981.1	-	804372	804321
Aae-1-3048	NW_001810681.1	+	1404357	1404409
Aae-1-3049	NW_001810033.1	-	124178	124126

Aae-1-3050	NW_001811018.1	+	2336180	2336232
Aae-1-3051	NW_001811168.1	-	24123	24071
Aae-1-3052	NW_001811414.1	-	571488	571436
Aae-1-3053	NW_001811418.1	-	302970	302918
Aae-1-3054	NW_001809896.1	-	132142	132090
Aae-1-3055	NW_001811713.1	-	367736	367684
Aae-1-3056	NW_001810134.1	+	2393389	2393441
Aae-1-3057	NW_001810638.1	+	745502	745554
Aae-1-3058	NW_001810782.1	+	4975063	4975115
Aae-1-3059	NW_001810254.1	-	1811101	1811055
Aae-1-3060	NW_001811429.1	+	406538	406589
Aae-1-3061	NW_001811122.1	+	423768	423816
Aae-1-3062	NW_001810981.1	+	517081	517131
Aae-1-3063	NW_001811719.1	+	66225	66279
Aae-1-3064	NW_001810868.1	-	792873	792819
Aae-1-3065	NW_001810996.1	+	2840239	2840290
Aae-1-3066	NW_001810242.1	-	861493	861442
Aae-1-3067	NW_001810242.1	+	2161080	2161134
Aae-1-3068	NW_001810167.1	-	600075	600024
Aae-1-3069	NW_001810034.1	+	2090761	2090815
Aae-1-3070	NW_001811762.1	+	217555	217607
Aae-1-3071	NW_001811754.1	-	126424	126372
Aae-1-3072	NW_001811684.1	+	840715	840768
Aae-1-3073	NW_001811530.1	+	82914	82966
Aae-1-3074	NW_001811527.1	-	277876	277824
Aae-1-3075	NW_001811514.1	-	123734	123682
Aae-1-3076	NW_001811175.1	-	464022	463970
Aae-1-3077	NW_001811502.1	-	491101	491049
Aae-1-3078	NW_001811477.1	+	317312	317364
Aae-1-3079	NW_001835868.1	-	3378	3326
Aae-1-3080	NW_001811385.1	-	83810	83758
Aae-1-3081	NW_001811370.1	+	182624	182676
Aae-1-3082	NW_001811351.1	-	4032785	4032733
Aae-1-3083	NW_001811322.1	-	571330	571278
Aae-1-3084	NW_001811318.1	+	744057	744109
Aae-1-3085	NW_001811315.1	+	237996	238048
Aae-1-3086	NW_001811225.1	+	420703	420755
Aae-1-3087	NW_001811222.1	-	962130	962078
Aae-1-3088	NW_001811170.1	-	701010	700958
Aae-1-3089	NW_001811168.1	+	943207	943259
Aae-1-3090	NW_001810000.1	-	1008678	1008626
Aae-1-3091	NW_001810805.1	-	657295	657243
Aae-1-3092	NW_001811051.1	-	1104927	1104875
Aae-1-3093	NW_001811380.1	+	238331	238383
Aae-1-3094	NW_001811640.1	-	1181335	1181283
Aae-1-3095	NW_001811662.1	+	1639945	1639997
Aae-1-3096	NW_001810041.1	+	89118	89170
Aae-1-3097	NW_001811154.1	-	106730	106678
Aae-1-3098	NW_001811135.1	+	375836	375888
Aae-1-3099	NW_001811110.1	+	171295	171347
Aae-1-3100	NW_001811096.1	+	3458294	3458346
Aae-1-3101	NW_001811077.1	+	1154544	1154596

Aae-1-3102	NW_001811071.1	-	1185345	1185293
Aae-1-3103	NW_001811055.1	+	208797	208849
Aae-1-3104	NW_001811054.1	+	430917	430969
Aae-1-3105	NW_001811042.1	-	848189	848135
Aae-1-3106	NW_001811019.1	+	1959069	1959121
Aae-1-3107	NW_001811018.1	-	2846876	2846824
Aae-1-3108	NW_001811007.1	+	1911737	1911789
Aae-1-3109	NW_001810998.1	-	806579	806527
Aae-1-3110	NW_001810984.1	+	136450	136502
Aae-1-3111	NW_001810963.1	-	508898	508846
Aae-1-3112	NW_001810954.1	-	956096	956044
Aae-1-3113	NW_001810895.1	-	872170	872118
Aae-1-3114	NW_001810783.1	-	1435624	1435572
Aae-1-3115	NW_001810721.1	-	783800	783748
Aae-1-3116	NW_001810675.1	-	383140	383088
Aae-1-3117	NW_001810629.1	+	403886	403938
Aae-1-3118	NW_001810304.1	-	1209	1157
Aae-1-3119	NW_001811551.1	-	2094492	2094440
Aae-1-3120	NW_001810182.1	+	162775	162827
Aae-1-3121	NW_001810134.1	-	891499	891445
Aae-1-3122	NW_001810042.1	+	193983	194035
Aae-1-3123	NW_001809989.1	+	875128	875180
Aae-1-3124	NW_001809935.1	+	972875	972927
Aae-1-3125	NW_001809879.1	+	2357464	2357516
Aae-1-3126	NW_001809857.1	+	143740	143792
Aae-1-3127	NW_001811670.1	-	490869	490815
Aae-1-3128	NW_001811576.1	+	181039	181093
Aae-1-3129	NW_001811477.1	-	91718	91664
Aae-1-3130	NW_001811442.1	+	70114	70168
Aae-1-3131	NW_001810592.1	+	664332	664386
Aae-1-3132	NW_001809912.1	+	3039260	3039314
Aae-1-3133	NW_001810122.1	-	2243304	2243250
Aae-1-3134	NW_001811351.1	+	2728885	2728939
Aae-1-3135	NW_001811440.1	-	1409501	1409447
Aae-1-3136	NW_001811348.1	-	238336	238282
Aae-1-3137	NW_001811296.1	+	1130	1185
Aae-1-3138	NW_001811295.1	-	76991	76937
Aae-1-3139	NW_001811252.1	+	1770618	1770672
Aae-1-3140	NW_001811203.1	-	43778	43724
Aae-1-3141	NW_001811133.1	-	995703	995649
Aae-1-3142	NW_001811099.1	-	79465	79411
Aae-1-3143	NW_001811070.1	+	1099669	1099723
Aae-1-3144	NW_001811019.1	-	495155	495101
Aae-1-3145	NW_001810929.1	+	968274	968328
Aae-1-3146	NW_001810852.1	-	652146	652092
Aae-1-3147	NW_001810813.1	-	304411	304357
Aae-1-3148	NW_001810763.1	-	912158	912104
Aae-1-3149	NW_001810714.1	-	2923461	2923407
Aae-1-3150	NW_001809951.1	+	145628	145682
Aae-1-3151	NW_001810960.1	-	395431	395377
Aae-1-3152	NW_001811463.1	+	1940874	1940928
Aae-1-3153	NW_001810702.1	+	1646261	1646315

Aae-1-3154	NW_001810575.1	-	1198903	1198849
Aae-1-3155	NW_001810481.1	-	36084	36030
Aae-1-3156	NW_001810459.1	-	2102994	2102940
Aae-1-3157	NW_001810089.1	+	901500	901554
Aae-1-3158	NW_001810133.1	+	2233520	2233570
Aae-1-3159	NW_001809817.1	-	95596	95546
Aae-1-3160	NW_001811087.1	+	503151	503205
Aae-1-3161	NW_001811610.1	-	512076	512025
Aae-1-3162	NW_001810178.1	+	2200619	2200670
Aae-1-3163	NW_001809803.1	+	1484770	1484821
Aae-1-3164	NW_001811035.1	-	504957	504905
Aae-1-3165	NW_001809922.1	+	190747	190799
Aae-1-3166	NW_001811290.1	+	354090	354142
Aae-1-3167	NW_001811651.1	+	1266596	1266650
Aae-1-3168	NW_001810133.1	-	101026	100974
Aae-1-3169	NW_001811010.1	-	893182	893130
Aae-1-3170	NW_001811076.1	-	355227	355175
Aae-1-3171	NW_001811107.1	+	582220	582272
Aae-1-3172	NW_001811429.1	-	604922	604868
Aae-1-3173	NW_001811129.1	+	3459173	3459225
Aae-1-3174	NW_001811507.1	-	855456	855404
Aae-1-3175	NW_001811103.1	-	1087236	1087184
Aae-1-3176	NW_001811036.1	-	213289	213237
Aae-1-3177	NW_001809979.1	+	1476544	1476596
Aae-1-3178	NW_001811327.1	+	477652	477704
Aae-1-3179	NW_001811337.1	-	213018	212966
Aae-1-3180	NW_001811363.1	+	1260938	1260990
Aae-1-3181	NW_001811585.1	+	2555174	2555226
Aae-1-3182	NW_001810157.1	+	48422	48474
Aae-1-3183	NW_001811793.1	-	229574	229520
Aae-1-3184	NW_001811596.1	+	1775613	1775665
Aae-1-3185	NW_001811337.1	-	614617	614565
Aae-1-3186	NW_001811138.1	+	485012	485064
Aae-1-3187	NW_001811252.1	+	781198	781250
Aae-1-3188	NW_001811207.1	+	385411	385463
Aae-1-3189	NW_001811059.1	-	572740	572688
Aae-1-3190	NW_001810972.1	-	444005	443953
Aae-1-3191	NW_001810941.1	+	420974	421026
Aae-1-3192	NW_001810371.1	+	749965	750017
Aae-1-3193	NW_001810371.1	-	749939	749887
Aae-1-3194	NW_001810213.1	+	14275	14327
Aae-1-3195	NW_001811019.1	+	2697081	2697133
Aae-1-3196	NW_001811228.1	+	663079	663131
Aae-1-3197	NW_001810254.1	-	1091087	1091035
Aae-1-3198	NW_001811285.1	-	509982	509930
Aae-1-3199	NW_001811112.1	+	1099187	1099239
Aae-1-3200	NW_001811003.1	+	777925	777977
Aae-1-3201	NW_001810189.1	+	435951	436003
Aae-1-3202	NW_001810864.1	+	892516	892570
Aae-1-3203	NW_001810971.1	+	948770	948824
Aae-1-3204	NW_001809986.1	+	135108	135162
Aae-1-3205	NW_001810880.1	+	2440689	2440741

Aae-1-3206	NW_001810977.1	+	670754	670806
Aae-1-3207	NW_001811141.1	+	112067	112119
Aae-1-3208	NW_001811301.1	+	573971	574023
Aae-1-3209	NW_001811276.1	+	104932	104984
Aae-1-3210	NW_001810847.1	-	1670251	1670199
Aae-1-3211	NW_001811229.1	+	1061551	1061605
Aae-1-3212	NW_001811105.1	+	314967	315021
Aae-1-3213	NW_001810945.1	+	345339	345393
Aae-1-3214	NW_001810945.1	+	346779	346833
Aae-1-3215	NW_001810945.1	+	158110	158164
Aae-1-3216	NW_001809824.1	-	112382	112328
Aae-1-3217	NW_001809801.1	-	5117112	5117058
Aae-1-3218	NW_001811329.1	-	1796648	1796594
Aae-1-3219	NW_001811340.1	-	2025165	2025111
Aae-1-3220	NW_001811206.1	-	204295	204241
Aae-1-3221	NW_001810681.1	-	1728527	1728473
Aae-1-3222	NW_001811115.1	+	688496	688550
Aae-1-3223	NW_001811141.1	+	789275	789329
Aae-1-3224	NW_001811573.1	+	4735676	4735730
Aae-1-3225	NW_001810555.1	-	1734066	1734012
Aae-1-3226	NW_001811018.1	+	3105173	3105227
Aae-1-3227	NW_001811018.1	+	3111751	3111805
Aae-1-3228	NW_001811018.1	-	3101823	3101769
Aae-1-3229	NW_001811027.1	-	93336	93282
Aae-1-3230	NW_001811041.1	+	1375323	1375377
Aae-1-3231	NW_001811585.1	-	2858159	2858105
Aae-1-3232	NW_001811620.1	+	491908	491962
Aae-1-3233	NW_001811729.1	+	1130959	1131013
Aae-1-3234	NW_001811770.1	+	345431	345485
Aae-1-3235	NW_001810459.1	+	2103002	2103056
Aae-1-3236	NW_001810307.1	-	2118782	2118728
Aae-1-3237	NW_001809894.1	+	192093	192147
Aae-1-3238	NW_001810891.1	+	1200773	1200827
Aae-1-3239	NW_001810020.1	-	32430	32379
Aae-1-3240	NW_001811251.1	-	738632	738581
Aae-1-3241	NW_001811470.1	-	256727	256673
Aae-1-3242	NW_001810855.1	+	242833	242887
Aae-1-3243	NW_001810895.1	+	284578	284632
Aae-1-3244	NW_001810996.1	-	1010412	1010358
Aae-1-3245	NW_001809817.1	+	95602	95656
Aae-1-3246	NW_001811251.1	+	801823	801877
Aae-1-3247	NW_001811048.1	+	103669	103723
Aae-1-3248	NW_001811299.1	+	253886	253940
Aae-1-3249	NW_001811419.1	+	528337	528391
Aae-1-3250	NW_001834983.1	-	1588	1534
Aae-1-3251	NW_001811099.1	+	79490	79542
Aae-1-3252	NW_001811260.1	+	62423	62475
Aae-1-3253	NW_001811137.1	-	602155	602103
Aae-1-3254	NW_001810944.1	+	419188	419240
Aae-1-3255	NW_001811224.1	+	721826	721878
Aae-1-3256	NW_001811274.1	+	1590688	1590741
Aae-1-3257	NW_001811034.1	+	691669	691721

Aae-1-3258	NW_001811152.1	+	742133	742185
Aae-1-3259	NW_001811468.1	+	300619	300671
Aae-1-3260	NW_001811318.1	-	1476458	1476406
Aae-1-3261	NW_001811412.1	-	376255	376203
Aae-1-3262	NW_001811640.1	-	330093	330041
Aae-1-3263	NW_001811574.1	-	2806887	2806835
Aae-1-3264	NW_001811303.1	+	683231	683283
Aae-1-3265	NW_001811317.1	-	425991	425939
Aae-1-3266	NW_001811460.1	-	158670	158618
Aae-1-3267	NW_001811449.1	-	374432	374380
Aae-1-3268	NW_001811405.1	+	389084	389136
Aae-1-3269	NW_001811385.1	-	2461170	2461118
Aae-1-3270	NW_001811385.1	+	2196797	2196849
Aae-1-3271	NW_001811369.1	+	208368	208420
Aae-1-3272	NW_001811308.1	+	701203	701255
Aae-1-3273	NW_001810286.1	-	1454552	1454500
Aae-1-3274	NW_001810984.1	-	886934	886882
Aae-1-3275	NW_001811163.1	+	390034	390086
Aae-1-3276	NW_001811181.1	-	296136	296084
Aae-1-3277	NW_001810023.1	-	2345094	2345042
Aae-1-3278	NW_001810054.1	+	142013	142065
Aae-1-3279	NW_001810111.1	-	1514327	1514275
Aae-1-3280	NW_001810791.1	+	1079396	1079448
Aae-1-3281	NW_001810952.1	+	1193489	1193541
Aae-1-3282	NW_001811718.1	-	2178839	2178787
Aae-1-3283	NW_001810034.1	-	1565223	1565171
Aae-1-3284	NW_001811003.1	-	234924	234872
Aae-1-3285	NW_001811115.1	+	1114967	1115019
Aae-1-3286	NW_001811339.1	+	33363	33415
Aae-1-3287	NW_001809913.1	-	636866	636813
Aae-1-3288	NW_001811021.1	+	1195632	1195684
Aae-1-3289	NW_001810681.1	-	1404141	1404087
Aae-1-3290	NW_001811045.1	+	952111	952163
Aae-1-3291	NW_001811007.1	+	2065257	2065309
Aae-1-3292	NW_001810852.1	-	1301714	1301662
Aae-1-3293	NW_001810783.1	+	766394	766446
Aae-1-3294	NW_001810462.1	+	2819	2871
Aae-1-3295	NW_001810414.1	-	453657	453605
Aae-1-3296	NW_001810221.1	+	1860176	1860228
Aae-1-3297	NW_001809847.1	-	1096174	1096122
Aae-1-3298	NW_001810174.1	-	14770	14718
Aae-1-3299	NW_001811156.1	+	631462	631514
Aae-1-3300	NW_001811562.1	+	2598175	2598227
Aae-1-3301	NW_001809861.1	-	54631	54579
Aae-1-3302	NW_001810990.1	-	1193038	1192986
Aae-1-3303	NW_001809825.1	+	997733	997785
Aae-1-3304	NW_001809814.1	-	1348197	1348145
Aae-1-3305	NW_001811180.1	-	65659	65607
Aae-1-3306	NW_001809816.1	+	42997	43049
Aae-1-3307	NW_001810027.1	-	97337	97285
Aae-1-3308	NW_001810157.1	+	18571	18623
Aae-1-3309	NW_001810556.1	+	2076033	2076085

Aae-1-3310	NW_001810912.1	-	3397203	3397151
Aae-1-3311	NW_001811163.1	+	1097089	1097141
Aae-1-3312	NW_001811543.1	-	213595	213543
Aae-1-3313	NW_001809879.1	-	1859371	1859319
Aae-1-3314	NW_001810783.1	-	2043496	2043444
Aae-1-3315	NW_001810929.1	-	731884	731832
Aae-1-3316	NW_001811022.1	+	1016530	1016582
Aae-1-3317	NW_001811074.1	+	707263	707315
Aae-1-3318	NW_001811351.1	-	1465857	1465805
Aae-1-3319	NW_001811443.1	-	361174	361122
Aae-1-3320	NW_001811229.1	+	1974944	1974996
Aae-1-3321	NW_001811344.1	-	416143	416091
Aae-1-3322	NW_001810192.1	-	46687	46635
Aae-1-3323	NW_001811391.1	-	128756	128704
Aae-1-3324	NW_001810592.1	-	58071	58017
Aae-1-3325	NW_001810637.1	-	2971742	2971690
Aae-1-3326	NW_001810998.1	-	1282249	1282197
Aae-1-3327	NW_001811029.1	-	160239	160187
Aae-1-3328	NW_001811474.1	-	2184634	2184582
Aae-1-3329	NW_001811452.1	+	551886	551938
Aae-1-3330	NW_001811761.1	-	1837933	1837881
Aae-1-3331	NW_001811596.1	-	1792068	1792016
Aae-1-3332	NW_001811565.1	+	36767	36819
Aae-1-3333	NW_001811525.1	+	99737	99789
Aae-1-3334	NW_001811506.1	+	441463	441515
Aae-1-3335	NW_001811010.1	-	517108	517056
Aae-1-3336	NW_001811188.1	-	769520	769468
Aae-1-3337	NW_001811263.1	-	3011203	3011151
Aae-1-3338	NW_001810784.1	+	1834240	1834292
Aae-1-3339	NW_001811098.1	-	709734	709682
Aae-1-3340	NW_001811070.1	+	752666	752718
Aae-1-3341	NW_001811216.1	-	334183	334131
Aae-1-3342	NW_001809945.1	-	207244	207192
Aae-1-3343	NW_001809979.1	+	959216	959268
Aae-1-3344	NW_001811375.1	+	433152	433204
Aae-1-3345	NW_001811202.1	-	589062	589010
Aae-1-3346	NW_001810134.1	-	2393354	2393302
Aae-1-3347	NW_001810782.1	-	4975028	4974976
Aae-1-3348	NW_001810855.1	+	459981	460033
Aae-1-3349	NW_001810981.1	+	662617	662669
Aae-1-3350	NW_001811098.1	-	246089	246037
Aae-1-3351	NW_001811380.1	-	621111	621059
Aae-1-3352	NW_001810952.1	-	1193452	1193400
Aae-1-3353	NW_001810967.1	-	864957	864905
Aae-1-3354	NW_001810929.1	+	1520809	1520861
Aae-1-3355	NW_001811067.1	+	107254	107306
Aae-1-3356	NW_001811540.1	+	2603986	2604038
Aae-1-3357	NW_001811421.1	-	427004	426952
Aae-1-3358	NW_001811418.1	-	2489671	2489619
Aae-1-3359	NW_001811311.1	-	123336	123284
Aae-1-3360	NW_001811292.1	+	82685	82737
Aae-1-3361	NW_001811280.1	-	311658	311606

Aae-1-3362	NW_001811254.1	+	231887	231939
Aae-1-3363	NW_001811446.1	+	537552	537604
Aae-1-3364	NW_001811718.1	+	89008	89060
Aae-1-3365	NW_001811252.1	+	538545	538597
Aae-1-3366	NW_001811101.1	-	720090	720038
Aae-1-3367	NW_001811665.1	+	338107	338159
Aae-1-3368	NW_001809957.1	+	1892276	1892328
Aae-1-3369	NW_001810784.1	+	1192665	1192717
Aae-1-3370	NW_001811152.1	+	3351578	3351630
Aae-1-3371	NW_001809910.1	-	132920	132868
Aae-1-3372	NW_001809913.1	+	255613	255665
Aae-1-3373	NW_001810111.1	+	64917	64969
Aae-1-3374	NW_001810782.1	-	1296527	1296475
Aae-1-3375	NW_001811010.1	+	1200522	1200574
Aae-1-3376	NW_001811037.1	-	854590	854538
Aae-1-3377	NW_001811398.1	-	89384	89332
Aae-1-3378	NW_001836538.1	-	1998	1946
Aae-1-3379	NW_001810830.1	+	908877	908929
Aae-1-3380	NW_001811045.1	-	366070	366017
Aae-1-3381	NW_001810782.1	+	4429779	4429831
Aae-1-3382	NW_001810837.1	-	2321471	2321419
Aae-1-3383	NW_001809915.1	+	55414	55466
Aae-1-3384	NW_001811024.1	+	361711	361763
Aae-1-3385	NW_001811470.1	+	37675	37727
Aae-1-3386	NW_001811079.1	-	681609	681557
Aae-1-3387	NW_001811478.1	-	262770	262718
Aae-1-3388	NW_001811064.1	+	1112500	1112552
Aae-1-3389	NW_001810275.1	+	668281	668333
Aae-1-3390	NW_001811319.1	+	189540	189592
Aae-1-3391	NW_001811351.1	-	903847	903795
Aae-1-3392	NW_001811119.1	-	412442	412390
Aae-1-3393	NW_001811115.1	-	611690	611638
Aae-1-3394	NW_001811039.1	+	880193	880245
Aae-1-3395	NW_001811017.1	-	610151	610099
Aae-1-3396	NW_001810974.1	+	556556	556608
Aae-1-3397	NW_001811504.1	-	484413	484359
Aae-1-3398	NW_001811647.1	-	212537	212483
Aae-1-3399	NW_001810950.1	+	1184148	1184202
Aae-1-3400	NW_001810648.1	+	645324	645378
Aae-1-3401	NW_001810232.1	+	101184	101238
Aae-1-3402	NW_001810920.1	+	62256	62310
Aae-1-3403	NW_001811029.1	+	836293	836347
Aae-1-3404	NW_001811040.1	+	1219365	1219419
Aae-1-3405	NW_001811201.1	-	439514	439460
Aae-1-3406	NW_001811662.1	+	2337220	2337274
Aae-1-3407	NW_001810111.1	-	826117	826063
Aae-1-3408	NW_001809961.1	-	26314	26260
Aae-1-3409	NW_001810519.1	-	491819	491765
Aae-1-3410	NW_001811207.1	+	1943249	1943303
Aae-1-3411	NW_001811607.1	+	488516	488570
Aae-1-3412	NW_001811684.1	+	3963179	3963233
Aae-1-3413	NW_001811757.1	-	162265	162211

Aae-1-3414	NW_001811764.1	+	312105	312159
Aae-1-3415	NW_001811772.1	+	2066628	2066682
Aae-1-3416	NW_001811242.1	-	297911	297857
Aae-1-3417	NW_001811217.1	+	801595	801649
Aae-1-3418	NW_001811147.1	+	1103978	1104032
Aae-1-3419	NW_001810696.1	+	1292719	1292773
Aae-1-3420	NW_001810647.1	+	34331	34385
Aae-1-3421	NW_001810264.1	-	2111285	2111231
Aae-1-3422	NW_001810436.1	-	630053	629999
Aae-1-3423	NW_001811225.1	+	371716	371770
Aae-1-3424	NW_001810350.1	+	1831642	1831696
Aae-1-3425	NW_001811261.1	-	354052	353998
Aae-1-3426	NW_001811153.1	+	798190	798244
Aae-1-3427	NW_001811112.1	-	15275	15221
Aae-1-3428	NW_001811109.1	+	1089428	1089482
Aae-1-3429	NW_001811033.1	+	343829	343883
Aae-1-3430	NW_001809803.1	+	2333239	2333293
Aae-1-3431	NW_001810022.1	-	2140558	2140504
Aae-1-3432	NW_001810458.1	-	3017501	3017447
Aae-1-3433	NW_001810555.1	-	4306081	4306027
Aae-1-3434	NW_001810675.1	+	101663	101717
Aae-1-3435	NW_001810838.1	-	803692	803638
Aae-1-3436	NW_001811385.1	-	1265123	1265069
Aae-1-3437	NW_001810616.1	-	1564708	1564654
Aae-1-3438	NW_001810935.1	+	473207	473261
Aae-1-3439	NW_001810955.1	-	1573689	1573635
Aae-1-3440	NW_001810696.1	-	590745	590691
Aae-1-3441	NW_001811373.1	+	89628	89682
Aae-1-3442	NW_001810447.1	-	255024	254970
Aae-1-3443	NW_001810508.1	+	771349	771403
Aae-1-3444	NW_001810795.1	+	496202	496256
Aae-1-3445	NW_001810666.1	+	1163358	1163412
Aae-1-3446	NW_001810050.1	+	112812	112866
Aae-1-3447	NW_001809901.1	+	301602	301656
Aae-1-3448	NW_001811252.1	+	381505	381559
Aae-1-3449	NW_001811316.1	+	125106	125160
Aae-1-3450	NW_001809803.1	-	514126	514072
Aae-1-3451	NW_001810980.1	-	577429	577375
Aae-1-3452	NW_001809803.1	-	2131573	2131519
Aae-1-3453	NW_001811044.1	+	987194	987248
Aae-1-3454	NW_001810963.1	+	1262619	1262673
Aae-1-3455	NW_001811457.1	+	346576	346627
Aae-1-3456	NW_001810019.1	+	141417	141471
Aae-1-3457	NW_001811418.1	+	2583945	2583999
Aae-1-3458	NW_001811462.1	-	3135760	3135706
Aae-1-3459	NW_001811545.1	+	114669	114723
Aae-1-3460	NW_001811334.1	-	697759	697704
Aae-1-3461	NW_001811761.1	-	310187	310133
Aae-1-3462	NW_001811224.1	-	637266	637214
Aae-1-3463	NW_001811152.1	+	856145	856197
Aae-1-3464	NW_001811662.1	+	1400393	1400445
Aae-1-3465	NW_001811269.1	-	941754	941702

Aae-1-3466	NW_001811241.1	-	738378	738326
Aae-1-3467	NW_001810783.1	-	3410396	3410344
Aae-1-3468	NW_001809957.1	+	968321	968373
Aae-1-3469	NW_001810783.1	-	2577294	2577242
Aae-1-3470	NW_001810051.1	-	2438	2386
Aae-1-3471	NW_001811542.1	-	60695	60643
Aae-1-3472	NW_001809801.1	-	2841208	2841156
Aae-1-3473	NW_001811183.1	-	308596	308544
Aae-1-3474	NW_001810537.1	-	1839565	1839513
Aae-1-3475	NW_001810328.1	+	1493832	1493884
Aae-1-3476	NW_001810156.1	-	1826661	1826609
Aae-1-3477	NW_001811281.1	+	273924	273976
Aae-1-3478	NW_001810307.1	+	2118800	2118852
Aae-1-3479	NW_001811211.1	+	124646	124698
Aae-1-3480	NW_001810805.1	-	868672	868620
Aae-1-3481	NW_001811377.1	+	545543	545595
Aae-1-3482	NW_001810913.1	+	1685678	1685730
Aae-1-3483	NW_001811168.1	+	893523	893575
Aae-1-3484	NW_001810967.1	+	362000	362052
Aae-1-3485	NW_001811218.1	-	3100166	3100114
Aae-1-3486	NW_001811093.1	+	670342	670394
Aae-1-3487	NW_001811007.1	+	2313028	2313080
Aae-1-3488	NW_001810714.1	-	1701680	1701628
Aae-1-3489	NW_001810996.1	+	455265	455317
Aae-1-3490	NW_001811084.1	+	668009	668061
Aae-1-3491	NW_001811285.1	+	3085544	3085596
Aae-1-3492	NW_001811483.1	-	347687	347635
Aae-1-3493	NW_001811640.1	-	1448037	1447985
Aae-1-3494	NW_001810078.1	+	921737	921789
Aae-1-3495	NW_001810960.1	-	237013	236961
Aae-1-3496	NW_001811514.1	+	410784	410836
Aae-1-3497	NW_001811130.1	+	1553851	1553903
Aae-1-3498	NW_001810296.1	-	1639659	1639607
Aae-1-3499	NW_001810984.1	+	3057276	3057328
Aae-1-3500	NW_001811252.1	+	1084858	1084910
Aae-1-3501	NW_001811288.1	+	622379	622431
Aae-1-3502	NW_001811351.1	-	3863650	3863598
Aae-1-3503	NW_001811462.1	+	2367418	2367470
Aae-1-3504	NW_001811077.1	-	392219	392167
Aae-1-3505	NW_001811083.1	-	339405	339353
Aae-1-3506	NW_001811385.1	-	2417066	2417014
Aae-1-3507	NW_001811578.1	+	176248	176300
Aae-1-3508	NW_001811651.1	-	1239276	1239222
Aae-1-3509	NW_001811263.1	-	2947676	2947624
Aae-1-3510	NW_001811717.1	-	298570	298517
Aae-1-3511	NW_001811655.1	+	123844	123896
Aae-1-3512	NW_001811054.1	-	430801	430749
Aae-1-3513	NW_001810837.1	-	2296554	2296502
Aae-1-3514	NW_001811309.1	+	764018	764070
Aae-1-3515	NW_001809854.1	-	51835	51783
Aae-1-3516	NW_001809901.1	-	1304965	1304913
Aae-1-3517	NW_001810190.1	-	34429	34377

Aae-1-3518	NW_001810275.1	+	1575248	1575300
Aae-1-3519	NW_001810805.1	-	872123	872071
Aae-1-3520	NW_001811008.1	+	1362589	1362641
Aae-1-3521	NW_001811028.1	+	767211	767263
Aae-1-3522	NW_001811607.1	-	2475817	2475765
Aae-1-3523	NW_001811696.1	-	1216322	1216270
Aae-1-3524	NW_001809992.1	-	27254	27202
Aae-1-3525	NW_001811512.1	-	426452	426400
Aae-1-3526	NW_001811619.1	+	137050	137102
Aae-1-3527	NW_001811063.1	-	3132410	3132354
Aae-1-3528	NW_001810211.1	-	441266	441214
Aae-1-3529	NW_001810638.1	+	1267911	1267963
Aae-1-3530	NW_001810656.1	+	189931	189983
Aae-1-3531	NW_001811079.1	+	670382	670434
Aae-1-3532	NW_001810891.1	+	456783	456837
Aae-1-3533	NW_001811603.1	-	376734	376682
Aae-1-3534	NW_001811569.1	+	175801	175853
Aae-1-3535	NW_001811514.1	-	293260	293208
Aae-1-3536	NW_001811260.1	+	773723	773775
Aae-1-3537	NW_001811429.1	-	748615	748561
Aae-1-3538	NW_001811385.1	+	143446	143500
Aae-1-3539	NW_001810078.1	-	2243434	2243382
Aae-1-3540	NW_001810459.1	-	1949790	1949738
Aae-1-3541	NW_001810791.1	-	1469286	1469234
Aae-1-3542	NW_001811116.1	+	784463	784515
Aae-1-3543	NW_001811440.1	-	2214940	2214888
Aae-1-3544	NW_001810458.1	-	2613706	2613654
Aae-1-3545	NW_001810995.1	-	370958	370906
Aae-1-3546	NW_001810976.1	-	382279	382227
Aae-1-3547	NW_001811385.1	+	504876	504928
Aae-1-3548	NW_001811481.1	-	319573	319519
Aae-1-3549	NW_001811205.1	-	722380	722326
Aae-1-3550	NW_001811096.1	-	2796203	2796151
Aae-1-3551	NW_001811462.1	+	4052076	4052128
Aae-1-3552	NW_001810318.1	+	1082425	1082477
Aae-1-3553	NW_001810339.1	-	1240405	1240353
Aae-1-3554	NW_001811331.1	+	616027	616079
Aae-1-3555	NW_001810350.1	-	1831625	1831573
Aae-1-3556	NW_001810392.1	+	912084	912136
Aae-1-3557	NW_001810537.1	+	1013465	1013517
Aae-1-3558	NW_001810919.1	-	356565	356513
Aae-1-3559	NW_001810955.1	+	3622349	3622401
Aae-1-3560	NW_001810966.1	-	1231742	1231690
Aae-1-3561	NW_001811120.1	+	160575	160627
Aae-1-3562	NW_001811633.1	+	36550	36602
Aae-1-3563	NW_001810990.1	-	76489	76437
Aae-1-3564	NW_001810805.1	+	1255820	1255872
Aae-1-3565	NW_001810750.1	-	848710	848658
Aae-1-3566	NW_001810623.1	-	1206586	1206534
Aae-1-3567	NW_001811098.1	-	810350	810298
Aae-1-3568	NW_001810231.1	+	68929	68981
Aae-1-3569	NW_001810102.1	+	81649	81701

Aae-1-3570	NW_001811185.1	+	1793404	1793456
Aae-1-3571	NW_001810022.1	-	900652	900600
Aae-1-3572	NW_001811065.1	-	784670	784618
Aae-1-3573	NW_001811546.1	-	292773	292721
Aae-1-3574	NW_001809801.1	-	4716282	4716230
Aae-1-3575	NW_001810575.1	-	662974	662922
Aae-1-3576	NW_001810425.1	-	1159039	1158981
Aae-1-3577	NW_001809825.1	-	1191141	1191089
Aae-1-3578	NW_001810425.1	+	376542	376594
Aae-1-3579	NW_001811130.1	-	1650285	1650233
Aae-1-3580	NW_001811607.1	-	488210	488158
Aae-1-3581	NW_001811694.1	-	203675	203623
Aae-1-3582	NW_001811703.1	+	207286	207338
Aae-1-3583	NW_001809913.1	-	2460008	2459956
Aae-1-3584	NW_001810666.1	-	1047603	1047551
Aae-1-3585	NW_001811063.1	+	2577454	2577506
Aae-1-3586	NW_001811096.1	-	2992456	2992404
Aae-1-3587	NW_001811158.1	+	983640	983692
Aae-1-3588	NW_001811431.1	+	460969	461021
Aae-1-3589	NW_001810575.1	+	842724	842776
Aae-1-3590	NW_001811764.1	-	311379	311327
Aae-1-3591	NW_001810995.1	-	733245	733191
Aae-1-3592	NW_001811297.1	-	904749	904695
Aae-1-3593	NW_001811116.1	-	786278	786224
Aae-1-3594	NW_001811173.1	+	511685	511739
Aae-1-3595	NW_001810984.1	+	221365	221419
Aae-1-3596	NW_001811010.1	-	1154143	1154089
Aae-1-3597	NW_001811407.1	+	644274	644328
Aae-1-3598	NW_001811288.1	-	397739	397685
Aae-1-3599	NW_001811271.1	-	114309	114255
Aae-1-3600	NW_001810078.1	+	183260	183314
Aae-1-3601	NW_001810714.1	-	2411937	2411883
Aae-1-3602	NW_001811651.1	+	1707475	1707529
Aae-1-3603	NW_001811346.1	-	363719	363668
Aae-1-3604	NW_001811394.1	-	642776	642729
Aae-1-3605	NW_001811282.1	+	572520	572574
Aae-1-3606	NW_001811346.1	+	342697	342751
Aae-1-3607	NW_001811354.1	-	127136	127082
Aae-1-3608	NW_001811783.1	-	465798	465744
Aae-1-3609	NW_001810242.1	-	2795867	2795816
Aae-1-3610	NW_001836342.1	-	6588	6533
Aae-1-3611	NW_001810318.1	-	2118890	2118836
Aae-1-3612	NW_001811004.1	-	1259238	1259184
Aae-1-3613	NW_001810955.1	+	1301808	1301862
Aae-1-3614	NW_001811159.1	+	916308	916362
Aae-1-3615	NW_001809901.1	-	2354308	2354254
Aae-1-3616	NW_001810952.1	+	157414	157464
Aae-1-3617	NW_001809834.1	+	162833	162886
Aae-1-3618	NW_001811529.1	+	50820	50873
Aae-1-3619	NW_001810381.1	-	992148	992094
Aae-1-3620	NW_001811562.1	+	765522	765576
Aae-1-3621	NW_001811068.1	-	235150	235096

Aae-1-3622	NW_001810350.1	-	2912553	2912499
Aae-1-3623	NW_001811063.1	+	3248757	3248811
Aae-1-3624	NW_001810480.1	-	2058116	2058062
Aae-1-3625	NW_001810459.1	+	1615410	1615464
Aae-1-3626	NW_001811031.1	-	693079	693025
Aae-1-3627	NW_001810243.1	+	658339	658393
Aae-1-3628	NW_001810648.1	-	539861	539807
Aae-1-3629	NW_001810999.1	+	489080	489134
Aae-1-3630	NW_001810231.1	-	620695	620641
Aae-1-3631	NW_001809946.1	+	579579	579633
Aae-1-3632	NW_001811769.1	+	132167	132221
Aae-1-3633	NW_001811250.1	-	409333	409279
Aae-1-3634	NW_001809901.1	-	1322964	1322912
Aae-1-3635	NW_001810656.1	+	1032550	1032600
Aae-1-3636	NW_001810688.1	-	199661	199609
Aae-1-3637	NW_001810242.1	+	526047	526100
Aae-1-3638	NW_001810714.1	-	2539509	2539456
Aae-1-3639	NW_001811003.1	+	269536	269590
Aae-1-3640	NW_001811051.1	-	444150	444096
Aae-1-3641	NW_001811158.1	+	916317	916372
Aae-1-3642	NW_001811201.1	-	383483	383429
Aae-1-3643	NW_001811596.1	+	1010364	1010416
Aae-1-3644	NW_001811352.1	+	664953	665007
Aae-1-3645	NW_001811318.1	+	2307072	2307126
Aae-1-3646	NW_001811485.1	+	2584244	2584298
Aae-1-3647	NW_001811443.1	+	267023	267077
Aae-1-3648	NW_001811016.1	+	19483	19537
Aae-1-3649	NW_001811074.1	-	3480340	3480286
Aae-1-3650	NW_001811067.1	-	749831	749777
Aae-1-3651	NW_001811022.1	-	220795	220741
Aae-1-3652	NW_001810997.1	+	21439	21493
Aae-1-3653	NW_001810984.1	+	3100509	3100563
Aae-1-3654	NW_001810852.1	+	57449	57503
Aae-1-3655	NW_001810656.1	+	1863632	1863686
Aae-1-3656	NW_001810242.1	+	4256694	4256748
Aae-1-3657	NW_001811134.1	+	1074468	1074522
Aae-1-3658	NW_001810069.1	+	76099	76153
Aae-1-3659	NW_001810864.1	-	5058	5004
Aae-1-3660	NW_001810067.1	-	937294	937240
Aae-1-3661	NW_001809801.1	+	3651648	3651702
Aae-1-3662	NW_001811398.1	+	589609	589675
Aae-1-3663	NW_001811398.1	-	584866	584800
Aae-1-3664	NW_001811027.1	+	51861	51915
Aae-1-3665	NW_001811306.1	-	505201	505147
Aae-1-3666	NW_001811150.1	+	64623	64675
Aae-1-3667	NW_001811638.1	+	145728	145782
Aae-1-3668	NW_001811638.1	-	145719	145665
Aae-1-3669	NW_001811629.1	+	2009211	2009265
Aae-1-3670	NW_001811440.1	+	1410055	1410109
Aae-1-3671	NW_001811142.1	+	1176644	1176698
Aae-1-3672	NW_001811284.1	+	785037	785091
Aae-1-3673	NW_001811250.1	-	105364	105310

Aae-1-3674	NW_001811329.1	+	1719503	1719557
Aae-1-3675	NW_001837310.1	-	5710	5656
Aae-1-3676	NW_001811213.1	+	397542	397596
Aae-1-3677	NW_001810837.1	+	1784038	1784092
Aae-1-3678	NW_001811351.1	-	4993119	4993065
Aae-1-3679	NW_001811185.1	+	39466	39520
Aae-1-3680	NW_001811183.1	+	23838	23892
Aae-1-3681	NW_001811152.1	-	2562249	2562195
Aae-1-3682	NW_001811145.1	+	70129	70183
Aae-1-3683	NW_001811129.1	+	2451179	2451233
Aae-1-3684	NW_001811118.1	-	2209523	2209469
Aae-1-3685	NW_001811117.1	-	282149	282095
Aae-1-3686	NW_001811107.1	+	1682271	1682325
Aae-1-3687	NW_001811090.1	+	698847	698901
Aae-1-3688	NW_001811055.1	+	732879	732933
Aae-1-3689	NW_001811005.1	+	1224781	1224835
Aae-1-3690	NW_001810981.1	+	1562834	1562888
Aae-1-3691	NW_001810958.1	-	1098396	1098342
Aae-1-3692	NW_001810912.1	-	1378285	1378231
Aae-1-3693	NW_001810893.1	+	1351430	1351484
Aae-1-3694	NW_001810837.1	-	2490434	2490380
Aae-1-3695	NW_001810795.1	+	1584630	1584684
Aae-1-3696	NW_001810637.1	-	14689	14635
Aae-1-3697	NW_001810459.1	+	485846	485900
Aae-1-3698	NW_001810381.1	+	247145	247199
Aae-1-3699	NW_001810361.1	-	1568598	1568544
Aae-1-3700	NW_001810200.1	-	1108085	1108031
Aae-1-3701	NW_001810191.1	+	75848	75902
Aae-1-3702	NW_001810178.1	+	1732437	1732491
Aae-1-3703	NW_001810143.1	-	88304	88250
Aae-1-3704	NW_001810132.1	+	126597	126651
Aae-1-3705	NW_001810022.1	-	2384514	2384459
Aae-1-3706	NW_001810003.1	+	54580	54634
Aae-1-3707	NW_001809844.1	+	275699	275753
Aae-1-3708	NW_001809836.1	+	1699432	1699486
Aae-1-3709	NW_001811393.1	+	158435	158489
Aae-1-3710	NW_001811672.1	+	1201	1255
Aae-1-3711	NW_001811292.1	-	189229	189179
Aae-1-3712	NW_001811103.1	+	25263	25317
Aae-1-3713	NW_001811085.1	-	3355210	3355156
Aae-1-3714	NW_001811046.1	+	523063	523117
Aae-1-3715	NW_001811031.1	-	681494	681439
Aae-1-3716	NW_001811005.1	-	1358116	1358062
Aae-1-3717	NW_001810898.1	+	742033	742087
Aae-1-3718	NW_001810782.1	-	230306	230254
Aae-1-3719	NW_001810459.1	-	2264459	2264405
Aae-1-3720	NW_001811703.1	-	272292	272237
Aae-1-3721	NW_001811518.1	-	1333532	1333478
Aae-1-3722	NW_001811684.1	+	1918801	1918855
Aae-1-3723	NW_001811329.1	+	2492624	2492678
Aae-1-3724	NW_001811203.1	-	377868	377815
Aae-1-3725	NW_001811222.1	+	605060	605113

Aae-1-3726	NW_001810527.1	-	1498434	1498380
Aae-1-3727	NW_001810490.1	-	1698000	1697946
Aae-1-3728	NW_001810078.1	-	367176	367122
Aae-1-3729	NW_001810000.1	-	1700666	1700612
Aae-1-3730	NW_001810953.1	+	102976	103028
Aae-1-3731	NW_001811040.1	+	1167889	1167943
Aae-1-3732	NW_001811108.1	-	193331	193278
Aae-1-3733	NW_001811463.1	+	1591435	1591489
Aae-1-3734	NW_001809964.1	+	18577	18631
Aae-1-3735	NW_001811077.1	-	384720	384665
Aae-1-3736	NW_001809838.1	-	57238	57184
Aae-1-3737	NW_001811129.1	+	190367	190421
Aae-1-3738	NW_001810708.1	+	1477887	1477941
Aae-1-3739	NW_001811776.1	-	295248	295194
Aae-1-3740	NW_001811578.1	-	331811	331757
Aae-1-3741	NW_001811440.1	+	1532818	1532872
Aae-1-3742	NW_001811441.1	+	481104	481158
Aae-1-3743	NW_001811307.1	+	188194	188248
Aae-1-3744	NW_001811363.1	+	1384819	1384873
Aae-1-3745	NW_001811220.1	+	556508	556563
Aae-1-3746	NW_001810939.1	-	472089	472035
Aae-1-3747	NW_001811163.1	+	1764620	1764674
Aae-1-3748	NW_001811088.1	-	370753	370699
Aae-1-3749	NW_001811017.1	-	354433	354379
Aae-1-3750	NW_001809816.1	+	184838	184892
Aae-1-3751	NW_001810998.1	-	1424202	1424148
Aae-1-3752	NW_001811071.1	+	891072	891126
Aae-1-3753	NW_001810982.1	-	972386	972332
Aae-1-3754	NW_001810111.1	-	1888056	1888002
Aae-1-3755	NW_001810447.1	-	1765460	1765406
Aae-1-3756	NW_001810912.1	+	2340556	2340610
Aae-1-3757	NW_001811141.1	+	1366090	1366144
Aae-1-3758	NW_001811778.1	-	29818	29764
Aae-1-3759	NW_001810971.1	-	1275267	1275213
Aae-1-3760	NW_001810855.1	+	107758	107812
Aae-1-3761	NW_001811618.1	-	1181119	1181065
Aae-1-3762	NW_001811196.1	+	2728458	2728512
Aae-1-3763	NW_001811009.1	-	364902	364841
Aae-1-3764	NW_001811329.1	-	2234170	2234116
Aae-1-3765	NW_001811429.1	-	2475458	2475404
Aae-1-3766	NW_001811277.1	-	564232	564178
Aae-1-3767	NW_001811021.1	-	1309513	1309459
Aae-1-3768	NW_001810980.1	-	430556	430502
Aae-1-3769	NW_001810913.1	+	401848	401902
Aae-1-3770	NW_001810947.1	+	1047797	1047851
Aae-1-3771	NW_001811124.1	+	156031	156085
Aae-1-3772	NW_001811163.1	+	1139886	1139940
Aae-1-3773	NW_001811185.1	-	2392492	2392438
Aae-1-3774	NW_001811396.1	-	2501420	2501366
Aae-1-3775	NW_001811065.1	+	828862	828916
Aae-1-3776	NW_001810816.1	+	1596052	1596106
Aae-1-3777	NW_001810909.1	+	1154687	1154741

Aae-1-3778	NW_001811498.1	+	2258	2312
Aae-1-3779	NW_001811096.1	+	1312346	1312400
Aae-1-3780	NW_001809945.1	-	200986	200932
Aae-1-3781	NW_001810078.1	+	367326	367380
Aae-1-3782	NW_001810242.1	+	346696	346750
Aae-1-3783	NW_001810782.1	+	667771	667825
Aae-1-3784	NW_001811456.1	+	320577	320631
Aae-1-3785	NW_001809857.1	+	458123	458177
Aae-1-3786	NW_001811129.1	-	3057141	3057087
Aae-1-3787	NW_001809912.1	-	2618072	2618018
Aae-1-3788	NW_001811585.1	+	828783	828837
Aae-1-3789	NW_001811360.1	+	531251	531305
Aae-1-3790	NW_001810350.1	-	3826093	3826039
Aae-1-3791	NW_001810927.1	+	405694	405748
Aae-1-3792	NW_001811055.1	-	1015334	1015280
Aae-1-3793	NW_001811743.1	-	121367	121313
Aae-1-3794	NW_001811573.1	-	3199851	3199797
Aae-1-3795	NW_001811739.1	-	274476	274422
Aae-1-3796	NW_001811739.1	-	2344734	2344680
Aae-1-3797	NW_001811662.1	-	1960028	1959974
Aae-1-3798	NW_001810964.1	+	741868	741922
Aae-1-3799	NW_001811448.1	-	408546	408492
Aae-1-3800	NW_001811613.1	+	35976	36030
Aae-1-3801	NW_001811448.1	-	122388	122334
Aae-1-3802	NW_001811251.1	-	28642	28588
Aae-1-3803	NW_001811096.1	-	2726545	2726491
Aae-1-3804	NW_001810404.1	+	47262	47316
Aae-1-3805	NW_001809801.1	-	1476087	1476033
Aae-1-3806	NW_001810242.1	-	1493338	1493284
Aae-1-3807	NW_001810459.1	-	1553846	1553792
Aae-1-3808	NW_001810984.1	-	717599	717545
Aae-1-3809	NW_001811122.1	-	457009	456955
Aae-1-3810	NW_001811274.1	-	1191066	1191012
Aae-1-3811	NW_001811562.1	-	48373	48319
Aae-1-3812	NW_001810006.1	+	21455	21509
Aae-1-3813	NW_001809825.1	-	2275293	2275239
Aae-1-3814	NW_001811314.1	+	513818	513872
Aae-1-3815	NW_001810001.1	-	102824	102770
Aae-1-3816	NW_001810178.1	-	1901254	1901200
Aae-1-3817	NW_001810133.1	-	391953	391899
Aae-1-3818	NW_001810459.1	-	2242624	2242570
Aae-1-3819	NW_001811240.1	+	1478733	1478787
Aae-1-3820	NW_001811066.1	-	928168	928114
Aae-1-3821	NW_001810970.1	-	3576129	3576075
Aae-1-3822	NW_001811029.1	+	326174	326228
Aae-1-3823	NW_001811164.1	+	81893	81947
Aae-1-3824	NW_001811533.1	-	341080	341026
Aae-1-3825	NW_001810373.1	+	53437	53491
Aae-1-3826	NW_001810847.1	-	399512	399458
Aae-1-3827	NW_001811433.1	-	126959	126905
Aae-1-3828	NW_001811576.1	+	137817	137871
Aae-1-3829	NW_001811562.1	+	1808573	1808627

Aae-1-3830	NW_001811535.1	+	346512	346566
Aae-1-3831	NW_001811433.1	-	217259	217205
Aae-1-3832	NW_001811340.1	-	2677611	2677557
Aae-1-3833	NW_001811226.1	-	317246	317192
Aae-1-3834	NW_001811201.1	-	642194	642140
Aae-1-3835	NW_001811196.1	-	3178952	3178898
Aae-1-3836	NW_001811179.1	-	320601	320547
Aae-1-3837	NW_001811163.1	-	3040365	3040311
Aae-1-3838	NW_001811225.1	+	435064	435118
Aae-1-3839	NW_001811150.1	+	661349	661403
Aae-1-3840	NW_001811149.1	+	164262	164316
Aae-1-3841	NW_001811120.1	+	401141	401195
Aae-1-3842	NW_001811108.1	+	1208634	1208688
Aae-1-3843	NW_001811096.1	+	3595024	3595078
Aae-1-3844	NW_001811080.1	-	1126258	1126204
Aae-1-3845	NW_001810991.1	+	575910	575964
Aae-1-3846	NW_001811320.1	-	775081	775027
Aae-1-3847	NW_001811729.1	+	295542	295596
Aae-1-3848	NW_001810967.1	-	715145	715091
Aae-1-3849	NW_001810880.1	+	283687	283741
Aae-1-3850	NW_001810783.1	+	1243958	1244012
Aae-1-3851	NW_001811193.1	-	601742	601688
Aae-1-3852	NW_001811318.1	+	468277	468331
Aae-1-3853	NW_001811007.1	-	2803667	2803613
Aae-1-3854	NW_001811136.1	-	982180	982126
Aae-1-3855	NW_001810964.1	-	1473956	1473902
Aae-1-3856	NW_001810958.1	-	590372	590318
Aae-1-3857	NW_001810901.1	-	1081072	1081018
Aae-1-3858	NW_001811618.1	+	1945427	1945481
Aae-1-3859	NW_001809912.1	+	1477042	1477096
Aae-1-3860	NW_001811051.1	+	921366	921420
Aae-1-3861	NW_001811491.1	+	248767	248821
Aae-1-3862	NW_001810381.1	+	1862371	1862425
Aae-1-3863	NW_001811030.1	+	2077966	2078020
Aae-1-3864	NW_001811081.1	-	884109	884055
Aae-1-3865	NW_001810023.1	-	415738	415684
Aae-1-3866	NW_001809912.1	-	2221573	2221519
Aae-1-3867	NW_001811407.1	-	1196199	1196145
Aae-1-3868	NW_001809935.1	+	635739	635793
Aae-1-3869	NW_001809957.1	+	1844623	1844677
Aae-1-3870	NW_001810756.1	-	1506817	1506763
Aae-1-3871	NW_001810134.1	+	472424	472478
Aae-1-3872	NW_001810000.1	-	417000	416946
Aae-1-3873	NW_001810339.1	-	1665183	1665129
Aae-1-3874	NW_001810417.1	-	9799	9745
Aae-1-3875	NW_001811056.1	+	759259	759313
Aae-1-3876	NW_001811233.1	-	4991	4937
Aae-1-3877	NW_001811462.1	-	4498132	4498078
Aae-1-3878	NW_001810134.1	+	1760240	1760294
Aae-1-3879	NW_001810769.1	-	1521292	1521238
Aae-1-3880	NW_001811597.1	+	161187	161241
Aae-1-3881	NW_001810714.1	-	3127151	3127097

Aae-1-3882	NW_001811060.1	+	155540	155594
Aae-1-3883	NW_001810490.1	+	282237	282291
Aae-1-3884	NW_001811170.1	+	949966	950020
Aae-1-3885	NW_001811351.1	+	106080	106134
Aae-1-3886	NW_001810067.1	+	2180912	2180966
Aae-1-3887	NW_001811126.1	+	37061	37115
Aae-1-3888	NW_001811267.1	-	286716	286662
Aae-1-3889	NW_001811568.1	-	147904	147850
Aae-1-3890	NW_001811064.1	-	743879	743825
Aae-1-3891	NW_001810371.1	-	892334	892280
Aae-1-3892	NW_001810022.1	+	913015	913069
Aae-1-3893	NW_001810018.1	+	13890	13944
Aae-1-3894	NW_001809903.1	-	37585	37531
Aae-1-3895	NW_001810503.1	-	21630	21576
Aae-1-3896	NW_001810607.1	-	710961	710907
Aae-1-3897	NW_001811621.1	-	68133	68079
Aae-1-3898	NW_001809854.1	-	33897	33843
Aae-1-3899	NW_001811626.1	+	81447	81501
Aae-1-3900	NW_001811591.1	-	98760	98703
Aae-1-3901	NW_001811540.1	+	752149	752203
Aae-1-3902	NW_001811438.1	-	173254	173200
Aae-1-3903	NW_001811201.1	-	724533	724479
Aae-1-3904	NW_001811166.1	-	855665	855611
Aae-1-3905	NW_001811063.1	-	108961	108911
Aae-1-3906	NW_001810023.1	+	465495	465549
Aae-1-3907	NW_001809801.1	-	3612625	3612571
Aae-1-3908	NW_001809821.1	+	33362	33416
Aae-1-3909	NW_001809822.1	+	236175	236229
Aae-1-3910	NW_001809825.1	+	1559492	1559546
Aae-1-3911	NW_001809825.1	+	2404025	2404079
Aae-1-3912	NW_001809863.1	+	253574	253628
Aae-1-3913	NW_001809868.1	-	1184907	1184853
Aae-1-3914	NW_001809878.1	+	257179	257233
Aae-1-3915	NW_001809879.1	-	1208376	1208322
Aae-1-3916	NW_001809890.1	+	214984	215038
Aae-1-3917	NW_001809912.1	+	2410051	2410105
Aae-1-3918	NW_001809912.1	-	2407647	2407593
Aae-1-3919	NW_001809912.1	-	1717610	1717556
Aae-1-3920	NW_001809928.1	+	190134	190188
Aae-1-3921	NW_001809931.1	-	68320	68266
Aae-1-3922	NW_001809935.1	+	2516906	2516960
Aae-1-3923	NW_001809946.1	+	259565	259619
Aae-1-3924	NW_001809947.1	+	219882	219936
Aae-1-3925	NW_001809947.1	-	4713	4659
Aae-1-3926	NW_001809957.1	+	762796	762850
Aae-1-3927	NW_001809957.1	-	2018075	2018021
Aae-1-3928	NW_001809981.1	-	125447	125393
Aae-1-3929	NW_001809988.1	-	184803	184749
Aae-1-3930	NW_001810022.1	-	4158558	4158504
Aae-1-3931	NW_001810056.1	-	1949917	1949863
Aae-1-3932	NW_001810099.1	+	32060	32114
Aae-1-3933	NW_001810100.1	+	1616838	1616892

Aae-1-3934	NW_001810128.1	+	37425	37479
Aae-1-3935	NW_001810128.1	-	36873	36819
Aae-1-3936	NW_001810134.1	+	261112	261166
Aae-1-3937	NW_001810221.1	+	2163903	2163957
Aae-1-3938	NW_001810231.1	-	1431022	1430968
Aae-1-3939	NW_001810275.1	+	1627111	1627165
Aae-1-3940	NW_001810275.1	-	1624552	1624498
Aae-1-3941	NW_001810296.1	-	1868495	1868441
Aae-1-3942	NW_001810296.1	-	593514	593460
Aae-1-3943	NW_001810392.1	-	1180016	1179962
Aae-1-3944	NW_001810414.1	+	304278	304332
Aae-1-3945	NW_001810430.1	+	32579	32633
Aae-1-3946	NW_001810436.1	+	1661525	1661579
Aae-1-3947	NW_001810436.1	-	1660106	1660052
Aae-1-3948	NW_001810447.1	+	389206	389260
Aae-1-3949	NW_001810469.1	-	2055892	2055838
Aae-1-3950	NW_001810475.1	-	57327	57273
Aae-1-3951	NW_001810499.1	+	225888	225942
Aae-1-3952	NW_001810519.1	-	570914	570860
Aae-1-3953	NW_001810527.1	+	471565	471619
Aae-1-3954	NW_001810565.1	+	1677938	1677992
Aae-1-3955	NW_001810565.1	+	1985375	1985429
Aae-1-3956	NW_001810592.1	+	1208799	1208853
Aae-1-3957	NW_001810592.1	-	1203111	1203057
Aae-1-3958	NW_001810607.1	-	733647	733593
Aae-1-3959	NW_001810607.1	-	698657	698603
Aae-1-3960	NW_001810651.1	+	15015	15069
Aae-1-3961	NW_001810688.1	-	1734466	1734412
Aae-1-3962	NW_001810714.1	-	212431	212377
Aae-1-3963	NW_001810714.1	-	54509	54455
Aae-1-3964	NW_001810728.1	+	433025	433079
Aae-1-3965	NW_001810736.1	-	697116	697062
Aae-1-3966	NW_001810744.1	-	1222067	1222013
Aae-1-3967	NW_001810763.1	+	304011	304065
Aae-1-3968	NW_001810778.1	-	34988	34934
Aae-1-3969	NW_001810778.1	-	30822	30768
Aae-1-3970	NW_001810791.1	-	1556895	1556841
Aae-1-3971	NW_001810795.1	+	812999	813053
Aae-1-3972	NW_001810800.1	-	1320459	1320405
Aae-1-3973	NW_001810837.1	+	3873128	3873182
Aae-1-3974	NW_001810837.1	+	3915539	3915593
Aae-1-3975	NW_001810838.1	+	833022	833076
Aae-1-3976	NW_001810844.1	+	179539	179593
Aae-1-3977	NW_001810852.1	+	1049084	1049138
Aae-1-3978	NW_001810855.1	+	972488	972542
Aae-1-3979	NW_001810868.1	-	275150	275096
Aae-1-3980	NW_001810880.1	+	398744	398798
Aae-1-3981	NW_001810880.1	+	400899	400953
Aae-1-3982	NW_001810880.1	-	2025763	2025709
Aae-1-3983	NW_001810893.1	-	1238299	1238245
Aae-1-3984	NW_001810909.1	+	1724250	1724304
Aae-1-3985	NW_001810912.1	-	2018331	2018277

Aae-1-3986	NW_001810912.1	-	1886438	1886384
Aae-1-3987	NW_001810923.1	-	495968	495914
Aae-1-3988	NW_001810939.1	+	1473998	1474052
Aae-1-3989	NW_001810939.1	-	1386986	1386932
Aae-1-3990	NW_001810945.1	+	131831	131885
Aae-1-3991	NW_001810945.1	-	573893	573839
Aae-1-3992	NW_001810950.1	+	537075	537129
Aae-1-3993	NW_001810952.1	-	487572	487518
Aae-1-3994	NW_001810955.1	+	2552822	2552876
Aae-1-3995	NW_001810959.1	+	832443	832497
Aae-1-3996	NW_001810960.1	+	717026	717080
Aae-1-3997	NW_001810962.1	+	1282955	1283009
Aae-1-3998	NW_001810964.1	-	942367	942313
Aae-1-3999	NW_001810970.1	+	2681054	2681108
Aae-1-4000	NW_001810970.1	-	2183712	2183658
Aae-1-4001	NW_001810975.1	-	190628	190574
Aae-1-4002	NW_001810984.1	-	441077	441023
Aae-1-4003	NW_001810993.1	+	1499537	1499591
Aae-1-4004	NW_001810994.1	-	1280086	1280032
Aae-1-4005	NW_001810995.1	-	426457	426403
Aae-1-4006	NW_001810998.1	-	1379695	1379641
Aae-1-4007	NW_001811006.1	+	1297330	1297384
Aae-1-4008	NW_001811007.1	-	3221806	3221752
Aae-1-4009	NW_001811009.1	+	128449	128503
Aae-1-4010	NW_001811019.1	-	1032606	1032552
Aae-1-4011	NW_001811020.1	-	119306	119252
Aae-1-4012	NW_001811027.1	+	44102	44156
Aae-1-4013	NW_001811027.1	-	513782	513728
Aae-1-4014	NW_001811030.1	-	3047603	3047549
Aae-1-4015	NW_001811031.1	-	138178	138124
Aae-1-4016	NW_001811034.1	-	151691	151637
Aae-1-4017	NW_001811038.1	+	1403739	1403793
Aae-1-4018	NW_001811038.1	-	1400815	1400761
Aae-1-4019	NW_001811041.1	+	1708722	1708776
Aae-1-4020	NW_001811041.1	+	3382459	3382513
Aae-1-4021	NW_001811046.1	+	378663	378717
Aae-1-4022	NW_001811053.1	+	745273	745327
Aae-1-4023	NW_001811054.1	+	997759	997813
Aae-1-4024	NW_001811054.1	-	986482	986428
Aae-1-4025	NW_001811058.1	+	705760	705814
Aae-1-4026	NW_001811059.1	+	105804	105858
Aae-1-4027	NW_001811059.1	+	882394	882448
Aae-1-4028	NW_001811059.1	-	881598	881544
Aae-1-4029	NW_001811060.1	-	642712	642658
Aae-1-4030	NW_001811061.1	-	65909	65855
Aae-1-4031	NW_001811062.1	-	673916	673862
Aae-1-4032	NW_001811063.1	-	513581	513527
Aae-1-4033	NW_001811064.1	+	1176265	1176319
Aae-1-4034	NW_001811064.1	-	1175335	1175281
Aae-1-4035	NW_001811071.1	+	219233	219287
Aae-1-4036	NW_001811071.1	-	891057	891003
Aae-1-4037	NW_001811079.1	+	108556	108610

Aae-1-4038	NW_001811081.1	+	617844	617898
Aae-1-4039	NW_001811082.1	-	1141966	1141912
Aae-1-4040	NW_001811085.1	+	499020	499074
Aae-1-4041	NW_001811088.1	+	372580	372634
Aae-1-4042	NW_001811090.1	-	102621	102567
Aae-1-4043	NW_001811092.1	+	326188	326242
Aae-1-4044	NW_001811095.1	+	146195	146249
Aae-1-4045	NW_001811098.1	+	694024	694078
Aae-1-4046	NW_001811100.1	+	912433	912487
Aae-1-4047	NW_001811107.1	+	2872095	2872149
Aae-1-4048	NW_001811107.1	-	592795	592741
Aae-1-4049	NW_001811108.1	+	128300	128354
Aae-1-4050	NW_001811111.1	-	90512	90458
Aae-1-4051	NW_001811112.1	+	688166	688220
Aae-1-4052	NW_001811118.1	-	3327876	3327822
Aae-1-4053	NW_001811125.1	+	443689	443743
Aae-1-4054	NW_001811127.1	+	708886	708940
Aae-1-4055	NW_001811129.1	+	4039687	4039741
Aae-1-4056	NW_001811133.1	+	795120	795174
Aae-1-4057	NW_001811135.1	+	594850	594904
Aae-1-4058	NW_001811135.1	+	942809	942863
Aae-1-4059	NW_001811141.1	+	2898714	2898768
Aae-1-4060	NW_001811141.1	-	2895351	2895297
Aae-1-4061	NW_001811142.1	+	1179452	1179506
Aae-1-4062	NW_001811142.1	-	1175105	1175051
Aae-1-4063	NW_001811143.1	-	284636	284582
Aae-1-4064	NW_001811150.1	+	352893	352947
Aae-1-4065	NW_001811152.1	-	3065194	3065140
Aae-1-4066	NW_001811159.1	+	622858	622912
Aae-1-4067	NW_001811163.1	+	3043066	3043120
Aae-1-4068	NW_001811174.1	-	675780	675726
Aae-1-4069	NW_001811179.1	+	576782	576836
Aae-1-4070	NW_001811179.1	+	982417	982471
Aae-1-4071	NW_001811179.1	-	980562	980508
Aae-1-4072	NW_001811180.1	-	338885	338831
Aae-1-4073	NW_001811183.1	+	527705	527759
Aae-1-4074	NW_001811185.1	-	2418281	2418227
Aae-1-4075	NW_001811191.1	+	115897	115951
Aae-1-4076	NW_001811191.1	-	722470	722416
Aae-1-4077	NW_001811194.1	+	273755	273809
Aae-1-4078	NW_001811195.1	+	191523	191577
Aae-1-4079	NW_001811195.1	-	888142	888088
Aae-1-4080	NW_001811195.1	-	207603	207549
Aae-1-4081	NW_001811197.1	-	940132	940078
Aae-1-4082	NW_001811200.1	+	716018	716072
Aae-1-4083	NW_001811207.1	+	610066	610120
Aae-1-4084	NW_001811207.1	-	608689	608635
Aae-1-4085	NW_001811212.1	+	858581	858635
Aae-1-4086	NW_001811229.1	+	2471561	2471615
Aae-1-4087	NW_001811229.1	-	2466924	2466870
Aae-1-4088	NW_001811252.1	+	973208	973262
Aae-1-4089	NW_001811271.1	+	73116	73170

Aae-1-4090	NW_001811271.1	-	458147	458093
Aae-1-4091	NW_001811273.1	+	107013	107067
Aae-1-4092	NW_001811274.1	+	3078214	3078268
Aae-1-4093	NW_001811288.1	-	91765	91711
Aae-1-4094	NW_001811296.1	+	142976	143030
Aae-1-4095	NW_001811296.1	-	2899250	2899196
Aae-1-4096	NW_001811296.1	-	2667469	2667415
Aae-1-4097	NW_001811301.1	+	169134	169188
Aae-1-4098	NW_001811301.1	-	167519	167465
Aae-1-4099	NW_001811318.1	+	2296121	2296175
Aae-1-4100	NW_001811321.1	+	558001	558055
Aae-1-4101	NW_001811321.1	-	554413	554359
Aae-1-4102	NW_001811329.1	+	393160	393214
Aae-1-4103	NW_001811329.1	+	1463559	1463613
Aae-1-4104	NW_001811329.1	-	1462583	1462529
Aae-1-4105	NW_001811340.1	+	2827475	2827529
Aae-1-4106	NW_001811341.1	-	242077	242023
Aae-1-4107	NW_001811346.1	-	82681	82627
Aae-1-4108	NW_001811347.1	-	366449	366395
Aae-1-4109	NW_001811350.1	+	332755	332809
Aae-1-4110	NW_001811350.1	+	335798	335852
Aae-1-4111	NW_001811352.1	-	2181350	2181296
Aae-1-4112	NW_001811362.1	+	194874	194928
Aae-1-4113	NW_001811363.1	-	2777405	2777351
Aae-1-4114	NW_001811374.1	+	722111	722165
Aae-1-4115	NW_001811376.1	-	108403	108349
Aae-1-4116	NW_001811392.1	+	463802	463856
Aae-1-4117	NW_001811398.1	+	585100	585154
Aae-1-4118	NW_001811411.1	+	544875	544929
Aae-1-4119	NW_001811422.1	-	34820	34766
Aae-1-4120	NW_001811431.1	+	404451	404505
Aae-1-4121	NW_001811432.1	-	381184	381130
Aae-1-4122	NW_001811437.1	+	368357	368411
Aae-1-4123	NW_001811440.1	+	2763604	2763658
Aae-1-4124	NW_001811440.1	-	2433993	2433939
Aae-1-4125	NW_001811441.1	-	389360	389306
Aae-1-4126	NW_001811449.1	+	375250	375304
Aae-1-4127	NW_001811451.1	+	1789634	1789688
Aae-1-4128	NW_001811455.1	-	98184	98130
Aae-1-4129	NW_001811462.1	+	281737	281791
Aae-1-4130	NW_001811462.1	+	997123	997177
Aae-1-4131	NW_001811462.1	-	994360	994306
Aae-1-4132	NW_001811474.1	-	2571752	2571698
Aae-1-4133	NW_001811476.1	+	266189	266243
Aae-1-4134	NW_001811481.1	-	267111	267057
Aae-1-4135	NW_001811496.1	+	866359	866413
Aae-1-4136	NW_001811496.1	-	863161	863107
Aae-1-4137	NW_001811507.1	+	2126054	2126108
Aae-1-4138	NW_001811511.1	-	67859	67805
Aae-1-4139	NW_001811514.1	-	317927	317873
Aae-1-4140	NW_001811522.1	+	265200	265254
Aae-1-4141	NW_001811527.1	+	341245	341299

Aae-1-4142	NW_001811529.1	+	215575	215629
Aae-1-4143	NW_001811529.1	+	2387795	2387849
Aae-1-4144	NW_001811529.1	-	2387163	2387109
Aae-1-4145	NW_001811529.1	-	2384900	2384846
Aae-1-4146	NW_001811533.1	-	444672	444618
Aae-1-4147	NW_001811540.1	+	4521	4575
Aae-1-4148	NW_001811568.1	+	241392	241446
Aae-1-4149	NW_001811574.1	+	452528	452582
Aae-1-4150	NW_001811578.1	+	370371	370425
Aae-1-4151	NW_001811579.1	+	300923	300977
Aae-1-4152	NW_001811580.1	-	384698	384644
Aae-1-4153	NW_001811586.1	+	129503	129557
Aae-1-4154	NW_001811587.1	+	167994	168048
Aae-1-4155	NW_001811594.1	+	192863	192917
Aae-1-4156	NW_001811596.1	+	1695502	1695556
Aae-1-4157	NW_001811598.1	+	321251	321305
Aae-1-4158	NW_001811607.1	-	2283908	2283854
Aae-1-4159	NW_001811623.1	-	173871	173817
Aae-1-4160	NW_001811629.1	+	1691059	1691113
Aae-1-4161	NW_001811661.1	+	360480	360534
Aae-1-4162	NW_001811678.1	+	168995	169049
Aae-1-4163	NW_001811678.1	-	166495	166441
Aae-1-4164	NW_001811713.1	-	351481	351427
Aae-1-4165	NW_001811716.1	-	35997	35943
Aae-1-4166	NW_001811721.1	+	304043	304097
Aae-1-4167	NW_001811731.1	-	202606	202552
Aae-1-4168	NW_001811739.1	+	2345120	2345174
Aae-1-4169	NW_001811750.1	-	1561019	1560965
Aae-1-4170	NW_001811757.1	+	105741	105795
Aae-1-4171	NW_001811761.1	+	2495496	2495550
Aae-1-4172	NW_001811769.1	-	128648	128594
Aae-1-4173	NW_001811783.1	+	1753163	1753217
Aae-1-4174	NW_001811789.1	-	43450	43396
Aae-1-4175	NW_001811790.1	+	4390	4444
Aae-1-4176	NW_001811790.1	+	60699	60753
Aae-1-4177	NW_001835775.1	-	188	134
Aae-1-4178	NW_001836839.1	+	2425	2479
Aae-1-4179	NW_001836839.1	+	7634	7688
Aae-1-4180	NW_001837425.1	-	18457	18403
Aae-1-4181	NW_001837430.1	+	22850	22904
Aae-1-4182	NW_001811163.1	-	49899	49846
Aae-1-4183	NW_001811007.1	+	2804724	2804780
Aae-1-4184	NW_001810990.1	+	1034793	1034847
Aae-1-4185	NW_001810984.1	-	1489446	1489392
Aae-1-4186	NW_001810964.1	-	1166126	1166072
Aae-1-4187	NW_001811061.1	+	226346	226400
Aae-1-4188	NW_001810944.1	-	395997	395943
Aae-1-4189	NW_001810813.1	-	1144360	1144306
Aae-1-4190	NW_001810714.1	+	3065708	3065762
Aae-1-4191	NW_001811462.1	+	1415941	1415995
Aae-1-4192	NW_001810499.1	+	1034931	1034985
Aae-1-4193	NW_001810999.1	-	549627	549573

Aae-1-4194	NW_001811580.1	-	551114	551060
Aae-1-4195	NW_001810351.1	+	1563491	1563545
Aae-1-4196	NW_001811200.1	-	721946	721892
Aae-1-4197	NW_001811296.1	-	239808	239754
Aae-1-4198	NW_001810351.1	+	1174078	1174132
Aae-1-4199	NW_001811355.1	+	346020	346074
Aae-1-4200	NW_001810034.1	+	124028	124082
Aae-1-4201	NW_001811351.1	+	878171	878225
Aae-1-4202	NW_001810022.1	-	4507577	4507523
Aae-1-4203	NW_001810454.1	+	29938	29992
Aae-1-4204	NW_001811062.1	+	1094146	1094200
Aae-1-4205	NW_001811406.1	+	536351	536405
Aae-1-4206	NW_001809952.1	-	211050	210996
Aae-1-4207	NW_001810728.1	+	1680133	1680187
Aae-1-4208	NW_001809933.1	+	44158	44212
Aae-1-4209	NW_001809881.1	-	117036	116982
Aae-1-4210	NW_001809825.1	+	1060787	1060842
Aae-1-4211	NW_001809814.1	+	1633501	1633552
Aae-1-4212	NW_001811507.1	+	331394	331440
Aae-1-4213	NW_001810242.1	+	1340905	1340959
Aae-1-4214	NW_001810243.1	-	1934699	1934645
Aae-1-4215	NW_001810527.1	-	541057	541003
Aae-1-4216	NW_001811019.1	+	2453342	2453396
Aae-1-4217	NW_001811429.1	-	1105840	1105785
Aae-1-4218	NW_001811514.1	+	182796	182850
Aae-1-4219	NW_001811576.1	+	268523	268577
Aae-1-4220	NW_001835677.1	-	5185	5131
Aae-1-4221	NW_001811432.1	-	287040	286986
Aae-1-4222	NW_001811352.1	+	2652642	2652696
Aae-1-4223	NW_001811478.1	-	393516	393462
Aae-1-4224	NW_001811274.1	+	2442197	2442251
Aae-1-4225	NW_001811167.1	-	659555	659501
Aae-1-4226	NW_001811144.1	-	1046663	1046609
Aae-1-4227	NW_001810156.1	-	1384901	1384847
Aae-1-4228	NW_001810800.1	-	1472283	1472229
Aae-1-4229	NW_001810100.1	-	668059	668005
Aae-1-4230	NW_001809957.1	-	518287	518233
Aae-1-4231	NW_001809935.1	+	577595	577649
Aae-1-4232	NW_001810078.1	+	142523	142577
Aae-1-4233	NW_001810156.1	-	1172430	1172376
Aae-1-4234	NW_001811179.1	+	672585	672639
Aae-1-4235	NW_001811478.1	+	9742	9796
Aae-1-4236	NW_001811098.1	-	820650	820596
Aae-1-4237	NW_001811250.1	-	674888	674834
Aae-1-4238	NW_001811190.1	+	425422	425476
Aae-1-4239	NW_001810991.1	-	913326	913272
Aae-1-4240	NW_001809968.1	-	2385538	2385484
Aae-1-4241	NW_001809900.1	-	83882	83828
Aae-1-4242	NW_001809979.1	-	718008	717954
Aae-1-4243	NW_001810383.1	+	33706	33760
Aae-1-4244	NW_001811437.1	-	362457	362403
Aae-1-4245	NW_001810981.1	+	881141	881195

Aae-1-4246	NW_001810637.1	+	1481981	1482035
Aae-1-4247	NW_001809912.1	+	2336756	2336811
Aae-1-4248	NW_001811276.1	+	704538	704592
Aae-1-4249	NW_001811757.1	+	105680	105735
Aae-1-4250	NW_001810078.1	+	56340	56394
Aae-1-4251	NW_001810307.1	-	1671879	1671825
Aae-1-4252	NW_001810459.1	+	267852	267906
Aae-1-4253	NW_001811076.1	-	454447	454393
Aae-1-4254	NW_001810286.1	+	474095	474149
Aae-1-4255	NW_001811375.1	+	561004	561058
Aae-1-4256	NW_001811381.1	-	92461	92407
Aae-1-4257	NW_001809801.1	-	5101959	5101905
Aae-1-4258	NW_001810162.1	-	18469	18415
Aae-1-4259	NW_001809801.1	-	2559844	2559790
Aae-1-4260	NW_001809968.1	-	1968185	1968137
Aae-1-4261	NW_001811396.1	-	1002447	1002393
Aae-1-4262	NW_001811028.1	+	1164728	1164782
Aae-1-4263	NW_001809802.1	-	3683151	3683097
Aae-1-4264	NW_001809803.1	+	1976327	1976381
Aae-1-4265	NW_001809814.1	+	2099681	2099735
Aae-1-4266	NW_001809957.1	+	1986447	1986501
Aae-1-4267	NW_001809979.1	-	515455	515401
Aae-1-4268	NW_001810072.1	+	57442	57496
Aae-1-4269	NW_001810072.1	+	124128	124182
Aae-1-4270	NW_001810111.1	+	699054	699108
Aae-1-4271	NW_001810155.1	+	64562	64616
Aae-1-4272	NW_001810254.1	+	16461	16515
Aae-1-4273	NW_001810392.1	-	433883	433829
Aae-1-4274	NW_001810414.1	+	111073	111127
Aae-1-4275	NW_001810425.1	+	575043	575097
Aae-1-4276	NW_001810436.1	+	700315	700369
Aae-1-4277	NW_001810508.1	+	674928	674982
Aae-1-4278	NW_001810638.1	-	1670904	1670850
Aae-1-4279	NW_001810721.1	+	956329	956383
Aae-1-4280	NW_001810783.1	-	325343	325289
Aae-1-4281	NW_001810852.1	+	204877	204931
Aae-1-4282	NW_001810966.1	-	1547763	1547709
Aae-1-4283	NW_001810972.1	+	1246081	1246135
Aae-1-4284	NW_001811044.1	+	55719	55773
Aae-1-4285	NW_001811062.1	-	966182	966128
Aae-1-4286	NW_001811069.1	-	325079	325025
Aae-1-4287	NW_001811073.1	+	744282	744336
Aae-1-4288	NW_001811086.1	-	593882	593828
Aae-1-4289	NW_001811096.1	-	2990842	2990788
Aae-1-4290	NW_001811096.1	-	1477831	1477777
Aae-1-4291	NW_001811096.1	-	542018	541964
Aae-1-4292	NW_001811097.1	+	1068931	1068985
Aae-1-4293	NW_001811140.1	-	888280	888226
Aae-1-4294	NW_001811150.1	+	732630	732684
Aae-1-4295	NW_001811163.1	+	2787522	2787576
Aae-1-4296	NW_001811168.1	+	408314	408368
Aae-1-4297	NW_001811241.1	+	654050	654104

Aae-1-4298	NW_001811246.1	+	329484	329538
Aae-1-4299	NW_001811268.1	+	98455	98509
Aae-1-4300	NW_001811299.1	+	477610	477664
Aae-1-4301	NW_001811305.1	+	281164	281218
Aae-1-4302	NW_001811307.1	-	2898935	2898881
Aae-1-4303	NW_001811351.1	+	3685965	3686019
Aae-1-4304	NW_001811429.1	-	959478	959424
Aae-1-4305	NW_001811458.1	+	440415	440469
Aae-1-4306	NW_001811474.1	-	750822	750768
Aae-1-4307	NW_001811474.1	-	264105	264051
Aae-1-4308	NW_001811486.1	-	397373	397319
Aae-1-4309	NW_001811506.1	-	547715	547661
Aae-1-4310	NW_001811593.1	+	314648	314702
Aae-1-4311	NW_001811618.1	-	1679792	1679738
Aae-1-4312	NW_001811629.1	-	1683455	1683401
Aae-1-4313	NW_001811761.1	+	473551	473605
Aae-1-4314	NW_001811023.1	-	560897	560846
Aae-1-4315	NW_001811560.1	-	275330	275276
Aae-1-4316	NW_001811001.1	-	812585	812531
Aae-1-4317	NW_001811240.1	+	4463314	4463368
Aae-1-4318	NW_001811529.1	-	2874035	2873981
Aae-1-4319	NW_001810721.1	-	1829630	1829577
Aae-1-4320	NW_001811640.1	-	1948316	1948266
Aae-1-4321	NW_001837020.1	+	8501	8554
Aae-1-4322	NW_001810971.1	-	510450	510396
Aae-1-4323	NW_001811407.1	-	1302599	1302545
Aae-1-4324	NW_001811350.1	+	515964	516018
Aae-1-4325	NW_001811363.1	+	2952135	2952189
Aae-1-4326	NW_001811397.1	+	505675	505729
Aae-1-4327	NW_001811596.1	+	276873	276927
Aae-1-4328	NW_001811600.1	-	289868	289814
Aae-1-4329	NW_001811222.1	+	287812	287866
Aae-1-4330	NW_001811562.1	-	2750346	2750292
Aae-1-4331	NW_001811554.1	+	6078	6132
Aae-1-4332	NW_001811389.1	+	220202	220256
Aae-1-4333	NW_001811421.1	-	385309	385255
Aae-1-4334	NW_001811458.1	-	482227	482175
Aae-1-4335	NW_001811201.1	-	513661	513607
Aae-1-4336	NW_001810111.1	-	1673262	1673208
Aae-1-4337	NW_001810537.1	-	45570	45516
Aae-1-4338	NW_001811110.1	+	218305	218361
Aae-1-4339	NW_001811634.1	-	266318	266264
Aae-1-4340	NW_001810638.1	+	1501113	1501167
Aae-1-4341	NW_001809989.1	+	1708557	1708608
Aae-1-4342	NW_001811585.1	+	2817768	2817823
Aae-1-4343	NW_001811116.1	-	321143	321090
Aae-1-4344	NW_001810984.1	-	1209788	1209733
Aae-1-4345	NW_001810898.1	-	273778	273720
Mmu-1-27	NT_039353.7	-	81355619	81355532
Mmu-1-28	NW_001030811.1	-	70596485	70596398
Mmu-1-29	AC_000028.1	-	130800358	130800271
Mmu-1-30	NC_000072.5	-	129042657	129042570

Hsa-1-20	NT_030059.13	-	66732480	66732397
Hsa-1-21	NW_924884.1	-	26660004	26659921
Hsa-1-22	NW_001838006.2	+	9076606	9076689
Hsa-1-23	NC_000010.10	-	115928016	115927933
Hsa-1-24	AC_000053.1	-	109657187	109657104
Hsa-1-25	AC_000142.1	-	109555078	109554995
Ptr-1-11	NW_001220742.1	-	11175449	11175366
Ptr-1-12	NC_006477.2	-	114862921	114862838
Mmu-2-11	NW_001125295.1	-	67249	67167
Ssc-1-3	NW_001885543.1	-	1780080	1779996
Ssc-1-4	NC_010456.1	-	121937569	121937485
Eca-1-15	NW_001867386.1	+	17650707	17650791
Eca-1-16	NC_009144.2	+	17650707	17650791
Bta-1-3	NW_001494374.2	-	1546405	1546321
Bta-1-4	NC_007327.3	-	35082886	35082802
Clu-1-1	NW_876285.1	-	25017142	25017058
Clu-1-2	NC_006610.2	-	28017142	28017058
Mdo-1-23	NW_001581859.1	+	47845879	47845964
Mdo-1-24	NC_008801.1	+	96595526	96595611
Tgu-1-1	NW_002198527.1	+	272297	272353
Oan-1-2	NW_001752350.1	-	533196	533145
Mmu-1-31	NT_109315.4	+	1757035	1757086
Mmu-1-32	NW_001030745.1	+	1112700	1112751
Mmu-1-33	AC_000026.1	+	43932398	43932449
Mmu-1-34	NC_000070.5	+	43917960	43918011
Rno-1-21	NW_047712.1	+	460589	460640
Rno-1-22	NW_001084836.1	+	32742106	32742157
Rno-1-23	AC_000073.1	+	56712834	56712885
Rno-1-24	NC_005104.2	+	60371361	60371412
Eca-1-17	NW_001867396.1	+	1321382	1321433
Eca-1-18	NC_009168.2	+	1321382	1321433
Mmu-2-12	NW_001101662.1	-	398175	398123
Mmu-2-13	NC_007872.1	-	41396244	41396192
Ptr-1-13	NW_001240439.1	+	36022474	36022526
Ptr-1-14	NC_006476.2	+	36817700	36817752
Hsa-1-26	NT_008413.18	+	36062692	36062744
Hsa-1-27	NW_924062.1	+	35890777	35890829
Hsa-1-28	NW_001839151.2	-	2712190	2712138
Hsa-1-29	NC_000009.11	+	36072692	36072744
Hsa-1-30	AC_000052.1	+	36006258	36006310
Hsa-1-31	AC_000141.1	+	36026909	36026961
Clu-1-3	NW_876253.1	+	43513672	43513724
Clu-1-4	NC_006593.2	+	55574374	55574426
Bta-1-5	NW_001495460.2	+	159066	159118
Bta-1-6	NC_007306.3	+	63111584	63111636
Gga-1-1	NW_001471637.1	+	2728482	2728534
Gga-1-2	NC_006089.2	+	57147256	57147308
Tgu-1-2	NW_002198271.1	-	8046200	8046148
Tgu-1-3	NC_011465.1	-	75586939	75586887
Mdo-1-25	NW_001581976.1	+	14018006	14018058
Mdo-1-26	NC_008806.1	+	26969510	26969562
Gga-1-3	NW_001471673.1	-	9425216	9425159

Gga-1-4	NC_006090.2	-	107580148	107580091
Oan-1-3	NW_001794184.1	-	6561960	6561876
Sma-1-17	NW_003037895.1	+	61029	61106
Sma-1-18	NW_003037895.1	+	97405	97482
Sma-1-19	NS_000200.1	+	142507231	142507308
Sma-1-20	NS_000200.1	+	142543607	142543684
Sma-1-21	NW_003038037.1	+	1679770	1679826
Sma-1-22	NS_000200.1	+	251835179	251835235
Sma-1-23	NW_003037912.1	+	25666	25743
Sma-1-24	NS_000200.1	+	156837241	156837318
Sma-1-25	NW_003024567.1	-	788	722
Sma-1-26	NW_003037885.1	+	1057664	1057730
Sma-1-27	NS_000200.1	+	129155611	129155677
Sma-1-28	NS_000200.1	-	348866783	348866717
Sma-1-29	NW_003037847.1	-	737945	737897
Sma-1-30	NS_000200.1	-	89389675	89389627
Sma-1-31	NW_003037795.1	+	467984	468032
Sma-1-32	NS_000200.1	+	6858867	6858915
Sma-1-33	NW_003037938.1	-	429352	429297
Sma-1-34	NS_000200.1	-	182466465	182466410
Sma-1-35	NW_003038034.1	-	246149	246082
Sma-1-36	NS_000200.1	-	247187905	247187838
Sma-1-37	NW_003037915.1	-	210060	210011
Sma-1-38	NS_000200.1	-	162110046	162109997
Sma-1-39	NW_003037854.1	-	691787	691738
Sma-1-40	NS_000200.1	-	97596208	97596159
Sma-1-41	NW_003038251.1	-	515986	515937
Sma-1-42	NS_000200.1	-	318847758	318847709
Sma-1-43	NW_003037944.1	+	491847	491924
Sma-1-44	NS_000200.1	+	188713200	188713277
Sma-1-45	NW_003037810.1	+	1198076	1198125
Sma-1-46	NS_000200.1	+	37802598	37802647
Sma-1-47	NW_003037906.1	+	135008	135076
Sma-1-48	NS_000200.1	+	150442787	150442855
Sma-1-49	NW_003037874.1	-	273823	273774
Sma-1-50	NS_000200.1	-	119698773	119698724
Sma-1-51	NW_003037891.1	+	1233848	1233897
Sma-1-52	NS_000200.1	+	139115126	139115175
Sma-1-53	NW_003037809.1	-	1043888	1043840
Sma-1-54	NS_000200.1	-	34708352	34708304
Sma-1-55	NW_003037943.1	-	1178364	1178315
Sma-1-56	NS_000200.1	-	188199897	188199848
Sma-1-57	NW_003037822.1	-	81583	81534
Sma-1-58	NS_000200.1	-	54389589	54389540
Sma-1-59	NW_003037915.1	-	1210512	1210463
Sma-1-60	NS_000200.1	-	163110498	163110449
Sma-1-61	NW_003034555.1	-	9365	9299
Sma-1-62	NS_000200.1	-	327351571	327351505
Sma-1-63	NW_003037809.1	-	1654723	1654674
Sma-1-64	NS_000200.1	-	35319187	35319138
Sma-1-65	NW_003037914.1	+	1195479	1195556
Sma-1-66	NS_000200.1	+	161339729	161339806

Sma-1-67	NW_003037818.1	+	872839	872888
Sma-1-68	NS_000200.1	+	49588246	49588295
Sma-1-69	NW_003038261.1	+	255564	255613
Sma-1-70	NS_000200.1	+	323869215	323869264
Sma-1-71	NW_003038482.1	-	36460	36411
Sma-1-72	NS_000200.1	-	333013911	333013862
Sma-1-73	NW_003038010.1	-	400809	400760
Sma-1-74	NS_000200.1	-	227219032	227218983
Sma-1-75	NW_003038224.1	+	49354	49403
Sma-1-76	NS_000200.1	+	309461082	309461131
Sma-1-77	NW_003037822.1	-	834751	834702
Sma-1-78	NS_000200.1	-	55142757	55142708
Sma-1-79	NW_003038033.1	+	842856	842905
Sma-1-80	NS_000200.1	+	246822858	246822907
Sma-1-81	NW_003037891.1	-	109102	109053
Sma-1-82	NS_000200.1	-	137990380	137990331
Sma-1-83	NW_003038244.1	+	34664	34713
Sma-1-84	NS_000200.1	+	315179869	315179918
Sma-1-85	NW_003038149.1	-	7740	7691
Sma-1-86	NS_000200.1	-	291036353	291036304
Sma-1-87	NW_003026507.1	+	165321	165370
Sma-1-88	NS_000200.1	+	279825472	279825521
Sma-1-89	NW_003037824.1	+	313203	313252
Sma-1-90	NW_003037912.1	+	283210	283259
Sma-1-91	NS_000200.1	+	57244143	57244192
Sma-1-92	NS_000200.1	+	157094785	157094834
Sma-1-93	NW_003037806.1	-	1470978	1470929
Sma-1-94	NW_003038230.1	-	302012	301963
Sma-1-95	NS_000200.1	-	311587583	311587534
Sma-1-96	NS_000200.1	-	29666192	29666143
Sma-1-97	NW_003037836.1	-	750996	750947
Sma-1-98	NS_000200.1	-	76308095	76308046
Sma-1-99	NW_003038147.1	-	269128	269079
Sma-1-100	NS_000200.1	-	289796871	289796822
Sma-1-101	NW_003037893.1	-	34012	33963
Sma-1-102	NS_000200.1	-	141148322	141148273
Sma-1-103	NW_003037838.1	-	536400	536351
Sma-1-104	NS_000200.1	-	78534963	78534914
Sma-1-105	NW_003037809.1	-	2580359	2580310
Sma-1-106	NS_000200.1	-	36244823	36244774
Sma-1-107	NW_003022765.1	-	1972	1923
Sma-1-108	NW_003037938.1	-	770736	770687
Sma-1-109	NS_000200.1	-	368059217	368059168
Sma-1-110	NS_000200.1	-	182807849	182807800
Sma-1-111	NW_003026466.1	+	17305	17354
Sma-1-112	NS_000200.1	+	215284412	215284461
Sma-1-113	NW_003037974.1	+	229488	229537
Sma-1-114	NS_000200.1	+	204904794	204904843
Sma-1-115	NW_003037838.1	+	619123	619172
Sma-1-116	NS_000200.1	+	78617686	78617735
Sma-1-117	NW_003038086.1	+	271283	271332
Sma-1-118	NS_000200.1	+	272519876	272519925

Sma-1-119	NW_003038240.1	+	234883	234932
Sma-1-120	NS_000200.1	+	314373814	314373863
Sma-1-121	NW_003038074.1	-	106320	106271
Sma-1-122	NS_000200.1	-	265997700	265997651
Sma-1-123	NW_003032120.1	-	22643	22594
Sma-1-124	NS_000200.1	-	377847920	377847871
Sma-1-125	NW_003038030.1	-	460189	460140
Sma-1-126	NS_000200.1	-	243596056	243596007
Sma-1-127	NW_003037809.1	+	1574000	1574049
Sma-1-128	NS_000200.1	+	35238464	35238513
Sma-1-129	NW_003037818.1	+	231995	232044
Sma-1-130	NS_000200.1	+	48947402	48947451
Sma-1-131	NW_003037937.1	+	22138	22187
Sma-1-132	NS_000200.1	+	181222047	181222096
Sma-1-133	NW_003037791.1	-	690360	690311
Sma-1-134	NS_000200.1	-	382395788	382395739
Sma-1-135	NW_003037978.1	+	59086	59135
Sma-1-136	NS_000200.1	+	209327096	209327145
Sma-1-137	NW_003038012.1	-	36974	36925
Sma-1-138	NS_000200.1	-	228360913	228360864
Sma-1-139	NW_003035881.1	+	146650	146699
Sma-1-140	NS_000200.1	+	341210855	341210904
Sma-1-141	NW_003022582.1	+	1011	1060
Sma-1-142	NS_000200.1	+	365225464	365225513
Sma-1-143	NW_003038038.1	-	576472	576423
Sma-1-144	NS_000200.1	-	252479865	252479816
Sma-1-145	NW_003038109.1	-	62618	62569
Sma-1-146	NS_000200.1	-	275745296	275745247
Sma-1-147	NW_003038471.1	+	54464	54513
Sma-1-148	NS_000200.1	+	331895378	331895427
Sma-1-149	NW_003037894.1	+	174067	174116
Sma-1-150	NW_003037894.1	-	176261	176212
Sma-1-151	NS_000200.1	+	142070640	142070689
Sma-1-152	NS_000200.1	-	142072834	142072785
Sma-1-153	NW_003037943.1	+	855932	855981
Sma-1-154	NS_000200.1	+	187877465	187877514
Sma-1-155	NW_003037795.1	-	1462071	1462022
Sma-1-156	NS_000200.1	-	7852954	7852905
Sma-1-157	NW_003038517.1	-	259398	259349
Sma-1-158	NS_000200.1	-	341023886	341023837
Sma-1-159	NW_003037818.1	+	832747	832796
Sma-1-160	NS_000200.1	+	49548154	49548203
Sma-1-161	NW_003037944.1	-	769933	769885
Sma-1-162	NS_000200.1	-	188991286	188991238
Sma-1-163	NW_003037833.1	-	667772	667724
Sma-1-164	NS_000200.1	-	72858868	72858820
Sma-1-165	NW_003037858.1	-	70965	70917
Sma-1-166	NS_000200.1	-	100735241	100735193
Sma-1-167	NW_003035382.1	-	877	829
Sma-1-168	NS_000200.1	-	367376881	367376833
Sma-1-169	NW_003037806.1	-	1397509	1397460
Sma-1-170	NS_000200.1	-	29592723	29592674

Sma-1-171	NW_003037852.1	-	1049049	1049000
Sma-1-172	NS_000200.1	-	95440944	95440895
Sma-1-173	NW_003037862.1	+	784838	784887
Sma-1-174	NS_000200.1	+	106436499	106436548
Sma-1-175	NW_003037797.1	+	238608	238657
Sma-1-176	NS_000200.1	+	10666468	10666517
Sma-1-177	NW_003037827.1	-	972088	972039
Sma-1-178	NS_000200.1	-	63594229	63594180
Sma-1-179	NW_003028140.1	-	2294	2245
Sma-1-180	NS_000200.1	-	374137882	374137833
Sma-1-181	NW_003038156.1	+	467465	467514
Sma-1-182	NS_000200.1	+	295199578	295199627
Sma-1-183	NW_003038033.1	-	381204	381155
Sma-1-184	NS_000200.1	-	246361206	246361157
Sma-1-185	NW_003037940.1	+	831177	831226
Sma-1-186	NS_000200.1	+	184573307	184573356
Sma-1-187	NW_003037938.1	-	701411	701362
Sma-1-188	NS_000200.1	-	182738524	182738475
Sma-1-189	NW_003037820.1	-	194078	194029
Sma-1-190	NS_000200.1	-	52467060	52467011
Sma-1-191	NW_003039129.1	-	403955	403906
Sma-1-192	NS_000200.1	-	229358763	229358714
Sma-1-193	NW_003037837.1	-	674530	674481
Sma-1-194	NS_000200.1	-	77480388	77480339
Sma-1-195	NW_003038457.1	+	10365	10414
Sma-1-196	NS_000200.1	+	331092229	331092278
Sma-1-197	NW_003037853.1	+	761422	761471
Sma-1-198	NS_000200.1	+	96253473	96253522
Sma-1-199	NW_003031339.1	+	742	791
Sma-1-200	NW_003026471.1	-	120208	120159
Sma-1-201	NS_000200.1	+	345779720	345779769
Sma-1-202	NS_000200.1	-	222232441	222232392
Sma-1-203	NW_003037911.1	-	286521	286472
Sma-1-204	NS_000200.1	-	156073874	156073825
Sma-1-205	NW_003031319.1	+	48	97
Sma-1-206	NS_000200.1	+	345743695	345743744
Sma-1-207	NW_003037881.1	-	614014	613965
Sma-1-208	NS_000200.1	-	125045805	125045756
Sma-1-209	NW_003037796.1	+	112408	112457
Sma-1-210	NS_000200.1	+	8322052	8322101
Sma-1-211	NW_003037814.1	+	1683197	1683246
Sma-1-212	NS_000200.1	+	44667057	44667106
Sma-1-213	NW_003038011.1	+	184433	184482
Sma-1-214	NS_000200.1	+	227712083	227712132
Sma-1-215	NW_003038030.1	+	381248	381297
Sma-1-216	NS_000200.1	+	243517115	243517164
Sma-1-217	NW_003027564.1	-	551	502
Sma-1-218	NS_000200.1	-	361628937	361628888
Sma-1-219	NW_003032095.1	-	3914	3865
Sma-1-220	NS_000200.1	-	377687260	377687211
Sma-1-221	NW_003037953.1	+	152189	152238
Sma-1-222	NS_000200.1	+	193065240	193065289

Sma-1-223	NW_003037972.1	+	167093	167142
Sma-1-224	NS_000200.1	+	202642797	202642846
Sma-1-225	NW_003037851.1	+	383270	383319
Sma-1-226	NS_000200.1	+	93786580	93786629
Sma-1-227	NW_003038232.1	+	292624	292673
Sma-1-228	NS_000200.1	+	312256415	312256464
Sma-1-229	NW_003037858.1	+	827763	827812
Sma-1-230	NS_000200.1	+	101492039	101492088
Sma-1-231	NW_003035643.1	-	5019	4970
Sma-1-232	NS_000200.1	-	330305454	330305405
Sma-1-233	NW_003037865.1	+	85717	85766
Sma-1-234	NS_000200.1	+	110507636	110507685
Sma-1-235	NW_003037901.1	+	602143	602192
Sma-1-236	NS_000200.1	+	146450604	146450653
Sma-1-237	NW_003037954.1	+	3901	3950
Sma-1-238	NS_000200.1	+	193233523	193233572
Sma-1-239	NW_003037952.1	-	2257	2208
Sma-1-240	NS_000200.1	-	192822390	192822341
Sma-1-241	NW_003037824.1	+	431744	431793
Sma-1-242	NS_000200.1	+	57362684	57362733
Sma-1-243	NW_003039127.1	+	2249263	2249312
Sma-1-244	NS_000200.1	+	115326762	115326811
Sma-1-245	NW_003032120.1	+	90300	90349
Sma-1-246	NS_000200.1	+	377915577	377915626
Sma-1-247	NW_003038254.1	+	151660	151709
Sma-1-248	NS_000200.1	+	320187218	320187267
Sma-1-249	NW_003038251.1	-	97714	97665
Sma-1-250	NS_000200.1	-	318429486	318429437
Sma-1-251	NW_003038462.1	-	50308	50259
Sma-1-252	NS_000200.1	-	331417971	331417922
Sma-1-253	NW_003037979.1	-	672776	672727
Sma-1-254	NS_000200.1	-	210733796	210733747
Sma-1-255	NW_003037939.1	+	466211	466260
Sma-1-256	NS_000200.1	+	183335965	183336014
Sma-1-257	NW_003026615.1	-	109697	109648
Sma-1-258	NS_000200.1	-	317104138	317104089
Sma-1-259	NW_003026597.1	-	36597	36548
Sma-1-260	NS_000200.1	-	305657783	305657734
Sma-1-261	NW_003037815.1	+	474425	474474
Sma-1-262	NS_000200.1	+	45737416	45737465
Sma-1-263	NW_003037872.1	+	234505	234554
Sma-1-264	NS_000200.1	+	118191511	118191560
Sma-1-265	NW_003037794.1	-	1812961	1812912
Sma-1-266	NS_000200.1	-	5992556	5992507
Sma-1-267	NW_003037828.1	-	776515	776466
Sma-1-268	NS_000200.1	-	65866000	65865951
Sma-1-269	NW_003037851.1	-	694079	694030
Sma-1-270	NS_000200.1	-	94097389	94097340
Sma-1-271	NW_003037884.1	-	1240855	1240806
Sma-1-272	NS_000200.1	-	128020520	128020471
Sma-1-273	NW_003037909.1	+	396654	396703
Sma-1-274	NS_000200.1	+	153938303	153938352

Sma-1-275	NW_003038142.1	-	387114	387065
Sma-1-276	NS_000200.1	-	287597550	287597501
Sma-1-277	NW_003038027.1	+	86006	86055
Sma-1-278	NS_000200.1	+	240456720	240456769
Sma-1-279	NW_003036423.1	-	753	704
Sma-1-280	NS_000200.1	-	359945478	359945429
Sma-1-281	NW_003038017.1	-	426124	426066
Sma-1-282	NS_000200.1	-	232192155	232192097
Sma-1-283	NW_003037797.1	+	2150502	2150551
Sma-1-284	NS_000200.1	+	12578362	12578411
Sma-1-285	NW_003038151.1	-	199495	199446
Sma-1-286	NS_000200.1	-	292106738	292106689
Sma-1-287	NW_003037797.1	-	2817884	2817835
Sma-1-288	NS_000200.1	-	13245744	13245695
Sma-1-289	NW_003037909.1	-	52348	52301
Sma-1-290	NS_000200.1	-	153593997	153593950
Sma-1-291	NW_003037891.1	-	2130259	2130211
Sma-1-292	NS_000200.1	-	140011537	140011489
Sma-1-293	NW_003038476.1	-	9097	9049
Sma-1-294	NS_000200.1	-	332397472	332397424
Sma-1-295	NW_003037870.1	-	62943	62878
Sma-1-296	NS_000200.1	-	116936608	116936543
Sma-1-297	NW_003038041.1	-	33873	33825
Sma-1-298	NS_000200.1	-	253701155	253701107
Sma-1-299	NW_003038086.1	+	79205	79254
Sma-1-300	NS_000200.1	+	272327798	272327847
Sma-1-301	NW_003038025.1	+	569355	569403
Sma-1-302	NS_000200.1	+	239427600	239427648
Sma-1-303	NW_003037811.1	+	1137033	1137082
Sma-1-304	NS_000200.1	+	39307275	39307324
Sma-1-305	NW_003026472.1	-	105618	105569
Sma-1-306	NS_000200.1	-	236517610	236517561
Sma-1-307	NW_003037839.1	-	1090703	1090654
Sma-1-308	NS_000200.1	-	80048853	80048804
Sma-1-309	NW_003028385.1	+	203	252
Sma-1-310	NS_000200.1	+	369491653	369491702
Sma-1-311	NW_003038149.1	-	47394	47345
Sma-1-312	NS_000200.1	-	291076007	291075958
Sma-1-313	NW_003038155.1	+	374584	374633
Sma-1-314	NS_000200.1	+	294497702	294497751
Sma-1-315	NW_003038252.1	-	126047	125998
Sma-1-316	NS_000200.1	-	318981635	318981586
Sma-1-317	NW_003022234.1	+	966	1015
Sma-1-318	NW_003037841.1	-	1101172	1101123
Sma-1-319	NS_000200.1	+	360981282	360981331
Sma-1-320	NS_000200.1	-	83712353	83712304
Sma-1-321	NW_003022092.1	+	121	170
Sma-1-322	NS_000200.1	+	355607555	355607604
Sma-1-323	NW_003038003.1	+	184335	184384
Sma-1-324	NS_000200.1	+	223683617	223683666
Sma-1-325	NW_003038161.1	-	469587	469538
Sma-1-326	NS_000200.1	-	297839438	297839389

Sma-1-327	NW_003037930.1	+	899450	899499
Sma-1-328	NS_000200.1	+	176757414	176757463
Sma-1-329	NW_003037907.1	-	93135	93086
Sma-1-330	NS_000200.1	-	151497624	151497575
Sma-1-331	NW_003037860.1	+	219605	219654
Sma-1-332	NS_000200.1	+	103962633	103962682
Sma-1-333	NW_003032160.1	-	42051	42002
Sma-1-334	NS_000200.1	-	381050137	381050088
Sma-1-335	NW_003037846.1	-	284376	284327
Sma-1-336	NS_000200.1	-	87918946	87918897
Sma-1-337	NW_003038508.1	+	49811	49860
Sma-1-338	NS_000200.1	+	337156779	337156828
Sma-1-339	NW_003026466.1	+	128780	128829
Sma-1-340	NS_000200.1	+	215395887	215395936
Sma-1-341	NW_003037821.1	-	223029	222980
Sma-1-342	NS_000200.1	-	53367513	53367464
Sma-1-343	NW_003038085.1	-	492485	492436
Sma-1-344	NS_000200.1	-	272182578	272182529
Sma-1-345	NW_003021210.1	+	55	104
Sma-1-346	NS_000200.1	+	371593684	371593733
Sma-1-347	NW_003037984.1	-	1940	1891
Sma-1-348	NS_000200.1	-	215073028	215072979
Sma-1-349	NW_003028291.1	-	849	800
Sma-1-350	NW_003038488.1	+	109424	109473
Sma-1-351	NS_000200.1	+	333706481	333706530
Sma-1-352	NS_000200.1	-	374487322	374487273
Sma-1-353	NW_003026458.1	-	719219	719170
Sma-1-354	NS_000200.1	-	102587449	102587400
Sma-1-355	NW_003037852.1	+	151815	151864
Sma-1-356	NS_000200.1	+	94543710	94543759
Sma-1-357	NW_003037795.1	+	353306	353355
Sma-1-358	NS_000200.1	+	6744189	6744238
Sma-1-359	NW_003038016.1	+	108073	108122
Sma-1-360	NS_000200.1	+	231224610	231224659
Sma-1-361	NW_003022661.1	+	2259	2308
Sma-1-362	NS_000200.1	+	365375070	365375119
Sma-1-363	NW_003037916.1	-	1351016	1350967
Sma-1-364	NS_000200.1	-	164544427	164544378
Sma-1-365	NW_003038001.1	-	376789	376740
Sma-1-366	NS_000200.1	-	223086350	223086301
Sma-1-367	NW_003037795.1	-	1162451	1162402
Sma-1-368	NS_000200.1	-	7553334	7553285
Sma-1-369	NW_003037796.1	-	94604	94555
Sma-1-370	NS_000200.1	-	8304248	8304199
Sma-1-371	NW_003038037.1	-	1435907	1435858
Sma-1-372	NS_000200.1	-	251591316	251591267
Sma-1-373	NW_003035881.1	-	317742	317693
Sma-1-374	NS_000200.1	-	341381947	341381898
Sma-1-375	NW_003037979.1	+	389130	389179
Sma-1-376	NS_000200.1	+	210450150	210450199
Sma-1-377	NW_003038037.1	-	1620357	1620308
Sma-1-378	NS_000200.1	-	251775766	251775717

Sma-1-379	NW_003038255.1	-	282592	282543
Sma-1-380	NS_000200.1	-	320671737	320671688
Sma-1-381	NW_003037807.1	+	176583	176632
Sma-1-382	NS_000200.1	+	30438099	30438148
Sma-1-383	NW_003032078.1	+	11481	11530
Sma-1-384	NS_000200.1	+	377345570	377345619
Sma-1-385	NW_003026517.1	+	406865	406914
Sma-1-386	NS_000200.1	+	300012874	300012923
Sma-1-387	NW_003037854.1	-	1512427	1512378
Sma-1-388	NS_000200.1	-	98416848	98416799
Sma-1-389	NW_003037838.1	+	319456	319505
Sma-1-390	NS_000200.1	+	78318019	78318068
Sma-1-391	NW_003038071.1	-	39365	39316
Sma-1-392	NS_000200.1	-	264842408	264842359
Sma-1-393	NW_003037940.1	-	283309	283260
Sma-1-394	NS_000200.1	-	184025439	184025390
Sma-1-395	NW_003020906.1	+	743	792
Sma-1-396	NS_000200.1	+	364673334	364673383
Sma-1-397	NW_003037837.1	+	596053	596102
Sma-1-398	NS_000200.1	+	77401911	77401960
Sma-1-399	NW_003037859.1	+	102808	102857
Sma-1-400	NS_000200.1	+	102781649	102781698
Sma-1-401	NW_003026517.1	+	612020	612069
Sma-1-402	NS_000200.1	+	300218029	300218078
Sma-1-403	NW_003037887.1	+	1155155	1155204
Sma-1-404	NS_000200.1	+	131619133	131619182
Sma-1-405	NW_003037999.1	+	128773	128822
Sma-1-406	NS_000200.1	+	221823327	221823376
Sma-1-407	NW_003038462.1	-	55831	55782
Sma-1-408	NS_000200.1	-	331423494	331423445
Sma-1-409	NW_003038023.1	+	433574	433623
Sma-1-410	NS_000200.1	+	237593865	237593914
Sma-1-411	NW_003038029.1	-	805670	805621
Sma-1-412	NS_000200.1	-	243006571	243006522
Sma-1-413	NW_003038033.1	-	40050	40001
Sma-1-414	NS_000200.1	-	246020052	246020003
Sma-1-415	NW_003037978.1	-	554314	554265
Sma-1-416	NS_000200.1	-	209822324	209822275
Sma-1-417	NW_003037906.1	+	90335	90384
Sma-1-418	NS_000200.1	+	150398114	150398163
Sma-1-419	NW_003038239.1	-	6372	6323
Sma-1-420	NS_000200.1	-	313847154	313847105
Sma-1-421	NW_003037901.1	+	514981	515030
Sma-1-422	NS_000200.1	+	146363442	146363491
Sma-1-423	NW_003037796.1	-	1729007	1728958
Sma-1-424	NS_000200.1	-	9938651	9938602
Sma-1-425	NW_003038013.1	+	207804	207853
Sma-1-426	NS_000200.1	+	229597430	229597479
Sma-1-427	NW_003038251.1	-	32119	32070
Sma-1-428	NS_000200.1	-	318363891	318363842
Sma-1-429	NW_003026517.1	+	402327	402376
Sma-1-430	NS_000200.1	+	300008336	300008385

Sma-1-431	NW_003038097.1	-	26917	26868
Sma-1-432	NS_000200.1	-	274277219	274277170
Sma-1-433	NW_003037909.1	+	496829	496879
Sma-1-434	NS_000200.1	+	154038478	154038528
Sma-1-435	NW_003037796.1	+	203604	203653
Sma-1-436	NS_000200.1	+	8413248	8413297
Sma-1-437	NW_003038023.1	-	317688	317639
Sma-1-438	NS_000200.1	-	237477979	237477930
Sma-1-439	NW_003026472.1	+	395223	395272
Sma-1-440	NS_000200.1	+	236807215	236807264
Sma-1-441	NW_003037944.1	-	540713	540663
Sma-1-442	NS_000200.1	-	188762066	188762016
Sma-1-443	NW_003037860.1	-	530752	530703
Sma-1-444	NS_000200.1	-	104273780	104273731
Sma-1-445	NW_003037836.1	+	751163	751212
Sma-1-446	NS_000200.1	+	76308262	76308311
Sma-1-447	NW_003038159.1	+	82041	82090
Sma-1-448	NS_000200.1	+	296470062	296470111
Sma-1-449	NW_003038005.1	-	295989	295940
Sma-1-450	NS_000200.1	-	224701943	224701894
Sma-1-451	NW_003037822.1	+	370352	370401
Sma-1-452	NS_000200.1	+	54678358	54678407
Sma-1-453	NW_003037996.1	-	225723	225674
Sma-1-454	NS_000200.1	-	220470599	220470550
Sma-1-455	NW_003037911.1	+	132358	132407
Sma-1-456	NS_000200.1	+	155919711	155919760
Sma-1-457	NW_003037818.1	-	361799	361750
Sma-1-458	NS_000200.1	-	49077206	49077157
Sma-1-459	NW_003038008.1	+	217739	217788
Sma-1-460	NS_000200.1	+	225890835	225890884
Sma-1-461	NW_003035869.1	-	154908	154859
Sma-1-462	NS_000200.1	-	335339108	335339059
Sma-1-463	NW_003037883.1	+	244213	244262
Sma-1-464	NS_000200.1	+	126307936	126307985
Sma-1-465	NW_003037858.1	-	1168105	1168056
Sma-1-466	NS_000200.1	-	101832381	101832332
Sma-1-467	NW_003037863.1	-	1028759	1028710
Sma-1-468	NS_000200.1	-	107787126	107787077
Sma-1-469	NW_003037836.1	-	671007	670958
Sma-1-470	NS_000200.1	-	76228106	76228057
Sma-1-471	NW_003037930.1	+	793533	793582
Sma-1-472	NS_000200.1	+	176651497	176651546
Sma-1-473	NW_003038106.1	-	40834	40785
Sma-1-474	NS_000200.1	-	275229599	275229550
Sma-1-475	NW_003038038.1	-	320028	319979
Sma-1-476	NS_000200.1	-	252223421	252223372
Sma-1-477	NW_003037797.1	+	407896	407945
Sma-1-478	NS_000200.1	+	10835756	10835805
Sma-1-479	NW_003037810.1	-	47052	47003
Sma-1-480	NS_000200.1	-	36651574	36651525
Sma-1-481	NW_003037850.1	-	1134801	1134752
Sma-1-482	NS_000200.1	-	93120831	93120782

Sma-1-483	NW_003026516.1	-	343050	343001
Sma-1-484	NS_000200.1	-	299451347	299451298
Sma-1-485	NW_003037818.1	+	2068554	2068603
Sma-1-486	NS_000200.1	+	50783961	50784010
Sma-1-487	NW_003038244.1	+	187257	187306
Sma-1-488	NS_000200.1	+	315332462	315332511
Sma-1-489	NW_003037683.1	+	842	891
Sma-1-490	NS_000200.1	+	375148418	375148467
Sma-1-491	NW_003037698.1	+	3292	3341
Sma-1-492	NS_000200.1	+	375499359	375499408
Sma-1-493	NW_003038085.1	+	501843	501892
Sma-1-494	NS_000200.1	+	272191936	272191985
Sma-1-495	NW_003037794.1	-	884879	884830
Sma-1-496	NS_000200.1	-	5064474	5064425
Sma-1-497	NW_003038161.1	+	239178	239227
Sma-1-498	NS_000200.1	+	297609029	297609078
Sma-1-499	NW_003037977.1	-	840532	840483
Sma-1-500	NS_000200.1	-	209126552	209126503
Sma-1-501	NW_003037966.1	-	478350	478301
Sma-1-502	NS_000200.1	-	199180213	199180164
Sma-1-503	NW_003038257.1	-	207309	207260
Sma-1-504	NS_000200.1	-	321381637	321381588
Sma-1-505	NW_003037853.1	-	611435	611386
Sma-1-506	NS_000200.1	-	96103486	96103437
Sma-1-507	NW_003037994.1	-	474647	474598
Sma-1-508	NS_000200.1	-	219006461	219006412
Sma-1-509	NW_003025858.1	+	51	100
Sma-1-510	NW_003037824.1	-	346002	345953
Sma-1-511	NS_000200.1	+	356643902	356643951
Sma-1-512	NS_000200.1	-	57276942	57276893
Sma-1-513	NW_003026621.1	-	2296	2247
Sma-1-514	NS_000200.1	-	324845690	324845641
Sma-1-515	NW_003032268.1	-	23915	23866
Sma-1-516	NS_000200.1	-	358284675	358284626
Sma-1-517	NW_003026608.1	+	40450	40499
Sma-1-518	NS_000200.1	+	309318570	309318619
Sma-1-519	NW_003033782.1	-	5600	5550
Sma-1-520	NS_000200.1	-	358957906	358957856
Sma-1-521	NW_003037940.1	+	573487	573536
Sma-1-522	NS_000200.1	+	184315617	184315666
Sma-1-523	NW_003037806.1	+	1105337	1105386
Sma-1-524	NS_000200.1	+	29300551	29300600
Sma-1-525	NW_003038070.1	+	396023	396072
Sma-1-526	NS_000200.1	+	264733264	264733313
Sma-1-527	NW_003038163.1	+	606832	606881
Sma-1-528	NS_000200.1	+	299007112	299007161
Sma-1-529	NW_003037881.1	-	352329	352280
Sma-1-530	NS_000200.1	-	124784120	124784071
Sma-1-531	NW_003037797.1	+	1635731	1635780
Sma-1-532	NS_000200.1	+	12063591	12063640
Sma-1-533	NW_003039127.1	+	366449	366498
Sma-1-534	NS_000200.1	+	113443948	113443997

Sma-1-535	NW_003026619.1	+	100641	100690
Sma-1-536	NS_000200.1	+	322291385	322291434
Sma-1-537	NW_003037978.1	+	456818	456867
Sma-1-538	NS_000200.1	+	209724828	209724877
Sma-1-539	NW_003026484.1	+	15526	15575
Sma-1-540	NS_000200.1	+	256978217	256978266
Sma-1-541	NW_003026474.1	-	65233	65184
Sma-1-542	NS_000200.1	-	253263808	253263759
Sma-1-543	NW_003037913.1	-	949788	949739
Sma-1-544	NS_000200.1	-	159513539	159513490
Sma-1-545	NW_003037835.1	-	514955	514906
Sma-1-546	NS_000200.1	-	74911463	74911414
Sma-1-547	NW_003033782.1	+	151	200
Sma-1-548	NW_003038001.1	+	379833	379882
Sma-1-549	NS_000200.1	+	223089394	223089443
Sma-1-550	NS_000200.1	+	358952457	358952506
Sma-1-551	NW_003038083.1	-	35564	35515
Sma-1-552	NS_000200.1	-	270278901	270278852
Sma-1-553	NW_003026465.1	+	547485	547534
Sma-1-554	NS_000200.1	+	207940079	207940128
Sma-1-555	NW_003037918.1	-	2137985	2137936
Sma-1-556	NS_000200.1	-	168704975	168704926
Sma-1-557	NW_003037805.1	-	1032214	1032165
Sma-1-558	NS_000200.1	-	27590058	27590009
env-78	AB231864.1	+	86311	86360
Sma-1-559	NW_003037980.1	+	263935	263984
Sma-1-560	NS_000200.1	+	211224797	211224846
Sma-1-561	NW_003037787.1	-	1385	1336
Sma-1-562	NS_000200.1	-	380249554	380249505
Sma-1-563	NW_003026456.1	-	301597	301548
Sma-1-564	NS_000200.1	-	58224733	58224684
Sma-1-565	NW_003038058.1	-	379088	379039
Sma-1-566	NS_000200.1	-	260210288	260210239
Sma-1-567	NW_003037971.1	+	630216	630265
Sma-1-568	NS_000200.1	+	202252266	202252315
Sma-1-569	NW_003026515.1	-	179612	179563
Sma-1-570	NS_000200.1	-	290824023	290823974
Sma-1-571	NW_003037823.1	+	245857	245906
Sma-1-572	NS_000200.1	+	56162476	56162525
Sma-1-573	NW_003026594.1	+	9623	9672
Sma-1-574	NS_000200.1	+	304953993	304954042
Sma-1-575	NW_003037918.1	+	1252048	1252097
Sma-1-576	NS_000200.1	+	167819038	167819087
Sma-1-577	NW_003037834.1	-	807224	807175
Sma-1-578	NS_000200.1	-	73967710	73967661
Sma-1-579	NW_003037788.1	+	107143	107192
Sma-1-580	NS_000200.1	+	380554381	380554430
Sma-1-581	NW_003037796.1	-	1676597	1676548
Sma-1-582	NS_000200.1	-	9886241	9886192
Sma-1-583	NW_003037823.1	+	251462	251511
Sma-1-584	NS_000200.1	+	56168081	56168130
Sma-1-585	NW_003037888.1	+	1217615	1217664

Sma-1-586	NS_000200.1	+	133426107	133426156
Sma-1-587	NW_003038084.1	+	118897	118946
Sma-1-588	NS_000200.1	+	271134141	271134190
Sma-1-589	NW_003038147.1	+	121550	121599
Sma-1-590	NS_000200.1	+	289649293	289649342
Sma-1-591	NW_003037797.1	+	2518052	2518101
Sma-1-592	NW_003037813.1	+	722755	722804
Sma-1-593	NS_000200.1	+	12945912	12945961
Sma-1-594	NS_000200.1	+	41709618	41709667
Sma-1-595	NW_003037916.1	+	625037	625086
Sma-1-596	NS_000200.1	+	163818448	163818497
Sma-1-597	NW_003037841.1	-	477218	477169
Sma-1-598	NW_003037868.1	+	480071	480120
Sma-1-599	NS_000200.1	+	116451783	116451832
Sma-1-600	NS_000200.1	-	83088399	83088350
Tcr-1-1	NZ_AAHK01026755.1	+	172	221
Sma-1-601	NW_003037916.1	-	1682574	1682525
Sma-1-602	NS_000200.1	-	164875985	164875936
Sma-1-603	NW_003037831.1	-	773070	773021
Sma-1-604	NS_000200.1	-	70222372	70222323
Sma-1-605	NW_003037822.1	-	314443	314394
Sma-1-606	NS_000200.1	-	54622449	54622400
Sma-1-607	NW_003037940.1	-	178299	178250
Sma-1-608	NS_000200.1	-	183920429	183920380
Sma-1-609	NW_003038151.1	+	214097	214146
Sma-1-610	NS_000200.1	+	292121340	292121389
Sma-1-611	NW_003029307.1	-	286	237
Sma-1-612	NS_000200.1	-	357258996	357258947
Sma-1-613	NW_003038224.1	-	68321	68272
Sma-1-614	NS_000200.1	-	309480049	309480000
Sma-1-615	NW_003037796.1	+	400256	400305
Sma-1-616	NS_000200.1	+	8609900	8609949
Sma-1-617	NW_003037971.1	+	840256	840305
Sma-1-618	NS_000200.1	+	202462306	202462355
Sma-1-619	NW_003037800.1	-	1705452	1705403
Sma-1-620	NS_000200.1	-	20334812	20334763
Sma-1-621	NW_003038161.1	-	490818	490769
Sma-1-622	NS_000200.1	-	297860669	297860620
Sma-1-623	NW_003038077.1	+	406768	406817
Sma-1-624	NS_000200.1	+	267178408	267178457
Sma-1-625	NW_003038024.1	+	738469	738518
Sma-1-626	NS_000200.1	+	238635498	238635547
Sma-1-627	NW_003037965.1	+	6715	6764
Sma-1-628	NS_000200.1	+	198295800	198295849
Sma-1-629	NW_003037798.1	+	480286	480335
Sma-1-630	NS_000200.1	+	14739444	14739493
Sma-1-631	NW_003037935.1	-	685278	685229
Sma-1-632	NS_000200.1	-	180101813	180101764
Sma-1-633	NW_003037916.1	-	837040	836991
Sma-1-634	NS_000200.1	-	164030451	164030402
Sma-1-635	NW_003038241.1	-	175695	175646
Sma-1-636	NS_000200.1	-	314638400	314638351

Sma-1-637	NW_003037895.1	+	292226	292275
Sma-1-638	NS_000200.1	+	142738428	142738477
Sma-1-639	NW_003037809.1	-	1397739	1397690
Sma-1-640	NS_000200.1	-	35062203	35062154
Sma-1-641	NW_003038131.1	-	211278	211229
Sma-1-642	NS_000200.1	-	282173122	282173073
Sma-1-643	NW_003037923.1	+	102090	102139
Sma-1-644	NS_000200.1	+	171158303	171158352
Sma-1-645	NW_003038013.1	-	239752	239703
Sma-1-646	NS_000200.1	-	229629378	229629329
Sma-1-647	NW_003037868.1	+	118548	118597
Sma-1-648	NS_000200.1	+	116090260	116090309
Sma-1-649	NW_003037917.1	+	1237998	1238047
Sma-1-650	NS_000200.1	+	166274802	166274851
Sma-1-651	NW_003037851.1	+	522070	522120
Sma-1-652	NS_000200.1	+	93925380	93925430
Sma-1-653	NW_003037844.1	-	282977	282928
Sma-1-654	NS_000200.1	-	86466423	86466374
Sma-1-655	NW_003038261.1	-	1056544	1056495
Sma-1-656	NS_000200.1	-	324670195	324670146
Sma-1-657	NW_003037807.1	-	1216446	1216397
Sma-1-658	NS_000200.1	-	31477962	31477913
Sma-1-659	NW_003038241.1	+	103854	103903
Sma-1-660	NS_000200.1	+	314566559	314566608
Sma-1-661	NW_003037808.1	-	615902	615853
Sma-1-662	NW_003038083.1	+	479087	479136
Sma-1-663	NS_000200.1	+	270722424	270722473
Sma-1-664	NS_000200.1	-	32402539	32402490
Sma-1-665	NW_003020312.1	+	989	1038
Sma-1-666	NS_000200.1	+	371397082	371397131
Sma-1-667	NW_003037917.1	-	374323	374274
Sma-1-668	NS_000200.1	-	165411127	165411078
Sma-1-669	NW_003037827.1	-	698322	698273
Sma-1-670	NS_000200.1	-	63320463	63320414
Sma-1-671	NW_003038083.1	-	757627	757578
Sma-1-672	NS_000200.1	-	271000964	271000915
Sma-1-673	NW_003038146.1	-	8367	8318
Sma-1-674	NS_000200.1	-	288989037	288988988
Sma-1-675	NW_003026460.1	+	124160	124210
Sma-1-676	NS_000200.1	+	170646366	170646416
Sma-1-677	NW_003037982.1	-	1265489	1265440
Sma-1-678	NS_000200.1	-	214924684	214924635
Sma-1-679	NW_003037903.1	-	953942	953893
Sma-1-680	NS_000200.1	-	148508023	148507974
Sma-1-681	NW_003037814.1	+	89354	89403
Sma-1-682	NS_000200.1	+	43073214	43073263
Sma-1-683	NW_003037824.1	-	208699	208650
Sma-1-684	NS_000200.1	-	57139639	57139590
Sma-1-685	NW_003037889.1	-	349927	349878
Sma-1-686	NS_000200.1	-	134126417	134126368
Sma-1-687	NW_003037841.1	+	1113939	1113988
Sma-1-688	NS_000200.1	+	83725120	83725169

Sma-1-689	NW_003037939.1	+	383304	383353
Sma-1-690	NS_000200.1	+	183253058	183253107
Sma-1-691	NW_003026482.1	-	17603	17554
Sma-1-692	NS_000200.1	-	255805349	255805300
Sma-1-693	NW_003038015.1	-	182316	182267
Sma-1-694	NS_000200.1	-	230505806	230505757
Sma-1-695	NW_003037962.1	+	475305	475354
Sma-1-696	NS_000200.1	+	196552148	196552197
Sma-1-697	NW_003037920.1	+	25381	25430
Sma-1-698	NS_000200.1	+	169206188	169206237
Sma-1-699	NW_003026462.1	+	230811	230860
Sma-1-700	NS_000200.1	+	196898314	196898363
Sma-1-701	NW_003037914.1	-	596233	596184
Sma-1-702	NS_000200.1	-	160740483	160740434
Sma-1-703	NW_003037919.1	+	18857	18906
Sma-1-704	NS_000200.1	+	168876786	168876835
Sma-1-705	NW_003037797.1	-	1381216	1381167
Sma-1-706	NS_000200.1	-	11809076	11809027
Sma-1-707	NW_003037904.1	+	172858	172907
Sma-1-708	NS_000200.1	+	148887357	148887406
Sma-1-709	NW_003038056.1	+	161042	161091
Sma-1-710	NS_000200.1	+	259138693	259138742
Sma-1-711	NW_003037938.1	+	84968	85017
Sma-1-712	NS_000200.1	+	182122081	182122130
Sma-1-713	NW_003038252.1	+	114690	114740
Sma-1-714	NS_000200.1	+	318970278	318970328
Sma-1-715	NW_003037935.1	+	875811	875861
Sma-1-716	NS_000200.1	+	180292346	180292396
Sma-1-717	NW_003037826.1	+	365309	365359
Sma-1-718	NS_000200.1	+	61364825	61364875
Sma-1-719	NW_003038028.1	+	644752	644801
Sma-1-720	NS_000200.1	+	241952326	241952375
env-79	AY167025.1	-	16892	16847
Sma-1-721	NW_003037928.1	+	285674	285723
Sma-1-722	NS_000200.1	+	174609627	174609676
Sma-1-723	NW_003037803.1	+	385354	385403
Sma-1-724	NS_000200.1	+	23885891	23885940
Sma-1-725	NW_003037995.1	-	210050	210003
Sma-1-726	NS_000200.1	-	219899818	219899771
Sma-1-727	NW_003038030.1	+	667484	667532
Sma-1-728	NS_000200.1	+	243803351	243803399
Sma-1-729	NW_003038095.1	-	31031	30983
Sma-1-730	NS_000200.1	-	274100073	274100025
Sma-1-731	NW_003037827.1	+	228036	228084
Sma-1-732	NS_000200.1	+	62850177	62850225
Sma-1-733	NW_003038161.1	+	393359	393407
Sma-1-734	NS_000200.1	+	297763210	297763258
Sma-1-735	NW_003038027.1	-	134680	134632
Sma-1-736	NS_000200.1	-	240505394	240505346
Sma-1-737	NW_003026618.1	+	1790	1839
Sma-1-738	NS_000200.1	+	321756792	321756841
Sma-1-739	NW_003037923.1	+	96656	96704

Sma-1-740	NS_000200.1	+	171152869	171152917
Sma-1-741	NW_003037792.1	-	246181	246133
Sma-1-742	NS_000200.1	-	382683525	382683477
Sma-1-743	NW_003037914.1	-	329788	329740
Sma-1-744	NS_000200.1	-	160474038	160473990
Sma-1-745	NW_003038541.1	-	1231	1182
Sma-1-746	NS_000200.1	-	343392080	343392031
Sma-1-747	NW_003037890.1	-	1104919	1104870
Sma-1-748	NS_000200.1	-	136788280	136788231
Sma-1-749	NW_003038246.1	+	173082	173131
Sma-1-750	NS_000200.1	+	315814210	315814259
Sma-1-751	NW_003030390.1	+	906	955
Sma-1-752	NS_000200.1	+	360009513	360009562
Sma-1-753	NW_003038163.1	-	634567	634518
Sma-1-754	NS_000200.1	-	299034847	299034798
Sma-1-755	NW_003037860.1	-	86443	86394
Sma-1-756	NS_000200.1	-	103829471	103829422
Sma-1-757	NW_003037909.1	-	733336	733287
Sma-1-758	NS_000200.1	-	154274985	154274936
Sma-1-759	NW_003038146.1	-	323449	323400
Sma-1-760	NS_000200.1	-	289304119	289304070
Sma-1-761	NW_003038081.1	-	263240	263191
Sma-1-762	NS_000200.1	-	269425283	269425234
Sma-1-763	NW_003038147.1	-	366843	366794
Sma-1-764	NS_000200.1	-	289894586	289894537
Sma-1-765	NW_003037841.1	+	1883161	1883210
Sma-1-766	NS_000200.1	+	84494342	84494391
Sma-1-767	NW_003037814.1	-	2130414	2130365
Sma-1-768	NS_000200.1	-	45114274	45114225
Sma-1-769	NW_003038250.1	+	187496	187545
Sma-1-770	NS_000200.1	+	318240173	318240222
Sma-1-771	NW_003037827.1	-	1727419	1727370
Sma-1-772	NS_000200.1	-	64349560	64349511
Sma-1-773	NW_003037976.1	+	203870	203919
Sma-1-774	NS_000200.1	+	206818185	206818234
Sma-1-775	NW_003037860.1	-	55782	55733
Sma-1-776	NS_000200.1	-	103798810	103798761
Sma-1-777	NW_003037896.1	+	476015	476064
Sma-1-778	NS_000200.1	+	143396438	143396487
Sma-1-779	NW_003035869.1	-	150960	150911
Sma-1-780	NS_000200.1	-	335335160	335335111
Sma-1-781	NW_003037834.1	-	671901	671852
Sma-1-782	NS_000200.1	-	73832387	73832338
env-80	AB231864.1	-	117137	117092
Sma-1-783	NW_003037980.1	-	295103	295058
Sma-1-784	NS_000200.1	-	211255965	211255920
Sma-1-785	NW_003037992.1	+	123764	123813
Sma-1-786	NS_000200.1	+	217965245	217965294
Sma-1-787	NW_003038124.1	+	51512	51561
Sma-1-788	NS_000200.1	+	279044156	279044205
Sma-1-789	NW_003037887.1	+	374452	374501
Sma-1-790	NS_000200.1	+	130838430	130838479

Sma-1-791	NW_003037981.1	-	340170	340121
Sma-1-792	NS_000200.1	-	212738038	212737989
Sma-1-793	NW_003037915.1	-	1109105	1109056
Sma-1-794	NS_000200.1	-	163009091	163009042
Sma-1-795	NW_003037824.1	-	767501	767452
Sma-1-796	NS_000200.1	-	57698441	57698392
Sma-1-797	NW_003038033.1	+	173736	173785
Sma-1-798	NS_000200.1	+	246153738	246153787
Sma-1-799	NW_003037806.1	+	1185389	1185438
Sma-1-800	NS_000200.1	+	29380603	29380652
Sma-1-801	NW_003037799.1	-	1480515	1480466
Sma-1-802	NS_000200.1	-	18470869	18470820
Sma-1-803	NW_003037846.1	+	723007	723056
Sma-1-804	NS_000200.1	+	88357577	88357626
Sma-1-805	NW_003037868.1	+	425983	426032
Sma-1-806	NS_000200.1	+	116397695	116397744
Sma-1-807	NW_003037911.1	+	836086	836135
Sma-1-808	NS_000200.1	+	156623439	156623488
Sma-1-809	NW_003037891.1	-	653679	653630
Sma-1-810	NS_000200.1	-	138534957	138534908
Sma-1-811	NW_003038005.1	-	359725	359676
Sma-1-812	NS_000200.1	-	224765679	224765630
Sma-1-813	NW_003037793.1	-	692365	692316
Sma-1-814	NS_000200.1	-	692365	692316
Sma-1-815	NW_003038023.1	+	724652	724701
Sma-1-816	NS_000200.1	+	237884943	237884992
Sma-1-817	NW_003039130.1	-	105886	105837
Sma-1-818	NS_000200.1	-	317780497	317780448
Sma-1-819	NW_003020984.1	+	506	555
Sma-1-820	NS_000200.1	+	367729338	367729387
Sma-1-821	NW_003038023.1	+	714692	714741
Sma-1-822	NS_000200.1	+	237874983	237875032
Sma-1-823	NW_003037814.1	-	1783694	1783645
Sma-1-824	NS_000200.1	-	44767554	44767505
Sma-1-825	NW_003037915.1	-	1204936	1204887
Sma-1-826	NS_000200.1	-	163104922	163104873
Sma-1-827	NW_003037839.1	+	401556	401605
Sma-1-828	NS_000200.1	+	79359706	79359755
Sma-1-829	NW_003037891.1	-	480129	480080
Sma-1-830	NS_000200.1	-	138361407	138361358
Sma-1-831	NW_003038261.1	+	769098	769147
Sma-1-832	NS_000200.1	+	324382749	324382798
Sma-1-833	NW_003026472.1	+	172306	172355
Sma-1-834	NS_000200.1	+	236584298	236584347
Sma-1-835	NW_003037884.1	+	968569	968618
Sma-1-836	NS_000200.1	+	127748234	127748283
Sma-1-837	NW_003037819.1	-	1140460	1140411
Sma-1-838	NS_000200.1	-	52228725	52228676
Sma-1-839	NW_003037859.1	+	369558	369608
Sma-1-840	NS_000200.1	+	103048399	103048449
Sma-1-841	NW_003038002.1	-	146929	146880
Sma-1-842	NS_000200.1	-	223267138	223267089

Sma-1-843	NW_003037787.1	+	192459	192508
Sma-1-844	NS_000200.1	+	380440628	380440677
Sma-1-845	NW_003037873.1	+	676403	676452
Sma-1-846	NS_000200.1	+	119415531	119415580
Sma-1-847	NW_003038001.1	+	376911	376960
Sma-1-848	NS_000200.1	+	223086472	223086521
Sma-1-849	NW_003038086.1	+	433581	433630
Sma-1-850	NS_000200.1	+	272682174	272682223
Sma-1-851	NW_003026607.1	+	145142	145191
Sma-1-852	NS_000200.1	+	308177527	308177576
Sma-1-853	NW_003038132.1	+	435081	435130
Sma-1-854	NS_000200.1	+	282855551	282855600
Sma-1-855	NW_003038196.1	+	48855	48904
Sma-1-856	NS_000200.1	+	303191138	303191187
Sma-1-857	NW_003038023.1	-	571731	571682
Sma-1-858	NS_000200.1	-	237732022	237731973
Sma-1-859	NW_003023030.1	+	3232	3281
Sma-1-860	NS_000200.1	+	372111452	372111501
Sma-1-861	NW_003038037.1	-	436580	436531
Sma-1-862	NS_000200.1	-	250591989	250591940
Sma-1-863	NW_003037977.1	-	339163	339114
Sma-1-864	NS_000200.1	-	208625183	208625134
Sma-1-865	NW_003037798.1	-	1316742	1316693
Sma-1-866	NS_000200.1	-	15575900	15575851
Sma-1-867	NW_003037884.1	+	1302266	1302315
Sma-1-868	NS_000200.1	+	128081931	128081980
Sma-1-869	NW_003038471.1	-	7877	7828
Sma-1-870	NS_000200.1	-	331848791	331848742
Sma-1-871	NW_003026458.1	-	278638	278589
Sma-1-872	NS_000200.1	-	102146868	102146819
Sma-1-873	NW_003037829.1	-	865507	865458
Sma-1-874	NS_000200.1	-	66766045	66765996
Sma-1-875	NW_003038094.1	+	23598	23647
Sma-1-876	NS_000200.1	+	274066912	274066961
Sma-1-877	NW_003037849.1	-	315767	315718
Sma-1-878	NS_000200.1	-	91292040	91291991
Sma-1-879	NW_003038229.1	+	40664	40713
Sma-1-880	NS_000200.1	+	311157886	311157935
Sma-1-881	NW_003037810.1	-	361470	361421
Sma-1-882	NS_000200.1	-	36965992	36965943
Sma-1-883	NW_003037831.1	+	1127260	1127309
Sma-1-884	NS_000200.1	+	70576562	70576611
Sma-1-885	NW_003026466.1	-	54368	54319
Sma-1-886	NS_000200.1	-	215321475	215321426
Sma-1-887	NW_003038161.1	+	181712	181761
Sma-1-888	NS_000200.1	+	297551563	297551612
Sma-1-889	NW_003037901.1	-	652283	652234
Sma-1-890	NS_000200.1	-	146500744	146500695
Sma-1-891	NW_003037890.1	-	378670	378621
Sma-1-892	NS_000200.1	-	136062031	136061982
Sma-1-893	NW_003038131.1	-	332170	332121
Sma-1-894	NS_000200.1	-	282294014	282293965

Sma-1-895	NW_003026514.1	-	112222	112173
Sma-1-896	NS_000200.1	-	288570909	288570860
Sma-1-897	NW_003037880.1	+	453315	453364
Sma-1-898	NS_000200.1	+	124136873	124136922
Sma-1-899	NW_003037827.1	-	2264732	2264683
Sma-1-900	NS_000200.1	-	64886873	64886824
Sma-1-901	NW_003037901.1	-	784013	783964
Sma-1-902	NS_000200.1	-	146632474	146632425
Sma-1-903	NW_003037888.1	-	780475	780426
Sma-1-904	NS_000200.1	-	132988967	132988918
Sma-1-905	NW_003037831.1	+	1533673	1533722
Sma-1-906	NS_000200.1	+	70982975	70983024
Sma-1-907	NW_003037916.1	+	1634486	1634535
Sma-1-908	NS_000200.1	+	164827897	164827946
Sma-1-909	NW_003037930.1	-	149774	149725
Sma-1-910	NS_000200.1	-	176007738	176007689
Sma-1-911	NW_003037831.1	-	1681192	1681143
Sma-1-912	NS_000200.1	-	71130494	71130445
Sma-1-913	NW_003037810.1	-	338398	338349
Sma-1-914	NS_000200.1	-	36942920	36942871
Sma-1-915	NW_003037798.1	-	1890980	1890931
Sma-1-916	NS_000200.1	-	16150138	16150089
Sma-1-917	NW_003037795.1	+	1575490	1575539
Sma-1-918	NS_000200.1	+	7966373	7966422
Sma-1-919	NW_003037788.1	-	172199	172150
Sma-1-920	NS_000200.1	-	380619437	380619388
Sma-1-921	NW_003037851.1	-	382933	382884
Sma-1-922	NS_000200.1	-	93786243	93786194
Sma-1-923	NW_003026456.1	+	553931	553980
Sma-1-924	NS_000200.1	+	58477067	58477116
Sma-1-925	NW_003037988.1	+	163393	163443
Sma-1-926	NS_000200.1	+	216157211	216157261
Sma-1-927	NW_003026515.1	+	289418	289467
Sma-1-928	NS_000200.1	+	290933829	290933878
Sma-1-929	NW_003038115.1	-	110616	110567
Sma-1-930	NS_000200.1	-	277165911	277165862
Sma-1-931	NW_003026616.1	+	314488	314537
Sma-1-932	NS_000200.1	+	317671087	317671136
Sma-1-933	NW_003026458.1	-	377559	377510
Sma-1-934	NS_000200.1	-	102245789	102245740
Sma-1-935	NW_003037834.1	-	155402	155350
Sma-1-936	NS_000200.1	-	73315888	73315836
Sma-1-937	NW_003037923.1	+	114176	114225
Sma-1-938	NS_000200.1	+	171170389	171170438
Sma-1-939	NW_003037801.1	-	483979	483930
Sma-1-940	NS_000200.1	-	21573459	21573410
Sma-1-941	NW_003026464.1	+	342279	342328
Sma-1-942	NS_000200.1	+	203642588	203642637
Sma-1-943	NW_003026608.1	+	52354	52403
Sma-1-944	NS_000200.1	+	309330474	309330523
Sma-1-945	NW_003037933.1	-	140143	140093
Sma-1-946	NS_000200.1	-	178553315	178553265

Sma-1-947	NW_003037814.1	-	999248	999199
Sma-1-948	NS_000200.1	-	43983108	43983059
Sma-1-949	NW_003037807.1	+	94897	94946
Sma-1-950	NS_000200.1	+	30356413	30356462
Sma-1-951	NW_003037793.1	+	3672925	3672973
Sma-1-952	NS_000200.1	+	3672925	3672973
Sma-1-953	NW_003037851.1	-	750733	750685
Sma-1-954	NS_000200.1	-	94154043	94153995
Sma-1-955	NW_003037797.1	+	2065268	2065317
Sma-1-956	NS_000200.1	+	12493128	12493177
Sma-1-957	NW_003037909.1	+	571470	571518
Sma-1-958	NS_000200.1	+	154113119	154113167
Sma-1-959	NW_003030366.1	+	575	623
Sma-1-960	NS_000200.1	+	362776720	362776768
Sma-1-961	NW_003038247.1	+	346889	346937
Sma-1-962	NS_000200.1	+	316295617	316295665
Sma-1-963	NW_003037840.1	-	1724147	1724098
Sma-1-964	NS_000200.1	-	82347471	82347422
Sma-1-965	NW_003037838.1	+	874480	874529
Sma-1-966	NS_000200.1	+	78873043	78873092
Sma-1-967	NW_003037901.1	+	378921	378969
Sma-1-968	NS_000200.1	+	146227382	146227430
Sma-1-969	NW_003037901.1	+	521120	521168
Sma-1-970	NS_000200.1	+	146369581	146369629
Sma-1-971	NW_003026456.1	+	150182	150230
Sma-1-972	NS_000200.1	+	58073318	58073366
Sma-1-973	NW_003037834.1	+	947163	947211
Sma-1-974	NS_000200.1	+	74107649	74107697
Sma-1-975	NW_003037982.1	-	951467	951419
Sma-1-976	NS_000200.1	-	214610662	214610614
Sma-1-977	NW_003026462.1	-	470155	470107
Sma-1-978	NS_000200.1	-	197137658	197137610
Sma-1-979	NW_003026459.1	+	234852	234901
Sma-1-980	NS_000200.1	+	141880125	141880174
Sma-1-981	NW_003037981.1	-	215031	214982
Sma-1-982	NS_000200.1	-	212612899	212612850
Sma-1-983	NW_003026456.1	-	56186	56137
Sma-1-984	NS_000200.1	-	57979322	57979273
Sma-1-985	NW_003037905.1	+	187901	187950
Sma-1-986	NS_000200.1	+	149456913	149456962
Sma-1-987	NW_003037980.1	+	1302643	1302692
Sma-1-988	NS_000200.1	+	212263505	212263554
Sma-1-989	NW_003025206.1	+	1541	1590
Sma-1-990	NS_000200.1	+	361395509	361395558
Sma-1-991	NW_003037836.1	-	273691	273642
Sma-1-992	NS_000200.1	-	75830790	75830741
Sma-1-993	NW_003025176.1	-	1324	1275
Sma-1-994	NW_003038015.1	+	713200	713249
Sma-1-995	NS_000200.1	+	231036690	231036739
Sma-1-996	NS_000200.1	-	361334941	361334892
Sma-1-997	NW_003037901.1	+	592169	592218
Sma-1-998	NS_000200.1	+	146440630	146440679

Sma-1-999	NW_003026469.1	-	205970	205921
Sma-1-1000	NS_000200.1	-	217827629	217827580
Sma-1-1001	NW_003037997.1	+	413357	413407
Sma-1-1002	NS_000200.1	+	221224799	221224849
Sma-1-1003	NW_003026470.1	-	492387	492338
Sma-1-1004	NS_000200.1	-	219616356	219616307
Sma-1-1005	NW_003037830.1	+	624040	624089
Sma-1-1006	NS_000200.1	+	68514411	68514460
Sma-1-1007	NW_003026484.1	+	74421	74470
Sma-1-1008	NS_000200.1	+	257037112	257037161
Sma-1-1009	NW_003037804.1	-	930368	930319
Sma-1-1010	NS_000200.1	-	26070388	26070339
Sma-1-1011	NW_003037841.1	+	500341	500390
Sma-1-1012	NS_000200.1	+	83111522	83111571
Sma-1-1013	NW_003037895.1	-	263173	263124
Sma-1-1014	NS_000200.1	-	142709375	142709326
Sma-1-1015	NW_003037844.1	+	415942	415991
Sma-1-1016	NS_000200.1	+	86599388	86599437
Sma-1-1017	NW_003026504.1	-	85760	85711
Sma-1-1018	NS_000200.1	-	277002526	277002477
Sma-1-1019	NW_003037829.1	-	1731785	1731736
Sma-1-1020	NS_000200.1	-	67632323	67632274
Sma-1-1021	NW_003037977.1	+	487557	487606
Sma-1-1022	NS_000200.1	+	208773577	208773626
Sma-1-1023	NW_003037891.1	+	931978	932027
Sma-1-1024	NS_000200.1	+	138813256	138813305
Sma-1-1025	NW_003037848.1	+	931306	931355
Sma-1-1026	NS_000200.1	+	90624377	90624426
Sma-1-1027	NW_003037928.1	+	152379	152428
Sma-1-1028	NS_000200.1	+	174476332	174476381
Sma-1-1029	NW_003037961.1	+	353436	353485
Sma-1-1030	NS_000200.1	+	195977678	195977727
Sma-1-1031	NW_003037891.1	+	1245677	1245726
Sma-1-1032	NS_000200.1	+	139126955	139127004
Sma-1-1033	NW_003037814.1	-	192634	192585
Sma-1-1034	NS_000200.1	-	43176494	43176445
Sma-1-1035	NW_003037794.1	+	687533	687582
Sma-1-1036	NS_000200.1	+	4867128	4867177
Sma-1-1037	NW_003037918.1	-	561420	561371
Sma-1-1038	NS_000200.1	-	167128410	167128361
Sma-1-1039	NW_003038130.1	+	106642	106691
Sma-1-1040	NS_000200.1	+	281661628	281661677
Sma-1-1041	NW_003037912.1	+	1723749	1723798
Sma-1-1042	NS_000200.1	+	158535324	158535373
Sma-1-1043	NW_003037818.1	-	1412574	1412525
Sma-1-1044	NS_000200.1	-	50127981	50127932
Sma-1-1045	NW_003038070.1	+	696	745
Sma-1-1046	NS_000200.1	+	264337937	264337986
Sma-1-1047	NW_003037852.1	+	1062494	1062543
Sma-1-1048	NS_000200.1	+	95454389	95454438
Sma-1-1049	NW_003037827.1	-	442783	442734
Sma-1-1050	NS_000200.1	-	63064924	63064875

Sma-1-1051	NW_003026577.1	+	15448	15497
Sma-1-1052	NS_000200.1	+	302686704	302686753
Sma-1-1053	NW_003037918.1	+	1043824	1043873
Sma-1-1054	NS_000200.1	+	167610814	167610863
Sma-1-1055	NW_003022920.1	-	559	510
Sma-1-1056	NS_000200.1	-	371883353	371883304
Sma-1-1057	NW_003033045.1	-	463	414
Sma-1-1058	NS_000200.1	-	326267351	326267302
Sma-1-1059	NW_003038023.1	-	566131	566082
Sma-1-1060	NS_000200.1	-	237726422	237726373
Sma-1-1061	NW_003037853.1	+	316625	316674
Sma-1-1062	NS_000200.1	+	95808676	95808725
Sma-1-1063	NW_003038014.1	-	223606	223557
Sma-1-1064	NS_000200.1	-	229964295	229964246
Sma-1-1065	NW_003026599.1	+	7012	7061
Sma-1-1066	NS_000200.1	+	305885341	305885390
Sma-1-1067	NW_003026605.1	-	12928	12879
Sma-1-1068	NS_000200.1	-	307691792	307691743
Sma-1-1069	NW_003037889.1	-	878970	878921
Sma-1-1070	NS_000200.1	-	134655460	134655411
Sma-1-1071	NW_003037908.1	+	263402	263451
Sma-1-1072	NW_003037908.1	-	264036	263987
Sma-1-1073	NS_000200.1	+	152736533	152736582
Sma-1-1074	NS_000200.1	-	152737167	152737118
Sma-1-1075	NW_003026611.1	+	126887	126936
Sma-1-1076	NS_000200.1	+	310696671	310696720
Sma-1-1077	NW_003037972.1	-	484572	484523
Sma-1-1078	NS_000200.1	-	202960276	202960227
Sma-1-1079	NW_003037795.1	-	1486300	1486251
Sma-1-1080	NS_000200.1	-	7877183	7877134
Sma-1-1081	NW_003037826.1	+	1350034	1350083
Sma-1-1082	NS_000200.1	+	62349550	62349599
Sma-1-1083	NW_003026616.1	+	140375	140424
Sma-1-1084	NS_000200.1	+	317496974	317497023
Sma-1-1085	NW_003038019.1	-	253653	253604
Sma-1-1086	NS_000200.1	-	233570471	233570422
Sma-1-1087	NW_003035689.1	-	10530	10481
Sma-1-1088	NS_000200.1	-	333095746	333095697
Sma-1-1089	NW_003038022.1	-	320209	320160
Sma-1-1090	NS_000200.1	-	236001051	236001002
Sma-1-1091	NW_003037971.1	-	639433	639384
Sma-1-1092	NS_000200.1	-	202261483	202261434
Sma-1-1093	NW_003037908.1	+	995446	995495
Sma-1-1094	NS_000200.1	+	153468577	153468626
Sma-1-1095	NW_003038070.1	+	133989	134038
Sma-1-1096	NS_000200.1	+	264471230	264471279
Sma-1-1097	NW_003026618.1	-	242417	242368
Sma-1-1098	NS_000200.1	-	321997419	321997370
Sma-1-1099	NW_003037825.1	+	80033	80082
Sma-1-1100	NS_000200.1	+	59209815	59209864
Sma-1-1101	NW_003037970.1	+	396607	396656
Sma-1-1102	NS_000200.1	+	201452797	201452846

Sma-1-1103	NW_003039127.1	-	2304473	2304424
Sma-1-1104	NS_000200.1	-	115381972	115381923
Sma-1-1105	NW_003026458.1	+	514192	514241
Sma-1-1106	NW_003026458.1	-	515765	515716
Sma-1-1107	NS_000200.1	+	102382422	102382471
Sma-1-1108	NS_000200.1	-	102383995	102383946
Sma-1-1109	NW_003026637.1	-	2923	2874
Sma-1-1110	NS_000200.1	-	325043959	325043910
Sma-1-1111	NW_003037969.1	+	257242	257291
Sma-1-1112	NW_003026484.1	+	10941	10990
Sma-1-1113	NS_000200.1	+	200863369	200863418
Sma-1-1114	NS_000200.1	+	256973632	256973681
Sma-1-1115	NW_003027763.1	-	892	843
Sma-1-1116	NS_000200.1	-	365898106	365898057
Sma-1-1117	NW_003037797.1	-	1990223	1990174
Sma-1-1118	NS_000200.1	-	12418083	12418034
Sma-1-1119	NW_003038139.1	+	9465	9514
Sma-1-1120	NS_000200.1	+	286049595	286049644
Sma-1-1121	NW_003037915.1	-	108059	108010
Sma-1-1122	NS_000200.1	-	162008045	162007996
Sma-1-1123	NW_003037825.1	+	1374253	1374302
Sma-1-1124	NS_000200.1	+	60504035	60504084
Sma-1-1125	NW_003038072.1	-	320697	320648
Sma-1-1126	NS_000200.1	-	265482925	265482876
Sma-1-1127	NW_003037825.1	+	1338856	1338905
Sma-1-1128	NS_000200.1	+	60468638	60468687
Sma-1-1129	NW_003038142.1	-	4051	4002
Sma-1-1130	NS_000200.1	-	287214487	287214438
Sma-1-1131	NW_003038024.1	+	782487	782536
Sma-1-1132	NS_000200.1	+	238679516	238679565
Sma-1-1133	NW_003037965.1	-	217293	217244
Sma-1-1134	NS_000200.1	-	198506378	198506329
Sma-1-1135	NW_003038025.1	-	107085	107036
Sma-1-1136	NS_000200.1	-	238965330	238965281
Sma-1-1137	NW_003037964.1	-	571963	571914
Sma-1-1138	NS_000200.1	-	198173485	198173436
Sma-1-1139	NW_003038025.1	-	285052	285003
Sma-1-1140	NW_003038246.1	+	226185	226234
Sma-1-1141	NS_000200.1	+	315867313	315867362
Sma-1-1142	NS_000200.1	-	239143297	239143248
Sma-1-1143	NW_003037926.1	+	287394	287443
Sma-1-1144	NW_003037926.1	-	288315	288266
Sma-1-1145	NS_000200.1	+	173455664	173455713
Sma-1-1146	NS_000200.1	-	173456585	173456536
Sma-1-1147	NW_003038240.1	+	113984	114033
Sma-1-1148	NS_000200.1	+	314252915	314252964
Sma-1-1149	NW_003037860.1	+	317949	317998
Sma-1-1150	NS_000200.1	+	104060977	104061026
Sma-1-1151	NW_003038145.1	+	7233	7282
Sma-1-1152	NS_000200.1	+	288618656	288618705
Sma-1-1153	NW_003038244.1	-	131181	131132
Sma-1-1154	NS_000200.1	-	315276386	315276337

Sma-1-1155	NW_003038026.1	-	676430	676381
Sma-1-1156	NS_000200.1	-	240184191	240184142
Sma-1-1157	NW_003038053.1	+	228964	229013
Sma-1-1158	NS_000200.1	+	257589059	257589108
Sma-1-1159	NW_003037962.1	+	478486	478536
Sma-1-1160	NS_000200.1	+	196555329	196555379
Sma-1-1161	NW_003038027.1	-	320813	320764
Sma-1-1162	NS_000200.1	-	240691527	240691478
Sma-1-1163	NW_003038027.1	-	39195	39146
Sma-1-1164	NS_000200.1	-	240409909	240409860
Sma-1-1165	NW_003037860.1	-	798314	798265
Sma-1-1166	NS_000200.1	-	104541342	104541293
Sma-1-1167	NW_003037937.1	+	566334	566383
Sma-1-1168	NS_000200.1	+	181766243	181766292
Sma-1-1169	NW_003031050.1	-	1163	1114
Sma-1-1170	NS_000200.1	-	325601478	325601429
Sma-1-1171	NW_003038028.1	+	544752	544801
Sma-1-1172	NS_000200.1	+	241852326	241852375
Sma-1-1173	NW_003037962.1	-	25133	25084
Sma-1-1174	NS_000200.1	-	196101976	196101927
Sma-1-1175	NW_003037797.1	+	295487	295536
Sma-1-1176	NS_000200.1	+	10723347	10723396
Sma-1-1177	NW_003037862.1	-	829554	829505
Sma-1-1178	NS_000200.1	-	106481215	106481166
Sma-1-1179	NW_003037863.1	-	1093526	1093477
Sma-1-1180	NS_000200.1	-	107851893	107851844
Sma-1-1181	NW_003039127.1	+	530675	530724
Sma-1-1182	NS_000200.1	+	113608174	113608223
Sma-1-1183	NW_003037911.1	+	772510	772559
Sma-1-1184	NS_000200.1	+	156559863	156559912
Sma-1-1185	NW_003038238.1	+	102163	102212
Sma-1-1186	NS_000200.1	+	313708944	313708993
Sma-1-1187	NW_003038030.1	+	384815	384864
Sma-1-1188	NS_000200.1	+	243520682	243520731
Sma-1-1189	NW_003037812.1	+	930065	930114
Sma-1-1190	NS_000200.1	+	40412804	40412853
Sma-1-1191	NW_003038030.1	+	640622	640671
Sma-1-1192	NS_000200.1	+	243776489	243776538
Sma-1-1193	NW_003038253.1	+	213757	213806
Sma-1-1194	NS_000200.1	+	319531554	319531603
Sma-1-1195	NW_003037953.1	-	252464	252414
Sma-1-1196	NS_000200.1	-	193165515	193165465
Sma-1-1197	NW_003037822.1	-	1495518	1495469
Sma-1-1198	NS_000200.1	-	55803524	55803475
Sma-1-1199	NW_003037886.1	+	452737	452786
Sma-1-1200	NS_000200.1	+	129708304	129708353
Sma-1-1201	NW_003033835.1	+	5493	5542
Sma-1-1202	NS_000200.1	+	359231235	359231284
Sma-1-1203	NW_003038151.1	-	221067	221018
Sma-1-1204	NS_000200.1	-	292128310	292128261
Sma-1-1205	NW_003038151.1	-	226810	226761
Sma-1-1206	NS_000200.1	-	292134053	292134004

Sma-1-1207	NW_003037794.1	+	1814090	1814139
Sma-1-1208	NS_000200.1	+	5993685	5993734
Sma-1-1209	NW_003038151.1	-	609280	609231
Sma-1-1210	NS_000200.1	-	292516523	292516474
Sma-1-1211	NW_003034096.1	+	69	118
Sma-1-1212	NS_000200.1	+	363729277	363729326
Sma-1-1213	NW_003037811.1	+	97742	97791
Sma-1-1214	NS_000200.1	+	38267984	38268033
Sma-1-1215	NW_003034560.1	+	4296	4345
Sma-1-1216	NW_003037818.1	+	149565	149614
Sma-1-1217	NS_000200.1	+	48864972	48865021
Sma-1-1218	NS_000200.1	+	327581754	327581803
Sma-1-1219	NW_003037821.1	+	329184	329233
Sma-1-1220	NS_000200.1	+	53473668	53473717
Sma-1-1221	NW_003037821.1	-	1120230	1120181
Sma-1-1222	NS_000200.1	-	54264714	54264665
Sma-1-1223	NW_003037946.1	-	483376	483327
Sma-1-1224	NS_000200.1	-	191177236	191177187
Sma-1-1225	NW_003039129.1	+	182272	182321
Sma-1-1226	NW_003039129.1	+	193918	193967
Sma-1-1227	NS_000200.1	+	229137080	229137129
Sma-1-1228	NS_000200.1	+	229148726	229148775
Sma-1-1229	NW_003038518.1	+	482171	482220
Sma-1-1230	NS_000200.1	+	342119004	342119053
Sma-1-1231	NW_003035641.1	+	18274	18323
Sma-1-1232	NS_000200.1	+	330255652	330255701
Sma-1-1233	NW_003037869.1	-	110342	110293
Sma-1-1234	NS_000200.1	-	116582687	116582638
Sma-1-1235	NW_003035869.1	-	165614	165565
Sma-1-1236	NS_000200.1	-	335349814	335349765
Sma-1-1237	NW_003037819.1	-	521153	521104
Sma-1-1238	NS_000200.1	-	51609418	51609369
Sma-1-1239	NW_003035876.1	+	135126	135175
Sma-1-1240	NS_000200.1	+	336966626	336966675
Sma-1-1241	NW_003037912.1	-	321586	321537
Sma-1-1242	NS_000200.1	-	157133161	157133112
Sma-1-1243	NW_003038035.1	-	163451	163402
Sma-1-1244	NS_000200.1	-	248024943	248024894
Sma-1-1245	NW_003037807.1	-	593337	593288
Sma-1-1246	NS_000200.1	-	30854853	30854804
Sma-1-1247	NW_003035880.1	+	256431	256480
Sma-1-1248	NS_000200.1	+	339497495	339497544
Sma-1-1249	NW_003038369.1	+	7932	7981
Sma-1-1250	NS_000200.1	+	327116871	327116920
Sma-1-1251	NW_003037796.1	-	1892092	1892043
Sma-1-1252	NS_000200.1	-	10101736	10101687
Sma-1-1253	NW_003037874.1	-	312460	312411
Sma-1-1254	NS_000200.1	-	119737410	119737361
Sma-1-1255	NW_003037912.1	+	489525	489574
Sma-1-1256	NS_000200.1	+	157301100	157301149
Sma-1-1257	NW_003038104.1	+	34395	34444
Sma-1-1258	NS_000200.1	+	274930411	274930460

Sma-1-1259	NW_003037880.1	-	638752	638703
Sma-1-1260	NS_000200.1	-	124322310	124322261
Sma-1-1261	NW_003038038.1	+	782667	782716
Sma-1-1262	NS_000200.1	+	252686060	252686109
Sma-1-1263	NW_003026470.1	+	434228	434277
Sma-1-1264	NS_000200.1	+	219558197	219558246
Sma-1-1265	NW_003037876.1	+	481325	481374
Sma-1-1266	NS_000200.1	+	121026961	121027010
Sma-1-1267	NW_003037882.1	+	233866	233915
Sma-1-1268	NW_003038259.1	-	256583	256534
Sma-1-1269	NS_000200.1	+	125525540	125525589
Sma-1-1270	NS_000200.1	-	323117182	323117133
Sma-1-1271	NW_003038055.1	+	9888	9937
Sma-1-1272	NS_000200.1	+	258709210	258709259
Sma-1-1273	NW_003037800.1	-	1311152	1311103
Sma-1-1274	NS_000200.1	-	19940512	19940463
Sma-1-1275	NW_003037924.1	-	139563	139514
Sma-1-1276	NS_000200.1	-	171846521	171846472
Sma-1-1277	NW_003037800.1	-	637253	637204
Sma-1-1278	NS_000200.1	-	19266613	19266564
Sma-1-1279	NW_003037917.1	-	1267331	1267282
Sma-1-1280	NS_000200.1	-	166304135	166304086
Sma-1-1281	NW_003037802.1	-	1021636	1021587
Sma-1-1282	NS_000200.1	-	23024430	23024381
Sma-1-1283	NW_003037803.1	+	1143974	1144023
Sma-1-1284	NS_000200.1	+	24644511	24644560
Sma-1-1285	NW_003037807.1	-	966160	966111
Sma-1-1286	NS_000200.1	-	31227676	31227627
Sma-1-1287	NW_003037814.1	-	416734	416685
Sma-1-1288	NS_000200.1	-	43400594	43400545
Sma-1-1289	NW_003037939.1	+	218579	218628
Sma-1-1290	NS_000200.1	+	183088333	183088382
Sma-1-1291	NW_003037890.1	+	1573556	1573605
Sma-1-1292	NW_003038084.1	-	110550	110501
Sma-1-1293	NS_000200.1	+	137256917	137256966
Sma-1-1294	NS_000200.1	-	271125794	271125745
Sma-1-1295	NW_003037891.1	+	1375297	1375346
Sma-1-1296	NS_000200.1	+	139256575	139256624
Sma-1-1297	NW_003037900.1	+	697952	698001
Sma-1-1298	NS_000200.1	+	145826818	145826867
Sma-1-1299	NW_003037834.1	-	1118612	1118563
Sma-1-1300	NS_000200.1	-	74279098	74279049
Sma-1-1301	NW_003037833.1	+	820105	820154
Sma-1-1302	NW_003038023.1	+	261903	261952
Sma-1-1303	NS_000200.1	+	73011201	73011250
Sma-1-1304	NS_000200.1	+	237422194	237422243
Sma-1-1305	NW_003037903.1	+	920292	920341
Sma-1-1306	NS_000200.1	+	148474373	148474422
Sma-1-1307	NW_003037903.1	+	944862	944911
Sma-1-1308	NS_000200.1	+	148498943	148498992
Sma-1-1309	NW_003037831.1	-	1686339	1686290
Sma-1-1310	NS_000200.1	-	71135641	71135592

Sma-1-1311	NW_003037930.1	-	909125	909076
Sma-1-1312	NS_000200.1	-	176767089	176767040
Sma-1-1313	NW_003037846.1	+	111432	111481
Sma-1-1314	NS_000200.1	+	87746002	87746051
Sma-1-1315	NW_003037814.1	-	521561	521512
Sma-1-1316	NS_000200.1	-	43505421	43505372
Sma-1-1317	NW_003037914.1	+	1270260	1270309
Sma-1-1318	NW_003037932.1	+	443293	443342
Sma-1-1319	NS_000200.1	+	161414510	161414559
Sma-1-1320	NS_000200.1	+	177976489	177976538
Sma-1-1321	NW_003037918.1	-	33169	33120
Sma-1-1322	NS_000200.1	-	166600159	166600110
Sma-1-1323	NW_003037859.1	+	293814	293863
Sma-1-1324	NS_000200.1	+	102972655	102972704
Sma-1-1325	NW_003037799.1	-	1173877	1173828
Sma-1-1326	NW_003037859.1	-	265733	265684
Sma-1-1327	NW_003037979.1	+	465403	465452
Sma-1-1328	NS_000200.1	+	210526423	210526472
Sma-1-1329	NS_000200.1	-	102944574	102944525
Sma-1-1330	NS_000200.1	-	18164231	18164182
Sma-1-1331	NW_003037933.1	-	426070	426021
Sma-1-1332	NS_000200.1	-	178839242	178839193
Sma-1-1333	NW_003037938.1	+	423869	423918
Sma-1-1334	NS_000200.1	+	182460982	182461031
Sma-1-1335	NW_003030922.1	+	576	625
Sma-1-1336	NS_000200.1	+	370524338	370524387
Sma-1-1337	NW_003026487.1	-	122798	122749
Sma-1-1338	NS_000200.1	-	261033492	261033443
Sma-1-1339	NW_003038020.1	-	702444	702395
Sma-1-1340	NS_000200.1	-	234669788	234669739
Sma-1-1341	NW_003037801.1	+	871260	871309
Sma-1-1342	NS_000200.1	+	21960740	21960789
Sma-1-1343	NW_003038008.1	-	317333	317284
Sma-1-1344	NS_000200.1	-	225990429	225990380
Sma-1-1345	NW_003038028.1	-	621800	621751
Sma-1-1346	NS_000200.1	-	241929374	241929325
Sma-1-1347	NW_003037889.1	+	1857913	1857962
Sma-1-1348	NS_000200.1	+	135634403	135634452
Sma-1-1349	NW_003037891.1	-	2839401	2839352
Sma-1-1350	NS_000200.1	-	140720679	140720630
Sma-1-1351	NW_003038073.1	-	55892	55840
Sma-1-1352	NS_000200.1	-	265578325	265578273
Sma-1-1353	NW_003038084.1	+	232186	232235
Sma-1-1354	NS_000200.1	+	271247430	271247479
Sma-1-1355	NW_003038253.1	+	72202	72251
Sma-1-1356	NW_003038258.1	-	213425	213376
Sma-1-1357	NW_003026615.1	+	96685	96734
Sma-1-1358	NW_003026458.1	+	176755	176804
Sma-1-1359	NW_003037794.1	+	448645	448694
Sma-1-1360	NW_003037800.1	-	1510807	1510758
Sma-1-1361	NW_003037827.1	-	363224	363175
Sma-1-1362	NW_003037839.1	-	978703	978654

Sma-1-1363	NW_003037839.1	-	894723	894674
Sma-1-1364	NW_003037918.1	+	1013603	1013652
Sma-1-1365	NW_003037946.1	-	1136325	1136276
Sma-1-1366	NW_003037963.1	+	231099	231148
Sma-1-1367	NW_003038003.1	-	135437	135388
Sma-1-1368	NW_003038031.1	+	693941	693990
Sma-1-1369	NW_003038035.1	+	585837	585886
Sma-1-1370	NW_003038069.1	-	59325	59276
Sma-1-1371	NW_003038261.1	+	20127	20176
Sma-1-1372	NW_003026488.1	-	300098	300049
Sma-1-1373	NS_000200.1	+	4628240	4628289
Sma-1-1374	NS_000200.1	+	102044985	102045034
Sma-1-1375	NS_000200.1	+	167580593	167580642
Sma-1-1376	NS_000200.1	+	197509311	197509360
Sma-1-1377	NS_000200.1	+	244971699	244971748
Sma-1-1378	NS_000200.1	+	248447329	248447378
Sma-1-1379	NS_000200.1	+	317091126	317091175
Sma-1-1380	NS_000200.1	+	319389999	319390048
Sma-1-1381	NS_000200.1	+	323633778	323633827
Sma-1-1382	NS_000200.1	-	322751935	322751886
Sma-1-1383	NS_000200.1	-	263902731	263902682
Sma-1-1384	NS_000200.1	-	263806592	263806543
Sma-1-1385	NS_000200.1	-	223634719	223634670
Sma-1-1386	NS_000200.1	-	191830185	191830136
Sma-1-1387	NS_000200.1	-	79936853	79936804
Sma-1-1388	NS_000200.1	-	79852873	79852824
Sma-1-1389	NS_000200.1	-	62985365	62985316
Sma-1-1390	NS_000200.1	-	20140167	20140118
Sma-1-1391	NW_003026456.1	-	283357	283308
Sma-1-1392	NS_000200.1	-	58206493	58206444
Sma-1-1393	NW_003037795.1	+	149107	149156
Sma-1-1394	NS_000200.1	+	6539990	6540039
Sma-1-1395	NW_003037930.1	+	580862	580911
Sma-1-1396	NW_003037930.1	-	580013	579964
Sma-1-1397	NS_000200.1	+	176438826	176438875
Sma-1-1398	NS_000200.1	-	176437977	176437928
Sma-1-1399	NW_003037794.1	+	227757	227806
Sma-1-1400	NS_000200.1	+	4407352	4407401
Sma-1-1401	NW_003037940.1	+	947378	947427
Sma-1-1402	NS_000200.1	+	184689508	184689557
Sma-1-1403	NW_003037978.1	-	282973	282924
Sma-1-1404	NS_000200.1	-	209550983	209550934
Sma-1-1405	NW_003037945.1	+	1292593	1292642
Sma-1-1406	NS_000200.1	+	190394557	190394606
Sma-1-1407	NW_003022908.1	-	948	899
Sma-1-1408	NS_000200.1	-	371859929	371859880
Sma-1-1409	NW_003037830.1	-	1503869	1503820
Sma-1-1410	NW_003037918.1	-	1644177	1644128
Sma-1-1411	NS_000200.1	-	168211167	168211118
Sma-1-1412	NS_000200.1	-	69394240	69394191
Sma-1-1413	NW_003037799.1	+	738237	738286
Sma-1-1414	NS_000200.1	+	17728591	17728640

Sma-1-1415	NW_003033033.1	-	2831	2782
Sma-1-1416	NW_003037851.1	+	512895	512944
Sma-1-1417	NS_000200.1	+	93916205	93916254
Sma-1-1418	NS_000200.1	-	326239284	326239235
Sma-1-1419	NW_003037967.1	-	160299	160250
Sma-1-1420	NS_000200.1	-	199400270	199400221
Sma-1-1421	NW_003038244.1	-	211206	211157
Sma-1-1422	NS_000200.1	-	315356411	315356362
Sma-1-1423	NW_003037914.1	-	1703691	1703642
Sma-1-1424	NW_003038025.1	-	74491	74442
Sma-1-1425	NS_000200.1	-	238932736	238932687
Sma-1-1426	NS_000200.1	-	161847941	161847892
Sma-1-1427	NW_003037818.1	-	2072319	2072270
Sma-1-1428	NS_000200.1	-	50787726	50787677
Sma-1-1429	NW_003037793.1	+	2547084	2547133
Sma-1-1430	NS_000200.1	+	2547084	2547133
Sma-1-1431	NW_003027917.1	-	219	170
Sma-1-1432	NW_003037796.1	-	443968	443919
Sma-1-1433	NS_000200.1	-	366149935	366149886
Sma-1-1434	NS_000200.1	-	8653612	8653563
Sma-1-1435	NW_003026462.1	-	475233	475184
Sma-1-1436	NS_000200.1	-	197142736	197142687
Sma-1-1437	NW_003037824.1	+	780141	780190
Sma-1-1438	NW_003037824.1	-	148709	148660
Sma-1-1439	NS_000200.1	+	57711081	57711130
Sma-1-1440	NS_000200.1	-	57079649	57079600
Sma-1-1441	NW_003037826.1	+	617521	617570
Sma-1-1442	NS_000200.1	+	61617037	61617086
Sma-1-1443	NW_003037915.1	-	294743	294694
Sma-1-1444	NS_000200.1	-	162194729	162194680
Sma-1-1445	NW_003037924.1	+	605505	605554
Sma-1-1446	NS_000200.1	+	172312463	172312512
Sma-1-1447	NW_003037938.1	+	491829	491878
Sma-1-1448	NS_000200.1	+	182528942	182528991
Sma-1-1449	NW_003038069.1	-	370606	370557
Sma-1-1450	NS_000200.1	-	264214012	264213963
Sma-1-1451	NW_003037888.1	+	832246	832295
Sma-1-1452	NS_000200.1	+	133040738	133040787
Sma-1-1453	NW_003037973.1	-	31785	31736
Sma-1-1454	NS_000200.1	-	204013512	204013463
Sma-1-1455	NW_003038010.1	-	411093	411044
Sma-1-1456	NS_000200.1	-	227229316	227229267
Sma-1-1457	NW_003038137.1	+	216161	216210
Sma-1-1458	NS_000200.1	+	285005057	285005106
Sma-1-1459	NW_003038232.1	+	335678	335727
Sma-1-1460	NS_000200.1	+	312299469	312299518
Sma-1-1461	NW_003026456.1	-	285089	285040
Sma-1-1462	NW_003037914.1	-	1250909	1250860
Sma-1-1463	NW_003038015.1	-	201800	201751
Sma-1-1464	NS_000200.1	-	230525290	230525241
Sma-1-1465	NS_000200.1	-	161395159	161395110
Sma-1-1466	NS_000200.1	-	58208225	58208176

Sma-1-1467	NW_003037977.1	-	655095	655046
Sma-1-1468	NS_000200.1	-	208941115	208941066
Sma-1-1469	NW_003038015.1	-	164138	164089
Sma-1-1470	NS_000200.1	-	230487628	230487579
Sma-1-1471	NW_003038148.1	-	89824	89775
Sma-1-1472	NS_000200.1	-	290163808	290163759
Sma-1-1473	NW_003038015.1	-	549843	549794
Sma-1-1474	NS_000200.1	-	230873333	230873284
Sma-1-1475	NW_003037845.1	+	13796	13845
Sma-1-1476	NS_000200.1	+	87015699	87015748
Sma-1-1477	NW_003038037.1	+	851159	851208
Sma-1-1478	NS_000200.1	+	251006568	251006617
Sma-1-1479	NW_003038196.1	+	15398	15447
Sma-1-1480	NS_000200.1	+	303157681	303157730
Sma-1-1481	NW_003037810.1	-	170332	170283
Sma-1-1482	NS_000200.1	-	36774854	36774805
Sma-1-1483	NW_003037981.1	-	984726	984677
Sma-1-1484	NS_000200.1	-	213382594	213382545
Sma-1-1485	NW_003037973.1	-	71341	71292
Sma-1-1486	NS_000200.1	-	204053068	204053019
Sma-1-1487	NW_003037913.1	+	887852	887901
Sma-1-1488	NS_000200.1	+	159451603	159451652
Sma-1-1489	NW_003039130.1	-	163397	163348
Sma-1-1490	NS_000200.1	-	317838008	317837959
Sma-1-1491	NW_003038260.1	+	283258	283307
Sma-1-1492	NS_000200.1	+	323455033	323455082
Sma-1-1493	NW_003037812.1	+	948266	948315
Sma-1-1494	NS_000200.1	+	40431005	40431054
Sma-1-1495	NW_003037809.1	+	881566	881615
Sma-1-1496	NS_000200.1	+	34546030	34546079
Sma-1-1497	NW_003037809.1	-	1503197	1503148
Sma-1-1498	NS_000200.1	-	35167661	35167612
Sma-1-1499	NW_003037808.1	+	237436	237485
Sma-1-1500	NS_000200.1	+	32024073	32024122
Sma-1-1501	NW_003037799.1	+	1093628	1093677
Sma-1-1502	NS_000200.1	+	18083982	18084031
Sma-1-1503	NW_003026456.1	+	242687	242736
Sma-1-1504	NS_000200.1	+	58165823	58165872
Sma-1-1505	NW_003037982.1	+	860137	860186
Sma-1-1506	NS_000200.1	+	214519332	214519381
Sma-1-1507	NW_003037943.1	+	604182	604231
Sma-1-1508	NS_000200.1	+	187625715	187625764
Sma-1-1509	NW_003037913.1	-	1326085	1326036
Sma-1-1510	NS_000200.1	-	159889836	159889787
Sma-1-1511	NW_003037785.1	+	84201	84250
Sma-1-1512	NS_000200.1	+	379982868	379982917
Sma-1-1513	NW_003037799.1	+	912715	912764
Sma-1-1514	NW_003037883.1	-	139074	139025
Sma-1-1515	NS_000200.1	+	17903069	17903118
Sma-1-1516	NS_000200.1	-	126202797	126202748
Sma-1-1517	NW_003037958.1	+	328356	328405
Sma-1-1518	NS_000200.1	+	194992540	194992589

Sma-1-1519	NW_003037964.1	-	39479	39430
Sma-1-1520	NS_000200.1	-	197641001	197640952
Sma-1-1521	NW_003037964.1	-	425668	425619
Sma-1-1522	NS_000200.1	-	198027190	198027141
Sma-1-1523	NW_003027978.1	-	750	701
Sma-1-1524	NW_003038148.1	+	398236	398285
Sma-1-1525	NS_000200.1	+	290472220	290472269
Sma-1-1526	NS_000200.1	-	369105026	369104977
Sma-1-1527	NW_003022658.1	+	34	83
Sma-1-1528	NW_003037822.1	+	236759	236808
Sma-1-1529	NW_003037905.1	+	497398	497447
Sma-1-1530	NW_003038037.1	-	732426	732377
Sma-1-1531	NS_000200.1	+	54544765	54544814
Sma-1-1532	NS_000200.1	+	149766410	149766459
Sma-1-1533	NS_000200.1	+	365367852	365367901
Sma-1-1534	NS_000200.1	-	250887835	250887786
Sma-1-1535	NW_003026465.1	+	246387	246436
Sma-1-1536	NS_000200.1	+	207638981	207639030
Sma-1-1537	NW_003038135.1	+	228603	228652
Sma-1-1538	NS_000200.1	+	284100942	284100991
Sma-1-1539	NW_003038028.1	-	683466	683417
Sma-1-1540	NS_000200.1	-	241991040	241990991
Sma-1-1541	NW_003038032.1	-	445762	445713
Sma-1-1542	NS_000200.1	-	245604922	245604873
Sma-1-1543	NW_003038509.1	+	92048	92097
Sma-1-1544	NW_003037934.1	-	132257	132208
Sma-1-1545	NW_003037793.1	+	757979	758028
Sma-1-1546	NW_003037928.1	-	72546	72497
Sma-1-1547	NW_003037943.1	-	1164722	1164673
Sma-1-1548	NW_003037981.1	+	831813	831862
Sma-1-1549	NW_003037989.1	+	169925	169974
Sma-1-1550	NW_003038007.1	+	371681	371730
Sma-1-1551	NW_003038015.1	+	264091	264140
Sma-1-1552	NW_003038238.1	-	104099	104050
Sma-1-1553	NW_003038099.1	+	3688	3737
Sma-1-1554	NW_003038127.1	+	200472	200521
Sma-1-1555	NS_000200.1	+	757979	758028
Sma-1-1556	NS_000200.1	+	213229681	213229730
Sma-1-1557	NS_000200.1	+	216780484	216780533
Sma-1-1558	NS_000200.1	+	225620236	225620285
Sma-1-1559	NS_000200.1	+	230587581	230587630
Sma-1-1560	NS_000200.1	+	274461901	274461950
Sma-1-1561	NS_000200.1	+	280592178	280592227
Sma-1-1562	NS_000200.1	+	337724500	337724549
Sma-1-1563	NS_000200.1	-	313710880	313710831
Sma-1-1564	NS_000200.1	-	188186255	188186206
Sma-1-1565	NS_000200.1	-	179179004	179178955
Sma-1-1566	NS_000200.1	-	174396499	174396450
Sma-1-1567	NW_003038441.1	-	3501	3452
Sma-1-1568	NS_000200.1	-	329750873	329750824
Sma-1-1569	NW_003037801.1	-	580141	580092
Sma-1-1570	NS_000200.1	-	21669621	21669572

Sma-1-1571	NW_003037928.1	+	553209	553258
Sma-1-1572	NS_000200.1	+	174877162	174877211
Sma-1-1573	NW_003037971.1	+	269047	269096
Sma-1-1574	NS_000200.1	+	201891097	201891146
Sma-1-1575	NW_003026517.1	+	129541	129590
Sma-1-1576	NS_000200.1	+	299735550	299735599
Sma-1-1577	NW_003038033.1	+	949242	949291
Sma-1-1578	NS_000200.1	+	246929244	246929293
Sma-1-1579	NW_003038505.1	+	257941	257990
Sma-1-1580	NS_000200.1	+	336337278	336337327
Sma-1-1581	NW_003037839.1	-	75147	75098
Sma-1-1582	NW_003038126.1	+	375352	375401
Sma-1-1583	NS_000200.1	+	280385764	280385813
Sma-1-1584	NS_000200.1	-	79033297	79033248
Sma-1-1585	NW_003037796.1	-	2113407	2113358
Sma-1-1586	NW_003037886.1	-	443847	443798
Sma-1-1587	NS_000200.1	-	129699414	129699365
Sma-1-1588	NS_000200.1	-	10323051	10323002
Sma-1-1589	NW_003037933.1	-	77587	77538
Sma-1-1590	NS_000200.1	-	178490759	178490710
Sma-1-1591	NW_003036918.1	+	1431	1480
Sma-1-1592	NS_000200.1	+	343396144	343396193
Sma-1-1593	NW_003037952.1	+	39217	39266
Sma-1-1594	NW_003037804.1	+	698705	698754
Sma-1-1595	NS_000200.1	+	25838725	25838774
Sma-1-1596	NS_000200.1	+	192859350	192859399
Sma-1-1597	NW_003037973.1	+	427749	427798
Sma-1-1598	NS_000200.1	+	204409476	204409525
Sma-1-1599	NW_003038251.1	+	480919	480968
Sma-1-1600	NS_000200.1	+	318812691	318812740
Sma-1-1601	NW_003038153.1	+	693273	693322
Sma-1-1602	NS_000200.1	+	293755322	293755371
Sma-1-1603	NW_003038056.1	+	110879	110928
Sma-1-1604	NS_000200.1	+	259088530	259088579
Sma-1-1605	NW_003038031.1	+	199879	199928
Sma-1-1606	NS_000200.1	+	244477637	244477686
Sma-1-1607	NW_003037817.1	-	457337	457288
Sma-1-1608	NS_000200.1	-	47839689	47839640
Sma-1-1609	NW_003037832.1	+	451956	452005
Sma-1-1610	NS_000200.1	+	71661387	71661436
Sma-1-1611	NW_003037853.1	+	756115	756164
Sma-1-1612	NS_000200.1	+	96248166	96248215
Sma-1-1613	NW_003037918.1	+	137607	137656
Sma-1-1614	NS_000200.1	+	166704597	166704646
Sma-1-1615	NW_003038010.1	-	268980	268931
Sma-1-1616	NS_000200.1	-	227087203	227087154
Sma-1-1617	NW_003038511.1	+	201279	201328
Sma-1-1618	NS_000200.1	+	338546491	338546540
Sma-1-1619	NW_003033994.1	+	929	978
Sma-1-1620	NS_000200.1	+	363378517	363378566
Sma-1-1621	NW_003037859.1	+	876521	876570
Sma-1-1622	NS_000200.1	+	103555362	103555411

Sma-1-1623	NW_003037863.1	+	838175	838224
Sma-1-1624	NW_003038037.1	+	1152941	1152990
Sma-1-1625	NS_000200.1	+	107596542	107596591
Sma-1-1626	NS_000200.1	+	251308350	251308399
Sma-1-1627	NW_003037865.1	+	606578	606627
Sma-1-1628	NW_003038067.1	+	122467	122516
Sma-1-1629	NS_000200.1	+	111028497	111028546
Sma-1-1630	NS_000200.1	+	262976520	262976569
Sma-1-1631	NW_003037871.1	-	384370	384321
Sma-1-1632	NS_000200.1	-	117832108	117832059
Sma-1-1633	NW_003038068.1	-	274743	274694
Sma-1-1634	NS_000200.1	-	263341951	263341902
Sma-1-1635	NW_003032235.1	+	1799	1848
Sma-1-1636	NW_003038258.1	+	279284	279333
Sma-1-1637	NS_000200.1	+	322817794	322817843
Sma-1-1638	NS_000200.1	+	357970176	357970225
Sma-1-1639	NW_003037940.1	-	39446	39397
Sma-1-1640	NW_003038053.1	-	408159	408110
Sma-1-1641	NW_003038161.1	-	267276	267227
Sma-1-1642	NS_000200.1	-	297637127	297637078
Sma-1-1643	NS_000200.1	-	257768254	257768205
Sma-1-1644	NS_000200.1	-	183781576	183781527
Sma-1-1645	NW_003037884.1	+	1250073	1250122
Sma-1-1646	NS_000200.1	+	128029738	128029787
Sma-1-1647	NW_003037796.1	+	400918	400966
Sma-1-1648	NS_000200.1	+	8610562	8610610
Sma-1-1649	NW_003037877.1	+	559038	559087
Sma-1-1650	NS_000200.1	+	121597600	121597649
Sma-1-1651	NW_003037877.1	+	538456	538505
Sma-1-1652	NS_000200.1	+	121577018	121577067
Sma-1-1653	NW_003038038.1	-	1142564	1142515
Sma-1-1654	NS_000200.1	-	253045957	253045908
Sma-1-1655	NW_003037818.1	-	1720656	1720607
Sma-1-1656	NS_000200.1	-	50436063	50436014
Sma-1-1657	NW_003037880.1	-	602588	602539
Sma-1-1658	NS_000200.1	-	124286146	124286097
Sma-1-1659	NS_000200.1	+	348664654	348664703
Sma-1-1660	NW_003037785.1	+	85401	85450
Sma-1-1661	NS_000200.1	+	379984068	379984117
Sma-1-1662	NW_003037787.1	+	91613	91662
Sma-1-1663	NS_000200.1	+	380339782	380339831
Sma-1-1664	NW_003037808.1	-	681640	681591
Sma-1-1665	NS_000200.1	-	32468277	32468228
Sma-1-1666	NW_003037863.1	-	657377	657328
Sma-1-1667	NS_000200.1	-	107415744	107415695
Sma-1-1668	NW_003037799.1	+	765165	765214
Sma-1-1669	NS_000200.1	+	17755519	17755568
Sma-1-1670	NW_003037788.1	-	221962	221913
Sma-1-1671	NS_000200.1	-	380669200	380669151
Sma-1-1672	NW_003037809.1	-	1783306	1783257
Sma-1-1673	NS_000200.1	-	35447770	35447721
Sma-1-1674	NW_003037800.1	-	1408400	1408351

Sma-1-1675	NS_000200.1	-	20037760	20037711
Sma-1-1676	NW_003037914.1	+	1185081	1185130
Sma-1-1677	NS_000200.1	+	161329331	161329380
Sma-1-1678	NW_003037803.1	-	454699	454650
Sma-1-1679	NS_000200.1	-	23955236	23955187
Sma-1-1680	NW_003038244.1	-	52103	52054
Sma-1-1681	NS_000200.1	-	315197308	315197259
Sma-1-1682	NW_003037813.1	+	733048	733097
Sma-1-1683	NS_000200.1	+	41719911	41719960
Sma-1-1684	NW_003037761.1	-	14281	14232
Sma-1-1685	NS_000200.1	-	377640427	377640378
Sma-1-1686	NW_003037797.1	+	2866010	2866059
Sma-1-1687	NS_000200.1	+	13293870	13293919
Sma-1-1688	NW_003037815.1	+	369540	369589
Sma-1-1689	NS_000200.1	+	45632531	45632580
Sma-1-1690	NW_003037882.1	-	268072	268023
Sma-1-1691	NS_000200.1	-	125559746	125559697
Sma-1-1692	NW_003037886.1	+	214242	214291
Sma-1-1693	NS_000200.1	+	129469809	129469858
Sma-1-1694	NW_003037830.1	-	1454761	1454712
Sma-1-1695	NS_000200.1	-	69345132	69345083
Sma-1-1696	NW_003037884.1	+	1054022	1054071
Sma-1-1697	NS_000200.1	+	127833687	127833736
Sma-1-1698	NW_003037887.1	+	648223	648272
Sma-1-1699	NS_000200.1	+	131112201	131112250
Sma-1-1700	NW_003037859.1	+	712916	712965
Sma-1-1701	NS_000200.1	+	103391757	103391806
Sma-1-1702	NW_003037822.1	-	983813	983764
Sma-1-1703	NS_000200.1	-	55291819	55291770
Sma-1-1704	NW_003037836.1	+	321663	321712
Sma-1-1705	NS_000200.1	+	75878762	75878811
Sma-1-1706	NW_003037851.1	-	787886	787837
Sma-1-1707	NS_000200.1	-	94191196	94191147
Sma-1-1708	NW_003037891.1	+	593631	593680
Sma-1-1709	NS_000200.1	+	138474909	138474958
Sma-1-1710	NW_003037841.1	-	1976130	1976081
Sma-1-1711	NS_000200.1	-	84587311	84587262
Sma-1-1712	NW_003037899.1	+	233550	233599
Sma-1-1713	NS_000200.1	+	144752661	144752710
Sma-1-1714	NW_003037837.1	-	449535	449486
Sma-1-1715	NS_000200.1	-	77255393	77255344
Sma-1-1716	NW_003037834.1	-	191861	191812
Sma-1-1717	NS_000200.1	-	73352347	73352298
Sma-1-1718	NW_003037833.1	+	77974	78023
Sma-1-1719	NS_000200.1	+	72269070	72269119
Sma-1-1720	NW_003037831.1	-	1292750	1292700
Sma-1-1721	NS_000200.1	-	70742052	70742002
Sma-1-1722	NW_003037905.1	-	69628	69579
Sma-1-1723	NS_000200.1	-	149338640	149338591
Sma-1-1724	NW_003038516.1	-	184786	184737
Sma-1-1725	NS_000200.1	-	340673191	340673142
Sma-1-1726	NW_003037825.1	+	90396	90445

Sma-1-1727	NS_000200.1	+	59220178	59220227
Sma-1-1728	NW_003037824.1	+	981756	981805
Sma-1-1729	NS_000200.1	+	57912696	57912745
Sma-1-1730	NW_003037885.1	-	733380	733331
Sma-1-1731	NS_000200.1	-	128831327	128831278
Sma-1-1732	NW_003037824.1	-	877306	877257
Sma-1-1733	NS_000200.1	-	57808246	57808197
Sma-1-1734	NW_003037982.1	+	825856	825905
Sma-1-1735	NS_000200.1	+	214485051	214485100
Sma-1-1736	NW_003026470.1	-	512917	512868
Sma-1-1737	NW_003037809.1	-	2606228	2606179
Sma-1-1738	NW_003037889.1	-	1687391	1687342
Sma-1-1739	NW_003037902.1	+	187099	187148
Sma-1-1740	NW_003037912.1	+	1070168	1070217
Sma-1-1741	NS_000200.1	+	146942333	146942382
Sma-1-1742	NS_000200.1	+	157881743	157881792
Sma-1-1743	NS_000200.1	-	219636886	219636837
Sma-1-1744	NS_000200.1	-	135463881	135463832
Sma-1-1745	NS_000200.1	-	36270692	36270643
Sma-1-1746	NW_003037798.1	+	2042370	2042419
Sma-1-1747	NS_000200.1	+	16301528	16301577
Sma-1-1748	NW_003037956.1	-	18136	18087
Sma-1-1749	NS_000200.1	-	194074591	194074542
Sma-1-1750	NW_003037974.1	+	865363	865412
Sma-1-1751	NS_000200.1	+	205540669	205540718
Sma-1-1752	NW_003037806.1	-	580214	580165
Sma-1-1753	NS_000200.1	-	28775428	28775379
Sma-1-1754	NW_003037795.1	-	594490	594441
Sma-1-1755	NS_000200.1	-	6985373	6985324
Sma-1-1756	NW_003037800.1	+	1966717	1966766
Sma-1-1757	NS_000200.1	+	20596077	20596126
Sma-1-1758	NW_003037818.1	+	1029741	1029790
Sma-1-1759	NS_000200.1	+	49745148	49745197
Sma-1-1760	NW_003037916.1	-	1502320	1502271
Sma-1-1761	NS_000200.1	-	164695731	164695682
Sma-1-1762	NW_003037829.1	-	1763089	1763040
Sma-1-1763	NS_000200.1	-	67663627	67663578
Sma-1-1764	NW_003037819.1	+	638760	638809
Sma-1-1765	NW_003037819.1	+	643503	643552
Sma-1-1766	NS_000200.1	+	51727025	51727074
Sma-1-1767	NS_000200.1	+	51731768	51731817
Sma-1-1768	NW_003038943.1	+	1126	1175
Sma-1-1769	NW_003037825.1	-	115841	115792
Sma-1-1770	NS_000200.1	+	356389319	356389368
Sma-1-1771	NS_000200.1	-	59245623	59245574
Sma-1-1772	NW_003038508.1	+	43080	43129
Sma-1-1773	NS_000200.1	+	337150048	337150097
Sma-1-1774	NW_003035869.1	+	118247	118296
Sma-1-1775	NW_003038019.1	+	186588	186637
Sma-1-1776	NS_000200.1	+	233503406	233503455
Sma-1-1777	NS_000200.1	+	335302447	335302496
Sma-1-1778	NW_003037802.1	+	51338	51387

Sma-1-1779	NS_000200.1	+	22054132	22054181
Sma-1-1780	NW_003037910.1	+	50310	50359
Sma-1-1781	NS_000200.1	+	154562285	154562334
Sma-1-1782	NW_003038252.1	-	198461	198412
Sma-1-1783	NS_000200.1	-	319054049	319054000
Sma-1-1784	NW_003037972.1	-	263599	263550
Sma-1-1785	NW_003038151.1	+	131595	131644
Sma-1-1786	NS_000200.1	+	292038838	292038887
Sma-1-1787	NS_000200.1	-	202739303	202739254
Sma-1-1788	NW_003037913.1	-	1466496	1466447
Sma-1-1789	NS_000200.1	-	160030247	160030198
Sma-1-1790	NW_003037918.1	-	1904169	1904120
Sma-1-1791	NS_000200.1	-	168471159	168471110
Sma-1-1792	NW_003037826.1	+	391383	391432
Sma-1-1793	NW_003037975.1	+	388638	388687
Sma-1-1794	NS_000200.1	+	61390899	61390948
Sma-1-1795	NS_000200.1	+	205970862	205970911
Sma-1-1796	NW_003037980.1	-	1267616	1267567
Sma-1-1797	NS_000200.1	-	212228478	212228429
Sma-1-1798	NW_003037833.1	-	642336	642287
Sma-1-1799	NS_000200.1	-	72833432	72833383
Sma-1-1800	NW_003037798.1	-	1993817	1993768
Sma-1-1801	NW_003037811.1	+	1310298	1310347
Sma-1-1802	NS_000200.1	+	39480540	39480589
Sma-1-1803	NS_000200.1	-	16252975	16252926
Sma-1-1804	NW_003037841.1	+	1548537	1548586
Sma-1-1805	NW_003038016.1	+	518172	518221
Sma-1-1806	NS_000200.1	+	84159718	84159767
Sma-1-1807	NS_000200.1	+	231634709	231634758
Sma-1-1808	NW_003037817.1	-	614726	614677
Sma-1-1809	NS_000200.1	-	47997078	47997029
Sma-1-1810	NW_003037809.1	-	63700	63650
Sma-1-1811	NS_000200.1	-	33728164	33728114
Sma-1-1812	NW_003037807.1	+	302363	302412
Sma-1-1813	NS_000200.1	+	30563879	30563928
Sma-1-1814	NW_003037801.1	-	489769	489720
Sma-1-1815	NS_000200.1	-	21579249	21579200
Sma-1-1816	NW_003037909.1	-	602983	602934
Sma-1-1817	NS_000200.1	-	154144632	154144583
Sma-1-1818	NW_003037954.1	-	14893	14844
Sma-1-1819	NW_003038148.1	-	343321	343272
Sma-1-1820	NS_000200.1	-	290417305	290417256
Sma-1-1821	NS_000200.1	-	193244515	193244466
Sma-1-1822	NW_003038083.1	+	683734	683783
Sma-1-1823	NS_000200.1	+	270927071	270927120
Sma-1-1824	NW_003026483.1	-	44613	44564
Sma-1-1825	NS_000200.1	-	256559137	256559088
Sma-1-1826	NW_003038628.1	-	1079	1030
Sma-1-1827	NW_003037795.1	-	1322389	1322340
Sma-1-1828	NS_000200.1	-	345644328	345644279
Sma-1-1829	NS_000200.1	-	7713272	7713223
Sma-1-1830	NW_003038131.1	+	165844	165893

Sma-1-1831	NS_000200.1	+	282127688	282127737
Sma-1-1832	NW_003035169.1	-	1367	1318
Sma-1-1833	NW_003037912.1	+	1691657	1691706
Sma-1-1834	NS_000200.1	+	158503232	158503281
Sma-1-1835	NS_000200.1	-	370730299	370730250
Sma-1-1836	NW_003037797.1	+	3249364	3249413
Sma-1-1837	NS_000200.1	+	13677224	13677273
Sma-1-1838	NW_003038031.1	-	197700	197651
Sma-1-1839	NS_000200.1	-	244475458	244475409
Sma-1-1840	NW_003038261.1	+	136724	136773
Sma-1-1841	NS_000200.1	+	323750375	323750424
Sma-1-1842	NW_003037819.1	-	739912	739863
Sma-1-1843	NS_000200.1	-	51828177	51828128
Sma-1-1844	NW_003026465.1	-	338438	338389
Sma-1-1845	NS_000200.1	-	207731032	207730983
Sma-1-1846	NW_003037928.1	-	242065	242016
Sma-1-1847	NS_000200.1	-	174566018	174565969
Sma-1-1848	NW_003026490.1	+	68697	68746
Sma-1-1849	NS_000200.1	+	267700413	267700462
Sma-1-1850	NW_003026460.1	-	241257	241208
Sma-1-1851	NS_000200.1	-	170763463	170763414
Sma-1-1852	NW_003037814.1	-	125591	125542
Sma-1-1853	NS_000200.1	-	43109451	43109402
Sma-1-1854	NW_003037804.1	-	822709	822660
Sma-1-1855	NS_000200.1	-	25962729	25962680
Sma-1-1856	NW_003037807.1	-	967602	967553
Sma-1-1857	NW_003037848.1	-	350114	350065
Sma-1-1858	NS_000200.1	-	90043185	90043136
Sma-1-1859	NS_000200.1	-	31229118	31229069
Sma-1-1860	NW_003038145.1	+	361834	361883
Sma-1-1861	NS_000200.1	+	288973257	288973306
Sma-1-1862	NW_003038059.1	-	90661	90612
Sma-1-1863	NS_000200.1	-	260332733	260332684
Sma-1-1864	NW_003037866.1	-	721741	721692
Sma-1-1865	NW_003037866.1	-	719518	719469
Sma-1-1866	NW_003037891.1	-	2831817	2831768
Sma-1-1867	NS_000200.1	-	140713095	140713046
Sma-1-1868	NS_000200.1	-	112209540	112209491
Sma-1-1869	NS_000200.1	-	112207317	112207268
Sma-1-1870	NW_003037793.1	-	3810466	3810417
Sma-1-1871	NS_000200.1	-	3810466	3810417
Sma-1-1872	NW_003037997.1	+	97187	97236
Sma-1-1873	NS_000200.1	+	220908629	220908678
Sma-1-1874	NW_003026464.1	+	127322	127371
Sma-1-1875	NS_000200.1	+	203427631	203427680
Sma-1-1876	NW_003026488.1	+	103439	103488
Sma-1-1877	NS_000200.1	+	263609933	263609982
Sma-1-1878	NW_003038156.1	-	99318	99269
Sma-1-1879	NS_000200.1	-	294831431	294831382
Sma-1-1880	NW_003035523.1	-	936	887
Sma-1-1881	NW_003037798.1	+	1534037	1534086
Sma-1-1882	NS_000200.1	+	15793195	15793244

Sma-1-1883	NS_000200.1	-	328339291	328339242
Sma-1-1884	NW_003037798.1	+	2052281	2052330
Sma-1-1885	NW_003038033.1	-	372829	372780
Sma-1-1886	NS_000200.1	+	16311439	16311488
Sma-1-1887	NS_000200.1	-	246352831	246352782
Sma-1-1888	NW_003038161.1	+	87603	87652
Sma-1-1889	NS_000200.1	+	297457454	297457503
Sma-1-1890	NW_003038013.1	-	329472	329423
Sma-1-1891	NS_000200.1	-	229719098	229719049
Sma-1-1892	NW_003038028.1	+	764543	764592
Sma-1-1893	NS_000200.1	+	242072117	242072166
Sma-1-1894	NW_003037825.1	+	1416870	1416919
Sma-1-1895	NW_003038030.1	+	448786	448835
Sma-1-1896	NS_000200.1	+	60546652	60546701
Sma-1-1897	NS_000200.1	+	243584653	243584702
Sma-1-1898	NW_003037985.1	+	62249	62298
Sma-1-1899	NW_003038489.1	+	78372	78421
Sma-1-1900	NW_003037793.1	-	2251263	2251214
Sma-1-1901	NW_003037807.1	-	319891	319842
Sma-1-1902	NW_003037809.1	-	2611876	2611827
Sma-1-1903	NW_003037815.1	+	362096	362145
Sma-1-1904	NW_003037839.1	-	353757	353708
Sma-1-1905	NW_003037860.1	-	451348	451299
Sma-1-1906	NW_003037881.1	+	753148	753197
Sma-1-1907	NW_003037912.1	+	1321216	1321265
Sma-1-1908	NW_003037923.1	+	206170	206219
Sma-1-1909	NW_003037930.1	-	194574	194525
Sma-1-1910	NW_003037943.1	-	1196663	1196614
Sma-1-1911	NW_003038016.1	-	484691	484642
Sma-1-1912	NW_003038086.1	+	327950	327999
Sma-1-1913	NW_003038119.1	-	184268	184219
Sma-1-1914	NW_003038140.1	+	121048	121097
Sma-1-1915	NS_000200.1	+	45625087	45625136
Sma-1-1916	NS_000200.1	+	125184939	125184988
Sma-1-1917	NS_000200.1	+	158132791	158132840
Sma-1-1918	NS_000200.1	+	171262383	171262432
Sma-1-1919	NS_000200.1	+	215189593	215189642
Sma-1-1920	NS_000200.1	+	272576543	272576592
Sma-1-1921	NS_000200.1	+	286648925	286648974
Sma-1-1922	NS_000200.1	+	333790655	333790704
Sma-1-1923	NS_000200.1	-	278091824	278091775
Sma-1-1924	NS_000200.1	-	231601228	231601179
Sma-1-1925	NS_000200.1	-	188218196	188218147
Sma-1-1926	NS_000200.1	-	176052538	176052489
Sma-1-1927	NS_000200.1	-	104194376	104194327
Sma-1-1928	NS_000200.1	-	79311907	79311858
Sma-1-1929	NS_000200.1	-	36276340	36276291
Sma-1-1930	NS_000200.1	-	30581407	30581358
Sma-1-1931	NS_000200.1	-	2251263	2251214
Sma-1-1932	NW_003037990.1	-	178630	178581
Sma-1-1933	NS_000200.1	-	217077389	217077340
Sma-1-1934	NW_003038008.1	+	264406	264455

Sma-1-1935	NS_000200.1	+	225937502	225937551
Sma-1-1936	NW_003037800.1	+	1113741	1113790
Sma-1-1937	NS_000200.1	+	19743101	19743150
Sma-1-1938	NW_003037816.1	-	943565	943516
Sma-1-1939	NS_000200.1	-	47152437	47152388
Sma-1-1940	NW_003037853.1	+	781401	781450
Sma-1-1941	NS_000200.1	+	96273452	96273501
Sma-1-1942	NW_003037860.1	-	51981	51932
Sma-1-1943	NS_000200.1	-	103795009	103794960
Sma-1-1944	NW_003037890.1	+	1161141	1161190
Sma-1-1945	NS_000200.1	+	136844502	136844551
Sma-1-1946	NW_003037851.1	-	572264	572215
Sma-1-1947	NS_000200.1	-	93975574	93975525
Sma-1-1948	NW_003037845.1	+	249741	249790
Sma-1-1949	NS_000200.1	+	87251644	87251693
Sma-1-1950	NW_003037896.1	+	304180	304229
Sma-1-1951	NS_000200.1	+	143224603	143224652
Sma-1-1952	NW_003037801.1	+	484672	484721
Sma-1-1953	NS_000200.1	+	21574152	21574201
Sma-1-1954	NW_003038150.1	+	101810	101859
Sma-1-1955	NS_000200.1	+	291580548	291580597
Sma-1-1956	NW_003028445.1	-	1683	1634
Sma-1-1957	NW_003038260.1	+	190499	190548
Sma-1-1958	NS_000200.1	+	323362274	323362323
Sma-1-1959	NS_000200.1	-	369655758	369655709
Sma-1-1960	NW_003038163.1	-	213337	213288
Sma-1-1961	NS_000200.1	-	298613617	298613568
Sma-1-1962	NW_003037899.1	-	213697	213648
Sma-1-1963	NS_000200.1	-	144732808	144732759
Sma-1-1964	NW_003023460.1	-	2150	2101
Sma-1-1965	NW_003030217.1	-	1887	1838
Sma-1-1966	NW_003038504.1	-	34542	34493
Sma-1-1967	NW_003034557.1	-	8437	8388
Sma-1-1968	NW_003037572.1	-	885	836
Sma-1-1969	NW_003037796.1	+	1247309	1247358
Sma-1-1970	NW_003037822.1	-	400549	400500
Sma-1-1971	NW_003037823.1	+	680321	680370
Sma-1-1972	NW_003037823.1	-	679931	679882
Sma-1-1973	NW_003037824.1	+	503724	503773
Sma-1-1974	NW_003037824.1	-	521220	521171
Sma-1-1975	NW_003037866.1	+	1467712	1467761
Sma-1-1976	NW_003037880.1	+	351969	352018
Sma-1-1977	NW_003037883.1	-	367878	367829
Sma-1-1978	NW_003037889.1	-	1690618	1690569
Sma-1-1979	NW_003037908.1	+	4023	4072
Sma-1-1980	NW_003037942.1	+	436156	436205
Sma-1-1981	NW_003037973.1	-	311290	311241
Sma-1-1982	NW_003037975.1	+	1028129	1028178
Sma-1-1983	NW_003038011.1	-	365630	365581
Sma-1-1984	NW_003038024.1	-	126619	126570
Sma-1-1985	NW_003038034.1	-	880488	880439
Sma-1-1986	NW_003038240.1	+	141237	141286

Sma-1-1987	NW_003026515.1	+	35104	35153
Sma-1-1988	NW_003038044.1	-	135993	135944
Sma-1-1989	NW_003038084.1	-	342717	342668
Sma-1-1990	NW_003038172.1	+	49301	49350
Sma-1-1991	NS_000200.1	+	9456953	9457002
Sma-1-1992	NS_000200.1	+	56596940	56596989
Sma-1-1993	NS_000200.1	+	57434664	57434713
Sma-1-1994	NS_000200.1	+	112955511	112955560
Sma-1-1995	NS_000200.1	+	124035527	124035576
Sma-1-1996	NS_000200.1	+	152477154	152477203
Sma-1-1997	NS_000200.1	+	186196064	186196113
Sma-1-1998	NS_000200.1	+	206610353	206610402
Sma-1-1999	NS_000200.1	+	290679515	290679564
Sma-1-2000	NS_000200.1	+	300722816	300722865
Sma-1-2001	NS_000200.1	+	314280168	314280217
Sma-1-2002	NS_000200.1	-	373440391	373440342
Sma-1-2003	NS_000200.1	-	372065905	372065856
Sma-1-2004	NS_000200.1	-	362367059	362367010
Sma-1-2005	NS_000200.1	-	335883074	335883025
Sma-1-2006	NS_000200.1	-	327436635	327436586
Sma-1-2007	NS_000200.1	-	271357961	271357912
Sma-1-2008	NS_000200.1	-	254760775	254760726
Sma-1-2009	NS_000200.1	-	247822244	247822195
Sma-1-2010	NS_000200.1	-	238023648	238023599
Sma-1-2011	NS_000200.1	-	227893280	227893231
Sma-1-2012	NS_000200.1	-	204293017	204292968
Sma-1-2013	NS_000200.1	-	135467108	135467059
Sma-1-2014	NS_000200.1	-	126431601	126431552
Sma-1-2015	NS_000200.1	-	57452160	57452111
Sma-1-2016	NS_000200.1	-	56596550	56596501
Sma-1-2017	NS_000200.1	-	54708555	54708506
Sma-1-2018	NW_003037908.1	+	976346	976396
Sma-1-2019	NS_000200.1	+	153449477	153449527
Sma-1-2020	NW_003037937.1	+	567977	568026
Sma-1-2021	NS_000200.1	+	181767886	181767935
Sma-1-2022	NW_003038261.1	+	131039	131088
Sma-1-2023	NS_000200.1	+	323744690	323744739
Sma-1-2024	NW_003037809.1	+	2855346	2855395
Sma-1-2025	NW_003037814.1	+	1048218	1048267
Sma-1-2026	NS_000200.1	+	36519810	36519859
Sma-1-2027	NS_000200.1	+	44032078	44032127
Sma-1-2028	NW_003031785.1	-	1293	1244
Sma-1-2029	NW_003037885.1	+	570294	570343
Sma-1-2030	NS_000200.1	+	128668241	128668290
Sma-1-2031	NS_000200.1	-	367010310	367010261
Sma-1-2032	NW_003037792.1	+	149923	149972
Sma-1-2033	NS_000200.1	+	382587267	382587316
Sma-1-2034	NW_003037976.1	-	9839	9790
Sma-1-2035	NS_000200.1	-	206624154	206624105
Sma-1-2036	NW_003037791.1	+	116584	116633
Sma-1-2037	NW_003037852.1	-	593179	593130
Sma-1-2038	NS_000200.1	+	381822012	381822061

Sma-1-2039	NS_000200.1	-	94985074	94985025
Sma-1-2040	NW_003026460.1	-	86029	85980
Sma-1-2041	NW_003026464.1	-	371354	371305
Sma-1-2042	NS_000200.1	-	203671663	203671614
Sma-1-2043	NS_000200.1	-	170608235	170608186
Sma-1-2044	NW_003034033.1	-	2555	2506
Sma-1-2045	NW_003037890.1	-	1585048	1584999
Sma-1-2046	NW_003037932.1	+	110327	110376
Sma-1-2047	NS_000200.1	+	177643523	177643572
Sma-1-2048	NS_000200.1	-	363538664	363538615
Sma-1-2049	NS_000200.1	-	137268409	137268360
Sma-1-2050	NW_003037801.1	-	729947	729898
Sma-1-2051	NS_000200.1	-	21819427	21819378
Sma-1-2052	NW_003038058.1	+	409152	409201
Sma-1-2053	NS_000200.1	+	260240352	260240401
Sma-1-2054	NW_003038035.1	-	49780	49731
Sma-1-2055	NS_000200.1	-	247911272	247911223
Sma-1-2056	NW_003028847.1	+	917	966
Sma-1-2057	NW_003034703.1	+	2546	2595
Sma-1-2058	NS_000200.1	+	328230510	328230559
Sma-1-2059	NS_000200.1	+	370077423	370077472
Sma-1-2060	NW_003037829.1	-	1701309	1701260
Sma-1-2061	NS_000200.1	-	67601847	67601798
Sma-1-2062	NW_003037822.1	-	528668	528619
Sma-1-2063	NS_000200.1	-	54836674	54836625
Sma-1-2064	NW_003037798.1	-	187410	187361
Sma-1-2065	NS_000200.1	-	14446568	14446519
Sma-1-2066	NW_003037797.1	+	566005	566054
Sma-1-2067	NS_000200.1	+	10993865	10993914
Sma-1-2068	NW_003039127.1	+	711206	711255
Sma-1-2069	NS_000200.1	+	113788705	113788754
Sma-1-2070	NW_003038153.1	+	712438	712487
Sma-1-2071	NS_000200.1	+	293774487	293774536
Sma-1-2072	NW_003037809.1	-	2778994	2778945
Sma-1-2073	NS_000200.1	-	36443458	36443409
Sma-1-2074	NW_003037813.1	-	1019255	1019206
Sma-1-2075	NS_000200.1	-	42006118	42006069
Sma-1-2076	NW_003037816.1	+	857050	857099
Sma-1-2077	NW_003037980.1	+	961675	961724
Sma-1-2078	NS_000200.1	+	47065922	47065971
Sma-1-2079	NS_000200.1	+	211922537	211922586
Sma-1-2080	NW_003037795.1	-	1133162	1133113
Sma-1-2081	NS_000200.1	-	7524045	7523996
Sma-1-2082	NW_003024026.1	+	555	604
Sma-1-2083	NS_000200.1	+	344477115	344477164
Sma-1-2084	NW_003038034.1	+	336300	336349
Sma-1-2085	NS_000200.1	+	247278056	247278105
Sma-1-2086	NW_003026618.1	-	157434	157385
Sma-1-2087	NW_003038505.1	-	279958	279909
Sma-1-2088	NS_000200.1	-	336359295	336359246
Sma-1-2089	NS_000200.1	-	321912436	321912387
Sma-1-2090	NW_003037980.1	-	1306287	1306238

Sma-1-2091	NS_000200.1	-	212267149	212267100
Sma-1-2092	NW_003037797.1	+	1447454	1447503
Sma-1-2093	NS_000200.1	+	11875314	11875363
Sma-1-2094	NW_003032891.1	-	402	353
Sma-1-2095	NW_003037849.1	-	944824	944775
Sma-1-2096	NW_003037976.1	+	605267	605316
Sma-1-2097	NW_003037976.1	+	606407	606456
Sma-1-2098	NW_003038086.1	+	64461	64510
Sma-1-2099	NS_000200.1	+	207219582	207219631
Sma-1-2100	NS_000200.1	+	207220722	207220771
Sma-1-2101	NS_000200.1	+	272313054	272313103
Sma-1-2102	NS_000200.1	-	350526925	350526876
Sma-1-2103	NS_000200.1	-	91921097	91921048
Sma-1-2104	NW_003026456.1	+	139637	139686
Sma-1-2105	NS_000200.1	+	58062773	58062822
Sma-1-2106	NW_003037918.1	+	2061896	2061945
Sma-1-2107	NS_000200.1	+	168628886	168628935
Sma-1-2108	NW_003026464.1	+	653408	653457
Sma-1-2109	NS_000200.1	+	203953717	203953766
Sma-1-2110	NW_003038035.1	+	198877	198926
Sma-1-2111	NS_000200.1	+	248060369	248060418
Sma-1-2112	NW_003038041.1	-	103631	103582
Sma-1-2113	NS_000200.1	-	253770913	253770864
Sma-1-2114	NW_003037834.1	+	306755	306804
Sma-1-2115	NS_000200.1	+	73467241	73467290
Sma-1-2116	NW_003038002.1	-	245490	245441
Sma-1-2117	NW_003037827.1	+	374992	375041
Sma-1-2118	NS_000200.1	+	62997133	62997182
Sma-1-2119	NS_000200.1	-	223365699	223365650
Sma-1-2120	NW_003026464.1	-	50874	50825
Sma-1-2121	NW_003037822.1	+	1521091	1521140
Sma-1-2122	NW_003037914.1	-	811240	811191
Sma-1-2123	NS_000200.1	+	55829097	55829146
Sma-1-2124	NS_000200.1	-	203351183	203351134
Sma-1-2125	NS_000200.1	-	160955490	160955441
Sma-1-2126	NW_003038229.1	-	157361	157311
Sma-1-2127	NS_000200.1	-	311274583	311274533
Sma-1-2128	NW_003038031.1	+	676548	676597
Sma-1-2129	NS_000200.1	+	244954306	244954355
Sma-1-2130	NW_003038114.1	+	148403	148452
Sma-1-2131	NS_000200.1	+	276874308	276874357
Sma-1-2132	NW_003037917.1	-	1182114	1182065
Sma-1-2133	NS_000200.1	-	166218918	166218869
Sma-1-2134	NW_003037803.1	+	467997	468046
Sma-1-2135	NS_000200.1	+	23968534	23968583
Sma-1-2136	NW_003038015.1	-	505577	505528
Sma-1-2137	NS_000200.1	-	230829067	230829018
Sma-1-2138	NW_003038122.1	-	15797	15748
Sma-1-2139	NS_000200.1	-	278535024	278534975
Sma-1-2140	NW_003037939.1	+	549434	549483
Sma-1-2141	NS_000200.1	+	183419188	183419237
Sma-1-2142	NW_003024494.1	-	584	535

Sma-1-2143	NW_003035502.1	-	2508	2459
Sma-1-2144	NS_000200.1	-	348733036	348732987
Sma-1-2145	NS_000200.1	-	328297251	328297202
Sma-1-2146	NW_003038033.1	-	421245	421196
Sma-1-2147	NS_000200.1	-	246401247	246401198
Sma-1-2148	NW_003037848.1	+	934138	934187
Sma-1-2149	NW_003037848.1	-	933948	933899
Sma-1-2150	NS_000200.1	+	90627209	90627258
Sma-1-2151	NS_000200.1	-	90627019	90626970
Sma-1-2152	NW_003037838.1	-	811294	811245
Sma-1-2153	NS_000200.1	-	78809857	78809808
Sma-1-2154	NW_003037847.1	-	988413	988364
Sma-1-2155	NS_000200.1	-	89640143	89640094
Sma-1-2156	NW_003037864.1	+	69434	69483
Sma-1-2157	NS_000200.1	+	108562008	108562057
Sma-1-2158	NW_003037880.1	+	682889	682938
Sma-1-2159	NS_000200.1	+	124366447	124366496
Sma-1-2160	NW_003037917.1	+	501122	501171
Sma-1-2161	NS_000200.1	+	165537926	165537975
Sma-1-2162	NW_003037792.1	+	230665	230714
Sma-1-2163	NW_003037917.1	-	480381	480332
Sma-1-2164	NW_003037945.1	-	727187	727138
Sma-1-2165	NS_000200.1	+	382668009	382668058
Sma-1-2166	NS_000200.1	-	189829151	189829102
Sma-1-2167	NS_000200.1	-	165517185	165517136
Sma-1-2168	NW_003037794.1	-	75205	75156
Sma-1-2169	NW_003037841.1	+	1588083	1588132
Sma-1-2170	NW_003037846.1	-	661737	661688
Sma-1-2171	NS_000200.1	+	84199264	84199313
Sma-1-2172	NS_000200.1	-	88296307	88296258
Sma-1-2173	NS_000200.1	-	4254800	4254751
Sma-1-2174	NW_003038006.1	+	253123	253171
Sma-1-2175	NS_000200.1	+	225137607	225137655
Sma-1-2176	NW_003037943.1	-	942181	942133
Sma-1-2177	NS_000200.1	-	187963714	187963666
Sma-1-2178	NW_003037806.1	-	1979049	1979001
Sma-1-2179	NS_000200.1	-	30174263	30174215
Sma-1-2180	NW_003037917.1	+	1115525	1115573
Sma-1-2181	NS_000200.1	+	166152329	166152377
Sma-1-2182	NW_003037797.1	+	97320	97369
Sma-1-2183	NS_000200.1	+	10525180	10525229
Sma-1-2184	NW_003037809.1	-	578093	578044
Sma-1-2185	NS_000200.1	-	34242557	34242508
Sma-1-2186	NW_003027832.1	-	333	284
Sma-1-2187	NW_003037936.1	-	708139	708090
Sma-1-2188	NS_000200.1	-	366002354	366002305
Sma-1-2189	NS_000200.1	-	181147868	181147819
Sma-1-2190	NW_003038038.1	-	1212853	1212804
Sma-1-2191	NS_000200.1	-	253116246	253116197
Sma-1-2192	NW_003038113.1	-	2249	2200
Sma-1-2193	NS_000200.1	-	276663111	276663062
Sma-1-2194	NW_003037969.1	+	435802	435851

Sma-1-2195	NS_000200.1	+	201041929	201041978
Sma-1-2196	NW_003037933.1	-	300662	300613
Sma-1-2197	NS_000200.1	-	178713834	178713785
Sma-1-2198	NW_003037981.1	+	284485	284534
Sma-1-2199	NS_000200.1	+	212682353	212682402
Sma-1-2200	NW_003021009.1	+	102	151
Sma-1-2201	NS_000200.1	+	367773667	367773716
Sma-1-2202	NW_003039058.1	-	1933	1884
Sma-1-2203	NW_003037841.1	+	1076927	1076976
Sma-1-2204	NS_000200.1	+	83688108	83688157
Sma-1-2205	NS_000200.1	-	359474196	359474147
Sma-1-2206	NW_003037793.1	+	869260	869309
Sma-1-2207	NS_000200.1	+	869260	869309
Sma-1-2208	NW_003037831.1	+	979076	979125
Sma-1-2209	NS_000200.1	+	70428378	70428427
Sma-1-2210	NW_003037900.1	+	124591	124640
Sma-1-2211	NS_000200.1	+	145253457	145253506
Sma-1-2212	NW_003037838.1	+	538099	538148
Sma-1-2213	NS_000200.1	+	78536662	78536711
Sma-1-2214	NW_003037837.1	-	432158	432109
Sma-1-2215	NS_000200.1	-	77238016	77237967
Sma-1-2216	NW_003037901.1	+	395305	395354
Sma-1-2217	NS_000200.1	+	146243766	146243815
Sma-1-2218	NW_003037793.1	-	2962214	2962165
Sma-1-2219	NS_000200.1	-	2962214	2962165
Sma-1-2220	NW_003026469.1	+	142681	142730
Sma-1-2221	NS_000200.1	+	217764340	217764389
Sma-1-2222	NW_003037962.1	+	320434	320483
Sma-1-2223	NS_000200.1	+	196397277	196397326
Sma-1-2224	NW_003038249.1	-	275301	275252
Sma-1-2225	NS_000200.1	-	316957554	316957505
Sma-1-2226	NW_003020333.1	-	61	12
Sma-1-2227	NS_000200.1	-	354921700	354921651
Sma-1-2228	NW_003031384.1	-	285	236
Sma-1-2229	NS_000200.1	-	345864385	345864336
Sma-1-2230	NW_003037804.1	-	797074	797025
Sma-1-2231	NS_000200.1	-	25937094	25937045
Sma-1-2232	NW_003037891.1	-	947222	947173
Sma-1-2233	NS_000200.1	-	138828500	138828451
Sma-1-2234	NW_003038037.1	-	1699945	1699896
Sma-1-2235	NS_000200.1	-	251855354	251855305
Sma-1-2236	NW_003037854.1	+	1499350	1499399
Sma-1-2237	NS_000200.1	+	98403771	98403820
Sma-1-2238	NW_003038234.1	+	152288	152337
Sma-1-2239	NS_000200.1	+	312989057	312989106
Sma-1-2240	NW_003038068.1	+	225910	225959
Sma-1-2241	NS_000200.1	+	263293118	263293167
Sma-1-2242	NW_003037849.1	+	611958	612007
Sma-1-2243	NS_000200.1	+	91588231	91588280
Sma-1-2244	NW_003038261.1	-	105044	104995
Sma-1-2245	NS_000200.1	-	323718695	323718646
Sma-1-2246	NW_003037793.1	+	2846112	2846161

Sma-1-2247	NW_003037793.1	-	2845707	2845658
Sma-1-2248	NS_000200.1	+	2846112	2846161
Sma-1-2249	NS_000200.1	-	2845707	2845658
Sma-1-2250	NW_003037981.1	-	608968	608919
Sma-1-2251	NS_000200.1	-	213006836	213006787
Sma-1-2252	NW_003038251.1	+	496125	496173
Sma-1-2253	NS_000200.1	+	318827897	318827945
Sma-1-2254	NW_003038211.1	+	76686	76734
Sma-1-2255	NS_000200.1	+	306096756	306096804
Sma-1-2256	NW_003026578.1	-	36171	36122
Sma-1-2257	NW_003037911.1	+	26114	26163
Sma-1-2258	NS_000200.1	+	155813467	155813516
Sma-1-2259	NS_000200.1	-	302753842	302753793
Sma-1-2260	NW_003037954.1	-	270739	270690
Sma-1-2261	NS_000200.1	-	193500361	193500312
Sma-1-2262	NW_003038032.1	+	255798	255847
Sma-1-2263	NS_000200.1	+	245414958	245415007
Sma-1-2264	NW_003026557.1	+	56438	56487
Sma-1-2265	NS_000200.1	+	301044481	301044530
Sma-1-2266	NW_003037956.1	-	96662	96613
Sma-1-2267	NS_000200.1	-	194153117	194153068
Sma-1-2268	NW_003037886.1	+	915252	915301
Sma-1-2269	NS_000200.1	+	130170819	130170868
Sma-1-2270	NW_003037822.1	+	672564	672613
Sma-1-2271	NS_000200.1	+	54980570	54980619
Sma-1-2272	NW_003038251.1	+	60989	61038
Sma-1-2273	NS_000200.1	+	318392761	318392810
Sma-1-2274	NW_003037814.1	-	1610542	1610493
Sma-1-2275	NS_000200.1	-	44594402	44594353
Sma-1-2276	NW_003037827.1	-	1820187	1820138
Sma-1-2277	NS_000200.1	-	64442328	64442279
Sma-1-2278	NW_003037810.1	+	444962	445011
Sma-1-2279	NS_000200.1	+	37049484	37049533
Sma-1-2280	NW_003031858.1	-	406	357
Sma-1-2281	NS_000200.1	-	367156902	367156853
Sma-1-2282	NW_003037862.1	+	992320	992369
Sma-1-2283	NS_000200.1	+	106643981	106644030
Sma-1-2284	NW_003026597.1	+	122283	122332
Sma-1-2285	NS_000200.1	+	305743469	305743518
Sma-1-2286	NW_003037793.1	+	2417943	2417992
Sma-1-2287	NS_000200.1	+	2417943	2417992
Sma-1-2288	NW_003037792.1	-	378446	378397
Sma-1-2289	NS_000200.1	-	382815790	382815741
Sma-1-2290	NW_003037803.1	-	1007699	1007650
Sma-1-2291	NS_000200.1	-	24508236	24508187
Sma-1-2292	NW_003037935.1	-	514441	514392
Sma-1-2293	NS_000200.1	-	179930976	179930927
Sma-1-2294	NW_003037909.1	+	812695	812744
Sma-1-2295	NS_000200.1	+	154354344	154354393
Sma-1-2296	NW_003038249.1	-	306899	306850
Sma-1-2297	NS_000200.1	-	316989152	316989103
Sma-1-2298	NW_003037823.1	+	226016	226065

Sma-1-2299	NS_000200.1	+	56142635	56142684
Sma-1-2300	NW_003038508.1	-	61897	61848
Sma-1-2301	NW_003035642.1	+	22084	22133
Sma-1-2302	NS_000200.1	+	330279760	330279809
Sma-1-2303	NS_000200.1	-	337168865	337168816
Sma-1-2304	NW_003037861.1	+	290258	290307
Sma-1-2305	NS_000200.1	+	105066712	105066761
Sma-1-2306	NW_003030610.1	-	1401	1352
Sma-1-2307	NS_000200.1	-	366573120	366573071
Sma-1-2308	NW_003037794.1	+	501511	501560
Sma-1-2309	NS_000200.1	+	4681106	4681155
Sma-1-2310	NW_003038085.1	+	68783	68832
Sma-1-2311	NS_000200.1	+	271758876	271758925
Sma-1-2312	NW_003037909.1	-	646390	646341
Sma-1-2313	NS_000200.1	-	154188039	154187990
Sma-1-2314	NW_003026495.1	+	13731	13780
Sma-1-2315	NS_000200.1	+	274339145	274339194
Sma-1-2316	NW_003037824.1	+	144128	144177
Sma-1-2317	NS_000200.1	+	57075068	57075117
Sma-1-2318	NW_003038027.1	+	305894	305943
Sma-1-2319	NS_000200.1	+	240676608	240676657
Sma-1-2320	NW_003037793.1	-	3086874	3086825
Sma-1-2321	NS_000200.1	-	3086874	3086825
Sma-1-2322	NW_003037895.1	-	290492	290443
Sma-1-2323	NS_000200.1	-	142736694	142736645
Sma-1-2324	NW_003037823.1	+	7547	7596
Sma-1-2325	NS_000200.1	+	55924166	55924215
Sma-1-2326	NW_003037932.1	+	574887	574936
Sma-1-2327	NS_000200.1	+	178108083	178108132
Sma-1-2328	NW_003037977.1	+	643997	644046
Sma-1-2329	NS_000200.1	+	208930017	208930066
Sma-1-2330	NW_003037894.1	+	453835	453884
Sma-1-2331	NS_000200.1	+	142350408	142350457
Sma-1-2332	NW_003026503.1	+	21030	21079
Sma-1-2333	NS_000200.1	+	276584205	276584254
Sma-1-2334	NW_003037841.1	-	2006503	2006454
Sma-1-2335	NS_000200.1	-	84617684	84617635
Sma-1-2336	NW_003037795.1	+	611290	611339
Sma-1-2337	NS_000200.1	+	7002173	7002222
Sma-1-2338	NW_003038028.1	-	690411	690362
Sma-1-2339	NS_000200.1	-	241997985	241997936
Sma-1-2340	NW_003038250.1	+	115043	115092
Sma-1-2341	NS_000200.1	+	318167720	318167769
Sma-1-2342	NW_003039053.1	-	107	58
Sma-1-2343	NS_000200.1	-	359338339	359338290
Sma-1-2344	NW_003031567.1	-	670	621
Sma-1-2345	NW_003038031.1	+	709998	710047
Sma-1-2346	NS_000200.1	+	244987756	244987805
Sma-1-2347	NS_000200.1	-	350202865	350202816
Sma-1-2348	NW_003037844.1	+	90083	90132
Sma-1-2349	NS_000200.1	+	86273529	86273578
Sma-1-2350	NW_003038244.1	+	146878	146927

Sma-1-2351	NS_000200.1	+	315292083	315292132
Sma-1-2352	NW_003037914.1	-	1483468	1483419
Sma-1-2353	NS_000200.1	-	161627718	161627669
Sma-1-2354	NW_003037914.1	-	1481276	1481227
Sma-1-2355	NS_000200.1	-	161625526	161625477
Sma-1-2356	NW_003037859.1	-	369238	369189
Sma-1-2357	NS_000200.1	-	103048079	103048030
Sma-1-2358	NW_003038069.1	+	69150	69199
Sma-1-2359	NS_000200.1	+	263912556	263912605
Sma-1-2360	NW_003037909.1	-	465732	465683
Sma-1-2361	NS_000200.1	-	154007381	154007332
Sma-1-2362	NW_003037982.1	-	1343309	1343260
Sma-1-2363	NS_000200.1	-	215002504	215002455
Sma-1-2364	NW_003038079.1	-	95557	95508
Sma-1-2365	NS_000200.1	-	268225545	268225496
Sma-1-2366	NW_003037962.1	+	69075	69124
Sma-1-2367	NS_000200.1	+	196145918	196145967
Sma-1-2368	NW_003038238.1	+	35314	35363
Sma-1-2369	NS_000200.1	+	313642095	313642144
Sma-1-2370	NW_003038251.1	+	102168	102217
Sma-1-2371	NS_000200.1	+	318433940	318433989
Sma-1-2372	NW_003037797.1	-	2585270	2585221
Sma-1-2373	NS_000200.1	-	13013130	13013081
Sma-1-2374	NW_003037824.1	-	757824	757775
Sma-1-2375	NS_000200.1	-	57688764	57688715
Sma-1-2376	NW_003037938.1	-	685235	685186
Sma-1-2377	NS_000200.1	-	182722348	182722299
Sma-1-2378	NW_003037977.1	+	616780	616829
Sma-1-2379	NS_000200.1	+	208902800	208902849
Sma-1-2380	NW_003038029.1	+	299650	299699
Sma-1-2381	NS_000200.1	+	242500551	242500600
Sma-1-2382	NW_003037860.1	-	328739	328690
Sma-1-2383	NS_000200.1	-	104071767	104071718
Sma-1-2384	NW_003037795.1	-	630192	630143
Sma-1-2385	NS_000200.1	-	7021075	7021026
Sma-1-2386	NW_003037915.1	-	710234	710185
Sma-1-2387	NS_000200.1	-	162610220	162610171
Sma-1-2388	NW_003038251.1	-	262948	262899
Sma-1-2389	NS_000200.1	-	318594720	318594671
Sma-1-2390	NW_003037827.1	+	1844928	1844977
Sma-1-2391	NS_000200.1	+	64467069	64467118
Sma-1-2392	NW_003037796.1	-	68319	68270
Sma-1-2393	NS_000200.1	-	8277963	8277914
Sma-1-2394	NW_003029653.1	+	1335	1384
Sma-1-2395	NS_000200.1	+	366356352	366356401
Sma-1-2396	NW_003039127.1	+	574937	574986
Sma-1-2397	NS_000200.1	+	113652436	113652485
Sma-1-2398	NW_003037897.1	+	105573	105622
Sma-1-2399	NS_000200.1	+	143587949	143587998
Sma-1-2400	NW_003037797.1	-	2555815	2555766
Sma-1-2401	NS_000200.1	-	12983675	12983626
Sma-1-2402	NW_003037797.1	-	3142014	3141964

Sma-1-2403	NS_000200.1	-	13569874	13569824
Sma-1-2404	NW_003037813.1	-	1587178	1587129
Sma-1-2405	NS_000200.1	-	42574041	42573992
Sma-1-2406	NW_003038013.1	-	280911	280860
Sma-1-2407	NS_000200.1	-	229670537	229670486
Sma-1-2408	NW_003037814.1	-	1658488	1658439
Sma-1-2409	NS_000200.1	-	44642348	44642299
Sma-1-2410	NW_003032155.1	-	51720	51671
Sma-1-2411	NS_000200.1	-	379602811	379602762
Sma-1-2412	NW_003038118.1	-	127744	127695
Sma-1-2413	NS_000200.1	-	277691871	277691822
Sma-1-2414	NW_003026507.1	+	243996	244045
Sma-1-2415	NS_000200.1	+	279904147	279904196
Sma-1-2416	NW_003038049.1	+	17367	17416
Sma-1-2417	NS_000200.1	+	255992894	255992943
Sma-1-2418	NW_003032160.1	+	252363	252412
Sma-1-2419	NS_000200.1	+	381260449	381260498
Sma-1-2420	NW_003037841.1	+	1518005	1518054
Sma-1-2421	NS_000200.1	+	84129186	84129235
Sma-1-2422	NW_003037886.1	-	858494	858445
Sma-1-2423	NS_000200.1	-	130114061	130114012
Sma-1-2424	NW_003037822.1	+	324928	324977
Sma-1-2425	NS_000200.1	+	54632934	54632983
Sma-1-2426	NW_003037893.1	+	497415	497464
Sma-1-2427	NS_000200.1	+	141611725	141611774
Sma-1-2428	NW_003029634.1	+	1313	1362
Sma-1-2429	NS_000200.1	+	366307616	366307665
Sma-1-2430	NW_003037812.1	+	1424021	1424071
Sma-1-2431	NS_000200.1	+	40906760	40906810
Sma-1-2432	NW_003037824.1	-	850520	850471
Sma-1-2433	NS_000200.1	-	57781460	57781411
Sma-1-2434	NW_003037817.1	-	977077	977028
Sma-1-2435	NS_000200.1	-	48359429	48359380
Sma-1-2436	NW_003037837.1	-	8419	8370
Sma-1-2437	NS_000200.1	-	76814277	76814228
Sma-1-2438	NW_003037932.1	-	315622	315573
Sma-1-2439	NS_000200.1	-	177848818	177848769
Sma-1-2440	NW_003038514.1	+	97178	97227
Sma-1-2441	NW_003038514.1	-	97967	97918
Sma-1-2442	NS_000200.1	+	339721717	339721766
Sma-1-2443	NS_000200.1	-	339722506	339722457
Sma-1-2444	NW_003038032.1	-	176791	176742
Sma-1-2445	NS_000200.1	-	245335951	245335902
Sma-1-2446	NW_003037846.1	-	202881	202832
Sma-1-2447	NS_000200.1	-	87837451	87837402
Sma-1-2448	NW_003037891.1	+	2065821	2065870
Sma-1-2449	NS_000200.1	+	139947099	139947148
Sma-1-2450	NW_003038008.1	+	397218	397267
Sma-1-2451	NS_000200.1	+	226070314	226070363
Sma-1-2452	NW_003037810.1	+	175036	175085
Sma-1-2453	NS_000200.1	+	36779558	36779607
Sma-1-2454	NW_003037864.1	-	369176	369127

Sma-1-2455	NS_000200.1	-	108861750	108861701
Sma-1-2456	NW_003037953.1	+	55999	56048
Sma-1-2457	NS_000200.1	+	192969050	192969099
Sma-1-2458	NW_003038120.1	-	75984	75935
Sma-1-2459	NS_000200.1	-	278261207	278261158
Sma-1-2460	NW_003037806.1	-	73008	72959
Sma-1-2461	NS_000200.1	-	28268222	28268173
Sma-1-2462	NW_003038083.1	+	300324	300373
Sma-1-2463	NS_000200.1	+	270543661	270543710
Sma-1-2464	NW_003037964.1	+	543275	543324
Sma-1-2465	NS_000200.1	+	198144797	198144846
Sma-1-2466	NW_003037806.1	-	844474	844425
Sma-1-2467	NW_003038159.1	+	359723	359772
Sma-1-2468	NS_000200.1	+	296747744	296747793
Sma-1-2469	NS_000200.1	-	29039688	29039639
Sma-1-2470	NW_003037914.1	-	753930	753881
Sma-1-2471	NS_000200.1	-	160898180	160898131
Sma-1-2472	NW_003037910.1	-	512831	512782
Sma-1-2473	NS_000200.1	-	155024806	155024757
Sma-1-2474	NW_003037865.1	+	452238	452287
Sma-1-2475	NS_000200.1	+	110874157	110874206
Sma-1-2476	NW_003026581.1	-	24305	24255
Sma-1-2477	NS_000200.1	-	302854293	302854243
Sma-1-2478	NW_003037910.1	+	726997	727046
Sma-1-2479	NS_000200.1	+	155238972	155239021
Sma-1-2480	NW_003037822.1	-	1095803	1095754
Sma-1-2481	NS_000200.1	-	55403809	55403760
Sma-1-2482	NW_003037846.1	+	329356	329405
Sma-1-2483	NS_000200.1	+	87963926	87963975
Sma-1-2484	NW_003037809.1	+	654316	654365
Sma-1-2485	NS_000200.1	+	34318780	34318829
Sma-1-2486	NW_003038114.1	+	171214	171263
Sma-1-2487	NS_000200.1	+	276897119	276897168
Sma-1-2488	NW_003037916.1	+	1314507	1314556
Sma-1-2489	NS_000200.1	+	164507918	164507967
Sma-1-2490	NW_003039129.1	-	149788	149739
Sma-1-2491	NS_000200.1	-	229104596	229104547
Sma-1-2492	NW_003038033.1	-	385960	385911
Sma-1-2493	NS_000200.1	-	246365962	246365913
Sma-1-2494	NW_003037917.1	-	488697	488648
Sma-1-2495	NS_000200.1	-	165525501	165525452
Sma-1-2496	NW_003038161.1	-	68407	68358
Sma-1-2497	NS_000200.1	-	297438258	297438209
Sma-1-2498	NW_003037948.1	-	158253	158204
Sma-1-2499	NS_000200.1	-	192178197	192178148
Sma-1-2500	NW_003037821.1	-	258990	258941
Sma-1-2501	NS_000200.1	-	53403474	53403425
Sma-1-2502	NW_003034945.1	-	5266	5217
Sma-1-2503	NW_003026463.1	-	72026	71977
Sma-1-2504	NW_003037854.1	+	1486524	1486573
Sma-1-2505	NS_000200.1	+	98390945	98390994
Sma-1-2506	NS_000200.1	-	351060495	351060446

Sma-1-2507	NS_000200.1	-	198687396	198687347
Sma-1-2508	NW_003037946.1	+	803037	803086
Sma-1-2509	NS_000200.1	+	191496897	191496946
Sma-1-2510	NW_003035301.1	-	753	704
Sma-1-2511	NW_003037876.1	-	166847	166798
Sma-1-2512	NS_000200.1	-	370973675	370973626
Sma-1-2513	NS_000200.1	-	120712483	120712434
Sma-1-2514	NW_003037886.1	+	1106855	1106904
Sma-1-2515	NS_000200.1	+	130362422	130362471
Sma-1-2516	NW_003037821.1	+	106109	106158
Sma-1-2517	NS_000200.1	+	53250593	53250642
Sma-1-2518	NW_003026481.1	+	128860	128909
Sma-1-2519	NS_000200.1	+	255528844	255528893
Sma-1-2520	NW_003037820.1	-	297736	297687
Sma-1-2521	NS_000200.1	-	52570718	52570669
Sma-1-2522	NW_003038143.1	+	474786	474835
Sma-1-2523	NS_000200.1	+	288234278	288234327
Sma-1-2524	NW_003037797.1	-	1489802	1489753
Sma-1-2525	NS_000200.1	-	11917662	11917613
Sma-1-2526	NW_003038475.1	-	44719	44670
Sma-1-2527	NS_000200.1	-	332361002	332360953
Sma-1-2528	NW_003037935.1	+	246807	246856
Sma-1-2529	NS_000200.1	+	179663342	179663391
Sma-1-2530	NW_003026602.1	+	36165	36214
Sma-1-2531	NS_000200.1	+	306956148	306956197
Sma-1-2532	NW_003037797.1	-	3763110	3763061
Sma-1-2533	NS_000200.1	-	14190970	14190921
Sma-1-2534	NW_003037909.1	+	368067	368116
Sma-1-2535	NS_000200.1	+	153909716	153909765
Sma-1-2536	NW_003038084.1	-	155684	155635
Sma-1-2537	NS_000200.1	-	271170928	271170879
Sma-1-2538	NW_003035618.1	+	13	62
Sma-1-2539	NS_000200.1	+	329412302	329412351
Sma-1-2540	NW_003037978.1	-	201454	201405
Sma-1-2541	NS_000200.1	-	209469464	209469415
Sma-1-2542	NW_003026476.1	-	90886	90837
Sma-1-2543	NS_000200.1	-	253639656	253639607
Sma-1-2544	NW_003037793.1	-	3061375	3061326
Sma-1-2545	NS_000200.1	-	3061375	3061326
Sma-1-2546	NW_003038131.1	-	359160	359111
Sma-1-2547	NS_000200.1	-	282321004	282320955
Sma-1-2548	NW_003038142.1	-	284357	284308
Sma-1-2549	NS_000200.1	-	287494793	287494744
Sma-1-2550	NW_003037807.1	+	1208874	1208923
Sma-1-2551	NS_000200.1	+	31470390	31470439
Sma-1-2552	NW_003020641.1	-	429	380
Sma-1-2553	NS_000200.1	-	360516105	360516056
Sma-1-2554	NW_003035682.1	+	18691	18740
Sma-1-2555	NS_000200.1	+	332640744	332640793
Sma-1-2556	NW_003037906.1	+	147761	147810
Sma-1-2557	NS_000200.1	+	150455540	150455589
Sma-1-2558	NW_003038005.1	-	431236	431187

Sma-1-2559	NS_000200.1	-	224837190	224837141
Sma-1-2560	NW_003038261.1	-	283089	283040
Sma-1-2561	NS_000200.1	-	323896740	323896691
Sma-1-2562	NW_003035781.1	-	983	934
Sma-1-2563	NS_000200.1	-	364075797	364075748
Sma-1-2564	NW_003038261.1	-	322004	321955
Sma-1-2565	NS_000200.1	-	323935655	323935606
Sma-1-2566	NW_003038034.1	-	336909	336860
Sma-1-2567	NS_000200.1	-	247278665	247278616
Sma-1-2568	NW_003037859.1	-	439613	439564
Sma-1-2569	NS_000200.1	-	103118454	103118405
Sma-1-2570	NW_003037797.1	+	3801284	3801333
Sma-1-2571	NS_000200.1	+	14229144	14229193
Sma-1-2572	NW_003038034.1	+	386996	387045
Sma-1-2573	NS_000200.1	+	247328752	247328801
Sma-1-2574	NW_003037912.1	+	1546020	1546069
Sma-1-2575	NS_000200.1	+	158357595	158357644
Sma-1-2576	NW_003037937.1	-	650368	650319
Sma-1-2577	NS_000200.1	-	181850277	181850228
Sma-1-2578	NW_003037871.1	-	19810	19761
Sma-1-2579	NS_000200.1	-	117467548	117467499
Sma-1-2580	NW_003037912.1	+	1674299	1674348
Sma-1-2581	NS_000200.1	+	158485874	158485923
Sma-1-2582	NW_003038508.1	-	43990	43941
Sma-1-2583	NS_000200.1	-	337150958	337150909
Sma-1-2584	NW_003037972.1	+	86093	86142
Sma-1-2585	NS_000200.1	+	202561797	202561846
Sma-1-2586	NW_003035870.1	+	70417	70467
Sma-1-2587	NS_000200.1	+	335435706	335435756
Sma-1-2588	NW_003038239.1	+	283184	283233
Sma-1-2589	NS_000200.1	+	314123966	314124015
Sma-1-2590	NW_003037871.1	-	410425	410376
Sma-1-2591	NS_000200.1	-	117858163	117858114
Sma-1-2592	NW_003026472.1	+	220146	220195
Sma-1-2593	NS_000200.1	+	236632138	236632187
Sma-1-2594	NW_003037905.1	-	897178	897129
Sma-1-2595	NS_000200.1	-	150166190	150166141
Sma-1-2596	NW_003035880.1	-	112784	112735
Sma-1-2597	NS_000200.1	-	339353848	339353799
Sma-1-2598	NW_003035880.1	-	187365	187316
Sma-1-2599	NS_000200.1	-	339428429	339428380
Sma-1-2600	NW_003037889.1	+	1900917	1900966
Sma-1-2601	NS_000200.1	+	135677407	135677456
Sma-1-2602	NW_003037797.1	-	218156	218107
Sma-1-2603	NS_000200.1	-	10646016	10645967
Sma-1-2604	NW_003038035.1	-	428601	428552
Sma-1-2605	NS_000200.1	-	248290093	248290044
Sma-1-2606	NW_003038035.1	+	570767	570816
Sma-1-2607	NS_000200.1	+	248432259	248432308
Sma-1-2608	NW_003022147.1	+	5955	6004
Sma-1-2609	NS_000200.1	+	360806500	360806549
Sma-1-2610	NW_003037819.1	-	113709	113660

Sma-1-2611	NS_000200.1	-	51201974	51201925
Sma-1-2612	NW_003038050.1	+	236143	236192
Sma-1-2613	NS_000200.1	+	256511037	256511086
Sma-1-2614	NW_003038386.1	-	3766	3717
Sma-1-2615	NS_000200.1	-	327865229	327865180
Sma-1-2616	NW_003038002.1	-	5878	5829
Sma-1-2617	NS_000200.1	-	223126087	223126038
Sma-1-2618	NW_003028271.1	+	1028	1077
Sma-1-2619	NS_000200.1	+	374437380	374437429
Sma-1-2620	NW_003037856.1	+	155099	155148
Sma-1-2621	NS_000200.1	+	99513055	99513104
Sma-1-2622	NW_003037819.1	-	1032154	1032105
Sma-1-2623	NS_000200.1	-	52120419	52120370
Sma-1-2624	NW_003038037.1	-	1089241	1089192
Sma-1-2625	NW_003038037.1	-	1087963	1087914
Sma-1-2626	NS_000200.1	-	251244650	251244601
Sma-1-2627	NS_000200.1	-	251243372	251243323
Sma-1-2628	NW_003037933.1	+	504623	504672
Sma-1-2629	NS_000200.1	+	178917795	178917844
Sma-1-2630	NW_003037853.1	-	321091	321042
Sma-1-2631	NS_000200.1	-	95813142	95813093
Sma-1-2632	NW_003038086.1	-	661340	661291
Sma-1-2633	NS_000200.1	-	272909933	272909884
Sma-1-2634	NW_003037943.1	-	746399	746350
Sma-1-2635	NS_000200.1	-	187767932	187767883
Sma-1-2636	NW_003037884.1	+	882398	882447
Sma-1-2637	NS_000200.1	+	127662063	127662112
Sma-1-2638	NW_003037809.1	-	542221	542172
Sma-1-2639	NS_000200.1	-	34206685	34206636
Sma-1-2640	NW_003037830.1	+	698055	698104
Sma-1-2641	NS_000200.1	+	68588426	68588475
Sma-1-2642	NW_003038452.1	+	1842	1891
Sma-1-2643	NS_000200.1	+	330579483	330579532
Sma-1-2644	NW_003037796.1	+	2003618	2003667
Sma-1-2645	NS_000200.1	+	10213262	10213311
Sma-1-2646	NW_003037838.1	-	190721	190672
Sma-1-2647	NS_000200.1	-	78189284	78189235
Sma-1-2648	NW_003037826.1	-	530061	530012
Sma-1-2649	NS_000200.1	-	61529577	61529528
Sma-1-2650	NW_003026471.1	+	108780	108829
Sma-1-2651	NS_000200.1	+	222221013	222221062
Sma-1-2652	NW_003038141.1	-	101066	101017
Sma-1-2653	NS_000200.1	-	286991045	286990996
Sma-1-2654	NW_003037816.1	-	678299	678250
Sma-1-2655	NS_000200.1	-	46887171	46887122
Sma-1-2656	NW_003037813.1	-	1966919	1966870
Sma-1-2657	NS_000200.1	-	42953782	42953733
Sma-1-2658	NW_003037847.1	-	872472	872423
Sma-1-2659	NS_000200.1	-	89524202	89524153
Sma-1-2660	NW_003038000.1	+	102498	102547
Sma-1-2661	NS_000200.1	+	222573276	222573325
Sma-1-2662	NW_003026470.1	+	84290	84339

Sma-1-2663	NS_000200.1	+	219208259	219208308
Sma-1-2664	NW_003036362.1	+	1651	1700
Sma-1-2665	NS_000200.1	+	359709267	359709316
Sma-1-2666	NW_003037942.1	+	829741	829790
Sma-1-2667	NS_000200.1	+	186589649	186589698
Sma-1-2668	NW_003038037.1	-	704949	704900
Sma-1-2669	NS_000200.1	-	250860358	250860309
Sma-1-2670	NW_003036403.1	-	474	425
Sma-1-2671	NW_003037943.1	-	1154921	1154872
Sma-1-2672	NS_000200.1	-	359882180	359882131
Sma-1-2673	NS_000200.1	-	188176454	188176405
Sma-1-2674	NW_003037969.1	-	182139	182090
Sma-1-2675	NS_000200.1	-	200788266	200788217
Sma-1-2676	NW_003038125.1	-	48628	48579
Sma-1-2677	NS_000200.1	-	279485584	279485535
Sma-1-2678	NW_003038146.1	+	70976	71025
Sma-1-2679	NS_000200.1	+	289051646	289051695
Sma-1-2680	NW_003037884.1	+	1244646	1244695
Sma-1-2681	NS_000200.1	+	128024311	128024360
Sma-1-2682	NW_003037884.1	+	1100813	1100862
Sma-1-2683	NS_000200.1	+	127880478	127880527
Sma-1-2684	NW_003038234.1	-	80520	80471
Sma-1-2685	NS_000200.1	-	312917289	312917240
Sma-1-2686	NW_003037818.1	-	593819	593770
Sma-1-2687	NS_000200.1	-	49309226	49309177
Sma-1-2688	NW_003037797.1	+	970460	970509
Sma-1-2689	NS_000200.1	+	11398320	11398369
Sma-1-2690	NW_003037969.1	-	212027	211978
Sma-1-2691	NS_000200.1	-	200818154	200818105
Sma-1-2692	NW_003037818.1	-	466814	466765
Sma-1-2693	NS_000200.1	-	49182221	49182172
Sma-1-2694	NW_003037798.1	-	117571	117522
Sma-1-2695	NS_000200.1	-	14376729	14376680
Sma-1-2696	NW_003038038.1	+	1000549	1000598
Sma-1-2697	NS_000200.1	+	252903942	252903991
Sma-1-2698	NW_003038476.1	-	63672	63623
Sma-1-2699	NS_000200.1	-	332452047	332451998
Sma-1-2700	NW_003038476.1	+	74652	74701
Sma-1-2701	NS_000200.1	+	332463027	332463076
Sma-1-2702	NW_003038102.1	-	24588	24539
Sma-1-2703	NS_000200.1	-	274703534	274703485
Sma-1-2704	NW_003037775.1	-	10465	10416
Sma-1-2705	NS_000200.1	-	378639631	378639582
Sma-1-2706	NW_003037969.1	+	392590	392639
Sma-1-2707	NS_000200.1	+	200998717	200998766
Sma-1-2708	NW_003038038.1	+	1011271	1011320
Sma-1-2709	NS_000200.1	+	252914664	252914713
Sma-1-2710	NW_003037881.1	+	654787	654836
Sma-1-2711	NS_000200.1	+	125086578	125086627
Sma-1-2712	NW_003037941.1	-	971974	971925
Sma-1-2713	NS_000200.1	-	185706448	185706399
Sma-1-2714	NW_003037359.1	+	741	790

Sma-1-2715	NW_003038120.1	+	21197	21246
Sma-1-2716	NS_000200.1	+	278206420	278206469
Sma-1-2717	NS_000200.1	+	366972933	366972982
Sma-1-2718	NW_003037836.1	-	540652	540603
Sma-1-2719	NS_000200.1	-	76097751	76097702
Sma-1-2720	NW_003027298.1	-	925	876
Sma-1-2721	NS_000200.1	-	353429207	353429158
Sma-1-2722	NW_003038137.1	-	7972	7923
Sma-1-2723	NS_000200.1	-	284796868	284796819
Sma-1-2724	NW_003038488.1	-	60847	60798
Sma-1-2725	NS_000200.1	-	333657904	333657855
Sma-1-2726	NW_003026468.1	+	218685	218734
Sma-1-2727	NS_000200.1	+	216412706	216412755
Sma-1-2728	NW_003026517.1	-	196412	196363
Sma-1-2729	NS_000200.1	-	299802421	299802372
Sma-1-2730	NW_003037940.1	+	422302	422351
Sma-1-2731	NS_000200.1	+	184164432	184164481
Sma-1-2732	NW_003037913.1	+	262298	262347
Sma-1-2733	NS_000200.1	+	158826049	158826098
Sma-1-2734	NW_003037972.1	+	473480	473529
Sma-1-2735	NS_000200.1	+	202949184	202949233
Sma-1-2736	NW_003037795.1	+	637910	637959
Sma-1-2737	NS_000200.1	+	7028793	7028842
Sma-1-2738	NW_003037879.1	+	136378	136427
Sma-1-2739	NS_000200.1	+	123138634	123138683
Sma-1-2740	NW_003038109.1	-	41790	41741
Sma-1-2741	NS_000200.1	-	275724468	275724419
Sma-1-2742	NW_003038512.1	-	77887	77838
Sma-1-2743	NS_000200.1	-	338634584	338634535
Sma-1-2744	NW_003039047.1	-	3665	3616
Sma-1-2745	NS_000200.1	-	359204367	359204318
Sma-1-2746	NW_003037879.1	+	331477	331526
Sma-1-2747	NS_000200.1	+	123333733	123333782
Sma-1-2748	NW_003038161.1	-	485775	485726
Sma-1-2749	NS_000200.1	-	297855626	297855577
Sma-1-2750	NW_003038011.1	-	188039	187990
Sma-1-2751	NS_000200.1	-	227715689	227715640
Sma-1-2752	NW_003026621.1	+	5209	5258
Sma-1-2753	NS_000200.1	+	324848603	324848652
Sma-1-2754	NW_003037835.1	+	983226	983275
Sma-1-2755	NS_000200.1	+	75379734	75379783
Sma-1-2756	NW_003037974.1	+	685800	685849
Sma-1-2757	NS_000200.1	+	205361106	205361155
Sma-1-2758	NW_003037805.1	+	1396719	1396768
Sma-1-2759	NS_000200.1	+	27954563	27954612
Sma-1-2760	NW_003037938.1	+	750909	750958
Sma-1-2761	NS_000200.1	+	182788022	182788071
Sma-1-2762	NW_003038137.1	-	223316	223267
Sma-1-2763	NS_000200.1	-	285012212	285012163
Sma-1-2764	NW_003038012.1	-	125123	125074
Sma-1-2765	NS_000200.1	-	228449062	228449013
Sma-1-2766	NW_003037850.1	-	205776	205727

Sma-1-2767	NS_000200.1	-	92191806	92191757
Sma-1-2768	NW_003037901.1	+	779563	779612
Sma-1-2769	NS_000200.1	+	146628024	146628073
Sma-1-2770	NW_003037777.1	+	43779	43828
Sma-1-2771	NS_000200.1	+	378861565	378861614
Sma-1-2772	NW_003026609.1	-	26381	26332
Sma-1-2773	NS_000200.1	-	309681373	309681324
Sma-1-2774	NW_003037882.1	+	135387	135436
Sma-1-2775	NS_000200.1	+	125427061	125427110
Sma-1-2776	NW_003026516.1	-	386497	386448
Sma-1-2777	NS_000200.1	-	299494794	299494745
Sma-1-2778	NS_000200.1	-	373500852	373500803
Sma-1-2779	NW_003037907.1	+	152697	152746
Sma-1-2780	NS_000200.1	+	151557186	151557235
Sma-1-2781	NW_003037994.1	+	272728	272777
Sma-1-2782	NS_000200.1	+	218804542	218804591
Sma-1-2783	NW_003026273.1	-	107	58
Sma-1-2784	NW_003037854.1	+	664372	664421
Sma-1-2785	NS_000200.1	+	97568793	97568842
Sma-1-2786	NS_000200.1	-	344652186	344652137
Sma-1-2787	NW_003038228.1	+	102959	103008
Sma-1-2788	NS_000200.1	+	310976239	310976288
Sma-1-2789	NW_003026465.1	-	627602	627553
Sma-1-2790	NS_000200.1	-	208020196	208020147
Sma-1-2791	NW_003039127.1	+	1921445	1921494
Sma-1-2792	NS_000200.1	+	114998944	114998993
Sma-1-2793	NW_003037856.1	-	580069	580020
Sma-1-2794	NS_000200.1	-	99938025	99937976
Sma-1-2795	NW_003037807.1	-	212802	212753
Sma-1-2796	NS_000200.1	-	30474318	30474269
Sma-1-2797	NW_003037850.1	-	554269	554220
Sma-1-2798	NS_000200.1	-	92540299	92540250
Sma-1-2799	NW_003037918.1	-	489526	489477
Sma-1-2800	NS_000200.1	-	167056516	167056467
Sma-1-2801	NW_003037804.1	+	683313	683362
Sma-1-2802	NS_000200.1	+	25823333	25823382
Sma-1-2803	NW_003026618.1	-	159218	159169
Sma-1-2804	NS_000200.1	-	321914220	321914171
Sma-1-2805	NW_003026618.1	-	125341	125292
Sma-1-2806	NS_000200.1	-	321880343	321880294
Sma-1-2807	NW_003037926.1	+	181498	181547
Sma-1-2808	NS_000200.1	+	173349768	173349817
Sma-1-2809	NW_003026456.1	+	301816	301865
Sma-1-2810	NS_000200.1	+	58224952	58225001
Sma-1-2811	NW_003037851.1	-	26502	26453
Sma-1-2812	NS_000200.1	-	93429812	93429763
Sma-1-2813	NW_003037940.1	-	396542	396493
Sma-1-2814	NS_000200.1	-	184138672	184138623
Sma-1-2815	NW_003026594.1	-	39558	39509
Sma-1-2816	NS_000200.1	-	304983928	304983879
Sma-1-2817	NW_003038171.1	+	29510	29559
Sma-1-2818	NS_000200.1	+	300669814	300669863

Sma-1-2819	NW_003038010.1	-	325684	325635
Sma-1-2820	NS_000200.1	-	227143907	227143858
Sma-1-2821	NW_003037831.1	-	1687715	1687666
Sma-1-2822	NS_000200.1	-	71137017	71136968
Sma-1-2823	NW_003037938.1	-	41075	41026
Sma-1-2824	NS_000200.1	-	182078188	182078139
Sma-1-2825	NW_003037809.1	+	2038136	2038185
Sma-1-2826	NS_000200.1	+	35702600	35702649
Sma-1-2827	NW_003037925.1	+	270313	270362
Sma-1-2828	NS_000200.1	+	172672316	172672365
Sma-1-2829	NW_003037918.1	+	490733	490782
Sma-1-2830	NS_000200.1	+	167057723	167057772
Sma-1-2831	NW_003037793.1	-	2621752	2621703
Sma-1-2832	NS_000200.1	-	2621752	2621703
Sma-1-2833	NW_003038224.1	-	72625	72576
Sma-1-2834	NS_000200.1	-	309484353	309484304
Sma-1-2835	NW_003037925.1	-	166533	166484
Sma-1-2836	NS_000200.1	-	172568536	172568487
Sma-1-2837	NW_003038011.1	-	481719	481670
Sma-1-2838	NS_000200.1	-	228009369	228009320
Sma-1-2839	NW_003037852.1	-	473415	473366
Sma-1-2840	NW_003038011.1	-	483003	482954
Sma-1-2841	NS_000200.1	-	228010653	228010604
Sma-1-2842	NS_000200.1	-	94865310	94865261
Sma-1-2843	NW_003037940.1	+	142466	142515
Sma-1-2844	NS_000200.1	+	183884596	183884645
Sma-1-2845	NW_003037809.1	+	1723962	1724011
Sma-1-2846	NS_000200.1	+	35388426	35388475
Sma-1-2847	NW_003037981.1	+	1025584	1025633
Sma-1-2848	NS_000200.1	+	213423452	213423501
Sma-1-2849	NW_003037918.1	+	1797515	1797564
Sma-1-2850	NS_000200.1	+	168364505	168364554
Sma-1-2851	NW_003026458.1	+	179491	179540
Sma-1-2852	NS_000200.1	+	102047721	102047770
Sma-1-2853	NW_003037798.1	+	2708064	2708113
Sma-1-2854	NS_000200.1	+	16967222	16967271
Sma-1-2855	NW_003037816.1	+	992686	992735
Sma-1-2856	NS_000200.1	+	47201558	47201607
Sma-1-2857	NW_003038050.1	+	226429	226478
Sma-1-2858	NS_000200.1	+	256501323	256501372
Sma-1-2859	NW_003026584.1	-	50950	50901
Sma-1-2860	NS_000200.1	-	303705698	303705649
Sma-1-2861	NW_003037800.1	-	2358577	2358528
Sma-1-2862	NS_000200.1	-	20987937	20987888
Sma-1-2863	NW_003037827.1	-	780693	780644
Sma-1-2864	NS_000200.1	-	63402834	63402785
Sma-1-2865	NW_003038921.1	+	1519	1568
Sma-1-2866	NW_003038023.1	+	244370	244419
Sma-1-2867	NS_000200.1	+	237404661	237404710
Sma-1-2868	NS_000200.1	+	355588690	355588739
Sma-1-2869	NW_003037809.1	+	1485838	1485889
Sma-1-2870	NS_000200.1	+	35150302	35150353

Sma-1-2871	NW_003026458.1	+	494264	494313
Sma-1-2872	NS_000200.1	+	102362494	102362543
Sma-1-2873	NW_003037903.1	+	134232	134281
Sma-1-2874	NS_000200.1	+	147688313	147688362
Sma-1-2875	NW_003038019.1	-	254792	254743
Sma-1-2876	NS_000200.1	-	233571610	233571561
Sma-1-2877	NW_003037908.1	-	974941	974892
Sma-1-2878	NS_000200.1	-	153448072	153448023
Sma-1-2879	NW_003037928.1	+	201896	201945
Sma-1-2880	NS_000200.1	+	174525849	174525898
Sma-1-2881	NW_003037808.1	-	797536	797487
Sma-1-2882	NS_000200.1	-	32584173	32584124
Sma-1-2883	NW_003026458.1	+	70616	70665
Sma-1-2884	NS_000200.1	+	101938846	101938895
Sma-1-2885	NW_003037926.1	-	586323	586274
Sma-1-2886	NS_000200.1	-	173754593	173754544
Sma-1-2887	NW_003037981.1	-	309706	309657
Sma-1-2888	NS_000200.1	-	212707574	212707525
Sma-1-2889	NW_003037972.1	-	445224	445175
Sma-1-2890	NS_000200.1	-	202920928	202920879
Sma-1-2891	NW_003037832.1	-	481949	481896
Sma-1-2892	NS_000200.1	-	71691380	71691327
Sma-1-2893	NW_003037992.1	+	109073	109122
Sma-1-2894	NS_000200.1	+	217950554	217950603
Sma-1-2895	NW_003031067.1	-	885	836
Sma-1-2896	NS_000200.1	-	325639272	325639223
Sma-1-2897	NW_003038219.1	+	18157	18206
Sma-1-2898	NS_000200.1	+	308198578	308198627
Sma-1-2899	NW_003037802.1	+	1022733	1022782
Sma-1-2900	NS_000200.1	+	23025527	23025576
Sma-1-2901	NW_003037974.1	-	660548	660499
Sma-1-2902	NS_000200.1	-	205335854	205335805
Sma-1-2903	NW_003037903.1	-	787915	787866
Sma-1-2904	NS_000200.1	-	148341996	148341947
Sma-1-2905	NW_003037982.1	+	1114843	1114892
Sma-1-2906	NS_000200.1	+	214774038	214774087
Sma-1-2907	NW_003037793.1	-	4123677	4123628
Sma-1-2908	NS_000200.1	-	4123677	4123628
Sma-1-2909	NW_003026460.1	+	379164	379214
Sma-1-2910	NS_000200.1	+	170901370	170901420
Sma-1-2911	NW_003038512.1	-	80176	80127
Sma-1-2912	NS_000200.1	-	338636873	338636824
Sma-1-2913	NW_003026460.1	+	491352	491401
Sma-1-2914	NS_000200.1	+	171013558	171013607
Sma-1-2915	NW_003037915.1	+	388894	388943
Sma-1-2916	NS_000200.1	+	162288880	162288929
Sma-1-2917	NW_003037799.1	+	1336808	1336858
Sma-1-2918	NS_000200.1	+	18327162	18327212
Sma-1-2919	NW_003038218.1	-	71202	71153
Sma-1-2920	NS_000200.1	-	307511500	307511451
Sma-1-2921	NW_003038135.1	-	121048	120999
Sma-1-2922	NS_000200.1	-	283993387	283993338

Sma-1-2923	NW_003037937.1	-	667692	667643
Sma-1-2924	NS_000200.1	-	181867601	181867552
Sma-1-2925	NW_003037982.1	-	1038207	1038158
Sma-1-2926	NS_000200.1	-	214697402	214697353
Sma-1-2927	NW_003037807.1	-	956732	956682
Sma-1-2928	NS_000200.1	-	31218248	31218198
Sma-1-2929	NW_003037917.1	+	327717	327766
Sma-1-2930	NS_000200.1	+	165364521	165364570
Sma-1-2931	NW_003026462.1	+	467584	467633
Sma-1-2932	NS_000200.1	+	197135087	197135136
Sma-1-2933	NW_003038000.1	+	55157	55207
Sma-1-2934	NS_000200.1	+	222525935	222525985
Sma-1-2935	NW_003037924.1	-	123908	123858
Sma-1-2936	NS_000200.1	-	171830866	171830816
Sma-1-2937	NW_003037796.1	+	974570	974619
Sma-1-2938	NS_000200.1	+	9184214	9184263
Sma-1-2939	NW_003038224.1	-	160160	160111
Sma-1-2940	NS_000200.1	-	309571888	309571839
Sma-1-2941	NW_003038517.1	-	171212	171163
Sma-1-2942	NS_000200.1	-	340935700	340935651
Sma-1-2943	NW_003038257.1	+	495667	495745
Sma-1-2944	NS_000200.1	+	321669995	321670073
Sma-1-2945	NW_003035866.1	+	71489	71538
Sma-1-2946	NS_000200.1	+	334165299	334165348
Sma-1-2947	NS_000200.1	+	365467216	365467265
env-81	AB231864.1	-	107811	107766
Sma-1-2948	NW_003022932.1	-	2211	2166
Sma-1-2949	NW_003037980.1	-	285776	285731
Sma-1-2950	NS_000200.1	-	371905439	371905394
Sma-1-2951	NS_000200.1	-	211246638	211246593
Sma-1-2952	NW_003038124.1	+	218524	218573
Sma-1-2953	NS_000200.1	+	279211168	279211217
Sma-1-2954	NW_003037904.1	+	231699	231747
Sma-1-2955	NS_000200.1	+	148946198	148946246
Sma-1-2956	NW_003037978.1	-	327690	327641
Sma-1-2957	NS_000200.1	-	209595700	209595651
Sma-1-2958	NW_003026510.1	+	42387	42436
Sma-1-2959	NS_000200.1	+	283482568	283482617
Sma-1-2960	NW_003037908.1	+	928012	928061
Sma-1-2961	NW_003037908.1	-	940027	939978
Sma-1-2962	NS_000200.1	+	153401143	153401192
Sma-1-2963	NS_000200.1	-	153413158	153413109
Sma-1-2964	NW_003037967.1	+	471923	471972
Sma-1-2965	NS_000200.1	+	199711894	199711943
Sma-1-2966	NW_003037821.1	+	1113666	1113715
Sma-1-2967	NS_000200.1	+	54258150	54258199
Sma-1-2968	NW_003038450.1	-	3079	3030
Sma-1-2969	NW_003037797.1	+	3213702	3213751
Sma-1-2970	NW_003037797.1	+	3217539	3217588
Sma-1-2971	NS_000200.1	+	13641562	13641611
Sma-1-2972	NS_000200.1	+	13645399	13645448
Sma-1-2973	NS_000200.1	-	330533980	330533931

Sma-1-2974	NW_003038087.1	+	126265	126314
Sma-1-2975	NS_000200.1	+	273154666	273154715
Sma-1-2976	NW_003037789.1	+	53182	53231
Sma-1-2977	NS_000200.1	+	380837802	380837851
Sma-1-2978	NW_003037851.1	-	502124	502075
Sma-1-2979	NS_000200.1	-	93905434	93905385
Sma-1-2980	NW_003026516.1	+	361215	361264
Sma-1-2981	NS_000200.1	+	299469512	299469561
Sma-1-2982	NW_003037812.1	-	935294	935245
Sma-1-2983	NS_000200.1	-	40418033	40417984
Sma-1-2984	NW_003035573.1	-	3522	3473
Sma-1-2985	NS_000200.1	-	328461731	328461682
Sma-1-2986	NW_003037882.1	-	227216	227167
Sma-1-2987	NS_000200.1	-	125518890	125518841
Sma-1-2988	NW_003037878.1	-	295978	295929
Sma-1-2989	NS_000200.1	-	122349456	122349407
Sma-1-2990	NW_003038028.1	-	757905	757857
Sma-1-2991	NS_000200.1	-	242065479	242065431
Sma-1-2992	NW_003026464.1	+	499923	499972
Sma-1-2993	NS_000200.1	+	203800232	203800281
Sma-1-2994	NW_003029000.1	+	1882	1931
Sma-1-2995	NS_000200.1	+	375129325	375129374
Sma-1-2996	NW_003037944.1	-	276186	276137
Sma-1-2997	NS_000200.1	-	188497539	188497490
Sma-1-2998	NW_003038120.1	-	123780	123731
Sma-1-2999	NS_000200.1	-	278309003	278308954
Sma-1-3000	NW_003037810.1	-	149359	149310
Sma-1-3001	NW_003038261.1	+	620681	620730
Sma-1-3002	NS_000200.1	+	324234332	324234381
Sma-1-3003	NS_000200.1	-	36753881	36753832
Sma-1-3004	NW_003037847.1	+	138571	138620
Sma-1-3005	NS_000200.1	+	88790301	88790350
Sma-1-3006	NW_003037860.1	+	342482	342531
Sma-1-3007	NS_000200.1	+	104085510	104085559
Sma-1-3008	NW_003037864.1	+	167011	167060
Sma-1-3009	NS_000200.1	+	108659585	108659634
Sma-1-3010	NW_003038252.1	+	31907	31956
Sma-1-3011	NS_000200.1	+	318887495	318887544
Sma-1-3012	NW_003020602.1	+	3616	3665
Sma-1-3013	NS_000200.1	+	360369865	360369914
Sma-1-3014	NW_003037833.1	-	695149	695100
Sma-1-3015	NS_000200.1	-	72886245	72886196
Sma-1-3016	NW_003037832.1	-	656951	656902
Sma-1-3017	NS_000200.1	-	71866382	71866333
Sma-1-3018	NW_003037975.1	+	357734	357783
Sma-1-3019	NS_000200.1	+	205939958	205940007
Sma-1-3020	NW_003024225.1	+	969	1018
Sma-1-3021	NS_000200.1	+	348588149	348588198
Sma-1-3022	NW_003028936.1	+	252	301
Sma-1-3023	NS_000200.1	+	370262939	370262988
Sma-1-3024	NW_003037816.1	+	40028	40077
Sma-1-3025	NS_000200.1	+	46248900	46248949

Sma-1-3026	NW_003037914.1	-	1643117	1643068
Sma-1-3027	NS_000200.1	-	161787367	161787318
Sma-1-3028	NW_003038033.1	+	93822	93871
Sma-1-3029	NS_000200.1	+	246073824	246073873
Sma-1-3030	NW_003037809.1	-	2452855	2452806
Sma-1-3031	NS_000200.1	-	36117319	36117270
Sma-1-3032	NW_003037846.1	-	938914	938865
Sma-1-3033	NS_000200.1	-	88573484	88573435
Sma-1-3034	NW_003037942.1	-	40441	40392
Sma-1-3035	NS_000200.1	-	185800349	185800300
Sma-1-3036	NW_003038042.1	-	159380	159331
Sma-1-3037	NS_000200.1	-	254104979	254104930
Sma-1-3038	NW_003037839.1	+	771497	771546
Sma-1-3039	NS_000200.1	+	79729647	79729696
Sma-1-3040	NW_003037903.1	+	699606	699655
Sma-1-3041	NS_000200.1	+	148253687	148253736
Sma-1-3042	NW_003037832.1	+	452361	452410
Sma-1-3043	NS_000200.1	+	71661792	71661841
Sma-1-3044	NW_003037904.1	-	260820	260771
Sma-1-3045	NS_000200.1	-	148975319	148975270
Sma-1-3046	NW_003037982.1	-	147501	147452
Sma-1-3047	NS_000200.1	-	213806696	213806647
Sma-1-3048	NW_003037981.1	+	165461	165510
Sma-1-3049	NS_000200.1	+	212563329	212563378
Sma-1-3050	NW_003037945.1	+	516918	516967
Sma-1-3051	NS_000200.1	+	189618882	189618931
Sma-1-3052	NW_003037800.1	-	1808209	1808160
Sma-1-3053	NS_000200.1	-	20437569	20437520
Sma-1-3054	NW_003038224.1	-	149383	149334
Sma-1-3055	NS_000200.1	-	309561111	309561062
Sma-1-3056	NW_003020184.1	+	316	365
Sma-1-3057	NS_000200.1	+	371153957	371154006
Sma-1-3058	NW_003026464.1	+	545270	545319
Sma-1-3059	NS_000200.1	+	203845579	203845628
Sma-1-3060	NW_003037903.1	+	663513	663562
Sma-1-3061	NS_000200.1	+	148217594	148217643
Sma-1-3062	NW_003037866.1	-	927962	927913
Sma-1-3063	NS_000200.1	-	112415761	112415712
Sma-1-3064	NW_003037827.1	-	1724632	1724583
Sma-1-3065	NS_000200.1	-	64346773	64346724
Sma-1-3066	NW_003037969.1	+	399857	399906
Sma-1-3067	NS_000200.1	+	201005984	201006033
Sma-1-3068	NW_003025258.1	-	614	565
Sma-1-3069	NS_000200.1	-	361497286	361497237
Sma-1-3070	NW_003038019.1	+	319566	319615
Sma-1-3071	NS_000200.1	+	233636384	233636433
Sma-1-3072	NW_003037944.1	+	624828	624877
Sma-1-3073	NS_000200.1	+	188846181	188846230
Sma-1-3074	NW_003037809.1	-	1667336	1667287
Sma-1-3075	NS_000200.1	-	35331800	35331751
Sma-1-3076	NW_003038148.1	+	92307	92356
Sma-1-3077	NS_000200.1	+	290166291	290166340

Sma-1-3078	NW_003037913.1	+	863599	863648
Sma-1-3079	NS_000200.1	+	159427350	159427399
Sma-1-3080	NW_003038036.1	+	258951	259000
Sma-1-3081	NS_000200.1	+	249192803	249192852
Sma-1-3082	NW_003038037.1	-	1565870	1565821
Sma-1-3083	NW_003038056.1	-	53994	53945
Sma-1-3084	NS_000200.1	-	259031645	259031596
Sma-1-3085	NS_000200.1	-	251721279	251721230
Sma-1-3086	NW_003037894.1	-	371707	371658
Sma-1-3087	NS_000200.1	-	142268280	142268231
Sma-1-3088	NW_003037943.1	-	886840	886791
Sma-1-3089	NS_000200.1	-	187908373	187908324
Sma-1-3090	NW_003037803.1	-	346822	346773
Sma-1-3091	NS_000200.1	-	23847359	23847310
Sma-1-3092	NW_003038243.1	-	194832	194783
Sma-1-3093	NS_000200.1	-	315133316	315133267
Sma-1-3094	NW_003038031.1	+	30052	30101
Sma-1-3095	NS_000200.1	+	244307810	244307859
Sma-1-3096	NW_003038244.1	-	278567	278518
Sma-1-3097	NS_000200.1	-	315423772	315423723
Sma-1-3098	NW_003037807.1	-	1304567	1304518
Sma-1-3099	NS_000200.1	-	31566083	31566034
Sma-1-3100	NW_003037999.1	-	128551	128502
Sma-1-3101	NS_000200.1	-	221823105	221823056
Sma-1-3102	NW_003026609.1	-	117900	117851
Sma-1-3103	NS_000200.1	-	309772892	309772843
Sma-1-3104	NW_003037854.1	-	151184	151135
Sma-1-3105	NW_003038152.1	-	226856	226807
Sma-1-3106	NS_000200.1	-	292832580	292832531
Sma-1-3107	NS_000200.1	-	97055605	97055556
Sma-1-3108	NW_003038224.1	-	156043	155994
Sma-1-3109	NS_000200.1	-	309567771	309567722
Sma-1-3110	NW_003027803.1	-	1135	1086
Sma-1-3111	NW_003037916.1	+	1284316	1284365
Sma-1-3112	NS_000200.1	+	164477727	164477776
Sma-1-3113	NS_000200.1	-	365958061	365958012
Sma-1-3114	NW_003037871.1	-	290855	290806
Sma-1-3115	NS_000200.1	-	117738593	117738544
Sma-1-3116	NW_003037864.1	+	157701	157750
Sma-1-3117	NS_000200.1	+	108650275	108650324
Sma-1-3118	NW_003037868.1	-	13957	13908
Sma-1-3119	NS_000200.1	-	115985669	115985620
Sma-1-3120	NW_003038502.1	+	173823	173872
Sma-1-3121	NS_000200.1	+	335172868	335172917
Sma-1-3122	NW_003037799.1	+	1128056	1128105
Sma-1-3123	NS_000200.1	+	18118410	18118459
Sma-1-3124	NW_003037809.1	+	2746232	2746281
Sma-1-3125	NS_000200.1	+	36410696	36410745
Sma-1-3126	NW_003038008.1	-	203654	203605
Sma-1-3127	NS_000200.1	-	225876750	225876701
Sma-1-3128	NW_003037915.1	+	193890	193939
Sma-1-3129	NS_000200.1	+	162093876	162093925

Sma-1-3130	NW_003037821.1	-	691687	691638
Sma-1-3131	NS_000200.1	-	53836171	53836122
Sma-1-3132	NW_003037854.1	-	1002530	1002481
Sma-1-3133	NS_000200.1	-	97906951	97906902
Sma-1-3134	NW_003037982.1	+	588444	588493
Sma-1-3135	NS_000200.1	+	214247639	214247688
Sma-1-3136	NW_003038216.1	+	86928	86977
Sma-1-3137	NS_000200.1	+	306872560	306872609
Sma-1-3138	NW_003037903.1	+	1031582	1031631
Sma-1-3139	NS_000200.1	+	148585663	148585712
Sma-1-3140	NW_003026458.1	-	431635	431586
Sma-1-3141	NS_000200.1	-	102299865	102299816
Sma-1-3142	NW_003037831.1	-	976781	976732
Sma-1-3143	NS_000200.1	-	70426083	70426034
Sma-1-3144	NW_003037902.1	+	374708	374757
Sma-1-3145	NS_000200.1	+	147129942	147129991
Sma-1-3146	NW_003037831.1	+	949404	949453
Sma-1-3147	NS_000200.1	+	70398706	70398755
Sma-1-3148	NW_003026458.1	+	391700	391749
Sma-1-3149	NS_000200.1	+	102259930	102259979
Sma-1-3150	NW_003037904.1	+	317896	317945
Sma-1-3151	NS_000200.1	+	149032395	149032444
Sma-1-3152	NW_003037902.1	+	229460	229509
Sma-1-3153	NS_000200.1	+	146984694	146984743
Sma-1-3154	NW_003037986.1	-	109970	109921
Sma-1-3155	NS_000200.1	-	215526722	215526673
Sma-1-3156	NW_003037996.1	+	368522	368571
Sma-1-3157	NW_003037996.1	-	281070	281021
Sma-1-3158	NS_000200.1	+	220613398	220613447
Sma-1-3159	NS_000200.1	-	220525946	220525897
Sma-1-3160	NW_003037981.1	-	1136217	1136168
Sma-1-3161	NS_000200.1	-	213534085	213534036
Sma-1-3162	NW_003026458.1	+	115045	115094
Sma-1-3163	NS_000200.1	+	101983275	101983324
Sma-1-3164	NW_003037841.1	+	1575510	1575559
Sma-1-3165	NS_000200.1	+	84186691	84186740
Sma-1-3166	NW_003026456.1	-	93700	93651
Sma-1-3167	NS_000200.1	-	58016836	58016787
Sma-1-3168	NW_003026605.1	+	121790	121839
Sma-1-3169	NS_000200.1	+	307800654	307800703
Sma-1-3170	NW_003026605.1	-	14128	14079
Sma-1-3171	NS_000200.1	-	307692992	307692943
Sma-1-3172	NW_003037954.1	-	47267	47218
Sma-1-3173	NS_000200.1	-	193276889	193276840
Sma-1-3174	NW_003037844.1	-	122735	122686
Sma-1-3175	NS_000200.1	-	86306181	86306132
Sma-1-3176	NW_003026456.1	-	739281	739232
Sma-1-3177	NS_000200.1	-	58662417	58662368
Sma-1-3178	NW_003037988.1	+	34134	34183
Sma-1-3179	NS_000200.1	+	216027952	216028001
Sma-1-3180	NW_003038515.1	-	82313	82264
Sma-1-3181	NS_000200.1	-	340166378	340166329

Sma-1-3182	NW_003037988.1	+	74137	74186
Sma-1-3183	NS_000200.1	+	216067955	216068004
Sma-1-3184	NW_003026456.1	+	434255	434304
Sma-1-3185	NW_003038030.1	-	476314	476265
Sma-1-3186	NS_000200.1	+	58357391	58357440
Sma-1-3187	NS_000200.1	-	243612181	243612132
Sma-1-3188	NW_003037989.1	+	108131	108180
Sma-1-3189	NS_000200.1	+	216718690	216718739
Sma-1-3190	NW_003037906.1	-	704905	704856
Sma-1-3191	NS_000200.1	-	151012684	151012635
Sma-1-3192	NW_003037885.1	-	829560	829511
Sma-1-3193	NS_000200.1	-	128927507	128927458
Sma-1-3194	NW_003038221.1	-	128613	128564
Sma-1-3195	NS_000200.1	-	308769877	308769828
Sma-1-3196	NW_003025257.1	+	6	55
Sma-1-3197	NS_000200.1	+	361495542	361495591
Sma-1-3198	NW_003026516.1	+	608	657
Sma-1-3199	NS_000200.1	+	299108905	299108954
Sma-1-3200	NW_003037814.1	-	1604488	1604439
Sma-1-3201	NS_000200.1	-	44588348	44588299
Sma-1-3202	NW_003038122.1	-	22380	22331
Sma-1-3203	NS_000200.1	-	278541607	278541558
Sma-1-3204	NW_003037890.1	+	1848568	1848617
Sma-1-3205	NS_000200.1	+	137531929	137531978
Sma-1-3206	NW_003037825.1	+	1030292	1030341
Sma-1-3207	NS_000200.1	+	60160074	60160123
Sma-1-3208	NW_003037858.1	+	639394	639443
Sma-1-3209	NS_000200.1	+	101303670	101303719
Sma-1-3210	NW_003038248.1	-	49038	48989
Sma-1-3211	NS_000200.1	-	316360305	316360256
Sma-1-3212	NW_003038028.1	+	727111	727160
Sma-1-3213	NS_000200.1	+	242034685	242034734
Sma-1-3214	NW_003037807.1	+	1438971	1439020
Sma-1-3215	NS_000200.1	+	31700487	31700536
Sma-1-3216	NW_003037993.1	-	183104	183055
Sma-1-3217	NS_000200.1	-	218369807	218369758
Sma-1-3218	NW_003037993.1	+	205900	205949
Sma-1-3219	NS_000200.1	+	218392603	218392652
Sma-1-3220	NW_003026456.1	-	159630	159581
Sma-1-3221	NS_000200.1	-	58082766	58082717
Sma-1-3222	NW_003037773.1	+	50608	50657
Sma-1-3223	NS_000200.1	+	378572964	378573013
Sma-1-3224	NW_003026430.1	+	30	79
Sma-1-3225	NS_000200.1	+	344998929	344998978
Sma-1-3226	NW_003037835.1	+	526163	526212
Sma-1-3227	NS_000200.1	+	74922671	74922720
Sma-1-3228	NW_003026465.1	-	663689	663640
Sma-1-3229	NS_000200.1	-	208056283	208056234
Sma-1-3230	NW_003037918.1	+	1308031	1308080
Sma-1-3231	NS_000200.1	+	167875021	167875070
Sma-1-3232	NW_003026303.1	+	6	55
Sma-1-3233	NS_000200.1	+	344700252	344700301

Sma-1-3234	NW_003025800.1	+	721	770
Sma-1-3235	NS_000200.1	+	356551922	356551971
Sma-1-3236	NW_003025628.1	-	931	882
Sma-1-3237	NW_003038028.1	+	52813	52862
Sma-1-3238	NS_000200.1	+	241360387	241360436
Sma-1-3239	NS_000200.1	-	356265189	356265140
Sma-1-3240	NW_003037836.1	-	199510	199461
Sma-1-3241	NS_000200.1	-	75756609	75756560
Sma-1-3242	NW_003037905.1	-	307300	307251
Sma-1-3243	NS_000200.1	-	149576312	149576263
Sma-1-3244	NW_003038021.1	-	414199	414150
Sma-1-3245	NS_000200.1	-	235242774	235242725
Sma-1-3246	NW_003037845.1	-	12697	12648
Sma-1-3247	NS_000200.1	-	87014600	87014551
Sma-1-3248	NW_003037836.1	+	199997	200046
Sma-1-3249	NS_000200.1	+	75757096	75757145
Sma-1-3250	NW_003037831.1	-	1219497	1219448
Sma-1-3251	NS_000200.1	-	70668799	70668750
Sma-1-3252	NW_003037868.1	+	7615	7664
Sma-1-3253	NS_000200.1	+	115979327	115979376
Sma-1-3254	NW_003037831.1	-	1166629	1166580
Sma-1-3255	NS_000200.1	-	70615931	70615882
Sma-1-3256	NW_003037994.1	-	552551	552502
Sma-1-3257	NS_000200.1	-	219084365	219084316
Sma-1-3258	NW_003037858.1	-	541098	541049
Sma-1-3259	NS_000200.1	-	101205374	101205325
Sma-1-3260	NW_003037994.1	-	566610	566561
Sma-1-3261	NS_000200.1	-	219098424	219098375
Sma-1-3262	NW_003037807.1	-	782247	782198
Sma-1-3263	NS_000200.1	-	31043763	31043714
Sma-1-3264	NW_003025267.1	-	533	484
Sma-1-3265	NS_000200.1	-	361512356	361512307
Sma-1-3266	NW_003037846.1	-	462817	462768
Sma-1-3267	NS_000200.1	-	88097387	88097338
Sma-1-3268	NW_003037996.1	-	175123	175074
Sma-1-3269	NS_000200.1	-	220419999	220419950
Sma-1-3270	NW_003034567.1	+	1740	1789
Sma-1-3271	NS_000200.1	+	327642634	327642683
Sma-1-3272	NW_003037793.1	-	3394110	3394061
Sma-1-3273	NW_003038517.1	+	244201	244250
Sma-1-3274	NS_000200.1	+	341008689	341008738
Sma-1-3275	NS_000200.1	-	3394110	3394061
Sma-1-3276	NW_003038153.1	+	555787	555836
Sma-1-3277	NS_000200.1	+	293617836	293617885
Sma-1-3278	NW_003037890.1	-	332928	332879
Sma-1-3279	NS_000200.1	-	136016289	136016240
Sma-1-3280	NW_003037918.1	+	440986	441035
Sma-1-3281	NS_000200.1	+	167007976	167008025
Sma-1-3282	NW_003038242.1	-	87184	87135
Sma-1-3283	NS_000200.1	-	314786330	314786281
Sma-1-3284	NW_003026469.1	+	207333	207382
Sma-1-3285	NS_000200.1	+	217828992	217829041

Sma-1-3286	NW_003026466.1	-	26389	26340
Sma-1-3287	NS_000200.1	-	215293496	215293447
Sma-1-3288	NW_003026466.1	+	26633	26682
Sma-1-3289	NS_000200.1	+	215293740	215293789
Sma-1-3290	NW_003026466.1	-	42469	42420
Sma-1-3291	NS_000200.1	-	215309576	215309527
Sma-1-3292	NW_003037831.1	-	1125182	1125133
Sma-1-3293	NS_000200.1	-	70574484	70574435
Sma-1-3294	NW_003037836.1	-	329103	329054
Sma-1-3295	NS_000200.1	-	75886202	75886153
Sma-1-3296	NW_003037995.1	-	237550	237501
Sma-1-3297	NS_000200.1	-	219927318	219927269
Sma-1-3298	NW_003037836.1	-	474542	474493
Sma-1-3299	NS_000200.1	-	76031641	76031592
Sma-1-3300	NW_003037817.1	-	524885	524835
Sma-1-3301	NS_000200.1	-	47907237	47907187
Sma-1-3302	NW_003037817.1	+	523360	523410
Sma-1-3303	NS_000200.1	+	47905712	47905762
Sma-1-3304	NW_003037995.1	+	468200	468249
Sma-1-3305	NS_000200.1	+	220157968	220158017
Sma-1-3306	NW_003026487.1	+	98128	98178
Sma-1-3307	NS_000200.1	+	261008822	261008872
Sma-1-3308	NW_003037996.1	-	205702	205653
Sma-1-3309	NS_000200.1	-	220450578	220450529
Sma-1-3310	NW_003038079.1	+	305570	305619
Sma-1-3311	NS_000200.1	+	268435558	268435607
Sma-1-3312	NW_003037937.1	-	615858	615809
Sma-1-3313	NS_000200.1	-	181815767	181815718
Sma-1-3314	NW_003037997.1	-	379504	379455
Sma-1-3315	NS_000200.1	-	221190946	221190897
Sma-1-3316	NW_003024257.1	+	546	595
Sma-1-3317	NS_000200.1	+	348650706	348650755
Sma-1-3318	NW_003038042.1	-	136204	136155
Sma-1-3319	NS_000200.1	-	254081803	254081754
Sma-1-3320	NW_003037979.1	-	60545	60496
Sma-1-3321	NS_000200.1	-	210121565	210121516
Sma-1-3322	NW_003024194.1	+	353	402
Sma-1-3323	NS_000200.1	+	348539414	348539463
Sma-1-3324	NW_003037999.1	+	110644	110693
Sma-1-3325	NS_000200.1	+	221805198	221805247
Sma-1-3326	NW_003037830.1	-	740979	740930
Sma-1-3327	NS_000200.1	-	68631350	68631301
Sma-1-3328	NW_003037837.1	-	227768	227719
Sma-1-3329	NS_000200.1	-	77033626	77033577
Sma-1-3330	NW_003023565.1	-	2908	2859
Sma-1-3331	NS_000200.1	-	373701837	373701788
Sma-1-3332	NW_003026471.1	+	103499	103548
Sma-1-3333	NS_000200.1	+	222215732	222215781
Sma-1-3334	NW_003026605.1	-	46896	46847
Sma-1-3335	NS_000200.1	-	307725760	307725711
Sma-1-3336	NW_003037837.1	+	58487	58536
Sma-1-3337	NW_003037837.1	+	84874	84923

Sma-1-3338	NW_003037837.1	-	105841	105792
Sma-1-3339	NS_000200.1	+	76864345	76864394
Sma-1-3340	NS_000200.1	+	76890732	76890781
Sma-1-3341	NS_000200.1	-	76911699	76911650
Sma-1-3342	NW_003037979.1	+	452870	452919
Sma-1-3343	NS_000200.1	+	210513890	210513939
Sma-1-3344	NW_003038000.1	-	109188	109139
Sma-1-3345	NS_000200.1	-	222579966	222579917
Sma-1-3346	NW_003026471.1	-	139952	139903
Sma-1-3347	NS_000200.1	-	222252185	222252136
Sma-1-3348	NW_003038000.1	+	80055	80104
Sma-1-3349	NS_000200.1	+	222550833	222550882
Sma-1-3350	NW_003037830.1	+	697208	697257
Sma-1-3351	NS_000200.1	+	68587579	68587628
Sma-1-3352	NW_003038001.1	+	31014	31063
Sma-1-3353	NS_000200.1	+	222740575	222740624
Sma-1-3354	NW_003037838.1	-	662206	662157
Sma-1-3355	NS_000200.1	-	78660769	78660720
Sma-1-3356	NW_003037900.1	-	243490	243441
Sma-1-3357	NS_000200.1	-	145372356	145372307
Sma-1-3358	NW_003035870.1	+	2409	2458
Sma-1-3359	NS_000200.1	+	335367698	335367747
Sma-1-3360	NW_003038010.1	-	392986	392937
Sma-1-3361	NS_000200.1	-	227211209	227211160
Sma-1-3362	NW_003038001.1	-	39492	39443
Sma-1-3363	NS_000200.1	-	222749053	222749004
Sma-1-3364	NW_003026471.1	-	43272	43223
Sma-1-3365	NS_000200.1	-	222155505	222155456
Sma-1-3366	NW_003037830.1	-	637263	637214
Sma-1-3367	NS_000200.1	-	68527634	68527585
Sma-1-3368	NW_003026471.1	+	94729	94778
Sma-1-3369	NS_000200.1	+	222206962	222207011
Sma-1-3370	NW_003038003.1	-	200362	200313
Sma-1-3371	NS_000200.1	-	223699644	223699595
Sma-1-3372	NW_003037911.1	+	663663	663712
Sma-1-3373	NS_000200.1	+	156451016	156451065
Sma-1-3374	NW_003026472.1	-	112078	112029
Sma-1-3375	NS_000200.1	-	236524070	236524021
Sma-1-3376	NW_003037905.1	+	839577	839626
Sma-1-3377	NS_000200.1	+	150108589	150108638
Sma-1-3378	NW_003037839.1	-	1068338	1068289
Sma-1-3379	NS_000200.1	-	80026488	80026439
Sma-1-3380	NW_003037799.1	-	1180084	1180035
Sma-1-3381	NS_000200.1	-	18170438	18170389
Sma-1-3382	NW_003037813.1	+	1965704	1965753
Sma-1-3383	NS_000200.1	+	42952567	42952616
Sma-1-3384	NW_003037830.1	-	353513	353464
Sma-1-3385	NS_000200.1	-	68243884	68243835
Sma-1-3386	NW_003037830.1	-	289543	289493
Sma-1-3387	NW_003037915.1	-	617533	617483
Sma-1-3388	NS_000200.1	-	162517519	162517469
Sma-1-3389	NS_000200.1	-	68179914	68179864

Sma-1-3390	NW_003037839.1	-	362267	362218
Sma-1-3391	NS_000200.1	-	79320417	79320368
Sma-1-3392	NW_003037839.1	-	1236532	1236483
Sma-1-3393	NS_000200.1	-	80194682	80194633
Sma-1-3394	NW_003021694.1	-	1030	981
Sma-1-3395	NS_000200.1	-	348318026	348317977
Sma-1-3396	NW_003037839.1	-	1366705	1366656
Sma-1-3397	NS_000200.1	-	80324855	80324806
Sma-1-3398	NW_003037859.1	-	91752	91703
Sma-1-3399	NS_000200.1	-	102770593	102770544
Sma-1-3400	NW_003026472.1	+	326398	326447
Sma-1-3401	NS_000200.1	+	236738390	236738439
Sma-1-3402	NW_003037899.1	+	152466	152515
Sma-1-3403	NS_000200.1	+	144671577	144671626
Sma-1-3404	NW_003038005.1	+	225771	225820
Sma-1-3405	NS_000200.1	+	224631725	224631774
Sma-1-3406	NW_003021258.1	-	1057	1008
Sma-1-3407	NW_003037794.1	-	676480	676431
Sma-1-3408	NS_000200.1	-	371720929	371720880
Sma-1-3409	NS_000200.1	-	4856075	4856026
Sma-1-3410	NW_003038261.1	+	405007	405056
Sma-1-3411	NS_000200.1	+	324018658	324018707
Sma-1-3412	NW_003038261.1	-	121228	121179
Sma-1-3413	NS_000200.1	-	323734879	323734830
Sma-1-3414	NW_003032691.1	-	592	543
Sma-1-3415	NW_003037800.1	-	436688	436639
Sma-1-3416	NS_000200.1	-	353868057	353868008
Sma-1-3417	NS_000200.1	-	19066048	19065999
Sma-1-3418	NW_003037829.1	+	1816085	1816134
Sma-1-3419	NS_000200.1	+	67716623	67716672
Sma-1-3420	NW_003038261.1	+	1004358	1004407
Sma-1-3421	NS_000200.1	+	324618009	324618058
Sma-1-3422	NW_003038032.1	+	79341	79390
Sma-1-3423	NS_000200.1	+	245238501	245238550
Sma-1-3424	NW_003038161.1	-	493495	493446
Sma-1-3425	NS_000200.1	-	297863346	297863297
Sma-1-3426	NW_003037851.1	-	461146	461097
Sma-1-3427	NS_000200.1	-	93864456	93864407
Sma-1-3428	NW_003026472.1	-	430650	430601
Sma-1-3429	NS_000200.1	-	236842642	236842593
Sma-1-3430	NW_003037830.1	+	161109	161158
Sma-1-3431	NS_000200.1	+	68051480	68051529
Sma-1-3432	NW_003038005.1	+	300219	300268
Sma-1-3433	NS_000200.1	+	224706173	224706222
Sma-1-3434	NW_003037978.1	-	50372	50323
Sma-1-3435	NS_000200.1	-	209318382	209318333
Sma-1-3436	NW_003037978.1	+	459370	459419
Sma-1-3437	NS_000200.1	+	209727380	209727429
Sma-1-3438	NW_003029816.1	-	990	941
Sma-1-3439	NS_000200.1	-	376137430	376137381
Sma-1-3440	NW_003037858.1	+	472182	472231
Sma-1-3441	NS_000200.1	+	101136458	101136507

Sma-1-3442	NW_003038027.1	+	568154	568203
Sma-1-3443	NS_000200.1	+	240938868	240938917
Sma-1-3444	NW_003037933.1	+	224671	224720
Sma-1-3445	NS_000200.1	+	178637843	178637892
Sma-1-3446	NW_003020821.1	+	1857	1906
Sma-1-3447	NS_000200.1	+	364522139	364522188
Sma-1-3448	NW_003037940.1	-	851787	851738
Sma-1-3449	NS_000200.1	-	184593917	184593868
Sma-1-3450	NW_003023619.1	+	4162	4211
Sma-1-3451	NS_000200.1	+	373867600	373867649
Sma-1-3452	NW_003020700.1	+	375	424
Sma-1-3453	NS_000200.1	+	360687979	360688028
Sma-1-3454	NW_003032147.1	-	986	937
Sma-1-3455	NS_000200.1	-	379063260	379063211
Sma-1-3456	NW_003020811.1	+	2495	2544
Sma-1-3457	NS_000200.1	+	364503734	364503783
Sma-1-3458	NW_003037830.1	-	1478044	1477995
Sma-1-3459	NS_000200.1	-	69368415	69368366
Sma-1-3460	NW_003037839.1	+	800093	800142
Sma-1-3461	NS_000200.1	+	79758243	79758292
Sma-1-3462	NW_003037839.1	+	872917	872966
Sma-1-3463	NS_000200.1	+	79831067	79831116
Sma-1-3464	NW_003037839.1	+	923932	923981
Sma-1-3465	NS_000200.1	+	79882082	79882131
Sma-1-3466	NW_003037840.1	-	1286964	1286915
Sma-1-3467	NS_000200.1	-	81910288	81910239
Sma-1-3468	NW_003038067.1	-	118630	118581
Sma-1-3469	NS_000200.1	-	262972683	262972634
Sma-1-3470	NW_003037844.1	+	231508	231557
Sma-1-3471	NS_000200.1	+	86414954	86415003
Sma-1-3472	NW_003020545.1	+	58	107
Sma-1-3473	NS_000200.1	+	360113453	360113502
Sma-1-3474	NW_003026478.1	-	22656	22607
Sma-1-3475	NS_000200.1	-	254224205	254224156
Sma-1-3476	NW_003038042.1	+	89357	89406
Sma-1-3477	NS_000200.1	+	254034956	254035005
Sma-1-3478	NW_003037840.1	+	1837805	1837854
Sma-1-3479	NS_000200.1	+	82461129	82461178
Sma-1-3480	NW_003037978.1	+	167836	167885
Sma-1-3481	NS_000200.1	+	209435846	209435895
Sma-1-3482	NW_003037977.1	+	976708	976757
Sma-1-3483	NS_000200.1	+	209262728	209262777
Sma-1-3484	NW_003037830.1	+	1121642	1121691
Sma-1-3485	NS_000200.1	+	69012013	69012062
Sma-1-3486	NW_003026481.1	+	81738	81787
Sma-1-3487	NS_000200.1	+	255481722	255481771
Sma-1-3488	NW_003020305.1	-	975	926
Sma-1-3489	NW_003034538.1	-	2932	2883
Sma-1-3490	NS_000200.1	-	371379835	371379786
Sma-1-3491	NS_000200.1	-	326882494	326882445
Sma-1-3492	NW_003037840.1	-	438708	438659
Sma-1-3493	NS_000200.1	-	81062032	81061983

Sma-1-3494	NW_003026483.1	+	120971	121020
Sma-1-3495	NS_000200.1	+	256635495	256635544
Tcr-1-2	NZ_AAHK01020178.1	-	695	646
Sma-1-3496	NW_003039071.1	+	648	697
Sma-1-3497	NS_000200.1	+	359889966	359890015
Sma-1-3498	NW_003037841.1	-	1240662	1240613
Sma-1-3499	NS_000200.1	-	83851843	83851794
Sma-1-3500	NW_003038509.1	-	304147	304098
Sma-1-3501	NS_000200.1	-	337936599	337936550
Sma-1-3502	NW_003037912.1	+	1068034	1068083
Sma-1-3503	NS_000200.1	+	157879609	157879658
Sma-1-3504	NW_003037937.1	-	374253	374204
Sma-1-3505	NS_000200.1	-	181574162	181574113
Sma-1-3506	NW_003038122.1	-	16729	16680
Sma-1-3507	NS_000200.1	-	278535956	278535907
Sma-1-3508	NW_003037976.1	+	155732	155781
Sma-1-3509	NS_000200.1	+	206770047	206770096
Sma-1-3510	NW_003036393.1	-	9111	9062
Sma-1-3511	NS_000200.1	-	359846466	359846417
Sma-1-3512	NW_003026456.1	-	232067	232018
Sma-1-3513	NS_000200.1	-	58155203	58155154
Sma-1-3514	NW_003026510.1	+	189125	189174
Sma-1-3515	NS_000200.1	+	283629306	283629355
Sma-1-3516	NW_003037805.1	+	1316575	1316624
Sma-1-3517	NS_000200.1	+	27874419	27874468
Sma-1-3518	NW_003037805.1	-	1343094	1343045
Sma-1-3519	NS_000200.1	-	27900938	27900889
Sma-1-3520	NW_003037906.1	+	580926	580975
Sma-1-3521	NS_000200.1	+	150888705	150888754
Sma-1-3522	NW_003026488.1	-	166644	166595
Sma-1-3523	NS_000200.1	-	263673138	263673089
Sma-1-3524	NW_003037793.1	+	2676664	2676713
Sma-1-3525	NS_000200.1	+	2676664	2676713
Sma-1-3526	NW_003037938.1	+	196884	196933
Sma-1-3527	NS_000200.1	+	182233997	182234046
Sma-1-3528	NW_003038082.1	+	627563	627612
Sma-1-3529	NS_000200.1	+	270162786	270162835
Sma-1-3530	NW_003037875.1	-	366655	366606
Sma-1-3531	NS_000200.1	-	120395324	120395275
Sma-1-3532	NW_003038008.1	-	604706	604657
Sma-1-3533	NS_000200.1	-	226277802	226277753
Sma-1-3534	NW_003037841.1	-	1770665	1770616
Sma-1-3535	NS_000200.1	-	84381846	84381797
Sma-1-3536	NW_003037896.1	-	386193	386144
Sma-1-3537	NS_000200.1	-	143306616	143306567
Sma-1-3538	NW_003037841.1	-	240621	240572
Sma-1-3539	NS_000200.1	-	82851802	82851753
Sma-1-3540	NW_003037917.1	+	929272	929321
Sma-1-3541	NS_000200.1	+	165966076	165966125
Sma-1-3542	NW_003037829.1	-	1892167	1892118
Sma-1-3543	NS_000200.1	-	67792705	67792656
Sma-1-3544	NW_003037842.1	-	15841	15792

Sma-1-3545	NS_000200.1	-	85115031	85114982
Sma-1-3546	NW_003038009.1	+	133971	134020
Sma-1-3547	NS_000200.1	+	226508034	226508083
Sma-1-3548	NW_003037844.1	-	274734	274685
Sma-1-3549	NS_000200.1	-	86458180	86458131
Sma-1-3550	NW_003037806.1	+	1511885	1511934
Sma-1-3551	NS_000200.1	+	29707099	29707148
Sma-1-3552	NW_003026502.1	+	114536	114585
Sma-1-3553	NS_000200.1	+	276150972	276151021
Sma-1-3554	NW_003037906.1	+	601129	601178
Sma-1-3555	NS_000200.1	+	150908908	150908957
Sma-1-3556	NW_003037844.1	-	404009	403960
Sma-1-3557	NS_000200.1	-	86587455	86587406
Sma-1-3558	NW_003038009.1	-	319060	319011
Sma-1-3559	NS_000200.1	-	226693123	226693074
Sma-1-3560	NW_003026504.1	+	67759	67808
Sma-1-3561	NS_000200.1	+	276984525	276984574
Sma-1-3562	NW_003037906.1	+	622547	622596
Sma-1-3563	NS_000200.1	+	150930326	150930375
Sma-1-3564	NW_003037894.1	+	359462	359511
Sma-1-3565	NS_000200.1	+	142256035	142256084
Sma-1-3566	NW_003037845.1	+	273039	273088
Sma-1-3567	NS_000200.1	+	87274942	87274991
Sma-1-3568	NW_003037791.1	+	690683	690732
Sma-1-3569	NS_000200.1	+	382396111	382396160
Sma-1-3570	NW_003037807.1	+	66184	66233
Sma-1-3571	NS_000200.1	+	30327700	30327749
Sma-1-3572	NW_003038044.1	+	129243	129292
Sma-1-3573	NS_000200.1	+	254754025	254754074
Sma-1-3574	NW_003037852.1	+	579517	579566
Sma-1-3575	NS_000200.1	+	94971412	94971461
Sma-1-3576	NW_003037793.1	+	3039247	3039296
Sma-1-3577	NS_000200.1	+	3039247	3039296
Sma-1-3578	NW_003037846.1	-	182616	182567
Sma-1-3579	NS_000200.1	-	87817186	87817137
Sma-1-3580	NW_003037874.1	-	179445	179396
Sma-1-3581	NS_000200.1	-	119604395	119604346
Sma-1-3582	NW_003037809.1	+	2107625	2107674
Sma-1-3583	NS_000200.1	+	35772089	35772138
Sma-1-3584	NW_003037806.1	-	253672	253623
Sma-1-3585	NS_000200.1	-	28448886	28448837
Sma-1-3586	NW_003037846.1	+	320334	320383
Sma-1-3587	NS_000200.1	+	87954904	87954953
Sma-1-3588	NW_003037797.1	+	1409156	1409205
Sma-1-3589	NS_000200.1	+	11837016	11837065
Sma-1-3590	NW_003037806.1	-	626355	626306
Sma-1-3591	NS_000200.1	-	28821569	28821520
Sma-1-3592	NW_003038070.1	+	51992	52041
Sma-1-3593	NS_000200.1	+	264389233	264389282
Sma-1-3594	NW_003037846.1	+	825755	825804
Sma-1-3595	NS_000200.1	+	88460325	88460374
Sma-1-3596	NW_003037846.1	+	920671	920720

Sma-1-3597	NS_000200.1	+	88555241	88555290
Sma-1-3598	NW_003037891.1	+	621926	621975
Sma-1-3599	NS_000200.1	+	138503204	138503253
Sma-1-3600	NW_003038014.1	-	470412	470363
Sma-1-3601	NS_000200.1	-	230211101	230211052
Sma-1-3602	NW_003038115.1	-	91363	91314
Sma-1-3603	NS_000200.1	-	277146658	277146609
Sma-1-3604	NW_003038052.1	+	7540	7589
Sma-1-3605	NS_000200.1	+	257055016	257055065
Sma-1-3606	NW_003037891.1	+	1594312	1594361
Sma-1-3607	NS_000200.1	+	139475590	139475639
Sma-1-3608	NW_003026511.1	-	184210	184161
Sma-1-3609	NS_000200.1	-	284567262	284567213
Sma-1-3610	NW_003037916.1	-	1478302	1478253
Sma-1-3611	NS_000200.1	-	164671713	164671664
Sma-1-3612	NW_003037848.1	-	1063038	1062989
Sma-1-3613	NS_000200.1	-	90756109	90756060
Sma-1-3614	NW_003037891.1	+	1956186	1956235
Sma-1-3615	NS_000200.1	+	139837464	139837513
Sma-1-3616	NW_003038025.1	-	65470	65421
Sma-1-3617	NS_000200.1	-	238923715	238923666
Sma-1-3618	NW_003026458.1	+	531893	531942
Sma-1-3619	NS_000200.1	+	102400123	102400172
Sma-1-3620	NW_003038009.1	+	371339	371388
Sma-1-3621	NW_003038009.1	-	368769	368720
Sma-1-3622	NW_003038009.1	-	367039	366990
Sma-1-3623	NS_000200.1	+	226745402	226745451
Sma-1-3624	NS_000200.1	-	226742832	226742783
Sma-1-3625	NS_000200.1	-	226741102	226741053
Sma-1-3626	NW_003037850.1	+	1289003	1289052
Sma-1-3627	NS_000200.1	+	93275033	93275082
Sma-1-3628	NW_003026514.1	-	25130	25081
Sma-1-3629	NS_000200.1	-	288483817	288483768
Sma-1-3630	NW_003037891.1	-	1835054	1835005
Sma-1-3631	NS_000200.1	-	139716332	139716283
Sma-1-3632	NW_003037891.1	+	1593439	1593488
Sma-1-3633	NS_000200.1	+	139474717	139474766
Sma-1-3634	NW_003037891.1	+	1599189	1599238
Sma-1-3635	NS_000200.1	+	139480467	139480516
Sma-1-3636	NW_003037946.1	+	850796	850845
Sma-1-3637	NS_000200.1	+	191544656	191544705
Sma-1-3638	NW_003037809.1	-	2492122	2492073
Sma-1-3639	NS_000200.1	-	36156586	36156537
Sma-1-3640	NW_003037909.1	+	273708	273757
Sma-1-3641	NS_000200.1	+	153815357	153815406
Sma-1-3642	NW_003037851.1	+	428453	428502
Sma-1-3643	NW_003037909.1	+	281086	281135
Sma-1-3644	NS_000200.1	+	93831763	93831812
Sma-1-3645	NS_000200.1	+	153822735	153822784
Sma-1-3646	NW_003037907.1	+	630930	630979
Sma-1-3647	NS_000200.1	+	152035419	152035468
Sma-1-3648	NW_003037827.1	-	678780	678731

Sma-1-3649	NS_000200.1	-	63300921	63300872
Sma-1-3650	NW_003026516.1	+	2701	2750
Sma-1-3651	NS_000200.1	+	299110998	299111047
Sma-1-3652	NW_003037806.1	+	612309	612358
Sma-1-3653	NS_000200.1	+	28807523	28807572
Sma-1-3654	NW_003037891.1	+	1214871	1214920
Sma-1-3655	NS_000200.1	+	139096149	139096198
Sma-1-3656	NW_003037976.1	-	284985	284936
Sma-1-3657	NS_000200.1	-	206899300	206899251
Sma-1-3658	NW_003037915.1	-	560523	560474
Sma-1-3659	NS_000200.1	-	162460509	162460460
Sma-1-3660	NW_003037890.1	+	987465	987514
Sma-1-3661	NS_000200.1	+	136670826	136670875
Sma-1-3662	NW_003037933.1	+	570144	570193
Sma-1-3663	NS_000200.1	+	178983316	178983365
Sma-1-3664	NW_003037938.1	+	182784	182833
Sma-1-3665	NS_000200.1	+	182219897	182219946
Sma-1-3666	NW_003037795.1	-	436316	436267
Sma-1-3667	NS_000200.1	-	6827199	6827150
Sma-1-3668	NW_003037969.1	-	236990	236941
Sma-1-3669	NS_000200.1	-	200843117	200843068
Sma-1-3670	NW_003038010.1	-	364929	364880
Sma-1-3671	NS_000200.1	-	227183152	227183103
Sma-1-3672	NW_003038161.1	+	476427	476476
Sma-1-3673	NS_000200.1	+	297846278	297846327
Sma-1-3674	NW_003038049.1	+	35383	35432
Sma-1-3675	NS_000200.1	+	256010910	256010959
Sma-1-3676	NW_003038010.1	+	385259	385308
Sma-1-3677	NS_000200.1	+	227203482	227203531
Sma-1-3678	NW_003037818.1	-	399174	399125
Sma-1-3679	NS_000200.1	-	49114581	49114532
Sma-1-3680	NW_003037810.1	-	376014	375965
Sma-1-3681	NS_000200.1	-	36980536	36980487
Sma-1-3682	NW_003037890.1	+	654234	654283
Sma-1-3683	NS_000200.1	+	136337595	136337644
Sma-1-3684	NW_003038122.1	-	37596	37547
Sma-1-3685	NS_000200.1	-	278556823	278556774
Sma-1-3686	NW_003037913.1	-	380109	380060
Sma-1-3687	NS_000200.1	-	158943860	158943811
Sma-1-3688	NW_003037878.1	+	765267	765316
Sma-1-3689	NS_000200.1	+	122818745	122818794
Sma-1-3690	NW_003026517.1	+	417898	417947
Sma-1-3691	NS_000200.1	+	300023907	300023956
Sma-1-3692	NW_003037861.1	+	113436	113485
Sma-1-3693	NS_000200.1	+	104889890	104889939
Sma-1-3694	NW_003038517.1	+	107049	107098
Sma-1-3695	NS_000200.1	+	340871537	340871586
Sma-1-3696	NW_003038511.1	-	195849	195800
Sma-1-3697	NS_000200.1	-	338541061	338541012
Sma-1-3698	NW_003026517.1	-	465373	465324
Sma-1-3699	NS_000200.1	-	300071382	300071333
Sma-1-3700	NW_003037827.1	-	406813	406764

Sma-1-3701	NS_000200.1	-	63028954	63028905
Sma-1-3702	NW_003038050.1	+	121392	121441
Sma-1-3703	NS_000200.1	+	256396286	256396335
Sma-1-3704	NW_003037795.1	-	1156933	1156884
Sma-1-3705	NS_000200.1	-	7547816	7547767
Sma-1-3706	NW_003037890.1	+	587552	587601
Sma-1-3707	NS_000200.1	+	136270913	136270962
Sma-1-3708	NW_003037852.1	-	464280	464231
Sma-1-3709	NS_000200.1	-	94856175	94856126
Sma-1-3710	NW_003038011.1	-	221041	220992
Sma-1-3711	NS_000200.1	-	227748691	227748642
Sma-1-3712	NW_003038011.1	-	270285	270236
Sma-1-3713	NS_000200.1	-	227797935	227797886
Sma-1-3714	NW_003037873.1	+	580610	580659
Sma-1-3715	NS_000200.1	+	119319738	119319787
Sma-1-3716	NW_003037915.1	+	737391	737440
Sma-1-3717	NS_000200.1	+	162637377	162637426
Sma-1-3718	NW_003026557.1	+	136	185
Sma-1-3719	NS_000200.1	+	300988179	300988228
Sma-1-3720	NW_003038163.1	-	241774	241725
Sma-1-3721	NS_000200.1	-	298642054	298642005
Sma-1-3722	NW_003038163.1	-	297939	297890
Sma-1-3723	NS_000200.1	-	298698219	298698170
Sma-1-3724	NW_003038011.1	+	626845	626894
Sma-1-3725	NS_000200.1	+	228154495	228154544
Sma-1-3726	NW_003038011.1	+	742725	742774
Sma-1-3727	NW_003038011.1	+	756557	756606
Sma-1-3728	NS_000200.1	+	228270375	228270424
Sma-1-3729	NS_000200.1	+	228284207	228284256
Sma-1-3730	NW_003038012.1	+	359002	359051
Sma-1-3731	NS_000200.1	+	228682941	228682990
Sma-1-3732	NW_003038060.1	+	235113	235162
Sma-1-3733	NS_000200.1	+	260800238	260800287
Sma-1-3734	NW_003037908.1	-	134129	134080
Sma-1-3735	NS_000200.1	-	152607260	152607211
Sma-1-3736	NW_003026581.1	+	3895	3944
Sma-1-3737	NS_000200.1	+	302833883	302833932
Sma-1-3738	NW_003037795.1	+	1224497	1224546
Sma-1-3739	NS_000200.1	+	7615380	7615429
Sma-1-3740	NW_003037975.1	+	399058	399107
Sma-1-3741	NS_000200.1	+	205981282	205981331
Sma-1-3742	NW_003037933.1	-	34891	34842
Sma-1-3743	NS_000200.1	-	178448063	178448014
Sma-1-3744	NW_003037853.1	+	576509	576558
Sma-1-3745	NS_000200.1	+	96068560	96068609
Sma-1-3746	NW_003037909.1	+	746399	746448
Sma-1-3747	NS_000200.1	+	154288048	154288097
Sma-1-3748	NW_003038252.1	-	112510	112461
Sma-1-3749	NS_000200.1	-	318968098	318968049
Sma-1-3750	NW_003037944.1	+	232294	232343
Sma-1-3751	NS_000200.1	+	188453647	188453696
Sma-1-3752	NW_003022712.1	+	409	458

Sma-1-3753	NS_000200.1	+	367967712	367967761
Sma-1-3754	NW_003037890.1	+	2055227	2055276
Sma-1-3755	NS_000200.1	+	137738588	137738637
Sma-1-3756	NW_003038187.1	+	131066	131115
Sma-1-3757	NS_000200.1	+	302082924	302082973
Sma-1-3758	NW_003038147.1	+	225188	225237
Sma-1-3759	NS_000200.1	+	289752931	289752980
Sma-1-3760	NW_003037825.1	+	1403677	1403726
Sma-1-3761	NS_000200.1	+	60533459	60533508
Sma-1-3762	NW_003033971.1	-	1222	1173
Sma-1-3763	NS_000200.1	-	363328822	363328773
Sma-1-3764	NW_003037871.1	-	369200	369151
Sma-1-3765	NW_003038261.1	-	433733	433684
Sma-1-3766	NS_000200.1	-	324047384	324047335
Sma-1-3767	NS_000200.1	-	117816938	117816889
Sma-1-3768	NW_003037938.1	+	821070	821119
Sma-1-3769	NS_000200.1	+	182858183	182858232
Sma-1-3770	NW_003037975.1	+	1017632	1017681
Sma-1-3771	NS_000200.1	+	206599856	206599905
Sma-1-3772	NW_003026596.1	-	179800	179751
Sma-1-3773	NS_000200.1	-	305450428	305450379
Sma-1-3774	NW_003038187.1	+	25006	25055
Sma-1-3775	NS_000200.1	+	301976864	301976913
Sma-1-3776	NW_003038013.1	-	230535	230486
Sma-1-3777	NS_000200.1	-	229620161	229620112
Sma-1-3778	NW_003037937.1	+	806236	806285
Sma-1-3779	NW_003037937.1	-	806520	806471
Sma-1-3780	NS_000200.1	+	182006145	182006194
Sma-1-3781	NS_000200.1	-	182006429	182006380
Sma-1-3782	NW_003038064.1	+	154693	154742
Sma-1-3783	NS_000200.1	+	261991270	261991319
Sma-1-3784	NW_003038192.1	-	5329	5280
Sma-1-3785	NS_000200.1	-	302567236	302567187
Sma-1-3786	NW_003035836.1	-	455	406
Sma-1-3787	NS_000200.1	-	364192196	364192147
Sma-1-3788	NW_003037852.1	+	675528	675577
Sma-1-3789	NS_000200.1	+	95067423	95067472
Sma-1-3790	NW_003038070.1	+	444723	444772
Sma-1-3791	NS_000200.1	+	264781964	264782013
Sma-1-3792	NW_003026598.1	+	70867	70916
Sma-1-3793	NS_000200.1	+	305841083	305841132
Sma-1-3794	NW_003038014.1	+	12335	12384
Sma-1-3795	NS_000200.1	+	229753024	229753073
Sma-1-3796	NW_003037853.1	+	545778	545827
Sma-1-3797	NS_000200.1	+	96037829	96037878
Sma-1-3798	NW_003026599.1	-	6942	6893
Sma-1-3799	NS_000200.1	-	305885271	305885222
Sma-1-3800	NW_003037933.1	-	217477	217428
Sma-1-3801	NS_000200.1	-	178630649	178630600
Sma-1-3802	NW_003037915.1	+	55600	55649
Sma-1-3803	NS_000200.1	+	161955586	161955635
Sma-1-3804	NW_003037908.1	+	177361	177410

Sma-1-3805	NS_000200.1	+	152650492	152650541
Sma-1-3806	NW_003038202.1	+	22663	22712
Sma-1-3807	NS_000200.1	+	304280909	304280958
Sma-1-3808	NW_003038131.1	-	65917	65868
Sma-1-3809	NS_000200.1	-	282027761	282027712
Sma-1-3810	NW_003038206.1	+	35290	35339
Sma-1-3811	NS_000200.1	+	304862054	304862103
Sma-1-3812	NW_003037973.1	-	545213	545164
Sma-1-3813	NS_000200.1	-	204526940	204526891
Sma-1-3814	NW_003037890.1	+	1150402	1150451
Sma-1-3815	NS_000200.1	+	136833763	136833812
Sma-1-3816	NW_003037810.1	-	240284	240235
Sma-1-3817	NS_000200.1	-	36844806	36844757
Sma-1-3818	NW_003037798.1	-	1263053	1263004
Sma-1-3819	NS_000200.1	-	15522211	15522162
Sma-1-3820	NW_003037918.1	-	2205442	2205393
Sma-1-3821	NS_000200.1	-	168772432	168772383
Sma-1-3822	NW_003026608.1	+	18456	18505
Sma-1-3823	NS_000200.1	+	309296576	309296625
Sma-1-3824	NW_003037889.1	-	881546	881497
Sma-1-3825	NS_000200.1	-	134658036	134657987
Sma-1-3826	NW_003037795.1	+	1398605	1398654
Sma-1-3827	NS_000200.1	+	7789488	7789537
Sma-1-3828	NW_003037889.1	+	868468	868517
Sma-1-3829	NS_000200.1	+	134644958	134645007
Sma-1-3830	NW_003037936.1	+	392531	392580
Sma-1-3831	NS_000200.1	+	180832260	180832309
Sma-1-3832	NW_003038014.1	+	394169	394218
Sma-1-3833	NS_000200.1	+	230134858	230134907
Sma-1-3834	NW_003037827.1	+	120114	120163
Sma-1-3835	NS_000200.1	+	62742255	62742304
Sma-1-3836	NW_003038014.1	+	515287	515336
Sma-1-3837	NW_003038014.1	+	515792	515841
Sma-1-3838	NS_000200.1	+	230255976	230256025
Sma-1-3839	NS_000200.1	+	230256481	230256530
Sma-1-3840	NW_003037854.1	-	1092632	1092583
Sma-1-3841	NS_000200.1	-	97997053	97997004
Sma-1-3842	NW_003037972.1	-	814848	814799
Sma-1-3843	NS_000200.1	-	203290552	203290503
Sma-1-3844	NW_003038015.1	+	48598	48647
Sma-1-3845	NS_000200.1	+	230372088	230372137
Sma-1-3846	NW_003037908.1	+	316568	316617
Sma-1-3847	NS_000200.1	+	152789699	152789748
Sma-1-3848	NW_003038015.1	+	509442	509491
Sma-1-3849	NS_000200.1	+	230832932	230832981
Sma-1-3850	NW_003038116.1	+	46451	46500
Sma-1-3851	NW_003038116.1	-	38233	38184
Sma-1-3852	NS_000200.1	+	277261729	277261778
Sma-1-3853	NS_000200.1	-	277253511	277253462
Sma-1-3854	NW_003026609.1	-	96983	96934
Sma-1-3855	NS_000200.1	-	309751975	309751926
Sma-1-3856	NW_003037889.1	+	647483	647532

Sma-1-3857	NS_000200.1	+	134423973	134424022
Sma-1-3858	NW_003037972.1	-	494873	494824
Sma-1-3859	NS_000200.1	-	202970577	202970528
Sma-1-3860	NW_003037797.1	-	1663278	1663229
Sma-1-3861	NS_000200.1	-	12091138	12091089
Sma-1-3862	NW_003038239.1	+	9500	9549
Sma-1-3863	NS_000200.1	+	313850282	313850331
Sma-1-3864	NW_003036007.1	+	1280	1329
Sma-1-3865	NS_000200.1	+	343250489	343250538
Sma-1-3866	NW_003037915.1	-	66544	66495
Sma-1-3867	NS_000200.1	-	161966530	161966481
Sma-1-3868	NW_003037972.1	+	485157	485206
Sma-1-3869	NS_000200.1	+	202960861	202960910
Sma-1-3870	NW_003037826.1	+	631933	631982
Sma-1-3871	NS_000200.1	+	61631449	61631498
Sma-1-3872	NW_003037908.1	+	911430	911479
Sma-1-3873	NS_000200.1	+	153384561	153384610
Sma-1-3874	NW_003038161.1	+	120603	120652
Sma-1-3875	NS_000200.1	+	297490454	297490503
Sma-1-3876	NW_003037889.1	-	1857781	1857732
Sma-1-3877	NS_000200.1	-	135634271	135634222
Sma-1-3878	NW_003026611.1	-	215167	215118
Sma-1-3879	NS_000200.1	-	310784951	310784902
Sma-1-3880	NW_003038068.1	-	192280	192231
Sma-1-3881	NS_000200.1	-	263259488	263259439
Sma-1-3882	NW_003037826.1	+	403308	403357
Sma-1-3883	NS_000200.1	+	61402824	61402873
Sma-1-3884	NW_003038018.1	-	637506	637457
Sma-1-3885	NS_000200.1	-	233038356	233038307
Sma-1-3886	NW_003026612.1	+	175203	175252
Sma-1-3887	NS_000200.1	+	312480590	312480639
Sma-1-3888	NW_003037936.1	-	260205	260156
Sma-1-3889	NS_000200.1	-	180699934	180699885
Sma-1-3890	NW_003037935.1	-	913758	913709
Sma-1-3891	NS_000200.1	-	180330293	180330244
Sma-1-3892	NW_003038254.1	-	341736	341687
Sma-1-3893	NS_000200.1	-	320377294	320377245
Sma-1-3894	NW_003037797.1	+	1458420	1458469
Sma-1-3895	NS_000200.1	+	11886280	11886329
Sma-1-3896	NW_003038068.1	+	248399	248448
Sma-1-3897	NS_000200.1	+	263315607	263315656
Sma-1-3898	NW_003037826.1	-	1798	1749
Sma-1-3899	NS_000200.1	-	61001314	61001265
Sma-1-3900	NW_003037826.1	+	173991	174040
Sma-1-3901	NS_000200.1	+	61173507	61173556
Sma-1-3902	NW_003038221.1	-	70795	70746
Sma-1-3903	NS_000200.1	-	308712059	308712010
Sma-1-3904	NW_003037854.1	-	232146	232097
Sma-1-3905	NS_000200.1	-	97136567	97136518
Sma-1-3906	NW_003038230.1	+	27567	27616
Sma-1-3907	NS_000200.1	+	311313138	311313187
Sma-1-3908	NW_003026465.1	-	500046	499997

Sma-1-3909	NS_000200.1	-	207892640	207892591
Sma-1-3910	NW_003038019.1	-	276325	276276
Sma-1-3911	NS_000200.1	-	233593143	233593094
Sma-1-3912	NW_003038019.1	+	350694	350743
Sma-1-3913	NS_000200.1	+	233667512	233667561
Sma-1-3914	NW_003037972.1	-	181465	181416
Sma-1-3915	NS_000200.1	-	202657169	202657120
Sma-1-3916	NW_003038068.1	+	48796	48845
Sma-1-3917	NS_000200.1	+	263116004	263116053
Sma-1-3918	NW_003037908.1	-	980531	980482
Sma-1-3919	NS_000200.1	-	153453662	153453613
Sma-1-3920	NW_003037810.1	-	1360322	1360273
Sma-1-3921	NS_000200.1	-	37964844	37964795
Sma-1-3922	NW_003038069.1	-	348446	348397
Sma-1-3923	NS_000200.1	-	264191852	264191803
Sma-1-3924	NW_003037803.1	+	63413	63462
Sma-1-3925	NS_000200.1	+	23563950	23563999
Sma-1-3926	NW_003037795.1	+	1592121	1592170
Sma-1-3927	NS_000200.1	+	7983004	7983053
Sma-1-3928	NW_003038020.1	+	828261	828310
Sma-1-3929	NS_000200.1	+	234795605	234795654
Sma-1-3930	NW_003037855.1	-	262736	262687
Sma-1-3931	NS_000200.1	-	99127486	99127437
Sma-1-3932	NW_003037812.1	-	981912	981863
Sma-1-3933	NS_000200.1	-	40464651	40464602
Sma-1-3934	NW_003037793.1	-	525269	525220
Sma-1-3935	NS_000200.1	-	525269	525220
Sma-1-3936	NW_003037831.1	-	1277500	1277451
Sma-1-3937	NS_000200.1	-	70726802	70726753
Sma-1-3938	NW_003037909.1	-	128463	128414
Sma-1-3939	NS_000200.1	-	153670112	153670063
Sma-1-3940	NW_003037797.1	-	1623052	1623003
Sma-1-3941	NS_000200.1	-	12050912	12050863
Sma-1-3942	NW_003026618.1	-	408151	408102
Sma-1-3943	NS_000200.1	-	322163153	322163104
Sma-1-3944	NW_003037804.1	+	506733	506782
Sma-1-3945	NS_000200.1	+	25646753	25646802
Sma-1-3946	NW_003038228.1	-	102767	102718
Sma-1-3947	NS_000200.1	-	310976047	310975998
Sma-1-3948	NW_003037797.1	+	1623269	1623318
Sma-1-3949	NS_000200.1	+	12051129	12051178
Sma-1-3950	NW_003038022.1	+	105272	105321
Sma-1-3951	NS_000200.1	+	235786114	235786163
Sma-1-3952	NW_003038022.1	-	133967	133918
Sma-1-3953	NS_000200.1	-	235814809	235814760
Sma-1-3954	NW_003037810.1	+	1335095	1335144
Sma-1-3955	NS_000200.1	+	37939617	37939666
Sma-1-3956	NW_003037970.1	+	349493	349543
Sma-1-3957	NS_000200.1	+	201405683	201405733
Sma-1-3958	NW_003026619.1	-	193808	193759
Sma-1-3959	NS_000200.1	-	322384552	322384503
Sma-1-3960	NW_003037970.1	+	313929	313978

Sma-1-3961	NS_000200.1	+	201370119	201370168
Sma-1-3962	NW_003038022.1	-	186954	186905
Sma-1-3963	NS_000200.1	-	235867796	235867747
Sma-1-3964	NW_003037970.1	+	310993	311042
Sma-1-3965	NS_000200.1	+	201367183	201367232
Sma-1-3966	NW_003037856.1	-	648523	648474
Sma-1-3967	NS_000200.1	-	100006479	100006430
Sma-1-3968	NW_003026619.1	-	96830	96781
Sma-1-3969	NS_000200.1	-	322287574	322287525
Sma-1-3970	NW_003039127.1	+	2118515	2118564
Sma-1-3971	NS_000200.1	+	115196014	115196063
Sma-1-3972	NW_003037813.1	+	800088	800137
Sma-1-3973	NS_000200.1	+	41786951	41787000
Sma-1-3974	NW_003038020.1	-	152304	152255
Sma-1-3975	NS_000200.1	-	234119648	234119599
Sma-1-3976	NW_003037825.1	-	742758	742709
Sma-1-3977	NS_000200.1	-	59872540	59872491
Sma-1-3978	NW_003037812.1	-	962489	962440
Sma-1-3979	NS_000200.1	-	40445228	40445179
Sma-1-3980	NW_003026642.1	+	506	555
Sma-1-3981	NS_000200.1	+	325066576	325066625
Sma-1-3982	NW_003026650.1	+	1128	1177
Sma-1-3983	NS_000200.1	+	325122560	325122609
Sma-1-3984	NW_003026653.1	-	2227	2178
Sma-1-3985	NW_003037901.1	-	296649	296600
Sma-1-3986	NS_000200.1	-	325140676	325140627
Sma-1-3987	NS_000200.1	-	146145110	146145061
Sma-1-3988	NW_003026714.1	+	1280	1329
Sma-1-3989	NS_000200.1	+	325375075	325375124
Sma-1-3990	NW_003036900.1	-	462	413
Sma-1-3991	NW_003038067.1	-	134619	134570
Sma-1-3992	NS_000200.1	-	347935711	347935662
Sma-1-3993	NS_000200.1	-	262988672	262988623
Sma-1-3994	NW_003037890.1	+	1813721	1813770
Sma-1-3995	NS_000200.1	+	137497082	137497131
Sma-1-3996	NW_003037805.1	+	1566299	1566348
Sma-1-3997	NS_000200.1	+	28124143	28124192
Sma-1-3998	NW_003037858.1	+	1113051	1113100
Sma-1-3999	NS_000200.1	+	101777327	101777376
Sma-1-4000	NW_003038081.1	+	330513	330562
Sma-1-4001	NS_000200.1	+	269492556	269492605
Sma-1-4002	NW_003037793.1	+	2886026	2886075
Sma-1-4003	NS_000200.1	+	2886026	2886075
Sma-1-4004	NW_003038037.1	+	76344	76393
Sma-1-4005	NS_000200.1	+	250231753	250231802
Sma-1-4006	NW_003038039.1	-	40121	40072
Sma-1-4007	NS_000200.1	-	253477967	253477918
Sma-1-4008	NW_003037825.1	-	552559	552510
Sma-1-4009	NS_000200.1	-	59682341	59682292
Sma-1-4010	NW_003037858.1	-	1125345	1125296
Sma-1-4011	NS_000200.1	-	101789621	101789572
Sma-1-4012	NW_003037970.1	-	143195	143146

Sma-1-4013	NS_000200.1	-	201199385	201199336
Sma-1-4014	NW_003037797.1	+	2130467	2130516
Sma-1-4015	NS_000200.1	+	12558327	12558376
Sma-1-4016	NW_003027532.1	+	747	796
Sma-1-4017	NS_000200.1	+	361560584	361560633
Sma-1-4018	NW_003037891.1	+	323534	323583
Sma-1-4019	NS_000200.1	+	138204812	138204861
Sma-1-4020	NW_003037856.1	+	437836	437885
Sma-1-4021	NS_000200.1	+	99795792	99795841
Sma-1-4022	NW_003037969.1	+	429080	429129
Sma-1-4023	NS_000200.1	+	201035207	201035256
Sma-1-4024	NW_003037825.1	+	427990	428039
Sma-1-4025	NS_000200.1	+	59557772	59557821
Sma-1-4026	NW_003027591.1	+	1221	1270
Sma-1-4027	NS_000200.1	+	361689404	361689453
Sma-1-4028	NW_003038025.1	-	194051	194002
Sma-1-4029	NS_000200.1	-	239052296	239052247
Sma-1-4030	NW_003021738.1	-	849	800
Sma-1-4031	NW_003026464.1	-	634433	634384
Sma-1-4032	NS_000200.1	-	351774973	351774924
Sma-1-4033	NS_000200.1	-	203934742	203934693
Sma-1-4034	NW_003027748.1	+	898	947
Sma-1-4035	NS_000200.1	+	365874344	365874393
Sma-1-4036	NW_003027772.1	-	218	169
Sma-1-4037	NW_003037970.1	-	179121	179072
Sma-1-4038	NS_000200.1	-	365910468	365910419
Sma-1-4039	NS_000200.1	-	201235311	201235262
Sma-1-4040	NW_003038232.1	-	137069	137020
Sma-1-4041	NS_000200.1	-	312100860	312100811
Sma-1-4042	NW_003037863.1	+	1069940	1069989
Sma-1-4043	NW_003037899.1	-	387736	387687
Sma-1-4044	NS_000200.1	+	107828307	107828356
Sma-1-4045	NS_000200.1	-	144906847	144906798
Sma-1-4046	NW_003037981.1	-	874769	874720
Sma-1-4047	NW_003038030.1	+	983020	983069
Sma-1-4048	NS_000200.1	+	244118887	244118936
Sma-1-4049	NS_000200.1	-	213272637	213272588
Sma-1-4050	NW_003027904.1	-	975	926
Sma-1-4051	NS_000200.1	-	366128962	366128913
Sma-1-4052	NW_003029016.1	+	1615	1664
Sma-1-4053	NS_000200.1	+	375169912	375169961
Sma-1-4054	NW_003037795.1	-	211733	211684
Sma-1-4055	NS_000200.1	-	6602616	6602567
Sma-1-4056	NW_003037838.1	+	718880	718929
Sma-1-4057	NS_000200.1	+	78717443	78717492
Sma-1-4058	NW_003037969.1	+	189595	189644
Sma-1-4059	NS_000200.1	+	200795722	200795771
Sma-1-4060	NW_003038080.1	+	408960	409009
Sma-1-4061	NS_000200.1	+	268861570	268861619
Sma-1-4062	NW_003037943.1	-	502501	502452
Sma-1-4063	NS_000200.1	-	187524034	187523985
Sma-1-4064	NW_003038260.1	+	292145	292194

Sma-1-4065	NS_000200.1	+	323463920	323463969
Sma-1-4066	NW_003038023.1	+	372278	372327
Sma-1-4067	NS_000200.1	+	237532569	237532618
Sma-1-4068	NW_003028065.1	-	77	28
Sma-1-4069	NS_000200.1	-	369275520	369275471
Sma-1-4070	NW_003038140.1	+	312662	312711
Sma-1-4071	NS_000200.1	+	286840539	286840588
Sma-1-4072	NW_003028216.1	+	7148	7197
Sma-1-4073	NW_003037941.1	+	271231	271280
Sma-1-4074	NS_000200.1	+	185005705	185005754
Sma-1-4075	NS_000200.1	+	374310412	374310461
Sma-1-4076	NW_003038071.1	-	34816	34767
Sma-1-4077	NS_000200.1	-	264837859	264837810
Sma-1-4078	NW_003037859.1	+	147151	147200
Sma-1-4079	NS_000200.1	+	102825992	102826041
Sma-1-4080	NW_003037797.1	-	2081493	2081444
Sma-1-4081	NS_000200.1	-	12509353	12509304
Sma-1-4082	NW_003039127.1	+	2221975	2222024
Sma-1-4083	NS_000200.1	+	115299474	115299523
Sma-1-4084	NW_003038237.1	-	4820	4771
Sma-1-4085	NS_000200.1	-	313444739	313444690
Sma-1-4086	NW_003037825.1	-	1272497	1272448
Sma-1-4087	NS_000200.1	-	60402279	60402230
Sma-1-4088	NW_003037825.1	-	1215020	1214971
Sma-1-4089	NS_000200.1	-	60344802	60344753
Sma-1-4090	NW_003037967.1	-	233369	233320
Sma-1-4091	NS_000200.1	-	199473340	199473291
Sma-1-4092	NW_003038239.1	-	12142	12093
Sma-1-4093	NS_000200.1	-	313852924	313852875
Sma-1-4094	NW_003037793.1	-	130173	130124
Sma-1-4095	NS_000200.1	-	130173	130124
Sma-1-4096	NW_003038010.1	-	162649	162600
Sma-1-4097	NS_000200.1	-	226980872	226980823
Sma-1-4098	NW_003037967.1	-	206057	206008
Sma-1-4099	NS_000200.1	-	199446028	199445979
Sma-1-4100	NW_003037793.1	-	724516	724467
Sma-1-4101	NS_000200.1	-	724516	724467
Sma-1-4102	NW_003038023.1	-	94103	94054
Sma-1-4103	NS_000200.1	-	237254394	237254345
Sma-1-4104	NW_003035869.1	+	128441	128490
Sma-1-4105	NS_000200.1	+	335312641	335312690
Sma-1-4106	NW_003038024.1	-	39544	39495
Sma-1-4107	NS_000200.1	-	237936573	237936524
Sma-1-4108	NW_003028876.1	-	610	561
Sma-1-4109	NS_000200.1	-	370133472	370133423
Sma-1-4110	NW_003028888.1	-	99	50
Sma-1-4111	NS_000200.1	-	370162913	370162864
Sma-1-4112	NW_003037915.1	+	1094220	1094269
Sma-1-4113	NS_000200.1	+	162994206	162994255
Sma-1-4114	NW_003037909.1	-	369994	369945
Sma-1-4115	NS_000200.1	-	153911643	153911594
Sma-1-4116	NW_003037881.1	-	453586	453537

Sma-1-4117	NS_000200.1	-	124885377	124885328
Sma-1-4118	NW_003038162.1	-	246099	246050
Sma-1-4119	NS_000200.1	-	298191554	298191505
Sma-1-4120	NW_003039097.1	-	1462	1413
Sma-1-4121	NS_000200.1	-	360501759	360501710
Sma-1-4122	NW_003037965.1	-	311446	311397
Sma-1-4123	NS_000200.1	-	198600531	198600482
Sma-1-4124	NW_003037859.1	-	615359	615310
Sma-1-4125	NS_000200.1	-	103294200	103294151
Sma-1-4126	NW_003037824.1	-	959010	958961
Sma-1-4127	NS_000200.1	-	57889950	57889901
Sma-1-4128	NW_003037888.1	-	1401379	1401330
Sma-1-4129	NS_000200.1	-	133609871	133609822
Sma-1-4130	NW_003038012.1	-	48081	48032
Sma-1-4131	NS_000200.1	-	228372020	228371971
Sma-1-4132	NW_003038124.1	+	46578	46627
Sma-1-4133	NS_000200.1	+	279039222	279039271
Sma-1-4134	NW_003037859.1	-	757963	757914
Sma-1-4135	NS_000200.1	-	103436804	103436755
Sma-1-4136	NW_003037964.1	+	78168	78217
Sma-1-4137	NS_000200.1	+	197679690	197679739
Sma-1-4138	NW_003038025.1	+	102415	102464
Sma-1-4139	NS_000200.1	+	238960660	238960709
Sma-1-4140	NW_003037824.1	-	946059	946010
Sma-1-4141	NS_000200.1	-	57876999	57876950
Sma-1-4142	NW_003029320.1	-	1849	1800
Sma-1-4143	NS_000200.1	-	357283963	357283914
Sma-1-4144	NW_003038073.1	+	288372	288421
Sma-1-4145	NS_000200.1	+	265810805	265810854
Sma-1-4146	NW_003037859.1	-	805579	805530
Sma-1-4147	NS_000200.1	-	103484420	103484371
Sma-1-4148	NW_003037881.1	+	490349	490398
Sma-1-4149	NS_000200.1	+	124922140	124922189
Sma-1-4150	NW_003038080.1	-	206423	206374
Sma-1-4151	NS_000200.1	-	268659033	268658984
Sma-1-4152	NW_003037822.1	-	26391	26342
Sma-1-4153	NS_000200.1	-	54334397	54334348
Sma-1-4154	NW_003037887.1	-	1245831	1245782
Sma-1-4155	NS_000200.1	-	131709809	131709760
Sma-1-4156	NW_003038034.1	+	523676	523725
Sma-1-4157	NS_000200.1	+	247465432	247465481
Sma-1-4158	NW_003037870.1	-	517111	517062
Sma-1-4159	NS_000200.1	-	117390776	117390727
Sma-1-4160	NW_003038025.1	+	539326	539375
Sma-1-4161	NS_000200.1	+	239397571	239397620
Sma-1-4162	NW_003038242.1	+	53021	53070
Sma-1-4163	NS_000200.1	+	314752167	314752216
Sma-1-4164	NW_003037795.1	-	320882	320833
Sma-1-4165	NS_000200.1	-	6711765	6711716
Sma-1-4166	NW_003037795.1	+	344661	344710
Sma-1-4167	NS_000200.1	+	6735544	6735593
Sma-1-4168	NW_003037793.1	-	3075847	3075798

Sma-1-4169	NS_000200.1	-	3075847	3075798
Sma-1-4170	NW_003038145.1	-	360563	360514
Sma-1-4171	NS_000200.1	-	288971986	288971937
Sma-1-4172	NW_003037805.1	-	2936	2887
Sma-1-4173	NS_000200.1	-	26560780	26560731
Sma-1-4174	NW_003037824.1	-	764739	764690
Sma-1-4175	NS_000200.1	-	57695679	57695630
Sma-1-4176	NW_003037964.1	+	26363	26412
Sma-1-4177	NS_000200.1	+	197627885	197627934
Sma-1-4178	NW_003036817.1	-	1006	957
Sma-1-4179	NS_000200.1	-	347708605	347708556
Sma-1-4180	NW_003038074.1	+	115440	115489
Sma-1-4181	NS_000200.1	+	266006820	266006869
Sma-1-4182	NW_003037824.1	+	642006	642055
Sma-1-4183	NS_000200.1	+	57572946	57572995
Sma-1-4184	NW_003037963.1	+	46379	46428
Sma-1-4185	NS_000200.1	+	197324591	197324640
Sma-1-4186	NW_003038145.1	+	94986	95035
Sma-1-4187	NS_000200.1	+	288706409	288706458
Sma-1-4188	NW_003037962.1	+	177725	177774
Sma-1-4189	NS_000200.1	+	196254568	196254617
Sma-1-4190	NW_003035193.1	-	1225	1176
Sma-1-4191	NS_000200.1	-	370780208	370780159
Sma-1-4192	NW_003038248.1	-	16668	16619
Sma-1-4193	NS_000200.1	-	316327935	316327886
Sma-1-4194	NW_003038077.1	-	158106	158057
Sma-1-4195	NS_000200.1	-	266929746	266929697
Sma-1-4196	NW_003037824.1	+	416305	416354
Sma-1-4197	NS_000200.1	+	57347245	57347294
Sma-1-4198	NW_003038255.1	-	274174	274125
Sma-1-4199	NS_000200.1	-	320663319	320663270
Sma-1-4200	NW_003037935.1	-	615382	615333
Sma-1-4201	NS_000200.1	-	180031917	180031868
Sma-1-4202	NW_003037938.1	+	652666	652715
Sma-1-4203	NS_000200.1	+	182689779	182689828
Sma-1-4204	NW_003037824.1	-	409258	409209
Sma-1-4205	NS_000200.1	-	57340198	57340149
Sma-1-4206	NW_003038077.1	-	191338	191289
Sma-1-4207	NS_000200.1	-	266962978	266962929
Sma-1-4208	NW_003037824.1	-	352531	352482
Sma-1-4209	NS_000200.1	-	57283471	57283422
Sma-1-4210	NW_003030353.1	-	1608	1559
Sma-1-4211	NW_003026608.1	-	115158	115109
Sma-1-4212	NS_000200.1	-	362749052	362749003
Sma-1-4213	NS_000200.1	-	309393278	309393229
Sma-1-4214	NW_003038027.1	+	39279	39328
Sma-1-4215	NS_000200.1	+	240409993	240410042
Sma-1-4216	NW_003037797.1	+	2766852	2766901
Sma-1-4217	NS_000200.1	+	13194712	13194761
Sma-1-4218	NW_003037909.1	+	614822	614871
Sma-1-4219	NS_000200.1	+	154156471	154156520
Sma-1-4220	NW_003037909.1	+	692146	692195

Sma-1-4221	NS_000200.1	+	154233795	154233844
Sma-1-4222	NW_003037962.1	+	456989	457038
Sma-1-4223	NS_000200.1	+	196533832	196533881
Sma-1-4224	NW_003037795.1	+	515547	515596
Sma-1-4225	NS_000200.1	+	6906430	6906479
Sma-1-4226	NW_003038028.1	+	145818	145867
Sma-1-4227	NS_000200.1	+	241453392	241453441
Sma-1-4228	NW_003037915.1	-	770452	770403
Sma-1-4229	NS_000200.1	-	162670438	162670389
Sma-1-4230	NW_003037932.1	-	646517	646468
Sma-1-4231	NS_000200.1	-	178179713	178179664
Sma-1-4232	NW_003038249.1	+	69766	69815
Sma-1-4233	NS_000200.1	+	316752019	316752068
Sma-1-4234	NW_003038145.1	+	97230	97279
Sma-1-4235	NS_000200.1	+	288708653	288708702
Sma-1-4236	NW_003037804.1	-	1333976	1333927
Sma-1-4237	NS_000200.1	-	26473996	26473947
Sma-1-4238	NW_003037861.1	+	322320	322369
Sma-1-4239	NS_000200.1	+	105098774	105098823
Sma-1-4240	NW_003037823.1	+	64296	64345
Sma-1-4241	NS_000200.1	+	55980915	55980964
Sma-1-4242	NW_003037795.1	+	583158	583207
Sma-1-4243	NS_000200.1	+	6974041	6974090
Sma-1-4244	NW_003037918.1	+	127703	127752
Sma-1-4245	NS_000200.1	+	166694693	166694742
Sma-1-4246	NW_003037932.1	+	349205	349254
Sma-1-4247	NW_003037932.1	+	350342	350391
Sma-1-4248	NS_000200.1	+	177882401	177882450
Sma-1-4249	NS_000200.1	+	177883538	177883587
Sma-1-4250	NW_003037887.1	-	630257	630208
Sma-1-4251	NS_000200.1	-	131094235	131094186
Sma-1-4252	NW_003037861.1	+	645142	645191
Sma-1-4253	NS_000200.1	+	105421596	105421645
Sma-1-4254	NW_003037918.1	+	1351831	1351880
Sma-1-4255	NS_000200.1	+	167918821	167918870
Sma-1-4256	NW_003037937.1	-	555048	554999
Sma-1-4257	NS_000200.1	-	181754957	181754908
Sma-1-4258	NW_003037823.1	+	123679	123728
Sma-1-4259	NS_000200.1	+	56040298	56040347
Sma-1-4260	NW_003038079.1	-	19909	19860
Sma-1-4261	NS_000200.1	-	268149897	268149848
Sma-1-4262	NW_003038028.1	-	595	546
Sma-1-4263	NS_000200.1	-	241308169	241308120
Sma-1-4264	NW_003037862.1	+	273120	273169
Sma-1-4265	NS_000200.1	+	105924781	105924830
Sma-1-4266	NW_003038028.1	-	714227	714178
Sma-1-4267	NS_000200.1	-	242021801	242021752
Sma-1-4268	NW_003038250.1	+	136849	136898
Sma-1-4269	NS_000200.1	+	318189526	318189575
Sma-1-4270	NW_003037797.1	+	2972556	2972605
Sma-1-4271	NS_000200.1	+	13400416	13400465
Sma-1-4272	NW_003037915.1	+	1009929	1009978

Sma-1-4273	NS_000200.1	+	162909915	162909964
Sma-1-4274	NW_003037810.1	+	1016162	1016211
Sma-1-4275	NS_000200.1	+	37620684	37620733
Sma-1-4276	NW_003037909.1	-	886257	886206
Sma-1-4277	NS_000200.1	-	154427906	154427855
Sma-1-4278	NW_003037962.1	+	115324	115373
Sma-1-4279	NS_000200.1	+	196192167	196192216
Sma-1-4280	NW_003037915.1	-	931725	931676
Sma-1-4281	NS_000200.1	-	162831711	162831662
Sma-1-4282	NW_003037961.1	-	450506	450457
Sma-1-4283	NS_000200.1	-	196074748	196074699
Sma-1-4284	NW_003038251.1	+	147685	147734
Sma-1-4285	NS_000200.1	+	318479457	318479506
Sma-1-4286	NW_003038251.1	+	258313	258362
Sma-1-4287	NS_000200.1	+	318590085	318590134
Sma-1-4288	NW_003037863.1	+	1199496	1199545
Sma-1-4289	NS_000200.1	+	107957863	107957912
Sma-1-4290	NW_003038080.1	-	136324	136275
Sma-1-4291	NS_000200.1	-	268588934	268588885
Sma-1-4292	NW_003037940.1	-	112461	112412
Sma-1-4293	NS_000200.1	-	183854591	183854542
Sma-1-4294	NW_003038249.1	+	211504	211553
Sma-1-4295	NS_000200.1	+	316893757	316893806
Sma-1-4296	NW_003032082.1	-	6260	6210
Sma-1-4297	NS_000200.1	-	377497873	377497823
Sma-1-4298	NW_003038261.1	-	554317	554268
Sma-1-4299	NS_000200.1	-	324167968	324167919
Sma-1-4300	NW_003035858.1	-	1811	1762
Sma-1-4301	NS_000200.1	-	364241715	364241666
Sma-1-4302	NW_003037961.1	-	260415	260366
Sma-1-4303	NS_000200.1	-	195884657	195884608
Sma-1-4304	NW_003037794.1	-	1682152	1682103
Sma-1-4305	NS_000200.1	-	5861747	5861698
Sma-1-4306	NW_003037809.1	+	612708	612757
Sma-1-4307	NS_000200.1	+	34277172	34277221
Sma-1-4308	NW_003038033.1	-	410885	410836
Sma-1-4309	NS_000200.1	-	246390887	246390838
Sma-1-4310	NW_003037814.1	-	112584	112535
Sma-1-4311	NS_000200.1	-	43096444	43096395
Sma-1-4312	NW_003032160.1	-	160474	160425
Sma-1-4313	NS_000200.1	-	381168560	381168511
Sma-1-4314	NW_003032160.1	-	195201	195152
Sma-1-4315	NS_000200.1	-	381203287	381203238
Sma-1-4316	NW_003037916.1	-	1024275	1024226
Sma-1-4317	NS_000200.1	-	164217686	164217637
Sma-1-4318	NW_003037863.1	-	730206	730157
Sma-1-4319	NS_000200.1	-	107488573	107488524
Sma-1-4320	NW_003037809.1	-	622007	621958
Sma-1-4321	NW_003037964.1	+	182812	182861
Sma-1-4322	NS_000200.1	+	197784334	197784383
Sma-1-4323	NS_000200.1	-	34286471	34286422
Sma-1-4324	NW_003038083.1	-	226704	226655

Sma-1-4325	NS_000200.1	-	270470041	270469992
Sma-1-4326	NW_003037797.1	+	3204257	3204306
Sma-1-4327	NS_000200.1	+	13632117	13632166
Sma-1-4328	NW_003037886.1	+	857238	857287
Sma-1-4329	NS_000200.1	+	130112805	130112854
Sma-1-4330	NW_003037822.1	-	36145	36096
Sma-1-4331	NS_000200.1	-	54344151	54344102
Sma-1-4332	NW_003037956.1	-	33678	33629
Sma-1-4333	NS_000200.1	-	194090133	194090084
Sma-1-4334	NW_003037886.1	+	774583	774632
Sma-1-4335	NS_000200.1	+	130030150	130030199
Sma-1-4336	NW_003037822.1	+	323127	323176
Sma-1-4337	NS_000200.1	+	54631133	54631182
Sma-1-4338	NW_003037819.1	+	1062757	1062806
Sma-1-4339	NS_000200.1	+	52151022	52151071
Sma-1-4340	NW_003037793.1	+	3204295	3204344
Sma-1-4341	NS_000200.1	+	3204295	3204344
Sma-1-4342	NW_003037864.1	-	1465255	1465206
Sma-1-4343	NS_000200.1	-	109957829	109957780
Sma-1-4344	NW_003037917.1	-	1458580	1458531
Sma-1-4345	NS_000200.1	-	166495384	166495335
Sma-1-4346	NW_003038253.1	+	187046	187095
Sma-1-4347	NW_003038029.1	+	813117	813166
Sma-1-4348	NS_000200.1	+	243014018	243014067
Sma-1-4349	NS_000200.1	+	319504843	319504892
Sma-1-4350	NW_003038083.1	+	11786	11835
Sma-1-4351	NS_000200.1	+	270255123	270255172
Sma-1-4352	NW_003038037.1	-	1731231	1731182
Sma-1-4353	NS_000200.1	-	251886640	251886591
Sma-1-4354	NW_003038032.1	-	122187	122138
Sma-1-4355	NS_000200.1	-	245281347	245281298
Sma-1-4356	NW_003038032.1	-	202866	202817
Sma-1-4357	NS_000200.1	-	245362026	245361977
Sma-1-4358	NW_003037955.1	-	176006	175957
Sma-1-4359	NS_000200.1	-	193802740	193802691
Sma-1-4360	NW_003038373.1	+	3606	3655
Sma-1-4361	NS_000200.1	+	327324477	327324526
Sma-1-4362	NW_003038053.1	+	436671	436720
Sma-1-4363	NS_000200.1	+	257796766	257796815
Sma-1-4364	NW_003038032.1	+	257222	257271
Sma-1-4365	NS_000200.1	+	245416382	245416431
Sma-1-4366	NW_003037864.1	-	23303	23254
Sma-1-4367	NS_000200.1	-	108515877	108515828
Sma-1-4368	NW_003037955.1	+	166482	166531
Sma-1-4369	NS_000200.1	+	193793216	193793265
Sma-1-4370	NW_003037822.1	+	1504367	1504416
Sma-1-4371	NS_000200.1	+	55812373	55812422
Sma-1-4372	NW_003037954.1	-	35154	35105
Sma-1-4373	NS_000200.1	-	193264776	193264727
Sma-1-4374	NW_003037864.1	-	58176	58127
Sma-1-4375	NS_000200.1	-	108550750	108550701
Sma-1-4376	NW_003037886.1	-	702575	702526

Sma-1-4377	NS_000200.1	-	129958142	129958093
Sma-1-4378	NW_003037953.1	+	203679	203728
Sma-1-4379	NS_000200.1	+	193116730	193116779
Sma-1-4380	NW_003033762.1	+	1483	1532
Sma-1-4381	NW_003038129.1	-	41332	41283
Sma-1-4382	NS_000200.1	+	358878123	358878172
Sma-1-4383	NS_000200.1	-	281294679	281294630
Sma-1-4384	NW_003037865.1	-	1039968	1039919
Sma-1-4385	NS_000200.1	-	111461887	111461838
Sma-1-4386	NW_003037935.1	+	269426	269475
Sma-1-4387	NS_000200.1	+	179685961	179686010
Sma-1-4388	NW_003038256.1	-	279505	279456
Sma-1-4389	NS_000200.1	-	321013331	321013282
Sma-1-4390	NW_003038257.1	+	188450	188499
Sma-1-4391	NW_003038257.1	-	183427	183378
Sma-1-4392	NS_000200.1	+	321362778	321362827
Sma-1-4393	NS_000200.1	-	321357755	321357706
Sma-1-4394	NW_003038257.1	+	228364	228413
Sma-1-4395	NS_000200.1	+	321402692	321402741
Sma-1-4396	NW_003038032.1	-	687155	687106
Sma-1-4397	NS_000200.1	-	245846315	245846266
Sma-1-4398	NW_003037937.1	-	176830	176781
Sma-1-4399	NS_000200.1	-	181376739	181376690
Sma-1-4400	NW_003037865.1	-	466011	465962
Sma-1-4401	NS_000200.1	-	110887930	110887881
Sma-1-4402	NW_003038083.1	-	638268	638219
Sma-1-4403	NS_000200.1	-	270881605	270881556
Sma-1-4404	NW_003034024.1	-	984	935
Sma-1-4405	NS_000200.1	-	363496873	363496824
Sma-1-4406	NW_003037865.1	+	51009	51058
Sma-1-4407	NS_000200.1	+	110472928	110472977
Sma-1-4408	NW_003037796.1	+	1072980	1073029
Sma-1-4409	NS_000200.1	+	9282624	9282673
Sma-1-4410	NW_003037916.1	+	1311791	1311840
Sma-1-4411	NS_000200.1	+	164505202	164505251
Sma-1-4412	NW_003037821.1	-	862468	862419
Sma-1-4413	NS_000200.1	-	54006952	54006903
Sma-1-4414	NW_003037886.1	+	1128642	1128691
Sma-1-4415	NS_000200.1	+	130384209	130384258
Sma-1-4416	NW_003037821.1	-	633329	633280
Sma-1-4417	NS_000200.1	-	53777813	53777764
Sma-1-4418	NW_003038033.1	+	238645	238694
Sma-1-4419	NS_000200.1	+	246218647	246218696
Sma-1-4420	NW_003037809.1	-	652669	652620
Sma-1-4421	NS_000200.1	-	34317133	34317084
Sma-1-4422	NW_003034553.1	+	6037	6086
Sma-1-4423	NS_000200.1	+	327186344	327186393
Sma-1-4424	NW_003037814.1	-	320796	320747
Sma-1-4425	NS_000200.1	-	43304656	43304607
Sma-1-4426	NW_003037809.1	+	2939887	2939936
Sma-1-4427	NS_000200.1	+	36604351	36604400
Sma-1-4428	NW_003037911.1	+	209351	209400

Sma-1-4429	NW_003037911.1	-	209560	209511
Sma-1-4430	NS_000200.1	+	155996704	155996753
Sma-1-4431	NS_000200.1	-	155996913	155996864
Sma-1-4432	NW_003034567.1	+	897	946
Sma-1-4433	NS_000200.1	+	327641791	327641840
Sma-1-4434	NW_003038033.1	+	322001	322050
Sma-1-4435	NS_000200.1	+	246302003	246302052
Sma-1-4436	NW_003038152.1	-	222520	222471
Sma-1-4437	NS_000200.1	-	292828244	292828195
Sma-1-4438	NW_003034742.1	+	175	224
Sma-1-4439	NW_003037953.1	-	233720	233671
Sma-1-4440	NS_000200.1	+	346907083	346907132
Sma-1-4441	NS_000200.1	-	193146771	193146722
Sma-1-4442	NW_003038517.1	-	245757	245708
Sma-1-4443	NS_000200.1	-	341010245	341010196
Sma-1-4444	NW_003038258.1	-	24741	24692
Sma-1-4445	NS_000200.1	-	322563251	322563202
Sma-1-4446	NW_003037886.1	+	1125907	1125956
Sma-1-4447	NS_000200.1	+	130381474	130381523
Sma-1-4448	NW_003038033.1	+	535067	535116
Sma-1-4449	NS_000200.1	+	246515069	246515118
Sma-1-4450	NW_003038258.1	-	98265	98216
Sma-1-4451	NS_000200.1	-	322636775	322636726
Sma-1-4452	NW_003038259.1	-	118168	118119
Sma-1-4453	NS_000200.1	-	322978767	322978718
Sma-1-4454	NW_003037946.1	-	775586	775537
Sma-1-4455	NS_000200.1	-	191469446	191469397
Sma-1-4456	NW_003038259.1	-	136331	136282
Sma-1-4457	NS_000200.1	-	322996930	322996881
Sma-1-4458	NW_003038259.1	-	275091	275042
Sma-1-4459	NS_000200.1	-	323135690	323135641
Sma-1-4460	NW_003037946.1	+	772888	772937
Sma-1-4461	NS_000200.1	+	191466748	191466797
Sma-1-4462	NW_003037866.1	+	697836	697885
Sma-1-4463	NS_000200.1	+	112185635	112185684
Sma-1-4464	NW_003038033.1	-	697841	697792
Sma-1-4465	NS_000200.1	-	246677843	246677794
Sma-1-4466	NW_003038033.1	+	72048	72097
Sma-1-4467	NS_000200.1	+	246052050	246052099
Sma-1-4468	NW_003038033.1	+	785981	786030
Sma-1-4469	NS_000200.1	+	246765983	246766032
Sma-1-4470	NW_003037911.1	-	629050	629001
Sma-1-4471	NS_000200.1	-	156416403	156416354
Sma-1-4472	NW_003037946.1	+	1027853	1027902
Sma-1-4473	NS_000200.1	+	191721713	191721762
Sma-1-4474	NW_003037867.1	+	23139	23188
Sma-1-4475	NS_000200.1	+	115623958	115624007
Sma-1-4476	NW_003038509.1	-	70633	70584
Sma-1-4477	NS_000200.1	-	337703085	337703036
Sma-1-4478	NW_003037810.1	+	297801	297850
Sma-1-4479	NS_000200.1	+	36902323	36902372
Sma-1-4480	NW_003037945.1	-	734696	734647

Sma-1-4481	NS_000200.1	-	189836660	189836611
Sma-1-4482	NW_003038033.1	+	812829	812878
Sma-1-4483	NS_000200.1	+	246792831	246792880
Sma-1-4484	NW_003038152.1	+	370616	370665
Sma-1-4485	NS_000200.1	+	292976340	292976389
Sma-1-4486	NW_003037809.1	+	2684674	2684723
Sma-1-4487	NS_000200.1	+	36349138	36349187
Sma-1-4488	NW_003037811.1	+	1271230	1271279
Sma-1-4489	NS_000200.1	+	39441472	39441521
Sma-1-4490	NW_003037806.1	-	28431	28382
Sma-1-4491	NS_000200.1	-	28223645	28223596
Sma-1-4492	NW_003037868.1	+	15518	15567
Sma-1-4493	NS_000200.1	+	115987230	115987279
Sma-1-4494	NW_003037945.1	+	1096676	1096725
Sma-1-4495	NS_000200.1	+	190198640	190198689
Sma-1-4496	NW_003037797.1	-	3773406	3773357
Sma-1-4497	NS_000200.1	-	14201266	14201217
Sma-1-4498	NW_003038033.1	-	890477	890428
Sma-1-4499	NS_000200.1	-	246870479	246870430
Sma-1-4500	NW_003038153.1	-	137714	137665
Sma-1-4501	NS_000200.1	-	293199763	293199714
Sma-1-4502	NW_003037796.1	+	1681983	1682032
Sma-1-4503	NS_000200.1	+	9891627	9891676
Sma-1-4504	NW_003037820.1	-	258196	258147
Sma-1-4505	NS_000200.1	-	52531178	52531129
Sma-1-4506	NW_003037868.1	-	86717	86668
Sma-1-4507	NS_000200.1	-	116058429	116058380
Sma-1-4508	NW_003038084.1	+	556542	556591
Sma-1-4509	NS_000200.1	+	271571786	271571835
Sma-1-4510	NW_003038261.1	+	280304	280353
Sma-1-4511	NS_000200.1	+	323893955	323894004
Sma-1-4512	NW_003035759.1	+	1871	1920
Sma-1-4513	NS_000200.1	+	364017215	364017264
Sma-1-4514	NW_003037870.1	+	250340	250389
Sma-1-4515	NS_000200.1	+	117124005	117124054
Sma-1-4516	NW_003037819.1	-	971501	971452
Sma-1-4517	NS_000200.1	-	52059766	52059717
Sma-1-4518	NW_003038034.1	+	308230	308279
Sma-1-4519	NS_000200.1	+	247249986	247250035
Sma-1-4520	NW_003038085.1	-	469709	469660
Sma-1-4521	NS_000200.1	-	272159802	272159753
Sma-1-4522	NW_003038252.1	+	4177	4226
Sma-1-4523	NS_000200.1	+	318859765	318859814
Sma-1-4524	NW_003035852.1	+	166	215
Sma-1-4525	NW_003037932.1	-	135162	135113
Sma-1-4526	NS_000200.1	+	364225226	364225275
Sma-1-4527	NS_000200.1	-	177668358	177668309
Sma-1-4528	NW_003037885.1	-	634446	634397
Sma-1-4529	NS_000200.1	-	128732393	128732344
Sma-1-4530	NW_003037944.1	+	583862	583911
Sma-1-4531	NS_000200.1	+	188805215	188805264
Sma-1-4532	NW_003037819.1	-	150513	150464

Sma-1-4533	NS_000200.1	-	51238778	51238729
Sma-1-4534	NW_003037871.1	+	209520	209569
Sma-1-4535	NS_000200.1	+	117657258	117657307
Sma-1-4536	NW_003037819.1	+	601877	601926
Sma-1-4537	NS_000200.1	+	51690142	51690191
Sma-1-4538	NW_003037793.1	-	3303317	3303268
Sma-1-4539	NS_000200.1	-	3303317	3303268
Sma-1-4540	NW_003035870.1	+	94606	94655
Sma-1-4541	NS_000200.1	+	335459895	335459944
Sma-1-4542	NW_003039130.1	-	130335	130286
Sma-1-4543	NS_000200.1	-	317804946	317804897
Sma-1-4544	NW_003038037.1	-	854217	854168
Sma-1-4545	NS_000200.1	-	251009626	251009577
Sma-1-4546	NW_003038261.1	+	576251	576300
Sma-1-4547	NW_003038261.1	+	577133	577182
Sma-1-4548	NS_000200.1	+	324189902	324189951
Sma-1-4549	NS_000200.1	+	324190784	324190833
Sma-1-4550	NW_003037885.1	+	519951	520000
Sma-1-4551	NS_000200.1	+	128617898	128617947
Sma-1-4552	NW_003037819.1	+	319214	319263
Sma-1-4553	NS_000200.1	+	51407479	51407528
Sma-1-4554	NW_003037819.1	-	2038	1989
Sma-1-4555	NS_000200.1	-	51090303	51090254
Sma-1-4556	NW_003037819.1	+	187141	187190
Sma-1-4557	NS_000200.1	+	51275406	51275455
Sma-1-4558	NW_003038261.1	-	696338	696289
Sma-1-4559	NS_000200.1	-	324309989	324309940
Sma-1-4560	NW_003037885.1	-	483038	482989
Sma-1-4561	NS_000200.1	-	128580985	128580936
Sma-1-4562	NW_003038035.1	+	394835	394884
Sma-1-4563	NS_000200.1	+	248256327	248256376
Sma-1-4564	NW_003038086.1	+	265891	265940
Sma-1-4565	NS_000200.1	+	272514484	272514533
Sma-1-4566	NW_003037937.1	+	541249	541298
Sma-1-4567	NS_000200.1	+	181741158	181741207
Sma-1-4568	NW_003037935.1	+	899653	899702
Sma-1-4569	NS_000200.1	+	180316188	180316237
Sma-1-4570	NW_003037924.1	+	541079	541128
Sma-1-4571	NS_000200.1	+	172248037	172248086
Sma-1-4572	NW_003038053.1	-	73665	73616
Sma-1-4573	NS_000200.1	-	257433760	257433711
Sma-1-4574	NW_003037915.1	-	297694	297645
Sma-1-4575	NS_000200.1	-	162197680	162197631
Sma-1-4576	NW_003037915.1	-	1043559	1043510
Sma-1-4577	NS_000200.1	-	162943545	162943496
Sma-1-4578	NW_003037873.1	-	508112	508063
Sma-1-4579	NS_000200.1	-	119247240	119247191
Sma-1-4580	NW_003037885.1	+	1018043	1018092
Sma-1-4581	NS_000200.1	+	129115990	129116039
Sma-1-4582	NW_003037819.1	+	135481	135530
Sma-1-4583	NS_000200.1	+	51223746	51223795
Sma-1-4584	NW_003038036.1	-	265977	265928

Sma-1-4585	NS_000200.1	-	249199829	249199780
Sma-1-4586	NW_003037819.1	+	1128555	1128604
Sma-1-4587	NS_000200.1	+	52216820	52216869
Sma-1-4588	NW_003037802.1	+	871019	871068
Sma-1-4589	NS_000200.1	+	22873813	22873862
Sma-1-4590	NW_003038036.1	+	311575	311624
Sma-1-4591	NS_000200.1	+	249245427	249245476
Sma-1-4592	NW_003035881.1	+	125353	125402
Sma-1-4593	NS_000200.1	+	341189558	341189607
Sma-1-4594	NW_003037819.1	+	1058318	1058367
Sma-1-4595	NS_000200.1	+	52146583	52146632
Sma-1-4596	NW_003037796.1	-	1810201	1810152
Sma-1-4597	NS_000200.1	-	10019845	10019796
Sma-1-4598	NW_003038036.1	-	902737	902688
Sma-1-4599	NS_000200.1	-	249836589	249836540
Sma-1-4600	NW_003038037.1	-	1307217	1307168
Sma-1-4601	NS_000200.1	-	251462626	251462577
Sma-1-4602	NW_003038037.1	+	1587024	1587073
Sma-1-4603	NS_000200.1	+	251742433	251742482
Sma-1-4604	NW_003038443.1	+	35868	35917
Sma-1-4605	NS_000200.1	+	330065514	330065563
Sma-1-4606	NW_003038086.1	+	764123	764172
Sma-1-4607	NS_000200.1	+	273012716	273012765
Sma-1-4608	NW_003037943.1	-	434828	434779
Sma-1-4609	NS_000200.1	-	187456361	187456312
Sma-1-4610	NW_003038156.1	-	158731	158682
Sma-1-4611	NS_000200.1	-	294890844	294890795
Sma-1-4612	NW_003037818.1	+	92235	92284
Sma-1-4613	NS_000200.1	+	48807642	48807691
Sma-1-4614	NW_003037818.1	-	917808	917759
Sma-1-4615	NS_000200.1	-	49633215	49633166
Sma-1-4616	NW_003038156.1	+	282831	282880
Sma-1-4617	NS_000200.1	+	295014944	295014993
Sma-1-4618	NW_003037943.1	+	1093946	1093995
Sma-1-4619	NS_000200.1	+	188115479	188115528
Sma-1-4620	NW_003037884.1	-	727223	727174
Sma-1-4621	NS_000200.1	-	127506888	127506839
Sma-1-4622	NW_003036292.1	-	284	235
Sma-1-4623	NS_000200.1	-	359576825	359576776
Sma-1-4624	NW_003038458.1	-	19843	19794
Sma-1-4625	NS_000200.1	-	331139496	331139447
Sma-1-4626	NW_003038156.1	+	490406	490455
Sma-1-4627	NS_000200.1	+	295222519	295222568
Sma-1-4628	NW_003038037.1	+	700637	700686
Sma-1-4629	NS_000200.1	+	250856046	250856095
Sma-1-4630	NW_003039130.1	+	5879	5928
Sma-1-4631	NS_000200.1	+	317680490	317680539
Sma-1-4632	NW_003038157.1	+	117803	117852
Sma-1-4633	NS_000200.1	+	295368723	295368772
Sma-1-4634	NW_003037912.1	+	942999	943048
Sma-1-4635	NS_000200.1	+	157754574	157754623
Sma-1-4636	NW_003038468.1	+	22802	22851

Sma-1-4637	NS_000200.1	+	331731072	331731121
Sma-1-4638	NW_003037875.1	+	447880	447929
Sma-1-4639	NS_000200.1	+	120476549	120476598
Sma-1-4640	NW_003037884.1	-	1069223	1069174
Sma-1-4641	NS_000200.1	-	127848888	127848839
Sma-1-4642	NW_003038124.1	-	34720	34671
Sma-1-4643	NS_000200.1	-	279027364	279027315
Sma-1-4644	NW_003038159.1	+	103277	103326
Sma-1-4645	NS_000200.1	+	296491298	296491347
Sma-1-4646	NW_003037807.1	+	176892	176941
Sma-1-4647	NS_000200.1	+	30438408	30438457
Sma-1-4648	NW_003038517.1	-	276953	276904
Sma-1-4649	NS_000200.1	-	341041441	341041392
Sma-1-4650	NW_003037806.1	-	245412	245363
Sma-1-4651	NS_000200.1	-	28440626	28440577
Sma-1-4652	NW_003037794.1	-	511630	511581
Sma-1-4653	NS_000200.1	-	4691225	4691176
Sma-1-4654	NW_003038481.1	+	87047	87096
Sma-1-4655	NS_000200.1	+	332870770	332870819
Sma-1-4656	NW_003038483.1	+	29024	29073
Sma-1-4657	NS_000200.1	+	333209282	333209331
Sma-1-4658	NW_003037818.1	+	2250627	2250676
Sma-1-4659	NS_000200.1	+	50966034	50966083
Sma-1-4660	NW_003037818.1	-	220753	220704
Sma-1-4661	NS_000200.1	-	48936160	48936111
Sma-1-4662	NW_003037550.1	-	1828	1779
Sma-1-4663	NS_000200.1	-	371617265	371617216
Sma-1-4664	NW_003038160.1	-	24925	24876
Sma-1-4665	NS_000200.1	-	296776856	296776807
Sma-1-4666	NW_003037882.1	+	97877	97926
Sma-1-4667	NS_000200.1	+	125389551	125389600
Sma-1-4668	NW_003037585.1	+	4636	4685
Sma-1-4669	NS_000200.1	+	372525807	372525856
Sma-1-4670	NW_003037913.1	+	279408	279457
Sma-1-4671	NS_000200.1	+	158843159	158843208
Sma-1-4672	NW_003038501.1	+	190062	190111
Sma-1-4673	NS_000200.1	+	334984114	334984163
Sma-1-4674	NW_003038038.1	+	381584	381633
Sma-1-4675	NS_000200.1	+	252284977	252285026
Sma-1-4676	NW_003037913.1	+	347690	347739
Sma-1-4677	NS_000200.1	+	158911441	158911490
Sma-1-4678	NW_003037641.1	+	204	253
Sma-1-4679	NW_003037794.1	+	293884	293933
Sma-1-4680	NW_003037865.1	+	61715	61764
Sma-1-4681	NS_000200.1	+	4473479	4473528
Sma-1-4682	NS_000200.1	+	110483634	110483683
Sma-1-4683	NS_000200.1	+	374259262	374259311
Sma-1-4684	NW_003038109.1	-	191225	191176
Sma-1-4685	NS_000200.1	-	275873903	275873854
Sma-1-4686	NW_003038057.1	-	293749	293700
Sma-1-4687	NS_000200.1	-	259499565	259499516
Sma-1-4688	NW_003038038.1	-	468155	468106

Sma-1-4689	NS_000200.1	-	252371548	252371499
Sma-1-4690	NW_003037940.1	-	825666	825617
Sma-1-4691	NS_000200.1	-	184567796	184567747
Sma-1-4692	NW_003037879.1	-	298375	298326
Sma-1-4693	NS_000200.1	-	123300631	123300582
Sma-1-4694	NW_003037793.1	-	2811076	2811027
Sma-1-4695	NS_000200.1	-	2811076	2811027
Sma-1-4696	NW_003037837.1	+	438827	438877
Sma-1-4697	NS_000200.1	+	77244685	77244735
Sma-1-4698	NW_003037915.1	-	903079	903029
Sma-1-4699	NS_000200.1	-	162803065	162803015
Sma-1-4700	NW_003037805.1	-	422380	422330
Sma-1-4701	NS_000200.1	-	26980224	26980174
Sma-1-4702	NW_003038514.1	-	102030	101980
Sma-1-4703	NS_000200.1	-	339726569	339726519
Sma-1-4704	NW_003037940.1	-	696847	696798
Sma-1-4705	NS_000200.1	-	184438977	184438928
Sma-1-4706	NW_003038502.1	+	66379	66428
Sma-1-4707	NS_000200.1	+	335065424	335065473
Sma-1-4708	NW_003037880.1	+	319854	319903
Sma-1-4709	NS_000200.1	+	124003412	124003461
Sma-1-4710	NW_003037798.1	-	1279395	1279346
Sma-1-4711	NS_000200.1	-	15538553	15538504
Sma-1-4712	NW_003038505.1	+	154080	154129
Sma-1-4713	NS_000200.1	+	336233417	336233466
Sma-1-4714	NW_003038110.1	+	121940	121989
Sma-1-4715	NS_000200.1	+	276288143	276288192
Sma-1-4716	NW_003037918.1	+	1711487	1711536
Sma-1-4717	NS_000200.1	+	168278477	168278526
Sma-1-4718	NW_003037913.1	-	699734	699685
Sma-1-4719	NS_000200.1	-	159263485	159263436
Sma-1-4720	NW_003037938.1	-	790604	790555
Sma-1-4721	NS_000200.1	-	182827717	182827668
Sma-1-4722	NW_003037882.1	-	169834	169785
Sma-1-4723	NS_000200.1	-	125461508	125461459
Sma-1-4724	NW_003037785.1	+	116153	116202
Sma-1-4725	NS_000200.1	+	380014820	380014869
Sma-1-4726	NW_003037817.1	-	65704	65655
Sma-1-4727	NS_000200.1	-	47448056	47448007
Sma-1-4728	NW_003037940.1	-	436818	436769
Sma-1-4729	NS_000200.1	-	184178948	184178899
Sma-1-4730	NW_003037806.1	-	1318910	1318861
Sma-1-4731	NS_000200.1	-	29514124	29514075
Sma-1-4732	NW_003037813.1	-	1726990	1726941
Sma-1-4733	NS_000200.1	-	42713853	42713804
Sma-1-4734	NW_003037787.1	+	194731	194780
Sma-1-4735	NS_000200.1	+	380442900	380442949
Sma-1-4736	NW_003037881.1	+	642318	642367
Sma-1-4737	NS_000200.1	+	125074109	125074158
Sma-1-4738	NW_003037918.1	+	2217112	2217161
Sma-1-4739	NS_000200.1	+	168784102	168784151
Sma-1-4740	NW_003037798.1	+	1901516	1901565

Sma-1-4741	NS_000200.1	+	16160674	16160723
Sma-1-4742	NW_003037817.1	-	599712	599663
Sma-1-4743	NS_000200.1	-	47982064	47982015
Sma-1-4744	NW_003037809.1	-	2212895	2212846
Sma-1-4745	NS_000200.1	-	35877359	35877310
Sma-1-4746	NW_003037925.1	+	357601	357650
Sma-1-4747	NS_000200.1	+	172759604	172759653
Sma-1-4748	NW_003037800.1	-	1166230	1166181
Sma-1-4749	NS_000200.1	-	19795590	19795541
Sma-1-4750	NW_003037788.1	-	209470	209421
Sma-1-4751	NS_000200.1	-	380656708	380656659
Sma-1-4752	NW_003037928.1	+	513693	513742
Sma-1-4753	NS_000200.1	+	174837646	174837695
Sma-1-4754	NW_003037925.1	+	207272	207321
Sma-1-4755	NS_000200.1	+	172609275	172609324
Sma-1-4756	NW_003037800.1	+	1436208	1436257
Sma-1-4757	NS_000200.1	+	20065568	20065617
Sma-1-4758	NW_003037804.1	-	766809	766760
Sma-1-4759	NW_003038169.1	+	780	829
Sma-1-4760	NS_000200.1	+	300483948	300483997
Sma-1-4761	NS_000200.1	-	25906829	25906780
Sma-1-4762	NW_003037809.1	-	1618936	1618887
Sma-1-4763	NS_000200.1	-	35283400	35283351
Sma-1-4764	NW_003037800.1	+	1768958	1769007
Sma-1-4765	NS_000200.1	+	20398318	20398367
Sma-1-4766	NW_003037800.1	-	1864537	1864488
Sma-1-4767	NS_000200.1	-	20493897	20493848
Sma-1-4768	NW_003037924.1	-	180668	180619
Sma-1-4769	NS_000200.1	-	171887626	171887577
Sma-1-4770	NW_003037939.1	-	764503	764454
Sma-1-4771	NS_000200.1	-	183634257	183634208
Sma-1-4772	NW_003037914.1	-	1407496	1407447
Sma-1-4773	NS_000200.1	-	161551746	161551697
Sma-1-4774	NW_003037800.1	+	2075041	2075090
Sma-1-4775	NS_000200.1	+	20704401	20704450
Sma-1-4776	NW_003037800.1	+	2155976	2156025
Sma-1-4777	NS_000200.1	+	20785336	20785385
Sma-1-4778	NW_003037800.1	-	321363	321314
Sma-1-4779	NS_000200.1	-	18950723	18950674
Sma-1-4780	NW_003037809.1	+	2778255	2778304
Sma-1-4781	NS_000200.1	+	36442719	36442768
Sma-1-4782	NW_003037816.1	+	403020	403069
Sma-1-4783	NS_000200.1	+	46611892	46611941
Sma-1-4784	NW_003037922.1	+	355184	355233
Sma-1-4785	NS_000200.1	+	170279181	170279230
Sma-1-4786	NW_003037816.1	-	283140	283091
Sma-1-4787	NS_000200.1	-	46492012	46491963
Sma-1-4788	NW_003037881.1	+	81326	81375
Sma-1-4789	NS_000200.1	+	124513117	124513166
Sma-1-4790	NW_003037922.1	-	177095	177046
Sma-1-4791	NS_000200.1	-	170101092	170101043
Sma-1-4792	NW_003037922.1	-	163631	163582

Sma-1-4793	NS_000200.1	-	170087628	170087579
Sma-1-4794	NW_003037926.1	-	307236	307187
Sma-1-4795	NS_000200.1	-	173475506	173475457
Sma-1-4796	NW_003037801.1	-	732022	731973
Sma-1-4797	NS_000200.1	-	21821502	21821453
Sma-1-4798	NW_003037801.1	-	821213	821164
Sma-1-4799	NS_000200.1	-	21910693	21910644
Sma-1-4800	NW_003037917.1	+	1399716	1399765
Sma-1-4801	NS_000200.1	+	166436520	166436569
Sma-1-4802	NW_003037793.1	+	1183068	1183118
Sma-1-4803	NS_000200.1	+	1183068	1183118
Sma-1-4804	NW_003037803.1	-	323566	323517
Sma-1-4805	NS_000200.1	-	23824103	23824054
Sma-1-4806	NW_003037929.1	+	323206	323255
Sma-1-4807	NS_000200.1	+	175563984	175564033
Sma-1-4808	NW_003037793.1	-	1432871	1432822
Sma-1-4809	NS_000200.1	-	1432871	1432822
Sma-1-4810	NW_003037802.1	+	62918	62967
Sma-1-4811	NS_000200.1	+	22065712	22065761
Sma-1-4812	NW_003037917.1	+	1407846	1407895
Sma-1-4813	NS_000200.1	+	166444650	166444699
Sma-1-4814	NW_003037793.1	+	1586301	1586350
Sma-1-4815	NS_000200.1	+	1586301	1586350
Sma-1-4816	NW_003037803.1	+	1000987	1001036
Sma-1-4817	NS_000200.1	+	24501524	24501573
Sma-1-4818	NW_003037914.1	+	41147	41196
Sma-1-4819	NS_000200.1	+	160185397	160185446
Sma-1-4820	NW_003037814.1	-	544738	544689
Sma-1-4821	NS_000200.1	-	43528598	43528549
Sma-1-4822	NW_003037814.1	+	502742	502791
Sma-1-4823	NS_000200.1	+	43486602	43486651
Sma-1-4824	NW_003038516.1	-	86319	86270
Sma-1-4825	NW_003038129.1	-	112133	112084
Sma-1-4826	NS_000200.1	-	340574724	340574675
Sma-1-4827	NS_000200.1	-	281365480	281365431
Sma-1-4828	NW_003037807.1	+	960701	960750
Sma-1-4829	NS_000200.1	+	31222217	31222266
Sma-1-4830	NW_003037939.1	+	191937	191986
Sma-1-4831	NS_000200.1	+	183061691	183061740
Sma-1-4832	NW_003037970.1	+	358461	358506
Sma-1-4833	NS_000200.1	+	201414651	201414696
env-82	DQ831701.1	+	213	258
env-83	AB231864.1	-	4604	4559
Sma-1-4834	NW_003037980.1	-	154040	153995
Sma-1-4835	NS_000200.1	-	211114902	211114857
Sma-1-4836	NW_003037829.1	-	781284	781235
Sma-1-4837	NS_000200.1	-	66681822	66681773
Sma-1-4838	NW_003037833.1	-	481086	481037
Sma-1-4839	NS_000200.1	-	72672182	72672133
Sma-1-4840	NW_003037844.1	-	564619	564571
Sma-1-4841	NS_000200.1	-	86748065	86748017
Sma-1-4842	NW_003037856.1	+	360122	360170

Sma-1-4843	NS_000200.1	+	99718078	99718126
Sma-1-4844	NW_003037881.1	-	645384	645335
Sma-1-4845	NS_000200.1	-	125077175	125077126
Sma-1-4846	NW_003037918.1	-	1656253	1656204
Sma-1-4847	NS_000200.1	-	168223243	168223194
Sma-1-4848	NW_003037782.1	-	177250	177201
Sma-1-4849	NS_000200.1	-	379500693	379500644
Sma-1-4850	NW_003037793.1	+	164115	164164
Sma-1-4851	NS_000200.1	+	164115	164164
Sma-1-4852	NW_003037864.1	-	638621	638572
Sma-1-4853	NS_000200.1	-	109131195	109131146
Sma-1-4854	NW_003037809.1	-	2403893	2403844
Sma-1-4855	NS_000200.1	-	36068357	36068308
Sma-1-4856	NW_003024547.1	+	151	200
Sma-1-4857	NW_003037865.1	-	85458	85409
Sma-1-4858	NS_000200.1	+	348832406	348832455
Sma-1-4859	NS_000200.1	-	110507377	110507328
Sma-1-4860	NW_003037914.1	+	1230130	1230179
Sma-1-4861	NS_000200.1	+	161374380	161374429
Sma-1-4862	NW_003037882.1	+	105183	105232
Sma-1-4863	NS_000200.1	+	125396857	125396906
Sma-1-4864	NW_003037880.1	+	724544	724593
Sma-1-4865	NS_000200.1	+	124408102	124408151
Sma-1-4866	NW_003037882.1	+	184311	184360
Sma-1-4867	NS_000200.1	+	125475985	125476034
Sma-1-4868	NW_003037882.1	-	214221	214172
Sma-1-4869	NS_000200.1	-	125505895	125505846
Sma-1-4870	NW_003037882.1	+	234496	234545
Sma-1-4871	NS_000200.1	+	125526170	125526219
Sma-1-4872	NW_003037882.1	-	258096	258047
Sma-1-4873	NS_000200.1	-	125549770	125549721
Sma-1-4874	NW_003037882.1	+	519862	519911
Sma-1-4875	NS_000200.1	+	125811536	125811585
Sma-1-4876	NW_003037878.1	-	459054	459005
Sma-1-4877	NS_000200.1	-	122512532	122512483
Sma-1-4878	NW_003037876.1	+	49504	49553
Sma-1-4879	NW_003037797.1	-	2899372	2899323
Sma-1-4880	NS_000200.1	+	120595140	120595189
Sma-1-4881	NS_000200.1	-	13327232	13327183
Sma-1-4882	NW_003037876.1	-	478592	478543
Sma-1-4883	NS_000200.1	-	121024228	121024179
Sma-1-4884	NW_003037876.1	-	26229	26180
Sma-1-4885	NS_000200.1	-	120571865	120571816
Sma-1-4886	NW_003037884.1	-	1246534	1246485
Sma-1-4887	NS_000200.1	-	128026199	128026150
Sma-1-4888	NW_003037882.1	-	396638	396589
Sma-1-4889	NS_000200.1	-	125688312	125688263
Sma-1-4890	NW_003037884.1	-	1288028	1287979
Sma-1-4891	NS_000200.1	-	128067693	128067644
Sma-1-4892	NW_003037858.1	-	410269	410220
Sma-1-4893	NW_003037918.1	-	526588	526539
Sma-1-4894	NS_000200.1	-	167093578	167093529

Sma-1-4895	NS_000200.1	-	101074545	101074496
Sma-1-4896	NW_003038150.1	-	235441	235392
Sma-1-4897	NS_000200.1	-	291714179	291714130
Sma-1-4898	NW_003037859.1	-	645692	645643
Sma-1-4899	NS_000200.1	-	103324533	103324484
Sma-1-4900	NW_003037885.1	-	1080806	1080757
Sma-1-4901	NS_000200.1	-	129178753	129178704
Sma-1-4902	NW_003037873.1	+	153629	153678
Sma-1-4903	NS_000200.1	+	118892757	118892806
Sma-1-4904	NW_003037871.1	-	472825	472776
Sma-1-4905	NS_000200.1	-	117920563	117920514
Sma-1-4906	NW_003037869.1	-	394055	394006
Sma-1-4907	NS_000200.1	-	116866400	116866351
Sma-1-4908	NW_003037865.1	+	760238	760287
Sma-1-4909	NS_000200.1	+	111182157	111182206
Sma-1-4910	NW_003037865.1	-	564355	564306
Sma-1-4911	NS_000200.1	-	110986274	110986225
Sma-1-4912	NW_003037863.1	+	998370	998419
Sma-1-4913	NS_000200.1	+	107756737	107756786
Sma-1-4914	NW_003037913.1	-	73572	73523
Sma-1-4915	NS_000200.1	-	158637323	158637274
Sma-1-4916	NW_003037589.1	+	16929	16978
Sma-1-4917	NS_000200.1	+	372665881	372665930
Sma-1-4918	NW_003038518.1	+	664834	664883
Sma-1-4919	NS_000200.1	+	342301667	342301716
Sma-1-4920	NW_003039129.1	-	241426	241377
Sma-1-4921	NS_000200.1	-	229196234	229196185
Sma-1-4922	NW_003038099.1	-	33109	33060
Sma-1-4923	NS_000200.1	-	274491322	274491273
Sma-1-4924	NW_003026517.1	+	374519	374568
Sma-1-4925	NS_000200.1	+	299980528	299980577
Sma-1-4926	NW_003038129.1	-	25982	25933
Sma-1-4927	NS_000200.1	-	281279329	281279280
Sma-1-4928	NW_003031890.1	+	439	488
Sma-1-4929	NW_003037982.1	+	1194458	1194507
Sma-1-4930	NW_003038141.1	+	164251	164300
Sma-1-4931	NS_000200.1	+	214853653	214853702
Sma-1-4932	NS_000200.1	+	287054230	287054279
Sma-1-4933	NS_000200.1	+	367216480	367216529
Sma-1-4934	NW_003026465.1	-	381101	381052
Sma-1-4935	NS_000200.1	-	207773695	207773646
Sma-1-4936	NW_003037929.1	+	111855	111904
Sma-1-4937	NW_003026456.1	-	562202	562153
Sma-1-4938	NW_003037804.1	-	776119	776070
Sma-1-4939	NW_003037841.1	-	558348	558299
Sma-1-4940	NW_003037889.1	-	1808388	1808339
Sma-1-4941	NW_003037982.1	-	240533	240484
Sma-1-4942	NS_000200.1	+	175352633	175352682
Sma-1-4943	NS_000200.1	-	213899728	213899679
Sma-1-4944	NS_000200.1	-	135584878	135584829
Sma-1-4945	NS_000200.1	-	83169529	83169480
Sma-1-4946	NS_000200.1	-	58485338	58485289

Sma-1-4947	NS_000200.1	-	25916139	25916090
Sma-1-4948	NW_003037818.1	+	2043635	2043684
Sma-1-4949	NS_000200.1	+	50759042	50759091
Sma-1-4950	NW_003037829.1	+	1088990	1089039
Sma-1-4951	NS_000200.1	+	66989528	66989577
Sma-1-4952	NW_003037863.1	+	1277013	1277062
Sma-1-4953	NS_000200.1	+	108035380	108035429
Sma-1-4954	NW_003037887.1	+	1453428	1453477
Sma-1-4955	NS_000200.1	+	131917406	131917455
Sma-1-4956	NW_003037887.1	+	1551844	1551893
Sma-1-4957	NW_003038158.1	+	88160	88209
Sma-1-4958	NS_000200.1	+	132015822	132015871
Sma-1-4959	NS_000200.1	+	295971328	295971377
Sma-1-4960	NW_003037862.1	+	757132	757181
Sma-1-4961	NS_000200.1	+	106408793	106408842
Sma-1-4962	NW_003037887.1	-	369381	369332
Sma-1-4963	NS_000200.1	-	130833359	130833310
Sma-1-4964	NW_003037860.1	-	525708	525659
Sma-1-4965	NS_000200.1	-	104268736	104268687
Sma-1-4966	NW_003037889.1	-	1247758	1247709
Sma-1-4967	NW_003038149.1	-	228143	228094
Sma-1-4968	NS_000200.1	-	291256756	291256707
Sma-1-4969	NS_000200.1	-	135024248	135024199
Sma-1-4970	NW_003037854.1	+	520090	520139
Sma-1-4971	NS_000200.1	+	97424511	97424560
Sma-1-4972	NW_003037890.1	+	780074	780123
Sma-1-4973	NS_000200.1	+	136463435	136463484
Sma-1-4974	NW_003037852.1	-	660595	660546
Sma-1-4975	NW_003037865.1	-	214865	214816
Sma-1-4976	NS_000200.1	-	110636784	110636735
Sma-1-4977	NS_000200.1	-	95052490	95052441
Sma-1-4978	NW_003037889.1	+	1844462	1844511
Sma-1-4979	NS_000200.1	+	135620952	135621001
Sma-1-4980	NW_003037889.1	-	412026	411977
Sma-1-4981	NS_000200.1	-	134188516	134188467
Sma-1-4982	NW_003026462.1	+	339942	339991
Sma-1-4983	NW_003037820.1	+	716416	716465
Sma-1-4984	NW_003037851.1	-	713394	713345
Sma-1-4985	NW_003037940.1	+	330419	330468
Sma-1-4986	NW_003038230.1	-	242924	242875
Sma-1-4987	NS_000200.1	+	52989398	52989447
Sma-1-4988	NS_000200.1	+	184072549	184072598
Sma-1-4989	NS_000200.1	+	197007445	197007494
Sma-1-4990	NS_000200.1	-	311528495	311528446
Sma-1-4991	NS_000200.1	-	94116704	94116655
Sma-1-4992	NW_003035431.1	+	218	267
Sma-1-4993	NS_000200.1	+	367454815	367454864
Sma-1-4994	NW_003037890.1	+	1392269	1392318
Sma-1-4995	NS_000200.1	+	137075630	137075679
Sma-1-4996	NW_003037853.1	+	176394	176443
Sma-1-4997	NS_000200.1	+	95668445	95668494
Sma-1-4998	NW_003037853.1	+	1048326	1048375

Sma-1-4999	NS_000200.1	+	96540377	96540426
Sma-1-5000	NW_003037822.1	+	460208	460257
Sma-1-5001	NS_000200.1	+	54768214	54768263
Sma-1-5002	NW_003026612.1	-	223365	223316
Sma-1-5003	NS_000200.1	-	312528752	312528703
Sma-1-5004	NW_003037866.1	+	766313	766362
Sma-1-5005	NW_003037899.1	+	326311	326360
Sma-1-5006	NS_000200.1	+	112254112	112254161
Sma-1-5007	NS_000200.1	+	144845422	144845471
Sma-1-5008	NW_003037890.1	-	1597752	1597703
Sma-1-5009	NS_000200.1	-	137281113	137281064
Sma-1-5010	NW_003024033.1	+	1156	1205
Sma-1-5011	NS_000200.1	+	344497301	344497350
Sma-1-5012	NW_003035881.1	-	139539	139490
Sma-1-5013	NW_003038125.1	-	85183	85134
Sma-1-5014	NS_000200.1	-	341203744	341203695
Sma-1-5015	NS_000200.1	-	279522139	279522090
Sma-1-5016	NW_003037821.1	+	223703	223752
Sma-1-5017	NS_000200.1	+	53368187	53368236
Sma-1-5018	NW_003037806.1	-	359575	359526
Sma-1-5019	NS_000200.1	-	28554789	28554740
Sma-1-5020	NW_003037799.1	+	574778	574827
Sma-1-5021	NS_000200.1	+	17565132	17565181
Sma-1-5022	NW_003037992.1	-	199851	199802
Sma-1-5023	NS_000200.1	-	218041332	218041283
Sma-1-5024	NW_003038005.1	-	469457	469408
Sma-1-5025	NS_000200.1	-	224875411	224875362
Sma-1-5026	NW_003038042.1	+	100747	100796
Sma-1-5027	NS_000200.1	+	254046346	254046395
Sma-1-5028	NW_003037918.1	-	600336	600287
Sma-1-5029	NS_000200.1	-	167167326	167167277
Sma-1-5030	NW_003038035.1	+	188280	188329
Sma-1-5031	NS_000200.1	+	248049772	248049821
Sma-1-5032	NW_003037822.1	+	400674	400723
Sma-1-5033	NS_000200.1	+	54708680	54708729
Sma-1-5034	NW_003037801.1	+	672107	672156
Sma-1-5035	NS_000200.1	+	21761587	21761636
Sma-1-5036	NW_003037945.1	+	393903	393952
Sma-1-5037	NS_000200.1	+	189495867	189495916
Sma-1-5038	NW_003034822.1	-	1765	1716
Sma-1-5039	NS_000200.1	-	347049489	347049440
Sma-1-5040	NW_003038258.1	+	283386	283435
Sma-1-5041	NS_000200.1	+	322821896	322821945
Sma-1-5042	NW_003020596.1	+	2136	2185
Sma-1-5043	NW_003026490.1	+	100854	100903
Sma-1-5044	NS_000200.1	+	267732570	267732619
Sma-1-5045	NS_000200.1	+	360344148	360344197
Sma-1-5046	NW_003037836.1	+	848190	848239
Sma-1-5047	NS_000200.1	+	76405289	76405338
Sma-1-5048	NW_003037851.1	+	572479	572528
Sma-1-5049	NS_000200.1	+	93975789	93975838
Sma-1-5050	NW_003037891.1	+	2138289	2138338

Sma-1-5051	NS_000200.1	+	140019567	140019616
Sma-1-5052	NW_003037841.1	-	1602688	1602639
Sma-1-5053	NW_003037916.1	+	1131211	1131260
Sma-1-5054	NS_000200.1	+	164324622	164324671
Sma-1-5055	NS_000200.1	-	84213869	84213820
Sma-1-5056	NW_003037840.1	+	367128	367177
Sma-1-5057	NS_000200.1	+	80990452	80990501
Sma-1-5058	NW_003037901.1	+	399662	399711
Sma-1-5059	NS_000200.1	+	146248123	146248172
Sma-1-5060	NW_003037906.1	+	484394	484443
Sma-1-5061	NS_000200.1	+	150792173	150792222
Sma-1-5062	NW_003037911.1	-	409179	409130
Sma-1-5063	NS_000200.1	-	156196532	156196483
Sma-1-5064	NW_003037913.1	-	939597	939548
Sma-1-5065	NS_000200.1	-	159503348	159503299
Sma-1-5066	NW_003037914.1	-	156352	156303
Sma-1-5067	NS_000200.1	-	160300602	160300553
Sma-1-5068	NW_003037925.1	+	138314	138363
Sma-1-5069	NS_000200.1	+	172540317	172540366
Sma-1-5070	NW_003038011.1	+	97261	97310
Sma-1-5071	NS_000200.1	+	227624911	227624960
Sma-1-5072	NW_003038014.1	+	108133	108182
Sma-1-5073	NS_000200.1	+	229848822	229848871
Sma-1-5074	NW_003038242.1	+	224957	225006
Sma-1-5075	NS_000200.1	+	314924103	314924152
Sma-1-5076	NW_003038321.1	-	280	231
Sma-1-5077	NS_000200.1	-	326143348	326143299
Sma-1-5078	NW_003038036.1	-	505927	505878
Sma-1-5079	NS_000200.1	-	249439779	249439730
Sma-1-5080	NW_003035639.1	+	11289	11338
Sma-1-5081	NW_003037795.1	+	1178969	1179018
Sma-1-5082	NW_003037825.1	+	292733	292782
Sma-1-5083	NW_003038246.1	-	176547	176498
Sma-1-5084	NW_003026481.1	-	80657	80608
Sma-1-5085	NW_003038058.1	-	129066	129017
Sma-1-5086	NW_003038083.1	+	764068	764117
Sma-1-5087	NS_000200.1	+	7569852	7569901
Sma-1-5088	NS_000200.1	+	59422515	59422564
Sma-1-5089	NS_000200.1	+	271007405	271007454
Sma-1-5090	NS_000200.1	+	329961729	329961778
Sma-1-5091	NS_000200.1	-	315817675	315817626
Sma-1-5092	NS_000200.1	-	259960266	259960217
Sma-1-5093	NS_000200.1	-	255480641	255480592
Sma-1-5094	NW_003037836.1	-	750392	750341
Sma-1-5095	NS_000200.1	-	76307491	76307440
Sma-1-5096	NW_003037803.1	-	1595899	1595850
Sma-1-5097	NS_000200.1	-	25096436	25096387
Sma-1-5098	NW_003037926.1	-	397236	397187
Sma-1-5099	NW_003038036.1	+	266312	266361
Sma-1-5100	NS_000200.1	+	249200164	249200213
Sma-1-5101	NS_000200.1	-	173565506	173565457
Sma-1-5102	NW_003037940.1	+	61866	61915

Sma-1-5103	NW_003038027.1	-	845466	845417
Sma-1-5104	NS_000200.1	+	183803996	183804045
Sma-1-5105	NS_000200.1	-	241216180	241216131
Sma-1-5106	NW_003037944.1	+	580779	580828
Sma-1-5107	NS_000200.1	+	188802132	188802181
Sma-1-5108	NW_003038014.1	+	531997	532046
Sma-1-5109	NS_000200.1	+	230272686	230272735
Sma-1-5110	NW_003038130.1	+	293856	293905
Sma-1-5111	NS_000200.1	+	281848842	281848891
Sma-1-5112	NW_003026610.1	-	3091	3042
Sma-1-5113	NW_003035748.1	+	629	678
Sma-1-5114	NW_003038508.1	-	18988	18939
Sma-1-5115	NW_003026456.1	-	281381	281332
Sma-1-5116	NW_003037798.1	-	89166	89117
Sma-1-5117	NW_003037814.1	-	1812699	1812650
Sma-1-5118	NW_003037836.1	+	4544	4593
Sma-1-5119	NW_003037887.1	+	1456525	1456574
Sma-1-5120	NW_003037938.1	+	738688	738737
Sma-1-5121	NW_003037938.1	+	739452	739501
Sma-1-5122	NW_003037970.1	-	401725	401676
Sma-1-5123	NW_003038023.1	+	401604	401653
Sma-1-5124	NW_003038037.1	-	97966	97917
Sma-1-5125	NW_003038071.1	-	22147	22098
Sma-1-5126	NW_003038068.1	+	190329	190378
Sma-1-5127	NS_000200.1	+	75561643	75561692
Sma-1-5128	NS_000200.1	+	131920503	131920552
Sma-1-5129	NS_000200.1	+	182775801	182775850
Sma-1-5130	NS_000200.1	+	182776565	182776614
Sma-1-5131	NS_000200.1	+	237561895	237561944
Sma-1-5132	NS_000200.1	+	263257537	263257586
Sma-1-5133	NS_000200.1	+	363995115	363995164
Sma-1-5134	NS_000200.1	-	337125956	337125907
Sma-1-5135	NS_000200.1	-	310158461	310158412
Sma-1-5136	NS_000200.1	-	264825190	264825141
Sma-1-5137	NS_000200.1	-	250253375	250253326
Sma-1-5138	NS_000200.1	-	201457915	201457866
Sma-1-5139	NS_000200.1	-	58204517	58204468
Sma-1-5140	NS_000200.1	-	44796559	44796510
Sma-1-5141	NS_000200.1	-	14348324	14348275
Sma-1-5142	NW_003037982.1	-	1206793	1206744
Sma-1-5143	NS_000200.1	-	214865988	214865939
Sma-1-5144	NW_003038117.1	-	167618	167569
Sma-1-5145	NS_000200.1	-	277485443	277485394
Sma-1-5146	NW_003038511.1	+	209002	209051
Sma-1-5147	NS_000200.1	+	338554214	338554263
Sma-1-5148	NW_003024925.1	-	595	546
Sma-1-5149	NW_003037836.1	-	796722	796673
Sma-1-5150	NW_003037837.1	+	19	68
Sma-1-5151	NW_003037931.1	-	45323	45274
Sma-1-5152	NW_003038148.1	-	486286	486237
Sma-1-5153	NS_000200.1	+	76805877	76805926
Sma-1-5154	NS_000200.1	-	355717955	355717906

Sma-1-5155	NS_000200.1	-	290560270	290560221
Sma-1-5156	NS_000200.1	-	176877990	176877941
Sma-1-5157	NS_000200.1	-	76353821	76353772
Sma-1-5158	NW_003026508.1	+	165305	165354
Sma-1-5159	NS_000200.1	+	281209919	281209968
Sma-1-5160	NW_003037796.1	-	1689328	1689279
Sma-1-5161	NS_000200.1	-	9898972	9898923
Sma-1-5162	NW_003037800.1	+	1558585	1558634
Sma-1-5163	NS_000200.1	+	20187945	20187994
Sma-1-5164	NW_003037832.1	+	568411	568460
Sma-1-5165	NW_003037859.1	+	346091	346140
Sma-1-5166	NW_003037859.1	+	351145	351194
Sma-1-5167	NS_000200.1	+	71777842	71777891
Sma-1-5168	NS_000200.1	+	103024932	103024981
Sma-1-5169	NS_000200.1	+	103029986	103030035
Sma-1-5170	NW_003037891.1	-	2053364	2053315
Sma-1-5171	NS_000200.1	-	139934642	139934593
Sma-1-5172	NW_003037847.1	-	339211	339162
Sma-1-5173	NS_000200.1	-	88990941	88990892
Sma-1-5174	NW_003026588.1	-	28389	28340
Sma-1-5175	NS_000200.1	-	303877532	303877483
Sma-1-5176	NW_003038150.1	+	318132	318181
Sma-1-5177	NS_000200.1	+	291796870	291796919
Sma-1-5178	NW_003038507.1	-	25395	25346
Sma-1-5179	NS_000200.1	-	336694289	336694240
Sma-1-5180	NW_003037827.1	-	218722	218673
Sma-1-5181	NS_000200.1	-	62840863	62840814
Sma-1-5182	NW_003026612.1	+	13238	13287
Sma-1-5183	NW_003038261.1	-	893184	893135
Sma-1-5184	NS_000200.1	+	312318625	312318674
Sma-1-5185	NS_000200.1	-	324506835	324506786
Sma-1-5186	NW_003037871.1	+	388077	388126
Sma-1-5187	NW_003037915.1	+	1216778	1216827
Sma-1-5188	NS_000200.1	+	117835815	117835864
Sma-1-5189	NS_000200.1	+	163116764	163116813
Sma-1-5190	NW_003038037.1	+	1227646	1227695
Sma-1-5191	NS_000200.1	+	251383055	251383104
Sma-1-5192	NW_003038042.1	+	201772	201821
Sma-1-5193	NW_003038080.1	+	596429	596478
Sma-1-5194	NS_000200.1	+	254147371	254147420
Sma-1-5195	NS_000200.1	+	269049039	269049088
Sma-1-5196	NW_003037898.1	-	458548	458499
Sma-1-5197	NS_000200.1	-	144442370	144442321
Sma-1-5198	NW_003037899.1	-	98864	98815
Sma-1-5199	NS_000200.1	-	144617975	144617926
Sma-1-5200	NW_003037837.1	-	209896	209847
Sma-1-5201	NS_000200.1	-	77015754	77015705
Sma-1-5202	NW_003038112.1	-	27508	27459
Sma-1-5203	NS_000200.1	-	276482686	276482637
Sma-1-5204	NW_003037791.1	+	719480	719529
Sma-1-5205	NS_000200.1	+	382424908	382424957
Sma-1-5206	NW_003038028.1	+	741297	741346

Sma-1-5207	NS_000200.1	+	242048871	242048920
Sma-1-5208	NW_003037804.1	+	1333048	1333097
Sma-1-5209	NS_000200.1	+	26473068	26473117
Sma-1-5210	NW_003037809.1	-	2676911	2676862
Sma-1-5211	NS_000200.1	-	36341375	36341326
Sma-1-5212	NW_003037905.1	+	218796	218845
Sma-1-5213	NW_003038518.1	+	72018	72067
Sma-1-5214	NS_000200.1	+	149487808	149487857
Sma-1-5215	NS_000200.1	+	341708851	341708900
Sma-1-5216	NW_003038004.1	-	140991	140942
Sma-1-5217	NS_000200.1	-	224308859	224308810
Sma-1-5218	NW_003037813.1	-	415692	415643
Sma-1-5219	NS_000200.1	-	41402555	41402506
Sma-1-5220	NW_003037905.1	+	297505	297554
Sma-1-5221	NS_000200.1	+	149566517	149566566
Sma-1-5222	NW_003037906.1	-	121130	121081
Sma-1-5223	NS_000200.1	-	150428909	150428860
Sma-1-5224	NW_003038058.1	-	292700	292651
Sma-1-5225	NS_000200.1	-	260123900	260123851
Sma-1-5226	NW_003037809.1	-	2522029	2521980
Sma-1-5227	NS_000200.1	-	36186493	36186444
Sma-1-5228	NW_003037829.1	+	1745363	1745412
Sma-1-5229	NS_000200.1	+	67645901	67645950
Sma-1-5230	NW_003037814.1	-	139140	139091
Sma-1-5231	NS_000200.1	-	43123000	43122951
Sma-1-5232	NW_003035881.1	+	346464	346513
Sma-1-5233	NW_003037914.1	+	1131354	1131403
Sma-1-5234	NW_003037974.1	-	134620	134571
Sma-1-5235	NW_003026507.1	-	279665	279616
Sma-1-5236	NS_000200.1	+	161275604	161275653
Sma-1-5237	NS_000200.1	+	341410669	341410718
Sma-1-5238	NS_000200.1	-	279939816	279939767
Sma-1-5239	NS_000200.1	-	204809926	204809877
Sma-1-5240	NW_003037793.1	-	2895766	2895717
Sma-1-5241	NS_000200.1	-	2895766	2895717
Sma-1-5242	NW_003037827.1	-	787713	787664
Sma-1-5243	NS_000200.1	-	63409854	63409805
Sma-1-5244	NW_003037907.1	+	947749	947798
Sma-1-5245	NW_003037982.1	+	240833	240882
Sma-1-5246	NS_000200.1	+	152352238	152352287
Sma-1-5247	NS_000200.1	+	213900028	213900077
Sma-1-5248	NW_003037908.1	+	866670	866719
Sma-1-5249	NS_000200.1	+	153339801	153339850
Sma-1-5250	NW_003037826.1	+	1274319	1274368
Sma-1-5251	NS_000200.1	+	62273835	62273884
Sma-1-5252	NW_003037825.1	-	1845764	1845715
Sma-1-5253	NS_000200.1	-	60975546	60975497
Sma-1-5254	NW_003037909.1	+	680565	680614
Sma-1-5255	NS_000200.1	+	154222214	154222263
Sma-1-5256	NW_003037909.1	-	943986	943937
Sma-1-5257	NS_000200.1	-	154485635	154485586
Sma-1-5258	NW_003038063.1	+	252548	252597

Sma-1-5259	NS_000200.1	+	261748826	261748875
Sma-1-5260	NW_003037821.1	-	417447	417398
Sma-1-5261	NW_003038028.1	+	502139	502188
Sma-1-5262	NS_000200.1	+	241809713	241809762
Sma-1-5263	NS_000200.1	-	53561931	53561882
Sma-1-5264	NW_003037915.1	-	1055382	1055333
Sma-1-5265	NS_000200.1	-	162955368	162955319
Sma-1-5266	NW_003037810.1	-	1081765	1081716
Sma-1-5267	NS_000200.1	-	37686287	37686238
Sma-1-5268	NW_003037944.1	+	743452	743501
Sma-1-5269	NS_000200.1	+	188964805	188964854
Sma-1-5270	NW_003037805.1	+	1093823	1093872
Sma-1-5271	NW_003037863.1	-	483454	483405
Sma-1-5272	NW_003038120.1	-	201357	201308
Sma-1-5273	NS_000200.1	+	27651667	27651716
Sma-1-5274	NS_000200.1	-	278386580	278386531
Sma-1-5275	NS_000200.1	-	107241821	107241772
Sma-1-5276	NW_003037797.1	+	1367445	1367494
Sma-1-5277	NS_000200.1	+	11795305	11795354
Sma-1-5278	NW_003038070.1	-	264767	264718
Sma-1-5279	NS_000200.1	-	264602008	264601959
Sma-1-5280	NW_003037815.1	-	519883	519834
Sma-1-5281	NW_003037990.1	-	237678	237629
Sma-1-5282	NW_003038076.1	+	524726	524775
Sma-1-5283	NS_000200.1	+	266741900	266741949
Sma-1-5284	NS_000200.1	-	217136437	217136388
Sma-1-5285	NS_000200.1	-	45782874	45782825
Sma-1-5286	NW_003026616.1	+	129659	129708
Sma-1-5287	NS_000200.1	+	317486258	317486307
Sma-1-5288	NW_003038252.1	-	418759	418710
Sma-1-5289	NW_003038117.1	+	206559	206608
Sma-1-5290	NS_000200.1	+	277524384	277524433
Sma-1-5291	NS_000200.1	-	319274347	319274298
Sma-1-5292	NW_003035872.1	+	7036	7085
Sma-1-5293	NS_000200.1	+	335628783	335628832
Sma-1-5294	NW_003038462.1	-	52372	52323
Sma-1-5295	NS_000200.1	-	331420035	331419986
Sma-1-5296	NW_003037890.1	+	254570	254619
Sma-1-5297	NS_000200.1	+	135937931	135937980
Sma-1-5298	NW_003038079.1	-	272649	272600
Sma-1-5299	NS_000200.1	-	268402637	268402588
Sma-1-5300	NW_003038034.1	-	539735	539686
Sma-1-5301	NW_003038261.1	-	193735	193686
Sma-1-5302	NS_000200.1	-	323807386	323807337
Sma-1-5303	NS_000200.1	-	247481491	247481442
Sma-1-5304	NW_003039129.1	+	17385	17434
Sma-1-5305	NW_003026587.1	+	29218	29267
Sma-1-5306	NW_003026587.1	-	32335	32286
Sma-1-5307	NW_003026456.1	+	361455	361504
Sma-1-5308	NW_003037798.1	+	1312961	1313010
Sma-1-5309	NW_003037807.1	+	420441	420490
Sma-1-5310	NW_003037812.1	-	791496	791447

Sma-1-5311	NW_003037812.1	-	791102	791053
Sma-1-5312	NW_003037814.1	-	2094	2045
Sma-1-5313	NW_003037827.1	+	1664783	1664832
Sma-1-5314	NW_003037850.1	-	1231171	1231122
Sma-1-5315	NW_003037865.1	+	817306	817355
Sma-1-5316	NW_003037880.1	-	292911	292862
Sma-1-5317	NW_003037889.1	-	950121	950072
Sma-1-5318	NW_003037906.1	+	26992	27041
Sma-1-5319	NW_003037994.1	+	567815	567864
Sma-1-5320	NW_003037997.1	-	56827	56778
Sma-1-5321	NW_003039127.1	+	1931579	1931628
Sma-1-5322	NW_003038086.1	+	330138	330187
Sma-1-5323	NW_003038141.1	-	249710	249661
Sma-1-5324	NW_003038152.1	+	322589	322638
Sma-1-5325	NW_003038187.1	+	103117	103166
Sma-1-5326	NS_000200.1	+	15572119	15572168
Sma-1-5327	NS_000200.1	+	30681957	30682006
Sma-1-5328	NS_000200.1	+	58284591	58284640
Sma-1-5329	NS_000200.1	+	64286924	64286973
Sma-1-5330	NS_000200.1	+	111239225	111239274
Sma-1-5331	NS_000200.1	+	115009078	115009127
Sma-1-5332	NS_000200.1	+	150334771	150334820
Sma-1-5333	NS_000200.1	+	219099629	219099678
Sma-1-5334	NS_000200.1	+	228972193	228972242
Sma-1-5335	NS_000200.1	+	272578731	272578780
Sma-1-5336	NS_000200.1	+	292928313	292928362
Sma-1-5337	NS_000200.1	+	302054975	302055024
Sma-1-5338	NS_000200.1	+	303842109	303842158
Sma-1-5339	NS_000200.1	-	303845226	303845177
Sma-1-5340	NS_000200.1	-	287139689	287139640
Sma-1-5341	NS_000200.1	-	220868269	220868220
Sma-1-5342	NS_000200.1	-	134726611	134726562
Sma-1-5343	NS_000200.1	-	123976469	123976420
Sma-1-5344	NS_000200.1	-	93217201	93217152
Sma-1-5345	NS_000200.1	-	42985954	42985905
Sma-1-5346	NS_000200.1	-	40274235	40274186
Sma-1-5347	NS_000200.1	-	40273841	40273792
Sma-1-5348	NW_003037927.1	+	70827	70876
Sma-1-5349	NS_000200.1	+	174048190	174048239
Sma-1-5350	NW_003037851.1	+	891156	891205
Sma-1-5351	NS_000200.1	+	94294466	94294515
Sma-1-5352	NW_003026618.1	-	232842	232793
Sma-1-5353	NS_000200.1	-	321987844	321987795
Sma-1-5354	NW_003038161.1	-	473417	473368
Sma-1-5355	NS_000200.1	-	297843268	297843219
Sma-1-5356	NW_003037916.1	-	870688	870639
Sma-1-5357	NW_003037978.1	+	539858	539907
Sma-1-5358	NS_000200.1	+	209807868	209807917
Sma-1-5359	NS_000200.1	-	164064099	164064050
Sma-1-5360	NW_003026610.1	+	5367	5416
Sma-1-5361	NW_003027748.1	+	1385	1434
Sma-1-5362	NW_003037873.1	+	317579	317628

Sma-1-5363	NW_003038502.1	-	184699	184650
Sma-1-5364	NW_003038502.1	-	178141	178092
Sma-1-5365	NW_003037842.1	+	82810	82859
Sma-1-5366	NW_003037887.1	-	1476832	1476783
Sma-1-5367	NW_003038019.1	+	412647	412696
Sma-1-5368	NW_003026510.1	+	85234	85283
Sma-1-5369	NS_000200.1	+	85182000	85182049
Sma-1-5370	NS_000200.1	+	119056707	119056756
Sma-1-5371	NS_000200.1	+	233729465	233729514
Sma-1-5372	NS_000200.1	+	283525415	283525464
Sma-1-5373	NS_000200.1	+	310160737	310160786
Sma-1-5374	NS_000200.1	+	365874831	365874880
Sma-1-5375	NS_000200.1	-	335183744	335183695
Sma-1-5376	NS_000200.1	-	335177186	335177137
Sma-1-5377	NS_000200.1	-	131940810	131940761
Sma-1-5378	NW_003039127.1	-	863989	863940
Sma-1-5379	NS_000200.1	-	113941488	113941439
Sma-1-5380	NW_003038118.1	-	5907	5858
Sma-1-5381	NS_000200.1	-	277570034	277569985
Sma-1-5382	NW_003020838.1	+	122	171
Sma-1-5383	NW_003022582.1	-	1539	1490
Sma-1-5384	NW_003024594.1	-	1622	1573
Sma-1-5385	NW_003037822.1	+	1267535	1267584
Sma-1-5386	NW_003037822.1	-	1264034	1263985
Sma-1-5387	NW_003037943.1	-	733436	733387
Sma-1-5388	NW_003038243.1	+	96011	96060
Sma-1-5389	NS_000200.1	+	55575541	55575590
Sma-1-5390	NS_000200.1	+	315034495	315034544
Sma-1-5391	NS_000200.1	+	364546126	364546175
Sma-1-5392	NS_000200.1	-	365225992	365225943
Sma-1-5393	NS_000200.1	-	348912918	348912869
Sma-1-5394	NS_000200.1	-	187754969	187754920
Sma-1-5395	NS_000200.1	-	55572040	55571991
Sma-1-5396	NW_003039129.1	+	392675	392724
Sma-1-5397	NS_000200.1	+	229347483	229347532
Sma-1-5398	NW_003027605.1	+	245	294
Sma-1-5399	NW_003026470.1	+	493185	493234
Sma-1-5400	NW_003037821.1	-	596573	596524
Sma-1-5401	NW_003037830.1	+	157042	157091
Sma-1-5402	NW_003037852.1	+	923632	923681
Sma-1-5403	NW_003037865.1	+	564830	564879
Sma-1-5404	NW_003037918.1	-	836110	836061
Sma-1-5405	NW_003037946.1	+	898258	898307
Sma-1-5406	NW_003037996.1	-	485745	485696
Sma-1-5407	NW_003038255.1	-	244416	244367
Sma-1-5408	NW_003038418.1	+	1037	1086
Sma-1-5409	NS_000200.1	+	68047413	68047462
Sma-1-5410	NS_000200.1	+	95315527	95315576
Sma-1-5411	NS_000200.1	+	110986749	110986798
Sma-1-5412	NS_000200.1	+	191592118	191592167
Sma-1-5413	NS_000200.1	+	219617154	219617203
Sma-1-5414	NS_000200.1	+	328865556	328865605

Sma-1-5415	NS_000200.1	+	361717485	361717534
Sma-1-5416	NS_000200.1	-	320633561	320633512
Sma-1-5417	NS_000200.1	-	220730621	220730572
Sma-1-5418	NS_000200.1	-	167403100	167403051
Sma-1-5419	NS_000200.1	-	53741057	53741008
Sma-1-5420	NW_003037979.1	-	27921	27872
Sma-1-5421	NS_000200.1	-	210088941	210088892
Sma-1-5422	NW_003037799.1	-	1529396	1529347
Sma-1-5423	NW_003037820.1	+	443320	443369
Sma-1-5424	NS_000200.1	+	52716302	52716351
Sma-1-5425	NS_000200.1	-	18519750	18519701
Sma-1-5426	NW_003037824.1	+	641603	641652
Sma-1-5427	NS_000200.1	+	57572543	57572592
Sma-1-5428	NW_003038133.1	+	50601	50650
Sma-1-5429	NS_000200.1	+	282922783	282922832
Sma-1-5430	NW_003038252.1	-	36073	36024
Sma-1-5431	NS_000200.1	-	318891661	318891612
Sma-1-5432	NW_003038156.1	+	471448	471497
Sma-1-5433	NS_000200.1	+	295203561	295203610
Sma-1-5434	NW_003020433.1	+	15	64
Sma-1-5435	NW_003038507.1	-	40472	40423
Sma-1-5436	NS_000200.1	+	355095622	355095671
Sma-1-5437	NS_000200.1	-	336709366	336709317
Sma-1-5438	NW_003026609.1	+	192756	192805
Sma-1-5439	NW_003037940.1	-	327297	327248
Sma-1-5440	NS_000200.1	+	309847748	309847797
Sma-1-5441	NS_000200.1	-	184069427	184069378
Sma-1-5442	NW_003037824.1	-	29031	28982
Sma-1-5443	NS_000200.1	-	56959971	56959922
Sma-1-5444	NW_003037821.1	+	160517	160566
Sma-1-5445	NW_003038071.1	-	164645	164596
Sma-1-5446	NS_000200.1	+	53305001	53305050
Sma-1-5447	NS_000200.1	-	264967688	264967639
Sma-1-5448	NW_003037822.1	+	1297221	1297270
Sma-1-5449	NS_000200.1	+	55605227	55605276
Sma-1-5450	NW_003037822.1	-	563151	563102
Sma-1-5451	NW_003037859.1	+	247124	247173
Sma-1-5452	NW_003038025.1	+	80921	80970
Sma-1-5453	NS_000200.1	+	102925965	102926014
Sma-1-5454	NS_000200.1	+	238939166	238939215
Sma-1-5455	NS_000200.1	-	54871157	54871108
Sma-1-5456	NW_003037822.1	+	432921	432970
Sma-1-5457	NS_000200.1	+	54740927	54740976
Sma-1-5458	NW_003037814.1	-	1679501	1679452
Sma-1-5459	NS_000200.1	-	44663361	44663312
Sma-1-5460	NW_003038514.1	-	92755	92706
Sma-1-5461	NS_000200.1	-	339717294	339717245
Sma-1-5462	NW_003037819.1	-	720465	720416
Sma-1-5463	NW_003038038.1	+	1004342	1004391
Sma-1-5464	NW_003038072.1	-	80120	80071
Sma-1-5465	NS_000200.1	+	252907735	252907784
Sma-1-5466	NS_000200.1	-	265242348	265242299

Sma-1-5467	NS_000200.1	-	51808730	51808681
Sma-1-5468	NW_003038049.1	+	204286	204335
Sma-1-5469	NS_000200.1	+	256179813	256179862
Sma-1-5470	NW_003037793.1	-	1567003	1566954
Sma-1-5471	NS_000200.1	-	1567003	1566954
Sma-1-5472	NW_003037793.1	-	388546	388497
Sma-1-5473	NS_000200.1	-	388546	388497
Sma-1-5474	NW_003037790.1	-	61768	61719
Sma-1-5475	NW_003037840.1	-	1749246	1749197
Sma-1-5476	NS_000200.1	-	381405940	381405891
Sma-1-5477	NS_000200.1	-	82372570	82372521
Sma-1-5478	NW_003037981.1	-	1054091	1054042
Sma-1-5479	NS_000200.1	-	213451959	213451910
Sma-1-5480	NW_003037907.1	+	64908	64957
Sma-1-5481	NS_000200.1	+	151469397	151469446
Sma-1-5482	NW_003026617.1	-	104457	104408
Sma-1-5483	NS_000200.1	-	319675316	319675267
Sma-1-5484	NW_003037824.1	+	612200	612249
Sma-1-5485	NS_000200.1	+	57543140	57543189
Sma-1-5486	NW_003037822.1	-	1195985	1195936
Sma-1-5487	NS_000200.1	-	55503991	55503942
Sma-1-5488	NW_003039127.1	-	257753	257704
Sma-1-5489	NS_000200.1	-	113335252	113335203
Sma-1-5490	NW_003037937.1	-	210193	210144
Sma-1-5491	NW_003038117.1	-	88100	88051
Sma-1-5492	NS_000200.1	-	277405925	277405876
Sma-1-5493	NS_000200.1	-	181410102	181410053
Sma-1-5494	NW_003037914.1	+	468760	468809
Sma-1-5495	NS_000200.1	+	160613010	160613059
Sma-1-5496	NW_003037916.1	+	1295281	1295330
Sma-1-5497	NS_000200.1	+	164488692	164488741
Sma-1-5498	NW_003037821.1	-	1037524	1037475
Sma-1-5499	NS_000200.1	-	54182008	54181959
Sma-1-5500	NW_003037916.1	-	1310814	1310765
Sma-1-5501	NS_000200.1	-	164504225	164504176
Sma-1-5502	NW_003037809.1	+	1252661	1252710
Sma-1-5503	NS_000200.1	+	34917125	34917174
Sma-1-5504	NW_003037917.1	-	1428714	1428665
Sma-1-5505	NS_000200.1	-	166465518	166465469
Sma-1-5506	NW_003026466.1	+	68045	68094
Sma-1-5507	NS_000200.1	+	215335152	215335201
Sma-1-5508	NW_003026596.1	-	183504	183455
Sma-1-5509	NS_000200.1	-	305454132	305454083
Sma-1-5510	NW_003038146.1	-	434026	433977
Sma-1-5511	NS_000200.1	-	289414696	289414647
Sma-1-5512	NW_003037830.1	+	692312	692361
Sma-1-5513	NS_000200.1	+	68582683	68582732
Sma-1-5514	NW_003026602.1	+	20689	20738
Sma-1-5515	NS_000200.1	+	306940672	306940721
Sma-1-5516	NW_003037796.1	-	402905	402856
Sma-1-5517	NS_000200.1	-	8612549	8612500
Sma-1-5518	NW_003038131.1	-	16688	16639

Sma-1-5519	NS_000200.1	-	281978532	281978483
Sma-1-5520	NW_003037912.1	+	1511453	1511502
Sma-1-5521	NS_000200.1	+	158323028	158323077
Sma-1-5522	NW_003037950.1	-	104996	104947
Sma-1-5523	NS_000200.1	-	192504104	192504055
Sma-1-5524	NW_003035873.1	+	14193	14242
Sma-1-5525	NW_003037918.1	+	2232364	2232413
Sma-1-5526	NS_000200.1	+	168799354	168799403
Sma-1-5527	NS_000200.1	+	335775195	335775244
Sma-1-5528	NW_003037794.1	+	83488	83537
Sma-1-5529	NS_000200.1	+	4263083	4263132
Sma-1-5530	NW_003037917.1	-	714097	714048
Sma-1-5531	NS_000200.1	-	165750901	165750852
Sma-1-5532	NW_003037805.1	-	1075366	1075317
Sma-1-5533	NS_000200.1	-	27633210	27633161
Sma-1-5534	NW_003037804.1	+	2718	2767
Sma-1-5535	NS_000200.1	+	25142738	25142787
Sma-1-5536	NW_003037803.1	-	902026	901977
Sma-1-5537	NS_000200.1	-	24402563	24402514
Sma-1-5538	NW_003037918.1	-	60519	60470
Sma-1-5539	NS_000200.1	-	166627509	166627460
Sma-1-5540	NW_003038832.1	+	2088	2137
Sma-1-5541	NS_000200.1	+	352368432	352368481
Sma-1-5542	NW_003037775.1	-	3442	3393
Sma-1-5543	NW_003038155.1	-	525447	525398
Sma-1-5544	NS_000200.1	-	378632608	378632559
Sma-1-5545	NS_000200.1	-	294648565	294648516
Sma-1-5546	NW_003037924.1	-	251253	251204
Sma-1-5547	NS_000200.1	-	171958211	171958162
Sma-1-5548	NW_003037800.1	-	132532	132483
Sma-1-5549	NS_000200.1	-	18761892	18761843
Sma-1-5550	NW_003037968.1	+	456090	456139
Sma-1-5551	NS_000200.1	+	200373260	200373309
Sma-1-5552	NW_003037484.1	+	1019	1068
Sma-1-5553	NS_000200.1	+	370138194	370138243
Sma-1-5554	NW_003037926.1	+	301623	301672
Sma-1-5555	NS_000200.1	+	173469893	173469942
Sma-1-5556	NW_003037936.1	-	407153	407104
Sma-1-5557	NS_000200.1	-	180846882	180846833
Sma-1-5558	NW_003038036.1	+	691138	691187
Sma-1-5559	NS_000200.1	+	249624990	249625039
Sma-1-5560	NW_003037943.1	+	1140833	1140882
Sma-1-5561	NS_000200.1	+	188162366	188162415
Sma-1-5562	NW_003037797.1	+	593105	593154
Sma-1-5563	NS_000200.1	+	11020965	11021014
Sma-1-5564	NW_003037795.1	+	1004	1053
Sma-1-5565	NS_000200.1	+	6391887	6391936
Sma-1-5566	NW_003037794.1	+	889727	889776
Sma-1-5567	NS_000200.1	+	5069322	5069371
Sma-1-5568	NW_003037937.1	-	402777	402728
Sma-1-5569	NW_003026516.1	-	373945	373896
Sma-1-5570	NS_000200.1	-	299482242	299482193

Sma-1-5571	NS_000200.1	-	181602686	181602637
Sma-1-5572	NW_003035880.1	+	323784	323833
Sma-1-5573	NS_000200.1	+	339564848	339564897
Sma-1-5574	NW_003037982.1	-	1176069	1176020
Sma-1-5575	NS_000200.1	-	214835264	214835215
Sma-1-5576	NW_003037995.1	-	365979	365930
Sma-1-5577	NS_000200.1	-	220055747	220055698
Sma-1-5578	NW_003037996.1	-	164079	164030
Sma-1-5579	NS_000200.1	-	220408955	220408906
Sma-1-5580	NW_003024939.1	-	693	644
Sma-1-5581	NW_003037809.1	-	1826255	1826206
Sma-1-5582	NW_003037848.1	-	400879	400830
Sma-1-5583	NW_003037905.1	+	665182	665231
Sma-1-5584	NS_000200.1	+	149934194	149934243
Sma-1-5585	NS_000200.1	-	355746057	355746008
Sma-1-5586	NS_000200.1	-	90093950	90093901
Sma-1-5587	NS_000200.1	-	35490719	35490670
Sma-1-5588	NW_003038033.1	+	643162	643211
Sma-1-5589	NS_000200.1	+	246623164	246623213
Sma-1-5590	NW_003037795.1	+	1731579	1731628
Sma-1-5591	NS_000200.1	+	8122462	8122511
Sma-1-5592	NW_003038033.1	-	644870	644821
Sma-1-5593	NS_000200.1	-	246624872	246624823
Sma-1-5594	NW_003037798.1	-	2326789	2326740
Sma-1-5595	NW_003037831.1	+	369159	369208
Sma-1-5596	NW_003037863.1	+	1320127	1320176
Sma-1-5597	NW_003038027.1	-	639431	639382
Sma-1-5598	NW_003038037.1	-	1653230	1653181
Sma-1-5599	NS_000200.1	+	69818461	69818510
Sma-1-5600	NS_000200.1	+	108078494	108078543
Sma-1-5601	NS_000200.1	-	251808639	251808590
Sma-1-5602	NS_000200.1	-	241010145	241010096
Sma-1-5603	NS_000200.1	-	16585947	16585898
Sma-1-5604	NW_003038002.1	+	237681	237730
Sma-1-5605	NS_000200.1	+	223357890	223357939
Sma-1-5606	NW_003032998.1	+	1156	1205
Sma-1-5607	NW_003037795.1	+	1032411	1032460
Sma-1-5608	NW_003037958.1	+	151620	151669
Sma-1-5609	NS_000200.1	+	7423294	7423343
Sma-1-5610	NS_000200.1	+	194815804	194815853
Sma-1-5611	NS_000200.1	+	326149261	326149310
Sma-1-5612	NW_003038509.1	+	322562	322611
Sma-1-5613	NS_000200.1	+	337955014	337955063
Sma-1-5614	NW_003038132.1	+	343625	343674
Sma-1-5615	NS_000200.1	+	282764095	282764144
Sma-1-5616	NW_003038037.1	-	1383033	1382984
Sma-1-5617	NS_000200.1	-	251538442	251538393
Sma-1-5618	NW_003027365.1	-	997	948
Sma-1-5619	NS_000200.1	-	356736493	356736444
Sma-1-5620	NW_003038035.1	-	182489	182440
Sma-1-5621	NS_000200.1	-	248043981	248043932
Sma-1-5622	NW_003029804.1	+	17357	17406

Sma-1-5623	NW_003033027.1	+	4260	4309
Sma-1-5624	NW_003038212.1	+	2328	2377
Sma-1-5625	NW_003037898.1	-	422859	422810
Sma-1-5626	NW_003026468.1	-	131536	131487
Sma-1-5627	NW_003037797.1	+	1028264	1028313
Sma-1-5628	NW_003037805.1	-	1182776	1182727
Sma-1-5629	NW_003037805.1	-	970601	970552
Sma-1-5630	NW_003037810.1	+	1273339	1273388
Sma-1-5631	NW_003037814.1	+	92444	92493
Sma-1-5632	NW_003037814.1	+	94667	94716
Sma-1-5633	NW_003037822.1	+	373956	374005
Sma-1-5634	NW_003037836.1	-	564533	564484
Sma-1-5635	NW_003037846.1	-	273266	273217
Sma-1-5636	NW_003037862.1	-	92435	92386
Sma-1-5637	NW_003037883.1	+	373460	373509
Sma-1-5638	NW_003038023.1	+	42624	42673
Sma-1-5639	NW_003026490.1	+	209219	209268
Sma-1-5640	NS_000200.1	+	11456124	11456173
Sma-1-5641	NS_000200.1	+	37877861	37877910
Sma-1-5642	NS_000200.1	+	43076304	43076353
Sma-1-5643	NS_000200.1	+	43078527	43078576
Sma-1-5644	NS_000200.1	+	54681962	54682011
Sma-1-5645	NS_000200.1	+	126437183	126437232
Sma-1-5646	NS_000200.1	+	237202915	237202964
Sma-1-5647	NS_000200.1	+	267840935	267840984
Sma-1-5648	NS_000200.1	+	306166219	306166268
Sma-1-5649	NS_000200.1	+	326219080	326219129
Sma-1-5650	NS_000200.1	+	375893963	375894012
Sma-1-5651	NS_000200.1	-	216325557	216325508
Sma-1-5652	NS_000200.1	-	144406681	144406632
Sma-1-5653	NS_000200.1	-	105744096	105744047
Sma-1-5654	NS_000200.1	-	87907836	87907787
Sma-1-5655	NS_000200.1	-	76121632	76121583
Sma-1-5656	NS_000200.1	-	27740620	27740571
Sma-1-5657	NS_000200.1	-	27528445	27528396
Sma-1-5658	NW_003021621.1	-	55	6
Sma-1-5659	NW_003037838.1	-	444443	444394
Sma-1-5660	NS_000200.1	-	348183556	348183507
Sma-1-5661	NS_000200.1	-	78443006	78442957
Sma-1-5662	NW_003033333.1	-	1087	1038
Sma-1-5663	NW_003038136.1	+	59438	59487
Sma-1-5664	NS_000200.1	+	284181507	284181556
Sma-1-5665	NS_000200.1	-	347999435	347999386
Sma-1-5666	NW_003037796.1	+	279159	279208
Sma-1-5667	NS_000200.1	+	8488803	8488852
Sma-1-5668	NW_003037962.1	-	298621	298572
Sma-1-5669	NS_000200.1	-	196375464	196375415
Sma-1-5670	NW_003037982.1	-	282946	282897
Sma-1-5671	NS_000200.1	-	213942141	213942092
Sma-1-5672	NW_003026485.1	+	121296	121345
Sma-1-5673	NW_003026485.1	+	142073	142122
Sma-1-5674	NS_000200.1	+	258476201	258476250

Sma-1-5675	NS_000200.1	+	258496978	258497027
Sma-1-5676	NW_003026604.1	+	110089	110138
Sma-1-5677	NS_000200.1	+	307355646	307355695
Sma-1-5678	NW_003037979.1	+	352589	352638
Sma-1-5679	NS_000200.1	+	210413609	210413658
Sma-1-5680	NW_003038028.1	+	82306	82355
Sma-1-5681	NS_000200.1	+	241389880	241389929
Sma-1-5682	NW_003038033.1	+	205578	205627
Sma-1-5683	NS_000200.1	+	246185580	246185629
Sma-1-5684	NW_003037914.1	-	1710319	1710270
Sma-1-5685	NS_000200.1	-	161854569	161854520
Sma-1-5686	NW_003038237.1	+	19564	19613
Sma-1-5687	NS_000200.1	+	313459483	313459532
Sma-1-5688	NW_003038037.1	+	1663193	1663242
Sma-1-5689	NS_000200.1	+	251818602	251818651
Sma-1-5690	NW_003024268.1	-	138	89
Sma-1-5691	NW_003037170.1	+	4325	4374
Sma-1-5692	NW_003026458.1	+	311601	311650
Sma-1-5693	NW_003037794.1	-	886440	886391
Sma-1-5694	NW_003037797.1	+	1382034	1382083
Sma-1-5695	NW_003037797.1	+	2507803	2507852
Sma-1-5696	NW_003037800.1	+	295289	295338
Sma-1-5697	NW_003037807.1	-	111052	111003
Sma-1-5698	NW_003037818.1	+	1122122	1122171
Sma-1-5699	NW_003037837.1	+	459204	459253
Sma-1-5700	NW_003037838.1	+	850420	850469
Sma-1-5701	NW_003037838.1	-	303752	303703
Sma-1-5702	NW_003037914.1	+	1712418	1712467
Sma-1-5703	NW_003037938.1	+	372893	372942
Sma-1-5704	NW_003037938.1	+	691643	691692
Sma-1-5705	NW_003037955.1	+	98597	98646
Sma-1-5706	NW_003037956.1	+	5919	5968
Sma-1-5707	NW_003037964.1	-	332610	332561
Sma-1-5708	NW_003037974.1	+	235606	235655
Sma-1-5709	NW_003038135.1	+	93888	93937
Sma-1-5710	NS_000200.1	+	11809894	11809943
Sma-1-5711	NS_000200.1	+	12935663	12935712
Sma-1-5712	NS_000200.1	+	18924649	18924698
Sma-1-5713	NS_000200.1	+	49837529	49837578
Sma-1-5714	NS_000200.1	+	77265062	77265111
Sma-1-5715	NS_000200.1	+	78848983	78849032
Sma-1-5716	NS_000200.1	+	102179831	102179880
Sma-1-5717	NS_000200.1	+	161856668	161856717
Sma-1-5718	NS_000200.1	+	182410006	182410055
Sma-1-5719	NS_000200.1	+	182728756	182728805
Sma-1-5720	NS_000200.1	+	193725331	193725380
Sma-1-5721	NS_000200.1	+	194062374	194062423
Sma-1-5722	NS_000200.1	+	204910912	204910961
Sma-1-5723	NS_000200.1	+	283966227	283966276
Sma-1-5724	NS_000200.1	+	362451922	362451971
Sma-1-5725	NS_000200.1	-	348669642	348669593
Sma-1-5726	NS_000200.1	-	197934132	197934083

Sma-1-5727	NS_000200.1	-	78302315	78302266
Sma-1-5728	NS_000200.1	-	30372568	30372519
Sma-1-5729	NS_000200.1	-	5066035	5065986
Sma-1-5730	NW_003038120.1	-	179086	179037
Sma-1-5731	NS_000200.1	-	278364309	278364260
Sma-1-5732	NW_003038221.1	+	136866	136915
Sma-1-5733	NS_000200.1	+	308778130	308778179
Sma-1-5734	NW_003026488.1	-	53182	53133
Sma-1-5735	NS_000200.1	-	263559676	263559627
Sma-1-5736	NW_003027905.1	+	46	95
Sma-1-5737	NS_000200.1	+	366129163	366129212
Sma-1-5738	NW_003037938.1	-	662613	662564
Sma-1-5739	NS_000200.1	-	182699726	182699677
Sma-1-5740	NW_003037839.1	-	1358863	1358814
Sma-1-5741	NW_003038060.1	+	94056	94105
Sma-1-5742	NW_003038060.1	-	93229	93180
Sma-1-5743	NW_003038076.1	-	272122	272073
Sma-1-5744	NS_000200.1	+	260659181	260659230
Sma-1-5745	NS_000200.1	-	266489296	266489247
Sma-1-5746	NS_000200.1	-	260658354	260658305
Sma-1-5747	NS_000200.1	-	80317013	80316964
Sma-1-5748	NW_003037800.1	-	1309377	1309328
Sma-1-5749	NS_000200.1	-	19938737	19938688
Sma-1-5750	NW_003037881.1	-	677105	677056
Sma-1-5751	NW_003037886.1	+	832237	832286
Sma-1-5752	NW_003038036.1	+	225994	226043
Sma-1-5753	NW_003038084.1	+	289542	289591
Sma-1-5754	NS_000200.1	+	130087804	130087853
Sma-1-5755	NS_000200.1	+	249159846	249159895
Sma-1-5756	NS_000200.1	+	271304786	271304835
Sma-1-5757	NS_000200.1	-	125108896	125108847
Sma-1-5758	NW_003038512.1	-	262012	261963
Sma-1-5759	NS_000200.1	-	338818709	338818660
Sma-1-5760	NW_003027354.1	+	353	402
Sma-1-5761	NW_003038258.1	-	243905	243856
Sma-1-5762	NW_003037904.1	-	547389	547340
Sma-1-5763	NW_003037969.1	-	364524	364475
Sma-1-5764	NS_000200.1	+	356717306	356717355
Sma-1-5765	NS_000200.1	-	322782415	322782366
Sma-1-5766	NS_000200.1	-	200970651	200970602
Sma-1-5767	NS_000200.1	-	149261888	149261839
Sma-1-5768	NW_003037799.1	-	1542994	1542945
Sma-1-5769	NS_000200.1	-	18533348	18533299
Sma-1-5770	NW_003035578.1	-	2657	2608
Sma-1-5771	NW_003038005.1	+	149743	149792
Sma-1-5772	NS_000200.1	+	224555697	224555746
Sma-1-5773	NS_000200.1	-	329027988	329027939
Sma-1-5774	NW_003037793.1	-	2802570	2802521
Sma-1-5775	NS_000200.1	-	2802570	2802521
Sma-1-5776	NW_003037797.1	-	1011217	1011168
Sma-1-5777	NS_000200.1	-	11439077	11439028
Sma-1-5778	NW_003038163.1	+	251269	251318

Sma-1-5779	NS_000200.1	+	298651549	298651598
Sma-1-5780	NW_003026619.1	+	64844	64893
Sma-1-5781	NW_003037862.1	-	45468	45419
Sma-1-5782	NS_000200.1	+	322255588	322255637
Sma-1-5783	NS_000200.1	-	105697129	105697080
Sma-1-5784	NW_003037931.1	+	597660	597709
Sma-1-5785	NS_000200.1	+	177430327	177430376
Sma-1-5786	NW_003037933.1	+	267989	268038
Sma-1-5787	NS_000200.1	+	178681161	178681210
Sma-1-5788	NW_003037795.1	-	1557173	1557124
Sma-1-5789	NS_000200.1	-	7948056	7948007
Sma-1-5790	NW_003037936.1	+	378087	378136
Sma-1-5791	NS_000200.1	+	180817816	180817865
Sma-1-5792	NW_003037793.1	-	3321378	3321329
Sma-1-5793	NS_000200.1	-	3321378	3321329
Sma-1-5794	NW_003035589.1	+	8532	8581
Sma-1-5795	NS_000200.1	+	329258005	329258054
Sma-1-5796	NW_003037808.1	+	250718	250767
Sma-1-5797	NW_003037881.1	+	454022	454071
Sma-1-5798	NS_000200.1	+	32037355	32037404
Sma-1-5799	NS_000200.1	+	124885813	124885862
Sma-1-5800	NW_003037791.1	+	400187	400236
Sma-1-5801	NW_003037797.1	-	960810	960761
Sma-1-5802	NS_000200.1	+	382105615	382105664
Sma-1-5803	NS_000200.1	-	11388670	11388621
Sma-1-5804	NW_003037940.1	+	563393	563442
Sma-1-5805	NS_000200.1	+	184305523	184305572
Sma-1-5806	NW_003037944.1	+	298039	298088
Sma-1-5807	NS_000200.1	+	188519392	188519441
Sma-1-5808	NW_003037974.1	-	639645	639596
Sma-1-5809	NW_003038240.1	+	56005	56054
Sma-1-5810	NS_000200.1	+	314194936	314194985
Sma-1-5811	NS_000200.1	-	205314951	205314902
Sma-1-5812	NW_003026596.1	+	179043	179092
Sma-1-5813	NS_000200.1	+	305449671	305449720
Sma-1-5814	NW_003026470.1	+	322476	322525
Sma-1-5815	NS_000200.1	+	219446445	219446494
Sma-1-5816	NW_003038928.1	-	837	788
Sma-1-5817	NS_000200.1	-	355820717	355820668
Sma-1-5818	NW_003020782.1	-	185	136
Sma-1-5819	NW_003026462.1	+	193838	193887
Sma-1-5820	NS_000200.1	+	196861341	196861390
Sma-1-5821	NS_000200.1	-	364447332	364447283
Sma-1-5822	NW_003026456.1	-	589477	589428
Sma-1-5823	NW_003038025.1	+	535419	535468
Sma-1-5824	NS_000200.1	+	239393664	239393713
Sma-1-5825	NS_000200.1	-	58512613	58512564
Sma-1-5826	NW_003038012.1	+	22089	22138
Sma-1-5827	NS_000200.1	+	228346028	228346077
Sma-1-5828	NW_003038068.1	-	180861	180812
Sma-1-5829	NS_000200.1	-	263248069	263248020
Sma-1-5830	NW_003037819.1	-	493155	493106

Sma-1-5831	NS_000200.1	-	51581420	51581371
Sma-1-5832	NW_003038006.1	+	234439	234488
Sma-1-5833	NS_000200.1	+	225118923	225118972
Sma-1-5834	NW_003038016.1	+	124373	124422
Sma-1-5835	NS_000200.1	+	231240910	231240959
Sma-1-5836	NW_003029805.1	-	4670	4621
Sma-1-5837	NW_003038009.1	+	148010	148059
Sma-1-5838	NW_003037824.1	+	766402	766451
Sma-1-5839	NW_003037855.1	-	333696	333647
Sma-1-5840	NW_003037866.1	+	626148	626197
Sma-1-5841	NW_003037879.1	+	654531	654580
Sma-1-5842	NW_003037974.1	-	110247	110198
Sma-1-5843	NW_003038017.1	-	281520	281471
Sma-1-5844	NW_003038030.1	+	444771	444820
Sma-1-5845	NW_003038033.1	-	711833	711784
Sma-1-5846	NW_003038118.1	-	45075	45026
Sma-1-5847	NW_003038151.1	+	465941	465990
Sma-1-5848	NW_003038160.1	-	520851	520802
Sma-1-5849	NS_000200.1	+	57697342	57697391
Sma-1-5850	NS_000200.1	+	112113947	112113996
Sma-1-5851	NS_000200.1	+	123656787	123656836
Sma-1-5852	NS_000200.1	+	226522073	226522122
Sma-1-5853	NS_000200.1	+	243580638	243580687
Sma-1-5854	NS_000200.1	+	292373184	292373233
Sma-1-5855	NS_000200.1	-	375910934	375910885
Sma-1-5856	NS_000200.1	-	297272782	297272733
Sma-1-5857	NS_000200.1	-	277609202	277609153
Sma-1-5858	NS_000200.1	-	246691835	246691786
Sma-1-5859	NS_000200.1	-	232047551	232047502
Sma-1-5860	NS_000200.1	-	204785553	204785504
Sma-1-5861	NS_000200.1	-	99198446	99198397
Sma-1-5862	NW_003037943.1	-	263173	263124
Sma-1-5863	NS_000200.1	-	187284706	187284657
Sma-1-5864	NW_003037813.1	+	1223323	1223372
Sma-1-5865	NW_003038042.1	+	217233	217282
Sma-1-5866	NS_000200.1	+	42210186	42210235
Sma-1-5867	NS_000200.1	+	254162832	254162881
Sma-1-5868	NW_003030794.1	-	91	42
Sma-1-5869	NS_000200.1	-	366923227	366923178
Sma-1-5870	NW_003026471.1	-	122215	122166
Sma-1-5871	NS_000200.1	-	222234448	222234399
Sma-1-5872	NW_003038151.1	+	50555	50604
Sma-1-5873	NS_000200.1	+	291957798	291957847
Sma-1-5874	NW_003038242.1	-	134692	134643
Sma-1-5875	NS_000200.1	-	314833838	314833789
Sma-1-5876	NW_003038509.1	+	171969	172018
Sma-1-5877	NS_000200.1	+	337804421	337804470
Sma-1-5878	NW_003037824.1	-	53523	53474
Sma-1-5879	NS_000200.1	-	56984463	56984414
Sma-1-5880	NW_003037961.1	-	5820	5771
Sma-1-5881	NS_000200.1	-	195630062	195630013
Sma-1-5882	NW_003029591.1	-	668	619

Sma-1-5883	NS_000200.1	-	366206010	366205961
Sma-1-5884	NW_003026588.1	+	27324	27373
Sma-1-5885	NS_000200.1	+	303876467	303876516
Sma-1-5886	NW_003037831.1	+	598889	598938
Sma-1-5887	NW_003037838.1	-	891474	891425
Sma-1-5888	NS_000200.1	+	70048191	70048240
Sma-1-5889	NS_000200.1	-	78890037	78889988
Sma-1-5890	NW_003037962.1	+	165890	165939
Sma-1-5891	NS_000200.1	+	196242733	196242782
Sma-1-5892	NW_003026483.1	+	192287	192336
Sma-1-5893	NS_000200.1	+	256706811	256706860
Sma-1-5894	NW_003037755.1	-	31159	31110
Sma-1-5895	NW_003026465.1	+	558650	558699
Sma-1-5896	NW_003037814.1	-	1603172	1603123
Sma-1-5897	NW_003037821.1	+	1110497	1110546
Sma-1-5898	NW_003037823.1	-	250562	250513
Sma-1-5899	NW_003037841.1	+	1100036	1100085
Sma-1-5900	NW_003037914.1	+	1673378	1673427
Sma-1-5901	NW_003037925.1	+	451886	451935
Sma-1-5902	NS_000200.1	+	54254981	54255030
Sma-1-5903	NS_000200.1	+	83711217	83711266
Sma-1-5904	NS_000200.1	+	161817628	161817677
Sma-1-5905	NS_000200.1	+	172853889	172853938
Sma-1-5906	NS_000200.1	+	207951244	207951293
Sma-1-5907	NS_000200.1	-	377437405	377437356
Sma-1-5908	NS_000200.1	-	56167181	56167132
Sma-1-5909	NS_000200.1	-	44587032	44586983
Sma-1-5910	NW_003029783.1	-	2113	2064
Sma-1-5911	NS_000200.1	-	375588599	375588550
Sma-1-5912	NW_003026616.1	+	144281	144330
Sma-1-5913	NW_003037809.1	-	1432620	1432571
Sma-1-5914	NS_000200.1	+	317500880	317500929
Sma-1-5915	NS_000200.1	-	35097084	35097035
Sma-1-5916	NW_003038258.1	+	159790	159839
Sma-1-5917	NS_000200.1	+	322698300	322698349
Sma-1-5918	NW_003037798.1	+	2398500	2398549
Sma-1-5919	NW_003037830.1	-	484800	484751
Sma-1-5920	NW_003037881.1	-	638213	638164
Sma-1-5921	NS_000200.1	+	16657658	16657707
Sma-1-5922	NS_000200.1	-	125070004	125069955
Sma-1-5923	NS_000200.1	-	68375171	68375122
Sma-1-5924	NW_003026456.1	-	532950	532901
Sma-1-5925	NS_000200.1	-	58456086	58456037
Sma-1-5926	NW_003038037.1	+	1505776	1505825
Sma-1-5927	NS_000200.1	+	251661185	251661234
Sma-1-5928	NW_003038187.1	+	137210	137259
Sma-1-5929	NS_000200.1	+	302089068	302089117
Sma-1-5930	NW_003038250.1	-	131659	131610
Sma-1-5931	NW_003037831.1	-	658394	658345
Sma-1-5932	NS_000200.1	-	318184336	318184287
Sma-1-5933	NS_000200.1	-	70107696	70107647
Sma-1-5934	NW_003028564.1	-	126	77

Sma-1-5935	NW_003026619.1	-	306112	306063
Sma-1-5936	NW_003037814.1	+	100690	100739
Sma-1-5937	NW_003037837.1	-	440417	440368
Sma-1-5938	NW_003037884.1	-	1255104	1255055
Sma-1-5939	NW_003037888.1	+	986195	986244
Sma-1-5940	NW_003037914.1	+	13357	13406
Sma-1-5941	NW_003037943.1	-	414321	414272
Sma-1-5942	NW_003037956.1	+	327956	328005
Sma-1-5943	NW_003037963.1	+	99758	99807
Sma-1-5944	NW_003038021.1	-	788060	788011
Sma-1-5945	NW_003038128.1	-	74707	74658
Sma-1-5946	NS_000200.1	+	43084550	43084599
Sma-1-5947	NS_000200.1	+	133194687	133194736
Sma-1-5948	NS_000200.1	+	160157607	160157656
Sma-1-5949	NS_000200.1	+	194384411	194384460
Sma-1-5950	NS_000200.1	+	197377970	197378019
Sma-1-5951	NS_000200.1	-	374644719	374644670
Sma-1-5952	NS_000200.1	-	322496856	322496807
Sma-1-5953	NS_000200.1	-	280792995	280792946
Sma-1-5954	NS_000200.1	-	235616635	235616586
Sma-1-5955	NS_000200.1	-	187435854	187435805
Sma-1-5956	NS_000200.1	-	128034769	128034720
Sma-1-5957	NS_000200.1	-	77246275	77246226
Sma-1-5958	NW_003022492.1	+	1424	1473
Sma-1-5959	NW_003026468.1	-	52826	52777
Sma-1-5960	NW_003038024.1	+	125879	125928
Sma-1-5961	NS_000200.1	+	238022908	238022957
Sma-1-5962	NS_000200.1	+	365045836	365045885
Sma-1-5963	NS_000200.1	-	216246847	216246798
Sma-1-5964	NW_003038014.1	-	184021	183972
Sma-1-5965	NS_000200.1	-	229924710	229924661
Sma-1-5966	NW_003037793.1	+	247954	248003
Sma-1-5967	NW_003037882.1	-	624700	624651
Sma-1-5968	NW_003037938.1	-	602016	601967
Sma-1-5969	NW_003038153.1	-	295641	295592
Sma-1-5970	NS_000200.1	+	247954	248003
Sma-1-5971	NS_000200.1	-	293357690	293357641
Sma-1-5972	NS_000200.1	-	182639129	182639080
Sma-1-5973	NS_000200.1	-	125916374	125916325
Sma-1-5974	NW_003037787.1	+	64038	64087
Sma-1-5975	NS_000200.1	+	380312207	380312256
Sma-1-5976	NW_003037807.1	+	1153606	1153655
Sma-1-5977	NS_000200.1	+	31415122	31415171
Sma-1-5978	NW_003037852.1	-	525278	525229
Sma-1-5979	NS_000200.1	-	94917173	94917124
Sma-1-5980	NW_003037827.1	+	1188787	1188836
Sma-1-5981	NS_000200.1	+	63810928	63810977
Sma-1-5982	NW_003037796.1	+	46970	47019
Sma-1-5983	NS_000200.1	+	8256614	8256663
Sma-1-5984	NW_003024301.1	-	2426	2377
Sma-1-5985	NS_000200.1	-	365474777	365474728
Sma-1-5986	NW_003035880.1	-	289103	289054

Sma-1-5987	NS_000200.1	-	339530167	339530118
Sma-1-5988	NW_003037832.1	-	550968	550919
Sma-1-5989	NS_000200.1	-	71760399	71760350
Sma-1-5990	NW_003037795.1	+	1339339	1339388
Sma-1-5991	NS_000200.1	+	7730222	7730271
Sma-1-5992	NW_003037986.1	+	93995	94044
Sma-1-5993	NS_000200.1	+	215510747	215510796
Sma-1-5994	NW_003026456.1	-	321515	321466
Sma-1-5995	NS_000200.1	-	58244651	58244602
Sma-1-5996	NW_003038147.1	-	364000	363951
Sma-1-5997	NS_000200.1	-	289891743	289891694
Sma-1-5998	NW_003038244.1	-	436559	436510
Sma-1-5999	NS_000200.1	-	315581764	315581715
Sma-1-6000	NW_003037844.1	+	406872	406921
Sma-1-6001	NS_000200.1	+	86590318	86590367
Sma-1-6002	NW_003037902.1	+	276733	276782
Sma-1-6003	NW_003037913.1	+	682315	682364
Sma-1-6004	NS_000200.1	+	147031967	147032016
Sma-1-6005	NS_000200.1	+	159246066	159246115
Sma-1-6006	NW_003037804.1	-	986982	986933
Sma-1-6007	NS_000200.1	-	26127002	26126953
Sma-1-6008	NW_003037798.1	+	1948184	1948233
Sma-1-6009	NS_000200.1	+	16207342	16207391
Sma-1-6010	NW_003038141.1	+	265095	265144
Sma-1-6011	NS_000200.1	+	287155074	287155123
Sma-1-6012	NW_003023322.1	-	1251	1202
Sma-1-6013	NS_000200.1	-	372961495	372961446
Sma-1-6014	NW_003038232.1	-	18320	18271
Sma-1-6015	NS_000200.1	-	311982111	311982062
Sma-1-6016	NW_003024936.1	-	647	598
Sma-1-6017	NW_003030988.1	+	412	461
Sma-1-6018	NW_003026471.1	-	108253	108204
Sma-1-6019	NW_003037928.1	+	487161	487210
Sma-1-6020	NS_000200.1	+	174811114	174811163
Sma-1-6021	NS_000200.1	+	370671035	370671084
Sma-1-6022	NS_000200.1	-	355741168	355741119
Sma-1-6023	NS_000200.1	-	222220486	222220437
Sma-1-6024	NW_003037848.1	+	521004	521053
Sma-1-6025	NS_000200.1	+	90214075	90214124
Sma-1-6026	NW_003037905.1	-	1036027	1035978
Sma-1-6027	NS_000200.1	-	150305039	150304990
Sma-1-6028	NW_003038234.1	+	101312	101361
Sma-1-6029	NS_000200.1	+	312938081	312938130
Sma-1-6030	NW_003026617.1	-	184119	184070
Sma-1-6031	NW_003037793.1	+	682993	683042
Sma-1-6032	NS_000200.1	+	682993	683042
Sma-1-6033	NS_000200.1	-	319754978	319754929
Sma-1-6034	NW_003038138.1	-	93932	93883
Sma-1-6035	NS_000200.1	-	285649247	285649198
Sma-1-6036	NW_003026513.1	-	68612	68563
Sma-1-6037	NS_000200.1	-	285483148	285483099
Sma-1-6038	NW_003037800.1	-	52071	52022

Sma-1-6039	NS_000200.1	-	18681431	18681382
Sma-1-6040	NW_003037941.1	+	483388	483437
Sma-1-6041	NS_000200.1	+	185217862	185217911
Sma-1-6042	NW_003027567.1	-	372	323
Sma-1-6043	NW_003037940.1	+	973321	973370
Sma-1-6044	NS_000200.1	+	184715451	184715500
Sma-1-6045	NS_000200.1	-	361637035	361636986
Sma-1-6046	NW_003026458.1	-	332457	332408
Sma-1-6047	NS_000200.1	-	102200687	102200638
Sma-1-6048	NW_003037824.1	-	367164	367115
Sma-1-6049	NS_000200.1	-	57298104	57298055
Sma-1-6050	NW_003038011.1	+	599526	599575
Sma-1-6051	NS_000200.1	+	228127176	228127225
Sma-1-6052	NW_003038261.1	-	208313	208264
Sma-1-6053	NS_000200.1	-	323821964	323821915
Sma-1-6054	NW_003037792.1	-	161498	161449
Sma-1-6055	NS_000200.1	-	382598842	382598793
Sma-1-6056	NW_003035880.1	-	347065	347016
Sma-1-6057	NW_003026458.1	-	705470	705421
Sma-1-6058	NS_000200.1	-	339588129	339588080
Sma-1-6059	NS_000200.1	-	102573700	102573651
Sma-1-6060	NW_003037714.1	+	15866	15915
Sma-1-6061	NS_000200.1	+	376047442	376047491
Sma-1-6062	NW_003037909.1	+	881834	881883
Sma-1-6063	NS_000200.1	+	154423483	154423532
Sma-1-6064	NW_003037818.1	-	2127727	2127678
Sma-1-6065	NS_000200.1	-	50843134	50843085
Sma-1-6066	NW_003037793.1	+	3428365	3428414
Sma-1-6067	NS_000200.1	+	3428365	3428414
Sma-1-6068	NW_003036447.1	-	986	937
Sma-1-6069	NS_000200.1	-	354579817	354579768
Sma-1-6070	NW_003035881.1	+	393414	393463
Sma-1-6071	NS_000200.1	+	341457619	341457668
Sma-1-6072	NW_003038010.1	-	376887	376838
Sma-1-6073	NS_000200.1	-	227195110	227195061
Sma-1-6074	NW_003031336.1	-	871	822
Sma-1-6075	NS_000200.1	-	345774841	345774792
Sma-1-6076	NW_003037953.1	-	113818	113769
Sma-1-6077	NS_000200.1	-	193026869	193026820
Sma-1-6078	NW_003027652.1	-	986	937
Sma-1-6079	NS_000200.1	-	361804786	361804737
Sma-1-6080	NW_003038261.1	+	683982	684031
Sma-1-6081	NS_000200.1	+	324297633	324297682
Sma-1-6082	NW_003038025.1	+	573372	573421
Sma-1-6083	NS_000200.1	+	239431617	239431666
Sma-1-6084	NW_003037980.1	-	103323	103274
Sma-1-6085	NS_000200.1	-	211064185	211064136
Sma-1-6086	NW_003037994.1	+	247873	247922
Sma-1-6087	NW_003038024.1	-	727081	727032
Sma-1-6088	NS_000200.1	+	218779687	218779736
Sma-1-6089	NS_000200.1	-	238624110	238624061
Sma-1-6090	NW_003038244.1	+	325207	325256

Sma-1-6091	NW_003037798.1	+	2406972	2407021
Sma-1-6092	NW_003037810.1	+	751811	751860
Sma-1-6093	NW_003037848.1	+	596139	596188
Sma-1-6094	NW_003037848.1	-	595723	595674
Sma-1-6095	NW_003037868.1	+	121604	121653
Sma-1-6096	NW_003037909.1	-	234017	233968
Sma-1-6097	NW_003037913.1	+	1022780	1022829
Sma-1-6098	NW_003037955.1	-	114935	114886
Sma-1-6099	NW_003037997.1	+	452305	452354
Sma-1-6100	NS_000200.1	+	16666130	16666179
Sma-1-6101	NS_000200.1	+	37356333	37356382
Sma-1-6102	NS_000200.1	+	90289210	90289259
Sma-1-6103	NS_000200.1	+	116093316	116093365
Sma-1-6104	NS_000200.1	+	159586531	159586580
Sma-1-6105	NS_000200.1	+	221263747	221263796
Sma-1-6106	NS_000200.1	+	315470412	315470461
Sma-1-6107	NS_000200.1	-	193741669	193741620
Sma-1-6108	NS_000200.1	-	153775666	153775617
Sma-1-6109	NS_000200.1	-	90288794	90288745
Sma-1-6110	NW_003038073.1	-	230484	230435
Sma-1-6111	NS_000200.1	-	265752917	265752868
Sma-1-6112	NW_003026472.1	+	33784	33833
Sma-1-6113	NS_000200.1	+	236445776	236445825
Sma-1-6114	NW_003037946.1	-	1142473	1142424
Sma-1-6115	NW_003038023.1	+	349775	349824
Sma-1-6116	NS_000200.1	+	237510066	237510115
Sma-1-6117	NS_000200.1	-	191836333	191836284
Sma-1-6118	NW_003026517.1	+	634049	634098
Sma-1-6119	NS_000200.1	+	300240058	300240107
Sma-1-6120	NW_003038120.1	-	205676	205627
Sma-1-6121	NS_000200.1	-	278390899	278390850
Sma-1-6122	NW_003020303.1	-	1463	1414
Sma-1-6123	NW_003038064.1	-	92274	92225
Sma-1-6124	NW_003038086.1	-	377071	377022
Sma-1-6125	NS_000200.1	-	371375853	371375804
Sma-1-6126	NS_000200.1	-	272625664	272625615
Sma-1-6127	NS_000200.1	-	261928851	261928802
Sma-1-6128	NW_003037798.1	-	1867674	1867625
Sma-1-6129	NW_003037848.1	-	570008	569959
Sma-1-6130	NS_000200.1	-	90263079	90263030
Sma-1-6131	NS_000200.1	-	16126832	16126783
Sma-1-6132	NW_003038053.1	+	63938	63987
Sma-1-6133	NS_000200.1	+	257424033	257424082
Sma-1-6134	NW_003037868.1	+	27091	27139
Sma-1-6135	NS_000200.1	+	115998803	115998851
Sma-1-6136	NW_003038033.1	+	840428	840477
Sma-1-6137	NS_000200.1	+	246820430	246820479
Sma-1-6138	NW_003037824.1	+	346196	346245
Sma-1-6139	NS_000200.1	+	57277136	57277185
Sma-1-6140	NW_003038215.1	+	41563	41612
Sma-1-6141	NS_000200.1	+	306654599	306654648
Sma-1-6142	NW_003037838.1	-	508305	508256

Sma-1-6143	NS_000200.1	-	78506868	78506819
Sma-1-6144	NW_003038132.1	+	328881	328930
Sma-1-6145	NS_000200.1	+	282749351	282749400
Sma-1-6146	NW_003038038.1	+	310502	310551
Sma-1-6147	NS_000200.1	+	252213895	252213944
Sma-1-6148	NW_003037822.1	-	318660	318611
Sma-1-6149	NW_003037980.1	+	1157399	1157448
Sma-1-6150	NS_000200.1	+	212118261	212118310
Sma-1-6151	NS_000200.1	-	54626666	54626617
Sma-1-6152	NW_003037992.1	+	219108	219157
Sma-1-6153	NS_000200.1	+	218060589	218060638
Sma-1-6154	NW_003038014.1	+	390785	390834
Sma-1-6155	NS_000200.1	+	230131474	230131523
Sma-1-6156	NW_003037853.1	+	649605	649654
Sma-1-6157	NW_003037942.1	+	428221	428270
Sma-1-6158	NS_000200.1	+	96141656	96141705
Sma-1-6159	NS_000200.1	+	186188129	186188178
Sma-1-6160	NW_003038010.1	-	455496	455447
Sma-1-6161	NS_000200.1	-	227273719	227273670
Sma-1-6162	NW_003037879.1	-	247850	247801
Sma-1-6163	NS_000200.1	-	123250106	123250057
Sma-1-6164	NW_003038114.1	-	138227	138178
Sma-1-6165	NS_000200.1	-	276864132	276864083
Sma-1-6166	NW_003037825.1	-	815311	815262
Sma-1-6167	NS_000200.1	-	59945093	59945044
Sma-1-6168	NW_003038005.1	-	455564	455515
Sma-1-6169	NS_000200.1	-	224861518	224861469
Sma-1-6170	NW_003038023.1	+	100314	100363
Sma-1-6171	NS_000200.1	+	237260605	237260654
Sma-1-6172	NW_003025689.1	+	663	712
Sma-1-6173	NW_003026472.1	-	29670	29621
Sma-1-6174	NW_003026460.1	-	303644	303595
Sma-1-6175	NW_003037818.1	+	383566	383615
Sma-1-6176	NW_003037822.1	+	716165	716214
Sma-1-6177	NW_003037822.1	-	713732	713683
Sma-1-6178	NW_003037845.1	+	559532	559581
Sma-1-6179	NW_003037892.1	+	294879	294928
Sma-1-6180	NW_003037909.1	-	374838	374789
Sma-1-6181	NW_003037979.1	+	708164	708213
Sma-1-6182	NW_003038257.1	-	251339	251290
Sma-1-6183	NW_003026516.1	+	326274	326323
Sma-1-6184	NS_000200.1	+	49098973	49099022
Sma-1-6185	NS_000200.1	+	55024171	55024220
Sma-1-6186	NS_000200.1	+	87561435	87561484
Sma-1-6187	NS_000200.1	+	141018106	141018155
Sma-1-6188	NS_000200.1	+	210769184	210769233
Sma-1-6189	NS_000200.1	+	299434571	299434620
Sma-1-6190	NS_000200.1	+	356364622	356364671
Sma-1-6191	NS_000200.1	-	321425667	321425618
Sma-1-6192	NS_000200.1	-	236441662	236441613
Sma-1-6193	NS_000200.1	-	170825850	170825801
Sma-1-6194	NS_000200.1	-	153916487	153916438

Sma-1-6195	NS_000200.1	-	55021738	55021689
Sma-1-6196	NW_003037830.1	-	221006	220956
Sma-1-6197	NS_000200.1	-	68111377	68111327
Sma-1-6198	NW_003026615.1	-	196023	195974
Sma-1-6199	NW_003037953.1	-	227011	226962
Sma-1-6200	NW_003038153.1	-	602933	602884
Sma-1-6201	NS_000200.1	-	317190464	317190415
Sma-1-6202	NS_000200.1	-	293664982	293664933
Sma-1-6203	NS_000200.1	-	193140062	193140013
Sma-1-6204	NW_003037873.1	+	32849	32898
Sma-1-6205	NW_003037831.1	-	1106560	1106511
Sma-1-6206	NW_003037890.1	-	186762	186713
Sma-1-6207	NW_003037891.1	+	1844226	1844275
Sma-1-6208	NW_003037919.1	-	91937	91888
Sma-1-6209	NW_003038159.1	-	152447	152398
Sma-1-6210	NS_000200.1	+	118771977	118772026
Sma-1-6211	NS_000200.1	+	139725504	139725553
Sma-1-6212	NS_000200.1	-	296540468	296540419
Sma-1-6213	NS_000200.1	-	168949866	168949817
Sma-1-6214	NS_000200.1	-	135870123	135870074
Sma-1-6215	NS_000200.1	-	70555862	70555813
Sma-1-6216	NW_003037861.1	-	866820	866771
Sma-1-6217	NS_000200.1	-	105643274	105643225
Sma-1-6218	NW_003037854.1	+	907587	907636
Sma-1-6219	NS_000200.1	+	97812008	97812057
Sma-1-6220	NW_003038212.1	-	11039	10990
Sma-1-6221	NS_000200.1	-	306174930	306174881
Sma-1-6222	NW_003029612.1	+	239	288
Sma-1-6223	NS_000200.1	+	366251356	366251405
Sma-1-6224	NW_003037844.1	+	30265	30314
Sma-1-6225	NS_000200.1	+	86213711	86213760
Sma-1-6226	NW_003037895.1	+	282999	283048
Sma-1-6227	NS_000200.1	+	142729201	142729250
Sma-1-6228	NW_003037838.1	-	829056	829007
Sma-1-6229	NS_000200.1	-	78827619	78827570
Sma-1-6230	NW_003038489.1	+	115254	115303
Sma-1-6231	NS_000200.1	+	333827537	333827586
Sma-1-6232	NW_003038086.1	-	413649	413600
Sma-1-6233	NS_000200.1	-	272662242	272662193
Sma-1-6234	NW_003037916.1	+	895808	895857
Sma-1-6235	NS_000200.1	+	164089219	164089268
Sma-1-6236	NW_003037943.1	+	742588	742637
Sma-1-6237	NS_000200.1	+	187764121	187764170
Sma-1-6238	NW_003038456.1	-	54402	54353
Sma-1-6239	NS_000200.1	-	330924666	330924617
Sma-1-6240	NW_003026618.1	+	83708	83757
Sma-1-6241	NS_000200.1	+	321838710	321838759
Sma-1-6242	NW_003029845.1	+	58	107
Sma-1-6243	NW_003038504.1	-	871	822
Sma-1-6244	NW_003037927.1	-	104878	104829
Sma-1-6245	NW_003026619.1	+	72010	72059
Sma-1-6246	NS_000200.1	+	322262754	322262803

Sma-1-6247	NS_000200.1	+	376401042	376401091
Sma-1-6248	NS_000200.1	-	335849403	335849354
Sma-1-6249	NS_000200.1	-	174082241	174082192
Sma-1-6250	NW_003038153.1	-	698066	698017
Sma-1-6251	NS_000200.1	-	293760115	293760066
Sma-1-6252	NW_003032147.1	-	5723	5674
Sma-1-6253	NW_003038443.1	-	25035	24986
Sma-1-6254	NW_003038002.1	+	37618	37667
Sma-1-6255	NW_003037813.1	-	1089086	1089037
Sma-1-6256	NW_003037846.1	+	971031	971080
Sma-1-6257	NW_003037879.1	-	667768	667719
Sma-1-6258	NW_003037881.1	-	431295	431246
Sma-1-6259	NW_003037885.1	-	418008	417959
Sma-1-6260	NW_003037902.1	+	498746	498795
Sma-1-6261	NW_003037980.1	-	827373	827324
Sma-1-6262	NW_003038027.1	+	439621	439670
Sma-1-6263	NW_003038133.1	+	157217	157266
Sma-1-6264	NW_003038133.1	-	156953	156904
Sma-1-6265	NS_000200.1	+	88605601	88605650
Sma-1-6266	NS_000200.1	+	147253980	147254029
Sma-1-6267	NS_000200.1	+	223157827	223157876
Sma-1-6268	NS_000200.1	+	240810335	240810384
Sma-1-6269	NS_000200.1	+	283029399	283029448
Sma-1-6270	NS_000200.1	-	379067997	379067948
Sma-1-6271	NS_000200.1	-	330054681	330054632
Sma-1-6272	NS_000200.1	-	283029135	283029086
Sma-1-6273	NS_000200.1	-	211788235	211788186
Sma-1-6274	NS_000200.1	-	128515955	128515906
Sma-1-6275	NS_000200.1	-	124863086	124863037
Sma-1-6276	NS_000200.1	-	123670024	123669975
Sma-1-6277	NS_000200.1	-	42075949	42075900
Sma-1-6278	NW_003038246.1	-	173619	173570
Sma-1-6279	NS_000200.1	-	315814747	315814698
Sma-1-6280	NW_003037866.1	+	1069531	1069580
Sma-1-6281	NS_000200.1	+	112557330	112557379
Sma-1-6282	NW_003037833.1	+	47084	47133
Sma-1-6283	NS_000200.1	+	72238180	72238229
Sma-1-6284	NW_003037825.1	+	379792	379841
Sma-1-6285	NS_000200.1	+	59509574	59509623
Sma-1-6286	NW_003037822.1	-	146486	146437
Sma-1-6287	NS_000200.1	-	54454492	54454443
Sma-1-6288	NW_003036796.1	-	498	449
Sma-1-6289	NW_003037793.1	+	306904	306953
Sma-1-6290	NS_000200.1	+	306904	306953
Sma-1-6291	NS_000200.1	-	347673221	347673172
Sma-1-6292	NW_003037978.1	-	109308	109259
Sma-1-6293	NS_000200.1	-	209377318	209377269
Sma-1-6294	NW_003038037.1	+	587883	587932
Sma-1-6295	NS_000200.1	+	250743292	250743341
Sma-1-6296	NW_003038249.1	+	70187	70236
Sma-1-6297	NS_000200.1	+	316752440	316752489
Sma-1-6298	NW_003026506.1	-	33553	33504

Sma-1-6299	NS_000200.1	-	279594618	279594569
Sma-1-6300	NW_003037809.1	+	2596321	2596370
Sma-1-6301	NS_000200.1	+	36260785	36260834
Sma-1-6302	NW_003037925.1	-	427922	427873
Sma-1-6303	NS_000200.1	-	172829925	172829876
Sma-1-6304	NW_003037940.1	+	74942	74991
Sma-1-6305	NS_000200.1	+	183817072	183817121
Sma-1-6306	NW_003035639.1	+	4577	4626
Sma-1-6307	NS_000200.1	+	329955017	329955066
Sma-1-6308	NW_003037825.1	-	1205177	1205128
Sma-1-6309	NS_000200.1	-	60334959	60334910
Sma-1-6310	NW_003035660.1	-	2263	2214
Sma-1-6311	NS_000200.1	-	330548576	330548527
Sma-1-6312	NW_003026465.1	+	850608	850657
Sma-1-6313	NW_003037889.1	+	1759862	1759911
Sma-1-6314	NS_000200.1	+	135536352	135536401
Sma-1-6315	NS_000200.1	+	208243202	208243251
Sma-1-6316	NW_003038132.1	-	352020	351971
Sma-1-6317	NS_000200.1	-	282772490	282772441
Sma-1-6318	NW_003038080.1	+	370066	370115
Sma-1-6319	NS_000200.1	+	268822676	268822725
Sma-1-6320	NW_003038033.1	+	441961	442010
Sma-1-6321	NS_000200.1	+	246421963	246422012
Sma-1-6322	NW_003038041.1	+	126950	126999
Sma-1-6323	NS_000200.1	+	253794232	253794281
Sma-1-6324	NW_003026617.1	-	109711	109662
Sma-1-6325	NW_003037951.1	+	54412	54461
Sma-1-6326	NW_003037816.1	-	437208	437159
Sma-1-6327	NW_003037836.1	-	814082	814033
Sma-1-6328	NW_003037861.1	-	650159	650110
Sma-1-6329	NW_003037896.1	+	465669	465718
Sma-1-6330	NW_003038037.1	+	190121	190170
Sma-1-6331	NW_003038257.1	+	467376	467425
Sma-1-6332	NS_000200.1	+	143386092	143386141
Sma-1-6333	NS_000200.1	+	192732137	192732186
Sma-1-6334	NS_000200.1	+	250345530	250345579
Sma-1-6335	NS_000200.1	+	321641704	321641753
Sma-1-6336	NS_000200.1	-	319680570	319680521
Sma-1-6337	NS_000200.1	-	105426613	105426564
Sma-1-6338	NS_000200.1	-	76371181	76371132
Sma-1-6339	NS_000200.1	-	46646080	46646031
Sma-1-6340	NW_003037883.1	-	323042	322993
Sma-1-6341	NS_000200.1	-	126386765	126386716
Sma-1-6342	NW_003037877.1	-	323177	323128
Sma-1-6343	NS_000200.1	-	121361739	121361690
Sma-1-6344	NW_003037884.1	+	1163544	1163593
Sma-1-6345	NS_000200.1	+	127943209	127943258
Sma-1-6346	NW_003037797.1	+	884246	884295
Sma-1-6347	NW_003037797.1	-	884549	884500
Sma-1-6348	NS_000200.1	+	11312106	11312155
Sma-1-6349	NS_000200.1	-	11312409	11312360
Sma-1-6350	NW_003037801.1	+	888703	888752

Sma-1-6351	NS_000200.1	+	21978183	21978232
Sma-1-6352	NW_003037964.1	+	110168	110217
Sma-1-6353	NS_000200.1	+	197711690	197711739
Sma-1-6354	NW_003026512.1	-	174856	174807
Sma-1-6355	NS_000200.1	-	285268896	285268847
Sma-1-6356	NW_003037992.1	-	249257	249208
Sma-1-6357	NS_000200.1	-	218090738	218090689
Sma-1-6358	NW_003037825.1	+	1082794	1082843
Sma-1-6359	NW_003037886.1	+	908471	908520
Sma-1-6360	NW_003037888.1	-	804244	804195
Sma-1-6361	NW_003037941.1	+	190517	190566
Sma-1-6362	NW_003038031.1	+	72339	72388
Sma-1-6363	NW_003038505.1	+	269090	269139
Sma-1-6364	NS_000200.1	+	60212576	60212625
Sma-1-6365	NS_000200.1	+	130164038	130164087
Sma-1-6366	NS_000200.1	+	184924991	184925040
Sma-1-6367	NS_000200.1	+	244350097	244350146
Sma-1-6368	NS_000200.1	+	336348427	336348476
Sma-1-6369	NS_000200.1	-	133012736	133012687
Sma-1-6370	NW_003037873.1	+	566487	566536
Sma-1-6371	NS_000200.1	+	119305615	119305664
Sma-1-6372	NW_003037871.1	+	173199	173248
Sma-1-6373	NS_000200.1	+	117620937	117620986
Sma-1-6374	NW_003037864.1	+	1269652	1269701
Sma-1-6375	NS_000200.1	+	109762226	109762275
Sma-1-6376	NW_003037902.1	-	489913	489864
Sma-1-6377	NS_000200.1	-	147245147	147245098
Sma-1-6378	NW_003037808.1	+	747644	747693
Sma-1-6379	NS_000200.1	+	32534281	32534330
Sma-1-6380	NW_003037800.1	-	372543	372494
Sma-1-6381	NS_000200.1	-	19001903	19001854
Sma-1-6382	NW_003026557.1	-	5830	5781
Sma-1-6383	NS_000200.1	-	300993873	300993824
Sma-1-6384	NW_003037852.1	+	1046886	1046935
Sma-1-6385	NS_000200.1	+	95438781	95438830
Sma-1-6386	NW_003036400.1	-	6106	6057
Sma-1-6387	NW_003037812.1	-	1416814	1416765
Sma-1-6388	NW_003037812.1	-	1412551	1412502
Sma-1-6389	NW_003037915.1	+	228028	228077
Sma-1-6390	NS_000200.1	+	162128014	162128063
Sma-1-6391	NS_000200.1	-	359876289	359876240
Sma-1-6392	NS_000200.1	-	40899553	40899504
Sma-1-6393	NS_000200.1	-	40895290	40895241
Sma-1-6394	NW_003037813.1	-	1481102	1481053
Sma-1-6395	NS_000200.1	-	42467965	42467916
Sma-1-6396	NW_003037906.1	+	485350	485399
Sma-1-6397	NS_000200.1	+	150793129	150793178
Sma-1-6398	NW_003026458.1	+	766512	766561
Sma-1-6399	NS_000200.1	+	102634742	102634791
Sma-1-6400	NW_003038369.1	-	9120	9071
Sma-1-6401	NW_003038222.1	+	38075	38124
Sma-1-6402	NW_003037803.1	+	187098	187147

Sma-1-6403	NW_003037848.1	-	1194245	1194196
Sma-1-6404	NW_003037917.1	-	474950	474901
Sma-1-6405	NW_003037976.1	-	504414	504365
Sma-1-6406	NS_000200.1	+	23687635	23687684
Sma-1-6407	NS_000200.1	+	308825739	308825788
Sma-1-6408	NS_000200.1	-	327118059	327118010
Sma-1-6409	NS_000200.1	-	207118729	207118680
Sma-1-6410	NS_000200.1	-	165511754	165511705
Sma-1-6411	NS_000200.1	-	90887316	90887267
Sma-1-6412	NW_003038145.1	-	8445	8396
Sma-1-6413	NS_000200.1	-	288619868	288619819
Sma-1-6414	NW_003038023.1	+	653499	653548
Sma-1-6415	NS_000200.1	+	237813790	237813839
Sma-1-6416	NW_003028440.1	+	645	694
Sma-1-6417	NW_003037821.1	-	929862	929813
Sma-1-6418	NS_000200.1	+	369635234	369635283
Sma-1-6419	NS_000200.1	-	54074346	54074297
Sma-1-6420	NW_003037917.1	-	1238743	1238694
Sma-1-6421	NS_000200.1	-	166275547	166275498
Sma-1-6422	NW_003037807.1	-	814679	814630
Sma-1-6423	NS_000200.1	-	31076195	31076146
Sma-1-6424	NW_003037806.1	-	273324	273275
Sma-1-6425	NW_003037854.1	+	1865553	1865602
Sma-1-6426	NW_003038028.1	+	693559	693608
Sma-1-6427	NS_000200.1	+	98769974	98770023
Sma-1-6428	NS_000200.1	+	242001133	242001182
Sma-1-6429	NS_000200.1	-	28468538	28468489
Sma-1-6430	NW_003037931.1	+	13006	13055
Sma-1-6431	NW_003037941.1	+	924658	924707
Sma-1-6432	NS_000200.1	+	176845673	176845722
Sma-1-6433	NS_000200.1	+	185659132	185659181
Sma-1-6434	NW_003037959.1	-	28293	28244
Sma-1-6435	NS_000200.1	-	195093926	195093877
Sma-1-6436	NW_003029656.1	+	124	173
Sma-1-6437	NW_003037822.1	+	1571995	1572044
Sma-1-6438	NW_003037844.1	+	782815	782864
Sma-1-6439	NW_003037897.1	+	107417	107466
Sma-1-6440	NS_000200.1	+	55880001	55880050
Sma-1-6441	NS_000200.1	+	86966261	86966310
Sma-1-6442	NS_000200.1	+	143589793	143589842
Sma-1-6443	NS_000200.1	+	366361312	366361361
Sma-1-6444	NW_003029802.1	-	3305	3256
Sma-1-6445	NW_003038478.1	-	6198	6149
Sma-1-6446	NW_003037817.1	-	377342	377293
Sma-1-6447	NW_003037836.1	+	298414	298463
Sma-1-6448	NW_003037866.1	+	527098	527147
Sma-1-6449	NW_003037902.1	+	604614	604663
Sma-1-6450	NW_003038032.1	-	577251	577202
Sma-1-6451	NS_000200.1	+	75855513	75855562
Sma-1-6452	NS_000200.1	+	112014897	112014946
Sma-1-6453	NS_000200.1	+	147359848	147359897
Sma-1-6454	NS_000200.1	-	375809829	375809780

Sma-1-6455	NS_000200.1	-	332581232	332581183
Sma-1-6456	NS_000200.1	-	245736411	245736362
Sma-1-6457	NS_000200.1	-	47759694	47759645
Sma-1-6458	NW_003037818.1	-	1930178	1930129
Sma-1-6459	NS_000200.1	-	50645585	50645536
Sma-1-6460	NW_003037854.1	-	1839306	1839257
Sma-1-6461	NS_000200.1	-	98743727	98743678
Sma-1-6462	NW_003037827.1	-	735235	735186
Sma-1-6463	NS_000200.1	-	63357376	63357327
Sma-1-6464	NW_003037817.1	+	116141	116190
Sma-1-6465	NS_000200.1	+	47498493	47498542
Sma-1-6466	NW_003038011.1	+	280591	280640
Sma-1-6467	NS_000200.1	+	227808241	227808290
Sma-1-6468	NW_003037918.1	-	612192	612143
Sma-1-6469	NS_000200.1	-	167179182	167179133
Sma-1-6470	NW_003037982.1	-	1325209	1325160
Sma-1-6471	NS_000200.1	-	214984404	214984355
Sma-1-6472	NW_003038034.1	-	313185	313136
Sma-1-6473	NS_000200.1	-	247254941	247254892
Sma-1-6474	NW_003026456.1	+	365998	366047
Sma-1-6475	NW_003038037.1	-	71631	71582
Sma-1-6476	NS_000200.1	+	58289134	58289183
Sma-1-6477	NS_000200.1	-	250227040	250226991
Sma-1-6478	NW_003037945.1	-	1087306	1087257
Sma-1-6479	NS_000200.1	-	190189270	190189221
Sma-1-6480	NW_003024566.1	+	143	192
Sma-1-6481	NW_003037863.1	-	1173458	1173409
Sma-1-6482	NS_000200.1	+	348865015	348865064
Sma-1-6483	NS_000200.1	-	107931825	107931776
Sma-1-6484	NW_003037881.1	-	595383	595334
Sma-1-6485	NW_003037890.1	+	2072753	2072802
Sma-1-6486	NW_003037976.1	-	132358	132309
Sma-1-6487	NS_000200.1	+	137756114	137756163
Sma-1-6488	NS_000200.1	-	206746673	206746624
Sma-1-6489	NS_000200.1	-	125027174	125027125
Sma-1-6490	NW_003037909.1	-	943359	943310
Sma-1-6491	NW_003037972.1	+	725668	725717
Sma-1-6492	NS_000200.1	+	203201372	203201421
Sma-1-6493	NS_000200.1	-	154485008	154484959
Sma-1-6494	NW_003037924.1	-	183769	183720
Sma-1-6495	NS_000200.1	-	171890727	171890678
Sma-1-6496	NW_003038003.1	-	377276	377227
Sma-1-6497	NS_000200.1	-	223876558	223876509
Sma-1-6498	NW_003037793.1	-	3124913	3124864
Sma-1-6499	NS_000200.1	-	3124913	3124864
Sma-1-6500	NW_003037827.1	+	2460475	2460524
Sma-1-6501	NW_003038124.1	+	8490	8539
Sma-1-6502	NS_000200.1	+	65082616	65082665
Sma-1-6503	NS_000200.1	+	279001134	279001183
Sma-1-6504	NW_003037841.1	+	1760892	1760941
Sma-1-6505	NW_003038077.1	-	282099	282050
Sma-1-6506	NS_000200.1	+	84372073	84372122

Sma-1-6507	NS_000200.1	-	267053739	267053690
Sma-1-6508	NW_003038034.1	+	25753	25802
Sma-1-6509	NS_000200.1	+	246967509	246967558
Sma-1-6510	NW_003026464.1	+	110120	110169
Sma-1-6511	NS_000200.1	+	203410429	203410478
Sma-1-6512	NW_003037788.1	-	18056	18007
Sma-1-6513	NS_000200.1	-	380465294	380465245
Sma-1-6514	NW_003038028.1	-	746257	746208
Sma-1-6515	NS_000200.1	-	242053831	242053782
Sma-1-6516	NW_003039127.1	+	1943795	1943844
Sma-1-6517	NW_003038055.1	-	235868	235819
Sma-1-6518	NS_000200.1	+	115021294	115021343
Sma-1-6519	NS_000200.1	-	258935190	258935141
Sma-1-6520	NW_003038017.1	+	146849	146898
Sma-1-6521	NS_000200.1	+	231912880	231912929
Sma-1-6522	NW_003038261.1	-	529082	529033
Sma-1-6523	NS_000200.1	-	324142733	324142684
Sma-1-6524	NW_003028386.1	+	722	771
Sma-1-6525	NW_003037876.1	-	232080	232031
Sma-1-6526	NW_003037791.1	-	264134	264085
Sma-1-6527	NW_003037795.1	+	1325758	1325807
Sma-1-6528	NW_003037797.1	+	1470634	1470683
Sma-1-6529	NW_003037797.1	-	1809005	1808956
Sma-1-6530	NW_003037800.1	-	524940	524891
Sma-1-6531	NW_003037802.1	+	1264698	1264747
Sma-1-6532	NW_003037810.1	+	100898	100947
Sma-1-6533	NW_003037837.1	+	990274	990323
Sma-1-6534	NW_003037841.1	-	2421204	2421155
Sma-1-6535	NW_003037848.1	-	566155	566106
Sma-1-6536	NW_003037887.1	+	240778	240827
Sma-1-6537	NW_003037888.1	-	239719	239670
Sma-1-6538	NW_003037909.1	-	606496	606447
Sma-1-6539	NW_003037909.1	-	23534	23485
Sma-1-6540	NW_003037942.1	-	394559	394510
Sma-1-6541	NW_003037945.1	-	680433	680384
Sma-1-6542	NW_003037973.1	-	10876	10827
Sma-1-6543	NW_003037975.1	-	14924	14875
Sma-1-6544	NW_003037976.1	+	621034	621083
Sma-1-6545	NW_003037988.1	+	139817	139866
Sma-1-6546	NW_003037997.1	+	65026	65075
Sma-1-6547	NW_003038018.1	+	359335	359384
Sma-1-6548	NW_003038227.1	-	409	360
Sma-1-6549	NW_003039127.1	-	2181792	2181743
Sma-1-6550	NW_003038084.1	+	216553	216602
Sma-1-6551	NW_003038095.1	+	89252	89301
Sma-1-6552	NS_000200.1	+	7716641	7716690
Sma-1-6553	NS_000200.1	+	11898494	11898543
Sma-1-6554	NS_000200.1	+	23267492	23267541
Sma-1-6555	NS_000200.1	+	36705420	36705469
Sma-1-6556	NS_000200.1	+	77796132	77796181
Sma-1-6557	NS_000200.1	+	130704756	130704805
Sma-1-6558	NS_000200.1	+	207235349	207235398

Sma-1-6559	NS_000200.1	+	216133635	216133684
Sma-1-6560	NS_000200.1	+	220876468	220876517
Sma-1-6561	NS_000200.1	+	232760185	232760234
Sma-1-6562	NS_000200.1	+	271231797	271231846
Sma-1-6563	NS_000200.1	+	274158294	274158343
Sma-1-6564	NS_000200.1	+	369493925	369493974
Sma-1-6565	NS_000200.1	-	381969562	381969513
Sma-1-6566	NS_000200.1	-	310281763	310281714
Sma-1-6567	NS_000200.1	-	205597148	205597099
Sma-1-6568	NS_000200.1	-	203992603	203992554
Sma-1-6569	NS_000200.1	-	189782397	189782348
Sma-1-6570	NS_000200.1	-	186154467	186154418
Sma-1-6571	NS_000200.1	-	154148145	154148096
Sma-1-6572	NS_000200.1	-	153565183	153565134
Sma-1-6573	NS_000200.1	-	132448211	132448162
Sma-1-6574	NS_000200.1	-	120777716	120777667
Sma-1-6575	NS_000200.1	-	115259291	115259242
Sma-1-6576	NS_000200.1	-	90259226	90259177
Sma-1-6577	NS_000200.1	-	85032385	85032336
Sma-1-6578	NS_000200.1	-	19154300	19154251
Sma-1-6579	NS_000200.1	-	12236865	12236816
Sma-1-6580	NW_003037898.1	-	238963	238914
Sma-1-6581	NW_003037858.1	-	118546	118497
Sma-1-6582	NS_000200.1	-	144222785	144222736
Sma-1-6583	NS_000200.1	-	100782822	100782773
Sma-1-6584	NW_003037901.1	-	417600	417551
Sma-1-6585	NS_000200.1	-	146266061	146266012
Sma-1-6586	NW_003037827.1	+	707185	707234
Sma-1-6587	NS_000200.1	+	63329326	63329375
Sma-1-6588	NW_003037826.1	+	581114	581163
Sma-1-6589	NS_000200.1	+	61580630	61580679
Sma-1-6590	NW_003037796.1	+	420054	420103
Sma-1-6591	NS_000200.1	+	8629698	8629747
Sma-1-6592	NW_003037062.1	+	152	201
Sma-1-6593	NS_000200.1	+	343761646	343761695
Sma-1-6594	NW_003038161.1	-	558775	558726
Sma-1-6595	NS_000200.1	-	297928626	297928577
Sma-1-6596	NW_003026612.1	+	26884	26933
Sma-1-6597	NS_000200.1	+	312332271	312332320
Sma-1-6598	NW_003032266.1	-	20257	20208
Sma-1-6599	NS_000200.1	-	358211004	358210955
Sma-1-6600	NW_003037795.1	+	986175	986224
Sma-1-6601	NS_000200.1	+	7377058	7377107
Sma-1-6602	NW_003026607.1	+	135964	136013
Sma-1-6603	NS_000200.1	+	308168349	308168398
Sma-1-6604	NW_003026515.1	+	164501	164550
Sma-1-6605	NS_000200.1	+	290808912	290808961
Sma-1-6606	NW_003037880.1	-	593228	593179
Sma-1-6607	NW_003037882.1	+	558210	558259
Sma-1-6608	NW_003037975.1	+	992341	992390
Sma-1-6609	NW_003026496.1	-	21913	21864
Sma-1-6610	NS_000200.1	+	125849884	125849933

Sma-1-6611	NS_000200.1	+	206574565	206574614
Sma-1-6612	NS_000200.1	-	274558654	274558605
Sma-1-6613	NS_000200.1	-	124276786	124276737
Sma-1-6614	NW_003037841.1	-	2055881	2055832
Sma-1-6615	NS_000200.1	-	84667062	84667013
Sma-1-6616	NW_003037839.1	+	329224	329273
Sma-1-6617	NS_000200.1	+	79287374	79287423
Sma-1-6618	NW_003037974.1	+	135632	135681
Sma-1-6619	NS_000200.1	+	204810938	204810987
Sma-1-6620	NW_003038034.1	+	279210	279259
Sma-1-6621	NS_000200.1	+	247220966	247221015
Sma-1-6622	NW_003037976.1	+	215433	215482
Sma-1-6623	NS_000200.1	+	206829748	206829797
Sma-1-6624	NW_003038018.1	+	694223	694272
Sma-1-6625	NS_000200.1	+	233095073	233095122
Sma-1-6626	NW_003034545.1	+	766	815
Sma-1-6627	NW_003037954.1	+	68198	68247
Sma-1-6628	NS_000200.1	+	193297820	193297869
Sma-1-6629	NS_000200.1	+	326895707	326895756
Sma-1-6630	NW_003038084.1	-	40670	40621
Sma-1-6631	NS_000200.1	-	271055914	271055865
Sma-1-6632	NW_003033027.1	-	504	455
Sma-1-6633	NW_003037795.1	+	1595761	1595810
Sma-1-6634	NW_003037797.1	+	1941280	1941329
Sma-1-6635	NW_003037806.1	-	350405	350356
Sma-1-6636	NW_003037863.1	-	1361104	1361055
Sma-1-6637	NW_003038261.1	+	974391	974440
Sma-1-6638	NS_000200.1	+	7986644	7986693
Sma-1-6639	NS_000200.1	+	12369140	12369189
Sma-1-6640	NS_000200.1	+	324588042	324588091
Sma-1-6641	NS_000200.1	-	326215324	326215275
Sma-1-6642	NS_000200.1	-	108119471	108119422
Sma-1-6643	NS_000200.1	-	28545619	28545570
Sma-1-6644	NW_003037916.1	+	1178263	1178312
Sma-1-6645	NS_000200.1	+	164371674	164371723
Sma-1-6646	NW_003037916.1	-	1703750	1703701
Sma-1-6647	NS_000200.1	-	164897161	164897112
Sma-1-6648	NW_003037796.1	-	76368	76319
Sma-1-6649	NS_000200.1	-	8286012	8285963
Sma-1-6650	NW_003026472.1	+	68308	68357
Sma-1-6651	NS_000200.1	+	236480300	236480349
env-84	AB231864.1	-	41943	41894
Sma-1-6652	NW_003022248.1	+	2146	2195
Sma-1-6653	NW_003028351.1	-	1174	1125
Sma-1-6654	NW_003031306.1	+	699	748
Sma-1-6655	NW_003035418.1	+	1132	1181
Sma-1-6656	NW_003038253.1	-	71982	71933
Sma-1-6657	NW_003037962.1	+	550819	550868
Sma-1-6658	NW_003037898.1	-	228168	228119
Sma-1-6659	NW_003037794.1	+	1859462	1859511
Sma-1-6660	NW_003037809.1	-	1823358	1823309
Sma-1-6661	NW_003037819.1	+	958856	958905

Sma-1-6662	NW_003037825.1	-	1630713	1630664
Sma-1-6663	NW_003037825.1	-	314107	314058
Sma-1-6664	NW_003037836.1	+	256643	256692
Sma-1-6665	NW_003037839.1	+	251414	251463
Sma-1-6666	NW_003037840.1	-	331020	330971
Sma-1-6667	NW_003037843.1	+	2347	2396
Sma-1-6668	NW_003037860.1	+	280489	280538
Sma-1-6669	NW_003037861.1	+	843455	843504
Sma-1-6670	NW_003037895.1	+	301682	301731
Sma-1-6671	NW_003037899.1	+	17203	17252
Sma-1-6672	NW_003037905.1	+	631805	631854
Sma-1-6673	NW_003037905.1	+	633772	633821
Sma-1-6674	NW_003037912.1	+	1226979	1227028
Sma-1-6675	NW_003037918.1	+	1644412	1644461
Sma-1-6676	NW_003037940.1	+	394637	394686
Sma-1-6677	NW_003037953.1	+	25478	25527
Sma-1-6678	NW_003037956.1	-	193368	193319
Sma-1-6679	NW_003037970.1	+	499048	499097
Sma-1-6680	NW_003037970.1	-	182784	182735
Sma-1-6681	NW_003037980.1	-	191412	191363
Sma-1-6682	NW_003037982.1	+	930462	930511
Sma-1-6683	NW_003038028.1	-	794094	794045
Sma-1-6684	NW_003038259.1	+	186439	186488
Sma-1-6685	NW_003026484.1	+	15126	15175
Sma-1-6686	NW_003026508.1	-	162329	162280
Sma-1-6687	NW_003038068.1	-	209871	209822
Sma-1-6688	NS_000200.1	+	6039057	6039106
Sma-1-6689	NS_000200.1	+	52047121	52047170
Sma-1-6690	NS_000200.1	+	75813742	75813791
Sma-1-6691	NS_000200.1	+	79209564	79209613
Sma-1-6692	NS_000200.1	+	85641654	85641703
Sma-1-6693	NS_000200.1	+	104023517	104023566
Sma-1-6694	NS_000200.1	+	105619909	105619958
Sma-1-6695	NS_000200.1	+	142747884	142747933
Sma-1-6696	NS_000200.1	+	144536314	144536363
Sma-1-6697	NS_000200.1	+	149900817	149900866
Sma-1-6698	NS_000200.1	+	149902784	149902833
Sma-1-6699	NS_000200.1	+	158038554	158038603
Sma-1-6700	NS_000200.1	+	168211402	168211451
Sma-1-6701	NS_000200.1	+	184136767	184136816
Sma-1-6702	NS_000200.1	+	192938529	192938578
Sma-1-6703	NS_000200.1	+	196627662	196627711
Sma-1-6704	NS_000200.1	+	201555238	201555287
Sma-1-6705	NS_000200.1	+	214589657	214589706
Sma-1-6706	NS_000200.1	+	256977817	256977866
Sma-1-6707	NS_000200.1	+	323047038	323047087
Sma-1-6708	NS_000200.1	+	345710555	345710604
Sma-1-6709	NS_000200.1	+	361014632	361014681
Sma-1-6710	NS_000200.1	+	367431900	367431949
Sma-1-6711	NS_000200.1	-	369429272	369429223
Sma-1-6712	NS_000200.1	-	319389779	319389730
Sma-1-6713	NS_000200.1	-	281206943	281206894

Sma-1-6714	NS_000200.1	-	263277079	263277030
Sma-1-6715	NS_000200.1	-	242101668	242101619
Sma-1-6716	NS_000200.1	-	211152274	211152225
Sma-1-6717	NS_000200.1	-	201238974	201238925
Sma-1-6718	NS_000200.1	-	194249823	194249774
Sma-1-6719	NS_000200.1	-	144211990	144211941
Sma-1-6720	NS_000200.1	-	80954344	80954295
Sma-1-6721	NS_000200.1	-	60760495	60760446
Sma-1-6722	NS_000200.1	-	59443889	59443840
Sma-1-6723	NS_000200.1	-	35487822	35487773
Sma-1-6724	NW_003038249.1	+	299260	299309
Sma-1-6725	NW_003037972.1	+	181731	181780
Sma-1-6726	NS_000200.1	+	202657435	202657484
Sma-1-6727	NS_000200.1	+	316981513	316981562
Sma-1-6728	NW_003038020.1	-	101205	101156
Sma-1-6729	NS_000200.1	-	234068549	234068500
Sma-1-6730	NW_003038191.1	+	2631	2680
Sma-1-6731	NS_000200.1	+	302489816	302489865
Sma-1-6732	NW_003037865.1	+	225008	225057
Sma-1-6733	NS_000200.1	+	110646927	110646976
Sma-1-6734	NW_003037863.1	+	974411	974460
Sma-1-6735	NS_000200.1	+	107732778	107732827
Sma-1-6736	NW_003037863.1	-	735228	735179
Sma-1-6737	NS_000200.1	-	107493595	107493546
Sma-1-6738	NW_003037863.1	-	1672325	1672276
Sma-1-6739	NS_000200.1	-	108430692	108430643
Sma-1-6740	NW_003037887.1	+	1456131	1456180
Sma-1-6741	NS_000200.1	+	131920109	131920158
Sma-1-6742	NW_003037887.1	+	404044	404093
Sma-1-6743	NS_000200.1	+	130868022	130868071
Sma-1-6744	NW_003037861.1	+	633620	633669
Sma-1-6745	NW_003037904.1	+	129607	129656
Sma-1-6746	NS_000200.1	+	105410074	105410123
Sma-1-6747	NS_000200.1	+	148844106	148844155
Sma-1-6748	NW_003037888.1	+	1179807	1179856
Sma-1-6749	NS_000200.1	+	133388299	133388348
Sma-1-6750	NW_003037803.1	+	1333237	1333286
Sma-1-6751	NS_000200.1	+	24833774	24833823
Sma-1-6752	NW_003037805.1	+	1551571	1551619
Sma-1-6753	NS_000200.1	+	28109415	28109463
Sma-1-6754	NW_003037939.1	+	468650	468699
Sma-1-6755	NW_003038035.1	+	60641	60690
Sma-1-6756	NW_003038453.1	+	21976	22025
Sma-1-6757	NS_000200.1	+	183338404	183338453
Sma-1-6758	NS_000200.1	+	247922133	247922182
Sma-1-6759	NS_000200.1	+	330603903	330603952
Sma-1-6760	NW_003037809.1	+	2644790	2644839
Sma-1-6761	NS_000200.1	+	36309254	36309303
Sma-1-6762	NW_003037996.1	-	526507	526458
Sma-1-6763	NS_000200.1	-	220771383	220771334
Sma-1-6764	NW_003037885.1	-	399155	399106
Sma-1-6765	NW_003037940.1	-	727135	727086

Sma-1-6766	NS_000200.1	-	184469265	184469216
Sma-1-6767	NS_000200.1	-	128497102	128497053
Sma-1-6768	NW_003026465.1	-	89600	89551
Sma-1-6769	NS_000200.1	-	207482194	207482145
Sma-1-6770	NW_003020818.1	-	777	728
Sma-1-6771	NS_000200.1	-	364515892	364515843
Sma-1-6772	NW_003038015.1	+	507832	507881
Sma-1-6773	NS_000200.1	+	230831322	230831371
Sma-1-6774	NW_003038215.1	+	143156	143205
Sma-1-6775	NS_000200.1	+	306756192	306756241
Sma-1-6776	NW_003038027.1	+	320868	320917
Sma-1-6777	NW_003038152.1	+	15661	15710
Sma-1-6778	NS_000200.1	+	240691582	240691631
Sma-1-6779	NS_000200.1	+	292621385	292621434
Sma-1-6780	NW_003038037.1	-	1334182	1334133
Sma-1-6781	NS_000200.1	-	251489591	251489542
Sma-1-6782	NW_003038150.1	+	90191	90240
Sma-1-6783	NW_003038162.1	+	279638	279687
Sma-1-6784	NS_000200.1	+	291568929	291568978
Sma-1-6785	NS_000200.1	+	298225093	298225142
Sma-1-6786	NW_003038254.1	+	58204	58253
Sma-1-6787	NS_000200.1	+	320093762	320093811
Sma-1-6788	NW_003038215.1	-	138355	138306
Sma-1-6789	NW_003035870.1	-	50278	50229
Sma-1-6790	NW_003037937.1	-	3497	3448
Sma-1-6791	NW_003037795.1	-	1368284	1368235
Sma-1-6792	NW_003037817.1	-	886290	886241
Sma-1-6793	NW_003037821.1	-	696783	696734
Sma-1-6794	NW_003037829.1	-	1034772	1034723
Sma-1-6795	NW_003037834.1	+	344963	345012
Sma-1-6796	NW_003037863.1	+	1501270	1501319
Sma-1-6797	NW_003037881.1	+	617391	617440
Sma-1-6798	NW_003037882.1	-	539921	539872
Sma-1-6799	NW_003037904.1	-	191787	191738
Sma-1-6800	NW_003037968.1	+	8936	8985
Sma-1-6801	NW_003038030.1	-	186920	186871
Sma-1-6802	NW_003038241.1	-	160442	160393
Sma-1-6803	NW_003026507.1	-	339434	339385
Sma-1-6804	NW_003038086.1	+	357069	357118
Sma-1-6805	NW_003038086.1	-	351397	351348
Sma-1-6806	NW_003038087.1	-	338388	338339
Sma-1-6807	NS_000200.1	+	73505449	73505498
Sma-1-6808	NS_000200.1	+	108259637	108259686
Sma-1-6809	NS_000200.1	+	125049182	125049231
Sma-1-6810	NS_000200.1	+	199926106	199926155
Sma-1-6811	NS_000200.1	+	272605662	272605711
Sma-1-6812	NS_000200.1	-	335415567	335415518
Sma-1-6813	NS_000200.1	-	314623147	314623098
Sma-1-6814	NS_000200.1	-	306751391	306751342
Sma-1-6815	NS_000200.1	-	279999585	279999536
Sma-1-6816	NS_000200.1	-	273366789	273366740
Sma-1-6817	NS_000200.1	-	272599990	272599941

Sma-1-6818	NS_000200.1	-	243322787	243322738
Sma-1-6819	NS_000200.1	-	181203406	181203357
Sma-1-6820	NS_000200.1	-	148906286	148906237
Sma-1-6821	NS_000200.1	-	125831595	125831546
Sma-1-6822	NS_000200.1	-	66935310	66935261
Sma-1-6823	NS_000200.1	-	53841267	53841218
Sma-1-6824	NS_000200.1	-	48268642	48268593
Sma-1-6825	NS_000200.1	-	7759167	7759118
Sma-1-6826	NW_003037860.1	-	277083	277034
Sma-1-6827	NS_000200.1	-	104020111	104020062
Sma-1-6828	NW_003037859.1	-	160338	160289
Sma-1-6829	NS_000200.1	-	102839179	102839130
Sma-1-6830	NW_003026619.1	+	188313	188362
Sma-1-6831	NS_000200.1	+	322379057	322379106
Sma-1-6832	NW_003037935.1	-	220062	220013
Sma-1-6833	NS_000200.1	-	179636597	179636548
Sma-1-6834	NW_003037861.1	+	643419	643468
Sma-1-6835	NS_000200.1	+	105419873	105419922
Sma-1-6836	NW_003037890.1	+	1368268	1368317
Sma-1-6837	NS_000200.1	+	137051629	137051678
Sma-1-6838	NW_003037853.1	+	496648	496697
Sma-1-6839	NS_000200.1	+	95988699	95988748
Sma-1-6840	NW_003038033.1	+	865997	866046
Sma-1-6841	NS_000200.1	+	246845999	246846048
Sma-1-6842	NW_003037950.1	-	9123	9074
Sma-1-6843	NW_003037906.1	+	385363	385412
Sma-1-6844	NW_003037974.1	-	145877	145828
Sma-1-6845	NS_000200.1	+	150693142	150693191
Sma-1-6846	NS_000200.1	-	204821183	204821134
Sma-1-6847	NS_000200.1	-	192408231	192408182
Sma-1-6848	NW_003037914.1	-	23048	22999
Sma-1-6849	NS_000200.1	-	160167298	160167249
Sma-1-6850	NW_003037845.1	+	83198	83247
Sma-1-6851	NW_003037845.1	-	79646	79597
Sma-1-6852	NW_003038033.1	+	806901	806950
Sma-1-6853	NS_000200.1	+	87085101	87085150
Sma-1-6854	NS_000200.1	+	246786903	246786952
Sma-1-6855	NS_000200.1	-	87081549	87081500
Sma-1-6856	NW_003037852.1	-	855768	855719
Sma-1-6857	NS_000200.1	-	95247663	95247614
Sma-1-6858	NW_003037814.1	+	478178	478227
Sma-1-6859	NS_000200.1	+	43462038	43462087
Sma-1-6860	NW_003037908.1	-	699459	699410
Sma-1-6861	NS_000200.1	-	153172590	153172541
Sma-1-6862	NW_003037890.1	+	646246	646295
Sma-1-6863	NS_000200.1	+	136329607	136329656
Sma-1-6864	NW_003037904.1	+	175191	175240
Sma-1-6865	NS_000200.1	+	148889690	148889739
Sma-1-6866	NW_003037859.1	-	759160	759111
Sma-1-6867	NS_000200.1	-	103438001	103437952
Sma-1-6868	NW_003037850.1	+	579442	579491
Sma-1-6869	NW_003037887.1	+	641628	641677

Sma-1-6870	NS_000200.1	+	92565472	92565521
Sma-1-6871	NS_000200.1	+	131105606	131105655
Sma-1-6872	NW_003037848.1	+	688866	688915
Sma-1-6873	NW_003038025.1	+	201578	201627
Sma-1-6874	NS_000200.1	+	90381937	90381986
Sma-1-6875	NS_000200.1	+	239059823	239059872
Sma-1-6876	NW_003037824.1	+	568455	568504
Sma-1-6877	NW_003037915.1	-	181951	181902
Sma-1-6878	NW_003038035.1	+	970172	970221
Sma-1-6879	NS_000200.1	+	57499395	57499444
Sma-1-6880	NS_000200.1	+	248831664	248831713
Sma-1-6881	NS_000200.1	-	162081937	162081888
Sma-1-6882	NW_003037937.1	+	426778	426827
Sma-1-6883	NS_000200.1	+	181626687	181626736
Sma-1-6884	NW_003037939.1	+	327019	327068
Sma-1-6885	NS_000200.1	+	183196773	183196822
Sma-1-6886	NW_003038079.1	+	215294	215343
Sma-1-6887	NS_000200.1	+	268345282	268345331
Sma-1-6888	NW_003026464.1	-	555944	555895
Sma-1-6889	NW_003037859.1	-	579083	579034
Sma-1-6890	NW_003038224.1	+	61131	61180
Sma-1-6891	NS_000200.1	+	309472859	309472908
Sma-1-6892	NS_000200.1	-	203856253	203856204
Sma-1-6893	NS_000200.1	-	103257924	103257875
Sma-1-6894	NW_003026618.1	+	38517	38566
Sma-1-6895	NS_000200.1	+	321793519	321793568
Sma-1-6896	NW_003037964.1	+	557566	557615
Sma-1-6897	NS_000200.1	+	198159088	198159137
Sma-1-6898	NW_003037920.1	+	276577	276626
Sma-1-6899	NW_003037821.1	-	246056	246007
Sma-1-6900	NS_000200.1	+	169457384	169457433
Sma-1-6901	NS_000200.1	-	53390540	53390491
Sma-1-6902	NW_003038301.1	+	766	815
Sma-1-6903	NS_000200.1	+	325714070	325714119
Sma-1-6904	NW_003026598.1	-	82347	82298
Sma-1-6905	NW_003026606.1	-	91929	91880
Sma-1-6906	NW_003037891.1	+	1527438	1527487
Sma-1-6907	NW_003038020.1	+	134041	134090
Sma-1-6908	NW_003038247.1	-	198334	198285
Sma-1-6909	NS_000200.1	+	139408716	139408765
Sma-1-6910	NS_000200.1	+	234101385	234101434
Sma-1-6911	NS_000200.1	-	316147062	316147013
Sma-1-6912	NS_000200.1	-	307947622	307947573
Sma-1-6913	NS_000200.1	-	305852563	305852514
Sma-1-6914	NW_003037912.1	+	654442	654491
Sma-1-6915	NW_003038418.1	+	633	682
Sma-1-6916	NW_003038513.1	+	7519	7568
Sma-1-6917	NW_003038131.1	-	77409	77360
Sma-1-6918	NS_000200.1	+	157466017	157466066
Sma-1-6919	NS_000200.1	+	328865152	328865201
Sma-1-6920	NS_000200.1	+	339016696	339016745
Sma-1-6921	NS_000200.1	-	282039253	282039204

Sma-1-6922	NW_003037914.1	+	1231152	1231201
Sma-1-6923	NS_000200.1	+	161375402	161375451
Sma-1-6924	NW_003037813.1	-	67448	67399
Sma-1-6925	NS_000200.1	-	41054311	41054262
Sma-1-6926	NW_003037915.1	-	395346	395297
Sma-1-6927	NS_000200.1	-	162295332	162295283
Sma-1-6928	NW_003037915.1	+	507291	507340
Sma-1-6929	NS_000200.1	+	162407277	162407326
Sma-1-6930	NW_003037920.1	+	312337	312386
Sma-1-6931	NS_000200.1	+	169493144	169493193
Sma-1-6932	NW_003037802.1	+	106837	106886
Sma-1-6933	NW_003037822.1	+	1296662	1296711
Sma-1-6934	NW_003037822.1	+	1440703	1440752
Sma-1-6935	NW_003037822.1	+	1450554	1450603
Sma-1-6936	NS_000200.1	+	22109631	22109680
Sma-1-6937	NS_000200.1	+	55604668	55604717
Sma-1-6938	NS_000200.1	+	55748709	55748758
Sma-1-6939	NS_000200.1	+	55758560	55758609
Sma-1-6940	NW_003037800.1	+	321436	321485
Sma-1-6941	NS_000200.1	+	18950796	18950845
Sma-1-6942	NW_003037799.1	-	737639	737590
Sma-1-6943	NS_000200.1	-	17727993	17727944
Sma-1-6944	NW_003037928.1	+	383869	383918
Sma-1-6945	NS_000200.1	+	174707822	174707871
Sma-1-6946	NW_003038011.1	+	37923	37972
Sma-1-6947	NS_000200.1	+	227565573	227565622
Sma-1-6948	NW_003037797.1	-	3259384	3259335
Sma-1-6949	NW_003037809.1	+	274883	274932
Sma-1-6950	NW_003037829.1	-	1007638	1007589
Sma-1-6951	NS_000200.1	+	33939347	33939396
Sma-1-6952	NS_000200.1	-	66908176	66908127
Sma-1-6953	NS_000200.1	-	13687244	13687195
Sma-1-6954	NW_003037972.1	-	281455	281406
Sma-1-6955	NS_000200.1	-	202757159	202757110
Sma-1-6956	NW_003037977.1	+	133445	133494
Sma-1-6957	NW_003037977.1	-	144128	144079
Sma-1-6958	NW_003038037.1	+	1106181	1106230
Sma-1-6959	NW_003038037.1	-	1392281	1392232
Sma-1-6960	NS_000200.1	+	208419465	208419514
Sma-1-6961	NS_000200.1	+	251261590	251261639
Sma-1-6962	NS_000200.1	-	251547690	251547641
Sma-1-6963	NS_000200.1	-	208430148	208430099
Sma-1-6964	NW_003038512.1	+	305750	305799
Sma-1-6965	NS_000200.1	+	338862447	338862496
Sma-1-6966	NW_003037982.1	+	830400	830449
Sma-1-6967	NS_000200.1	+	214489595	214489644
Sma-1-6968	NW_003037818.1	+	1464345	1464394
Sma-1-6969	NW_003037825.1	-	694641	694592
Sma-1-6970	NS_000200.1	+	50179752	50179801
Sma-1-6971	NS_000200.1	-	59824423	59824374
Sma-1-6972	NW_003038086.1	-	141944	141895
Sma-1-6973	NS_000200.1	-	272390537	272390488

Sma-1-6974	NW_003038253.1	-	47703	47654
Sma-1-6975	NS_000200.1	-	319365500	319365451
Sma-1-6976	NW_003038225.1	-	120360	120311
Sma-1-6977	NS_000200.1	-	309975024	309974975
Sma-1-6978	NW_003038072.1	+	224255	224304
Sma-1-6979	NS_000200.1	+	265386483	265386532
Sma-1-6980	NW_003038455.1	-	60059	60010
Sma-1-6981	NW_003037774.1	-	43608	43559
Sma-1-6982	NW_003037797.1	-	133995	133946
Sma-1-6983	NW_003037827.1	-	131181	131132
Sma-1-6984	NW_003037839.1	+	908672	908721
Sma-1-6985	NW_003037852.1	-	908819	908770
Sma-1-6986	NW_003038008.1	+	337916	337965
Sma-1-6987	NW_003038233.1	+	21423	21472
Sma-1-6988	NW_003038083.1	-	311078	311029
Sma-1-6989	NW_003038086.1	+	142982	143031
Sma-1-6990	NS_000200.1	+	79866822	79866871
Sma-1-6991	NS_000200.1	+	226011012	226011061
Sma-1-6992	NS_000200.1	+	272391575	272391624
Sma-1-6993	NS_000200.1	+	312604522	312604571
Sma-1-6994	NS_000200.1	-	378628985	378628936
Sma-1-6995	NS_000200.1	-	330842110	330842061
Sma-1-6996	NS_000200.1	-	270554415	270554366
Sma-1-6997	NS_000200.1	-	95300714	95300665
Sma-1-6998	NS_000200.1	-	62753322	62753273
Sma-1-6999	NS_000200.1	-	10561855	10561806
Sma-1-7000	NW_003038086.1	-	142624	142575
Sma-1-7001	NS_000200.1	-	272391217	272391168
Sma-1-7002	NW_003026458.1	+	498723	498772
Sma-1-7003	NS_000200.1	+	102366953	102367002
Sma-1-7004	NW_003038032.1	+	372496	372545
Sma-1-7005	NS_000200.1	+	245531656	245531705
Sma-1-7006	NW_003037873.1	-	543872	543823
Sma-1-7007	NW_003035880.1	+	352499	352548
Sma-1-7008	NW_003026611.1	+	215586	215635
Sma-1-7009	NW_003037937.1	-	606550	606501
Sma-1-7010	NW_003037793.1	-	2563942	2563893
Sma-1-7011	NW_003037795.1	+	265128	265177
Sma-1-7012	NW_003037799.1	-	1252547	1252498
Sma-1-7013	NW_003037819.1	-	378254	378205
Sma-1-7014	NW_003037836.1	-	447750	447701
Sma-1-7015	NW_003037850.1	+	222723	222772
Sma-1-7016	NW_003037853.1	-	1003451	1003402
Sma-1-7017	NW_003037861.1	-	189604	189555
Sma-1-7018	NW_003037881.1	+	452321	452370
Sma-1-7019	NW_003037893.1	-	299224	299175
Sma-1-7020	NW_003037903.1	-	329083	329034
Sma-1-7021	NW_003037910.1	+	1040315	1040364
Sma-1-7022	NW_003037911.1	-	370317	370268
Sma-1-7023	NW_003037938.1	-	403261	403212
Sma-1-7024	NW_003037938.1	-	222356	222307
Sma-1-7025	NW_003038037.1	+	693037	693086

Sma-1-7026	NW_003038037.1	+	1723280	1723329
Sma-1-7027	NW_003038261.1	+	912048	912097
Sma-1-7028	NW_003038518.1	+	629037	629086
Sma-1-7029	NW_003026508.1	+	64531	64580
Sma-1-7030	NW_003026517.1	-	575960	575911
Sma-1-7031	NW_003038058.1	+	327042	327091
Sma-1-7032	NW_003038077.1	+	338672	338721
Sma-1-7033	NW_003038084.1	+	325948	325997
Sma-1-7034	NS_000200.1	+	6656011	6656060
Sma-1-7035	NS_000200.1	+	92208753	92208802
Sma-1-7036	NS_000200.1	+	124884112	124884161
Sma-1-7037	NS_000200.1	+	155552290	155552339
Sma-1-7038	NS_000200.1	+	250848446	250848495
Sma-1-7039	NS_000200.1	+	251878689	251878738
Sma-1-7040	NS_000200.1	+	260158242	260158291
Sma-1-7041	NS_000200.1	+	267110312	267110361
Sma-1-7042	NS_000200.1	+	271341192	271341241
Sma-1-7043	NS_000200.1	+	281109145	281109194
Sma-1-7044	NS_000200.1	+	310785370	310785419
Sma-1-7045	NS_000200.1	+	324525699	324525748
Sma-1-7046	NS_000200.1	+	339593563	339593612
Sma-1-7047	NS_000200.1	+	342265870	342265919
Sma-1-7048	NS_000200.1	-	300181969	300181920
Sma-1-7049	NS_000200.1	-	182440374	182440325
Sma-1-7050	NS_000200.1	-	182259469	182259420
Sma-1-7051	NS_000200.1	-	181806459	181806410
Sma-1-7052	NS_000200.1	-	156157670	156157621
Sma-1-7053	NS_000200.1	-	147883164	147883115
Sma-1-7054	NS_000200.1	-	141413534	141413485
Sma-1-7055	NS_000200.1	-	119283000	119282951
Sma-1-7056	NS_000200.1	-	104966058	104966009
Sma-1-7057	NS_000200.1	-	96495502	96495453
Sma-1-7058	NS_000200.1	-	76004849	76004800
Sma-1-7059	NS_000200.1	-	51466519	51466470
Sma-1-7060	NS_000200.1	-	18242901	18242852
Sma-1-7061	NS_000200.1	-	2563942	2563893
Sma-1-7062	NW_003037793.1	+	4026372	4026421
Sma-1-7063	NS_000200.1	+	4026372	4026421
Sma-1-7064	NW_003037797.1	+	2991336	2991385
Sma-1-7065	NS_000200.1	+	13419196	13419245
Sma-1-7066	NW_003037797.1	+	1877907	1877956
Sma-1-7067	NW_003037897.1	+	376944	376993
Sma-1-7068	NS_000200.1	+	12305767	12305816
Sma-1-7069	NS_000200.1	+	143859320	143859369
Sma-1-7070	NW_003038020.1	+	395067	395116
Sma-1-7071	NW_003038068.1	+	125120	125169
Sma-1-7072	NS_000200.1	+	234362411	234362460
Sma-1-7073	NS_000200.1	+	263192328	263192377
Sma-1-7074	NW_003020691.1	+	122	171
Sma-1-7075	NS_000200.1	+	360671311	360671360
Sma-1-7076	NW_003037918.1	+	432533	432582
Sma-1-7077	NW_003037991.1	-	211198	211149

Sma-1-7078	NS_000200.1	+	166999523	166999572
Sma-1-7079	NS_000200.1	-	217360766	217360717
Sma-1-7080	NW_003038014.1	-	284622	284573
Sma-1-7081	NS_000200.1	-	230025311	230025262
Sma-1-7082	NW_003038145.1	+	112713	112762
Sma-1-7083	NS_000200.1	+	288724136	288724185
Sma-1-7084	NW_003038081.1	+	255410	255459
Sma-1-7085	NS_000200.1	+	269417453	269417502
Sma-1-7086	NW_003037793.1	-	1378920	1378871
Sma-1-7087	NW_003038254.1	+	272895	272944
Sma-1-7088	NW_003038254.1	-	271504	271455
Sma-1-7089	NW_003038119.1	-	192611	192562
Sma-1-7090	NS_000200.1	+	320308453	320308502
Sma-1-7091	NS_000200.1	-	320307062	320307013
Sma-1-7092	NS_000200.1	-	278100167	278100118
Sma-1-7093	NS_000200.1	-	1378920	1378871
Sma-1-7094	NW_003032136.1	-	1730	1681
Sma-1-7095	NS_000200.1	-	378268231	378268182
Sma-1-7096	NW_003037826.1	+	341164	341213
Sma-1-7097	NW_003038257.1	+	291298	291347
Sma-1-7098	NS_000200.1	+	61340680	61340729
Sma-1-7099	NS_000200.1	+	321465626	321465675
Sma-1-7100	NW_003037891.1	-	1762805	1762756
Sma-1-7101	NS_000200.1	-	139644083	139644034
Sma-1-7102	NW_003037938.1	-	90146	90097
Sma-1-7103	NW_003037972.1	-	337590	337541
Sma-1-7104	NW_003037980.1	+	763626	763675
Sma-1-7105	NS_000200.1	+	211724488	211724537
Sma-1-7106	NS_000200.1	-	202813294	202813245
Sma-1-7107	NS_000200.1	-	182127259	182127210
Sma-1-7108	NW_003037798.1	+	1251379	1251428
Sma-1-7109	NS_000200.1	+	15510537	15510586
Sma-1-7110	NW_003037846.1	-	655333	655284
Sma-1-7111	NS_000200.1	-	88289903	88289854
Sma-1-7112	NW_003037904.1	-	186346	186297
Sma-1-7113	NS_000200.1	-	148900845	148900796
Sma-1-7114	NW_003037819.1	-	965313	965264
Sma-1-7115	NS_000200.1	-	52053578	52053529
Sma-1-7116	NW_003037908.1	-	948734	948685
Sma-1-7117	NS_000200.1	-	153421865	153421816
Sma-1-7118	NW_003037846.1	+	790385	790434
Sma-1-7119	NS_000200.1	+	88424955	88425004
Sma-1-7120	NW_003037839.1	-	1237578	1237529
Sma-1-7121	NS_000200.1	-	80195728	80195679
Sma-1-7122	NW_003037901.1	-	330651	330602
Sma-1-7123	NS_000200.1	-	146179112	146179063
Sma-1-7124	NW_003037906.1	+	732768	732817
Sma-1-7125	NS_000200.1	+	151040547	151040596
Sma-1-7126	NW_003037918.1	+	1294365	1294414
Sma-1-7127	NS_000200.1	+	167861355	167861404
Sma-1-7128	NW_003037807.1	-	765166	765117
Sma-1-7129	NS_000200.1	-	31026682	31026633

Sma-1-7130	NW_003037822.1	-	1583103	1583054
Sma-1-7131	NW_003037930.1	+	262133	262182
Sma-1-7132	NS_000200.1	+	176120097	176120146
Sma-1-7133	NS_000200.1	-	55891109	55891060
Sma-1-7134	NW_003038041.1	+	179299	179348
Sma-1-7135	NS_000200.1	+	253846581	253846630
Sma-1-7136	NW_003037836.1	+	306141	306190
Sma-1-7137	NS_000200.1	+	75863240	75863289
Sma-1-7138	NW_003037833.1	+	738130	738179
Sma-1-7139	NS_000200.1	+	72929226	72929275
Sma-1-7140	NW_003037903.1	-	999270	999221
Sma-1-7141	NS_000200.1	-	148553351	148553302
Sma-1-7142	NW_003037832.1	-	668133	668084
Sma-1-7143	NW_003037911.1	+	306714	306763
Sma-1-7144	NW_003037912.1	+	1068892	1068941
Sma-1-7145	NS_000200.1	+	156094067	156094116
Sma-1-7146	NS_000200.1	+	157880467	157880516
Sma-1-7147	NS_000200.1	-	71877564	71877515
Sma-1-7148	NW_003037831.1	-	1573312	1573263
Sma-1-7149	NS_000200.1	-	71022614	71022565
Sma-1-7150	NW_003037830.1	-	184453	184404
Sma-1-7151	NS_000200.1	-	68074824	68074775
Sma-1-7152	NW_003037931.1	-	44666	44617
Sma-1-7153	NS_000200.1	-	176877333	176877284
Sma-1-7154	NW_003038241.1	+	13768	13817
Sma-1-7155	NS_000200.1	+	314476473	314476522
Sma-1-7156	NW_003026456.1	+	103496	103545
Sma-1-7157	NS_000200.1	+	58026632	58026681
Sma-1-7158	NW_003037907.1	+	280417	280466
Sma-1-7159	NS_000200.1	+	151684906	151684955
Sma-1-7160	NW_003038223.1	+	63153	63202
Sma-1-7161	NW_003038037.1	+	594264	594313
Sma-1-7162	NS_000200.1	+	250749673	250749722
Sma-1-7163	NS_000200.1	+	309131587	309131636
Sma-1-7164	NW_003026515.1	-	117264	117215
Sma-1-7165	NS_000200.1	-	290761675	290761626
Sma-1-7166	NW_003037808.1	-	777742	777693
Sma-1-7167	NS_000200.1	-	32564379	32564330
Sma-1-7168	NW_003037821.1	+	437691	437740
Sma-1-7169	NS_000200.1	+	53582175	53582224
Sma-1-7170	NW_003037993.1	-	183987	183938
Sma-1-7171	NS_000200.1	-	218370690	218370641
Sma-1-7172	NW_003037962.1	-	429898	429849
Sma-1-7173	NS_000200.1	-	196506741	196506692
Sma-1-7174	NW_003037798.1	-	1257416	1257367
Sma-1-7175	NS_000200.1	-	15516574	15516525
Sma-1-7176	NW_003038068.1	-	119541	119492
Sma-1-7177	NS_000200.1	-	263186749	263186700
Sma-1-7178	NW_003024325.1	-	304	255
Sma-1-7179	NW_003029802.1	+	16495	16544
Sma-1-7180	NW_003026624.1	-	19970	19921
Sma-1-7181	NW_003037808.1	+	568546	568595

Sma-1-7182	NW_003037810.1	-	1237729	1237680
Sma-1-7183	NW_003037890.1	+	1327581	1327630
Sma-1-7184	NS_000200.1	+	32355183	32355232
Sma-1-7185	NS_000200.1	+	137010942	137010991
Sma-1-7186	NS_000200.1	+	375823019	375823068
Sma-1-7187	NS_000200.1	-	365524062	365524013
Sma-1-7188	NS_000200.1	-	324890140	324890091
Sma-1-7189	NS_000200.1	-	37842251	37842202
Sma-1-7190	NW_003037935.1	+	188540	188589
Sma-1-7191	NS_000200.1	+	179605075	179605124
Sma-1-7192	NW_003037816.1	+	941166	941215
Sma-1-7193	NW_003037854.1	-	1842738	1842689
Sma-1-7194	NS_000200.1	+	47150038	47150087
Sma-1-7195	NS_000200.1	-	98747159	98747110
Sma-1-7196	NW_003037807.1	+	947986	948035
Sma-1-7197	NS_000200.1	+	31209502	31209551
Sma-1-7198	NW_003038036.1	+	667140	667189
Sma-1-7199	NS_000200.1	+	249600992	249601041
Sma-1-7200	NW_003037858.1	+	1146748	1146797
Sma-1-7201	NW_003037914.1	-	245073	245024
Sma-1-7202	NS_000200.1	+	101811024	101811073
Sma-1-7203	NS_000200.1	-	160389323	160389274
Sma-1-7204	NW_003037795.1	-	485165	485116
Sma-1-7205	NS_000200.1	-	6876048	6875999
Sma-1-7206	NW_003037829.1	-	749248	749199
Sma-1-7207	NW_003037890.1	+	1290543	1290592
Sma-1-7208	NS_000200.1	+	136973904	136973953
Sma-1-7209	NS_000200.1	-	66649786	66649737
Sma-1-7210	NW_003037942.1	+	394842	394891
Sma-1-7211	NS_000200.1	+	186154750	186154799
Sma-1-7212	NW_003026490.1	+	76910	76959
Sma-1-7213	NS_000200.1	+	267708626	267708675
Sma-1-7214	NW_003037941.1	-	104386	104337
Sma-1-7215	NS_000200.1	-	184838860	184838811
Sma-1-7216	NW_003026464.1	+	69346	69395
Sma-1-7217	NS_000200.1	+	203369655	203369704
Sma-1-7218	NW_003037995.1	+	32573	32622
Sma-1-7219	NW_003038014.1	-	357176	357127
Sma-1-7220	NS_000200.1	+	219722341	219722390
Sma-1-7221	NS_000200.1	-	230097865	230097816
Sma-1-7222	NW_003026458.1	-	313070	313021
Sma-1-7223	NW_003037829.1	+	1145052	1145101
Sma-1-7224	NW_003037975.1	+	182744	182793
Sma-1-7225	NW_003038102.1	-	33181	33132
Sma-1-7226	NS_000200.1	+	67045590	67045639
Sma-1-7227	NS_000200.1	+	205764968	205765017
Sma-1-7228	NS_000200.1	-	274712127	274712078
Sma-1-7229	NS_000200.1	-	102181300	102181251
Sma-1-7230	NW_003037793.1	+	3946332	3946381
Sma-1-7231	NW_003026517.1	-	409273	409224
Sma-1-7232	NS_000200.1	+	3946332	3946381
Sma-1-7233	NS_000200.1	-	300015282	300015233

Sma-1-7234	NW_003026468.1	-	265149	265100
Sma-1-7235	NS_000200.1	-	216459170	216459121
Sma-1-7236	NW_003037800.1	-	1525362	1525313
Sma-1-7237	NW_003037913.1	+	236967	237016
Sma-1-7238	NW_003037913.1	+	242051	242100
Sma-1-7239	NW_003037940.1	-	899383	899334
Sma-1-7240	NS_000200.1	+	158800718	158800767
Sma-1-7241	NS_000200.1	+	158805802	158805851
Sma-1-7242	NS_000200.1	-	184641513	184641464
Sma-1-7243	NS_000200.1	-	20154722	20154673
Sma-1-7244	NW_003037914.1	-	1706928	1706879
Sma-1-7245	NS_000200.1	-	161851178	161851129
Sma-1-7246	NW_003037816.1	-	286404	286355
Sma-1-7247	NS_000200.1	-	46495276	46495227
Sma-1-7248	NW_003037915.1	-	1199702	1199653
Sma-1-7249	NW_003038152.1	-	111986	111937
Sma-1-7250	NS_000200.1	-	292717710	292717661
Sma-1-7251	NS_000200.1	-	163099688	163099639
Sma-1-7252	NW_003032160.1	-	48078	48029
Sma-1-7253	NS_000200.1	-	381056164	381056115
Sma-1-7254	NW_003037792.1	+	235696	235745
Sma-1-7255	NS_000200.1	+	382673040	382673089
Sma-1-7256	NW_003037810.1	-	1210610	1210561
Sma-1-7257	NW_003037849.1	+	397443	397492
Sma-1-7258	NW_003037889.1	+	918748	918797
Sma-1-7259	NW_003038148.1	+	401719	401768
Sma-1-7260	NS_000200.1	+	91373716	91373765
Sma-1-7261	NS_000200.1	+	134695238	134695287
Sma-1-7262	NS_000200.1	+	290475703	290475752
Sma-1-7263	NS_000200.1	-	37815132	37815083
Sma-1-7264	NW_003037812.1	-	930666	930617
Sma-1-7265	NS_000200.1	-	40413405	40413356
Sma-1-7266	NW_003037917.1	+	626803	626852
Sma-1-7267	NS_000200.1	+	165663607	165663656
Sma-1-7268	NW_003038150.1	+	6304	6353
Sma-1-7269	NS_000200.1	+	291485042	291485091
Sma-1-7270	NW_003026605.1	-	21011	20962
Sma-1-7271	NS_000200.1	-	307699875	307699826
Sma-1-7272	NW_003031852.1	+	168	217
Sma-1-7273	NW_003037966.1	+	448507	448556
Sma-1-7274	NW_003026515.1	-	34934	34885
Sma-1-7275	NW_003038157.1	-	119871	119822
Sma-1-7276	NS_000200.1	+	199150370	199150419
Sma-1-7277	NS_000200.1	+	367147433	367147482
Sma-1-7278	NS_000200.1	-	295370791	295370742
Sma-1-7279	NS_000200.1	-	290679345	290679296
Sma-1-7280	NW_003037829.1	+	1149315	1149364
Sma-1-7281	NS_000200.1	+	67049853	67049902
Sma-1-7282	NW_003037916.1	-	143377	143328
Sma-1-7283	NS_000200.1	-	163336788	163336739
Sma-1-7284	NW_003037806.1	-	51031	50982
Sma-1-7285	NS_000200.1	-	28246245	28246196

Sma-1-7286	NW_003037806.1	-	1458001	1457952
Sma-1-7287	NW_003038024.1	+	583764	583813
Sma-1-7288	NS_000200.1	+	238480793	238480842
Sma-1-7289	NS_000200.1	-	29653215	29653166
Sma-1-7290	NW_003026472.1	-	112829	112780
Sma-1-7291	NW_003038131.1	+	111507	111556
Sma-1-7292	NS_000200.1	+	282073351	282073400
Sma-1-7293	NS_000200.1	-	236524821	236524772
Sma-1-7294	NW_003038235.1	+	46922	46971
Sma-1-7295	NW_003038161.1	+	414496	414545
Sma-1-7296	NS_000200.1	+	297784347	297784396
Sma-1-7297	NS_000200.1	+	313112513	313112562
Sma-1-7298	NW_003038070.1	+	454343	454392
Sma-1-7299	NW_003037889.1	+	954550	954599
Sma-1-7300	NW_003038050.1	-	189913	189864
Sma-1-7301	NS_000200.1	+	134731040	134731089
Sma-1-7302	NS_000200.1	+	264791584	264791633
Sma-1-7303	NS_000200.1	-	256464807	256464758
Sma-1-7304	NW_003035655.1	+	3282	3331
Sma-1-7305	NS_000200.1	+	330513880	330513929
Sma-1-7306	NW_003037930.1	-	580491	580442
Sma-1-7307	NS_000200.1	-	176438455	176438406
Sma-1-7308	NW_003037930.1	-	614210	614161
Sma-1-7309	NS_000200.1	-	176472174	176472125
Sma-1-7310	NW_003037930.1	-	799674	799625
Sma-1-7311	NW_003038035.1	-	189913	189864
Sma-1-7312	NS_000200.1	-	248051405	248051356
Sma-1-7313	NS_000200.1	-	176657638	176657589
Sma-1-7314	NW_003037932.1	-	507622	507573
Sma-1-7315	NS_000200.1	-	178040818	178040769
Sma-1-7316	NW_003037797.1	+	1606349	1606398
Sma-1-7317	NS_000200.1	+	12034209	12034258
Sma-1-7318	NW_003037992.1	-	139248	139199
Sma-1-7319	NS_000200.1	-	217980729	217980680
Sma-1-7320	NW_003037585.1	+	5033	5082
Sma-1-7321	NW_003038037.1	-	1682491	1682442
Sma-1-7322	NS_000200.1	+	372526204	372526253
Sma-1-7323	NS_000200.1	-	251837900	251837851
Sma-1-7324	NW_003038029.1	+	763003	763052
Sma-1-7325	NS_000200.1	+	242963904	242963953
Sma-1-7326	NW_003038157.1	+	280288	280337
Sma-1-7327	NS_000200.1	+	295531208	295531257
Sma-1-7328	NW_003037891.1	+	2447183	2447232
Sma-1-7329	NW_003037902.1	-	380167	380118
Sma-1-7330	NW_003037913.1	+	1471773	1471822
Sma-1-7331	NW_003037915.1	-	1093621	1093572
Sma-1-7332	NW_003038161.1	-	238708	238659
Sma-1-7333	NS_000200.1	+	140328461	140328510
Sma-1-7334	NS_000200.1	+	160035524	160035573
Sma-1-7335	NS_000200.1	-	297608559	297608510
Sma-1-7336	NS_000200.1	-	162993607	162993558
Sma-1-7337	NS_000200.1	-	147135401	147135352

Sma-1-7338	NW_003037933.1	-	45824	45775
Sma-1-7339	NS_000200.1	-	178458996	178458947
Sma-1-7340	NW_003037788.1	+	204190	204239
Sma-1-7341	NS_000200.1	+	380651428	380651477
Sma-1-7342	NW_003038003.1	+	360088	360137
Sma-1-7343	NS_000200.1	+	223859370	223859419
Sma-1-7344	NW_003038023.1	+	256760	256809
Sma-1-7345	NS_000200.1	+	237417051	237417100
Sma-1-7346	NW_003038033.1	-	393517	393468
Sma-1-7347	NW_003026510.1	+	80612	80661
Sma-1-7348	NS_000200.1	+	283520793	283520842
Sma-1-7349	NS_000200.1	-	246373519	246373470
Sma-1-7350	NW_003038037.1	+	1194181	1194230
Sma-1-7351	NS_000200.1	+	251349590	251349639
Sma-1-7352	NW_003037795.1	-	206588	206539
Sma-1-7353	NS_000200.1	-	6597471	6597422
Sma-1-7354	NW_003037851.1	-	507217	507168
Sma-1-7355	NW_003037891.1	+	2571087	2571136
Sma-1-7356	NS_000200.1	+	140452365	140452414
Sma-1-7357	NS_000200.1	-	93910527	93910478
Sma-1-7358	NW_003038146.1	-	294504	294455
Sma-1-7359	NS_000200.1	-	289275174	289275125
Sma-1-7360	NW_003038214.1	+	65286	65335
Sma-1-7361	NW_003038112.1	-	71043	70994
Sma-1-7362	NS_000200.1	+	306604922	306604971
Sma-1-7363	NS_000200.1	-	276526221	276526172
Sma-1-7364	NW_003037937.1	-	233216	233167
Sma-1-7365	NW_003037863.1	-	999591	999542
Sma-1-7366	NS_000200.1	-	181433125	181433076
Sma-1-7367	NS_000200.1	-	107757958	107757909
Sma-1-7368	NW_003038045.1	-	52041	51992
Sma-1-7369	NS_000200.1	-	254829652	254829603
Sma-1-7370	NW_003037938.1	+	15735	15784
Sma-1-7371	NS_000200.1	+	182052848	182052897
Sma-1-7372	NW_003037938.1	+	324614	324663
Sma-1-7373	NS_000200.1	+	182361727	182361776
Sma-1-7374	NW_003026510.1	+	317576	317625
Sma-1-7375	NS_000200.1	+	283757757	283757806
Sma-1-7376	NW_003026465.1	-	52778	52729
Sma-1-7377	NS_000200.1	-	207445372	207445323
Sma-1-7378	NW_003037819.1	+	740123	740172
Sma-1-7379	NW_003037964.1	+	451460	451509
Sma-1-7380	NS_000200.1	+	51828388	51828437
Sma-1-7381	NS_000200.1	+	198052982	198053031
Sma-1-7382	NW_003037941.1	-	473047	472998
Sma-1-7383	NS_000200.1	-	185207521	185207472
Sma-1-7384	NW_003037941.1	+	959379	959428
Sma-1-7385	NS_000200.1	+	185693853	185693902
Sma-1-7386	NW_003037944.1	-	522340	522291
Sma-1-7387	NS_000200.1	-	188743693	188743644
Sma-1-7388	NW_003037973.1	-	545782	545733
Sma-1-7389	NS_000200.1	-	204527509	204527460

Sma-1-7390	NW_003026517.1	+	331383	331432
Sma-1-7391	NS_000200.1	+	299937392	299937441
Sma-1-7392	NW_003021666.1	+	1709	1758
Sma-1-7393	NW_003031162.1	-	152	103
Sma-1-7394	NW_003033991.1	+	2362	2411
Sma-1-7395	NW_003038249.1	-	87788	87739
Sma-1-7396	NW_003037793.1	+	3190615	3190664
Sma-1-7397	NW_003037797.1	-	3397989	3397940
Sma-1-7398	NW_003037797.1	-	534863	534814
Sma-1-7399	NW_003037817.1	-	507803	507754
Sma-1-7400	NW_003037830.1	+	403644	403693
Sma-1-7401	NW_003037831.1	+	260453	260502
Sma-1-7402	NW_003037864.1	+	484344	484393
Sma-1-7403	NW_003037885.1	-	381206	381157
Sma-1-7404	NW_003037896.1	+	76319	76368
Sma-1-7405	NW_003038037.1	-	511068	511019
Sma-1-7406	NW_003038038.1	-	90811	90762
Sma-1-7407	NW_003038115.1	-	34912	34863
Sma-1-7408	NW_003038148.1	-	103696	103647
Sma-1-7409	NS_000200.1	+	3190615	3190664
Sma-1-7410	NS_000200.1	+	68294015	68294064
Sma-1-7411	NS_000200.1	+	69709755	69709804
Sma-1-7412	NS_000200.1	+	108976918	108976967
Sma-1-7413	NS_000200.1	+	142996742	142996791
Sma-1-7414	NS_000200.1	+	348263849	348263898
Sma-1-7415	NS_000200.1	+	363370158	363370207
Sma-1-7416	NS_000200.1	-	325880893	325880844
Sma-1-7417	NS_000200.1	-	316770041	316769992
Sma-1-7418	NS_000200.1	-	290177680	290177631
Sma-1-7419	NS_000200.1	-	277090207	277090158
Sma-1-7420	NS_000200.1	-	251994204	251994155
Sma-1-7421	NS_000200.1	-	250666477	250666428
Sma-1-7422	NS_000200.1	-	128479153	128479104
Sma-1-7423	NS_000200.1	-	47890155	47890106
Sma-1-7424	NS_000200.1	-	13825849	13825800
Sma-1-7425	NS_000200.1	-	10962723	10962674
Sma-1-7426	NW_003026460.1	+	394406	394455
Sma-1-7427	NW_003037884.1	-	78031	77982
Sma-1-7428	NS_000200.1	+	170916612	170916661
Sma-1-7429	NS_000200.1	-	126857696	126857647
Sma-1-7430	NW_003028350.1	+	931	980
Sma-1-7431	NW_003028670.1	+	36	85
Sma-1-7432	NW_003038044.1	-	125039	124990
Sma-1-7433	NS_000200.1	+	369427731	369427780
Sma-1-7434	NS_000200.1	+	374852193	374852242
Sma-1-7435	NS_000200.1	-	254749821	254749772
Sma-1-7436	NW_003038161.1	+	476772	476821
Sma-1-7437	NS_000200.1	+	297846623	297846672
Sma-1-7438	NW_003038268.1	+	8081	8130
Sma-1-7439	NS_000200.1	+	324905392	324905441
Sma-1-7440	NW_003037970.1	+	343654	343703
Sma-1-7441	NS_000200.1	+	201399844	201399893

Sma-1-7442	NW_003037970.1	+	410759	410808
Sma-1-7443	NS_000200.1	+	201466949	201466998
Sma-1-7444	NW_003038120.1	-	104212	104163
Sma-1-7445	NS_000200.1	-	278289435	278289386
Sma-1-7446	NW_003024095.1	+	429	478
Sma-1-7447	NW_003026456.1	-	420693	420644
Sma-1-7448	NW_003037890.1	+	392054	392103
Sma-1-7449	NS_000200.1	+	136075415	136075464
Sma-1-7450	NS_000200.1	+	348355156	348355205
Sma-1-7451	NS_000200.1	-	58343829	58343780
Sma-1-7452	NW_003037940.1	+	226281	226330
Sma-1-7453	NS_000200.1	+	183968411	183968460
Sma-1-7454	NW_003033027.1	-	3893	3844
Sma-1-7455	NS_000200.1	-	326218713	326218664
Sma-1-7456	NW_003037922.1	-	60559	60510
Sma-1-7457	NS_000200.1	-	169984556	169984507
Sma-1-7458	NW_003037807.1	+	1439677	1439726
Sma-1-7459	NS_000200.1	+	31701193	31701242
Sma-1-7460	NW_003035869.1	-	119157	119108
Sma-1-7461	NS_000200.1	-	335303357	335303308
Sma-1-7462	NW_003037817.1	+	885254	885303
Sma-1-7463	NW_003037890.1	+	920948	920997
Sma-1-7464	NS_000200.1	+	48267606	48267655
Sma-1-7465	NS_000200.1	+	136604309	136604358
Sma-1-7466	NW_003037797.1	+	2536828	2536877
Sma-1-7467	NS_000200.1	+	12964688	12964737
Sma-1-7468	NW_003038070.1	+	97961	98010
Sma-1-7469	NS_000200.1	+	264435202	264435251
Sma-1-7470	NW_003037809.1	-	1006202	1006153
Sma-1-7471	NS_000200.1	-	34670666	34670617
Sma-1-7472	NW_003038029.1	-	773293	773244
Sma-1-7473	NS_000200.1	-	242974194	242974145
Sma-1-7474	NW_003038462.1	+	45702	45751
Sma-1-7475	NS_000200.1	+	331413365	331413414
Sma-1-7476	NW_003032613.1	+	1524	1573
Sma-1-7477	NW_003026460.1	-	137023	136974
Sma-1-7478	NW_003037804.1	+	880703	880752
Sma-1-7479	NW_003037831.1	-	1137528	1137479
Sma-1-7480	NW_003037956.1	+	153446	153495
Sma-1-7481	NW_003038036.1	-	432741	432692
Sma-1-7482	NS_000200.1	+	26020723	26020772
Sma-1-7483	NS_000200.1	+	194209901	194209950
Sma-1-7484	NS_000200.1	+	350423653	350423702
Sma-1-7485	NS_000200.1	-	249366593	249366544
Sma-1-7486	NS_000200.1	-	170659229	170659180
Sma-1-7487	NS_000200.1	-	70586830	70586781
Sma-1-7488	NW_003037767.1	-	38494	38445
Sma-1-7489	NW_003037910.1	+	1169986	1170035
Sma-1-7490	NS_000200.1	+	155681961	155682010
Sma-1-7491	NS_000200.1	-	378086374	378086325
Sma-1-7492	NW_003026464.1	-	637409	637360
Sma-1-7493	NW_003038219.1	-	79273	79224

Sma-1-7494	NW_003038149.1	-	169810	169761
Sma-1-7495	NS_000200.1	-	308259694	308259645
Sma-1-7496	NS_000200.1	-	291198423	291198374
Sma-1-7497	NS_000200.1	-	203937718	203937669
Sma-1-7498	NW_003037944.1	-	180377	180329
Sma-1-7499	NS_000200.1	-	188401730	188401682
Sma-1-7500	NW_003034207.1	-	394	345
Sma-1-7501	NW_003037797.1	-	2506829	2506780
Sma-1-7502	NS_000200.1	-	326536375	326536326
Sma-1-7503	NS_000200.1	-	12934689	12934640
Sma-1-7504	NW_003020180.1	+	97	146
Sma-1-7505	NS_000200.1	+	371147048	371147097
Sma-1-7506	NW_003027814.1	-	585	536
Sma-1-7507	NW_003030818.1	-	920	871
Sma-1-7508	NW_003037815.1	-	350383	350334
Sma-1-7509	NW_003037935.1	+	536411	536460
Sma-1-7510	NW_003037938.1	+	170144	170193
Sma-1-7511	NW_003038077.1	-	107313	107264
Sma-1-7512	NS_000200.1	+	179952946	179952995
Sma-1-7513	NS_000200.1	+	182207257	182207306
Sma-1-7514	NS_000200.1	-	370309964	370309915
Sma-1-7515	NS_000200.1	-	365975909	365975860
Sma-1-7516	NS_000200.1	-	266878953	266878904
Sma-1-7517	NS_000200.1	-	45613374	45613325
Sma-1-7518	NW_003038020.1	+	255693	255742
Sma-1-7519	NS_000200.1	+	234223037	234223086
Sma-1-7520	NW_003038042.1	+	165123	165172
Sma-1-7521	NS_000200.1	+	254110722	254110771
Sma-1-7522	NW_003037925.1	-	103245	103196
Sma-1-7523	NS_000200.1	-	172505248	172505199
Sma-1-7524	NW_003037793.1	+	239541	239590
Sma-1-7525	NW_003037830.1	+	1454973	1455022
Sma-1-7526	NW_003037841.1	-	1970714	1970665
Sma-1-7527	NS_000200.1	+	239541	239590
Sma-1-7528	NS_000200.1	+	69345344	69345393
Sma-1-7529	NS_000200.1	-	84581895	84581846
Sma-1-7530	NW_003037958.1	-	382050	382001
Sma-1-7531	NS_000200.1	-	195046234	195046185
Sma-1-7532	NW_003038067.1	-	145777	145728
Sma-1-7533	NS_000200.1	-	262999830	262999781
Sma-1-7534	NW_003038065.1	+	292645	292694
Sma-1-7535	NS_000200.1	+	262444262	262444311
Sma-1-7536	NW_003037803.1	-	36889	36840
Sma-1-7537	NW_003037882.1	+	189429	189478
Sma-1-7538	NW_003037967.1	-	492200	492151
Sma-1-7539	NW_003038111.1	+	39303	39352
Sma-1-7540	NW_003038130.1	-	244785	244736
Sma-1-7541	NS_000200.1	+	125481103	125481152
Sma-1-7542	NS_000200.1	+	276359066	276359115
Sma-1-7543	NS_000200.1	-	281799771	281799722
Sma-1-7544	NS_000200.1	-	199732171	199732122
Sma-1-7545	NS_000200.1	-	23537426	23537377

Sma-1-7546	NW_003038152.1	-	163377	163328
Sma-1-7547	NS_000200.1	-	292769101	292769052
Sma-1-7548	NW_003037975.1	-	366471	366422
Sma-1-7549	NW_003037980.1	-	1134535	1134486
Sma-1-7550	NS_000200.1	-	212095397	212095348
Sma-1-7551	NS_000200.1	-	205948695	205948646
Sma-1-7552	NW_003037962.1	+	120534	120583
Sma-1-7553	NW_003037847.1	-	1003576	1003527
Sma-1-7554	NS_000200.1	+	196197377	196197426
Sma-1-7555	NS_000200.1	-	89655306	89655257
Sma-1-7556	NW_003037993.1	-	203666	203617
Sma-1-7557	NS_000200.1	-	218390369	218390320
Sma-1-7558	NW_003037796.1	-	415100	415051
Sma-1-7559	NW_003037861.1	+	318177	318226
Sma-1-7560	NS_000200.1	+	105094631	105094680
Sma-1-7561	NS_000200.1	-	8624744	8624695
Sma-1-7562	NW_003024449.1	+	42	91
Sma-1-7563	NS_000200.1	+	365761512	365761561
Sma-1-7564	NW_003038002.1	-	142474	142425
Sma-1-7565	NW_003026616.1	+	188457	188506
Sma-1-7566	NW_003037796.1	+	1025047	1025096
Sma-1-7567	NW_003037825.1	+	1390419	1390468
Sma-1-7568	NW_003037837.1	-	460770	460721
Sma-1-7569	NW_003037883.1	+	316720	316769
Sma-1-7570	NW_003037903.1	-	417220	417171
Sma-1-7571	NW_003037975.1	+	749069	749118
Sma-1-7572	NW_003037979.1	-	702673	702624
Sma-1-7573	NW_003037994.1	+	281697	281746
Sma-1-7574	NW_003026481.1	-	157619	157570
Sma-1-7575	NW_003038083.1	+	448099	448148
Sma-1-7576	NW_003038150.1	+	166112	166161
Sma-1-7577	NW_003038155.1	+	482128	482177
Sma-1-7578	NS_000200.1	+	9234691	9234740
Sma-1-7579	NS_000200.1	+	60520201	60520250
Sma-1-7580	NS_000200.1	+	126380443	126380492
Sma-1-7581	NS_000200.1	+	206331293	206331342
Sma-1-7582	NS_000200.1	+	218813511	218813560
Sma-1-7583	NS_000200.1	+	270691436	270691485
Sma-1-7584	NS_000200.1	+	291644850	291644899
Sma-1-7585	NS_000200.1	+	294605246	294605295
Sma-1-7586	NS_000200.1	+	317545056	317545105
Sma-1-7587	NS_000200.1	-	255557603	255557554
Sma-1-7588	NS_000200.1	-	223262683	223262634
Sma-1-7589	NS_000200.1	-	210763693	210763644
Sma-1-7590	NS_000200.1	-	147971301	147971252
Sma-1-7591	NS_000200.1	-	77266628	77266579
Sma-1-7592	NW_003022382.1	+	1496	1545
Sma-1-7593	NW_003037896.1	-	156215	156166
Sma-1-7594	NW_003037917.1	+	1379398	1379447
Sma-1-7595	NS_000200.1	+	166416202	166416251
Sma-1-7596	NS_000200.1	+	364839817	364839866
Sma-1-7597	NS_000200.1	-	143076638	143076589

Sma-1-7598	NW_003038005.1	+	301413	301462
Sma-1-7599	NS_000200.1	+	224707367	224707416
Sma-1-7600	NW_003021096.1	-	159	110
Sma-1-7601	NW_003021302.1	-	1636	1587
Sma-1-7602	NW_003025204.1	-	1486	1437
Sma-1-7603	NW_003026502.1	-	5640	5591
Sma-1-7604	NW_003026628.1	-	4987	4938
Sma-1-7605	NW_003029722.1	-	935	886
Sma-1-7606	NW_003029802.1	-	1470	1421
Sma-1-7607	NW_003029993.1	-	1859	1810
Sma-1-7608	NW_003034555.1	+	8655	8704
Sma-1-7609	NW_003034571.1	-	1504	1455
Sma-1-7610	NW_003035663.1	-	5889	5840
Sma-1-7611	NW_003035928.1	+	1213	1262
Sma-1-7612	NW_003036308.1	+	1143	1192
Sma-1-7613	NW_003037874.1	+	172368	172417
Sma-1-7614	NW_003037876.1	+	389895	389944
Sma-1-7615	NW_003035880.1	+	165905	165954
Sma-1-7616	NW_003037670.1	+	1692	1741
Sma-1-7617	NW_003037293.1	+	1688	1737
Sma-1-7618	NW_003026611.1	+	256077	256126
Sma-1-7619	NW_003038002.1	-	23785	23736
Sma-1-7620	NW_003038381.1	-	7001	6952
Sma-1-7621	NW_003026618.1	-	219334	219285
Sma-1-7622	NW_003035576.1	+	12102	12151
Sma-1-7623	NW_003026460.1	+	157696	157745
Sma-1-7624	NW_003037790.1	+	233114	233163
Sma-1-7625	NW_003037790.1	-	241545	241496
Sma-1-7626	NW_003038258.1	+	120840	120889
Sma-1-7627	NW_003037929.1	+	12688	12737
Sma-1-7628	NW_003037937.1	+	582051	582100
Sma-1-7629	NW_003039005.1	-	49962	49913
Sma-1-7630	NW_003038250.1	+	39267	39316
Sma-1-7631	NW_003026456.1	+	422105	422154
Sma-1-7632	NW_003035881.1	+	451225	451274
Sma-1-7633	NW_003037791.1	+	496605	496654
Sma-1-7634	NW_003037793.1	+	1313949	1313998
Sma-1-7635	NW_003037793.1	+	3002683	3002732
Sma-1-7636	NW_003037793.1	-	4122349	4122300
Sma-1-7637	NW_003037793.1	-	2894335	2894286
Sma-1-7638	NW_003037793.1	-	2841680	2841631
Sma-1-7639	NW_003037793.1	-	312734	312685
Sma-1-7640	NW_003037795.1	+	1297757	1297806
Sma-1-7641	NW_003037795.1	-	603419	603370
Sma-1-7642	NW_003037796.1	+	1683974	1684023
Sma-1-7643	NW_003037797.1	+	1241621	1241670
Sma-1-7644	NW_003037797.1	+	1803982	1804031
Sma-1-7645	NW_003037798.1	+	487793	487842
Sma-1-7646	NW_003037798.1	+	2567621	2567670
Sma-1-7647	NW_003037801.1	+	616747	616796
Sma-1-7648	NW_003037802.1	-	1029474	1029425
Sma-1-7649	NW_003037803.1	+	346967	347016

Sma-1-7650	NW_003037803.1	-	1000845	1000796
Sma-1-7651	NW_003037804.1	-	228896	228847
Sma-1-7652	NW_003037805.1	-	1040248	1040199
Sma-1-7653	NW_003037806.1	+	693027	693076
Sma-1-7654	NW_003037806.1	-	72624	72575
Sma-1-7655	NW_003037807.1	+	798192	798241
Sma-1-7656	NW_003037807.1	+	835262	835311
Sma-1-7657	NW_003037807.1	+	853056	853105
Sma-1-7658	NW_003037807.1	+	1031197	1031246
Sma-1-7659	NW_003037807.1	-	857880	857831
Sma-1-7660	NW_003037809.1	+	694328	694377
Sma-1-7661	NW_003037809.1	+	2634283	2634332
Sma-1-7662	NW_003037809.1	+	2736626	2736675
Sma-1-7663	NW_003037809.1	-	1971050	1971001
Sma-1-7664	NW_003037810.1	-	1034340	1034291
Sma-1-7665	NW_003037811.1	+	734335	734384
Sma-1-7666	NW_003037811.1	-	332010	331961
Sma-1-7667	NW_003037813.1	+	711545	711594
Sma-1-7668	NW_003037814.1	-	1592234	1592185
Sma-1-7669	NW_003037816.1	+	73111	73160
Sma-1-7670	NW_003037819.1	+	493486	493535
Sma-1-7671	NW_003037823.1	-	782605	782556
Sma-1-7672	NW_003037823.1	-	277544	277495
Sma-1-7673	NW_003037825.1	-	480780	480731
Sma-1-7674	NW_003037827.1	+	94832	94881
Sma-1-7675	NW_003037830.1	-	1534344	1534295
Sma-1-7676	NW_003037831.1	-	897534	897485
Sma-1-7677	NW_003037833.1	+	284344	284393
Sma-1-7678	NW_003037833.1	-	950938	950889
Sma-1-7679	NW_003037834.1	+	727663	727712
Sma-1-7680	NW_003037838.1	+	905576	905625
Sma-1-7681	NW_003037838.1	-	85166	85117
Sma-1-7682	NW_003037839.1	-	936377	936328
Sma-1-7683	NW_003037848.1	-	1193149	1193100
Sma-1-7684	NW_003037849.1	+	242462	242511
Sma-1-7685	NW_003037850.1	+	1362961	1363010
Sma-1-7686	NW_003037850.1	-	342251	342202
Sma-1-7687	NW_003037854.1	+	1001623	1001672
Sma-1-7688	NW_003037859.1	+	661671	661720
Sma-1-7689	NW_003037863.1	+	275997	276046
Sma-1-7690	NW_003037863.1	+	391559	391608
Sma-1-7691	NW_003037863.1	+	665951	666000
Sma-1-7692	NW_003037863.1	+	668613	668662
Sma-1-7693	NW_003037863.1	-	1509211	1509162
Sma-1-7694	NW_003037864.1	-	643513	643464
Sma-1-7695	NW_003037865.1	+	545810	545859
Sma-1-7696	NW_003037870.1	-	442237	442188
Sma-1-7697	NW_003037871.1	+	342452	342501
Sma-1-7698	NW_003037884.1	-	443192	443143
Sma-1-7699	NW_003037887.1	+	175510	175559
Sma-1-7700	NW_003037888.1	+	990603	990652
Sma-1-7701	NW_003037890.1	+	1555381	1555430

Sma-1-7702	NW_003037890.1	-	2125184	2125135
Sma-1-7703	NW_003037899.1	-	429968	429919
Sma-1-7704	NW_003037901.1	-	863274	863225
Sma-1-7705	NW_003037901.1	-	853714	853665
Sma-1-7706	NW_003037902.1	-	195046	194997
Sma-1-7707	NW_003037904.1	-	436288	436239
Sma-1-7708	NW_003037906.1	-	36458	36409
Sma-1-7709	NW_003037907.1	+	83409	83458
Sma-1-7710	NW_003037911.1	+	675597	675646
Sma-1-7711	NW_003037912.1	-	1337254	1337205
Sma-1-7712	NW_003037912.1	-	57990	57941
Sma-1-7713	NW_003037914.1	+	288592	288641
Sma-1-7714	NW_003037914.1	-	434570	434521
Sma-1-7715	NW_003037916.1	+	1573574	1573623
Sma-1-7716	NW_003037924.1	-	365564	365515
Sma-1-7717	NW_003037926.1	+	232010	232059
Sma-1-7718	NW_003037930.1	+	923622	923671
Sma-1-7719	NW_003037930.1	-	925174	925125
Sma-1-7720	NW_003037932.1	-	517165	517116
Sma-1-7721	NW_003037938.1	+	522035	522084
Sma-1-7722	NW_003037939.1	-	578449	578400
Sma-1-7723	NW_003037942.1	-	427683	427634
Sma-1-7724	NW_003037943.1	+	403911	403960
Sma-1-7725	NW_003037944.1	+	611525	611574
Sma-1-7726	NW_003037944.1	-	858804	858755
Sma-1-7727	NW_003037945.1	+	1083554	1083603
Sma-1-7728	NW_003037968.1	+	199018	199067
Sma-1-7729	NW_003037972.1	+	591419	591468
Sma-1-7730	NW_003037975.1	+	703256	703305
Sma-1-7731	NW_003037978.1	-	122730	122681
Sma-1-7732	NW_003037980.1	+	377621	377670
Sma-1-7733	NW_003037980.1	-	377427	377378
Sma-1-7734	NW_003037982.1	-	234293	234244
Sma-1-7735	NW_003037994.1	+	299923	299972
Sma-1-7736	NW_003038010.1	-	466160	466111
Sma-1-7737	NW_003038014.1	-	416404	416355
Sma-1-7738	NW_003038018.1	-	523563	523514
Sma-1-7739	NW_003038020.1	-	57952	57903
Sma-1-7740	NW_003038021.1	-	481161	481112
Sma-1-7741	NW_003038025.1	+	131147	131196
Sma-1-7742	NW_003038026.1	-	761111	761062
Sma-1-7743	NW_003038028.1	+	672149	672198
Sma-1-7744	NW_003038029.1	-	676791	676742
Sma-1-7745	NW_003038031.1	+	440581	440630
Sma-1-7746	NW_003038033.1	+	119718	119767
Sma-1-7747	NW_003038034.1	+	566227	566276
Sma-1-7748	NW_003038034.1	-	564879	564830
Sma-1-7749	NW_003038038.1	-	926154	926105
Sma-1-7750	NW_003038230.1	+	205027	205076
Sma-1-7751	NW_003038248.1	+	71923	71972
Sma-1-7752	NW_003038251.1	+	221543	221592
Sma-1-7753	NW_003038261.1	-	744200	744151

Sma-1-7754	NW_003038505.1	+	147126	147175
Sma-1-7755	NW_003038517.1	+	116311	116360
Sma-1-7756	NW_003039127.1	+	1252019	1252068
Sma-1-7757	NW_003026511.1	-	350253	350204
Sma-1-7758	NW_003038044.1	-	85921	85872
Sma-1-7759	NW_003038053.1	+	289066	289115
Sma-1-7760	NW_003038058.1	+	387942	387991
Sma-1-7761	NW_003038064.1	+	123752	123801
Sma-1-7762	NW_003038064.1	-	60101	60052
Sma-1-7763	NW_003038067.1	-	93491	93442
Sma-1-7764	NW_003038083.1	-	478939	478890
Sma-1-7765	NW_003038114.1	-	175976	175927
Sma-1-7766	NW_003038128.1	-	32107	32058
Sma-1-7767	NW_003038133.1	+	177867	177916
Sma-1-7768	NW_003038138.1	-	173350	173301
Sma-1-7769	NW_003038148.1	-	115662	115613
Sma-1-7770	NW_003038151.1	+	161685	161734
Sma-1-7771	NW_003038161.1	-	196478	196429
Sma-1-7772	NW_003038163.1	+	39763	39812
Sma-1-7773	NW_003038163.1	-	510702	510653
Sma-1-7774	NW_003038191.1	+	66079	66128
Sma-1-7775	NS_000200.1	+	1313949	1313998
Sma-1-7776	NS_000200.1	+	3002683	3002732
Sma-1-7777	NS_000200.1	+	7688640	7688689
Sma-1-7778	NS_000200.1	+	9893618	9893667
Sma-1-7779	NS_000200.1	+	11669481	11669530
Sma-1-7780	NS_000200.1	+	12231842	12231891
Sma-1-7781	NS_000200.1	+	14746951	14747000
Sma-1-7782	NS_000200.1	+	16826779	16826828
Sma-1-7783	NS_000200.1	+	21706227	21706276
Sma-1-7784	NS_000200.1	+	23847504	23847553
Sma-1-7785	NS_000200.1	+	28888241	28888290
Sma-1-7786	NS_000200.1	+	31059708	31059757
Sma-1-7787	NS_000200.1	+	31096778	31096827
Sma-1-7788	NS_000200.1	+	31114572	31114621
Sma-1-7789	NS_000200.1	+	31292713	31292762
Sma-1-7790	NS_000200.1	+	34358792	34358841
Sma-1-7791	NS_000200.1	+	36298747	36298796
Sma-1-7792	NS_000200.1	+	36401090	36401139
Sma-1-7793	NS_000200.1	+	38904577	38904626
Sma-1-7794	NS_000200.1	+	41698408	41698457
Sma-1-7795	NS_000200.1	+	46281983	46282032
Sma-1-7796	NS_000200.1	+	51581751	51581800
Sma-1-7797	NS_000200.1	+	58345241	58345290
Sma-1-7798	NS_000200.1	+	62716973	62717022
Sma-1-7799	NS_000200.1	+	72475440	72475489
Sma-1-7800	NS_000200.1	+	73888149	73888198
Sma-1-7801	NS_000200.1	+	78904139	78904188
Sma-1-7802	NS_000200.1	+	91218735	91218784
Sma-1-7803	NS_000200.1	+	93348991	93349040
Sma-1-7804	NS_000200.1	+	97906044	97906093
Sma-1-7805	NS_000200.1	+	103340512	103340561

Sma-1-7806	NS_000200.1	+	107034364	107034413
Sma-1-7807	NS_000200.1	+	107149926	107149975
Sma-1-7808	NS_000200.1	+	107424318	107424367
Sma-1-7809	NS_000200.1	+	107426980	107427029
Sma-1-7810	NS_000200.1	+	110967729	110967778
Sma-1-7811	NS_000200.1	+	114329518	114329567
Sma-1-7812	NS_000200.1	+	117790190	117790239
Sma-1-7813	NS_000200.1	+	119597318	119597367
Sma-1-7814	NS_000200.1	+	120935531	120935580
Sma-1-7815	NS_000200.1	+	130639488	130639537
Sma-1-7816	NS_000200.1	+	133199095	133199144
Sma-1-7817	NS_000200.1	+	137238742	137238791
Sma-1-7818	NS_000200.1	+	151487898	151487947
Sma-1-7819	NS_000200.1	+	156462950	156462999
Sma-1-7820	NS_000200.1	+	160432842	160432891
Sma-1-7821	NS_000200.1	+	164766985	164767034
Sma-1-7822	NS_000200.1	+	170679902	170679951
Sma-1-7823	NS_000200.1	+	173400280	173400329
Sma-1-7824	NS_000200.1	+	175253466	175253515
Sma-1-7825	NS_000200.1	+	176781586	176781635
Sma-1-7826	NS_000200.1	+	181781960	181782009
Sma-1-7827	NS_000200.1	+	182559148	182559197
Sma-1-7828	NS_000200.1	+	187425444	187425493
Sma-1-7829	NS_000200.1	+	188832878	188832927
Sma-1-7830	NS_000200.1	+	190185518	190185567
Sma-1-7831	NS_000200.1	+	200116188	200116237
Sma-1-7832	NS_000200.1	+	203067123	203067172
Sma-1-7833	NS_000200.1	+	206285480	206285529
Sma-1-7834	NS_000200.1	+	211338483	211338532
Sma-1-7835	NS_000200.1	+	218831737	218831786
Sma-1-7836	NS_000200.1	+	238989392	238989441
Sma-1-7837	NS_000200.1	+	241979723	241979772
Sma-1-7838	NS_000200.1	+	244718339	244718388
Sma-1-7839	NS_000200.1	+	246099720	246099769
Sma-1-7840	NS_000200.1	+	247507983	247508032
Sma-1-7841	NS_000200.1	+	257649161	257649210
Sma-1-7842	NS_000200.1	+	260219142	260219191
Sma-1-7843	NS_000200.1	+	261960329	261960378
Sma-1-7844	NS_000200.1	+	283050049	283050098
Sma-1-7845	NS_000200.1	+	292068928	292068977
Sma-1-7846	NS_000200.1	+	298440043	298440092
Sma-1-7847	NS_000200.1	+	302553264	302553313
Sma-1-7848	NS_000200.1	+	310825861	310825910
Sma-1-7849	NS_000200.1	+	311490598	311490647
Sma-1-7850	NS_000200.1	+	316383190	316383239
Sma-1-7851	NS_000200.1	+	318091944	318091993
Sma-1-7852	NS_000200.1	+	318553315	318553364
Sma-1-7853	NS_000200.1	+	322659350	322659399
Sma-1-7854	NS_000200.1	+	327350861	327350910
Sma-1-7855	NS_000200.1	+	329002473	329002522
Sma-1-7856	NS_000200.1	+	336226463	336226512
Sma-1-7857	NS_000200.1	+	339406969	339407018

Sma-1-7858	NS_000200.1	+	340880799	340880848
Sma-1-7859	NS_000200.1	+	341515430	341515479
Sma-1-7860	NS_000200.1	+	343049026	343049075
Sma-1-7861	NS_000200.1	+	359604278	359604327
Sma-1-7862	NS_000200.1	+	365411291	365411340
Sma-1-7863	NS_000200.1	+	374840302	374840351
Sma-1-7864	NS_000200.1	+	381577286	381577335
Sma-1-7865	NS_000200.1	+	382202033	382202082
Sma-1-7866	NS_000200.1	-	381585717	381585668
Sma-1-7867	NS_000200.1	-	375807994	375807945
Sma-1-7868	NS_000200.1	-	371821499	371821450
Sma-1-7869	NS_000200.1	-	367926913	367926864
Sma-1-7870	NS_000200.1	-	366484324	366484275
Sma-1-7871	NS_000200.1	-	361392565	361392516
Sma-1-7872	NS_000200.1	-	358076903	358076854
Sma-1-7873	NS_000200.1	-	357394776	357394727
Sma-1-7874	NS_000200.1	-	331272654	331272605
Sma-1-7875	NS_000200.1	-	327675636	327675587
Sma-1-7876	NS_000200.1	-	327588990	327588941
Sma-1-7877	NS_000200.1	-	324974552	324974503
Sma-1-7878	NS_000200.1	-	324357851	324357802
Sma-1-7879	NS_000200.1	-	321974336	321974287
Sma-1-7880	NS_000200.1	-	298910982	298910933
Sma-1-7881	NS_000200.1	-	297566329	297566280
Sma-1-7882	NS_000200.1	-	290189646	290189597
Sma-1-7883	NS_000200.1	-	285728665	285728616
Sma-1-7884	NS_000200.1	-	284733305	284733256
Sma-1-7885	NS_000200.1	-	280750395	280750346
Sma-1-7886	NS_000200.1	-	276901881	276901832
Sma-1-7887	NS_000200.1	-	276042076	276042027
Sma-1-7888	NS_000200.1	-	270722276	270722227
Sma-1-7889	NS_000200.1	-	262947544	262947495
Sma-1-7890	NS_000200.1	-	261896678	261896629
Sma-1-7891	NS_000200.1	-	254710703	254710654
Sma-1-7892	NS_000200.1	-	252829547	252829498
Sma-1-7893	NS_000200.1	-	247506635	247506586
Sma-1-7894	NS_000200.1	-	242877692	242877643
Sma-1-7895	NS_000200.1	-	240268872	240268823
Sma-1-7896	NS_000200.1	-	235309736	235309687
Sma-1-7897	NS_000200.1	-	234025296	234025247
Sma-1-7898	NS_000200.1	-	232924413	232924364
Sma-1-7899	NS_000200.1	-	230157093	230157044
Sma-1-7900	NS_000200.1	-	227284383	227284334
Sma-1-7901	NS_000200.1	-	223143994	223143945
Sma-1-7902	NS_000200.1	-	213893488	213893439
Sma-1-7903	NS_000200.1	-	211338289	211338240
Sma-1-7904	NS_000200.1	-	209390740	209390691
Sma-1-7905	NS_000200.1	-	189080157	189080108
Sma-1-7906	NS_000200.1	-	186187591	186187542
Sma-1-7907	NS_000200.1	-	183448203	183448154
Sma-1-7908	NS_000200.1	-	178050361	178050312
Sma-1-7909	NS_000200.1	-	176783138	176783089

Sma-1-7910	NS_000200.1	-	172072522	172072473
Sma-1-7911	NS_000200.1	-	160578820	160578771
Sma-1-7912	NS_000200.1	-	158148829	158148780
Sma-1-7913	NS_000200.1	-	156869565	156869516
Sma-1-7914	NS_000200.1	-	150344237	150344188
Sma-1-7915	NS_000200.1	-	149150787	149150738
Sma-1-7916	NS_000200.1	-	146950280	146950231
Sma-1-7917	NS_000200.1	-	146711735	146711686
Sma-1-7918	NS_000200.1	-	146702175	146702126
Sma-1-7919	NS_000200.1	-	144949079	144949030
Sma-1-7920	NS_000200.1	-	137808545	137808496
Sma-1-7921	NS_000200.1	-	127222857	127222808
Sma-1-7922	NS_000200.1	-	117315902	117315853
Sma-1-7923	NS_000200.1	-	109136087	109136038
Sma-1-7924	NS_000200.1	-	108267578	108267529
Sma-1-7925	NS_000200.1	-	92328281	92328232
Sma-1-7926	NS_000200.1	-	90886220	90886171
Sma-1-7927	NS_000200.1	-	79894527	79894478
Sma-1-7928	NS_000200.1	-	78083729	78083680
Sma-1-7929	NS_000200.1	-	73142034	73141985
Sma-1-7930	NS_000200.1	-	70346836	70346787
Sma-1-7931	NS_000200.1	-	69424715	69424666
Sma-1-7932	NS_000200.1	-	59610562	59610513
Sma-1-7933	NS_000200.1	-	56699224	56699175
Sma-1-7934	NS_000200.1	-	56194163	56194114
Sma-1-7935	NS_000200.1	-	44576094	44576045
Sma-1-7936	NS_000200.1	-	38502252	38502203
Sma-1-7937	NS_000200.1	-	37638862	37638813
Sma-1-7938	NS_000200.1	-	35635514	35635465
Sma-1-7939	NS_000200.1	-	31119396	31119347
Sma-1-7940	NS_000200.1	-	28267838	28267789
Sma-1-7941	NS_000200.1	-	27598092	27598043
Sma-1-7942	NS_000200.1	-	25368916	25368867
Sma-1-7943	NS_000200.1	-	24501382	24501333
Sma-1-7944	NS_000200.1	-	23032268	23032219
Sma-1-7945	NS_000200.1	-	6994302	6994253
Sma-1-7946	NS_000200.1	-	4122349	4122300
Sma-1-7947	NS_000200.1	-	2894335	2894286
Sma-1-7948	NS_000200.1	-	2841680	2841631
Sma-1-7949	NS_000200.1	-	312734	312685
env-85	JCVI_SCAF_1096628084112	+	139	188
Sma-1-7950	NW_003038006.1	-	71278	71228
Sma-1-7951	NS_000200.1	-	224955762	224955712
Sma-1-7952	NW_003038009.1	+	238586	238635
Sma-1-7953	NS_000200.1	+	226612649	226612698
Sma-1-7954	NW_003038011.1	+	425468	425517
Sma-1-7955	NW_003038011.1	-	424753	424704
Sma-1-7956	NS_000200.1	+	227953118	227953167
Sma-1-7957	NS_000200.1	-	227952403	227952354
Sma-1-7958	NW_003038020.1	-	106616	106567
Sma-1-7959	NS_000200.1	-	234073960	234073911
Sma-1-7960	NW_003037800.1	-	1570095	1570046

Sma-1-7961	NS_000200.1	-	20199455	20199406
Sma-1-7962	NW_003038241.1	-	79473	79424
Sma-1-7963	NS_000200.1	-	314542178	314542129
Sma-1-7964	NW_003029512.1	-	324	275
Sma-1-7965	NW_003038198.1	+	7519	7568
Sma-1-7966	NW_003037817.1	-	988965	988916
Sma-1-7967	NS_000200.1	+	303438479	303438528
Sma-1-7968	NS_000200.1	-	362191529	362191480
Sma-1-7969	NS_000200.1	-	48371317	48371268
Sma-1-7970	NW_003038161.1	+	244927	244976
Sma-1-7971	NS_000200.1	+	297614778	297614827
Sma-1-7972	NW_003020651.1	-	643	594
Sma-1-7973	NW_003035436.1	-	1512	1463
Sma-1-7974	NW_003038420.1	-	16157	16108
Sma-1-7975	NW_003038256.1	+	225215	225264
Sma-1-7976	NW_003038070.1	+	171503	171552
Sma-1-7977	NW_003037797.1	-	1638676	1638627
Sma-1-7978	NW_003037799.1	-	742839	742790
Sma-1-7979	NW_003037805.1	-	1763	1714
Sma-1-7980	NW_003037809.1	-	688083	688034
Sma-1-7981	NW_003037814.1	-	2120766	2120717
Sma-1-7982	NW_003037814.1	-	807048	806999
Sma-1-7983	NW_003037838.1	+	350920	350969
Sma-1-7984	NW_003037841.1	-	932757	932708
Sma-1-7985	NW_003037847.1	+	354174	354223
Sma-1-7986	NW_003037866.1	+	1106772	1106821
Sma-1-7987	NW_003037887.1	-	1192361	1192312
Sma-1-7988	NW_003037888.1	-	1384196	1384147
Sma-1-7989	NW_003037924.1	-	313576	313527
Sma-1-7990	NW_003037928.1	-	907829	907780
Sma-1-7991	NW_003037978.1	-	69612	69563
Sma-1-7992	NW_003037980.1	+	1274182	1274231
Sma-1-7993	NW_003037982.1	-	473311	473262
Sma-1-7994	NW_003038239.1	-	7134	7085
Sma-1-7995	NW_003038518.1	+	906157	906206
Sma-1-7996	NS_000200.1	+	78349483	78349532
Sma-1-7997	NS_000200.1	+	89005904	89005953
Sma-1-7998	NS_000200.1	+	112594571	112594620
Sma-1-7999	NS_000200.1	+	212235044	212235093
Sma-1-8000	NS_000200.1	+	264508744	264508793
Sma-1-8001	NS_000200.1	+	320959041	320959090
Sma-1-8002	NS_000200.1	+	342542990	342543039
Sma-1-8003	NS_000200.1	-	367464174	367464125
Sma-1-8004	NS_000200.1	-	360555039	360554990
Sma-1-8005	NS_000200.1	-	328938188	328938139
Sma-1-8006	NS_000200.1	-	313847916	313847867
Sma-1-8007	NS_000200.1	-	214132506	214132457
Sma-1-8008	NS_000200.1	-	209337622	209337573
Sma-1-8009	NS_000200.1	-	175231782	175231733
Sma-1-8010	NS_000200.1	-	172020534	172020485
Sma-1-8011	NS_000200.1	-	133592688	133592639
Sma-1-8012	NS_000200.1	-	131656339	131656290

Sma-1-8013	NS_000200.1	-	83543938	83543889
Sma-1-8014	NS_000200.1	-	45104626	45104577
Sma-1-8015	NS_000200.1	-	43790908	43790859
Sma-1-8016	NS_000200.1	-	34352547	34352498
Sma-1-8017	NS_000200.1	-	26559607	26559558
Sma-1-8018	NS_000200.1	-	17733193	17733144
Sma-1-8019	NS_000200.1	-	12066536	12066487
Sma-1-8020	NW_003031824.1	+	1129	1178
Sma-1-8021	NS_000200.1	+	367101584	367101633
Sma-1-8022	NW_003037833.1	+	902344	902393
Sma-1-8023	NS_000200.1	+	73093440	73093489
Sma-1-8024	NW_003037827.1	-	537437	537388
Sma-1-8025	NS_000200.1	-	63159578	63159529
Sma-1-8026	NW_003037827.1	+	367264	367313
Sma-1-8027	NS_000200.1	+	62989405	62989454
Sma-1-8028	NW_003037917.1	+	726027	726076
Sma-1-8029	NS_000200.1	+	165762831	165762880
Sma-1-8030	NW_003037822.1	-	1447190	1447141
Sma-1-8031	NS_000200.1	-	55755196	55755147
Sma-1-8032	NW_003037809.1	-	725364	725315
Sma-1-8033	NW_003037815.1	+	445526	445575
Sma-1-8034	NS_000200.1	+	45708517	45708566
Sma-1-8035	NS_000200.1	-	34389828	34389779
Sma-1-8036	NW_003026581.1	+	25889	25938
Sma-1-8037	NS_000200.1	+	302855877	302855926
Sma-1-8038	NW_003026515.1	+	269482	269531
Sma-1-8039	NS_000200.1	+	290913893	290913942
Sma-1-8040	NW_003026464.1	+	125437	125486
Sma-1-8041	NS_000200.1	+	203425746	203425795
Sma-1-8042	NW_003026594.1	-	18593	18544
Sma-1-8043	NS_000200.1	-	304962963	304962914
Sma-1-8044	NW_003023534.1	-	4182	4133
Sma-1-8045	NS_000200.1	-	373586856	373586807
Sma-1-8046	NW_003038005.1	+	454534	454583
Sma-1-8047	NS_000200.1	+	224860488	224860537
Sma-1-8048	NW_003038240.1	+	296867	296916
Sma-1-8049	NS_000200.1	+	314435798	314435847
Sma-1-8050	NW_003038268.1	+	11603	11652
Sma-1-8051	NS_000200.1	+	324908914	324908963
Sma-1-8052	NW_003036361.1	+	482	531
Sma-1-8053	NW_003037902.1	+	246752	246801
Sma-1-8054	NW_003038155.1	+	501308	501357
Sma-1-8055	NS_000200.1	+	147001986	147002035
Sma-1-8056	NS_000200.1	+	294624426	294624475
Sma-1-8057	NS_000200.1	+	359705286	359705335
Sma-1-8058	NW_003023365.1	+	349	398
Sma-1-8059	NW_003038256.1	-	269719	269670
Sma-1-8060	NW_003037937.1	-	201148	201099
Sma-1-8061	NW_003037894.1	-	171233	171184
Sma-1-8062	NW_003037933.1	+	340870	340919
Sma-1-8063	NS_000200.1	+	178754042	178754091
Sma-1-8064	NS_000200.1	+	373139623	373139672

Sma-1-8065	NS_000200.1	-	321003545	321003496
Sma-1-8066	NS_000200.1	-	181401057	181401008
Sma-1-8067	NS_000200.1	-	142067806	142067757
Sma-1-8068	NW_003037905.1	+	883456	883505
Sma-1-8069	NS_000200.1	+	150152468	150152517
Sma-1-8070	NW_003037835.1	-	149987	149938
Sma-1-8071	NS_000200.1	-	74546495	74546446
Sma-1-8072	NW_003037905.1	-	674734	674685
Sma-1-8073	NS_000200.1	-	149943746	149943697
Sma-1-8074	NW_003037980.1	+	1032573	1032622
Sma-1-8075	NS_000200.1	+	211993435	211993484
Sma-1-8076	NW_003037905.1	-	388485	388436
Sma-1-8077	NS_000200.1	-	149657497	149657448
Sma-1-8078	NW_003037943.1	+	1006917	1006966
Sma-1-8079	NS_000200.1	+	188028450	188028499
Sma-1-8080	NW_003037915.1	-	209659	209610
Sma-1-8081	NW_003037974.1	-	902865	902816
Sma-1-8082	NS_000200.1	-	205578171	205578122
Sma-1-8083	NS_000200.1	-	162109645	162109596
Sma-1-8084	NW_003037853.1	+	115358	115407
Sma-1-8085	NS_000200.1	+	95607409	95607458
Sma-1-8086	NW_003037918.1	-	508493	508444
Sma-1-8087	NS_000200.1	-	167075483	167075434
Sma-1-8088	NW_003037970.1	+	357055	357104
Sma-1-8089	NS_000200.1	+	201413245	201413294
Sma-1-8090	NW_003038007.1	-	285073	285024
Sma-1-8091	NS_000200.1	-	225533628	225533579
Sma-1-8092	NW_003037962.1	+	527671	527720
Sma-1-8093	NW_003038033.1	-	556241	556192
Sma-1-8094	NW_003038037.1	-	1178058	1178009
Sma-1-8095	NS_000200.1	+	196604514	196604563
Sma-1-8096	NS_000200.1	-	251333467	251333418
Sma-1-8097	NS_000200.1	-	246536243	246536194
Sma-1-8098	NW_003037815.1	-	452357	452308
Sma-1-8099	NS_000200.1	-	45715348	45715299
Sma-1-8100	NW_003037819.1	+	162733	162782
Sma-1-8101	NS_000200.1	+	51250998	51251047
Sma-1-8102	NW_003037827.1	+	1089358	1089407
Sma-1-8103	NS_000200.1	+	63711499	63711548
Sma-1-8104	NW_003037917.1	+	389624	389673
Sma-1-8105	NS_000200.1	+	165426428	165426477
Sma-1-8106	NW_003037937.1	-	420636	420587
Sma-1-8107	NS_000200.1	-	181620545	181620496
Sma-1-8108	NW_003038002.1	-	145991	145942
Sma-1-8109	NS_000200.1	-	223266200	223266151
Sma-1-8110	NW_003038261.1	+	202209	202258
Sma-1-8111	NS_000200.1	+	323815860	323815909
Sma-1-8112	NW_003038037.1	-	1559592	1559543
Sma-1-8113	NS_000200.1	-	251715001	251714952
Sma-1-8114	NW_003038242.1	+	34368	34417
Sma-1-8115	NW_003037937.1	+	153164	153213
Sma-1-8116	NW_003037844.1	+	653367	653416

Sma-1-8117	NS_000200.1	+	86836813	86836862
Sma-1-8118	NS_000200.1	+	181353073	181353122
Sma-1-8119	NS_000200.1	+	314733514	314733563
Sma-1-8120	NW_003038031.1	-	708004	707955
Sma-1-8121	NS_000200.1	-	244985762	244985713
Sma-1-8122	NW_003037791.1	-	421695	421646
Sma-1-8123	NW_003038153.1	+	6216	6265
Sma-1-8124	NS_000200.1	+	293068265	293068314
Sma-1-8125	NS_000200.1	-	382127123	382127074
Sma-1-8126	NW_003038014.1	-	121193	121144
Sma-1-8127	NS_000200.1	-	229861882	229861833
Sma-1-8128	NW_003038023.1	+	716974	717023
Sma-1-8129	NS_000200.1	+	237877265	237877314
Sma-1-8130	NW_003038478.1	+	32685	32734
Sma-1-8131	NS_000200.1	+	332607719	332607768
Sma-1-8132	NW_003028247.1	-	968	919
Sma-1-8133	NW_003028876.1	-	1301	1252
Sma-1-8134	NW_003033005.1	-	90	41
Sma-1-8135	NW_003037806.1	-	1930648	1930599
Sma-1-8136	NW_003037806.1	-	1928469	1928420
Sma-1-8137	NW_003037837.1	+	707935	707984
Sma-1-8138	NW_003037854.1	+	1646617	1646666
Sma-1-8139	NW_003037882.1	+	388142	388191
Sma-1-8140	NW_003037911.1	+	8177	8226
Sma-1-8141	NW_003037914.1	-	651007	650958
Sma-1-8142	NW_003037955.1	+	161573	161622
Sma-1-8143	NW_003038023.1	-	409888	409839
Sma-1-8144	NW_003026517.1	+	335783	335832
Sma-1-8145	NW_003038077.1	-	65683	65634
Sma-1-8146	NS_000200.1	+	77513793	77513842
Sma-1-8147	NS_000200.1	+	98551038	98551087
Sma-1-8148	NS_000200.1	+	125679816	125679865
Sma-1-8149	NS_000200.1	+	155795530	155795579
Sma-1-8150	NS_000200.1	+	193788307	193788356
Sma-1-8151	NS_000200.1	+	299941792	299941841
Sma-1-8152	NS_000200.1	-	374383386	374383337
Sma-1-8153	NS_000200.1	-	370134163	370134114
Sma-1-8154	NS_000200.1	-	326162457	326162408
Sma-1-8155	NS_000200.1	-	266837323	266837274
Sma-1-8156	NS_000200.1	-	237570179	237570130
Sma-1-8157	NS_000200.1	-	160795257	160795208
Sma-1-8158	NS_000200.1	-	30125862	30125813
Sma-1-8159	NS_000200.1	-	30123683	30123634
Sma-1-8160	NW_003037985.1	-	136452	136403
Sma-1-8161	NS_000200.1	-	215263796	215263747
Sma-1-8162	NW_003037818.1	+	215330	215379
Sma-1-8163	NS_000200.1	+	48930737	48930786
Sma-1-8164	NW_003036271.1	+	1115	1164
Sma-1-8165	NW_003037798.1	+	2576143	2576192
Sma-1-8166	NW_003038151.1	-	220781	220732
Sma-1-8167	NS_000200.1	+	16835301	16835350
Sma-1-8168	NS_000200.1	+	359527101	359527150

Sma-1-8169	NS_000200.1	-	292128024	292127975
Sma-1-8170	NW_003037914.1	-	37733	37684
Sma-1-8171	NS_000200.1	-	160181983	160181934
Sma-1-8172	NW_003037814.1	-	549843	549794
Sma-1-8173	NW_003037842.1	-	196549	196500
Sma-1-8174	NW_003037914.1	-	41044	40995
Sma-1-8175	NS_000200.1	-	160185294	160185245
Sma-1-8176	NS_000200.1	-	85295739	85295690
Sma-1-8177	NS_000200.1	-	43533703	43533654
Sma-1-8178	NW_003037795.1	+	653683	653732
Sma-1-8179	NS_000200.1	+	7044566	7044615
Sma-1-8180	NW_003037880.1	-	79892	79843
Sma-1-8181	NS_000200.1	-	123763450	123763401
Sma-1-8182	NW_003037810.1	+	112654	112703
Sma-1-8183	NS_000200.1	+	36717176	36717225
Sma-1-8184	NW_003037916.1	+	1782785	1782834
Sma-1-8185	NS_000200.1	+	164976196	164976245
Sma-1-8186	NW_003037925.1	+	63977	64026
Sma-1-8187	NS_000200.1	+	172465980	172466029
Sma-1-8188	NW_003037799.1	-	1510235	1510186
Sma-1-8189	NW_003038018.1	+	227449	227498
Sma-1-8190	NS_000200.1	+	232628299	232628348
Sma-1-8191	NS_000200.1	-	18500589	18500540
Sma-1-8192	NW_003038067.1	-	66594	66545
Sma-1-8193	NS_000200.1	-	262920647	262920598
Sma-1-8194	NW_003037846.1	-	886031	885982
Sma-1-8195	NS_000200.1	-	88520601	88520552
Sma-1-8196	NW_003037933.1	-	222502	222453
Sma-1-8197	NS_000200.1	-	178635674	178635625
Sma-1-8198	NW_003037942.1	+	96797	96846
Sma-1-8199	NS_000200.1	+	185856705	185856754
Sma-1-8200	NW_003029322.1	+	604	653
Sma-1-8201	NS_000200.1	+	357286913	357286962
Sma-1-8202	NW_003027744.1	+	488	537
Sma-1-8203	NS_000200.1	+	365868316	365868365
Sma-1-8204	NW_003038259.1	+	304063	304112
Sma-1-8205	NS_000200.1	+	323164662	323164711
Sma-1-8206	NW_003037806.1	-	1715965	1715916
Sma-1-8207	NS_000200.1	-	29911179	29911130
Sma-1-8208	NW_003038009.1	+	276093	276142
Sma-1-8209	NS_000200.1	+	226650156	226650205
Sma-1-8210	NW_003038012.1	-	358736	358687
Sma-1-8211	NS_000200.1	-	228682675	228682626
Sma-1-8212	NW_003037874.1	+	277714	277763
Sma-1-8213	NW_003038509.1	-	54332	54283
Sma-1-8214	NW_003038005.1	-	131389	131340
Sma-1-8215	NW_003037937.1	+	561828	561877
Sma-1-8216	NW_003032160.1	+	267916	267965
Sma-1-8217	NW_003037794.1	+	509311	509360
Sma-1-8218	NW_003037822.1	+	31318	31367
Sma-1-8219	NW_003037827.1	-	823389	823340
Sma-1-8220	NW_003037838.1	-	631649	631600

Sma-1-8221	NW_003037850.1	-	1341342	1341293
Sma-1-8222	NW_003037896.1	-	541679	541630
Sma-1-8223	NW_003037910.1	-	987956	987907
Sma-1-8224	NW_003038036.1	-	1130502	1130453
Sma-1-8225	NW_003038069.1	-	373738	373689
Sma-1-8226	NW_003038240.1	-	207506	207457
Sma-1-8227	NW_003026515.1	-	88559	88510
Sma-1-8228	NW_003038046.1	+	112780	112829
Sma-1-8229	NW_003038143.1	-	313638	313589
Sma-1-8230	NS_000200.1	+	4688906	4688955
Sma-1-8231	NS_000200.1	+	54339324	54339373
Sma-1-8232	NS_000200.1	+	119702664	119702713
Sma-1-8233	NS_000200.1	+	181761737	181761786
Sma-1-8234	NS_000200.1	+	255186162	255186211
Sma-1-8235	NS_000200.1	+	381276002	381276051
Sma-1-8236	NS_000200.1	-	337686784	337686735
Sma-1-8237	NS_000200.1	-	314346437	314346388
Sma-1-8238	NS_000200.1	-	290732970	290732921
Sma-1-8239	NS_000200.1	-	288073130	288073081
Sma-1-8240	NS_000200.1	-	264217144	264217095
Sma-1-8241	NS_000200.1	-	250064354	250064305
Sma-1-8242	NS_000200.1	-	224537343	224537294
Sma-1-8243	NS_000200.1	-	155499931	155499882
Sma-1-8244	NS_000200.1	-	143462102	143462053
Sma-1-8245	NS_000200.1	-	93327372	93327323
Sma-1-8246	NS_000200.1	-	78630212	78630163
Sma-1-8247	NS_000200.1	-	63445530	63445481
Sma-1-8248	NW_003037708.1	+	25961	26010
Sma-1-8249	NS_000200.1	+	375745883	375745932
Sma-1-8250	NW_003037988.1	-	47377	47328
Sma-1-8251	NS_000200.1	-	216041195	216041146
Sma-1-8252	NW_003037996.1	+	336238	336287
Sma-1-8253	NS_000200.1	+	220581114	220581163
Sma-1-8254	NW_003037962.1	-	465626	465577
Sma-1-8255	NW_003037871.1	+	95615	95664
Sma-1-8256	NW_003037908.1	+	279705	279754
Sma-1-8257	NW_003037909.1	-	603390	603341
Sma-1-8258	NW_003037914.1	+	45450	45499
Sma-1-8259	NS_000200.1	+	117543353	117543402
Sma-1-8260	NS_000200.1	+	152752836	152752885
Sma-1-8261	NS_000200.1	+	160189700	160189749
Sma-1-8262	NS_000200.1	-	196542469	196542420
Sma-1-8263	NS_000200.1	-	154145039	154144990
Sma-1-8264	NW_003026459.1	+	225848	225897
Sma-1-8265	NS_000200.1	+	141871121	141871170
Sma-1-8266	NW_003035642.1	-	22723	22674
Sma-1-8267	NS_000200.1	-	330280399	330280350
Sma-1-8268	NW_003038152.1	+	424667	424716
Sma-1-8269	NS_000200.1	+	293030391	293030440
Sma-1-8270	NW_003039127.1	+	1228478	1228527
Sma-1-8271	NS_000200.1	+	114305977	114306026
Sma-1-8272	NW_003037860.1	+	777063	777112

Sma-1-8273	NS_000200.1	+	104520091	104520140
Sma-1-8274	NW_003037868.1	+	113815	113864
Sma-1-8275	NS_000200.1	+	116085527	116085576
Sma-1-8276	NW_003037854.1	+	388459	388508
Sma-1-8277	NW_003037854.1	-	390563	390514
Sma-1-8278	NS_000200.1	+	97292880	97292929
Sma-1-8279	NS_000200.1	-	97294984	97294935
Sma-1-8280	NW_003037890.1	-	1326021	1325972
Sma-1-8281	NS_000200.1	-	137009382	137009333
Sma-1-8282	NW_003037853.1	+	650966	651015
Sma-1-8283	NS_000200.1	+	96143017	96143066
Sma-1-8284	NW_003037824.1	-	923066	923017
Sma-1-8285	NW_003037977.1	-	954611	954562
Sma-1-8286	NS_000200.1	-	209240631	209240582
Sma-1-8287	NS_000200.1	-	57854006	57853957
Sma-1-8288	NW_003037806.1	+	1900599	1900648
Sma-1-8289	NS_000200.1	+	30095813	30095862
Sma-1-8290	NW_003038230.1	+	152091	152140
Sma-1-8291	NS_000200.1	+	311437662	311437711
Sma-1-8292	NW_003037797.1	+	13100	13149
Sma-1-8293	NS_000200.1	+	10440960	10441009
Sma-1-8294	NW_003037940.1	+	915014	915063
Sma-1-8295	NS_000200.1	+	184657144	184657193
Sma-1-8296	NW_003035874.1	+	26436	26485
Sma-1-8297	NW_003026458.1	+	372185	372234
Sma-1-8298	NS_000200.1	+	102240415	102240464
Sma-1-8299	NS_000200.1	+	336003980	336004029
Sma-1-8300	NW_003037945.1	-	749844	749795
Sma-1-8301	NS_000200.1	-	189851808	189851759
Sma-1-8302	NW_003037969.1	+	209368	209417
Sma-1-8303	NS_000200.1	+	200815495	200815544
Sma-1-8304	NW_003037997.1	+	409372	409421
Sma-1-8305	NW_003038032.1	-	803486	803437
Sma-1-8306	NW_003038117.1	-	178538	178489
Sma-1-8307	NS_000200.1	+	221220814	221220863
Sma-1-8308	NS_000200.1	-	277496363	277496314
Sma-1-8309	NS_000200.1	-	245962646	245962597
Sma-1-8310	NW_003039127.1	-	2285651	2285602
Sma-1-8311	NS_000200.1	-	115363150	115363101
Sma-1-8312	NW_003036831.1	+	815	864
Sma-1-8313	NW_003037827.1	-	2204539	2204490
Sma-1-8314	NW_003037859.1	+	997648	997697
Sma-1-8315	NW_003037862.1	+	272407	272456
Sma-1-8316	NS_000200.1	+	103676489	103676538
Sma-1-8317	NS_000200.1	+	105924068	105924117
Sma-1-8318	NS_000200.1	+	347810507	347810556
Sma-1-8319	NS_000200.1	-	64826680	64826631
Sma-1-8320	NW_003026504.1	+	43705	43754
Sma-1-8321	NS_000200.1	+	276960471	276960520
Sma-1-8322	NW_003037986.1	-	49934	49885
Sma-1-8323	NS_000200.1	-	215466686	215466637
Sma-1-8324	NW_003023469.1	+	239	288

Sma-1-8325	NW_003037966.1	-	13533	13484
Sma-1-8326	NW_003038032.1	-	673911	673862
Sma-1-8327	NS_000200.1	+	373464649	373464698
Sma-1-8328	NS_000200.1	-	245833071	245833022
Sma-1-8329	NS_000200.1	-	198715396	198715347
Sma-1-8330	NW_003038073.1	-	297746	297697
Sma-1-8331	NW_003037812.1	+	1262740	1262789
Sma-1-8332	NW_003038015.1	-	467546	467497
Sma-1-8333	NW_003038036.1	-	1002842	1002793
Sma-1-8334	NS_000200.1	+	40745479	40745528
Sma-1-8335	NS_000200.1	-	265820179	265820130
Sma-1-8336	NS_000200.1	-	249936694	249936645
Sma-1-8337	NS_000200.1	-	230791036	230790987
Sma-1-8338	NW_003038037.1	+	51560	51609
Sma-1-8339	NS_000200.1	+	250206969	250207018
Sma-1-8340	NW_003028049.1	+	937	986
Sma-1-8341	NW_003034042.1	+	85	134
Sma-1-8342	NW_003038509.1	+	283359	283408
Sma-1-8343	NW_003037800.1	+	60097	60146
Sma-1-8344	NW_003037807.1	+	516474	516523
Sma-1-8345	NW_003037809.1	+	2298416	2298465
Sma-1-8346	NW_003037822.1	+	1592337	1592386
Sma-1-8347	NW_003037823.1	+	677651	677700
Sma-1-8348	NW_003037827.1	-	229814	229765
Sma-1-8349	NW_003037831.1	+	1240985	1241034
Sma-1-8350	NW_003037831.1	-	881024	880975
Sma-1-8351	NW_003037840.1	+	238351	238400
Sma-1-8352	NW_003037846.1	+	426720	426769
Sma-1-8353	NW_003037851.1	+	282914	282963
Sma-1-8354	NW_003037869.1	+	64360	64409
Sma-1-8355	NW_003037881.1	-	134762	134713
Sma-1-8356	NW_003037918.1	-	1845649	1845600
Sma-1-8357	NW_003037964.1	-	638506	638457
Sma-1-8358	NW_003038020.1	+	525306	525355
Sma-1-8359	NW_003038081.1	+	84602	84651
Sma-1-8360	NS_000200.1	+	18689457	18689506
Sma-1-8361	NS_000200.1	+	30777990	30778039
Sma-1-8362	NS_000200.1	+	35962880	35962929
Sma-1-8363	NS_000200.1	+	55900343	55900392
Sma-1-8364	NS_000200.1	+	56594270	56594319
Sma-1-8365	NS_000200.1	+	70690287	70690336
Sma-1-8366	NS_000200.1	+	80861675	80861724
Sma-1-8367	NS_000200.1	+	88061290	88061339
Sma-1-8368	NS_000200.1	+	93686224	93686273
Sma-1-8369	NS_000200.1	+	116536705	116536754
Sma-1-8370	NS_000200.1	+	234492650	234492699
Sma-1-8371	NS_000200.1	+	269246645	269246694
Sma-1-8372	NS_000200.1	+	337915811	337915860
Sma-1-8373	NS_000200.1	+	363574049	363574098
Sma-1-8374	NS_000200.1	+	369235392	369235441
Sma-1-8375	NS_000200.1	-	198240028	198239979
Sma-1-8376	NS_000200.1	-	168412639	168412590

Sma-1-8377	NS_000200.1	-	124566553	124566504
Sma-1-8378	NS_000200.1	-	70330326	70330277
Sma-1-8379	NS_000200.1	-	62851955	62851906
Sma-1-8380	NW_003037611.1	-	1239	1190
Sma-1-8381	NW_003037822.1	+	951765	951814
Sma-1-8382	NS_000200.1	+	55259771	55259820
Sma-1-8383	NS_000200.1	-	373369616	373369567
Sma-1-8384	NW_003037800.1	-	2029325	2029276
Sma-1-8385	NW_003037975.1	+	450448	450497
Sma-1-8386	NS_000200.1	+	206032672	206032721
Sma-1-8387	NS_000200.1	-	20658685	20658636
Sma-1-8388	NW_003037870.1	-	498898	498849
Sma-1-8389	NS_000200.1	-	117372563	117372514
Sma-1-8390	NW_003037853.1	-	590623	590574
Sma-1-8391	NS_000200.1	-	96082674	96082625
Sma-1-8392	NW_003037852.1	-	672649	672600
Sma-1-8393	NS_000200.1	-	95064544	95064495
Sma-1-8394	NW_003038155.1	+	406111	406160
Sma-1-8395	NS_000200.1	+	294529229	294529278
Sma-1-8396	NW_003037807.1	+	998470	998519
Sma-1-8397	NS_000200.1	+	31259986	31260035
Sma-1-8398	NW_003037917.1	-	632170	632121
Sma-1-8399	NS_000200.1	-	165668974	165668925
Sma-1-8400	NW_003037796.1	+	351488	351537
Sma-1-8401	NS_000200.1	+	8561132	8561181
Sma-1-8402	NW_003030849.1	-	1613	1564
Sma-1-8403	NS_000200.1	-	370366029	370365980
Sma-1-8404	NW_003038155.1	+	245657	245706
Sma-1-8405	NS_000200.1	+	294368775	294368824
Sma-1-8406	NW_003026462.1	+	41404	41453
Sma-1-8407	NW_003037793.1	-	3820752	3820703
Sma-1-8408	NW_003038012.1	+	369183	369232
Sma-1-8409	NW_003038014.1	-	243952	243903
Sma-1-8410	NS_000200.1	+	196708907	196708956
Sma-1-8411	NS_000200.1	+	228693122	228693171
Sma-1-8412	NS_000200.1	-	229984641	229984592
Sma-1-8413	NS_000200.1	-	3820752	3820703
Sma-1-8414	NW_003038079.1	-	34064	34015
Sma-1-8415	NS_000200.1	-	268164052	268164003
Sma-1-8416	NW_003029086.1	-	818	769
Sma-1-8417	NW_003038516.1	+	27797	27846
Sma-1-8418	NW_003026470.1	-	78414	78365
Sma-1-8419	NW_003037808.1	+	1405017	1405066
Sma-1-8420	NW_003037904.1	-	469512	469463
Sma-1-8421	NW_003037928.1	+	387023	387072
Sma-1-8422	NW_003038037.1	-	1158054	1158005
Sma-1-8423	NW_003038153.1	-	574955	574906
Sma-1-8424	NS_000200.1	+	33191654	33191703
Sma-1-8425	NS_000200.1	+	174710976	174711025
Sma-1-8426	NS_000200.1	+	340516202	340516251
Sma-1-8427	NS_000200.1	-	375355544	375355495
Sma-1-8428	NS_000200.1	-	293637004	293636955

Sma-1-8429	NS_000200.1	-	251313463	251313414
Sma-1-8430	NS_000200.1	-	219202383	219202334
Sma-1-8431	NS_000200.1	-	149184011	149183962
Sma-1-8432	NW_003039129.1	+	134737	134786
Sma-1-8433	NS_000200.1	+	229089545	229089594
Sma-1-8434	NW_003038249.1	+	64882	64931
Sma-1-8435	NS_000200.1	+	316747135	316747184
Sma-1-8436	NW_003037915.1	+	814209	814258
Sma-1-8437	NW_003038058.1	-	191416	191367
Sma-1-8438	NS_000200.1	+	162714195	162714244
Sma-1-8439	NS_000200.1	-	260022616	260022567
Sma-1-8440	NW_003037818.1	+	1310470	1310519
Sma-1-8441	NS_000200.1	+	50025877	50025926
Sma-1-8442	NW_003037917.1	-	1504993	1504944
Sma-1-8443	NS_000200.1	-	166541797	166541748
Sma-1-8444	NW_003037939.1	+	497390	497439
Sma-1-8445	NS_000200.1	+	183367144	183367193
Sma-1-8446	NW_003037804.1	-	763473	763424
Sma-1-8447	NW_003037939.1	+	558585	558634
Sma-1-8448	NW_003026504.1	+	69698	69747
Sma-1-8449	NS_000200.1	+	183428339	183428388
Sma-1-8450	NS_000200.1	+	276986464	276986513
Sma-1-8451	NS_000200.1	-	25903493	25903444
Sma-1-8452	NW_003037874.1	-	502061	502012
Sma-1-8453	NS_000200.1	-	119927011	119926962
Sma-1-8454	NW_003037875.1	-	327820	327771
Sma-1-8455	NW_003026604.1	-	64329	64280
Sma-1-8456	NW_003038079.1	+	51419	51468
Sma-1-8457	NS_000200.1	+	268181407	268181456
Sma-1-8458	NS_000200.1	-	307309886	307309837
Sma-1-8459	NS_000200.1	-	120356489	120356440
Sma-1-8460	NW_003037874.1	+	599052	599101
Sma-1-8461	NS_000200.1	+	120024002	120024051
Sma-1-8462	NW_003037914.1	-	1227845	1227796
Sma-1-8463	NS_000200.1	-	161372095	161372046
Sma-1-8464	NW_003037818.1	-	1310086	1310037
Sma-1-8465	NS_000200.1	-	50025493	50025444
Sma-1-8466	NW_003037868.1	-	278875	278826
Sma-1-8467	NS_000200.1	-	116250587	116250538
Sma-1-8468	NW_003037886.1	+	1167366	1167415
Sma-1-8469	NS_000200.1	+	130422933	130422982
Sma-1-8470	NW_003037864.1	+	23551	23600
Sma-1-8471	NS_000200.1	+	108516125	108516174
Sma-1-8472	NW_003037862.1	+	7987	8036
Sma-1-8473	NS_000200.1	+	105659648	105659697
Sma-1-8474	NW_003037862.1	+	237926	237975
Sma-1-8475	NS_000200.1	+	105889587	105889636
Sma-1-8476	NW_003037861.1	-	462320	462271
Sma-1-8477	NS_000200.1	-	105238774	105238725
Sma-1-8478	NW_003037831.1	+	1314129	1314178
Sma-1-8479	NS_000200.1	+	70763431	70763480
Sma-1-8480	NW_003037851.1	-	898718	898669

Sma-1-8481	NS_000200.1	-	94302028	94301979
Sma-1-8482	NW_003037912.1	+	1281277	1281326
Sma-1-8483	NS_000200.1	+	158092852	158092901
Sma-1-8484	NW_003037913.1	-	1151903	1151854
Sma-1-8485	NW_003037933.1	+	74441	74490
Sma-1-8486	NS_000200.1	+	178487613	178487662
Sma-1-8487	NS_000200.1	-	159715654	159715605
Sma-1-8488	NW_003037916.1	+	1815549	1815598
Sma-1-8489	NS_000200.1	+	165008960	165009009
Sma-1-8490	NW_003037797.1	+	1636765	1636814
Sma-1-8491	NS_000200.1	+	12064625	12064674
Sma-1-8492	NW_003037852.1	+	460288	460337
Sma-1-8493	NS_000200.1	+	94852183	94852232
Sma-1-8494	NW_003037917.1	-	416984	416935
Sma-1-8495	NS_000200.1	-	165453788	165453739
Sma-1-8496	NW_003037929.1	+	52729	52778
Sma-1-8497	NS_000200.1	+	175293507	175293556
Sma-1-8498	NW_003035879.1	-	128634	128585
Sma-1-8499	NW_003035879.1	-	128069	128020
Sma-1-8500	NS_000200.1	-	338095961	338095912
Sma-1-8501	NS_000200.1	-	338095396	338095347
Sma-1-8502	NW_003035870.1	+	48875	48924
Sma-1-8503	NS_000200.1	+	335414164	335414213
Sma-1-8504	NW_003026469.1	-	151215	151166
Sma-1-8505	NS_000200.1	-	217772874	217772825
Sma-1-8506	NW_003037812.1	-	876394	876345
Sma-1-8507	NW_003038020.1	-	687994	687945
Sma-1-8508	NS_000200.1	-	234655338	234655289
Sma-1-8509	NS_000200.1	-	40359133	40359084
Sma-1-8510	NW_003037795.1	+	879342	879391
Sma-1-8511	NS_000200.1	+	7270225	7270274
Sma-1-8512	NW_003037937.1	-	119645	119596
Sma-1-8513	NW_003037937.1	-	117876	117827
Sma-1-8514	NS_000200.1	-	181319554	181319505
Sma-1-8515	NS_000200.1	-	181317785	181317736
Sma-1-8516	NW_003037940.1	-	257423	257374
Sma-1-8517	NS_000200.1	-	183999553	183999504
Sma-1-8518	NW_003037978.1	-	336640	336591
Sma-1-8519	NS_000200.1	-	209604650	209604601
Sma-1-8520	NW_003038512.1	-	278348	278299
Sma-1-8521	NS_000200.1	-	338835045	338834996
Sma-1-8522	NW_003038003.1	-	580008	579959
Sma-1-8523	NS_000200.1	-	224079290	224079241
Sma-1-8524	NW_003038478.1	+	26572	26621
Sma-1-8525	NW_003038032.1	-	81002	80953
Sma-1-8526	NS_000200.1	+	332601606	332601655
Sma-1-8527	NS_000200.1	-	245240162	245240113
Sma-1-8528	NW_003020378.1	+	832	881
Sma-1-8529	NS_000200.1	+	355001029	355001078
Sma-1-8530	NW_003026474.1	-	164757	164708
Sma-1-8531	NS_000200.1	-	253363332	253363283
Sma-1-8532	NW_003037976.1	+	570613	570662

Sma-1-8533	NS_000200.1	+	207184928	207184977
Sma-1-8534	NW_003038478.1	-	26424	26375
Sma-1-8535	NS_000200.1	-	332601458	332601409
Sma-1-8536	NW_003037809.1	+	2786860	2786909
Sma-1-8537	NW_003037863.1	+	1447403	1447452
Sma-1-8538	NW_003038038.1	-	272410	272361
Sma-1-8539	NS_000200.1	+	36451324	36451373
Sma-1-8540	NS_000200.1	+	108205770	108205819
Sma-1-8541	NS_000200.1	-	252175803	252175754
Sma-1-8542	NW_003026617.1	+	184611	184660
Sma-1-8543	NW_003037797.1	-	16915	16866
Sma-1-8544	NW_003037816.1	-	2978	2929
Sma-1-8545	NW_003037824.1	+	186027	186076
Sma-1-8546	NW_003037824.1	-	70613	70564
Sma-1-8547	NW_003037826.1	-	1621818	1621769
Sma-1-8548	NW_003037830.1	-	189390	189341
Sma-1-8549	NW_003037840.1	+	1629036	1629085
Sma-1-8550	NW_003037896.1	+	533769	533818
Sma-1-8551	NW_003037904.1	+	115274	115323
Sma-1-8552	NW_003037941.1	-	649214	649165
Sma-1-8553	NW_003037976.1	-	582544	582495
Sma-1-8554	NW_003037978.1	-	342890	342841
Sma-1-8555	NW_003038038.1	+	320096	320145
Sma-1-8556	NW_003038261.1	+	553299	553348
Sma-1-8557	NW_003026510.1	+	77614	77663
Sma-1-8558	NW_003038187.1	+	56808	56857
Sma-1-8559	NS_000200.1	+	57116967	57117016
Sma-1-8560	NS_000200.1	+	82252360	82252409
Sma-1-8561	NS_000200.1	+	143454192	143454241
Sma-1-8562	NS_000200.1	+	148829773	148829822
Sma-1-8563	NS_000200.1	+	252223489	252223538
Sma-1-8564	NS_000200.1	+	283517795	283517844
Sma-1-8565	NS_000200.1	+	302008666	302008715
Sma-1-8566	NS_000200.1	+	319755470	319755519
Sma-1-8567	NS_000200.1	+	324166950	324166999
Sma-1-8568	NS_000200.1	-	209610900	209610851
Sma-1-8569	NS_000200.1	-	207196859	207196810
Sma-1-8570	NS_000200.1	-	185383688	185383639
Sma-1-8571	NS_000200.1	-	68079761	68079712
Sma-1-8572	NS_000200.1	-	62621334	62621285
Sma-1-8573	NS_000200.1	-	57001553	57001504
Sma-1-8574	NS_000200.1	-	46211850	46211801
Sma-1-8575	NS_000200.1	-	10444775	10444726
Sma-1-8576	NW_003037977.1	+	805822	805871
Sma-1-8577	NS_000200.1	+	209091842	209091891
Sma-1-8578	NW_003038518.1	+	482910	482959
Sma-1-8579	NS_000200.1	+	342119743	342119792
Sma-1-8580	NW_003038028.1	+	153884	153933
Sma-1-8581	NS_000200.1	+	241461458	241461507
Sma-1-8582	NW_003038037.1	+	602815	602864
Sma-1-8583	NS_000200.1	+	250758224	250758273
Sma-1-8584	NW_003035578.1	+	8956	9005

Sma-1-8585	NW_003037839.1	-	1183502	1183453
Sma-1-8586	NW_003037858.1	-	818370	818321
Sma-1-8587	NW_003037864.1	+	27095	27144
Sma-1-8588	NW_003037896.1	-	443154	443105
Sma-1-8589	NW_003026481.1	+	41372	41421
Sma-1-8590	NW_003038074.1	+	111762	111811
Sma-1-8591	NS_000200.1	+	108519669	108519718
Sma-1-8592	NS_000200.1	+	255441356	255441405
Sma-1-8593	NS_000200.1	+	266003142	266003191
Sma-1-8594	NS_000200.1	+	329034287	329034336
Sma-1-8595	NS_000200.1	-	143363577	143363528
Sma-1-8596	NS_000200.1	-	101482646	101482597
Sma-1-8597	NS_000200.1	-	80141652	80141603
Sma-1-8598	NW_003038254.1	-	239264	239215
Sma-1-8599	NS_000200.1	-	320274822	320274773
Sma-1-8600	NW_003038011.1	+	65825	65874
Sma-1-8601	NS_000200.1	+	227593475	227593524
Sma-1-8602	NW_003029713.1	-	1049	1000
Sma-1-8603	NW_003035393.1	+	1005	1054
Sma-1-8604	NW_003026466.1	+	70136	70185
Sma-1-8605	NW_003037794.1	+	827786	827835
Sma-1-8606	NW_003038037.1	-	1497602	1497553
Sma-1-8607	NW_003038087.1	+	14958	15007
Sma-1-8608	NS_000200.1	+	5007381	5007430
Sma-1-8609	NS_000200.1	+	215337243	215337292
Sma-1-8610	NS_000200.1	+	273043359	273043408
Sma-1-8611	NS_000200.1	+	367391011	367391060
Sma-1-8612	NS_000200.1	-	366467997	366467948
Sma-1-8613	NS_000200.1	-	251653011	251652962
Sma-1-8614	NW_003037807.1	-	127814	127765
Sma-1-8615	NS_000200.1	-	30389330	30389281
Sma-1-8616	NW_003037824.1	-	847877	847828
Sma-1-8617	NS_000200.1	-	57778817	57778768
Sma-1-8618	NW_003037853.1	+	827594	827643
Sma-1-8619	NW_003037869.1	+	100747	100796
Sma-1-8620	NS_000200.1	+	96319645	96319694
Sma-1-8621	NS_000200.1	+	116573092	116573141
Sma-1-8622	NW_003037888.1	-	930552	930503
Sma-1-8623	NS_000200.1	-	133139044	133138995
Sma-1-8624	NW_003037888.1	+	930910	930959
Sma-1-8625	NS_000200.1	+	133139402	133139451
Sma-1-8626	NW_003037889.1	+	114958	115007
Sma-1-8627	NS_000200.1	+	133891448	133891497
Sma-1-8628	NW_003037889.1	+	365661	365710
Sma-1-8629	NS_000200.1	+	134142151	134142200
Sma-1-8630	NW_003037889.1	+	504981	505030
Sma-1-8631	NS_000200.1	+	134281471	134281520
Sma-1-8632	NW_003037854.1	-	1198812	1198763
Sma-1-8633	NS_000200.1	-	98103233	98103184
Sma-1-8634	NW_003037853.1	-	985219	985170
Sma-1-8635	NS_000200.1	-	96477270	96477221
Sma-1-8636	NW_003037853.1	-	477169	477120

Sma-1-8637	NS_000200.1	-	95969220	95969171
Sma-1-8638	NW_003037850.1	+	178036	178085
Sma-1-8639	NS_000200.1	+	92164066	92164115
Sma-1-8640	NW_003037849.1	+	88954	89003
Sma-1-8641	NS_000200.1	+	91065227	91065276
Sma-1-8642	NW_003037849.1	+	188962	189011
Sma-1-8643	NS_000200.1	+	91165235	91165284
Sma-1-8644	NW_003037891.1	+	457988	458037
Sma-1-8645	NS_000200.1	+	138339266	138339315
Sma-1-8646	NW_003037893.1	-	39646	39597
Sma-1-8647	NW_003037943.1	-	350971	350922
Sma-1-8648	NW_003038135.1	-	105089	105040
Sma-1-8649	NW_003038163.1	+	95542	95591
Sma-1-8650	NS_000200.1	+	298495822	298495871
Sma-1-8651	NS_000200.1	-	283977428	283977379
Sma-1-8652	NS_000200.1	-	187372504	187372455
Sma-1-8653	NS_000200.1	-	141153956	141153907
Sma-1-8654	NW_003037844.1	+	805125	805174
Sma-1-8655	NW_003038152.1	-	78669	78620
Sma-1-8656	NS_000200.1	+	86988571	86988620
Sma-1-8657	NS_000200.1	-	292684393	292684344
Sma-1-8658	NW_003037843.1	-	105626	105577
Sma-1-8659	NW_003037917.1	-	76683	76634
Sma-1-8660	NS_000200.1	-	165113487	165113438
Sma-1-8661	NS_000200.1	-	85744933	85744884
Sma-1-8662	NW_003037922.1	+	15510	15559
Sma-1-8663	NS_000200.1	+	169939507	169939556
Sma-1-8664	NW_003037918.1	+	352321	352370
Sma-1-8665	NS_000200.1	+	166919311	166919360
Sma-1-8666	NW_003037841.1	-	282034	281985
Sma-1-8667	NS_000200.1	-	82893215	82893166
Sma-1-8668	NW_003037916.1	+	1251223	1251272
Sma-1-8669	NS_000200.1	+	164444634	164444683
Sma-1-8670	NW_003037797.1	-	3383281	3383232
Sma-1-8671	NS_000200.1	-	13811141	13811092
Sma-1-8672	NW_003037797.1	-	3363854	3363805
Sma-1-8673	NS_000200.1	-	13791714	13791665
Sma-1-8674	NW_003037797.1	+	3386529	3386578
Sma-1-8675	NS_000200.1	+	13814389	13814438
Sma-1-8676	NW_003037875.1	+	400865	400914
Sma-1-8677	NW_003037913.1	+	479544	479593
Sma-1-8678	NW_003037913.1	-	479150	479101
Sma-1-8679	NS_000200.1	+	120429534	120429583
Sma-1-8680	NS_000200.1	+	159043295	159043344
Sma-1-8681	NS_000200.1	-	159042901	159042852
Sma-1-8682	NW_003037830.1	-	281391	281342
Sma-1-8683	NS_000200.1	-	68171762	68171713
Sma-1-8684	NW_003037843.1	-	335477	335428
Sma-1-8685	NS_000200.1	-	85974784	85974735
Sma-1-8686	NW_003037839.1	-	929150	929101
Sma-1-8687	NS_000200.1	-	79887300	79887251
Sma-1-8688	NW_003037839.1	+	599122	599171

Sma-1-8689	NS_000200.1	+	79557272	79557321
Sma-1-8690	NW_003037839.1	-	1094375	1094326
Sma-1-8691	NS_000200.1	-	80052525	80052476
Sma-1-8692	NW_003037835.1	+	599637	599686
Sma-1-8693	NS_000200.1	+	74996145	74996194
Sma-1-8694	NW_003037902.1	-	250115	250066
Sma-1-8695	NS_000200.1	-	147005349	147005300
Sma-1-8696	NW_003037933.1	-	512151	512102
Sma-1-8697	NS_000200.1	-	178925323	178925274
Sma-1-8698	NW_003037821.1	-	774329	774280
Sma-1-8699	NS_000200.1	-	53918813	53918764
Sma-1-8700	NW_003037903.1	-	457873	457824
Sma-1-8701	NS_000200.1	-	148011954	148011905
Sma-1-8702	NW_003037833.1	+	612899	612948
Sma-1-8703	NW_003037854.1	+	1516120	1516169
Sma-1-8704	NS_000200.1	+	72803995	72804044
Sma-1-8705	NS_000200.1	+	98420541	98420590
Sma-1-8706	NW_003037904.1	-	307468	307419
Sma-1-8707	NS_000200.1	-	149021967	149021918
Sma-1-8708	NW_003037831.1	+	603230	603279
Sma-1-8709	NW_003038238.1	+	120626	120675
Sma-1-8710	NS_000200.1	+	70052532	70052581
Sma-1-8711	NS_000200.1	+	313727407	313727456
Sma-1-8712	NW_003037913.1	-	1327082	1327033
Sma-1-8713	NS_000200.1	-	159890833	159890784
Sma-1-8714	NW_003037831.1	-	944490	944441
Sma-1-8715	NS_000200.1	-	70393792	70393743
Sma-1-8716	NW_003038069.1	+	226308	226357
Sma-1-8717	NS_000200.1	+	264069714	264069763
Sma-1-8718	NW_003038259.1	-	271310	271261
Sma-1-8719	NS_000200.1	-	323131909	323131860
Sma-1-8720	NW_003022267.1	-	764	715
Sma-1-8721	NS_000200.1	-	361061638	361061589
Sma-1-8722	NW_003038030.1	+	22513	22562
Sma-1-8723	NS_000200.1	+	243158380	243158429
Sma-1-8724	NW_003037968.1	-	331617	331568
Sma-1-8725	NS_000200.1	-	200248787	200248738
Sma-1-8726	NW_003026616.1	+	29924	29973
Sma-1-8727	NW_003038250.1	+	229949	229998
Sma-1-8728	NW_003037901.1	+	790014	790063
Sma-1-8729	NW_003037932.1	-	638688	638639
Sma-1-8730	NW_003037945.1	+	508072	508121
Sma-1-8731	NW_003038069.1	-	226089	226040
Sma-1-8732	NW_003038083.1	-	466745	466696
Sma-1-8733	NS_000200.1	+	146638475	146638524
Sma-1-8734	NS_000200.1	+	189610036	189610085
Sma-1-8735	NS_000200.1	+	317386523	317386572
Sma-1-8736	NS_000200.1	+	318282626	318282675
Sma-1-8737	NS_000200.1	-	270710082	270710033
Sma-1-8738	NS_000200.1	-	264069495	264069446
Sma-1-8739	NS_000200.1	-	178171884	178171835
Sma-1-8740	NW_003026506.1	-	18115	18066

Sma-1-8741	NS_000200.1	-	279579180	279579131
Sma-1-8742	NW_003037807.1	-	495516	495467
Sma-1-8743	NS_000200.1	-	30757032	30756983
Sma-1-8744	NW_003037873.1	-	73593	73544
Sma-1-8745	NS_000200.1	-	118812721	118812672
Sma-1-8746	NW_003037888.1	+	260903	260952
Sma-1-8747	NW_003037891.1	-	1026785	1026736
Sma-1-8748	NS_000200.1	+	132469395	132469444
Sma-1-8749	NS_000200.1	-	138908063	138908014
Sma-1-8750	NW_003037831.1	-	1168993	1168944
Sma-1-8751	NS_000200.1	-	70618295	70618246
Sma-1-8752	NW_003037905.1	+	829402	829451
Sma-1-8753	NS_000200.1	+	150098414	150098463
Sma-1-8754	NW_003038514.1	-	17602	17553
Sma-1-8755	NS_000200.1	-	339642141	339642092
Sma-1-8756	NW_003037908.1	-	402541	402492
Sma-1-8757	NS_000200.1	-	152875672	152875623
Sma-1-8758	NW_003037982.1	+	671975	672024
Sma-1-8759	NS_000200.1	+	214331170	214331219
Sma-1-8760	NW_003039127.1	+	1250310	1250359
Sma-1-8761	NS_000200.1	+	114327809	114327858
Sma-1-8762	NW_003037911.1	+	755992	756041
Sma-1-8763	NW_003037913.1	+	1006220	1006269
Sma-1-8764	NW_003038240.1	+	185660	185709
Sma-1-8765	NW_003038482.1	-	92904	92855
Sma-1-8766	NS_000200.1	+	156543345	156543394
Sma-1-8767	NS_000200.1	+	159569971	159570020
Sma-1-8768	NS_000200.1	+	314324591	314324640
Sma-1-8769	NS_000200.1	-	333070355	333070306
Sma-1-8770	NW_003037822.1	+	815468	815517
Sma-1-8771	NS_000200.1	+	55123474	55123523
Sma-1-8772	NW_003037813.1	+	1218817	1218866
Sma-1-8773	NS_000200.1	+	42205680	42205729
Sma-1-8774	NW_003038149.1	-	350854	350805
Sma-1-8775	NS_000200.1	-	291379467	291379418
Sma-1-8776	NW_003037820.1	+	223337	223386
Sma-1-8777	NW_003037917.1	+	505744	505793
Sma-1-8778	NS_000200.1	+	52496319	52496368
Sma-1-8779	NS_000200.1	+	165542548	165542597
Sma-1-8780	NW_003037809.1	+	1663178	1663227
Sma-1-8781	NS_000200.1	+	35327642	35327691
Sma-1-8782	NW_003037967.1	+	427170	427219
Sma-1-8783	NS_000200.1	+	199667141	199667190
Sma-1-8784	NW_003026598.1	+	8662	8711
Sma-1-8785	NS_000200.1	+	305778878	305778927
Sma-1-8786	NW_003038261.1	-	128714	128665
Sma-1-8787	NS_000200.1	-	323742365	323742316
Sma-1-8788	NW_003028415.1	-	388	339
Sma-1-8789	NW_003029350.1	+	1517	1566
Sma-1-8790	NW_003038000.1	+	34615	34664
Sma-1-8791	NW_003037797.1	-	2603352	2603303
Sma-1-8792	NW_003037829.1	-	1142533	1142484

Sma-1-8793	NW_003037830.1	-	923269	923220
Sma-1-8794	NW_003037871.1	+	110861	110910
Sma-1-8795	NW_003037905.1	-	631513	631464
Sma-1-8796	NW_003037917.1	+	248750	248799
Sma-1-8797	NW_003038037.1	+	564528	564577
Sma-1-8798	NW_003038074.1	+	9290	9339
Sma-1-8799	NW_003038074.1	+	56468	56517
Sma-1-8800	NS_000200.1	+	117558599	117558648
Sma-1-8801	NS_000200.1	+	165285554	165285603
Sma-1-8802	NS_000200.1	+	222505393	222505442
Sma-1-8803	NS_000200.1	+	250719937	250719986
Sma-1-8804	NS_000200.1	+	265900670	265900719
Sma-1-8805	NS_000200.1	+	265947848	265947897
Sma-1-8806	NS_000200.1	+	357350668	357350717
Sma-1-8807	NS_000200.1	-	369571633	369571584
Sma-1-8808	NS_000200.1	-	149900525	149900476
Sma-1-8809	NS_000200.1	-	68813640	68813591
Sma-1-8810	NS_000200.1	-	67043071	67043022
Sma-1-8811	NS_000200.1	-	13031212	13031163
Sma-1-8812	NW_003038067.1	+	102034	102083
Sma-1-8813	NS_000200.1	+	262956087	262956136
Sma-1-8814	NW_003037804.1	-	412049	412000
Sma-1-8815	NS_000200.1	-	25552069	25552020
Sma-1-8816	NW_003037793.1	-	3562025	3561976
Sma-1-8817	NS_000200.1	-	3562025	3561976
Sma-1-8818	NW_003032078.1	+	16605	16654
Sma-1-8819	NS_000200.1	+	377350694	377350743
Sma-1-8820	NW_003038014.1	+	138195	138244
Sma-1-8821	NS_000200.1	+	229878884	229878933
Sma-1-8822	NW_003029650.1	+	215	264
Sma-1-8823	NW_003037902.1	-	179184	179135
Sma-1-8824	NS_000200.1	+	366350275	366350324
Sma-1-8825	NS_000200.1	-	146934418	146934369
Sma-1-8826	NW_003038008.1	+	103351	103400
Sma-1-8827	NS_000200.1	+	225776447	225776496
Sma-1-8828	NW_003026462.1	+	301089	301138
Sma-1-8829	NW_003038038.1	+	1238938	1238987
Sma-1-8830	NS_000200.1	+	196968592	196968641
Sma-1-8831	NS_000200.1	+	253142331	253142380
Sma-1-8832	NW_003029807.1	-	18286	18237
Sma-1-8833	NW_003037789.1	+	46821	46870
Sma-1-8834	NW_003026514.1	+	142415	142464
Sma-1-8835	NS_000200.1	+	288601102	288601151
Sma-1-8836	NS_000200.1	+	380831441	380831490
Sma-1-8837	NS_000200.1	-	375967646	375967597
Sma-1-8838	NW_003037977.1	+	510458	510507
Sma-1-8839	NS_000200.1	+	208796478	208796527
Sma-1-8840	NW_003037872.1	+	718459	718508
Sma-1-8841	NW_003037872.1	-	718109	718060
Sma-1-8842	NS_000200.1	+	118675465	118675514
Sma-1-8843	NS_000200.1	-	118675115	118675066
Sma-1-8844	NW_003037928.1	-	293521	293472

Sma-1-8845	NW_003038149.1	-	69909	69860
Sma-1-8846	NS_000200.1	-	291098522	291098473
Sma-1-8847	NS_000200.1	-	174617474	174617425
Sma-1-8848	NW_003036956.1	+	1467	1516
Sma-1-8849	NS_000200.1	+	343552964	343553013
Sma-1-8850	NW_003038155.1	+	384550	384599
Sma-1-8851	NS_000200.1	+	294507668	294507717
Sma-1-8852	NW_003038135.1	-	231021	230972
Sma-1-8853	NS_000200.1	-	284103360	284103311
Sma-1-8854	NW_003037919.1	-	121350	121301
Sma-1-8855	NS_000200.1	-	168979279	168979230
Sma-1-8856	NW_003038087.1	+	312056	312105
Sma-1-8857	NS_000200.1	+	273340457	273340506
Sma-1-8858	NW_003037791.1	-	365558	365509
Sma-1-8859	NS_000200.1	-	382070986	382070937
Sma-1-8860	NW_003026456.1	+	658367	658416
Sma-1-8861	NS_000200.1	+	58581503	58581552
Sma-1-8862	NW_003038005.1	+	159414	159463
Sma-1-8863	NW_003037806.1	-	1902858	1902809
Sma-1-8864	NW_003037844.1	+	656391	656440
Sma-1-8865	NS_000200.1	+	86839837	86839886
Sma-1-8866	NS_000200.1	+	224565368	224565417
Sma-1-8867	NS_000200.1	-	30098072	30098023
Sma-1-8868	NW_003022520.1	+	2898	2947
Sma-1-8869	NS_000200.1	+	365102844	365102893
Sma-1-8870	NW_003037800.1	-	155788	155739
Sma-1-8871	NS_000200.1	-	18785148	18785099
Sma-1-8872	NW_003037818.1	-	912437	912388
Sma-1-8873	NS_000200.1	-	49627844	49627795
Sma-1-8874	NW_003037820.1	-	733788	733739
Sma-1-8875	NS_000200.1	-	53006770	53006721
Sma-1-8876	NW_003037914.1	+	1107144	1107193
Sma-1-8877	NS_000200.1	+	161251394	161251443
Sma-1-8878	NW_003026517.1	-	134018	133969
Sma-1-8879	NS_000200.1	-	299740027	299739978
Sma-1-8880	NW_003037800.1	-	620251	620202
Sma-1-8881	NS_000200.1	-	19249611	19249562
Sma-1-8882	NW_003038442.1	-	7190	7141
Sma-1-8883	NW_003037801.1	-	533584	533535
Sma-1-8884	NS_000200.1	-	329863243	329863194
Sma-1-8885	NS_000200.1	-	21623064	21623015
Sma-1-8886	NW_003026464.1	+	674371	674420
Sma-1-8887	NS_000200.1	+	203974680	203974729
Sma-1-8888	NW_003037809.1	-	265534	265485
Sma-1-8889	NS_000200.1	-	33929998	33929949
Sma-1-8890	NW_003037823.1	-	229708	229659
Sma-1-8891	NW_003038034.1	-	241768	241719
Sma-1-8892	NS_000200.1	-	247183524	247183475
Sma-1-8893	NS_000200.1	-	56146327	56146278
Sma-1-8894	NW_003037854.1	-	418523	418474
Sma-1-8895	NS_000200.1	-	97322944	97322895
Sma-1-8896	NW_003037913.1	+	390945	390994

Sma-1-8897	NS_000200.1	+	158954696	158954745
Sma-1-8898	NW_003037914.1	-	1690566	1690517
Sma-1-8899	NS_000200.1	-	161834816	161834767
Sma-1-8900	NW_003037632.1	+	3209	3258
Sma-1-8901	NS_000200.1	+	373977789	373977838
Sma-1-8902	NW_003037821.1	+	416421	416470
Sma-1-8903	NS_000200.1	+	53560905	53560954
Sma-1-8904	NW_003037937.1	+	318379	318428
Sma-1-8905	NW_003037822.1	+	1528151	1528200
Sma-1-8906	NS_000200.1	+	55836157	55836206
Sma-1-8907	NS_000200.1	+	181518288	181518337
Sma-1-8908	NW_003037806.1	-	749232	749183
Sma-1-8909	NS_000200.1	-	28944446	28944397
Sma-1-8910	NW_003037805.1	+	1046060	1046109
Sma-1-8911	NS_000200.1	+	27603904	27603953
Sma-1-8912	NW_003037918.1	+	2208964	2209013
Sma-1-8913	NS_000200.1	+	168775954	168776003
Sma-1-8914	NW_003038149.1	-	319483	319434
Sma-1-8915	NS_000200.1	-	291348096	291348047
Sma-1-8916	NW_003037812.1	+	278289	278338
Sma-1-8917	NW_003037862.1	-	833659	833610
Sma-1-8918	NW_003037961.1	+	176810	176859
Sma-1-8919	NS_000200.1	+	39761028	39761077
Sma-1-8920	NS_000200.1	+	195801052	195801101
Sma-1-8921	NS_000200.1	-	106485320	106485271
Sma-1-8922	NW_003020571.1	+	1754	1803
Sma-1-8923	NS_000200.1	+	360237654	360237703
Sma-1-8924	NW_003037992.1	+	231997	232046
Sma-1-8925	NW_003038247.1	-	102588	102539
Sma-1-8926	NS_000200.1	+	218073478	218073527
Sma-1-8927	NS_000200.1	-	316051316	316051267
Sma-1-8928	NW_003026609.1	+	169018	169067
Sma-1-8929	NS_000200.1	+	309824010	309824059
Sma-1-8930	NW_003037933.1	+	259719	259768
Sma-1-8931	NS_000200.1	+	178672891	178672940
Sma-1-8932	NW_003037793.1	-	3383885	3383836
Sma-1-8933	NW_003037883.1	-	190609	190560
Sma-1-8934	NW_003037887.1	+	538626	538675
Sma-1-8935	NW_003038037.1	-	61202	61153
Sma-1-8936	NS_000200.1	+	131002604	131002653
Sma-1-8937	NS_000200.1	-	250216611	250216562
Sma-1-8938	NS_000200.1	-	126254332	126254283
Sma-1-8939	NS_000200.1	-	3383885	3383836
Sma-1-8940	NW_003037793.1	-	2555701	2555652
Sma-1-8941	NS_000200.1	-	2555701	2555652
Sma-1-8942	NW_003037793.1	-	1228438	1228389
Sma-1-8943	NW_003037808.1	-	763421	763372
Sma-1-8944	NW_003037914.1	+	109340	109389
Sma-1-8945	NS_000200.1	+	160253590	160253639
Sma-1-8946	NS_000200.1	-	32550058	32550009
Sma-1-8947	NS_000200.1	-	1228438	1228389
Sma-1-8948	NW_003038001.1	-	55888	55839

Sma-1-8949	NS_000200.1	-	222765449	222765400
Sma-1-8950	NW_003026512.1	-	262596	262547
Sma-1-8951	NS_000200.1	-	285356636	285356587
Sma-1-8952	NW_003026458.1	+	354548	354597
Sma-1-8953	NW_003038080.1	-	9317	9268
Sma-1-8954	NS_000200.1	+	102222778	102222827
Sma-1-8955	NS_000200.1	-	268461927	268461878
Sma-1-8956	NW_003027990.1	-	383	334
Sma-1-8957	NW_003030773.1	-	1071	1022
Sma-1-8958	NW_003026464.1	-	352157	352108
Sma-1-8959	NW_003037793.1	+	1953950	1953999
Sma-1-8960	NW_003037796.1	-	202000	201951
Sma-1-8961	NW_003037810.1	+	969132	969181
Sma-1-8962	NW_003037824.1	+	29319	29368
Sma-1-8963	NW_003037830.1	-	244770	244721
Sma-1-8964	NW_003037832.1	+	563360	563409
Sma-1-8965	NW_003037863.1	+	399859	399908
Sma-1-8966	NW_003037880.1	-	118456	118407
Sma-1-8967	NW_003037900.1	-	315927	315878
Sma-1-8968	NW_003037905.1	-	430361	430312
Sma-1-8969	NW_003037918.1	+	2124131	2124180
Sma-1-8970	NW_003037925.1	-	532371	532322
Sma-1-8971	NW_003038024.1	+	692615	692664
Sma-1-8972	NW_003038029.1	-	311078	311029
Sma-1-8973	NW_003038042.1	+	24907	24956
Sma-1-8974	NW_003038156.1	-	506099	506050
Sma-1-8975	NS_000200.1	+	1953950	1953999
Sma-1-8976	NS_000200.1	+	37573654	37573703
Sma-1-8977	NS_000200.1	+	56960259	56960308
Sma-1-8978	NS_000200.1	+	71772791	71772840
Sma-1-8979	NS_000200.1	+	107158226	107158275
Sma-1-8980	NS_000200.1	+	168691121	168691170
Sma-1-8981	NS_000200.1	+	238589644	238589693
Sma-1-8982	NS_000200.1	+	253970506	253970555
Sma-1-8983	NS_000200.1	-	369125501	369125452
Sma-1-8984	NS_000200.1	-	366886017	366885968
Sma-1-8985	NS_000200.1	-	295238212	295238163
Sma-1-8986	NS_000200.1	-	242511979	242511930
Sma-1-8987	NS_000200.1	-	203652466	203652417
Sma-1-8988	NS_000200.1	-	172934374	172934325
Sma-1-8989	NS_000200.1	-	149699373	149699324
Sma-1-8990	NS_000200.1	-	145444793	145444744
Sma-1-8991	NS_000200.1	-	123802014	123801965
Sma-1-8992	NS_000200.1	-	68135141	68135092
Sma-1-8993	NS_000200.1	-	8411644	8411595
Sma-1-8994	NW_003038237.1	+	118190	118239
Sma-1-8995	NS_000200.1	+	313558109	313558158
Sma-1-8996	NW_003026458.1	+	510445	510494
Sma-1-8997	NW_003026458.1	-	510808	510759
Sma-1-8998	NS_000200.1	+	102378675	102378724
Sma-1-8999	NS_000200.1	-	102379038	102378989
Sma-1-9000	NW_003037918.1	-	463540	463491

Sma-1-9001	NS_000200.1	-	167030530	167030481
Sma-1-9002	NW_003037920.1	-	30329	30280
Sma-1-9003	NS_000200.1	-	169211136	169211087
Sma-1-9004	NW_003037921.1	+	22150	22199
Sma-1-9005	NS_000200.1	+	169552285	169552334
Sma-1-9006	NW_003038512.1	+	333870	333919
Sma-1-9007	NS_000200.1	+	338890567	338890616
Sma-1-9008	NW_003037923.1	-	374000	373951
Sma-1-9009	NW_003026484.1	+	61848	61897
Sma-1-9010	NS_000200.1	+	257024539	257024588
Sma-1-9011	NS_000200.1	-	171430213	171430164
Sma-1-9012	NW_003037800.1	+	1406862	1406911
Sma-1-9013	NS_000200.1	+	20036222	20036271
Sma-1-9014	NW_003037927.1	+	106173	106222
Sma-1-9015	NS_000200.1	+	174083536	174083585
Sma-1-9016	NW_003037797.1	-	3714122	3714073
Sma-1-9017	NW_003038177.1	-	52524	52475
Sma-1-9018	NS_000200.1	-	300944964	300944915
Sma-1-9019	NS_000200.1	-	14141982	14141933
Sma-1-9020	NW_003037797.1	+	2700053	2700102
Sma-1-9021	NS_000200.1	+	13127913	13127962
Sma-1-9022	NW_003037796.1	-	157577	157528
Sma-1-9023	NS_000200.1	-	8367221	8367172
Sma-1-9024	NW_003037839.1	-	1560112	1560063
Sma-1-9025	NW_003037878.1	+	196741	196790
Sma-1-9026	NW_003037938.1	-	657966	657917
Sma-1-9027	NW_003026482.1	-	55777	55728
Sma-1-9028	NW_003038064.1	+	80628	80677
Sma-1-9029	NS_000200.1	+	122250219	122250268
Sma-1-9030	NS_000200.1	+	261917205	261917254
Sma-1-9031	NS_000200.1	-	255843523	255843474
Sma-1-9032	NS_000200.1	-	182695079	182695030
Sma-1-9033	NS_000200.1	-	80518262	80518213
Sma-1-9034	NW_003037821.1	-	177703	177654
Sma-1-9035	NW_003038260.1	+	55886	55935
Sma-1-9036	NS_000200.1	+	323227661	323227710
Sma-1-9037	NS_000200.1	-	53322187	53322138
Sma-1-9038	NW_003037796.1	-	895150	895101
Sma-1-9039	NS_000200.1	-	9104794	9104745
Sma-1-9040	NW_003037935.1	+	897375	897424
Sma-1-9041	NS_000200.1	+	180313910	180313959
Sma-1-9042	NW_003035206.1	-	836	787
Sma-1-9043	NW_003037808.1	+	805478	805527
Sma-1-9044	NW_003037940.1	+	191411	191460
Sma-1-9045	NS_000200.1	+	32592115	32592164
Sma-1-9046	NS_000200.1	+	183933541	183933590
Sma-1-9047	NS_000200.1	-	370803424	370803375
Sma-1-9048	NW_003037796.1	+	1729821	1729870
Sma-1-9049	NW_003037806.1	-	1431944	1431895
Sma-1-9050	NW_003037891.1	-	1480476	1480427
Sma-1-9051	NW_003037918.1	-	490506	490457
Sma-1-9052	NW_003026517.1	-	177312	177263

Sma-1-9053	NS_000200.1	+	9939465	9939514
Sma-1-9054	NS_000200.1	-	299783321	299783272
Sma-1-9055	NS_000200.1	-	167057496	167057447
Sma-1-9056	NS_000200.1	-	139361754	139361705
Sma-1-9057	NS_000200.1	-	29627158	29627109
Sma-1-9058	NW_003035559.1	-	2127	2078
Sma-1-9059	NW_003037845.1	-	601722	601673
Sma-1-9060	NW_003037888.1	+	1387117	1387166
Sma-1-9061	NS_000200.1	+	133595609	133595658
Sma-1-9062	NS_000200.1	-	328427133	328427084
Sma-1-9063	NS_000200.1	-	87603625	87603576
Sma-1-9064	NW_003026592.1	-	137580	137531
Sma-1-9065	NS_000200.1	-	304257238	304257189
Sma-1-9066	NW_003038032.1	+	805023	805072
Sma-1-9067	NS_000200.1	+	245964183	245964232
Sma-1-9068	NW_003036115.1	+	285	334
Sma-1-9069	NW_003037974.1	-	722442	722393
Sma-1-9070	NS_000200.1	+	347309545	347309594
Sma-1-9071	NS_000200.1	-	205397748	205397699
Sma-1-9072	NW_003034600.1	-	1095	1046
Sma-1-9073	NS_000200.1	-	327828735	327828686
Sma-1-9074	NW_003037951.1	+	8537	8586
Sma-1-9075	NW_003037796.1	-	1730980	1730931
Sma-1-9076	NW_003037824.1	-	103485	103436
Sma-1-9077	NW_003037887.1	-	1515022	1514973
Sma-1-9078	NW_003038021.1	-	415619	415570
Sma-1-9079	NS_000200.1	+	192686262	192686311
Sma-1-9080	NS_000200.1	-	235244194	235244145
Sma-1-9081	NS_000200.1	-	131979000	131978951
Sma-1-9082	NS_000200.1	-	57034425	57034376
Sma-1-9083	NS_000200.1	-	9940624	9940575
Sma-1-9084	NW_003037922.1	+	254134	254183
Sma-1-9085	NS_000200.1	+	170178131	170178180
Sma-1-9086	NW_003037943.1	-	473206	473158
Sma-1-9087	NS_000200.1	-	187494739	187494691
Sma-1-9088	NW_003032243.1	+	1376	1425
Sma-1-9089	NW_003026592.1	+	13068	13117
Sma-1-9090	NW_003026619.1	+	242190	242239
Sma-1-9091	NS_000200.1	+	304132726	304132775
Sma-1-9092	NS_000200.1	+	322432934	322432983
Sma-1-9093	NS_000200.1	+	357987598	357987647
Sma-1-9094	NW_003031512.1	-	1476	1427
Sma-1-9095	NW_003037966.1	-	57950	57901
Sma-1-9096	NS_000200.1	-	350106788	350106739
Sma-1-9097	NS_000200.1	-	198759813	198759764
Sma-1-9098	NW_003028960.1	+	1426	1475
Sma-1-9099	NW_003037833.1	-	98561	98512
Sma-1-9100	NS_000200.1	+	374989613	374989662
Sma-1-9101	NS_000200.1	-	72289657	72289608
Sma-1-9102	NW_003038014.1	-	248098	248049
Sma-1-9103	NS_000200.1	-	229988787	229988738
Sma-1-9104	NW_003037856.1	-	626531	626482

Sma-1-9105	NS_000200.1	-	99984487	99984438
Sma-1-9106	NW_003037982.1	-	274113	274064
Sma-1-9107	NW_003038083.1	-	136231	136182
Sma-1-9108	NS_000200.1	-	270379568	270379519
Sma-1-9109	NS_000200.1	-	213933308	213933259
Sma-1-9110	NW_003038198.1	+	12055	12104
Sma-1-9111	NS_000200.1	+	303443015	303443064
Sma-1-9112	NW_003028816.1	+	389	438
Sma-1-9113	NW_003037997.1	+	161502	161551
Sma-1-9114	NW_003038028.1	-	347341	347292
Sma-1-9115	NW_003038261.1	-	958224	958175
Sma-1-9116	NW_003038077.1	-	129510	129461
Sma-1-9117	NW_003038157.1	+	241878	241927
Sma-1-9118	NS_000200.1	+	220972944	220972993
Sma-1-9119	NS_000200.1	+	295492798	295492847
Sma-1-9120	NS_000200.1	+	370000197	370000246
Sma-1-9121	NS_000200.1	-	324571875	324571826
Sma-1-9122	NS_000200.1	-	266901150	266901101
Sma-1-9123	NS_000200.1	-	241654915	241654866
Sma-1-9124	NW_003038043.1	-	155117	155068
Sma-1-9125	NW_003038147.1	+	292258	292307
Sma-1-9126	NS_000200.1	+	289820001	289820050
Sma-1-9127	NS_000200.1	-	254465159	254465110
Sma-1-9128	NW_003038025.1	+	561958	562007
Sma-1-9129	NS_000200.1	+	239420203	239420252
Sma-1-9130	NW_003038027.1	-	478644	478595
Sma-1-9131	NS_000200.1	-	240849358	240849309
Sma-1-9132	NW_003026468.1	+	387133	387182
Sma-1-9133	NW_003037793.1	+	764936	764985
Sma-1-9134	NW_003037794.1	-	1040344	1040295
Sma-1-9135	NW_003037797.1	-	2067419	2067370
Sma-1-9136	NW_003037809.1	-	849317	849268
Sma-1-9137	NW_003037813.1	+	369200	369249
Sma-1-9138	NW_003037813.1	-	368700	368651
Sma-1-9139	NW_003037863.1	-	953351	953302
Sma-1-9140	NW_003037886.1	-	250126	250077
Sma-1-9141	NW_003037889.1	+	645523	645572
Sma-1-9142	NW_003037940.1	+	80393	80442
Sma-1-9143	NW_003037944.1	+	198301	198350
Sma-1-9144	NW_003037978.1	+	171123	171172
Sma-1-9145	NW_003038008.1	+	385372	385421
Sma-1-9146	NW_003038014.1	-	109688	109639
Sma-1-9147	NW_003038025.1	+	564295	564344
Sma-1-9148	NW_003038032.1	+	204124	204173
Sma-1-9149	NW_003038034.1	-	377293	377244
Sma-1-9150	NW_003026512.1	+	205873	205922
Sma-1-9151	NW_003038178.1	-	14178	14129
Sma-1-9152	NS_000200.1	+	764936	764985
Sma-1-9153	NS_000200.1	+	41356063	41356112
Sma-1-9154	NS_000200.1	+	134422013	134422062
Sma-1-9155	NS_000200.1	+	183822523	183822572
Sma-1-9156	NS_000200.1	+	188419654	188419703

Sma-1-9157	NS_000200.1	+	209439133	209439182
Sma-1-9158	NS_000200.1	+	216581154	216581203
Sma-1-9159	NS_000200.1	+	226058468	226058517
Sma-1-9160	NS_000200.1	+	239422540	239422589
Sma-1-9161	NS_000200.1	+	245363284	245363333
Sma-1-9162	NS_000200.1	+	285299913	285299962
Sma-1-9163	NS_000200.1	-	301074923	301074874
Sma-1-9164	NS_000200.1	-	247319049	247319000
Sma-1-9165	NS_000200.1	-	229850377	229850328
Sma-1-9166	NS_000200.1	-	129505693	129505644
Sma-1-9167	NS_000200.1	-	107711718	107711669
Sma-1-9168	NS_000200.1	-	41355563	41355514
Sma-1-9169	NS_000200.1	-	34513781	34513732
Sma-1-9170	NS_000200.1	-	12495279	12495230
Sma-1-9171	NS_000200.1	-	5219939	5219890
Sma-1-9172	NW_003038131.1	+	158058	158107
Sma-1-9173	NS_000200.1	+	282119902	282119951
Sma-1-9174	NW_003039130.1	+	55922	55971
Sma-1-9175	NS_000200.1	+	317730533	317730582
Sma-1-9176	NW_003038243.1	+	106957	107006
Sma-1-9177	NW_003038261.1	+	309024	309073
Sma-1-9178	NS_000200.1	+	315045441	315045490
Sma-1-9179	NS_000200.1	+	323922675	323922724
Sma-1-9180	NW_003037793.1	-	3730856	3730807
Sma-1-9181	NW_003037839.1	-	776013	775964
Sma-1-9182	NW_003026474.1	+	67201	67250
Sma-1-9183	NS_000200.1	+	253265776	253265825
Sma-1-9184	NS_000200.1	-	79734163	79734114
Sma-1-9185	NS_000200.1	-	3730856	3730807
Sma-1-9186	NW_003026619.1	-	346078	346029
Sma-1-9187	NS_000200.1	-	322536822	322536773
Sma-1-9188	NW_003026469.1	+	203138	203187
Sma-1-9189	NS_000200.1	+	217824797	217824846
Sma-1-9190	NW_003037988.1	+	180512	180561
Sma-1-9191	NS_000200.1	+	216174330	216174379
Sma-1-9192	NW_003038227.1	-	113363	113314
Sma-1-9193	NS_000200.1	-	310394717	310394668
Sma-1-9194	NW_003038228.1	-	156452	156403
Sma-1-9195	NW_003037800.1	+	1603793	1603842
Sma-1-9196	NW_003037946.1	-	1020116	1020067
Sma-1-9197	NS_000200.1	+	20233153	20233202
Sma-1-9198	NS_000200.1	-	311029732	311029683
Sma-1-9199	NS_000200.1	-	191713976	191713927
Sma-1-9200	NW_003038022.1	-	682758	682709
Sma-1-9201	NS_000200.1	-	236363600	236363551
Sma-1-9202	NW_003038024.1	+	515150	515199
Sma-1-9203	NS_000200.1	+	238412179	238412228
Sma-1-9204	NW_003038024.1	-	682477	682428
Sma-1-9205	NS_000200.1	-	238579506	238579457
Sma-1-9206	NW_003038073.1	-	186234	186185
Sma-1-9207	NS_000200.1	-	265708667	265708618
Sma-1-9208	NW_003038119.1	+	19037	19086

Sma-1-9209	NS_000200.1	+	277926593	277926642
Sma-1-9210	NW_003038251.1	-	66649	66600
Sma-1-9211	NS_000200.1	-	318398421	318398372
Sma-1-9212	NW_003038032.1	+	142311	142360
Sma-1-9213	NS_000200.1	+	245301471	245301520
Sma-1-9214	NW_003038254.1	-	340050	340001
Sma-1-9215	NS_000200.1	-	320375608	320375559
Sma-1-9216	NW_003038161.1	-	393016	392967
Sma-1-9217	NS_000200.1	-	297762867	297762818
Sma-1-9218	NW_003037904.1	-	416469	416420
Sma-1-9219	NW_003037972.1	+	688598	688647
Sma-1-9220	NS_000200.1	+	203164302	203164351
Sma-1-9221	NS_000200.1	-	149130968	149130919
Sma-1-9222	NW_003037887.1	-	1127536	1127487
Sma-1-9223	NW_003038131.1	+	443604	443653
Sma-1-9224	NS_000200.1	+	282405448	282405497
Sma-1-9225	NS_000200.1	-	131591514	131591465
Sma-1-9226	NW_003037874.1	+	22864	22913
Sma-1-9227	NS_000200.1	+	119447814	119447863
Sma-1-9228	NW_003036385.1	-	1531	1482
Sma-1-9229	NS_000200.1	-	359785470	359785421
Sma-1-9230	NW_003034097.1	+	1002	1051
Sma-1-9231	NS_000200.1	+	363734673	363734722
Sma-1-9232	NW_003032014.1	-	621	572
Sma-1-9233	NW_003038369.1	-	7633	7584
Sma-1-9234	NW_003037867.1	+	19017	19066
Sma-1-9235	NW_003037929.1	+	19896	19945
Sma-1-9236	NW_003038244.1	-	406741	406692
Sma-1-9237	NW_003037798.1	+	1925526	1925575
Sma-1-9238	NW_003037798.1	-	1684504	1684455
Sma-1-9239	NW_003037813.1	-	851395	851346
Sma-1-9240	NW_003037833.1	+	511020	511069
Sma-1-9241	NW_003037851.1	+	790504	790553
Sma-1-9242	NW_003037860.1	+	65157	65206
Sma-1-9243	NW_003037866.1	+	1345932	1345981
Sma-1-9244	NW_003037905.1	-	644915	644866
Sma-1-9245	NW_003037912.1	-	74618	74569
Sma-1-9246	NW_003037917.1	+	810779	810828
Sma-1-9247	NW_003037925.1	-	191084	191035
Sma-1-9248	NW_003037940.1	+	138733	138782
Sma-1-9249	NW_003038012.1	+	366937	366986
Sma-1-9250	NW_003038024.1	+	874734	874783
Sma-1-9251	NW_003038031.1	+	49804	49853
Sma-1-9252	NW_003038037.1	+	427831	427880
Sma-1-9253	NW_003038257.1	-	300688	300639
Sma-1-9254	NW_003038046.1	+	60466	60515
Sma-1-9255	NW_003038114.1	+	158721	158770
Sma-1-9256	NW_003038116.1	+	45753	45802
Sma-1-9257	NW_003038146.1	+	528822	528871
Sma-1-9258	NS_000200.1	+	16184684	16184733
Sma-1-9259	NS_000200.1	+	72702116	72702165
Sma-1-9260	NS_000200.1	+	94193814	94193863

Sma-1-9261	NS_000200.1	+	103808185	103808234
Sma-1-9262	NS_000200.1	+	112833731	112833780
Sma-1-9263	NS_000200.1	+	115619836	115619885
Sma-1-9264	NS_000200.1	+	165847583	165847632
Sma-1-9265	NS_000200.1	+	175260674	175260723
Sma-1-9266	NS_000200.1	+	183880863	183880912
Sma-1-9267	NS_000200.1	+	228690876	228690925
Sma-1-9268	NS_000200.1	+	238771763	238771812
Sma-1-9269	NS_000200.1	+	244327562	244327611
Sma-1-9270	NS_000200.1	+	250583240	250583289
Sma-1-9271	NS_000200.1	+	255133848	255133897
Sma-1-9272	NS_000200.1	+	276884626	276884675
Sma-1-9273	NS_000200.1	+	277261031	277261080
Sma-1-9274	NS_000200.1	+	289509492	289509541
Sma-1-9275	NS_000200.1	-	353816693	353816644
Sma-1-9276	NS_000200.1	-	327116572	327116523
Sma-1-9277	NS_000200.1	-	321475016	321474967
Sma-1-9278	NS_000200.1	-	315551946	315551897
Sma-1-9279	NS_000200.1	-	172593087	172593038
Sma-1-9280	NS_000200.1	-	156886193	156886144
Sma-1-9281	NS_000200.1	-	149913927	149913878
Sma-1-9282	NS_000200.1	-	41838258	41838209
Sma-1-9283	NS_000200.1	-	15943662	15943613
Sma-1-9284	NW_003031251.1	-	751	699
Sma-1-9285	NS_000200.1	-	345610155	345610103
Sma-1-9286	NW_003030221.1	+	38	87
Sma-1-9287	NW_003038253.1	+	72603	72652
Sma-1-9288	NW_003038253.1	-	71583	71534
Sma-1-9289	NW_003037936.1	+	626903	626952
Sma-1-9290	NW_003038489.1	-	67851	67802
Sma-1-9291	NW_003037792.1	-	301044	300995
Sma-1-9292	NW_003037801.1	+	503789	503838
Sma-1-9293	NW_003037809.1	-	1713905	1713856
Sma-1-9294	NW_003037814.1	+	2055050	2055099
Sma-1-9295	NW_003037818.1	+	1665024	1665073
Sma-1-9296	NW_003037822.1	+	1607932	1607981
Sma-1-9297	NW_003037829.1	-	1306347	1306298
Sma-1-9298	NW_003037846.1	-	613946	613897
Sma-1-9299	NW_003037853.1	+	751340	751389
Sma-1-9300	NW_003037932.1	+	396884	396933
Sma-1-9301	NW_003037932.1	+	401130	401179
Sma-1-9302	NW_003037938.1	+	352370	352419
Sma-1-9303	NW_003037972.1	-	489724	489675
Sma-1-9304	NW_003037978.1	+	382146	382195
Sma-1-9305	NW_003038011.1	-	307563	307514
Sma-1-9306	NW_003038014.1	+	183249	183298
Sma-1-9307	NW_003038014.1	+	221528	221577
Sma-1-9308	NW_003038210.1	-	97426	97377
Sma-1-9309	NW_003038261.1	+	575196	575245
Sma-1-9310	NW_003038057.1	+	355696	355745
Sma-1-9311	NW_003038160.1	+	236802	236851
Sma-1-9312	NS_000200.1	+	21593269	21593318

Sma-1-9313	NS_000200.1	+	45038910	45038959
Sma-1-9314	NS_000200.1	+	50380431	50380480
Sma-1-9315	NS_000200.1	+	55915938	55915987
Sma-1-9316	NS_000200.1	+	96243391	96243440
Sma-1-9317	NS_000200.1	+	177930080	177930129
Sma-1-9318	NS_000200.1	+	177934326	177934375
Sma-1-9319	NS_000200.1	+	181066632	181066681
Sma-1-9320	NS_000200.1	+	182389483	182389532
Sma-1-9321	NS_000200.1	+	209650156	209650205
Sma-1-9322	NS_000200.1	+	229923938	229923987
Sma-1-9323	NS_000200.1	+	229962217	229962266
Sma-1-9324	NS_000200.1	+	259561512	259561561
Sma-1-9325	NS_000200.1	+	296988733	296988782
Sma-1-9326	NS_000200.1	+	319390400	319390449
Sma-1-9327	NS_000200.1	+	324188847	324188896
Sma-1-9328	NS_000200.1	+	362374952	362375001
Sma-1-9329	NS_000200.1	-	382738388	382738339
Sma-1-9330	NS_000200.1	-	333780134	333780085
Sma-1-9331	NS_000200.1	-	319389380	319389331
Sma-1-9332	NS_000200.1	-	305559325	305559276
Sma-1-9333	NS_000200.1	-	227835213	227835164
Sma-1-9334	NS_000200.1	-	202965428	202965379
Sma-1-9335	NS_000200.1	-	88248516	88248467
Sma-1-9336	NS_000200.1	-	67206885	67206836
Sma-1-9337	NS_000200.1	-	35378369	35378320
Sma-1-9338	NW_003027916.1	-	1721	1672
Sma-1-9339	NW_003026601.1	+	14915	14964
Sma-1-9340	NW_003037367.1	+	439	488
Sma-1-9341	NW_003038226.1	+	30984	31033
Sma-1-9342	NW_003038235.1	-	96395	96346
Sma-1-9343	NW_003037927.1	+	73635	73684
Sma-1-9344	NW_003026468.1	+	231088	231137
Sma-1-9345	NW_003037937.1	-	97785	97736
Sma-1-9346	NW_003037794.1	+	1888879	1888928
Sma-1-9347	NW_003037794.1	-	1886618	1886569
Sma-1-9348	NW_003037794.1	-	1261303	1261254
Sma-1-9349	NW_003037805.1	-	1559920	1559871
Sma-1-9350	NW_003037807.1	+	885864	885913
Sma-1-9351	NW_003037809.1	+	2080094	2080143
Sma-1-9352	NW_003037809.1	-	2080675	2080626
Sma-1-9353	NW_003037822.1	+	1109711	1109760
Sma-1-9354	NW_003037850.1	+	635929	635978
Sma-1-9355	NW_003037854.1	+	699493	699542
Sma-1-9356	NW_003037856.1	+	544418	544467
Sma-1-9357	NW_003037863.1	+	1509582	1509631
Sma-1-9358	NW_003037864.1	-	1143466	1143417
Sma-1-9359	NW_003037885.1	+	892349	892398
Sma-1-9360	NW_003037908.1	-	287660	287611
Sma-1-9361	NW_003037911.1	-	290629	290580
Sma-1-9362	NW_003037928.1	+	57707	57756
Sma-1-9363	NW_003037928.1	-	6507	6458
Sma-1-9364	NW_003037953.1	-	10356	10307

Sma-1-9365	NW_003037971.1	-	283488	283439
Sma-1-9366	NW_003037977.1	-	596825	596776
Sma-1-9367	NW_003037980.1	-	675969	675920
Sma-1-9368	NW_003038023.1	+	485109	485158
Sma-1-9369	NW_003038037.1	+	552368	552417
Sma-1-9370	NW_003038229.1	+	11681	11730
Sma-1-9371	NW_003038229.1	-	10123	10074
Sma-1-9372	NW_003038507.1	+	8441	8490
Sma-1-9373	NW_003026516.1	+	474600	474649
Sma-1-9374	NW_003038128.1	-	296298	296249
Sma-1-9375	NW_003038161.1	+	123088	123137
Sma-1-9376	NS_000200.1	+	6068474	6068523
Sma-1-9377	NS_000200.1	+	31147380	31147429
Sma-1-9378	NS_000200.1	+	35744558	35744607
Sma-1-9379	NS_000200.1	+	55417717	55417766
Sma-1-9380	NS_000200.1	+	92621959	92622008
Sma-1-9381	NS_000200.1	+	97603914	97603963
Sma-1-9382	NS_000200.1	+	99902374	99902423
Sma-1-9383	NS_000200.1	+	108267949	108267998
Sma-1-9384	NS_000200.1	+	128990296	128990345
Sma-1-9385	NS_000200.1	+	174050998	174051047
Sma-1-9386	NS_000200.1	+	174381660	174381709
Sma-1-9387	NS_000200.1	+	216425109	216425158
Sma-1-9388	NS_000200.1	+	237645400	237645449
Sma-1-9389	NS_000200.1	+	250707777	250707826
Sma-1-9390	NS_000200.1	+	297492939	297492988
Sma-1-9391	NS_000200.1	+	299582897	299582946
Sma-1-9392	NS_000200.1	+	306396524	306396573
Sma-1-9393	NS_000200.1	+	310062018	310062067
Sma-1-9394	NS_000200.1	+	311128903	311128952
Sma-1-9395	NS_000200.1	+	336677335	336677384
Sma-1-9396	NS_000200.1	+	367086188	367086237
Sma-1-9397	NS_000200.1	-	366149614	366149565
Sma-1-9398	NS_000200.1	-	313161986	313161937
Sma-1-9399	NS_000200.1	-	311127345	311127296
Sma-1-9400	NS_000200.1	-	281014586	281014537
Sma-1-9401	NS_000200.1	-	211636831	211636782
Sma-1-9402	NS_000200.1	-	208882845	208882796
Sma-1-9403	NS_000200.1	-	201905538	201905489
Sma-1-9404	NS_000200.1	-	192923407	192923358
Sma-1-9405	NS_000200.1	-	181297694	181297645
Sma-1-9406	NS_000200.1	-	174330460	174330411
Sma-1-9407	NS_000200.1	-	156077982	156077933
Sma-1-9408	NS_000200.1	-	152760791	152760742
Sma-1-9409	NS_000200.1	-	109636040	109635991
Sma-1-9410	NS_000200.1	-	35745139	35745090
Sma-1-9411	NS_000200.1	-	28117764	28117715
Sma-1-9412	NS_000200.1	-	6066213	6066164
Sma-1-9413	NS_000200.1	-	5440898	5440849
Sma-1-9414	NW_003026624.1	+	21462	21511
Sma-1-9415	NS_000200.1	+	324891632	324891681
Sma-1-9416	NW_003026472.1	-	126728	126679

Sma-1-9417	NS_000200.1	-	236538720	236538671
Sma-1-9418	NW_003026471.1	+	177497	177546
Sma-1-9419	NW_003037976.1	-	5915	5866
Sma-1-9420	NW_003038037.1	-	1282022	1281973
Sma-1-9421	NW_003039127.1	-	2512342	2512293
Sma-1-9422	NS_000200.1	+	222289730	222289779
Sma-1-9423	NS_000200.1	-	251437431	251437382
Sma-1-9424	NS_000200.1	-	206620230	206620181
Sma-1-9425	NS_000200.1	-	115589841	115589792
Sma-1-9426	NW_003025519.1	-	729	680
Sma-1-9427	NW_003032158.1	-	20334	20285
Sma-1-9428	NW_003038514.1	-	290746	290697
Sma-1-9429	NW_003038516.1	+	15007	15056
Sma-1-9430	NW_003038420.1	+	2934	2983
Sma-1-9431	NW_003026472.1	-	399439	399390
Sma-1-9432	NW_003026618.1	+	161825	161874
Sma-1-9433	NW_003026458.1	-	101810	101761
Sma-1-9434	NW_003037800.1	+	1879691	1879740
Sma-1-9435	NW_003037800.1	-	1556487	1556438
Sma-1-9436	NW_003037819.1	-	791759	791710
Sma-1-9437	NW_003037844.1	+	567106	567155
Sma-1-9438	NW_003037853.1	-	452293	452244
Sma-1-9439	NW_003037910.1	-	957759	957710
Sma-1-9440	NW_003037912.1	-	525480	525431
Sma-1-9441	NW_003037944.1	+	184262	184311
Sma-1-9442	NW_003037977.1	+	200836	200885
Sma-1-9443	NW_003038027.1	+	404105	404154
Sma-1-9444	NW_003038027.1	-	359897	359848
Sma-1-9445	NW_003038072.1	-	108599	108550
Sma-1-9446	NW_003038072.1	-	55074	55025
Sma-1-9447	NW_003039127.1	+	710804	710853
Sma-1-9448	NS_000200.1	+	20509051	20509100
Sma-1-9449	NS_000200.1	+	86750552	86750601
Sma-1-9450	NS_000200.1	+	113788303	113788352
Sma-1-9451	NS_000200.1	+	188405615	188405664
Sma-1-9452	NS_000200.1	+	208486856	208486905
Sma-1-9453	NS_000200.1	+	240774819	240774868
Sma-1-9454	NS_000200.1	+	321916827	321916876
Sma-1-9455	NS_000200.1	+	328924965	328925014
Sma-1-9456	NS_000200.1	+	340503412	340503461
Sma-1-9457	NS_000200.1	-	380752613	380752564
Sma-1-9458	NS_000200.1	-	356086267	356086218
Sma-1-9459	NS_000200.1	-	339915285	339915236
Sma-1-9460	NS_000200.1	-	265270827	265270778
Sma-1-9461	NS_000200.1	-	265217302	265217253
Sma-1-9462	NS_000200.1	-	240730611	240730562
Sma-1-9463	NS_000200.1	-	236811431	236811382
Sma-1-9464	NS_000200.1	-	157337055	157337006
Sma-1-9465	NS_000200.1	-	155469734	155469685
Sma-1-9466	NS_000200.1	-	101970040	101969991
Sma-1-9467	NS_000200.1	-	95944344	95944295
Sma-1-9468	NS_000200.1	-	51880024	51879975

Sma-1-9469	NS_000200.1	-	20185847	20185798
Sma-1-9470	NW_003025336.1	-	978	929
Sma-1-9471	NW_003025336.1	-	575	526
Sma-1-9472	NW_003025336.1	-	174	125
Sma-1-9473	NW_003029208.1	+	211	260
Sma-1-9474	NW_003030317.1	-	782	733
Sma-1-9475	NW_003030317.1	-	379	330
Sma-1-9476	NW_003030824.1	+	1280	1329
Sma-1-9477	NW_003036643.1	-	1243	1194
Sma-1-9478	NW_003036643.1	-	840	791
Sma-1-9479	NW_003036643.1	-	437	388
Sma-1-9480	NW_003039029.1	+	4071	4120
Sma-1-9481	NW_003038211.1	+	143022	143071
Sma-1-9482	NW_003037810.1	+	45783	45832
Sma-1-9483	NW_003037847.1	+	117492	117541
Sma-1-9484	NW_003037892.1	+	207102	207151
Sma-1-9485	NW_003037953.1	+	218691	218740
Sma-1-9486	NW_003037972.1	-	325643	325594
Sma-1-9487	NW_003037980.1	-	1245453	1245404
Sma-1-9488	NW_003038038.1	+	51859	51908
Sma-1-9489	NW_003026488.1	-	221636	221587
Sma-1-9490	NW_003026517.1	-	681293	681244
Sma-1-9491	NW_003026517.1	-	679901	679852
Sma-1-9492	NW_003038150.1	-	411436	411387
Sma-1-9493	NS_000200.1	+	36650305	36650354
Sma-1-9494	NS_000200.1	+	88769222	88769271
Sma-1-9495	NS_000200.1	+	140930329	140930378
Sma-1-9496	NS_000200.1	+	193131742	193131791
Sma-1-9497	NS_000200.1	+	251955252	251955301
Sma-1-9498	NS_000200.1	+	306163092	306163141
Sma-1-9499	NS_000200.1	+	357084776	357084825
Sma-1-9500	NS_000200.1	+	358819950	358819999
Sma-1-9501	NS_000200.1	+	370320079	370320128
Sma-1-9502	NS_000200.1	-	362663724	362663675
Sma-1-9503	NS_000200.1	-	362663321	362663272
Sma-1-9504	NS_000200.1	-	352533862	352533813
Sma-1-9505	NS_000200.1	-	352533459	352533410
Sma-1-9506	NS_000200.1	-	352533058	352533009
Sma-1-9507	NS_000200.1	-	351460847	351460798
Sma-1-9508	NS_000200.1	-	351460444	351460395
Sma-1-9509	NS_000200.1	-	351460041	351459992
Sma-1-9510	NS_000200.1	-	300287302	300287253
Sma-1-9511	NS_000200.1	-	300285910	300285861
Sma-1-9512	NS_000200.1	-	291890174	291890125
Sma-1-9513	NS_000200.1	-	263728130	263728081
Sma-1-9514	NS_000200.1	-	212206315	212206266
Sma-1-9515	NS_000200.1	-	202801347	202801298
Sma-1-9516	NW_003021533.1	-	502	453
Sma-1-9517	NW_003026873.1	+	701	750
Sma-1-9518	NW_003037888.1	+	224850	224899
Sma-1-9519	NW_003037905.1	-	460061	460012
Sma-1-9520	NW_003038023.1	-	730049	730000

Sma-1-9521	NS_000200.1	+	132433342	132433391
Sma-1-9522	NS_000200.1	+	345360203	345360252
Sma-1-9523	NS_000200.1	-	348033658	348033609
Sma-1-9524	NS_000200.1	-	237890340	237890291
Sma-1-9525	NS_000200.1	-	149729073	149729024
Sma-1-9526	NW_003020183.1	+	315	364
Sma-1-9527	NW_003020246.1	+	57	106
Sma-1-9528	NW_003020616.1	-	1624	1575
Sma-1-9529	NW_003021276.1	+	90	139
Sma-1-9530	NW_003021839.1	-	673	624
Sma-1-9531	NW_003021839.1	-	275	226
Sma-1-9532	NW_003022032.1	+	3462	3511
Sma-1-9533	NW_003022422.1	-	1115	1066
Sma-1-9534	NW_003022520.1	-	2104	2055
Sma-1-9535	NW_003022558.1	-	1396	1347
Sma-1-9536	NW_003023481.1	-	367	318
Sma-1-9537	NW_003024376.1	+	854	903
Sma-1-9538	NW_003024631.1	+	55	104
Sma-1-9539	NW_003024827.1	-	924	875
Sma-1-9540	NW_003024827.1	-	526	477
Sma-1-9541	NW_003024827.1	-	128	79
Sma-1-9542	NW_003025543.1	-	536	487
Sma-1-9543	NW_003025543.1	-	134	85
Sma-1-9544	NW_003025661.1	-	971	922
Sma-1-9545	NW_003026297.1	-	133	84
Sma-1-9546	NW_003026353.1	+	382	431
Sma-1-9547	NW_003026461.1	-	51875	51826
Sma-1-9548	NW_003026567.1	+	14673	14722
Sma-1-9549	NW_003026728.1	+	56	105
Sma-1-9550	NW_003026732.1	-	439	390
Sma-1-9551	NW_003027394.1	-	691	642
Sma-1-9552	NW_003029270.1	+	233	282
Sma-1-9553	NW_003029818.1	+	2933	2982
Sma-1-9554	NW_003030106.1	-	649	600
Sma-1-9555	NW_003031024.1	+	1173	1222
Sma-1-9556	NW_003031945.1	+	276	325
Sma-1-9557	NW_003032158.1	-	29640	29591
Sma-1-9558	NW_003032444.1	-	688	639
Sma-1-9559	NW_003032444.1	-	287	238
Sma-1-9560	NW_003032991.1	+	47	96
Sma-1-9561	NW_003033041.1	-	265	216
Sma-1-9562	NW_003033917.1	+	287	336
Sma-1-9563	NW_003034071.1	-	937	888
Sma-1-9564	NW_003034601.1	-	2594	2545
Sma-1-9565	NW_003034607.1	-	4265	4216
Sma-1-9566	NW_003035148.1	-	1719	1670
Sma-1-9567	NW_003035192.1	+	1024	1073
Sma-1-9568	NW_003035209.1	-	1017	968
Sma-1-9569	NW_003035219.1	-	781	732
Sma-1-9570	NW_003035219.1	-	375	326
Sma-1-9571	NW_003035403.1	+	638	687
Sma-1-9572	NW_003035417.1	+	707	756

Sma-1-9573	NW_003035623.1	-	901	852
Sma-1-9574	NW_003035641.1	+	5648	5697
Sma-1-9575	NW_003035693.1	+	123	172
Sma-1-9576	NW_003035707.1	-	2746	2697
Sma-1-9577	NW_003036064.1	-	87	38
Sma-1-9578	NW_003036176.1	+	2437	2486
Sma-1-9579	NW_003036786.1	-	1324	1275
Sma-1-9580	NW_003026617.1	+	48677	48726
Sma-1-9581	NW_003035872.1	+	108444	108493
Sma-1-9582	NW_003038253.1	+	62592	62641
Sma-1-9583	NW_003038262.1	-	42005	41956
Sma-1-9584	NW_003038504.1	+	31055	31104
Sma-1-9585	NW_003038264.1	+	17959	18008
Sma-1-9586	NW_003037875.1	+	508931	508980
Sma-1-9587	NW_003037875.1	-	166910	166861
Sma-1-9588	NW_003037876.1	-	115514	115465
Sma-1-9589	NW_003037960.1	+	4305	4354
Sma-1-9590	NW_003038511.1	-	18786	18737
Sma-1-9591	NW_003026596.1	-	152213	152164
Sma-1-9592	NW_003035636.1	+	49069	49118
Sma-1-9593	NW_003035880.1	-	25251	25202
Sma-1-9594	NW_003037965.1	-	142368	142319
Sma-1-9595	NW_003039028.1	-	1539	1490
Sma-1-9596	NW_003035639.1	+	51948	51997
Sma-1-9597	NW_003038276.1	+	3941	3990
Sma-1-9598	NW_003026472.1	+	129280	129329
Sma-1-9599	NW_003026472.1	+	320834	320883
Sma-1-9600	NW_003026472.1	-	241686	241637
Sma-1-9601	NW_003026472.1	-	152537	152488
Sma-1-9602	NW_003037898.1	-	387972	387923
Sma-1-9603	NW_003038457.1	-	27369	27320
Sma-1-9604	NW_003026471.1	-	226861	226812
Sma-1-9605	NW_003038377.1	-	4565	4516
Sma-1-9606	NW_003037771.1	+	43067	43116
Sma-1-9607	NW_003026611.1	-	103801	103752
Sma-1-9608	NW_003037774.1	+	26093	26142
Sma-1-9609	NW_003037774.1	-	22744	22695
Sma-1-9610	NW_003037920.1	+	192115	192164
Sma-1-9611	NW_003037920.1	-	191871	191822
Sma-1-9612	NW_003037217.1	+	1628	1677
Sma-1-9613	NW_003038005.1	+	394581	394630
Sma-1-9614	NW_003038231.1	+	2844	2893
Sma-1-9615	NW_003026618.1	+	178630	178679
Sma-1-9616	NW_003026618.1	+	223777	223826
Sma-1-9617	NW_003026612.1	-	207079	207030
Sma-1-9618	NW_003038009.1	+	68213	68262
Sma-1-9619	NW_003038235.1	+	89820	89869
Sma-1-9620	NW_003037466.1	-	1430	1381
Sma-1-9621	NW_003038917.1	+	849	898
Sma-1-9622	NW_003038917.1	+	1902	1951
Sma-1-9623	NW_003037259.1	+	100	149
Sma-1-9624	NW_003038473.1	+	34328	34377

Sma-1-9625	NW_003038473.1	-	33897	33848
Sma-1-9626	NW_003038473.1	-	32055	32006
Sma-1-9627	NW_003026465.1	+	327099	327148
Sma-1-9628	NW_003026465.1	+	335196	335245
Sma-1-9629	NW_003026465.1	-	334795	334746
Sma-1-9630	NW_003037790.1	+	317139	317188
Sma-1-9631	NW_003035870.1	-	46982	46933
Sma-1-9632	NW_003038258.1	+	1441	1490
Sma-1-9633	NW_003037929.1	+	287456	287505
Sma-1-9634	NW_003037929.1	-	75153	75104
Sma-1-9635	NW_003037934.1	-	96376	96327
Sma-1-9636	NW_003037934.1	-	75851	75802
Sma-1-9637	NW_003038244.1	-	333262	333213
Sma-1-9638	NW_003038244.1	-	176888	176839
Sma-1-9639	NW_003026469.1	+	23964	24013
Sma-1-9640	NW_003026469.1	-	23555	23506
Sma-1-9641	NW_003037936.1	-	239697	239648
Sma-1-9642	NW_003037937.1	+	307888	307937
Sma-1-9643	NW_003037995.1	-	390568	390519
Sma-1-9644	NW_003037995.1	-	269957	269908
Sma-1-9645	NW_003037995.1	-	48437	48388
Sma-1-9646	NW_003038698.1	+	101	150
Sma-1-9647	NW_003035866.1	-	51328	51279
Sma-1-9648	NW_003038070.1	-	281939	281890
Sma-1-9649	NW_003038250.1	-	95201	95152
Sma-1-9650	NW_003026462.1	+	495706	495755
Sma-1-9651	NW_003026464.1	-	672181	672132
Sma-1-9652	NW_003026470.1	+	115935	115984
Sma-1-9653	NW_003026470.1	-	312493	312444
Sma-1-9654	NW_003026470.1	-	124876	124827
Sma-1-9655	NW_003026604.1	+	155724	155773
Sma-1-9656	NW_003026604.1	+	175441	175490
Sma-1-9657	NW_003026619.1	+	135618	135667
Sma-1-9658	NW_003037793.1	+	399042	399091
Sma-1-9659	NW_003037793.1	+	1699116	1699165
Sma-1-9660	NW_003037793.1	+	2627558	2627607
Sma-1-9661	NW_003037793.1	+	3869488	3869537
Sma-1-9662	NW_003037793.1	+	4163649	4163698
Sma-1-9663	NW_003037793.1	-	3339965	3339916
Sma-1-9664	NW_003037793.1	-	2825011	2824962
Sma-1-9665	NW_003037793.1	-	1568160	1568111
Sma-1-9666	NW_003037793.1	-	1078388	1078339
Sma-1-9667	NW_003037793.1	-	441706	441657
Sma-1-9668	NW_003037794.1	+	411054	411103
Sma-1-9669	NW_003037794.1	+	475128	475177
Sma-1-9670	NW_003037794.1	+	681309	681358
Sma-1-9671	NW_003037794.1	+	1025586	1025635
Sma-1-9672	NW_003037794.1	-	1077023	1076974
Sma-1-9673	NW_003037794.1	-	878975	878926
Sma-1-9674	NW_003037795.1	+	517140	517189
Sma-1-9675	NW_003037795.1	+	828559	828608
Sma-1-9676	NW_003037795.1	+	1461545	1461594

Sma-1-9677	NW_003037795.1	-	1035752	1035703
Sma-1-9678	NW_003037795.1	-	516859	516810
Sma-1-9679	NW_003037796.1	-	1557310	1557261
Sma-1-9680	NW_003037796.1	-	1237619	1237570
Sma-1-9681	NW_003037796.1	-	1237219	1237170
Sma-1-9682	NW_003037796.1	-	1178425	1178376
Sma-1-9683	NW_003037797.1	+	5016	5065
Sma-1-9684	NW_003037797.1	+	620580	620629
Sma-1-9685	NW_003037797.1	+	1287677	1287726
Sma-1-9686	NW_003037797.1	+	1897273	1897322
Sma-1-9687	NW_003037797.1	+	2410508	2410557
Sma-1-9688	NW_003037797.1	+	3469116	3469165
Sma-1-9689	NW_003037797.1	+	3723081	3723130
Sma-1-9690	NW_003037797.1	-	2502658	2502609
Sma-1-9691	NW_003037798.1	+	628770	628819
Sma-1-9692	NW_003037798.1	+	1036931	1036980
Sma-1-9693	NW_003037798.1	+	1678446	1678495
Sma-1-9694	NW_003037798.1	+	2034553	2034602
Sma-1-9695	NW_003037798.1	-	2037117	2037068
Sma-1-9696	NW_003037798.1	-	733161	733112
Sma-1-9697	NW_003037798.1	-	388763	388714
Sma-1-9698	NW_003037799.1	+	643737	643786
Sma-1-9699	NW_003037799.1	-	1413449	1413400
Sma-1-9700	NW_003037799.1	-	1141189	1141140
Sma-1-9701	NW_003037800.1	-	1934672	1934623
Sma-1-9702	NW_003037800.1	-	756714	756665
Sma-1-9703	NW_003037801.1	+	326834	326883
Sma-1-9704	NW_003037801.1	-	808101	808052
Sma-1-9705	NW_003037802.1	+	352231	352280
Sma-1-9706	NW_003037803.1	-	817744	817695
Sma-1-9707	NW_003037803.1	-	186420	186371
Sma-1-9708	NW_003037804.1	-	377345	377296
Sma-1-9709	NW_003037806.1	+	82145	82194
Sma-1-9710	NW_003037807.1	+	787761	787810
Sma-1-9711	NW_003037808.1	+	745771	745820
Sma-1-9712	NW_003037808.1	+	1867310	1867359
Sma-1-9713	NW_003037808.1	-	745563	745514
Sma-1-9714	NW_003037808.1	-	249364	249315
Sma-1-9715	NW_003037808.1	-	26211	26162
Sma-1-9716	NW_003037809.1	+	644145	644194
Sma-1-9717	NW_003037809.1	+	731505	731554
Sma-1-9718	NW_003037809.1	+	1326880	1326929
Sma-1-9719	NW_003037809.1	+	2589116	2589165
Sma-1-9720	NW_003037809.1	-	1994765	1994716
Sma-1-9721	NW_003037809.1	-	1516056	1516007
Sma-1-9722	NW_003037809.1	-	513022	512973
Sma-1-9723	NW_003037809.1	-	65763	65714
Sma-1-9724	NW_003037810.1	+	752212	752261
Sma-1-9725	NW_003037810.1	-	292920	292871
Sma-1-9726	NW_003037812.1	+	1249951	1250000
Sma-1-9727	NW_003037813.1	-	67930	67881
Sma-1-9728	NW_003037814.1	-	1320639	1320590

Sma-1-9729	NW_003037815.1	+	467542	467591
Sma-1-9730	NW_003037816.1	-	618597	618548
Sma-1-9731	NW_003037816.1	-	444106	444057
Sma-1-9732	NW_003037817.1	-	261994	261945
Sma-1-9733	NW_003037818.1	+	1472842	1472891
Sma-1-9734	NW_003037818.1	+	1665426	1665475
Sma-1-9735	NW_003037818.1	-	1230819	1230770
Sma-1-9736	NW_003037819.1	+	1136681	1136730
Sma-1-9737	NW_003037820.1	+	607998	608047
Sma-1-9738	NW_003037820.1	+	665839	665888
Sma-1-9739	NW_003037820.1	+	666837	666886
Sma-1-9740	NW_003037820.1	-	753336	753287
Sma-1-9741	NW_003037821.1	+	254608	254657
Sma-1-9742	NW_003037821.1	+	783237	783286
Sma-1-9743	NW_003037821.1	+	992518	992567
Sma-1-9744	NW_003037821.1	-	777792	777743
Sma-1-9745	NW_003037822.1	-	1451604	1451555
Sma-1-9746	NW_003037822.1	-	381903	381854
Sma-1-9747	NW_003037822.1	-	378187	378138
Sma-1-9748	NW_003037824.1	-	515590	515541
Sma-1-9749	NW_003037825.1	+	943168	943217
Sma-1-9750	NW_003037825.1	+	1644574	1644623
Sma-1-9751	NW_003037825.1	-	1830096	1830047
Sma-1-9752	NW_003037825.1	-	942887	942838
Sma-1-9753	NW_003037825.1	-	407161	407112
Sma-1-9754	NW_003037826.1	+	1123657	1123706
Sma-1-9755	NW_003037826.1	-	1126098	1126049
Sma-1-9756	NW_003037827.1	+	789267	789316
Sma-1-9757	NW_003037827.1	+	1441526	1441575
Sma-1-9758	NW_003037827.1	+	2268494	2268543
Sma-1-9759	NW_003037827.1	-	551669	551620
Sma-1-9760	NW_003037827.1	-	426390	426341
Sma-1-9761	NW_003037829.1	-	1685205	1685156
Sma-1-9762	NW_003037830.1	-	1337413	1337364
Sma-1-9763	NW_003037830.1	-	267804	267755
Sma-1-9764	NW_003037831.1	+	1352567	1352616
Sma-1-9765	NW_003037831.1	-	1712324	1712275
Sma-1-9766	NW_003037831.1	-	1159756	1159707
Sma-1-9767	NW_003037832.1	+	839485	839534
Sma-1-9768	NW_003037832.1	-	120332	120283
Sma-1-9769	NW_003037834.1	+	96882	96931
Sma-1-9770	NW_003037834.1	+	710868	710917
Sma-1-9771	NW_003037835.1	-	777125	777076
Sma-1-9772	NW_003037836.1	+	912517	912566
Sma-1-9773	NW_003037837.1	+	1130348	1130397
Sma-1-9774	NW_003037837.1	-	591867	591818
Sma-1-9775	NW_003037838.1	+	740444	740493
Sma-1-9776	NW_003037839.1	-	1632412	1632363
Sma-1-9777	NW_003037839.1	-	1173283	1173234
Sma-1-9778	NW_003037839.1	-	766498	766449
Sma-1-9779	NW_003037840.1	+	368434	368483
Sma-1-9780	NW_003037840.1	+	616975	617024

Sma-1-9781	NW_003037840.1	+	1865572	1865621
Sma-1-9782	NW_003037840.1	-	777266	777217
Sma-1-9783	NW_003037841.1	+	196355	196404
Sma-1-9784	NW_003037841.1	+	1424053	1424102
Sma-1-9785	NW_003037841.1	-	1099422	1099373
Sma-1-9786	NW_003037841.1	-	480504	480455
Sma-1-9787	NW_003037842.1	+	483577	483626
Sma-1-9788	NW_003037842.1	-	65189	65140
Sma-1-9789	NW_003037845.1	+	230906	230955
Sma-1-9790	NW_003037845.1	-	344254	344205
Sma-1-9791	NW_003037846.1	+	189010	189059
Sma-1-9792	NW_003037846.1	-	75243	75194
Sma-1-9793	NW_003037846.1	-	74842	74793
Sma-1-9794	NW_003037846.1	-	74441	74392
Sma-1-9795	NW_003037846.1	-	62587	62538
Sma-1-9796	NW_003037847.1	+	625177	625226
Sma-1-9797	NW_003037847.1	-	678837	678788
Sma-1-9798	NW_003037847.1	-	442705	442656
Sma-1-9799	NW_003037848.1	+	561611	561660
Sma-1-9800	NW_003037848.1	-	404260	404211
Sma-1-9801	NW_003037849.1	-	241798	241749
Sma-1-9802	NW_003037853.1	+	332689	332738
Sma-1-9803	NW_003037853.1	+	1043228	1043277
Sma-1-9804	NW_003037853.1	-	332467	332418
Sma-1-9805	NW_003037858.1	-	896459	896410
Sma-1-9806	NW_003037859.1	+	692580	692629
Sma-1-9807	NW_003037859.1	-	787658	787609
Sma-1-9808	NW_003037860.1	+	15002	15051
Sma-1-9809	NW_003037860.1	+	416488	416537
Sma-1-9810	NW_003037860.1	-	415496	415447
Sma-1-9811	NW_003037861.1	+	855200	855249
Sma-1-9812	NW_003037861.1	-	163832	163783
Sma-1-9813	NW_003037862.1	-	967350	967301
Sma-1-9814	NW_003037863.1	+	291919	291968
Sma-1-9815	NW_003037863.1	+	1170759	1170808
Sma-1-9816	NW_003037863.1	+	1711522	1711571
Sma-1-9817	NW_003037863.1	-	501514	501465
Sma-1-9818	NW_003037864.1	+	156862	156911
Sma-1-9819	NW_003037864.1	+	748792	748841
Sma-1-9820	NW_003037864.1	+	1736604	1736653
Sma-1-9821	NW_003037864.1	-	1736147	1736098
Sma-1-9822	NW_003037866.1	+	1252768	1252817
Sma-1-9823	NW_003037866.1	-	1372444	1372395
Sma-1-9824	NW_003037866.1	-	1250241	1250192
Sma-1-9825	NW_003037866.1	-	1220931	1220882
Sma-1-9826	NW_003037866.1	-	867250	867201
Sma-1-9827	NW_003037869.1	-	113192	113143
Sma-1-9828	NW_003037871.1	+	197826	197875
Sma-1-9829	NW_003037877.1	+	682788	682837
Sma-1-9830	NW_003037878.1	-	450594	450545
Sma-1-9831	NW_003037882.1	+	151317	151366
Sma-1-9832	NW_003037882.1	-	236123	236074

Sma-1-9833	NW_003037882.1	-	133355	133306
Sma-1-9834	NW_003037884.1	-	2858	2809
Sma-1-9835	NW_003037885.1	-	294024	293975
Sma-1-9836	NW_003037886.1	-	802026	801977
Sma-1-9837	NW_003037888.1	+	252578	252627
Sma-1-9838	NW_003037888.1	-	990349	990300
Sma-1-9839	NW_003037888.1	-	258122	258073
Sma-1-9840	NW_003037889.1	+	201179	201228
Sma-1-9841	NW_003037889.1	+	788879	788928
Sma-1-9842	NW_003037889.1	+	1695147	1695196
Sma-1-9843	NW_003037889.1	-	365586	365537
Sma-1-9844	NW_003037890.1	+	1095281	1095330
Sma-1-9845	NW_003037890.1	+	1174629	1174678
Sma-1-9846	NW_003037890.1	+	2007444	2007493
Sma-1-9847	NW_003037890.1	-	64961	64912
Sma-1-9848	NW_003037891.1	+	1269508	1269557
Sma-1-9849	NW_003037891.1	+	1975794	1975843
Sma-1-9850	NW_003037891.1	-	2012528	2012479
Sma-1-9851	NW_003037891.1	-	1269345	1269296
Sma-1-9852	NW_003037891.1	-	268091	268042
Sma-1-9853	NW_003037892.1	-	82175	82126
Sma-1-9854	NW_003037894.1	+	152464	152513
Sma-1-9855	NW_003037899.1	+	551287	551336
Sma-1-9856	NW_003037899.1	-	69461	69412
Sma-1-9857	NW_003037901.1	+	805191	805240
Sma-1-9858	NW_003037903.1	+	715716	715765
Sma-1-9859	NW_003037903.1	-	715318	715269
Sma-1-9860	NW_003037904.1	-	345961	345912
Sma-1-9861	NW_003037905.1	+	261751	261800
Sma-1-9862	NW_003037905.1	+	262151	262200
Sma-1-9863	NW_003037905.1	+	266992	267041
Sma-1-9864	NW_003037905.1	-	261471	261422
Sma-1-9865	NW_003037907.1	+	345078	345127
Sma-1-9866	NW_003037907.1	+	801440	801489
Sma-1-9867	NW_003037908.1	-	706237	706188
Sma-1-9868	NW_003037909.1	+	267529	267578
Sma-1-9869	NW_003037909.1	+	634922	634971
Sma-1-9870	NW_003037909.1	-	272672	272623
Sma-1-9871	NW_003037911.1	+	952644	952693
Sma-1-9872	NW_003037911.1	-	832225	832176
Sma-1-9873	NW_003037911.1	-	604427	604378
Sma-1-9874	NW_003037911.1	-	197313	197264
Sma-1-9875	NW_003037911.1	-	196912	196863
Sma-1-9876	NW_003037912.1	+	383982	384031
Sma-1-9877	NW_003037912.1	-	382954	382905
Sma-1-9878	NW_003037913.1	+	405406	405455
Sma-1-9879	NW_003037916.1	+	158153	158202
Sma-1-9880	NW_003037916.1	+	278190	278239
Sma-1-9881	NW_003037916.1	-	1466781	1466732
Sma-1-9882	NW_003037916.1	-	692823	692774
Sma-1-9883	NW_003037916.1	-	371013	370964
Sma-1-9884	NW_003037917.1	+	1116550	1116599

Sma-1-9885	NW_003037918.1	+	792749	792798
Sma-1-9886	NW_003037918.1	+	1656394	1656443
Sma-1-9887	NW_003037918.1	-	1963748	1963699
Sma-1-9888	NW_003037918.1	-	1943973	1943924
Sma-1-9889	NW_003037918.1	-	1846404	1846355
Sma-1-9890	NW_003037918.1	-	1015396	1015347
Sma-1-9891	NW_003037918.1	-	82667	82618
Sma-1-9892	NW_003037921.1	-	103909	103860
Sma-1-9893	NW_003037921.1	-	49052	49003
Sma-1-9894	NW_003037922.1	+	216252	216301
Sma-1-9895	NW_003037924.1	+	338821	338870
Sma-1-9896	NW_003037925.1	+	238504	238553
Sma-1-9897	NW_003037928.1	+	170389	170438
Sma-1-9898	NW_003037928.1	+	764753	764802
Sma-1-9899	NW_003037928.1	-	45280	45231
Sma-1-9900	NW_003037930.1	-	207227	207178
Sma-1-9901	NW_003037931.1	-	597468	597419
Sma-1-9902	NW_003037932.1	+	616036	616085
Sma-1-9903	NW_003037932.1	-	819114	819065
Sma-1-9904	NW_003037933.1	+	304755	304804
Sma-1-9905	NW_003037933.1	+	348980	349029
Sma-1-9906	NW_003037935.1	-	600365	600316
Sma-1-9907	NW_003037935.1	-	287722	287673
Sma-1-9908	NW_003037938.1	+	188678	188727
Sma-1-9909	NW_003037938.1	+	349439	349488
Sma-1-9910	NW_003037938.1	+	754107	754156
Sma-1-9911	NW_003037938.1	-	554259	554210
Sma-1-9912	NW_003037938.1	-	186758	186709
Sma-1-9913	NW_003037939.1	+	596516	596565
Sma-1-9914	NW_003037939.1	-	228432	228383
Sma-1-9915	NW_003037940.1	-	468249	468200
Sma-1-9916	NW_003037940.1	-	383128	383079
Sma-1-9917	NW_003037943.1	+	1088970	1089019
Sma-1-9918	NW_003037943.1	-	1077189	1077140
Sma-1-9919	NW_003037943.1	-	473302	473253
Sma-1-9920	NW_003037943.1	-	285294	285245
Sma-1-9921	NW_003037944.1	-	527361	527312
Sma-1-9922	NW_003037945.1	-	445079	445030
Sma-1-9923	NW_003037945.1	-	374296	374247
Sma-1-9924	NW_003037946.1	+	280009	280058
Sma-1-9925	NW_003037946.1	+	880606	880655
Sma-1-9926	NW_003037948.1	-	173706	173657
Sma-1-9927	NW_003037953.1	+	168409	168458
Sma-1-9928	NW_003037954.1	-	275279	275230
Sma-1-9929	NW_003037961.1	+	341569	341618
Sma-1-9930	NW_003037963.1	-	230327	230278
Sma-1-9931	NW_003037963.1	-	184425	184376
Sma-1-9932	NW_003037964.1	-	383937	383888
Sma-1-9933	NW_003037966.1	+	341915	341964
Sma-1-9934	NW_003037966.1	+	384123	384172
Sma-1-9935	NW_003037966.1	-	84994	84945
Sma-1-9936	NW_003037967.1	+	89512	89561

Sma-1-9937	NW_003037970.1	+	188901	188950
Sma-1-9938	NW_003037972.1	+	305924	305973
Sma-1-9939	NW_003037972.1	+	460770	460819
Sma-1-9940	NW_003037972.1	-	579869	579820
Sma-1-9941	NW_003037972.1	-	459872	459823
Sma-1-9942	NW_003037972.1	-	305567	305518
Sma-1-9943	NW_003037972.1	-	302841	302792
Sma-1-9944	NW_003037973.1	-	433783	433734
Sma-1-9945	NW_003037973.1	-	197247	197198
Sma-1-9946	NW_003037974.1	-	510040	509991
Sma-1-9947	NW_003037975.1	-	580870	580821
Sma-1-9948	NW_003037977.1	-	939432	939383
Sma-1-9949	NW_003037977.1	-	338582	338533
Sma-1-9950	NW_003037979.1	+	257948	257997
Sma-1-9951	NW_003037979.1	-	257650	257601
Sma-1-9952	NW_003037980.1	+	1260880	1260929
Sma-1-9953	NW_003037980.1	-	1166155	1166106
Sma-1-9954	NW_003037981.1	+	529302	529351
Sma-1-9955	NW_003037981.1	+	838489	838538
Sma-1-9956	NW_003037981.1	+	857249	857298
Sma-1-9957	NW_003037981.1	-	1178811	1178762
Sma-1-9958	NW_003037981.1	-	625324	625275
Sma-1-9959	NW_003037981.1	-	511492	511443
Sma-1-9960	NW_003037981.1	-	497436	497387
Sma-1-9961	NW_003037981.1	-	444655	444606
Sma-1-9962	NW_003037981.1	-	86773	86724
Sma-1-9963	NW_003037993.1	+	79281	79330
Sma-1-9964	NW_003037994.1	+	263338	263387
Sma-1-9965	NW_003037994.1	-	284792	284743
Sma-1-9966	NW_003037997.1	+	451904	451953
Sma-1-9967	NW_003037997.1	-	433819	433770
Sma-1-9968	NW_003037997.1	-	143354	143305
Sma-1-9969	NW_003037999.1	-	352422	352373
Sma-1-9970	NW_003038001.1	+	7315	7364
Sma-1-9971	NW_003038001.1	+	30176	30225
Sma-1-9972	NW_003038001.1	+	104803	104852
Sma-1-9973	NW_003038008.1	+	131432	131481
Sma-1-9974	NW_003038008.1	+	348150	348199
Sma-1-9975	NW_003038010.1	+	441215	441264
Sma-1-9976	NW_003038014.1	+	486861	486910
Sma-1-9977	NW_003038015.1	-	414897	414848
Sma-1-9978	NW_003038018.1	+	17841	17890
Sma-1-9979	NW_003038018.1	+	368530	368579
Sma-1-9980	NW_003038018.1	-	848763	848714
Sma-1-9981	NW_003038018.1	-	549077	549028
Sma-1-9982	NW_003038019.1	-	374888	374839
Sma-1-9983	NW_003038020.1	+	524689	524738
Sma-1-9984	NW_003038020.1	-	524534	524485
Sma-1-9985	NW_003038023.1	+	617829	617878
Sma-1-9986	NW_003038027.1	+	662461	662510
Sma-1-9987	NW_003038027.1	-	273576	273527
Sma-1-9988	NW_003038028.1	+	653297	653346

Sma-1-9989	NW_003038028.1	+	755689	755738
Sma-1-9990	NW_003038031.1	+	358992	359041
Sma-1-9991	NW_003038031.1	-	863672	863623
Sma-1-9992	NW_003038032.1	-	524322	524273
Sma-1-9993	NW_003038033.1	-	199150	199101
Sma-1-9994	NW_003038033.1	-	50039	49990
Sma-1-9995	NW_003038035.1	-	1052514	1052465
Sma-1-9996	NW_003038035.1	-	1033237	1033188
Sma-1-9997	NW_003038036.1	+	4621	4670
Sma-1-9998	NW_003038036.1	+	271136	271185
Sma-1-9999	NW_003038037.1	-	1629119	1629070
Sma-1-10000	NW_003038037.1	-	842282	842233
Sma-1-10001	NW_003038037.1	-	695977	695928
Sma-1-10002	NW_003038038.1	-	523127	523078
Sma-1-10003	NW_003038072.1	-	200145	200096
Sma-1-10004	NW_003038230.1	+	1406	1455
Sma-1-10005	NW_003038230.1	-	282309	282260
Sma-1-10006	NW_003038230.1	-	278651	278602
Sma-1-10007	NW_003038233.1	-	21201	21152
Sma-1-10008	NW_003038237.1	-	114463	114414
Sma-1-10009	NW_003038238.1	+	111821	111870
Sma-1-10010	NW_003038239.1	-	216088	216039
Sma-1-10011	NW_003038239.1	-	34600	34551
Sma-1-10012	NW_003038240.1	-	86360	86311
Sma-1-10013	NW_003038247.1	+	361430	361479
Sma-1-10014	NW_003038247.1	-	339782	339733
Sma-1-10015	NW_003038251.1	-	236126	236077
Sma-1-10016	NW_003038251.1	-	235723	235674
Sma-1-10017	NW_003038254.1	-	4901	4852
Sma-1-10018	NW_003038257.1	+	318105	318154
Sma-1-10019	NW_003038257.1	-	559126	559077
Sma-1-10020	NW_003038260.1	-	321345	321296
Sma-1-10021	NW_003038261.1	+	16709	16758
Sma-1-10022	NW_003038261.1	+	1014479	1014528
Sma-1-10023	NW_003038261.1	-	481087	481038
Sma-1-10024	NW_003038513.1	+	143729	143778
Sma-1-10025	NW_003039127.1	+	2844	2893
Sma-1-10026	NW_003039127.1	-	901316	901267
Sma-1-10027	NW_003039127.1	-	515624	515575
Sma-1-10028	NW_003039127.1	-	515223	515174
Sma-1-10029	NW_003039127.1	-	262952	262903
Sma-1-10030	NW_003026474.1	+	85592	85641
Sma-1-10031	NW_003026474.1	-	124381	124332
Sma-1-10032	NW_003026483.1	-	136975	136926
Sma-1-10033	NW_003026490.1	-	425088	425039
Sma-1-10034	NW_003026503.1	+	55120	55169
Sma-1-10035	NW_003026515.1	+	75625	75674
Sma-1-10036	NW_003026516.1	+	278901	278950
Sma-1-10037	NW_003026517.1	-	724123	724074
Sma-1-10038	NW_003038041.1	+	84446	84495
Sma-1-10039	NW_003038042.1	-	79283	79234
Sma-1-10040	NW_003038044.1	+	136166	136215

Sma-1-10041	NW_003038046.1	+	164571	164620
Sma-1-10042	NW_003038050.1	-	236913	236864
Sma-1-10043	NW_003038050.1	-	65892	65843
Sma-1-10044	NW_003038051.1	-	40808	40759
Sma-1-10045	NW_003038053.1	-	344773	344724
Sma-1-10046	NW_003038055.1	+	38880	38929
Sma-1-10047	NW_003038057.1	-	185908	185859
Sma-1-10048	NW_003038059.1	-	303751	303702
Sma-1-10049	NW_003038077.1	+	417421	417470
Sma-1-10050	NW_003038077.1	-	268463	268414
Sma-1-10051	NW_003038080.1	+	550111	550160
Sma-1-10052	NW_003038081.1	+	71815	71864
Sma-1-10053	NW_003038082.1	+	239234	239283
Sma-1-10054	NW_003038082.1	+	681281	681330
Sma-1-10055	NW_003038082.1	-	680679	680630
Sma-1-10056	NW_003038082.1	-	6613	6564
Sma-1-10057	NW_003038084.1	+	151300	151349
Sma-1-10058	NW_003038084.1	-	151012	150963
Sma-1-10059	NW_003038086.1	+	366504	366553
Sma-1-10060	NW_003038086.1	-	164115	164066
Sma-1-10061	NW_003038105.1	+	51458	51507
Sma-1-10062	NW_003038105.1	-	51273	51224
Sma-1-10063	NW_003038108.1	+	39953	40002
Sma-1-10064	NW_003038108.1	+	107062	107111
Sma-1-10065	NW_003038110.1	+	118615	118664
Sma-1-10066	NW_003038110.1	-	118367	118318
Sma-1-10067	NW_003038114.1	-	139203	139154
Sma-1-10068	NW_003038119.1	-	221076	221027
Sma-1-10069	NW_003038119.1	-	175284	175235
Sma-1-10070	NW_003038126.1	+	135581	135630
Sma-1-10071	NW_003038126.1	+	135981	136030
Sma-1-10072	NW_003038126.1	+	136381	136430
Sma-1-10073	NW_003038126.1	+	159189	159238
Sma-1-10074	NW_003038126.1	-	135183	135134
Sma-1-10075	NW_003038126.1	-	134783	134734
Sma-1-10076	NW_003038127.1	+	96458	96507
Sma-1-10077	NW_003038129.1	+	87	136
Sma-1-10078	NW_003038129.1	+	36782	36831
Sma-1-10079	NW_003038130.1	-	315256	315207
Sma-1-10080	NW_003038131.1	+	53328	53377
Sma-1-10081	NW_003038131.1	-	374171	374122
Sma-1-10082	NW_003038131.1	-	344731	344682
Sma-1-10083	NW_003038132.1	+	184386	184435
Sma-1-10084	NW_003038132.1	-	259661	259612
Sma-1-10085	NW_003038136.1	+	19933	19982
Sma-1-10086	NW_003038136.1	+	69343	69392
Sma-1-10087	NW_003038138.1	-	461714	461665
Sma-1-10088	NW_003038147.1	+	359712	359761
Sma-1-10089	NW_003038151.1	-	169941	169892
Sma-1-10090	NW_003038155.1	+	334398	334447
Sma-1-10091	NW_003038155.1	+	501714	501763
Sma-1-10092	NW_003038157.1	+	250092	250141

Sma-1-10093	NW_003038163.1	+	61002	61051
Sma-1-10094	NW_003038163.1	+	61401	61450
Sma-1-10095	NW_003038163.1	-	467069	467020
Sma-1-10096	NW_003038169.1	+	39677	39726
Sma-1-10097	NW_003038169.1	-	52651	52602
Sma-1-10098	NW_003038183.1	+	68654	68703
Sma-1-10099	NW_003038196.1	+	91692	91741
Sma-1-10100	NS_000200.1	+	399042	399091
Sma-1-10101	NS_000200.1	+	1699116	1699165
Sma-1-10102	NS_000200.1	+	2627558	2627607
Sma-1-10103	NS_000200.1	+	3869488	3869537
Sma-1-10104	NS_000200.1	+	4163649	4163698
Sma-1-10105	NS_000200.1	+	4590649	4590698
Sma-1-10106	NS_000200.1	+	4654723	4654772
Sma-1-10107	NS_000200.1	+	4860904	4860953
Sma-1-10108	NS_000200.1	+	5205181	5205230
Sma-1-10109	NS_000200.1	+	6908023	6908072
Sma-1-10110	NS_000200.1	+	7219442	7219491
Sma-1-10111	NS_000200.1	+	7852428	7852477
Sma-1-10112	NS_000200.1	+	10432876	10432925
Sma-1-10113	NS_000200.1	+	11048440	11048489
Sma-1-10114	NS_000200.1	+	11715537	11715586
Sma-1-10115	NS_000200.1	+	12325133	12325182
Sma-1-10116	NS_000200.1	+	12838368	12838417
Sma-1-10117	NS_000200.1	+	13896976	13897025
Sma-1-10118	NS_000200.1	+	14150941	14150990
Sma-1-10119	NS_000200.1	+	14887928	14887977
Sma-1-10120	NS_000200.1	+	15296089	15296138
Sma-1-10121	NS_000200.1	+	15937604	15937653
Sma-1-10122	NS_000200.1	+	16293711	16293760
Sma-1-10123	NS_000200.1	+	17634091	17634140
Sma-1-10124	NS_000200.1	+	21416314	21416363
Sma-1-10125	NS_000200.1	+	22355025	22355074
Sma-1-10126	NS_000200.1	+	28277359	28277408
Sma-1-10127	NS_000200.1	+	31049277	31049326
Sma-1-10128	NS_000200.1	+	32532408	32532457
Sma-1-10129	NS_000200.1	+	33653947	33653996
Sma-1-10130	NS_000200.1	+	34308609	34308658
Sma-1-10131	NS_000200.1	+	34395969	34396018
Sma-1-10132	NS_000200.1	+	34991344	34991393
Sma-1-10133	NS_000200.1	+	36253580	36253629
Sma-1-10134	NS_000200.1	+	37356734	37356783
Sma-1-10135	NS_000200.1	+	40732690	40732739
Sma-1-10136	NS_000200.1	+	45730533	45730582
Sma-1-10137	NS_000200.1	+	50188249	50188298
Sma-1-10138	NS_000200.1	+	50380833	50380882
Sma-1-10139	NS_000200.1	+	52224946	52224995
Sma-1-10140	NS_000200.1	+	52880980	52881029
Sma-1-10141	NS_000200.1	+	52938821	52938870
Sma-1-10142	NS_000200.1	+	52939819	52939868
Sma-1-10143	NS_000200.1	+	53399092	53399141
Sma-1-10144	NS_000200.1	+	53927721	53927770

Sma-1-10145	NS_000200.1	+	54137002	54137051
Sma-1-10146	NS_000200.1	+	60072950	60072999
Sma-1-10147	NS_000200.1	+	60774356	60774405
Sma-1-10148	NS_000200.1	+	62123173	62123222
Sma-1-10149	NS_000200.1	+	63411408	63411457
Sma-1-10150	NS_000200.1	+	64063667	64063716
Sma-1-10151	NS_000200.1	+	64890635	64890684
Sma-1-10152	NS_000200.1	+	70801869	70801918
Sma-1-10153	NS_000200.1	+	72048916	72048965
Sma-1-10154	NS_000200.1	+	73257368	73257417
Sma-1-10155	NS_000200.1	+	73871354	73871403
Sma-1-10156	NS_000200.1	+	76469616	76469665
Sma-1-10157	NS_000200.1	+	77936206	77936255
Sma-1-10158	NS_000200.1	+	78739007	78739056
Sma-1-10159	NS_000200.1	+	80991758	80991807
Sma-1-10160	NS_000200.1	+	81240299	81240348
Sma-1-10161	NS_000200.1	+	82488896	82488945
Sma-1-10162	NS_000200.1	+	82807536	82807585
Sma-1-10163	NS_000200.1	+	84035234	84035283
Sma-1-10164	NS_000200.1	+	85582767	85582816
Sma-1-10165	NS_000200.1	+	87232809	87232858
Sma-1-10166	NS_000200.1	+	87823580	87823629
Sma-1-10167	NS_000200.1	+	89276907	89276956
Sma-1-10168	NS_000200.1	+	90254682	90254731
Sma-1-10169	NS_000200.1	+	95824740	95824789
Sma-1-10170	NS_000200.1	+	96535279	96535328
Sma-1-10171	NS_000200.1	+	103371421	103371470
Sma-1-10172	NS_000200.1	+	103758030	103758079
Sma-1-10173	NS_000200.1	+	104159516	104159565
Sma-1-10174	NS_000200.1	+	105631654	105631703
Sma-1-10175	NS_000200.1	+	107050286	107050335
Sma-1-10176	NS_000200.1	+	107929126	107929175
Sma-1-10177	NS_000200.1	+	108469889	108469938
Sma-1-10178	NS_000200.1	+	108649436	108649485
Sma-1-10179	NS_000200.1	+	109241366	109241415
Sma-1-10180	NS_000200.1	+	110229178	110229227
Sma-1-10181	NS_000200.1	+	112740567	112740616
Sma-1-10182	NS_000200.1	+	113080343	113080392
Sma-1-10183	NS_000200.1	+	117645564	117645613
Sma-1-10184	NS_000200.1	+	120537600	120537649
Sma-1-10185	NS_000200.1	+	121721350	121721399
Sma-1-10186	NS_000200.1	+	125442991	125443040
Sma-1-10187	NS_000200.1	+	132461070	132461119
Sma-1-10188	NS_000200.1	+	133977669	133977718
Sma-1-10189	NS_000200.1	+	134565369	134565418
Sma-1-10190	NS_000200.1	+	135471637	135471686
Sma-1-10191	NS_000200.1	+	136778642	136778691
Sma-1-10192	NS_000200.1	+	136857990	136858039
Sma-1-10193	NS_000200.1	+	137690805	137690854
Sma-1-10194	NS_000200.1	+	139150786	139150835
Sma-1-10195	NS_000200.1	+	139857072	139857121
Sma-1-10196	NS_000200.1	+	142049037	142049086

Sma-1-10197	NS_000200.1	+	145070398	145070447
Sma-1-10198	NS_000200.1	+	146653652	146653701
Sma-1-10199	NS_000200.1	+	148269797	148269846
Sma-1-10200	NS_000200.1	+	149530763	149530812
Sma-1-10201	NS_000200.1	+	149531163	149531212
Sma-1-10202	NS_000200.1	+	149536004	149536053
Sma-1-10203	NS_000200.1	+	151749567	151749616
Sma-1-10204	NS_000200.1	+	152205929	152205978
Sma-1-10205	NS_000200.1	+	153809178	153809227
Sma-1-10206	NS_000200.1	+	154176571	154176620
Sma-1-10207	NS_000200.1	+	156739997	156740046
Sma-1-10208	NS_000200.1	+	157195557	157195606
Sma-1-10209	NS_000200.1	+	158969157	158969206
Sma-1-10210	NS_000200.1	+	163351564	163351613
Sma-1-10211	NS_000200.1	+	163471601	163471650
Sma-1-10212	NS_000200.1	+	166153354	166153403
Sma-1-10213	NS_000200.1	+	167359739	167359788
Sma-1-10214	NS_000200.1	+	168223384	168223433
Sma-1-10215	NS_000200.1	+	169372922	169372971
Sma-1-10216	NS_000200.1	+	170140249	170140298
Sma-1-10217	NS_000200.1	+	172045779	172045828
Sma-1-10218	NS_000200.1	+	172640507	172640556
Sma-1-10219	NS_000200.1	+	174494342	174494391
Sma-1-10220	NS_000200.1	+	175088706	175088755
Sma-1-10221	NS_000200.1	+	175528234	175528283
Sma-1-10222	NS_000200.1	+	178149232	178149281
Sma-1-10223	NS_000200.1	+	178717927	178717976
Sma-1-10224	NS_000200.1	+	178762152	178762201
Sma-1-10225	NS_000200.1	+	181507797	181507846
Sma-1-10226	NS_000200.1	+	182225791	182225840
Sma-1-10227	NS_000200.1	+	182386552	182386601
Sma-1-10228	NS_000200.1	+	182791220	182791269
Sma-1-10229	NS_000200.1	+	183466270	183466319
Sma-1-10230	NS_000200.1	+	188110503	188110552
Sma-1-10231	NS_000200.1	+	190973869	190973918
Sma-1-10232	NS_000200.1	+	191574466	191574515
Sma-1-10233	NS_000200.1	+	193081460	193081509
Sma-1-10234	NS_000200.1	+	195446455	195446504
Sma-1-10235	NS_000200.1	+	195965811	195965860
Sma-1-10236	NS_000200.1	+	197163209	197163258
Sma-1-10237	NS_000200.1	+	199043778	199043827
Sma-1-10238	NS_000200.1	+	199085986	199086035
Sma-1-10239	NS_000200.1	+	199329483	199329532
Sma-1-10240	NS_000200.1	+	201245091	201245140
Sma-1-10241	NS_000200.1	+	202781628	202781677
Sma-1-10242	NS_000200.1	+	202936474	202936523
Sma-1-10243	NS_000200.1	+	207719693	207719742
Sma-1-10244	NS_000200.1	+	207727790	207727839
Sma-1-10245	NS_000200.1	+	210318968	210319017
Sma-1-10246	NS_000200.1	+	212221742	212221791
Sma-1-10247	NS_000200.1	+	212927170	212927219
Sma-1-10248	NS_000200.1	+	213236357	213236406

Sma-1-10249	NS_000200.1	+	213255117	213255166
Sma-1-10250	NS_000200.1	+	217645623	217645672
Sma-1-10251	NS_000200.1	+	218265984	218266033
Sma-1-10252	NS_000200.1	+	218795152	218795201
Sma-1-10253	NS_000200.1	+	219239904	219239953
Sma-1-10254	NS_000200.1	+	221263346	221263395
Sma-1-10255	NS_000200.1	+	222716876	222716925
Sma-1-10256	NS_000200.1	+	222739737	222739786
Sma-1-10257	NS_000200.1	+	222814364	222814413
Sma-1-10258	NS_000200.1	+	224800535	224800584
Sma-1-10259	NS_000200.1	+	225804528	225804577
Sma-1-10260	NS_000200.1	+	226021246	226021295
Sma-1-10261	NS_000200.1	+	226442276	226442325
Sma-1-10262	NS_000200.1	+	227259438	227259487
Sma-1-10263	NS_000200.1	+	230227550	230227599
Sma-1-10264	NS_000200.1	+	232418691	232418740
Sma-1-10265	NS_000200.1	+	232769380	232769429
Sma-1-10266	NS_000200.1	+	234492033	234492082
Sma-1-10267	NS_000200.1	+	236541272	236541321
Sma-1-10268	NS_000200.1	+	236732826	236732875
Sma-1-10269	NS_000200.1	+	237778120	237778169
Sma-1-10270	NS_000200.1	+	241033175	241033224
Sma-1-10271	NS_000200.1	+	241960871	241960920
Sma-1-10272	NS_000200.1	+	242063263	242063312
Sma-1-10273	NS_000200.1	+	244636750	244636799
Sma-1-10274	NS_000200.1	+	248938473	248938522
Sma-1-10275	NS_000200.1	+	249204988	249205037
Sma-1-10276	NS_000200.1	+	253284167	253284216
Sma-1-10277	NS_000200.1	+	253751728	253751777
Sma-1-10278	NS_000200.1	+	254760948	254760997
Sma-1-10279	NS_000200.1	+	255237953	255238002
Sma-1-10280	NS_000200.1	+	258738202	258738251
Sma-1-10281	NS_000200.1	+	267189061	267189110
Sma-1-10282	NS_000200.1	+	269002721	269002770
Sma-1-10283	NS_000200.1	+	269233858	269233907
Sma-1-10284	NS_000200.1	+	269774457	269774506
Sma-1-10285	NS_000200.1	+	270216504	270216553
Sma-1-10286	NS_000200.1	+	271166544	271166593
Sma-1-10287	NS_000200.1	+	272615097	272615146
Sma-1-10288	NS_000200.1	+	275141607	275141656
Sma-1-10289	NS_000200.1	+	275474481	275474530
Sma-1-10290	NS_000200.1	+	275541590	275541639
Sma-1-10291	NS_000200.1	+	276284818	276284867
Sma-1-10292	NS_000200.1	+	276618295	276618344
Sma-1-10293	NS_000200.1	+	280145993	280146042
Sma-1-10294	NS_000200.1	+	280146393	280146442
Sma-1-10295	NS_000200.1	+	280146793	280146842
Sma-1-10296	NS_000200.1	+	280169601	280169650
Sma-1-10297	NS_000200.1	+	280488164	280488213
Sma-1-10298	NS_000200.1	+	281253434	281253483
Sma-1-10299	NS_000200.1	+	281290129	281290178
Sma-1-10300	NS_000200.1	+	282015172	282015221

Sma-1-10301	NS_000200.1	+	282604856	282604905
Sma-1-10302	NS_000200.1	+	284142002	284142051
Sma-1-10303	NS_000200.1	+	284191412	284191461
Sma-1-10304	NS_000200.1	+	289887455	289887504
Sma-1-10305	NS_000200.1	+	290720036	290720085
Sma-1-10306	NS_000200.1	+	294457516	294457565
Sma-1-10307	NS_000200.1	+	294624832	294624881
Sma-1-10308	NS_000200.1	+	295501012	295501061
Sma-1-10309	NS_000200.1	+	298461282	298461331
Sma-1-10310	NS_000200.1	+	298461681	298461730
Sma-1-10311	NS_000200.1	+	299387198	299387247
Sma-1-10312	NS_000200.1	+	300522845	300522894
Sma-1-10313	NS_000200.1	+	301289215	301289264
Sma-1-10314	NS_000200.1	+	301587335	301587384
Sma-1-10315	NS_000200.1	+	303233975	303234024
Sma-1-10316	NS_000200.1	+	307401281	307401330
Sma-1-10317	NS_000200.1	+	307420998	307421047
Sma-1-10318	NS_000200.1	+	311286977	311287026
Sma-1-10319	NS_000200.1	+	311610997	311611046
Sma-1-10320	NS_000200.1	+	313155411	313155460
Sma-1-10321	NS_000200.1	+	313718602	313718651
Sma-1-10322	NS_000200.1	+	316310158	316310207
Sma-1-10323	NS_000200.1	+	319380389	319380438
Sma-1-10324	NS_000200.1	+	319619536	319619585
Sma-1-10325	NS_000200.1	+	321492433	321492482
Sma-1-10326	NS_000200.1	+	321933632	321933681
Sma-1-10327	NS_000200.1	+	321978779	321978828
Sma-1-10328	NS_000200.1	+	322326362	322326411
Sma-1-10329	NS_000200.1	+	322539951	322540000
Sma-1-10330	NS_000200.1	+	323630360	323630409
Sma-1-10331	NS_000200.1	+	324628130	324628179
Sma-1-10332	NS_000200.1	+	324762770	324762819
Sma-1-10333	NS_000200.1	+	325164296	325164345
Sma-1-10334	NS_000200.1	+	325466291	325466340
Sma-1-10335	NS_000200.1	+	325543095	325543144
Sma-1-10336	NS_000200.1	+	326130321	326130370
Sma-1-10337	NS_000200.1	+	329521131	329521180
Sma-1-10338	NS_000200.1	+	330002388	330002437
Sma-1-10339	NS_000200.1	+	330243026	330243075
Sma-1-10340	NS_000200.1	+	332204613	332204662
Sma-1-10341	NS_000200.1	+	335730191	335730240
Sma-1-10342	NS_000200.1	+	335879587	335879636
Sma-1-10343	NS_000200.1	+	339152906	339152955
Sma-1-10344	NS_000200.1	+	344794366	344794415
Sma-1-10345	NS_000200.1	+	347416423	347416472
Sma-1-10346	NS_000200.1	+	348038190	348038239
Sma-1-10347	NS_000200.1	+	348979309	348979358
Sma-1-10348	NS_000200.1	+	355480024	355480073
Sma-1-10349	NS_000200.1	+	355481077	355481126
Sma-1-10350	NS_000200.1	+	355501481	355501530
Sma-1-10351	NS_000200.1	+	357196183	357196232
Sma-1-10352	NS_000200.1	+	357732341	357732390

Sma-1-10353	NS_000200.1	+	359474722	359474771
Sma-1-10354	NS_000200.1	+	363553852	363553901
Sma-1-10355	NS_000200.1	+	363883674	363883723
Sma-1-10356	NS_000200.1	+	364455488	364455537
Sma-1-10357	NS_000200.1	+	365610492	365610541
Sma-1-10358	NS_000200.1	+	367406513	367406562
Sma-1-10359	NS_000200.1	+	367429947	367429996
Sma-1-10360	NS_000200.1	+	370777071	370777120
Sma-1-10361	NS_000200.1	+	371150853	371150902
Sma-1-10362	NS_000200.1	+	371273888	371273937
Sma-1-10363	NS_000200.1	+	371755824	371755873
Sma-1-10364	NS_000200.1	+	376154858	376154907
Sma-1-10365	NS_000200.1	+	378343973	378344022
Sma-1-10366	NS_000200.1	+	378611470	378611519
Sma-1-10367	NS_000200.1	+	381661311	381661360
Sma-1-10368	NS_000200.1	-	380761919	380761870
Sma-1-10369	NS_000200.1	-	378608121	378608072
Sma-1-10370	NS_000200.1	-	373488557	373488508
Sma-1-10371	NS_000200.1	-	370823518	370823469
Sma-1-10372	NS_000200.1	-	370823112	370823063
Sma-1-10373	NS_000200.1	-	370807200	370807151
Sma-1-10374	NS_000200.1	-	370687465	370687416
Sma-1-10375	NS_000200.1	-	369720680	369720631
Sma-1-10376	NS_000200.1	-	365180225	365180176
Sma-1-10377	NS_000200.1	-	365102050	365102001
Sma-1-10378	NS_000200.1	-	364913478	364913429
Sma-1-10379	NS_000200.1	-	363921150	363921101
Sma-1-10380	NS_000200.1	-	363660850	363660801
Sma-1-10381	NS_000200.1	-	360445888	360445839
Sma-1-10382	NS_000200.1	-	358796742	358796693
Sma-1-10383	NS_000200.1	-	357609130	357609081
Sma-1-10384	NS_000200.1	-	356781117	356781068
Sma-1-10385	NS_000200.1	-	356319697	356319648
Sma-1-10386	NS_000200.1	-	356127395	356127346
Sma-1-10387	NS_000200.1	-	356126993	356126944
Sma-1-10388	NS_000200.1	-	352328801	352328752
Sma-1-10389	NS_000200.1	-	352328403	352328354
Sma-1-10390	NS_000200.1	-	352328005	352327956
Sma-1-10391	NS_000200.1	-	351954041	351953992
Sma-1-10392	NS_000200.1	-	351953643	351953594
Sma-1-10393	NS_000200.1	-	347650336	347650287
Sma-1-10394	NS_000200.1	-	347222552	347222503
Sma-1-10395	NS_000200.1	-	346092357	346092308
Sma-1-10396	NS_000200.1	-	346091956	346091907
Sma-1-10397	NS_000200.1	-	345067896	345067847
Sma-1-10398	NS_000200.1	-	344691450	344691401
Sma-1-10399	NS_000200.1	-	339266315	339266266
Sma-1-10400	NS_000200.1	-	338363998	338363949
Sma-1-10401	NS_000200.1	-	335412271	335412222
Sma-1-10402	NS_000200.1	-	334145138	334145089
Sma-1-10403	NS_000200.1	-	332204182	332204133
Sma-1-10404	NS_000200.1	-	332202340	332202291

Sma-1-10405	NS_000200.1	-	331109233	331109184
Sma-1-10406	NS_000200.1	-	329429104	329429055
Sma-1-10407	NS_000200.1	-	327918450	327918401
Sma-1-10408	NS_000200.1	-	327831974	327831925
Sma-1-10409	NS_000200.1	-	327473086	327473037
Sma-1-10410	NS_000200.1	-	326259946	326259897
Sma-1-10411	NS_000200.1	-	324720606	324720557
Sma-1-10412	NS_000200.1	-	324094738	324094689
Sma-1-10413	NS_000200.1	-	323493120	323493071
Sma-1-10414	NS_000200.1	-	321733454	321733405
Sma-1-10415	NS_000200.1	-	320040459	320040410
Sma-1-10416	NS_000200.1	-	318567898	318567849
Sma-1-10417	NS_000200.1	-	318567495	318567446
Sma-1-10418	NS_000200.1	-	318147878	318147829
Sma-1-10419	NS_000200.1	-	316288510	316288461
Sma-1-10420	NS_000200.1	-	315478467	315478418
Sma-1-10421	NS_000200.1	-	315322093	315322044
Sma-1-10422	NS_000200.1	-	314225291	314225242
Sma-1-10423	NS_000200.1	-	314056870	314056821
Sma-1-10424	NS_000200.1	-	313875382	313875333
Sma-1-10425	NS_000200.1	-	313554382	313554333
Sma-1-10426	NS_000200.1	-	312604300	312604251
Sma-1-10427	NS_000200.1	-	312512466	312512417
Sma-1-10428	NS_000200.1	-	311567880	311567831
Sma-1-10429	NS_000200.1	-	311564222	311564173
Sma-1-10430	NS_000200.1	-	310673585	310673536
Sma-1-10431	NS_000200.1	-	305422841	305422792
Sma-1-10432	NS_000200.1	-	300535819	300535770
Sma-1-10433	NS_000200.1	-	300330132	300330083
Sma-1-10434	NS_000200.1	-	298867349	298867300
Sma-1-10435	NS_000200.1	-	292077184	292077135
Sma-1-10436	NS_000200.1	-	286017029	286016980
Sma-1-10437	NS_000200.1	-	282680131	282680082
Sma-1-10438	NS_000200.1	-	282336015	282335966
Sma-1-10439	NS_000200.1	-	282306575	282306526
Sma-1-10440	NS_000200.1	-	281870242	281870193
Sma-1-10441	NS_000200.1	-	280145595	280145546
Sma-1-10442	NS_000200.1	-	280145195	280145146
Sma-1-10443	NS_000200.1	-	278128632	278128583
Sma-1-10444	NS_000200.1	-	278082840	278082791
Sma-1-10445	NS_000200.1	-	276865108	276865059
Sma-1-10446	NS_000200.1	-	276284570	276284521
Sma-1-10447	NS_000200.1	-	275141422	275141373
Sma-1-10448	NS_000200.1	-	272412708	272412659
Sma-1-10449	NS_000200.1	-	271166256	271166207
Sma-1-10450	NS_000200.1	-	270215902	270215853
Sma-1-10451	NS_000200.1	-	269541836	269541787
Sma-1-10452	NS_000200.1	-	268056804	268056755
Sma-1-10453	NS_000200.1	-	267040103	267040054
Sma-1-10454	NS_000200.1	-	265362373	265362324
Sma-1-10455	NS_000200.1	-	264619180	264619131
Sma-1-10456	NS_000200.1	-	260545823	260545774

Sma-1-10457	NS_000200.1	-	259391724	259391675
Sma-1-10458	NS_000200.1	-	257704868	257704819
Sma-1-10459	NS_000200.1	-	256783179	256783130
Sma-1-10460	NS_000200.1	-	256651499	256651450
Sma-1-10461	NS_000200.1	-	256511807	256511758
Sma-1-10462	NS_000200.1	-	256340786	256340737
Sma-1-10463	NS_000200.1	-	254024882	254024833
Sma-1-10464	NS_000200.1	-	253322956	253322907
Sma-1-10465	NS_000200.1	-	252426520	252426471
Sma-1-10466	NS_000200.1	-	251784528	251784479
Sma-1-10467	NS_000200.1	-	250997691	250997642
Sma-1-10468	NS_000200.1	-	250851386	250851337
Sma-1-10469	NS_000200.1	-	248914006	248913957
Sma-1-10470	NS_000200.1	-	248894729	248894680
Sma-1-10471	NS_000200.1	-	246179152	246179103
Sma-1-10472	NS_000200.1	-	246030041	246029992
Sma-1-10473	NS_000200.1	-	245683482	245683433
Sma-1-10474	NS_000200.1	-	245141430	245141381
Sma-1-10475	NS_000200.1	-	240644290	240644241
Sma-1-10476	NS_000200.1	-	236653678	236653629
Sma-1-10477	NS_000200.1	-	236564529	236564480
Sma-1-10478	NS_000200.1	-	234491878	234491829
Sma-1-10479	NS_000200.1	-	233691706	233691657
Sma-1-10480	NS_000200.1	-	233249613	233249564
Sma-1-10481	NS_000200.1	-	232949927	232949878
Sma-1-10482	NS_000200.1	-	230738387	230738338
Sma-1-10483	NS_000200.1	-	222339094	222339045
Sma-1-10484	NS_000200.1	-	222046976	222046927
Sma-1-10485	NS_000200.1	-	221245261	221245212
Sma-1-10486	NS_000200.1	-	220954796	220954747
Sma-1-10487	NS_000200.1	-	220080336	220080287
Sma-1-10488	NS_000200.1	-	219959725	219959676
Sma-1-10489	NS_000200.1	-	219738205	219738156
Sma-1-10490	NS_000200.1	-	219436462	219436413
Sma-1-10491	NS_000200.1	-	219248845	219248796
Sma-1-10492	NS_000200.1	-	218816606	218816557
Sma-1-10493	NS_000200.1	-	217645214	217645165
Sma-1-10494	NS_000200.1	-	213576679	213576630
Sma-1-10495	NS_000200.1	-	213023192	213023143
Sma-1-10496	NS_000200.1	-	212909360	212909311
Sma-1-10497	NS_000200.1	-	212895304	212895255
Sma-1-10498	NS_000200.1	-	212842523	212842474
Sma-1-10499	NS_000200.1	-	212484641	212484592
Sma-1-10500	NS_000200.1	-	212127017	212126968
Sma-1-10501	NS_000200.1	-	210318670	210318621
Sma-1-10502	NS_000200.1	-	209225452	209225403
Sma-1-10503	NS_000200.1	-	208624602	208624553
Sma-1-10504	NS_000200.1	-	207727389	207727340
Sma-1-10505	NS_000200.1	-	206163094	206163045
Sma-1-10506	NS_000200.1	-	205185346	205185297
Sma-1-10507	NS_000200.1	-	204415510	204415461
Sma-1-10508	NS_000200.1	-	204178974	204178925

Sma-1-10509	NS_000200.1	-	203972490	203972441
Sma-1-10510	NS_000200.1	-	203055573	203055524
Sma-1-10511	NS_000200.1	-	202935576	202935527
Sma-1-10512	NS_000200.1	-	202781271	202781222
Sma-1-10513	NS_000200.1	-	202778545	202778496
Sma-1-10514	NS_000200.1	-	198786857	198786808
Sma-1-10515	NS_000200.1	-	198431453	198431404
Sma-1-10516	NS_000200.1	-	197985459	197985410
Sma-1-10517	NS_000200.1	-	197508539	197508490
Sma-1-10518	NS_000200.1	-	197462637	197462588
Sma-1-10519	NS_000200.1	-	193504901	193504852
Sma-1-10520	NS_000200.1	-	192627718	192627669
Sma-1-10521	NS_000200.1	-	192193650	192193601
Sma-1-10522	NS_000200.1	-	189547043	189546994
Sma-1-10523	NS_000200.1	-	189476260	189476211
Sma-1-10524	NS_000200.1	-	188748714	188748665
Sma-1-10525	NS_000200.1	-	188098722	188098673
Sma-1-10526	NS_000200.1	-	187494835	187494786
Sma-1-10527	NS_000200.1	-	187306827	187306778
Sma-1-10528	NS_000200.1	-	184210379	184210330
Sma-1-10529	NS_000200.1	-	184125258	184125209
Sma-1-10530	NS_000200.1	-	183098186	183098137
Sma-1-10531	NS_000200.1	-	182591372	182591323
Sma-1-10532	NS_000200.1	-	182223871	182223822
Sma-1-10533	NS_000200.1	-	180679426	180679377
Sma-1-10534	NS_000200.1	-	180016900	180016851
Sma-1-10535	NS_000200.1	-	179704257	179704208
Sma-1-10536	NS_000200.1	-	179143123	179143074
Sma-1-10537	NS_000200.1	-	179122598	179122549
Sma-1-10538	NS_000200.1	-	178352310	178352261
Sma-1-10539	NS_000200.1	-	177430135	177430086
Sma-1-10540	NS_000200.1	-	176065191	176065142
Sma-1-10541	NS_000200.1	-	175315931	175315882
Sma-1-10542	NS_000200.1	-	174369233	174369184
Sma-1-10543	NS_000200.1	-	169634044	169633995
Sma-1-10544	NS_000200.1	-	169579187	169579138
Sma-1-10545	NS_000200.1	-	169372678	169372629
Sma-1-10546	NS_000200.1	-	168530738	168530689
Sma-1-10547	NS_000200.1	-	168510963	168510914
Sma-1-10548	NS_000200.1	-	168413394	168413345
Sma-1-10549	NS_000200.1	-	167582386	167582337
Sma-1-10550	NS_000200.1	-	166649657	166649608
Sma-1-10551	NS_000200.1	-	164660192	164660143
Sma-1-10552	NS_000200.1	-	163886234	163886185
Sma-1-10553	NS_000200.1	-	163564424	163564375
Sma-1-10554	NS_000200.1	-	157194529	157194480
Sma-1-10555	NS_000200.1	-	156619578	156619529
Sma-1-10556	NS_000200.1	-	156391780	156391731
Sma-1-10557	NS_000200.1	-	155984666	155984617
Sma-1-10558	NS_000200.1	-	155984265	155984216
Sma-1-10559	NS_000200.1	-	153814321	153814272
Sma-1-10560	NS_000200.1	-	153179368	153179319

Sma-1-10561	NS_000200.1	-	149530483	149530434
Sma-1-10562	NS_000200.1	-	149060460	149060411
Sma-1-10563	NS_000200.1	-	148269399	148269350
Sma-1-10564	NS_000200.1	-	144588572	144588523
Sma-1-10565	NS_000200.1	-	144371794	144371745
Sma-1-10566	NS_000200.1	-	140805402	140805353
Sma-1-10567	NS_000200.1	-	139893806	139893757
Sma-1-10568	NS_000200.1	-	139150623	139150574
Sma-1-10569	NS_000200.1	-	138149369	138149320
Sma-1-10570	NS_000200.1	-	135748322	135748273
Sma-1-10571	NS_000200.1	-	134142076	134142027
Sma-1-10572	NS_000200.1	-	133198841	133198792
Sma-1-10573	NS_000200.1	-	132466614	132466565
Sma-1-10574	NS_000200.1	-	130057593	130057544
Sma-1-10575	NS_000200.1	-	128391971	128391922
Sma-1-10576	NS_000200.1	-	126782523	126782474
Sma-1-10577	NS_000200.1	-	125527797	125527748
Sma-1-10578	NS_000200.1	-	125425029	125424980
Sma-1-10579	NS_000200.1	-	122504072	122504023
Sma-1-10580	NS_000200.1	-	120661150	120661101
Sma-1-10581	NS_000200.1	-	120195579	120195530
Sma-1-10582	NS_000200.1	-	116585537	116585488
Sma-1-10583	NS_000200.1	-	113978815	113978766
Sma-1-10584	NS_000200.1	-	113593123	113593074
Sma-1-10585	NS_000200.1	-	113592722	113592673
Sma-1-10586	NS_000200.1	-	113340451	113340402
Sma-1-10587	NS_000200.1	-	112860243	112860194
Sma-1-10588	NS_000200.1	-	112738040	112737991
Sma-1-10589	NS_000200.1	-	112708730	112708681
Sma-1-10590	NS_000200.1	-	112355049	112355000
Sma-1-10591	NS_000200.1	-	110228721	110228672
Sma-1-10592	NS_000200.1	-	107259881	107259832
Sma-1-10593	NS_000200.1	-	106619011	106618962
Sma-1-10594	NS_000200.1	-	104940286	104940237
Sma-1-10595	NS_000200.1	-	104158524	104158475
Sma-1-10596	NS_000200.1	-	103466499	103466450
Sma-1-10597	NS_000200.1	-	101560735	101560686
Sma-1-10598	NS_000200.1	-	95824518	95824469
Sma-1-10599	NS_000200.1	-	91218071	91218022
Sma-1-10600	NS_000200.1	-	90097331	90097282
Sma-1-10601	NS_000200.1	-	89330567	89330518
Sma-1-10602	NS_000200.1	-	89094435	89094386
Sma-1-10603	NS_000200.1	-	87709813	87709764
Sma-1-10604	NS_000200.1	-	87709412	87709363
Sma-1-10605	NS_000200.1	-	87709011	87708962
Sma-1-10606	NS_000200.1	-	87697157	87697108
Sma-1-10607	NS_000200.1	-	87346157	87346108
Sma-1-10608	NS_000200.1	-	85164379	85164330
Sma-1-10609	NS_000200.1	-	83710603	83710554
Sma-1-10610	NS_000200.1	-	83091685	83091636
Sma-1-10611	NS_000200.1	-	81400590	81400541
Sma-1-10612	NS_000200.1	-	80590562	80590513

Sma-1-10613	NS_000200.1	-	80131433	80131384
Sma-1-10614	NS_000200.1	-	79724648	79724599
Sma-1-10615	NS_000200.1	-	77397725	77397676
Sma-1-10616	NS_000200.1	-	75173633	75173584
Sma-1-10617	NS_000200.1	-	71329763	71329714
Sma-1-10618	NS_000200.1	-	71161626	71161577
Sma-1-10619	NS_000200.1	-	70609058	70609009
Sma-1-10620	NS_000200.1	-	69227784	69227735
Sma-1-10621	NS_000200.1	-	68158175	68158126
Sma-1-10622	NS_000200.1	-	67585743	67585694
Sma-1-10623	NS_000200.1	-	63173810	63173761
Sma-1-10624	NS_000200.1	-	63048531	63048482
Sma-1-10625	NS_000200.1	-	62125614	62125565
Sma-1-10626	NS_000200.1	-	60959878	60959829
Sma-1-10627	NS_000200.1	-	60072669	60072620
Sma-1-10628	NS_000200.1	-	59536943	59536894
Sma-1-10629	NS_000200.1	-	57446530	57446481
Sma-1-10630	NS_000200.1	-	55759610	55759561
Sma-1-10631	NS_000200.1	-	54689909	54689860
Sma-1-10632	NS_000200.1	-	54686193	54686144
Sma-1-10633	NS_000200.1	-	53922276	53922227
Sma-1-10634	NS_000200.1	-	53026318	53026269
Sma-1-10635	NS_000200.1	-	49946226	49946177
Sma-1-10636	NS_000200.1	-	47644346	47644297
Sma-1-10637	NS_000200.1	-	46827469	46827420
Sma-1-10638	NS_000200.1	-	46652978	46652929
Sma-1-10639	NS_000200.1	-	44304499	44304450
Sma-1-10640	NS_000200.1	-	41054793	41054744
Sma-1-10641	NS_000200.1	-	36897442	36897393
Sma-1-10642	NS_000200.1	-	35659229	35659180
Sma-1-10643	NS_000200.1	-	35180520	35180471
Sma-1-10644	NS_000200.1	-	34177486	34177437
Sma-1-10645	NS_000200.1	-	33730227	33730178
Sma-1-10646	NS_000200.1	-	32532200	32532151
Sma-1-10647	NS_000200.1	-	32036001	32035952
Sma-1-10648	NS_000200.1	-	31812848	31812799
Sma-1-10649	NS_000200.1	-	25517365	25517316
Sma-1-10650	NS_000200.1	-	24318281	24318232
Sma-1-10651	NS_000200.1	-	23686957	23686908
Sma-1-10652	NS_000200.1	-	21897581	21897532
Sma-1-10653	NS_000200.1	-	20564032	20563983
Sma-1-10654	NS_000200.1	-	19386074	19386025
Sma-1-10655	NS_000200.1	-	18403803	18403754
Sma-1-10656	NS_000200.1	-	18131543	18131494
Sma-1-10657	NS_000200.1	-	16296275	16296226
Sma-1-10658	NS_000200.1	-	14992319	14992270
Sma-1-10659	NS_000200.1	-	14647921	14647872
Sma-1-10660	NS_000200.1	-	12930518	12930469
Sma-1-10661	NS_000200.1	-	9766954	9766905
Sma-1-10662	NS_000200.1	-	9447263	9447214
Sma-1-10663	NS_000200.1	-	9446863	9446814
Sma-1-10664	NS_000200.1	-	9388069	9388020

Sma-1-10665	NS_000200.1	-	7426635	7426586
Sma-1-10666	NS_000200.1	-	6907742	6907693
Sma-1-10667	NS_000200.1	-	5256618	5256569
Sma-1-10668	NS_000200.1	-	5058570	5058521
Sma-1-10669	NS_000200.1	-	3339965	3339916
Sma-1-10670	NS_000200.1	-	2825011	2824962
Sma-1-10671	NS_000200.1	-	1568160	1568111
Sma-1-10672	NS_000200.1	-	1078388	1078339
Sma-1-10673	NS_000200.1	-	441706	441657
Sma-1-10674	NW_003037872.1	-	379721	379676
Sma-1-10675	NS_000200.1	-	118336727	118336682
Sma-1-10676	NW_003037834.1	-	902781	902732
Sma-1-10677	NW_003037914.1	+	679313	679362
Sma-1-10678	NW_003037981.1	+	489699	489748
Sma-1-10679	NS_000200.1	+	160823563	160823612
Sma-1-10680	NS_000200.1	+	212887567	212887616
Sma-1-10681	NS_000200.1	-	74063267	74063218
Sma-1-10682	NW_003037874.1	-	18742	18693
Sma-1-10683	NS_000200.1	-	119443692	119443643
Sma-1-10684	NW_003037977.1	-	209986	209937
Sma-1-10685	NS_000200.1	-	208496006	208495957
Sma-1-10686	NW_003037995.1	-	540367	540318
Sma-1-10687	NS_000200.1	-	220230135	220230086
Sma-1-10688	NW_003038073.1	+	282342	282394
Sma-1-10689	NW_003038305.1	-	1625	1573
Sma-1-10690	NS_000200.1	+	265804775	265804827
Sma-1-10691	NS_000200.1	-	325748411	325748359
Sma-1-10692	NW_003038124.1	+	19225	19274
Sma-1-10693	NS_000200.1	+	279011869	279011918
Sma-1-10694	NW_003038217.1	-	50924	50875
Sma-1-10695	NW_003037913.1	+	652070	652119
Sma-1-10696	NS_000200.1	+	159215821	159215870
Sma-1-10697	NS_000200.1	-	307212732	307212683
Sma-1-10698	NW_003038111.1	-	19921	19872
Sma-1-10699	NS_000200.1	-	276339684	276339635
Sma-1-10700	NW_003030236.1	+	3903	3952
Sma-1-10701	NW_003037831.1	+	1739393	1739442
Sma-1-10702	NW_003037845.1	+	176793	176842
Sma-1-10703	NW_003037917.1	+	572224	572273
Sma-1-10704	NS_000200.1	+	71188695	71188744
Sma-1-10705	NS_000200.1	+	87178696	87178745
Sma-1-10706	NS_000200.1	+	165609028	165609077
Sma-1-10707	NS_000200.1	+	362438384	362438433
Sma-1-10708	NW_003037937.1	+	556449	556498
Sma-1-10709	NW_003037937.1	+	559055	559104
Sma-1-10710	NS_000200.1	+	181756358	181756407
Sma-1-10711	NS_000200.1	+	181758964	181759013
Sma-1-10712	NW_003037938.1	-	787859	787810
Sma-1-10713	NS_000200.1	-	182824972	182824923
Sma-1-10714	NW_003026471.1	+	122786	122835
Sma-1-10715	NS_000200.1	+	222235019	222235068
Sma-1-10716	NW_003038037.1	+	611372	611421

Sma-1-10717	NS_000200.1	+	250766781	250766830
Sma-1-10718	NW_003038014.1	+	363585	363634
Sma-1-10719	NS_000200.1	+	230104274	230104323
Sma-1-10720	NW_003038070.1	+	29331	29380
Sma-1-10721	NS_000200.1	+	264366572	264366621
Sma-1-10722	NW_003037795.1	-	1183182	1183133
Sma-1-10723	NS_000200.1	-	7574065	7574016
Sma-1-10724	NW_003037797.1	-	3334456	3334407
Sma-1-10725	NS_000200.1	-	13762316	13762267
Sma-1-10726	NW_003037948.1	+	233230	233279
Sma-1-10727	NS_000200.1	+	192253174	192253223
Sma-1-10728	NW_003038228.1	+	148852	148901
Sma-1-10729	NS_000200.1	+	311022132	311022181
Sma-1-10730	NW_003037794.1	+	434017	434066
Sma-1-10731	NS_000200.1	+	4613612	4613661
Sma-1-10732	NW_003037841.1	-	554898	554849
Sma-1-10733	NS_000200.1	-	83166079	83166030
Sma-1-10734	NW_003024070.1	+	1530	1579
Sma-1-10735	NW_003037836.1	-	848026	847977
Sma-1-10736	NS_000200.1	+	344578940	344578989
Sma-1-10737	NS_000200.1	-	76405125	76405076
Sma-1-10738	NW_003037841.1	-	229427	229378
Sma-1-10739	NS_000200.1	-	82840608	82840559
Sma-1-10740	NW_003037850.1	+	157636	157685
Sma-1-10741	NW_003037976.1	-	204900	204851
Sma-1-10742	NW_003037980.1	-	903464	903415
Sma-1-10743	NW_003038017.1	-	95771	95722
Sma-1-10744	NS_000200.1	+	92143666	92143715
Sma-1-10745	NS_000200.1	-	231861802	231861753
Sma-1-10746	NS_000200.1	-	211864326	211864277
Sma-1-10747	NS_000200.1	-	206819215	206819166
Sma-1-10748	NW_003026456.1	-	306023	305974
Sma-1-10749	NW_003037853.1	-	1320574	1320525
Sma-1-10750	NS_000200.1	-	96812625	96812576
Sma-1-10751	NS_000200.1	-	58229159	58229110
Sma-1-10752	NW_003026466.1	-	61141	61092
Sma-1-10753	NS_000200.1	-	215328248	215328199
Sma-1-10754	NW_003038261.1	+	437098	437147
Sma-1-10755	NS_000200.1	+	324050749	324050798
Sma-1-10756	NW_003038036.1	+	371837	371886
Sma-1-10757	NS_000200.1	+	249305689	249305738
Sma-1-10758	NW_003026618.1	+	101642	101691
Sma-1-10759	NW_003026466.1	-	33500	33451
Sma-1-10760	NS_000200.1	+	321856644	321856693
Sma-1-10761	NS_000200.1	-	215300607	215300558
Sma-1-10762	NW_003037937.1	+	453259	453308
Sma-1-10763	NS_000200.1	+	181653168	181653217
Sma-1-10764	NW_003037942.1	+	338169	338218
Sma-1-10765	NS_000200.1	+	186098077	186098126
Sma-1-10766	NW_003039130.1	+	152962	153011
Sma-1-10767	NS_000200.1	+	317827573	317827622
Sma-1-10768	NW_003037809.1	+	173158	173207

Sma-1-10769	NW_003038015.1	+	473034	473083
Sma-1-10770	NW_003038149.1	-	41103	41054
Sma-1-10771	NS_000200.1	+	33837622	33837671
Sma-1-10772	NS_000200.1	+	230796524	230796573
Sma-1-10773	NS_000200.1	-	291069716	291069667
Sma-1-10774	NW_003031903.1	+	141	190
Sma-1-10775	NW_003026471.1	+	67393	67442
Sma-1-10776	NW_003026456.1	+	325247	325296
Sma-1-10777	NW_003037794.1	-	1005373	1005324
Sma-1-10778	NW_003037794.1	-	952684	952635
Sma-1-10779	NW_003037795.1	+	1104760	1104809
Sma-1-10780	NW_003037847.1	-	881089	881040
Sma-1-10781	NW_003037866.1	+	1415446	1415495
Sma-1-10782	NS_000200.1	+	7495643	7495692
Sma-1-10783	NS_000200.1	+	58248383	58248432
Sma-1-10784	NS_000200.1	+	112903245	112903294
Sma-1-10785	NS_000200.1	+	222179626	222179675
Sma-1-10786	NS_000200.1	+	367238650	367238699
Sma-1-10787	NS_000200.1	-	89532819	89532770
Sma-1-10788	NS_000200.1	-	5184968	5184919
Sma-1-10789	NS_000200.1	-	5132279	5132230
Sma-1-10790	NW_003037798.1	-	2217106	2217057
Sma-1-10791	NW_003037859.1	+	679615	679664
Sma-1-10792	NS_000200.1	+	103358456	103358505
Sma-1-10793	NS_000200.1	-	16476264	16476215
Sma-1-10794	NW_003037973.1	-	82326	82277
Sma-1-10795	NS_000200.1	-	204064053	204064004
Sma-1-10796	NW_003037873.1	+	678822	678871
Sma-1-10797	NW_003026467.1	-	26706	26657
Sma-1-10798	NW_003037799.1	+	770664	770713
Sma-1-10799	NW_003037917.1	-	609401	609352
Sma-1-10800	NW_003037935.1	+	782950	782999
Sma-1-10801	NS_000200.1	+	17761018	17761067
Sma-1-10802	NS_000200.1	+	119417950	119417999
Sma-1-10803	NS_000200.1	+	180199485	180199534
Sma-1-10804	NS_000200.1	-	215619923	215619874
Sma-1-10805	NS_000200.1	-	165646205	165646156
Sma-1-10806	NW_003038505.1	-	162450	162401
Sma-1-10807	NS_000200.1	-	336241787	336241738
Sma-1-10808	NW_003037801.1	-	513206	513157
Sma-1-10809	NW_003037832.1	+	487866	487915
Sma-1-10810	NW_003037946.1	-	269446	269397
Sma-1-10811	NS_000200.1	+	71697297	71697346
Sma-1-10812	NS_000200.1	-	190963306	190963257
Sma-1-10813	NS_000200.1	-	21602686	21602637
Sma-1-10814	NW_003038509.1	-	25377	25328
Sma-1-10815	NS_000200.1	-	337657829	337657780
Sma-1-10816	NW_003037901.1	-	15956	15907
Sma-1-10817	NS_000200.1	-	145864417	145864368
Sma-1-10818	NW_003037854.1	-	1912071	1912022
Sma-1-10819	NW_003037969.1	+	229812	229861
Sma-1-10820	NS_000200.1	+	200835939	200835988

Sma-1-10821	NS_000200.1	-	98816492	98816443
Sma-1-10822	NW_003038067.1	+	21001	21050
Sma-1-10823	NS_000200.1	+	262875054	262875103
Sma-1-10824	NW_003037797.1	-	2208145	2208096
Sma-1-10825	NS_000200.1	-	12636005	12635956
Sma-1-10826	NW_003030509.1	-	92	43
Sma-1-10827	NW_003037923.1	-	156606	156557
Sma-1-10828	NS_000200.1	-	363076112	363076063
Sma-1-10829	NS_000200.1	-	171212819	171212770
Sma-1-10830	NW_003035881.1	-	295440	295391
Sma-1-10831	NS_000200.1	-	341359645	341359596
Sma-1-10832	NW_003037798.1	-	82520	82471
Sma-1-10833	NS_000200.1	-	14341678	14341629
Sma-1-10834	NW_003037891.1	-	1093230	1093181
Sma-1-10835	NS_000200.1	-	138974508	138974459
Sma-1-10836	NW_003037846.1	+	912534	912583
Sma-1-10837	NS_000200.1	+	88547104	88547153
Sma-1-10838	NW_003037944.1	+	208730	208779
Sma-1-10839	NS_000200.1	+	188430083	188430132
Sma-1-10840	NW_003037794.1	-	1086679	1086630
Sma-1-10841	NW_003037858.1	+	1131509	1131558
Sma-1-10842	NS_000200.1	+	101795785	101795834
Sma-1-10843	NS_000200.1	-	5266274	5266225
Sma-1-10844	NW_003037845.1	-	295475	295426
Sma-1-10845	NW_003038160.1	-	33071	33022
Sma-1-10846	NS_000200.1	-	296785002	296784953
Sma-1-10847	NS_000200.1	-	87297378	87297329
Sma-1-10848	NW_003037844.1	-	432736	432687
Sma-1-10849	NS_000200.1	-	86616182	86616133
Sma-1-10850	NW_003037841.1	+	987938	987987
Sma-1-10851	NW_003037909.1	-	236929	236880
Sma-1-10852	NS_000200.1	+	83599119	83599168
Sma-1-10853	NS_000200.1	-	153778578	153778529
Sma-1-10854	NW_003037900.1	+	62615	62664
Sma-1-10855	NS_000200.1	+	145191481	145191530
Sma-1-10856	NW_003037902.1	-	206734	206685
Sma-1-10857	NW_003037909.1	+	708760	708809
Sma-1-10858	NS_000200.1	+	154250409	154250458
Sma-1-10859	NS_000200.1	-	146961968	146961919
Sma-1-10860	NW_003037830.1	-	720211	720162
Sma-1-10861	NS_000200.1	-	68610582	68610533
Sma-1-10862	NW_003037829.1	-	1696400	1696351
Sma-1-10863	NS_000200.1	-	67596938	67596889
Sma-1-10864	NW_003037809.1	+	1499907	1499956
Sma-1-10865	NS_000200.1	+	35164371	35164420
Sma-1-10866	NW_003037935.1	+	22444	22493
Sma-1-10867	NS_000200.1	+	179438979	179439028
Sma-1-10868	NW_003026599.1	-	135643	135594
Sma-1-10869	NS_000200.1	-	306013972	306013923
Sma-1-10870	NW_003026599.1	-	129875	129826
Sma-1-10871	NS_000200.1	-	306008204	306008155
Sma-1-10872	NW_003028946.1	-	2236	2187

Sma-1-10873	NW_003037859.1	+	480760	480809
Sma-1-10874	NW_003037967.1	+	439757	439806
Sma-1-10875	NS_000200.1	+	103159601	103159650
Sma-1-10876	NS_000200.1	+	199679728	199679777
Sma-1-10877	NS_000200.1	-	374957102	374957053
Sma-1-10878	NW_003037942.1	+	326932	326981
Sma-1-10879	NS_000200.1	+	186086840	186086889
Sma-1-10880	NW_003037798.1	+	701896	701945
Sma-1-10881	NS_000200.1	+	14961054	14961103
Sma-1-10882	NW_003037814.1	-	830181	830132
Sma-1-10883	NS_000200.1	-	43814041	43813992
Sma-1-10884	NW_003037820.1	+	207659	207708
Sma-1-10885	NW_003037885.1	+	407240	407289
Sma-1-10886	NW_003026516.1	+	277124	277173
Sma-1-10887	NS_000200.1	+	52480641	52480690
Sma-1-10888	NS_000200.1	+	128505187	128505236
Sma-1-10889	NS_000200.1	+	299385421	299385470
Sma-1-10890	NW_003037827.1	-	24510	24461
Sma-1-10891	NS_000200.1	-	62646651	62646602
Sma-1-10892	NW_003037827.1	+	1461544	1461593
Sma-1-10893	NS_000200.1	+	64083685	64083734
Sma-1-10894	NW_003037823.1	+	395757	395806
Sma-1-10895	NS_000200.1	+	56312376	56312425
Sma-1-10896	NW_003037818.1	-	664576	664527
Sma-1-10897	NS_000200.1	-	49379983	49379934
Sma-1-10898	NW_003037829.1	-	698862	698813
Sma-1-10899	NS_000200.1	-	66599400	66599351
Sma-1-10900	NW_003037923.1	+	252464	252513
Sma-1-10901	NS_000200.1	+	171308677	171308726
Sma-1-10902	NW_003036826.1	-	1574	1525
Sma-1-10903	NS_000200.1	-	347801463	347801414
Sma-1-10904	NW_003037836.1	+	932096	932145
Sma-1-10905	NW_003038518.1	-	318646	318597
Sma-1-10906	NS_000200.1	+	76489195	76489244
Sma-1-10907	NS_000200.1	-	341955479	341955430
Sma-1-10908	NW_003037926.1	+	40323	40372
Sma-1-10909	NS_000200.1	+	173208593	173208642
Sma-1-10910	NW_003037837.1	-	1012084	1012035
Sma-1-10911	NW_003037866.1	-	1030624	1030575
Sma-1-10912	NW_003037941.1	-	370391	370342
Sma-1-10913	NW_003038065.1	+	26768	26817
Sma-1-10914	NS_000200.1	+	262178385	262178434
Sma-1-10915	NS_000200.1	-	185104865	185104816
Sma-1-10916	NS_000200.1	-	112518423	112518374
Sma-1-10917	NS_000200.1	-	77817942	77817893
Sma-1-10918	NW_003037797.1	-	2930107	2930058
Sma-1-10919	NS_000200.1	-	13357967	13357918
Sma-1-10920	NW_003026471.1	-	175055	175006
Sma-1-10921	NW_003037995.1	+	16022	16071
Sma-1-10922	NS_000200.1	+	219705790	219705839
Sma-1-10923	NS_000200.1	-	222287288	222287239
Sma-1-10924	NW_003026471.1	+	176693	176742

Sma-1-10925	NS_000200.1	+	222288926	222288975
Sma-1-10926	NW_003037943.1	+	1125383	1125432
Sma-1-10927	NS_000200.1	+	188146916	188146965
Sma-1-10928	NW_003038145.1	-	229783	229734
Sma-1-10929	NS_000200.1	-	288841206	288841157
Sma-1-10930	NW_003026512.1	+	293425	293474
Sma-1-10931	NS_000200.1	+	285387465	285387514
Sma-1-10932	NW_003026502.1	+	32469	32518
Sma-1-10933	NW_003034610.1	-	1181	1132
Sma-1-10934	NW_003037979.1	+	181744	181793
Sma-1-10935	NS_000200.1	+	210242764	210242813
Sma-1-10936	NS_000200.1	+	276068905	276068954
Sma-1-10937	NS_000200.1	-	327927256	327927207
Sma-1-10938	NW_003034610.1	-	1974	1925
Sma-1-10939	NS_000200.1	-	327928049	327928000
Sma-1-10940	NW_003037963.1	-	41904	41855
Sma-1-10941	NS_000200.1	-	197320116	197320067
Sma-1-10942	NW_003038147.1	+	24004	24053
Sma-1-10943	NS_000200.1	+	289551747	289551796
Sma-1-10944	NW_003037831.1	+	1637362	1637411
Sma-1-10945	NW_003037831.1	-	845788	845739
Sma-1-10946	NS_000200.1	+	71086664	71086713
Sma-1-10947	NS_000200.1	-	70295090	70295041
Sma-1-10948	NW_003037853.1	-	833972	833923
Sma-1-10949	NS_000200.1	-	96326023	96325974
Sma-1-10950	NW_003038258.1	-	154191	154142
Sma-1-10951	NS_000200.1	-	322692701	322692652
Sma-1-10952	NW_003032045.1	-	4985	4936
Sma-1-10953	NW_003038221.1	-	102084	102035
Sma-1-10954	NW_003037795.1	-	1227767	1227718
Sma-1-10955	NW_003037797.1	-	3072642	3072593
Sma-1-10956	NW_003037807.1	+	301196	301245
Sma-1-10957	NW_003037887.1	+	842464	842513
Sma-1-10958	NW_003037889.1	+	1214122	1214171
Sma-1-10959	NW_003037890.1	-	1491190	1491141
Sma-1-10960	NW_003037912.1	-	159393	159344
Sma-1-10961	NW_003037940.1	+	806224	806273
Sma-1-10962	NW_003037981.1	+	619392	619441
Sma-1-10963	NW_003038014.1	+	213431	213480
Sma-1-10964	NW_003038224.1	+	63179	63228
Sma-1-10965	NW_003038252.1	+	33238	33287
Sma-1-10966	NW_003038083.1	-	625901	625852
Sma-1-10967	NW_003038152.1	-	384258	384209
Sma-1-10968	NS_000200.1	+	30562712	30562761
Sma-1-10969	NS_000200.1	+	131306442	131306491
Sma-1-10970	NS_000200.1	+	134990612	134990661
Sma-1-10971	NS_000200.1	+	184548354	184548403
Sma-1-10972	NS_000200.1	+	213017260	213017309
Sma-1-10973	NS_000200.1	+	229954120	229954169
Sma-1-10974	NS_000200.1	+	309474907	309474956
Sma-1-10975	NS_000200.1	+	318888826	318888875
Sma-1-10976	NS_000200.1	-	376985063	376985014

Sma-1-10977	NS_000200.1	-	308743348	308743299
Sma-1-10978	NS_000200.1	-	292989982	292989933
Sma-1-10979	NS_000200.1	-	270869238	270869189
Sma-1-10980	NS_000200.1	-	156970968	156970919
Sma-1-10981	NS_000200.1	-	137174551	137174502
Sma-1-10982	NS_000200.1	-	13500502	13500453
Sma-1-10983	NS_000200.1	-	7618650	7618601
Sma-1-10984	NW_003035881.1	-	74970	74921
Sma-1-10985	NS_000200.1	-	341139175	341139126
Sma-1-10986	NW_003038147.1	+	149855	149904
Sma-1-10987	NS_000200.1	+	289677598	289677647
Sma-1-10988	NW_003039130.1	+	189679	189728
Sma-1-10989	NS_000200.1	+	317864290	317864339
Sma-1-10990	NW_003020966.1	+	480	529
Sma-1-10991	NW_003023078.1	+	1196	1245
Sma-1-10992	NW_003037789.1	+	25055	25104
Sma-1-10993	NW_003037921.1	-	379324	379275
Sma-1-10994	NW_003037978.1	+	406121	406170
Sma-1-10995	NW_003038072.1	+	3624	3673
Sma-1-10996	NS_000200.1	+	209674131	209674180
Sma-1-10997	NS_000200.1	+	265165852	265165901
Sma-1-10998	NS_000200.1	+	367698557	367698606
Sma-1-10999	NS_000200.1	+	372719992	372720041
Sma-1-11000	NS_000200.1	+	380809675	380809724
Sma-1-11001	NS_000200.1	-	169909459	169909410
Sma-1-11002	NW_003030576.1	-	1276	1227
Sma-1-11003	NW_003033936.1	+	557	606
Sma-1-11004	NW_003037798.1	+	1076427	1076476
Sma-1-11005	NW_003037808.1	+	1717344	1717393
Sma-1-11006	NW_003037827.1	-	691741	691692
Sma-1-11007	NW_003037841.1	+	558680	558729
Sma-1-11008	NW_003037866.1	+	301288	301337
Sma-1-11009	NW_003037911.1	-	675379	675330
Sma-1-11010	NW_003037964.1	-	497555	497506
Sma-1-11011	NW_003038037.1	+	18980	19029
Sma-1-11012	NW_003038476.1	-	56210	56161
Sma-1-11013	NW_003026486.1	-	138168	138119
Sma-1-11014	NW_003026511.1	-	193604	193555
Sma-1-11015	NW_003038152.1	-	44244	44195
Sma-1-11016	NS_000200.1	+	15335585	15335634
Sma-1-11017	NS_000200.1	+	33503981	33504030
Sma-1-11018	NS_000200.1	+	83169861	83169910
Sma-1-11019	NS_000200.1	+	111789087	111789136
Sma-1-11020	NS_000200.1	+	250174389	250174438
Sma-1-11021	NS_000200.1	+	363265607	363265656
Sma-1-11022	NS_000200.1	-	363207535	363207486
Sma-1-11023	NS_000200.1	-	332444585	332444536
Sma-1-11024	NS_000200.1	-	292649968	292649919
Sma-1-11025	NS_000200.1	-	284576656	284576607
Sma-1-11026	NS_000200.1	-	259759287	259759238
Sma-1-11027	NS_000200.1	-	198099077	198099028
Sma-1-11028	NS_000200.1	-	156462732	156462683

Sma-1-11029	NS_000200.1	-	63313882	63313833
Sma-1-11030	NW_003038028.1	+	407766	407815
Sma-1-11031	NS_000200.1	+	241715340	241715389
Sma-1-11032	NW_003037786.1	-	137514	137465
Sma-1-11033	NW_003037847.1	-	740579	740530
Sma-1-11034	NW_003038506.1	-	304799	304750
Sma-1-11035	NW_003038518.1	-	735765	735716
Sma-1-11036	NS_000200.1	-	380230874	380230825
Sma-1-11037	NS_000200.1	-	342372598	342372549
Sma-1-11038	NS_000200.1	-	336666638	336666589
Sma-1-11039	NS_000200.1	-	89392309	89392260
Sma-1-11040	NW_003039130.1	-	203468	203419
Sma-1-11041	NW_003037853.1	+	236492	236541
Sma-1-11042	NW_003038011.1	-	599265	599216
Sma-1-11043	NW_003038025.1	+	18870	18919
Sma-1-11044	NW_003038145.1	+	26442	26491
Sma-1-11045	NS_000200.1	+	95728543	95728592
Sma-1-11046	NS_000200.1	+	238877115	238877164
Sma-1-11047	NS_000200.1	+	288637865	288637914
Sma-1-11048	NS_000200.1	-	317878079	317878030
Sma-1-11049	NS_000200.1	-	228126915	228126866
Sma-1-11050	NW_003038453.1	+	100322	100371
Sma-1-11051	NS_000200.1	+	330682249	330682298
Sma-1-11052	NW_003020433.1	+	419	468
Sma-1-11053	NW_003020433.1	+	824	873
Sma-1-11054	NW_003020471.1	-	1504	1455
Sma-1-11055	NW_003021417.1	-	1544	1495
Sma-1-11056	NW_003021590.1	-	561	512
Sma-1-11057	NW_003021590.1	-	156	107
Sma-1-11058	NW_003021871.1	-	955	906
Sma-1-11059	NW_003021871.1	-	550	501
Sma-1-11060	NW_003021871.1	-	145	96
Sma-1-11061	NW_003022765.1	+	1538	1587
Sma-1-11062	NW_003023439.1	+	1202	1251
Sma-1-11063	NW_003023454.1	+	2123	2172
Sma-1-11064	NW_003023505.1	-	1506	1457
Sma-1-11065	NW_003023983.1	+	736	785
Sma-1-11066	NW_003024058.1	-	994	945
Sma-1-11067	NW_003024070.1	+	1125	1174
Sma-1-11068	NW_003025400.1	+	147	196
Sma-1-11069	NW_003026129.1	-	1691	1642
Sma-1-11070	NW_003026614.1	-	8232	8183
Sma-1-11071	NW_003026722.1	+	1964	2013
Sma-1-11072	NW_003026722.1	+	10072	10121
Sma-1-11073	NW_003029509.1	-	1026	977
Sma-1-11074	NW_003029807.1	-	3614	3565
Sma-1-11075	NW_003030507.1	+	541	590
Sma-1-11076	NW_003031439.1	-	939	890
Sma-1-11077	NW_003031439.1	-	534	485
Sma-1-11078	NW_003031439.1	-	129	80
Sma-1-11079	NW_003031608.1	+	226	275
Sma-1-11080	NW_003031608.1	+	631	680

Sma-1-11081	NW_003031608.1	+	1036	1085
Sma-1-11082	NW_003033323.1	-	1519	1470
Sma-1-11083	NW_003033740.1	+	34	83
Sma-1-11084	NW_003033991.1	+	1347	1396
Sma-1-11085	NW_003034191.1	+	63	112
Sma-1-11086	NW_003034191.1	+	468	517
Sma-1-11087	NW_003034191.1	+	873	922
Sma-1-11088	NW_003034655.1	+	123	172
Sma-1-11089	NW_003035112.1	-	745	696
Sma-1-11090	NW_003035112.1	-	340	291
Sma-1-11091	NW_003035282.1	-	1041	992
Sma-1-11092	NW_003035930.1	+	1717	1766
Sma-1-11093	NW_003036571.1	-	1597	1548
Sma-1-11094	NW_003036571.1	-	1192	1143
Sma-1-11095	NW_003036571.1	-	787	738
Sma-1-11096	NW_003036571.1	-	382	333
Sma-1-11097	NW_003036692.1	-	1416	1367
Sma-1-11098	NW_003036692.1	-	1011	962
Sma-1-11099	NW_003036692.1	-	606	557
Sma-1-11100	NW_003036692.1	-	201	152
Sma-1-11101	NW_003038253.1	-	70738	70689
Sma-1-11102	NW_003037873.1	+	37062	37111
Sma-1-11103	NW_003037873.1	-	317388	317339
Sma-1-11104	NW_003035878.1	-	281266	281217
Sma-1-11105	NW_003037586.1	-	11494	11445
Sma-1-11106	NW_003039108.1	-	6744	6695
Sma-1-11107	NW_003037962.1	-	318218	318169
Sma-1-11108	NW_003035636.1	+	48262	48311
Sma-1-11109	NW_003035636.1	+	48667	48716
Sma-1-11110	NW_003035880.1	+	348035	348084
Sma-1-11111	NW_003038514.1	-	83593	83544
Sma-1-11112	NW_003037898.1	+	62503	62552
Sma-1-11113	NW_003038218.1	+	75018	75067
Sma-1-11114	NW_003026611.1	+	178516	178565
Sma-1-11115	NW_003037772.1	-	59334	59285
Sma-1-11116	NW_003038228.1	+	147444	147493
Sma-1-11117	NW_003026618.1	+	382895	382944
Sma-1-11118	NW_003037779.1	+	33267	33316
Sma-1-11119	NW_003037779.1	-	31484	31435
Sma-1-11120	NW_003035869.1	+	57073	57122
Sma-1-11121	NW_003037470.1	+	279	328
Sma-1-11122	NW_003026460.1	+	460763	460812
Sma-1-11123	NW_003026465.1	-	698562	698513
Sma-1-11124	NW_003026465.1	-	558378	558329
Sma-1-11125	NW_003035690.1	-	41430	41381
Sma-1-11126	NW_003037238.1	-	196	147
Sma-1-11127	NW_003038326.1	+	2288	2337
Sma-1-11128	NW_003037995.1	+	118714	118763
Sma-1-11129	NW_003037995.1	+	119119	119168
Sma-1-11130	NW_003039130.1	+	249668	249717
Sma-1-11131	NW_003039130.1	+	250073	250122
Sma-1-11132	NW_003038070.1	-	231932	231883

Sma-1-11133	NW_003038250.1	-	264435	264386
Sma-1-11134	NW_003026456.1	-	82603	82554
Sma-1-11135	NW_003037791.1	+	70453	70502
Sma-1-11136	NW_003037793.1	+	763399	763448
Sma-1-11137	NW_003037793.1	+	3173608	3173657
Sma-1-11138	NW_003037793.1	-	4124224	4124175
Sma-1-11139	NW_003037793.1	-	218360	218311
Sma-1-11140	NW_003037794.1	-	1864888	1864839
Sma-1-11141	NW_003037795.1	+	1003244	1003293
Sma-1-11142	NW_003037795.1	+	1571953	1572002
Sma-1-11143	NW_003037796.1	-	1845391	1845342
Sma-1-11144	NW_003037797.1	-	373646	373597
Sma-1-11145	NW_003037798.1	+	1194881	1194930
Sma-1-11146	NW_003037798.1	-	2624108	2624059
Sma-1-11147	NW_003037799.1	-	1250084	1250035
Sma-1-11148	NW_003037799.1	-	1046738	1046689
Sma-1-11149	NW_003037799.1	-	857921	857872
Sma-1-11150	NW_003037799.1	-	178208	178159
Sma-1-11151	NW_003037801.1	+	582479	582528
Sma-1-11152	NW_003037801.1	-	474100	474051
Sma-1-11153	NW_003037803.1	+	467778	467827
Sma-1-11154	NW_003037805.1	+	1380391	1380440
Sma-1-11155	NW_003037806.1	+	288957	289006
Sma-1-11156	NW_003037806.1	-	797923	797874
Sma-1-11157	NW_003037808.1	+	1326285	1326334
Sma-1-11158	NW_003037808.1	-	568140	568091
Sma-1-11159	NW_003037809.1	+	2189085	2189134
Sma-1-11160	NW_003037809.1	-	2757120	2757071
Sma-1-11161	NW_003037809.1	-	1249559	1249510
Sma-1-11162	NW_003037810.1	+	50331	50380
Sma-1-11163	NW_003037810.1	+	1531046	1531095
Sma-1-11164	NW_003037812.1	-	628332	628283
Sma-1-11165	NW_003037813.1	+	1534784	1534833
Sma-1-11166	NW_003037817.1	+	70102	70151
Sma-1-11167	NW_003037820.1	-	716190	716141
Sma-1-11168	NW_003037825.1	-	1031361	1031312
Sma-1-11169	NW_003037830.1	-	176880	176831
Sma-1-11170	NW_003037833.1	+	80424	80473
Sma-1-11171	NW_003037835.1	+	60372	60421
Sma-1-11172	NW_003037836.1	-	4411	4362
Sma-1-11173	NW_003037837.1	-	114073	114024
Sma-1-11174	NW_003037838.1	-	480916	480867
Sma-1-11175	NW_003037840.1	+	961612	961661
Sma-1-11176	NW_003037841.1	-	570524	570475
Sma-1-11177	NW_003037842.1	+	344869	344918
Sma-1-11178	NW_003037842.1	-	115015	114966
Sma-1-11179	NW_003037847.1	+	975218	975267
Sma-1-11180	NW_003037848.1	+	72369	72418
Sma-1-11181	NW_003037848.1	+	439630	439679
Sma-1-11182	NW_003037848.1	+	1188780	1188829
Sma-1-11183	NW_003037849.1	+	297263	297312
Sma-1-11184	NW_003037849.1	-	812079	812030

Sma-1-11185	NW_003037851.1	+	451151	451200
Sma-1-11186	NW_003037851.1	-	450505	450456
Sma-1-11187	NW_003037853.1	+	348493	348542
Sma-1-11188	NW_003037853.1	+	1016442	1016491
Sma-1-11189	NW_003037853.1	+	1016848	1016897
Sma-1-11190	NW_003037853.1	-	1333977	1333928
Sma-1-11191	NW_003037858.1	-	471860	471811
Sma-1-11192	NW_003037858.1	-	411695	411646
Sma-1-11193	NW_003037864.1	+	1562412	1562461
Sma-1-11194	NW_003037864.1	-	488106	488057
Sma-1-11195	NW_003037866.1	+	224416	224465
Sma-1-11196	NW_003037866.1	+	1534745	1534794
Sma-1-11197	NW_003037866.1	-	790166	790117
Sma-1-11198	NW_003037866.1	-	224142	224093
Sma-1-11199	NW_003037866.1	-	77074	77025
Sma-1-11200	NW_003037878.1	-	48426	48377
Sma-1-11201	NW_003037884.1	-	1156666	1156617
Sma-1-11202	NW_003037885.1	+	537924	537973
Sma-1-11203	NW_003037885.1	-	773185	773136
Sma-1-11204	NW_003037896.1	-	75427	75378
Sma-1-11205	NW_003037899.1	-	497249	497200
Sma-1-11206	NW_003037905.1	-	393828	393779
Sma-1-11207	NW_003037906.1	+	449327	449376
Sma-1-11208	NW_003037906.1	+	597025	597074
Sma-1-11209	NW_003037906.1	-	648944	648895
Sma-1-11210	NW_003037909.1	-	626832	626783
Sma-1-11211	NW_003037911.1	-	865542	865493
Sma-1-11212	NW_003037912.1	-	942731	942682
Sma-1-11213	NW_003037913.1	-	1252298	1252249
Sma-1-11214	NW_003037914.1	+	600721	600770
Sma-1-11215	NW_003037914.1	+	1112075	1112124
Sma-1-11216	NW_003037914.1	-	1110968	1110919
Sma-1-11217	NW_003037915.1	+	1080910	1080959
Sma-1-11218	NW_003037915.1	+	1091428	1091477
Sma-1-11219	NW_003037918.1	-	2061250	2061201
Sma-1-11220	NW_003037918.1	-	1556666	1556617
Sma-1-11221	NW_003037918.1	-	1473041	1472992
Sma-1-11222	NW_003037918.1	-	133367	133318
Sma-1-11223	NW_003037938.1	+	770302	770351
Sma-1-11224	NW_003037939.1	-	97887	97838
Sma-1-11225	NW_003037943.1	+	773666	773715
Sma-1-11226	NW_003037943.1	-	715841	715792
Sma-1-11227	NW_003037944.1	-	719407	719358
Sma-1-11228	NW_003037945.1	-	1026581	1026532
Sma-1-11229	NW_003037946.1	+	157274	157323
Sma-1-11230	NW_003037949.1	+	116493	116542
Sma-1-11231	NW_003037966.1	+	113579	113628
Sma-1-11232	NW_003037966.1	-	272190	272141
Sma-1-11233	NW_003037972.1	+	725088	725137
Sma-1-11234	NW_003037973.1	+	646525	646574
Sma-1-11235	NW_003037975.1	-	607085	607036
Sma-1-11236	NW_003037976.1	-	36529	36480

Sma-1-11237	NW_003037981.1	+	8255	8304
Sma-1-11238	NW_003037996.1	+	221557	221606
Sma-1-11239	NW_003037996.1	-	114742	114693
Sma-1-11240	NW_003038001.1	+	600	649
Sma-1-11241	NW_003038003.1	+	247626	247675
Sma-1-11242	NW_003038008.1	-	211653	211604
Sma-1-11243	NW_003038011.1	-	581852	581803
Sma-1-11244	NW_003038014.1	+	197306	197355
Sma-1-11245	NW_003038019.1	+	168517	168566
Sma-1-11246	NW_003038019.1	+	412243	412292
Sma-1-11247	NW_003038019.1	-	412145	412096
Sma-1-11248	NW_003038024.1	+	39756	39805
Sma-1-11249	NW_003038028.1	+	494091	494140
Sma-1-11250	NW_003038032.1	+	293151	293200
Sma-1-11251	NW_003038032.1	+	604656	604705
Sma-1-11252	NW_003038033.1	+	124044	124093
Sma-1-11253	NW_003038033.1	+	817854	817903
Sma-1-11254	NW_003038036.1	-	161513	161464
Sma-1-11255	NW_003038037.1	+	677121	677170
Sma-1-11256	NW_003038038.1	+	1106223	1106272
Sma-1-11257	NW_003038224.1	-	208790	208741
Sma-1-11258	NW_003038229.1	+	103162	103211
Sma-1-11259	NW_003038229.1	-	103540	103491
Sma-1-11260	NW_003038259.1	+	308915	308964
Sma-1-11261	NW_003038515.1	+	398871	398920
Sma-1-11262	NW_003038518.1	-	316293	316244
Sma-1-11263	NW_003039127.1	-	200805	200756
Sma-1-11264	NW_003026474.1	+	63759	63808
Sma-1-11265	NW_003026480.1	-	22913	22864
Sma-1-11266	NW_003026505.1	+	172773	172822
Sma-1-11267	NW_003026510.1	+	88387	88436
Sma-1-11268	NW_003026515.1	-	194589	194540
Sma-1-11269	NW_003026515.1	-	149179	149130
Sma-1-11270	NW_003026574.1	+	58684	58733
Sma-1-11271	NW_003038055.1	+	163923	163972
Sma-1-11272	NW_003038068.1	+	124310	124359
Sma-1-11273	NW_003038068.1	+	124715	124764
Sma-1-11274	NW_003038075.1	-	15887	15838
Sma-1-11275	NW_003038080.1	+	343451	343500
Sma-1-11276	NW_003038083.1	-	708163	708114
Sma-1-11277	NW_003038083.1	-	444603	444554
Sma-1-11278	NW_003038084.1	-	22318	22269
Sma-1-11279	NW_003038086.1	+	366099	366148
Sma-1-11280	NW_003038086.1	-	428107	428058
Sma-1-11281	NW_003038102.1	+	67776	67825
Sma-1-11282	NW_003038109.1	-	66449	66400
Sma-1-11283	NW_003038114.1	+	141396	141445
Sma-1-11284	NW_003038115.1	-	86992	86943
Sma-1-11285	NW_003038132.1	+	400072	400121
Sma-1-11286	NW_003038134.1	-	55996	55947
Sma-1-11287	NW_003038148.1	-	80867	80818
Sma-1-11288	NW_003038153.1	+	287911	287960

Sma-1-11289	NS_000200.1	+	763399	763448
Sma-1-11290	NS_000200.1	+	3173608	3173657
Sma-1-11291	NS_000200.1	+	7394127	7394176
Sma-1-11292	NS_000200.1	+	7962836	7962885
Sma-1-11293	NS_000200.1	+	15454039	15454088
Sma-1-11294	NS_000200.1	+	21671959	21672008
Sma-1-11295	NS_000200.1	+	23968315	23968364
Sma-1-11296	NS_000200.1	+	27938235	27938284
Sma-1-11297	NS_000200.1	+	28484171	28484220
Sma-1-11298	NS_000200.1	+	33112922	33112971
Sma-1-11299	NS_000200.1	+	35853549	35853598
Sma-1-11300	NS_000200.1	+	36654853	36654902
Sma-1-11301	NS_000200.1	+	38135568	38135617
Sma-1-11302	NS_000200.1	+	42521647	42521696
Sma-1-11303	NS_000200.1	+	47452454	47452503
Sma-1-11304	NS_000200.1	+	72271520	72271569
Sma-1-11305	NS_000200.1	+	74456880	74456929
Sma-1-11306	NS_000200.1	+	81584936	81584985
Sma-1-11307	NS_000200.1	+	85444059	85444108
Sma-1-11308	NS_000200.1	+	89626948	89626997
Sma-1-11309	NS_000200.1	+	89765440	89765489
Sma-1-11310	NS_000200.1	+	90132701	90132750
Sma-1-11311	NS_000200.1	+	90881851	90881900
Sma-1-11312	NS_000200.1	+	91273536	91273585
Sma-1-11313	NS_000200.1	+	93854461	93854510
Sma-1-11314	NS_000200.1	+	95840544	95840593
Sma-1-11315	NS_000200.1	+	96508493	96508542
Sma-1-11316	NS_000200.1	+	96508899	96508948
Sma-1-11317	NS_000200.1	+	110054986	110055035
Sma-1-11318	NS_000200.1	+	111712215	111712264
Sma-1-11319	NS_000200.1	+	113022544	113022593
Sma-1-11320	NS_000200.1	+	118776190	118776239
Sma-1-11321	NS_000200.1	+	128635871	128635920
Sma-1-11322	NS_000200.1	+	144046325	144046374
Sma-1-11323	NS_000200.1	+	150757106	150757155
Sma-1-11324	NS_000200.1	+	150904804	150904853
Sma-1-11325	NS_000200.1	+	160744971	160745020
Sma-1-11326	NS_000200.1	+	161256325	161256374
Sma-1-11327	NS_000200.1	+	162980896	162980945
Sma-1-11328	NS_000200.1	+	162991414	162991463
Sma-1-11329	NS_000200.1	+	170982969	170983018
Sma-1-11330	NS_000200.1	+	182807415	182807464
Sma-1-11331	NS_000200.1	+	187795199	187795248
Sma-1-11332	NS_000200.1	+	190851134	190851183
Sma-1-11333	NS_000200.1	+	192378540	192378589
Sma-1-11334	NS_000200.1	+	198815442	198815491
Sma-1-11335	NS_000200.1	+	203200792	203200841
Sma-1-11336	NS_000200.1	+	204628252	204628301
Sma-1-11337	NS_000200.1	+	212406123	212406172
Sma-1-11338	NS_000200.1	+	219808482	219808531
Sma-1-11339	NS_000200.1	+	219808887	219808936
Sma-1-11340	NS_000200.1	+	220466433	220466482

Sma-1-11341	NS_000200.1	+	222710161	222710210
Sma-1-11342	NS_000200.1	+	223746908	223746957
Sma-1-11343	NS_000200.1	+	229937995	229938044
Sma-1-11344	NS_000200.1	+	233485335	233485384
Sma-1-11345	NS_000200.1	+	233729061	233729110
Sma-1-11346	NS_000200.1	+	237936785	237936834
Sma-1-11347	NS_000200.1	+	241801665	241801714
Sma-1-11348	NS_000200.1	+	245452311	245452360
Sma-1-11349	NS_000200.1	+	245763816	245763865
Sma-1-11350	NS_000200.1	+	246104046	246104095
Sma-1-11351	NS_000200.1	+	246797856	246797905
Sma-1-11352	NS_000200.1	+	250832530	250832579
Sma-1-11353	NS_000200.1	+	253009616	253009665
Sma-1-11354	NS_000200.1	+	253262334	253262383
Sma-1-11355	NS_000200.1	+	258863245	258863294
Sma-1-11356	NS_000200.1	+	263191518	263191567
Sma-1-11357	NS_000200.1	+	263191923	263191972
Sma-1-11358	NS_000200.1	+	268796061	268796110
Sma-1-11359	NS_000200.1	+	272614692	272614741
Sma-1-11360	NS_000200.1	+	274746722	274746771
Sma-1-11361	NS_000200.1	+	276867301	276867350
Sma-1-11362	NS_000200.1	+	279425895	279425944
Sma-1-11363	NS_000200.1	+	282820542	282820591
Sma-1-11364	NS_000200.1	+	283528568	283528617
Sma-1-11365	NS_000200.1	+	293349960	293350009
Sma-1-11366	NS_000200.1	+	301866017	301866066
Sma-1-11367	NS_000200.1	+	307515316	307515365
Sma-1-11368	NS_000200.1	+	310748300	310748349
Sma-1-11369	NS_000200.1	+	311020724	311020773
Sma-1-11370	NS_000200.1	+	311220384	311220433
Sma-1-11371	NS_000200.1	+	317924279	317924328
Sma-1-11372	NS_000200.1	+	317924684	317924733
Sma-1-11373	NS_000200.1	+	322137897	322137946
Sma-1-11374	NS_000200.1	+	323169514	323169563
Sma-1-11375	NS_000200.1	+	325413227	325413276
Sma-1-11376	NS_000200.1	+	325421335	325421384
Sma-1-11377	NS_000200.1	+	326191277	326191326
Sma-1-11378	NS_000200.1	+	326506952	326507001
Sma-1-11379	NS_000200.1	+	326507357	326507406
Sma-1-11380	NS_000200.1	+	326507762	326507811
Sma-1-11381	NS_000200.1	+	328039131	328039180
Sma-1-11382	NS_000200.1	+	329520324	329520373
Sma-1-11383	NS_000200.1	+	329520729	329520778
Sma-1-11384	NS_000200.1	+	335241273	335241322
Sma-1-11385	NS_000200.1	+	339589099	339589148
Sma-1-11386	NS_000200.1	+	340482936	340482985
Sma-1-11387	NS_000200.1	+	343053940	343053989
Sma-1-11388	NS_000200.1	+	344365749	344365798
Sma-1-11389	NS_000200.1	+	344578535	344578584
Sma-1-11390	NS_000200.1	+	352642129	352642178
Sma-1-11391	NS_000200.1	+	353520528	353520577
Sma-1-11392	NS_000200.1	+	353520933	353520982

Sma-1-11393	NS_000200.1	+	353521338	353521387
Sma-1-11394	NS_000200.1	+	355096026	355096075
Sma-1-11395	NS_000200.1	+	355096431	355096480
Sma-1-11396	NS_000200.1	+	358812458	358812507
Sma-1-11397	NS_000200.1	+	363074177	363074226
Sma-1-11398	NS_000200.1	+	363369143	363369192
Sma-1-11399	NS_000200.1	+	368058783	368058832
Sma-1-11400	NS_000200.1	+	369846043	369846092
Sma-1-11401	NS_000200.1	+	373387858	373387907
Sma-1-11402	NS_000200.1	+	373428971	373429020
Sma-1-11403	NS_000200.1	+	378984429	378984478
Sma-1-11404	NS_000200.1	+	381775881	381775930
Sma-1-11405	NS_000200.1	-	378982646	378982597
Sma-1-11406	NS_000200.1	-	378496693	378496644
Sma-1-11407	NS_000200.1	-	375952974	375952925
Sma-1-11408	NS_000200.1	-	372540838	372540789
Sma-1-11409	NS_000200.1	-	370944073	370944024
Sma-1-11410	NS_000200.1	-	365453139	365453090
Sma-1-11411	NS_000200.1	-	364011453	364011404
Sma-1-11412	NS_000200.1	-	362187942	362187893
Sma-1-11413	NS_000200.1	-	360643261	360643212
Sma-1-11414	NS_000200.1	-	355166310	355166261
Sma-1-11415	NS_000200.1	-	354797173	354797124
Sma-1-11416	NS_000200.1	-	354796768	354796719
Sma-1-11417	NS_000200.1	-	354796363	354796314
Sma-1-11418	NS_000200.1	-	354795958	354795909
Sma-1-11419	NS_000200.1	-	352006418	352006369
Sma-1-11420	NS_000200.1	-	352006013	352005964
Sma-1-11421	NS_000200.1	-	352005608	352005559
Sma-1-11422	NS_000200.1	-	351544817	351544768
Sma-1-11423	NS_000200.1	-	351544412	351544363
Sma-1-11424	NS_000200.1	-	351544007	351543958
Sma-1-11425	NS_000200.1	-	351543602	351543553
Sma-1-11426	NS_000200.1	-	351373435	351373386
Sma-1-11427	NS_000200.1	-	351373030	351372981
Sma-1-11428	NS_000200.1	-	349975750	349975701
Sma-1-11429	NS_000200.1	-	349975345	349975296
Sma-1-11430	NS_000200.1	-	349974940	349974891
Sma-1-11431	NS_000200.1	-	349264589	349264540
Sma-1-11432	NS_000200.1	-	348133535	348133486
Sma-1-11433	NS_000200.1	-	348133130	348133081
Sma-1-11434	NS_000200.1	-	347984961	347984912
Sma-1-11435	NS_000200.1	-	344557695	344557646
Sma-1-11436	NS_000200.1	-	344036011	344035962
Sma-1-11437	NS_000200.1	-	341953126	341953077
Sma-1-11438	NS_000200.1	-	339708132	339708083
Sma-1-11439	NS_000200.1	-	337491076	337491027
Sma-1-11440	NS_000200.1	-	333180062	333180013
Sma-1-11441	NS_000200.1	-	319388535	319388486
Sma-1-11442	NS_000200.1	-	318317112	318317063
Sma-1-11443	NS_000200.1	-	315904963	315904914
Sma-1-11444	NS_000200.1	-	311220762	311220713

Sma-1-11445	NS_000200.1	-	309620518	309620469
Sma-1-11446	NS_000200.1	-	290839000	290838951
Sma-1-11447	NS_000200.1	-	290793590	290793541
Sma-1-11448	NS_000200.1	-	290154851	290154802
Sma-1-11449	NS_000200.1	-	283247113	283247064
Sma-1-11450	NS_000200.1	-	277142287	277142238
Sma-1-11451	NS_000200.1	-	275749127	275749078
Sma-1-11452	NS_000200.1	-	272676700	272676651
Sma-1-11453	NS_000200.1	-	271037562	271037513
Sma-1-11454	NS_000200.1	-	270951500	270951451
Sma-1-11455	NS_000200.1	-	270687940	270687891
Sma-1-11456	NS_000200.1	-	266134098	266134049
Sma-1-11457	NS_000200.1	-	264569173	264569124
Sma-1-11458	NS_000200.1	-	254908470	254908421
Sma-1-11459	NS_000200.1	-	249095365	249095316
Sma-1-11460	NS_000200.1	-	233728963	233728914
Sma-1-11461	NS_000200.1	-	228109502	228109453
Sma-1-11462	NS_000200.1	-	225884749	225884700
Sma-1-11463	NS_000200.1	-	220359618	220359569
Sma-1-11464	NS_000200.1	-	208091156	208091107
Sma-1-11465	NS_000200.1	-	207950972	207950923
Sma-1-11466	NS_000200.1	-	206650844	206650795
Sma-1-11467	NS_000200.1	-	206189309	206189260
Sma-1-11468	NS_000200.1	-	198974053	198974004
Sma-1-11469	NS_000200.1	-	196395061	196395012
Sma-1-11470	NS_000200.1	-	190128545	190128496
Sma-1-11471	NS_000200.1	-	188940760	188940711
Sma-1-11472	NS_000200.1	-	187737374	187737325
Sma-1-11473	NS_000200.1	-	182967641	182967592
Sma-1-11474	NS_000200.1	-	168628240	168628191
Sma-1-11475	NS_000200.1	-	168123656	168123607
Sma-1-11476	NS_000200.1	-	168040031	168039982
Sma-1-11477	NS_000200.1	-	166700357	166700308
Sma-1-11478	NS_000200.1	-	161255218	161255169
Sma-1-11479	NS_000200.1	-	159816049	159816000
Sma-1-11480	NS_000200.1	-	157754306	157754257
Sma-1-11481	NS_000200.1	-	156652895	156652846
Sma-1-11482	NS_000200.1	-	154168481	154168432
Sma-1-11483	NS_000200.1	-	150956723	150956674
Sma-1-11484	NS_000200.1	-	149662840	149662791
Sma-1-11485	NS_000200.1	-	145016360	145016311
Sma-1-11486	NS_000200.1	-	142995850	142995801
Sma-1-11487	NS_000200.1	-	128871132	128871083
Sma-1-11488	NS_000200.1	-	127936331	127936282
Sma-1-11489	NS_000200.1	-	122101904	122101855
Sma-1-11490	NS_000200.1	-	119056516	119056467
Sma-1-11491	NS_000200.1	-	113278304	113278255
Sma-1-11492	NS_000200.1	-	112277965	112277916
Sma-1-11493	NS_000200.1	-	111711941	111711892
Sma-1-11494	NS_000200.1	-	111564873	111564824
Sma-1-11495	NS_000200.1	-	108980680	108980631
Sma-1-11496	NS_000200.1	-	101136136	101136087

Sma-1-11497	NS_000200.1	-	101075971	101075922
Sma-1-11498	NS_000200.1	-	96826028	96825979
Sma-1-11499	NS_000200.1	-	93853815	93853766
Sma-1-11500	NS_000200.1	-	91788352	91788303
Sma-1-11501	NS_000200.1	-	85214205	85214156
Sma-1-11502	NS_000200.1	-	83181705	83181656
Sma-1-11503	NS_000200.1	-	78479479	78479430
Sma-1-11504	NS_000200.1	-	76919931	76919882
Sma-1-11505	NS_000200.1	-	75561510	75561461
Sma-1-11506	NS_000200.1	-	68067251	68067202
Sma-1-11507	NS_000200.1	-	60161143	60161094
Sma-1-11508	NS_000200.1	-	58005739	58005690
Sma-1-11509	NS_000200.1	-	52989172	52989123
Sma-1-11510	NS_000200.1	-	40111071	40111022
Sma-1-11511	NS_000200.1	-	36421584	36421535
Sma-1-11512	NS_000200.1	-	34914023	34913974
Sma-1-11513	NS_000200.1	-	32354777	32354728
Sma-1-11514	NS_000200.1	-	28993137	28993088
Sma-1-11515	NS_000200.1	-	21563580	21563531
Sma-1-11516	NS_000200.1	-	18240438	18240389
Sma-1-11517	NS_000200.1	-	18037092	18037043
Sma-1-11518	NS_000200.1	-	17848275	17848226
Sma-1-11519	NS_000200.1	-	17168562	17168513
Sma-1-11520	NS_000200.1	-	16883266	16883217
Sma-1-11521	NS_000200.1	-	10801506	10801457
Sma-1-11522	NS_000200.1	-	10055035	10054986
Sma-1-11523	NS_000200.1	-	6044483	6044434
Sma-1-11524	NS_000200.1	-	4124224	4124175
Sma-1-11525	NS_000200.1	-	218360	218311
Tcr-1-3	NZ_AAHK01022669.1	+	344	393
Tcr-1-4	NZ_AAHK01022669.1	+	749	798
Sma-1-11526	NW_003035894.1	-	160	111
Sma-1-11527	NS_000200.1	-	342932262	342932213
Sma-1-11528	NW_003037859.1	+	854165	854214
Sma-1-11529	NS_000200.1	+	103533006	103533055
Sma-1-11530	NW_003037865.1	+	544369	544418
Sma-1-11531	NS_000200.1	+	110966288	110966337
Sma-1-11532	NW_003037844.1	-	774063	774014
Sma-1-11533	NS_000200.1	-	86957509	86957460
Sma-1-11534	NW_003037835.1	+	1001116	1001165
Sma-1-11535	NS_000200.1	+	75397624	75397673
Sma-1-11536	NW_003037833.1	-	638325	638276
Sma-1-11537	NS_000200.1	-	72829421	72829372
Sma-1-11538	NW_003037904.1	-	108842	108793
Sma-1-11539	NS_000200.1	-	148823341	148823292
Sma-1-11540	NW_003037832.1	-	684747	684698
Sma-1-11541	NS_000200.1	-	71894178	71894129
Sma-1-11542	NW_003037796.1	+	1675092	1675141
Sma-1-11543	NS_000200.1	+	9884736	9884785
Sma-1-11544	NW_003037808.1	-	1325901	1325852
Sma-1-11545	NS_000200.1	-	33112538	33112489
Sma-1-11546	NW_003037825.1	+	1393987	1394036

Sma-1-11547	NS_000200.1	+	60523769	60523818
Sma-1-11548	NW_003037920.1	-	282995	282946
Sma-1-11549	NS_000200.1	-	169463802	169463753
Sma-1-11550	NW_003037923.1	-	294674	294625
Sma-1-11551	NS_000200.1	-	171350887	171350838
Sma-1-11552	NW_003026472.1	-	71070	71021
Sma-1-11553	NS_000200.1	-	236483062	236483013
Sma-1-11554	NW_003038261.1	-	192091	192042
Sma-1-11555	NS_000200.1	-	323805742	323805693
Sma-1-11556	NW_003038258.1	-	165109	165060
Sma-1-11557	NS_000200.1	-	322703619	322703570
Sma-1-11558	NW_003037797.1	+	1691515	1691564
Sma-1-11559	NW_003037809.1	-	858507	858458
Sma-1-11560	NW_003037910.1	+	957995	958044
Sma-1-11561	NW_003038072.1	+	86335	86384
Sma-1-11562	NS_000200.1	+	12119375	12119424
Sma-1-11563	NS_000200.1	+	155469970	155470019
Sma-1-11564	NS_000200.1	+	265248563	265248612
Sma-1-11565	NS_000200.1	-	34522971	34522922
Sma-1-11566	NW_003037971.1	-	823047	822998
Sma-1-11567	NS_000200.1	-	202445097	202445048
Sma-1-11568	NW_003026517.1	-	110929	110880
Sma-1-11569	NS_000200.1	-	299716938	299716889
Sma-1-11570	NW_003038260.1	-	285470	285421
Sma-1-11571	NS_000200.1	-	323457245	323457196
Sma-1-11572	NW_003026469.1	-	157321	157272
Sma-1-11573	NS_000200.1	-	217778980	217778931
Sma-1-11574	NW_003038023.1	+	239816	239865
Sma-1-11575	NW_003038261.1	-	909082	909033
Sma-1-11576	NS_000200.1	+	237400107	237400156
Sma-1-11577	NS_000200.1	-	324522733	324522684
Sma-1-11578	NW_003026606.1	-	93180	93131
Sma-1-11579	NS_000200.1	-	307948873	307948824
Sma-1-11580	NW_003038512.1	-	135670	135621
Sma-1-11581	NW_003037819.1	-	340192	340143
Sma-1-11582	NW_003037912.1	-	513409	513360
Sma-1-11583	NS_000200.1	-	338692367	338692318
Sma-1-11584	NS_000200.1	-	157324984	157324935
Sma-1-11585	NS_000200.1	-	51428457	51428408
Sma-1-11586	NW_003027511.1	+	345	394
Sma-1-11587	NS_000200.1	+	356980198	356980247
Sma-1-11588	NW_003038145.1	-	109619	109570
Sma-1-11589	NS_000200.1	-	288721042	288720993
Sma-1-11590	NW_003037797.1	-	3302726	3302677
Sma-1-11591	NW_003037813.1	+	1979912	1979961
Sma-1-11592	NW_003026574.1	+	54191	54240
Sma-1-11593	NS_000200.1	+	42966775	42966824
Sma-1-11594	NS_000200.1	+	301861524	301861573
Sma-1-11595	NS_000200.1	-	13730586	13730537
Sma-1-11596	NW_003026465.1	+	392556	392605
Sma-1-11597	NW_003037997.1	-	74566	74517
Sma-1-11598	NS_000200.1	+	207785150	207785199

Sma-1-11599	NS_000200.1	-	220886008	220885959
Sma-1-11600	NW_003038008.1	+	93559	93608
Sma-1-11601	NS_000200.1	+	225766655	225766704
Sma-1-11602	NW_003037849.1	-	951499	951450
Sma-1-11603	NW_003037896.1	+	538809	538858
Sma-1-11604	NW_003037916.1	+	405500	405549
Sma-1-11605	NW_003037943.1	+	529452	529501
Sma-1-11606	NW_003037943.1	+	530270	530319
Sma-1-11607	NS_000200.1	+	143459232	143459281
Sma-1-11608	NS_000200.1	+	163598911	163598960
Sma-1-11609	NS_000200.1	+	187550985	187551034
Sma-1-11610	NS_000200.1	+	187551803	187551852
Sma-1-11611	NS_000200.1	-	91927772	91927723
Sma-1-11612	NW_003038256.1	+	170282	170331
Sma-1-11613	NW_003038242.1	-	151719	151670
Sma-1-11614	NW_003037815.1	+	347794	347843
Sma-1-11615	NW_003037829.1	-	955251	955202
Sma-1-11616	NW_003037945.1	+	551540	551589
Sma-1-11617	NW_003037980.1	+	976673	976722
Sma-1-11618	NW_003037980.1	-	100900	100851
Sma-1-11619	NW_003038011.1	+	694065	694114
Sma-1-11620	NW_003038037.1	-	217215	217166
Sma-1-11621	NS_000200.1	+	45610785	45610834
Sma-1-11622	NS_000200.1	+	189653504	189653553
Sma-1-11623	NS_000200.1	+	211937535	211937584
Sma-1-11624	NS_000200.1	+	228221715	228221764
Sma-1-11625	NS_000200.1	+	320904108	320904157
Sma-1-11626	NS_000200.1	-	314850865	314850816
Sma-1-11627	NS_000200.1	-	250372624	250372575
Sma-1-11628	NS_000200.1	-	211061762	211061713
Sma-1-11629	NS_000200.1	-	66855789	66855740
Sma-1-11630	NW_003038258.1	-	12547	12498
Sma-1-11631	NS_000200.1	-	322551057	322551008
Sma-1-11632	NW_003026619.1	-	141223	141174
Sma-1-11633	NS_000200.1	-	322331967	322331918
Sma-1-11634	NW_003021096.1	-	543	494
Sma-1-11635	NS_000200.1	-	367927297	367927248
Sma-1-11636	NW_003037977.1	+	520828	520877
Sma-1-11637	NS_000200.1	+	208806848	208806897
Sma-1-11638	NW_003034337.1	-	738	689
Sma-1-11639	NW_003037811.1	-	236920	236871
Sma-1-11640	NW_003038064.1	-	169949	169900
Sma-1-11641	NS_000200.1	-	346558020	346557971
Sma-1-11642	NS_000200.1	-	262006526	262006477
Sma-1-11643	NS_000200.1	-	38407162	38407113
Sma-1-11644	NW_003037920.1	+	198293	198342
Sma-1-11645	NS_000200.1	+	169379100	169379149
Sma-1-11646	NW_003037944.1	-	234725	234676
Sma-1-11647	NS_000200.1	-	188456078	188456029
Sma-1-11648	NW_003038008.1	+	24172	24221
Sma-1-11649	NS_000200.1	+	225697268	225697317
Sma-1-11650	NW_003038016.1	+	529832	529881

Sma-1-11651	NS_000200.1	+	231646369	231646418
Sma-1-11652	NW_003039127.1	-	1927613	1927564
Sma-1-11653	NS_000200.1	-	115005112	115005063
Sma-1-11654	NW_003037539.1	+	273	322
Sma-1-11655	NS_000200.1	+	371306395	371306444
Sma-1-11656	NW_003037797.1	-	3675490	3675441
Sma-1-11657	NS_000200.1	-	14103350	14103301
Sma-1-11658	NW_003037879.1	+	560671	560720
Sma-1-11659	NS_000200.1	+	123562927	123562976
Sma-1-11660	NW_003037880.1	-	715330	715281
Sma-1-11661	NW_003037880.1	-	705690	705641
Sma-1-11662	NS_000200.1	-	124398888	124398839
Sma-1-11663	NS_000200.1	-	124389248	124389199
Sma-1-11664	NW_003037881.1	+	738671	738720
Sma-1-11665	NS_000200.1	+	125170462	125170511
Sma-1-11666	NW_003037881.1	-	642915	642866
Sma-1-11667	NW_003038035.1	-	316628	316579
Sma-1-11668	NS_000200.1	-	248178120	248178071
Sma-1-11669	NS_000200.1	-	125074706	125074657
Sma-1-11670	NW_003037880.1	-	459501	459452
Sma-1-11671	NS_000200.1	-	124143059	124143010
Sma-1-11672	NW_003037879.1	-	278817	278768
Sma-1-11673	NS_000200.1	-	123281073	123281024
Sma-1-11674	NW_003037806.1	-	49650	49601
Sma-1-11675	NS_000200.1	-	28244864	28244815
Sma-1-11676	NW_003037838.1	+	86949	86998
Sma-1-11677	NS_000200.1	+	78085512	78085561
Sma-1-11678	NW_003037882.1	+	583425	583474
Sma-1-11679	NS_000200.1	+	125875099	125875148
Sma-1-11680	NW_003037878.1	+	508063	508112
Sma-1-11681	NW_003038141.1	+	246601	246650
Sma-1-11682	NS_000200.1	+	122561541	122561590
Sma-1-11683	NS_000200.1	+	287136580	287136629
Sma-1-11684	NW_003037878.1	-	470103	470054
Sma-1-11685	NS_000200.1	-	122523581	122523532
Sma-1-11686	NW_003037877.1	-	796530	796481
Sma-1-11687	NS_000200.1	-	121835092	121835043
Sma-1-11688	NW_003037883.1	+	462669	462718
Sma-1-11689	NS_000200.1	+	126526392	126526441
Sma-1-11690	NW_003037876.1	+	489921	489970
Sma-1-11691	NW_003037802.1	-	305151	305102
Sma-1-11692	NW_003037812.1	+	1391199	1391248
Sma-1-11693	NW_003037821.1	-	859767	859718
Sma-1-11694	NW_003037847.1	+	995156	995205
Sma-1-11695	NW_003037882.1	-	325087	325038
Sma-1-11696	NW_003037918.1	+	2370	2419
Sma-1-11697	NW_003026575.1	-	71061	71012
Sma-1-11698	NS_000200.1	+	40873938	40873987
Sma-1-11699	NS_000200.1	+	89646886	89646935
Sma-1-11700	NS_000200.1	+	121035557	121035606
Sma-1-11701	NS_000200.1	+	166569360	166569409
Sma-1-11702	NS_000200.1	-	302302727	302302678

Sma-1-11703	NS_000200.1	-	125616761	125616712
Sma-1-11704	NS_000200.1	-	54004251	54004202
Sma-1-11705	NS_000200.1	-	22307945	22307896
Sma-1-11706	NW_003037875.1	+	450334	450383
Sma-1-11707	NS_000200.1	+	120479003	120479052
Sma-1-11708	NW_003037871.1	+	352077	352126
Sma-1-11709	NS_000200.1	+	117799815	117799864
Sma-1-11710	NW_003037870.1	+	276656	276705
Sma-1-11711	NS_000200.1	+	117150321	117150370
Sma-1-11712	NW_003026472.1	-	535430	535381
Sma-1-11713	NW_003037803.1	-	555281	555232
Sma-1-11714	NW_003037806.1	-	612108	612059
Sma-1-11715	NW_003037853.1	-	475881	475832
Sma-1-11716	NW_003037924.1	-	288393	288344
Sma-1-11717	NW_003038022.1	-	165675	165626
Sma-1-11718	NW_003038230.1	+	46512	46561
Sma-1-11719	NW_003038128.1	+	310599	310648
Sma-1-11720	NS_000200.1	+	281028887	281028936
Sma-1-11721	NS_000200.1	+	311332083	311332132
Sma-1-11722	NS_000200.1	-	236947422	236947373
Sma-1-11723	NS_000200.1	-	235846517	235846468
Sma-1-11724	NS_000200.1	-	171995351	171995302
Sma-1-11725	NS_000200.1	-	95967932	95967883
Sma-1-11726	NS_000200.1	-	28807322	28807273
Sma-1-11727	NS_000200.1	-	24055818	24055769
Sma-1-11728	NW_003028446.1	-	1270	1221
Sma-1-11729	NW_003038057.1	+	288257	288306
Sma-1-11730	NS_000200.1	+	259494073	259494122
Sma-1-11731	NS_000200.1	-	369657318	369657269
Sma-1-11732	NW_003037865.1	+	806342	806391
Sma-1-11733	NS_000200.1	+	111228261	111228310
Sma-1-11734	NW_003037824.1	+	957167	957216
Sma-1-11735	NW_003037866.1	+	1502652	1502701
Sma-1-11736	NS_000200.1	+	57888107	57888156
Sma-1-11737	NS_000200.1	+	112990451	112990500
Sma-1-11738	NW_003037824.1	+	970246	970295
Sma-1-11739	NS_000200.1	+	57901186	57901235
Sma-1-11740	NW_003037823.1	+	46225	46274
Sma-1-11741	NS_000200.1	+	55962844	55962893
Sma-1-11742	NW_003037803.1	+	1356705	1356754
Sma-1-11743	NS_000200.1	+	24857242	24857291
Sma-1-11744	NW_003037971.1	+	401589	401638
Sma-1-11745	NS_000200.1	+	202023639	202023688
Sma-1-11746	NW_003021869.1	-	372	323
Sma-1-11747	NW_003037977.1	-	302394	302345
Sma-1-11748	NW_003038059.1	+	215535	215584
Sma-1-11749	NS_000200.1	+	260457607	260457656
Sma-1-11750	NS_000200.1	-	352002574	352002525
Sma-1-11751	NS_000200.1	-	208588414	208588365
Sma-1-11752	NW_003038025.1	-	217217	217168
Sma-1-11753	NS_000200.1	-	239075462	239075413
Sma-1-11754	NW_003038070.1	+	149977	150026

Sma-1-11755	NS_000200.1	+	264487218	264487267
Sma-1-11756	NW_003038024.1	-	277005	276956
Sma-1-11757	NS_000200.1	-	238174034	238173985
Sma-1-11758	NW_003037833.1	+	96674	96723
Sma-1-11759	NS_000200.1	+	72287770	72287819
Sma-1-11760	NW_003027449.1	-	1873	1824
Sma-1-11761	NW_003037846.1	-	657873	657824
Sma-1-11762	NS_000200.1	-	356881049	356881000
Sma-1-11763	NS_000200.1	-	88292443	88292394
Sma-1-11764	NW_003035881.1	-	331892	331843
Sma-1-11765	NW_003037859.1	+	925213	925262
Sma-1-11766	NS_000200.1	+	103604054	103604103
Sma-1-11767	NS_000200.1	-	341396097	341396048
Sma-1-11768	NW_003029528.1	-	301	252
Sma-1-11769	NW_003026594.1	-	27213	27164
Sma-1-11770	NS_000200.1	-	362218181	362218132
Sma-1-11771	NS_000200.1	-	304971583	304971534
Sma-1-11772	NW_003037863.1	+	842618	842667
Sma-1-11773	NS_000200.1	+	107600985	107601034
Sma-1-11774	NW_003037863.1	-	1708637	1708588
Sma-1-11775	NS_000200.1	-	108467004	108466955
Sma-1-11776	NW_003037862.1	-	83532	83483
Sma-1-11777	NS_000200.1	-	105735193	105735144
Sma-1-11778	NW_003037887.1	+	360942	360991
Sma-1-11779	NW_003037916.1	+	1351308	1351357
Sma-1-11780	NS_000200.1	+	130824920	130824969
Sma-1-11781	NS_000200.1	+	164544719	164544768
Sma-1-11782	NW_003037888.1	-	1481901	1481852
Sma-1-11783	NS_000200.1	-	133690393	133690344
Sma-1-11784	NW_003037888.1	+	799722	799771
Sma-1-11785	NS_000200.1	+	133008214	133008263
Sma-1-11786	NW_003038225.1	-	100621	100572
Sma-1-11787	NS_000200.1	-	309955285	309955236
Sma-1-11788	NW_003037800.1	+	1520945	1520994
Sma-1-11789	NS_000200.1	+	20150305	20150354
Sma-1-11790	NW_003037908.1	+	916161	916210
Sma-1-11791	NS_000200.1	+	153389292	153389341
Sma-1-11792	NW_003037908.1	-	964591	964542
Sma-1-11793	NS_000200.1	-	153437722	153437673
Sma-1-11794	NW_003037814.1	-	268229	268180
Sma-1-11795	NS_000200.1	-	43252089	43252040
Sma-1-11796	NW_003038010.1	-	554924	554875
Sma-1-11797	NS_000200.1	-	227373147	227373098
Sma-1-11798	NW_003037830.1	-	1508506	1508457
Sma-1-11799	NS_000200.1	-	69398877	69398828
Sma-1-11800	NW_003038023.1	+	377311	377360
Sma-1-11801	NS_000200.1	+	237537602	237537651
Sma-1-11802	NW_003038036.1	+	553743	553792
Sma-1-11803	NS_000200.1	+	249487595	249487644
Sma-1-11804	NW_003037912.1	+	1335685	1335734
Sma-1-11805	NS_000200.1	+	158147260	158147309
Sma-1-11806	NW_003037856.1	+	419069	419118

Sma-1-11807	NS_000200.1	+	99777025	99777074
Sma-1-11808	NW_003037854.1	+	700077	700126
Sma-1-11809	NS_000200.1	+	97604498	97604547
Sma-1-11810	NW_003038033.1	+	382431	382480
Sma-1-11811	NS_000200.1	+	246362433	246362482
Sma-1-11812	NW_003020317.1	-	292	243
Sma-1-11813	NW_003037915.1	-	173461	173412
Sma-1-11814	NS_000200.1	-	368991032	368990983
Sma-1-11815	NS_000200.1	-	162073447	162073398
Sma-1-11816	NW_003038150.1	-	45277	45228
Sma-1-11817	NS_000200.1	-	291524015	291523966
Sma-1-11818	NW_003038080.1	-	130397	130348
Sma-1-11819	NS_000200.1	-	268583007	268582958
Sma-1-11820	NW_003038023.1	+	652766	652815
Sma-1-11821	NS_000200.1	+	237813057	237813106
Sma-1-11822	NW_003037914.1	+	475263	475312
Sma-1-11823	NS_000200.1	+	160619513	160619562
Sma-1-11824	NW_003037889.1	-	350915	350866
Sma-1-11825	NS_000200.1	-	134127405	134127356
Sma-1-11826	NW_003037841.1	-	2453245	2453196
Sma-1-11827	NS_000200.1	-	85064426	85064377
Sma-1-11828	NW_003037854.1	+	1910085	1910134
Sma-1-11829	NS_000200.1	+	98814506	98814555
Sma-1-11830	NW_003037799.1	+	1038296	1038345
Sma-1-11831	NS_000200.1	+	18028650	18028699
Sma-1-11832	NW_003037819.1	+	135886	135935
Sma-1-11833	NS_000200.1	+	51224151	51224200
Sma-1-11834	NW_003037853.1	-	520095	520046
Sma-1-11835	NS_000200.1	-	96012146	96012097
Sma-1-11836	NW_003037853.1	+	252258	252307
Sma-1-11837	NS_000200.1	+	95744309	95744358
Sma-1-11838	NW_003037851.1	-	89716	89667
Sma-1-11839	NS_000200.1	-	93493026	93492977
Sma-1-11840	NW_003037901.1	-	203670	203621
Sma-1-11841	NS_000200.1	-	146052131	146052082
Sma-1-11842	NW_003037901.1	-	263272	263223
Sma-1-11843	NS_000200.1	-	146111733	146111684
Sma-1-11844	NW_003037907.1	-	604943	604894
Sma-1-11845	NS_000200.1	-	152009432	152009383
Sma-1-11846	NW_003037817.1	+	866885	866934
Sma-1-11847	NW_003038023.1	+	632720	632769
Sma-1-11848	NS_000200.1	+	48249237	48249286
Sma-1-11849	NS_000200.1	+	237793011	237793060
Sma-1-11850	NW_003036289.1	+	427	476
Sma-1-11851	NS_000200.1	+	359572499	359572548
Sma-1-11852	NW_003037813.1	+	1095767	1095816
Sma-1-11853	NS_000200.1	+	42082630	42082679
Sma-1-11854	NW_003037924.1	-	352998	352946
Sma-1-11855	NS_000200.1	-	172059956	172059904
Sma-1-11856	NW_003037831.1	+	1533311	1533360
Sma-1-11857	NW_003037831.1	-	1533150	1533101
Sma-1-11858	NS_000200.1	+	70982613	70982662

Sma-1-11859	NS_000200.1	-	70982452	70982403
Sma-1-11860	NW_003038002.1	-	65213	65164
Sma-1-11861	NW_003037845.1	+	313252	313301
Sma-1-11862	NS_000200.1	+	87315155	87315204
Sma-1-11863	NS_000200.1	-	223185422	223185373
Sma-1-11864	NW_003026464.1	-	439131	439082
Sma-1-11865	NS_000200.1	-	203739440	203739391
Sma-1-11866	NW_003037808.1	-	242096	242047
Sma-1-11867	NS_000200.1	-	32028733	32028684
Sma-1-11868	NW_003037809.1	+	682675	682724
Sma-1-11869	NS_000200.1	+	34347139	34347188
Sma-1-11870	NW_003038077.1	+	186472	186521
Sma-1-11871	NS_000200.1	+	266958112	266958161
Sma-1-11872	NW_003028484.1	-	671	622
Sma-1-11873	NW_003028484.1	-	267	218
Sma-1-11874	NW_003037809.1	-	1980097	1980048
Sma-1-11875	NS_000200.1	-	369742458	369742409
Sma-1-11876	NS_000200.1	-	369742054	369742005
Sma-1-11877	NS_000200.1	-	35644561	35644512
Sma-1-11878	NW_003038139.1	-	194575	194526
Sma-1-11879	NS_000200.1	-	286234705	286234656
Sma-1-11880	NW_003026199.1	+	1204	1253
Sma-1-11881	NW_003039129.1	-	81311	81262
Sma-1-11882	NW_003026460.1	+	511816	511865
Sma-1-11883	NW_003037796.1	+	760616	760665
Sma-1-11884	NW_003037802.1	-	1002761	1002712
Sma-1-11885	NW_003038245.1	-	1647	1598
Sma-1-11886	NS_000200.1	+	8970260	8970309
Sma-1-11887	NS_000200.1	+	171034022	171034071
Sma-1-11888	NS_000200.1	+	349423473	349423522
Sma-1-11889	NS_000200.1	-	315590437	315590388
Sma-1-11890	NS_000200.1	-	229036119	229036070
Sma-1-11891	NS_000200.1	-	23005555	23005506
Sma-1-11892	NW_003026456.1	+	457031	457080
Sma-1-11893	NS_000200.1	+	58380167	58380216
Sma-1-11894	NW_003037809.1	-	2297291	2297242
Sma-1-11895	NS_000200.1	-	35961755	35961706
Sma-1-11896	NW_003037918.1	-	521592	521543
Sma-1-11897	NS_000200.1	-	167088582	167088533
Sma-1-11898	NW_003037791.1	-	251784	251735
Sma-1-11899	NS_000200.1	-	381957212	381957163
Sma-1-11900	NW_003037942.1	+	160451	160500
Sma-1-11901	NS_000200.1	+	185920359	185920408
Sma-1-11902	NW_003037982.1	+	198874	198923
Sma-1-11903	NS_000200.1	+	213858069	213858118
Sma-1-11904	NW_003037819.1	+	340481	340530
Sma-1-11905	NW_003037853.1	-	957039	956990
Sma-1-11906	NW_003037926.1	-	231660	231611
Sma-1-11907	NS_000200.1	+	51428746	51428795
Sma-1-11908	NS_000200.1	-	173399930	173399881
Sma-1-11909	NS_000200.1	-	96449090	96449041
env-86	AB231864.1	-	50263	50214

Sma-1-11910	NW_003033999.1	+	1310	1359
Sma-1-11911	NS_000200.1	+	363395644	363395693
Sma-1-11912	NW_003037939.1	+	272005	272054
Sma-1-11913	NW_003037982.1	-	1214537	1214488
Sma-1-11914	NS_000200.1	+	183141759	183141808
Sma-1-11915	NS_000200.1	-	214873732	214873683
Sma-1-11916	NW_003037980.1	-	204236	204187
Sma-1-11917	NS_000200.1	-	211165098	211165049
Sma-1-11918	NW_003038131.1	+	314668	314717
Sma-1-11919	NS_000200.1	+	282276512	282276561
Sma-1-11920	NW_003037846.1	-	447544	447495
Sma-1-11921	NS_000200.1	-	88082114	88082065
Sma-1-11922	NW_003038258.1	-	139765	139716
Sma-1-11923	NS_000200.1	-	322678275	322678226
Sma-1-11924	NW_003026516.1	-	166342	166293
Sma-1-11925	NS_000200.1	-	299274639	299274590
Sma-1-11926	NW_003037800.1	+	2054320	2054369
Sma-1-11927	NS_000200.1	+	20683680	20683729
Sma-1-11928	NW_003037967.1	+	185196	185245
Sma-1-11929	NW_003038020.1	-	265711	265662
Sma-1-11930	NS_000200.1	+	199425167	199425216
Sma-1-11931	NS_000200.1	-	234233055	234233006
Sma-1-11932	NW_003038068.1	-	304464	304415
Sma-1-11933	NS_000200.1	-	263371672	263371623
Sma-1-11934	NW_003037866.1	-	445045	444996
Sma-1-11935	NW_003037982.1	-	1344873	1344824
Sma-1-11936	NS_000200.1	-	215004068	215004019
Sma-1-11937	NS_000200.1	-	111932844	111932795
Sma-1-11938	NW_003037970.1	+	139089	139138
Sma-1-11939	NS_000200.1	+	201195279	201195328
Sma-1-11940	NW_003037974.1	-	331128	331079
Sma-1-11941	NS_000200.1	-	205006434	205006385
Sma-1-11942	NW_003037992.1	-	258039	257990
Sma-1-11943	NS_000200.1	-	218099520	218099471
Sma-1-11944	NW_003038042.1	-	5512	5463
Sma-1-11945	NS_000200.1	-	253951111	253951062
Sma-1-11946	NW_003037818.1	+	10135	10184
Sma-1-11947	NS_000200.1	+	48725542	48725591
Sma-1-11948	NW_003038251.1	+	410461	410510
Sma-1-11949	NS_000200.1	+	318742233	318742282
Sma-1-11950	NW_003037834.1	+	1162034	1162083
Sma-1-11951	NW_003037850.1	+	832326	832375
Sma-1-11952	NW_003037866.1	+	790588	790637
Sma-1-11953	NW_003037881.1	+	470969	471018
Sma-1-11954	NW_003037924.1	+	489303	489352
Sma-1-11955	NW_003038038.1	+	919884	919933
Sma-1-11956	NW_003038038.1	-	919760	919711
Sma-1-11957	NW_003038128.1	+	310190	310239
Sma-1-11958	NW_003038144.1	-	14662	14613
Sma-1-11959	NS_000200.1	+	74322520	74322569
Sma-1-11960	NS_000200.1	+	92818356	92818405
Sma-1-11961	NS_000200.1	+	112278387	112278436

Sma-1-11962	NS_000200.1	+	124902760	124902809
Sma-1-11963	NS_000200.1	+	172196261	172196310
Sma-1-11964	NS_000200.1	+	252823277	252823326
Sma-1-11965	NS_000200.1	+	281028478	281028527
Sma-1-11966	NS_000200.1	-	288279038	288278989
Sma-1-11967	NS_000200.1	-	252823153	252823104
Sma-1-11968	NW_003024681.1	-	922	873
Sma-1-11969	NW_003037806.1	-	490502	490453
Sma-1-11970	NW_003038252.1	+	377455	377504
Sma-1-11971	NS_000200.1	+	319233043	319233092
Sma-1-11972	NS_000200.1	-	349103287	349103238
Sma-1-11973	NS_000200.1	-	28685716	28685667
Sma-1-11974	NW_003037999.1	-	236824	236775
Sma-1-11975	NS_000200.1	-	221931378	221931329
Sma-1-11976	NW_003038014.1	+	441061	441110
Sma-1-11977	NS_000200.1	+	230181750	230181799
Sma-1-11978	NW_003037857.1	-	603016	602967
Sma-1-11979	NS_000200.1	-	100623373	100623324
Sma-1-11980	NW_003038032.1	-	390250	390201
Sma-1-11981	NW_003038084.1	+	359632	359681
Sma-1-11982	NS_000200.1	+	271374876	271374925
Sma-1-11983	NS_000200.1	-	245549410	245549361
Sma-1-11984	NW_003037798.1	-	2195806	2195757
Sma-1-11985	NW_003037813.1	-	963534	963485
Sma-1-11986	NW_003037841.1	+	1352208	1352257
Sma-1-11987	NW_003037853.1	+	994182	994231
Sma-1-11988	NW_003038034.1	+	792019	792068
Sma-1-11989	NW_003026490.1	-	415585	415536
Sma-1-11990	NW_003038102.1	-	45191	45142
Sma-1-11991	NS_000200.1	+	83963389	83963438
Sma-1-11992	NS_000200.1	+	96486233	96486282
Sma-1-11993	NS_000200.1	+	247733775	247733824
Sma-1-11994	NS_000200.1	-	274724137	274724088
Sma-1-11995	NS_000200.1	-	268047301	268047252
Sma-1-11996	NS_000200.1	-	41950397	41950348
Sma-1-11997	NS_000200.1	-	16454964	16454915
Sma-1-11998	NW_003037771.1	+	55041	55090
Sma-1-11999	NS_000200.1	+	378355947	378355996
Sma-1-12000	NW_003037797.1	-	2882915	2882866
Sma-1-12001	NS_000200.1	-	13310775	13310726
Sma-1-12002	NW_003037913.1	-	40456	40407
Sma-1-12003	NS_000200.1	-	158604207	158604158
Sma-1-12004	NW_003038001.1	+	392324	392373
Sma-1-12005	NS_000200.1	+	223101885	223101934
Sma-1-12006	NW_003037793.1	-	1588518	1588469
Sma-1-12007	NW_003037795.1	-	657716	657667
Sma-1-12008	NW_003037823.1	-	198352	198303
Sma-1-12009	NS_000200.1	-	56114971	56114922
Sma-1-12010	NS_000200.1	-	7048599	7048550
Sma-1-12011	NS_000200.1	-	1588518	1588469
Sma-1-12012	NW_003038148.1	-	502989	502940
Sma-1-12013	NS_000200.1	-	290576973	290576924

Sma-1-12014	NW_003028832.1	-	104	55
Sma-1-12015	NW_003029671.1	+	296	345
Sma-1-12016	NW_003030625.1	-	131	82
Sma-1-12017	NW_003031026.1	-	1543	1494
Sma-1-12018	NW_003037929.1	+	54035	54084
Sma-1-12019	NW_003037937.1	-	232749	232700
Sma-1-12020	NW_003037800.1	+	1870369	1870418
Sma-1-12021	NW_003037810.1	-	1217791	1217742
Sma-1-12022	NW_003037813.1	-	1190613	1190564
Sma-1-12023	NW_003037817.1	-	861019	860970
Sma-1-12024	NW_003037845.1	+	629481	629530
Sma-1-12025	NW_003037854.1	-	1467776	1467727
Sma-1-12026	NW_003037866.1	+	251888	251937
Sma-1-12027	NW_003037871.1	+	118505	118554
Sma-1-12028	NW_003037881.1	-	318355	318306
Sma-1-12029	NW_003037887.1	-	117812	117763
Sma-1-12030	NW_003037891.1	+	1331941	1331990
Sma-1-12031	NW_003037916.1	+	825727	825776
Sma-1-12032	NW_003037916.1	+	891413	891462
Sma-1-12033	NW_003038008.1	-	690449	690400
Sma-1-12034	NW_003038012.1	+	354647	354696
Sma-1-12035	NW_003038012.1	-	193348	193299
Sma-1-12036	NW_003026487.1	+	33345	33394
Sma-1-12037	NW_003038055.1	-	36073	36024
Sma-1-12038	NW_003038087.1	-	390969	390920
Sma-1-12039	NS_000200.1	+	20499729	20499778
Sma-1-12040	NS_000200.1	+	87631384	87631433
Sma-1-12041	NS_000200.1	+	111739687	111739736
Sma-1-12042	NS_000200.1	+	117566243	117566292
Sma-1-12043	NS_000200.1	+	139213219	139213268
Sma-1-12044	NS_000200.1	+	164019138	164019187
Sma-1-12045	NS_000200.1	+	164084824	164084873
Sma-1-12046	NS_000200.1	+	175294813	175294862
Sma-1-12047	NS_000200.1	+	228678586	228678635
Sma-1-12048	NS_000200.1	+	260944039	260944088
Sma-1-12049	NS_000200.1	+	366389170	366389219
Sma-1-12050	NS_000200.1	-	370046279	370046230
Sma-1-12051	NS_000200.1	-	366599671	366599622
Sma-1-12052	NS_000200.1	-	325552718	325552669
Sma-1-12053	NS_000200.1	-	273419370	273419321
Sma-1-12054	NS_000200.1	-	258735395	258735346
Sma-1-12055	NS_000200.1	-	228517287	228517238
Sma-1-12056	NS_000200.1	-	226363545	226363496
Sma-1-12057	NS_000200.1	-	181432658	181432609
Sma-1-12058	NS_000200.1	-	130581790	130581741
Sma-1-12059	NS_000200.1	-	124750146	124750097
Sma-1-12060	NS_000200.1	-	98372197	98372148
Sma-1-12061	NS_000200.1	-	48243371	48243322
Sma-1-12062	NS_000200.1	-	42177476	42177427
Sma-1-12063	NS_000200.1	-	37822313	37822264
Sma-1-12064	NW_003037891.1	+	166409	166458
Sma-1-12065	NS_000200.1	+	138047687	138047736

Sma-1-12066	NW_003037891.1	-	251711	251662
Sma-1-12067	NS_000200.1	-	138132989	138132940
Sma-1-12068	NW_003037847.1	-	122909	122860
Sma-1-12069	NS_000200.1	-	88774639	88774590
Sma-1-12070	NW_003038442.1	-	14487	14435
Sma-1-12071	NS_000200.1	-	329870540	329870488
Sma-1-12072	NW_003037883.1	+	452704	452753
Sma-1-12073	NW_003037945.1	-	406309	406260
Sma-1-12074	NW_003038094.1	+	19068	19117
Sma-1-12075	NS_000200.1	+	126516427	126516476
Sma-1-12076	NS_000200.1	+	274062382	274062431
Sma-1-12077	NS_000200.1	-	189508273	189508224
Sma-1-12078	NW_003037896.1	-	154775	154726
Sma-1-12079	NW_003038116.1	+	95245	95294
Sma-1-12080	NS_000200.1	+	277310523	277310572
Sma-1-12081	NS_000200.1	-	143075198	143075149
Sma-1-12082	NW_003038044.1	+	123126	123175
Sma-1-12083	NS_000200.1	+	254747908	254747957
Sma-1-12084	NW_003037894.1	-	520807	520758
Sma-1-12085	NW_003037961.1	+	344017	344066
Sma-1-12086	NS_000200.1	+	195968259	195968308
Sma-1-12087	NS_000200.1	-	142417380	142417331
Sma-1-12088	NW_003037843.1	-	257895	257846
Sma-1-12089	NS_000200.1	-	85897202	85897153
Sma-1-12090	NW_003037839.1	-	1124400	1124351
Sma-1-12091	NS_000200.1	-	80082550	80082501
Sma-1-12092	NW_003037904.1	+	287086	287135
Sma-1-12093	NS_000200.1	+	149001585	149001634
Sma-1-12094	NW_003037904.1	+	511993	512042
Sma-1-12095	NW_003038037.1	-	1008195	1008146
Sma-1-12096	NS_000200.1	+	149226492	149226541
Sma-1-12097	NS_000200.1	-	251163604	251163555
Sma-1-12098	NW_003037818.1	-	1943573	1943524
Sma-1-12099	NS_000200.1	-	50658980	50658931
Sma-1-12100	NW_003037831.1	+	1138281	1138330
Sma-1-12101	NW_003026517.1	-	437205	437156
Sma-1-12102	NS_000200.1	+	70587583	70587632
Sma-1-12103	NS_000200.1	-	300043214	300043165
Sma-1-12104	NW_003023040.1	-	2512	2463
Sma-1-12105	NS_000200.1	-	372139354	372139305
Sma-1-12106	NW_003037912.1	-	1339979	1339930
Sma-1-12107	NS_000200.1	-	158151554	158151505
Sma-1-12108	NW_003025649.1	+	141	190
Sma-1-12109	NW_003037880.1	+	522416	522465
Sma-1-12110	NW_003037942.1	+	649425	649474
Sma-1-12111	NS_000200.1	+	124205974	124206023
Sma-1-12112	NS_000200.1	+	186409333	186409382
Sma-1-12113	NS_000200.1	+	356299602	356299651
Sma-1-12114	NW_003038083.1	-	79454	79405
Sma-1-12115	NS_000200.1	-	270322791	270322742
Sma-1-12116	NW_003026458.1	+	212618	212667
Sma-1-12117	NS_000200.1	+	102080848	102080897

Sma-1-12118	NW_003029652.1	+	74	123
Sma-1-12119	NW_003037977.1	-	713818	713769
Sma-1-12120	NW_003038019.1	+	36086	36135
Sma-1-12121	NW_003038026.1	+	593009	593058
Sma-1-12122	NS_000200.1	+	233352904	233352953
Sma-1-12123	NS_000200.1	+	240100770	240100819
Sma-1-12124	NS_000200.1	+	366353414	366353463
Sma-1-12125	NS_000200.1	-	208999838	208999789
Sma-1-12126	NW_003037800.1	-	1388204	1388155
Sma-1-12127	NS_000200.1	-	20017564	20017515
Sma-1-12128	NW_003037810.1	+	292684	292733
Sma-1-12129	NS_000200.1	+	36897206	36897255
Sma-1-12130	NW_003034094.1	+	2309	2358
Sma-1-12131	NS_000200.1	+	363724257	363724306
Sma-1-12132	NW_003026510.1	-	56349	56300
Sma-1-12133	NS_000200.1	-	283496530	283496481
Sma-1-12134	NW_003037939.1	+	605910	605959
Sma-1-12135	NS_000200.1	+	183475664	183475713
Sma-1-12136	NW_003037995.1	+	274630	274679
Sma-1-12137	NW_003038008.1	-	210965	210916
Sma-1-12138	NS_000200.1	+	219964398	219964447
Sma-1-12139	NS_000200.1	-	225884061	225884012
Sma-1-12140	NW_003038131.1	+	40479	40528
Sma-1-12141	NS_000200.1	+	282002323	282002372
Sma-1-12142	NW_003026515.1	+	163590	163639
Sma-1-12143	NW_003026517.1	-	283101	283052
Sma-1-12144	NW_003038152.1	+	265861	265910
Sma-1-12145	NS_000200.1	+	290808001	290808050
Sma-1-12146	NS_000200.1	+	292871585	292871634
Sma-1-12147	NS_000200.1	-	299889110	299889061
Sma-1-12148	NW_003035870.1	-	11140	11091
Sma-1-12149	NW_003037862.1	+	83761	83810
Sma-1-12150	NW_003037918.1	-	1457746	1457697
Sma-1-12151	NS_000200.1	+	105735422	105735471
Sma-1-12152	NS_000200.1	-	335376429	335376380
Sma-1-12153	NS_000200.1	-	168024736	168024687
Sma-1-12154	NW_003037800.1	+	1310260	1310309
Sma-1-12155	NS_000200.1	+	19939620	19939669
Sma-1-12156	NW_003037911.1	-	550066	550017
Sma-1-12157	NS_000200.1	-	156337419	156337370
Sma-1-12158	NW_003037797.1	+	1465620	1465669
Sma-1-12159	NS_000200.1	+	11893480	11893529
Sma-1-12160	NW_003037799.1	+	1191413	1191462
Sma-1-12161	NS_000200.1	+	18181767	18181816
Sma-1-12162	NW_003037928.1	-	349837	349788
Sma-1-12163	NS_000200.1	-	174673790	174673741
Sma-1-12164	NW_003037803.1	+	83386	83435
Sma-1-12165	NS_000200.1	+	23583923	23583972
Sma-1-12166	NW_003037817.1	-	1208172	1208123
Sma-1-12167	NS_000200.1	-	48590524	48590475
Sma-1-12168	NW_003037918.1	+	1786313	1786362
Sma-1-12169	NS_000200.1	+	168353303	168353352

Sma-1-12170	NW_003037805.1	-	756680	756631
Sma-1-12171	NW_003037827.1	-	738036	737987
Sma-1-12172	NS_000200.1	-	63360177	63360128
Sma-1-12173	NS_000200.1	-	27314524	27314475
Sma-1-12174	NW_003037926.1	+	75334	75383
Sma-1-12175	NS_000200.1	+	173243604	173243653
Sma-1-12176	NW_003037935.1	-	100503	100454
Sma-1-12177	NS_000200.1	-	179517038	179516989
Sma-1-12178	NW_003037937.1	+	124189	124238
Sma-1-12179	NW_003037798.1	-	2389401	2389352
Sma-1-12180	NW_003037810.1	-	208959	208910
Sma-1-12181	NW_003037813.1	-	929753	929704
Sma-1-12182	NW_003037827.1	+	195205	195254
Sma-1-12183	NW_003037827.1	+	734432	734481
Sma-1-12184	NW_003037939.1	-	609177	609128
Sma-1-12185	NW_003037941.1	+	957158	957207
Sma-1-12186	NW_003038017.1	-	174133	174084
Sma-1-12187	NW_003038025.1	-	258177	258128
Sma-1-12188	NW_003038028.1	-	700675	700626
Sma-1-12189	NW_003038252.1	-	167787	167738
Sma-1-12190	NW_003026517.1	-	428410	428361
Sma-1-12191	NS_000200.1	+	62817346	62817395
Sma-1-12192	NS_000200.1	+	63356573	63356622
Sma-1-12193	NS_000200.1	+	181324098	181324147
Sma-1-12194	NS_000200.1	+	185691632	185691681
Sma-1-12195	NS_000200.1	-	319023375	319023326
Sma-1-12196	NS_000200.1	-	300034419	300034370
Sma-1-12197	NS_000200.1	-	242008249	242008200
Sma-1-12198	NS_000200.1	-	239116422	239116373
Sma-1-12199	NS_000200.1	-	231940164	231940115
Sma-1-12200	NS_000200.1	-	183478931	183478882
Sma-1-12201	NS_000200.1	-	41916616	41916567
Sma-1-12202	NS_000200.1	-	36813481	36813432
Sma-1-12203	NS_000200.1	-	16648559	16648510
Sma-1-12204	NW_003037937.1	+	196473	196522
Sma-1-12205	NS_000200.1	+	181396382	181396431
Sma-1-12206	NW_003038033.1	-	249504	249455
Sma-1-12207	NS_000200.1	-	246229506	246229457
Sma-1-12208	NW_003029920.1	+	1218	1267
Sma-1-12209	NW_003037798.1	+	1943255	1943304
Sma-1-12210	NW_003037803.1	-	796927	796878
Sma-1-12211	NW_003037807.1	-	377673	377624
Sma-1-12212	NW_003037831.1	+	850648	850697
Sma-1-12213	NW_003037909.1	+	311923	311972
Sma-1-12214	NW_003037909.1	-	362979	362930
Sma-1-12215	NW_003037961.1	+	14019	14068
Sma-1-12216	NW_003037964.1	+	418340	418389
Sma-1-12217	NW_003038011.1	+	487461	487510
Sma-1-12218	NS_000200.1	+	16202413	16202462
Sma-1-12219	NS_000200.1	+	70299950	70299999
Sma-1-12220	NS_000200.1	+	153853572	153853621
Sma-1-12221	NS_000200.1	+	195638261	195638310

Sma-1-12222	NS_000200.1	+	198019862	198019911
Sma-1-12223	NS_000200.1	+	228015111	228015160
Sma-1-12224	NS_000200.1	+	376793179	376793228
Sma-1-12225	NS_000200.1	-	153904628	153904579
Sma-1-12226	NS_000200.1	-	30639189	30639140
Sma-1-12227	NS_000200.1	-	24297464	24297415
Sma-1-12228	NW_003038502.1	-	102979	102930
Sma-1-12229	NS_000200.1	-	335102024	335101975
Sma-1-12230	NW_003038083.1	-	52413	52364
Sma-1-12231	NS_000200.1	-	270295750	270295701
Sma-1-12232	NW_003026472.1	-	291908	291859
Sma-1-12233	NS_000200.1	-	236703900	236703851
Sma-1-12234	NW_003038733.1	+	5540	5589
Sma-1-12235	NS_000200.1	+	348987333	348987382
Sma-1-12236	NW_003038066.1	-	342470	342421
Sma-1-12237	NS_000200.1	-	262811414	262811365
Sma-1-12238	NW_003038023.1	-	731401	731352
Sma-1-12239	NS_000200.1	-	237891692	237891643
Sma-1-12240	NW_003037798.1	-	1533727	1533678
Sma-1-12241	NS_000200.1	-	15792885	15792836
Sma-1-12242	NW_003026470.1	+	211070	211119
Sma-1-12243	NS_000200.1	+	219335039	219335088
Sma-1-12244	NW_003037800.1	+	632571	632620
Sma-1-12245	NS_000200.1	+	19261931	19261980
Sma-1-12246	NW_003037747.1	+	16805	16854
Sma-1-12247	NW_003038511.1	-	1354	1305
Sma-1-12248	NW_003026471.1	+	191436	191485
Sma-1-12249	NW_003037801.1	-	555650	555601
Sma-1-12250	NW_003037810.1	-	718387	718338
Sma-1-12251	NW_003037814.1	+	386365	386414
Sma-1-12252	NW_003037832.1	-	480963	480914
Sma-1-12253	NW_003037915.1	+	1093720	1093769
Sma-1-12254	NW_003038133.1	-	267440	267391
Sma-1-12255	NW_003038187.1	+	115656	115705
Sma-1-12256	NS_000200.1	+	43370225	43370274
Sma-1-12257	NS_000200.1	+	162993706	162993755
Sma-1-12258	NS_000200.1	+	222303669	222303718
Sma-1-12259	NS_000200.1	+	302067514	302067563
Sma-1-12260	NS_000200.1	+	376942004	376942053
Sma-1-12261	NS_000200.1	-	338346566	338346517
Sma-1-12262	NS_000200.1	-	283139622	283139573
Sma-1-12263	NS_000200.1	-	71690394	71690345
Sma-1-12264	NS_000200.1	-	37322909	37322860
Sma-1-12265	NS_000200.1	-	21645130	21645081
Sma-1-12266	NW_003037946.1	-	81220	81171
Sma-1-12267	NS_000200.1	-	190775080	190775031
Sma-1-12268	NW_003037970.1	+	106448	106497
Sma-1-12269	NS_000200.1	+	201162638	201162687
Sma-1-12270	NW_003037799.1	+	1174156	1174205
Sma-1-12271	NW_003037918.1	-	2164475	2164426
Sma-1-12272	NS_000200.1	+	18164510	18164559
Sma-1-12273	NS_000200.1	-	168731465	168731416

Sma-1-12274	NW_003039129.1	-	408983	408934
Sma-1-12275	NS_000200.1	-	229363791	229363742
Sma-1-12276	NW_003037881.1	-	639228	639179
Sma-1-12277	NS_000200.1	-	125071019	125070970
Sma-1-12278	NW_003037982.1	-	1249333	1249284
Sma-1-12279	NS_000200.1	-	214908528	214908479
Sma-1-12280	NW_003037982.1	+	1329433	1329482
Sma-1-12281	NS_000200.1	+	214988628	214988677
Sma-1-12282	NW_003037999.1	-	340378	340329
Sma-1-12283	NS_000200.1	-	222034932	222034883
Sma-1-12284	NW_003038132.1	+	411341	411390
Sma-1-12285	NS_000200.1	+	282831811	282831860
Sma-1-12286	NW_003020690.1	-	897	848
Sma-1-12287	NW_003038261.1	+	200733	200782
Sma-1-12288	NS_000200.1	+	323814384	323814433
Sma-1-12289	NS_000200.1	-	360670296	360670247
Sma-1-12290	NW_003038240.1	+	48647	48696
Sma-1-12291	NS_000200.1	+	314187578	314187627
Sma-1-12292	NW_003037851.1	-	259962	259913
Sma-1-12293	NS_000200.1	-	93663272	93663223
Sma-1-12294	NW_003038033.1	+	325626	325675
Sma-1-12295	NS_000200.1	+	246305628	246305677
Sma-1-12296	NW_003038037.1	+	749219	749268
Sma-1-12297	NS_000200.1	+	250904628	250904677
Sma-1-12298	NW_003037885.1	-	811541	811492
Sma-1-12299	NS_000200.1	-	128909488	128909439
Sma-1-12300	NW_003037913.1	-	956112	956063
Sma-1-12301	NW_003037913.1	-	951941	951892
Sma-1-12302	NS_000200.1	-	159519863	159519814
Sma-1-12303	NS_000200.1	-	159515692	159515643
Sma-1-12304	NW_003037796.1	-	1526798	1526749
Sma-1-12305	NS_000200.1	-	9736442	9736393
Sma-1-12306	NW_003037873.1	-	220744	220695
Sma-1-12307	NW_003037795.1	-	641090	641041
Sma-1-12308	NS_000200.1	-	118959872	118959823
Sma-1-12309	NS_000200.1	-	7031973	7031924
Sma-1-12310	NW_003037891.1	+	1094117	1094166
Sma-1-12311	NS_000200.1	+	138975395	138975444
Sma-1-12312	NW_003037847.1	+	312470	312519
Sma-1-12313	NS_000200.1	+	88964200	88964249
Sma-1-12314	NW_003037846.1	+	373843	373892
Sma-1-12315	NS_000200.1	+	88008413	88008462
Sma-1-12316	NW_003037840.1	+	1302121	1302170
Sma-1-12317	NS_000200.1	+	81925445	81925494
Sma-1-12318	NW_003037834.1	+	872343	872392
Sma-1-12319	NS_000200.1	+	74032829	74032878
Sma-1-12320	NW_003038252.1	-	302071	302022
Sma-1-12321	NS_000200.1	-	319157659	319157610
Sma-1-12322	NW_003037829.1	+	1860869	1860918
Sma-1-12323	NS_000200.1	+	67761407	67761456
Sma-1-12324	NW_003037833.1	+	101066	101115
Sma-1-12325	NS_000200.1	+	72292162	72292211

Sma-1-12326	NW_003037808.1	-	91429	91380
Sma-1-12327	NS_000200.1	-	31878066	31878017
Sma-1-12328	NW_003038509.1	+	137232	137281
Sma-1-12329	NS_000200.1	+	337769684	337769733
Sma-1-12330	NW_003037863.1	+	46237	46286
Sma-1-12331	NS_000200.1	+	106804604	106804653
Sma-1-12332	NW_003037924.1	-	413217	413168
Sma-1-12333	NS_000200.1	-	172120175	172120126
Sma-1-12334	NW_003037798.1	-	1873563	1873514
Sma-1-12335	NS_000200.1	-	16132721	16132672
Sma-1-12336	NW_003038489.1	-	109457	109408
Sma-1-12337	NS_000200.1	-	333821740	333821691
Sma-1-12338	NW_003031885.1	+	737	786
Sma-1-12339	NW_003035682.1	+	50347	50396
Sma-1-12340	NW_003037787.1	+	186032	186081
Sma-1-12341	NW_003037797.1	-	1803150	1803101
Sma-1-12342	NW_003037807.1	+	1449519	1449568
Sma-1-12343	NW_003037868.1	+	22515	22564
Sma-1-12344	NW_003037884.1	-	1127242	1127193
Sma-1-12345	NW_003037893.1	+	220380	220429
Sma-1-12346	NW_003037902.1	-	607664	607615
Sma-1-12347	NW_003037911.1	+	591072	591121
Sma-1-12348	NW_003037914.1	-	193117	193068
Sma-1-12349	NW_003037924.1	-	531347	531298
Sma-1-12350	NW_003037958.1	+	353197	353246
Sma-1-12351	NW_003037977.1	-	967003	966954
Sma-1-12352	NS_000200.1	+	31711035	31711084
Sma-1-12353	NS_000200.1	+	115994227	115994276
Sma-1-12354	NS_000200.1	+	141334690	141334739
Sma-1-12355	NS_000200.1	+	156378425	156378474
Sma-1-12356	NS_000200.1	+	195017381	195017430
Sma-1-12357	NS_000200.1	+	332672400	332672449
Sma-1-12358	NS_000200.1	+	367208477	367208526
Sma-1-12359	NS_000200.1	+	380434201	380434250
Sma-1-12360	NS_000200.1	-	209253023	209252974
Sma-1-12361	NS_000200.1	-	172238305	172238256
Sma-1-12362	NS_000200.1	-	160337367	160337318
Sma-1-12363	NS_000200.1	-	147362898	147362849
Sma-1-12364	NS_000200.1	-	127906907	127906858
Sma-1-12365	NS_000200.1	-	12231010	12230961
Sma-1-12366	NW_003038072.1	+	73197	73246
Sma-1-12367	NS_000200.1	+	265235425	265235474
Sma-1-12368	NW_003037918.1	+	2188336	2188385
Sma-1-12369	NS_000200.1	+	168755326	168755375
Sma-1-12370	NW_003037906.1	+	513559	513608
Sma-1-12371	NS_000200.1	+	150821338	150821387
Sma-1-12372	NW_003037876.1	-	172254	172205
Sma-1-12373	NW_003037807.1	-	170377	170328
Sma-1-12374	NW_003038037.1	-	178829	178780
Sma-1-12375	NS_000200.1	-	250334238	250334189
Sma-1-12376	NS_000200.1	-	120717890	120717841
Sma-1-12377	NS_000200.1	-	30431893	30431844

Sma-1-12378	NW_003037794.1	+	278684	278733
Sma-1-12379	NS_000200.1	+	4458279	4458328
Sma-1-12380	NW_003037939.1	+	494465	494514
Sma-1-12381	NS_000200.1	+	183364219	183364268
Sma-1-12382	NW_003037881.1	+	828639	828688
Sma-1-12383	NW_003037942.1	+	1126150	1126199
Sma-1-12384	NS_000200.1	+	125260430	125260479
Sma-1-12385	NS_000200.1	+	186886058	186886107
Sma-1-12386	NW_003026464.1	-	559658	559609
Sma-1-12387	NS_000200.1	-	203859967	203859918
Sma-1-12388	NW_003037933.1	-	195063	195014
Sma-1-12389	NS_000200.1	-	178608235	178608186
Sma-1-12390	NW_003037793.1	+	3016214	3016263
Sma-1-12391	NS_000200.1	+	3016214	3016263
Sma-1-12392	NW_003037939.1	-	705081	705032
Sma-1-12393	NS_000200.1	-	183574835	183574786
Sma-1-12394	NW_003037792.1	-	274538	274489
Sma-1-12395	NS_000200.1	-	382711882	382711833
Sma-1-12396	NW_003037832.1	-	337867	337818
Sma-1-12397	NS_000200.1	-	71547298	71547249
Sma-1-12398	NW_003037833.1	+	830542	830591
Sma-1-12399	NS_000200.1	+	73021638	73021687
Sma-1-12400	NW_003037917.1	+	1086388	1086437
Sma-1-12401	NS_000200.1	+	166123192	166123241
Sma-1-12402	NW_003037793.1	+	3117564	3117613
Sma-1-12403	NS_000200.1	+	3117564	3117613
Sma-1-12404	NW_003037793.1	+	1566431	1566480
Sma-1-12405	NS_000200.1	+	1566431	1566480
Sma-1-12406	NW_003038014.1	+	368599	368648
Sma-1-12407	NS_000200.1	+	230109288	230109337
Sma-1-12408	NW_003038014.1	+	376592	376641
Sma-1-12409	NS_000200.1	+	230117281	230117330
Sma-1-12410	NW_003037797.1	-	2546787	2546738
Sma-1-12411	NS_000200.1	-	12974647	12974598
Sma-1-12412	NW_003026465.1	-	618439	618390
Sma-1-12413	NW_003037799.1	-	1042976	1042927
Sma-1-12414	NW_003038097.1	-	11368	11319
Sma-1-12415	NS_000200.1	-	274261670	274261621
Sma-1-12416	NS_000200.1	-	208011033	208010984
Sma-1-12417	NS_000200.1	-	18033330	18033281
Sma-1-12418	NW_003026458.1	+	98808	98857
Sma-1-12419	NS_000200.1	+	101967038	101967087
Sma-1-12420	NW_003037906.1	+	528716	528765
Sma-1-12421	NS_000200.1	+	150836495	150836544
Sma-1-12422	NW_003037913.1	-	982966	982917
Sma-1-12423	NS_000200.1	-	159546717	159546668
Sma-1-12424	NW_003038050.1	-	226258	226209
Sma-1-12425	NS_000200.1	-	256501152	256501103
Sma-1-12426	NW_003038508.1	+	61259	61308
Sma-1-12427	NW_003026472.1	+	273956	274005
Sma-1-12428	NS_000200.1	+	236685948	236685997
Sma-1-12429	NS_000200.1	+	337168227	337168276

Sma-1-12430	NW_003037800.1	-	25303	25254
Sma-1-12431	NS_000200.1	-	18654663	18654614
Sma-1-12432	NW_003037914.1	+	1710738	1710787
Sma-1-12433	NS_000200.1	+	161854988	161855037
Sma-1-12434	NW_003037814.1	+	187689	187738
Sma-1-12435	NS_000200.1	+	43171549	43171598
Sma-1-12436	NW_003037804.1	-	1308578	1308529
Sma-1-12437	NS_000200.1	-	26448598	26448549
Sma-1-12438	NW_003037923.1	-	187346	187297
Sma-1-12439	NS_000200.1	-	171243559	171243510
Sma-1-12440	NW_003026605.1	-	8584	8535
Sma-1-12441	NS_000200.1	-	307687448	307687399
Sma-1-12442	NW_003037825.1	+	983424	983473
Sma-1-12443	NS_000200.1	+	60113206	60113255
Sma-1-12444	NW_003037988.1	-	150587	150538
Sma-1-12445	NS_000200.1	-	216144405	216144356
Sma-1-12446	NW_003038027.1	+	237753	237802
Sma-1-12447	NS_000200.1	+	240608467	240608516
Sma-1-12448	NW_003038035.1	-	185414	185365
Sma-1-12449	NS_000200.1	-	248046906	248046857
Sma-1-12450	NW_003038086.1	+	404890	404939
Sma-1-12451	NS_000200.1	+	272653483	272653532
Sma-1-12452	NW_003037850.1	-	1208518	1208469
Sma-1-12453	NS_000200.1	-	93194548	93194499
Sma-1-12454	NW_003037974.1	-	818015	817966
Sma-1-12455	NS_000200.1	-	205493321	205493272
Sma-1-12456	NW_003034567.1	+	10982	11031
Sma-1-12457	NW_003035881.1	+	332054	332103
Sma-1-12458	NS_000200.1	+	327651876	327651925
Sma-1-12459	NS_000200.1	+	341396259	341396308
Sma-1-12460	NW_003038014.1	+	533567	533616
Sma-1-12461	NS_000200.1	+	230274256	230274305
Sma-1-12462	NW_003038499.1	-	47460	47411
Sma-1-12463	NS_000200.1	-	334579994	334579945
Sma-1-12464	NW_003022703.1	-	1163	1114
Sma-1-12465	NW_003037821.1	+	624161	624210
Sma-1-12466	NW_003038008.1	-	499106	499057
Sma-1-12467	NS_000200.1	+	53768645	53768694
Sma-1-12468	NS_000200.1	-	365449719	365449670
Sma-1-12469	NS_000200.1	-	226172202	226172153
env-87	AB231864.1	+	33804	33853
Sma-1-12470	NW_003033780.1	-	3463	3414
Sma-1-12471	NW_003038013.1	-	330441	330392
Sma-1-12472	NW_003026456.1	-	511262	511213
Sma-1-12473	NW_003037795.1	-	785234	785185
Sma-1-12474	NW_003037796.1	+	275303	275352
Sma-1-12475	NW_003037797.1	+	1885676	1885725
Sma-1-12476	NW_003037816.1	+	526302	526351
Sma-1-12477	NW_003037825.1	+	1396103	1396152
Sma-1-12478	NW_003037831.1	-	499026	498977
Sma-1-12479	NW_003037852.1	-	185363	185314
Sma-1-12480	NW_003037894.1	-	427467	427418

Sma-1-12481	NW_003037906.1	+	17788	17837
Sma-1-12482	NW_003037913.1	+	469171	469220
Sma-1-12483	NW_003037917.1	-	853785	853736
Sma-1-12484	NW_003037932.1	+	823376	823425
Sma-1-12485	NW_003037939.1	-	870356	870307
Sma-1-12486	NW_003037978.1	-	104369	104320
Sma-1-12487	NW_003037980.1	+	183348	183397
Sma-1-12488	NW_003038008.1	-	18784	18735
Sma-1-12489	NW_003038015.1	+	92406	92455
Sma-1-12490	NW_003038015.1	+	188145	188194
Sma-1-12491	NW_003038033.1	+	148806	148855
Sma-1-12492	NW_003038069.1	+	129632	129681
Sma-1-12493	NW_003038252.1	+	164417	164466
Sma-1-12494	NW_003038255.1	-	275992	275943
Sma-1-12495	NW_003038055.1	+	265255	265304
Sma-1-12496	NW_003038136.1	-	162994	162945
Sma-1-12497	NS_000200.1	+	8484947	8484996
Sma-1-12498	NS_000200.1	+	12313536	12313585
Sma-1-12499	NS_000200.1	+	46735174	46735223
Sma-1-12500	NS_000200.1	+	60525885	60525934
Sma-1-12501	NS_000200.1	+	150325567	150325616
Sma-1-12502	NS_000200.1	+	159032922	159032971
Sma-1-12503	NS_000200.1	+	178356572	178356621
Sma-1-12504	NS_000200.1	+	211144210	211144259
Sma-1-12505	NS_000200.1	+	230415896	230415945
Sma-1-12506	NS_000200.1	+	230511635	230511684
Sma-1-12507	NS_000200.1	+	246128808	246128857
Sma-1-12508	NS_000200.1	+	258964577	258964626
Sma-1-12509	NS_000200.1	+	263973038	263973087
Sma-1-12510	NS_000200.1	+	319020005	319020054
Sma-1-12511	NS_000200.1	-	358944219	358944170
Sma-1-12512	NS_000200.1	-	320665137	320665088
Sma-1-12513	NS_000200.1	-	284285063	284285014
Sma-1-12514	NS_000200.1	-	229720067	229720018
Sma-1-12515	NS_000200.1	-	225691880	225691831
Sma-1-12516	NS_000200.1	-	209372379	209372330
Sma-1-12517	NS_000200.1	-	183740110	183740061
Sma-1-12518	NS_000200.1	-	165890589	165890540
Sma-1-12519	NS_000200.1	-	142324040	142323991
Sma-1-12520	NS_000200.1	-	94577258	94577209
Sma-1-12521	NS_000200.1	-	69948328	69948279
Sma-1-12522	NS_000200.1	-	58434398	58434349
Sma-1-12523	NS_000200.1	-	7176117	7176068
Sma-1-12524	NW_003038333.1	+	602	651
Sma-1-12525	NS_000200.1	+	326253793	326253842
Sma-1-12526	NW_003038211.1	+	143425	143474
Sma-1-12527	NW_003038224.1	+	107264	107313
Sma-1-12528	NW_003038086.1	+	164406	164455
Sma-1-12529	NW_003038102.1	-	45594	45545
Sma-1-12530	NS_000200.1	+	272412999	272413048
Sma-1-12531	NS_000200.1	+	306163495	306163544
Sma-1-12532	NS_000200.1	+	309518992	309519041

Sma-1-12533	NS_000200.1	-	274724540	274724491
Sma-1-12534	NW_003038022.1	-	468265	468216
Sma-1-12535	NS_000200.1	-	236149107	236149058
Sma-1-12536	NW_003038195.1	+	4250	4299
Sma-1-12537	NS_000200.1	+	302993022	302993071
Sma-1-12538	NW_003021533.1	-	98	49
Sma-1-12539	NW_003032444.1	-	1088	1039
Sma-1-12540	NW_003035313.1	-	417	368
Sma-1-12541	NS_000200.1	-	370994997	370994948
Sma-1-12542	NS_000200.1	-	348033254	348033205
Sma-1-12543	NS_000200.1	-	346092757	346092708
Sma-1-12544	NW_003037795.1	-	1137309	1137260
Sma-1-12545	NS_000200.1	-	7528192	7528143
Sma-1-12546	NW_003037812.1	+	1492642	1492691
Sma-1-12547	NW_003037822.1	+	1027831	1027880
Sma-1-12548	NW_003037825.1	-	1844147	1844098
Sma-1-12549	NW_003037863.1	+	1791	1840
Sma-1-12550	NW_003037879.1	-	370595	370546
Sma-1-12551	NW_003037906.1	-	584064	584015
Sma-1-12552	NS_000200.1	+	40975381	40975430
Sma-1-12553	NS_000200.1	+	55335837	55335886
Sma-1-12554	NS_000200.1	+	106760158	106760207
Sma-1-12555	NS_000200.1	-	150891843	150891794
Sma-1-12556	NS_000200.1	-	123372851	123372802
Sma-1-12557	NS_000200.1	-	60973929	60973880
Sma-1-12558	NW_003037937.1	+	4614	4663
Sma-1-12559	NS_000200.1	+	181204523	181204572
Sma-1-12560	NW_003037791.1	+	320321	320370
Sma-1-12561	NS_000200.1	+	382025749	382025798
Sma-1-12562	NW_003033261.1	+	462	511
Sma-1-12563	NW_003037795.1	-	1299655	1299606
Sma-1-12564	NW_003037797.1	-	1456150	1456101
Sma-1-12565	NW_003037846.1	-	717531	717482
Sma-1-12566	NW_003037911.1	+	817411	817460
Sma-1-12567	NW_003037981.1	-	187697	187648
Sma-1-12568	NS_000200.1	+	156604764	156604813
Sma-1-12569	NS_000200.1	+	346454891	346454940
Sma-1-12570	NS_000200.1	-	212585565	212585516
Sma-1-12571	NS_000200.1	-	88352101	88352052
Sma-1-12572	NS_000200.1	-	11884010	11883961
Sma-1-12573	NS_000200.1	-	7690538	7690489
Sma-1-12574	NW_003037069.1	+	1668	1717
Sma-1-12575	NW_003038078.1	+	15815	15864
Sma-1-12576	NS_000200.1	+	267222018	267222067
Sma-1-12577	NS_000200.1	+	343778301	343778350
Sma-1-12578	NW_003038020.1	+	40070	40119
Sma-1-12579	NS_000200.1	+	234007414	234007463
Sma-1-12580	NW_003033935.1	-	1118	1069
Sma-1-12581	NW_003037972.1	+	233615	233664
Sma-1-12582	NS_000200.1	+	202709319	202709368
Sma-1-12583	NS_000200.1	-	363264217	363264168
Sma-1-12584	NW_003037794.1	-	1230672	1230623

Sma-1-12585	NW_003037939.1	+	453578	453627
Sma-1-12586	NW_003038077.1	+	369574	369623
Sma-1-12587	NS_000200.1	+	183323332	183323381
Sma-1-12588	NS_000200.1	+	267141214	267141263
Sma-1-12589	NS_000200.1	-	5410267	5410218
Sma-1-12590	NW_003028448.1	+	1718	1767
Sma-1-12591	NS_000200.1	+	369661364	369661413
Sma-1-12592	NW_003037794.1	-	671905	671856
Sma-1-12593	NS_000200.1	-	4851500	4851451
Sma-1-12594	NW_003037814.1	-	722374	722325
Sma-1-12595	NS_000200.1	-	43706234	43706185
Sma-1-12596	NW_003037827.1	+	1152293	1152342
Sma-1-12597	NS_000200.1	+	63774434	63774483
Sma-1-12598	NW_003037817.1	+	869467	869516
Sma-1-12599	NW_003037831.1	-	1731318	1731269
Sma-1-12600	NW_003037849.1	-	263508	263459
Sma-1-12601	NS_000200.1	+	48251819	48251868
Sma-1-12602	NS_000200.1	-	91239781	91239732
Sma-1-12603	NS_000200.1	-	71180620	71180571
Sma-1-12604	NW_003037816.1	-	700235	700186
Sma-1-12605	NS_000200.1	-	46909107	46909058
Sma-1-12606	NW_003037814.1	+	1561734	1561783
Sma-1-12607	NS_000200.1	+	44545594	44545643
Sma-1-12608	NW_003037916.1	+	1597366	1597415
Sma-1-12609	NS_000200.1	+	164790777	164790826
Sma-1-12610	NW_003038070.1	-	422891	422842
Sma-1-12611	NS_000200.1	-	264760132	264760083
Sma-1-12612	NW_003038029.1	+	528116	528165
Sma-1-12613	NS_000200.1	+	242729017	242729066
Sma-1-12614	NW_003037809.1	+	951479	951528
Sma-1-12615	NW_003037910.1	+	1016536	1016585
Sma-1-12616	NS_000200.1	+	34615943	34615992
Sma-1-12617	NS_000200.1	+	155528511	155528560
Sma-1-12618	NW_003026458.1	+	631364	631413
Sma-1-12619	NS_000200.1	+	102499594	102499643
Sma-1-12620	NW_003037978.1	+	32323	32372
Sma-1-12621	NS_000200.1	+	209300333	209300382
Sma-1-12622	NW_003037794.1	-	2149953	2149904
Sma-1-12623	NS_000200.1	-	6329548	6329499
Sma-1-12624	NW_003037809.1	-	853536	853487
Sma-1-12625	NW_003037910.1	+	430287	430336
Sma-1-12626	NW_003038260.1	+	301048	301097
Sma-1-12627	NS_000200.1	+	154942262	154942311
Sma-1-12628	NS_000200.1	+	323472823	323472872
Sma-1-12629	NS_000200.1	-	34518000	34517951
Sma-1-12630	NW_003037918.1	+	859253	859302
Sma-1-12631	NS_000200.1	+	167426243	167426292
Sma-1-12632	NW_003037801.1	+	682539	682588
Sma-1-12633	NW_003037905.1	+	507666	507715
Sma-1-12634	NS_000200.1	+	21772019	21772068
Sma-1-12635	NS_000200.1	+	149776678	149776727
Sma-1-12636	NW_003038029.1	+	839715	839764

Sma-1-12637	NS_000200.1	+	243040616	243040665
Sma-1-12638	NW_003037851.1	+	11513	11562
Sma-1-12639	NW_003037866.1	+	528542	528591
Sma-1-12640	NS_000200.1	+	93414823	93414872
Sma-1-12641	NS_000200.1	+	112016341	112016390
Sma-1-12642	NW_003026465.1	+	185105	185154
Sma-1-12643	NS_000200.1	+	207577699	207577748
Sma-1-12644	NW_003038067.1	-	116814	116765
Sma-1-12645	NS_000200.1	-	262970867	262970818
Sma-1-12646	NW_003037793.1	+	992663	992712
Sma-1-12647	NW_003037838.1	+	725340	725389
Sma-1-12648	NW_003037907.1	+	130785	130834
Sma-1-12649	NW_003037910.1	-	970840	970791
Sma-1-12650	NS_000200.1	+	992663	992712
Sma-1-12651	NS_000200.1	+	78723903	78723952
Sma-1-12652	NS_000200.1	+	151535274	151535323
Sma-1-12653	NS_000200.1	-	155482815	155482766
Sma-1-12654	NW_003035639.1	-	38862	38813
Sma-1-12655	NS_000200.1	-	329989302	329989253
Sma-1-12656	NW_003037978.1	-	504069	504020
Sma-1-12657	NS_000200.1	-	209772079	209772030
Sma-1-12658	NW_003038505.1	+	234354	234403
Sma-1-12659	NS_000200.1	+	336313691	336313740
Sma-1-12660	NW_003022201.1	-	496	447
Sma-1-12661	NW_003029097.1	-	470	421
Sma-1-12662	NW_003033906.1	+	156	205
Sma-1-12663	NW_003037873.1	-	549766	549717
Sma-1-12664	NW_003026599.1	-	115042	114993
Sma-1-12665	NW_003038242.1	-	57728	57679
Sma-1-12666	NW_003026616.1	-	120640	120591
Sma-1-12667	NW_003037798.1	+	2092616	2092665
Sma-1-12668	NW_003037815.1	-	944508	944459
Sma-1-12669	NW_003037821.1	+	461880	461929
Sma-1-12670	NW_003037822.1	+	946439	946488
Sma-1-12671	NW_003037829.1	+	1751071	1751120
Sma-1-12672	NW_003037834.1	+	279603	279652
Sma-1-12673	NW_003037846.1	+	475774	475823
Sma-1-12674	NW_003037853.1	-	524167	524118
Sma-1-12675	NW_003037857.1	-	554366	554317
Sma-1-12676	NW_003037858.1	+	461503	461552
Sma-1-12677	NW_003037863.1	+	351919	351968
Sma-1-12678	NW_003037863.1	-	351397	351348
Sma-1-12679	NW_003037865.1	-	982053	982004
Sma-1-12680	NW_003037877.1	+	179608	179657
Sma-1-12681	NW_003037882.1	+	260147	260196
Sma-1-12682	NW_003037887.1	+	1589703	1589752
Sma-1-12683	NW_003037889.1	-	1735743	1735694
Sma-1-12684	NW_003037895.1	+	246236	246285
Sma-1-12685	NW_003037896.1	-	341663	341614
Sma-1-12686	NW_003037901.1	+	782018	782067
Sma-1-12687	NW_003037942.1	-	863200	863151
Sma-1-12688	NW_003037964.1	-	61297	61248

Sma-1-12689	NW_003037972.1	+	533255	533304
Sma-1-12690	NW_003038031.1	-	35114	35065
Sma-1-12691	NW_003038072.1	-	87891	87842
Sma-1-12692	NW_003038199.1	+	47077	47126
Sma-1-12693	NW_003038255.1	+	193925	193974
Sma-1-12694	NW_003039127.1	+	1160017	1160066
Sma-1-12695	NS_000200.1	+	16351774	16351823
Sma-1-12696	NS_000200.1	+	53606364	53606413
Sma-1-12697	NS_000200.1	+	55254445	55254494
Sma-1-12698	NS_000200.1	+	67651609	67651658
Sma-1-12699	NS_000200.1	+	73440089	73440138
Sma-1-12700	NS_000200.1	+	88110344	88110393
Sma-1-12701	NS_000200.1	+	101125779	101125828
Sma-1-12702	NS_000200.1	+	107110286	107110335
Sma-1-12703	NS_000200.1	+	114237516	114237565
Sma-1-12704	NS_000200.1	+	121218170	121218219
Sma-1-12705	NS_000200.1	+	125551821	125551870
Sma-1-12706	NS_000200.1	+	132053681	132053730
Sma-1-12707	NS_000200.1	+	142692438	142692487
Sma-1-12708	NS_000200.1	+	146630479	146630528
Sma-1-12709	NS_000200.1	+	203008959	203009008
Sma-1-12710	NS_000200.1	+	303566715	303566764
Sma-1-12711	NS_000200.1	+	320583070	320583119
Sma-1-12712	NS_000200.1	+	359448551	359448600
Sma-1-12713	NS_000200.1	-	375374393	375374344
Sma-1-12714	NS_000200.1	-	360920098	360920049
Sma-1-12715	NS_000200.1	-	317477239	317477190
Sma-1-12716	NS_000200.1	-	314756874	314756825
Sma-1-12717	NS_000200.1	-	305993371	305993322
Sma-1-12718	NS_000200.1	-	265250119	265250070
Sma-1-12719	NS_000200.1	-	244312872	244312823
Sma-1-12720	NS_000200.1	-	197662819	197662770
Sma-1-12721	NS_000200.1	-	186623108	186623059
Sma-1-12722	NS_000200.1	-	143262086	143262037
Sma-1-12723	NS_000200.1	-	135512233	135512184
Sma-1-12724	NS_000200.1	-	119288894	119288845
Sma-1-12725	NS_000200.1	-	111403972	111403923
Sma-1-12726	NS_000200.1	-	107109764	107109715
Sma-1-12727	NS_000200.1	-	100574723	100574674
Sma-1-12728	NS_000200.1	-	96016218	96016169
Sma-1-12729	NS_000200.1	-	46207499	46207450
Sma-1-12730	NW_003038029.1	+	504501	504550
Sma-1-12731	NS_000200.1	+	242705402	242705451
Sma-1-12732	NW_003037803.1	+	1327109	1327158
Sma-1-12733	NW_003037810.1	-	634738	634689
Sma-1-12734	NW_003038056.1	-	110393	110344
Sma-1-12735	NS_000200.1	+	24827646	24827695
Sma-1-12736	NS_000200.1	-	259088044	259087995
Sma-1-12737	NS_000200.1	-	37239260	37239211
Sma-1-12738	NW_003037939.1	+	827482	827531
Sma-1-12739	NS_000200.1	+	183697236	183697285
Sma-1-12740	NW_003037788.1	+	161985	162034

Sma-1-12741	NW_003037821.1	+	346555	346604
Sma-1-12742	NS_000200.1	+	53491039	53491088
Sma-1-12743	NS_000200.1	+	380609223	380609272
Sma-1-12744	NW_003037809.1	-	2497976	2497927
Sma-1-12745	NS_000200.1	-	36162440	36162391
Sma-1-12746	NW_003038131.1	-	101126	101077
Sma-1-12747	NS_000200.1	-	282062970	282062921
Sma-1-12748	NW_003037903.1	-	30175	30126
Sma-1-12749	NS_000200.1	-	147584256	147584207
Sma-1-12750	NW_003026618.1	+	50163	50212
Sma-1-12751	NS_000200.1	+	321805165	321805214
Sma-1-12752	NW_003038147.1	+	508056	508105
Sma-1-12753	NS_000200.1	+	290035799	290035848
Sma-1-12754	NW_003038256.1	-	113959	113910
Sma-1-12755	NW_003037818.1	+	664908	664957
Sma-1-12756	NW_003037981.1	-	1027942	1027893
Sma-1-12757	NS_000200.1	+	49380315	49380364
Sma-1-12758	NS_000200.1	-	320847785	320847736
Sma-1-12759	NS_000200.1	-	213425810	213425761
Sma-1-12760	NW_003023978.1	+	585	634
Sma-1-12761	NW_003027562.1	-	484	435
Sma-1-12762	NW_003033810.1	-	2098	2049
Sma-1-12763	NW_003037951.1	+	84148	84197
Sma-1-12764	NW_003037788.1	-	264273	264224
Sma-1-12765	NW_003037790.1	-	231061	231012
Sma-1-12766	NW_003026615.1	+	114889	114938
Sma-1-12767	NW_003026616.1	+	128714	128763
Sma-1-12768	NW_003037797.1	+	2503011	2503060
Sma-1-12769	NW_003037801.1	+	687825	687874
Sma-1-12770	NW_003037822.1	+	655294	655343
Sma-1-12771	NW_003037909.1	-	832283	832234
Sma-1-12772	NW_003037914.1	-	1199163	1199114
Sma-1-12773	NW_003037916.1	+	690830	690879
Sma-1-12774	NW_003037943.1	+	1167087	1167136
Sma-1-12775	NW_003037996.1	+	325569	325618
Sma-1-12776	NW_003038037.1	+	1676865	1676914
Sma-1-12777	NW_003039127.1	+	313569	313618
Sma-1-12778	NW_003039127.1	+	827994	828043
Sma-1-12779	NW_003038149.1	+	392947	392996
Sma-1-12780	NS_000200.1	+	12930871	12930920
Sma-1-12781	NS_000200.1	+	21777305	21777354
Sma-1-12782	NS_000200.1	+	54963300	54963349
Sma-1-12783	NS_000200.1	+	113391068	113391117
Sma-1-12784	NS_000200.1	+	113905493	113905542
Sma-1-12785	NS_000200.1	+	163884241	163884290
Sma-1-12786	NS_000200.1	+	188188620	188188669
Sma-1-12787	NS_000200.1	+	192761873	192761922
Sma-1-12788	NS_000200.1	+	220570445	220570494
Sma-1-12789	NS_000200.1	+	251832274	251832323
Sma-1-12790	NS_000200.1	+	291421560	291421609
Sma-1-12791	NS_000200.1	+	317109330	317109379
Sma-1-12792	NS_000200.1	+	317485313	317485362

Sma-1-12793	NS_000200.1	+	344357602	344357651
Sma-1-12794	NS_000200.1	-	381575233	381575184
Sma-1-12795	NS_000200.1	-	380711511	380711462
Sma-1-12796	NS_000200.1	-	361625963	361625914
Sma-1-12797	NS_000200.1	-	359089223	359089174
Sma-1-12798	NS_000200.1	-	161343413	161343364
Sma-1-12799	NS_000200.1	-	154373932	154373883
Sma-1-12800	NW_003037793.1	-	2828745	2828696
Sma-1-12801	NW_003037798.1	-	105212	105163
Sma-1-12802	NW_003037841.1	-	1993094	1993045
Sma-1-12803	NS_000200.1	-	84604275	84604226
Sma-1-12804	NS_000200.1	-	14364370	14364321
Sma-1-12805	NS_000200.1	-	2828745	2828696
Sma-1-12806	NW_003037809.1	-	2241066	2241017
Sma-1-12807	NW_003037911.1	-	409704	409655
Sma-1-12808	NW_003037918.1	+	1880642	1880691
Sma-1-12809	NS_000200.1	+	168447632	168447681
Sma-1-12810	NS_000200.1	-	156197057	156197008
Sma-1-12811	NS_000200.1	-	35905530	35905481
Sma-1-12812	NW_003037825.1	-	926171	926122
Sma-1-12813	NS_000200.1	-	60055953	60055904
Sma-1-12814	NW_003037909.1	+	512043	512092
Sma-1-12815	NS_000200.1	+	154053692	154053741
Sma-1-12816	NW_003037810.1	+	40676	40725
Sma-1-12817	NS_000200.1	+	36645198	36645247
Sma-1-12818	NW_003037814.1	+	1330130	1330179
Sma-1-12819	NS_000200.1	+	44313990	44314039
Sma-1-12820	NW_003038129.1	-	33598	33549
Sma-1-12821	NS_000200.1	-	281286945	281286896
Sma-1-12822	NW_003037827.1	+	368457	368506
Sma-1-12823	NS_000200.1	+	62990598	62990647
Sma-1-12824	NW_003026465.1	-	410570	410521
Sma-1-12825	NS_000200.1	-	207803164	207803115
Sma-1-12826	NW_003037795.1	-	278446	278397
Sma-1-12827	NS_000200.1	-	6669329	6669280
Sma-1-12828	NW_003037945.1	+	252613	252662
Sma-1-12829	NS_000200.1	+	189354577	189354626
Sma-1-12830	NW_003037829.1	+	1655963	1656012
Sma-1-12831	NS_000200.1	+	67556501	67556550
Sma-1-12832	NW_003037967.1	+	72414	72463
Sma-1-12833	NS_000200.1	+	199312385	199312434
Sma-1-12834	NW_003030121.1	+	34	83
Sma-1-12835	NW_003037841.1	+	182718	182767
Sma-1-12836	NW_003037939.1	-	334866	334817
Sma-1-12837	NS_000200.1	+	82793899	82793948
Sma-1-12838	NS_000200.1	+	357636263	357636312
Sma-1-12839	NS_000200.1	-	183204620	183204571
Sma-1-12840	NW_003038069.1	+	133426	133475
Sma-1-12841	NS_000200.1	+	263976832	263976881
Sma-1-12842	NW_003038163.1	-	623184	623135
Sma-1-12843	NS_000200.1	-	299023464	299023415
Sma-1-12844	NW_003037814.1	-	996896	996847

Sma-1-12845	NS_000200.1	-	43980756	43980707
Sma-1-12846	NW_003037803.1	+	1011696	1011745
Sma-1-12847	NS_000200.1	+	24512233	24512282
Sma-1-12848	NW_003037846.1	-	178122	178073
Sma-1-12849	NW_003026516.1	-	278437	278388
Sma-1-12850	NS_000200.1	-	299386734	299386685
Sma-1-12851	NS_000200.1	-	87812692	87812643
Sma-1-12852	NW_003037808.1	+	1047395	1047444
Sma-1-12853	NS_000200.1	+	32834032	32834081
Sma-1-12854	NW_003038068.1	+	204414	204463
Sma-1-12855	NS_000200.1	+	263271622	263271671
Sma-1-12856	NW_003037846.1	-	71912	71863
Sma-1-12857	NW_003037913.1	+	1506270	1506319
Sma-1-12858	NS_000200.1	+	160070021	160070070
Sma-1-12859	NS_000200.1	-	87706482	87706433
Sma-1-12860	NW_003037810.1	-	1430374	1430325
Sma-1-12861	NS_000200.1	-	38034896	38034847
Sma-1-12862	NW_003037932.1	+	739746	739795
Sma-1-12863	NS_000200.1	+	178272942	178272991
Sma-1-12864	NW_003037841.1	+	172737	172786
Sma-1-12865	NW_003038132.1	+	162479	162528
Sma-1-12866	NS_000200.1	+	82783918	82783967
Sma-1-12867	NS_000200.1	+	282582949	282582998
Sma-1-12868	NW_003037795.1	-	150467	150418
Sma-1-12869	NS_000200.1	-	6541350	6541301
Sma-1-12870	NW_003037971.1	-	594021	593972
Sma-1-12871	NS_000200.1	-	202216071	202216022
Sma-1-12872	NW_003038025.1	+	582940	582989
Sma-1-12873	NS_000200.1	+	239441185	239441234
Sma-1-12874	NW_003038214.1	+	5993	6042
Sma-1-12875	NW_003037793.1	-	2910049	2910000
Sma-1-12876	NS_000200.1	+	306545629	306545678
Sma-1-12877	NS_000200.1	-	2910049	2910000
Sma-1-12878	NW_003038025.1	-	271808	271759
Sma-1-12879	NS_000200.1	-	239130053	239130004
Sma-1-12880	NW_003038632.1	-	1565	1516
Sma-1-12881	NW_003037807.1	+	676623	676672
Sma-1-12882	NW_003037926.1	+	90237	90286
Sma-1-12883	NW_003038025.1	+	588414	588463
Sma-1-12884	NW_003038025.1	+	604348	604397
Sma-1-12885	NW_003026515.1	+	292757	292806
Sma-1-12886	NS_000200.1	+	30938139	30938188
Sma-1-12887	NS_000200.1	+	173258507	173258556
Sma-1-12888	NS_000200.1	+	239446659	239446708
Sma-1-12889	NS_000200.1	+	239462593	239462642
Sma-1-12890	NS_000200.1	+	290937168	290937217
Sma-1-12891	NS_000200.1	-	345742824	345742775
Sma-1-12892	NW_003037813.1	-	1208139	1208090
Sma-1-12893	NS_000200.1	-	42195002	42194953
Sma-1-12894	NW_003037810.1	+	70791	70840
Sma-1-12895	NS_000200.1	+	36675313	36675362
Sma-1-12896	NW_003037809.1	-	153712	153663

Sma-1-12897	NS_000200.1	-	33818176	33818127
Sma-1-12898	NW_003026609.1	+	78571	78620
Sma-1-12899	NS_000200.1	+	309733563	309733612
Sma-1-12900	NW_003037890.1	-	582066	582017
Sma-1-12901	NS_000200.1	-	136265427	136265378
Sma-1-12902	NW_003038064.1	+	277945	277994
Sma-1-12903	NS_000200.1	+	262114522	262114571
Sma-1-12904	NW_003038249.1	+	157090	157139
Sma-1-12905	NS_000200.1	+	316839343	316839392
Sma-1-12906	NW_003037818.1	-	250239	250190
Sma-1-12907	NS_000200.1	-	48965646	48965597
Sma-1-12908	NW_003026472.1	-	610882	610833
Sma-1-12909	NW_003037890.1	-	1437786	1437737
Sma-1-12910	NW_003037988.1	-	98010	97961
Sma-1-12911	NW_003038072.1	-	246632	246583
Sma-1-12912	NS_000200.1	-	265408860	265408811
Sma-1-12913	NS_000200.1	-	237022874	237022825
Sma-1-12914	NS_000200.1	-	216091828	216091779
Sma-1-12915	NS_000200.1	-	137121147	137121098
Sma-1-12916	NW_003038243.1	-	163800	163751
Sma-1-12917	NS_000200.1	-	315102284	315102235
Sma-1-12918	NW_003037786.1	+	78783	78832
Sma-1-12919	NW_003037841.1	+	1569182	1569231
Sma-1-12920	NW_003038137.1	-	196346	196297
Sma-1-12921	NS_000200.1	+	84180363	84180412
Sma-1-12922	NS_000200.1	+	380172143	380172192
Sma-1-12923	NS_000200.1	-	284985242	284985193
Sma-1-12924	NW_003032921.1	-	1031	982
Sma-1-12925	NW_003034577.1	+	1219	1268
Sma-1-12926	NS_000200.1	+	327708892	327708941
Sma-1-12927	NS_000200.1	-	325984269	325984220
Sma-1-12928	NW_003026619.1	-	299630	299581
Sma-1-12929	NW_003037863.1	-	1036402	1036353
Sma-1-12930	NW_003037964.1	-	340214	340165
Sma-1-12931	NS_000200.1	-	322490374	322490325
Sma-1-12932	NS_000200.1	-	197941736	197941687
Sma-1-12933	NS_000200.1	-	107794769	107794720
Sma-1-12934	NW_003037970.1	-	520291	520242
Sma-1-12935	NS_000200.1	-	201576481	201576432
Sma-1-12936	NW_003037973.1	-	514776	514727
Sma-1-12937	NS_000200.1	-	204496503	204496454
Sma-1-12938	NW_003037974.1	+	869655	869704
Sma-1-12939	NS_000200.1	+	205544961	205545010
Sma-1-12940	NW_003038129.1	+	187455	187504
Sma-1-12941	NS_000200.1	+	281440802	281440851
Sma-1-12942	NW_003024476.1	+	59	108
Sma-1-12943	NW_003037915.1	+	146540	146589
Sma-1-12944	NW_003038024.1	-	582815	582766
Sma-1-12945	NS_000200.1	+	162046526	162046575
Sma-1-12946	NS_000200.1	+	365807455	365807504
Sma-1-12947	NS_000200.1	-	238479844	238479795
Sma-1-12948	NW_003038210.1	-	59182	59133

Sma-1-12949	NS_000200.1	-	305521081	305521032
Sma-1-12950	NW_003037856.1	+	596856	596905
Sma-1-12951	NS_000200.1	+	99954812	99954861
Sma-1-12952	NW_003038003.1	-	361586	361537
Sma-1-12953	NS_000200.1	-	223860868	223860819
Sma-1-12954	NW_003038005.1	+	161255	161304
Sma-1-12955	NW_003037908.1	-	849576	849527
Sma-1-12956	NW_003038163.1	+	278616	278665
Sma-1-12957	NS_000200.1	+	224567209	224567258
Sma-1-12958	NS_000200.1	+	298678896	298678945
Sma-1-12959	NS_000200.1	-	153322707	153322658
Sma-1-12960	NW_003038027.1	-	327005	326956
Sma-1-12961	NS_000200.1	-	240697719	240697670
Sma-1-12962	NW_003038120.1	+	175389	175438
Sma-1-12963	NS_000200.1	+	278360612	278360661
Sma-1-12964	NW_003037821.1	-	236628	236579
Sma-1-12965	NS_000200.1	-	53381112	53381063
Sma-1-12966	NW_003029425.1	+	862	911
Sma-1-12967	NW_003038070.1	-	249726	249677
Sma-1-12968	NW_003037815.1	-	785372	785323
Sma-1-12969	NW_003037833.1	+	555536	555585
Sma-1-12970	NW_003037848.1	-	549244	549195
Sma-1-12971	NW_003037925.1	-	615197	615148
Sma-1-12972	NW_003037945.1	+	554921	554970
Sma-1-12973	NW_003038064.1	-	93775	93726
Sma-1-12974	NS_000200.1	+	72746632	72746681
Sma-1-12975	NS_000200.1	+	189656885	189656934
Sma-1-12976	NS_000200.1	+	362039901	362039950
Sma-1-12977	NS_000200.1	-	264586967	264586918
Sma-1-12978	NS_000200.1	-	261930352	261930303
Sma-1-12979	NS_000200.1	-	173017200	173017151
Sma-1-12980	NS_000200.1	-	90242315	90242266
Sma-1-12981	NS_000200.1	-	46048363	46048314
Sma-1-12982	NW_003020614.1	-	624	575
Sma-1-12983	NW_003037878.1	+	187906	187955
Sma-1-12984	NS_000200.1	+	122241384	122241433
Sma-1-12985	NS_000200.1	-	360433329	360433280
Sma-1-12986	NW_003038457.1	+	21224	21273
Sma-1-12987	NW_003037818.1	+	1316358	1316407
Sma-1-12988	NW_003037988.1	-	107151	107102
Sma-1-12989	NW_003038025.1	-	333406	333357
Sma-1-12990	NS_000200.1	+	50031765	50031814
Sma-1-12991	NS_000200.1	+	331103088	331103137
Sma-1-12992	NS_000200.1	-	239191651	239191602
Sma-1-12993	NS_000200.1	-	216100969	216100920
Sma-1-12994	NW_003038256.1	-	167278	167229
Sma-1-12995	NS_000200.1	-	320901104	320901055
Sma-1-12996	NW_003038511.1	-	183193	183144
Sma-1-12997	NS_000200.1	-	338528405	338528356
Sma-1-12998	NW_003038064.1	+	92516	92565
Sma-1-12999	NS_000200.1	+	261929093	261929142
Sma-1-13000	NW_003038043.1	+	133032	133081

Sma-1-13001	NS_000200.1	+	254443074	254443123
Sma-1-13002	NW_003038232.1	-	338884	338835
Sma-1-13003	NS_000200.1	-	312302675	312302626
Sma-1-13004	NW_003038028.1	+	654491	654540
Sma-1-13005	NS_000200.1	+	241962065	241962114
Sma-1-13006	NW_003035682.1	+	13005	13054
Sma-1-13007	NW_003026617.1	+	51872	51921
Sma-1-13008	NW_003035879.1	+	252645	252694
Sma-1-13009	NW_003037800.1	+	1353334	1353383
Sma-1-13010	NW_003037809.1	-	584745	584696
Sma-1-13011	NW_003037833.1	+	586301	586350
Sma-1-13012	NW_003037848.1	-	135857	135808
Sma-1-13013	NW_003037901.1	+	312254	312303
Sma-1-13014	NW_003037918.1	-	113224	113175
Sma-1-13015	NW_003037954.1	-	18269	18220
Sma-1-13016	NW_003038027.1	-	817900	817851
Sma-1-13017	NW_003038032.1	-	162037	161988
Sma-1-13018	NW_003038453.1	+	95114	95163
Sma-1-13019	NW_003038042.1	-	184919	184870
Sma-1-13020	NW_003038124.1	-	161402	161353
Sma-1-13021	NW_003038143.1	-	409736	409687
Sma-1-13022	NW_003038145.1	+	124797	124846
Sma-1-13023	NW_003038151.1	+	133116	133165
Sma-1-13024	NW_003038155.1	-	381630	381581
Sma-1-13025	NS_000200.1	+	19982694	19982743
Sma-1-13026	NS_000200.1	+	72777397	72777446
Sma-1-13027	NS_000200.1	+	146160715	146160764
Sma-1-13028	NS_000200.1	+	288736220	288736269
Sma-1-13029	NS_000200.1	+	292040359	292040408
Sma-1-13030	NS_000200.1	+	319622731	319622780
Sma-1-13031	NS_000200.1	+	330677041	330677090
Sma-1-13032	NS_000200.1	+	332635058	332635107
Sma-1-13033	NS_000200.1	+	338219972	338220021
Sma-1-13034	NS_000200.1	-	294504748	294504699
Sma-1-13035	NS_000200.1	-	288169228	288169179
Sma-1-13036	NS_000200.1	-	279154046	279153997
Sma-1-13037	NS_000200.1	-	254130518	254130469
Sma-1-13038	NS_000200.1	-	245321197	245321148
Sma-1-13039	NS_000200.1	-	241188614	241188565
Sma-1-13040	NS_000200.1	-	193247891	193247842
Sma-1-13041	NS_000200.1	-	166680214	166680165
Sma-1-13042	NS_000200.1	-	89828928	89828879
Sma-1-13043	NS_000200.1	-	34249209	34249160
Sma-1-13044	NW_003037889.1	-	892432	892384
Sma-1-13045	NS_000200.1	-	134668922	134668874
Sma-1-13046	NW_003037836.1	+	1118921	1118970
Sma-1-13047	NW_003037840.1	-	1509871	1509822
Sma-1-13048	NW_003038020.1	+	698683	698732
Sma-1-13049	NW_003038033.1	-	248390	248341
Sma-1-13050	NW_003026510.1	+	48972	49021
Sma-1-13051	NS_000200.1	+	76676020	76676069
Sma-1-13052	NS_000200.1	+	234666027	234666076

Sma-1-13053	NS_000200.1	+	283489153	283489202
Sma-1-13054	NS_000200.1	-	246228392	246228343
Sma-1-13055	NS_000200.1	-	82133195	82133146
Sma-1-13056	NW_003038250.1	+	278672	278721
Sma-1-13057	NW_003038152.1	+	317901	317950
Sma-1-13058	NS_000200.1	+	292923625	292923674
Sma-1-13059	NS_000200.1	+	318331349	318331398
Sma-1-13060	NW_003030738.1	+	775	824
Sma-1-13061	NS_000200.1	+	366824476	366824525
Sma-1-13062	NW_003037846.1	-	93020	92971
Sma-1-13063	NS_000200.1	-	87727590	87727541
Sma-1-13064	NW_003037869.1	-	119960	119911
Sma-1-13065	NS_000200.1	-	116592305	116592256
Sma-1-13066	NW_003037891.1	-	170064	170015
Sma-1-13067	NS_000200.1	-	138051342	138051293
Sma-1-13068	NW_003037841.1	+	1780688	1780737
Sma-1-13069	NS_000200.1	+	84391869	84391918
Sma-1-13070	NW_003037916.1	+	1728916	1728965
Sma-1-13071	NW_003038037.1	-	929450	929401
Sma-1-13072	NS_000200.1	+	164922327	164922376
Sma-1-13073	NS_000200.1	-	251084859	251084810
Sma-1-13074	NW_003037936.1	+	390563	390612
Sma-1-13075	NW_003037794.1	+	869614	869663
Sma-1-13076	NS_000200.1	+	5049209	5049258
Sma-1-13077	NS_000200.1	+	180830292	180830341
Sma-1-13078	NW_003037941.1	+	898629	898678
Sma-1-13079	NW_003037972.1	+	622226	622275
Sma-1-13080	NW_003037976.1	-	165808	165759
Sma-1-13081	NS_000200.1	+	185633103	185633152
Sma-1-13082	NS_000200.1	+	203097930	203097979
Sma-1-13083	NS_000200.1	-	206780123	206780074
Sma-1-13084	NW_003038244.1	+	407106	407155
Sma-1-13085	NS_000200.1	+	315552311	315552360
Sma-1-13086	NW_003038032.1	+	181242	181291
Sma-1-13087	NS_000200.1	+	245340402	245340451
Sma-1-13088	NW_003037923.1	+	278716	278765
Sma-1-13089	NS_000200.1	+	171334929	171334978
Sma-1-13090	NW_003037800.1	+	1610531	1610580
Sma-1-13091	NS_000200.1	+	20239891	20239940
Sma-1-13092	NW_003037937.1	+	832490	832539
Sma-1-13093	NS_000200.1	+	182032399	182032448
Sma-1-13094	NW_003037970.1	-	416255	416206
Sma-1-13095	NS_000200.1	-	201472445	201472396
Sma-1-13096	NW_003037376.1	+	438	487
Sma-1-13097	NW_003032160.1	-	210566	210517
Sma-1-13098	NW_003037866.1	-	1499327	1499278
Sma-1-13099	NW_003037871.1	+	432111	432160
Sma-1-13100	NW_003037912.1	+	1036693	1036742
Sma-1-13101	NW_003037918.1	-	2190875	2190826
Sma-1-13102	NW_003038037.1	-	1665753	1665704
Sma-1-13103	NW_003038517.1	-	77659	77610
Sma-1-13104	NW_003026512.1	+	133119	133168

Sma-1-13105	NS_000200.1	+	117879849	117879898
Sma-1-13106	NS_000200.1	+	157848268	157848317
Sma-1-13107	NS_000200.1	+	285227159	285227208
Sma-1-13108	NS_000200.1	+	367306665	367306714
Sma-1-13109	NS_000200.1	-	381218652	381218603
Sma-1-13110	NS_000200.1	-	340842147	340842098
Sma-1-13111	NS_000200.1	-	251821162	251821113
Sma-1-13112	NS_000200.1	-	168757865	168757816
Sma-1-13113	NS_000200.1	-	112987126	112987077
Sma-1-13114	NW_003037796.1	+	505313	505362
Sma-1-13115	NS_000200.1	+	8714957	8715006
Sma-1-13116	NW_003037854.1	-	1840436	1840387
Sma-1-13117	NS_000200.1	-	98744857	98744808
Sma-1-13118	NW_003037795.1	-	625684	625635
Sma-1-13119	NS_000200.1	-	7016567	7016518
Sma-1-13120	NW_003037961.1	+	338128	338177
Sma-1-13121	NS_000200.1	+	195962370	195962419
Sma-1-13122	NW_003037809.1	-	55198	55149
Sma-1-13123	NS_000200.1	-	33719662	33719613
Sma-1-13124	NW_003037881.1	+	462157	462206
Sma-1-13125	NW_003037977.1	+	654252	654301
Sma-1-13126	NW_003038153.1	+	577473	577522
Sma-1-13127	NS_000200.1	+	124893948	124893997
Sma-1-13128	NS_000200.1	+	208940272	208940321
Sma-1-13129	NS_000200.1	+	293639522	293639571
Sma-1-13130	NW_003037879.1	+	175817	175866
Sma-1-13131	NS_000200.1	+	123178073	123178122
Sma-1-13132	NW_003037882.1	-	536259	536210
Sma-1-13133	NS_000200.1	-	125827933	125827884
Sma-1-13134	NW_003037878.1	-	504818	504769
Sma-1-13135	NS_000200.1	-	122558296	122558247
Sma-1-13136	NW_003037840.1	-	1291967	1291918
Sma-1-13137	NS_000200.1	-	81915291	81915242
Sma-1-13138	NW_003037796.1	+	555074	555123
Sma-1-13139	NS_000200.1	+	8764718	8764767
Sma-1-13140	NW_003021447.1	-	443	394
Sma-1-13141	NW_003026612.1	+	235826	235875
Sma-1-13142	NW_003037823.1	-	873300	873251
Sma-1-13143	NS_000200.1	+	312541213	312541262
Sma-1-13144	NS_000200.1	-	344085727	344085678
Sma-1-13145	NS_000200.1	-	56789919	56789870
Sma-1-13146	NW_003037810.1	+	1137663	1137712
Sma-1-13147	NW_003037839.1	+	1271051	1271100
Sma-1-13148	NW_003038036.1	-	229133	229084
Sma-1-13149	NS_000200.1	+	37742185	37742234
Sma-1-13150	NS_000200.1	+	80229201	80229250
Sma-1-13151	NS_000200.1	-	249162985	249162936
Sma-1-13152	NW_003037818.1	+	1021453	1021502
Sma-1-13153	NW_003038153.1	+	678506	678555
Sma-1-13154	NS_000200.1	+	49736860	49736909
Sma-1-13155	NS_000200.1	+	293740555	293740604
Sma-1-13156	NW_003037877.1	+	675254	675303

Sma-1-13157	NS_000200.1	+	121713816	121713865
Sma-1-13158	NW_003037884.1	-	1123674	1123625
Sma-1-13159	NS_000200.1	-	127903339	127903290
Sma-1-13160	NW_003037875.1	-	399239	399190
Sma-1-13161	NS_000200.1	-	120427908	120427859
Sma-1-13162	NW_003037884.1	-	4141	4092
Sma-1-13163	NS_000200.1	-	126783806	126783757
Sma-1-13164	NW_003037884.1	-	923851	923802
Sma-1-13165	NW_003038014.1	+	291755	291804
Sma-1-13166	NS_000200.1	+	230032444	230032493
Sma-1-13167	NS_000200.1	-	127703516	127703467
Sma-1-13168	NW_003037873.1	-	314019	313970
Sma-1-13169	NS_000200.1	-	119053147	119053098
Sma-1-13170	NW_003037930.1	-	290461	290412
Sma-1-13171	NS_000200.1	-	176148425	176148376
Sma-1-13172	NW_003037840.1	+	1557022	1557071
Sma-1-13173	NW_003038124.1	-	179162	179113
Sma-1-13174	NS_000200.1	+	82180346	82180395
Sma-1-13175	NS_000200.1	-	279171806	279171757
Sma-1-13176	NW_003037840.1	-	1560544	1560495
Sma-1-13177	NS_000200.1	-	82183868	82183819
Sma-1-13178	NW_003037796.1	+	2010388	2010437
Sma-1-13179	NS_000200.1	+	10220032	10220081
Sma-1-13180	NW_003037786.1	-	94551	94502
Sma-1-13181	NW_003037858.1	-	1131052	1131003
Sma-1-13182	NS_000200.1	-	380187911	380187862
Sma-1-13183	NS_000200.1	-	101795328	101795279
Sma-1-13184	NW_003030981.1	+	1663	1712
Sma-1-13185	NW_003038151.1	+	27202	27251
Sma-1-13186	NS_000200.1	+	291934445	291934494
Sma-1-13187	NS_000200.1	+	370656840	370656889
Sma-1-13188	NW_003026517.1	-	233997	233948
Sma-1-13189	NS_000200.1	-	299840006	299839957
Sma-1-13190	NW_003038227.1	-	226903	226854
Sma-1-13191	NS_000200.1	-	310508257	310508208
Sma-1-13192	NW_003029251.1	+	59	108
Sma-1-13193	NS_000200.1	+	357164269	357164318
Sma-1-13194	NW_003038037.1	+	709395	709444
Sma-1-13195	NS_000200.1	+	250864804	250864853
Sma-1-13196	NW_003038210.1	-	78123	78074
Sma-1-13197	NS_000200.1	-	305540022	305539973
Sma-1-13198	NW_003038163.1	-	118879	118830
Sma-1-13199	NS_000200.1	-	298519159	298519110
Sma-1-13200	NW_003037923.1	+	448020	448069
Sma-1-13201	NW_003037971.1	+	453686	453735
Sma-1-13202	NS_000200.1	+	171504233	171504282
Sma-1-13203	NS_000200.1	+	202075736	202075785
Sma-1-13204	NW_003038218.1	-	207367	207318
Sma-1-13205	NS_000200.1	-	307647665	307647616
Sma-1-13206	NW_003038305.1	-	2044	1995
Sma-1-13207	NS_000200.1	-	325748830	325748781
Sma-1-13208	NW_003038037.1	+	531251	531300

Sma-1-13209	NS_000200.1	+	250686660	250686709
Sma-1-13210	NW_003026465.1	+	377562	377611
Sma-1-13211	NW_003037798.1	-	497661	497612
Sma-1-13212	NW_003037806.1	+	236779	236828
Sma-1-13213	NW_003037809.1	+	1365142	1365191
Sma-1-13214	NW_003037817.1	-	698146	698097
Sma-1-13215	NW_003037821.1	-	543829	543780
Sma-1-13216	NW_003037833.1	+	926431	926480
Sma-1-13217	NW_003037847.1	+	1015943	1015992
Sma-1-13218	NW_003037849.1	+	935501	935550
Sma-1-13219	NW_003037891.1	+	2814045	2814094
Sma-1-13220	NW_003037894.1	+	273067	273116
Sma-1-13221	NW_003037905.1	+	735797	735846
Sma-1-13222	NW_003037972.1	+	625786	625835
Sma-1-13223	NW_003038518.1	-	242718	242669
Sma-1-13224	NS_000200.1	+	28431993	28432042
Sma-1-13225	NS_000200.1	+	35029606	35029655
Sma-1-13226	NS_000200.1	+	73117527	73117576
Sma-1-13227	NS_000200.1	+	89667673	89667722
Sma-1-13228	NS_000200.1	+	91911774	91911823
Sma-1-13229	NS_000200.1	+	140695323	140695372
Sma-1-13230	NS_000200.1	+	142169640	142169689
Sma-1-13231	NS_000200.1	+	150004809	150004858
Sma-1-13232	NS_000200.1	+	203101490	203101539
Sma-1-13233	NS_000200.1	+	207770156	207770205
Sma-1-13234	NS_000200.1	-	341879551	341879502
Sma-1-13235	NS_000200.1	-	53688313	53688264
Sma-1-13236	NS_000200.1	-	48080498	48080449
Sma-1-13237	NS_000200.1	-	14756819	14756770
Sma-1-13238	NW_003024030.1	+	418	467
Sma-1-13239	NW_003037800.1	+	2176341	2176390
Sma-1-13240	NW_003037906.1	+	470847	470896
Sma-1-13241	NS_000200.1	+	20805701	20805750
Sma-1-13242	NS_000200.1	+	150778626	150778675
Sma-1-13243	NS_000200.1	+	344488899	344488948
Sma-1-13244	NW_003034025.1	+	3913	3962
Sma-1-13245	NW_003037874.1	-	171009	170960
Sma-1-13246	NW_003037937.1	+	268914	268963
Sma-1-13247	NW_003037816.1	-	856720	856671
Sma-1-13248	NW_003037829.1	-	997891	997842
Sma-1-13249	NW_003037917.1	-	234121	234072
Sma-1-13250	NW_003037922.1	+	73475	73524
Sma-1-13251	NS_000200.1	+	169997472	169997521
Sma-1-13252	NS_000200.1	+	181468823	181468872
Sma-1-13253	NS_000200.1	+	363502710	363502759
Sma-1-13254	NS_000200.1	-	165270925	165270876
Sma-1-13255	NS_000200.1	-	119595959	119595910
Sma-1-13256	NS_000200.1	-	66898429	66898380
Sma-1-13257	NS_000200.1	-	47065592	47065543
Sma-1-13258	NW_003039127.1	+	825682	825731
Sma-1-13259	NS_000200.1	+	113903181	113903230
Sma-1-13260	NW_003037806.1	+	34181	34230

Sma-1-13261	NS_000200.1	+	28229395	28229444
Sma-1-13262	NW_003037966.1	-	290133	290084
Sma-1-13263	NS_000200.1	-	198991996	198991947
Sma-1-13264	NW_003037793.1	+	2746402	2746451
Sma-1-13265	NW_003037866.1	-	1397280	1397231
Sma-1-13266	NW_003038015.1	-	705178	705129
Sma-1-13267	NS_000200.1	+	2746402	2746451
Sma-1-13268	NS_000200.1	-	231028668	231028619
Sma-1-13269	NS_000200.1	-	112885079	112885030
Sma-1-13270	NW_003037885.1	-	527270	527221
Sma-1-13271	NS_000200.1	-	128625217	128625168
Sma-1-13272	NW_003037886.1	-	1042867	1042818
Sma-1-13273	NS_000200.1	-	130298434	130298385
Sma-1-13274	NW_003037868.1	+	360001	360050
Sma-1-13275	NS_000200.1	+	116331713	116331762
Sma-1-13276	NW_003037886.1	+	121004	121053
Sma-1-13277	NS_000200.1	+	129376571	129376620
Sma-1-13278	NW_003037864.1	+	144563	144612
Sma-1-13279	NS_000200.1	+	108637137	108637186
Sma-1-13280	NW_003037887.1	+	1397188	1397237
Sma-1-13281	NS_000200.1	+	131861166	131861215
Sma-1-13282	NW_003037887.1	+	432373	432422
Sma-1-13283	NS_000200.1	+	130896351	130896400
Sma-1-13284	NW_003037838.1	+	517373	517422
Sma-1-13285	NS_000200.1	+	78515936	78515985
Sma-1-13286	NW_003037865.1	+	283314	283363
Sma-1-13287	NW_003037899.1	-	102046	101997
Sma-1-13288	NW_003037978.1	-	250254	250205
Sma-1-13289	NS_000200.1	+	110705233	110705282
Sma-1-13290	NS_000200.1	-	209518264	209518215
Sma-1-13291	NS_000200.1	-	144621157	144621108
Sma-1-13292	NW_003037861.1	-	64115	64066
Sma-1-13293	NS_000200.1	-	104840569	104840520
Sma-1-13294	NW_003037887.1	+	919597	919646
Sma-1-13295	NS_000200.1	+	131383575	131383624
Sma-1-13296	NW_003037888.1	-	174968	174919
Sma-1-13297	NW_003038195.1	-	27776	27727
Sma-1-13298	NS_000200.1	-	303016548	303016499
Sma-1-13299	NS_000200.1	-	132383460	132383411
Sma-1-13300	NW_003037888.1	+	829169	829218
Sma-1-13301	NS_000200.1	+	133037661	133037710
Sma-1-13302	NW_003037856.1	-	152008	151959
Sma-1-13303	NS_000200.1	-	99509964	99509915
Sma-1-13304	NW_003037854.1	+	1511726	1511775
Sma-1-13305	NS_000200.1	+	98416147	98416196
Sma-1-13306	NW_003037854.1	+	1498835	1498884
Sma-1-13307	NW_003037918.1	-	26295	26246
Sma-1-13308	NS_000200.1	+	98403256	98403305
Sma-1-13309	NS_000200.1	-	166593285	166593236
Sma-1-13310	NW_003037854.1	+	1408510	1408559
Sma-1-13311	NS_000200.1	+	98312931	98312980
Sma-1-13312	NW_003038140.1	+	189933	189982

Sma-1-13313	NS_000200.1	+	286717810	286717859
Sma-1-13314	NW_003038156.1	+	38054	38103
Sma-1-13315	NS_000200.1	+	294770167	294770216
Sma-1-13316	NW_003026597.1	-	116623	116574
Sma-1-13317	NW_003037803.1	+	108502	108551
Sma-1-13318	NS_000200.1	+	23609039	23609088
Sma-1-13319	NS_000200.1	-	305737809	305737760
Sma-1-13320	NW_003037806.1	-	1792704	1792655
Sma-1-13321	NS_000200.1	-	29987918	29987869
Sma-1-13322	NW_003038155.1	-	412503	412454
Sma-1-13323	NS_000200.1	-	294535621	294535572
Sma-1-13324	NW_003037814.1	-	1557929	1557880
Sma-1-13325	NS_000200.1	-	44541789	44541740
Sma-1-13326	NW_003037852.1	+	707216	707265
Sma-1-13327	NS_000200.1	+	95099111	95099160
Sma-1-13328	NW_003037830.1	-	1367719	1367670
Sma-1-13329	NS_000200.1	-	69258090	69258041
Sma-1-13330	NW_003037827.1	+	107389	107438
Sma-1-13331	NS_000200.1	+	62729530	62729579
Sma-1-13332	NW_003037818.1	-	893105	893056
Sma-1-13333	NW_003038151.1	+	228700	228749
Sma-1-13334	NS_000200.1	+	292135943	292135992
Sma-1-13335	NS_000200.1	-	49608512	49608463
Sma-1-13336	NW_003037799.1	+	611883	611932
Sma-1-13337	NW_003037854.1	+	995091	995140
Sma-1-13338	NS_000200.1	+	17602237	17602286
Sma-1-13339	NS_000200.1	+	97899512	97899561
Sma-1-13340	NW_003037932.1	+	625159	625208
Sma-1-13341	NS_000200.1	+	178158355	178158404
Sma-1-13342	NW_003038187.1	+	4676	4725
Sma-1-13343	NS_000200.1	+	301956534	301956583
Sma-1-13344	NW_003026615.1	+	21835	21884
Sma-1-13345	NS_000200.1	+	317016276	317016325
Sma-1-13346	NW_003037979.1	+	600238	600287
Sma-1-13347	NS_000200.1	+	210661258	210661307
Sma-1-13348	NW_003038221.1	-	112010	111961
Sma-1-13349	NS_000200.1	-	308753274	308753225
Sma-1-13350	NW_003037810.1	-	883083	883034
Sma-1-13351	NW_003037810.1	-	879764	879715
Sma-1-13352	NW_003037901.1	-	355283	355234
Sma-1-13353	NW_003026510.1	-	309734	309685
Sma-1-13354	NS_000200.1	-	283749915	283749866
Sma-1-13355	NS_000200.1	-	146203744	146203695
Sma-1-13356	NS_000200.1	-	37487605	37487556
Sma-1-13357	NS_000200.1	-	37484286	37484237
Sma-1-13358	NW_003026517.1	-	574857	574808
Sma-1-13359	NS_000200.1	-	300180866	300180817
Sma-1-13360	NW_003038037.1	+	1272873	1272922
Sma-1-13361	NS_000200.1	+	251428282	251428331
Sma-1-13362	NW_003037841.1	-	374688	374639
Sma-1-13363	NS_000200.1	-	82985869	82985820
Sma-1-13364	NW_003038242.1	+	6890	6939

Sma-1-13365	NS_000200.1	+	314706036	314706085
Sma-1-13366	NW_003030423.1	+	939	988
Sma-1-13367	NS_000200.1	+	362869740	362869789
Sma-1-13368	NW_003037807.1	-	1404256	1404207
Sma-1-13369	NS_000200.1	-	31665772	31665723
Sma-1-13370	NW_003037806.1	+	54411	54460
Sma-1-13371	NS_000200.1	+	28249625	28249674
Sma-1-13372	NW_003037933.1	+	609511	609560
Sma-1-13373	NS_000200.1	+	179022683	179022732
Sma-1-13374	NW_003038032.1	+	410398	410447
Sma-1-13375	NW_003038050.1	-	90231	90182
Sma-1-13376	NS_000200.1	+	245569558	245569607
Sma-1-13377	NS_000200.1	-	256365125	256365076
Sma-1-13378	NW_003038038.1	+	1252123	1252172
Sma-1-13379	NS_000200.1	+	253155516	253155565
Sma-1-13380	NW_003038471.1	-	45575	45526
Sma-1-13381	NW_003038050.1	-	85544	85495
Sma-1-13382	NS_000200.1	-	331886489	331886440
Sma-1-13383	NS_000200.1	-	256360438	256360389
Sma-1-13384	NW_003037797.1	-	2834937	2834888
Sma-1-13385	NW_003037894.1	+	246882	246931
Sma-1-13386	NS_000200.1	+	142143455	142143504
Sma-1-13387	NS_000200.1	-	13262797	13262748
Sma-1-13388	NW_003037935.1	+	641639	641688
Sma-1-13389	NW_003037942.1	-	378048	377999
Sma-1-13390	NS_000200.1	+	180058174	180058223
Sma-1-13391	NS_000200.1	-	186137956	186137907
Sma-1-13392	NW_003037940.1	+	219589	219638
Sma-1-13393	NS_000200.1	+	183961719	183961768
Sma-1-13394	NW_003032100.1	-	1844	1795
Sma-1-13395	NS_000200.1	-	377734643	377734594
Sma-1-13396	NW_003038228.1	-	120331	120282
Sma-1-13397	NS_000200.1	-	310993611	310993562
Sma-1-13398	NW_003038075.1	-	26083	26034
Sma-1-13399	NS_000200.1	-	266144294	266144245
Sma-1-13400	NW_003038511.1	-	128840	128791
Sma-1-13401	NW_003037862.1	-	298088	298039
Sma-1-13402	NW_003037941.1	-	479000	478951
Sma-1-13403	NW_003039127.1	-	1047578	1047529
Sma-1-13404	NS_000200.1	-	338474052	338474003
Sma-1-13405	NS_000200.1	-	185213474	185213425
Sma-1-13406	NS_000200.1	-	114125077	114125028
Sma-1-13407	NS_000200.1	-	105949749	105949700
Sma-1-13408	NW_003039129.1	+	231288	231337
Sma-1-13409	NS_000200.1	+	229186096	229186145
Sma-1-13410	NW_003037796.1	-	1996602	1996553
Sma-1-13411	NS_000200.1	-	10206246	10206197
Sma-1-13412	NW_003037827.1	+	454750	454799
Sma-1-13413	NS_000200.1	+	63076891	63076940
Sma-1-13414	NW_003037852.1	+	764492	764541
Sma-1-13415	NS_000200.1	+	95156387	95156436
Sma-1-13416	NW_003037891.1	-	1118215	1118166

Sma-1-13417	NS_000200.1	-	138999493	138999444
Sma-1-13418	NW_003037912.1	-	133633	133584
Sma-1-13419	NS_000200.1	-	156945208	156945159
Sma-1-13420	NW_003038037.1	-	833603	833554
Sma-1-13421	NS_000200.1	-	250989012	250988963
Sma-1-13422	NW_003038119.1	-	134949	134900
Sma-1-13423	NS_000200.1	-	278042505	278042456
Sma-1-13424	NW_003035880.1	-	42568	42519
Sma-1-13425	NS_000200.1	-	339283632	339283583
Sma-1-13426	NW_003038016.1	-	154719	154670
Sma-1-13427	NS_000200.1	-	231271256	231271207
Sma-1-13428	NW_003037821.1	-	34230	34181
Sma-1-13429	NS_000200.1	-	53178714	53178665
Sma-1-13430	NW_003037850.1	-	388241	388192
Sma-1-13431	NS_000200.1	-	92374271	92374222
Sma-1-13432	NW_003037850.1	-	442740	442691
Sma-1-13433	NW_003037967.1	-	78452	78403
Sma-1-13434	NS_000200.1	-	199318423	199318374
Sma-1-13435	NS_000200.1	-	92428770	92428721
Sma-1-13436	NW_003037852.1	-	892206	892157
Sma-1-13437	NW_003037887.1	+	964216	964265
Sma-1-13438	NW_003026498.1	-	23892	23843
Sma-1-13439	NW_003038163.1	-	664157	664108
Sma-1-13440	NS_000200.1	+	131428194	131428243
Sma-1-13441	NS_000200.1	-	299064437	299064388
Sma-1-13442	NS_000200.1	-	275011747	275011698
Sma-1-13443	NS_000200.1	-	95284101	95284052
Sma-1-13444	NW_003038251.1	+	328137	328186
Sma-1-13445	NS_000200.1	+	318659909	318659958
Sma-1-13446	NW_003037799.1	-	1502483	1502434
Sma-1-13447	NS_000200.1	-	18492837	18492788
Sma-1-13448	NW_003038067.1	+	89791	89840
Sma-1-13449	NS_000200.1	+	262943844	262943893
Sma-1-13450	NW_003037822.1	-	29840	29791
Sma-1-13451	NW_003037909.1	+	334459	334508
Sma-1-13452	NS_000200.1	+	153876108	153876157
Sma-1-13453	NS_000200.1	-	54337846	54337797
Sma-1-13454	NW_003037894.1	-	232999	232950
Sma-1-13455	NW_003038082.1	+	219572	219621
Sma-1-13456	NS_000200.1	+	269754795	269754844
Sma-1-13457	NS_000200.1	-	142129572	142129523
Sma-1-13458	NW_003037894.1	-	446164	446115
Sma-1-13459	NS_000200.1	-	142342737	142342688
Sma-1-13460	NW_003029195.1	-	1036	987
Sma-1-13461	NS_000200.1	-	357064591	357064542
Sma-1-13462	NW_003035328.1	+	220	269
Sma-1-13463	NS_000200.1	+	367276384	367276433
Sma-1-13464	NW_003038514.1	+	93402	93451
Sma-1-13465	NS_000200.1	+	339717941	339717990
Sma-1-13466	NW_003037898.1	-	157543	157494
Sma-1-13467	NW_003037993.1	+	38938	38987
Sma-1-13468	NS_000200.1	+	218225641	218225690

Sma-1-13469	NS_000200.1	-	144141365	144141316
Sma-1-13470	NW_003037899.1	-	152396	152347
Sma-1-13471	NS_000200.1	-	144671507	144671458
Sma-1-13472	NW_003037899.1	-	550954	550905
Sma-1-13473	NW_003037904.1	+	163352	163401
Sma-1-13474	NW_003038057.1	+	183682	183731
Sma-1-13475	NS_000200.1	+	148877851	148877900
Sma-1-13476	NS_000200.1	+	259389498	259389547
Sma-1-13477	NS_000200.1	-	145070065	145070016
Sma-1-13478	NW_003037836.1	+	365891	365940
Sma-1-13479	NS_000200.1	+	75922990	75923039
Sma-1-13480	NW_003037910.1	+	1217323	1217372
Sma-1-13481	NW_003038513.1	+	108604	108653
Sma-1-13482	NS_000200.1	+	155729298	155729347
Sma-1-13483	NS_000200.1	+	339117781	339117830
Sma-1-13484	NW_003037911.1	+	17905	17954
Sma-1-13485	NS_000200.1	+	155805258	155805307
Sma-1-13486	NW_003037946.1	+	589399	589448
Sma-1-13487	NS_000200.1	+	191283259	191283308
Sma-1-13488	NW_003026464.1	-	150022	149973
Sma-1-13489	NS_000200.1	-	203450331	203450282
Sma-1-13490	NW_003025461.1	+	184	233
Sma-1-13491	NW_003027227.1	+	290	339
Sma-1-13492	NW_003038078.1	+	40626	40675
Sma-1-13493	NS_000200.1	+	267246829	267246878
Sma-1-13494	NS_000200.1	+	352750690	352750739
Sma-1-13495	NS_000200.1	+	353298574	353298623
Sma-1-13496	NW_003025431.1	+	207	256
Sma-1-13497	NS_000200.1	+	352691282	352691331
Sma-1-13498	NW_003038011.1	+	437987	438036
Sma-1-13499	NW_003038057.1	-	155715	155666
Sma-1-13500	NS_000200.1	+	227965637	227965686
Sma-1-13501	NS_000200.1	-	259361531	259361482
Sma-1-13502	NW_003038020.1	-	258150	258101
Sma-1-13503	NW_003038261.1	+	486544	486593
Sma-1-13504	NS_000200.1	+	324100195	324100244
Sma-1-13505	NS_000200.1	-	234225494	234225445
Sma-1-13506	NW_003023422.1	+	232	281
Sma-1-13507	NW_003029015.1	-	1583	1534
Sma-1-13508	NW_003032240.1	-	934	885
Sma-1-13509	NW_003026458.1	-	239738	239689
Sma-1-13510	NW_003037797.1	+	133385	133434
Sma-1-13511	NW_003037797.1	+	1603662	1603711
Sma-1-13512	NW_003037807.1	-	538542	538493
Sma-1-13513	NW_003037853.1	-	1368179	1368130
Sma-1-13514	NW_003037886.1	+	256524	256573
Sma-1-13515	NW_003037908.1	+	656674	656723
Sma-1-13516	NW_003037915.1	+	377052	377101
Sma-1-13517	NW_003037923.1	-	357653	357604
Sma-1-13518	NW_003038018.1	-	76700	76651
Sma-1-13519	NW_003038028.1	+	457647	457696
Sma-1-13520	NW_003038067.1	-	25324	25275

Sma-1-13521	NW_003038117.1	-	164623	164574
Sma-1-13522	NW_003038130.1	+	357280	357329
Sma-1-13523	NS_000200.1	+	10561245	10561294
Sma-1-13524	NS_000200.1	+	12031522	12031571
Sma-1-13525	NS_000200.1	+	129512091	129512140
Sma-1-13526	NS_000200.1	+	153129805	153129854
Sma-1-13527	NS_000200.1	+	162277038	162277087
Sma-1-13528	NS_000200.1	+	241765221	241765270
Sma-1-13529	NS_000200.1	+	281912266	281912315
Sma-1-13530	NS_000200.1	+	373336911	373336960
Sma-1-13531	NS_000200.1	-	375167044	375166995
Sma-1-13532	NS_000200.1	-	357982211	357982162
Sma-1-13533	NS_000200.1	-	277482448	277482399
Sma-1-13534	NS_000200.1	-	262879377	262879328
Sma-1-13535	NS_000200.1	-	232477550	232477501
Sma-1-13536	NS_000200.1	-	171413866	171413817
Sma-1-13537	NS_000200.1	-	102107968	102107919
Sma-1-13538	NS_000200.1	-	96860230	96860181
Sma-1-13539	NS_000200.1	-	30800058	30800009
Sma-1-13540	NW_003026517.1	-	446323	446274
Sma-1-13541	NS_000200.1	-	300052332	300052283
Sma-1-13542	NW_003037835.1	+	556059	556108
Sma-1-13543	NS_000200.1	+	74952567	74952616
Sma-1-13544	NW_003037933.1	+	212152	212201
Sma-1-13545	NS_000200.1	+	178625324	178625373
Sma-1-13546	NW_003037936.1	-	703952	703903
Sma-1-13547	NS_000200.1	-	181143681	181143632
Sma-1-13548	NW_003029748.1	-	1107	1058
Sma-1-13549	NS_000200.1	-	366534262	366534213
Sma-1-13550	NW_003038023.1	+	470355	470404
Sma-1-13551	NS_000200.1	+	237630646	237630695
Sma-1-13552	NW_003037871.1	+	165746	165795
Sma-1-13553	NS_000200.1	+	117613484	117613533
Sma-1-13554	NW_003026490.1	-	138140	138091
Sma-1-13555	NS_000200.1	-	267769856	267769807
Sma-1-13556	NW_003037806.1	-	1291607	1291558
Sma-1-13557	NS_000200.1	-	29486821	29486772
Sma-1-13558	NW_003037810.1	-	1007738	1007689
Sma-1-13559	NW_003038150.1	+	12268	12317
Sma-1-13560	NS_000200.1	+	291491006	291491055
Sma-1-13561	NS_000200.1	-	37612260	37612211
Sma-1-13562	NW_003026617.1	+	329940	329989
Sma-1-13563	NS_000200.1	+	319900799	319900848
Sma-1-13564	NW_003037978.1	+	377849	377898
Sma-1-13565	NS_000200.1	+	209645859	209645908
Sma-1-13566	NW_003038077.1	+	208316	208365
Sma-1-13567	NS_000200.1	+	266979956	266980005
Sma-1-13568	NW_003022752.1	+	521	570
Sma-1-13569	NW_003038137.1	+	114671	114720
Sma-1-13570	NS_000200.1	+	284903567	284903616
Sma-1-13571	NS_000200.1	+	368037715	368037764
Sma-1-13572	NW_003037800.1	-	1659163	1659114

Sma-1-13573	NS_000200.1	-	20288523	20288474
Sma-1-13574	NW_003037809.1	+	128522	128571
Sma-1-13575	NS_000200.1	+	33792986	33793035
Sma-1-13576	NW_003037834.1	-	643397	643348
Sma-1-13577	NS_000200.1	-	73803883	73803834
Sma-1-13578	NW_003037834.1	-	293596	293547
Sma-1-13579	NW_003026508.1	-	32648	32599
Sma-1-13580	NS_000200.1	-	281077262	281077213
Sma-1-13581	NS_000200.1	-	73454082	73454033
Sma-1-13582	NW_003037834.1	-	119940	119891
Sma-1-13583	NS_000200.1	-	73280426	73280377
Sma-1-13584	NW_003037903.1	-	1011285	1011236
Sma-1-13585	NS_000200.1	-	148565366	148565317
Sma-1-13586	NW_003038163.1	-	642305	642256
Sma-1-13587	NS_000200.1	-	299042585	299042536
Sma-1-13588	NW_003038153.1	+	224713	224762
Sma-1-13589	NS_000200.1	+	293286762	293286811
Sma-1-13590	NW_003035166.1	+	140	189
Sma-1-13591	NW_003037981.1	+	338646	338695
Sma-1-13592	NS_000200.1	+	212736514	212736563
Sma-1-13593	NS_000200.1	+	370722718	370722767
Sma-1-13594	NW_003037830.1	+	544475	544524
Sma-1-13595	NS_000200.1	+	68434846	68434895
Sma-1-13596	NW_003037830.1	+	408313	408362
Sma-1-13597	NS_000200.1	+	68298684	68298733
Sma-1-13598	NW_003037906.1	-	482336	482287
Sma-1-13599	NS_000200.1	-	150790115	150790066
Sma-1-13600	NW_003037827.1	+	2455813	2455862
Sma-1-13601	NS_000200.1	+	65077954	65078003
Sma-1-13602	NW_003037825.1	-	1427300	1427251
Sma-1-13603	NS_000200.1	-	60557082	60557033
Sma-1-13604	NW_003037821.1	+	63256	63305
Sma-1-13605	NS_000200.1	+	53207740	53207789
Sma-1-13606	NW_003037820.1	-	62691	62642
Sma-1-13607	NW_003026488.1	+	300526	300575
Sma-1-13608	NS_000200.1	+	263807020	263807069
Sma-1-13609	NS_000200.1	-	52335673	52335624
Sma-1-13610	NW_003037918.1	+	1799589	1799638
Sma-1-13611	NS_000200.1	+	168366579	168366628
Sma-1-13612	NW_003037141.1	-	752	703
Sma-1-13613	NS_000200.1	-	361699489	361699440
Sma-1-13614	NW_003026458.1	-	490705	490656
Sma-1-13615	NS_000200.1	-	102358935	102358886
Sma-1-13616	NW_003038024.1	+	531974	532023
Sma-1-13617	NS_000200.1	+	238429003	238429052
Sma-1-13618	NW_003038031.1	+	248053	248102
Sma-1-13619	NS_000200.1	+	244525811	244525860
Sma-1-13620	NW_003037873.1	-	76869	76820
Sma-1-13621	NS_000200.1	-	118815997	118815948
Sma-1-13622	NW_003037912.1	+	662243	662292
Sma-1-13623	NS_000200.1	+	157473818	157473867
Sma-1-13624	NW_003037914.1	-	807270	807221

Sma-1-13625	NW_003026487.1	+	122837	122886
Sma-1-13626	NS_000200.1	+	261033531	261033580
Sma-1-13627	NS_000200.1	-	160951520	160951471
Sma-1-13628	NW_003037814.1	+	1376793	1376842
Sma-1-13629	NS_000200.1	+	44360653	44360702
Sma-1-13630	NW_003037813.1	-	972764	972715
Sma-1-13631	NS_000200.1	-	41959627	41959578
Sma-1-13632	NW_003037809.1	+	495665	495714
Sma-1-13633	NS_000200.1	+	34160129	34160178
Sma-1-13634	NW_003037917.1	+	738605	738654
Sma-1-13635	NS_000200.1	+	165775409	165775458
Sma-1-13636	NW_003037918.1	+	1522361	1522410
Sma-1-13637	NS_000200.1	+	168089351	168089400
Sma-1-13638	NW_003037918.1	+	904959	905008
Sma-1-13639	NS_000200.1	+	167471949	167471998
Sma-1-13640	NW_003037789.1	+	44775	44824
Sma-1-13641	NS_000200.1	+	380829395	380829444
Sma-1-13642	NW_003038240.1	+	21595	21644
Sma-1-13643	NS_000200.1	+	314160526	314160575
Sma-1-13644	NW_003023042.1	+	4883	4932
Sma-1-13645	NS_000200.1	+	372231547	372231596
Sma-1-13646	NW_003038462.1	+	43094	43143
Sma-1-13647	NS_000200.1	+	331410757	331410806
Sma-1-13648	NW_003038056.1	+	100137	100186
Sma-1-13649	NS_000200.1	+	259077788	259077837
Sma-1-13650	NW_003039127.1	-	2058270	2058221
Sma-1-13651	NS_000200.1	-	115135769	115135720
Sma-1-13652	NW_003035817.1	-	4369	4320
Sma-1-13653	NW_003037992.1	+	188970	189019
Sma-1-13654	NS_000200.1	+	218030451	218030500
Sma-1-13655	NS_000200.1	-	364155238	364155189
Sma-1-13656	NW_003038008.1	-	415049	415000
Sma-1-13657	NS_000200.1	-	226088145	226088096
Sma-1-13658	NW_003037803.1	+	1309619	1309668
Sma-1-13659	NS_000200.1	+	24810156	24810205
Sma-1-13660	NW_003037805.1	+	1621369	1621418
Sma-1-13661	NW_003037825.1	-	1666855	1666806
Sma-1-13662	NW_003037890.1	+	845791	845840
Sma-1-13663	NW_003038024.1	-	803814	803765
Sma-1-13664	NS_000200.1	+	28179213	28179262
Sma-1-13665	NS_000200.1	+	136529152	136529201
Sma-1-13666	NS_000200.1	-	238700843	238700794
Sma-1-13667	NS_000200.1	-	60796637	60796588
Sma-1-13668	NW_003037926.1	-	5211	5162
Sma-1-13669	NS_000200.1	-	173173481	173173432
Sma-1-13670	NW_003037798.1	+	770179	770228
Sma-1-13671	NS_000200.1	+	15029337	15029386
Sma-1-13672	NW_003037934.1	+	224800	224849
Sma-1-13673	NS_000200.1	+	179271547	179271596
Sma-1-13674	NW_003037118.1	+	46	95
Sma-1-13675	NW_003038015.1	+	187736	187785
Sma-1-13676	NS_000200.1	+	230511226	230511275

Sma-1-13677	NS_000200.1	+	361201123	361201172
Sma-1-13678	NW_003037787.1	-	3748	3699
Sma-1-13679	NS_000200.1	-	380251917	380251868
Sma-1-13680	NW_003039127.1	+	1297552	1297601
Sma-1-13681	NS_000200.1	+	114375051	114375100
Sma-1-13682	NW_003037831.1	+	854375	854424
Sma-1-13683	NS_000200.1	+	70303677	70303726
Sma-1-13684	NW_003033063.1	+	227	276
Sma-1-13685	NW_003037944.1	+	229924	229973
Sma-1-13686	NS_000200.1	+	188451277	188451326
Sma-1-13687	NS_000200.1	+	326302004	326302053
Sma-1-13688	NW_003037965.1	-	140300	140251
Sma-1-13689	NS_000200.1	-	198429385	198429336
Sma-1-13690	NW_003026458.1	-	471925	471876
Sma-1-13691	NS_000200.1	-	102340155	102340106
Sma-1-13692	NW_003037918.1	+	1525376	1525425
Sma-1-13693	NW_003038115.1	+	75847	75896
Sma-1-13694	NS_000200.1	+	168092366	168092415
Sma-1-13695	NS_000200.1	+	277131142	277131191
Sma-1-13696	NW_003026574.1	+	90393	90442
Sma-1-13697	NS_000200.1	+	301897726	301897775
Sma-1-13698	NW_003037998.1	-	209273	209224
Sma-1-13699	NS_000200.1	-	221495869	221495820
Sma-1-13700	NW_003039130.1	+	24710	24759
Sma-1-13701	NS_000200.1	+	317699321	317699370
Sma-1-13702	NW_003037914.1	-	1254991	1254942
Sma-1-13703	NS_000200.1	-	161399241	161399192
Sma-1-13704	NW_003022684.1	-	372	323
Sma-1-13705	NW_003037809.1	+	1792027	1792076
Sma-1-13706	NW_003037826.1	+	1183473	1183522
Sma-1-13707	NW_003037932.1	-	514623	514574
Sma-1-13708	NW_003037932.1	-	511750	511701
Sma-1-13709	NW_003038037.1	+	66554	66603
Sma-1-13710	NW_003038239.1	-	19574	19525
Sma-1-13711	NW_003038068.1	+	148319	148368
Sma-1-13712	NW_003038079.1	-	269172	269123
Sma-1-13713	NS_000200.1	+	35456491	35456540
Sma-1-13714	NS_000200.1	+	62182989	62183038
Sma-1-13715	NS_000200.1	+	250221963	250222012
Sma-1-13716	NS_000200.1	+	263215527	263215576
Sma-1-13717	NS_000200.1	-	365418554	365418505
Sma-1-13718	NS_000200.1	-	313860356	313860307
Sma-1-13719	NS_000200.1	-	268399160	268399111
Sma-1-13720	NS_000200.1	-	178047819	178047770
Sma-1-13721	NS_000200.1	-	178044946	178044897
Sma-1-13722	NW_003026511.1	+	30	79
Sma-1-13723	NS_000200.1	+	284383082	284383131
Sma-1-13724	NW_003038053.1	-	329879	329830
Sma-1-13725	NS_000200.1	-	257689974	257689925
Sma-1-13726	NW_003038145.1	+	327865	327914
Sma-1-13727	NS_000200.1	+	288939288	288939337
Sma-1-13728	NW_003026456.1	+	947605	947654

Sma-1-13729	NW_003037811.1	-	676686	676637
Sma-1-13730	NW_003038042.1	-	157434	157385
Sma-1-13731	NS_000200.1	+	58870741	58870790
Sma-1-13732	NS_000200.1	-	254103033	254102984
Sma-1-13733	NS_000200.1	-	38846928	38846879
Sma-1-13734	NW_003038067.1	+	149622	149671
Sma-1-13735	NS_000200.1	+	263003675	263003724
Sma-1-13736	NW_003037920.1	+	280697	280746
Sma-1-13737	NW_003038235.1	+	48974	49023
Sma-1-13738	NW_003037796.1	+	2013049	2013098
Sma-1-13739	NW_003037832.1	+	611551	611600
Sma-1-13740	NW_003037835.1	+	696452	696501
Sma-1-13741	NW_003037891.1	+	391619	391668
Sma-1-13742	NW_003037905.1	+	1000242	1000291
Sma-1-13743	NW_003037913.1	-	1028887	1028838
Sma-1-13744	NW_003037913.1	-	764126	764077
Sma-1-13745	NW_003037923.1	+	394070	394119
Sma-1-13746	NW_003038083.1	+	17099	17148
Sma-1-13747	NW_003038083.1	-	17009	16960
Sma-1-13748	NS_000200.1	+	10222693	10222742
Sma-1-13749	NS_000200.1	+	71820982	71821031
Sma-1-13750	NS_000200.1	+	75092960	75093009
Sma-1-13751	NS_000200.1	+	138272897	138272946
Sma-1-13752	NS_000200.1	+	150269254	150269303
Sma-1-13753	NS_000200.1	+	169461504	169461553
Sma-1-13754	NS_000200.1	+	171450283	171450332
Sma-1-13755	NS_000200.1	+	270260436	270260485
Sma-1-13756	NS_000200.1	+	313114565	313114614
Sma-1-13757	NS_000200.1	-	270260346	270260297
Sma-1-13758	NS_000200.1	-	159592638	159592589
Sma-1-13759	NS_000200.1	-	159327877	159327828
Sma-1-13760	NW_003037944.1	+	24144	24193
Sma-1-13761	NS_000200.1	+	188245497	188245546
Sma-1-13762	NW_003035793.1	-	560	511
Sma-1-13763	NS_000200.1	-	364101546	364101497
Sma-1-13764	NW_003037797.1	-	2908999	2908950
Sma-1-13765	NS_000200.1	-	13336859	13336810
Sma-1-13766	NW_003037827.1	+	1106559	1106608
Sma-1-13767	NS_000200.1	+	63728700	63728749
Sma-1-13768	NW_003037937.1	+	239581	239630
Sma-1-13769	NS_000200.1	+	181439490	181439539
Sma-1-13770	NW_003038163.1	+	623521	623570
Sma-1-13771	NS_000200.1	+	299023801	299023850
Sma-1-13772	NW_003024922.1	+	186	235
Sma-1-13773	NW_003038026.1	+	488039	488088
Sma-1-13774	NS_000200.1	+	239995800	239995849
Sma-1-13775	NS_000200.1	+	355711170	355711219
Sma-1-13776	NW_003037796.1	+	1033836	1033885
Sma-1-13777	NS_000200.1	+	9243480	9243529
Sma-1-13778	NW_003037793.1	-	880165	880116
Sma-1-13779	NS_000200.1	-	880165	880116
Sma-1-13780	NW_003037939.1	-	823658	823609

Sma-1-13781	NS_000200.1	-	183693412	183693363
Sma-1-13782	NW_003038518.1	-	220447	220398
Sma-1-13783	NS_000200.1	-	341857280	341857231
Sma-1-13784	NW_003037945.1	+	942790	942839
Sma-1-13785	NS_000200.1	+	190044754	190044803
Sma-1-13786	NW_003037962.1	-	315038	314989
Sma-1-13787	NS_000200.1	-	196391881	196391832
Sma-1-13788	NW_003038025.1	+	429241	429290
Sma-1-13789	NS_000200.1	+	239287486	239287535
Sma-1-13790	NW_003037829.1	-	1567055	1567006
Sma-1-13791	NW_003037909.1	+	750665	750714
Sma-1-13792	NS_000200.1	+	154292314	154292363
Sma-1-13793	NS_000200.1	-	67467593	67467544
Sma-1-13794	NW_003038034.1	+	819582	819631
Sma-1-13795	NS_000200.1	+	247761338	247761387
Sma-1-13796	NW_003035879.1	-	55226	55177
Sma-1-13797	NS_000200.1	-	338022553	338022504
Sma-1-13798	NW_003037971.1	-	619016	618967
Sma-1-13799	NS_000200.1	-	202241066	202241017
Sma-1-13800	NW_003026617.1	+	353026	353075
Sma-1-13801	NS_000200.1	+	319923885	319923934
Sma-1-13802	NW_003037982.1	-	674728	674679
Sma-1-13803	NS_000200.1	-	214333923	214333874
Sma-1-13804	NW_003037940.1	+	789314	789363
Sma-1-13805	NW_003037974.1	+	832167	832216
Sma-1-13806	NS_000200.1	+	184531444	184531493
Sma-1-13807	NS_000200.1	+	205507473	205507522
Sma-1-13808	NW_003038149.1	+	7980	8029
Sma-1-13809	NS_000200.1	+	291036593	291036642
Sma-1-13810	NW_003022248.1	+	5033	5082
Sma-1-13811	NS_000200.1	+	361017519	361017568
Sma-1-13812	NW_003037981.1	-	332814	332765
Sma-1-13813	NS_000200.1	-	212730682	212730633
Sma-1-13814	NW_003038009.1	+	296189	296238
Sma-1-13815	NS_000200.1	+	226670252	226670301
Sma-1-13816	NW_003037793.1	-	2022964	2022915
Sma-1-13817	NS_000200.1	-	2022964	2022915
Sma-1-13818	NW_003038007.1	-	263798	263749
Sma-1-13819	NS_000200.1	-	225512353	225512304
Sma-1-13820	NW_003038224.1	+	108211	108260
Sma-1-13821	NS_000200.1	+	309519939	309519988
Sma-1-13822	NW_003036041.1	-	3258	3209
Sma-1-13823	NW_003026599.1	+	130567	130616
Sma-1-13824	NW_003026458.1	-	394277	394228
Sma-1-13825	NW_003037886.1	+	1117130	1117179
Sma-1-13826	NW_003037939.1	+	592036	592085
Sma-1-13827	NW_003038515.1	+	215328	215377
Sma-1-13828	NW_003038076.1	+	252363	252412
Sma-1-13829	NW_003038141.1	+	218545	218594
Sma-1-13830	NW_003038141.1	+	219754	219803
Sma-1-13831	NW_003038141.1	+	270607	270656
Sma-1-13832	NS_000200.1	+	130372697	130372746

Sma-1-13833	NS_000200.1	+	183461790	183461839
Sma-1-13834	NS_000200.1	+	266469537	266469586
Sma-1-13835	NS_000200.1	+	287108524	287108573
Sma-1-13836	NS_000200.1	+	287109733	287109782
Sma-1-13837	NS_000200.1	+	287160586	287160635
Sma-1-13838	NS_000200.1	+	306008896	306008945
Sma-1-13839	NS_000200.1	+	340299393	340299442
Sma-1-13840	NS_000200.1	-	343333336	343333287
Sma-1-13841	NS_000200.1	-	102262507	102262458
Sma-1-13842	NW_003038020.1	+	293266	293315
Sma-1-13843	NS_000200.1	+	234260610	234260659
Sma-1-13844	NW_003038034.1	-	902646	902597
Sma-1-13845	NS_000200.1	-	247844402	247844353
Sma-1-13846	NW_003038080.1	-	215249	215200
Sma-1-13847	NS_000200.1	-	268667859	268667810
Sma-1-13848	NW_003023398.1	+	3361	3410
Sma-1-13849	NW_003028168.1	-	202	153
Sma-1-13850	NW_003037800.1	+	1351811	1351860
Sma-1-13851	NW_003037809.1	-	655757	655708
Sma-1-13852	NW_003037825.1	-	886946	886897
Sma-1-13853	NW_003037836.1	-	386405	386356
Sma-1-13854	NW_003037836.1	-	384903	384854
Sma-1-13855	NW_003037888.1	+	36863	36912
Sma-1-13856	NW_003038016.1	+	354940	354989
Sma-1-13857	NW_003038018.1	-	697845	697796
Sma-1-13858	NW_003038021.1	+	722686	722735
Sma-1-13859	NW_003038023.1	-	493303	493254
Sma-1-13860	NW_003039127.1	-	1966529	1966480
Sma-1-13861	NW_003038063.1	+	157350	157399
Sma-1-13862	NS_000200.1	+	19981171	19981220
Sma-1-13863	NS_000200.1	+	132245355	132245404
Sma-1-13864	NS_000200.1	+	231471477	231471526
Sma-1-13865	NS_000200.1	+	235551261	235551310
Sma-1-13866	NS_000200.1	+	261653628	261653677
Sma-1-13867	NS_000200.1	+	373274767	373274816
Sma-1-13868	NS_000200.1	-	374198178	374198129
Sma-1-13869	NS_000200.1	-	237653594	237653545
Sma-1-13870	NS_000200.1	-	233098695	233098646
Sma-1-13871	NS_000200.1	-	115044028	115043979
Sma-1-13872	NS_000200.1	-	75943504	75943455
Sma-1-13873	NS_000200.1	-	75942002	75941953
Sma-1-13874	NS_000200.1	-	60016728	60016679
Sma-1-13875	NS_000200.1	-	34320221	34320172
Sma-1-13876	NW_003037832.1	+	686107	686156
Sma-1-13877	NS_000200.1	+	71895538	71895587
Sma-1-13878	NW_003037806.1	+	1477013	1477062
Sma-1-13879	NS_000200.1	+	29672227	29672276
Sma-1-13880	NW_003037912.1	-	46732	46683
Sma-1-13881	NS_000200.1	-	156858307	156858258
Sma-1-13882	NW_003037839.1	-	554312	554263
Sma-1-13883	NS_000200.1	-	79512462	79512413
Sma-1-13884	NW_003026596.1	-	126007	125958

Sma-1-13885	NS_000200.1	-	305396635	305396586
Sma-1-13886	NW_003035648.1	-	885	836
Sma-1-13887	NS_000200.1	-	330443222	330443173
Sma-1-13888	NW_003034684.1	-	1350	1301
Sma-1-13889	NW_003037889.1	-	1071854	1071805
Sma-1-13890	NS_000200.1	-	328162177	328162128
Sma-1-13891	NS_000200.1	-	134848344	134848295
Sma-1-13892	NW_003037798.1	-	1251286	1251237
Sma-1-13893	NS_000200.1	-	15510444	15510395
Sma-1-13894	NW_003038142.1	-	394435	394386
Sma-1-13895	NS_000200.1	-	287604871	287604822
Sma-1-13896	NW_003037798.1	+	1666957	1667006
Sma-1-13897	NS_000200.1	+	15926115	15926164
Sma-1-13898	NW_003037982.1	-	825616	825567
Sma-1-13899	NS_000200.1	-	214484811	214484762
Sma-1-13900	NW_003026481.1	-	171390	171341
Sma-1-13901	NS_000200.1	-	255571374	255571325
Sma-1-13902	NW_003026517.1	+	311335	311384
Sma-1-13903	NS_000200.1	+	299917344	299917393
Sma-1-13904	NW_003037863.1	-	409642	409593
Sma-1-13905	NW_003038505.1	+	26264	26313
Sma-1-13906	NS_000200.1	+	336105601	336105650
Sma-1-13907	NS_000200.1	-	107168009	107167960
Sma-1-13908	NW_003038038.1	+	82894	82943
Sma-1-13909	NS_000200.1	+	251986287	251986336
Sma-1-13910	NW_003038151.1	+	183761	183810
Sma-1-13911	NW_003038156.1	+	253490	253539
Sma-1-13912	NS_000200.1	+	292091004	292091053
Sma-1-13913	NS_000200.1	+	294985603	294985652
Sma-1-13914	NW_003038147.1	-	397550	397501
Sma-1-13915	NS_000200.1	-	289925293	289925244
Sma-1-13916	NW_003038148.1	+	201581	201630
Sma-1-13917	NS_000200.1	+	290275565	290275614
Sma-1-13918	NW_003038025.1	-	543958	543909
Sma-1-13919	NS_000200.1	-	239402203	239402154
Sma-1-13920	NW_003026608.1	+	60160	60209
Sma-1-13921	NS_000200.1	+	309338280	309338329
Sma-1-13922	NW_003026481.1	+	2525	2574
Sma-1-13923	NS_000200.1	+	255402509	255402558
Sma-1-13924	NW_003037790.1	-	216356	216307
Sma-1-13925	NW_003037797.1	+	2956679	2956728
Sma-1-13926	NW_003037798.1	-	1790179	1790130
Sma-1-13927	NW_003037805.1	-	310917	310868
Sma-1-13928	NW_003037809.1	+	217403	217452
Sma-1-13929	NW_003037827.1	+	2196398	2196447
Sma-1-13930	NW_003037834.1	+	1073938	1073987
Sma-1-13931	NW_003037908.1	-	791249	791200
Sma-1-13932	NW_003037914.1	+	1225267	1225316
Sma-1-13933	NW_003038010.1	-	397805	397756
Sma-1-13934	NW_003038011.1	+	227072	227121
Sma-1-13935	NW_003038028.1	-	55156	55107
Sma-1-13936	NW_003038037.1	+	672091	672140

Sma-1-13937	NW_003038038.1	-	594818	594769
Sma-1-13938	NW_003039127.1	+	1622744	1622793
Sma-1-13939	NW_003026576.1	+	11734	11783
Sma-1-13940	NS_000200.1	+	13384539	13384588
Sma-1-13941	NS_000200.1	+	33881867	33881916
Sma-1-13942	NS_000200.1	+	64818539	64818588
Sma-1-13943	NS_000200.1	+	74234424	74234473
Sma-1-13944	NS_000200.1	+	114700243	114700292
Sma-1-13945	NS_000200.1	+	161369517	161369566
Sma-1-13946	NS_000200.1	+	227754722	227754771
Sma-1-13947	NS_000200.1	+	250827500	250827549
Sma-1-13948	NS_000200.1	+	302639310	302639359
Sma-1-13949	NS_000200.1	-	381560528	381560479
Sma-1-13950	NS_000200.1	-	252498211	252498162
Sma-1-13951	NS_000200.1	-	241362730	241362681
Sma-1-13952	NS_000200.1	-	227216028	227215979
Sma-1-13953	NS_000200.1	-	153264380	153264331
Sma-1-13954	NS_000200.1	-	26868761	26868712
Sma-1-13955	NS_000200.1	-	16049337	16049288
Sma-1-13956	NW_003037836.1	-	233304	233255
Sma-1-13957	NS_000200.1	-	75790403	75790354
Sma-1-13958	NW_003037810.1	+	276877	276926
Sma-1-13959	NS_000200.1	+	36881399	36881448
Sma-1-13960	NW_003037909.1	-	131386	131337
Sma-1-13961	NS_000200.1	-	153673035	153672986
Sma-1-13962	NW_003038029.1	-	198615	198566
Sma-1-13963	NS_000200.1	-	242399516	242399467
Sma-1-13964	NW_003038240.1	-	241570	241521
Sma-1-13965	NS_000200.1	-	314380501	314380452
Sma-1-13966	NW_003039127.1	+	2307511	2307560
Sma-1-13967	NS_000200.1	+	115385010	115385059
Sma-1-13968	NW_003037824.1	+	22617	22666
Sma-1-13969	NS_000200.1	+	56953557	56953606
Sma-1-13970	NW_003037840.1	-	1567480	1567431
Sma-1-13971	NS_000200.1	-	82190804	82190755
Sma-1-13972	NW_003037831.1	-	1108765	1108716
Sma-1-13973	NS_000200.1	-	70558067	70558018
Sma-1-13974	NW_003037935.1	-	739557	739508
Sma-1-13975	NS_000200.1	-	180156092	180156043
Sma-1-13976	NW_003038077.1	-	250421	250372
Sma-1-13977	NS_000200.1	-	267022061	267022012
Sma-1-13978	NW_003037812.1	+	987506	987555
Sma-1-13979	NS_000200.1	+	40470245	40470294
Sma-1-13980	NW_003038123.1	+	218375	218424
Sma-1-13981	NW_003038135.1	-	84687	84638
Sma-1-13982	NW_003038143.1	-	416420	416371
Sma-1-13983	NS_000200.1	+	278966542	278966591
Sma-1-13984	NS_000200.1	-	288175912	288175863
Sma-1-13985	NS_000200.1	-	283957026	283956977
Sma-1-13986	NW_003026608.1	-	96111	96062
Sma-1-13987	NW_003038487.1	+	45375	45424
Sma-1-13988	NW_003026470.1	+	97192	97241

Sma-1-13989	NW_003026609.1	+	90906	90955
Sma-1-13990	NW_003037814.1	+	265921	265970
Sma-1-13991	NW_003037830.1	-	1242603	1242554
Sma-1-13992	NW_003037847.1	-	270014	269965
Sma-1-13993	NW_003037854.1	-	1627504	1627455
Sma-1-13994	NW_003037923.1	+	202217	202266
Sma-1-13995	NW_003037935.1	+	648984	649033
Sma-1-13996	NW_003038033.1	-	928210	928161
Sma-1-13997	NW_003038454.1	-	68344	68295
Sma-1-13998	NW_003026483.1	+	75781	75830
Sma-1-13999	NS_000200.1	+	43249781	43249830
Sma-1-14000	NS_000200.1	+	171258430	171258479
Sma-1-14001	NS_000200.1	+	180065519	180065568
Sma-1-14002	NS_000200.1	+	219221161	219221210
Sma-1-14003	NS_000200.1	+	256590305	256590354
Sma-1-14004	NS_000200.1	+	309745898	309745947
Sma-1-14005	NS_000200.1	+	333501162	333501211
Sma-1-14006	NS_000200.1	-	330780694	330780645
Sma-1-14007	NS_000200.1	-	309374231	309374182
Sma-1-14008	NS_000200.1	-	246908212	246908163
Sma-1-14009	NS_000200.1	-	98531925	98531876
Sma-1-14010	NS_000200.1	-	88921744	88921695
Sma-1-14011	NS_000200.1	-	69132974	69132925
Sma-1-14012	NW_003022199.1	+	795	844
Sma-1-14013	NS_000200.1	+	360916729	360916778
Sma-1-14014	NW_003037851.1	-	581809	581760
Sma-1-14015	NS_000200.1	-	93985119	93985070
Sma-1-14016	NW_003037966.1	-	478930	478881
Sma-1-14017	NS_000200.1	-	199180793	199180744
Sma-1-14018	NW_003037846.1	+	273515	273564
Sma-1-14019	NW_003038028.1	-	819038	818989
Sma-1-14020	NS_000200.1	+	87908085	87908134
Sma-1-14021	NS_000200.1	-	242126612	242126563
Sma-1-14022	NW_003037892.1	+	345899	345948
Sma-1-14023	NS_000200.1	+	141069126	141069175
Sma-1-14024	NW_003037846.1	+	649005	649054
Sma-1-14025	NS_000200.1	+	88283575	88283624
Sma-1-14026	NW_003037894.1	-	292854	292805
Sma-1-14027	NS_000200.1	-	142189427	142189378
Sma-1-14028	NW_003037904.1	-	253065	253016
Sma-1-14029	NS_000200.1	-	148967564	148967515
Sma-1-14030	NW_003037827.1	+	1091365	1091414
Sma-1-14031	NS_000200.1	+	63713506	63713555
Sma-1-14032	NW_003037909.1	+	425014	425063
Sma-1-14033	NS_000200.1	+	153966663	153966712
Sma-1-14034	NW_003037934.1	-	189134	189085
Sma-1-14035	NS_000200.1	-	179235881	179235832
Sma-1-14036	NW_003037888.1	-	656570	656521
Sma-1-14037	NS_000200.1	-	132865062	132865013
Sma-1-14038	NW_003037820.1	-	490418	490369
Sma-1-14039	NW_003037820.1	-	486763	486714
Sma-1-14040	NS_000200.1	-	52763400	52763351

Sma-1-14041	NS_000200.1	-	52759745	52759696
Sma-1-14042	NW_003037819.1	+	1123393	1123442
Sma-1-14043	NS_000200.1	+	52211658	52211707
Sma-1-14044	NW_003037918.1	-	440396	440347
Sma-1-14045	NS_000200.1	-	167007386	167007337
Sma-1-14046	NW_003037932.1	+	249773	249822
Sma-1-14047	NS_000200.1	+	177782969	177783018
Sma-1-14048	NW_003037936.1	-	229980	229931
Sma-1-14049	NW_003037850.1	-	93057	93008
Sma-1-14050	NS_000200.1	-	180669709	180669660
Sma-1-14051	NS_000200.1	-	92079087	92079038
Sma-1-14052	NW_003037589.1	-	14773	14724
Sma-1-14053	NS_000200.1	-	372663725	372663676
Sma-1-14054	NW_003028779.1	-	1375	1326
Sma-1-14055	NS_000200.1	-	369922714	369922665
Sma-1-14056	NW_003026471.1	-	214881	214832
Sma-1-14057	NW_003037807.1	+	140162	140211
Sma-1-14058	NW_003037807.1	-	141383	141334
Sma-1-14059	NS_000200.1	+	30401678	30401727
Sma-1-14060	NS_000200.1	-	222327114	222327065
Sma-1-14061	NS_000200.1	-	30402899	30402850
Sma-1-14062	NW_003038067.1	-	108558	108509
Sma-1-14063	NS_000200.1	-	262962611	262962562
Sma-1-14064	NW_003037894.1	-	508706	508657
Sma-1-14065	NS_000200.1	-	142405279	142405230
Sma-1-14066	NW_003038507.1	+	98799	98848
Sma-1-14067	NS_000200.1	+	336767693	336767742
Sma-1-14068	NW_003037810.1	-	400247	400198
Sma-1-14069	NW_003038135.1	+	191361	191410
Sma-1-14070	NS_000200.1	+	284063700	284063749
Sma-1-14071	NS_000200.1	-	37004769	37004720
Sma-1-14072	NW_003038146.1	+	337242	337291
Sma-1-14073	NS_000200.1	+	289317912	289317961
Sma-1-14074	NW_003035580.1	+	7380	7429
Sma-1-14075	NS_000200.1	+	329088974	329089023
Sma-1-14076	NW_003038037.1	+	777305	777354
Sma-1-14077	NS_000200.1	+	250932714	250932763
Sma-1-14078	NW_003026617.1	+	242981	243030
Sma-1-14079	NW_003037818.1	+	206344	206393
Sma-1-14080	NW_003037862.1	-	265524	265475
Sma-1-14081	NW_003037918.1	-	253979	253930
Sma-1-14082	NW_003037987.1	-	34389	34340
Sma-1-14083	NW_003038018.1	+	76876	76925
Sma-1-14084	NW_003038037.1	-	1618528	1618479
Sma-1-14085	NW_003038254.1	+	193984	194033
Sma-1-14086	NS_000200.1	+	48921751	48921800
Sma-1-14087	NS_000200.1	+	232477726	232477775
Sma-1-14088	NS_000200.1	+	319813840	319813889
Sma-1-14089	NS_000200.1	+	320229542	320229591
Sma-1-14090	NS_000200.1	-	251773937	251773888
Sma-1-14091	NS_000200.1	-	215787010	215786961
Sma-1-14092	NS_000200.1	-	166820969	166820920

Sma-1-14093	NS_000200.1	-	105917185	105917136
Sma-1-14094	NW_003032503.1	-	238	189
Sma-1-14095	NW_003026608.1	-	18196	18147
Sma-1-14096	NW_003037793.1	+	3238922	3238971
Sma-1-14097	NW_003037914.1	-	1645200	1645151
Sma-1-14098	NS_000200.1	+	3238922	3238971
Sma-1-14099	NS_000200.1	-	346196952	346196903
Sma-1-14100	NS_000200.1	-	309296316	309296267
Sma-1-14101	NS_000200.1	-	161789450	161789401
Sma-1-14102	NW_003037938.1	+	4378	4427
Sma-1-14103	NW_003038035.1	-	908420	908371
Sma-1-14104	NS_000200.1	+	182041491	182041540
Sma-1-14105	NS_000200.1	-	248769912	248769863
Sma-1-14106	NW_003026456.1	+	311783	311832
Sma-1-14107	NS_000200.1	+	58234919	58234968
Sma-1-14108	NW_003038024.1	+	701376	701425
Sma-1-14109	NS_000200.1	+	238598405	238598454
Sma-1-14110	NW_003038037.1	-	528498	528449
Sma-1-14111	NS_000200.1	-	250683907	250683858
Sma-1-14112	NW_003021469.1	-	245	196
Sma-1-14113	NW_003038070.1	+	75857	75906
Sma-1-14114	NW_003037809.1	-	585508	585459
Sma-1-14115	NW_003037923.1	-	393722	393673
Sma-1-14116	NW_003038067.1	-	94970	94921
Sma-1-14117	NS_000200.1	+	264413098	264413147
Sma-1-14118	NS_000200.1	-	344131267	344131218
Sma-1-14119	NS_000200.1	-	262949023	262948974
Sma-1-14120	NS_000200.1	-	171449935	171449886
Sma-1-14121	NS_000200.1	-	34249972	34249923
Sma-1-14122	NW_003037818.1	+	2019735	2019784
Sma-1-14123	NS_000200.1	+	50735142	50735191
Sma-1-14124	NW_003038037.1	-	1211748	1211699
Sma-1-14125	NS_000200.1	-	251367157	251367108
Sma-1-14126	NW_003037844.1	-	27053	27004
Sma-1-14127	NS_000200.1	-	86210499	86210450
Sma-1-14128	NW_003037793.1	+	153640	153689
Sma-1-14129	NS_000200.1	+	153640	153689
Sma-1-14130	NW_003037796.1	-	243166	243117
Sma-1-14131	NS_000200.1	-	8452810	8452761
Sma-1-14132	NW_003037792.1	-	265664	265615
Sma-1-14133	NS_000200.1	-	382703008	382702959
Sma-1-14134	NW_003037812.1	+	1082029	1082078
Sma-1-14135	NS_000200.1	+	40564768	40564817
Sma-1-14136	NW_003037911.1	-	20086	20037
Sma-1-14137	NW_003038240.1	+	92467	92516
Sma-1-14138	NW_003038086.1	-	610608	610559
Sma-1-14139	NS_000200.1	+	314231398	314231447
Sma-1-14140	NS_000200.1	-	272859201	272859152
Sma-1-14141	NS_000200.1	-	155807439	155807390
Sma-1-14142	NW_003037911.1	-	334468	334419
Sma-1-14143	NS_000200.1	-	156121821	156121772
Sma-1-14144	NW_003037817.1	+	987740	987789

Sma-1-14145	NS_000200.1	+	48370092	48370141
Sma-1-14146	NW_003037918.1	+	184571	184620
Sma-1-14147	NS_000200.1	+	166751561	166751610
Sma-1-14148	NW_003038010.1	+	517711	517760
Sma-1-14149	NS_000200.1	+	227335934	227335983
Sma-1-14150	NW_003035879.1	+	186726	186775
Sma-1-14151	NW_003039026.1	-	3537	3488
Sma-1-14152	NW_003035639.1	-	39876	39827
Sma-1-14153	NW_003035661.1	-	35158	35109
Sma-1-14154	NW_003037809.1	-	2430954	2430905
Sma-1-14155	NW_003037812.1	-	1121364	1121315
Sma-1-14156	NW_003037827.1	-	1610691	1610642
Sma-1-14157	NW_003037827.1	-	1604393	1604344
Sma-1-14158	NW_003037851.1	+	278701	278750
Sma-1-14159	NW_003037865.1	-	78035	77986
Sma-1-14160	NW_003037877.1	-	403597	403548
Sma-1-14161	NW_003037888.1	-	229921	229872
Sma-1-14162	NW_003037928.1	+	183851	183900
Sma-1-14163	NW_003037928.1	+	549429	549478
Sma-1-14164	NW_003037942.1	+	662527	662576
Sma-1-14165	NW_003038030.1	+	234172	234221
Sma-1-14166	NW_003038037.1	+	1136384	1136433
Sma-1-14167	NW_003038071.1	-	202021	201972
Sma-1-14168	NW_003038506.1	-	184074	184025
Sma-1-14169	NW_003026488.1	-	70740	70691
Sma-1-14170	NW_003038074.1	+	124828	124877
Sma-1-14171	NW_003038086.1	-	351798	351749
Sma-1-14172	NW_003038156.1	-	475619	475570
Sma-1-14173	NS_000200.1	+	93682011	93682060
Sma-1-14174	NS_000200.1	+	174507804	174507853
Sma-1-14175	NS_000200.1	+	174873382	174873431
Sma-1-14176	NS_000200.1	+	186422435	186422484
Sma-1-14177	NS_000200.1	+	243370039	243370088
Sma-1-14178	NS_000200.1	+	251291793	251291842
Sma-1-14179	NS_000200.1	+	266016208	266016257
Sma-1-14180	NS_000200.1	+	338154053	338154102
Sma-1-14181	NS_000200.1	-	358752482	358752433
Sma-1-14182	NS_000200.1	-	336545913	336545864
Sma-1-14183	NS_000200.1	-	330985844	330985795
Sma-1-14184	NS_000200.1	-	329990316	329990267
Sma-1-14185	NS_000200.1	-	295207732	295207683
Sma-1-14186	NS_000200.1	-	272600391	272600342
Sma-1-14187	NS_000200.1	-	265005064	265005015
Sma-1-14188	NS_000200.1	-	263577234	263577185
Sma-1-14189	NS_000200.1	-	132438413	132438364
Sma-1-14190	NS_000200.1	-	121442159	121442110
Sma-1-14191	NS_000200.1	-	110499954	110499905
Sma-1-14192	NS_000200.1	-	64232832	64232783
Sma-1-14193	NS_000200.1	-	64226534	64226485
Sma-1-14194	NS_000200.1	-	40604103	40604054
Sma-1-14195	NS_000200.1	-	36095418	36095369
Sma-1-14196	NW_003037915.1	-	1056094	1056045

Sma-1-14197	NS_000200.1	-	162956080	162956031
Sma-1-14198	NW_003037928.1	+	337917	337966
Sma-1-14199	NS_000200.1	+	174661870	174661919
Sma-1-14200	NW_003037849.1	-	965341	965292
Sma-1-14201	NS_000200.1	-	91941614	91941565
Sma-1-14202	NW_003037804.1	-	653838	653789
Sma-1-14203	NW_003037900.1	-	638614	638565
Sma-1-14204	NS_000200.1	-	145767480	145767431
Sma-1-14205	NS_000200.1	-	25793858	25793809
Sma-1-14206	NW_003038396.1	-	4125	4076
Sma-1-14207	NW_003037839.1	+	1273342	1273391
Sma-1-14208	NS_000200.1	+	80231492	80231541
Sma-1-14209	NS_000200.1	-	328073555	328073506
Sma-1-14210	NW_003038050.1	+	219187	219236
Sma-1-14211	NS_000200.1	+	256494081	256494130
Sma-1-14212	NW_003037977.1	-	187586	187537
Sma-1-14213	NS_000200.1	-	208473606	208473557
Sma-1-14214	NW_003026512.1	-	247167	247118
Sma-1-14215	NS_000200.1	-	285341207	285341158
Sma-1-14216	NW_003038259.1	+	55591	55640
Sma-1-14217	NS_000200.1	+	322916190	322916239
Sma-1-14218	NW_003038084.1	+	288533	288582
Sma-1-14219	NS_000200.1	+	271303777	271303826
Sma-1-14220	NW_003037975.1	-	37132	37083
Sma-1-14221	NS_000200.1	-	205619356	205619307
Sma-1-14222	NW_003037810.1	-	1270174	1270125
Sma-1-14223	NS_000200.1	-	37874696	37874647
Sma-1-14224	NW_003037918.1	-	221674	221625
Sma-1-14225	NS_000200.1	-	166788664	166788615
Sma-1-14226	NW_003037928.1	-	599225	599176
Sma-1-14227	NS_000200.1	-	174923178	174923129
Sma-1-14228	NW_003037795.1	-	387377	387328
Sma-1-14229	NS_000200.1	-	6778260	6778211
Sma-1-14230	NW_003037377.1	+	2	51
Sma-1-14231	NS_000200.1	+	367309654	367309703
Sma-1-14232	NW_003038005.1	-	150923	150874
Sma-1-14233	NW_003037822.1	+	1227083	1227132
Sma-1-14234	NS_000200.1	+	55535089	55535138
Sma-1-14235	NS_000200.1	-	224556877	224556828
Sma-1-14236	NW_003020783.1	-	1634	1585
Sma-1-14237	NS_000200.1	-	364450146	364450097
Sma-1-14238	NW_003037809.1	-	1799596	1799547
Sma-1-14239	NS_000200.1	-	35464060	35464011
Sma-1-14240	NW_003037928.1	-	605906	605857
Sma-1-14241	NS_000200.1	-	174929859	174929810
Sma-1-14242	NW_003038011.1	+	395874	395923
Sma-1-14243	NS_000200.1	+	227923524	227923573
Sma-1-14244	NW_003038113.1	+	35373	35422
Sma-1-14245	NS_000200.1	+	276696235	276696284
Sma-1-14246	NW_003037795.1	+	834289	834338
Sma-1-14247	NS_000200.1	+	7225172	7225221
Sma-1-14248	NW_003037944.1	-	707387	707338

Sma-1-14249	NS_000200.1	-	188928740	188928691
Sma-1-14250	NW_003029798.1	-	2468	2419
Sma-1-14251	NS_000200.1	-	375636214	375636165
Sma-1-14252	NW_003037820.1	+	64400	64449
Sma-1-14253	NW_003037825.1	-	1395868	1395819
Sma-1-14254	NW_003037923.1	-	119163	119114
Sma-1-14255	NS_000200.1	+	52337382	52337431
Sma-1-14256	NS_000200.1	-	171175376	171175327
Sma-1-14257	NS_000200.1	-	60525650	60525601
Sma-1-14258	NW_003037967.1	-	447378	447329
Sma-1-14259	NS_000200.1	-	199687349	199687300
Sma-1-14260	NW_003037875.1	-	487237	487188
Sma-1-14261	NW_003037797.1	-	778083	778034
Sma-1-14262	NW_003037804.1	+	237296	237345
Sma-1-14263	NW_003037886.1	+	150702	150751
Sma-1-14264	NS_000200.1	+	25377316	25377365
Sma-1-14265	NS_000200.1	+	129406269	129406318
Sma-1-14266	NS_000200.1	-	120515906	120515857
Sma-1-14267	NS_000200.1	-	11205943	11205894
Sma-1-14268	NW_003027937.1	-	1303	1254
Sma-1-14269	NW_003037790.1	-	225047	224998
Sma-1-14270	NS_000200.1	-	381569219	381569170
Sma-1-14271	NS_000200.1	-	369030159	369030110
Sma-1-14272	NW_003026619.1	+	16786	16835
Sma-1-14273	NS_000200.1	+	322207530	322207579
Sma-1-14274	NW_003037976.1	-	64926	64877
Sma-1-14275	NS_000200.1	-	206679241	206679192
Sma-1-14276	NW_003024200.1	-	799	750
Sma-1-14277	NW_003037873.1	-	58187	58138
Sma-1-14278	NW_003037798.1	-	1926752	1926703
Sma-1-14279	NS_000200.1	-	348549703	348549654
Sma-1-14280	NS_000200.1	-	118797315	118797266
Sma-1-14281	NS_000200.1	-	16185910	16185861
Sma-1-14282	NW_003026477.1	-	37151	37102
Sma-1-14283	NS_000200.1	-	253914560	253914511
Sma-1-14284	NW_003026498.1	-	37845	37796
Sma-1-14285	NS_000200.1	-	275025700	275025651
Sma-1-14286	NW_003038086.1	+	667472	667521
Sma-1-14287	NS_000200.1	+	272916065	272916114
Sma-1-14288	NW_003037833.1	-	903171	903122
Sma-1-14289	NW_003037954.1	-	20852	20803
Sma-1-14290	NW_003038086.1	+	666025	666074
Sma-1-14291	NS_000200.1	+	272914618	272914667
Sma-1-14292	NS_000200.1	-	193250474	193250425
Sma-1-14293	NS_000200.1	-	73094267	73094218
Sma-1-14294	NW_003026462.1	-	542072	542023
Sma-1-14295	NS_000200.1	-	197209575	197209526
Sma-1-14296	NW_003026460.1	+	17138	17187
Sma-1-14297	NS_000200.1	+	170539344	170539393
Sma-1-14298	NW_003037995.1	+	172215	172264
Sma-1-14299	NS_000200.1	+	219861983	219862032
Sma-1-14300	NW_003024960.1	-	393	344

Sma-1-14301	NW_003035672.1	-	9384	9335
Sma-1-14302	NW_003035880.1	+	34200	34249
Sma-1-14303	NW_003037890.1	-	1591647	1591598
Sma-1-14304	NS_000200.1	+	339275264	339275313
Sma-1-14305	NS_000200.1	-	355811638	355811589
Sma-1-14306	NS_000200.1	-	331679474	331679425
Sma-1-14307	NS_000200.1	-	137275008	137274959
Sma-1-14308	NW_003037997.1	-	386852	386803
Sma-1-14309	NS_000200.1	-	221198294	221198245
Sma-1-14310	NW_003038032.1	-	441123	441074
Sma-1-14311	NS_000200.1	-	245600283	245600234
Sma-1-14312	NW_003021735.1	+	99	148
Sma-1-14313	NW_003022562.1	+	1729	1778
Sma-1-14314	NW_003032958.1	-	1669	1620
Sma-1-14315	NW_003035821.1	+	1946	1995
Sma-1-14316	NW_003036001.1	+	1375	1424
Sma-1-14317	NW_003036329.1	-	87	38
Sma-1-14318	NW_003036788.1	-	1068	1019
Sma-1-14319	NW_003037751.1	-	690	641
Sma-1-14320	NW_003032145.1	+	101335	101384
Sma-1-14321	NW_003032145.1	+	101741	101790
Sma-1-14322	NW_003026465.1	-	446097	446048
Sma-1-14323	NW_003037723.1	+	4281	4330
Sma-1-14324	NW_003026459.1	-	204312	204263
Sma-1-14325	NW_003037793.1	-	386201	386152
Sma-1-14326	NW_003037795.1	-	367894	367845
Sma-1-14327	NW_003037797.1	+	614399	614448
Sma-1-14328	NW_003037809.1	+	1789140	1789189
Sma-1-14329	NW_003037810.1	+	1223281	1223330
Sma-1-14330	NW_003037838.1	+	739522	739571
Sma-1-14331	NW_003037846.1	+	887105	887154
Sma-1-14332	NW_003037850.1	-	238102	238053
Sma-1-14333	NW_003037858.1	-	457684	457635
Sma-1-14334	NW_003037871.1	+	218274	218323
Sma-1-14335	NW_003037909.1	+	337286	337335
Sma-1-14336	NW_003037914.1	+	63209	63258
Sma-1-14337	NW_003037916.1	+	41099	41148
Sma-1-14338	NW_003037916.1	+	1736966	1737015
Sma-1-14339	NW_003037926.1	+	397495	397544
Sma-1-14340	NW_003038020.1	+	419883	419932
Sma-1-14341	NW_003038033.1	-	709884	709835
Sma-1-14342	NW_003038037.1	+	711938	711987
Sma-1-14343	NW_003038037.1	+	1163364	1163413
Sma-1-14344	NW_003038513.1	+	162925	162974
Sma-1-14345	NW_003026474.1	+	40576	40625
Sma-1-14346	NW_003026515.1	-	131565	131516
Sma-1-14347	NW_003038136.1	-	260707	260658
Sma-1-14348	NS_000200.1	+	11042259	11042308
Sma-1-14349	NS_000200.1	+	35453604	35453653
Sma-1-14350	NS_000200.1	+	37827803	37827852
Sma-1-14351	NS_000200.1	+	78738085	78738134
Sma-1-14352	NS_000200.1	+	88521675	88521724

Sma-1-14353	NS_000200.1	+	117666012	117666061
Sma-1-14354	NS_000200.1	+	153878935	153878984
Sma-1-14355	NS_000200.1	+	160207459	160207508
Sma-1-14356	NS_000200.1	+	163234510	163234559
Sma-1-14357	NS_000200.1	+	164930377	164930426
Sma-1-14358	NS_000200.1	+	173565765	173565814
Sma-1-14359	NS_000200.1	+	234387227	234387276
Sma-1-14360	NS_000200.1	+	250867347	250867396
Sma-1-14361	NS_000200.1	+	251318773	251318822
Sma-1-14362	NS_000200.1	+	253239151	253239200
Sma-1-14363	NS_000200.1	+	339172102	339172151
Sma-1-14364	NS_000200.1	+	343238530	343238579
Sma-1-14365	NS_000200.1	+	351768069	351768118
Sma-1-14366	NS_000200.1	+	364166555	364166604
Sma-1-14367	NS_000200.1	+	365186685	365186734
Sma-1-14368	NS_000200.1	+	376385800	376385849
Sma-1-14369	NS_000200.1	+	378800900	378800949
Sma-1-14370	NS_000200.1	+	378801306	378801355
Sma-1-14371	NS_000200.1	-	377039135	377039086
Sma-1-14372	NS_000200.1	-	359642897	359642848
Sma-1-14373	NS_000200.1	-	347654077	347654028
Sma-1-14374	NS_000200.1	-	326079170	326079121
Sma-1-14375	NS_000200.1	-	290775976	290775927
Sma-1-14376	NS_000200.1	-	284382776	284382727
Sma-1-14377	NS_000200.1	-	246689886	246689837
Sma-1-14378	NS_000200.1	-	207838691	207838642
Sma-1-14379	NS_000200.1	-	141849585	141849536
Sma-1-14380	NS_000200.1	-	101121960	101121911
Sma-1-14381	NS_000200.1	-	92224132	92224083
Sma-1-14382	NS_000200.1	-	6758777	6758728
Sma-1-14383	NS_000200.1	-	386201	386152
Sma-1-14384	NW_003038084.1	+	355089	355138
Sma-1-14385	NS_000200.1	+	271370333	271370382
Sma-1-14386	NW_003023837.1	+	32	81
Sma-1-14387	NW_003037800.1	+	1017718	1017767
Sma-1-14388	NS_000200.1	+	19647078	19647127
Sma-1-14389	NS_000200.1	+	368888301	368888350
Sma-1-14390	NW_003037798.1	+	2278824	2278873
Sma-1-14391	NS_000200.1	+	16537982	16538031
Sma-1-14392	NW_003037807.1	+	412718	412767
Sma-1-14393	NS_000200.1	+	30674234	30674283
Sma-1-14394	NW_003037888.1	-	1501658	1501609
Sma-1-14395	NS_000200.1	-	133710150	133710101
Sma-1-14396	NW_003037891.1	+	2158220	2158269
Sma-1-14397	NS_000200.1	+	140039498	140039547
Sma-1-14398	NW_003037873.1	+	553954	554003
Sma-1-14399	NW_003026472.1	+	699301	699350
Sma-1-14400	NW_003037998.1	+	9428	9477
Sma-1-14401	NS_000200.1	+	119293082	119293131
Sma-1-14402	NS_000200.1	+	221296024	221296073
Sma-1-14403	NS_000200.1	+	237111293	237111342
Sma-1-14404	NW_003037841.1	+	191345	191394

Sma-1-14405	NS_000200.1	+	82802526	82802575
Sma-1-14406	NW_003037851.1	-	612467	612418
Sma-1-14407	NS_000200.1	-	94015777	94015728
Sma-1-14408	NW_003037861.1	-	436660	436611
Sma-1-14409	NS_000200.1	-	105213114	105213065
Sma-1-14410	NW_003037863.1	-	341150	341101
Sma-1-14411	NS_000200.1	-	107099517	107099468
Sma-1-14412	NW_003037863.1	+	1031140	1031189
Sma-1-14413	NS_000200.1	+	107789507	107789556
Sma-1-14414	NW_003037887.1	+	1618716	1618765
Sma-1-14415	NW_003038150.1	+	411929	411978
Sma-1-14416	NS_000200.1	+	132082694	132082743
Sma-1-14417	NS_000200.1	+	291890667	291890716
Sma-1-14418	NW_003037888.1	+	117113	117162
Sma-1-14419	NS_000200.1	+	132325605	132325654
Sma-1-14420	NW_003037860.1	+	830550	830599
Sma-1-14421	NS_000200.1	+	104573578	104573627
Sma-1-14422	NW_003038247.1	+	117149	117198
Sma-1-14423	NS_000200.1	+	316065877	316065926
Sma-1-14424	NW_003037800.1	+	1260306	1260355
Sma-1-14425	NS_000200.1	+	19889666	19889715
Sma-1-14426	NW_003038131.1	-	138637	138588
Sma-1-14427	NS_000200.1	-	282100481	282100432
Sma-1-14428	NW_003037807.1	-	595020	594971
Sma-1-14429	NW_003037827.1	-	2331489	2331440
Sma-1-14430	NS_000200.1	-	64953630	64953581
Sma-1-14431	NS_000200.1	-	30856536	30856487
Sma-1-14432	NW_003037827.1	-	969529	969480
Sma-1-14433	NS_000200.1	-	63591670	63591621
Sma-1-14434	NW_003037967.1	-	55477	55428
Sma-1-14435	NS_000200.1	-	199295448	199295399
Sma-1-14436	NW_003037916.1	-	196078	196029
Sma-1-14437	NS_000200.1	-	163389489	163389440
Sma-1-14438	NW_003037829.1	-	627837	627788
Sma-1-14439	NS_000200.1	-	66528375	66528326
Sma-1-14440	NW_003037799.1	-	1019328	1019279
Sma-1-14441	NS_000200.1	-	18009682	18009633
Sma-1-14442	NW_003037970.1	-	158725	158676
Sma-1-14443	NS_000200.1	-	201214915	201214866
Sma-1-14444	NW_003038250.1	-	71737	71688
Sma-1-14445	NW_003037967.1	-	647608	647559
Sma-1-14446	NS_000200.1	-	318124414	318124365
Sma-1-14447	NS_000200.1	-	199887579	199887530
Sma-1-14448	NW_003037793.1	-	3467381	3467332
Sma-1-14449	NS_000200.1	-	3467381	3467332
Sma-1-14450	NW_003037913.1	-	318111	318062
Sma-1-14451	NS_000200.1	-	158881862	158881813
Sma-1-14452	NW_003037808.1	-	508935	508886
Sma-1-14453	NS_000200.1	-	32295572	32295523
Sma-1-14454	NW_003037917.1	-	1447945	1447896
Sma-1-14455	NS_000200.1	-	166484749	166484700
Sma-1-14456	NW_003037806.1	-	653568	653519

Sma-1-14457	NS_000200.1	-	28848782	28848733
Sma-1-14458	NW_003037973.1	-	16303	16254
Sma-1-14459	NS_000200.1	-	203998030	203997981
Sma-1-14460	NW_003038086.1	-	265571	265522
Sma-1-14461	NS_000200.1	-	272514164	272514115
Sma-1-14462	NW_003026456.1	+	98342	98391
Sma-1-14463	NW_003037982.1	+	1076392	1076441
Sma-1-14464	NS_000200.1	+	58021478	58021527
Sma-1-14465	NS_000200.1	+	214735587	214735636
Sma-1-14466	NW_003037928.1	+	44106	44155
Sma-1-14467	NS_000200.1	+	174368059	174368108
Sma-1-14468	NW_003037969.1	+	208602	208651
Sma-1-14469	NS_000200.1	+	200814729	200814778
Sma-1-14470	NW_003026460.1	+	141083	141132
Sma-1-14471	NS_000200.1	+	170663289	170663338
Sma-1-14472	NW_003038151.1	-	84160	84111
Sma-1-14473	NS_000200.1	-	291991403	291991354
Sma-1-14474	NW_003038042.1	+	94769	94818
Sma-1-14475	NS_000200.1	+	254040368	254040417
Sma-1-14476	NW_003023253.1	-	176	127
Sma-1-14477	NW_003038514.1	+	194131	194180
Sma-1-14478	NW_003026618.1	+	426788	426837
Sma-1-14479	NW_003026465.1	+	741113	741162
Sma-1-14480	NW_003037794.1	-	1832658	1832609
Sma-1-14481	NW_003037821.1	-	969315	969266
Sma-1-14482	NW_003037833.1	-	539325	539276
Sma-1-14483	NW_003037863.1	-	579855	579806
Sma-1-14484	NW_003037916.1	+	462538	462587
Sma-1-14485	NW_003037916.1	+	493508	493557
Sma-1-14486	NW_003037925.1	-	314375	314326
Sma-1-14487	NW_003038008.1	-	622955	622906
Sma-1-14488	NW_003038251.1	-	62452	62403
Sma-1-14489	NW_003038507.1	+	84405	84454
Sma-1-14490	NW_003026574.1	+	96204	96253
Sma-1-14491	NW_003038138.1	-	311469	311420
Sma-1-14492	NS_000200.1	+	163655949	163655998
Sma-1-14493	NS_000200.1	+	163686919	163686968
Sma-1-14494	NS_000200.1	+	208133707	208133756
Sma-1-14495	NS_000200.1	+	301903537	301903586
Sma-1-14496	NS_000200.1	+	322181790	322181839
Sma-1-14497	NS_000200.1	+	336753299	336753348
Sma-1-14498	NS_000200.1	+	339818670	339818719
Sma-1-14499	NS_000200.1	-	368563216	368563167
Sma-1-14500	NS_000200.1	-	318394224	318394175
Sma-1-14501	NS_000200.1	-	285866784	285866735
Sma-1-14502	NS_000200.1	-	226296051	226296002
Sma-1-14503	NS_000200.1	-	172716378	172716329
Sma-1-14504	NS_000200.1	-	107338222	107338173
Sma-1-14505	NS_000200.1	-	72730421	72730372
Sma-1-14506	NS_000200.1	-	54113799	54113750
Sma-1-14507	NS_000200.1	-	6012253	6012204
Sma-1-14508	NW_003037797.1	-	3263590	3263541

Sma-1-14509	NS_000200.1	-	13691450	13691401
Sma-1-14510	NW_003038016.1	+	289331	289380
Sma-1-14511	NS_000200.1	+	231405868	231405917
Sma-1-14512	NW_003038020.1	+	726502	726551
Sma-1-14513	NS_000200.1	+	234693846	234693895
Sma-1-14514	NW_003037906.1	+	68613	68662
Sma-1-14515	NS_000200.1	+	150376392	150376441
Sma-1-14516	NW_003038049.1	-	173401	173352
Sma-1-14517	NS_000200.1	-	256148928	256148879
Sma-1-14518	NW_003037577.1	+	4	53
Sma-1-14519	NS_000200.1	+	372211081	372211130
Sma-1-14520	NW_003037999.1	+	270322	270373
Sma-1-14521	NS_000200.1	+	221964876	221964927
Sma-1-14522	NW_003037818.1	+	1731145	1731194
Sma-1-14523	NW_003037853.1	-	322333	322284
Sma-1-14524	NS_000200.1	+	50446552	50446601
Sma-1-14525	NS_000200.1	-	95814384	95814335
Sma-1-14526	NW_003037814.1	-	171911	171862
Sma-1-14527	NS_000200.1	-	43155771	43155722
Sma-1-14528	NW_003037873.1	+	576594	576643
Sma-1-14529	NS_000200.1	+	119315722	119315771
Sma-1-14530	NW_003037908.1	-	129409	129360
Sma-1-14531	NS_000200.1	-	152602540	152602491
Sma-1-14532	NW_003037896.1	-	331130	331081
Sma-1-14533	NS_000200.1	-	143251553	143251504
Sma-1-14534	NW_003037838.1	-	565883	565834
Sma-1-14535	NS_000200.1	-	78564446	78564397
Sma-1-14536	NW_003037827.1	-	2266185	2266136
Sma-1-14537	NS_000200.1	-	64888326	64888277
Sma-1-14538	NW_003037825.1	-	359004	358955
Sma-1-14539	NW_003037825.1	-	358859	358810
Sma-1-14540	NS_000200.1	-	59488786	59488737
Sma-1-14541	NS_000200.1	-	59488641	59488592
Sma-1-14542	NW_003037824.1	-	243241	243192
Sma-1-14543	NS_000200.1	-	57174181	57174132
Sma-1-14544	NW_003037819.1	-	908765	908716
Sma-1-14545	NS_000200.1	-	51997030	51996981
Sma-1-14546	NW_003038129.1	+	34027	34076
Sma-1-14547	NS_000200.1	+	281287374	281287423
Sma-1-14548	NW_003038053.1	+	440461	440510
Sma-1-14549	NS_000200.1	+	257800556	257800605
Sma-1-14550	NW_003038087.1	-	524220	524171
Sma-1-14551	NS_000200.1	-	273552621	273552572
Sma-1-14552	NW_003038160.1	-	246452	246403
Sma-1-14553	NS_000200.1	-	296998383	296998334
Sma-1-14554	NW_003037831.1	-	826452	826403
Sma-1-14555	NW_003037887.1	-	965757	965708
Sma-1-14556	NS_000200.1	-	131429735	131429686
Sma-1-14557	NS_000200.1	-	70275754	70275705
Sma-1-14558	NW_003037915.1	+	611921	611970
Sma-1-14559	NS_000200.1	+	162511907	162511956
Sma-1-14560	NW_003037916.1	-	248284	248235

Sma-1-14561	NS_000200.1	-	163441695	163441646
Sma-1-14562	NW_003037800.1	-	252311	252262
Sma-1-14563	NS_000200.1	-	18881671	18881622
Sma-1-14564	NW_003037939.1	+	768317	768366
Sma-1-14565	NS_000200.1	+	183638071	183638120
Sma-1-14566	NW_003037827.1	+	456903	456952
Sma-1-14567	NW_003037938.1	+	411262	411311
Sma-1-14568	NS_000200.1	+	63079044	63079093
Sma-1-14569	NS_000200.1	+	182448375	182448424
Sma-1-14570	NW_003035677.1	-	59	10
Sma-1-14571	NS_000200.1	-	331792728	331792679
Sma-1-14572	NW_003037953.1	-	170802	170753
Sma-1-14573	NS_000200.1	-	193083853	193083804
Sma-1-14574	NW_003037979.1	-	172139	172090
Sma-1-14575	NS_000200.1	-	210233159	210233110
Sma-1-14576	NW_003038257.1	+	352553	352602
Sma-1-14577	NS_000200.1	+	321526881	321526930
Sma-1-14578	NW_003037846.1	+	384765	384814
Sma-1-14579	NW_003037904.1	-	479579	479530
Sma-1-14580	NW_003038252.1	-	354992	354943
Sma-1-14581	NS_000200.1	+	88019335	88019384
Sma-1-14582	NS_000200.1	-	319210580	319210531
Sma-1-14583	NS_000200.1	-	149194078	149194029
Sma-1-14584	NW_003038032.1	-	308726	308677
Sma-1-14585	NS_000200.1	-	245467886	245467837
Sma-1-14586	NW_003038123.1	+	230627	230676
Sma-1-14587	NS_000200.1	+	278978794	278978843
Sma-1-14588	NW_003037793.1	+	3619368	3619417
Sma-1-14589	NW_003037863.1	-	994684	994635
Sma-1-14590	NW_003037890.1	+	747967	748016
Sma-1-14591	NS_000200.1	+	3619368	3619417
Sma-1-14592	NS_000200.1	+	136431328	136431377
Sma-1-14593	NS_000200.1	-	107753051	107753002
Sma-1-14594	NW_003037806.1	+	1595121	1595170
Sma-1-14595	NS_000200.1	+	29790335	29790384
Sma-1-14596	NW_003037804.1	+	1364892	1364941
Sma-1-14597	NS_000200.1	+	26504912	26504961
Sma-1-14598	NW_003037923.1	+	247182	247231
Sma-1-14599	NS_000200.1	+	171303395	171303444
Sma-1-14600	NW_003035449.1	+	248	297
Sma-1-14601	NW_003037798.1	-	2386743	2386694
Sma-1-14602	NS_000200.1	+	367483755	367483804
Sma-1-14603	NS_000200.1	-	16645901	16645852
Sma-1-14604	NW_003037828.1	-	457531	457482
Sma-1-14605	NW_003037841.1	+	2100329	2100378
Sma-1-14606	NW_003037856.1	+	409982	410031
Sma-1-14607	NW_003037884.1	-	519427	519378
Sma-1-14608	NW_003037948.1	+	186540	186589
Sma-1-14609	NW_003038037.1	-	788204	788155
Sma-1-14610	NS_000200.1	+	84711510	84711559
Sma-1-14611	NS_000200.1	+	99767938	99767987
Sma-1-14612	NS_000200.1	+	192206484	192206533

Sma-1-14613	NS_000200.1	-	250943613	250943564
Sma-1-14614	NS_000200.1	-	127299092	127299043
Sma-1-14615	NS_000200.1	-	65547016	65546967
Sma-1-14616	NW_003037795.1	+	1220826	1220875
Sma-1-14617	NS_000200.1	+	7611709	7611758
Sma-1-14618	NW_003037973.1	-	152114	152065
Sma-1-14619	NS_000200.1	-	204133841	204133792
Sma-1-14620	NW_003037981.1	-	902105	902056
Sma-1-14621	NS_000200.1	-	213299973	213299924
Sma-1-14622	NW_003038478.1	-	38017	37968
Sma-1-14623	NW_003037831.1	+	1759319	1759368
Sma-1-14624	NW_003038076.1	-	138395	138346
Sma-1-14625	NS_000200.1	+	71208621	71208670
Sma-1-14626	NS_000200.1	-	332613051	332613002
Sma-1-14627	NS_000200.1	-	266355569	266355520
Sma-1-14628	NW_003038509.1	+	259255	259304
Sma-1-14629	NS_000200.1	+	337891707	337891756
Sma-1-14630	NW_003026466.1	-	130232	130183
Sma-1-14631	NS_000200.1	-	215397339	215397290
Sma-1-14632	NW_003026456.1	-	148687	148638
Sma-1-14633	NW_003037816.1	+	500295	500344
Sma-1-14634	NW_003038261.1	-	733105	733056
Sma-1-14635	NW_003026516.1	+	341204	341253
Sma-1-14636	NW_003038041.1	-	8756	8707
Sma-1-14637	NW_003038156.1	+	106735	106784
Sma-1-14638	NS_000200.1	+	46709167	46709216
Sma-1-14639	NS_000200.1	+	294838848	294838897
Sma-1-14640	NS_000200.1	+	299449501	299449550
Sma-1-14641	NS_000200.1	-	324346756	324346707
Sma-1-14642	NS_000200.1	-	253676038	253675989
Sma-1-14643	NS_000200.1	-	58071823	58071774
Sma-1-14644	NW_003021804.1	+	996	1045
Sma-1-14645	NS_000200.1	+	351888076	351888125
Sma-1-14646	NW_003026456.1	+	410419	410468
Sma-1-14647	NW_003037890.1	-	1175289	1175240
Sma-1-14648	NW_003037972.1	+	237833	237882
Sma-1-14649	NW_003037978.1	+	470107	470156
Sma-1-14650	NW_003026576.1	-	4670	4621
Sma-1-14651	NS_000200.1	+	58333555	58333604
Sma-1-14652	NS_000200.1	+	202713537	202713586
Sma-1-14653	NS_000200.1	+	209738117	209738166
Sma-1-14654	NS_000200.1	-	302632246	302632197
Sma-1-14655	NS_000200.1	-	136858650	136858601
Sma-1-14656	NW_003039127.1	+	362631	362680
Sma-1-14657	NS_000200.1	+	113440130	113440179
Sma-1-14658	NW_003038369.1	+	6889	6938
Sma-1-14659	NS_000200.1	+	327115828	327115877
Sma-1-14660	NW_003025279.1	-	477	428
Sma-1-14661	NW_003034511.1	-	1088	1039
Sma-1-14662	NW_003037995.1	+	307553	307602
Sma-1-14663	NW_003037793.1	+	2731136	2731185
Sma-1-14664	NW_003037795.1	+	1587293	1587342

Sma-1-14665	NW_003037795.1	+	1608481	1608530
Sma-1-14666	NW_003037797.1	-	2699823	2699774
Sma-1-14667	NW_003037798.1	+	1837390	1837439
Sma-1-14668	NW_003037798.1	-	1332824	1332775
Sma-1-14669	NW_003037800.1	+	489183	489232
Sma-1-14670	NW_003037800.1	+	1609373	1609422
Sma-1-14671	NW_003037802.1	-	456626	456577
Sma-1-14672	NW_003037804.1	-	1275123	1275074
Sma-1-14673	NW_003037814.1	-	1359142	1359093
Sma-1-14674	NW_003037830.1	+	296847	296896
Sma-1-14675	NW_003037838.1	-	930386	930337
Sma-1-14676	NW_003037863.1	-	1019512	1019463
Sma-1-14677	NW_003037903.1	-	41163	41114
Sma-1-14678	NW_003037905.1	-	437814	437765
Sma-1-14679	NW_003037909.1	-	200916	200867
Sma-1-14680	NW_003037924.1	-	93219	93170
Sma-1-14681	NW_003037925.1	-	346877	346828
Sma-1-14682	NW_003037935.1	+	390107	390156
Sma-1-14683	NW_003037938.1	+	165507	165556
Sma-1-14684	NW_003037938.1	-	84504	84455
Sma-1-14685	NW_003037942.1	+	947693	947742
Sma-1-14686	NW_003037953.1	+	39209	39258
Sma-1-14687	NW_003037977.1	+	814248	814297
Sma-1-14688	NW_003038029.1	+	530878	530927
Sma-1-14689	NW_003038031.1	+	446461	446510
Sma-1-14690	NW_003038031.1	-	439672	439623
Sma-1-14691	NW_003038033.1	-	174198	174149
Sma-1-14692	NW_003038033.1	-	152293	152244
Sma-1-14693	NW_003038238.1	+	42155	42204
Sma-1-14694	NW_003038505.1	+	213015	213064
Sma-1-14695	NW_003038086.1	+	51657	51706
Sma-1-14696	NW_003038131.1	+	109359	109408
Sma-1-14697	NW_003038137.1	-	120983	120934
Sma-1-14698	NW_003038148.1	+	503097	503146
Sma-1-14699	NS_000200.1	+	2731136	2731185
Sma-1-14700	NS_000200.1	+	7978176	7978225
Sma-1-14701	NS_000200.1	+	7999364	7999413
Sma-1-14702	NS_000200.1	+	16096548	16096597
Sma-1-14703	NS_000200.1	+	19118543	19118592
Sma-1-14704	NS_000200.1	+	20238733	20238782
Sma-1-14705	NS_000200.1	+	68187218	68187267
Sma-1-14706	NS_000200.1	+	179806642	179806691
Sma-1-14707	NS_000200.1	+	182202620	182202669
Sma-1-14708	NS_000200.1	+	186707601	186707650
Sma-1-14709	NS_000200.1	+	192952260	192952309
Sma-1-14710	NS_000200.1	+	209100268	209100317
Sma-1-14711	NS_000200.1	+	219997321	219997370
Sma-1-14712	NS_000200.1	+	242731779	242731828
Sma-1-14713	NS_000200.1	+	244724219	244724268
Sma-1-14714	NS_000200.1	+	272300250	272300299
Sma-1-14715	NS_000200.1	+	282071203	282071252
Sma-1-14716	NS_000200.1	+	290577081	290577130

Sma-1-14717	NS_000200.1	+	313648936	313648985
Sma-1-14718	NS_000200.1	+	336292352	336292401
Sma-1-14719	NS_000200.1	-	361531199	361531150
Sma-1-14720	NS_000200.1	-	346855793	346855744
Sma-1-14721	NS_000200.1	-	284909879	284909830
Sma-1-14722	NS_000200.1	-	246154200	246154151
Sma-1-14723	NS_000200.1	-	246132295	246132246
Sma-1-14724	NS_000200.1	-	244717430	244717381
Sma-1-14725	NS_000200.1	-	182121617	182121568
Sma-1-14726	NS_000200.1	-	172748880	172748831
Sma-1-14727	NS_000200.1	-	171800177	171800128
Sma-1-14728	NS_000200.1	-	153742565	153742516
Sma-1-14729	NS_000200.1	-	149706826	149706777
Sma-1-14730	NS_000200.1	-	147595244	147595195
Sma-1-14731	NS_000200.1	-	107777879	107777830
Sma-1-14732	NS_000200.1	-	78928949	78928900
Sma-1-14733	NS_000200.1	-	44343002	44342953
Sma-1-14734	NS_000200.1	-	26415143	26415094
Sma-1-14735	NS_000200.1	-	22459420	22459371
Sma-1-14736	NS_000200.1	-	15591982	15591933
Sma-1-14737	NS_000200.1	-	13127683	13127634
Sma-1-14738	NW_003022296.1	-	1395	1346
Sma-1-14739	NW_003023545.1	+	1259	1308
Sma-1-14740	NW_003026545.1	+	640	689
Sma-1-14741	NW_003036329.1	+	711	760
Sma-1-14742	NW_003038504.1	+	106500	106549
Sma-1-14743	NW_003026458.1	-	256458	256409
Sma-1-14744	NW_003037810.1	+	1434826	1434875
Sma-1-14745	NW_003037818.1	+	1474757	1474806
Sma-1-14746	NW_003037827.1	+	2260801	2260850
Sma-1-14747	NW_003037830.1	+	187082	187131
Sma-1-14748	NW_003037834.1	+	465400	465449
Sma-1-14749	NW_003037839.1	-	1095610	1095561
Sma-1-14750	NW_003037840.1	+	475084	475133
Sma-1-14751	NW_003037844.1	+	460919	460968
Sma-1-14752	NW_003037854.1	+	390858	390907
Sma-1-14753	NW_003037864.1	+	1827607	1827656
Sma-1-14754	NW_003037879.1	-	590942	590893
Sma-1-14755	NW_003037887.1	+	413272	413321
Sma-1-14756	NW_003037894.1	-	230038	229989
Sma-1-14757	NW_003037906.1	+	583174	583223
Sma-1-14758	NW_003037914.1	-	1640491	1640442
Sma-1-14759	NW_003037930.1	-	221128	221079
Sma-1-14760	NW_003037943.1	-	885266	885217
Sma-1-14761	NW_003037976.1	-	570454	570405
Sma-1-14762	NW_003037978.1	-	107220	107171
Sma-1-14763	NW_003037980.1	-	141315	141266
Sma-1-14764	NW_003038003.1	+	350663	350712
Sma-1-14765	NW_003038030.1	-	211130	211081
Sma-1-14766	NW_003038037.1	-	1646825	1646776
Sma-1-14767	NW_003038260.1	+	53803	53852
Sma-1-14768	NW_003026517.1	+	182290	182339

Sma-1-14769	NW_003038077.1	-	423276	423227
Sma-1-14770	NW_003038153.1	+	597190	597239
Sma-1-14771	NW_003038177.1	-	43730	43681
Sma-1-14772	NS_000200.1	+	38039348	38039397
Sma-1-14773	NS_000200.1	+	50190164	50190213
Sma-1-14774	NS_000200.1	+	64882942	64882991
Sma-1-14775	NS_000200.1	+	68077453	68077502
Sma-1-14776	NS_000200.1	+	73625886	73625935
Sma-1-14777	NS_000200.1	+	81098408	81098457
Sma-1-14778	NS_000200.1	+	86644365	86644414
Sma-1-14779	NS_000200.1	+	97295279	97295328
Sma-1-14780	NS_000200.1	+	110320181	110320230
Sma-1-14781	NS_000200.1	+	130877250	130877299
Sma-1-14782	NS_000200.1	+	150890953	150891002
Sma-1-14783	NS_000200.1	+	223849945	223849994
Sma-1-14784	NS_000200.1	+	293659239	293659288
Sma-1-14785	NS_000200.1	+	299788299	299788348
Sma-1-14786	NS_000200.1	+	300479960	300480009
Sma-1-14787	NS_000200.1	+	323225578	323225627
Sma-1-14788	NS_000200.1	+	335955032	335955081
Sma-1-14789	NS_000200.1	+	359643521	359643570
Sma-1-14790	NS_000200.1	+	373614794	373614843
Sma-1-14791	NS_000200.1	-	361125851	361125802
Sma-1-14792	NS_000200.1	-	300936170	300936121
Sma-1-14793	NS_000200.1	-	267194916	267194867
Sma-1-14794	NS_000200.1	-	251802234	251802185
Sma-1-14795	NS_000200.1	-	243346997	243346948
Sma-1-14796	NS_000200.1	-	211102177	211102128
Sma-1-14797	NS_000200.1	-	209375230	209375181
Sma-1-14798	NS_000200.1	-	207184769	207184720
Sma-1-14799	NS_000200.1	-	187906799	187906750
Sma-1-14800	NS_000200.1	-	176079092	176079043
Sma-1-14801	NS_000200.1	-	161784741	161784692
Sma-1-14802	NS_000200.1	-	142126611	142126562
Sma-1-14803	NS_000200.1	-	123593198	123593149
Sma-1-14804	NS_000200.1	-	102124688	102124639
Sma-1-14805	NS_000200.1	-	80053760	80053711
Sma-1-14806	NW_003023042.1	+	992	1041
Sma-1-14807	NS_000200.1	+	372227656	372227705
Sma-1-14808	NW_003037911.1	+	512642	512691
Sma-1-14809	NS_000200.1	+	156299995	156300044
Sma-1-14810	NW_003037809.1	+	1318213	1318262
Sma-1-14811	NS_000200.1	+	34982677	34982726
Sma-1-14812	NW_003037909.1	+	718505	718554
Sma-1-14813	NW_003038513.1	+	44698	44747
Sma-1-14814	NS_000200.1	+	154260154	154260203
Sma-1-14815	NS_000200.1	+	339053875	339053924
Sma-1-14816	NW_003037849.1	+	83881	83930
Sma-1-14817	NS_000200.1	+	91060154	91060203
Sma-1-14818	NW_003037853.1	-	998028	997979
Sma-1-14819	NS_000200.1	-	96490079	96490030
Sma-1-14820	NW_003038161.1	-	537253	537204

Sma-1-14821	NS_000200.1	-	297907104	297907055
Sma-1-14822	NW_003020123.1	+	770	819
Sma-1-14823	NW_003038026.1	+	796200	796249
Sma-1-14824	NS_000200.1	+	240303961	240304010
Sma-1-14825	NS_000200.1	+	371048493	371048542
Sma-1-14826	NW_003026517.1	+	744978	745027
Sma-1-14827	NS_000200.1	+	300350987	300351036
Sma-1-14828	NW_003035580.1	-	13474	13425
Sma-1-14829	NW_003037822.1	-	1175367	1175318
Sma-1-14830	NW_003038029.1	+	861954	862003
Sma-1-14831	NW_003038029.1	-	863508	863459
Sma-1-14832	NS_000200.1	+	243062855	243062904
Sma-1-14833	NS_000200.1	-	329095068	329095019
Sma-1-14834	NS_000200.1	-	243064409	243064360
Sma-1-14835	NS_000200.1	-	55483373	55483324
Sma-1-14836	NW_003037793.1	-	4109326	4109277
Sma-1-14837	NS_000200.1	-	4109326	4109277
Sma-1-14838	NW_003037794.1	-	485879	485830
Sma-1-14839	NS_000200.1	-	4665474	4665425
Sma-1-14840	NW_003039130.1	-	249094	249045
Sma-1-14841	NS_000200.1	-	317923705	317923656
Sma-1-14842	NW_003037793.1	-	690754	690705
Sma-1-14843	NW_003038252.1	-	178081	178032
Sma-1-14844	NW_003038109.1	-	25940	25891
Sma-1-14845	NS_000200.1	-	319033669	319033620
Sma-1-14846	NS_000200.1	-	275708618	275708569
Sma-1-14847	NS_000200.1	-	690754	690705
Sma-1-14848	NW_003037793.1	+	2572457	2572506
Sma-1-14849	NW_003037940.1	+	316253	316302
Sma-1-14850	NW_003026487.1	+	120425	120474
Sma-1-14851	NS_000200.1	+	2572457	2572506
Sma-1-14852	NS_000200.1	+	184058383	184058432
Sma-1-14853	NS_000200.1	+	261031119	261031168
Sma-1-14854	NW_003037861.1	-	843357	843308
Sma-1-14855	NS_000200.1	-	105619811	105619762
Sma-1-14856	NW_003037870.1	-	360222	360173
Sma-1-14857	NS_000200.1	-	117233887	117233838
Sma-1-14858	NW_003037904.1	+	515735	515784
Sma-1-14859	NS_000200.1	+	149230234	149230283
Sma-1-14860	NW_003038030.1	+	254557	254606
Sma-1-14861	NS_000200.1	+	243390424	243390473
Sma-1-14862	NW_003038079.1	+	204468	204517
Sma-1-14863	NS_000200.1	+	268334456	268334505
Sma-1-14864	NW_003037800.1	-	2279044	2278995
Sma-1-14865	NS_000200.1	-	20908404	20908355
Sma-1-14866	NW_003037851.1	+	115766	115815
Sma-1-14867	NW_003037945.1	-	1504436	1504387
Sma-1-14868	NS_000200.1	+	93519076	93519125
Sma-1-14869	NS_000200.1	-	190606400	190606351
Sma-1-14870	NW_003037847.1	-	933260	933211
Sma-1-14871	NW_003038027.1	-	676357	676308
Sma-1-14872	NS_000200.1	-	241047071	241047022

Sma-1-14873	NS_000200.1	-	89584990	89584941
Sma-1-14874	NW_003039127.1	+	1273906	1273955
Sma-1-14875	NS_000200.1	+	114351405	114351454
Sma-1-14876	NW_003037889.1	+	1076567	1076616
Sma-1-14877	NS_000200.1	+	134853057	134853106
Sma-1-14878	NW_003026611.1	-	224108	224059
Sma-1-14879	NW_003037796.1	+	2076118	2076167
Sma-1-14880	NW_003037861.1	+	845295	845344
Sma-1-14881	NS_000200.1	+	10285762	10285811
Sma-1-14882	NS_000200.1	+	105621749	105621798
Sma-1-14883	NS_000200.1	-	310793892	310793843
Sma-1-14884	NW_003026464.1	-	140556	140507
Sma-1-14885	NS_000200.1	-	203440865	203440816
Sma-1-14886	NW_003037990.1	+	206895	206944
Sma-1-14887	NS_000200.1	+	217105654	217105703
Sma-1-14888	NW_003038244.1	-	219756	219707
Sma-1-14889	NW_003037809.1	-	365942	365893
Sma-1-14890	NS_000200.1	-	315364961	315364912
Sma-1-14891	NS_000200.1	-	34030406	34030357
Sma-1-14892	NW_003038257.1	+	334711	334760
Sma-1-14893	NS_000200.1	+	321509039	321509088
Sma-1-14894	NW_003038261.1	+	37435	37484
Sma-1-14895	NS_000200.1	+	323651086	323651135
Sma-1-14896	NW_003032033.1	+	315	364
Sma-1-14897	NW_003026617.1	+	127106	127155
Sma-1-14898	NW_003037874.1	+	519417	519466
Sma-1-14899	NW_003026594.1	+	49640	49689
Sma-1-14900	NW_003038013.1	-	264062	264013
Sma-1-14901	NW_003038070.1	-	43673	43624
Sma-1-14902	NW_003037793.1	-	2793520	2793471
Sma-1-14903	NW_003037819.1	+	934051	934100
Sma-1-14904	NW_003037824.1	-	639575	639526
Sma-1-14905	NW_003037825.1	-	1303555	1303506
Sma-1-14906	NW_003037827.1	+	2238996	2239045
Sma-1-14907	NW_003037834.1	+	63594	63643
Sma-1-14908	NW_003037842.1	-	117363	117314
Sma-1-14909	NW_003037858.1	-	1022960	1022911
Sma-1-14910	NW_003037862.1	-	781544	781495
Sma-1-14911	NW_003037869.1	-	325276	325227
Sma-1-14912	NW_003037878.1	+	450882	450931
Sma-1-14913	NW_003037888.1	+	1235467	1235516
Sma-1-14914	NW_003037894.1	+	354925	354974
Sma-1-14915	NW_003037905.1	-	1000764	1000715
Sma-1-14916	NW_003037917.1	-	342662	342613
Sma-1-14917	NW_003037918.1	-	455706	455657
Sma-1-14918	NW_003037956.1	+	127285	127334
Sma-1-14919	NW_003037967.1	+	330081	330130
Sma-1-14920	NW_003037970.1	-	5031	4982
Sma-1-14921	NW_003038014.1	-	47083	47034
Sma-1-14922	NW_003038015.1	-	149797	149748
Sma-1-14923	NW_003038037.1	+	982246	982295
Sma-1-14924	NW_003038037.1	+	1213826	1213875

Sma-1-14925	NW_003038037.1	-	854928	854879
Sma-1-14926	NW_003038047.1	+	78260	78309
Sma-1-14927	NW_003038047.1	-	77771	77722
Sma-1-14928	NW_003038096.1	+	24419	24468
Sma-1-14929	NW_003038151.1	+	154496	154545
Sma-1-14930	NS_000200.1	+	52022316	52022365
Sma-1-14931	NS_000200.1	+	64861137	64861186
Sma-1-14932	NS_000200.1	+	73224080	73224129
Sma-1-14933	NS_000200.1	+	119944367	119944416
Sma-1-14934	NS_000200.1	+	122504360	122504409
Sma-1-14935	NS_000200.1	+	133443959	133444008
Sma-1-14936	NS_000200.1	+	142251498	142251547
Sma-1-14937	NS_000200.1	+	194183740	194183789
Sma-1-14938	NS_000200.1	+	199570052	199570101
Sma-1-14939	NS_000200.1	+	251137655	251137704
Sma-1-14940	NS_000200.1	+	251369235	251369284
Sma-1-14941	NS_000200.1	+	255328259	255328308
Sma-1-14942	NS_000200.1	+	274193412	274193461
Sma-1-14943	NS_000200.1	+	292061739	292061788
Sma-1-14944	NS_000200.1	+	304994010	304994059
Sma-1-14945	NS_000200.1	+	319697965	319698014
Sma-1-14946	NS_000200.1	+	376897472	376897521
Sma-1-14947	NS_000200.1	-	264380914	264380865
Sma-1-14948	NS_000200.1	-	255327770	255327721
Sma-1-14949	NS_000200.1	-	251010337	251010288
Sma-1-14950	NS_000200.1	-	230473287	230473238
Sma-1-14951	NS_000200.1	-	229787772	229787723
Sma-1-14952	NS_000200.1	-	229653688	229653639
Sma-1-14953	NS_000200.1	-	201061221	201061172
Sma-1-14954	NS_000200.1	-	167022696	167022647
Sma-1-14955	NS_000200.1	-	165379466	165379417
Sma-1-14956	NS_000200.1	-	150269776	150269727
Sma-1-14957	NS_000200.1	-	116797621	116797572
Sma-1-14958	NS_000200.1	-	106433205	106433156
Sma-1-14959	NS_000200.1	-	101687236	101687187
Sma-1-14960	NS_000200.1	-	85216553	85216504
Sma-1-14961	NS_000200.1	-	60433337	60433288
Sma-1-14962	NS_000200.1	-	57570515	57570466
Sma-1-14963	NS_000200.1	-	2793520	2793471
Sma-1-14964	NW_003038156.1	+	10515	10564
Sma-1-14965	NS_000200.1	+	294742628	294742677
Sma-1-14966	NW_003037925.1	-	203418	203369
Sma-1-14967	NS_000200.1	-	172605421	172605372
Sma-1-14968	NW_003037836.1	+	270253	270302
Sma-1-14969	NS_000200.1	+	75827352	75827401
Sma-1-14970	NW_003037930.1	-	276960	276911
Sma-1-14971	NS_000200.1	-	176134924	176134875
Sma-1-14972	NW_003037806.1	+	1073982	1074031
Sma-1-14973	NS_000200.1	+	29269196	29269245
Sma-1-14974	NW_003037819.1	-	998905	998856
Sma-1-14975	NW_003037914.1	+	1595568	1595617
Sma-1-14976	NS_000200.1	+	161739818	161739867

Sma-1-14977	NS_000200.1	-	52087170	52087121
Sma-1-14978	NW_003038184.1	-	7405	7356
Sma-1-14979	NS_000200.1	-	301597547	301597498
Sma-1-14980	NW_003026460.1	+	374323	374372
Sma-1-14981	NW_003026460.1	+	376849	376898
Sma-1-14982	NS_000200.1	+	170896529	170896578
Sma-1-14983	NS_000200.1	+	170899055	170899104
Sma-1-14984	NW_003037907.1	-	158797	158748
Sma-1-14985	NS_000200.1	-	151563286	151563237
Sma-1-14986	NW_003037799.1	+	910922	910971
Sma-1-14987	NS_000200.1	+	17901276	17901325
Sma-1-14988	NW_003037851.1	+	355072	355121
Sma-1-14989	NW_003037976.1	+	20792	20841
Sma-1-14990	NW_003037992.1	+	234657	234706
Sma-1-14991	NW_003038042.1	+	95771	95820
Sma-1-14992	NS_000200.1	+	93758382	93758431
Sma-1-14993	NS_000200.1	+	206635107	206635156
Sma-1-14994	NS_000200.1	+	218076138	218076187
Sma-1-14995	NS_000200.1	+	254041370	254041419
Sma-1-14996	NW_003037874.1	+	194167	194216
Sma-1-14997	NS_000200.1	+	119619117	119619166
Sma-1-14998	NW_003037874.1	-	286975	286926
Sma-1-14999	NS_000200.1	-	119711925	119711876
Sma-1-15000	NW_003037874.1	-	591547	591498
Sma-1-15001	NS_000200.1	-	120016497	120016448
Sma-1-15002	NW_003037875.1	+	332346	332395
Sma-1-15003	NS_000200.1	+	120361015	120361064
Sma-1-15004	NW_003038252.1	-	6327	6278
Sma-1-15005	NS_000200.1	-	318861915	318861866
Sma-1-15006	NW_003037793.1	-	3164641	3164593
Sma-1-15007	NS_000200.1	-	3164641	3164593
Sma-1-15008	NW_003037923.1	+	326296	326345
Sma-1-15009	NS_000200.1	+	171382509	171382558
Sma-1-15010	NW_003037803.1	+	1283002	1283050
Sma-1-15011	NS_000200.1	+	24783539	24783587
Sma-1-15012	NW_003037820.1	-	282140	282092
Sma-1-15013	NS_000200.1	-	52555122	52555074
Sma-1-15014	NW_003037980.1	+	947079	947128
Sma-1-15015	NS_000200.1	+	211907941	211907990
Sma-1-15016	NW_003037829.1	-	744814	744765
Sma-1-15017	NS_000200.1	-	66645352	66645303
Sma-1-15018	NW_003038442.1	+	840	892
Sma-1-15019	NS_000200.1	+	329856893	329856945
Sma-1-15020	NW_003038073.1	+	3964	4016
Sma-1-15021	NW_003038073.1	-	5324	5272
Sma-1-15022	NS_000200.1	+	265526397	265526449
Sma-1-15023	NS_000200.1	-	265527757	265527705
Sma-1-15024	NW_003038261.1	-	974287	974238
Sma-1-15025	NS_000200.1	-	324587938	324587889
Sma-1-15026	NW_003038152.1	-	92888	92839
Sma-1-15027	NS_000200.1	-	292698612	292698563
Sma-1-15028	NW_003038508.1	-	69689	69640

Sma-1-15029	NW_003038976.1	-	1273	1224
Sma-1-15030	NS_000200.1	-	357174454	357174405
Sma-1-15031	NS_000200.1	-	337176657	337176608
Sma-1-15032	NW_003037841.1	-	1291430	1291381
Sma-1-15033	NS_000200.1	-	83902611	83902562
Sma-1-15034	NW_003037849.1	+	459154	459203
Sma-1-15035	NS_000200.1	+	91435427	91435476
Sma-1-15036	NW_003026462.1	-	224617	224568
Sma-1-15037	NS_000200.1	-	196892120	196892071
Sma-1-15038	NW_003039130.1	-	138337	138287
Sma-1-15039	NS_000200.1	-	317812948	317812898
Sma-1-15040	NW_003037523.1	-	794	745
Sma-1-15041	NS_000200.1	-	370842084	370842035
Sma-1-15042	NW_003038462.1	+	46667	46716
Sma-1-15043	NS_000200.1	+	331414330	331414379
Sma-1-15044	NW_003037979.1	-	66911	66862
Sma-1-15045	NS_000200.1	-	210127931	210127882
Sma-1-15046	NW_003037804.1	+	798530	798579
Sma-1-15047	NS_000200.1	+	25938550	25938599
Sma-1-15048	NW_003037816.1	+	538155	538205
Sma-1-15049	NS_000200.1	+	46747027	46747077
Sma-1-15050	NW_003037800.1	-	2167381	2167333
Sma-1-15051	NS_000200.1	-	20796741	20796693
Sma-1-15052	NW_003037942.1	+	980838	980886
Sma-1-15053	NS_000200.1	+	186740746	186740794
Sma-1-15054	NW_003038156.1	-	280574	280526
Sma-1-15055	NS_000200.1	-	295012687	295012639
Sma-1-15056	NW_003021529.1	+	91	139
Sma-1-15057	NS_000200.1	+	348025990	348026038
Sma-1-15058	NW_003037905.1	+	828588	828636
Sma-1-15059	NS_000200.1	+	150097600	150097648
Sma-1-15060	NW_003038038.1	-	528772	528722
Sma-1-15061	NS_000200.1	-	252432165	252432115
Sma-1-15062	NW_003038152.1	-	111465	111415
Sma-1-15063	NS_000200.1	-	292717189	292717139
Sma-1-15064	NW_003038163.1	+	322069	322118
Sma-1-15065	NS_000200.1	+	298722349	298722398
Sma-1-15066	NW_003037917.1	+	347103	347152
Sma-1-15067	NS_000200.1	+	165383907	165383956
Sma-1-15068	NW_003037915.1	+	115193	115242
Sma-1-15069	NS_000200.1	+	162015179	162015228
Sma-1-15070	NW_003026517.1	-	340589	340540
Sma-1-15071	NS_000200.1	-	299946598	299946549
Sma-1-15072	NW_003037937.1	-	795971	795922
Sma-1-15073	NS_000200.1	-	181995880	181995831
Sma-1-15074	NW_003038249.1	-	238805	238756
Sma-1-15075	NS_000200.1	-	316921058	316921009
Sma-1-15076	NW_003026591.1	-	46217	46168
Sma-1-15077	NS_000200.1	-	303995045	303994996
Sma-1-15078	NW_003026517.1	+	283544	283593
Sma-1-15079	NS_000200.1	+	299889553	299889602
Sma-1-15080	NW_003038027.1	-	751168	751119

Sma-1-15081	NS_000200.1	-	241121882	241121833
Sma-1-15082	NW_003038505.1	-	195716	195667
Sma-1-15083	NS_000200.1	-	336275053	336275004
Sma-1-15084	NW_003038027.1	-	868522	868474
Sma-1-15085	NS_000200.1	-	241239236	241239188
Sma-1-15086	NW_003037886.1	+	1079935	1079983
Sma-1-15087	NS_000200.1	+	130335502	130335550
Sma-1-15088	NW_003038031.1	-	662724	662676
Sma-1-15089	NS_000200.1	-	244940482	244940434
Sma-1-15090	NW_003037888.1	-	70639	70590
Sma-1-15091	NS_000200.1	-	132279131	132279082
Sma-1-15092	NW_003026459.1	-	245102	245053
Sma-1-15093	NS_000200.1	-	141890375	141890326
Sma-1-15094	NW_003032051.1	-	368	317
Sma-1-15095	NS_000200.1	-	377004531	377004480
Sma-1-15096	NW_003037797.1	+	3439771	3439819
Sma-1-15097	NS_000200.1	+	13867631	13867679
Sma-1-15098	NW_003039127.1	-	724127	724050
Sma-1-15099	NS_000200.1	-	113801626	113801549
Sma-1-15100	NW_003037980.1	+	1256927	1257004
Sma-1-15101	NS_000200.1	+	212217789	212217866
Sma-1-15102	NW_003038028.1	-	755361	755284
Sma-1-15103	NS_000200.1	-	242062935	242062858
Nve-1-172	NW_001834409.1	+	519287	519335
Nve-1-173	NW_001834348.1	-	450000	449952
Nve-1-174	NW_001834300.1	-	629072	629027
Nve-1-175	NW_001827811.1	+	868	916
Nve-1-176	NW_001834180.1	-	275733	275685
Nve-1-177	NW_001834333.1	-	684521	684473
Nve-1-178	NW_001834362.1	-	515646	515598
Nve-1-179	NW_001834396.1	-	401172	401126
Nve-1-180	NW_001828687.1	+	71	118
Nve-1-181	NW_001834410.1	+	2514185	2514232
Nve-1-182	NW_001834075.1	+	31649	31697
Nve-1-183	NW_001834045.1	-	51750	51701
Nve-1-184	NW_001834291.1	-	96651	96603
Nve-1-185	NW_001834336.1	+	693860	693908
Nve-1-186	NW_001833989.1	-	26445	26396
Nve-1-187	NW_001834300.1	+	229171	229219
Nve-1-188	NW_001834204.1	+	401293	401341
Nve-1-189	NW_001834237.1	+	493	541
Nve-1-190	NW_001834359.1	+	1061534	1061582
Nve-1-191	NW_001834284.1	+	504381	504429
Nve-1-192	NW_001834287.1	+	339219	339267
Nve-1-193	NW_001834353.1	-	66232	66184
Nve-1-194	NW_001834393.1	-	1589950	1589902
Nve-1-195	NW_001834400.1	-	960257	960209
Nve-1-196	NW_001834402.1	+	5	53
Nve-1-197	NW_001834409.1	+	1966176	1966225
Nve-1-198	NW_001834411.1	+	2584674	2584722
Nve-1-199	NW_001833976.1	+	55150	55199
Nve-1-200	NW_001834140.1	+	121642	121691

Nve-1-201	NW_001832857.1	+	4362	4408
Nve-1-202	NW_001834212.1	-	295324	295273
Nve-1-203	NW_001825820.1	-	439	393
Nve-1-204	NW_001834093.1	-	129941	129895
Nve-1-205	NW_001834315.1	-	176930	176881
Nve-1-206	NW_001834331.1	-	348983	348934
Nve-1-207	NW_001834339.1	-	360768	360721
Nve-1-208	NW_001834123.1	-	91429	91382
Nve-1-209	NW_001834106.1	+	233614	233662
Nve-1-210	NW_001834348.1	-	116614	116566
Nve-1-211	NW_001834362.1	+	625452	625500
Nve-1-212	NW_001834409.1	+	285421	285469
Nve-1-213	NW_001834352.1	-	589991	589943
Nve-1-214	NW_001834394.1	-	130164	130116
Nve-1-215	NW_001834329.1	+	256845	256893
Nve-1-216	NW_001833889.1	+	36409	36457
Nve-1-217	NW_001834014.1	-	129399	129351
Nve-1-218	NW_001834195.1	+	255667	255715
Nve-1-219	NW_001834160.1	+	68964	69012
Nve-1-220	NW_001834377.1	+	691392	691440
Nve-1-221	NW_001834157.1	-	312036	311988
Nve-1-222	NW_001823964.1	+	290	338
Nve-1-223	NW_001827969.1	+	1633	1681
Nve-1-224	NW_001833817.1	+	13653	13701
Nve-1-225	NW_001833817.1	+	14012	14060
Nve-1-226	NW_001833817.1	+	14339	14387
Nve-1-227	NW_001833817.1	+	14693	14741
Nve-1-228	NW_001833903.1	+	12780	12828
Nve-1-229	NW_001833903.1	+	15273	15321
Nve-1-230	NW_001833934.1	-	130984	130936
Nve-1-231	NW_001833934.1	-	127083	127035
Nve-1-232	NW_001833934.1	-	126727	126679
Nve-1-233	NW_001833934.1	-	126372	126324
Nve-1-234	NW_001833934.1	-	126017	125969
Nve-1-235	NW_001833934.1	-	125662	125614
Nve-1-236	NW_001833934.1	-	125307	125259
Nve-1-237	NW_001833934.1	-	124951	124903
Nve-1-238	NW_001833989.1	-	26090	26042
Nve-1-239	NW_001833998.1	+	110246	110294
Nve-1-240	NW_001834016.1	-	96284	96236
Nve-1-241	NW_001834016.1	-	95930	95882
Nve-1-242	NW_001834044.1	+	186338	186386
Nve-1-243	NW_001834044.1	+	186692	186740
Nve-1-244	NW_001834044.1	+	187720	187768
Nve-1-245	NW_001834044.1	+	188074	188122
Nve-1-246	NW_001834081.1	-	9057	9009
Nve-1-247	NW_001834081.1	-	8703	8655
Nve-1-248	NW_001834193.1	+	342358	342406
Nve-1-249	NW_001834193.1	+	342713	342761
Nve-1-250	NW_001834193.1	+	343066	343114
Nve-1-251	NW_001834193.1	+	343421	343469
Nve-1-252	NW_001834193.1	+	343776	343824

Nve-1-253	NW_001834212.1	+	186510	186558
Nve-1-254	NW_001834212.1	+	186864	186912
Nve-1-255	NW_001834315.1	-	176571	176523
Nve-1-256	NW_001834355.1	-	258795	258747
Nve-1-257	NW_001834394.1	-	131983	131935
Nve-1-258	NW_001834394.1	-	130518	130470
Nve-1-259	NW_001834409.1	-	1095577	1095529
Nve-1-260	NW_001832444.1	+	15592	15640
Nve-1-261	NW_001834000.1	+	77564	77612
Nve-1-262	NW_001834252.1	-	488487	488439
Nve-1-263	NW_001834280.1	+	547077	547125
Nve-1-264	NW_001834352.1	-	589637	589589
Nve-1-265	NW_001834399.1	-	1280612	1280564
Nve-1-266	NW_001834402.1	+	359	407
Nve-1-267	NW_001833820.1	-	11840	11792
Nve-1-268	NW_001834411.1	+	54433	54481
Nve-1-269	NW_001825686.1	+	447	495
Nve-1-270	NW_001834402.1	-	1787006	1786958
Nve-1-271	NW_001834168.1	+	122822	122870
Nve-1-272	NW_001834168.1	+	323855	323903
Nve-1-273	NW_001834193.1	-	341001	340953
Nve-1-274	NW_001834272.1	+	114134	114182
Nve-1-275	NW_001834285.1	+	169699	169747
Nve-1-276	NW_001834274.1	+	15718	15766
Nve-1-277	NW_001834274.1	+	16072	16120
Nve-1-278	NW_001834274.1	+	28844	28892
Nve-1-279	NW_001834274.1	+	29552	29600
Nve-1-280	NW_001834391.1	+	1300956	1301004
Nve-1-281	NW_001834016.1	+	67712	67760
Nve-1-282	NW_001828027.1	-	3618	3570
Nve-1-283	NW_001833409.1	+	37400	37448
Nve-1-284	NW_001834016.1	+	98381	98429
Nve-1-285	NW_001834157.1	-	331331	331283
Nve-1-286	NW_001833422.1	-	58	10
Nve-1-287	NW_001824545.1	-	1082	1034
Nve-1-288	NW_001834244.1	-	474243	474195
Nve-1-289	NW_001834296.1	+	382794	382842
Nve-1-290	NW_001834388.1	-	1307215	1307167
Nve-1-291	NW_001834333.1	-	81235	81186
Nve-1-292	NW_001834396.1	-	400835	400788
Nve-1-293	NW_001834339.1	-	235427	235379
Nve-1-294	NW_001834380.1	+	35694	35742
Nve-1-295	NW_001832756.1	+	11773	11821
Nve-1-296	NW_001834219.1	+	127097	127145
Nve-1-297	NW_001834397.1	-	1090783	1090735
Nve-1-298	NW_001834200.1	-	209948	209900
Nve-1-299	NW_001833707.1	+	17040	17088
Nve-1-300	NW_001834166.1	-	190350	190302
Nve-1-301	NW_001833707.1	+	17394	17442
Nve-1-302	NW_001834166.1	-	231041	230993
Nve-1-303	NW_001834166.1	-	230333	230285
Nve-1-304	NW_001834339.1	+	754416	754464

Nve-1-305	NW_001832614.1	-	10332	10284
Nve-1-306	NW_001834295.1	+	395430	395478
Nve-1-307	NW_001834295.1	+	399458	399506
Nve-1-308	NW_001834412.1	+	1268476	1268524
Nve-1-309	NW_001834298.1	-	633408	633360
Nve-1-310	NW_001834369.1	-	1067371	1067323
Nve-1-311	NW_001834402.1	-	147248	147200
Nve-1-312	NW_001833923.1	+	82526	82574
Nve-1-313	NW_001834166.1	-	231395	231347
Nve-1-314	NW_001834383.1	-	861661	861613
Nve-1-315	NW_001834368.1	-	614456	614408
Nve-1-316	NW_001834361.1	-	281046	280997
Nve-1-317	NW_001834392.1	+	1285841	1285890
Nve-1-318	NW_001833631.1	-	19254	19205
Nve-1-319	NW_001834204.1	-	282061	282012
Nve-1-320	NW_001834404.1	+	217218	217267
Nve-1-321	NW_001834404.1	+	1946468	1946517
Nve-1-322	NW_001834304.1	+	315768	315817
Nve-1-323	NW_001834294.1	-	106646	106598
Nve-1-324	NW_001830134.1	-	6880	6832
Nve-1-325	NW_001832797.1	-	9426	9378
Nve-1-326	NW_001833807.1	-	65583	65535
Nve-1-327	NW_001834226.1	+	54821	54869
Nve-1-328	NW_001833934.1	-	36018	35970
Nve-1-329	NW_001834347.1	-	836767	836719
Nve-1-330	NW_001834249.1	-	143465	143417
Nve-1-331	NW_001832410.1	+	4633	4681
Nve-1-332	NW_001826320.1	-	1152	1104
Nve-1-333	NW_001834163.1	+	13886	13934
Nve-1-334	NW_001834413.1	+	950886	950934
Nve-1-335	NW_001831272.1	-	8136	8088
Nve-1-336	NW_001834299.1	-	375315	375267
Nve-1-337	NW_001832256.1	+	10966	11014
Nve-1-338	NW_001833835.1	-	1564	1516
Nve-1-339	NW_001834406.1	-	1261735	1261687
Nve-1-340	NW_001834149.1	+	271450	271498
Nve-1-341	NW_001834326.1	-	448399	448351
Nve-1-342	NW_001834291.1	-	622001	621953
Nve-1-343	NW_001834310.1	+	518739	518787
Nve-1-344	NW_001834404.1	-	149626	149578
Nve-1-345	NW_001834343.1	-	573534	573486
Nve-1-346	NW_001834166.1	-	231749	231701
Nve-1-347	NW_001834166.1	-	222915	222867
Nve-1-348	NW_001834373.1	-	774860	774812
Nve-1-349	NW_001834166.1	-	230686	230638
Nve-1-350	NW_001834404.1	-	349043	348995
Nve-1-351	NW_001832257.1	-	4946	4898
Nve-1-352	NW_001834378.1	-	1024300	1024252
Nve-1-353	NW_001834306.1	+	549175	549220
Nve-1-354	NW_001834398.1	+	56680	56728
Nve-1-355	NW_001834378.1	-	906453	906405
Nve-1-356	NW_001826742.1	+	529	577

Nve-1-357	NW_001834296.1	+	596770	596818
Nve-1-358	NW_001834357.1	+	37516	37564
Nve-1-359	NW_001833077.1	+	1722	1770
Nve-1-360	NW_001833077.1	+	2042	2090
Nve-1-361	NW_001833864.1	-	699	651
Nve-1-362	NW_001833864.1	-	344	296
Nve-1-363	NW_001833934.1	-	35669	35621
Nve-1-364	NW_001833955.1	+	66558	66606
Nve-1-365	NW_001834177.1	+	133396	133444
Nve-1-366	NW_001834220.1	-	62018	61970
Nve-1-367	NW_001834306.1	-	548901	548853
Nve-1-368	NW_001834306.1	-	188834	188786
Nve-1-369	NW_001834348.1	+	116756	116804
Nve-1-370	NW_001834408.1	-	305453	305405
Nve-1-371	NW_001834410.1	-	1649158	1649110
Nve-1-372	NW_001834393.1	-	816593	816545
Nve-1-373	NW_001834086.1	+	61754	61802
Nve-1-374	NW_001834216.1	+	145685	145733
Nve-1-375	NW_001834234.1	-	410112	410064
Nve-1-376	NW_001834401.1	-	1804231	1804183
Nve-1-377	NW_001834401.1	-	1799980	1799932
Nve-1-378	NW_001833827.1	-	38808	38760
Nve-1-379	NW_001834186.1	+	99741	99789
Nve-1-380	NW_001834225.1	-	222694	222646
Nve-1-381	NW_001834352.1	-	344105	344057
Nve-1-382	NW_001834408.1	+	305974	306022
Nve-1-383	NW_001834176.1	-	116628	116580
Nve-1-384	NW_001834132.1	+	39241	39289
Nve-1-385	NW_001834110.1	-	44953	44905
Nve-1-386	NW_001834014.1	-	34908	34860
Nve-1-387	NW_001833780.1	+	15710	15758
Nve-1-388	NW_001834024.1	-	94775	94727
Nve-1-389	NW_001834222.1	-	303173	303125
Nve-1-390	NW_001834234.1	-	423927	423879
Nve-1-391	NW_001834234.1	-	423578	423530
Nve-1-392	NW_001834256.1	+	350442	350490
Nve-1-393	NW_001834256.1	-	511850	511802
Nve-1-394	NW_001834336.1	-	303230	303182
Nve-1-395	NW_001834383.1	-	627145	627097
Nve-1-396	NW_001834291.1	-	622355	622307
Nve-1-397	NW_001834396.1	+	402347	402395
Nve-1-398	NW_001834406.1	-	1261380	1261332
Nve-1-399	NW_001833771.1	+	35376	35424
Nve-1-400	NW_001834027.1	+	93993	94041
Nve-1-401	NW_001834381.1	-	896250	896202
Nve-1-402	NW_001833678.1	-	49318	49270
Nve-1-403	NW_001834304.1	-	262973	262925
Nve-1-404	NW_001832852.1	-	13691	13643
Nve-1-405	NW_001832852.1	-	9847	9799
Nve-1-406	NW_001824109.1	+	463	511
Nve-1-407	NW_001834222.1	+	126418	126466
Nve-1-408	NW_001834222.1	-	125595	125547

Nve-1-409	NW_001834362.1	+	1073047	1073095
Nve-1-410	NW_001834377.1	+	690683	690731
Nve-1-411	NW_001834378.1	+	726932	726980
Nve-1-412	NW_001833814.1	+	49014	49065
Nve-1-413	NW_001834234.1	+	204301	204349
Nve-1-414	NW_001834180.1	+	52282	52330
Nve-1-415	NW_001834216.1	+	5589	5637
Nve-1-416	NW_001834170.1	-	139333	139285
Nve-1-417	NW_001834293.1	-	219242	219194
Nve-1-418	NW_001834355.1	-	1026407	1026359
Nve-1-419	NW_001834216.1	+	3789	3837
Nve-1-420	NW_001834348.1	+	956860	956908
Nve-1-421	NW_001834216.1	+	6258	6306
Nve-1-422	NW_001834239.1	-	45882	45834
Nve-1-423	NW_001834293.1	-	225369	225321
Nve-1-424	NW_001834293.1	-	219595	219547
Nve-1-425	NW_001833971.1	-	32933	32885
Nve-1-426	NW_001834020.1	+	148988	149036
Nve-1-427	NW_001834220.1	-	278118	278070
Nve-1-428	NW_001834395.1	-	1111234	1111186
Nve-1-429	NW_001834062.1	+	100334	100382
Nve-1-430	NW_001834246.1	+	181973	182021
Nve-1-431	NW_001834278.1	+	406850	406898
Nve-1-432	NW_001834300.1	-	626622	626574
Nve-1-433	NW_001825290.1	-	1015	967
Nve-1-434	NW_001834338.1	+	354215	354263
Nve-1-435	NW_001830916.1	-	6308	6260
Nve-1-436	NW_001833027.1	+	271	319
Nve-1-437	NW_001834103.1	+	24742	24790
Nve-1-438	NW_001834103.1	-	128932	128884
Nve-1-439	NW_001834103.1	-	128578	128530
Nve-1-440	NW_001834269.1	+	295803	295851
Nve-1-441	NW_001834272.1	+	111468	111516
Nve-1-442	NW_001834372.1	-	247942	247894
Nve-1-443	NW_001834094.1	+	50417	50465
Nve-1-444	NW_001834339.1	+	360842	360890
Nve-1-445	NW_001834341.1	-	384673	384625
Nve-1-446	NW_001834368.1	+	445297	445345
Nve-1-447	NW_001834388.1	+	1038723	1038771
Nve-1-448	NW_001833387.1	-	2082	2034
Nve-1-449	NW_001834166.1	-	223271	223223
Nve-1-450	NW_001834319.1	-	301207	301159
Nve-1-451	NW_001834410.1	-	1763358	1763310
Nve-1-452	NW_001834397.1	+	742775	742823
Nve-1-453	NW_001833257.1	+	23042	23090
Nve-1-454	NW_001834016.1	+	382	430
Nve-1-455	NW_001834157.1	-	299778	299730
Nve-1-456	NW_001834157.1	-	236817	236769
Nve-1-457	NW_001831433.1	-	2488	2440
Nve-1-458	NW_001829737.1	+	3388	3436
Nve-1-459	NW_001834016.1	+	44172	44220
Nve-1-460	NW_001834407.1	+	1609098	1609146

Nve-1-461	NW_001832900.1	-	9836	9788
Nve-1-462	NW_001828601.1	+	6068	6116
Nve-1-463	NW_001833889.1	+	54718	54766
Nve-1-464	NW_001827890.1	-	4602	4552
Nve-1-465	NW_001834036.1	-	83720	83670
Nve-1-466	NW_001834354.1	-	738521	738473
Nve-1-467	NW_001833951.1	-	118666	118618
Nve-1-468	NW_001833985.1	-	165627	165579
Nve-1-469	NW_001834027.1	-	115594	115548
Nve-1-470	NW_001834406.1	+	631305	631353
Nve-1-471	NW_001834153.1	+	79252	79300
Sma-1-15104	NW_003037827.1	+	371686	371753
Sma-1-15105	NS_000200.1	+	62993827	62993894
Sma-1-15106	NW_003037940.1	+	240616	240664
Sma-1-15107	NS_000200.1	+	183982746	183982794
Sma-1-15108	NW_003037859.1	+	806499	806547
Sma-1-15109	NS_000200.1	+	103485340	103485388
Sma-1-15110	NW_003037967.1	-	177216	177168
Sma-1-15111	NS_000200.1	-	199417187	199417139
Sma-1-15112	NW_003037964.1	-	499883	499835
Sma-1-15113	NS_000200.1	-	198101405	198101357
Sma-1-15114	NW_003035869.1	-	93093	93044
Sma-1-15115	NS_000200.1	-	335277293	335277244
Sma-1-15116	NW_003038152.1	-	167406	167359
Sma-1-15117	NS_000200.1	-	292773130	292773083
Sma-1-15118	NW_003037854.1	+	1123017	1123065
Sma-1-15119	NS_000200.1	+	98027438	98027486
Sma-1-15120	NW_003037977.1	+	455964	456012
Sma-1-15121	NS_000200.1	+	208741984	208742032
Sma-1-15122	NW_003038083.1	+	709359	709407
Sma-1-15123	NS_000200.1	+	270952696	270952744
Sma-1-15124	NW_003037966.1	-	275383	275335
Sma-1-15125	NS_000200.1	-	198977246	198977198
Sma-1-15126	NW_003037889.1	+	1511959	1512007
Sma-1-15127	NS_000200.1	+	135288449	135288497
Sma-1-15128	NW_003038083.1	-	19421	19373
Sma-1-15129	NS_000200.1	-	270262758	270262710
Sma-1-15130	NW_003038037.1	+	801657	801705
Sma-1-15131	NS_000200.1	+	250957066	250957114
Sma-1-15132	NW_003029567.1	-	321	273
Sma-1-15133	NW_003037977.1	+	941648	941696
Sma-1-15134	NS_000200.1	+	209227668	209227716
Sma-1-15135	NS_000200.1	-	366168666	366168618
Sma-1-15136	NW_003037937.1	-	814752	814703
Sma-1-15137	NS_000200.1	-	182014661	182014612
Sma-1-15138	NW_003037851.1	-	296220	296172
Sma-1-15139	NS_000200.1	-	93699530	93699482
Sma-1-15140	NW_003037807.1	-	503999	503951
Sma-1-15141	NS_000200.1	-	30765515	30765467
Sma-1-15142	NW_003037865.1	-	382918	382870
Sma-1-15143	NS_000200.1	-	110804837	110804789
Sma-1-15144	NW_003037834.1	-	301195	301147

Sma-1-15145	NS_000200.1	-	73461681	73461633
Sma-1-15146	NW_003038464.1	+	27183	27231
Sma-1-15147	NS_000200.1	+	331532450	331532498
Sma-1-15148	NW_003037976.1	+	286268	286316
Sma-1-15149	NS_000200.1	+	206900583	206900631
Sma-1-15150	NW_003037831.1	-	1446545	1446497
Sma-1-15151	NS_000200.1	-	70895847	70895799
Sma-1-15152	NW_003038149.1	+	78281	78331
Sma-1-15153	NS_000200.1	+	291106894	291106944
Sma-1-15154	NW_003037809.1	-	1706917	1706868
Sma-1-15155	NS_000200.1	-	35371381	35371332
Sma-1-15156	NW_003038135.1	+	79770	79821
Sma-1-15157	NS_000200.1	+	283952109	283952160
Sma-1-15158	NW_003038143.1	+	322332	322383
Sma-1-15159	NS_000200.1	+	288081824	288081875
Sma-1-15160	NW_003037814.1	+	254288	254339
Sma-1-15161	NS_000200.1	+	43238148	43238199
Sma-1-15162	NW_003037981.1	-	376137	376086
Sma-1-15163	NS_000200.1	-	212774005	212773954
Sma-1-15164	NW_003037859.1	-	965351	965303
Sma-1-15165	NS_000200.1	-	103644192	103644144
Sma-1-15166	NW_003038002.1	-	253434	253390
Sma-1-15167	NS_000200.1	-	223373643	223373599
Sma-1-15168	NW_003037874.1	-	67762	67714
Sma-1-15169	NS_000200.1	-	119492712	119492664
Sma-1-15170	NW_003038456.1	-	31934	31886
Sma-1-15171	NS_000200.1	-	330902198	330902150
Sma-1-15172	NW_003038252.1	+	9637	9685
Sma-1-15173	NS_000200.1	+	318865225	318865273
Sma-1-15174	NW_003038020.1	-	49930	49882
Sma-1-15175	NS_000200.1	-	234017274	234017226
Sma-1-15176	NW_003037907.1	+	364600	364648
Sma-1-15177	NS_000200.1	+	151769089	151769137
Sma-1-15178	NW_003037915.1	-	1233207	1233159
Sma-1-15179	NS_000200.1	-	163133193	163133145
Sma-1-15180	NW_003038012.1	+	538547	538595
Sma-1-15181	NS_000200.1	+	228862486	228862534
Sma-1-15182	NW_003038028.1	-	264426	264378
Sma-1-15183	NS_000200.1	-	241572000	241571952
Sma-1-15184	NW_003037979.1	-	5181	5133
Sma-1-15185	NS_000200.1	-	210066201	210066153
Sma-1-15186	NW_003026616.1	+	155937	155985
Sma-1-15187	NS_000200.1	+	317512536	317512584
Sma-1-15188	NW_003038012.1	+	588739	588787
Sma-1-15189	NS_000200.1	+	228912678	228912726
Sma-1-15190	NW_003037903.1	-	673097	673048
Sma-1-15191	NS_000200.1	-	148227178	148227129
Sma-1-15192	NW_003037851.1	+	525071	525119
Sma-1-15193	NS_000200.1	+	93928381	93928429
Sma-1-15194	NW_003037927.1	-	72315	72267
Sma-1-15195	NS_000200.1	-	174049678	174049630
Sma-1-15196	NW_003038455.1	-	87640	87592

Sma-1-15197	NW_003037854.1	-	1889558	1889510
Sma-1-15198	NS_000200.1	-	330869691	330869643
Sma-1-15199	NS_000200.1	-	98793979	98793931
Sma-1-15200	NW_003037887.1	+	968965	969013
Sma-1-15201	NS_000200.1	+	131432943	131432991
Sma-1-15202	NW_003037971.1	-	652131	652083
Sma-1-15203	NS_000200.1	-	202274181	202274133
Sma-1-15204	NW_003037831.1	-	779774	779725
Sma-1-15205	NS_000200.1	-	70229076	70229027
Sma-1-15206	NW_003037946.1	-	1023462	1023414
Sma-1-15207	NS_000200.1	-	191717322	191717274
Sma-1-15208	NW_003037961.1	-	230921	230873
Sma-1-15209	NS_000200.1	-	195855163	195855115
Sma-1-15210	NW_003037961.1	+	219790	219838
Sma-1-15211	NS_000200.1	+	195844032	195844080
Sma-1-15212	NW_003038048.1	+	187964	188012
Sma-1-15213	NS_000200.1	+	255765795	255765843
Sma-1-15214	NW_003038120.1	+	206303	206351
Sma-1-15215	NS_000200.1	+	278391526	278391574
Sma-1-15216	NW_003038016.1	-	87980	87932
Sma-1-15217	NS_000200.1	-	231204517	231204469
Sma-1-15218	NW_003037821.1	-	301014	300966
Sma-1-15219	NS_000200.1	-	53445498	53445450
Sma-1-15220	NW_003038033.1	+	598525	598573
Sma-1-15221	NS_000200.1	+	246578527	246578575
Sma-1-15222	NW_003038037.1	+	1000620	1000668
Sma-1-15223	NS_000200.1	+	251156029	251156077
Sma-1-15224	NW_003020275.1	+	597	645
Sma-1-15225	NS_000200.1	+	371326588	371326636
Sma-1-15226	NW_003037859.1	-	622597	622549
Sma-1-15227	NS_000200.1	-	103301438	103301390
Sma-1-15228	NW_003038032.1	+	136285	136333
Sma-1-15229	NS_000200.1	+	245295445	245295493
Sma-1-15230	NW_003037868.1	+	309209	309257
Sma-1-15231	NS_000200.1	+	116280921	116280969
Sma-1-15232	NW_003037868.1	+	40068	40116
Sma-1-15233	NS_000200.1	+	116011780	116011828
Sma-1-15234	NW_003037937.1	+	552015	552063
Sma-1-15235	NS_000200.1	+	181751924	181751972
Sma-1-15236	NW_003037888.1	+	542519	542567
Sma-1-15237	NS_000200.1	+	132751011	132751059
Sma-1-15238	NW_003037888.1	-	650132	650084
Sma-1-15239	NS_000200.1	-	132858624	132858576
Sma-1-15240	NW_003037933.1	+	207205	207253
Sma-1-15241	NS_000200.1	+	178620377	178620425
Sma-1-15242	NW_003021247.1	+	882	930
Sma-1-15243	NS_000200.1	+	371690048	371690096
Sma-1-15244	NW_003026510.1	-	305505	305457
Sma-1-15245	NS_000200.1	-	283745686	283745638
Sma-1-15246	NW_003038261.1	+	618661	618709
Sma-1-15247	NS_000200.1	+	324232312	324232360
Sma-1-15248	NW_003037830.1	-	187349	187301

Sma-1-15249	NS_000200.1	-	68077720	68077672
Sma-1-15250	NW_003021693.1	+	793	841
Sma-1-15251	NS_000200.1	+	348316529	348316577
Sma-1-15252	NW_003037937.1	-	382621	382573
Sma-1-15253	NS_000200.1	-	181582530	181582482
Sma-1-15254	NW_003037943.1	+	749856	749904
Sma-1-15255	NS_000200.1	+	187771389	187771437
Sma-1-15256	NW_003037813.1	-	1024889	1024841
Sma-1-15257	NS_000200.1	-	42011752	42011704
Sma-1-15258	NW_003037891.1	-	331795	331747
Sma-1-15259	NS_000200.1	-	138213073	138213025
Sma-1-15260	NW_003038244.1	+	120205	120253
Sma-1-15261	NS_000200.1	+	315265410	315265458
Sma-1-15262	NW_003037825.1	-	1440401	1440353
Sma-1-15263	NS_000200.1	-	60570183	60570135
Sma-1-15264	NW_003037798.1	-	1896026	1895979
Sma-1-15265	NS_000200.1	-	16155184	16155137
Sma-1-15266	NW_003037859.1	+	848435	848483
Sma-1-15267	NS_000200.1	+	103527276	103527324
Sma-1-15268	NW_003038028.1	+	811525	811573
Sma-1-15269	NS_000200.1	+	242119099	242119147
Sma-1-15270	NW_003038502.1	+	112809	112857
Sma-1-15271	NS_000200.1	+	335111854	335111902
Sma-1-15272	NW_003037807.1	+	65560	65608
Sma-1-15273	NS_000200.1	+	30327076	30327124
Sma-1-15274	NW_003038118.1	+	178058	178106
Sma-1-15275	NS_000200.1	+	277742185	277742233
Sma-1-15276	NW_003037905.1	-	452982	452934
Sma-1-15277	NS_000200.1	-	149721994	149721946
Sma-1-15278	NW_003038505.1	+	101837	101885
Sma-1-15279	NS_000200.1	+	336181174	336181222
Sma-1-15280	NW_003037913.1	-	910496	910448
Sma-1-15281	NS_000200.1	-	159474247	159474199
Sma-1-15282	NW_003026505.1	+	130135	130183
Sma-1-15283	NS_000200.1	+	279383257	279383305
Sma-1-15284	NW_003035671.1	+	11080	11128
Sma-1-15285	NS_000200.1	+	331644459	331644507
Sma-1-15286	NW_003037786.1	+	83377	83425
Sma-1-15287	NS_000200.1	+	380176737	380176785
Sma-1-15288	NW_003037851.1	-	900375	900327
Sma-1-15289	NS_000200.1	-	94303685	94303637
Sma-1-15290	NW_003038518.1	+	886590	886638
Sma-1-15291	NS_000200.1	+	342523423	342523471
Sma-1-15292	NW_003031186.1	-	813	765
Sma-1-15293	NS_000200.1	-	325961636	325961588
Sma-1-15294	NW_003037824.1	+	443334	443382
Sma-1-15295	NS_000200.1	+	57374274	57374322
Sma-1-15296	NW_003037834.1	-	1062572	1062524
Sma-1-15297	NS_000200.1	-	74223058	74223010
Sma-1-15298	NW_003026460.1	+	396455	396503
Sma-1-15299	NS_000200.1	+	170918661	170918709
Sma-1-15300	NW_003037844.1	-	3211	3163

Sma-1-15301	NS_000200.1	-	86186657	86186609
Sma-1-15302	NW_003037793.1	+	440043	440091
Sma-1-15303	NS_000200.1	+	440043	440091
Sma-1-15304	NW_003038225.1	+	95861	95909
Sma-1-15305	NS_000200.1	+	309950525	309950573
Sma-1-15306	NW_003038069.1	-	62191	62143
Sma-1-15307	NS_000200.1	-	263905597	263905549
Sma-1-15308	NW_003026619.1	-	319496	319448
Sma-1-15309	NS_000200.1	-	322510240	322510192
Sma-1-15310	NW_003038022.1	+	239680	239728
Sma-1-15311	NS_000200.1	+	235920522	235920570
Sma-1-15312	NW_003038229.1	+	4075	4123
Sma-1-15313	NS_000200.1	+	311121297	311121345
Sma-1-15314	NW_003037909.1	-	170586	170538
Sma-1-15315	NS_000200.1	-	153712235	153712187
Sma-1-15316	NW_003038071.1	+	132667	132715
Sma-1-15317	NS_000200.1	+	264935710	264935758
Sma-1-15318	NW_003038254.1	+	167496	167544
Sma-1-15319	NS_000200.1	+	320203054	320203102
Sma-1-15320	NW_003038152.1	+	329257	329305
Sma-1-15321	NS_000200.1	+	292934981	292935029
Sma-1-15322	NW_003037816.1	-	781185	781137
Sma-1-15323	NS_000200.1	-	46990057	46990009
Sma-1-15324	NW_003037802.1	-	894290	894242
Sma-1-15325	NS_000200.1	-	22897084	22897036
Sma-1-15326	NW_003038473.1	-	27944	27896
Sma-1-15327	NW_003038473.1	-	6119	6071
Sma-1-15328	NW_003038189.1	-	7907	7859
Sma-1-15329	NS_000200.1	-	332198229	332198181
Sma-1-15330	NS_000200.1	-	332176404	332176356
Sma-1-15331	NS_000200.1	-	302345019	302344971
Sma-1-15332	NW_003037847.1	-	26585	26537
Sma-1-15333	NS_000200.1	-	88678315	88678267
Sma-1-15334	NW_003037967.1	-	589528	589480
Sma-1-15335	NS_000200.1	-	199829499	199829451
Sma-1-15336	NW_003037859.1	-	437181	437133
Sma-1-15337	NS_000200.1	-	103116022	103115974
Sma-1-15338	NW_003037932.1	-	757839	757791
Sma-1-15339	NS_000200.1	-	178291035	178290987
Sma-1-15340	NW_003038024.1	+	590121	590169
Sma-1-15341	NS_000200.1	+	238487150	238487198
Sma-1-15342	NW_003037846.1	+	639027	639075
Sma-1-15343	NS_000200.1	+	88273597	88273645
Sma-1-15344	NW_003038073.1	+	356799	356847
Sma-1-15345	NS_000200.1	+	265879232	265879280
Sma-1-15346	NW_003037846.1	-	223240	223192
Sma-1-15347	NS_000200.1	-	87857810	87857762
Sma-1-15348	NW_003037932.1	-	706626	706578
Sma-1-15349	NS_000200.1	-	178239822	178239774
Sma-1-15350	NW_003037964.1	-	433707	433659
Sma-1-15351	NS_000200.1	-	198035229	198035181
Sma-1-15352	NW_003037844.1	-	565637	565589

Sma-1-15353	NS_000200.1	-	86749083	86749035
Sma-1-15354	NW_003038247.1	+	296451	296499
Sma-1-15355	NS_000200.1	+	316245179	316245227
Sma-1-15356	NW_003037842.1	+	460444	460492
Sma-1-15357	NS_000200.1	+	85559634	85559682
Sma-1-15358	NW_003037823.1	-	874736	874688
Sma-1-15359	NS_000200.1	-	56791355	56791307
Sma-1-15360	NW_003037861.1	-	393307	393259
Sma-1-15361	NS_000200.1	-	105169761	105169713
Sma-1-15362	NW_003038008.1	+	672332	672380
Sma-1-15363	NS_000200.1	+	226345428	226345476
Sma-1-15364	NW_003031741.1	+	109	157
Sma-1-15365	NS_000200.1	+	364302179	364302227
Sma-1-15366	NW_003037810.1	-	109986	109938
Sma-1-15367	NS_000200.1	-	36714508	36714460
Sma-1-15368	NW_003038147.1	-	418977	418929
Sma-1-15369	NS_000200.1	-	289946720	289946672
Sma-1-15370	NW_003038079.1	+	86204	86252
Sma-1-15371	NS_000200.1	+	268216192	268216240
Sma-1-15372	NW_003032034.1	-	1084	1036
Sma-1-15373	NS_000200.1	-	376900758	376900710
Sma-1-15374	NW_003038030.1	-	281892	281844
Sma-1-15375	NS_000200.1	-	243417759	243417711
Sma-1-15376	NW_003037882.1	+	244922	244970
Sma-1-15377	NS_000200.1	+	125536596	125536644
Sma-1-15378	NW_003037822.1	+	1527270	1527318
Sma-1-15379	NS_000200.1	+	55835276	55835324
Sma-1-15380	NW_003038151.1	+	200076	200124
Sma-1-15381	NS_000200.1	+	292107319	292107367
Sma-1-15382	NW_003022484.1	-	2275	2227
Sma-1-15383	NW_003037977.1	+	548208	548256
Sma-1-15384	NW_003037977.1	-	547911	547863
Sma-1-15385	NS_000200.1	+	208834228	208834276
Sma-1-15386	NS_000200.1	-	365023706	365023658
Sma-1-15387	NS_000200.1	-	208833931	208833883
Sma-1-15388	NW_003038149.1	-	400079	400031
Sma-1-15389	NS_000200.1	-	291428692	291428644
Sma-1-15390	NW_003037916.1	+	1217062	1217111
Sma-1-15391	NS_000200.1	+	164410473	164410522
Sma-1-15392	NW_003037886.1	-	221335	221287
Sma-1-15393	NS_000200.1	-	129476902	129476854
Sma-1-15394	NW_003037867.1	+	130891	130939
Sma-1-15395	NS_000200.1	+	115731710	115731758
Sma-1-15396	NW_003038152.1	-	43405	43357
Sma-1-15397	NS_000200.1	-	292649129	292649081
Sma-1-15398	NW_003037868.1	-	215848	215800
Sma-1-15399	NS_000200.1	-	116187560	116187512
Sma-1-15400	NW_003021177.1	-	1647	1599
Sma-1-15401	NS_000200.1	-	371516692	371516644
Sma-1-15402	NW_003037797.1	+	518460	518508
Sma-1-15403	NS_000200.1	+	10946320	10946368
Sma-1-15404	NW_003037944.1	-	531346	531298

Sma-1-15405	NS_000200.1	-	188752699	188752651
Sma-1-15406	NW_003038448.1	-	18645	18597
Sma-1-15407	NW_003038033.1	+	224698	224746
Sma-1-15408	NS_000200.1	+	246204700	246204748
Sma-1-15409	NS_000200.1	-	330395441	330395393
Sma-1-15410	NW_003037804.1	-	1174689	1174641
Sma-1-15411	NS_000200.1	-	26314709	26314661
Sma-1-15412	NW_003039130.1	-	142018	141970
Sma-1-15413	NS_000200.1	-	317816629	317816581
Sma-1-15414	NW_003035880.1	+	267121	267169
Sma-1-15415	NS_000200.1	+	339508185	339508233
Sma-1-15416	NW_003037944.1	+	196283	196331
Sma-1-15417	NS_000200.1	+	188417636	188417684
Sma-1-15418	NW_003038001.1	-	93417	93369
Sma-1-15419	NS_000200.1	-	222802978	222802930
Sma-1-15420	NW_003035881.1	+	341830	341878
Sma-1-15421	NS_000200.1	+	341406035	341406083
Sma-1-15422	NW_003037797.1	+	809069	809117
Sma-1-15423	NS_000200.1	+	11236929	11236977
Sma-1-15424	NW_003023016.1	-	728	680
Sma-1-15425	NS_000200.1	-	372063553	372063505
Sma-1-15426	NW_003023151.1	-	528	480
Sma-1-15427	NW_003037822.1	+	1108847	1108895
Sma-1-15428	NS_000200.1	+	55416853	55416901
Sma-1-15429	NS_000200.1	-	368382617	368382569
Sma-1-15430	NW_003037830.1	-	753208	753160
Sma-1-15431	NS_000200.1	-	68643579	68643531
Sma-1-15432	NW_003026469.1	+	181105	181153
Sma-1-15433	NS_000200.1	+	217802764	217802812
Sma-1-15434	NW_003024303.1	+	1395	1443
Sma-1-15435	NS_000200.1	+	365477701	365477749
Sma-1-15436	NW_003037878.1	-	248837	248789
Sma-1-15437	NS_000200.1	-	122302315	122302267
Sma-1-15438	NW_003037940.1	-	81044	80996
Sma-1-15439	NS_000200.1	-	183823174	183823126
Sma-1-15440	NW_003037774.1	-	16136	16088
Sma-1-15441	NS_000200.1	-	378601513	378601465
Sma-1-15442	NW_003037917.1	+	394268	394316
Sma-1-15443	NS_000200.1	+	165431072	165431120
Sma-1-15444	NW_003037940.1	-	397407	397359
Sma-1-15445	NS_000200.1	-	184139537	184139489
Sma-1-15446	NW_003037928.1	+	85163	85211
Sma-1-15447	NS_000200.1	+	174409116	174409164
Sma-1-15448	NW_003037988.1	+	40534	40582
Sma-1-15449	NS_000200.1	+	216034352	216034400
Sma-1-15450	NW_003037800.1	+	1067777	1067825
Sma-1-15451	NS_000200.1	+	19697137	19697185
Sma-1-15452	NW_003026601.1	-	14327	14279
Sma-1-15453	NS_000200.1	-	306395936	306395888
Sma-1-15454	NW_003037801.1	-	860541	860493
Sma-1-15455	NS_000200.1	-	21950021	21949973
Sma-1-15456	NW_003022125.1	+	1626	1674

Sma-1-15457	NS_000200.1	+	360760326	360760374
Sma-1-15458	NW_003037782.1	-	179924	179876
Sma-1-15459	NS_000200.1	-	379503367	379503319
Sma-1-15460	NW_003024365.1	+	395	443
Sma-1-15461	NW_003037846.1	-	722512	722464
Sma-1-15462	NW_003037921.1	-	194590	194542
Sma-1-15463	NW_003038014.1	+	289934	289982
Sma-1-15464	NW_003038027.1	-	906429	906381
Sma-1-15465	NW_003038034.1	+	863860	863908
Sma-1-15466	NW_003038128.1	+	53945	53993
Sma-1-15467	NW_003038128.1	+	66104	66152
Sma-1-15468	NS_000200.1	+	230030623	230030671
Sma-1-15469	NS_000200.1	+	247805616	247805664
Sma-1-15470	NS_000200.1	+	280772233	280772281
Sma-1-15471	NS_000200.1	+	280784392	280784440
Sma-1-15472	NS_000200.1	+	365592588	365592636
Sma-1-15473	NS_000200.1	-	241277143	241277095
Sma-1-15474	NS_000200.1	-	169724725	169724677
Sma-1-15475	NS_000200.1	-	88357082	88357034
Sma-1-15476	NW_003038005.1	+	390811	390859
Sma-1-15477	NS_000200.1	+	224796765	224796813
Sma-1-15478	NW_003032038.1	+	218	266
Sma-1-15479	NW_003038036.1	+	421005	421053
Sma-1-15480	NS_000200.1	+	249354857	249354905
Sma-1-15481	NS_000200.1	+	376922455	376922503
Sma-1-15482	NW_003037937.1	-	156465	156417
Sma-1-15483	NS_000200.1	-	181356374	181356326
Sma-1-15484	NW_003037800.1	-	1561500	1561452
Sma-1-15485	NS_000200.1	-	20190860	20190812
Sma-1-15486	NW_003037800.1	-	1708861	1708813
Sma-1-15487	NS_000200.1	-	20338221	20338173
Sma-1-15488	NW_003037939.1	+	857812	857860
Sma-1-15489	NS_000200.1	+	183727566	183727614
Sma-1-15490	NW_003037800.1	+	20907	20955
Sma-1-15491	NS_000200.1	+	18650267	18650315
Sma-1-15492	NW_003037914.1	+	1531131	1531179
Sma-1-15493	NS_000200.1	+	161675381	161675429
Sma-1-15494	NW_003026464.1	+	368271	368319
Sma-1-15495	NS_000200.1	+	203668580	203668628
Sma-1-15496	NW_003037807.1	+	307720	307768
Sma-1-15497	NS_000200.1	+	30569236	30569284
Sma-1-15498	NW_003037814.1	-	148889	148841
Sma-1-15499	NS_000200.1	-	43132749	43132701
Sma-1-15500	NW_003037937.1	+	560803	560851
Sma-1-15501	NW_003037904.1	+	301279	301327
Sma-1-15502	NS_000200.1	+	149015778	149015826
Sma-1-15503	NS_000200.1	+	181760712	181760760
Sma-1-15504	NW_003037797.1	+	3000757	3000805
Sma-1-15505	NS_000200.1	+	13428617	13428665
Sma-1-15506	NW_003037809.1	+	2199131	2199179
Sma-1-15507	NW_003037822.1	+	290577	290625
Sma-1-15508	NW_003037829.1	-	1019466	1019418

Sma-1-15509	NW_003037953.1	+	38565	38613
Sma-1-15510	NW_003038482.1	+	3242	3290
Sma-1-15511	NS_000200.1	+	35863595	35863643
Sma-1-15512	NS_000200.1	+	54598583	54598631
Sma-1-15513	NS_000200.1	+	192951616	192951664
Sma-1-15514	NS_000200.1	+	332980693	332980741
Sma-1-15515	NS_000200.1	-	66920004	66919956
Sma-1-15516	NW_003037800.1	-	617649	617601
Sma-1-15517	NS_000200.1	-	19247009	19246961
Sma-1-15518	NW_003037808.1	+	906879	906927
Sma-1-15519	NW_003037953.1	-	115522	115474
Sma-1-15520	NS_000200.1	+	32693516	32693564
Sma-1-15521	NS_000200.1	-	193028573	193028525
Sma-1-15522	NW_003037795.1	-	312303	312255
Sma-1-15523	NS_000200.1	-	6703186	6703138
Sma-1-15524	NW_003037799.1	+	984757	984805
Sma-1-15525	NS_000200.1	+	17975111	17975159
Sma-1-15526	NW_003037800.1	+	1422932	1422980
Sma-1-15527	NS_000200.1	+	20052292	20052340
Sma-1-15528	NW_003037928.1	+	867602	867650
Sma-1-15529	NW_003037981.1	+	1251143	1251191
Sma-1-15530	NS_000200.1	+	175191555	175191603
Sma-1-15531	NS_000200.1	+	213649011	213649059
Sma-1-15532	NW_003037825.1	+	356306	356354
Sma-1-15533	NW_003038025.1	-	524651	524603
Sma-1-15534	NW_003038150.1	+	329491	329539
Sma-1-15535	NS_000200.1	+	59486088	59486136
Sma-1-15536	NS_000200.1	+	291808229	291808277
Sma-1-15537	NS_000200.1	-	239382896	239382848
Sma-1-15538	NW_003037874.1	-	267876	267828
Sma-1-15539	NS_000200.1	-	119692826	119692778
Sma-1-15540	NW_003026472.1	-	34830	34782
Sma-1-15541	NS_000200.1	-	236446822	236446774
Sma-1-15542	NW_003038032.1	-	735281	735233
Sma-1-15543	NS_000200.1	-	245894441	245894393
Sma-1-15544	NW_003037788.1	+	199857	199905
Sma-1-15545	NS_000200.1	+	380647095	380647143
Sma-1-15546	NW_003037795.1	+	1347384	1347432
Sma-1-15547	NW_003037849.1	-	809742	809694
Sma-1-15548	NS_000200.1	+	7738267	7738315
Sma-1-15549	NS_000200.1	-	91786015	91785967
Sma-1-15550	NW_003037935.1	+	591007	591055
Sma-1-15551	NS_000200.1	+	180007542	180007590
Sma-1-15552	NW_003037867.1	+	13130	13178
Sma-1-15553	NS_000200.1	+	115613949	115613997
Sma-1-15554	NW_003026465.1	+	775796	775844
Sma-1-15555	NS_000200.1	+	208168390	208168438
Sma-1-15556	NW_003037906.1	-	234194	234146
Sma-1-15557	NW_003038030.1	-	268082	268034
Sma-1-15558	NS_000200.1	-	243403949	243403901
Sma-1-15559	NS_000200.1	-	150541973	150541925
Sma-1-15560	NW_003038257.1	-	244398	244350

Sma-1-15561	NS_000200.1	-	321418726	321418678
Sma-1-15562	NW_003029535.1	-	99	51
Sma-1-15563	NW_003038514.1	-	79753	79705
Sma-1-15564	NW_003026466.1	+	63110	63158
Sma-1-15565	NW_003037794.1	-	139997	139949
Sma-1-15566	NW_003037862.1	-	264176	264128
Sma-1-15567	NW_003037935.1	+	578097	578145
Sma-1-15568	NW_003037981.1	+	128562	128610
Sma-1-15569	NW_003038034.1	+	832067	832115
Sma-1-15570	NW_003038072.1	+	79204	79252
Sma-1-15571	NW_003038224.1	+	221200	221248
Sma-1-15572	NW_003038152.1	-	154121	154073
Sma-1-15573	NS_000200.1	+	179994632	179994680
Sma-1-15574	NS_000200.1	+	212526430	212526478
Sma-1-15575	NS_000200.1	+	215330217	215330265
Sma-1-15576	NS_000200.1	+	247773823	247773871
Sma-1-15577	NS_000200.1	+	265241432	265241480
Sma-1-15578	NS_000200.1	+	309632928	309632976
Sma-1-15579	NS_000200.1	-	362227835	362227787
Sma-1-15580	NS_000200.1	-	339704292	339704244
Sma-1-15581	NS_000200.1	-	292759845	292759797
Sma-1-15582	NS_000200.1	-	105915837	105915789
Sma-1-15583	NS_000200.1	-	4319592	4319544
Sma-1-15584	NW_003038028.1	-	264767	264719
Sma-1-15585	NS_000200.1	-	241572341	241572293
Sma-1-15586	NW_003038032.1	-	768057	768009
Sma-1-15587	NW_003038117.1	-	90276	90228
Sma-1-15588	NS_000200.1	-	277408101	277408053
Sma-1-15589	NS_000200.1	-	245927217	245927169
Sma-1-15590	NW_003038073.1	-	39763	39715
Sma-1-15591	NS_000200.1	-	265562196	265562148
Sma-1-15592	NW_003037801.1	+	568594	568642
Sma-1-15593	NS_000200.1	+	21658074	21658122
Sma-1-15594	NW_003037915.1	+	498618	498666
Sma-1-15595	NS_000200.1	+	162398604	162398652
Sma-1-15596	NW_003037909.1	+	603874	603922
Sma-1-15597	NS_000200.1	+	154145523	154145571
Sma-1-15598	NW_003025187.1	+	256	304
Sma-1-15599	NW_003037977.1	-	589812	589764
Sma-1-15600	NS_000200.1	+	361355848	361355896
Sma-1-15601	NS_000200.1	-	208875832	208875784
Sma-1-15602	NW_003038143.1	+	413127	413175
Sma-1-15603	NS_000200.1	+	288172619	288172667
Sma-1-15604	NW_003038261.1	-	965956	965908
Sma-1-15605	NS_000200.1	-	324579607	324579559
Sma-1-15606	NW_003030815.1	+	775	823
Sma-1-15607	NW_003037805.1	-	1223386	1223338
Sma-1-15608	NS_000200.1	+	370303974	370304022
Sma-1-15609	NS_000200.1	-	27781230	27781182
Sma-1-15610	NW_003037786.1	+	123890	123938
Sma-1-15611	NS_000200.1	+	380217250	380217298
Sma-1-15612	NW_003037810.1	+	454571	454619

Sma-1-15613	NW_003037810.1	-	439308	439260
Sma-1-15614	NW_003037866.1	+	1342491	1342539
Sma-1-15615	NW_003037897.1	+	289554	289602
Sma-1-15616	NW_003037905.1	+	41721	41769
Sma-1-15617	NW_003037909.1	+	868196	868244
Sma-1-15618	NW_003037930.1	+	817675	817723
Sma-1-15619	NW_003038008.1	-	354466	354418
Sma-1-15620	NS_000200.1	+	37059093	37059141
Sma-1-15621	NS_000200.1	+	112830290	112830338
Sma-1-15622	NS_000200.1	+	143771930	143771978
Sma-1-15623	NS_000200.1	+	149310733	149310781
Sma-1-15624	NS_000200.1	+	154409845	154409893
Sma-1-15625	NS_000200.1	+	176675639	176675687
Sma-1-15626	NS_000200.1	-	226027562	226027514
Sma-1-15627	NS_000200.1	-	37043830	37043782
Sma-1-15628	NW_003037934.1	+	194679	194727
Sma-1-15629	NW_003037916.1	-	1109503	1109455
Sma-1-15630	NS_000200.1	+	179241426	179241474
Sma-1-15631	NS_000200.1	-	164302914	164302866
Sma-1-15632	NW_003037794.1	+	1081830	1081878
Sma-1-15633	NW_003038172.1	-	43972	43924
Sma-1-15634	NS_000200.1	+	5261425	5261473
Sma-1-15635	NS_000200.1	-	300717487	300717439
Sma-1-15636	NW_003029979.1	-	1050	1002
Sma-1-15637	NS_000200.1	-	370271236	370271188
Sma-1-15638	NW_003038161.1	+	36688	36736
Sma-1-15639	NS_000200.1	+	297406539	297406587
Sma-1-15640	NW_003032374.1	-	944	896
Sma-1-15641	NW_003037809.1	+	46796	46844
Sma-1-15642	NW_003038055.1	+	18920	18968
Sma-1-15643	NS_000200.1	+	33711260	33711308
Sma-1-15644	NS_000200.1	+	258718242	258718290
Sma-1-15645	NS_000200.1	-	345973859	345973811
Sma-1-15646	NW_003037820.1	+	462318	462366
Sma-1-15647	NW_003037977.1	-	600125	600077
Sma-1-15648	NS_000200.1	+	52735300	52735348
Sma-1-15649	NS_000200.1	-	208886145	208886097
Sma-1-15650	NW_003026464.1	+	129482	129530
Sma-1-15651	NS_000200.1	+	203429791	203429839
Sma-1-15652	NW_003026458.1	-	518916	518868
Sma-1-15653	NS_000200.1	-	102387146	102387098
Sma-1-15654	NW_003026458.1	+	528458	528506
Sma-1-15655	NS_000200.1	+	102396688	102396736
Sma-1-15656	NW_003038457.1	+	15251	15299
Sma-1-15657	NS_000200.1	+	331097115	331097163
Sma-1-15658	NW_003038034.1	+	680496	680544
Sma-1-15659	NS_000200.1	+	247622252	247622300
Sma-1-15660	NW_003037880.1	-	291665	291617
Sma-1-15661	NS_000200.1	-	123975223	123975175
Sma-1-15662	NW_003026616.1	-	180994	180946
Sma-1-15663	NW_003037909.1	-	544510	544462
Sma-1-15664	NW_003038010.1	-	444944	444896

Sma-1-15665	NS_000200.1	-	317537593	317537545
Sma-1-15666	NS_000200.1	-	227263167	227263119
Sma-1-15667	NS_000200.1	-	154086159	154086111
Sma-1-15668	NW_003038152.1	+	338797	338845
Sma-1-15669	NS_000200.1	+	292944521	292944569
Sma-1-15670	NW_003035881.1	+	269137	269185
Sma-1-15671	NS_000200.1	+	341333342	341333390
Sma-1-15672	NW_003038161.1	+	273099	273147
Sma-1-15673	NS_000200.1	+	297642950	297642998
Sma-1-15674	NW_003037797.1	-	2506232	2506184
Sma-1-15675	NW_003037894.1	-	451859	451811
Sma-1-15676	NW_003037943.1	-	1151813	1151765
Sma-1-15677	NW_003038020.1	-	712889	712841
Sma-1-15678	NW_003026512.1	-	275879	275831
Sma-1-15679	NW_003038058.1	+	155518	155566
Sma-1-15680	NW_003038058.1	-	155300	155252
Sma-1-15681	NS_000200.1	+	259986718	259986766
Sma-1-15682	NS_000200.1	-	285369919	285369871
Sma-1-15683	NS_000200.1	-	259986500	259986452
Sma-1-15684	NS_000200.1	-	234680233	234680185
Sma-1-15685	NS_000200.1	-	188173346	188173298
Sma-1-15686	NS_000200.1	-	142348432	142348384
Sma-1-15687	NS_000200.1	-	12934092	12934044
Sma-1-15688	NW_003037885.1	-	1102856	1102808
Sma-1-15689	NS_000200.1	-	129200803	129200755
Sma-1-15690	NW_003038058.1	+	204647	204695
Sma-1-15691	NW_003038058.1	-	167470	167422
Sma-1-15692	NS_000200.1	+	260035847	260035895
Sma-1-15693	NS_000200.1	-	259998670	259998622
Sma-1-15694	NW_003032228.1	-	1564	1516
Sma-1-15695	NW_003037818.1	-	1106780	1106732
Sma-1-15696	NW_003037946.1	+	1039176	1039224
Sma-1-15697	NS_000200.1	+	191733036	191733084
Sma-1-15698	NS_000200.1	-	357951592	357951544
Sma-1-15699	NS_000200.1	-	49822187	49822139
Sma-1-15700	NW_003037915.1	-	628843	628795
Sma-1-15701	NS_000200.1	-	162528829	162528781
Sma-1-15702	NW_003037796.1	-	976349	976301
Sma-1-15703	NS_000200.1	-	9185993	9185945
Sma-1-15704	NW_003037796.1	-	1493390	1493342
Sma-1-15705	NS_000200.1	-	9703034	9702986
Sma-1-15706	NW_003037937.1	+	205453	205501
Sma-1-15707	NW_003037831.1	-	1389926	1389878
Sma-1-15708	NS_000200.1	+	181405362	181405410
Sma-1-15709	NS_000200.1	-	70839228	70839180
Sma-1-15710	NW_003037797.1	+	2604703	2604751
Sma-1-15711	NS_000200.1	+	13032563	13032611
Sma-1-15712	NW_003037977.1	-	932488	932440
Sma-1-15713	NS_000200.1	-	209218508	209218460
Sma-1-15714	NW_003037866.1	+	1116404	1116452
Sma-1-15715	NS_000200.1	+	112604203	112604251
Sma-1-15716	NW_003026468.1	+	381366	381414

Sma-1-15717	NW_003037882.1	-	544583	544535
Sma-1-15718	NS_000200.1	+	216575387	216575435
Sma-1-15719	NS_000200.1	-	125836257	125836209
Sma-1-15720	NW_003037879.1	-	550010	549962
Sma-1-15721	NS_000200.1	-	123552266	123552218
Sma-1-15722	NW_003026470.1	+	540419	540467
Sma-1-15723	NS_000200.1	+	219664388	219664436
Sma-1-15724	NW_003037951.1	+	15538	15586
Sma-1-15725	NW_003038006.1	+	133635	133683
Sma-1-15726	NS_000200.1	+	192693263	192693311
Sma-1-15727	NS_000200.1	+	225018119	225018167
Sma-1-15728	NW_003034096.1	+	1302	1350
Sma-1-15729	NS_000200.1	+	363730510	363730558
Sma-1-15730	NW_003034029.1	-	4590	4542
Sma-1-15731	NS_000200.1	-	363526453	363526405
Sma-1-15732	NW_003038147.1	-	386364	386316
Sma-1-15733	NS_000200.1	-	289914107	289914059
Sma-1-15734	NW_003037965.1	-	35227	35179
Sma-1-15735	NW_003037823.1	-	414926	414878
Sma-1-15736	NW_003038163.1	-	666383	666335
Sma-1-15737	NS_000200.1	-	299066663	299066615
Sma-1-15738	NS_000200.1	-	198324312	198324264
Sma-1-15739	NS_000200.1	-	56331545	56331497
Sma-1-15740	NW_003037830.1	+	977033	977081
Sma-1-15741	NS_000200.1	+	68867404	68867452
Sma-1-15742	NW_003038056.1	+	42619	42667
Sma-1-15743	NW_003038119.1	+	261645	261693
Sma-1-15744	NS_000200.1	+	259020270	259020318
Sma-1-15745	NS_000200.1	+	278169201	278169249
Sma-1-15746	NW_003037796.1	-	760243	760195
Sma-1-15747	NW_003037901.1	-	793219	793171
Sma-1-15748	NW_003038518.1	-	564478	564430
Sma-1-15749	NS_000200.1	-	342201311	342201263
Sma-1-15750	NS_000200.1	-	146641680	146641632
Sma-1-15751	NS_000200.1	-	8969887	8969839
Sma-1-15752	NW_003038257.1	+	121860	121908
Sma-1-15753	NS_000200.1	+	321296188	321296236
Sma-1-15754	NW_003026369.1	-	1228	1180
Sma-1-15755	NW_003038009.1	+	1961	2009
Sma-1-15756	NW_003038009.1	-	164552	164504
Sma-1-15757	NW_003038013.1	+	245800	245848
Sma-1-15758	NW_003037792.1	-	23069	23021
Sma-1-15759	NW_003037804.1	-	1356089	1356041
Sma-1-15760	NW_003037810.1	+	1274173	1274221
Sma-1-15761	NW_003037824.1	+	437048	437096
Sma-1-15762	NW_003037846.1	+	824456	824504
Sma-1-15763	NW_003037864.1	+	1019924	1019972
Sma-1-15764	NW_003037881.1	+	729410	729458
Sma-1-15765	NW_003037899.1	+	155031	155079
Sma-1-15766	NW_003037925.1	+	182255	182303
Sma-1-15767	NW_003038011.1	+	354250	354298
Sma-1-15768	NW_003038143.1	-	313239	313191

Sma-1-15769	NS_000200.1	+	37878695	37878743
Sma-1-15770	NS_000200.1	+	57367988	57368036
Sma-1-15771	NS_000200.1	+	88459026	88459074
Sma-1-15772	NS_000200.1	+	109512498	109512546
Sma-1-15773	NS_000200.1	+	125161201	125161249
Sma-1-15774	NS_000200.1	+	144674142	144674190
Sma-1-15775	NS_000200.1	+	172584258	172584306
Sma-1-15776	NS_000200.1	+	226376024	226376072
Sma-1-15777	NS_000200.1	+	227881900	227881948
Sma-1-15778	NS_000200.1	+	229635426	229635474
Sma-1-15779	NS_000200.1	-	382460413	382460365
Sma-1-15780	NS_000200.1	-	344836315	344836267
Sma-1-15781	NS_000200.1	-	288072731	288072683
Sma-1-15782	NS_000200.1	-	226538615	226538567
Sma-1-15783	NS_000200.1	-	26496109	26496061
Sma-1-15784	NW_003026472.1	-	58981	58933
Sma-1-15785	NS_000200.1	-	236470973	236470925
Sma-1-15786	NW_003037837.1	+	19253	19301
Sma-1-15787	NW_003038023.1	-	207977	207929
Sma-1-15788	NS_000200.1	+	76825111	76825159
Sma-1-15789	NS_000200.1	-	237368268	237368220
Sma-1-15790	NW_003038021.1	+	325575	325623
Sma-1-15791	NS_000200.1	+	235154150	235154198
Sma-1-15792	NW_003037833.1	-	746728	746680
Sma-1-15793	NS_000200.1	-	72937824	72937776
Sma-1-15794	NW_003037793.1	-	2778360	2778312
Sma-1-15795	NW_003037813.1	+	775533	775581
Sma-1-15796	NW_003037914.1	-	1487049	1487001
Sma-1-15797	NS_000200.1	+	41762396	41762444
Sma-1-15798	NS_000200.1	-	161631299	161631251
Sma-1-15799	NS_000200.1	-	2778360	2778312
Sma-1-15800	NW_003037863.1	-	1657940	1657892
Sma-1-15801	NS_000200.1	-	108416307	108416259
Sma-1-15802	NW_003037866.1	-	1116211	1116163
Sma-1-15803	NS_000200.1	-	112604010	112603962
Sma-1-15804	NW_003037885.1	+	484688	484736
Sma-1-15805	NS_000200.1	+	128582635	128582683
Sma-1-15806	NW_003038514.1	-	216283	216235
Sma-1-15807	NS_000200.1	-	339840822	339840774
Sma-1-15808	NW_003038038.1	+	780372	780420
Sma-1-15809	NS_000200.1	+	252683765	252683813
Sma-1-15810	NW_003037821.1	+	134605	134653
Sma-1-15811	NW_003037830.1	-	484336	484288
Sma-1-15812	NW_003039127.1	-	2192478	2192430
Sma-1-15813	NS_000200.1	+	53279089	53279137
Sma-1-15814	NS_000200.1	-	115269977	115269929
Sma-1-15815	NS_000200.1	-	68374707	68374659
Sma-1-15816	NW_003037887.1	-	37290	37242
Sma-1-15817	NS_000200.1	-	130501268	130501220
Sma-1-15818	NW_003038252.1	-	5844	5796
Sma-1-15819	NS_000200.1	-	318861432	318861384
Sma-1-15820	NW_003037877.1	+	479979	480027

Sma-1-15821	NS_000200.1	+	121518541	121518589
Sma-1-15822	NW_003038369.1	+	16123	16171
Sma-1-15823	NS_000200.1	+	327125062	327125110
Sma-1-15824	NW_003026624.1	-	24768	24720
Sma-1-15825	NS_000200.1	-	324894938	324894890
Sma-1-15826	NW_003037869.1	-	44741	44693
Sma-1-15827	NS_000200.1	-	116517086	116517038
Sma-1-15828	NW_003038444.1	+	48005	48053
Sma-1-15829	NS_000200.1	+	330122224	330122272
Sma-1-15830	NW_003037883.1	+	428860	428908
Sma-1-15831	NS_000200.1	+	126492583	126492631
Sma-1-15832	NW_003026612.1	-	143626	143578
Sma-1-15833	NW_003037804.1	-	1174337	1174289
Sma-1-15834	NW_003037830.1	-	432539	432491
Sma-1-15835	NW_003037833.1	-	22125	22077
Sma-1-15836	NW_003037912.1	+	720880	720928
Sma-1-15837	NW_003037923.1	-	639745	639697
Sma-1-15838	NW_003037932.1	-	822174	822126
Sma-1-15839	NW_003038038.1	-	52695	52647
Sma-1-15840	NW_003038257.1	+	191580	191628
Sma-1-15841	NW_003026515.1	+	176038	176086
Sma-1-15842	NS_000200.1	+	157532455	157532503
Sma-1-15843	NS_000200.1	+	290820449	290820497
Sma-1-15844	NS_000200.1	+	321365908	321365956
Sma-1-15845	NS_000200.1	-	312449013	312448965
Sma-1-15846	NS_000200.1	-	251956088	251956040
Sma-1-15847	NS_000200.1	-	178355370	178355322
Sma-1-15848	NS_000200.1	-	171695958	171695910
Sma-1-15849	NS_000200.1	-	72213221	72213173
Sma-1-15850	NS_000200.1	-	68322910	68322862
Sma-1-15851	NS_000200.1	-	26314357	26314309
Sma-1-15852	NW_003037927.1	-	69510	69462
Sma-1-15853	NS_000200.1	-	174046873	174046825
Sma-1-15854	NW_003037933.1	+	215023	215071
Sma-1-15855	NS_000200.1	+	178628195	178628243
Sma-1-15856	NW_003038029.1	-	570791	570743
Sma-1-15857	NS_000200.1	-	242771692	242771644
Sma-1-15858	NW_003037911.1	+	22277	22325
Sma-1-15859	NS_000200.1	+	155809630	155809678
Sma-1-15860	NW_003037935.1	-	353561	353513
Sma-1-15861	NW_003038161.1	-	310237	310189
Sma-1-15862	NW_003038161.1	-	302500	302452
Sma-1-15863	NS_000200.1	-	297680088	297680040
Sma-1-15864	NS_000200.1	-	297672351	297672303
Sma-1-15865	NS_000200.1	-	179770096	179770048
Sma-1-15866	NW_003037795.1	+	1319868	1319916
Sma-1-15867	NS_000200.1	+	7710751	7710799
Sma-1-15868	NW_003038037.1	-	986309	986261
Sma-1-15869	NS_000200.1	-	251141718	251141670
Sma-1-15870	NW_003038030.1	-	594555	594507
Sma-1-15871	NS_000200.1	-	243730422	243730374
Sma-1-15872	NW_003037795.1	+	1263194	1263242

Sma-1-15873	NS_000200.1	+	7654077	7654125
Sma-1-15874	NW_003037814.1	+	1471184	1471232
Sma-1-15875	NS_000200.1	+	44455044	44455092
Sma-1-15876	NW_003038032.1	+	565841	565889
Sma-1-15877	NS_000200.1	+	245725001	245725049
Sma-1-15878	NW_003037995.1	-	362765	362717
Sma-1-15879	NS_000200.1	-	220052533	220052485
Sma-1-15880	NW_003037793.1	+	2073048	2073096
Sma-1-15881	NS_000200.1	+	2073048	2073096
Sma-1-15882	NW_003038077.1	-	318161	318113
Sma-1-15883	NS_000200.1	-	267089801	267089753
Sma-1-15884	NW_003038261.1	+	42989	43037
Sma-1-15885	NS_000200.1	+	323656640	323656688
Sma-1-15886	NW_003038260.1	-	5558	5510
Sma-1-15887	NS_000200.1	-	323177333	323177285
Sma-1-15888	NW_003037941.1	+	295751	295799
Sma-1-15889	NS_000200.1	+	185030225	185030273
Sma-1-15890	NW_003037944.1	+	676662	676710
Sma-1-15891	NS_000200.1	+	188898015	188898063
Sma-1-15892	NW_003023212.1	-	2326	2278
Sma-1-15893	NW_003038033.1	-	221464	221416
Sma-1-15894	NS_000200.1	-	368492585	368492537
Sma-1-15895	NS_000200.1	-	246201466	246201418
Sma-1-15896	NW_003023538.1	-	578	530
Sma-1-15897	NW_003037898.1	+	419707	419755
Sma-1-15898	NS_000200.1	+	144403529	144403577
Sma-1-15899	NS_000200.1	-	373595936	373595888
Sma-1-15900	NW_003037797.1	-	3165405	3165357
Sma-1-15901	NS_000200.1	-	13593265	13593217
Sma-1-15902	NW_003036206.1	+	795	843
Sma-1-15903	NW_003038251.1	-	132159	132111
Sma-1-15904	NS_000200.1	+	347533648	347533696
Sma-1-15905	NS_000200.1	-	318463931	318463883
Sma-1-15906	NW_003037944.1	-	565429	565381
Sma-1-15907	NS_000200.1	-	188786782	188786734
Sma-1-15908	NW_003037824.1	+	989695	989743
Sma-1-15909	NS_000200.1	+	57920635	57920683
Sma-1-15910	NW_003026472.1	+	328941	328989
Sma-1-15911	NS_000200.1	+	236740933	236740981
Sma-1-15912	NW_003037898.1	+	447390	447438
Sma-1-15913	NW_003037969.1	-	252924	252876
Sma-1-15914	NS_000200.1	+	144431212	144431260
Sma-1-15915	NS_000200.1	-	200859051	200859003
Sma-1-15916	NW_003038081.1	+	245543	245591
Sma-1-15917	NS_000200.1	+	269407586	269407634
Sma-1-15918	NW_003038066.1	-	251118	251070
Sma-1-15919	NS_000200.1	-	262720062	262720014
Sma-1-15920	NW_003038033.1	-	959839	959791
Sma-1-15921	NS_000200.1	-	246939841	246939793
Sma-1-15922	NW_003026464.1	-	387612	387564
Sma-1-15923	NW_003037804.1	-	1130492	1130444
Sma-1-15924	NW_003037805.1	-	1613408	1613360

Sma-1-15925	NW_003037816.1	-	562663	562615
Sma-1-15926	NW_003037890.1	-	2057427	2057379
Sma-1-15927	NW_003037914.1	+	1636125	1636173
Sma-1-15928	NS_000200.1	+	161780375	161780423
Sma-1-15929	NS_000200.1	-	203687921	203687873
Sma-1-15930	NS_000200.1	-	137740788	137740740
Sma-1-15931	NS_000200.1	-	46771535	46771487
Sma-1-15932	NS_000200.1	-	28171252	28171204
Sma-1-15933	NS_000200.1	-	26270512	26270464
Sma-1-15934	NW_003037847.1	+	775868	775916
Sma-1-15935	NS_000200.1	+	89427598	89427646
Sma-1-15936	NW_003026458.1	+	7824	7872
Sma-1-15937	NW_003035881.1	-	194505	194457
Sma-1-15938	NS_000200.1	+	101876054	101876102
Sma-1-15939	NS_000200.1	-	341258710	341258662
Sma-1-15940	NW_003037805.1	+	1543595	1543643
Sma-1-15941	NS_000200.1	+	28101439	28101487
Sma-1-15942	NW_003038224.1	+	121840	121888
Sma-1-15943	NS_000200.1	+	309533568	309533616
Sma-1-15944	NW_003037858.1	+	84918	84966
Sma-1-15945	NW_003037916.1	+	1793649	1793697
Sma-1-15946	NS_000200.1	+	100749194	100749242
Sma-1-15947	NS_000200.1	+	164987060	164987108
Sma-1-15948	NW_003029782.1	+	145	193
Sma-1-15949	NS_000200.1	+	375568968	375569016
Sma-1-15950	NW_003037980.1	+	1265576	1265624
Sma-1-15951	NS_000200.1	+	212226438	212226486
Sma-1-15952	NW_003021785.1	-	566	518
Sma-1-15953	NS_000200.1	-	351851605	351851557
Sma-1-15954	NW_003037850.1	+	164137	164185
Sma-1-15955	NS_000200.1	+	92150167	92150215
Sma-1-15956	NW_003038069.1	-	128959	128911
Sma-1-15957	NS_000200.1	-	263972365	263972317
Sma-1-15958	NW_003038257.1	+	245509	245557
Sma-1-15959	NS_000200.1	+	321419837	321419885
Sma-1-15960	NW_003037873.1	+	169678	169726
Sma-1-15961	NS_000200.1	+	118908806	118908854
Sma-1-15962	NW_003021525.1	+	199	247
Sma-1-15963	NW_003021525.1	+	542	590
Sma-1-15964	NW_003021525.1	+	885	933
Sma-1-15965	NW_003021525.1	+	1228	1276
Sma-1-15966	NW_003021525.1	+	1571	1619
Sma-1-15967	NW_003021785.1	-	909	861
Sma-1-15968	NW_003021785.1	-	223	175
Sma-1-15969	NW_003024092.1	-	971	923
Sma-1-15970	NW_003024092.1	-	628	580
Sma-1-15971	NW_003024092.1	-	285	237
Sma-1-15972	NW_003025589.1	-	604	556
Sma-1-15973	NW_003025589.1	-	261	213
Sma-1-15974	NW_003025783.1	+	212	260
Sma-1-15975	NW_003025783.1	+	555	603
Sma-1-15976	NW_003025783.1	+	898	946

Sma-1-15977	NW_003030033.1	+	292	340
Sma-1-15978	NW_003030033.1	+	635	683
Sma-1-15979	NW_003032809.1	+	310	358
Sma-1-15980	NW_003032809.1	+	653	701
Sma-1-15981	NW_003032809.1	+	996	1044
Sma-1-15982	NW_003032809.1	+	1339	1387
Sma-1-15983	NW_003037934.1	+	230781	230829
Sma-1-15984	NW_003037797.1	+	1536134	1536182
Sma-1-15985	NW_003037869.1	+	156541	156589
Sma-1-15986	NW_003037911.1	-	365939	365891
Sma-1-15987	NW_003037935.1	-	286398	286350
Sma-1-15988	NW_003038025.1	+	628905	628953
Sma-1-15989	NW_003038238.1	-	156544	156496
Sma-1-15990	NW_003038238.1	-	156201	156153
Sma-1-15991	NW_003038148.1	-	396103	396055
Sma-1-15992	NW_003038152.1	+	389026	389074
Sma-1-15993	NS_000200.1	+	11963994	11964042
Sma-1-15994	NS_000200.1	+	116628886	116628934
Sma-1-15995	NS_000200.1	+	179277528	179277576
Sma-1-15996	NS_000200.1	+	239487150	239487198
Sma-1-15997	NS_000200.1	+	292994750	292994798
Sma-1-15998	NS_000200.1	+	348019553	348019601
Sma-1-15999	NS_000200.1	+	348019896	348019944
Sma-1-16000	NS_000200.1	+	348020239	348020287
Sma-1-16001	NS_000200.1	+	348020582	348020630
Sma-1-16002	NS_000200.1	+	348020925	348020973
Sma-1-16003	NS_000200.1	+	354080865	354080913
Sma-1-16004	NS_000200.1	+	354081208	354081256
Sma-1-16005	NS_000200.1	+	354081551	354081599
Sma-1-16006	NS_000200.1	+	354081894	354081942
Sma-1-16007	NS_000200.1	+	356525493	356525541
Sma-1-16008	NS_000200.1	+	356525836	356525884
Sma-1-16009	NS_000200.1	+	356526179	356526227
Sma-1-16010	NS_000200.1	+	357466688	357466736
Sma-1-16011	NS_000200.1	+	357467031	357467079
Sma-1-16012	NS_000200.1	-	356203815	356203767
Sma-1-16013	NS_000200.1	-	356203472	356203424
Sma-1-16014	NS_000200.1	-	351851948	351851900
Sma-1-16015	NS_000200.1	-	351851262	351851214
Sma-1-16016	NS_000200.1	-	348351099	348351051
Sma-1-16017	NS_000200.1	-	348350756	348350708
Sma-1-16018	NS_000200.1	-	348350413	348350365
Sma-1-16019	NS_000200.1	-	313763325	313763277
Sma-1-16020	NS_000200.1	-	313762982	313762934
Sma-1-16021	NS_000200.1	-	290470087	290470039
Sma-1-16022	NS_000200.1	-	179702933	179702885
Sma-1-16023	NS_000200.1	-	156153292	156153244
Sma-1-16024	NW_003038261.1	+	390544	390592
Sma-1-16025	NS_000200.1	+	324004195	324004243
Sma-1-16026	NW_003038537.1	-	764	716
Sma-1-16027	NW_003037914.1	-	1554290	1554242
Sma-1-16028	NS_000200.1	-	343334776	343334728

Sma-1-16029	NS_000200.1	-	161698540	161698492
Sma-1-16030	NW_003026502.1	+	11534	11582
Sma-1-16031	NS_000200.1	+	276047970	276048018
Sma-1-16032	NW_003030033.1	+	978	1026
Sma-1-16033	NS_000200.1	+	357467374	357467422
Sma-1-16034	NW_003037884.1	-	924945	924897
Sma-1-16035	NS_000200.1	-	127704610	127704562
Sma-1-16036	NW_003038249.1	-	160302	160254
Sma-1-16037	NS_000200.1	-	316842555	316842507
Sma-1-16038	NW_003037860.1	+	85967	86015
Sma-1-16039	NS_000200.1	+	103828995	103829043
Sma-1-16040	NW_003038132.1	+	196758	196806
Sma-1-16041	NS_000200.1	+	282617228	282617276
Sma-1-16042	NW_003037817.1	+	305015	305063
Sma-1-16043	NW_003037878.1	+	522579	522627
Sma-1-16044	NS_000200.1	+	47687367	47687415
Sma-1-16045	NS_000200.1	+	122576057	122576105
Sma-1-16046	NW_003037785.1	+	94364	94412
Sma-1-16047	NS_000200.1	+	379993031	379993079
Sma-1-16048	NW_003037920.1	-	224812	224764
Sma-1-16049	NW_003037972.1	-	618836	618788
Sma-1-16050	NS_000200.1	-	203094540	203094492
Sma-1-16051	NS_000200.1	-	169405619	169405571
Sma-1-16052	NW_003038046.1	+	113144	113192
Sma-1-16053	NS_000200.1	+	255186526	255186574
Sma-1-16054	NW_003023438.1	-	4508	4460
Sma-1-16055	NS_000200.1	-	373385396	373385348
Sma-1-16056	NW_003037916.1	+	1650586	1650634
Sma-1-16057	NS_000200.1	+	164843997	164844045
Sma-1-16058	NW_003039131.1	-	21978	21930
Sma-1-16059	NS_000200.1	-	328978883	328978835
Sma-1-16060	NW_003038034.1	-	831872	831824
Sma-1-16061	NS_000200.1	-	247773628	247773580
Sma-1-16062	NW_003038464.1	+	26318	26366
Sma-1-16063	NS_000200.1	+	331531585	331531633
Sma-1-16064	NW_003038010.1	-	335866	335816
Sma-1-16065	NS_000200.1	-	227154089	227154039
Sma-1-16066	NW_003037794.1	+	887824	887872
Sma-1-16067	NS_000200.1	+	5067419	5067467
Sma-1-16068	NW_003038038.1	+	582674	582722
Sma-1-16069	NS_000200.1	+	252486067	252486115
Sma-1-16070	NW_003038482.1	+	3584	3632
Sma-1-16071	NS_000200.1	+	332981035	332981083
Sma-1-16072	NW_003026609.1	-	37085	37037
Sma-1-16073	NS_000200.1	-	309692077	309692029
Sma-1-16074	NW_003037818.1	+	2278074	2278122
Sma-1-16075	NS_000200.1	+	50993481	50993529
Sma-1-16076	NW_003036430.1	-	1539	1491
Sma-1-16077	NS_000200.1	-	359969226	359969178
Sma-1-16078	NW_003035869.1	+	164409	164457
Sma-1-16079	NS_000200.1	+	335348609	335348657
Sma-1-16080	NW_003038081.1	-	331223	331175

Sma-1-16081	NS_000200.1	-	269493266	269493218
Sma-1-16082	NW_003038155.1	-	438905	438857
Sma-1-16083	NS_000200.1	-	294562023	294561975
Sma-1-16084	NW_003038286.1	+	1611	1659
Sma-1-16085	NW_003037885.1	+	1061954	1062002
Sma-1-16086	NS_000200.1	+	129159901	129159949
Sma-1-16087	NS_000200.1	+	325349480	325349528
Sma-1-16088	NW_003037818.1	+	914247	914295
Sma-1-16089	NS_000200.1	+	49629654	49629702
Sma-1-16090	NW_003035877.1	-	27746	27698
Sma-1-16091	NS_000200.1	-	337008280	337008232
Sma-1-16092	NW_003037944.1	+	674220	674268
Sma-1-16093	NS_000200.1	+	188895573	188895621
Sma-1-16094	NW_003035829.1	-	1048	1000
Sma-1-16095	NS_000200.1	-	364179514	364179466
Sma-1-16096	NW_003037911.1	-	981809	981761
Sma-1-16097	NW_003038518.1	-	323564	323516
Sma-1-16098	NS_000200.1	-	341960397	341960349
Sma-1-16099	NS_000200.1	-	156769162	156769114
Sma-1-16100	NW_003037824.1	-	973243	973195
Sma-1-16101	NW_003037930.1	+	626482	626530
Sma-1-16102	NS_000200.1	+	176484446	176484494
Sma-1-16103	NS_000200.1	-	57904183	57904135
Sma-1-16104	NW_003038163.1	+	681655	681703
Sma-1-16105	NS_000200.1	+	299081935	299081983
Sma-1-16106	NW_003037803.1	+	1328082	1328130
Sma-1-16107	NW_003037812.1	-	1406484	1406436
Sma-1-16108	NW_003037822.1	+	55198	55246
Sma-1-16109	NW_003037822.1	-	812302	812254
Sma-1-16110	NW_003038038.1	-	1170713	1170665
Sma-1-16111	NS_000200.1	+	24828619	24828667
Sma-1-16112	NS_000200.1	+	54363204	54363252
Sma-1-16113	NS_000200.1	-	253074106	253074058
Sma-1-16114	NS_000200.1	-	55120308	55120260
Sma-1-16115	NS_000200.1	-	40889223	40889175
Sma-1-16116	NW_003037864.1	+	531314	531362
Sma-1-16117	NS_000200.1	+	109023888	109023936
Sma-1-16118	NW_003038514.1	+	99281	99329
Sma-1-16119	NW_003037824.1	-	807554	807506
Sma-1-16120	NW_003037895.1	+	89607	89655
Sma-1-16121	NS_000200.1	+	142535809	142535857
Sma-1-16122	NS_000200.1	+	339723820	339723868
Sma-1-16123	NS_000200.1	-	57738494	57738446
Sma-1-16124	NW_003037823.1	-	960416	960368
Sma-1-16125	NW_003037832.1	+	749387	749435
Sma-1-16126	NW_003037832.1	-	782775	782727
Sma-1-16127	NW_003037906.1	-	745622	745574
Sma-1-16128	NW_003037975.1	-	678958	678910
Sma-1-16129	NS_000200.1	+	71958818	71958866
Sma-1-16130	NS_000200.1	-	206261182	206261134
Sma-1-16131	NS_000200.1	-	151053401	151053353
Sma-1-16132	NS_000200.1	-	71992206	71992158

Sma-1-16133	NS_000200.1	-	56877035	56876987
Sma-1-16134	NW_003037793.1	-	3347351	3347303
Sma-1-16135	NW_003037915.1	-	946758	946710
Sma-1-16136	NW_003038071.1	-	132620	132572
Sma-1-16137	NW_003038147.1	+	98143	98191
Sma-1-16138	NS_000200.1	+	289625886	289625934
Sma-1-16139	NS_000200.1	-	264935663	264935615
Sma-1-16140	NS_000200.1	-	162846744	162846696
Sma-1-16141	NS_000200.1	-	3347351	3347303
Sma-1-16142	NW_003026485.1	-	162234	162185
Sma-1-16143	NS_000200.1	-	258517139	258517090
Sma-1-16144	NW_003038072.1	+	51165	51213
Sma-1-16145	NW_003038120.1	-	20042	19994
Sma-1-16146	NS_000200.1	+	265213393	265213441
Sma-1-16147	NS_000200.1	-	278205265	278205217
Sma-1-16148	NW_003037991.1	+	315173	315221
Sma-1-16149	NS_000200.1	+	217464741	217464789
Sma-1-16150	NW_003037859.1	+	9985	10033
Sma-1-16151	NW_003037908.1	+	901826	901874
Sma-1-16152	NS_000200.1	+	102688826	102688874
Sma-1-16153	NS_000200.1	+	153374957	153375005
Sma-1-16154	NW_003038033.1	-	635362	635314
Sma-1-16155	NS_000200.1	-	246615364	246615316
Sma-1-16156	NW_003037978.1	-	23371	23323
Sma-1-16157	NS_000200.1	-	209291381	209291333
Sma-1-16158	NW_003031870.1	+	76	124
Sma-1-16159	NS_000200.1	+	367181565	367181613
Sma-1-16160	NW_003036157.1	+	91	139
Sma-1-16161	NW_003037809.1	+	947358	947406
Sma-1-16162	NW_003037813.1	-	328542	328494
Sma-1-16163	NW_003037824.1	+	300284	300332
Sma-1-16164	NW_003037839.1	-	1240227	1240179
Sma-1-16165	NW_003037866.1	+	1332210	1332258
Sma-1-16166	NW_003037906.1	+	6030	6078
Sma-1-16167	NW_003037933.1	-	269235	269187
Sma-1-16168	NW_003038255.1	+	23390	23438
Sma-1-16169	NS_000200.1	+	34611822	34611870
Sma-1-16170	NS_000200.1	+	57231224	57231272
Sma-1-16171	NS_000200.1	+	112820009	112820057
Sma-1-16172	NS_000200.1	+	150313809	150313857
Sma-1-16173	NS_000200.1	+	320412535	320412583
Sma-1-16174	NS_000200.1	+	347377277	347377325
Sma-1-16175	NS_000200.1	-	178682407	178682359
Sma-1-16176	NS_000200.1	-	80198377	80198329
Sma-1-16177	NS_000200.1	-	41315405	41315357
Sma-1-16178	NW_003038034.1	-	875187	875139
Sma-1-16179	NS_000200.1	-	247816943	247816895
Sma-1-16180	NW_003030133.1	-	962	914
Sma-1-16181	NW_003038081.1	-	108626	108578
Sma-1-16182	NS_000200.1	-	357657450	357657402
Sma-1-16183	NS_000200.1	-	269270669	269270621
Sma-1-16184	NW_003037831.1	-	1215409	1215361

Sma-1-16185	NS_000200.1	-	70664711	70664663
Sma-1-16186	NW_003037875.1	+	414845	414893
Sma-1-16187	NS_000200.1	+	120443514	120443562
Sma-1-16188	NW_003032263.1	+	5793	5841
Sma-1-16189	NW_003037795.1	-	683120	683072
Sma-1-16190	NW_003037809.1	-	2767100	2767052
Sma-1-16191	NW_003037819.1	+	496324	496372
Sma-1-16192	NW_003037866.1	+	649703	649751
Sma-1-16193	NW_003037885.1	-	731333	731285
Sma-1-16194	NW_003037901.1	-	677637	677589
Sma-1-16195	NW_003038042.1	+	67388	67436
Sma-1-16196	NS_000200.1	+	51584589	51584637
Sma-1-16197	NS_000200.1	+	112137502	112137550
Sma-1-16198	NS_000200.1	+	254012987	254013035
Sma-1-16199	NS_000200.1	+	358085143	358085191
Sma-1-16200	NS_000200.1	-	146526098	146526050
Sma-1-16201	NS_000200.1	-	128829280	128829232
Sma-1-16202	NS_000200.1	-	36431564	36431516
Sma-1-16203	NS_000200.1	-	7074003	7073955
Sma-1-16204	NW_003037958.1	-	133909	133861
Sma-1-16205	NS_000200.1	-	194798093	194798045
Sma-1-16206	NW_003031283.1	+	1095	1143
Sma-1-16207	NS_000200.1	+	345667183	345667231
Sma-1-16208	NW_003037859.1	+	140135	140183
Sma-1-16209	NW_003037859.1	-	139838	139790
Sma-1-16210	NS_000200.1	+	102818976	102819024
Sma-1-16211	NS_000200.1	-	102818679	102818631
Sma-1-16212	NW_003037938.1	-	566072	566024
Sma-1-16213	NS_000200.1	-	182603185	182603137
Sma-1-16214	NW_003037822.1	+	268609	268657
Sma-1-16215	NS_000200.1	+	54576615	54576663
Sma-1-16216	NW_003037853.1	+	683427	683475
Sma-1-16217	NS_000200.1	+	96175478	96175526
Sma-1-16218	NW_003021503.1	-	692	644
Sma-1-16219	NW_003035880.1	-	18935	18887
Sma-1-16220	NS_000200.1	-	344188827	344188779
Sma-1-16221	NS_000200.1	-	339259999	339259951
Sma-1-16222	NW_003031400.1	+	702	750
Sma-1-16223	NW_003038084.1	+	373007	373055
Sma-1-16224	NS_000200.1	+	271388251	271388299
Sma-1-16225	NS_000200.1	+	349903991	349904039
Sma-1-16226	NW_003031022.1	+	1350	1398
Sma-1-16227	NS_000200.1	+	325539197	325539245
Sma-1-16228	NW_003038257.1	-	191248	191200
Sma-1-16229	NS_000200.1	-	321365576	321365528
Sma-1-16230	NW_003037795.1	+	276262	276310
Sma-1-16231	NW_003037795.1	+	286014	286062
Sma-1-16232	NS_000200.1	+	6667145	6667193
Sma-1-16233	NS_000200.1	+	6676897	6676945
Sma-1-16234	NW_003037909.1	-	597950	597902
Sma-1-16235	NS_000200.1	-	154139599	154139551
Sma-1-16236	NW_003026618.1	+	367355	367403

Sma-1-16237	NS_000200.1	+	322122357	322122405
Sma-1-16238	NW_003037833.1	-	736607	736559
Sma-1-16239	NS_000200.1	-	72927703	72927655
Sma-1-16240	NW_003037924.1	+	26362	26410
Sma-1-16241	NW_003037945.1	-	882297	882249
Sma-1-16242	NW_003038020.1	+	226952	227000
Sma-1-16243	NS_000200.1	+	171733320	171733368
Sma-1-16244	NS_000200.1	+	234194296	234194344
Sma-1-16245	NS_000200.1	-	189984261	189984213
Sma-1-16246	NW_003037972.1	-	148422	148374
Sma-1-16247	NS_000200.1	-	202624126	202624078
Sma-1-16248	NW_003037826.1	+	137718	137766
Sma-1-16249	NS_000200.1	+	61137234	61137282
Sma-1-16250	NW_003038131.1	+	145677	145725
Sma-1-16251	NS_000200.1	+	282107521	282107569
Sma-1-16252	NW_003026606.1	-	149342	149294
Sma-1-16253	NW_003037911.1	-	451611	451563
Sma-1-16254	NW_003037971.1	+	530011	530059
Sma-1-16255	NW_003038024.1	+	729926	729974
Sma-1-16256	NS_000200.1	+	202152061	202152109
Sma-1-16257	NS_000200.1	+	238626955	238627003
Sma-1-16258	NS_000200.1	-	308005035	308004987
Sma-1-16259	NS_000200.1	-	156238964	156238916
Sma-1-16260	NW_003037901.1	-	855680	855632
Sma-1-16261	NS_000200.1	-	146704141	146704093
Sma-1-16262	NW_003037904.1	-	118228	118180
Sma-1-16263	NW_003038037.1	+	543157	543205
Sma-1-16264	NS_000200.1	+	250698566	250698614
Sma-1-16265	NS_000200.1	-	148832727	148832679
Sma-1-16266	NW_003037979.1	+	132689	132737
Sma-1-16267	NS_000200.1	+	210193709	210193757
Sma-1-16268	NW_003037905.1	+	49748	49796
Sma-1-16269	NS_000200.1	+	149318760	149318808
Sma-1-16270	NW_003037831.1	-	1067067	1067019
Sma-1-16271	NS_000200.1	-	70516369	70516321
Sma-1-16272	NW_003037980.1	-	493605	493557
Sma-1-16273	NS_000200.1	-	211454467	211454419
Sma-1-16274	NW_003038005.1	+	157697	157745
Sma-1-16275	NS_000200.1	+	224563651	224563699
Sma-1-16276	NW_003022852.1	-	939	891
Sma-1-16277	NW_003037934.1	-	230415	230367
Sma-1-16278	NW_003037800.1	-	2407465	2407417
Sma-1-16279	NW_003037914.1	-	330914	330866
Sma-1-16280	NS_000200.1	-	368212625	368212577
Sma-1-16281	NS_000200.1	-	179277162	179277114
Sma-1-16282	NS_000200.1	-	160475164	160475116
Sma-1-16283	NS_000200.1	-	21036825	21036777
Sma-1-16284	NW_003037831.1	+	416076	416124
Sma-1-16285	NS_000200.1	+	69865378	69865426
Sma-1-16286	NW_003037885.1	+	337939	337987
Sma-1-16287	NS_000200.1	+	128435886	128435934
Sma-1-16288	NW_003037896.1	-	127231	127183

Sma-1-16289	NS_000200.1	-	143047654	143047606
Sma-1-16290	NW_003026458.1	-	410335	410287
Sma-1-16291	NW_003037796.1	+	1064110	1064158
Sma-1-16292	NW_003038143.1	+	253533	253581
Sma-1-16293	NS_000200.1	+	9273754	9273802
Sma-1-16294	NS_000200.1	+	288013025	288013073
Sma-1-16295	NS_000200.1	-	102278565	102278517
Sma-1-16296	NW_003026283.1	+	360	408
Sma-1-16297	NS_000200.1	+	344667869	344667917
Sma-1-16298	NW_003037886.1	-	411838	411790
Sma-1-16299	NS_000200.1	-	129667405	129667357
Sma-1-16300	NW_003038261.1	-	458395	458347
Sma-1-16301	NW_003026490.1	+	69766	69814
Sma-1-16302	NS_000200.1	+	267701482	267701530
Sma-1-16303	NS_000200.1	-	324072046	324071998
Sma-1-16304	NW_003026609.1	-	152821	152773
Sma-1-16305	NW_003037807.1	-	322256	322208
Sma-1-16306	NW_003037814.1	-	281635	281587
Sma-1-16307	NW_003037816.1	+	562864	562912
Sma-1-16308	NW_003037838.1	-	277769	277721
Sma-1-16309	NW_003037839.1	+	804345	804393
Sma-1-16310	NW_003037964.1	-	181253	181205
Sma-1-16311	NW_003038037.1	+	537157	537205
Sma-1-16312	NW_003038037.1	-	536860	536812
Sma-1-16313	NW_003038069.1	+	57346	57394
Sma-1-16314	NW_003038224.1	+	41129	41177
Sma-1-16315	NW_003038065.1	+	193285	193333
Sma-1-16316	NS_000200.1	+	46771736	46771784
Sma-1-16317	NS_000200.1	+	79762495	79762543
Sma-1-16318	NS_000200.1	+	250692566	250692614
Sma-1-16319	NS_000200.1	+	262344902	262344950
Sma-1-16320	NS_000200.1	+	263900752	263900800
Sma-1-16321	NS_000200.1	+	309452857	309452905
Sma-1-16322	NS_000200.1	-	309807813	309807765
Sma-1-16323	NS_000200.1	-	250692269	250692221
Sma-1-16324	NS_000200.1	-	197782775	197782727
Sma-1-16325	NS_000200.1	-	78276332	78276284
Sma-1-16326	NS_000200.1	-	43265495	43265447
Sma-1-16327	NS_000200.1	-	30583772	30583724
Sma-1-16328	NW_003038152.1	+	109040	109088
Sma-1-16329	NS_000200.1	+	292714764	292714812
Sma-1-16330	NW_003037795.1	-	944082	944034
Sma-1-16331	NW_003038257.1	+	99479	99527
Sma-1-16332	NS_000200.1	+	321273807	321273855
Sma-1-16333	NS_000200.1	-	7334965	7334917
Sma-1-16334	NW_003025589.1	-	951	902
Sma-1-16335	NS_000200.1	-	356204162	356204113
Sma-1-16336	NW_003037858.1	-	365646	365598
Sma-1-16337	NW_003037886.1	+	535916	535964
Sma-1-16338	NS_000200.1	+	129791483	129791531
Sma-1-16339	NS_000200.1	-	101029922	101029874
Sma-1-16340	NW_003039022.1	-	1441	1393

Sma-1-16341	NW_003037978.1	+	488883	488931
Sma-1-16342	NS_000200.1	+	209756893	209756941
Sma-1-16343	NS_000200.1	-	358644946	358644898
Sma-1-16344	NW_003037856.1	-	354083	354035
Sma-1-16345	NS_000200.1	-	99712039	99711991
Sma-1-16346	NW_003037792.1	-	56822	56774
Sma-1-16347	NS_000200.1	-	382494166	382494118
Sma-1-16348	NW_003037853.1	+	143260	143308
Sma-1-16349	NW_003037910.1	+	28632	28680
Sma-1-16350	NS_000200.1	+	95635311	95635359
Sma-1-16351	NS_000200.1	+	154540607	154540655
Sma-1-16352	NW_003038018.1	+	655939	655987
Sma-1-16353	NS_000200.1	+	233056789	233056837
Sma-1-16354	NW_003037864.1	-	537624	537576
Sma-1-16355	NS_000200.1	-	109030198	109030150
Sma-1-16356	NW_003038259.1	+	32150	32198
Sma-1-16357	NS_000200.1	+	322892749	322892797
Sma-1-16358	NW_003038034.1	+	703190	703238
Sma-1-16359	NS_000200.1	+	247644946	247644994
Sma-1-16360	NW_003021503.1	-	361	313
Sma-1-16361	NW_003035880.1	-	18603	18555
Sma-1-16362	NW_003037834.1	+	723766	723814
Sma-1-16363	NS_000200.1	+	73884252	73884300
Sma-1-16364	NS_000200.1	-	344188496	344188448
Sma-1-16365	NS_000200.1	-	339259667	339259619
Sma-1-16366	NW_003038261.1	+	457913	457961
Sma-1-16367	NS_000200.1	+	324071564	324071612
Sma-1-16368	NW_003020214.1	+	651	699
Sma-1-16369	NW_003022681.1	-	1985	1937
Sma-1-16370	NW_003024359.1	-	1171	1123
Sma-1-16371	NW_003026497.1	+	3	51
Sma-1-16372	NW_003026497.1	+	346	394
Sma-1-16373	NW_003026497.1	+	689	737
Sma-1-16374	NW_003026497.1	+	1032	1080
Sma-1-16375	NW_003026557.1	+	63562	63610
Sma-1-16376	NW_003026571.1	+	18068	18116
Sma-1-16377	NW_003029352.1	-	1865	1817
Sma-1-16378	NW_003032152.1	+	24388	24436
Sma-1-16379	NW_003033784.1	-	2886	2838
Sma-1-16380	NW_003037033.1	-	1423	1375
Sma-1-16381	NW_003026617.1	+	341776	341824
Sma-1-16382	NW_003026617.1	-	341538	341490
Sma-1-16383	NW_003038073.1	-	9448	9400
Sma-1-16384	NW_003037873.1	+	147010	147058
Sma-1-16385	NW_003037876.1	-	204211	204163
Sma-1-16386	NW_003026601.1	-	12252	12204
Sma-1-16387	NW_003026602.1	+	43640	43688
Sma-1-16388	NW_003038222.1	-	113550	113502
Sma-1-16389	NW_003037867.1	-	131699	131651
Sma-1-16390	NW_003038225.1	+	170760	170808
Sma-1-16391	NW_003038226.1	-	32812	32764
Sma-1-16392	NW_003038228.1	+	228919	228967

Sma-1-16393	NW_003038005.1	+	234182	234230
Sma-1-16394	NW_003038005.1	+	433395	433443
Sma-1-16395	NW_003038013.1	-	201253	201205
Sma-1-16396	NW_003039068.1	+	1480	1528
Sma-1-16397	NW_003037708.1	-	8546	8498
Sma-1-16398	NW_003037929.1	+	137531	137579
Sma-1-16399	NW_003037929.1	-	9654	9606
Sma-1-16400	NW_003037934.1	-	142943	142895
Sma-1-16401	NW_003037937.1	+	114050	114098
Sma-1-16402	NW_003026458.1	-	491897	491849
Sma-1-16403	NW_003038070.1	+	49014	49062
Sma-1-16404	NW_003038415.1	+	8770	8818
Sma-1-16405	NW_003026456.1	-	271424	271376
Sma-1-16406	NW_003026464.1	-	161118	161070
Sma-1-16407	NW_003037793.1	+	1611648	1611696
Sma-1-16408	NW_003037793.1	-	1359641	1359593
Sma-1-16409	NW_003037794.1	+	696475	696523
Sma-1-16410	NW_003037796.1	-	1765513	1765465
Sma-1-16411	NW_003037797.1	-	2906565	2906517
Sma-1-16412	NW_003037797.1	-	1825343	1825295
Sma-1-16413	NW_003037799.1	-	1185289	1185241
Sma-1-16414	NW_003037800.1	+	465598	465646
Sma-1-16415	NW_003037800.1	-	1124644	1124596
Sma-1-16416	NW_003037806.1	+	292662	292710
Sma-1-16417	NW_003037809.1	+	1376083	1376131
Sma-1-16418	NW_003037809.1	+	2119500	2119548
Sma-1-16419	NW_003037809.1	+	2404329	2404377
Sma-1-16420	NW_003037809.1	-	1545276	1545228
Sma-1-16421	NW_003037810.1	+	697843	697891
Sma-1-16422	NW_003037815.1	-	409944	409896
Sma-1-16423	NW_003037818.1	-	575452	575404
Sma-1-16424	NW_003037821.1	+	86346	86394
Sma-1-16425	NW_003037821.1	+	589310	589358
Sma-1-16426	NW_003037821.1	+	1098262	1098310
Sma-1-16427	NW_003037824.1	+	676687	676735
Sma-1-16428	NW_003037824.1	-	564472	564424
Sma-1-16429	NW_003037825.1	-	1827847	1827799
Sma-1-16430	NW_003037825.1	-	1029401	1029353
Sma-1-16431	NW_003037827.1	+	52898	52946
Sma-1-16432	NW_003037827.1	+	1235084	1235132
Sma-1-16433	NW_003037827.1	+	1351947	1351995
Sma-1-16434	NW_003037827.1	-	1234787	1234739
Sma-1-16435	NW_003037829.1	-	1676244	1676196
Sma-1-16436	NW_003037829.1	-	342884	342836
Sma-1-16437	NW_003037831.1	+	640348	640396
Sma-1-16438	NW_003037835.1	+	979695	979743
Sma-1-16439	NW_003037835.1	-	875739	875691
Sma-1-16440	NW_003037836.1	+	1079407	1079455
Sma-1-16441	NW_003037836.1	-	910844	910796
Sma-1-16442	NW_003037837.1	+	652917	652965
Sma-1-16443	NW_003037842.1	+	94979	95027
Sma-1-16444	NW_003037842.1	+	240436	240484

Sma-1-16445	NW_003037843.1	+	48508	48556
Sma-1-16446	NW_003037844.1	+	382462	382510
Sma-1-16447	NW_003037846.1	-	656578	656530
Sma-1-16448	NW_003037848.1	+	1270506	1270554
Sma-1-16449	NW_003037848.1	-	1270224	1270176
Sma-1-16450	NW_003037851.1	+	315770	315818
Sma-1-16451	NW_003037852.1	+	879256	879304
Sma-1-16452	NW_003037853.1	-	1405508	1405460
Sma-1-16453	NW_003037853.1	-	682365	682317
Sma-1-16454	NW_003037855.1	+	19615	19663
Sma-1-16455	NW_003037863.1	+	434081	434129
Sma-1-16456	NW_003037864.1	+	774885	774933
Sma-1-16457	NW_003037865.1	-	75539	75491
Sma-1-16458	NW_003037880.1	+	510412	510460
Sma-1-16459	NW_003037883.1	+	55228	55276
Sma-1-16460	NW_003037883.1	+	155564	155612
Sma-1-16461	NW_003037884.1	+	776339	776387
Sma-1-16462	NW_003037885.1	+	433946	433994
Sma-1-16463	NW_003037885.1	+	1102962	1103010
Sma-1-16464	NW_003037885.1	-	1137407	1137359
Sma-1-16465	NW_003037885.1	-	69583	69535
Sma-1-16466	NW_003037891.1	-	2776175	2776127
Sma-1-16467	NW_003037892.1	+	23220	23268
Sma-1-16468	NW_003037892.1	-	23013	22965
Sma-1-16469	NW_003037894.1	+	156402	156450
Sma-1-16470	NW_003037894.1	+	176419	176467
Sma-1-16471	NW_003037897.1	+	217393	217441
Sma-1-16472	NW_003037899.1	-	379102	379054
Sma-1-16473	NW_003037900.1	+	208884	208932
Sma-1-16474	NW_003037904.1	+	500	548
Sma-1-16475	NW_003037909.1	-	784915	784867
Sma-1-16476	NW_003037911.1	+	541644	541692
Sma-1-16477	NW_003037912.1	+	720534	720582
Sma-1-16478	NW_003037912.1	-	534383	534335
Sma-1-16479	NW_003037913.1	-	344237	344189
Sma-1-16480	NW_003037916.1	+	542961	543009
Sma-1-16481	NW_003037916.1	+	751461	751509
Sma-1-16482	NW_003037917.1	+	361113	361161
Sma-1-16483	NW_003037917.1	-	771872	771824
Sma-1-16484	NW_003037917.1	-	47636	47588
Sma-1-16485	NW_003037924.1	-	32295	32247
Sma-1-16486	NW_003037928.1	+	176625	176673
Sma-1-16487	NW_003037928.1	+	497346	497394
Sma-1-16488	NW_003037930.1	-	156203	156155
Sma-1-16489	NW_003037935.1	-	72900	72852
Sma-1-16490	NW_003037939.1	+	871826	871874
Sma-1-16491	NW_003037939.1	-	857729	857681
Sma-1-16492	NW_003037940.1	-	580017	579969
Sma-1-16493	NW_003037945.1	-	1032070	1032022
Sma-1-16494	NW_003037964.1	+	138	186
Sma-1-16495	NW_003037966.1	+	477182	477230
Sma-1-16496	NW_003037972.1	+	515546	515594

Sma-1-16497	NW_003037975.1	-	678638	678590
Sma-1-16498	NW_003037977.1	-	200014	199966
Sma-1-16499	NW_003037979.1	+	260471	260519
Sma-1-16500	NW_003037979.1	-	132375	132327
Sma-1-16501	NW_003038003.1	+	38940	38988
Sma-1-16502	NW_003038011.1	+	736346	736394
Sma-1-16503	NW_003038012.1	+	236343	236391
Sma-1-16504	NW_003038012.1	-	553359	553311
Sma-1-16505	NW_003038015.1	-	543980	543932
Sma-1-16506	NW_003038015.1	-	155931	155883
Sma-1-16507	NW_003038016.1	-	388902	388854
Sma-1-16508	NW_003038018.1	+	538069	538117
Sma-1-16509	NW_003038018.1	+	608346	608394
Sma-1-16510	NW_003038018.1	-	537736	537688
Sma-1-16511	NW_003038021.1	+	94491	94539
Sma-1-16512	NW_003038021.1	-	94302	94254
Sma-1-16513	NW_003038028.1	-	634603	634555
Sma-1-16514	NW_003038033.1	+	260784	260832
Sma-1-16515	NW_003038034.1	-	812125	812077
Sma-1-16516	NW_003038034.1	-	680233	680185
Sma-1-16517	NW_003038072.1	+	5439	5487
Sma-1-16518	NW_003038210.1	+	98234	98282
Sma-1-16519	NW_003038239.1	-	142205	142157
Sma-1-16520	NW_003038243.1	-	173157	173109
Sma-1-16521	NW_003038254.1	+	311037	311085
Sma-1-16522	NW_003038261.1	+	407929	407977
Sma-1-16523	NW_003038482.1	-	9717	9669
Sma-1-16524	NW_003039127.1	+	2299865	2299913
Sma-1-16525	NW_003026478.1	+	39985	40033
Sma-1-16526	NW_003026490.1	-	358688	358640
Sma-1-16527	NW_003026512.1	-	261019	260971
Sma-1-16528	NW_003038041.1	-	130825	130777
Sma-1-16529	NW_003038042.1	+	200385	200433
Sma-1-16530	NW_003038045.1	-	7948	7900
Sma-1-16531	NW_003038049.1	-	119722	119674
Sma-1-16532	NW_003038049.1	-	34844	34796
Sma-1-16533	NW_003038050.1	+	126372	126420
Sma-1-16534	NW_003038050.1	-	165113	165065
Sma-1-16535	NW_003038053.1	+	577383	577431
Sma-1-16536	NW_003038055.1	+	221072	221120
Sma-1-16537	NW_003038059.1	+	75295	75343
Sma-1-16538	NW_003038064.1	-	30775	30727
Sma-1-16539	NW_003038076.1	-	501552	501504
Sma-1-16540	NW_003038085.1	+	482366	482414
Sma-1-16541	NW_003038124.1	+	136319	136367
Sma-1-16542	NW_003038130.1	-	179445	179397
Sma-1-16543	NW_003038141.1	-	170836	170788
Sma-1-16544	NW_003038145.1	-	134379	134331
Sma-1-16545	NW_003038149.1	-	328229	328181
Sma-1-16546	NW_003038150.1	+	277118	277166
Sma-1-16547	NS_000200.1	+	1611648	1611696
Sma-1-16548	NS_000200.1	+	4876070	4876118

Sma-1-16549	NS_000200.1	+	19094958	19095006
Sma-1-16550	NS_000200.1	+	28487876	28487924
Sma-1-16551	NS_000200.1	+	35040547	35040595
Sma-1-16552	NS_000200.1	+	35783964	35784012
Sma-1-16553	NS_000200.1	+	36068793	36068841
Sma-1-16554	NS_000200.1	+	37302365	37302413
Sma-1-16555	NS_000200.1	+	53230830	53230878
Sma-1-16556	NS_000200.1	+	53733794	53733842
Sma-1-16557	NS_000200.1	+	54242746	54242794
Sma-1-16558	NS_000200.1	+	57607627	57607675
Sma-1-16559	NS_000200.1	+	62675039	62675087
Sma-1-16560	NS_000200.1	+	63857225	63857273
Sma-1-16561	NS_000200.1	+	63974088	63974136
Sma-1-16562	NS_000200.1	+	70089650	70089698
Sma-1-16563	NS_000200.1	+	75376203	75376251
Sma-1-16564	NS_000200.1	+	76636506	76636554
Sma-1-16565	NS_000200.1	+	77458775	77458823
Sma-1-16566	NS_000200.1	+	85194169	85194217
Sma-1-16567	NS_000200.1	+	85339626	85339674
Sma-1-16568	NS_000200.1	+	85687815	85687863
Sma-1-16569	NS_000200.1	+	86565908	86565956
Sma-1-16570	NS_000200.1	+	90963577	90963625
Sma-1-16571	NS_000200.1	+	93719080	93719128
Sma-1-16572	NS_000200.1	+	95271151	95271199
Sma-1-16573	NS_000200.1	+	98884365	98884413
Sma-1-16574	NS_000200.1	+	107192448	107192496
Sma-1-16575	NS_000200.1	+	109267459	109267507
Sma-1-16576	NS_000200.1	+	115377364	115377412
Sma-1-16577	NS_000200.1	+	118886138	118886186
Sma-1-16578	NS_000200.1	+	124193970	124194018
Sma-1-16579	NS_000200.1	+	126118951	126118999
Sma-1-16580	NS_000200.1	+	126219287	126219335
Sma-1-16581	NS_000200.1	+	127556004	127556052
Sma-1-16582	NS_000200.1	+	128531893	128531941
Sma-1-16583	NS_000200.1	+	129200909	129200957
Sma-1-16584	NS_000200.1	+	140746447	140746495
Sma-1-16585	NS_000200.1	+	142052975	142053023
Sma-1-16586	NS_000200.1	+	142072992	142073040
Sma-1-16587	NS_000200.1	+	143699769	143699817
Sma-1-16588	NS_000200.1	+	145337750	145337798
Sma-1-16589	NS_000200.1	+	148714999	148715047
Sma-1-16590	NS_000200.1	+	156328997	156329045
Sma-1-16591	NS_000200.1	+	157532109	157532157
Sma-1-16592	NS_000200.1	+	163736372	163736420
Sma-1-16593	NS_000200.1	+	163944872	163944920
Sma-1-16594	NS_000200.1	+	165397917	165397965
Sma-1-16595	NS_000200.1	+	174500578	174500626
Sma-1-16596	NS_000200.1	+	174821299	174821347
Sma-1-16597	NS_000200.1	+	175378309	175378357
Sma-1-16598	NS_000200.1	+	181313959	181314007
Sma-1-16599	NS_000200.1	+	183741580	183741628
Sma-1-16600	NS_000200.1	+	197601660	197601708

Sma-1-16601	NS_000200.1	+	199179045	199179093
Sma-1-16602	NS_000200.1	+	202991250	202991298
Sma-1-16603	NS_000200.1	+	210321491	210321539
Sma-1-16604	NS_000200.1	+	223538222	223538270
Sma-1-16605	NS_000200.1	+	224640136	224640184
Sma-1-16606	NS_000200.1	+	224839349	224839397
Sma-1-16607	NS_000200.1	+	228263996	228264044
Sma-1-16608	NS_000200.1	+	228560282	228560330
Sma-1-16609	NS_000200.1	+	232938919	232938967
Sma-1-16610	NS_000200.1	+	233009196	233009244
Sma-1-16611	NS_000200.1	+	234923066	234923114
Sma-1-16612	NS_000200.1	+	246240786	246240834
Sma-1-16613	NS_000200.1	+	254145984	254146032
Sma-1-16614	NS_000200.1	+	254241534	254241582
Sma-1-16615	NS_000200.1	+	256401266	256401314
Sma-1-16616	NS_000200.1	+	257937478	257937526
Sma-1-16617	NS_000200.1	+	258920394	258920442
Sma-1-16618	NS_000200.1	+	260317367	260317415
Sma-1-16619	NS_000200.1	+	264386255	264386303
Sma-1-16620	NS_000200.1	+	265167667	265167715
Sma-1-16621	NS_000200.1	+	272172459	272172507
Sma-1-16622	NS_000200.1	+	274660195	274660243
Sma-1-16623	NS_000200.1	+	274660538	274660586
Sma-1-16624	NS_000200.1	+	274660881	274660929
Sma-1-16625	NS_000200.1	+	274661224	274661272
Sma-1-16626	NS_000200.1	+	279128963	279129011
Sma-1-16627	NS_000200.1	+	291755856	291755904
Sma-1-16628	NS_000200.1	+	301051605	301051653
Sma-1-16629	NS_000200.1	+	301720083	301720131
Sma-1-16630	NS_000200.1	+	305560133	305560181
Sma-1-16631	NS_000200.1	+	306963623	306963671
Sma-1-16632	NS_000200.1	+	310025424	310025472
Sma-1-16633	NS_000200.1	+	311102199	311102247
Sma-1-16634	NS_000200.1	+	319912635	319912683
Sma-1-16635	NS_000200.1	+	320346595	320346643
Sma-1-16636	NS_000200.1	+	324021580	324021628
Sma-1-16637	NS_000200.1	+	328775394	328775442
Sma-1-16638	NS_000200.1	+	359826500	359826548
Sma-1-16639	NS_000200.1	+	371213780	371213828
Sma-1-16640	NS_000200.1	+	379254909	379254957
Sma-1-16641	NS_000200.1	-	375728468	375728420
Sma-1-16642	NS_000200.1	-	365583550	365583502
Sma-1-16643	NS_000200.1	-	365414729	365414681
Sma-1-16644	NS_000200.1	-	358967208	358967160
Sma-1-16645	NS_000200.1	-	357357826	357357778
Sma-1-16646	NS_000200.1	-	343703583	343703535
Sma-1-16647	NS_000200.1	-	332987168	332987120
Sma-1-16648	NS_000200.1	-	319912397	319912349
Sma-1-16649	NS_000200.1	-	315111641	315111593
Sma-1-16650	NS_000200.1	-	313982987	313982939
Sma-1-16651	NS_000200.1	-	310063846	310063798
Sma-1-16652	NS_000200.1	-	308901214	308901166

Sma-1-16653	NS_000200.1	-	306393861	306393813
Sma-1-16654	NS_000200.1	-	291356842	291356794
Sma-1-16655	NS_000200.1	-	288745802	288745754
Sma-1-16656	NS_000200.1	-	287060815	287060767
Sma-1-16657	NS_000200.1	-	285355059	285355011
Sma-1-16658	NS_000200.1	-	281734431	281734383
Sma-1-16659	NS_000200.1	-	267990404	267990356
Sma-1-16660	NS_000200.1	-	266718726	266718678
Sma-1-16661	NS_000200.1	-	265531881	265531833
Sma-1-16662	NS_000200.1	-	261867352	261867304
Sma-1-16663	NS_000200.1	-	256440007	256439959
Sma-1-16664	NS_000200.1	-	256095249	256095201
Sma-1-16665	NS_000200.1	-	256010371	256010323
Sma-1-16666	NS_000200.1	-	254785559	254785511
Sma-1-16667	NS_000200.1	-	253798107	253798059
Sma-1-16668	NS_000200.1	-	247753881	247753833
Sma-1-16669	NS_000200.1	-	247621989	247621941
Sma-1-16670	NS_000200.1	-	241942177	241942129
Sma-1-16671	NS_000200.1	-	234922877	234922829
Sma-1-16672	NS_000200.1	-	232938586	232938538
Sma-1-16673	NS_000200.1	-	231505439	231505391
Sma-1-16674	NS_000200.1	-	230867470	230867422
Sma-1-16675	NS_000200.1	-	230479421	230479373
Sma-1-16676	NS_000200.1	-	229590879	229590831
Sma-1-16677	NS_000200.1	-	228877298	228877250
Sma-1-16678	NS_000200.1	-	210193395	210193347
Sma-1-16679	NS_000200.1	-	208486034	208485986
Sma-1-16680	NS_000200.1	-	206260862	206260814
Sma-1-16681	NS_000200.1	-	203461427	203461379
Sma-1-16682	NS_000200.1	-	190134034	190133986
Sma-1-16683	NS_000200.1	-	184322147	184322099
Sma-1-16684	NS_000200.1	-	183727483	183727435
Sma-1-16685	NS_000200.1	-	179489435	179489387
Sma-1-16686	NS_000200.1	-	179189690	179189642
Sma-1-16687	NS_000200.1	-	176014167	176014119
Sma-1-16688	NS_000200.1	-	175250432	175250384
Sma-1-16689	NS_000200.1	-	171739253	171739205
Sma-1-16690	NS_000200.1	-	165808676	165808628
Sma-1-16691	NS_000200.1	-	165084440	165084392
Sma-1-16692	NS_000200.1	-	158907988	158907940
Sma-1-16693	NS_000200.1	-	157345958	157345910
Sma-1-16694	NS_000200.1	-	154326564	154326516
Sma-1-16695	NS_000200.1	-	144898213	144898165
Sma-1-16696	NS_000200.1	-	140746240	140746192
Sma-1-16697	NS_000200.1	-	140657453	140657405
Sma-1-16698	NS_000200.1	-	129235354	129235306
Sma-1-16699	NS_000200.1	-	128167530	128167482
Sma-1-16700	NS_000200.1	-	120749847	120749799
Sma-1-16701	NS_000200.1	-	115732518	115732470
Sma-1-16702	NS_000200.1	-	110497458	110497410
Sma-1-16703	NS_000200.1	-	102360127	102360079
Sma-1-16704	NS_000200.1	-	96897559	96897511

Sma-1-16705	NS_000200.1	-	96174416	96174368
Sma-1-16706	NS_000200.1	-	90963295	90963247
Sma-1-16707	NS_000200.1	-	88291148	88291100
Sma-1-16708	NS_000200.1	-	76467943	76467895
Sma-1-16709	NS_000200.1	-	75272247	75272199
Sma-1-16710	NS_000200.1	-	67576782	67576734
Sma-1-16711	NS_000200.1	-	66243422	66243374
Sma-1-16712	NS_000200.1	-	63856928	63856880
Sma-1-16713	NS_000200.1	-	60957629	60957581
Sma-1-16714	NS_000200.1	-	60159183	60159135
Sma-1-16715	NS_000200.1	-	58194560	58194512
Sma-1-16716	NS_000200.1	-	57495412	57495364
Sma-1-16717	NS_000200.1	-	49290859	49290811
Sma-1-16718	NS_000200.1	-	45672935	45672887
Sma-1-16719	NS_000200.1	-	35209740	35209692
Sma-1-16720	NS_000200.1	-	19754004	19753956
Sma-1-16721	NS_000200.1	-	18175643	18175595
Sma-1-16722	NS_000200.1	-	13334425	13334377
Sma-1-16723	NS_000200.1	-	12253203	12253155
Sma-1-16724	NS_000200.1	-	9975157	9975109
Sma-1-16725	NS_000200.1	-	1359641	1359593
Sma-1-16726	NW_003038009.1	-	324106	324058
Sma-1-16727	NS_000200.1	-	226698169	226698121
Sma-1-16728	NW_003026594.1	+	63283	63331
Sma-1-16729	NW_003037819.1	-	180365	180317
Sma-1-16730	NS_000200.1	+	305007653	305007701
Sma-1-16731	NS_000200.1	-	51268630	51268582
Sma-1-16732	NW_003037838.1	-	659927	659879
Sma-1-16733	NW_003037871.1	+	409476	409524
Sma-1-16734	NS_000200.1	+	117857214	117857262
Sma-1-16735	NS_000200.1	-	78658490	78658442
Sma-1-16736	NW_003037839.1	-	1308240	1308192
Sma-1-16737	NS_000200.1	-	80266390	80266342
Sma-1-16738	NW_003037840.1	-	1229292	1229244
Sma-1-16739	NS_000200.1	-	81852616	81852568
Sma-1-16740	NW_003038249.1	+	80966	81014
Sma-1-16741	NS_000200.1	+	316763219	316763267
Sma-1-16742	NW_003027588.1	+	287	335
Sma-1-16743	NW_003037841.1	-	188116	188068
Sma-1-16744	NW_003037850.1	-	297755	297707
Sma-1-16745	NW_003037942.1	+	623273	623321
Sma-1-16746	NW_003038011.1	-	720320	720272
Sma-1-16747	NS_000200.1	+	186383181	186383229
Sma-1-16748	NS_000200.1	+	361682883	361682931
Sma-1-16749	NS_000200.1	-	228247970	228247922
Sma-1-16750	NS_000200.1	-	92283785	92283737
Sma-1-16751	NS_000200.1	-	82799297	82799249
Sma-1-16752	NW_003037851.1	+	959702	959750
Sma-1-16753	NW_003037853.1	-	1025127	1025079
Sma-1-16754	NS_000200.1	+	94363012	94363060
Sma-1-16755	NS_000200.1	-	96517178	96517130
Sma-1-16756	NW_003038028.1	-	343097	343049

Sma-1-16757	NS_000200.1	-	241650671	241650623
Sma-1-16758	NW_003026458.1	-	243732	243684
Sma-1-16759	NW_003037804.1	+	872575	872623
Sma-1-16760	NW_003037835.1	+	1064525	1064573
Sma-1-16761	NW_003037848.1	+	338975	339023
Sma-1-16762	NW_003037848.1	-	338591	338543
Sma-1-16763	NW_003037914.1	+	1665820	1665868
Sma-1-16764	NW_003037915.1	+	438376	438424
Sma-1-16765	NW_003037915.1	-	981507	981459
Sma-1-16766	NW_003037932.1	-	497648	497600
Sma-1-16767	NW_003037945.1	+	1028129	1028177
Sma-1-16768	NW_003037967.1	-	581152	581104
Sma-1-16769	NW_003038039.1	-	78103	78055
Sma-1-16770	NS_000200.1	+	26012595	26012643
Sma-1-16771	NS_000200.1	+	75461033	75461081
Sma-1-16772	NS_000200.1	+	90032046	90032094
Sma-1-16773	NS_000200.1	+	161810070	161810118
Sma-1-16774	NS_000200.1	+	162338362	162338410
Sma-1-16775	NS_000200.1	+	190130093	190130141
Sma-1-16776	NS_000200.1	-	253515949	253515901
Sma-1-16777	NS_000200.1	-	199821123	199821075
Sma-1-16778	NS_000200.1	-	178030844	178030796
Sma-1-16779	NS_000200.1	-	162881493	162881445
Sma-1-16780	NS_000200.1	-	102111962	102111914
Sma-1-16781	NS_000200.1	-	90031662	90031614
Sma-1-16782	NW_003037940.1	+	742747	742795
Sma-1-16783	NS_000200.1	+	184484877	184484925
Sma-1-16784	NW_003037800.1	+	552223	552271
Sma-1-16785	NS_000200.1	+	19181583	19181631
Sma-1-16786	NW_003037922.1	-	390136	390088
Sma-1-16787	NS_000200.1	-	170314133	170314085
Sma-1-16788	NW_003037802.1	+	868052	868100
Sma-1-16789	NS_000200.1	+	22870846	22870894
Sma-1-16790	NW_003037804.1	+	1203600	1203648
Sma-1-16791	NS_000200.1	+	26343620	26343668
Sma-1-16792	NW_003037804.1	+	800334	800382
Sma-1-16793	NS_000200.1	+	25940354	25940402
Sma-1-16794	NW_003037930.1	+	886488	886536
Sma-1-16795	NS_000200.1	+	176744452	176744500
Sma-1-16796	NW_003035682.1	+	47105	47153
Sma-1-16797	NW_003038228.1	+	206254	206302
Sma-1-16798	NS_000200.1	+	311079534	311079582
Sma-1-16799	NS_000200.1	+	332669158	332669206
Sma-1-16800	NW_003028654.1	+	95	143
Sma-1-16801	NS_000200.1	+	374820031	374820079
Sma-1-16802	NW_003037886.1	-	48529	48481
Sma-1-16803	NW_003037886.1	-	13279	13231
Sma-1-16804	NS_000200.1	-	129304096	129304048
Sma-1-16805	NS_000200.1	-	129268846	129268798
Sma-1-16806	NW_003022970.1	+	83	131
Sma-1-16807	NW_003026459.1	-	233031	232983
Sma-1-16808	NW_003037795.1	-	1263070	1263022

Sma-1-16809	NW_003038247.1	-	359410	359362
Sma-1-16810	NW_003038187.1	-	5260	5212
Sma-1-16811	NS_000200.1	+	371971437	371971485
Sma-1-16812	NS_000200.1	-	316308138	316308090
Sma-1-16813	NS_000200.1	-	301957118	301957070
Sma-1-16814	NS_000200.1	-	141878304	141878256
Sma-1-16815	NS_000200.1	-	7653953	7653905
Sma-1-16816	NW_003037795.1	+	1185702	1185750
Sma-1-16817	NS_000200.1	+	7576585	7576633
Sma-1-16818	NW_003037939.1	+	332739	332787
Sma-1-16819	NS_000200.1	+	183202493	183202541
Sma-1-16820	NW_003037928.1	-	6301	6253
Sma-1-16821	NW_003038041.1	-	131171	131123
Sma-1-16822	NS_000200.1	-	253798453	253798405
Sma-1-16823	NS_000200.1	-	174330254	174330206
Sma-1-16824	NW_003037944.1	-	805071	805023
Sma-1-16825	NW_003038152.1	+	389369	389417
Sma-1-16826	NS_000200.1	+	292995093	292995141
Sma-1-16827	NS_000200.1	-	189026424	189026376
Sma-1-16828	NW_003037918.1	+	601843	601891
Sma-1-16829	NS_000200.1	+	167168833	167168881
Sma-1-16830	NW_003037903.1	+	732640	732688
Sma-1-16831	NS_000200.1	+	148286721	148286769
Sma-1-16832	NW_003037837.1	-	960574	960526
Sma-1-16833	NS_000200.1	-	77766432	77766384
Sma-1-16834	NW_003037844.1	-	772471	772423
Sma-1-16835	NS_000200.1	-	86955917	86955869
Sma-1-16836	NW_003038022.1	+	518389	518437
Sma-1-16837	NS_000200.1	+	236199231	236199279
Sma-1-16838	NW_003039127.1	+	1126139	1126187
Sma-1-16839	NS_000200.1	+	114203638	114203686
Sma-1-16840	NW_003039090.1	-	3716	3668
Sma-1-16841	NS_000200.1	-	360275396	360275348
Sma-1-16842	NW_003023592.1	-	1126	1078
Sma-1-16843	NS_000200.1	-	373766057	373766009
Sma-1-16844	NW_003020795.1	+	1291	1339
Sma-1-16845	NW_003022599.1	+	960	1008
Sma-1-16846	NW_003037937.1	+	61124	61172
Sma-1-16847	NW_003037937.1	-	60827	60779
Sma-1-16848	NW_003037807.1	-	492756	492708
Sma-1-16849	NW_003037822.1	-	428914	428866
Sma-1-16850	NW_003037903.1	+	419668	419716
Sma-1-16851	NW_003037911.1	+	544388	544436
Sma-1-16852	NS_000200.1	+	147973749	147973797
Sma-1-16853	NS_000200.1	+	156331741	156331789
Sma-1-16854	NS_000200.1	+	181261033	181261081
Sma-1-16855	NS_000200.1	+	364474639	364474687
Sma-1-16856	NS_000200.1	+	365255347	365255395
Sma-1-16857	NS_000200.1	-	181260736	181260688
Sma-1-16858	NS_000200.1	-	54736920	54736872
Sma-1-16859	NS_000200.1	-	30754272	30754224
Sma-1-16860	NW_003026459.1	-	215723	215675

Sma-1-16861	NS_000200.1	-	141860996	141860948
Sma-1-16862	NW_003026480.1	-	63311	63263
Sma-1-16863	NW_003026507.1	+	142213	142261
Sma-1-16864	NS_000200.1	+	279802364	279802412
Sma-1-16865	NS_000200.1	-	254948868	254948820
Sma-1-16866	NW_003038013.1	-	329096	329048
Sma-1-16867	NS_000200.1	-	229718722	229718674
Sma-1-16868	NW_003038412.1	-	14728	14680
Sma-1-16869	NW_003037871.1	-	396623	396575
Sma-1-16870	NS_000200.1	-	328582820	328582772
Sma-1-16871	NS_000200.1	-	117844361	117844313
Sma-1-16872	NW_003038080.1	+	148697	148745
Sma-1-16873	NS_000200.1	+	268601307	268601355
Sma-1-16874	NW_003038023.1	+	297805	297853
Sma-1-16875	NW_003038148.1	-	122062	122014
Sma-1-16876	NS_000200.1	+	237458096	237458144
Sma-1-16877	NS_000200.1	-	290196046	290195998
Sma-1-16878	NW_003037932.1	+	129965	130013
Sma-1-16879	NS_000200.1	+	177663161	177663209
Sma-1-16880	NW_003037798.1	-	688223	688175
Sma-1-16881	NS_000200.1	-	14947381	14947333
Sma-1-16882	NW_003037797.1	-	2409441	2409393
Sma-1-16883	NS_000200.1	-	12837301	12837253
Sma-1-16884	NW_003037807.1	+	1423270	1423318
Sma-1-16885	NW_003037807.1	-	1423000	1422952
Sma-1-16886	NS_000200.1	+	31684786	31684834
Sma-1-16887	NS_000200.1	-	31684516	31684468
Sma-1-16888	NW_003038010.1	+	182865	182913
Sma-1-16889	NS_000200.1	+	227001088	227001136
Sma-1-16890	NW_003037888.1	+	635265	635313
Sma-1-16891	NS_000200.1	+	132843757	132843805
Sma-1-16892	NW_003037818.1	+	1083141	1083189
Sma-1-16893	NS_000200.1	+	49798548	49798596
Sma-1-16894	NW_003037793.1	+	2729198	2729246
Sma-1-16895	NW_003037857.1	+	245861	245909
Sma-1-16896	NW_003038193.1	+	15389	15437
Sma-1-16897	NS_000200.1	+	2729198	2729246
Sma-1-16898	NS_000200.1	+	100266218	100266266
Sma-1-16899	NS_000200.1	+	302895269	302895317
Sma-1-16900	NW_003037819.1	-	546267	546219
Sma-1-16901	NS_000200.1	-	51634532	51634484
Sma-1-16902	NW_003037814.1	+	809562	809610
Sma-1-16903	NW_003037872.1	+	605995	606043
Sma-1-16904	NW_003037974.1	+	428975	429023
Sma-1-16905	NS_000200.1	+	43793422	43793470
Sma-1-16906	NS_000200.1	+	118563001	118563049
Sma-1-16907	NS_000200.1	+	205104281	205104329
Sma-1-16908	NW_003037884.1	-	1067607	1067559
Sma-1-16909	NS_000200.1	-	127847272	127847224
Sma-1-16910	NW_003038037.1	-	355770	355722
Sma-1-16911	NS_000200.1	-	250511179	250511131
Sma-1-16912	NW_003037943.1	+	1103067	1103115

Sma-1-16913	NS_000200.1	+	188124600	188124648
Sma-1-16914	NW_003032029.1	-	1148	1100
Sma-1-16915	NS_000200.1	-	376890170	376890122
Sma-1-16916	NW_003037797.1	+	2891719	2891767
Sma-1-16917	NS_000200.1	+	13319579	13319627
Sma-1-16918	NW_003038017.1	-	257924	257876
Sma-1-16919	NS_000200.1	-	232023955	232023907
Sma-1-16920	NW_003038014.1	+	242739	242787
Sma-1-16921	NS_000200.1	+	229983428	229983476
Sma-1-16922	NW_003037796.1	+	473978	474026
Sma-1-16923	NS_000200.1	+	8683622	8683670
Sma-1-16924	NW_003038192.1	+	20253	20301
Sma-1-16925	NS_000200.1	+	302582160	302582208
Sma-1-16926	NW_003039127.1	+	1551626	1551674
Sma-1-16927	NS_000200.1	+	114629125	114629173
Sma-1-16928	NW_003038163.1	-	503439	503391
Sma-1-16929	NS_000200.1	-	298903719	298903671
Sma-1-16930	NW_003037810.1	-	364021	363973
Sma-1-16931	NS_000200.1	-	36968543	36968495
Sma-1-16932	NW_003037915.1	+	1226978	1227026
Sma-1-16933	NS_000200.1	+	163126964	163127012
Sma-1-16934	NW_003037940.1	+	312213	312261
Sma-1-16935	NS_000200.1	+	184054343	184054391
Sma-1-16936	NW_003037878.1	+	249457	249505
Sma-1-16937	NS_000200.1	+	122302935	122302983
Sma-1-16938	NW_003038037.1	+	608474	608522
Sma-1-16939	NS_000200.1	+	250763883	250763931
Sma-1-16940	NW_003038257.1	+	379133	379181
Sma-1-16941	NS_000200.1	+	321553461	321553509
Sma-1-16942	NW_003026474.1	+	66750	66798
Sma-1-16943	NS_000200.1	+	253265325	253265373
Sma-1-16944	NW_003037890.1	+	752075	752123
Sma-1-16945	NS_000200.1	+	136435436	136435484
Sma-1-16946	NW_003026622.1	+	2568	2616
Sma-1-16947	NS_000200.1	+	324856935	324856983
Sma-1-16948	NW_003037910.1	-	161262	161214
Sma-1-16949	NS_000200.1	-	154673237	154673189
Sma-1-16950	NW_003037804.1	+	1176163	1176211
Sma-1-16951	NS_000200.1	+	26316183	26316231
Sma-1-16952	NW_003038192.1	+	10970	11018
Sma-1-16953	NS_000200.1	+	302572877	302572925
Sma-1-16954	NW_003026609.1	+	12817	12865
Sma-1-16955	NS_000200.1	+	309667809	309667857
Sma-1-16956	NW_003037825.1	+	789324	789372
Sma-1-16957	NS_000200.1	+	59919106	59919154
Sma-1-16958	NW_003038005.1	-	178683	178635
Sma-1-16959	NS_000200.1	-	224584637	224584589
Sma-1-16960	NW_003037852.1	-	475771	475723
Sma-1-16961	NS_000200.1	-	94867666	94867618
Sma-1-16962	NW_003037797.1	+	3237072	3237120
Sma-1-16963	NS_000200.1	+	13664932	13664980
Sma-1-16964	NW_003037886.1	+	1108772	1108820

Sma-1-16965	NS_000200.1	+	130364339	130364387
Sma-1-16966	NW_003038149.1	+	17689	17737
Sma-1-16967	NS_000200.1	+	291046302	291046350
Sma-1-16968	NW_003026472.1	+	417344	417392
Sma-1-16969	NS_000200.1	+	236829336	236829384
Sma-1-16970	NW_003038001.1	+	375147	375195
Sma-1-16971	NS_000200.1	+	223084708	223084756
Sma-1-16972	NW_003026619.1	-	187054	187006
Sma-1-16973	NS_000200.1	-	322377798	322377750
Sma-1-16974	NW_003037909.1	+	494799	494847
Sma-1-16975	NS_000200.1	+	154036448	154036496
Sma-1-16976	NW_003037817.1	+	596963	597011
Sma-1-16977	NS_000200.1	+	47979315	47979363
Sma-1-16978	NW_003037814.1	+	1021684	1021733
Sma-1-16979	NS_000200.1	+	44005544	44005593
Sma-1-16980	NW_003037813.1	+	1634075	1634123
Sma-1-16981	NS_000200.1	+	42620938	42620986
Sma-1-16982	NW_003037859.1	-	903327	903279
Sma-1-16983	NS_000200.1	-	103582168	103582120
Sma-1-16984	NW_003037886.1	+	944718	944766
Sma-1-16985	NS_000200.1	+	130200285	130200333
Sma-1-16986	NW_003038514.1	+	189173	189221
Sma-1-16987	NS_000200.1	+	339813712	339813760
Sma-1-16988	NW_003026458.1	+	337259	337307
Sma-1-16989	NS_000200.1	+	102205489	102205537
Sma-1-16990	NW_003038118.1	-	276797	276749
Sma-1-16991	NS_000200.1	-	277840924	277840876
Sma-1-16992	NW_003037830.1	-	763599	763551
Sma-1-16993	NS_000200.1	-	68653970	68653922
Sma-1-16994	NW_003037854.1	-	241368	241320
Sma-1-16995	NS_000200.1	-	97145789	97145741
Sma-1-16996	NW_003037830.1	-	1370438	1370360
Sma-1-16997	NS_000200.1	-	69260809	69260731
Sma-1-16998	NW_003038147.1	+	398705	398753
Sma-1-16999	NS_000200.1	+	289926448	289926496
Sma-1-17000	NW_003037898.1	-	120154	120106
Sma-1-17001	NS_000200.1	-	144103976	144103928
Sma-1-17002	NW_003037830.1	-	166308	166260
Sma-1-17003	NS_000200.1	-	68056679	68056631
Sma-1-17004	NW_003037915.1	+	282877	282925
Sma-1-17005	NS_000200.1	+	162182863	162182911
Sma-1-17006	NW_003037890.1	-	1103007	1102959
Sma-1-17007	NS_000200.1	-	136786368	136786320
Sma-1-17008	NW_003026517.1	-	544499	544451
Sma-1-17009	NS_000200.1	-	300150508	300150460
Sma-1-17010	NW_003037808.1	+	504301	504349
Sma-1-17011	NS_000200.1	+	32290938	32290986
Sma-1-17012	NW_003037815.1	+	681084	681132
Sma-1-17013	NS_000200.1	+	45944075	45944123
Sma-1-17014	NW_003037846.1	-	657103	657055
Sma-1-17015	NS_000200.1	-	88291673	88291625
Sma-1-17016	NW_003038007.1	+	153703	153751

Sma-1-17017	NS_000200.1	+	225402258	225402306
Sma-1-17018	NW_003037977.1	+	809459	809507
Sma-1-17019	NS_000200.1	+	209095479	209095527
Sma-1-17020	NW_003031712.1	+	346	394
Sma-1-17021	NS_000200.1	+	353705996	353706044
Sma-1-17022	NW_003038112.1	+	41374	41422
Sma-1-17023	NS_000200.1	+	276496552	276496600
Sma-1-17024	NW_003037907.1	-	120981	120933
Sma-1-17025	NS_000200.1	-	151525470	151525422
Sma-1-17026	NW_003037865.1	-	847990	847942
Sma-1-17027	NS_000200.1	-	111269909	111269861
Sma-1-17028	NW_003037917.1	-	944729	944681
Sma-1-17029	NS_000200.1	-	165981533	165981485
Sma-1-17030	NW_003037886.1	-	747368	747320
Sma-1-17031	NS_000200.1	-	130002935	130002887
Sma-1-17032	NW_003038145.1	-	100780	100732
Sma-1-17033	NS_000200.1	-	288712203	288712155
Sma-1-17034	NW_003037173.1	-	7443	7395
Sma-1-17035	NS_000200.1	-	362521471	362521423
Sma-1-17036	NW_003037890.1	-	1892233	1892184
Sma-1-17037	NS_000200.1	-	137575594	137575545
Sma-1-17038	NW_003037938.1	+	202138	202189
Sma-1-17039	NS_000200.1	+	182239251	182239302
Sma-1-17040	NW_003037803.1	+	450658	450706
Sma-1-17041	NS_000200.1	+	23951195	23951243
Sma-1-17042	NW_003038034.1	-	828426	828378
Sma-1-17043	NS_000200.1	-	247770182	247770134
Sma-1-17044	NW_003037970.1	+	215394	215441
Sma-1-17045	NS_000200.1	+	201271584	201271631
Sma-1-17046	NW_003037915.1	-	76285	76238
Sma-1-17047	NS_000200.1	-	161976271	161976224
Sma-1-17048	NW_003038001.1	+	302159	302206
Sma-1-17049	NS_000200.1	+	223011720	223011767
Sma-1-17050	NW_003037925.1	+	159168	159215
Sma-1-17051	NS_000200.1	+	172561171	172561218
Sma-1-17052	NW_003037793.1	+	2757203	2757251
Sma-1-17053	NS_000200.1	+	2757203	2757251
Sma-1-17054	NW_003037902.1	+	226629	226676
Sma-1-17055	NS_000200.1	+	146981863	146981910
Sma-1-17056	NW_003038036.1	+	409024	409072
Sma-1-17057	NS_000200.1	+	249342876	249342924
Sma-1-17058	NW_003038260.1	-	29398	29351
Sma-1-17059	NS_000200.1	-	323201173	323201126
Sma-1-17060	NW_003037889.1	+	1831489	1831536
Sma-1-17061	NS_000200.1	+	135607979	135608026
Sma-1-17062	NW_003037910.1	+	1036647	1036695
Sma-1-17063	NS_000200.1	+	155548622	155548670
Sma-1-17064	NW_003024296.1	+	232	280
Sma-1-17065	NS_000200.1	+	365462860	365462908
Sma-1-17066	NW_003037822.1	-	829159	829111
Sma-1-17067	NS_000200.1	-	55137165	55137117
Sma-1-17068	NW_003037906.1	+	938155	938203

Sma-1-17069	NS_000200.1	+	151245934	151245982
Sma-1-17070	NW_003038241.1	+	49775	49823
Sma-1-17071	NS_000200.1	+	314512480	314512528
Sma-1-17072	NW_003037804.1	+	852455	852503
Sma-1-17073	NS_000200.1	+	25992475	25992523
Sma-1-17074	NW_003038022.1	-	694312	694264
Sma-1-17075	NS_000200.1	-	236375154	236375106
Sma-1-17076	NW_003038160.1	+	243284	243332
Sma-1-17077	NS_000200.1	+	296995215	296995263
Sma-1-17078	NW_003021473.1	-	193	145
Sma-1-17079	NW_003037877.1	+	165457	165505
Sma-1-17080	NS_000200.1	+	121204019	121204067
Sma-1-17081	NS_000200.1	-	344137104	344137056
Sma-1-17082	NW_003037796.1	+	1095721	1095769
Sma-1-17083	NS_000200.1	+	9305365	9305413
Sma-1-17084	NW_003038038.1	+	1168124	1168172
Sma-1-17085	NS_000200.1	+	253071517	253071565
Sma-1-17086	NW_003037941.1	+	91629	91677
Sma-1-17087	NS_000200.1	+	184826103	184826151
Sma-1-17088	NW_003037831.1	-	1113593	1113545
Sma-1-17089	NS_000200.1	-	70562895	70562847
Sma-1-17090	NW_003037893.1	+	336637	336685
Sma-1-17091	NS_000200.1	+	141450947	141450995
Sma-1-17092	NW_003026468.1	-	145074	145026
Sma-1-17093	NS_000200.1	-	216339095	216339047
Sma-1-17094	NW_003038038.1	+	377443	377491
Sma-1-17095	NS_000200.1	+	252280836	252280884
Sma-1-17096	NW_003037901.1	-	613513	613465
Sma-1-17097	NS_000200.1	-	146461974	146461926
Sma-1-17098	NW_003037891.1	-	2053012	2052964
Sma-1-17099	NS_000200.1	-	139934290	139934242
Sma-1-17100	NW_003038137.1	+	243638	243686
Sma-1-17101	NS_000200.1	+	285032534	285032582
Sma-1-17102	NW_003037884.1	+	226399	226447
Sma-1-17103	NS_000200.1	+	127006064	127006112
Sma-1-17104	NW_003038250.1	+	194771	194822
Sma-1-17105	NS_000200.1	+	318247448	318247499
Sma-1-17106	NW_003037862.1	-	824641	824593
Sma-1-17107	NS_000200.1	-	106476302	106476254
Sma-1-17108	NW_003037871.1	-	117266	117218
Sma-1-17109	NS_000200.1	-	117565004	117564956
Sma-1-17110	NW_003037918.1	-	2204168	2204120
Sma-1-17111	NS_000200.1	-	168771158	168771110
Sma-1-17112	NW_003037859.1	-	894947	894899
Sma-1-17113	NS_000200.1	-	103573788	103573740
Sma-1-17114	NW_003037940.1	+	806741	806789
Sma-1-17115	NS_000200.1	+	184548871	184548919
Sma-1-17116	NW_003037836.1	-	1163002	1162953
Sma-1-17117	NS_000200.1	-	76720101	76720052
Sma-1-17118	NW_003037879.1	+	568846	568894
Sma-1-17119	NS_000200.1	+	123571102	123571150
Sma-1-17120	NW_003037819.1	+	909600	909648

Sma-1-17121	NS_000200.1	+	51997865	51997913
Sma-1-17122	NW_003037932.1	+	257203	257251
Sma-1-17123	NS_000200.1	+	177790399	177790447
Sma-1-17124	NW_003037976.1	+	153501	153549
Sma-1-17125	NS_000200.1	+	206767816	206767864
Sma-1-17126	NW_003037882.1	-	124328	124280
Sma-1-17127	NS_000200.1	-	125416002	125415954
Sma-1-17128	NW_003038037.1	-	371292	371244
Sma-1-17129	NS_000200.1	-	250526701	250526653
Sma-1-17130	NW_003037859.1	+	447652	447700
Sma-1-17131	NS_000200.1	+	103126493	103126541
Sma-1-17132	NW_003038043.1	+	20999	21047
Sma-1-17133	NS_000200.1	+	254331041	254331089
Sma-1-17134	NW_003037844.1	-	426616	426568
Sma-1-17135	NS_000200.1	-	86610062	86610014
Sma-1-17136	NW_003038063.1	+	7720	7768
Sma-1-17137	NS_000200.1	+	261503998	261504046
Sma-1-17138	NW_003037924.1	-	661143	661095
Sma-1-17139	NS_000200.1	-	172368101	172368053
Sma-1-17140	NW_003037933.1	-	205356	205308
Sma-1-17141	NS_000200.1	-	178618528	178618480
Sma-1-17142	NW_003038076.1	-	295838	295790
Sma-1-17143	NS_000200.1	-	266513012	266512964
Sma-1-17144	NW_003037822.1	-	529056	529008
Sma-1-17145	NS_000200.1	-	54837062	54837014
Sma-1-17146	NW_003026618.1	+	345180	345229
Sma-1-17147	NS_000200.1	+	322100182	322100231
Sma-1-17148	NW_003037810.1	-	969978	969930
Sma-1-17149	NS_000200.1	-	37574500	37574452
Sma-1-17150	NW_003037968.1	+	243786	243834
Sma-1-17151	NS_000200.1	+	200160956	200161004
Sma-1-17152	NW_003037823.1	-	111138	111090
Sma-1-17153	NS_000200.1	-	56027757	56027709
Sma-1-17154	NW_003037889.1	-	1068495	1068447
Sma-1-17155	NS_000200.1	-	134844985	134844937
Sma-1-17156	NW_003037874.1	-	58528	58480
Sma-1-17157	NS_000200.1	-	119483478	119483430
Sma-1-17158	NW_003038258.1	+	184757	184805
Sma-1-17159	NS_000200.1	+	322723267	322723315
Sma-1-17160	NW_003037993.1	+	280897	280945
Sma-1-17161	NS_000200.1	+	218467600	218467648
Sma-1-17162	NW_003038151.1	+	233561	233609
Sma-1-17163	NS_000200.1	+	292140804	292140852
Sma-1-17164	NW_003038131.1	-	60855	60807
Sma-1-17165	NS_000200.1	-	282022699	282022651
Sma-1-17166	NW_003037823.1	-	138309	138261
Sma-1-17167	NS_000200.1	-	56054928	56054880
Sma-1-17168	NW_003037862.1	+	247760	247811
Sma-1-17169	NS_000200.1	+	105899421	105899472
Sma-1-17170	NW_003038083.1	+	238983	239031
Sma-1-17171	NS_000200.1	+	270482320	270482368
Sma-1-17172	NW_003032307.1	+	165	213

Sma-1-17173	NS_000200.1	+	358743069	358743117
Sma-1-17174	NW_003037889.1	-	1297543	1297495
Sma-1-17175	NS_000200.1	-	135074033	135073985
Sma-1-17176	NW_003023016.1	-	1684	1636
Sma-1-17177	NS_000200.1	-	372064509	372064461
Sma-1-17178	NW_003037917.1	-	402824	402776
Sma-1-17179	NS_000200.1	-	165439628	165439580
Sma-1-17180	NW_003037961.1	-	269190	269142
Sma-1-17181	NS_000200.1	-	195893432	195893384
Sma-1-17182	NW_003037925.1	-	72156	72108
Sma-1-17183	NS_000200.1	-	172474159	172474111
Sma-1-17184	NW_003037841.1	-	227451	227403
Sma-1-17185	NS_000200.1	-	82838632	82838584
Sma-1-17186	NW_003037796.1	-	1739813	1739765
Sma-1-17187	NS_000200.1	-	9949457	9949409
Sma-1-17188	NW_003038014.1	-	294866	294818
Sma-1-17189	NS_000200.1	-	230035555	230035507
Sma-1-17190	NW_003037807.1	+	319199	319247
Sma-1-17191	NS_000200.1	+	30580715	30580763
Sma-1-17192	NW_003037866.1	-	1234199	1234151
Sma-1-17193	NS_000200.1	-	112721998	112721950
Sma-1-17194	NW_003026476.1	-	107727	107679
Sma-1-17195	NS_000200.1	-	253656497	253656449
Sma-1-17196	NW_003037891.1	+	1379214	1379262
Sma-1-17197	NS_000200.1	+	139260492	139260540
Sma-1-17198	NW_003037896.1	+	150003	150051
Sma-1-17199	NS_000200.1	+	143070426	143070474
Sma-1-17200	NW_003037854.1	-	715034	714986
Sma-1-17201	NS_000200.1	-	97619455	97619407
Sma-1-17202	NW_003037868.1	-	356675	356627
Sma-1-17203	NS_000200.1	-	116328387	116328339
Sma-1-17204	NW_003037971.1	+	641294	641342
Sma-1-17205	NS_000200.1	+	202263344	202263392
Sma-1-17206	NW_003037916.1	-	166683	166635
Sma-1-17207	NS_000200.1	-	163360094	163360046
Sma-1-17208	NW_003038020.1	-	166843	166795
Sma-1-17209	NS_000200.1	-	234134187	234134139
Sma-1-17210	NW_003037788.1	-	26051	26003
Sma-1-17211	NS_000200.1	-	380473289	380473241
Sma-1-17212	NW_003037977.1	+	815505	815553
Sma-1-17213	NS_000200.1	+	209101525	209101573
Sma-1-17214	NW_003037918.1	-	1895506	1895458
Sma-1-17215	NS_000200.1	-	168462496	168462448
Sma-1-17216	NW_003038244.1	-	119812	119764
Sma-1-17217	NS_000200.1	-	315265017	315264969
Sma-1-17218	NW_003037873.1	-	180614	180566
Sma-1-17219	NS_000200.1	-	118919742	118919694
Sma-1-17220	NW_003037898.1	+	293710	293758
Sma-1-17221	NS_000200.1	+	144277532	144277580
Sma-1-17222	NW_003037809.1	-	1687526	1687478
Sma-1-17223	NS_000200.1	-	35351990	35351942
Sma-1-17224	NW_003037911.1	+	282235	282283

Sma-1-17225	NS_000200.1	+	156069588	156069636
Sma-1-17226	NW_003037798.1	+	2700643	2700691
Sma-1-17227	NS_000200.1	+	16959801	16959849
Sma-1-17228	NW_003037916.1	+	722653	722701
Sma-1-17229	NS_000200.1	+	163916064	163916112
Sma-1-17230	NW_003037812.1	-	1143456	1143408
Sma-1-17231	NS_000200.1	-	40626195	40626147
Sma-1-17232	NW_003037814.1	-	1593141	1593093
Sma-1-17233	NS_000200.1	-	44577001	44576953
Sma-1-17234	NW_003037826.1	-	1184638	1184561
Sma-1-17235	NS_000200.1	-	62184154	62184077
Sma-1-17236	NW_003037846.1	+	312564	312612
Sma-1-17237	NS_000200.1	+	87947134	87947182
Sma-1-17238	NW_003037832.1	+	393341	393389
Sma-1-17239	NS_000200.1	+	71602772	71602820
Sma-1-17240	NW_003037809.1	+	1037021	1037069
Sma-1-17241	NS_000200.1	+	34701485	34701533
Sma-1-17242	NW_003030881.1	-	2871	2823
Sma-1-17243	NS_000200.1	-	370443679	370443631
Sma-1-17244	NW_003037897.1	-	115023	114975
Sma-1-17245	NS_000200.1	-	143597399	143597351
Sma-1-17246	NW_003037982.1	+	1043547	1043595
Sma-1-17247	NS_000200.1	+	214702742	214702790
Sma-1-17248	NW_003026464.1	-	384881	384833
Sma-1-17249	NS_000200.1	-	203685190	203685142
Sma-1-17250	NW_003037800.1	+	638891	638939
Sma-1-17251	NS_000200.1	+	19268251	19268299
Sma-1-17252	NW_003038003.1	-	414438	414390
Sma-1-17253	NS_000200.1	-	223913720	223913672
Sma-1-17254	NW_003026472.1	+	117204	117252
Sma-1-17255	NS_000200.1	+	236529196	236529244
Sma-1-17256	NW_003026464.1	+	361565	361613
Sma-1-17257	NS_000200.1	+	203661874	203661922
Sma-1-17258	NW_003037826.1	-	1606533	1606485
Sma-1-17259	NS_000200.1	-	62606049	62606001
Sma-1-17260	NW_003026508.1	+	46480	46528
Sma-1-17261	NS_000200.1	+	281091094	281091142
Sma-1-17262	NW_003037939.1	+	483245	483293
Sma-1-17263	NS_000200.1	+	183352999	183353047
Sma-1-17264	NW_003038086.1	+	29092	29140
Sma-1-17265	NS_000200.1	+	272277685	272277733
Sma-1-17266	NW_003038192.1	-	894	846
Sma-1-17267	NS_000200.1	-	302562801	302562753
Sma-1-17268	NW_003037917.1	+	1274262	1274310
Sma-1-17269	NS_000200.1	+	166311066	166311114
Sma-1-17270	NW_003026459.1	+	206854	206902
Sma-1-17271	NS_000200.1	+	141852127	141852175
Sma-1-17272	NW_003037921.1	-	367272	367224
Sma-1-17273	NS_000200.1	-	169897407	169897359
Sma-1-17274	NW_003037801.1	+	813186	813234
Sma-1-17275	NS_000200.1	+	21902666	21902714
Sma-1-17276	NW_003037895.1	-	255629	255581

Sma-1-17277	NS_000200.1	-	142701831	142701783
Sma-1-17278	NW_003037796.1	-	280229	280181
Sma-1-17279	NS_000200.1	-	8489873	8489825
Sma-1-17280	NW_003037795.1	-	287906	287858
Sma-1-17281	NS_000200.1	-	6678789	6678741
Sma-1-17282	NW_003037795.1	-	990302	990254
Sma-1-17283	NS_000200.1	-	7381185	7381137
Sma-1-17284	NW_003037932.1	-	666573	666525
Sma-1-17285	NS_000200.1	-	178199769	178199721
Sma-1-17286	NW_003037844.1	-	225222	225174
Sma-1-17287	NS_000200.1	-	86408668	86408620
Sma-1-17288	NW_003037939.1	+	411423	411471
Sma-1-17289	NS_000200.1	+	183281177	183281225
Sma-1-17290	NW_003032670.1	-	370	322
Sma-1-17291	NW_003037798.1	-	1315680	1315632
Sma-1-17292	NS_000200.1	-	350591469	350591421
Sma-1-17293	NS_000200.1	-	15574838	15574790
Sma-1-17294	NW_003038081.1	-	258249	258201
Sma-1-17295	NS_000200.1	-	269420292	269420244
Sma-1-17296	NW_003037975.1	+	959721	959769
Sma-1-17297	NS_000200.1	+	206541945	206541993
Sma-1-17298	NW_003037867.1	+	131905	131953
Sma-1-17299	NS_000200.1	+	115732724	115732772
Sma-1-17300	NW_003038130.1	+	99737	99786
Sma-1-17301	NS_000200.1	+	281654723	281654772
Sma-1-17302	NW_003038246.1	+	215391	215439
Sma-1-17303	NS_000200.1	+	315856519	315856567
Sma-1-17304	NW_003037803.1	+	204614	204662
Sma-1-17305	NS_000200.1	+	23705151	23705199
Sma-1-17306	NW_003037972.1	+	486417	486466
Sma-1-17307	NS_000200.1	+	202962121	202962170
Sma-1-17308	NW_003038010.1	-	186677	186626
Sma-1-17309	NS_000200.1	-	227004900	227004849
Sma-1-17310	NW_003026471.1	-	353422	353371
Sma-1-17311	NS_000200.1	-	222465655	222465604
Sma-1-17312	NW_003037953.1	+	163504	163555
Sma-1-17313	NS_000200.1	+	193076555	193076606
Sma-1-17314	NW_003037809.1	-	1923208	1923157
Sma-1-17315	NS_000200.1	-	35587672	35587621
Sma-1-17316	NW_003038033.1	-	903429	903381
Sma-1-17317	NS_000200.1	-	246883431	246883383
Sma-1-17318	NW_003037819.1	-	1019612	1019564
Sma-1-17319	NS_000200.1	-	52107877	52107829
Sma-1-17320	NW_003037962.1	-	10552	10504
Sma-1-17321	NS_000200.1	-	196087395	196087347
Sma-1-17322	NW_003037797.1	-	296122	296074
Sma-1-17323	NS_000200.1	-	10723982	10723934
Sma-1-17324	NW_003037901.1	-	864740	864692
Sma-1-17325	NS_000200.1	-	146713201	146713153
Sma-1-17326	NW_003037844.1	-	402502	402454
Sma-1-17327	NS_000200.1	-	86585948	86585900
Sma-1-17328	NW_003033827.1	+	1423	1471

Sma-1-17329	NS_000200.1	+	359198569	359198617
Sma-1-17330	NW_003038034.1	+	352681	352729
Sma-1-17331	NS_000200.1	+	247294437	247294485
Sma-1-17332	NW_003037918.1	+	2084257	2084305
Sma-1-17333	NS_000200.1	+	168651247	168651295
Sma-1-17334	NW_003037915.1	+	208551	208602
Sma-1-17335	NS_000200.1	+	162108537	162108588
Sma-1-17336	NW_003037865.1	-	493953	493905
Sma-1-17337	NS_000200.1	-	110915872	110915824
Sma-1-17338	NW_003037910.1	-	69577	69529
Sma-1-17339	NS_000200.1	-	154581552	154581504
Sma-1-17340	NW_003037886.1	-	410260	410212
Sma-1-17341	NS_000200.1	-	129665827	129665779
Sma-1-17342	NW_003037889.1	+	191386	191434
Sma-1-17343	NS_000200.1	+	133967876	133967924
Sma-1-17344	NW_003038212.1	-	3137	3089
Sma-1-17345	NS_000200.1	-	306167028	306166980
Sma-1-17346	NW_003037852.1	-	501804	501756
Sma-1-17347	NS_000200.1	-	94893699	94893651
Sma-1-17348	NW_003037886.1	+	449647	449695
Sma-1-17349	NS_000200.1	+	129705214	129705262
Sma-1-17350	NW_003038025.1	+	237257	237305
Sma-1-17351	NS_000200.1	+	239095502	239095550
Sma-1-17352	NW_003037865.1	+	280655	280703
Sma-1-17353	NS_000200.1	+	110702574	110702622
Sma-1-17354	NW_003037890.1	-	440241	440193
Sma-1-17355	NS_000200.1	-	136123602	136123554
Sma-1-17356	NW_003038063.1	+	36965	37013
Sma-1-17357	NS_000200.1	+	261533243	261533291
Sma-1-17358	NW_003037824.1	+	895468	895516
Sma-1-17359	NS_000200.1	+	57826408	57826456
Sma-1-17360	NW_003037827.1	+	2336121	2336169
Sma-1-17361	NS_000200.1	+	64958262	64958310
Sma-1-17362	NW_003026507.1	-	303108	303060
Sma-1-17363	NS_000200.1	-	279963259	279963211
Sma-1-17364	NW_003037908.1	+	1000951	1000999
Sma-1-17365	NS_000200.1	+	153474082	153474130
Sma-1-17366	NW_003037795.1	+	309652	309700
Sma-1-17367	NS_000200.1	+	6700535	6700583
Sma-1-17368	NW_003037821.1	+	867706	867754
Sma-1-17369	NS_000200.1	+	54012190	54012238
Sma-1-17370	NW_003037804.1	-	1351524	1351476
Sma-1-17371	NS_000200.1	-	26491544	26491496
Sma-1-17372	NW_003026465.1	-	700058	700010
Sma-1-17373	NS_000200.1	-	208092652	208092604
Sma-1-17374	NW_003026465.1	+	702926	702974
Sma-1-17375	NS_000200.1	+	208095520	208095568
Sma-1-17376	NW_003038025.1	+	11144	11192
Sma-1-17377	NS_000200.1	+	238869389	238869437
Sma-1-17378	NW_003038049.1	+	229709	229757
Sma-1-17379	NS_000200.1	+	256205236	256205284
Sma-1-17380	NW_003037821.1	-	445542	445494

Sma-1-17381	NS_000200.1	-	53590026	53589978
Sma-1-17382	NW_003038032.1	-	604935	604887
Sma-1-17383	NS_000200.1	-	245764095	245764047
Sma-1-17384	NW_003038014.1	+	120658	120706
Sma-1-17385	NS_000200.1	+	229861347	229861395
Sma-1-17386	NW_003037981.1	+	1253624	1253672
Sma-1-17387	NS_000200.1	+	213651492	213651540
env-88	AF036395.1	+	119	163
Sma-1-17388	NW_003037951.1	-	125660	125612
Sma-1-17389	NS_000200.1	-	192803385	192803337
Sma-1-17390	NW_003038070.1	-	458837	458789
Sma-1-17391	NS_000200.1	-	264796078	264796030
Sma-1-17392	NW_003037866.1	+	1035105	1035153
Sma-1-17393	NS_000200.1	+	112522904	112522952
Sma-1-17394	NW_003037953.1	-	255576	255528
Sma-1-17395	NS_000200.1	-	193168627	193168579
Sma-1-17396	NW_003037810.1	-	1267722	1267674
Sma-1-17397	NS_000200.1	-	37872244	37872196
Sma-1-17398	NW_003026484.1	-	17057	17009
Sma-1-17399	NS_000200.1	-	256979748	256979700
Sma-1-17400	NW_003037827.1	-	1085837	1085789
Sma-1-17401	NS_000200.1	-	63707978	63707930
Sma-1-17402	NW_003038129.1	+	235231	235279
Sma-1-17403	NS_000200.1	+	281488578	281488626
Sma-1-17404	NW_003037795.1	-	309545	309497
Sma-1-17405	NS_000200.1	-	6700428	6700380
Sma-1-17406	NW_003037841.1	-	1306395	1306347
Sma-1-17407	NS_000200.1	-	83917576	83917528
Sma-1-17408	NW_003037796.1	+	1521294	1521342
Sma-1-17409	NS_000200.1	+	9730938	9730986
Sma-1-17410	NW_003037795.1	-	1219095	1219047
Sma-1-17411	NS_000200.1	-	7609978	7609930
Sma-1-17412	NW_003037824.1	+	950672	950720
Sma-1-17413	NS_000200.1	+	57881612	57881660
Sma-1-17414	NW_003020123.1	-	148	100
Sma-1-17415	NW_003038254.1	+	221891	221939
Sma-1-17416	NS_000200.1	+	320257449	320257497
Sma-1-17417	NS_000200.1	-	371047871	371047823
Sma-1-17418	NW_003037829.1	+	924112	924160
Sma-1-17419	NS_000200.1	+	66824650	66824698
Sma-1-17420	NW_003038007.1	+	251370	251418
Sma-1-17421	NS_000200.1	+	225499925	225499973
Sma-1-17422	NW_003026483.1	+	110944	110992
Sma-1-17423	NS_000200.1	+	256625468	256625516
Sma-1-17424	NW_003038260.1	-	367351	367303
Sma-1-17425	NS_000200.1	-	323539126	323539078
Sma-1-17426	NW_003037867.1	-	252909	252861
Sma-1-17427	NS_000200.1	-	115853728	115853680
Sma-1-17428	NW_003038518.1	+	640160	640208
Sma-1-17429	NS_000200.1	+	342276993	342277041
Sma-1-17430	NW_003037945.1	-	821449	821401
Sma-1-17431	NS_000200.1	-	189923413	189923365

Sma-1-17432	NW_003037800.1	-	2251075	2251027
Sma-1-17433	NS_000200.1	-	20880435	20880387
Sma-1-17434	NW_003026482.1	+	136211	136259
Sma-1-17435	NS_000200.1	+	255923957	255924005
Sma-1-17436	NW_003038032.1	+	225181	225229
Sma-1-17437	NS_000200.1	+	245384341	245384389
Sma-1-17438	NW_003037846.1	-	662616	662568
Sma-1-17439	NS_000200.1	-	88297186	88297138
Sma-1-17440	NW_003037793.1	-	3513656	3513608
Sma-1-17441	NS_000200.1	-	3513656	3513608
Sma-1-17442	NW_003038074.1	+	106769	106817
Sma-1-17443	NS_000200.1	+	265998149	265998197
Sma-1-17444	NW_003035602.1	+	8265	8313
Sma-1-17445	NS_000200.1	+	329347459	329347507
Sma-1-17446	NW_003037859.1	+	542156	542204
Sma-1-17447	NS_000200.1	+	103220997	103221045
Sma-1-17448	NW_003038007.1	-	122161	122078
Sma-1-17449	NS_000200.1	-	225370716	225370633
Sma-1-17450	NW_003038025.1	+	559506	559554
Sma-1-17451	NS_000200.1	+	239417751	239417799
Sma-1-17452	NW_003038033.1	+	838300	838348
Sma-1-17453	NS_000200.1	+	246818302	246818350
Sma-1-17454	NW_003037830.1	+	1383440	1383488
Sma-1-17455	NS_000200.1	+	69273811	69273859
Sma-1-17456	NW_003037945.1	+	1567543	1567591
Sma-1-17457	NS_000200.1	+	190669507	190669555
Sma-1-17458	NW_003038261.1	-	191601	191553
Sma-1-17459	NS_000200.1	-	323805252	323805204
Sma-1-17460	NW_003038033.1	+	91411	91459
Sma-1-17461	NS_000200.1	+	246071413	246071461
Sma-1-17462	NW_003037820.1	-	27040	26992
Sma-1-17463	NS_000200.1	-	52300022	52299974
Sma-1-17464	NW_003037820.1	+	268425	268473
Sma-1-17465	NS_000200.1	+	52541407	52541455
Sma-1-17466	NW_003038006.1	+	124897	124945
Sma-1-17467	NS_000200.1	+	225009381	225009429
Sma-1-17468	NW_003038005.1	+	463622	463670
Sma-1-17469	NS_000200.1	+	224869576	224869624
Sma-1-17470	NW_003037809.1	-	2193593	2193545
Sma-1-17471	NS_000200.1	-	35858057	35858009
Sma-1-17472	NW_003037800.1	+	750410	750458
Sma-1-17473	NS_000200.1	+	19379770	19379818
Sma-1-17474	NW_003037798.1	-	1364277	1364229
Sma-1-17475	NS_000200.1	-	15623435	15623387
Sma-1-17476	NW_003037797.1	+	3791675	3791723
Sma-1-17477	NS_000200.1	+	14219535	14219583
Sma-1-17478	NW_003029633.1	-	781	733
Sma-1-17479	NS_000200.1	-	366303473	366303425
Sma-1-17480	NW_003037881.1	+	456279	456327
Sma-1-17481	NS_000200.1	+	124888070	124888118
Sma-1-17482	NW_003037886.1	+	1083099	1083147
Sma-1-17483	NS_000200.1	+	130338666	130338714

Sma-1-17484	NW_003038063.1	+	232727	232775
Sma-1-17485	NS_000200.1	+	261729005	261729053
Sma-1-17486	NW_003037898.1	+	427698	427746
Sma-1-17487	NS_000200.1	+	144411520	144411568
Sma-1-17488	NW_003037864.1	+	1629348	1629396
Sma-1-17489	NS_000200.1	+	110121922	110121970
Sma-1-17490	NW_003037918.1	+	1665243	1665291
Sma-1-17491	NS_000200.1	+	168232233	168232281
Sma-1-17492	NW_003038258.1	+	114473	114521
Sma-1-17493	NS_000200.1	+	322652983	322653031
Sma-1-17494	NW_003038505.1	+	223154	223202
Sma-1-17495	NS_000200.1	+	336302491	336302539
Sma-1-17496	NW_003026462.1	+	288476	288524
Sma-1-17497	NS_000200.1	+	196955979	196956027
Sma-1-17498	NW_003037820.1	+	223975	224023
Sma-1-17499	NS_000200.1	+	52496957	52497005
Sma-1-17500	NW_003038153.1	+	550185	550233
Sma-1-17501	NS_000200.1	+	293612234	293612282
Sma-1-17502	NW_003038024.1	+	725381	725429
Sma-1-17503	NS_000200.1	+	238622410	238622458
Sma-1-17504	NW_003037846.1	-	789372	789324
Sma-1-17505	NS_000200.1	-	88423942	88423894
Sma-1-17506	NW_003026546.1	+	1402	1450
Sma-1-17507	NS_000200.1	+	300481907	300481955
Sma-1-17508	NW_003038005.1	-	384320	384272
Sma-1-17509	NS_000200.1	-	224790274	224790226
Sma-1-17510	NW_003038085.1	+	380334	380382
Sma-1-17511	NS_000200.1	+	272070427	272070475
Sma-1-17512	NW_003037794.1	+	911460	911508
Sma-1-17513	NS_000200.1	+	5091055	5091103
Sma-1-17514	NW_003038252.1	-	432658	432610
Sma-1-17515	NS_000200.1	-	319288246	319288198
Sma-1-17516	NW_003038085.1	-	510903	510855
Sma-1-17517	NS_000200.1	-	272200996	272200948
Sma-1-17518	NW_003037793.1	-	979478	979430
Sma-1-17519	NW_003037814.1	+	953949	953997
Sma-1-17520	NW_003038023.1	-	285632	285584
Sma-1-17521	NS_000200.1	+	43937809	43937857
Sma-1-17522	NS_000200.1	-	237445923	237445875
Sma-1-17523	NS_000200.1	-	979478	979430
Sma-1-17524	NW_003037902.1	-	517103	517055
Sma-1-17525	NS_000200.1	-	147272337	147272289
Sma-1-17526	NW_003038015.1	+	528551	528599
Sma-1-17527	NS_000200.1	+	230852041	230852089
Sma-1-17528	NW_003025194.1	-	1456	1407
Sma-1-17529	NS_000200.1	-	361375104	361375055
Sma-1-17530	NW_003037915.1	-	269624	269576
Sma-1-17531	NS_000200.1	-	162169610	162169562
Sma-1-17532	NW_003038252.1	-	396803	396755
Sma-1-17533	NS_000200.1	-	319252391	319252343
Sma-1-17534	NW_003026472.1	-	408567	408519
Sma-1-17535	NS_000200.1	-	236820559	236820511

Sma-1-17536	NW_003037910.1	-	1110653	1110605
Sma-1-17537	NS_000200.1	-	155622628	155622580
Sma-1-17538	NW_003037839.1	-	641288	641240
Sma-1-17539	NS_000200.1	-	79599438	79599390
Sma-1-17540	NW_003037906.1	-	126243	126195
Sma-1-17541	NS_000200.1	-	150434022	150433974
Sma-1-17542	NW_003038030.1	+	778802	778850
Sma-1-17543	NS_000200.1	+	243914669	243914717
Sma-1-17544	NW_003037906.1	-	105150	105102
Sma-1-17545	NS_000200.1	-	150412929	150412881
Sma-1-17546	NW_003037827.1	-	221010	220962
Sma-1-17547	NS_000200.1	-	62843151	62843103
Sma-1-17548	NW_003038086.1	+	289299	289347
Sma-1-17549	NS_000200.1	+	272537892	272537940
Sma-1-17550	NW_003037908.1	+	1046899	1046947
Sma-1-17551	NS_000200.1	+	153520030	153520078
Sma-1-17552	NW_003037885.1	+	1143306	1143354
Sma-1-17553	NS_000200.1	+	129241253	129241301
Sma-1-17554	NW_003037908.1	-	1031017	1030969
Sma-1-17555	NS_000200.1	-	153504148	153504100
Sma-1-17556	NW_003038086.1	-	362572	362524
Sma-1-17557	NS_000200.1	-	272611165	272611117
Sma-1-17558	NW_003026609.1	-	81137	81089
Sma-1-17559	NS_000200.1	-	309736129	309736081
Sma-1-17560	NW_003037886.1	-	980054	980006
Sma-1-17561	NS_000200.1	-	130235621	130235573
Sma-1-17562	NW_003037891.1	+	433785	433833
Sma-1-17563	NS_000200.1	+	138315063	138315111
Sma-1-17564	NW_003038003.1	-	229649	229601
Sma-1-17565	NS_000200.1	-	223728931	223728883
Sma-1-17566	NW_003037796.1	+	1767386	1767434
Sma-1-17567	NS_000200.1	+	9977030	9977078
Sma-1-17568	NW_003037932.1	+	692399	692447
Sma-1-17569	NS_000200.1	+	178225595	178225643
Sma-1-17570	NW_003022225.1	+	321	369
Sma-1-17571	NS_000200.1	+	360961686	360961734
Sma-1-17572	NW_003035880.1	-	27319	27271
Sma-1-17573	NS_000200.1	-	339268383	339268335
Sma-1-17574	NW_003037829.1	+	692890	692938
Sma-1-17575	NS_000200.1	+	66593428	66593476
Sma-1-17576	NW_003026610.1	+	103557	103605
Sma-1-17577	NS_000200.1	+	310258927	310258975
Sma-1-17578	NW_003037873.1	-	589469	589421
Sma-1-17579	NS_000200.1	-	119328597	119328549
Sma-1-17580	NW_003037861.1	+	647233	647281
Sma-1-17581	NS_000200.1	+	105423687	105423735
Sma-1-17582	NW_003037838.1	+	751205	751253
Sma-1-17583	NS_000200.1	+	78749768	78749816
Sma-1-17584	NW_003037854.1	-	1773820	1773772
Sma-1-17585	NS_000200.1	-	98678241	98678193
Sma-1-17586	NW_003038414.1	-	43693	43645
Sma-1-17587	NS_000200.1	-	328760713	328760665

Sma-1-17588	NW_003037793.1	-	3536543	3536495
Sma-1-17589	NS_000200.1	-	3536543	3536495
Sma-1-17590	NW_003035881.1	+	187982	188030
Sma-1-17591	NS_000200.1	+	341252187	341252235
Sma-1-17592	NW_003037845.1	-	28672	28624
Sma-1-17593	NS_000200.1	-	87030575	87030527
Sma-1-17594	NW_003037915.1	+	220429	220480
Sma-1-17595	NS_000200.1	+	162120415	162120466
Sma-1-17596	NW_003039127.1	+	202213	202261
Sma-1-17597	NS_000200.1	+	113279712	113279760
Sma-1-17598	NW_003037797.1	+	624300	624348
Sma-1-17599	NS_000200.1	+	11052160	11052208
Sma-1-17600	NW_003026488.1	+	267486	267534
Sma-1-17601	NS_000200.1	+	263773980	263774028
Sma-1-17602	NW_003038124.1	+	217214	217262
Sma-1-17603	NS_000200.1	+	279209858	279209906
Sma-1-17604	NW_003037884.1	-	959447	959399
Sma-1-17605	NS_000200.1	-	127739112	127739064
Sma-1-17606	NW_003037873.1	+	78342	78390
Sma-1-17607	NS_000200.1	+	118817470	118817518
Sma-1-17608	NW_003037847.1	-	31516	31468
Sma-1-17609	NS_000200.1	-	88683246	88683198
Sma-1-17610	NW_003037934.1	+	194419	194467
Sma-1-17611	NS_000200.1	+	179241166	179241214
Sma-1-17612	NW_003037863.1	-	519042	518994
Sma-1-17613	NS_000200.1	-	107277409	107277361
Sma-1-17614	NW_003037979.1	-	273067	273019
Sma-1-17615	NS_000200.1	-	210334087	210334039
Sma-1-17616	NW_003037874.1	-	135989	135941
Sma-1-17617	NS_000200.1	-	119560939	119560891
Sma-1-17618	NW_003037968.1	+	12942	12990
Sma-1-17619	NS_000200.1	+	199930112	199930160
Sma-1-17620	NW_003037915.1	+	1159037	1159088
Sma-1-17621	NS_000200.1	+	163059023	163059074
Sma-1-17622	NW_003038016.1	-	314249	314201
Sma-1-17623	NS_000200.1	-	231430786	231430738
Sma-1-17624	NW_003038037.1	+	1593656	1593704
Sma-1-17625	NS_000200.1	+	251749065	251749113
Sma-1-17626	NW_003037943.1	+	537641	537690
Sma-1-17627	NS_000200.1	+	187559174	187559223
Sma-1-17628	NW_003037900.1	+	38659	38707
Sma-1-17629	NS_000200.1	+	145167525	145167573
Sma-1-17630	NW_003035890.1	-	10745	10697
Sma-1-17631	NS_000200.1	-	342867243	342867195
Sma-1-17632	NW_003037884.1	+	839937	839985
Sma-1-17633	NS_000200.1	+	127619602	127619650
Sma-1-17634	NW_003037979.1	-	457164	457116
Sma-1-17635	NS_000200.1	-	210518184	210518136
Sma-1-17636	NW_003038119.1	+	222517	222565
Sma-1-17637	NS_000200.1	+	278130073	278130121
Sma-1-17638	NW_003037848.1	-	158971	158923
Sma-1-17639	NS_000200.1	-	89852042	89851994

Sma-1-17640	NW_003023739.1	-	859	811
Sma-1-17641	NS_000200.1	-	368728525	368728477
Sma-1-17642	NW_003037942.1	-	952585	952536
Sma-1-17643	NS_000200.1	-	186712493	186712444
Sma-1-17644	NW_003038023.1	+	562709	562757
Sma-1-17645	NS_000200.1	+	237723000	237723048
Sma-1-17646	NW_003026470.1	-	332424	332376
Sma-1-17647	NS_000200.1	-	219456393	219456345
Sma-1-17648	NW_003037884.1	-	1301726	1301678
Sma-1-17649	NS_000200.1	-	128081391	128081343
Sma-1-17650	NW_003037806.1	-	1615055	1615007
Sma-1-17651	NS_000200.1	-	29810269	29810221
Sma-1-17652	NW_003037818.1	-	721788	721740
Sma-1-17653	NS_000200.1	-	49437195	49437147
Sma-1-17654	NW_003037814.1	+	2138963	2139011
Sma-1-17655	NS_000200.1	+	45122823	45122871
Sma-1-17656	NW_003037793.1	-	2764524	2764476
Sma-1-17657	NS_000200.1	-	2764524	2764476
Sma-1-17658	NW_003037858.1	-	798375	798327
Sma-1-17659	NS_000200.1	-	101462651	101462603
Sma-1-17660	NW_003037969.1	-	193372	193324
Sma-1-17661	NS_000200.1	-	200799499	200799451
Sma-1-17662	NW_003024185.1	+	769	817
Sma-1-17663	NS_000200.1	+	348522203	348522251
Sma-1-17664	NW_003038037.1	-	996118	996069
Sma-1-17665	NS_000200.1	-	251151527	251151478
Sma-1-17666	NW_003026470.1	-	127025	126977
Sma-1-17667	NS_000200.1	-	219250994	219250946
Sma-1-17668	NW_003037818.1	-	554938	554890
Sma-1-17669	NS_000200.1	-	49270345	49270297
Sma-1-17670	NW_003026469.1	+	16050	16098
Sma-1-17671	NS_000200.1	+	217637709	217637757
Sma-1-17672	NW_003037961.1	+	372952	373000
Sma-1-17673	NS_000200.1	+	195997194	195997242
Sma-1-17674	NW_003036859.1	-	2782	2734
Sma-1-17675	NS_000200.1	-	347862624	347862576
Sma-1-17676	NW_003037977.1	+	494721	494769
Sma-1-17677	NS_000200.1	+	208780741	208780789
Sma-1-17678	NW_003038157.1	+	619663	619711
Sma-1-17679	NS_000200.1	+	295870583	295870631
Sma-1-17680	NW_003037996.1	-	505018	504970
Sma-1-17681	NS_000200.1	-	220749894	220749846
Sma-1-17682	NW_003037793.1	+	950533	950581
Sma-1-17683	NS_000200.1	+	950533	950581
Sma-1-17684	NW_003037918.1	+	539752	539800
Sma-1-17685	NS_000200.1	+	167106742	167106790
Sma-1-17686	NW_003037806.1	+	1477930	1477978
Sma-1-17687	NS_000200.1	+	29673144	29673192
Sma-1-17688	NW_003037909.1	+	220385	220433
Sma-1-17689	NS_000200.1	+	153762034	153762082
Sma-1-17690	NW_003037826.1	+	555851	555899
Sma-1-17691	NS_000200.1	+	61555367	61555415

Sma-1-17692	NW_003038038.1	-	113983	113935
Sma-1-17693	NS_000200.1	-	252017376	252017328
Sma-1-17694	NW_003037877.1	+	728009	728057
Sma-1-17695	NS_000200.1	+	121766571	121766619
Sma-1-17696	NW_003037794.1	-	580326	580278
Sma-1-17697	NS_000200.1	-	4759921	4759873
Sma-1-17698	NW_003037878.1	+	237595	237643
Sma-1-17699	NS_000200.1	+	122291073	122291121
Sma-1-17700	NW_003037793.1	-	3807648	3807600
Sma-1-17701	NS_000200.1	-	3807648	3807600
Sma-1-17702	NW_003038028.1	+	726825	726873
Sma-1-17703	NS_000200.1	+	242034399	242034447
Sma-1-17704	NW_003037941.1	+	915728	915776
Sma-1-17705	NS_000200.1	+	185650202	185650250
Sma-1-17706	NW_003037977.1	-	847809	847761
Sma-1-17707	NS_000200.1	-	209133829	209133781
Sma-1-17708	NW_003037806.1	-	1501826	1501778
Sma-1-17709	NS_000200.1	-	29697040	29696992
Sma-1-17710	NW_003037977.1	-	135738	135690
Sma-1-17711	NS_000200.1	-	208421758	208421710
Sma-1-17712	NW_003038038.1	-	264969	264921
Sma-1-17713	NS_000200.1	-	252168362	252168314
Sma-1-17714	NW_003037795.1	-	1303943	1303895
Sma-1-17715	NS_000200.1	-	7694826	7694778
Sma-1-17716	NW_003037941.1	-	32133	32085
Sma-1-17717	NS_000200.1	-	184766607	184766559
Sma-1-17718	NW_003037797.1	+	198007	198055
Sma-1-17719	NS_000200.1	+	10625867	10625915
Sma-1-17720	NW_003038498.1	-	98498	98450
Sma-1-17721	NS_000200.1	-	334518710	334518662
Sma-1-17722	NW_003037822.1	-	860346	860296
Sma-1-17723	NS_000200.1	-	55168352	55168302
Sma-1-17724	NW_003037822.1	-	88591	88543
Sma-1-17725	NS_000200.1	-	54396597	54396549
Sma-1-17726	NW_003037913.1	-	286774	286726
Sma-1-17727	NS_000200.1	-	158850525	158850477
Sma-1-17728	NW_003037797.1	-	1912876	1912828
Sma-1-17729	NS_000200.1	-	12340736	12340688
Sma-1-17730	NW_003037930.1	-	204679	204631
Sma-1-17731	NS_000200.1	-	176062643	176062595
Sma-1-17732	NW_003037887.1	+	239777	239825
Sma-1-17733	NS_000200.1	+	130703755	130703803
Sma-1-17734	NW_003026512.1	+	255544	255592
Sma-1-17735	NS_000200.1	+	285349584	285349632
Sma-1-17736	NW_003037857.1	+	542409	542457
Sma-1-17737	NS_000200.1	+	100562766	100562814
Sma-1-17738	NW_003037795.1	+	1410519	1410567
Sma-1-17739	NS_000200.1	+	7801402	7801450
Sma-1-17740	NW_003037901.1	+	758360	758408
Sma-1-17741	NS_000200.1	+	146606821	146606869
Sma-1-17742	NW_003037795.1	+	622621	622669
Sma-1-17743	NS_000200.1	+	7013504	7013552

Sma-1-17744	NW_003026517.1	+	460921	460969
Sma-1-17745	NS_000200.1	+	300066930	300066978
Sma-1-17746	NW_003037836.1	+	1214466	1214514
Sma-1-17747	NS_000200.1	+	76771565	76771613
Sma-1-17748	NW_003038028.1	+	714281	714329
Sma-1-17749	NS_000200.1	+	242021855	242021903
Sma-1-17750	NW_003037913.1	-	569613	569565
Sma-1-17751	NS_000200.1	-	159133364	159133316
Sma-1-17752	NW_003037932.1	+	81495	81543
Sma-1-17753	NS_000200.1	+	177614691	177614739
Sma-1-17754	NW_003037841.1	-	275059	275011
Sma-1-17755	NS_000200.1	-	82886240	82886192
Sma-1-17756	NW_003038022.1	+	265287	265335
Sma-1-17757	NS_000200.1	+	235946129	235946177
Sma-1-17758	NW_003038505.1	-	210583	210535
Sma-1-17759	NS_000200.1	-	336289920	336289872
Sma-1-17760	NW_003038018.1	+	362704	362752
Sma-1-17761	NS_000200.1	+	232763554	232763602
Sma-1-17762	NW_003037976.1	-	415630	415582
Sma-1-17763	NS_000200.1	-	207029945	207029897
Sma-1-17764	NW_003037975.1	-	521185	521137
Sma-1-17765	NS_000200.1	-	206103409	206103361
Sma-1-17766	NW_003037796.1	+	412278	412326
Sma-1-17767	NS_000200.1	+	8621922	8621970
Sma-1-17768	NW_003037862.1	-	495729	495681
Sma-1-17769	NS_000200.1	-	106147390	106147342
Sma-1-17770	NW_003037890.1	+	1114294	1114342
Sma-1-17771	NS_000200.1	+	136797655	136797703
Sma-1-17772	NW_003037890.1	+	180751	180799
Sma-1-17773	NS_000200.1	+	135864112	135864160
Sma-1-17774	NW_003037970.1	-	381454	381401
Sma-1-17775	NS_000200.1	-	201437644	201437591
Sma-1-17776	NW_003026618.1	-	72051	72002
Sma-1-17777	NS_000200.1	-	321827053	321827004
Sma-1-17778	NW_003037888.1	+	856127	856175
Sma-1-17779	NS_000200.1	+	133064619	133064667
Sma-1-17780	NW_003037881.1	-	846459	846411
Sma-1-17781	NS_000200.1	-	125278250	125278202
Sma-1-17782	NW_003037805.1	+	401073	401121
Sma-1-17783	NS_000200.1	+	26958917	26958965
Sma-1-17784	NW_003039127.1	+	1029650	1029698
Sma-1-17785	NS_000200.1	+	114107149	114107197
Sma-1-17786	NW_003038010.1	-	371417	371369
Sma-1-17787	NS_000200.1	-	227189640	227189592
Sma-1-17788	NW_003037807.1	+	63788	63836
Sma-1-17789	NS_000200.1	+	30325304	30325352
Sma-1-17790	NW_003037835.1	-	177740	177692
Sma-1-17791	NS_000200.1	-	74574248	74574200
Sma-1-17792	NW_003037850.1	+	514449	514497
Sma-1-17793	NS_000200.1	+	92500479	92500527
Sma-1-17794	NW_003037794.1	-	1218492	1218444
Sma-1-17795	NS_000200.1	-	5398087	5398039

Sma-1-17796	NW_003037791.1	-	403295	403247
Sma-1-17797	NS_000200.1	-	382108723	382108675
Sma-1-17798	NW_003037878.1	+	297921	297969
Sma-1-17799	NS_000200.1	+	122351399	122351447
Sma-1-17800	NW_003037825.1	+	879718	879766
Sma-1-17801	NS_000200.1	+	60009500	60009548
Sma-1-17802	NW_003037852.1	-	494424	494376
Sma-1-17803	NS_000200.1	-	94886319	94886271
Sma-1-17804	NW_003037913.1	+	951511	951559
Sma-1-17805	NS_000200.1	+	159515262	159515310
Sma-1-17806	NW_003038028.1	+	4248	4296
Sma-1-17807	NS_000200.1	+	241311822	241311870
Sma-1-17808	NW_003037814.1	-	1595201	1595153
Sma-1-17809	NS_000200.1	-	44579061	44579013
Sma-1-17810	NW_003037809.1	-	2393865	2393817
Sma-1-17811	NS_000200.1	-	36058329	36058281
Sma-1-17812	NW_003037938.1	+	643136	643184
Sma-1-17813	NS_000200.1	+	182680249	182680297
Sma-1-17814	NW_003026611.1	-	165210	165162
Sma-1-17815	NS_000200.1	-	310734994	310734946
Sma-1-17816	NW_003038043.1	-	157369	157321
Sma-1-17817	NS_000200.1	-	254467411	254467363
Sma-1-17818	NW_003037808.1	-	506891	506843
Sma-1-17819	NS_000200.1	-	32293528	32293480
Sma-1-17820	NW_003037873.1	+	66619	66667
Sma-1-17821	NW_003037873.1	-	63553	63505
Sma-1-17822	NS_000200.1	+	118805747	118805795
Sma-1-17823	NS_000200.1	-	118802681	118802633
Sma-1-17824	NW_003037873.1	-	64091	64043
Sma-1-17825	NS_000200.1	-	118803219	118803171
Sma-1-17826	NW_003038043.1	+	167555	167603
Sma-1-17827	NS_000200.1	+	254477597	254477645
Sma-1-17828	NW_003026465.1	+	41813	41861
Sma-1-17829	NS_000200.1	+	207434407	207434455
Sma-1-17830	NW_003037851.1	+	373778	373826
Sma-1-17831	NS_000200.1	+	93777088	93777136
Sma-1-17832	NW_003026456.1	+	537223	537271
Sma-1-17833	NS_000200.1	+	58460359	58460407
Sma-1-17834	NW_003037800.1	-	1134480	1134432
Sma-1-17835	NS_000200.1	-	19763840	19763792
Sma-1-17836	NW_003038249.1	+	240583	240631
Sma-1-17837	NS_000200.1	+	316922836	316922884
Sma-1-17838	NW_003026572.1	+	22899	22947
Sma-1-17839	NS_000200.1	+	301748988	301749036
Sma-1-17840	NW_003037928.1	+	340286	340334
Sma-1-17841	NS_000200.1	+	174664239	174664287
Sma-1-17842	NW_003037928.1	+	226053	226101
Sma-1-17843	NS_000200.1	+	174550006	174550054
Sma-1-17844	NW_003037914.1	-	1167143	1167095
Sma-1-17845	NS_000200.1	-	161311393	161311345
Sma-1-17846	NW_003037800.1	-	1574126	1574078
Sma-1-17847	NS_000200.1	-	20203486	20203438

Sma-1-17848	NW_003038078.1	+	18165	18213
Sma-1-17849	NS_000200.1	+	267224368	267224416
Sma-1-17850	NW_003038077.1	+	66909	66957
Sma-1-17851	NS_000200.1	+	266838549	266838597
Sma-1-17852	NW_003037889.1	+	1397483	1397534
Sma-1-17853	NS_000200.1	+	135173973	135174024
Sma-1-17854	NW_003026594.1	-	38490	38442
Sma-1-17855	NS_000200.1	-	304982860	304982812
Sma-1-17856	NW_003037973.1	-	13880	13830
Sma-1-17857	NS_000200.1	-	203995607	203995557
Sma-1-17858	NW_003037981.1	+	1249662	1249710
Sma-1-17859	NS_000200.1	+	213647530	213647578
Sma-1-17860	NW_003038028.1	+	17	65
Sma-1-17861	NS_000200.1	+	241307591	241307639
Sma-1-17862	NW_003026490.1	+	74450	74498
Sma-1-17863	NS_000200.1	+	267706166	267706214
Sma-1-17864	NW_003037980.1	+	38999	39047
Sma-1-17865	NS_000200.1	+	210999861	210999909
Sma-1-17866	NW_003037844.1	-	385739	385689
Sma-1-17867	NS_000200.1	-	86569185	86569135
Sma-1-17868	NW_003037800.1	-	389152	389104
Sma-1-17869	NS_000200.1	-	19018512	19018464
Sma-1-17870	NW_003038019.1	-	264706	264658
Sma-1-17871	NS_000200.1	-	233581524	233581476
Sma-1-17872	NW_003037939.1	-	677940	677892
Sma-1-17873	NS_000200.1	-	183547694	183547646
Sma-1-17874	NW_003037792.1	+	172818	172866
Sma-1-17875	NS_000200.1	+	382610162	382610210
Sma-1-17876	NW_003037939.1	-	504880	504832
Sma-1-17877	NS_000200.1	-	183374634	183374586
Sma-1-17878	NW_003037889.1	+	1711946	1711994
Sma-1-17879	NS_000200.1	+	135488436	135488484
Sma-1-17880	NW_003037908.1	-	945956	945908
Sma-1-17881	NS_000200.1	-	153419087	153419039
Sma-1-17882	NW_003037972.1	-	366750	366702
Sma-1-17883	NS_000200.1	-	202842454	202842406
Sma-1-17884	NW_003037976.1	-	149627	149579
Sma-1-17885	NS_000200.1	-	206763942	206763894
Sma-1-17886	NW_003038063.1	-	213687	213639
Sma-1-17887	NS_000200.1	-	261709965	261709917
Sma-1-17888	NW_003037917.1	-	1342692	1342644
Sma-1-17889	NS_000200.1	-	166379496	166379448
Sma-1-17890	NW_003037921.1	-	17755	17707
Sma-1-17891	NS_000200.1	-	169547890	169547842
Sma-1-17892	NW_003037814.1	-	980953	980905
Sma-1-17893	NS_000200.1	-	43964813	43964765
Sma-1-17894	NW_003037814.1	+	92242	92290
Sma-1-17895	NS_000200.1	+	43076102	43076150
Sma-1-17896	NW_003037915.1	+	1148119	1148167
Sma-1-17897	NS_000200.1	+	163048105	163048153
Sma-1-17898	NW_003026497.1	+	14380	14428
Sma-1-17899	NW_003037889.1	+	777570	777618

Sma-1-17900	NS_000200.1	+	134554060	134554108
Sma-1-17901	NS_000200.1	+	274674572	274674620
Sma-1-17902	NW_003037908.1	-	1031685	1031637
Sma-1-17903	NS_000200.1	-	153504816	153504768
Sma-1-17904	NW_003037939.1	-	394144	394096
Sma-1-17905	NS_000200.1	-	183263898	183263850
Sma-1-17906	NW_003037918.1	-	71632	71584
Sma-1-17907	NS_000200.1	-	166638622	166638574
Sma-1-17908	NW_003037832.1	+	61648	61696
Sma-1-17909	NS_000200.1	+	71271079	71271127
Sma-1-17910	NW_003037889.1	-	1756402	1756354
Sma-1-17911	NS_000200.1	-	135532892	135532844
Sma-1-17912	NW_003037939.1	-	362926	362878
Sma-1-17913	NS_000200.1	-	183232680	183232632
Sma-1-17914	NW_003030306.1	-	1155	1107
Sma-1-17915	NS_000200.1	-	362638438	362638390
Sma-1-17916	NW_003037982.1	-	1037347	1037299
Sma-1-17917	NS_000200.1	-	214696542	214696494
Sma-1-17918	NW_003037797.1	+	272201	272249
Sma-1-17919	NS_000200.1	+	10700061	10700109
Sma-1-17920	NW_003030458.1	-	885	837
Sma-1-17921	NS_000200.1	-	362953158	362953110
Sma-1-17922	NW_003037962.1	+	472433	472481
Sma-1-17923	NS_000200.1	+	196549276	196549324
Sma-1-17924	NW_003037803.1	-	169666	169618
Sma-1-17925	NS_000200.1	-	23670203	23670155
Sma-1-17926	NW_003037827.1	-	390120	390072
Sma-1-17927	NS_000200.1	-	63012261	63012213
Sma-1-17928	NW_003037793.1	+	250341	250389
Sma-1-17929	NS_000200.1	+	250341	250389
Sma-1-17930	NW_003022670.1	+	258	307
Sma-1-17931	NS_000200.1	+	365389328	365389377
Sma-1-17932	NW_003037913.1	-	1327791	1327742
Sma-1-17933	NS_000200.1	-	159891542	159891493
Sma-1-17934	NW_003026517.1	-	313005	312956
Sma-1-17935	NS_000200.1	-	299919014	299918965
Sma-1-17936	NW_003038025.1	-	26340	26292
Sma-1-17937	NS_000200.1	-	238884585	238884537
Sma-1-17938	NW_003037893.1	-	232857	232808
Sma-1-17939	NS_000200.1	-	141347167	141347118
Sma-1-17940	NW_003037975.1	-	48240	48191
Sma-1-17941	NS_000200.1	-	205630464	205630415
Sma-1-17942	NW_003037833.1	-	749872	749823
Sma-1-17943	NS_000200.1	-	72940968	72940919
Sma-1-17944	NW_003037914.1	-	186229	186180
Sma-1-17945	NS_000200.1	-	160330479	160330430
Sma-1-17946	NW_003037801.1	+	824593	824642
Sma-1-17947	NS_000200.1	+	21914073	21914122
Sma-1-17948	NW_003038135.1	-	187210	187159
Sma-1-17949	NW_003038135.1	-	181517	181466
Sma-1-17950	NS_000200.1	-	284059549	284059498
Sma-1-17951	NS_000200.1	-	284053856	284053805

Sma-1-17952	NW_003037856.1	+	622481	622533
Sma-1-17953	NS_000200.1	+	99980437	99980489
Sma-1-17954	NW_003026619.1	+	148375	148426
Sma-1-17955	NS_000200.1	+	322339119	322339170
Sma-1-17956	NW_003037970.1	+	306002	306053
Sma-1-17957	NS_000200.1	+	201362192	201362243
Sma-1-17958	NW_003038077.1	+	206624	206675
Sma-1-17959	NS_000200.1	+	266978264	266978315
Sma-1-17960	NW_003038077.1	+	60344	60395
Sma-1-17961	NS_000200.1	+	266831984	266832035
Sma-1-17962	NW_003038249.1	-	245340	245289
Sma-1-17963	NS_000200.1	-	316927593	316927542
Sma-1-17964	NW_003024539.1	+	70	121
Sma-1-17965	NS_000200.1	+	348816488	348816539
Sma-1-17966	NW_003038261.1	+	995488	995539
Sma-1-17967	NS_000200.1	+	324609139	324609190
Sma-1-17968	NW_003038044.1	+	96164	96215
Sma-1-17969	NS_000200.1	+	254720946	254720997
Sma-1-17970	NW_003037809.1	+	2572393	2572444
Sma-1-17971	NS_000200.1	+	36236857	36236908
Sma-1-17972	NW_003037827.1	+	2444700	2444748
Sma-1-17973	NS_000200.1	+	65066841	65066889
Sma-1-17974	NW_003037796.1	-	302932	302884
Sma-1-17975	NS_000200.1	-	8512576	8512528
Sma-1-17976	NW_003037915.1	+	1150559	1150607
Sma-1-17977	NS_000200.1	+	163050545	163050593
Sma-1-17978	NW_003037961.1	+	354921	354969
Sma-1-17979	NS_000200.1	+	195979163	195979211
Sma-1-17980	NW_003038514.1	-	208068	208020
Sma-1-17981	NS_000200.1	-	339832607	339832559
Sma-1-17982	NW_003037866.1	+	1085169	1085217
Sma-1-17983	NS_000200.1	+	112572968	112573016
Sma-1-17984	NW_003035874.1	-	22585	22537
Sma-1-17985	NS_000200.1	-	336000129	336000081
Sma-1-17986	NW_003037925.1	+	360299	360347
Sma-1-17987	NS_000200.1	+	172762302	172762350
Sma-1-17988	NW_003037914.1	+	1373667	1373715
Sma-1-17989	NS_000200.1	+	161517917	161517965
env-89	AB231864.1	+	52925	52969
Sma-1-17990	NW_003026458.1	-	123293	123245
Sma-1-17991	NS_000200.1	-	101991523	101991475
Sma-1-17992	NW_003038086.1	+	315242	315288
Sma-1-17993	NS_000200.1	+	272563835	272563881
Sma-1-17994	NW_003037809.1	+	236194	236245
Sma-1-17995	NS_000200.1	+	33900658	33900709
Sma-1-17996	NW_003037977.1	-	131024	130977
Sma-1-17997	NS_000200.1	-	208417044	208416997
Sma-1-17998	NW_003026510.1	-	208119	208072
Sma-1-17999	NS_000200.1	-	283648300	283648253
Sma-1-18000	NW_003037793.1	+	3121800	3121847
Sma-1-18001	NS_000200.1	+	3121800	3121847
Sma-1-18002	NW_003037910.1	+	1127760	1127807

Sma-1-18003	NS_000200.1	+	155639735	155639782
Sma-1-18004	NW_003037936.1	-	605295	605247
Sma-1-18005	NS_000200.1	-	181045024	181044976
Sma-1-18006	NW_003037796.1	-	39066	39018
Sma-1-18007	NS_000200.1	-	8248710	8248662
Sma-1-18008	NW_003037853.1	-	490473	490425
Sma-1-18009	NS_000200.1	-	95982524	95982476
Sma-1-18010	NW_003038161.1	-	433960	433912
Sma-1-18011	NS_000200.1	-	297803811	297803763
Sma-1-18012	NW_003037851.1	+	84886	84934
Sma-1-18013	NS_000200.1	+	93488196	93488244
Sma-1-18014	NW_003038161.1	+	411620	411668
Sma-1-18015	NS_000200.1	+	297781471	297781519
Sma-1-18016	NW_003026612.1	+	114277	114326
Sma-1-18017	NS_000200.1	+	312419664	312419713
Sma-1-18018	NW_003037889.1	+	1361301	1361349
Sma-1-18019	NW_003038141.1	-	278733	278685
Sma-1-18020	NS_000200.1	+	135137791	135137839
Sma-1-18021	NS_000200.1	-	287168712	287168664
Sma-1-18022	NW_003037932.1	-	843532	843484
Sma-1-18023	NS_000200.1	-	178376728	178376680
Sma-1-18024	NW_003037848.1	+	1043444	1043492
Sma-1-18025	NS_000200.1	+	90736515	90736563
Sma-1-18026	NW_003037884.1	-	1152058	1152010
Sma-1-18027	NS_000200.1	-	127931723	127931675
Sma-1-18028	NW_003037822.1	-	1408894	1408846
Sma-1-18029	NS_000200.1	-	55716900	55716852
Sma-1-18030	NW_003038063.1	+	70286	70334
Sma-1-18031	NS_000200.1	+	261566564	261566612
Sma-1-18032	NW_003037850.1	-	394094	394046
Sma-1-18033	NS_000200.1	-	92380124	92380076
Sma-1-18034	NW_003037961.1	-	152073	152025
Sma-1-18035	NS_000200.1	-	195776315	195776267
Sma-1-18036	NW_003037819.1	-	931518	931470
Sma-1-18037	NS_000200.1	-	52019783	52019735
Sma-1-18038	NW_003037891.1	+	2782044	2782092
Sma-1-18039	NW_003037935.1	-	729045	728997
Sma-1-18040	NS_000200.1	+	140663322	140663370
Sma-1-18041	NS_000200.1	-	180145580	180145532
Sma-1-18042	NW_003037838.1	+	379390	379438
Sma-1-18043	NW_003037891.1	+	1240167	1240215
Sma-1-18044	NS_000200.1	+	78377953	78378001
Sma-1-18045	NS_000200.1	+	139121445	139121493
Sma-1-18046	NW_003028426.1	+	24	72
Sma-1-18047	NS_000200.1	+	369593969	369594017
Sma-1-18048	NW_003037825.1	+	1167355	1167403
Sma-1-18049	NS_000200.1	+	60297137	60297185
Sma-1-18050	NW_003028854.1	+	1424	1472
Sma-1-18051	NS_000200.1	+	370089049	370089097
Sma-1-18052	NW_003037793.1	+	888204	888252
Sma-1-18053	NS_000200.1	+	888204	888252
Sma-1-18054	NW_003037845.1	-	235715	235667

Sma-1-18055	NS_000200.1	-	87237618	87237570
Sma-1-18056	NW_003037865.1	-	92888	92840
Sma-1-18057	NS_000200.1	-	110514807	110514759
Sma-1-18058	NW_003037825.1	+	1200550	1200598
Sma-1-18059	NS_000200.1	+	60330332	60330380
Sma-1-18060	NW_003026497.1	-	8447	8399
Sma-1-18061	NS_000200.1	-	274668639	274668591
Sma-1-18062	NW_003037843.1	+	488735	488783
Sma-1-18063	NS_000200.1	+	86128042	86128090
Sma-1-18064	NW_003026490.1	+	28862	28910
Sma-1-18065	NS_000200.1	+	267660578	267660626
Sma-1-18066	NW_003037932.1	+	531597	531645
Sma-1-18067	NS_000200.1	+	178064793	178064841
Sma-1-18068	NW_003037841.1	-	1637708	1637660
Sma-1-18069	NS_000200.1	-	84248889	84248841
Sma-1-18070	NW_003037863.1	-	520411	520363
Sma-1-18071	NW_003038079.1	+	105320	105368
Sma-1-18072	NS_000200.1	+	268235308	268235356
Sma-1-18073	NS_000200.1	-	107278778	107278730
Sma-1-18074	NW_003037794.1	+	1792448	1792496
Sma-1-18075	NS_000200.1	+	5972043	5972091
Sma-1-18076	NW_003037932.1	+	254082	254130
Sma-1-18077	NS_000200.1	+	177787278	177787326
Sma-1-18078	NW_003038033.1	-	610512	610464
Sma-1-18079	NS_000200.1	-	246590514	246590466
Sma-1-18080	NW_003035557.1	-	1067	1019
Sma-1-18081	NS_000200.1	-	328423226	328423178
Sma-1-18082	NW_003037886.1	+	1071136	1071184
Sma-1-18083	NS_000200.1	+	130326703	130326751
Sma-1-18084	NW_003038033.1	+	858744	858792
Sma-1-18085	NS_000200.1	+	246838746	246838794
Sma-1-18086	NW_003037840.1	+	1566873	1566921
Sma-1-18087	NS_000200.1	+	82190197	82190245
Sma-1-18088	NW_003037945.1	-	403255	403207
Sma-1-18089	NS_000200.1	-	189505219	189505171
Sma-1-18090	NW_003037886.1	-	1010135	1010087
Sma-1-18091	NS_000200.1	-	130265702	130265654
Sma-1-18092	NW_003035866.1	-	33026	32978
Sma-1-18093	NS_000200.1	-	334126836	334126788
Sma-1-18094	NW_003037871.1	-	33815	33767
Sma-1-18095	NS_000200.1	-	117481553	117481505
Sma-1-18096	NW_003035879.1	+	195194	195242
Sma-1-18097	NS_000200.1	+	338162521	338162569
Sma-1-18098	NW_003022240.1	-	1052	1004
Sma-1-18099	NW_003037819.1	-	158589	158541
Sma-1-18100	NS_000200.1	-	360996228	360996180
Sma-1-18101	NS_000200.1	-	51246854	51246806
Sma-1-18102	NW_003037819.1	+	1004782	1004830
Sma-1-18103	NS_000200.1	+	52093047	52093095
Sma-1-18104	NW_003037873.1	+	593287	593335
Sma-1-18105	NS_000200.1	+	119332415	119332463
Sma-1-18106	NW_003038378.1	+	15308	15356

Sma-1-18107	NS_000200.1	+	327517493	327517541
Sma-1-18108	NW_003037874.1	+	297188	297236
Sma-1-18109	NS_000200.1	+	119722138	119722186
Sma-1-18110	NW_003037901.1	-	317763	317715
Sma-1-18111	NS_000200.1	-	146166224	146166176
Sma-1-18112	NW_003038000.1	+	52692	52740
Sma-1-18113	NS_000200.1	+	222523470	222523518
Sma-1-18114	NW_003038087.1	-	368833	368785
Sma-1-18115	NS_000200.1	-	273397234	273397186
Sma-1-18116	NW_003037997.1	+	420315	420363
Sma-1-18117	NS_000200.1	+	221231757	221231805
Sma-1-18118	NW_003038487.1	-	57134	57086
Sma-1-18119	NS_000200.1	-	333512921	333512873
Sma-1-18120	NW_003037443.1	-	380	332
Sma-1-18121	NW_003037944.1	-	281583	281535
Sma-1-18122	NS_000200.1	-	369331982	369331934
Sma-1-18123	NS_000200.1	-	188502936	188502888
Sma-1-18124	NW_003037796.1	+	2142889	2142937
Sma-1-18125	NS_000200.1	+	10352533	10352581
Sma-1-18126	NW_003037913.1	+	455701	455749
Sma-1-18127	NS_000200.1	+	159019452	159019500
Sma-1-18128	NW_003038038.1	-	547908	547859
Sma-1-18129	NS_000200.1	-	252451301	252451252
Sma-1-18130	NW_003037809.1	+	1383962	1384010
Sma-1-18131	NS_000200.1	+	35048426	35048474
Sma-1-18132	NW_003037800.1	+	622107	622156
Sma-1-18133	NS_000200.1	+	19251467	19251516
Sma-1-18134	NW_003037792.1	-	138401	138351
Sma-1-18135	NS_000200.1	-	382575745	382575695
Sma-1-18136	NW_003037809.1	-	2450654	2450606
Sma-1-18137	NS_000200.1	-	36115118	36115070
Sma-1-18138	NW_003037805.1	-	400949	400901
Sma-1-18139	NS_000200.1	-	26958793	26958745
Sma-1-18140	NW_003037832.1	+	554480	554526
Sma-1-18141	NS_000200.1	+	71763911	71763957
Sma-1-18142	NW_003026607.1	-	40833	40785
Sma-1-18143	NS_000200.1	-	308073218	308073170
Sma-1-18144	NW_003037942.1	+	235743	235791
Sma-1-18145	NS_000200.1	+	185995651	185995699
Sma-1-18146	NW_003037901.1	+	349808	349856
Sma-1-18147	NS_000200.1	+	146198269	146198317
Sma-1-18148	NW_003037986.1	+	123567	123613
Sma-1-18149	NS_000200.1	+	215540319	215540365
Sma-1-18150	NW_003037809.1	-	2024102	2024054
Sma-1-18151	NS_000200.1	-	35688566	35688518
Sma-1-18152	NW_003037810.1	+	31871	31919
Sma-1-18153	NS_000200.1	+	36636393	36636441
Sma-1-18154	NW_003037915.1	+	271503	271551
Sma-1-18155	NS_000200.1	+	162171489	162171537
Sma-1-18156	NW_003037915.1	+	1187406	1187454
Sma-1-18157	NS_000200.1	+	163087392	163087440
Sma-1-18158	NW_003037813.1	-	888146	888098

Sma-1-18159	NS_000200.1	-	41875009	41874961
Sma-1-18160	NW_003038016.1	-	338198	338150
Sma-1-18161	NS_000200.1	-	231454735	231454687
Sma-1-18162	NW_003037816.1	+	322945	322993
Sma-1-18163	NS_000200.1	+	46531817	46531865
Sma-1-18164	NW_003026507.1	-	56699	56651
Sma-1-18165	NS_000200.1	-	279716850	279716802
Sma-1-18166	NW_003037816.1	+	319720	319768
Sma-1-18167	NS_000200.1	+	46528592	46528640
Sma-1-18168	NW_003037964.1	-	101266	101218
Sma-1-18169	NS_000200.1	-	197702788	197702740
Sma-1-18170	NW_003037995.1	-	20876	20828
Sma-1-18171	NS_000200.1	-	219710644	219710596
Sma-1-18172	NW_003038142.1	-	426518	426470
Sma-1-18173	NS_000200.1	-	287636954	287636906
Sma-1-18174	NW_003037804.1	-	464358	464310
Sma-1-18175	NW_003037885.1	-	395554	395506
Sma-1-18176	NW_003037964.1	+	72141	72189
Sma-1-18177	NW_003037973.1	-	625869	625821
Sma-1-18178	NS_000200.1	+	197673663	197673711
Sma-1-18179	NS_000200.1	-	204607596	204607548
Sma-1-18180	NS_000200.1	-	128493501	128493453
Sma-1-18181	NS_000200.1	-	25604378	25604330
Sma-1-18182	NW_003037820.1	-	685620	685572
Sma-1-18183	NW_003037864.1	+	154042	154090
Sma-1-18184	NW_003038007.1	-	143527	143479
Sma-1-18185	NS_000200.1	+	108646616	108646664
Sma-1-18186	NS_000200.1	-	225392082	225392034
Sma-1-18187	NS_000200.1	-	52958602	52958554
Sma-1-18188	NW_003025257.1	-	797	749
Sma-1-18189	NS_000200.1	-	361496333	361496285
Sma-1-18190	NW_003037917.1	-	1431664	1431616
Sma-1-18191	NS_000200.1	-	166468468	166468420
Sma-1-18192	NW_003037876.1	+	280592	280640
Sma-1-18193	NS_000200.1	+	120826228	120826276
Sma-1-18194	NW_003037803.1	+	462545	462593
Sma-1-18195	NS_000200.1	+	23963082	23963130
Sma-1-18196	NW_003037849.1	+	487755	487803
Sma-1-18197	NS_000200.1	+	91464028	91464076
Sma-1-18198	NW_003037848.1	-	1062113	1062065
Sma-1-18199	NS_000200.1	-	90755184	90755136
Sma-1-18200	NW_003038022.1	+	538735	538783
Sma-1-18201	NS_000200.1	+	236219577	236219625
Sma-1-18202	NW_003037831.1	-	1057305	1057257
Sma-1-18203	NS_000200.1	-	70506607	70506559
Sma-1-18204	NW_003037888.1	-	1420478	1420430
Sma-1-18205	NS_000200.1	-	133628970	133628922
Sma-1-18206	NW_003035282.1	-	834	786
Sma-1-18207	NS_000200.1	-	370943866	370943818
Sma-1-18208	NW_003026481.1	+	32296	32344
Sma-1-18209	NS_000200.1	+	255432280	255432328
Sma-1-18210	NW_003038025.1	+	98060	98110

Sma-1-18211	NS_000200.1	+	238956305	238956355
Sma-1-18212	NW_003038253.1	-	16391	16343
Sma-1-18213	NS_000200.1	-	319334188	319334140
Sma-1-18214	NW_003037804.1	-	1096830	1096782
Sma-1-18215	NW_003038036.1	-	530952	530904
Sma-1-18216	NS_000200.1	-	249464804	249464756
Sma-1-18217	NS_000200.1	-	26236850	26236802
Sma-1-18218	NW_003037854.1	-	387543	387495
Sma-1-18219	NS_000200.1	-	97291964	97291916
Sma-1-18220	NW_003037867.1	+	370057	370105
Sma-1-18221	NW_003026456.1	+	368472	368520
Sma-1-18222	NW_003037836.1	+	738848	738896
Sma-1-18223	NW_003037853.1	+	901121	901169
Sma-1-18224	NS_000200.1	+	58291608	58291656
Sma-1-18225	NS_000200.1	+	76295947	76295995
Sma-1-18226	NS_000200.1	+	96393172	96393220
Sma-1-18227	NS_000200.1	+	115970876	115970924
Sma-1-18228	NW_003038487.1	+	58845	58893
Sma-1-18229	NW_003037796.1	-	316539	316491
Sma-1-18230	NW_003037822.1	+	41805	41853
Sma-1-18231	NW_003037916.1	+	204324	204372
Sma-1-18232	NW_003037976.1	+	136897	136945
Sma-1-18233	NS_000200.1	+	54349811	54349859
Sma-1-18234	NS_000200.1	+	163397735	163397783
Sma-1-18235	NS_000200.1	+	206751212	206751260
Sma-1-18236	NS_000200.1	+	333514632	333514680
Sma-1-18237	NS_000200.1	-	8526183	8526135
Sma-1-18238	NW_003037878.1	+	789837	789885
Sma-1-18239	NW_003038032.1	+	796833	796881
Sma-1-18240	NS_000200.1	+	122843315	122843363
Sma-1-18241	NS_000200.1	+	245955993	245956041
Sma-1-18242	NW_003037881.1	+	639550	639598
Sma-1-18243	NS_000200.1	+	125071341	125071389
Sma-1-18244	NW_003031002.1	+	139	187
Sma-1-18245	NS_000200.1	+	325491373	325491421
Sma-1-18246	NW_003026477.1	+	12415	12463
Sma-1-18247	NW_003030975.1	-	153	105
Sma-1-18248	NW_003034628.1	+	285	333
Sma-1-18249	NW_003035151.1	-	1162	1114
Sma-1-18250	NW_003035675.1	+	9721	9769
Sma-1-18251	NW_003038514.1	+	437566	437614
Sma-1-18252	NW_003038443.1	-	23147	23099
Sma-1-18253	NW_003026605.1	+	99962	100010
Sma-1-18254	NW_003037766.1	+	24963	25011
Sma-1-18255	NW_003037867.1	-	269266	269218
Sma-1-18256	NW_003038460.1	-	25484	25436
Sma-1-18257	NW_003037920.1	+	61422	61470
Sma-1-18258	NW_003038258.1	+	126375	126423
Sma-1-18259	NW_003038258.1	+	166391	166439
Sma-1-18260	NW_003037995.1	+	205091	205139
Sma-1-18261	NW_003038250.1	+	206503	206551
Sma-1-18262	NW_003035876.1	+	126226	126274

Sma-1-18263	NW_003037793.1	-	3375173	3375125
Sma-1-18264	NW_003037796.1	-	778036	777988
Sma-1-18265	NW_003037796.1	-	188785	188737
Sma-1-18266	NW_003037797.1	+	1500922	1500970
Sma-1-18267	NW_003037800.1	-	250663	250615
Sma-1-18268	NW_003037806.1	-	748355	748307
Sma-1-18269	NW_003037814.1	+	765692	765740
Sma-1-18270	NW_003037826.1	+	628530	628578
Sma-1-18271	NW_003037829.1	-	508936	508888
Sma-1-18272	NW_003037838.1	+	301278	301326
Sma-1-18273	NW_003037844.1	-	260236	260188
Sma-1-18274	NW_003037852.1	+	319892	319940
Sma-1-18275	NW_003037854.1	+	743105	743153
Sma-1-18276	NW_003037854.1	+	1486901	1486949
Sma-1-18277	NW_003037866.1	-	524854	524806
Sma-1-18278	NW_003037880.1	+	642097	642145
Sma-1-18279	NW_003037905.1	-	195870	195822
Sma-1-18280	NW_003037914.1	+	1684626	1684674
Sma-1-18281	NW_003037917.1	-	1268354	1268306
Sma-1-18282	NW_003037928.1	-	163973	163925
Sma-1-18283	NW_003037940.1	-	88777	88729
Sma-1-18284	NW_003037964.1	-	455812	455764
Sma-1-18285	NW_003037969.1	-	262509	262461
Sma-1-18286	NW_003037972.1	+	680571	680619
Sma-1-18287	NW_003037978.1	-	329189	329141
Sma-1-18288	NW_003037994.1	-	402050	402002
Sma-1-18289	NW_003038017.1	-	543032	542984
Sma-1-18290	NW_003038028.1	+	677708	677756
Sma-1-18291	NW_003026515.1	-	197836	197788
Sma-1-18292	NW_003026517.1	+	293638	293686
Sma-1-18293	NW_003026517.1	+	459733	459781
Sma-1-18294	NW_003038161.1	+	204256	204304
Sma-1-18295	NS_000200.1	+	11928782	11928830
Sma-1-18296	NS_000200.1	+	43749552	43749600
Sma-1-18297	NS_000200.1	+	61628046	61628094
Sma-1-18298	NS_000200.1	+	78299841	78299889
Sma-1-18299	NS_000200.1	+	94711787	94711835
Sma-1-18300	NS_000200.1	+	97647526	97647574
Sma-1-18301	NS_000200.1	+	98391322	98391370
Sma-1-18302	NS_000200.1	+	124325655	124325703
Sma-1-18303	NS_000200.1	+	161828876	161828924
Sma-1-18304	NS_000200.1	+	169242229	169242277
Sma-1-18305	NS_000200.1	+	203156275	203156323
Sma-1-18306	NS_000200.1	+	219894859	219894907
Sma-1-18307	NS_000200.1	+	241985282	241985330
Sma-1-18308	NS_000200.1	+	253889824	253889872
Sma-1-18309	NS_000200.1	+	297574107	297574155
Sma-1-18310	NS_000200.1	+	299899647	299899695
Sma-1-18311	NS_000200.1	+	300065742	300065790
Sma-1-18312	NS_000200.1	+	307778826	307778874
Sma-1-18313	NS_000200.1	+	318259180	318259228
Sma-1-18314	NS_000200.1	+	322664885	322664933

Sma-1-18315	NS_000200.1	+	322704901	322704949
Sma-1-18316	NS_000200.1	+	327969698	327969746
Sma-1-18317	NS_000200.1	+	331787066	331787114
Sma-1-18318	NS_000200.1	+	336957726	336957774
Sma-1-18319	NS_000200.1	+	340062105	340062153
Sma-1-18320	NS_000200.1	+	377996361	377996409
Sma-1-18321	NS_000200.1	-	370693337	370693289
Sma-1-18322	NS_000200.1	-	370640155	370640107
Sma-1-18323	NS_000200.1	-	331327376	331327328
Sma-1-18324	NS_000200.1	-	330052793	330052745
Sma-1-18325	NS_000200.1	-	290842247	290842199
Sma-1-18326	NS_000200.1	-	232309063	232309015
Sma-1-18327	NS_000200.1	-	218933864	218933816
Sma-1-18328	NS_000200.1	-	209597199	209597151
Sma-1-18329	NS_000200.1	-	200868636	200868588
Sma-1-18330	NS_000200.1	-	198057334	198057286
Sma-1-18331	NS_000200.1	-	183830907	183830859
Sma-1-18332	NS_000200.1	-	174487926	174487878
Sma-1-18333	NS_000200.1	-	166305158	166305110
Sma-1-18334	NS_000200.1	-	149464882	149464834
Sma-1-18335	NS_000200.1	-	115870085	115870037
Sma-1-18336	NS_000200.1	-	112012653	112012605
Sma-1-18337	NS_000200.1	-	86443682	86443634
Sma-1-18338	NS_000200.1	-	66409474	66409426
Sma-1-18339	NS_000200.1	-	28943569	28943521
Sma-1-18340	NS_000200.1	-	18880023	18879975
Sma-1-18341	NS_000200.1	-	8987680	8987632
Sma-1-18342	NS_000200.1	-	8398429	8398381
Sma-1-18343	NS_000200.1	-	3375173	3375125
Sma-1-18344	NW_003037806.1	+	230115	230162
Sma-1-18345	NS_000200.1	+	28425329	28425376
Sma-1-18346	NW_003037908.1	+	922692	922740
Sma-1-18347	NW_003038106.1	+	45058	45106
Sma-1-18348	NS_000200.1	+	153395823	153395871
Sma-1-18349	NS_000200.1	+	275233823	275233871
Sma-1-18350	NW_003037880.1	-	642020	641972
Sma-1-18351	NS_000200.1	-	124325578	124325530
Sma-1-18352	NW_003038055.1	+	224798	224846
Sma-1-18353	NS_000200.1	+	258924120	258924168
Sma-1-18354	NW_003037873.1	+	410133	410181
Sma-1-18355	NS_000200.1	+	119149261	119149309
Sma-1-18356	NW_003037854.1	-	1865178	1865130
Sma-1-18357	NS_000200.1	-	98769599	98769551
Sma-1-18358	NW_003038118.1	+	277955	278003
Sma-1-18359	NS_000200.1	+	277842082	277842130
Sma-1-18360	NW_003026618.1	-	7016	6968
Sma-1-18361	NS_000200.1	-	321762018	321761970
Sma-1-18362	NW_003038006.1	+	54736	54784
Sma-1-18363	NW_003037975.1	-	407380	407332
Sma-1-18364	NS_000200.1	+	224939220	224939268
Sma-1-18365	NS_000200.1	-	205989604	205989556
Sma-1-18366	NW_003037854.1	-	30670	30622

Sma-1-18367	NS_000200.1	-	96935091	96935043
Sma-1-18368	NW_003036058.1	+	124	172
Sma-1-18369	NS_000200.1	+	343369538	343369586
Sma-1-18370	NW_003037794.1	-	1887346	1887298
Sma-1-18371	NS_000200.1	-	6066941	6066893
Sma-1-18372	NW_003038132.1	-	310660	310612
Sma-1-18373	NS_000200.1	-	282731130	282731082
Sma-1-18374	NW_003026462.1	-	497771	497723
Sma-1-18375	NS_000200.1	-	197165274	197165226
Sma-1-18376	NW_003037841.1	+	231003	231051
Sma-1-18377	NS_000200.1	+	82842184	82842232
Sma-1-18378	NW_003037863.1	+	1006616	1006664
Sma-1-18379	NW_003038008.1	+	193208	193256
Sma-1-18380	NS_000200.1	+	107764983	107765031
Sma-1-18381	NS_000200.1	+	225866304	225866352
Sma-1-18382	NW_003020781.1	+	207	255
Sma-1-18383	NS_000200.1	+	364445682	364445730
Sma-1-18384	NW_003026612.1	-	160478	160430
Sma-1-18385	NS_000200.1	-	312465865	312465817
Sma-1-18386	NW_003037900.1	+	147539	147587
Sma-1-18387	NS_000200.1	+	145276405	145276453
Sma-1-18388	NW_003037825.1	-	1767093	1767045
Sma-1-18389	NW_003037826.1	-	4506	4458
Sma-1-18390	NW_003037885.1	-	724769	724721
Sma-1-18391	NW_003037907.1	+	781032	781080
Sma-1-18392	NS_000200.1	+	152185521	152185569
Sma-1-18393	NS_000200.1	-	128822716	128822668
Sma-1-18394	NS_000200.1	-	61004022	61003974
Sma-1-18395	NS_000200.1	-	60896875	60896827
Sma-1-18396	NW_003037787.1	+	75974	76022
Sma-1-18397	NS_000200.1	+	380324143	380324191
Sma-1-18398	NW_003037939.1	-	389904	389856
Sma-1-18399	NS_000200.1	-	183259658	183259610
Sma-1-18400	NW_003037793.1	+	2976887	2976935
Sma-1-18401	NS_000200.1	+	2976887	2976935
Sma-1-18402	NW_003037793.1	+	2980103	2980151
Sma-1-18403	NS_000200.1	+	2980103	2980151
Sma-1-18404	NW_003037973.1	-	656937	656889
Sma-1-18405	NS_000200.1	-	204638664	204638616
Sma-1-18406	NW_003037973.1	+	438412	438460
Sma-1-18407	NW_003038011.1	+	580922	580970
Sma-1-18408	NS_000200.1	+	204420139	204420187
Sma-1-18409	NS_000200.1	+	228108572	228108620
Sma-1-18410	NW_003037797.1	+	740032	740080
Sma-1-18411	NS_000200.1	+	11167892	11167940
Sma-1-18412	NW_003037914.1	+	1672937	1672985
Sma-1-18413	NS_000200.1	+	161817187	161817235
Sma-1-18414	NW_003037854.1	+	1472754	1472802
Sma-1-18415	NS_000200.1	+	98377175	98377223
Sma-1-18416	NW_003038249.1	+	299706	299754
Sma-1-18417	NS_000200.1	+	316981959	316982007
Sma-1-18418	NW_003037887.1	-	258692	258644

Sma-1-18419	NS_000200.1	-	130722670	130722622
Sma-1-18420	NW_003037917.1	-	487774	487726
Sma-1-18421	NS_000200.1	-	165524578	165524530
Sma-1-18422	NW_003037816.1	+	536690	536738
Sma-1-18423	NW_003037838.1	+	357488	357536
Sma-1-18424	NS_000200.1	+	46745562	46745610
Sma-1-18425	NS_000200.1	+	78356051	78356099
Sma-1-18426	NW_003037791.1	-	680698	680650
Sma-1-18427	NS_000200.1	-	382386126	382386078
Sma-1-18428	NW_003037913.1	+	1326770	1326818
Sma-1-18429	NS_000200.1	+	159890521	159890569
Sma-1-18430	NW_003033840.1	-	807	759
Sma-1-18431	NW_003037938.1	+	548338	548386
Sma-1-18432	NS_000200.1	+	182585451	182585499
Sma-1-18433	NS_000200.1	-	359245234	359245186
Sma-1-18434	NW_003038033.1	-	862187	862139
Sma-1-18435	NS_000200.1	-	246842189	246842141
Sma-1-18436	NW_003037932.1	-	874892	874844
Sma-1-18437	NS_000200.1	-	178408088	178408040
Sma-1-18438	NW_003037827.1	-	2259063	2259015
Sma-1-18439	NS_000200.1	-	64881204	64881156
Sma-1-18440	NW_003037825.1	-	1210388	1210340
Sma-1-18441	NW_003037909.1	+	297722	297770
Sma-1-18442	NS_000200.1	+	153839371	153839419
Sma-1-18443	NS_000200.1	-	60340170	60340122
Sma-1-18444	NW_003037976.1	+	54347	54395
Sma-1-18445	NS_000200.1	+	206668662	206668710
Sma-1-18446	NW_003037882.1	+	405708	405756
Sma-1-18447	NS_000200.1	+	125697382	125697430
Sma-1-18448	NW_003033883.1	+	626	674
Sma-1-18449	NS_000200.1	+	359388340	359388388
Sma-1-18450	NW_003037835.1	-	1150569	1150521
Sma-1-18451	NS_000200.1	-	75547077	75547029
Sma-1-18452	NW_003022453.1	+	1062	1110
Sma-1-18453	NW_003037812.1	+	1103635	1103683
Sma-1-18454	NW_003037852.1	-	753660	753612
Sma-1-18455	NS_000200.1	+	40586374	40586422
Sma-1-18456	NS_000200.1	+	364970827	364970875
Sma-1-18457	NS_000200.1	-	95145555	95145507
Sma-1-18458	NW_003037918.1	-	1852154	1852106
Sma-1-18459	NS_000200.1	-	168419144	168419096
Sma-1-18460	NW_003037905.1	+	342022	342070
Sma-1-18461	NS_000200.1	+	149611034	149611082
Sma-1-18462	NW_003038515.1	+	223798	223846
Sma-1-18463	NS_000200.1	+	340307863	340307911
Sma-1-18464	NW_003037874.1	+	260913	260961
Sma-1-18465	NW_003037797.1	-	1864035	1863987
Sma-1-18466	NS_000200.1	+	119685863	119685911
Sma-1-18467	NS_000200.1	-	12291895	12291847
Sma-1-18468	NW_003038260.1	-	2040	1992
Sma-1-18469	NS_000200.1	-	323173815	323173767
Sma-1-18470	NW_003038497.1	-	4218	4170

Sma-1-18471	NS_000200.1	-	334389076	334389028
Sma-1-18472	NW_003039130.1	-	13837	13789
Sma-1-18473	NS_000200.1	-	317688448	317688400
Sma-1-18474	NW_003026464.1	-	556883	556835
Sma-1-18475	NS_000200.1	-	203857192	203857144
Sma-1-18476	NW_003037790.1	-	198656	198608
Sma-1-18477	NS_000200.1	-	381542828	381542780
Sma-1-18478	NW_003037827.1	+	394348	394396
Sma-1-18479	NW_003037827.1	+	1577892	1577940
Sma-1-18480	NW_003037850.1	+	797513	797561
Sma-1-18481	NS_000200.1	+	63016489	63016537
Sma-1-18482	NS_000200.1	+	64200033	64200081
Sma-1-18483	NS_000200.1	+	92783543	92783591
Sma-1-18484	NW_003037833.1	+	167586	167634
Sma-1-18485	NW_003037860.1	-	568720	568672
Sma-1-18486	NS_000200.1	+	72358682	72358730
Sma-1-18487	NS_000200.1	-	104311748	104311700
Sma-1-18488	NW_003035880.1	+	270143	270191
Sma-1-18489	NS_000200.1	+	339511207	339511255
Sma-1-18490	NW_003037794.1	+	1352061	1352107
Sma-1-18491	NS_000200.1	+	5531656	5531702
Sma-1-18492	NW_003038137.1	-	133749	133701
Sma-1-18493	NS_000200.1	-	284922645	284922597
Sma-1-18494	NW_003038027.1	+	673224	673272
Sma-1-18495	NS_000200.1	+	241043938	241043986
Sma-1-18496	NW_003037962.1	+	286864	286912
Sma-1-18497	NS_000200.1	+	196363707	196363755
Sma-1-18498	NW_003037873.1	+	30934	30982
Sma-1-18499	NW_003037795.1	-	1340980	1340932
Sma-1-18500	NW_003038239.1	+	241112	241160
Sma-1-18501	NS_000200.1	+	118770062	118770110
Sma-1-18502	NS_000200.1	+	314081894	314081942
Sma-1-18503	NS_000200.1	-	7731863	7731815
Sma-1-18504	NW_003038028.1	+	95312	95360
Sma-1-18505	NS_000200.1	+	241402886	241402934
Sma-1-18506	NW_003038041.1	-	170849	170801
Sma-1-18507	NS_000200.1	-	253838131	253838083
Sma-1-18508	NW_003028347.1	-	393	345
Sma-1-18509	NW_003035872.1	+	34546	34594
Sma-1-18510	NW_003035880.1	+	57047	57095
Sma-1-18511	NW_003038521.1	+	5434	5482
Sma-1-18512	NW_003037774.1	+	42345	42393
Sma-1-18513	NW_003037929.1	-	547891	547843
Sma-1-18514	NW_003026615.1	-	42977	42929
Sma-1-18515	NW_003032160.1	+	64924	64972
Sma-1-18516	NW_003037797.1	-	2556169	2556121
Sma-1-18517	NW_003037809.1	-	2187304	2187256
Sma-1-18518	NW_003037811.1	+	748280	748328
Sma-1-18519	NW_003037812.1	-	454830	454782
Sma-1-18520	NW_003037819.1	-	1167445	1167397
Sma-1-18521	NW_003037825.1	+	1463835	1463883
Sma-1-18522	NW_003037825.1	+	1486979	1487027

Sma-1-18523	NW_003037830.1	+	43619	43667
Sma-1-18524	NW_003037830.1	-	1548970	1548922
Sma-1-18525	NW_003037830.1	-	42478	42430
Sma-1-18526	NW_003037845.1	+	585685	585733
Sma-1-18527	NW_003037854.1	+	707301	707349
Sma-1-18528	NW_003037904.1	-	214638	214590
Sma-1-18529	NW_003037933.1	+	447436	447484
Sma-1-18530	NW_003038019.1	-	226071	226023
Sma-1-18531	NS_000200.1	+	38918522	38918570
Sma-1-18532	NS_000200.1	+	60593617	60593665
Sma-1-18533	NS_000200.1	+	60616761	60616809
Sma-1-18534	NS_000200.1	+	67933990	67934038
Sma-1-18535	NS_000200.1	+	87587588	87587636
Sma-1-18536	NS_000200.1	+	97611722	97611770
Sma-1-18537	NS_000200.1	+	178860608	178860656
Sma-1-18538	NS_000200.1	+	335656293	335656341
Sma-1-18539	NS_000200.1	+	339298111	339298159
Sma-1-18540	NS_000200.1	+	342873181	342873229
Sma-1-18541	NS_000200.1	+	378627722	378627770
Sma-1-18542	NS_000200.1	+	381073010	381073058
Sma-1-18543	NS_000200.1	-	369423293	369423245
Sma-1-18544	NS_000200.1	-	317037418	317037370
Sma-1-18545	NS_000200.1	-	233542889	233542841
Sma-1-18546	NS_000200.1	-	175788669	175788621
Sma-1-18547	NS_000200.1	-	148929137	148929089
Sma-1-18548	NS_000200.1	-	69439341	69439293
Sma-1-18549	NS_000200.1	-	67932849	67932801
Sma-1-18550	NS_000200.1	-	52255710	52255662
Sma-1-18551	NS_000200.1	-	39937569	39937521
Sma-1-18552	NS_000200.1	-	35851768	35851720
Sma-1-18553	NS_000200.1	-	12984029	12983981
Sma-1-18554	NW_003037851.1	-	613878	613830
Sma-1-18555	NS_000200.1	-	94017188	94017140
Sma-1-18556	NW_003038037.1	-	837776	837728
Sma-1-18557	NS_000200.1	-	250993185	250993137
Sma-1-18558	NW_003037924.1	-	135229	135181
Sma-1-18559	NS_000200.1	-	171842187	171842139
Sma-1-18560	NW_003037964.1	+	192665	192713
Sma-1-18561	NS_000200.1	+	197794187	197794235
Sma-1-18562	NW_003037831.1	-	306039	305991
Sma-1-18563	NS_000200.1	-	69755341	69755293
Sma-1-18564	NW_003038442.1	+	47115	47166
Sma-1-18565	NS_000200.1	+	329903168	329903219
Sma-1-18566	NW_003037981.1	+	944254	944302
Sma-1-18567	NS_000200.1	+	213342122	213342170
Sma-1-18568	NW_003025155.1	+	753	801
Sma-1-18569	NW_003037866.1	-	1047221	1047173
Sma-1-18570	NW_003037918.1	+	610956	611004
Sma-1-18571	NW_003037933.1	-	602274	602226
Sma-1-18572	NS_000200.1	+	167177946	167177994
Sma-1-18573	NS_000200.1	+	361281888	361281936
Sma-1-18574	NS_000200.1	-	179015446	179015398

Sma-1-18575	NS_000200.1	-	112535020	112534972
Sma-1-18576	NW_003038042.1	+	195095	195143
Sma-1-18577	NS_000200.1	+	254140694	254140742
Sma-1-18578	NW_003037933.1	-	155166	155118
Sma-1-18579	NS_000200.1	-	178568338	178568290
Sma-1-18580	NW_003037935.1	-	183833	183785
Sma-1-18581	NS_000200.1	-	179600368	179600320
Sma-1-18582	NW_003026471.1	+	173441	173489
Sma-1-18583	NS_000200.1	+	222285674	222285722
Sma-1-18584	NW_003037886.1	+	1043422	1043470
Sma-1-18585	NS_000200.1	+	130298989	130299037
Sma-1-18586	NW_003038141.1	-	239103	239052
Sma-1-18587	NS_000200.1	-	287129082	287129031
Sma-1-18588	NW_003037834.1	-	864038	863990
Sma-1-18589	NS_000200.1	-	74024524	74024476
Sma-1-18590	NW_003038157.1	+	502219	502267
Sma-1-18591	NS_000200.1	+	295753139	295753187
Sma-1-18592	NW_003039127.1	-	433590	433542
Sma-1-18593	NS_000200.1	-	113511089	113511041
Sma-1-18594	NW_003037854.1	-	401791	401743
Sma-1-18595	NS_000200.1	-	97306212	97306164
Sma-1-18596	NW_003038025.1	+	67332	67380
Sma-1-18597	NS_000200.1	+	238925577	238925625
Sma-1-18598	NW_003038255.1	-	29442	29394
Sma-1-18599	NS_000200.1	-	320418587	320418539
Sma-1-18600	NW_003037793.1	+	2895297	2895345
Sma-1-18601	NW_003037854.1	+	761075	761123
Sma-1-18602	NS_000200.1	+	2895297	2895345
Sma-1-18603	NS_000200.1	+	97665496	97665544
Sma-1-18604	NW_003026471.1	+	129107	129155
Sma-1-18605	NS_000200.1	+	222241340	222241388
Sma-1-18606	NW_003037796.1	-	1027354	1027306
Sma-1-18607	NS_000200.1	-	9236998	9236950
Sma-1-18608	NW_003037796.1	+	1100359	1100407
Sma-1-18609	NS_000200.1	+	9310003	9310051
Sma-1-18610	NW_003037801.1	-	499670	499622
Sma-1-18611	NW_003037942.1	-	660404	660356
Sma-1-18612	NS_000200.1	-	186420312	186420264
Sma-1-18613	NS_000200.1	-	21589150	21589102
Sma-1-18614	NW_003037819.1	+	929924	929972
Sma-1-18615	NS_000200.1	+	52018189	52018237
Sma-1-18616	NW_003037804.1	+	1195397	1195445
Sma-1-18617	NS_000200.1	+	26335417	26335465
Sma-1-18618	NW_003037804.1	-	785974	785926
Sma-1-18619	NW_003037973.1	+	317542	317590
Sma-1-18620	NS_000200.1	+	204299269	204299317
Sma-1-18621	NS_000200.1	-	25925994	25925946
Sma-1-18622	NW_003037808.1	-	281288	281240
Sma-1-18623	NS_000200.1	-	32067925	32067877
Sma-1-18624	NW_003037797.1	+	3224504	3224552
Sma-1-18625	NS_000200.1	+	13652364	13652412
Sma-1-18626	NW_003037797.1	+	1876397	1876445

Sma-1-18627	NS_000200.1	+	12304257	12304305
Sma-1-18628	NW_003037793.1	-	242463	242415
Sma-1-18629	NS_000200.1	-	242463	242415
Sma-1-18630	NW_003038133.1	+	244663	244711
Sma-1-18631	NS_000200.1	+	283116845	283116893
Sma-1-18632	NW_003038013.1	-	74285	74237
Sma-1-18633	NS_000200.1	-	229463911	229463863
Sma-1-18634	NW_003038192.1	-	64209	64161
Sma-1-18635	NS_000200.1	-	302626116	302626068
Sma-1-18636	NW_003037975.1	-	619243	619195
Sma-1-18637	NS_000200.1	-	206201467	206201419
Sma-1-18638	NW_003037806.1	-	257704	257656
Sma-1-18639	NS_000200.1	-	28452918	28452870
Sma-1-18640	NW_003037917.1	-	1501950	1501902
Sma-1-18641	NS_000200.1	-	166538754	166538706
Sma-1-18642	NW_003037793.1	+	2066624	2066672
Sma-1-18643	NS_000200.1	+	2066624	2066672
Sma-1-18644	NW_003037874.1	-	427384	427336
Sma-1-18645	NS_000200.1	-	119852334	119852286
Sma-1-18646	NW_003037798.1	+	308979	309027
Sma-1-18647	NS_000200.1	+	14568137	14568185
Sma-1-18648	NW_003037924.1	+	606409	606457
Sma-1-18649	NS_000200.1	+	172313367	172313415
Sma-1-18650	NW_003037852.1	-	660030	659982
Sma-1-18651	NS_000200.1	-	95051925	95051877
Sma-1-18652	NW_003038059.1	+	307570	307618
Sma-1-18653	NS_000200.1	+	260549642	260549690
Sma-1-18654	NW_003037827.1	+	302690	302738
Sma-1-18655	NS_000200.1	+	62924831	62924879
Sma-1-18656	NW_003038511.1	+	45606	45654
Sma-1-18657	NS_000200.1	+	338390818	338390866
Sma-1-18658	NW_003037852.1	-	659395	659347
Sma-1-18659	NS_000200.1	-	95051290	95051242
Sma-1-18660	NW_003037796.1	+	344328	344376
Sma-1-18661	NS_000200.1	+	8553972	8554020
Sma-1-18662	NW_003037936.1	+	544315	544363
Sma-1-18663	NS_000200.1	+	180984044	180984092
Sma-1-18664	NW_003037933.1	-	47050	47002
Sma-1-18665	NS_000200.1	-	178460222	178460174
Sma-1-18666	NW_003038251.1	+	404809	404857
Sma-1-18667	NS_000200.1	+	318736581	318736629
Sma-1-18668	NW_003032075.1	-	7064	7016
Sma-1-18669	NS_000200.1	-	377096660	377096612
Sma-1-18670	NW_003038016.1	-	128700	128652
Sma-1-18671	NS_000200.1	-	231245237	231245189
Sma-1-18672	NW_003038115.1	-	102173	102125
Sma-1-18673	NS_000200.1	-	277157468	277157420
Sma-1-18674	NW_003037975.1	-	506325	506277
Sma-1-18675	NS_000200.1	-	206088549	206088501
Sma-1-18676	NW_003038063.1	+	112059	112107
Sma-1-18677	NS_000200.1	+	261608337	261608385
Sma-1-18678	NW_003026591.1	-	23869	23821

Sma-1-18679	NS_000200.1	-	303972697	303972649
Sma-1-18680	NW_003038131.1	-	17141	17093
Sma-1-18681	NS_000200.1	-	281978985	281978937
Sma-1-18682	NW_003037795.1	+	220062	220110
Sma-1-18683	NS_000200.1	+	6610945	6610993
Sma-1-18684	NW_003037816.1	+	204582	204630
Sma-1-18685	NS_000200.1	+	46413454	46413502
Sma-1-18686	NW_003037857.1	+	353778	353826
Sma-1-18687	NS_000200.1	+	100374135	100374183
Sma-1-18688	NW_003037853.1	+	143611	143659
Sma-1-18689	NS_000200.1	+	95635662	95635710
Sma-1-18690	NW_003037862.1	+	581651	581699
Sma-1-18691	NS_000200.1	+	106233312	106233360
Sma-1-18692	NW_003037805.1	-	1360415	1360367
Sma-1-18693	NS_000200.1	-	27918259	27918211
Sma-1-18694	NW_003037805.1	-	1362243	1362195
Sma-1-18695	NS_000200.1	-	27920087	27920039
Sma-1-18696	NW_003038011.1	+	13905	13953
Sma-1-18697	NS_000200.1	+	227541555	227541603
Sma-1-18698	NW_003037853.1	-	174834	174786
Sma-1-18699	NS_000200.1	-	95666885	95666837
Sma-1-18700	NW_003037975.1	+	1006552	1006600
Sma-1-18701	NS_000200.1	+	206588776	206588824
Sma-1-18702	NW_003035361.1	-	467	419
Sma-1-18703	NW_003038137.1	-	234535	234487
Sma-1-18704	NS_000200.1	-	367338375	367338327
Sma-1-18705	NS_000200.1	-	285023431	285023383
Sma-1-18706	NW_003039127.1	+	2510113	2510161
Sma-1-18707	NS_000200.1	+	115587612	115587660
Sma-1-18708	NW_003037825.1	-	450261	450213
Sma-1-18709	NS_000200.1	-	59580043	59579995
Sma-1-18710	NW_003037971.1	-	488443	488395
Sma-1-18711	NS_000200.1	-	202110493	202110445
Sma-1-18712	NW_003037847.1	+	986091	986139
Sma-1-18713	NS_000200.1	+	89637821	89637869
Sma-1-18714	NW_003026516.1	+	162760	162808
Sma-1-18715	NS_000200.1	+	299271057	299271105
Sma-1-18716	NW_003037853.1	-	407033	406985
Sma-1-18717	NS_000200.1	-	95899084	95899036
Sma-1-18718	NW_003038188.1	+	74555	74603
Sma-1-18719	NS_000200.1	+	302168298	302168346
Sma-1-18720	NW_003037852.1	-	229294	229246
Sma-1-18721	NS_000200.1	-	94621189	94621141
Sma-1-18722	NW_003037933.1	-	291388	291340
Sma-1-18723	NS_000200.1	-	178704560	178704512
Sma-1-18724	NW_003037890.1	+	1335551	1335599
Sma-1-18725	NS_000200.1	+	137018912	137018960
Sma-1-18726	NW_003037884.1	-	533197	533149
Sma-1-18727	NS_000200.1	-	127312862	127312814
Sma-1-18728	NW_003037910.1	+	1128092	1128140
Sma-1-18729	NS_000200.1	+	155640067	155640115
Sma-1-18730	NW_003037793.1	+	3784362	3784410

Sma-1-18731	NS_000200.1	+	3784362	3784410
Sma-1-18732	NW_003038007.1	+	153026	153074
Sma-1-18733	NS_000200.1	+	225401581	225401629
Sma-1-18734	NW_003037837.1	+	287844	287892
Sma-1-18735	NW_003038047.1	+	92098	92146
Sma-1-18736	NS_000200.1	+	77093702	77093750
Sma-1-18737	NS_000200.1	+	255342097	255342145
Sma-1-18738	NW_003038144.1	-	109989	109941
Sma-1-18739	NS_000200.1	-	288374365	288374317
Sma-1-18740	NW_003038065.1	+	277227	277275
Sma-1-18741	NS_000200.1	+	262428844	262428892
Sma-1-18742	NW_003026607.1	+	124748	124796
Sma-1-18743	NS_000200.1	+	308157133	308157181
Sma-1-18744	NW_003038200.1	-	10120	10072
Sma-1-18745	NS_000200.1	-	303609414	303609366
Sma-1-18746	NW_003037813.1	+	1181778	1181826
Sma-1-18747	NS_000200.1	+	42168641	42168689
Sma-1-18748	NW_003037827.1	-	2252123	2252075
Sma-1-18749	NS_000200.1	-	64874264	64874216
Sma-1-18750	NW_003037975.1	-	997171	997123
Sma-1-18751	NS_000200.1	-	206579395	206579347
Sma-1-18752	NW_003037827.1	-	2199024	2198976
Sma-1-18753	NS_000200.1	-	64821165	64821117
Sma-1-18754	NW_003037827.1	+	192584	192632
Sma-1-18755	NS_000200.1	+	62814725	62814773
Sma-1-18756	NW_003037853.1	-	710150	710102
Sma-1-18757	NS_000200.1	-	96202201	96202153
Sma-1-18758	NW_003037827.1	+	1713119	1713167
Sma-1-18759	NS_000200.1	+	64335260	64335308
Sma-1-18760	NW_003037854.1	-	1472137	1472089
Sma-1-18761	NS_000200.1	-	98376558	98376510
Sma-1-18762	NW_003038014.1	-	369951	369903
Sma-1-18763	NS_000200.1	-	230110640	230110592
Sma-1-18764	NW_003037795.1	-	1333130	1333082
Sma-1-18765	NS_000200.1	-	7724013	7723965
Sma-1-18766	NW_003037827.1	-	1471907	1471859
Sma-1-18767	NS_000200.1	-	64094048	64094000
Sma-1-18768	NW_003037915.1	-	1267414	1267366
Sma-1-18769	NS_000200.1	-	163167400	163167352
Sma-1-18770	NW_003038049.1	-	243396	243348
Sma-1-18771	NS_000200.1	-	256218923	256218875
Sma-1-18772	NW_003034442.1	-	1499	1451
Sma-1-18773	NS_000200.1	-	346732099	346732051
Sma-1-18774	NW_003037937.1	-	721569	721521
Sma-1-18775	NS_000200.1	-	181921478	181921430
Sma-1-18776	NW_003038067.1	-	90939	90891
Sma-1-18777	NS_000200.1	-	262944992	262944944
Sma-1-18778	NW_003038241.1	-	186494	186446
Sma-1-18779	NS_000200.1	-	314649199	314649151
Sma-1-18780	NW_003021345.1	+	2620	2668
Sma-1-18781	NS_000200.1	+	343904618	343904666
Sma-1-18782	NW_003037937.1	+	666310	666358

Sma-1-18783	NS_000200.1	+	181866219	181866267
Sma-1-18784	NW_003037809.1	-	2164321	2164273
Sma-1-18785	NW_003037862.1	+	2649	2697
Sma-1-18786	NS_000200.1	+	105654310	105654358
Sma-1-18787	NS_000200.1	-	35828785	35828737
Sma-1-18788	NW_003037795.1	+	1131624	1131672
Sma-1-18789	NS_000200.1	+	7522507	7522555
Sma-1-18790	NW_003037859.1	+	767441	767489
Sma-1-18791	NS_000200.1	+	103446282	103446330
Sma-1-18792	NW_003037982.1	+	1326496	1326544
Sma-1-18793	NS_000200.1	+	214985691	214985739
Sma-1-18794	NW_003037807.1	-	616044	615996
Sma-1-18795	NS_000200.1	-	30877560	30877512
Sma-1-18796	NW_003026609.1	-	48141	48093
Sma-1-18797	NS_000200.1	-	309703133	309703085
Sma-1-18798	NW_003038015.1	-	715276	715228
Sma-1-18799	NS_000200.1	-	231038766	231038718
Sma-1-18800	NW_003037884.1	-	894284	894236
Sma-1-18801	NW_003026575.1	+	82841	82889
Sma-1-18802	NS_000200.1	+	302314507	302314555
Sma-1-18803	NS_000200.1	-	127673949	127673901
Sma-1-18804	NW_003038152.1	+	345878	345926
Sma-1-18805	NS_000200.1	+	292951602	292951650
Sma-1-18806	NW_003037909.1	+	238837	238885
Sma-1-18807	NS_000200.1	+	153780486	153780534
Sma-1-18808	NW_003037871.1	+	405778	405826
Sma-1-18809	NS_000200.1	+	117853516	117853564
Sma-1-18810	NW_003037967.1	-	589802	589754
Sma-1-18811	NS_000200.1	-	199829773	199829725
Sma-1-18812	NW_003037890.1	-	823811	823763
Sma-1-18813	NS_000200.1	-	136507172	136507124
Sma-1-18814	NW_003037908.1	-	702618	702570
Sma-1-18815	NS_000200.1	-	153175749	153175701
Sma-1-18816	NW_003038135.1	+	235262	235310
Sma-1-18817	NS_000200.1	+	284107601	284107649
Sma-1-18818	NW_003037972.1	+	249319	249367
Sma-1-18819	NS_000200.1	+	202725023	202725071
Sma-1-18820	NW_003037890.1	+	975245	975293
Sma-1-18821	NS_000200.1	+	136658606	136658654
Sma-1-18822	NW_003026610.1	-	52593	52545
Sma-1-18823	NS_000200.1	-	310207963	310207915
Sma-1-18824	NW_003037794.1	+	1967421	1967469
Sma-1-18825	NS_000200.1	+	6147016	6147064
Sma-1-18826	NW_003038022.1	-	43880	43832
Sma-1-18827	NS_000200.1	-	235724722	235724674
Sma-1-18828	NW_003038161.1	+	45016	45064
Sma-1-18829	NS_000200.1	+	297414867	297414915
Sma-1-18830	NW_003038017.1	+	459375	459423
Sma-1-18831	NS_000200.1	+	232225406	232225454
Sma-1-18832	NW_003037916.1	-	328577	328529
Sma-1-18833	NS_000200.1	-	163521988	163521940
Sma-1-18834	NW_003037830.1	+	72267	72315

Sma-1-18835	NS_000200.1	+	67962638	67962686
Sma-1-18836	NW_003037796.1	-	1360075	1360027
Sma-1-18837	NS_000200.1	-	9569719	9569671
Sma-1-18838	NW_003026611.1	+	148669	148717
Sma-1-18839	NS_000200.1	+	310718453	310718501
Sma-1-18840	NW_003037797.1	+	1318573	1318621
Sma-1-18841	NW_003037804.1	+	855633	855681
Sma-1-18842	NS_000200.1	+	11746433	11746481
Sma-1-18843	NS_000200.1	+	25995653	25995701
Sma-1-18844	NW_003026611.1	+	171580	171628
Sma-1-18845	NS_000200.1	+	310741364	310741412
Sma-1-18846	NW_003037793.1	+	4106378	4106426
Sma-1-18847	NS_000200.1	+	4106378	4106426
Sma-1-18848	NW_003038216.1	-	89765	89717
Sma-1-18849	NS_000200.1	-	306875397	306875349
Sma-1-18850	NW_003026611.1	-	95630	95582
Sma-1-18851	NS_000200.1	-	310665414	310665366
Sma-1-18852	NW_003038068.1	-	246817	246769
Sma-1-18853	NS_000200.1	-	263314025	263313977
Sma-1-18854	NW_003037907.1	-	793678	793630
Sma-1-18855	NS_000200.1	-	152198167	152198119
Sma-1-18856	NW_003038018.1	-	910090	910042
Sma-1-18857	NS_000200.1	-	233310940	233310892
Sma-1-18858	NW_003038019.1	+	106800	106848
Sma-1-18859	NS_000200.1	+	233423618	233423666
Sma-1-18860	NW_003037793.1	+	3042547	3042595
Sma-1-18861	NS_000200.1	+	3042547	3042595
Sma-1-18862	NW_003026615.1	+	185393	185441
Sma-1-18863	NW_003026615.1	+	187242	187290
Sma-1-18864	NS_000200.1	+	317179834	317179882
Sma-1-18865	NS_000200.1	+	317181683	317181731
Sma-1-18866	NW_003026617.1	+	290141	290189
Sma-1-18867	NW_003037887.1	+	1080014	1080062
Sma-1-18868	NS_000200.1	+	131543992	131544040
Sma-1-18869	NS_000200.1	+	319861000	319861048
Sma-1-18870	NW_003037810.1	-	1427531	1427483
Sma-1-18871	NS_000200.1	-	38032053	38032005
Sma-1-18872	NW_003026490.1	-	112133	112085
Sma-1-18873	NS_000200.1	-	267743849	267743801
Sma-1-18874	NW_003033882.1	+	181	229
Sma-1-18875	NS_000200.1	+	359384764	359384812
Sma-1-18876	NW_003037931.1	+	57181	57229
Sma-1-18877	NS_000200.1	+	176889848	176889896
Sma-1-18878	NW_003037931.1	-	42575	42527
Sma-1-18879	NS_000200.1	-	176875242	176875194
Sma-1-18880	NW_003035880.1	-	100905	100857
Sma-1-18881	NS_000200.1	-	339341969	339341921
Sma-1-18882	NW_003035880.1	-	238230	238182
Sma-1-18883	NS_000200.1	-	339479294	339479246
Sma-1-18884	NW_003037797.1	+	1494850	1494898
Sma-1-18885	NS_000200.1	+	11922710	11922758
Sma-1-18886	NW_003026460.1	-	276996	276948

Sma-1-18887	NW_003037861.1	+	779471	779519
Sma-1-18888	NW_003037896.1	+	188769	188817
Sma-1-18889	NW_003038034.1	+	306293	306341
Sma-1-18890	NS_000200.1	+	105555925	105555973
Sma-1-18891	NS_000200.1	+	143109192	143109240
Sma-1-18892	NS_000200.1	+	247248049	247248097
Sma-1-18893	NS_000200.1	-	170799202	170799154
Sma-1-18894	NW_003029310.1	+	1488	1536
Sma-1-18895	NW_003037891.1	-	2729485	2729437
Sma-1-18896	NS_000200.1	+	357269126	357269174
Sma-1-18897	NS_000200.1	-	140610763	140610715
Sma-1-18898	NW_003037909.1	-	227223	227175
Sma-1-18899	NS_000200.1	-	153768872	153768824
Sma-1-18900	NW_003037935.1	+	89550	89598
Sma-1-18901	NS_000200.1	+	179506085	179506133
Sma-1-18902	NW_003037822.1	+	769677	769725
Sma-1-18903	NS_000200.1	+	55077683	55077731
Sma-1-18904	NW_003038019.1	+	598263	598311
Sma-1-18905	NS_000200.1	+	233915081	233915129
Sma-1-18906	NW_003038109.1	-	101164	101116
Sma-1-18907	NS_000200.1	-	275783842	275783794
Sma-1-18908	NW_003037822.1	+	1037459	1037507
Sma-1-18909	NS_000200.1	+	55345465	55345513
Sma-1-18910	NW_003037979.1	-	265722	265674
Sma-1-18911	NS_000200.1	-	210326742	210326694
Sma-1-18912	NW_003037863.1	-	1119510	1119462
Sma-1-18913	NS_000200.1	-	107877877	107877829
Sma-1-18914	NW_003038069.1	-	186346	186298
Sma-1-18915	NS_000200.1	-	264029752	264029704
Sma-1-18916	NW_003037854.1	+	867605	867653
Sma-1-18917	NS_000200.1	+	97772026	97772074
Sma-1-18918	NW_003037795.1	-	1561225	1561177
Sma-1-18919	NS_000200.1	-	7952108	7952060
Sma-1-18920	NW_003037851.1	-	333976	333928
Sma-1-18921	NS_000200.1	-	93737286	93737238
Sma-1-18922	NW_003037824.1	-	83920	83872
Sma-1-18923	NS_000200.1	-	57014860	57014812
Sma-1-18924	NW_003037909.1	-	329902	329854
Sma-1-18925	NS_000200.1	-	153871551	153871503
Sma-1-18926	NW_003038010.1	+	132173	132221
Sma-1-18927	NS_000200.1	+	226950396	226950444
Sma-1-18928	NW_003037891.1	+	1548920	1548968
Sma-1-18929	NS_000200.1	+	139430198	139430246
Sma-1-18930	NW_003038069.1	+	72410	72458
Sma-1-18931	NS_000200.1	+	263915816	263915864
Sma-1-18932	NW_003037850.1	+	574621	574669
Sma-1-18933	NS_000200.1	+	92560651	92560699
Sma-1-18934	NW_003026465.1	+	756865	756913
Sma-1-18935	NS_000200.1	+	208149459	208149507
Sma-1-18936	NW_003038224.1	-	151373	151325
Sma-1-18937	NS_000200.1	-	309563101	309563053
Sma-1-18938	NW_003037805.1	-	1424194	1424146

Sma-1-18939	NS_000200.1	-	27982038	27981990
Sma-1-18940	NW_003038070.1	+	162497	162545
Sma-1-18941	NS_000200.1	+	264499738	264499786
Sma-1-18942	NW_003037825.1	+	82321	82369
Sma-1-18943	NS_000200.1	+	59212103	59212151
Sma-1-18944	NW_003037982.1	-	1307006	1306958
Sma-1-18945	NS_000200.1	-	214966201	214966153
Sma-1-18946	NW_003038161.1	+	102526	102574
Sma-1-18947	NS_000200.1	+	297472377	297472425
Sma-1-18948	NW_003037828.1	-	31585	31537
Sma-1-18949	NS_000200.1	-	65121070	65121022
Sma-1-18950	NW_003038113.1	-	9087	9039
Sma-1-18951	NS_000200.1	-	276669949	276669901
Sma-1-18952	NW_003038038.1	-	1219873	1219825
Sma-1-18953	NS_000200.1	-	253123266	253123218
Sma-1-18954	NW_003029826.1	+	7411	7459
Sma-1-18955	NW_003032094.1	-	3441	3393
Sma-1-18956	NS_000200.1	+	376278288	376278336
Sma-1-18957	NS_000200.1	-	377675026	377674978
Sma-1-18958	NW_003026596.1	-	19117	19069
Sma-1-18959	NS_000200.1	-	305289745	305289697
Sma-1-18960	NW_003038070.1	-	457382	457334
Sma-1-18961	NS_000200.1	-	264794623	264794575
Sma-1-18962	NW_003038006.1	+	293279	293327
Sma-1-18963	NS_000200.1	+	225177763	225177811
Sma-1-18964	NW_003037912.1	-	1269313	1269265
Sma-1-18965	NS_000200.1	-	158080888	158080840
Sma-1-18966	NW_003037812.1	-	1000624	1000576
Sma-1-18967	NS_000200.1	-	40483363	40483315
Sma-1-18968	NW_003038030.1	+	1003515	1003563
Sma-1-18969	NS_000200.1	+	244139382	244139430
Sma-1-18970	NW_003037944.1	+	418145	418193
Sma-1-18971	NS_000200.1	+	188639498	188639546
Sma-1-18972	NW_003038594.1	-	2469	2421
Sma-1-18973	NS_000200.1	-	344790831	344790783
Sma-1-18974	NW_003037904.1	+	71774	71822
Sma-1-18975	NS_000200.1	+	148786273	148786321
Sma-1-18976	NW_003037810.1	+	993310	993358
Sma-1-18977	NS_000200.1	+	37597832	37597880
Sma-1-18978	NW_003026722.1	-	10981	10933
Sma-1-18979	NS_000200.1	-	325422244	325422196
Sma-1-18980	NW_003037825.1	-	719243	719195
Sma-1-18981	NS_000200.1	-	59849025	59848977
Sma-1-18982	NW_003027899.1	+	828	876
Sma-1-18983	NS_000200.1	+	366120968	366121016
Sma-1-18984	NW_003026512.1	+	189607	189655
Sma-1-18985	NS_000200.1	+	285283647	285283695
Sma-1-18986	NW_003037848.1	+	915601	915649
Sma-1-18987	NS_000200.1	+	90608672	90608720
Sma-1-18988	NW_003037837.1	-	838892	838844
Sma-1-18989	NS_000200.1	-	77644750	77644702
Sma-1-18990	NW_003037962.1	-	51942	51894

Sma-1-18991	NS_000200.1	-	196128785	196128737
Sma-1-18992	NW_003037825.1	+	293857	293905
Sma-1-18993	NS_000200.1	+	59423639	59423687
Sma-1-18994	NW_003037907.1	+	1040753	1040801
Sma-1-18995	NS_000200.1	+	152445242	152445290
Sma-1-18996	NW_003037797.1	+	2038769	2038817
Sma-1-18997	NS_000200.1	+	12466629	12466677
Sma-1-18998	NW_003026456.1	+	572915	572963
Sma-1-18999	NS_000200.1	+	58496051	58496099
Sma-1-19000	NW_003038234.1	+	89258	89306
Sma-1-19001	NS_000200.1	+	312926027	312926075
Sma-1-19002	NW_003037825.1	+	1843872	1843920
Sma-1-19003	NS_000200.1	+	60973654	60973702
Sma-1-19004	NW_003038008.1	-	559916	559868
Sma-1-19005	NS_000200.1	-	226233012	226232964
Sma-1-19006	NW_003037801.1	+	704852	704900
Sma-1-19007	NS_000200.1	+	21794332	21794380
Sma-1-19008	NW_003037936.1	-	631912	631864
Sma-1-19009	NS_000200.1	-	181071641	181071593
Sma-1-19010	NW_003037877.1	+	963731	963779
Sma-1-19011	NS_000200.1	+	122002293	122002341
Sma-1-19012	NW_003037821.1	+	248931	248979
Sma-1-19013	NS_000200.1	+	53393415	53393463
Sma-1-19014	NW_003037830.1	+	1500771	1500819
Sma-1-19015	NS_000200.1	+	69391142	69391190
Sma-1-19016	NW_003037887.1	-	431577	431529
Sma-1-19017	NS_000200.1	-	130895555	130895507
Sma-1-19018	NW_003037794.1	+	855574	855622
Sma-1-19019	NS_000200.1	+	5035169	5035217
Sma-1-19020	NW_003037859.1	-	1043897	1043849
Sma-1-19021	NS_000200.1	-	103722738	103722690
Sma-1-19022	NW_003037909.1	+	238503	238551
Sma-1-19023	NS_000200.1	+	153780152	153780200
Sma-1-19024	NW_003038141.1	-	233357	233309
Sma-1-19025	NS_000200.1	-	287123336	287123288
Sma-1-19026	NW_003037935.1	+	780926	780974
Sma-1-19027	NS_000200.1	+	180197461	180197509
Sma-1-19028	NW_003037847.1	-	735141	735093
Sma-1-19029	NS_000200.1	-	89386871	89386823
Sma-1-19030	NW_003037859.1	+	26208	26256
Sma-1-19031	NS_000200.1	+	102705049	102705097
Sma-1-19032	NW_003037825.1	+	1341482	1341530
Sma-1-19033	NS_000200.1	+	60471264	60471312
Sma-1-19034	NW_003038072.1	+	325272	325320
Sma-1-19035	NS_000200.1	+	265487500	265487548
Sma-1-19036	NW_003037891.1	-	383239	383191
Sma-1-19037	NS_000200.1	-	138264517	138264469
Sma-1-19038	NW_003037800.1	+	641389	641437
Sma-1-19039	NS_000200.1	+	19270749	19270797
Sma-1-19040	NW_003038048.1	-	178160	178112
Sma-1-19041	NS_000200.1	-	255755991	255755943
Sma-1-19042	NW_003032312.1	-	1949	1901

Sma-1-19043	NS_000200.1	-	358762787	358762739
Sma-1-19044	NW_003037809.1	-	673892	673844
Sma-1-19045	NS_000200.1	-	34338356	34338308
Sma-1-19046	NW_003038255.1	+	266745	266793
Sma-1-19047	NS_000200.1	+	320655890	320655938
Sma-1-19048	NW_003038476.1	+	11049	11097
Sma-1-19049	NS_000200.1	+	332399424	332399472
Sma-1-19050	NW_003025089.1	-	697	649
Sma-1-19051	NS_000200.1	-	361156844	361156796
Sma-1-19052	NW_003038034.1	-	530691	530643
Sma-1-19053	NS_000200.1	-	247472447	247472399
Sma-1-19054	NW_003038023.1	-	68555	68507
Sma-1-19055	NS_000200.1	-	237228846	237228798
Sma-1-19056	NW_003037967.1	-	538284	538235
Sma-1-19057	NS_000200.1	-	199778255	199778206
Sma-1-19058	NW_003037967.1	+	51763	51812
Sma-1-19059	NS_000200.1	+	199291734	199291783
Sma-1-19060	NW_003037794.1	+	1981781	1981829
Sma-1-19061	NS_000200.1	+	6161376	6161424
Sma-1-19062	NW_003037824.1	+	636245	636293
Sma-1-19063	NS_000200.1	+	57567185	57567233
Sma-1-19064	NW_003037825.1	-	1189464	1189416
Sma-1-19065	NS_000200.1	-	60319246	60319198
Sma-1-19066	NW_003028777.1	-	1176	1128
Sma-1-19067	NS_000200.1	-	369919421	369919373
Sma-1-19068	NW_003037966.1	-	88086	88038
Sma-1-19069	NS_000200.1	-	198789949	198789901
Sma-1-19070	NW_003037784.1	+	383	431
Sma-1-19071	NS_000200.1	+	379829250	379829298
Sma-1-19072	NW_003038258.1	+	108424	108472
Sma-1-19073	NS_000200.1	+	322646934	322646982
Sma-1-19074	NW_003037859.1	-	524841	524793
Sma-1-19075	NS_000200.1	-	103203682	103203634
Sma-1-19076	NW_003038240.1	-	31397	31349
Sma-1-19077	NS_000200.1	-	314170328	314170280
Sma-1-19078	NW_003029025.1	+	1354	1402
Sma-1-19079	NW_003032116.1	+	1122	1170
Sma-1-19080	NW_003026463.1	+	54490	54538
Sma-1-19081	NS_000200.1	+	198669860	198669908
Sma-1-19082	NS_000200.1	+	375204863	375204911
Sma-1-19083	NS_000200.1	+	377801696	377801744
Sma-1-19084	NW_003037797.1	+	240300	240348
Sma-1-19085	NS_000200.1	+	10668160	10668208
Sma-1-19086	NW_003026470.1	+	477108	477156
Sma-1-19087	NS_000200.1	+	219601077	219601125
Sma-1-19088	NW_003037974.1	+	725116	725164
Sma-1-19089	NS_000200.1	+	205400422	205400470
Sma-1-19090	NW_003037829.1	-	1678478	1678430
Sma-1-19091	NS_000200.1	-	67579016	67578968
Sma-1-19092	NW_003038118.1	-	329178	329130
Sma-1-19093	NS_000200.1	-	277893305	277893257
Sma-1-19094	NW_003037888.1	-	61628	61580

Sma-1-19095	NS_000200.1	-	132270120	132270072
Sma-1-19096	NW_003037833.1	+	115643	115691
Sma-1-19097	NS_000200.1	+	72306739	72306787
Sma-1-19098	NW_003037807.1	+	808365	808413
Sma-1-19099	NS_000200.1	+	31069881	31069929
Sma-1-19100	NW_003037846.1	-	42888	42840
Sma-1-19101	NS_000200.1	-	87677458	87677410
Sma-1-19102	NW_003037859.1	+	796311	796359
Sma-1-19103	NS_000200.1	+	103475152	103475200
Sma-1-19104	NW_003026508.1	-	155164	155116
Sma-1-19105	NS_000200.1	-	281199778	281199730
Sma-1-19106	NW_003037846.1	+	349466	349514
Sma-1-19107	NS_000200.1	+	87984036	87984084
Sma-1-19108	NW_003037859.1	+	799759	799807
Sma-1-19109	NS_000200.1	+	103478600	103478648
Sma-1-19110	NW_003038032.1	+	669591	669639
Sma-1-19111	NS_000200.1	+	245828751	245828799
Sma-1-19112	NW_003029519.1	+	394	442
Sma-1-19113	NS_000200.1	+	362203205	362203253
Sma-1-19114	NW_003037893.1	-	384992	384944
Sma-1-19115	NS_000200.1	-	141499302	141499254
Sma-1-19116	NW_003037888.1	-	606133	606085
Sma-1-19117	NS_000200.1	-	132814625	132814577
Sma-1-19118	NW_003026507.1	+	252758	252806
Sma-1-19119	NS_000200.1	+	279912909	279912957
Sma-1-19120	NW_003037814.1	+	1514215	1514263
Sma-1-19121	NS_000200.1	+	44498075	44498123
Sma-1-19122	NW_003037894.1	-	310317	310269
Sma-1-19123	NS_000200.1	-	142206890	142206842
Sma-1-19124	NW_003037859.1	-	963483	963435
Sma-1-19125	NS_000200.1	-	103642324	103642276
Sma-1-19126	NW_003037860.1	-	257932	257884
Sma-1-19127	NS_000200.1	-	104000960	104000912
Sma-1-19128	NW_003037964.1	+	312	360
Sma-1-19129	NS_000200.1	+	197601834	197601882
Sma-1-19130	NW_003037814.1	+	1700182	1700230
Sma-1-19131	NS_000200.1	+	44684042	44684090
Sma-1-19132	NW_003037814.1	+	1689694	1689742
Sma-1-19133	NS_000200.1	+	44673554	44673602
Sma-1-19134	NW_003037861.1	+	195571	195619
Sma-1-19135	NS_000200.1	+	104972025	104972073
Sma-1-19136	NW_003037996.1	-	204101	204053
Sma-1-19137	NS_000200.1	-	220448977	220448929
Sma-1-19138	NW_003037890.1	+	136386	136434
Sma-1-19139	NS_000200.1	+	135819747	135819795
Sma-1-19140	NW_003037909.1	-	556087	556039
Sma-1-19141	NS_000200.1	-	154097736	154097688
Sma-1-19142	NW_003038254.1	-	249336	249288
Sma-1-19143	NS_000200.1	-	320284894	320284846
Sma-1-19144	NW_003037905.1	-	506118	506070
Sma-1-19145	NS_000200.1	-	149775130	149775082
Sma-1-19146	NW_003038056.1	-	52550	52502

Sma-1-19147	NS_000200.1	-	259030201	259030153
Sma-1-19148	NW_003037844.1	-	51771	51723
Sma-1-19149	NS_000200.1	-	86235217	86235169
Sma-1-19150	NW_003029861.1	+	325	373
Sma-1-19151	NS_000200.1	+	376513122	376513170
Sma-1-19152	NW_003037900.1	-	19382	19334
Sma-1-19153	NS_000200.1	-	145148248	145148200
Sma-1-19154	NW_003038030.1	+	686635	686683
Sma-1-19155	NS_000200.1	+	243822502	243822550
Sma-1-19156	NW_003037894.1	-	432213	432165
Sma-1-19157	NS_000200.1	-	142328786	142328738
Sma-1-19158	NW_003038027.1	-	193722	193674
Sma-1-19159	NS_000200.1	-	240564436	240564388
Sma-1-19160	NW_003038027.1	-	209163	209115
Sma-1-19161	NS_000200.1	-	240579877	240579829
Sma-1-19162	NW_003037977.1	+	71235	71283
Sma-1-19163	NS_000200.1	+	208357255	208357303
Sma-1-19164	NW_003037793.1	+	954765	954813
Sma-1-19165	NS_000200.1	+	954765	954813
Sma-1-19166	NW_003037794.1	-	851416	851368
Sma-1-19167	NS_000200.1	-	5031011	5030963
Sma-1-19168	NW_003037894.1	+	462393	462441
Sma-1-19169	NS_000200.1	+	142358966	142359014
Sma-1-19170	NW_003038077.1	-	186807	186759
Sma-1-19171	NS_000200.1	-	266958447	266958399
Sma-1-19172	NW_003037797.1	-	3361659	3361611
Sma-1-19173	NS_000200.1	-	13789519	13789471
Sma-1-19174	NW_003038536.1	-	6829	6781
Sma-1-19175	NS_000200.1	-	343326852	343326804
Sma-1-19176	NW_003022054.1	-	1373	1325
Sma-1-19177	NS_000200.1	-	355537402	355537354
Sma-1-19178	NW_003037825.1	+	560035	560083
Sma-1-19179	NS_000200.1	+	59689817	59689865
Sma-1-19180	NW_003038518.1	+	981441	981489
Sma-1-19181	NS_000200.1	+	342618274	342618322
Sma-1-19182	NW_003037962.1	+	440590	440638
Sma-1-19183	NS_000200.1	+	196517433	196517481
Sma-1-19184	NW_003025159.1	+	47	95
Sma-1-19185	NS_000200.1	+	361286867	361286915
Sma-1-19186	NW_003038153.1	-	189727	189679
Sma-1-19187	NS_000200.1	-	293251776	293251728
Sma-1-19188	NW_003038009.1	-	129626	129578
Sma-1-19189	NS_000200.1	-	226503689	226503641
Sma-1-19190	NW_003030628.1	-	1168	1120
Sma-1-19191	NS_000200.1	-	366607265	366607217
Sma-1-19192	NW_003037823.1	+	996076	996124
Sma-1-19193	NS_000200.1	+	56912695	56912743
Sma-1-19194	NW_003026490.1	-	74359	74311
Sma-1-19195	NS_000200.1	-	267706075	267706027
Sma-1-19196	NW_003037874.1	-	603247	603199
Sma-1-19197	NS_000200.1	-	120028197	120028149
Sma-1-19198	NW_003037932.1	+	637124	637172

Sma-1-19199	NS_000200.1	+	178170320	178170368
Sma-1-19200	NW_003037918.1	+	128924	128972
Sma-1-19201	NS_000200.1	+	166695914	166695962
Sma-1-19202	NW_003037795.1	-	210294	210246
Sma-1-19203	NS_000200.1	-	6601177	6601129
Sma-1-19204	NW_003037841.1	-	785994	785946
Sma-1-19205	NS_000200.1	-	83397175	83397127
Sma-1-19206	NW_003037896.1	+	124692	124740
Sma-1-19207	NS_000200.1	+	143045115	143045163
Sma-1-19208	NW_003037823.1	-	68820	68772
Sma-1-19209	NS_000200.1	-	55985439	55985391
Sma-1-19210	NW_003037861.1	-	393838	393790
Sma-1-19211	NS_000200.1	-	105170292	105170244
Sma-1-19212	NW_003038053.1	-	399586	399538
Sma-1-19213	NS_000200.1	-	257759681	257759633
Sma-1-19214	NW_003037823.1	+	6111	6159
Sma-1-19215	NS_000200.1	+	55922730	55922778
Sma-1-19216	NW_003037909.1	-	740071	740023
Sma-1-19217	NS_000200.1	-	154281720	154281672
Sma-1-19218	NW_003037829.1	+	604493	604541
Sma-1-19219	NS_000200.1	+	66505031	66505079
Sma-1-19220	NW_003031119.1	+	55	103
Sma-1-19221	NS_000200.1	+	325774419	325774467
Sma-1-19222	NW_003038028.1	+	503375	503423
Sma-1-19223	NS_000200.1	+	241810949	241810997
Sma-1-19224	NW_003031278.1	+	302	350
Sma-1-19225	NW_003035818.1	+	3458	3506
Sma-1-19226	NS_000200.1	+	345658521	345658569
Sma-1-19227	NS_000200.1	+	364158885	364158933
Sma-1-19228	NW_003031299.1	-	1245	1197
Sma-1-19229	NS_000200.1	-	345694692	345694644
Sma-1-19230	NW_003038028.1	-	642867	642819
Sma-1-19231	NS_000200.1	-	241950441	241950393
Sma-1-19232	NW_003037862.1	+	298826	298874
Sma-1-19233	NS_000200.1	+	105950487	105950535
Sma-1-19234	NW_003037887.1	+	1279182	1279230
Sma-1-19235	NS_000200.1	+	131743160	131743208
Sma-1-19236	NW_003037794.1	+	1307613	1307661
Sma-1-19237	NS_000200.1	+	5487208	5487256
Sma-1-19238	NW_003037831.1	+	1220198	1220246
Sma-1-19239	NS_000200.1	+	70669500	70669548
Sma-1-19240	NW_003037932.1	+	878291	878339
Sma-1-19241	NS_000200.1	+	178411487	178411535
Sma-1-19242	NW_003037905.1	-	180383	180333
Sma-1-19243	NS_000200.1	-	149449395	149449345
Sma-1-19244	NW_003037887.1	+	1259381	1259429
Sma-1-19245	NS_000200.1	+	131723359	131723407
Sma-1-19246	NW_003037793.1	-	2691611	2691563
Sma-1-19247	NS_000200.1	-	2691611	2691563
Sma-1-19248	NW_003031789.1	-	1140	1092
Sma-1-19249	NS_000200.1	-	367015914	367015866
Sma-1-19250	NW_003037911.1	+	80760	80808

Sma-1-19251	NS_000200.1	+	155868113	155868161
Sma-1-19252	NW_003037881.1	-	829259	829211
Sma-1-19253	NS_000200.1	-	125261050	125261002
Sma-1-19254	NW_003026638.1	+	88	136
Sma-1-19255	NS_000200.1	+	325044771	325044819
Sma-1-19256	NW_003038148.1	+	202227	202275
Sma-1-19257	NS_000200.1	+	290276211	290276259
Sma-1-19258	NW_003037805.1	+	975779	975827
Sma-1-19259	NS_000200.1	+	27533623	27533671
Sma-1-19260	NW_003038148.1	+	640	688
Sma-1-19261	NW_003038148.1	+	36706	36754
Sma-1-19262	NS_000200.1	+	290074624	290074672
Sma-1-19263	NS_000200.1	+	290110690	290110738
Sma-1-19264	NW_003037961.1	-	340446	340398
Sma-1-19265	NS_000200.1	-	195964688	195964640
Sma-1-19266	NW_003037863.1	-	1280280	1280232
Sma-1-19267	NS_000200.1	-	108038647	108038599
Sma-1-19268	NW_003037863.1	-	1306522	1306474
Sma-1-19269	NS_000200.1	-	108064889	108064841
Sma-1-19270	NW_003037815.1	-	341911	341863
Sma-1-19271	NS_000200.1	-	45604902	45604854
Sma-1-19272	NW_003035880.1	-	102094	102046
Sma-1-19273	NS_000200.1	-	339343158	339343110
Sma-1-19274	NW_003037863.1	+	172067	172115
Sma-1-19275	NS_000200.1	+	106930434	106930482
Sma-1-19276	NW_003037794.1	+	1673655	1673703
Sma-1-19277	NS_000200.1	+	5853250	5853298
Sma-1-19278	NW_003038030.1	+	402820	402868
Sma-1-19279	NS_000200.1	+	243538687	243538735
Sma-1-19280	NW_003037793.1	+	3581717	3581765
Sma-1-19281	NS_000200.1	+	3581717	3581765
Sma-1-19282	NW_003037806.1	+	1033226	1033274
Sma-1-19283	NS_000200.1	+	29228440	29228488
Sma-1-19284	NW_003038030.1	-	454534	454486
Sma-1-19285	NS_000200.1	-	243590401	243590353
Sma-1-19286	NW_003037814.1	+	374931	374979
Sma-1-19287	NS_000200.1	+	43358791	43358839
Sma-1-19288	NW_003037796.1	+	353448	353496
Sma-1-19289	NS_000200.1	+	8563092	8563140
Sma-1-19290	NW_003037829.1	+	621917	621966
Sma-1-19291	NS_000200.1	+	66522455	66522504
Sma-1-19292	NW_003038030.1	-	511325	511277
Sma-1-19293	NS_000200.1	-	243647192	243647144
Sma-1-19294	NW_003037793.1	+	3517517	3517565
Sma-1-19295	NS_000200.1	+	3517517	3517565
Sma-1-19296	NW_003038056.1	+	114987	115035
Sma-1-19297	NS_000200.1	+	259092638	259092686
Sma-1-19298	NW_003024373.1	+	1167	1215
Sma-1-19299	NS_000200.1	+	365606979	365607027
Sma-1-19300	NW_003038074.1	-	113629	113581
Sma-1-19301	NS_000200.1	-	266005009	266004961
Sma-1-19302	NW_003030487.1	-	2062	2014

Sma-1-19303	NS_000200.1	-	363032614	363032566
Sma-1-19304	NW_003037912.1	+	18753	18801
Sma-1-19305	NS_000200.1	+	156830328	156830376
Sma-1-19306	NW_003037862.1	+	794355	794403
Sma-1-19307	NS_000200.1	+	106446016	106446064
Sma-1-19308	NW_003038030.1	-	764707	764659
Sma-1-19309	NS_000200.1	-	243900574	243900526
Sma-1-19310	NW_003038030.1	+	78246	78294
Sma-1-19311	NS_000200.1	+	243214113	243214161
Sma-1-19312	NW_003037822.1	+	385632	385680
Sma-1-19313	NS_000200.1	+	54693638	54693686
Sma-1-19314	NW_003038031.1	-	384010	383962
Sma-1-19315	NS_000200.1	-	244661768	244661720
Sma-1-19316	NW_003037870.1	+	247795	247843
Sma-1-19317	NS_000200.1	+	117121460	117121508
Sma-1-19318	NW_003038031.1	+	434433	434481
Sma-1-19319	NS_000200.1	+	244712191	244712239
Sma-1-19320	NW_003038031.1	-	549764	549716
Sma-1-19321	NS_000200.1	-	244827522	244827474
Sma-1-19322	NW_003037864.1	+	1748434	1748482
Sma-1-19323	NS_000200.1	+	110241008	110241056
Sma-1-19324	NW_003037839.1	-	97784	97736
Sma-1-19325	NS_000200.1	-	79055934	79055886
Sma-1-19326	NW_003037916.1	+	196975	197023
Sma-1-19327	NS_000200.1	+	163390386	163390434
Sma-1-19328	NW_003038155.1	-	120251	120203
Sma-1-19329	NS_000200.1	-	294243369	294243321
Sma-1-19330	NW_003029434.1	+	1955	2003
Sma-1-19331	NW_003034123.1	+	359	407
Sma-1-19332	NS_000200.1	+	362054661	362054709
Sma-1-19333	NS_000200.1	+	363864536	363864584
Sma-1-19334	NW_003038254.1	+	196849	196897
Sma-1-19335	NS_000200.1	+	320232407	320232455
Sma-1-19336	NW_003037956.1	-	290266	290218
Sma-1-19337	NS_000200.1	-	194346721	194346673
Sma-1-19338	NW_003037822.1	-	209404	209356
Sma-1-19339	NS_000200.1	-	54517410	54517362
Sma-1-19340	NW_003038008.1	-	21918	21870
Sma-1-19341	NS_000200.1	-	225695014	225694966
Sma-1-19342	NW_003037864.1	+	47365	47413
Sma-1-19343	NS_000200.1	+	108539939	108539987
Sma-1-19344	NW_003037864.1	+	704296	704344
Sma-1-19345	NS_000200.1	+	109196870	109196918
Sma-1-19346	NW_003037865.1	+	270702	270750
Sma-1-19347	NS_000200.1	+	110692621	110692669
Sma-1-19348	NW_003037805.1	+	135067	135115
Sma-1-19349	NS_000200.1	+	26692911	26692959
Sma-1-19350	NW_003038256.1	-	229309	229261
Sma-1-19351	NS_000200.1	-	320963135	320963087
Sma-1-19352	NW_003033786.1	-	604	556
Sma-1-19353	NS_000200.1	-	358970129	358970081
Sma-1-19354	NW_003037977.1	+	866076	866124

Sma-1-19355	NS_000200.1	+	209152096	209152144
Sma-1-19356	NW_003033800.1	-	878	830
Sma-1-19357	NS_000200.1	-	359014492	359014444
Sma-1-19358	NW_003026486.1	-	72203	72155
Sma-1-19359	NS_000200.1	-	259693322	259693274
Sma-1-19360	NW_003038083.1	+	609940	609988
Sma-1-19361	NW_003038083.1	-	620486	620438
Sma-1-19362	NS_000200.1	+	270853277	270853325
Sma-1-19363	NS_000200.1	-	270863823	270863775
Sma-1-19364	NW_003037917.1	+	691415	691463
Sma-1-19365	NS_000200.1	+	165728219	165728267
Sma-1-19366	NW_003034018.1	+	727	775
Sma-1-19367	NS_000200.1	+	363482302	363482350
Sma-1-19368	NW_003037795.1	-	1140716	1140668
Sma-1-19369	NS_000200.1	-	7531599	7531551
Sma-1-19370	NW_003037817.1	+	622702	622750
Sma-1-19371	NS_000200.1	+	48005054	48005102
Sma-1-19372	NW_003037859.1	+	494572	494620
Sma-1-19373	NS_000200.1	+	103173413	103173461
Sma-1-19374	NW_003037913.1	-	376032	375984
Sma-1-19375	NS_000200.1	-	158939783	158939735
Sma-1-19376	NW_003037822.1	+	1031027	1031075
Sma-1-19377	NW_003038236.1	+	52035	52083
Sma-1-19378	NS_000200.1	+	55339033	55339081
Sma-1-19379	NS_000200.1	+	313297705	313297753
Sma-1-19380	NW_003037813.1	-	920736	920688
Sma-1-19381	NS_000200.1	-	41907599	41907551
Sma-1-19382	NW_003037809.1	+	658466	658514
Sma-1-19383	NS_000200.1	+	34322930	34322978
Sma-1-19384	NW_003038122.1	-	20742	20694
Sma-1-19385	NS_000200.1	-	278539969	278539921
Sma-1-19386	NW_003038152.1	-	150908	150860
Sma-1-19387	NS_000200.1	-	292756632	292756584
Sma-1-19388	NW_003038152.1	-	240839	240791
Sma-1-19389	NS_000200.1	-	292846563	292846515
Sma-1-19390	NW_003037821.1	+	256253	256301
Sma-1-19391	NS_000200.1	+	53400737	53400785
Sma-1-19392	NW_003037811.1	+	611293	611341
Sma-1-19393	NW_003037813.1	-	1038722	1038674
Sma-1-19394	NS_000200.1	+	38781535	38781583
Sma-1-19395	NS_000200.1	-	42025585	42025537
Sma-1-19396	NW_003038258.1	+	285225	285273
Sma-1-19397	NS_000200.1	+	322823735	322823783
Sma-1-19398	NW_003038259.1	-	123770	123722
Sma-1-19399	NS_000200.1	-	322984369	322984321
Sma-1-19400	NW_003037977.1	+	909275	909323
Sma-1-19401	NS_000200.1	+	209195295	209195343
Sma-1-19402	NW_003037866.1	+	1395812	1395860
Sma-1-19403	NS_000200.1	+	112883611	112883659
Sma-1-19404	NW_003037911.1	-	507772	507724
Sma-1-19405	NS_000200.1	-	156295125	156295077
Sma-1-19406	NW_003037798.1	-	1562311	1562263

Sma-1-19407	NS_000200.1	-	15821469	15821421
Sma-1-19408	NW_003038018.1	-	593299	593251
Sma-1-19409	NS_000200.1	-	232994149	232994101
Sma-1-19410	NW_003035432.1	-	155	107
Sma-1-19411	NW_003037793.1	+	459544	459592
Sma-1-19412	NS_000200.1	+	459544	459592
Sma-1-19413	NS_000200.1	-	367455961	367455913
Sma-1-19414	NW_003037946.1	-	1180602	1180554
Sma-1-19415	NS_000200.1	-	191874462	191874414
Sma-1-19416	NW_003037867.1	+	111277	111325
Sma-1-19417	NS_000200.1	+	115712096	115712144
Sma-1-19418	NW_003037820.1	-	532353	532305
Sma-1-19419	NS_000200.1	-	52805335	52805287
Sma-1-19420	NW_003038001.1	-	220843	220795
Sma-1-19421	NS_000200.1	-	222930404	222930356
Sma-1-19422	NW_003037820.1	+	488037	488085
Sma-1-19423	NS_000200.1	+	52761019	52761067
Sma-1-19424	NW_003037797.1	-	3726009	3725961
Sma-1-19425	NS_000200.1	-	14153869	14153821
Sma-1-19426	NW_003037820.1	-	480669	480621
Sma-1-19427	NS_000200.1	-	52753651	52753603
Sma-1-19428	NW_003038053.1	+	61204	61252
Sma-1-19429	NS_000200.1	+	257421299	257421347
Sma-1-19430	NW_003037935.1	-	200090	200042
Sma-1-19431	NS_000200.1	-	179616625	179616577
Sma-1-19432	NW_003037840.1	-	277883	277835
Sma-1-19433	NS_000200.1	-	80901207	80901159
Sma-1-19434	NW_003035622.1	-	4390	4341
Sma-1-19435	NS_000200.1	-	329425997	329425948
Sma-1-19436	NW_003035637.1	-	16241	16193
Sma-1-19437	NS_000200.1	-	329817483	329817435
Sma-1-19438	NW_003037898.1	+	225033	225081
Sma-1-19439	NS_000200.1	+	144208855	144208903
Sma-1-19440	NW_003037868.1	-	268782	268734
Sma-1-19441	NS_000200.1	-	116240494	116240446
Sma-1-19442	NW_003038006.1	+	249251	249299
Sma-1-19443	NS_000200.1	+	225133735	225133783
Sma-1-19444	NW_003037944.1	+	737568	737616
Sma-1-19445	NS_000200.1	+	188958921	188958969
Sma-1-19446	NW_003037840.1	+	1015949	1015997
Sma-1-19447	NS_000200.1	+	81639273	81639321
Sma-1-19448	NW_003020746.1	+	149	197
Sma-1-19449	NS_000200.1	+	364369082	364369130
Sma-1-19450	NW_003037935.1	+	128170	128218
Sma-1-19451	NS_000200.1	+	179544705	179544753
Sma-1-19452	NW_003035688.1	-	3285	3237
Sma-1-19453	NS_000200.1	-	332905763	332905715
Sma-1-19454	NW_003037898.1	-	430215	430167
Sma-1-19455	NS_000200.1	-	144414037	144413989
Sma-1-19456	NW_003038003.1	-	12948	12900
Sma-1-19457	NS_000200.1	-	223512230	223512182
Sma-1-19458	NW_003038085.1	+	16108	16156

Sma-1-19459	NS_000200.1	+	271706201	271706249
Sma-1-19460	NW_003037819.1	-	757729	757681
Sma-1-19461	NS_000200.1	-	51845994	51845946
Sma-1-19462	NW_003038085.1	+	532496	532544
Sma-1-19463	NS_000200.1	+	272222589	272222637
Sma-1-19464	NW_003037878.1	-	689020	688972
Sma-1-19465	NS_000200.1	-	122742498	122742450
Sma-1-19466	NW_003023437.1	+	356	404
Sma-1-19467	NS_000200.1	+	373379519	373379567
Sma-1-19468	NW_003023408.1	+	557	605
Sma-1-19469	NS_000200.1	+	373299654	373299702
Sma-1-19470	NW_003026517.1	+	290197	290245
Sma-1-19471	NS_000200.1	+	299896206	299896254
Sma-1-19472	NW_003037871.1	+	347710	347758
Sma-1-19473	NS_000200.1	+	117795448	117795496
Sma-1-19474	NW_003021169.1	-	1104	1056
Sma-1-19475	NW_003037812.1	-	980185	980137
Sma-1-19476	NS_000200.1	-	371504702	371504654
Sma-1-19477	NS_000200.1	-	40462924	40462876
Sma-1-19478	NW_003026472.1	-	364054	364006
Sma-1-19479	NS_000200.1	-	236776046	236775998
Sma-1-19480	NW_003021218.1	-	1513	1465
Sma-1-19481	NS_000200.1	-	371614812	371614764
Sma-1-19482	NW_003038261.1	+	681637	681685
Sma-1-19483	NS_000200.1	+	324295288	324295336
Sma-1-19484	NW_003038154.1	+	161171	161219
Sma-1-19485	NS_000200.1	+	293958880	293958928
Sma-1-19486	NW_003021346.1	+	100	148
Sma-1-19487	NW_003032238.1	-	2514	2466
Sma-1-19488	NW_003035241.1	+	454	502
Sma-1-19489	NS_000200.1	+	343906794	343906842
Sma-1-19490	NS_000200.1	+	370865370	370865418
Sma-1-19491	NS_000200.1	-	357975959	357975911
Sma-1-19492	NW_003035877.1	+	115437	115485
Sma-1-19493	NS_000200.1	+	337095971	337096019
Sma-1-19494	NW_003038261.1	+	913339	913387
Sma-1-19495	NW_003038261.1	+	921832	921880
Sma-1-19496	NS_000200.1	+	324526990	324527038
Sma-1-19497	NS_000200.1	+	324535483	324535531
Sma-1-19498	NW_003037899.1	-	119176	119128
Sma-1-19499	NS_000200.1	-	144638287	144638239
Sma-1-19500	NW_003037839.1	+	307828	307876
Sma-1-19501	NS_000200.1	+	79265978	79266026
Sma-1-19502	NW_003038261.1	+	952732	952780
Sma-1-19503	NS_000200.1	+	324566383	324566431
Sma-1-19504	NW_003035881.1	+	277728	277776
Sma-1-19505	NS_000200.1	+	341341933	341341981
Sma-1-19506	NW_003035878.1	+	420327	420375
Sma-1-19507	NS_000200.1	+	337630137	337630185
Sma-1-19508	NW_003039056.1	+	1359	1407
Sma-1-19509	NS_000200.1	+	359424437	359424485
Sma-1-19510	NW_003038035.1	-	1061842	1061794

Sma-1-19511	NS_000200.1	-	248923334	248923286
Sma-1-19512	NW_003037899.1	+	451569	451617
Sma-1-19513	NS_000200.1	+	144970680	144970728
Sma-1-19514	NW_003037899.1	+	464779	464827
Sma-1-19515	NS_000200.1	+	144983890	144983938
Sma-1-19516	NW_003035879.1	-	120208	120160
Sma-1-19517	NS_000200.1	-	338087535	338087487
Sma-1-19518	NW_003038003.1	-	4438	4390
Sma-1-19519	NS_000200.1	-	223503720	223503672
Sma-1-19520	NW_003035880.1	+	206872	206920
Sma-1-19521	NS_000200.1	+	339447936	339447984
Sma-1-19522	NW_003038003.1	+	325145	325193
Sma-1-19523	NS_000200.1	+	223824427	223824475
Sma-1-19524	NW_003037885.1	+	1060355	1060403
Sma-1-19525	NS_000200.1	+	129158302	129158350
Sma-1-19526	NW_003037938.1	+	807658	807706
Sma-1-19527	NS_000200.1	+	182844771	182844819
Sma-1-19528	NW_003022184.1	+	918	966
Sma-1-19529	NS_000200.1	+	360888777	360888825
Sma-1-19530	NW_003037813.1	-	1398625	1398577
Sma-1-19531	NW_003037909.1	+	467363	467411
Sma-1-19532	NS_000200.1	+	154009012	154009060
Sma-1-19533	NS_000200.1	-	42385488	42385440
Sma-1-19534	NW_003037830.1	+	614050	614098
Sma-1-19535	NS_000200.1	+	68504421	68504469
Sma-1-19536	NW_003038036.1	+	12466	12514
Sma-1-19537	NS_000200.1	+	248946318	248946366
Sma-1-19538	NW_003037912.1	-	47941	47893
Sma-1-19539	NS_000200.1	-	156859516	156859468
Sma-1-19540	NW_003022274.1	+	1024	1072
Sma-1-19541	NS_000200.1	+	361074710	361074758
Sma-1-19542	NW_003022372.1	-	313	265
Sma-1-19543	NS_000200.1	-	364823002	364822954
Sma-1-19544	NW_003035880.1	+	63845	63894
Sma-1-19545	NS_000200.1	+	339304909	339304958
Sma-1-19546	NW_003038124.1	-	210298	210250
Sma-1-19547	NS_000200.1	-	279202942	279202894
Sma-1-19548	NW_003022490.1	-	6707	6659
Sma-1-19549	NW_003037853.1	-	531816	531768
Sma-1-19550	NS_000200.1	-	365039988	365039940
Sma-1-19551	NS_000200.1	-	96023867	96023819
Sma-1-19552	NW_003037931.1	-	343455	343407
Sma-1-19553	NS_000200.1	-	177176122	177176074
Sma-1-19554	NW_003022550.1	-	413	365
Sma-1-19555	NS_000200.1	-	365162232	365162184
Sma-1-19556	NW_003038036.1	-	864954	864906
Sma-1-19557	NS_000200.1	-	249798806	249798758
Sma-1-19558	NW_003025819.1	-	102	54
Sma-1-19559	NS_000200.1	-	356584585	356584537
Sma-1-19560	NW_003037797.1	-	503406	503358
Sma-1-19561	NS_000200.1	-	10931266	10931218
Sma-1-19562	NW_003038037.1	-	1008722	1008674

Sma-1-19563	NS_000200.1	-	251164131	251164083
Sma-1-19564	NW_003022663.1	+	1127	1175
Sma-1-19565	NW_003037936.1	-	250004	249956
Sma-1-19566	NS_000200.1	+	365377731	365377779
Sma-1-19567	NS_000200.1	-	180689733	180689685
Sma-1-19568	NW_003037797.1	+	663853	663901
Sma-1-19569	NS_000200.1	+	11091713	11091761
Sma-1-19570	NW_003037905.1	+	663771	663819
Sma-1-19571	NS_000200.1	+	149932783	149932831
Sma-1-19572	NW_003038421.1	-	12348	12300
Sma-1-19573	NS_000200.1	-	329059892	329059844
Sma-1-19574	NW_003038155.1	+	467174	467222
Sma-1-19575	NS_000200.1	+	294590292	294590340
Sma-1-19576	NW_003038055.1	-	162158	162110
Sma-1-19577	NS_000200.1	-	258861480	258861432
Sma-1-19578	NW_003038037.1	+	1242125	1242173
Sma-1-19579	NS_000200.1	+	251397534	251397582
Sma-1-19580	NW_003037813.1	-	629518	629470
Sma-1-19581	NS_000200.1	-	41616381	41616333
Sma-1-19582	NW_003022936.1	-	749	701
Sma-1-19583	NS_000200.1	-	371912400	371912352
Sma-1-19584	NW_003037819.1	-	1003906	1003858
Sma-1-19585	NS_000200.1	-	52092171	52092123
Sma-1-19586	NW_003039130.1	+	213474	213522
Sma-1-19587	NS_000200.1	+	317888085	317888133
Sma-1-19588	NW_003037943.1	+	365777	365825
Sma-1-19589	NS_000200.1	+	187387310	187387358
Sma-1-19590	NW_003038000.1	+	59035	59083
Sma-1-19591	NS_000200.1	+	222529813	222529861
Sma-1-19592	NW_003037884.1	+	854409	854457
Sma-1-19593	NS_000200.1	+	127634074	127634122
Sma-1-19594	NW_003037916.1	+	378325	378373
Sma-1-19595	NS_000200.1	+	163571736	163571784
Sma-1-19596	NW_003037801.1	+	704519	704567
Sma-1-19597	NS_000200.1	+	21793999	21794047
Sma-1-19598	NW_003038075.1	+	44073	44121
Sma-1-19599	NS_000200.1	+	266162284	266162332
Sma-1-19600	NW_003038131.1	-	51930	51882
Sma-1-19601	NS_000200.1	-	282013774	282013726
Sma-1-19602	NW_003037912.1	-	491304	491256
Sma-1-19603	NS_000200.1	-	157302879	157302831
Sma-1-19604	NW_003037809.1	-	509598	509550
Sma-1-19605	NS_000200.1	-	34174062	34174014
Sma-1-19606	NW_003037908.1	-	223983	223935
Sma-1-19607	NS_000200.1	-	152697114	152697066
Sma-1-19608	NW_003036284.1	+	449	497
Sma-1-19609	NS_000200.1	+	359562230	359562278
Sma-1-19610	NW_003038086.1	-	96598	96550
Sma-1-19611	NS_000200.1	-	272345191	272345143
Sma-1-19612	NW_003036309.1	+	1328	1376
Sma-1-19613	NS_000200.1	+	359606339	359606387
Sma-1-19614	NW_003037915.1	+	1050721	1050769

Sma-1-19615	NW_003037915.1	+	1058868	1058916
Sma-1-19616	NS_000200.1	+	162950707	162950755
Sma-1-19617	NS_000200.1	+	162958854	162958902
Sma-1-19618	NW_003038087.1	+	295937	295985
Sma-1-19619	NS_000200.1	+	273324338	273324386
Sma-1-19620	NW_003037818.1	+	835564	835612
Sma-1-19621	NS_000200.1	+	49550971	49551019
Sma-1-19622	NW_003038464.1	+	29874	29922
Sma-1-19623	NS_000200.1	+	331535141	331535189
Sma-1-19624	NW_003039130.1	-	82803	82755
Sma-1-19625	NS_000200.1	-	317757414	317757366
Sma-1-19626	NW_003037942.1	-	1170975	1170927
Sma-1-19627	NS_000200.1	-	186930883	186930835
Sma-1-19628	NW_003037836.1	-	93487	93439
Sma-1-19629	NS_000200.1	-	75650586	75650538
Sma-1-19630	NW_003037876.1	+	459735	459783
Sma-1-19631	NS_000200.1	+	121005371	121005419
Sma-1-19632	NW_003037836.1	-	529023	528973
Sma-1-19633	NS_000200.1	-	76086122	76086072
Sma-1-19634	NW_003038118.1	-	267947	267899
Sma-1-19635	NS_000200.1	-	277832074	277832026
Sma-1-19636	NW_003038498.1	-	62306	62258
Sma-1-19637	NS_000200.1	-	334482518	334482470
Sma-1-19638	NW_003037865.1	-	82369	82321
Sma-1-19639	NS_000200.1	-	110504288	110504240
Sma-1-19640	NW_003037884.1	-	1062083	1062035
Sma-1-19641	NS_000200.1	-	127841748	127841700
Sma-1-19642	NW_003037037.1	+	442	490
Sma-1-19643	NS_000200.1	+	343709638	343709686
Sma-1-19644	NW_003037809.1	+	50509	50557
Sma-1-19645	NS_000200.1	+	33714973	33715021
Sma-1-19646	NW_003037877.1	+	80961	81009
Sma-1-19647	NS_000200.1	+	121119523	121119571
Sma-1-19648	NW_003037918.1	-	863771	863723
Sma-1-19649	NS_000200.1	-	167430761	167430713
Sma-1-19650	NW_003038079.1	-	316935	316887
Sma-1-19651	NS_000200.1	-	268446923	268446875
Sma-1-19652	NW_003037878.1	-	213387	213339
Sma-1-19653	NS_000200.1	-	122266865	122266817
Sma-1-19654	NW_003037916.1	-	1398405	1398357
Sma-1-19655	NS_000200.1	-	164591816	164591768
Sma-1-19656	NW_003038016.1	+	529404	529452
Sma-1-19657	NS_000200.1	+	231645941	231645989
Sma-1-19658	NW_003038013.1	-	114210	114162
Sma-1-19659	NS_000200.1	-	229503836	229503788
Sma-1-19660	NW_003037941.1	-	673146	673098
Sma-1-19661	NS_000200.1	-	185407620	185407572
Sma-1-19662	NW_003025056.1	-	1507	1459
Sma-1-19663	NS_000200.1	-	355977907	355977859
Sma-1-19664	NW_003037831.1	+	1113771	1113819
Sma-1-19665	NS_000200.1	+	70563073	70563121
Sma-1-19666	NW_003038107.1	-	27390	27342

Sma-1-19667	NS_000200.1	-	275330477	275330429
Sma-1-19668	NW_003037940.1	+	912723	912771
Sma-1-19669	NS_000200.1	+	184654853	184654901
Sma-1-19670	NW_003038109.1	-	103380	103332
Sma-1-19671	NS_000200.1	-	275786058	275786010
Sma-1-19672	NW_003038502.1	-	176147	176099
Sma-1-19673	NS_000200.1	-	335175192	335175144
Sma-1-19674	NW_003026466.1	+	1269	1317
Sma-1-19675	NS_000200.1	+	215268376	215268424
Sma-1-19676	NW_003026466.1	+	119628	119676
Sma-1-19677	NS_000200.1	+	215386735	215386783
Sma-1-19678	NW_003037801.1	-	812848	812800
Sma-1-19679	NS_000200.1	-	21902328	21902280
Sma-1-19680	NW_003037795.1	-	164886	164838
Sma-1-19681	NS_000200.1	-	6555769	6555721
Sma-1-19682	NW_003026465.1	-	74923	74875
Sma-1-19683	NS_000200.1	-	207467517	207467469
Sma-1-19684	NW_003037818.1	+	1532092	1532140
Sma-1-19685	NS_000200.1	+	50247499	50247547
Sma-1-19686	NW_003038502.1	-	181943	181895
Sma-1-19687	NS_000200.1	-	335180988	335180940
Sma-1-19688	NW_003037798.1	-	1259595	1259547
Sma-1-19689	NS_000200.1	-	15518753	15518705
Sma-1-19690	NW_003037913.1	-	629357	629309
Sma-1-19691	NS_000200.1	-	159193108	159193060
Sma-1-19692	NW_003037835.1	+	963470	963518
Sma-1-19693	NS_000200.1	+	75359978	75360026
Sma-1-19694	NW_003037835.1	-	910173	910125
Sma-1-19695	NS_000200.1	-	75306681	75306633
Sma-1-19696	NW_003025858.1	+	955	1003
Sma-1-19697	NW_003037824.1	-	345096	345048
Sma-1-19698	NS_000200.1	+	356644806	356644854
Sma-1-19699	NS_000200.1	-	57276036	57275988
Sma-1-19700	NS_000200.1	-	369420051	369420003
Sma-1-19701	NW_003037785.1	+	149994	150042
Sma-1-19702	NS_000200.1	+	380048661	380048709
Sma-1-19703	NW_003037817.1	+	770975	771023
Sma-1-19704	NS_000200.1	+	48153327	48153375
Sma-1-19705	NW_003037901.1	-	798321	798273
Sma-1-19706	NS_000200.1	-	146646782	146646734
Sma-1-19707	NW_003037835.1	-	1020632	1020584
Sma-1-19708	NW_003038145.1	+	69486	69534
Sma-1-19709	NS_000200.1	+	288680909	288680957
Sma-1-19710	NS_000200.1	-	75417140	75417092
Sma-1-19711	NW_003037918.1	+	226537	226585
Sma-1-19712	NS_000200.1	+	166793527	166793575
Sma-1-19713	NW_003037806.1	+	1390035	1390083
Sma-1-19714	NS_000200.1	+	29585249	29585297
Sma-1-19715	NW_003038039.1	-	5346	5298
Sma-1-19716	NS_000200.1	-	253443192	253443144
Sma-1-19717	NW_003037809.1	+	2418859	2418907
Sma-1-19718	NS_000200.1	+	36083323	36083371

Sma-1-19719	NW_003037925.1	+	530721	530769
Sma-1-19720	NS_000200.1	+	172932724	172932772
Sma-1-19721	NW_003037980.1	+	690593	690641
Sma-1-19722	NS_000200.1	+	211651455	211651503
Sma-1-19723	NW_003037916.1	+	1664438	1664486
Sma-1-19724	NS_000200.1	+	164857849	164857897
Sma-1-19725	NW_003037788.1	+	206358	206406
Sma-1-19726	NS_000200.1	+	380653596	380653644
Sma-1-19727	NW_003037928.1	+	390029	390077
Sma-1-19728	NS_000200.1	+	174713982	174714030
Sma-1-19729	NW_003037928.1	+	910321	910369
Sma-1-19730	NS_000200.1	+	175234274	175234322
Sma-1-19731	NW_003037916.1	+	655203	655251
Sma-1-19732	NS_000200.1	+	163848614	163848662
Sma-1-19733	NW_003037940.1	+	132450	132498
Sma-1-19734	NS_000200.1	+	183874580	183874628
Sma-1-19735	NW_003037986.1	+	29300	29348
Sma-1-19736	NS_000200.1	+	215446052	215446100
Sma-1-19737	NW_003037904.1	-	393570	393522
Sma-1-19738	NS_000200.1	-	149108069	149108021
Sma-1-19739	NW_003037817.1	-	195688	195640
Sma-1-19740	NS_000200.1	-	47578040	47577992
Sma-1-19741	NW_003037791.1	+	239564	239612
Sma-1-19742	NS_000200.1	+	381944992	381945040
Sma-1-19743	NW_003037798.1	+	2538054	2538102
Sma-1-19744	NS_000200.1	+	16797212	16797260
Sma-1-19745	NW_003026465.1	-	113920	113872
Sma-1-19746	NS_000200.1	-	207506514	207506466
Sma-1-19747	NW_003037939.1	-	771382	771334
Sma-1-19748	NS_000200.1	-	183641136	183641088
Sma-1-19749	NW_003037924.1	+	176114	176162
Sma-1-19750	NS_000200.1	+	171883072	171883120
Sma-1-19751	NW_003037981.1	-	132216	132168
Sma-1-19752	NS_000200.1	-	212530084	212530036
Sma-1-19753	NW_003037834.1	+	153618	153666
Sma-1-19754	NS_000200.1	+	73314104	73314152
Sma-1-19755	NW_003037809.1	+	1501649	1501697
Sma-1-19756	NS_000200.1	+	35166113	35166161
Sma-1-19757	NW_003037800.1	+	293841	293889
Sma-1-19758	NS_000200.1	+	18923201	18923249
Sma-1-19759	NW_003026458.1	+	460346	460394
Sma-1-19760	NS_000200.1	+	102328576	102328624
Sma-1-19761	NW_003037981.1	-	133271	133223
Sma-1-19762	NS_000200.1	-	212531139	212531091
Sma-1-19763	NW_003037917.1	-	356140	356092
Sma-1-19764	NS_000200.1	-	165392944	165392896
Sma-1-19765	NW_003037809.1	+	1401547	1401595
Sma-1-19766	NS_000200.1	+	35066011	35066059
Sma-1-19767	NW_003037798.1	+	1686046	1686094
Sma-1-19768	NS_000200.1	+	15945204	15945252
Sma-1-19769	NW_003037800.1	+	624477	624525
Sma-1-19770	NS_000200.1	+	19253837	19253885

Sma-1-19771	NW_003037982.1	-	1356898	1356850
Sma-1-19772	NS_000200.1	-	215016093	215016045
Sma-1-19773	NW_003037800.1	-	918973	918925
Sma-1-19774	NS_000200.1	-	19548333	19548285
Sma-1-19775	NW_003037801.1	+	511406	511454
Sma-1-19776	NS_000200.1	+	21600886	21600934
Sma-1-19777	NW_003026459.1	+	155403	155451
Sma-1-19778	NS_000200.1	+	141800676	141800724
Sma-1-19779	NW_003037903.1	+	700175	700223
Sma-1-19780	NS_000200.1	+	148254256	148254304
Sma-1-19781	NW_003037808.1	-	353366	353318
Sma-1-19782	NS_000200.1	-	32140003	32139955
Sma-1-19783	NW_003037921.1	-	112800	112752
Sma-1-19784	NS_000200.1	-	169642935	169642887
Sma-1-19785	NW_003037793.1	-	1367780	1367732
Sma-1-19786	NS_000200.1	-	1367780	1367732
Sma-1-19787	NW_003037802.1	+	32284	32332
Sma-1-19788	NS_000200.1	+	22035078	22035126
Sma-1-19789	NW_003037981.1	+	514457	514505
Sma-1-19790	NS_000200.1	+	212912325	212912373
Sma-1-19791	NW_003037920.1	+	75672	75720
Sma-1-19792	NS_000200.1	+	169256479	169256527
Sma-1-19793	NW_003037803.1	+	1015763	1015811
Sma-1-19794	NS_000200.1	+	24516300	24516348
Sma-1-19795	NW_003026464.1	+	107277	107325
Sma-1-19796	NS_000200.1	+	203407586	203407634
Sma-1-19797	NW_003037793.1	+	2081343	2081391
Sma-1-19798	NS_000200.1	+	2081343	2081391
Sma-1-19799	NW_003037803.1	+	1319386	1319434
Sma-1-19800	NS_000200.1	+	24819923	24819971
Sma-1-19801	NW_003037803.1	+	1578512	1578560
Sma-1-19802	NS_000200.1	+	25079049	25079097
Sma-1-19803	NW_003037803.1	+	1606043	1606091
Sma-1-19804	NS_000200.1	+	25106580	25106628
Sma-1-19805	NW_003026609.1	+	29449	29498
Sma-1-19806	NS_000200.1	+	309684441	309684490
Sma-1-19807	NW_003037856.1	+	442674	442723
Sma-1-19808	NS_000200.1	+	99800630	99800679
Sma-1-19809	NW_003038027.1	-	216609	216560
Sma-1-19810	NS_000200.1	-	240587323	240587274
Sma-1-19811	NW_003038044.1	-	87210	87161
Sma-1-19812	NS_000200.1	-	254711992	254711943
Sma-1-19813	NW_003038037.1	-	1283477	1283428
Sma-1-19814	NS_000200.1	-	251438886	251438837
Sma-1-19815	NW_003037800.1	+	153303	153352
Sma-1-19816	NS_000200.1	+	18782663	18782712
Sma-1-19817	NW_003037918.1	+	94694	94743
Sma-1-19818	NS_000200.1	+	166661684	166661733
Sma-1-19819	NW_003037799.1	+	1415871	1415920
Sma-1-19820	NS_000200.1	+	18406225	18406274
Sma-1-19821	NW_003037942.1	+	183435	183488
Sma-1-19822	NS_000200.1	+	185943343	185943396

Sma-1-19823	NW_003036688.1	-	780	729
Sma-1-19824	NW_003037799.1	+	768349	768400
Sma-1-19825	NS_000200.1	+	17758703	17758754
Sma-1-19826	NS_000200.1	-	351536688	351536637
Sma-1-19827	NW_003037970.1	+	367303	367354
Sma-1-19828	NS_000200.1	+	201423493	201423544
Sma-1-19829	NW_003026460.1	-	398030	397977
Sma-1-19830	NS_000200.1	-	170920236	170920183
Sma-1-19831	NW_003037839.1	+	40968	41019
Sma-1-19832	NS_000200.1	+	78999118	78999169
Sma-1-19833	NW_003038073.1	+	11131	11182
Sma-1-19834	NS_000200.1	+	265533564	265533615
Sma-1-19835	NW_003037860.1	-	70153	70102
Sma-1-19836	NS_000200.1	-	103813181	103813130
Sma-1-19837	NW_003037940.1	-	72709	72658
Sma-1-19838	NS_000200.1	-	183814839	183814788
Sma-1-19839	NW_003038077.1	+	298987	299038
Sma-1-19840	NS_000200.1	+	267070627	267070678
Sma-1-19841	NW_003038502.1	+	101750	101801
Sma-1-19842	NS_000200.1	+	335100795	335100846
Sma-1-19843	NW_003037809.1	+	2099520	2099571
Sma-1-19844	NS_000200.1	+	35763984	35764035
Sma-1-19845	NW_003037915.1	-	1154197	1154149
Sma-1-19846	NS_000200.1	-	163054183	163054135
Sma-1-19847	NW_003037793.1	+	168445	168493
Sma-1-19848	NS_000200.1	+	168445	168493
Sma-1-19849	NW_003037824.1	+	949376	949424
Sma-1-19850	NS_000200.1	+	57880316	57880364
Sma-1-19851	NW_003026617.1	-	303518	303470
Sma-1-19852	NS_000200.1	-	319874377	319874329
Sma-1-19853	NW_003037837.1	+	127085	127133
Sma-1-19854	NS_000200.1	+	76932943	76932991
Sma-1-19855	NW_003026458.1	+	446717	446765
Sma-1-19856	NW_003037880.1	+	720651	720699
Sma-1-19857	NS_000200.1	+	102314947	102314995
Sma-1-19858	NS_000200.1	+	124404209	124404257
Sma-1-19859	NW_003028525.1	+	1312	1360
Sma-1-19860	NS_000200.1	+	374562059	374562107
Sma-1-19861	NW_003035881.1	+	115688	115736
Sma-1-19862	NS_000200.1	+	341179893	341179941
Sma-1-19863	NW_003037976.1	-	256278	256230
Sma-1-19864	NS_000200.1	-	206870593	206870545
Sma-1-19865	NW_003026489.1	-	29719	29671
Sma-1-19866	NS_000200.1	-	264254939	264254891
Sma-1-19867	NW_003037831.1	-	995259	995211
Sma-1-19868	NS_000200.1	-	70444561	70444513
Sma-1-19869	NW_003038157.1	-	334298	334250
Sma-1-19870	NS_000200.1	-	295585218	295585170
Sma-1-19871	NW_003037796.1	+	51843	51891
Sma-1-19872	NS_000200.1	+	8261487	8261535
Sma-1-19873	NW_003037896.1	-	316029	315981
Sma-1-19874	NS_000200.1	-	143236452	143236404

Sma-1-19875	NW_003037895.1	-	127504	127456
Sma-1-19876	NS_000200.1	-	142573706	142573658
Sma-1-19877	NW_003037853.1	+	501171	501219
Sma-1-19878	NS_000200.1	+	95993222	95993270
Sma-1-19879	NW_003037854.1	-	1005559	1005511
Sma-1-19880	NS_000200.1	-	97909980	97909932
Sma-1-19881	NW_003038067.1	+	109326	109374
Sma-1-19882	NS_000200.1	+	262963379	262963427
Sma-1-19883	NW_003038023.1	+	252374	252422
Sma-1-19884	NS_000200.1	+	237412665	237412713
Sma-1-19885	NW_003038247.1	+	60492	60540
Sma-1-19886	NS_000200.1	+	316009220	316009268
Sma-1-19887	NW_003038248.1	+	70378	70426
Sma-1-19888	NS_000200.1	+	316381645	316381693
Sma-1-19889	NW_003038032.1	+	662983	663031
Sma-1-19890	NS_000200.1	+	245822143	245822191
Sma-1-19891	NW_003037885.1	-	378956	378908
Sma-1-19892	NS_000200.1	-	128476903	128476855
Sma-1-19893	NW_003038443.1	+	11756	11804
Sma-1-19894	NS_000200.1	+	330041402	330041450
Sma-1-19895	NW_003037995.1	+	84192	84239
Sma-1-19896	NS_000200.1	+	219773960	219774007
Sma-1-19897	NW_003037798.1	-	2423467	2423420
Sma-1-19898	NW_003038037.1	+	1181644	1181691
Sma-1-19899	NS_000200.1	+	251337053	251337100
Sma-1-19900	NS_000200.1	-	16682625	16682578
Sma-1-19901	NW_003037916.1	-	706659	706612
Sma-1-19902	NS_000200.1	-	163900070	163900023
Sma-1-19903	NW_003037933.1	-	85678	85631
Sma-1-19904	NS_000200.1	-	178498850	178498803
Sma-1-19905	NW_003037818.1	-	1008291	1008244
Sma-1-19906	NS_000200.1	-	49723698	49723651
Sma-1-19907	NW_003036961.1	-	1871	1823
Sma-1-19908	NS_000200.1	-	343562244	343562196
Sma-1-19909	NW_003037917.1	-	812218	812170
Sma-1-19910	NS_000200.1	-	165849022	165848974
Sma-1-19911	NW_003037918.1	+	2048219	2048267
Sma-1-19912	NS_000200.1	+	168615209	168615257
Sma-1-19913	NW_003037804.1	+	974576	974624
Sma-1-19914	NW_003037804.1	+	992443	992491
Sma-1-19915	NS_000200.1	+	26114596	26114644
Sma-1-19916	NS_000200.1	+	26132463	26132511
Sma-1-19917	NW_003037917.1	+	560056	560104
Sma-1-19918	NS_000200.1	+	165596860	165596908
Sma-1-19919	NW_003037804.1	-	946765	946717
Sma-1-19920	NW_003037918.1	+	1301984	1302032
Sma-1-19921	NS_000200.1	+	167868974	167869022
Sma-1-19922	NS_000200.1	-	26086785	26086737
Sma-1-19923	NW_003038053.1	-	417229	417181
Sma-1-19924	NS_000200.1	-	257777324	257777276
Sma-1-19925	NW_003038079.1	-	105213	105165
Sma-1-19926	NS_000200.1	-	268235201	268235153

Sma-1-19927	NW_003037804.1	+	530818	530866
Sma-1-19928	NS_000200.1	+	25670838	25670886
Sma-1-19929	NW_003037804.1	-	527388	527340
Sma-1-19930	NS_000200.1	-	25667408	25667360
Sma-1-19931	NW_003037804.1	-	1361129	1361081
Sma-1-19932	NS_000200.1	-	26501149	26501101
Sma-1-19933	NW_003037804.1	+	1113640	1113688
Sma-1-19934	NS_000200.1	+	26253660	26253708
Sma-1-19935	NW_003037882.1	+	531994	532042
Sma-1-19936	NW_003038035.1	-	654667	654619
Sma-1-19937	NS_000200.1	+	125823668	125823716
Sma-1-19938	NS_000200.1	-	248516159	248516111
Sma-1-19939	NW_003039129.1	-	246860	246812
Sma-1-19940	NS_000200.1	-	229201668	229201620
Sma-1-19941	NW_003037802.1	+	754132	754180
Sma-1-19942	NW_003037824.1	-	634254	634206
Sma-1-19943	NW_003037880.1	-	432643	432595
Sma-1-19944	NS_000200.1	+	22756926	22756974
Sma-1-19945	NS_000200.1	-	124116201	124116153
Sma-1-19946	NS_000200.1	-	57565194	57565146
Sma-1-19947	NW_003037972.1	-	622634	622586
Sma-1-19948	NS_000200.1	-	203098338	203098290
Sma-1-19949	NW_003037800.1	-	1960116	1960068
Sma-1-19950	NS_000200.1	-	20589476	20589428
Sma-1-19951	NW_003037801.1	+	677807	677855
Sma-1-19952	NS_000200.1	+	21767287	21767335
Sma-1-19953	NW_003026575.1	-	62669	62621
Sma-1-19954	NS_000200.1	-	302294335	302294287
Sma-1-19955	NW_003037825.1	-	1663637	1663589
Sma-1-19956	NS_000200.1	-	60793419	60793371
Sma-1-19957	NW_003026618.1	+	212366	212414
Sma-1-19958	NS_000200.1	+	321967368	321967416
Sma-1-19959	NW_003026516.1	+	180265	180313
Sma-1-19960	NS_000200.1	+	299288562	299288610
Sma-1-19961	NW_003037890.1	+	1801372	1801420
Sma-1-19962	NS_000200.1	+	137484733	137484781
Sma-1-19963	NW_003038118.1	-	330594	330546
Sma-1-19964	NS_000200.1	-	277894721	277894673
Sma-1-19965	NW_003038212.1	+	13470	13518
Sma-1-19966	NS_000200.1	+	306177361	306177409
Sma-1-19967	NW_003038518.1	-	329325	329277
Sma-1-19968	NS_000200.1	-	341966158	341966110
Sma-1-19969	NW_003037793.1	-	4134685	4134637
Sma-1-19970	NS_000200.1	-	4134685	4134637
Sma-1-19971	NW_003035877.1	+	34539	34587
Sma-1-19972	NS_000200.1	+	337015073	337015121
Sma-1-19973	NW_003026516.1	-	215495	215447
Sma-1-19974	NS_000200.1	-	299323792	299323744
Sma-1-19975	NW_003026515.1	+	98886	98934
Sma-1-19976	NS_000200.1	+	290743297	290743345
Sma-1-19977	NW_003037904.1	-	466557	466509
Sma-1-19978	NS_000200.1	-	149181056	149181008

Sma-1-19979	NW_003037908.1	-	859042	858994
Sma-1-19980	NW_003037942.1	+	227444	227492
Sma-1-19981	NS_000200.1	+	185987352	185987400
Sma-1-19982	NS_000200.1	-	153332173	153332125
Sma-1-19983	NW_003037831.1	-	1695135	1695087
Sma-1-19984	NS_000200.1	-	71144437	71144389
Sma-1-19985	NW_003037880.1	+	288108	288156
Sma-1-19986	NS_000200.1	+	123971666	123971714
Sma-1-19987	NW_003038260.1	-	432682	432634
Sma-1-19988	NS_000200.1	-	323604457	323604409
Sma-1-19989	NW_003037946.1	+	882687	882735
Sma-1-19990	NS_000200.1	+	191576547	191576595
Sma-1-19991	NW_003037958.1	-	144788	144740
Sma-1-19992	NW_003037978.1	+	9772	9820
Sma-1-19993	NS_000200.1	+	209277782	209277830
Sma-1-19994	NS_000200.1	-	194808972	194808924
Sma-1-19995	NW_003038032.1	+	394902	394950
Sma-1-19996	NS_000200.1	+	245554062	245554110
Sma-1-19997	NW_003038000.1	-	125013	124965
Sma-1-19998	NS_000200.1	-	222595791	222595743
Sma-1-19999	NW_003037870.1	-	277450	277402
Sma-1-20000	NS_000200.1	-	117151115	117151067
Sma-1-20001	NW_003026460.1	-	146212	146164
Sma-1-20002	NW_003037986.1	-	111703	111655
Sma-1-20003	NW_003038506.1	-	237438	237390
Sma-1-20004	NS_000200.1	-	336599277	336599229
Sma-1-20005	NS_000200.1	-	215528455	215528407
Sma-1-20006	NS_000200.1	-	170668418	170668370
Sma-1-20007	NW_003037797.1	-	2571944	2571896
Sma-1-20008	NS_000200.1	-	12999804	12999756
Sma-1-20009	NW_003037900.1	-	231347	231299
Sma-1-20010	NS_000200.1	-	145360213	145360165
Sma-1-20011	NW_003037848.1	-	764232	764184
Sma-1-20012	NS_000200.1	-	90457303	90457255
Sma-1-20013	NW_003038059.1	+	187836	187884
Sma-1-20014	NS_000200.1	+	260429908	260429956
Sma-1-20015	NW_003038079.1	-	25191	25144
Sma-1-20016	NS_000200.1	-	268155179	268155132
Sma-1-20017	NW_003038502.1	+	146304	146352
Sma-1-20018	NS_000200.1	+	335145349	335145397
Sma-1-20019	NW_003037831.1	-	1694801	1694753
Sma-1-20020	NS_000200.1	-	71144103	71144055
Sma-1-20021	NW_003037917.1	-	1222828	1222780
Sma-1-20022	NS_000200.1	-	166259632	166259584
Sma-1-20023	NW_003037851.1	-	265509	265461
Sma-1-20024	NS_000200.1	-	93668819	93668771
Sma-1-20025	NW_003037928.1	+	5498	5546
Sma-1-20026	NS_000200.1	+	174329451	174329499
Sma-1-20027	NW_003037916.1	+	928996	929044
Sma-1-20028	NS_000200.1	+	164122407	164122455
Sma-1-20029	NW_003037924.1	-	259965	259917
Sma-1-20030	NS_000200.1	-	171966923	171966875

Sma-1-20031	NW_003026610.1	+	10252	10300
Sma-1-20032	NS_000200.1	+	310165622	310165670
Sma-1-20033	NW_003037793.1	+	3514873	3514921
Sma-1-20034	NW_003038022.1	-	285885	285837
Sma-1-20035	NS_000200.1	+	3514873	3514921
Sma-1-20036	NS_000200.1	-	235966727	235966679
Sma-1-20037	NW_003037962.1	+	149751	149799
Sma-1-20038	NW_003037829.1	-	619895	619847
Sma-1-20039	NW_003037975.1	+	578693	578741
Sma-1-20040	NW_003038503.1	+	66234	66282
Sma-1-20041	NS_000200.1	+	196226594	196226642
Sma-1-20042	NS_000200.1	+	206160917	206160965
Sma-1-20043	NS_000200.1	+	335592284	335592332
Sma-1-20044	NS_000200.1	-	66520433	66520385
Sma-1-20045	NW_003037800.1	-	1884848	1884800
Sma-1-20046	NS_000200.1	-	20514208	20514160
Sma-1-20047	NW_003037827.1	-	678204	678156
Sma-1-20048	NS_000200.1	-	63300345	63300297
Sma-1-20049	NW_003026615.1	-	133212	133164
Sma-1-20050	NW_003037863.1	+	576344	576392
Sma-1-20051	NS_000200.1	+	107334711	107334759
Sma-1-20052	NS_000200.1	-	317127653	317127605
Sma-1-20053	NW_003037928.1	-	771530	771482
Sma-1-20054	NS_000200.1	-	175095483	175095435
Sma-1-20055	NW_003037814.1	-	1411248	1411200
Sma-1-20056	NW_003037902.1	-	752898	752850
Sma-1-20057	NW_003037915.1	+	860101	860149
Sma-1-20058	NW_003038007.1	-	344328	344280
Sma-1-20059	NW_003038077.1	-	327361	327313
Sma-1-20060	NS_000200.1	+	162760087	162760135
Sma-1-20061	NS_000200.1	-	267099001	267098953
Sma-1-20062	NS_000200.1	-	225592883	225592835
Sma-1-20063	NS_000200.1	-	147508132	147508084
Sma-1-20064	NS_000200.1	-	44395108	44395060
Sma-1-20065	NW_003037796.1	+	260509	260557
Sma-1-20066	NS_000200.1	+	8470153	8470201
Sma-1-20067	NW_003037800.1	-	1020647	1020599
Sma-1-20068	NS_000200.1	-	19650007	19649959
Sma-1-20069	NW_003037925.1	+	342091	342139
Sma-1-20070	NS_000200.1	+	172744094	172744142
Sma-1-20071	NW_003037809.1	+	2231013	2231061
Sma-1-20072	NW_003037910.1	-	987549	987501
Sma-1-20073	NS_000200.1	+	35895477	35895525
Sma-1-20074	NS_000200.1	-	155499524	155499476
Sma-1-20075	NW_003037799.1	-	714158	714110
Sma-1-20076	NS_000200.1	-	17704512	17704464
Sma-1-20077	NW_003037916.1	+	1735333	1735381
Sma-1-20078	NS_000200.1	+	164928744	164928792
Sma-1-20079	NW_003037931.1	-	641474	641426
Sma-1-20080	NS_000200.1	-	177474141	177474093
Sma-1-20081	NW_003038016.1	-	376192	376141
Sma-1-20082	NW_003038016.1	-	363371	363320

Sma-1-20083	NS_000200.1	-	231492729	231492678
Sma-1-20084	NS_000200.1	-	231479908	231479857
Sma-1-20085	NW_003038224.1	-	113268	113217
Sma-1-20086	NS_000200.1	-	309524996	309524945
Sma-1-20087	NW_003037793.1	+	4157763	4157814
Sma-1-20088	NS_000200.1	+	4157763	4157814
Sma-1-20089	NW_003037881.1	-	416240	416189
Sma-1-20090	NS_000200.1	-	124848031	124847980
Sma-1-20091	NW_003038010.1	-	554577	554526
Sma-1-20092	NS_000200.1	-	227372800	227372749
Sma-1-20093	NW_003032160.1	-	226462	226411
Sma-1-20094	NS_000200.1	-	381234548	381234497
Sma-1-20095	NW_003037851.1	+	890100	890151
Sma-1-20096	NS_000200.1	+	94293410	94293461
Sma-1-20097	NW_003037902.1	-	144567	144516
Sma-1-20098	NS_000200.1	-	146899801	146899750
Sma-1-20099	NW_003037889.1	+	1323185	1323236
Sma-1-20100	NS_000200.1	+	135099675	135099726
Sma-1-20101	NW_003038044.1	+	116995	117046
Sma-1-20102	NS_000200.1	+	254741777	254741828
Sma-1-20103	NW_003038044.1	-	123568	123517
Sma-1-20104	NS_000200.1	-	254748350	254748299
Sma-1-20105	NW_003037846.1	+	172523	172574
Sma-1-20106	NS_000200.1	+	87807093	87807144
Sma-1-20107	NW_003037781.1	-	1748	1697
Sma-1-20108	NS_000200.1	-	379082168	379082117
Sma-1-20109	NW_003037802.1	-	1358559	1358508
Sma-1-20110	NS_000200.1	-	23361353	23361302
Sma-1-20111	NW_003038517.1	+	248521	248572
Sma-1-20112	NS_000200.1	+	341013009	341013060
Sma-1-20113	NW_003038141.1	+	289305	289356
Sma-1-20114	NS_000200.1	+	287179284	287179335
Sma-1-20115	NW_003037809.1	+	1522856	1522907
Sma-1-20116	NS_000200.1	+	35187320	35187371
Sma-1-20117	NW_003035681.1	+	35869	35920
Sma-1-20118	NW_003035681.1	+	55216	55267
Sma-1-20119	NS_000200.1	+	332047309	332047360
Sma-1-20120	NS_000200.1	+	332066656	332066707
Sma-1-20121	NW_003032160.1	+	51201	51252
Sma-1-20122	NW_003032160.1	-	49223	49172
Sma-1-20123	NW_003037899.1	+	496036	496087
Sma-1-20124	NS_000200.1	+	145015147	145015198
Sma-1-20125	NS_000200.1	+	381059287	381059338
Sma-1-20126	NS_000200.1	-	381057309	381057258
Sma-1-20127	NW_003026616.1	+	82303	82354
Sma-1-20128	NS_000200.1	+	317438902	317438953
Sma-1-20129	NW_003026516.1	+	491653	491704
Sma-1-20130	NS_000200.1	+	299599950	299600001
Sma-1-20131	NW_003038143.1	-	230666	230615
Sma-1-20132	NS_000200.1	-	287990158	287990107
Sma-1-20133	NW_003038249.1	-	238344	238293
Sma-1-20134	NS_000200.1	-	316920597	316920546

Sma-1-20135	NW_003038249.1	-	87235	87184
Sma-1-20136	NS_000200.1	-	316769488	316769437
Sma-1-20137	NW_003034233.1	+	2343	2394
Sma-1-20138	NW_003026469.1	+	158273	158324
Sma-1-20139	NW_003037914.1	+	1600862	1600913
Sma-1-20140	NW_003038507.1	-	15793	15742
Sma-1-20141	NW_003038077.1	+	110640	110691
Sma-1-20142	NS_000200.1	+	161745112	161745163
Sma-1-20143	NS_000200.1	+	217779932	217779983
Sma-1-20144	NS_000200.1	+	266882280	266882331
Sma-1-20145	NS_000200.1	+	326601348	326601399
Sma-1-20146	NS_000200.1	-	336684687	336684636
Sma-1-20147	NW_003038038.1	-	989949	989899
Sma-1-20148	NS_000200.1	-	252893342	252893292
Sma-1-20149	NW_003037915.1	+	843546	843594
Sma-1-20150	NS_000200.1	+	162743532	162743580
Sma-1-20151	NW_003037810.1	+	1105391	1105439
Sma-1-20152	NS_000200.1	+	37709913	37709961
Sma-1-20153	NW_003037810.1	-	1136661	1136613
Sma-1-20154	NW_003026510.1	-	238418	238370
Sma-1-20155	NS_000200.1	-	283678599	283678551
Sma-1-20156	NS_000200.1	-	37741183	37741135
Sma-1-20157	NW_003037964.1	+	481915	481963
Sma-1-20158	NS_000200.1	+	198083437	198083485
Sma-1-20159	NW_003037898.1	-	383293	383245
Sma-1-20160	NW_003037872.1	+	24403	24451
Sma-1-20161	NS_000200.1	+	117981409	117981457
Sma-1-20162	NS_000200.1	-	144367115	144367067
Sma-1-20163	NW_003026471.1	-	74037	73989
Sma-1-20164	NS_000200.1	-	222186270	222186222
Sma-1-20165	NW_003037812.1	-	1260287	1260239
Sma-1-20166	NS_000200.1	-	40743026	40742978
Sma-1-20167	NW_003037812.1	-	1261083	1261035
Sma-1-20168	NW_003038041.1	-	75385	75337
Sma-1-20169	NS_000200.1	-	253742667	253742619
Sma-1-20170	NS_000200.1	-	40743822	40743774
Sma-1-20171	NW_003037935.1	+	439589	439637
Sma-1-20172	NS_000200.1	+	179856124	179856172
Sma-1-20173	NW_003030309.1	+	462	510
Sma-1-20174	NS_000200.1	+	362644069	362644117
Sma-1-20175	NW_003037962.1	+	265915	265963
Sma-1-20176	NS_000200.1	+	196342758	196342806
Sma-1-20177	NW_003037915.1	-	135060	135012
Sma-1-20178	NS_000200.1	-	162035046	162034998
Sma-1-20179	NW_003037813.1	+	1331489	1331537
Sma-1-20180	NS_000200.1	+	42318352	42318400
Sma-1-20181	NW_003037795.1	+	1135240	1135288
Sma-1-20182	NS_000200.1	+	7526123	7526171
Sma-1-20183	NW_003037814.1	+	13784	13832
Sma-1-20184	NS_000200.1	+	42997644	42997692
Sma-1-20185	NW_003037803.1	+	1582648	1582696
Sma-1-20186	NS_000200.1	+	25083185	25083233

Sma-1-20187	NW_003037971.1	+	310700	310748
Sma-1-20188	NS_000200.1	+	201932750	201932798
Sma-1-20189	NW_003037703.1	+	279	327
Sma-1-20190	NS_000200.1	+	375582281	375582329
Sma-1-20191	NW_003037814.1	-	1084546	1084498
Sma-1-20192	NS_000200.1	-	44068406	44068358
Sma-1-20193	NW_003037814.1	-	1093414	1093366
Sma-1-20194	NS_000200.1	-	44077274	44077226
Sma-1-20195	NW_003037794.1	-	1803966	1803918
Sma-1-20196	NW_003038515.1	+	335953	336001
Sma-1-20197	NS_000200.1	+	340420018	340420066
Sma-1-20198	NS_000200.1	-	5983561	5983513
Sma-1-20199	NW_003037793.1	-	3303691	3303643
Sma-1-20200	NS_000200.1	-	3303691	3303643
Sma-1-20201	NW_003037814.1	-	2061791	2061743
Sma-1-20202	NS_000200.1	-	45045651	45045603
Sma-1-20203	NW_003037814.1	+	227665	227713
Sma-1-20204	NS_000200.1	+	43211525	43211573
Sma-1-20205	NW_003026460.1	-	368184	368136
Sma-1-20206	NW_003038026.1	-	488792	488744
Sma-1-20207	NS_000200.1	-	239996553	239996505
Sma-1-20208	NS_000200.1	-	170890390	170890342
Sma-1-20209	NW_003037940.1	+	471012	471060
Sma-1-20210	NS_000200.1	+	184213142	184213190
Sma-1-20211	NW_003037942.1	+	521406	521454
Sma-1-20212	NS_000200.1	+	186281314	186281362
Sma-1-20213	NW_003037793.1	-	2592068	2592020
Sma-1-20214	NS_000200.1	-	2592068	2592020
Sma-1-20215	NW_003037814.1	-	602608	602560
Sma-1-20216	NS_000200.1	-	43586468	43586420
Sma-1-20217	NW_003037939.1	-	430814	430766
Sma-1-20218	NS_000200.1	-	183300568	183300520
Sma-1-20219	NW_003037792.1	-	454975	454927
Sma-1-20220	NW_003037824.1	+	615183	615231
Sma-1-20221	NS_000200.1	+	57546123	57546171
Sma-1-20222	NS_000200.1	-	382892319	382892271
Sma-1-20223	NW_003037958.1	-	337489	337441
Sma-1-20224	NS_000200.1	-	195001673	195001625
Sma-1-20225	NW_003037918.1	-	92603	92555
Sma-1-20226	NS_000200.1	-	166659593	166659545
Sma-1-20227	NW_003037962.1	+	387419	387467
Sma-1-20228	NS_000200.1	+	196464262	196464310
Sma-1-20229	NW_003038018.1	-	325998	325950
Sma-1-20230	NS_000200.1	-	232726848	232726800
Sma-1-20231	NW_003035734.1	-	2326	2278
Sma-1-20232	NW_003035849.1	-	1023	975
Sma-1-20233	NW_003037937.1	-	135024	134976
Sma-1-20234	NW_003037995.1	+	91893	91941
Sma-1-20235	NW_003037995.1	+	256469	256517
Sma-1-20236	NW_003037809.1	-	1009710	1009662
Sma-1-20237	NW_003037825.1	-	1429513	1429465
Sma-1-20238	NW_003037838.1	-	891741	891693

Sma-1-20239	NW_003037860.1	-	449751	449703
Sma-1-20240	NW_003037872.1	+	59704	59752
Sma-1-20241	NW_003037887.1	-	1573022	1572974
Sma-1-20242	NW_003037918.1	-	1990693	1990645
Sma-1-20243	NW_003037923.1	+	100334	100382
Sma-1-20244	NW_003037931.1	+	317447	317495
Sma-1-20245	NW_003037932.1	+	705486	705534
Sma-1-20246	NW_003037941.1	+	701846	701894
Sma-1-20247	NW_003037981.1	+	342252	342300
Sma-1-20248	NW_003037981.1	-	139165	139117
Sma-1-20249	NW_003038227.1	+	275580	275628
Sma-1-20250	NW_003039127.1	-	599531	599483
Sma-1-20251	NW_003026483.1	-	50809	50761
Sma-1-20252	NW_003038042.1	-	153775	153727
Sma-1-20253	NW_003038153.1	-	554871	554823
Sma-1-20254	NS_000200.1	+	118016710	118016758
Sma-1-20255	NS_000200.1	+	171156547	171156595
Sma-1-20256	NS_000200.1	+	177150114	177150162
Sma-1-20257	NS_000200.1	+	178238682	178238730
Sma-1-20258	NS_000200.1	+	185436320	185436368
Sma-1-20259	NS_000200.1	+	212740120	212740168
Sma-1-20260	NS_000200.1	+	219781661	219781709
Sma-1-20261	NS_000200.1	+	219946237	219946285
Sma-1-20262	NS_000200.1	+	310556934	310556982
Sma-1-20263	NS_000200.1	-	364222474	364222426
Sma-1-20264	NS_000200.1	-	363971559	363971511
Sma-1-20265	NS_000200.1	-	293616920	293616872
Sma-1-20266	NS_000200.1	-	256565333	256565285
Sma-1-20267	NS_000200.1	-	254099374	254099326
Sma-1-20268	NS_000200.1	-	212537033	212536985
Sma-1-20269	NS_000200.1	-	181334933	181334885
Sma-1-20270	NS_000200.1	-	168557683	168557635
Sma-1-20271	NS_000200.1	-	132037000	132036952
Sma-1-20272	NS_000200.1	-	113677030	113676982
Sma-1-20273	NS_000200.1	-	104192779	104192731
Sma-1-20274	NS_000200.1	-	78890304	78890256
Sma-1-20275	NS_000200.1	-	60559295	60559247
Sma-1-20276	NS_000200.1	-	34674174	34674126
Sma-1-20277	NW_003037815.1	-	606106	606058
Sma-1-20278	NW_003037822.1	+	775835	775883
Sma-1-20279	NW_003037844.1	+	456069	456117
Sma-1-20280	NW_003038083.1	-	9584	9536
Sma-1-20281	NS_000200.1	+	55083841	55083889
Sma-1-20282	NS_000200.1	+	86639515	86639563
Sma-1-20283	NS_000200.1	-	270252921	270252873
Sma-1-20284	NS_000200.1	-	45869097	45869049
Sma-1-20285	NW_003037817.1	-	484698	484650
Sma-1-20286	NW_003038033.1	+	146057	146105
Sma-1-20287	NS_000200.1	+	246126059	246126107
Sma-1-20288	NS_000200.1	-	47867050	47867002
Sma-1-20289	NW_003035810.1	+	1286	1334
Sma-1-20290	NW_003037815.1	+	832789	832837

Sma-1-20291	NS_000200.1	+	46095780	46095828
Sma-1-20292	NS_000200.1	+	364135410	364135458
Sma-1-20293	NW_003038230.1	-	246142	246094
Sma-1-20294	NS_000200.1	-	311531713	311531665
Sma-1-20295	NW_003037827.1	+	181498	181546
Sma-1-20296	NS_000200.1	+	62803639	62803687
Sma-1-20297	NW_003037896.1	-	488454	488404
Sma-1-20298	NS_000200.1	-	143408877	143408827
Sma-1-20299	NW_003037874.1	+	58988	59036
Sma-1-20300	NS_000200.1	+	119483938	119483986
Sma-1-20301	NW_003029267.1	-	503	455
Sma-1-20302	NS_000200.1	-	357192321	357192273
Sma-1-20303	NW_003037945.1	-	998570	998522
Sma-1-20304	NS_000200.1	-	190100534	190100486
Sma-1-20305	NW_003038027.1	-	330859	330811
Sma-1-20306	NS_000200.1	-	240701573	240701525
Sma-1-20307	NW_003035880.1	+	5730	5778
Sma-1-20308	NS_000200.1	+	339246794	339246842
Sma-1-20309	NW_003037951.1	+	134657	134705
Sma-1-20310	NS_000200.1	+	192812382	192812430
Sma-1-20311	NW_003037910.1	-	943479	943431
Sma-1-20312	NS_000200.1	-	155455454	155455406
Sma-1-20313	NW_003037821.1	+	703284	703332
Sma-1-20314	NS_000200.1	+	53847768	53847816
Sma-1-20315	NW_003037910.1	-	1201251	1201203
Sma-1-20316	NW_003038157.1	-	630695	630647
Sma-1-20317	NS_000200.1	-	295881615	295881567
Sma-1-20318	NS_000200.1	-	155713226	155713178
Sma-1-20319	NW_003037822.1	-	3357	3309
Sma-1-20320	NS_000200.1	-	54311363	54311315
Sma-1-20321	NW_003037964.1	-	657394	657346
Sma-1-20322	NS_000200.1	-	198258916	198258868
Sma-1-20323	NW_003037908.1	-	992499	992451
Sma-1-20324	NS_000200.1	-	153465630	153465582
Sma-1-20325	NW_003029072.1	-	961	913
Sma-1-20326	NS_000200.1	-	375331226	375331178
Sma-1-20327	NW_003037824.1	+	973910	973958
Sma-1-20328	NS_000200.1	+	57904850	57904898
Sma-1-20329	NW_003037970.1	-	215209	215161
Sma-1-20330	NS_000200.1	-	201271399	201271351
Sma-1-20331	NW_003037971.1	+	405703	405751
Sma-1-20332	NS_000200.1	+	202027753	202027801
Sma-1-20333	NW_003026617.1	-	33193	33145
Sma-1-20334	NS_000200.1	-	319604052	319604004
Sma-1-20335	NW_003037902.1	+	364600	364648
Sma-1-20336	NS_000200.1	+	147119834	147119882
Sma-1-20337	NW_003038131.1	-	178565	178517
Sma-1-20338	NS_000200.1	-	282140409	282140361
Sma-1-20339	NW_003037896.1	+	172469	172517
Sma-1-20340	NS_000200.1	+	143092892	143092940
Sma-1-20341	NW_003026615.1	+	93385	93433
Sma-1-20342	NS_000200.1	+	317087826	317087874

Sma-1-20343	NW_003037866.1	-	433155	433107
Sma-1-20344	NS_000200.1	-	111920954	111920906
Sma-1-20345	NW_003038011.1	+	541245	541293
Sma-1-20346	NS_000200.1	+	228068895	228068943
Sma-1-20347	NW_003037829.1	-	1510413	1510365
Sma-1-20348	NW_003039127.1	-	509757	509709
Sma-1-20349	NW_003038132.1	+	336697	336745
Sma-1-20350	NS_000200.1	+	282757167	282757215
Sma-1-20351	NS_000200.1	-	113587256	113587208
Sma-1-20352	NS_000200.1	-	67410951	67410903
Sma-1-20353	NW_003034051.1	-	268	220
Sma-1-20354	NW_003037797.1	-	288091	288043
Sma-1-20355	NS_000200.1	-	363610785	363610737
Sma-1-20356	NS_000200.1	-	10715951	10715903
Sma-1-20357	NW_003037942.1	+	653668	653716
Sma-1-20358	NS_000200.1	+	186413576	186413624
Sma-1-20359	NW_003037826.1	+	590429	590477
Sma-1-20360	NS_000200.1	+	61589945	61589993
Sma-1-20361	NW_003037827.1	+	1144166	1144214
Sma-1-20362	NW_003038039.1	-	86460	86412
Sma-1-20363	NS_000200.1	+	63766307	63766355
Sma-1-20364	NS_000200.1	-	253524306	253524258
Sma-1-20365	NW_003037834.1	+	890035	890083
Sma-1-20366	NS_000200.1	+	74050521	74050569
Sma-1-20367	NW_003037981.1	-	16212	16164
Sma-1-20368	NS_000200.1	-	212414080	212414032
Sma-1-20369	NW_003037973.1	-	120209	120161
Sma-1-20370	NS_000200.1	-	204101936	204101888
Sma-1-20371	NW_003037974.1	+	721556	721604
Sma-1-20372	NS_000200.1	+	205396862	205396910
Sma-1-20373	NW_003026597.1	-	146686	146638
Sma-1-20374	NS_000200.1	-	305767872	305767824
Sma-1-20375	NW_003026517.1	+	600520	600568
Sma-1-20376	NS_000200.1	+	300206529	300206577
Sma-1-20377	NW_003037827.1	-	699699	699651
Sma-1-20378	NS_000200.1	-	63321840	63321792
Sma-1-20379	NW_003037907.1	+	611284	611332
Sma-1-20380	NS_000200.1	+	152015773	152015821
Sma-1-20381	NW_003037829.1	+	1101831	1101879
Sma-1-20382	NS_000200.1	+	67002369	67002417
Sma-1-20383	NW_003026472.1	+	608348	608396
Sma-1-20384	NS_000200.1	+	237020340	237020388
Sma-1-20385	NW_003037830.1	-	86610	86562
Sma-1-20386	NS_000200.1	-	67976981	67976933
Sma-1-20387	NW_003026469.1	+	147353	147401
Sma-1-20388	NS_000200.1	+	217769012	217769060
Sma-1-20389	NW_003037979.1	+	95723	95771
Sma-1-20390	NW_003038505.1	+	18894	18942
Sma-1-20391	NS_000200.1	+	210156743	210156791
Sma-1-20392	NS_000200.1	+	336098231	336098279
Sma-1-20393	NW_003026465.1	+	884352	884400
Sma-1-20394	NS_000200.1	+	208276946	208276994

Sma-1-20395	NW_003037831.1	+	1652332	1652380
Sma-1-20396	NW_003037838.1	-	174344	174296
Sma-1-20397	NW_003037897.1	-	68436	68388
Sma-1-20398	NW_003037933.1	+	577644	577692
Sma-1-20399	NW_003037987.1	-	30385	30337
Sma-1-20400	NW_003038031.1	+	509597	509645
Sma-1-20401	NW_003038037.1	+	1166905	1166953
Sma-1-20402	NS_000200.1	+	71101634	71101682
Sma-1-20403	NS_000200.1	+	178990816	178990864
Sma-1-20404	NS_000200.1	+	244787355	244787403
Sma-1-20405	NS_000200.1	+	251322314	251322362
Sma-1-20406	NS_000200.1	-	215783006	215782958
Sma-1-20407	NS_000200.1	-	143550812	143550764
Sma-1-20408	NS_000200.1	-	78172907	78172859
Sma-1-20409	NW_003026465.1	+	533550	533598
Sma-1-20410	NS_000200.1	+	207926144	207926192
Sma-1-20411	NW_003037839.1	+	1149134	1149182
Sma-1-20412	NS_000200.1	+	80107284	80107332
Sma-1-20413	NW_003038249.1	+	259940	259988
Sma-1-20414	NS_000200.1	+	316942193	316942241
Sma-1-20415	NW_003037935.1	-	306733	306685
Sma-1-20416	NW_003037941.1	+	960657	960705
Sma-1-20417	NW_003038087.1	+	417472	417520
Sma-1-20418	NS_000200.1	+	185695131	185695179
Sma-1-20419	NS_000200.1	+	273445873	273445921
Sma-1-20420	NS_000200.1	-	179723268	179723220
Sma-1-20421	NW_003038145.1	+	194212	194260
Sma-1-20422	NS_000200.1	+	288805635	288805683
Sma-1-20423	NW_003037852.1	+	776036	776084
Sma-1-20424	NS_000200.1	+	95167931	95167979
Sma-1-20425	NW_003026456.1	+	1036009	1036057
Sma-1-20426	NW_003037935.1	+	764987	765035
Sma-1-20427	NS_000200.1	+	58959145	58959193
Sma-1-20428	NS_000200.1	+	180181522	180181570
Sma-1-20429	NW_003038250.1	-	51448	51400
Sma-1-20430	NS_000200.1	-	318104125	318104077
Sma-1-20431	NW_003024620.1	-	1450	1402
Sma-1-20432	NW_003030267.1	+	216	264
Sma-1-20433	NW_003031917.1	-	752	704
Sma-1-20434	NW_003033790.1	+	4171	4219
Sma-1-20435	NW_003034089.1	+	919	967
Sma-1-20436	NW_003035788.1	-	970	922
Sma-1-20437	NW_003035874.1	+	33929	33977
Sma-1-20438	NW_003037874.1	+	379964	380012
Sma-1-20439	NW_003037898.1	+	472629	472677
Sma-1-20440	NW_003038005.1	+	406850	406898
Sma-1-20441	NW_003026619.1	+	254716	254764
Sma-1-20442	NW_003037793.1	+	1467308	1467356
Sma-1-20443	NW_003037795.1	-	1111677	1111629
Sma-1-20444	NW_003037798.1	-	1367266	1367218
Sma-1-20445	NW_003037818.1	+	2214507	2214555
Sma-1-20446	NW_003037822.1	+	1379889	1379937

Sma-1-20447	NW_003037835.1	-	687440	687392
Sma-1-20448	NW_003037840.1	+	545581	545629
Sma-1-20449	NW_003037840.1	-	1463832	1463784
Sma-1-20450	NW_003037841.1	-	177318	177270
Sma-1-20451	NW_003037844.1	+	56435	56483
Sma-1-20452	NW_003037886.1	-	1063280	1063232
Sma-1-20453	NW_003037890.1	+	287273	287321
Sma-1-20454	NW_003037912.1	+	595646	595694
Sma-1-20455	NW_003037917.1	-	967349	967301
Sma-1-20456	NW_003037933.1	+	615715	615763
Sma-1-20457	NW_003037941.1	+	946463	946511
Sma-1-20458	NW_003037953.1	+	194422	194470
Sma-1-20459	NW_003037976.1	-	574519	574471
Sma-1-20460	NW_003038015.1	-	233107	233059
Sma-1-20461	NW_003038029.1	+	783323	783371
Sma-1-20462	NW_003038029.1	-	789502	789454
Sma-1-20463	NW_003038033.1	-	436594	436546
Sma-1-20464	NW_003038033.1	-	430944	430896
Sma-1-20465	NW_003038037.1	-	328964	328916
Sma-1-20466	NW_003039127.1	-	1982826	1982778
Sma-1-20467	NW_003038151.1	+	470828	470876
Sma-1-20468	NS_000200.1	+	1467308	1467356
Sma-1-20469	NS_000200.1	+	50929914	50929962
Sma-1-20470	NS_000200.1	+	55687895	55687943
Sma-1-20471	NS_000200.1	+	81168905	81168953
Sma-1-20472	NS_000200.1	+	86239881	86239929
Sma-1-20473	NS_000200.1	+	119804914	119804962
Sma-1-20474	NS_000200.1	+	135970634	135970682
Sma-1-20475	NS_000200.1	+	144456451	144456499
Sma-1-20476	NS_000200.1	+	157407221	157407269
Sma-1-20477	NS_000200.1	+	179028887	179028935
Sma-1-20478	NS_000200.1	+	185680937	185680985
Sma-1-20479	NS_000200.1	+	193107473	193107521
Sma-1-20480	NS_000200.1	+	224812804	224812852
Sma-1-20481	NS_000200.1	+	242984224	242984272
Sma-1-20482	NS_000200.1	+	292378071	292378119
Sma-1-20483	NS_000200.1	+	322445460	322445508
Sma-1-20484	NS_000200.1	+	336011473	336011521
Sma-1-20485	NS_000200.1	+	358984457	358984505
Sma-1-20486	NS_000200.1	+	362535791	362535839
Sma-1-20487	NS_000200.1	+	363711150	363711198
Sma-1-20488	NS_000200.1	-	367268853	367268805
Sma-1-20489	NS_000200.1	-	364092627	364092579
Sma-1-20490	NS_000200.1	-	348959615	348959567
Sma-1-20491	NS_000200.1	-	250484373	250484325
Sma-1-20492	NS_000200.1	-	246416596	246416548
Sma-1-20493	NS_000200.1	-	246410946	246410898
Sma-1-20494	NS_000200.1	-	242990403	242990355
Sma-1-20495	NS_000200.1	-	230556597	230556549
Sma-1-20496	NS_000200.1	-	207188834	207188786
Sma-1-20497	NS_000200.1	-	166004153	166004105
Sma-1-20498	NS_000200.1	-	130318847	130318799

Sma-1-20499	NS_000200.1	-	115060325	115060277
Sma-1-20500	NS_000200.1	-	82788499	82788451
Sma-1-20501	NS_000200.1	-	82087156	82087108
Sma-1-20502	NS_000200.1	-	75083948	75083900
Sma-1-20503	NS_000200.1	-	15626424	15626376
Sma-1-20504	NS_000200.1	-	7502560	7502512
Sma-1-20505	NW_003037833.1	+	949597	949645
Sma-1-20506	NW_003037833.1	-	950189	950141
Sma-1-20507	NS_000200.1	+	73140693	73140741
Sma-1-20508	NS_000200.1	-	73141285	73141237
Sma-1-20509	NW_003037982.1	+	693321	693369
Sma-1-20510	NS_000200.1	+	214352516	214352564
Sma-1-20511	NW_003037902.1	+	378211	378259
Sma-1-20512	NS_000200.1	+	147133445	147133493
Sma-1-20513	NW_003037834.1	+	258201	258249
Sma-1-20514	NS_000200.1	+	73418687	73418735
Sma-1-20515	NW_003037984.1	-	51719	51671
Sma-1-20516	NW_003037955.1	-	355681	355633
Sma-1-20517	NS_000200.1	-	215122807	215122759
Sma-1-20518	NS_000200.1	-	193982415	193982367
Sma-1-20519	NW_003037836.1	+	154247	154295
Sma-1-20520	NS_000200.1	+	75711346	75711394
Sma-1-20521	NW_003025565.1	+	176	224
Sma-1-20522	NW_003025565.1	-	631	583
Sma-1-20523	NS_000200.1	+	356168019	356168067
Sma-1-20524	NS_000200.1	-	356168474	356168426
Sma-1-20525	NW_003037844.1	-	36488	36440
Sma-1-20526	NS_000200.1	-	86219934	86219886
Sma-1-20527	NW_003037794.1	-	398863	398815
Sma-1-20528	NW_003037904.1	+	381347	381395
Sma-1-20529	NS_000200.1	+	149095846	149095894
Sma-1-20530	NS_000200.1	-	4578458	4578410
Sma-1-20531	NW_003035163.1	+	1252	1300
Sma-1-20532	NW_003037825.1	-	279765	279717
Sma-1-20533	NW_003037826.1	+	654645	654693
Sma-1-20534	NW_003037840.1	-	1921201	1921153
Sma-1-20535	NW_003037864.1	-	1790244	1790196
Sma-1-20536	NW_003037918.1	-	1057856	1057808
Sma-1-20537	NW_003037958.1	-	294288	294240
Sma-1-20538	NW_003037977.1	-	465542	465494
Sma-1-20539	NW_003038050.1	+	58305	58353
Sma-1-20540	NS_000200.1	+	61654161	61654209
Sma-1-20541	NS_000200.1	+	256333199	256333247
Sma-1-20542	NS_000200.1	+	370719636	370719684
Sma-1-20543	NS_000200.1	-	208751562	208751514
Sma-1-20544	NS_000200.1	-	194958472	194958424
Sma-1-20545	NS_000200.1	-	167624846	167624798
Sma-1-20546	NS_000200.1	-	110282818	110282770
Sma-1-20547	NS_000200.1	-	82544525	82544477
Sma-1-20548	NS_000200.1	-	59409547	59409499
Sma-1-20549	NW_003038130.1	+	57474	57523
Sma-1-20550	NS_000200.1	+	281612460	281612509

Sma-1-20551	NW_003037901.1	+	584054	584102
Sma-1-20552	NS_000200.1	+	146432515	146432563
Sma-1-20553	NW_003037837.1	+	1124388	1124436
Sma-1-20554	NS_000200.1	+	77930246	77930294
Sma-1-20555	NW_003023600.1	+	2480	2528
Sma-1-20556	NW_003026583.1	+	86242	86290
Sma-1-20557	NW_003035869.1	-	113188	113140
Sma-1-20558	NW_003037789.1	+	35567	35615
Sma-1-20559	NW_003037789.1	-	39915	39867
Sma-1-20560	NS_000200.1	+	303425870	303425918
Sma-1-20561	NS_000200.1	+	373805029	373805077
Sma-1-20562	NS_000200.1	+	380820187	380820235
Sma-1-20563	NS_000200.1	-	380824535	380824487
Sma-1-20564	NS_000200.1	-	335297388	335297340
Sma-1-20565	NW_003037837.1	+	404728	404776
Sma-1-20566	NS_000200.1	+	77210586	77210634
Sma-1-20567	NW_003037882.1	-	724751	724703
Sma-1-20568	NW_003037912.1	+	837146	837194
Sma-1-20569	NW_003038471.1	+	70718	70766
Sma-1-20570	NS_000200.1	+	157648721	157648769
Sma-1-20571	NS_000200.1	+	331911632	331911680
Sma-1-20572	NS_000200.1	-	126016425	126016377
Sma-1-20573	NW_003038259.1	+	69570	69618
Sma-1-20574	NS_000200.1	+	322930169	322930217
Sma-1-20575	NW_003023144.1	+	894	942
Sma-1-20576	NS_000200.1	+	368372020	368372068
Sma-1-20577	NW_003037898.1	+	191697	191745
Sma-1-20578	NS_000200.1	+	144175519	144175567
Sma-1-20579	NW_003037840.1	-	473759	473711
Sma-1-20580	NW_003037868.1	-	75741	75693
Sma-1-20581	NS_000200.1	-	116047453	116047405
Sma-1-20582	NS_000200.1	-	81097083	81097035
Sma-1-20583	NW_003037841.1	+	1254935	1254983
Sma-1-20584	NS_000200.1	+	83866116	83866164
Sma-1-20585	NW_003038007.1	+	360991	361039
Sma-1-20586	NS_000200.1	+	225609546	225609594
Sma-1-20587	NW_003038056.1	+	160532	160580
Sma-1-20588	NS_000200.1	+	259138183	259138231
Sma-1-20589	NW_003037841.1	+	1741346	1741394
Sma-1-20590	NS_000200.1	+	84352527	84352575
Sma-1-20591	NW_003037896.1	-	214510	214462
Sma-1-20592	NS_000200.1	-	143134933	143134885
Sma-1-20593	NW_003037891.1	+	1965322	1965370
Sma-1-20594	NS_000200.1	+	139846600	139846648
Sma-1-20595	NW_003038009.1	-	250828	250780
Sma-1-20596	NW_003038252.1	+	345444	345492
Sma-1-20597	NS_000200.1	+	319201032	319201080
Sma-1-20598	NS_000200.1	-	226624891	226624843
Sma-1-20599	NW_003038010.1	-	7380	7332
Sma-1-20600	NS_000200.1	-	226825603	226825555
Sma-1-20601	NW_003038013.1	-	108513	108465
Sma-1-20602	NW_003037841.1	+	299128	299176

Sma-1-20603	NS_000200.1	+	82910309	82910357
Sma-1-20604	NS_000200.1	-	229498139	229498091
Sma-1-20605	NW_003038083.1	+	679672	679720
Sma-1-20606	NS_000200.1	+	270923009	270923057
Sma-1-20607	NW_003034722.1	-	1244	1196
Sma-1-20608	NW_003037962.1	+	38537	38585
Sma-1-20609	NW_003035880.1	+	195679	195727
Sma-1-20610	NW_003037790.1	-	66501	66453
Sma-1-20611	NW_003026489.1	+	68130	68178
Sma-1-20612	NW_003037806.1	-	1360025	1359977
Sma-1-20613	NW_003037816.1	+	633379	633427
Sma-1-20614	NW_003037830.1	+	991736	991784
Sma-1-20615	NW_003037840.1	-	752470	752422
Sma-1-20616	NW_003037878.1	+	685050	685098
Sma-1-20617	NW_003037881.1	-	722122	722074
Sma-1-20618	NW_003037883.1	-	13817	13769
Sma-1-20619	NW_003037905.1	-	225957	225909
Sma-1-20620	NW_003037914.1	-	1710975	1710927
Sma-1-20621	NW_003037972.1	-	247377	247329
Sma-1-20622	NW_003038031.1	+	302629	302677
Sma-1-20623	NW_003039127.1	+	2198005	2198053
Sma-1-20624	NW_003038161.1	+	499680	499728
Sma-1-20625	NS_000200.1	+	46842251	46842299
Sma-1-20626	NS_000200.1	+	68882107	68882155
Sma-1-20627	NS_000200.1	+	115275504	115275552
Sma-1-20628	NS_000200.1	+	122738528	122738576
Sma-1-20629	NS_000200.1	+	196115380	196115428
Sma-1-20630	NS_000200.1	+	244580387	244580435
Sma-1-20631	NS_000200.1	+	264293350	264293398
Sma-1-20632	NS_000200.1	+	297869531	297869579
Sma-1-20633	NS_000200.1	+	339436743	339436791
Sma-1-20634	NS_000200.1	-	381410673	381410625
Sma-1-20635	NS_000200.1	-	328263064	328263016
Sma-1-20636	NS_000200.1	-	202723081	202723033
Sma-1-20637	NS_000200.1	-	161855225	161855177
Sma-1-20638	NS_000200.1	-	149494969	149494921
Sma-1-20639	NS_000200.1	-	126077540	126077492
Sma-1-20640	NS_000200.1	-	125153913	125153865
Sma-1-20641	NS_000200.1	-	81375794	81375746
Sma-1-20642	NS_000200.1	-	29555239	29555191
Sma-1-20643	NW_003037849.1	-	649015	648967
Sma-1-20644	NW_003038120.1	+	55216	55264
Sma-1-20645	NS_000200.1	+	278240439	278240487
Sma-1-20646	NS_000200.1	-	91625288	91625240
Sma-1-20647	NW_003037891.1	+	1549146	1549194
Sma-1-20648	NW_003037891.1	+	1553280	1553328
Sma-1-20649	NW_003037891.1	-	1548661	1548613
Sma-1-20650	NW_003037904.1	+	426104	426152
Sma-1-20651	NS_000200.1	+	139430424	139430472
Sma-1-20652	NS_000200.1	+	139434558	139434606
Sma-1-20653	NS_000200.1	+	149140603	149140651
Sma-1-20654	NS_000200.1	-	139429939	139429891

Sma-1-20655	NW_003037853.1	-	866623	866575
Sma-1-20656	NW_003038032.1	+	337794	337842
Sma-1-20657	NS_000200.1	+	245496954	245497002
Sma-1-20658	NS_000200.1	-	96358674	96358626
Sma-1-20659	NW_003038020.1	-	636630	636582
Sma-1-20660	NS_000200.1	-	234603974	234603926
Sma-1-20661	NW_003038137.1	+	206407	206455
Sma-1-20662	NS_000200.1	+	284995303	284995351
Sma-1-20663	NW_003038030.1	+	748392	748440
Sma-1-20664	NS_000200.1	+	243884259	243884307
Sma-1-20665	NW_003037794.1	+	1292742	1292790
Sma-1-20666	NW_003037829.1	+	914673	914721
Sma-1-20667	NW_003037847.1	-	848314	848266
Sma-1-20668	NW_003037907.1	-	254629	254581
Sma-1-20669	NW_003037909.1	+	112990	113038
Sma-1-20670	NW_003038033.1	+	910635	910683
Sma-1-20671	NW_003038084.1	+	183017	183065
Sma-1-20672	NW_003038087.1	+	338047	338095
Sma-1-20673	NS_000200.1	+	5472337	5472385
Sma-1-20674	NS_000200.1	+	66815211	66815259
Sma-1-20675	NS_000200.1	+	153654639	153654687
Sma-1-20676	NS_000200.1	+	246890637	246890685
Sma-1-20677	NS_000200.1	+	271198261	271198309
Sma-1-20678	NS_000200.1	+	273366448	273366496
Sma-1-20679	NS_000200.1	-	151659118	151659070
Sma-1-20680	NS_000200.1	-	89500044	89499996
Sma-1-20681	NW_003037873.1	-	683111	683063
Sma-1-20682	NS_000200.1	-	119422239	119422191
Sma-1-20683	NW_003035689.1	-	17750	17702
Sma-1-20684	NS_000200.1	-	333102966	333102918
Sma-1-20685	NW_003038241.1	+	151218	151266
Sma-1-20686	NS_000200.1	+	314613923	314613971
Sma-1-20687	NW_003037843.1	+	416428	416476
Sma-1-20688	NS_000200.1	+	86055735	86055783
Sma-1-20689	NW_003037891.1	+	1183466	1183514
Sma-1-20690	NS_000200.1	+	139064744	139064792
Sma-1-20691	NW_003037852.1	+	237215	237263
Sma-1-20692	NS_000200.1	+	94629110	94629158
Sma-1-20693	NW_003021574.1	-	918	870
Sma-1-20694	NS_000200.1	-	348105118	348105070
Sma-1-20695	NW_003038011.1	-	278500	278452
Sma-1-20696	NS_000200.1	-	227806150	227806102
Sma-1-20697	NW_003037885.1	-	702621	702573
Sma-1-20698	NS_000200.1	-	128800568	128800520
Sma-1-20699	NW_003030600.1	-	958	910
Sma-1-20700	NW_003037791.1	-	414004	413956
Sma-1-20701	NW_003037794.1	+	1145256	1145304
Sma-1-20702	NW_003037796.1	+	1355354	1355402
Sma-1-20703	NW_003037804.1	-	1347199	1347151
Sma-1-20704	NW_003037805.1	-	1068610	1068562
Sma-1-20705	NW_003037809.1	+	1471855	1471903
Sma-1-20706	NW_003037837.1	+	23782	23830

Sma-1-20707	NW_003037855.1	-	298637	298589
Sma-1-20708	NW_003037861.1	+	657797	657845
Sma-1-20709	NW_003037910.1	-	104369	104321
Sma-1-20710	NW_003037913.1	+	78574	78622
Sma-1-20711	NW_003037916.1	+	1358643	1358691
Sma-1-20712	NW_003037970.1	-	507120	507072
Sma-1-20713	NW_003037978.1	+	637497	637545
Sma-1-20714	NW_003037978.1	+	637829	637877
Sma-1-20715	NW_003037978.1	-	637185	637137
Sma-1-20716	NW_003038018.1	-	190932	190884
Sma-1-20717	NW_003038517.1	+	289848	289896
Sma-1-20718	NW_003038517.1	-	63725	63677
Sma-1-20719	NW_003038056.1	-	36579	36531
Sma-1-20720	NW_003038086.1	-	203615	203567
Sma-1-20721	NW_003038134.1	+	187107	187155
Sma-1-20722	NS_000200.1	+	5324851	5324899
Sma-1-20723	NS_000200.1	+	9564998	9565046
Sma-1-20724	NS_000200.1	+	35136319	35136367
Sma-1-20725	NS_000200.1	+	76829640	76829688
Sma-1-20726	NS_000200.1	+	105434251	105434299
Sma-1-20727	NS_000200.1	+	158642325	158642373
Sma-1-20728	NS_000200.1	+	164552054	164552102
Sma-1-20729	NS_000200.1	+	209905507	209905555
Sma-1-20730	NS_000200.1	+	209905839	209905887
Sma-1-20731	NS_000200.1	+	283378224	283378272
Sma-1-20732	NS_000200.1	+	341054336	341054384
Sma-1-20733	NS_000200.1	-	382119432	382119384
Sma-1-20734	NS_000200.1	-	366552920	366552872
Sma-1-20735	NS_000200.1	-	340828213	340828165
Sma-1-20736	NS_000200.1	-	272452208	272452160
Sma-1-20737	NS_000200.1	-	259014230	259014182
Sma-1-20738	NS_000200.1	-	232591782	232591734
Sma-1-20739	NS_000200.1	-	209905195	209905147
Sma-1-20740	NS_000200.1	-	201563310	201563262
Sma-1-20741	NS_000200.1	-	154616344	154616296
Sma-1-20742	NS_000200.1	-	99163387	99163339
Sma-1-20743	NS_000200.1	-	27626454	27626406
Sma-1-20744	NS_000200.1	-	26487219	26487171
Sma-1-20745	NW_003038092.1	-	21398	21350
Sma-1-20746	NS_000200.1	-	273993034	273992986
Sma-1-20747	NW_003038241.1	-	123842	123794
Sma-1-20748	NW_003038041.1	+	80632	80680
Sma-1-20749	NS_000200.1	+	253747914	253747962
Sma-1-20750	NS_000200.1	-	314586547	314586499
Sma-1-20751	NW_003037850.1	+	1097844	1097892
Sma-1-20752	NS_000200.1	+	93083874	93083922
Sma-1-20753	NW_003038059.1	-	77701	77653
Sma-1-20754	NS_000200.1	-	260319773	260319725
Sma-1-20755	NW_003037995.1	-	212798	212750
Sma-1-20756	NS_000200.1	-	219902566	219902518
Sma-1-20757	NW_003037894.1	-	372658	372610
Sma-1-20758	NS_000200.1	-	142269231	142269183

Sma-1-20759	NW_003037894.1	-	368542	368494
Sma-1-20760	NS_000200.1	-	142265115	142265067
Sma-1-20761	NW_003037851.1	+	291959	292007
Sma-1-20762	NS_000200.1	+	93695269	93695317
Sma-1-20763	NW_003038057.1	-	289953	289905
Sma-1-20764	NS_000200.1	-	259495769	259495721
Sma-1-20765	NW_003037853.1	+	1041946	1041994
Sma-1-20766	NW_003038031.1	-	211642	211594
Sma-1-20767	NW_003038157.1	-	134308	134260
Sma-1-20768	NS_000200.1	+	96533997	96534045
Sma-1-20769	NS_000200.1	-	295385228	295385180
Sma-1-20770	NS_000200.1	-	244489400	244489352
Sma-1-20771	NW_003037853.1	-	1057257	1057209
Sma-1-20772	NW_003038027.1	-	211531	211483
Sma-1-20773	NS_000200.1	-	240582245	240582197
Sma-1-20774	NS_000200.1	-	96549308	96549260
Sma-1-20775	NW_003038243.1	+	99094	99142
Sma-1-20776	NS_000200.1	+	315037578	315037626
Sma-1-20777	NW_003033991.1	+	111	159
Sma-1-20778	NW_003035689.1	+	41289	41337
Sma-1-20779	NS_000200.1	+	333126505	333126553
Sma-1-20780	NS_000200.1	+	363367907	363367955
Sma-1-20781	NW_003037854.1	-	1795336	1795288
Sma-1-20782	NS_000200.1	-	98699757	98699709
Sma-1-20783	NW_003037857.1	+	134307	134355
Sma-1-20784	NW_003038034.1	-	349954	349906
Sma-1-20785	NW_003038138.1	-	379356	379308
Sma-1-20786	NS_000200.1	+	100154664	100154712
Sma-1-20787	NS_000200.1	-	285934671	285934623
Sma-1-20788	NS_000200.1	-	247291710	247291662
Sma-1-20789	NW_003038138.1	+	293383	293431
Sma-1-20790	NS_000200.1	+	285848698	285848746
Sma-1-20791	NW_003038237.1	-	112849	112801
Sma-1-20792	NW_003039127.1	-	2070615	2070567
Sma-1-20793	NS_000200.1	-	313552768	313552720
Sma-1-20794	NS_000200.1	-	115148114	115148066
Sma-1-20795	NW_003038237.1	+	122722	122770
Sma-1-20796	NS_000200.1	+	313562641	313562689
Sma-1-20797	NW_003038142.1	+	106639	106687
Sma-1-20798	NS_000200.1	+	287317075	287317123
Sma-1-20799	NW_003038026.1	-	519898	519850
Sma-1-20800	NS_000200.1	-	240027659	240027611
Sma-1-20801	NW_003038244.1	+	13048	13096
Sma-1-20802	NS_000200.1	+	315158253	315158301
Sma-1-20803	NW_003038247.1	-	286292	286244
Sma-1-20804	NS_000200.1	-	316235020	316234972
Sma-1-20805	NW_003038147.1	+	10854	10902
Sma-1-20806	NS_000200.1	+	289538597	289538645
Sma-1-20807	NW_003038086.1	-	82306	82258
Sma-1-20808	NS_000200.1	-	272330899	272330851
Sma-1-20809	NW_003037976.1	-	117794	117746
Sma-1-20810	NS_000200.1	-	206732109	206732061

Sma-1-20811	NW_003037854.1	+	396608	396655
Sma-1-20812	NS_000200.1	+	97301029	97301076
Sma-1-20813	NW_003037905.1	-	997163	997115
Sma-1-20814	NW_003037913.1	-	395638	395590
Sma-1-20815	NS_000200.1	-	158959389	158959341
Sma-1-20816	NS_000200.1	-	150266175	150266127
Sma-1-20817	NW_003038034.1	-	216559	216511
Sma-1-20818	NS_000200.1	-	247158315	247158267
Sma-1-20819	NW_003037885.1	-	904500	904452
Sma-1-20820	NS_000200.1	-	129002447	129002399
Sma-1-20821	NW_003037870.1	-	278820	278772
Sma-1-20822	NS_000200.1	-	117152485	117152437
Sma-1-20823	NW_003038055.1	+	162258	162306
Sma-1-20824	NS_000200.1	+	258861580	258861628
Sma-1-20825	NW_003038210.1	+	118486	118534
Sma-1-20826	NW_003038083.1	-	623528	623480
Sma-1-20827	NS_000200.1	+	305580385	305580433
Sma-1-20828	NS_000200.1	-	270866865	270866817
Sma-1-20829	NW_003038035.1	+	454418	454466
Sma-1-20830	NS_000200.1	+	248315910	248315958
Sma-1-20831	NW_003037881.1	-	674520	674472
Sma-1-20832	NS_000200.1	-	125106311	125106263
Sma-1-20833	NW_003038258.1	+	276980	277028
Sma-1-20834	NS_000200.1	+	322815490	322815538
Sma-1-20835	NW_003038146.1	-	463400	463352
Sma-1-20836	NS_000200.1	-	289444070	289444022
Sma-1-20837	NW_003038077.1	-	326376	326328
Sma-1-20838	NS_000200.1	-	267098016	267097968
Sma-1-20839	NW_003038065.1	-	295106	295058
Sma-1-20840	NW_003038146.1	+	131377	131425
Sma-1-20841	NW_003038146.1	-	133601	133553
Sma-1-20842	NS_000200.1	+	289112047	289112095
Sma-1-20843	NS_000200.1	-	289114271	289114223
Sma-1-20844	NS_000200.1	-	262446723	262446675
Sma-1-20845	NW_003038037.1	+	1288675	1288723
Sma-1-20846	NS_000200.1	+	251444084	251444132
Sma-1-20847	NW_003037978.1	-	176135	176087
Sma-1-20848	NS_000200.1	-	209444145	209444097
Sma-1-20849	NW_003037915.1	+	731695	731743
Sma-1-20850	NS_000200.1	+	162631681	162631729
Sma-1-20851	NW_003037914.1	+	349558	349606
Sma-1-20852	NW_003037915.1	+	724420	724468
Sma-1-20853	NS_000200.1	+	160493808	160493856
Sma-1-20854	NS_000200.1	+	162624406	162624454
Sma-1-20855	NW_003037893.1	-	380863	380815
Sma-1-20856	NS_000200.1	-	141495173	141495125
Sma-1-20857	NW_003037851.1	-	116026	115978
Sma-1-20858	NS_000200.1	-	93519336	93519288
Sma-1-20859	NW_003037841.1	+	1347872	1347920
Sma-1-20860	NS_000200.1	+	83959053	83959101
Sma-1-20861	NW_003037840.1	+	417361	417409
Sma-1-20862	NS_000200.1	+	81040685	81040733

Sma-1-20863	NW_003037794.1	+	2038609	2038657
Sma-1-20864	NW_003038517.1	-	65745	65697
Sma-1-20865	NS_000200.1	+	6218204	6218252
Sma-1-20866	NS_000200.1	-	340830233	340830185
Sma-1-20867	NW_003037820.1	+	138670	138717
Sma-1-20868	NS_000200.1	+	52411652	52411699
Sma-1-20869	NW_003037816.1	-	419668	419620
Sma-1-20870	NS_000200.1	-	46628540	46628492
Sma-1-20871	NW_003037814.1	+	1189665	1189713
Sma-1-20872	NS_000200.1	+	44173525	44173573
Sma-1-20873	NW_003037804.1	+	1177454	1177502
Sma-1-20874	NS_000200.1	+	26317474	26317522
Sma-1-20875	NW_003037793.1	-	2442204	2442156
Sma-1-20876	NW_003037800.1	+	980342	980390
Sma-1-20877	NW_003037815.1	-	514574	514526
Sma-1-20878	NW_003037824.1	-	742482	742434
Sma-1-20879	NW_003037834.1	-	650708	650660
Sma-1-20880	NW_003037919.1	-	245666	245618
Sma-1-20881	NW_003038008.1	-	620369	620321
Sma-1-20882	NS_000200.1	+	19609702	19609750
Sma-1-20883	NS_000200.1	-	226293465	226293417
Sma-1-20884	NS_000200.1	-	169103595	169103547
Sma-1-20885	NS_000200.1	-	73811194	73811146
Sma-1-20886	NS_000200.1	-	57673422	57673374
Sma-1-20887	NS_000200.1	-	45777565	45777517
Sma-1-20888	NS_000200.1	-	2442204	2442156
Sma-1-20889	NW_003037066.1	-	2146	2098
Sma-1-20890	NS_000200.1	-	343769374	343769326
Sma-1-20891	NW_003032564.1	+	273	321
Sma-1-20892	NW_003032564.1	+	1640	1688
Sma-1-20893	NW_003034564.1	+	8473	8521
Sma-1-20894	NW_003026617.1	-	162330	162282
Sma-1-20895	NW_003020531.1	+	5317	5365
Sma-1-20896	NW_003037809.1	-	2148953	2148905
Sma-1-20897	NW_003037832.1	+	333274	333322
Sma-1-20898	NW_003037837.1	+	74883	74931
Sma-1-20899	NW_003037878.1	+	407231	407279
Sma-1-20900	NW_003037881.1	+	749929	749977
Sma-1-20901	NW_003026511.1	-	368826	368778
Sma-1-20902	NW_003038046.1	+	162868	162916
Sma-1-20903	NS_000200.1	+	71542705	71542753
Sma-1-20904	NS_000200.1	+	76880741	76880789
Sma-1-20905	NS_000200.1	+	122460709	122460757
Sma-1-20906	NS_000200.1	+	125181720	125181768
Sma-1-20907	NS_000200.1	+	255236250	255236298
Sma-1-20908	NS_000200.1	+	327621120	327621168
Sma-1-20909	NS_000200.1	+	350333126	350333174
Sma-1-20910	NS_000200.1	+	350334493	350334541
Sma-1-20911	NS_000200.1	+	360079094	360079142
Sma-1-20912	NS_000200.1	-	319733189	319733141
Sma-1-20913	NS_000200.1	-	284751878	284751830
Sma-1-20914	NS_000200.1	-	35813417	35813369

Sma-1-20915	NW_003031802.1	+	528	576
Sma-1-20916	NS_000200.1	+	367041715	367041763
Sma-1-20917	NW_003030081.1	-	980	932
Sma-1-20918	NW_003037875.1	-	262064	262016
Sma-1-20919	NW_003037829.1	+	1096961	1097009
Sma-1-20920	NW_003037830.1	-	1549968	1549920
Sma-1-20921	NW_003037840.1	-	375396	375348
Sma-1-20922	NW_003037848.1	-	1044787	1044739
Sma-1-20923	NW_003037890.1	-	1623405	1623357
Sma-1-20924	NW_003038026.1	+	719835	719883
Sma-1-20925	NW_003038153.1	-	487765	487717
Sma-1-20926	NS_000200.1	+	66997499	66997547
Sma-1-20927	NS_000200.1	+	240227596	240227644
Sma-1-20928	NS_000200.1	-	357555948	357555900
Sma-1-20929	NS_000200.1	-	293549814	293549766
Sma-1-20930	NS_000200.1	-	137306766	137306718
Sma-1-20931	NS_000200.1	-	120290733	120290685
Sma-1-20932	NS_000200.1	-	90737858	90737810
Sma-1-20933	NS_000200.1	-	80998720	80998672
Sma-1-20934	NS_000200.1	-	69440339	69440291
Sma-1-20935	NW_003027775.1	-	841	793
Sma-1-20936	NW_003037871.1	-	200844	200796
Sma-1-20937	NS_000200.1	-	365915050	365915002
Sma-1-20938	NS_000200.1	-	117648582	117648534
Sma-1-20939	NW_003026955.1	-	850	802
Sma-1-20940	NW_003037896.1	+	329226	329274
Sma-1-20941	NW_003037980.1	+	110381	110429
Sma-1-20942	NW_003038010.1	-	549008	548960
Sma-1-20943	NS_000200.1	+	143249649	143249697
Sma-1-20944	NS_000200.1	+	211071243	211071291
Sma-1-20945	NS_000200.1	-	349581786	349581738
Sma-1-20946	NS_000200.1	-	227367231	227367183
Sma-1-20947	NW_003026611.1	-	103432	103384
Sma-1-20948	NS_000200.1	-	310673216	310673168
Sma-1-20949	NW_003026466.1	+	103513	103561
Sma-1-20950	NS_000200.1	+	215370620	215370668
env-90	AF036747.1	+	117	160
Sma-1-20951	NW_003020254.1	+	26	74
Sma-1-20952	NW_003020977.1	+	1050	1098
Sma-1-20953	NW_003020977.1	+	1383	1431
Sma-1-20954	NW_003021866.1	-	2992	2944
Sma-1-20955	NW_003021866.1	-	2659	2611
Sma-1-20956	NW_003021866.1	-	2326	2278
Sma-1-20957	NW_003021866.1	-	1993	1945
Sma-1-20958	NW_003022190.1	+	663	711
Sma-1-20959	NW_003023045.1	+	13731	13779
Sma-1-20960	NW_003023075.1	+	3399	3447
Sma-1-20961	NW_003023381.1	+	2035	2083
Sma-1-20962	NW_003023795.1	-	579	531
Sma-1-20963	NW_003024785.1	+	122	170
Sma-1-20964	NW_003024785.1	+	455	503
Sma-1-20965	NW_003024945.1	+	217	265

Sma-1-20966	NW_003025129.1	+	170	218
Sma-1-20967	NW_003025653.1	+	470	518
Sma-1-20968	NW_003025832.1	+	862	910
Sma-1-20969	NW_003025832.1	+	1195	1243
Sma-1-20970	NW_003026477.1	-	44055	44007
Sma-1-20971	NW_003026566.1	-	3818	3770
Sma-1-20972	NW_003026578.1	-	31618	31570
Sma-1-20973	NW_003026583.1	+	24294	24342
Sma-1-20974	NW_003026614.1	-	5140	5092
Sma-1-20975	NW_003027140.1	-	1061	1013
Sma-1-20976	NW_003028110.1	+	688	736
Sma-1-20977	NW_003028255.1	-	1536	1488
Sma-1-20978	NW_003028567.1	-	914	866
Sma-1-20979	NW_003028749.1	+	199	247
Sma-1-20980	NW_003028941.1	+	224	272
Sma-1-20981	NW_003029209.1	-	779	731
Sma-1-20982	NW_003029281.1	+	563	611
Sma-1-20983	NW_003029509.1	+	236	284
Sma-1-20984	NW_003029645.1	-	1239	1191
Sma-1-20985	NW_003029859.1	-	1110	1062
Sma-1-20986	NW_003030020.1	-	641	593
Sma-1-20987	NW_003030059.1	-	705	657
Sma-1-20988	NW_003030181.1	+	15	63
Sma-1-20989	NW_003031250.1	-	1369	1321
Sma-1-20990	NW_003031848.1	-	97	49
Sma-1-20991	NW_003031889.1	+	128	176
Sma-1-20992	NW_003032126.1	-	7290	7242
Sma-1-20993	NW_003032319.1	+	2864	2912
Sma-1-20994	NW_003033836.1	+	468	516
Sma-1-20995	NW_003034050.1	-	243	195
Sma-1-20996	NW_003034083.1	+	10	58
Sma-1-20997	NW_003034267.1	+	341	389
Sma-1-20998	NW_003034267.1	-	72	24
Sma-1-20999	NW_003034296.1	+	766	814
Sma-1-21000	NW_003034327.1	-	5211	5163
Sma-1-21001	NW_003034794.1	-	916	868
Sma-1-21002	NW_003035328.1	+	1062	1110
Sma-1-21003	NW_003035562.1	-	841	793
Sma-1-21004	NW_003035779.1	+	1866	1914
Sma-1-21005	NW_003036685.1	+	299	347
Sma-1-21006	NW_003039132.1	-	727	679
Sma-1-21007	NW_003026617.1	+	162631	162679
Sma-1-21008	NW_003037165.1	+	3012	3060
Sma-1-21009	NW_003037165.1	-	4423	4375
Sma-1-21010	NW_003038253.1	+	16755	16803
Sma-1-21011	NW_003038262.1	-	30991	30943
Sma-1-21012	NW_003037583.1	+	7054	7102
Sma-1-21013	NW_003037873.1	+	62364	62412
Sma-1-21014	NW_003037873.1	+	107750	107798
Sma-1-21015	NW_003037874.1	+	229772	229820
Sma-1-21016	NW_003037874.1	+	371643	371691
Sma-1-21017	NW_003037875.1	+	331234	331282

Sma-1-21018	NW_003035878.1	+	88559	88607
Sma-1-21019	NW_003038509.1	-	56751	56703
Sma-1-21020	NW_003038337.1	-	1654	1606
Sma-1-21021	NW_003037962.1	+	73409	73457
Sma-1-21022	NW_003038511.1	+	191914	191962
Sma-1-21023	NW_003038512.1	-	445776	445728
Sma-1-21024	NW_003026598.1	-	41275	41227
Sma-1-21025	NW_003038441.1	+	21012	21060
Sma-1-21026	NW_003038514.1	+	272467	272515
Sma-1-21027	NW_003038514.1	-	104361	104313
Sma-1-21028	NW_003026472.1	-	173907	173859
Sma-1-21029	NW_003038366.1	+	529	577
Sma-1-21030	NW_003037898.1	+	374948	374996
Sma-1-21031	NW_003037898.1	-	353924	353876
Sma-1-21032	NW_003026607.1	+	94721	94769
Sma-1-21033	NW_003026607.1	-	114162	114114
Sma-1-21034	NW_003038370.1	-	28480	28432
Sma-1-21035	NW_003038221.1	+	48792	48840
Sma-1-21036	NW_003038222.1	+	131648	131696
Ehi-1-1	NW_001916244.1	-	616	568
Sma-1-21037	NW_003038850.1	-	638	590
Sma-1-21038	NW_003038002.1	-	310715	310667
Sma-1-21039	NW_003038745.1	-	2543	2495
Sma-1-21040	NW_003038004.1	-	160588	160540
Sma-1-21041	NW_003038005.1	-	428203	428155
Sma-1-21042	NW_003026584.1	+	20252	20300
Sma-1-21043	NW_003026618.1	+	171931	171979
Sma-1-21044	NW_003037778.1	+	32053	32101
Sma-1-21045	NW_003038009.1	-	250495	250447
Sma-1-21046	NW_003038470.1	+	11195	11243
Sma-1-21047	NW_003032155.1	+	104137	104185
Sma-1-21048	NW_003035869.1	+	71244	71292
Sma-1-21049	NW_003026460.1	+	257026	257074
Sma-1-21050	NW_003026465.1	+	361493	361541
Sma-1-21051	NW_003026465.1	-	339264	339216
Sma-1-21052	NW_003037985.1	+	58583	58631
Sma-1-21053	NW_003038478.1	+	9319	9367
Sma-1-21054	NW_003037929.1	+	548171	548219
Sma-1-21055	NW_003035690.1	+	25927	25975
Sma-1-21056	NW_003038244.1	-	171690	171642
Sma-1-21057	NW_003037936.1	-	73005	72957
Sma-1-21058	NW_003037580.1	-	13439	13391
Sma-1-21059	NW_003037736.1	-	2273	2225
Sma-1-21060	NW_003037937.1	+	304737	304785
Sma-1-21061	NW_003037937.1	-	685296	685248
Sma-1-21062	NW_003026615.1	+	121108	121156
Sma-1-21063	NW_003026616.1	-	113375	113327
Sma-1-21064	NW_003039130.1	+	168235	168283
Sma-1-21065	NW_003039130.1	-	112931	112883
Sma-1-21066	NW_003035866.1	+	52882	52930
Sma-1-21067	NW_003038070.1	+	154668	154716
Sma-1-21068	NW_003038070.1	-	370830	370782

Sma-1-21069	NW_003037785.1	+	87319	87367
Sma-1-21070	NW_003037785.1	+	95484	95532
Sma-1-21071	NW_003026462.1	-	582796	582748
Sma-1-21072	NW_003026470.1	+	251678	251726
Sma-1-21073	NW_003026470.1	+	306001	306049
Sma-1-21074	NW_003026619.1	+	83769	83817
Sma-1-21075	NW_003026619.1	-	296988	296940
Sma-1-21076	NW_003026619.1	-	19964	19916
Sma-1-21077	NW_003035876.1	+	32784	32832
Sma-1-21078	NW_003037791.1	-	390500	390452
Sma-1-21079	NW_003037792.1	-	207170	207122
Sma-1-21080	NW_003037793.1	+	2442512	2442560
Sma-1-21081	NW_003037793.1	+	2675609	2675657
Sma-1-21082	NW_003037793.1	-	3463476	3463428
Sma-1-21083	NW_003037793.1	-	2311860	2311812
Sma-1-21084	NW_003037793.1	-	2299500	2299452
Sma-1-21085	NW_003037793.1	-	711079	711031
Sma-1-21086	NW_003037793.1	-	24825	24777
Sma-1-21087	NW_003037794.1	+	35052	35100
Sma-1-21088	NW_003037794.1	+	1184749	1184797
Sma-1-21089	NW_003037794.1	-	1948914	1948866
Sma-1-21090	NW_003037794.1	-	1788755	1788707
Sma-1-21091	NW_003037795.1	+	639082	639130
Sma-1-21092	NW_003037795.1	+	1329850	1329898
Sma-1-21093	NW_003037796.1	-	1161550	1161502
Sma-1-21094	NW_003037796.1	-	587554	587506
Sma-1-21095	NW_003037796.1	-	308551	308503
Sma-1-21096	NW_003037796.1	-	59629	59581
Sma-1-21097	NW_003037797.1	+	2324716	2324764
Sma-1-21098	NW_003037797.1	+	2419541	2419589
Sma-1-21099	NW_003037797.1	-	1637382	1637334
Sma-1-21100	NW_003037797.1	-	1058159	1058111
Sma-1-21101	NW_003037797.1	-	652698	652650
Sma-1-21102	NW_003037798.1	+	260304	260352
Sma-1-21103	NW_003037798.1	+	432224	432272
Sma-1-21104	NW_003037798.1	+	2646194	2646242
Sma-1-21105	NW_003037798.1	-	2645626	2645578
Sma-1-21106	NW_003037798.1	-	1409395	1409347
Sma-1-21107	NW_003037798.1	-	18253	18205
Sma-1-21108	NW_003037799.1	-	261434	261386
Sma-1-21109	NW_003037800.1	+	1530556	1530604
Sma-1-21110	NW_003037800.1	+	2066003	2066051
Sma-1-21111	NW_003037800.1	-	309252	309204
Sma-1-21112	NW_003037803.1	+	133363	133411
Sma-1-21113	NW_003037803.1	+	1336477	1336525
Sma-1-21114	NW_003037803.1	-	1211820	1211772
Sma-1-21115	NW_003037804.1	+	563708	563756
Sma-1-21116	NW_003037804.1	-	473845	473797
Sma-1-21117	NW_003037805.1	-	1420200	1420152
Sma-1-21118	NW_003037806.1	-	1820498	1820450
Sma-1-21119	NW_003037806.1	-	1076913	1076865
Sma-1-21120	NW_003037806.1	-	412525	412477

Sma-1-21121	NW_003037807.1	+	1148191	1148239
Sma-1-21122	NW_003037807.1	-	1421272	1421224
Sma-1-21123	NW_003037808.1	+	925982	926030
Sma-1-21124	NW_003037809.1	+	1051772	1051820
Sma-1-21125	NW_003037809.1	+	2543608	2543656
Sma-1-21126	NW_003037809.1	-	2151881	2151833
Sma-1-21127	NW_003037810.1	-	170700	170652
Sma-1-21128	NW_003037811.1	-	878068	878020
Sma-1-21129	NW_003037812.1	+	189751	189799
Sma-1-21130	NW_003037812.1	+	1240518	1240566
Sma-1-21131	NW_003037812.1	-	297528	297480
Sma-1-21132	NW_003037813.1	+	642122	642170
Sma-1-21133	NW_003037813.1	-	1929839	1929791
Sma-1-21134	NW_003037814.1	+	656053	656101
Sma-1-21135	NW_003037814.1	-	13105	13057
Sma-1-21136	NW_003037815.1	+	149572	149620
Sma-1-21137	NW_003037815.1	+	543552	543600
Sma-1-21138	NW_003037815.1	+	690587	690635
Sma-1-21139	NW_003037817.1	+	506178	506226
Sma-1-21140	NW_003037817.1	+	956072	956120
Sma-1-21141	NW_003037817.1	-	981862	981814
Sma-1-21142	NW_003037818.1	+	692815	692863
Sma-1-21143	NW_003037818.1	+	2267424	2267472
Sma-1-21144	NW_003037818.1	-	350280	350232
Sma-1-21145	NW_003037819.1	-	477012	476964
Sma-1-21146	NW_003037820.1	-	516588	516540
Sma-1-21147	NW_003037822.1	-	1322042	1321994
Sma-1-21148	NW_003037822.1	-	294229	294181
Sma-1-21149	NW_003037822.1	-	259652	259604
Sma-1-21150	NW_003037824.1	-	614913	614865
Sma-1-21151	NW_003037825.1	+	363299	363347
Sma-1-21152	NW_003037825.1	+	776740	776788
Sma-1-21153	NW_003037825.1	+	1429842	1429890
Sma-1-21154	NW_003037825.1	-	1552411	1552363
Sma-1-21155	NW_003037825.1	-	445659	445611
Sma-1-21156	NW_003037826.1	+	238583	238631
Sma-1-21157	NW_003037826.1	+	286984	287032
Sma-1-21158	NW_003037826.1	+	526038	526086
Sma-1-21159	NW_003037826.1	+	566573	566621
Sma-1-21160	NW_003037826.1	-	324098	324050
Sma-1-21161	NW_003037827.1	+	447972	448020
Sma-1-21162	NW_003037827.1	+	2367505	2367553
Sma-1-21163	NW_003037827.1	-	1815589	1815541
Sma-1-21164	NW_003037827.1	-	553484	553436
Sma-1-21165	NW_003037827.1	-	553151	553103
Sma-1-21166	NW_003037829.1	+	96289	96337
Sma-1-21167	NW_003037829.1	+	652338	652386
Sma-1-21168	NW_003037830.1	+	315866	315914
Sma-1-21169	NW_003037830.1	+	1454095	1454143
Sma-1-21170	NW_003037831.1	-	1053318	1053270
Sma-1-21171	NW_003037832.1	+	332093	332141
Sma-1-21172	NW_003037832.1	-	327818	327770

Sma-1-21173	NW_003037834.1	-	1112735	1112687
Sma-1-21174	NW_003037836.1	+	1159313	1159361
Sma-1-21175	NW_003037838.1	+	343478	343526
Sma-1-21176	NW_003037838.1	+	690367	690415
Sma-1-21177	NW_003037838.1	+	903722	903770
Sma-1-21178	NW_003037839.1	+	964976	965024
Sma-1-21179	NW_003037839.1	+	986581	986629
Sma-1-21180	NW_003037839.1	-	968373	968325
Sma-1-21181	NW_003037839.1	-	40459	40411
Sma-1-21182	NW_003037839.1	-	40126	40078
Sma-1-21183	NW_003037840.1	+	1186949	1186997
Sma-1-21184	NW_003037840.1	-	458685	458637
Sma-1-21185	NW_003037846.1	-	275438	275390
Sma-1-21186	NW_003037847.1	+	413613	413661
Sma-1-21187	NW_003037848.1	+	201232	201280
Sma-1-21188	NW_003037848.1	+	236966	237014
Sma-1-21189	NW_003037849.1	+	834673	834721
Sma-1-21190	NW_003037849.1	-	317924	317876
Sma-1-21191	NW_003037849.1	-	247094	247046
Sma-1-21192	NW_003037850.1	+	1072073	1072121
Sma-1-21193	NW_003037850.1	-	193750	193702
Sma-1-21194	NW_003037852.1	+	670667	670715
Sma-1-21195	NW_003037852.1	-	272533	272485
Sma-1-21196	NW_003037853.1	+	152624	152672
Sma-1-21197	NW_003037853.1	+	429003	429051
Sma-1-21198	NW_003037853.1	-	1047445	1047397
Sma-1-21199	NW_003037853.1	-	421070	421022
Sma-1-21200	NW_003037854.1	+	1091915	1091963
Sma-1-21201	NW_003037854.1	-	1517280	1517232
Sma-1-21202	NW_003037855.1	+	157110	157158
Sma-1-21203	NW_003037856.1	+	657251	657299
Sma-1-21204	NW_003037859.1	+	127841	127889
Sma-1-21205	NW_003037859.1	+	200884	200932
Sma-1-21206	NW_003037859.1	-	558048	558000
Sma-1-21207	NW_003037860.1	+	861525	861573
Sma-1-21208	NW_003037864.1	+	1127863	1127911
Sma-1-21209	NW_003037864.1	-	1448547	1448499
Sma-1-21210	NW_003037865.1	+	590101	590149
Sma-1-21211	NW_003037865.1	+	596972	597020
Sma-1-21212	NW_003037865.1	+	854248	854296
Sma-1-21213	NW_003037869.1	+	156059	156107
Sma-1-21214	NW_003037872.1	-	296631	296583
Sma-1-21215	NW_003037872.1	-	41884	41836
Sma-1-21216	NW_003037877.1	+	896834	896882
Sma-1-21217	NW_003037879.1	-	169645	169597
Sma-1-21218	NW_003037879.1	-	3263	3215
Sma-1-21219	NW_003037881.1	-	74536	74488
Sma-1-21220	NW_003037882.1	-	520572	520524
Sma-1-21221	NW_003037884.1	+	287327	287375
Sma-1-21222	NW_003037885.1	-	625836	625788
Sma-1-21223	NW_003037886.1	+	1013707	1013755
Sma-1-21224	NW_003037886.1	-	606344	606296

Sma-1-21225	NW_003037887.1	-	1092432	1092384
Sma-1-21226	NW_003037889.1	+	436446	436494
Sma-1-21227	NW_003037889.1	+	466770	466818
Sma-1-21228	NW_003037889.1	+	469296	469344
Sma-1-21229	NW_003037889.1	+	977962	978010
Sma-1-21230	NW_003037889.1	-	1164074	1164026
Sma-1-21231	NW_003037889.1	-	483816	483768
Sma-1-21232	NW_003037890.1	+	1914685	1914733
Sma-1-21233	NW_003037890.1	+	2017176	2017224
Sma-1-21234	NW_003037890.1	-	2052355	2052307
Sma-1-21235	NW_003037891.1	+	852690	852738
Sma-1-21236	NW_003037891.1	+	2024519	2024567
Sma-1-21237	NW_003037891.1	+	2301626	2301674
Sma-1-21238	NW_003037891.1	-	1949002	1948954
Sma-1-21239	NW_003037893.1	+	37147	37195
Sma-1-21240	NW_003037893.1	+	83486	83534
Sma-1-21241	NW_003037893.1	-	361011	360963
Sma-1-21242	NW_003037895.1	+	2558	2606
Sma-1-21243	NW_003037895.1	+	355926	355974
Sma-1-21244	NW_003037896.1	-	321155	321107
Sma-1-21245	NW_003037900.1	-	684820	684772
Sma-1-21246	NW_003037902.1	+	780628	780676
Sma-1-21247	NW_003037902.1	-	619826	619778
Sma-1-21248	NW_003037904.1	+	137746	137794
Sma-1-21249	NW_003037904.1	-	314828	314780
Sma-1-21250	NW_003037905.1	-	160782	160734
Sma-1-21251	NW_003037905.1	-	135322	135274
Sma-1-21252	NW_003037907.1	-	335495	335447
Sma-1-21253	NW_003037907.1	-	101484	101436
Sma-1-21254	NW_003037907.1	-	101151	101103
Sma-1-21255	NW_003037908.1	+	257552	257600
Sma-1-21256	NW_003037908.1	+	357240	357288
Sma-1-21257	NW_003037909.1	+	706915	706963
Sma-1-21258	NW_003037909.1	-	29281	29233
Sma-1-21259	NW_003037910.1	+	428922	428970
Sma-1-21260	NW_003037910.1	+	936577	936625
Sma-1-21261	NW_003037910.1	+	936910	936958
Sma-1-21262	NW_003037910.1	-	840112	840064
Sma-1-21263	NW_003037911.1	-	873180	873132
Sma-1-21264	NW_003037911.1	-	872847	872799
Sma-1-21265	NW_003037911.1	-	872514	872466
Sma-1-21266	NW_003037913.1	-	1353043	1352995
Sma-1-21267	NW_003037913.1	-	1295292	1295244
Sma-1-21268	NW_003037914.1	+	1694256	1694304
Sma-1-21269	NW_003037914.1	-	1717323	1717275
Sma-1-21270	NW_003037914.1	-	319939	319891
Sma-1-21271	NW_003037914.1	-	285200	285152
Sma-1-21272	NW_003037914.1	-	247184	247136
Sma-1-21273	NW_003037915.1	-	1249433	1249385
Sma-1-21274	NW_003037915.1	-	906220	906172
Sma-1-21275	NW_003037915.1	-	397327	397279
Sma-1-21276	NW_003037915.1	-	68039	67991

Sma-1-21277	NW_003037916.1	+	1221430	1221478
Sma-1-21278	NW_003037916.1	-	1522566	1522518
Sma-1-21279	NW_003037916.1	-	391791	391743
Sma-1-21280	NW_003037916.1	-	281598	281550
Sma-1-21281	NW_003037917.1	+	1464900	1464948
Sma-1-21282	NW_003037917.1	-	459570	459522
Sma-1-21283	NW_003037918.1	+	1049867	1049915
Sma-1-21284	NW_003037918.1	+	1760023	1760071
Sma-1-21285	NW_003037922.1	+	153912	153960
Sma-1-21286	NW_003037922.1	-	147675	147627
Sma-1-21287	NW_003037925.1	-	728984	728936
Sma-1-21288	NW_003037925.1	-	728651	728603
Sma-1-21289	NW_003037926.1	+	159800	159848
Sma-1-21290	NW_003037926.1	-	159516	159468
Sma-1-21291	NW_003037930.1	+	214553	214601
Sma-1-21292	NW_003037932.1	-	381254	381206
Sma-1-21293	NW_003037938.1	+	609397	609445
Sma-1-21294	NW_003037939.1	-	46601	46553
Sma-1-21295	NW_003037942.1	+	91118	91166
Sma-1-21296	NW_003037942.1	+	605536	605584
Sma-1-21297	NW_003037942.1	+	1129622	1129670
Sma-1-21298	NW_003037945.1	-	1449542	1449494
Sma-1-21299	NW_003037945.1	-	1219554	1219506
Sma-1-21300	NW_003037945.1	-	1114852	1114804
Sma-1-21301	NW_003037946.1	+	494215	494263
Sma-1-21302	NW_003037946.1	+	835379	835427
Sma-1-21303	NW_003037946.1	+	1147325	1147373
Sma-1-21304	NW_003037956.1	+	43386	43434
Sma-1-21305	NW_003037958.1	-	394888	394840
Sma-1-21306	NW_003037958.1	-	297812	297764
Sma-1-21307	NW_003037964.1	-	192300	192252
Sma-1-21308	NW_003037964.1	-	130779	130731
Sma-1-21309	NW_003037964.1	-	85488	85440
Sma-1-21310	NW_003037966.1	+	536139	536187
Sma-1-21311	NW_003037967.1	-	122964	122916
Sma-1-21312	NW_003037967.1	-	62989	62941
Sma-1-21313	NW_003037969.1	+	174166	174214
Sma-1-21314	NW_003037971.1	+	308417	308465
Sma-1-21315	NW_003037972.1	+	132168	132216
Sma-1-21316	NW_003037972.1	+	449416	449464
Sma-1-21317	NW_003037972.1	+	449749	449797
Sma-1-21318	NW_003037975.1	+	15844	15892
Sma-1-21319	NW_003037975.1	+	839235	839283
Sma-1-21320	NW_003037975.1	-	752586	752538
Sma-1-21321	NW_003037975.1	-	733616	733568
Sma-1-21322	NW_003037976.1	-	368453	368405
Sma-1-21323	NW_003037976.1	-	338970	338922
Sma-1-21324	NW_003037977.1	-	701090	701042
Sma-1-21325	NW_003037978.1	+	392854	392902
Sma-1-21326	NW_003037979.1	+	201589	201637
Sma-1-21327	NW_003037979.1	-	338385	338337
Sma-1-21328	NW_003037980.1	+	1158834	1158882

Sma-1-21329	NW_003037980.1	-	1127844	1127796
Sma-1-21330	NW_003037980.1	-	701960	701912
Sma-1-21331	NW_003037981.1	+	773642	773690
Sma-1-21332	NW_003037981.1	-	773383	773335
Sma-1-21333	NW_003037982.1	-	598165	598117
Sma-1-21334	NW_003037990.1	+	195433	195481
Sma-1-21335	NW_003037996.1	+	547805	547853
Sma-1-21336	NW_003037996.1	-	484540	484492
Sma-1-21337	NW_003037997.1	+	395832	395880
Sma-1-21338	NW_003037998.1	-	291753	291705
Sma-1-21339	NW_003038003.1	-	511519	511471
Sma-1-21340	NW_003038008.1	-	569887	569839
Sma-1-21341	NW_003038010.1	+	109379	109427
Sma-1-21342	NW_003038010.1	+	513932	513980
Sma-1-21343	NW_003038011.1	+	602102	602150
Sma-1-21344	NW_003038011.1	-	740437	740389
Sma-1-21345	NW_003038011.1	-	246283	246235
Sma-1-21346	NW_003038012.1	+	407138	407186
Sma-1-21347	NW_003038012.1	-	511118	511070
Sma-1-21348	NW_003038018.1	-	216178	216130
Sma-1-21349	NW_003038021.1	-	127773	127725
Sma-1-21350	NW_003038022.1	-	514859	514811
Sma-1-21351	NW_003038023.1	-	478823	478775
Sma-1-21352	NW_003038026.1	+	271684	271732
Sma-1-21353	NW_003038027.1	-	804882	804834
Sma-1-21354	NW_003038028.1	-	221088	221040
Sma-1-21355	NW_003038029.1	+	523056	523104
Sma-1-21356	NW_003038029.1	+	675552	675600
Sma-1-21357	NW_003038030.1	+	469561	469609
Sma-1-21358	NW_003038031.1	+	652367	652415
Sma-1-21359	NW_003038031.1	+	686461	686509
Sma-1-21360	NW_003038031.1	-	686188	686140
Sma-1-21361	NW_003038031.1	-	652029	651981
Sma-1-21362	NW_003038034.1	-	563522	563474
Sma-1-21363	NW_003038035.1	-	989771	989723
Sma-1-21364	NW_003038035.1	-	848868	848820
Sma-1-21365	NW_003038036.1	-	401212	401164
Sma-1-21366	NW_003038037.1	+	750185	750233
Sma-1-21367	NW_003038037.1	-	1542085	1542037
Sma-1-21368	NW_003038037.1	-	1271635	1271587
Sma-1-21369	NW_003038037.1	-	1156076	1156028
Sma-1-21370	NW_003038037.1	-	1064710	1064662
Sma-1-21371	NW_003038038.1	-	736982	736934
Sma-1-21372	NW_003038219.1	+	121883	121931
Sma-1-21373	NW_003038229.1	-	83825	83777
Sma-1-21374	NW_003038233.1	+	26531	26579
Sma-1-21375	NW_003038233.1	+	208132	208180
Sma-1-21376	NW_003038239.1	+	119566	119614
Sma-1-21377	NW_003038239.1	+	138410	138458
Sma-1-21378	NW_003038239.1	-	144696	144648
Sma-1-21379	NW_003038239.1	-	138153	138105
Sma-1-21380	NW_003038246.1	+	98129	98177

Sma-1-21381	NW_003038246.1	-	97982	97934
Sma-1-21382	NW_003038248.1	-	109339	109291
Sma-1-21383	NW_003038251.1	+	360193	360241
Sma-1-21384	NW_003038251.1	+	390181	390229
Sma-1-21385	NW_003038251.1	-	398150	398102
Sma-1-21386	NW_003038251.1	-	389988	389940
Sma-1-21387	NW_003038254.1	+	214803	214851
Sma-1-21388	NW_003038255.1	-	257191	257143
Sma-1-21389	NW_003038259.1	-	188165	188117
Sma-1-21390	NW_003038260.1	+	54663	54711
Sma-1-21391	NW_003038260.1	-	173376	173328
Sma-1-21392	NW_003038261.1	+	937592	937640
Sma-1-21393	NW_003038410.1	-	23056	23008
Sma-1-21394	NW_003038518.1	+	899671	899719
Sma-1-21395	NW_003038518.1	-	337262	337214
Sma-1-21396	NW_003039127.1	+	911931	911979
Sma-1-21397	NW_003039127.1	+	1751477	1751525
Sma-1-21398	NW_003039127.1	-	2490539	2490491
Sma-1-21399	NW_003039127.1	-	913156	913108
Sma-1-21400	NW_003026474.1	+	71035	71083
Sma-1-21401	NW_003026474.1	-	69834	69786
Sma-1-21402	NW_003026474.1	-	61474	61426
Sma-1-21403	NW_003026482.1	-	34263	34215
Sma-1-21404	NW_003026485.1	-	141056	141008
Sma-1-21405	NW_003026490.1	+	234704	234752
Sma-1-21406	NW_003026506.1	-	47933	47885
Sma-1-21407	NW_003026507.1	+	328491	328539
Sma-1-21408	NW_003026510.1	-	54677	54629
Sma-1-21409	NW_003026514.1	-	137625	137577
Sma-1-21410	NW_003026516.1	+	307381	307429
Sma-1-21411	NW_003026517.1	-	165266	165218
Sma-1-21412	NW_003026575.1	+	83174	83222
Sma-1-21413	NW_003038042.1	-	8509	8461
Sma-1-21414	NW_003038050.1	+	203035	203083
Sma-1-21415	NW_003038052.1	-	234229	234181
Sma-1-21416	NW_003038052.1	-	213975	213927
Sma-1-21417	NW_003038055.1	+	188105	188153
Sma-1-21418	NW_003038057.1	+	382970	383018
Sma-1-21419	NW_003038059.1	-	223904	223856
Sma-1-21420	NW_003038060.1	-	205504	205456
Sma-1-21421	NW_003038062.1	-	45787	45739
Sma-1-21422	NW_003038064.1	+	192095	192143
Sma-1-21423	NW_003038064.1	+	198491	198539
Sma-1-21424	NW_003038065.1	-	94107	94059
Sma-1-21425	NW_003038076.1	-	391390	391342
Sma-1-21426	NW_003038078.1	+	79273	79321
Sma-1-21427	NW_003038078.1	-	164754	164706
Sma-1-21428	NW_003038079.1	+	149278	149326
Sma-1-21429	NW_003038081.1	+	31554	31602
Sma-1-21430	NW_003038081.1	+	237647	237695
Sma-1-21431	NW_003038082.1	+	189095	189143
Sma-1-21432	NW_003038083.1	+	60692	60740

Sma-1-21433	NW_003038083.1	+	81281	81329
Sma-1-21434	NW_003038085.1	-	558358	558310
Sma-1-21435	NW_003038086.1	+	468983	469031
Sma-1-21436	NW_003038086.1	+	760099	760147
Sma-1-21437	NW_003038103.1	+	7881	7929
Sma-1-21438	NW_003038115.1	+	25501	25549
Sma-1-21439	NW_003038118.1	+	281698	281746
Sma-1-21440	NW_003038122.1	+	131098	131146
Sma-1-21441	NW_003038131.1	-	15172	15124
Sma-1-21442	NW_003038132.1	+	415737	415785
Sma-1-21443	NW_003038134.1	+	17932	17980
Sma-1-21444	NW_003038134.1	-	14667	14619
Sma-1-21445	NW_003038136.1	-	47588	47540
Sma-1-21446	NW_003038139.1	+	215475	215523
Sma-1-21447	NW_003038139.1	+	222378	222426
Sma-1-21448	NW_003038142.1	-	331099	331051
Sma-1-21449	NW_003038147.1	+	59967	60015
Sma-1-21450	NW_003038149.1	+	204473	204521
Sma-1-21451	NW_003038150.1	-	108602	108554
Sma-1-21452	NW_003038151.1	+	271141	271189
Sma-1-21453	NW_003038152.1	-	140253	140205
Sma-1-21454	NW_003038152.1	-	49754	49706
Sma-1-21455	NW_003038152.1	-	45909	45861
Sma-1-21456	NW_003038154.1	+	86137	86185
Sma-1-21457	NW_003038156.1	+	56820	56868
Sma-1-21458	NW_003038156.1	-	270697	270649
Sma-1-21459	NW_003038157.1	+	107360	107408
Sma-1-21460	NW_003038157.1	+	517460	517508
Sma-1-21461	NW_003038157.1	-	201750	201702
Sma-1-21462	NW_003038158.1	-	317227	317179
Sma-1-21463	NW_003038159.1	+	231338	231386
Sma-1-21464	NW_003038159.1	-	231000	230952
Sma-1-21465	NW_003038160.1	+	255302	255350
Sma-1-21466	NW_003038160.1	-	588344	588296
Sma-1-21467	NW_003038160.1	-	585365	585317
Sma-1-21468	NW_003038160.1	-	47591	47543
Sma-1-21469	NW_003038177.1	+	93433	93481
Sma-1-21470	NW_003038196.1	+	22553	22601
Sma-1-21471	NS_000200.1	+	2442512	2442560
Sma-1-21472	NS_000200.1	+	2675609	2675657
Sma-1-21473	NS_000200.1	+	4214647	4214695
Sma-1-21474	NS_000200.1	+	5364344	5364392
Sma-1-21475	NS_000200.1	+	7029965	7030013
Sma-1-21476	NS_000200.1	+	7720733	7720781
Sma-1-21477	NS_000200.1	+	12752576	12752624
Sma-1-21478	NS_000200.1	+	12847401	12847449
Sma-1-21479	NS_000200.1	+	14519462	14519510
Sma-1-21480	NS_000200.1	+	14691382	14691430
Sma-1-21481	NS_000200.1	+	16905352	16905400
Sma-1-21482	NS_000200.1	+	20159916	20159964
Sma-1-21483	NS_000200.1	+	20695363	20695411
Sma-1-21484	NS_000200.1	+	23633900	23633948

Sma-1-21485	NS_000200.1	+	24837014	24837062
Sma-1-21486	NS_000200.1	+	25703728	25703776
Sma-1-21487	NS_000200.1	+	31409707	31409755
Sma-1-21488	NS_000200.1	+	32712619	32712667
Sma-1-21489	NS_000200.1	+	34716236	34716284
Sma-1-21490	NS_000200.1	+	36208072	36208120
Sma-1-21491	NS_000200.1	+	39672490	39672538
Sma-1-21492	NS_000200.1	+	40723257	40723305
Sma-1-21493	NS_000200.1	+	41628985	41629033
Sma-1-21494	NS_000200.1	+	43639913	43639961
Sma-1-21495	NS_000200.1	+	45412563	45412611
Sma-1-21496	NS_000200.1	+	45806543	45806591
Sma-1-21497	NS_000200.1	+	45953578	45953626
Sma-1-21498	NS_000200.1	+	47888530	47888578
Sma-1-21499	NS_000200.1	+	48338424	48338472
Sma-1-21500	NS_000200.1	+	49408222	49408270
Sma-1-21501	NS_000200.1	+	50982831	50982879
Sma-1-21502	NS_000200.1	+	59493081	59493129
Sma-1-21503	NS_000200.1	+	59906522	59906570
Sma-1-21504	NS_000200.1	+	60559624	60559672
Sma-1-21505	NS_000200.1	+	61238099	61238147
Sma-1-21506	NS_000200.1	+	61286500	61286548
Sma-1-21507	NS_000200.1	+	61525554	61525602
Sma-1-21508	NS_000200.1	+	61566089	61566137
Sma-1-21509	NS_000200.1	+	63070113	63070161
Sma-1-21510	NS_000200.1	+	64989646	64989694
Sma-1-21511	NS_000200.1	+	65996827	65996875
Sma-1-21512	NS_000200.1	+	66552876	66552924
Sma-1-21513	NS_000200.1	+	68206237	68206285
Sma-1-21514	NS_000200.1	+	69344466	69344514
Sma-1-21515	NS_000200.1	+	71541524	71541572
Sma-1-21516	NS_000200.1	+	76716412	76716460
Sma-1-21517	NS_000200.1	+	78342041	78342089
Sma-1-21518	NS_000200.1	+	78688930	78688978
Sma-1-21519	NS_000200.1	+	78902285	78902333
Sma-1-21520	NS_000200.1	+	79923126	79923174
Sma-1-21521	NS_000200.1	+	79944731	79944779
Sma-1-21522	NS_000200.1	+	81810273	81810321
Sma-1-21523	NS_000200.1	+	89065343	89065391
Sma-1-21524	NS_000200.1	+	89894303	89894351
Sma-1-21525	NS_000200.1	+	89930037	89930085
Sma-1-21526	NS_000200.1	+	91810946	91810994
Sma-1-21527	NS_000200.1	+	93058103	93058151
Sma-1-21528	NS_000200.1	+	95062562	95062610
Sma-1-21529	NS_000200.1	+	95644675	95644723
Sma-1-21530	NS_000200.1	+	95921054	95921102
Sma-1-21531	NS_000200.1	+	97996336	97996384
Sma-1-21532	NS_000200.1	+	99021860	99021908
Sma-1-21533	NS_000200.1	+	100015207	100015255
Sma-1-21534	NS_000200.1	+	102806682	102806730
Sma-1-21535	NS_000200.1	+	102879725	102879773
Sma-1-21536	NS_000200.1	+	104604553	104604601

Sma-1-21537	NS_000200.1	+	109620437	109620485
Sma-1-21538	NS_000200.1	+	111012020	111012068
Sma-1-21539	NS_000200.1	+	111018891	111018939
Sma-1-21540	NS_000200.1	+	111276167	111276215
Sma-1-21541	NS_000200.1	+	113989430	113989478
Sma-1-21542	NS_000200.1	+	114828976	114829024
Sma-1-21543	NS_000200.1	+	116628404	116628452
Sma-1-21544	NS_000200.1	+	118801492	118801540
Sma-1-21545	NS_000200.1	+	118846878	118846926
Sma-1-21546	NS_000200.1	+	119654722	119654770
Sma-1-21547	NS_000200.1	+	119796593	119796641
Sma-1-21548	NS_000200.1	+	120359903	120359951
Sma-1-21549	NS_000200.1	+	121935396	121935444
Sma-1-21550	NS_000200.1	+	127066992	127067040
Sma-1-21551	NS_000200.1	+	130269274	130269322
Sma-1-21552	NS_000200.1	+	134212936	134212984
Sma-1-21553	NS_000200.1	+	134243260	134243308
Sma-1-21554	NS_000200.1	+	134245786	134245834
Sma-1-21555	NS_000200.1	+	134754452	134754500
Sma-1-21556	NS_000200.1	+	137598046	137598094
Sma-1-21557	NS_000200.1	+	137700537	137700585
Sma-1-21558	NS_000200.1	+	138733968	138734016
Sma-1-21559	NS_000200.1	+	139905797	139905845
Sma-1-21560	NS_000200.1	+	140182904	140182952
Sma-1-21561	NS_000200.1	+	141151457	141151505
Sma-1-21562	NS_000200.1	+	141197796	141197844
Sma-1-21563	NS_000200.1	+	142448760	142448808
Sma-1-21564	NS_000200.1	+	142802128	142802176
Sma-1-21565	NS_000200.1	+	144358770	144358818
Sma-1-21566	NS_000200.1	+	147535862	147535910
Sma-1-21567	NS_000200.1	+	148852245	148852293
Sma-1-21568	NS_000200.1	+	152730683	152730731
Sma-1-21569	NS_000200.1	+	152830371	152830419
Sma-1-21570	NS_000200.1	+	154248564	154248612
Sma-1-21571	NS_000200.1	+	154940897	154940945
Sma-1-21572	NS_000200.1	+	155448552	155448600
Sma-1-21573	NS_000200.1	+	155448885	155448933
Sma-1-21574	NS_000200.1	+	161838506	161838554
Sma-1-21575	NS_000200.1	+	164414841	164414889
Sma-1-21576	NS_000200.1	+	166501704	166501752
Sma-1-21577	NS_000200.1	+	167616857	167616905
Sma-1-21578	NS_000200.1	+	168327013	168327061
Sma-1-21579	NS_000200.1	+	170077909	170077957
Sma-1-21580	NS_000200.1	+	170779232	170779280
Sma-1-21581	NS_000200.1	+	173328070	173328118
Sma-1-21582	NS_000200.1	+	175788949	175788997
Sma-1-21583	NS_000200.1	+	176072517	176072565
Sma-1-21584	NS_000200.1	+	181504646	181504694
Sma-1-21585	NS_000200.1	+	182646510	182646558
Sma-1-21586	NS_000200.1	+	185851026	185851074
Sma-1-21587	NS_000200.1	+	186365444	186365492
Sma-1-21588	NS_000200.1	+	186889530	186889578

Sma-1-21589	NS_000200.1	+	191188075	191188123
Sma-1-21590	NS_000200.1	+	191529239	191529287
Sma-1-21591	NS_000200.1	+	191841185	191841233
Sma-1-21592	NS_000200.1	+	194099841	194099889
Sma-1-21593	NS_000200.1	+	196150252	196150300
Sma-1-21594	NS_000200.1	+	199238002	199238050
Sma-1-21595	NS_000200.1	+	200780293	200780341
Sma-1-21596	NS_000200.1	+	201930467	201930515
Sma-1-21597	NS_000200.1	+	202607872	202607920
Sma-1-21598	NS_000200.1	+	202925120	202925168
Sma-1-21599	NS_000200.1	+	202925453	202925501
Sma-1-21600	NS_000200.1	+	205598068	205598116
Sma-1-21601	NS_000200.1	+	206421459	206421507
Sma-1-21602	NS_000200.1	+	207754087	207754135
Sma-1-21603	NS_000200.1	+	209660864	209660912
Sma-1-21604	NS_000200.1	+	210262609	210262657
Sma-1-21605	NS_000200.1	+	212119696	212119744
Sma-1-21606	NS_000200.1	+	213171510	213171558
Sma-1-21607	NS_000200.1	+	215185927	215185975
Sma-1-21608	NS_000200.1	+	217094192	217094240
Sma-1-21609	NS_000200.1	+	219375647	219375695
Sma-1-21610	NS_000200.1	+	219429970	219430018
Sma-1-21611	NS_000200.1	+	220792681	220792729
Sma-1-21612	NS_000200.1	+	221207274	221207322
Sma-1-21613	NS_000200.1	+	226927602	226927650
Sma-1-21614	NS_000200.1	+	227332155	227332203
Sma-1-21615	NS_000200.1	+	228129752	228129800
Sma-1-21616	NS_000200.1	+	228731077	228731125
Sma-1-21617	NS_000200.1	+	239779445	239779493
Sma-1-21618	NS_000200.1	+	242723957	242724005
Sma-1-21619	NS_000200.1	+	242876453	242876501
Sma-1-21620	NS_000200.1	+	243605428	243605476
Sma-1-21621	NS_000200.1	+	244930125	244930173
Sma-1-21622	NS_000200.1	+	244964219	244964267
Sma-1-21623	NS_000200.1	+	250905594	250905642
Sma-1-21624	NS_000200.1	+	253269610	253269658
Sma-1-21625	NS_000200.1	+	256477929	256477977
Sma-1-21626	NS_000200.1	+	258887427	258887475
Sma-1-21627	NS_000200.1	+	259588786	259588834
Sma-1-21628	NS_000200.1	+	262028672	262028720
Sma-1-21629	NS_000200.1	+	262035068	262035116
Sma-1-21630	NS_000200.1	+	264491909	264491957
Sma-1-21631	NS_000200.1	+	267285476	267285524
Sma-1-21632	NS_000200.1	+	267866420	267866468
Sma-1-21633	NS_000200.1	+	268279266	268279314
Sma-1-21634	NS_000200.1	+	269193597	269193645
Sma-1-21635	NS_000200.1	+	269399690	269399738
Sma-1-21636	NS_000200.1	+	269724318	269724366
Sma-1-21637	NS_000200.1	+	270304029	270304077
Sma-1-21638	NS_000200.1	+	270324618	270324666
Sma-1-21639	NS_000200.1	+	272717576	272717624
Sma-1-21640	NS_000200.1	+	273008692	273008740

Sma-1-21641	NS_000200.1	+	274802772	274802820
Sma-1-21642	NS_000200.1	+	277080796	277080844
Sma-1-21643	NS_000200.1	+	277845825	277845873
Sma-1-21644	NS_000200.1	+	278650325	278650373
Sma-1-21645	NS_000200.1	+	279988642	279988690
Sma-1-21646	NS_000200.1	+	282836207	282836255
Sma-1-21647	NS_000200.1	+	283209049	283209097
Sma-1-21648	NS_000200.1	+	286255605	286255653
Sma-1-21649	NS_000200.1	+	286262508	286262556
Sma-1-21650	NS_000200.1	+	289587710	289587758
Sma-1-21651	NS_000200.1	+	291233086	291233134
Sma-1-21652	NS_000200.1	+	292178384	292178432
Sma-1-21653	NS_000200.1	+	293883846	293883894
Sma-1-21654	NS_000200.1	+	294788933	294788981
Sma-1-21655	NS_000200.1	+	295358280	295358328
Sma-1-21656	NS_000200.1	+	295768380	295768428
Sma-1-21657	NS_000200.1	+	296619359	296619407
Sma-1-21658	NS_000200.1	+	297007233	297007281
Sma-1-21659	NS_000200.1	+	299415678	299415726
Sma-1-21660	NS_000200.1	+	300985873	300985921
Sma-1-21661	NS_000200.1	+	302314840	302314888
Sma-1-21662	NS_000200.1	+	303164836	303164884
Sma-1-21663	NS_000200.1	+	303363922	303363970
Sma-1-21664	NS_000200.1	+	303675000	303675048
Sma-1-21665	NS_000200.1	+	308127106	308127154
Sma-1-21666	NS_000200.1	+	308302304	308302352
Sma-1-21667	NS_000200.1	+	308690056	308690104
Sma-1-21668	NS_000200.1	+	308919312	308919360
Sma-1-21669	NS_000200.1	+	312609630	312609678
Sma-1-21670	NS_000200.1	+	312791231	312791279
Sma-1-21671	NS_000200.1	+	313960348	313960396
Sma-1-21672	NS_000200.1	+	313979192	313979240
Sma-1-21673	NS_000200.1	+	315739257	315739305
Sma-1-21674	NS_000200.1	+	317115549	317115597
Sma-1-21675	NS_000200.1	+	317842846	317842894
Sma-1-21676	NS_000200.1	+	318691965	318692013
Sma-1-21677	NS_000200.1	+	318721953	318722001
Sma-1-21678	NS_000200.1	+	319334552	319334600
Sma-1-21679	NS_000200.1	+	319733490	319733538
Sma-1-21680	NS_000200.1	+	320250361	320250409
Sma-1-21681	NS_000200.1	+	321926933	321926981
Sma-1-21682	NS_000200.1	+	322274513	322274561
Sma-1-21683	NS_000200.1	+	323226438	323226486
Sma-1-21684	NS_000200.1	+	324551243	324551291
Sma-1-21685	NS_000200.1	+	326704752	326704800
Sma-1-21686	NS_000200.1	+	326771287	326771335
Sma-1-21687	NS_000200.1	+	326907445	326907493
Sma-1-21688	NS_000200.1	+	329768384	329768432
Sma-1-21689	NS_000200.1	+	331835929	331835977
Sma-1-21690	NS_000200.1	+	332584353	332584401
Sma-1-21691	NS_000200.1	+	333164559	333164607
Sma-1-21692	NS_000200.1	+	334146692	334146740

Sma-1-21693	NS_000200.1	+	335255444	335255492
Sma-1-21694	NS_000200.1	+	336864284	336864332
Sma-1-21695	NS_000200.1	+	337298369	337298417
Sma-1-21696	NS_000200.1	+	338537126	338537174
Sma-1-21697	NS_000200.1	+	339897006	339897054
Sma-1-21698	NS_000200.1	+	342536504	342536552
Sma-1-21699	NS_000200.1	+	345887810	345887858
Sma-1-21700	NS_000200.1	+	351531880	351531928
Sma-1-21701	NS_000200.1	+	352260068	352260116
Sma-1-21702	NS_000200.1	+	352260401	352260449
Sma-1-21703	NS_000200.1	+	355762363	355762411
Sma-1-21704	NS_000200.1	+	356307632	356307680
Sma-1-21705	NS_000200.1	+	356605311	356605359
Sma-1-21706	NS_000200.1	+	356605644	356605692
Sma-1-21707	NS_000200.1	+	357213368	357213416
Sma-1-21708	NS_000200.1	+	359233551	359233599
Sma-1-21709	NS_000200.1	+	360897977	360898025
Sma-1-21710	NS_000200.1	+	361222676	361222724
Sma-1-21711	NS_000200.1	+	362187152	362187200
Sma-1-21712	NS_000200.1	+	362276646	362276694
Sma-1-21713	NS_000200.1	+	362389493	362389541
Sma-1-21714	NS_000200.1	+	363686005	363686053
Sma-1-21715	NS_000200.1	+	364072257	364072305
Sma-1-21716	NS_000200.1	+	367214972	367215020
Sma-1-21717	NS_000200.1	+	367277226	367277274
Sma-1-21718	NS_000200.1	+	367718093	367718141
Sma-1-21719	NS_000200.1	+	367718426	367718474
Sma-1-21720	NS_000200.1	+	369372637	369372685
Sma-1-21721	NS_000200.1	+	369867859	369867907
Sma-1-21722	NS_000200.1	+	371288067	371288115
Sma-1-21723	NS_000200.1	+	372358940	372358988
Sma-1-21724	NS_000200.1	+	372458745	372458793
Sma-1-21725	NS_000200.1	+	372699541	372699589
Sma-1-21726	NS_000200.1	+	373208495	373208543
Sma-1-21727	NS_000200.1	+	374939628	374939676
Sma-1-21728	NS_000200.1	+	378944561	378944609
Sma-1-21729	NS_000200.1	+	379655228	379655276
Sma-1-21730	NS_000200.1	+	379985986	379986034
Sma-1-21731	NS_000200.1	+	379994151	379994199
Sma-1-21732	NS_000200.1	-	382644514	382644466
Sma-1-21733	NS_000200.1	-	382095928	382095880
Sma-1-21734	NS_000200.1	-	378189045	378188997
Sma-1-21735	NS_000200.1	-	376682645	376682597
Sma-1-21736	NS_000200.1	-	376507188	376507140
Sma-1-21737	NS_000200.1	-	374651635	374651587
Sma-1-21738	NS_000200.1	-	374403457	374403409
Sma-1-21739	NS_000200.1	-	372316653	372316605
Sma-1-21740	NS_000200.1	-	368818408	368818360
Sma-1-21741	NS_000200.1	-	367140947	367140899
Sma-1-21742	NS_000200.1	-	366339630	366339582
Sma-1-21743	NS_000200.1	-	363609433	363609385
Sma-1-21744	NS_000200.1	-	362390904	362390856

Sma-1-21745	NS_000200.1	-	357507637	357507589
Sma-1-21746	NS_000200.1	-	357449648	357449600
Sma-1-21747	NS_000200.1	-	357086503	357086455
Sma-1-21748	NS_000200.1	-	353145494	353145446
Sma-1-21749	NS_000200.1	-	353022048	353022000
Sma-1-21750	NS_000200.1	-	351999094	351999046
Sma-1-21751	NS_000200.1	-	351998761	351998713
Sma-1-21752	NS_000200.1	-	351998428	351998380
Sma-1-21753	NS_000200.1	-	351998095	351998047
Sma-1-21754	NS_000200.1	-	349317818	349317770
Sma-1-21755	NS_000200.1	-	347003592	347003544
Sma-1-21756	NS_000200.1	-	345686322	345686274
Sma-1-21757	NS_000200.1	-	345609133	345609085
Sma-1-21758	NS_000200.1	-	341974095	341974047
Sma-1-21759	NS_000200.1	-	339728900	339728852
Sma-1-21760	NS_000200.1	-	339002473	339002425
Sma-1-21761	NS_000200.1	-	337689203	337689155
Sma-1-21762	NS_000200.1	-	328489634	328489586
Sma-1-21763	NS_000200.1	-	328434663	328434615
Sma-1-21764	NS_000200.1	-	327171796	327171748
Sma-1-21765	NS_000200.1	-	326855892	326855844
Sma-1-21766	NS_000200.1	-	326704483	326704435
Sma-1-21767	NS_000200.1	-	326341708	326341660
Sma-1-21768	NS_000200.1	-	324709592	324709544
Sma-1-21769	NS_000200.1	-	323345151	323345103
Sma-1-21770	NS_000200.1	-	323048764	323048716
Sma-1-21771	NS_000200.1	-	322487732	322487684
Sma-1-21772	NS_000200.1	-	322210708	322210660
Sma-1-21773	NS_000200.1	-	320646336	320646288
Sma-1-21774	NS_000200.1	-	318729922	318729874
Sma-1-21775	NS_000200.1	-	318721760	318721712
Sma-1-21776	NS_000200.1	-	317787542	317787494
Sma-1-21777	NS_000200.1	-	317469974	317469926
Sma-1-21778	NS_000200.1	-	316420606	316420558
Sma-1-21779	NS_000200.1	-	315901871	315901823
Sma-1-21780	NS_000200.1	-	315739110	315739062
Sma-1-21781	NS_000200.1	-	315316895	315316847
Sma-1-21782	NS_000200.1	-	313985478	313985430
Sma-1-21783	NS_000200.1	-	313978935	313978887
Sma-1-21784	NS_000200.1	-	311201047	311200999
Sma-1-21785	NS_000200.1	-	308146547	308146499
Sma-1-21786	NS_000200.1	-	305811491	305811443
Sma-1-21787	NS_000200.1	-	302749289	302749241
Sma-1-21788	NS_000200.1	-	301255422	301255374
Sma-1-21789	NS_000200.1	-	299771275	299771227
Sma-1-21790	NS_000200.1	-	297340275	297340227
Sma-1-21791	NS_000200.1	-	297337296	297337248
Sma-1-21792	NS_000200.1	-	296799522	296799474
Sma-1-21793	NS_000200.1	-	296619021	296618973
Sma-1-21794	NS_000200.1	-	296200395	296200347
Sma-1-21795	NS_000200.1	-	295452670	295452622
Sma-1-21796	NS_000200.1	-	295002810	295002762

Sma-1-21797	NS_000200.1	-	292745977	292745929
Sma-1-21798	NS_000200.1	-	292655478	292655430
Sma-1-21799	NS_000200.1	-	292651633	292651585
Sma-1-21800	NS_000200.1	-	291587340	291587292
Sma-1-21801	NS_000200.1	-	288596312	288596264
Sma-1-21802	NS_000200.1	-	287541535	287541487
Sma-1-21803	NS_000200.1	-	284169657	284169609
Sma-1-21804	NS_000200.1	-	283494858	283494810
Sma-1-21805	NS_000200.1	-	283205784	283205736
Sma-1-21806	NS_000200.1	-	281977016	281976968
Sma-1-21807	NS_000200.1	-	279608998	279608950
Sma-1-21808	NS_000200.1	-	272248451	272248403
Sma-1-21809	NS_000200.1	-	267370957	267370909
Sma-1-21810	NS_000200.1	-	266608564	266608516
Sma-1-21811	NS_000200.1	-	264708071	264708023
Sma-1-21812	NS_000200.1	-	262245724	262245676
Sma-1-21813	NS_000200.1	-	261397773	261397725
Sma-1-21814	NS_000200.1	-	260770629	260770581
Sma-1-21815	NS_000200.1	-	260465976	260465928
Sma-1-21816	NS_000200.1	-	258495961	258495913
Sma-1-21817	NS_000200.1	-	257281705	257281657
Sma-1-21818	NS_000200.1	-	257261451	257261403
Sma-1-21819	NS_000200.1	-	255822009	255821961
Sma-1-21820	NS_000200.1	-	253954108	253954060
Sma-1-21821	NS_000200.1	-	253921464	253921416
Sma-1-21822	NS_000200.1	-	253268409	253268361
Sma-1-21823	NS_000200.1	-	253260049	253260001
Sma-1-21824	NS_000200.1	-	252640375	252640327
Sma-1-21825	NS_000200.1	-	251697494	251697446
Sma-1-21826	NS_000200.1	-	251427044	251426996
Sma-1-21827	NS_000200.1	-	251311485	251311437
Sma-1-21828	NS_000200.1	-	251220119	251220071
Sma-1-21829	NS_000200.1	-	249335064	249335016
Sma-1-21830	NS_000200.1	-	248851263	248851215
Sma-1-21831	NS_000200.1	-	248710360	248710312
Sma-1-21832	NS_000200.1	-	247505278	247505230
Sma-1-21833	NS_000200.1	-	244963946	244963898
Sma-1-21834	NS_000200.1	-	244929787	244929739
Sma-1-21835	NS_000200.1	-	241528662	241528614
Sma-1-21836	NS_000200.1	-	241175596	241175548
Sma-1-21837	NS_000200.1	-	237639114	237639066
Sma-1-21838	NS_000200.1	-	236585899	236585851
Sma-1-21839	NS_000200.1	-	236195701	236195653
Sma-1-21840	NS_000200.1	-	234956348	234956300
Sma-1-21841	NS_000200.1	-	232617028	232616980
Sma-1-21842	NS_000200.1	-	228835057	228835009
Sma-1-21843	NS_000200.1	-	228268087	228268039
Sma-1-21844	NS_000200.1	-	227773933	227773885
Sma-1-21845	NS_000200.1	-	226624558	226624510
Sma-1-21846	NS_000200.1	-	226242983	226242935
Sma-1-21847	NS_000200.1	-	224834157	224834109
Sma-1-21848	NS_000200.1	-	224328456	224328408

Sma-1-21849	NS_000200.1	-	224010801	224010753
Sma-1-21850	NS_000200.1	-	223430924	223430876
Sma-1-21851	NS_000200.1	-	221578349	221578301
Sma-1-21852	NS_000200.1	-	220729416	220729368
Sma-1-21853	NS_000200.1	-	214257360	214257312
Sma-1-21854	NS_000200.1	-	213171251	213171203
Sma-1-21855	NS_000200.1	-	212088706	212088658
Sma-1-21856	NS_000200.1	-	211662822	211662774
Sma-1-21857	NS_000200.1	-	210399405	210399357
Sma-1-21858	NS_000200.1	-	208987110	208987062
Sma-1-21859	NS_000200.1	-	207731858	207731810
Sma-1-21860	NS_000200.1	-	206982768	206982720
Sma-1-21861	NS_000200.1	-	206953285	206953237
Sma-1-21862	NS_000200.1	-	206334810	206334762
Sma-1-21863	NS_000200.1	-	206315840	206315792
Sma-1-21864	NS_000200.1	-	199362935	199362887
Sma-1-21865	NS_000200.1	-	199302960	199302912
Sma-1-21866	NS_000200.1	-	197793822	197793774
Sma-1-21867	NS_000200.1	-	197732301	197732253
Sma-1-21868	NS_000200.1	-	197687010	197686962
Sma-1-21869	NS_000200.1	-	197250299	197250251
Sma-1-21870	NS_000200.1	-	195059072	195059024
Sma-1-21871	NS_000200.1	-	194961996	194961948
Sma-1-21872	NS_000200.1	-	190551506	190551458
Sma-1-21873	NS_000200.1	-	190321518	190321470
Sma-1-21874	NS_000200.1	-	190216816	190216768
Sma-1-21875	NS_000200.1	-	182916355	182916307
Sma-1-21876	NS_000200.1	-	181885205	181885157
Sma-1-21877	NS_000200.1	-	180512734	180512686
Sma-1-21878	NS_000200.1	-	177914450	177914402
Sma-1-21879	NS_000200.1	-	173327786	173327738
Sma-1-21880	NS_000200.1	-	173130987	173130939
Sma-1-21881	NS_000200.1	-	173130654	173130606
Sma-1-21882	NS_000200.1	-	170071672	170071624
Sma-1-21883	NS_000200.1	-	165496374	165496326
Sma-1-21884	NS_000200.1	-	164715977	164715929
Sma-1-21885	NS_000200.1	-	163585202	163585154
Sma-1-21886	NS_000200.1	-	163475009	163474961
Sma-1-21887	NS_000200.1	-	163149419	163149371
Sma-1-21888	NS_000200.1	-	162806206	162806158
Sma-1-21889	NS_000200.1	-	162297313	162297265
Sma-1-21890	NS_000200.1	-	161968025	161967977
Sma-1-21891	NS_000200.1	-	161861573	161861525
Sma-1-21892	NS_000200.1	-	160464189	160464141
Sma-1-21893	NS_000200.1	-	160429450	160429402
Sma-1-21894	NS_000200.1	-	160391434	160391386
Sma-1-21895	NS_000200.1	-	159916794	159916746
Sma-1-21896	NS_000200.1	-	159859043	159858995
Sma-1-21897	NS_000200.1	-	156660533	156660485
Sma-1-21898	NS_000200.1	-	156660200	156660152
Sma-1-21899	NS_000200.1	-	156659867	156659819
Sma-1-21900	NS_000200.1	-	155352087	155352039

Sma-1-21901	NS_000200.1	-	153570930	153570882
Sma-1-21902	NS_000200.1	-	151739984	151739936
Sma-1-21903	NS_000200.1	-	151505973	151505925
Sma-1-21904	NS_000200.1	-	151505640	151505592
Sma-1-21905	NS_000200.1	-	149429794	149429746
Sma-1-21906	NS_000200.1	-	149404334	149404286
Sma-1-21907	NS_000200.1	-	149029327	149029279
Sma-1-21908	NS_000200.1	-	147375060	147375012
Sma-1-21909	NS_000200.1	-	145813686	145813638
Sma-1-21910	NS_000200.1	-	144337746	144337698
Sma-1-21911	NS_000200.1	-	143241578	143241530
Sma-1-21912	NS_000200.1	-	141475321	141475273
Sma-1-21913	NS_000200.1	-	139830280	139830232
Sma-1-21914	NS_000200.1	-	137735716	137735668
Sma-1-21915	NS_000200.1	-	134940564	134940516
Sma-1-21916	NS_000200.1	-	134260306	134260258
Sma-1-21917	NS_000200.1	-	131556410	131556362
Sma-1-21918	NS_000200.1	-	129861911	129861863
Sma-1-21919	NS_000200.1	-	128723783	128723735
Sma-1-21920	NS_000200.1	-	125812246	125812198
Sma-1-21921	NS_000200.1	-	124506327	124506279
Sma-1-21922	NS_000200.1	-	123171901	123171853
Sma-1-21923	NS_000200.1	-	123005519	123005471
Sma-1-21924	NS_000200.1	-	118253637	118253589
Sma-1-21925	NS_000200.1	-	117998890	117998842
Sma-1-21926	NS_000200.1	-	115568038	115567990
Sma-1-21927	NS_000200.1	-	113990655	113990607
Sma-1-21928	NS_000200.1	-	109941121	109941073
Sma-1-21929	NS_000200.1	-	103236889	103236841
Sma-1-21930	NS_000200.1	-	98421701	98421653
Sma-1-21931	NS_000200.1	-	96539496	96539448
Sma-1-21932	NS_000200.1	-	95913121	95913073
Sma-1-21933	NS_000200.1	-	94664428	94664380
Sma-1-21934	NS_000200.1	-	92179780	92179732
Sma-1-21935	NS_000200.1	-	91294197	91294149
Sma-1-21936	NS_000200.1	-	91223367	91223319
Sma-1-21937	NS_000200.1	-	87910008	87909960
Sma-1-21938	NS_000200.1	-	81082009	81081961
Sma-1-21939	NS_000200.1	-	79926523	79926475
Sma-1-21940	NS_000200.1	-	78998609	78998561
Sma-1-21941	NS_000200.1	-	78998276	78998228
Sma-1-21942	NS_000200.1	-	74273221	74273173
Sma-1-21943	NS_000200.1	-	71537249	71537201
Sma-1-21944	NS_000200.1	-	70502620	70502572
Sma-1-21945	NS_000200.1	-	64437730	64437682
Sma-1-21946	NS_000200.1	-	63175625	63175577
Sma-1-21947	NS_000200.1	-	63175292	63175244
Sma-1-21948	NS_000200.1	-	61323614	61323566
Sma-1-21949	NS_000200.1	-	60682193	60682145
Sma-1-21950	NS_000200.1	-	59575441	59575393
Sma-1-21951	NS_000200.1	-	57545853	57545805
Sma-1-21952	NS_000200.1	-	55630048	55630000

Sma-1-21953	NS_000200.1	-	54602235	54602187
Sma-1-21954	NS_000200.1	-	54567658	54567610
Sma-1-21955	NS_000200.1	-	52789570	52789522
Sma-1-21956	NS_000200.1	-	51565277	51565229
Sma-1-21957	NS_000200.1	-	49065687	49065639
Sma-1-21958	NS_000200.1	-	48364214	48364166
Sma-1-21959	NS_000200.1	-	42996965	42996917
Sma-1-21960	NS_000200.1	-	42916702	42916654
Sma-1-21961	NS_000200.1	-	39780267	39780219
Sma-1-21962	NS_000200.1	-	39048310	39048262
Sma-1-21963	NS_000200.1	-	36775222	36775174
Sma-1-21964	NS_000200.1	-	35816345	35816297
Sma-1-21965	NS_000200.1	-	31682788	31682740
Sma-1-21966	NS_000200.1	-	30015712	30015664
Sma-1-21967	NS_000200.1	-	29272127	29272079
Sma-1-21968	NS_000200.1	-	28607739	28607691
Sma-1-21969	NS_000200.1	-	27978044	27977996
Sma-1-21970	NS_000200.1	-	25613865	25613817
Sma-1-21971	NS_000200.1	-	24712357	24712309
Sma-1-21972	NS_000200.1	-	18938612	18938564
Sma-1-21973	NS_000200.1	-	17251788	17251740
Sma-1-21974	NS_000200.1	-	16904784	16904736
Sma-1-21975	NS_000200.1	-	15668553	15668505
Sma-1-21976	NS_000200.1	-	14277411	14277363
Sma-1-21977	NS_000200.1	-	12065242	12065194
Sma-1-21978	NS_000200.1	-	11486019	11485971
Sma-1-21979	NS_000200.1	-	11080558	11080510
Sma-1-21980	NS_000200.1	-	9371194	9371146
Sma-1-21981	NS_000200.1	-	8797198	8797150
Sma-1-21982	NS_000200.1	-	8518195	8518147
Sma-1-21983	NS_000200.1	-	8269273	8269225
Sma-1-21984	NS_000200.1	-	6128509	6128461
Sma-1-21985	NS_000200.1	-	5968350	5968302
Sma-1-21986	NS_000200.1	-	3463476	3463428
Sma-1-21987	NS_000200.1	-	2311860	2311812
Sma-1-21988	NS_000200.1	-	2299500	2299452
Sma-1-21989	NS_000200.1	-	711079	711031
Sma-1-21990	NS_000200.1	-	24825	24777
Sma-1-21991	NW_003020957.1	-	452	404
Sma-1-21992	NW_003029647.1	-	2929	2881
Sma-1-21993	NW_003034530.1	-	289	241
Sma-1-21994	NW_003038244.1	+	136522	136570
Sma-1-21995	NW_003037796.1	-	509471	509423
Sma-1-21996	NW_003037798.1	+	1221327	1221375
Sma-1-21997	NW_003037805.1	+	224869	224917
Sma-1-21998	NW_003037806.1	-	116316	116268
Sma-1-21999	NW_003037844.1	+	555418	555466
Sma-1-22000	NW_003037863.1	+	149113	149161
Sma-1-22001	NW_003037921.1	-	21606	21558
Sma-1-22002	NW_003037922.1	+	555116	555164
Sma-1-22003	NW_003037942.1	-	1045757	1045709
Sma-1-22004	NW_003037977.1	+	974284	974332

Sma-1-22005	NW_003038016.1	+	533861	533909
Sma-1-22006	NW_003038031.1	+	384145	384193
Sma-1-22007	NW_003038260.1	-	71845	71797
Sma-1-22008	NW_003039127.1	-	424303	424255
Sma-1-22009	NW_003026515.1	+	334936	334984
Sma-1-22010	NW_003038081.1	-	250511	250463
Sma-1-22011	NW_003038126.1	-	86861	86813
Sma-1-22012	NS_000200.1	+	15480485	15480533
Sma-1-22013	NS_000200.1	+	26782713	26782761
Sma-1-22014	NS_000200.1	+	86738864	86738912
Sma-1-22015	NS_000200.1	+	106907480	106907528
Sma-1-22016	NS_000200.1	+	170479113	170479161
Sma-1-22017	NS_000200.1	+	209260304	209260352
Sma-1-22018	NS_000200.1	+	231650398	231650446
Sma-1-22019	NS_000200.1	+	244661903	244661951
Sma-1-22020	NS_000200.1	+	290979347	290979395
Sma-1-22021	NS_000200.1	+	315281727	315281775
Sma-1-22022	NS_000200.1	-	367684293	367684245
Sma-1-22023	NS_000200.1	-	366344663	366344615
Sma-1-22024	NS_000200.1	-	326867632	326867584
Sma-1-22025	NS_000200.1	-	323243620	323243572
Sma-1-22026	NS_000200.1	-	280097273	280097225
Sma-1-22027	NS_000200.1	-	269412554	269412506
Sma-1-22028	NS_000200.1	-	186805665	186805617
Sma-1-22029	NS_000200.1	-	169551741	169551693
Sma-1-22030	NS_000200.1	-	113501802	113501754
Sma-1-22031	NS_000200.1	-	28311530	28311482
Sma-1-22032	NS_000200.1	-	8719115	8719067
Sma-1-22033	NW_003022720.1	+	1070	1118
Sma-1-22034	NW_003037920.1	-	248839	248791
Sma-1-22035	NW_003037853.1	+	735914	735962
Sma-1-22036	NW_003037888.1	+	592933	592981
Sma-1-22037	NW_003037896.1	-	91741	91693
Sma-1-22038	NW_003037974.1	-	6026	5978
Sma-1-22039	NW_003038260.1	-	258282	258234
Sma-1-22040	NW_003038498.1	+	37686	37734
Sma-1-22041	NS_000200.1	+	96227965	96228013
Sma-1-22042	NS_000200.1	+	132801425	132801473
Sma-1-22043	NS_000200.1	+	334457898	334457946
Sma-1-22044	NS_000200.1	+	367983461	367983509
Sma-1-22045	NS_000200.1	-	323430057	323430009
Sma-1-22046	NS_000200.1	-	204681332	204681284
Sma-1-22047	NS_000200.1	-	169429646	169429598
Sma-1-22048	NS_000200.1	-	143012164	143012116
Sma-1-22049	NW_003024967.1	-	1111	1063
Sma-1-22050	NW_003025215.1	+	466	514
Sma-1-22051	NW_003026614.1	-	49922	49874
Sma-1-22052	NW_003038514.1	-	153112	153064
Sma-1-22053	NW_003026607.1	-	106579	106531
Sma-1-22054	NW_003038009.1	-	69031	68983
Sma-1-22055	NW_003037795.1	+	1033271	1033319
Sma-1-22056	NW_003037796.1	-	1463422	1463374

Sma-1-22057	NW_003037797.1	+	539612	539660
Sma-1-22058	NW_003037806.1	+	904251	904299
Sma-1-22059	NW_003037821.1	-	877383	877335
Sma-1-22060	NW_003037823.1	+	932686	932734
Sma-1-22061	NW_003037891.1	-	2768752	2768704
Sma-1-22062	NW_003037907.1	-	75404	75356
Sma-1-22063	NS_000200.1	+	7424154	7424202
Sma-1-22064	NS_000200.1	+	10967472	10967520
Sma-1-22065	NS_000200.1	+	29099465	29099513
Sma-1-22066	NS_000200.1	+	56849305	56849353
Sma-1-22067	NS_000200.1	+	361409437	361409485
Sma-1-22068	NS_000200.1	-	355827014	355826966
Sma-1-22069	NS_000200.1	-	339777651	339777603
Sma-1-22070	NS_000200.1	-	315946653	315946605
Sma-1-22071	NS_000200.1	-	308138964	308138916
Sma-1-22072	NS_000200.1	-	226443094	226443046
Sma-1-22073	NS_000200.1	-	151479893	151479845
Sma-1-22074	NS_000200.1	-	140650030	140649982
Sma-1-22075	NS_000200.1	-	54021867	54021819
Sma-1-22076	NS_000200.1	-	9673066	9673018
Sma-1-22077	NW_003025837.1	-	904	856
Sma-1-22078	NW_003037794.1	-	1961893	1961845
Sma-1-22079	NW_003037804.1	-	530551	530503
Sma-1-22080	NW_003037981.1	-	1147508	1147460
Sma-1-22081	NW_003038038.1	+	825977	826025
Sma-1-22082	NS_000200.1	+	252729370	252729418
Sma-1-22083	NS_000200.1	-	356613835	356613787
Sma-1-22084	NS_000200.1	-	213545376	213545328
Sma-1-22085	NS_000200.1	-	25670571	25670523
Sma-1-22086	NS_000200.1	-	6141488	6141440
Sma-1-22087	NW_003026465.1	+	362861	362909
Sma-1-22088	NS_000200.1	+	207755455	207755503
Sma-1-22089	NW_003037913.1	+	567628	567676
Sma-1-22090	NS_000200.1	+	159131379	159131427
Sma-1-22091	NW_003037952.1	-	30307	30259
Sma-1-22092	NW_003037866.1	-	441130	441082
Sma-1-22093	NW_003037972.1	-	413633	413585
Sma-1-22094	NW_003037975.1	-	752253	752205
Sma-1-22095	NS_000200.1	-	206334477	206334429
Sma-1-22096	NS_000200.1	-	202889337	202889289
Sma-1-22097	NS_000200.1	-	192850440	192850392
Sma-1-22098	NS_000200.1	-	111928929	111928881
Sma-1-22099	NW_003037980.1	+	249044	249092
Sma-1-22100	NS_000200.1	+	211209906	211209954
Sma-1-22101	NW_003037831.1	+	685594	685642
Sma-1-22102	NS_000200.1	+	70134896	70134944
Sma-1-22103	NW_003026464.1	+	71244	71292
Sma-1-22104	NW_003026577.1	+	26261	26309
Sma-1-22105	NS_000200.1	+	203371553	203371601
Sma-1-22106	NS_000200.1	+	302697517	302697565
Sma-1-22107	NW_003037833.1	+	904118	904166
Sma-1-22108	NS_000200.1	+	73095214	73095262

Sma-1-22109	NW_003037982.1	+	583713	583761
Sma-1-22110	NS_000200.1	+	214242908	214242956
Sma-1-22111	NW_003037898.1	-	207320	207272
Sma-1-22112	NS_000200.1	-	144191142	144191094
Sma-1-22113	NW_003038250.1	+	272760	272808
Sma-1-22114	NS_000200.1	+	318325437	318325485
Sma-1-22115	NW_003037823.1	+	879059	879107
Sma-1-22116	NW_003037935.1	-	532835	532787
Sma-1-22117	NW_003037946.1	+	815155	815203
Sma-1-22118	NW_003038261.1	-	306381	306333
Sma-1-22119	NS_000200.1	+	56795678	56795726
Sma-1-22120	NS_000200.1	+	191509015	191509063
Sma-1-22121	NS_000200.1	-	323920032	323919984
Sma-1-22122	NS_000200.1	-	179949370	179949322
Sma-1-22123	NW_003024576.1	+	244	292
Sma-1-22124	NW_003037839.1	+	635109	635157
Sma-1-22125	NW_003037893.1	+	402131	402179
Sma-1-22126	NW_003038012.1	-	295364	295316
Sma-1-22127	NS_000200.1	+	79593259	79593307
Sma-1-22128	NS_000200.1	+	141516441	141516489
Sma-1-22129	NS_000200.1	+	348885373	348885421
Sma-1-22130	NS_000200.1	-	228619303	228619255
Sma-1-22131	NW_003037854.1	-	1238982	1238934
Sma-1-22132	NW_003037953.1	+	87586	87634
Sma-1-22133	NW_003038035.1	+	809017	809065
Sma-1-22134	NS_000200.1	+	193000637	193000685
Sma-1-22135	NS_000200.1	+	248670509	248670557
Sma-1-22136	NS_000200.1	-	98143403	98143355
Sma-1-22137	NW_003037887.1	-	272783	272735
Sma-1-22138	NS_000200.1	-	130736761	130736713
Sma-1-22139	NW_003037504.1	+	2012	2060
Sma-1-22140	NW_003038443.1	+	13475	13523
Sma-1-22141	NW_003038216.1	+	50005	50053
Sma-1-22142	NW_003037793.1	+	2307370	2307418
Sma-1-22143	NW_003037795.1	-	627979	627931
Sma-1-22144	NW_003037814.1	+	207009	207057
Sma-1-22145	NW_003037852.1	-	93918	93870
Sma-1-22146	NW_003037877.1	+	177872	177920
Sma-1-22147	NW_003037884.1	-	1110269	1110221
Sma-1-22148	NW_003037885.1	+	483721	483769
Sma-1-22149	NW_003037916.1	+	470995	471043
Sma-1-22150	NW_003037926.1	+	789351	789399
Sma-1-22151	NW_003037976.1	+	421281	421329
Sma-1-22152	NW_003037978.1	-	39644	39596
Sma-1-22153	NW_003037981.1	+	903270	903318
Sma-1-22154	NW_003038038.1	+	384471	384519
Sma-1-22155	NW_003038257.1	-	354514	354466
Sma-1-22156	NW_003038161.1	+	262645	262693
Sma-1-22157	NS_000200.1	+	2307370	2307418
Sma-1-22158	NS_000200.1	+	43190869	43190917
Sma-1-22159	NS_000200.1	+	121216434	121216482
Sma-1-22160	NS_000200.1	+	128581668	128581716

Sma-1-22161	NS_000200.1	+	163664406	163664454
Sma-1-22162	NS_000200.1	+	173957621	173957669
Sma-1-22163	NS_000200.1	+	207035596	207035644
Sma-1-22164	NS_000200.1	+	213301138	213301186
Sma-1-22165	NS_000200.1	+	252287864	252287912
Sma-1-22166	NS_000200.1	+	297632496	297632544
Sma-1-22167	NS_000200.1	+	306835637	306835685
Sma-1-22168	NS_000200.1	+	330043121	330043169
Sma-1-22169	NS_000200.1	+	370603327	370603375
Sma-1-22170	NS_000200.1	-	321528842	321528794
Sma-1-22171	NS_000200.1	-	209307654	209307606
Sma-1-22172	NS_000200.1	-	127889934	127889886
Sma-1-22173	NS_000200.1	-	94485813	94485765
Sma-1-22174	NS_000200.1	-	7018862	7018814
Sma-1-22175	NW_003038006.1	+	295110	295158
Sma-1-22176	NS_000200.1	+	225179594	225179642
Sma-1-22177	NW_003022118.1	+	209	257
Sma-1-22178	NW_003036940.1	-	3308	3260
Sma-1-22179	NS_000200.1	+	360742241	360742289
Sma-1-22180	NS_000200.1	-	343512582	343512534
Sma-1-22181	NW_003038008.1	-	575134	575086
Sma-1-22182	NS_000200.1	-	226248230	226248182
Sma-1-22183	NW_003037886.1	-	749654	749606
Sma-1-22184	NS_000200.1	-	130005221	130005173
Sma-1-22185	NW_003038014.1	+	37315	37363
Sma-1-22186	NS_000200.1	+	229778004	229778052
Sma-1-22187	NW_003037884.1	+	101499	101547
Sma-1-22188	NS_000200.1	+	126881164	126881212
Sma-1-22189	NW_003032361.1	+	855	903
Sma-1-22190	NW_003037838.1	-	580987	580939
Sma-1-22191	NW_003037966.1	-	256404	256356
Sma-1-22192	NS_000200.1	+	345951715	345951763
Sma-1-22193	NS_000200.1	-	198958267	198958219
Sma-1-22194	NS_000200.1	-	78579550	78579502
Sma-1-22195	NW_003037944.1	-	563542	563494
Sma-1-22196	NS_000200.1	-	188784895	188784847
Sma-1-22197	NW_003038055.1	+	216916	216964
Sma-1-22198	NS_000200.1	+	258916238	258916286
Sma-1-22199	NW_003037801.1	+	620247	620295
Sma-1-22200	NS_000200.1	+	21709727	21709775
Sma-1-22201	NW_003038019.1	+	577536	577585
Sma-1-22202	NS_000200.1	+	233894354	233894403
Sma-1-22203	NW_003038157.1	-	510950	510901
Sma-1-22204	NS_000200.1	-	295761870	295761821
Sma-1-22205	NW_003038117.1	+	136911	136960
Sma-1-22206	NS_000200.1	+	277454736	277454785
Sma-1-22207	NW_003026619.1	-	127477	127428
Sma-1-22208	NS_000200.1	-	322318221	322318172
Sma-1-22209	NW_003037886.1	+	106168	106217
Sma-1-22210	NS_000200.1	+	129361735	129361784
Sma-1-22211	NW_003037888.1	-	132476	132427
Sma-1-22212	NS_000200.1	-	132340968	132340919

Sma-1-22213	NW_003037794.1	+	306280	306328
Sma-1-22214	NS_000200.1	+	4485875	4485923
Sma-1-22215	NW_003037794.1	+	2098671	2098719
Sma-1-22216	NS_000200.1	+	6278266	6278314
Sma-1-22217	NW_003037793.1	+	3819600	3819648
Sma-1-22218	NS_000200.1	+	3819600	3819648
Sma-1-22219	NW_003037818.1	+	1455631	1455679
Sma-1-22220	NW_003037884.1	-	771168	771120
Sma-1-22221	NS_000200.1	+	50171038	50171086
Sma-1-22222	NS_000200.1	-	127550833	127550785
Sma-1-22223	NW_003035881.1	-	342496	342448
Sma-1-22224	NS_000200.1	-	341406701	341406653
Sma-1-22225	NW_003037910.1	-	980270	980222
Sma-1-22226	NS_000200.1	-	155492245	155492197
Sma-1-22227	NW_003032266.1	-	52829	52781
Sma-1-22228	NS_000200.1	-	358243576	358243528
Sma-1-22229	NW_003031291.1	-	263	215
Sma-1-22230	NW_003037807.1	+	790107	790155
Sma-1-22231	NS_000200.1	+	31051623	31051671
Sma-1-22232	NS_000200.1	-	345678998	345678950
Sma-1-22233	NW_003031132.1	+	2117	2165
Sma-1-22234	NS_000200.1	+	325808837	325808885
Sma-1-22235	NW_003037825.1	+	566552	566600
Sma-1-22236	NS_000200.1	+	59696334	59696382
Sma-1-22237	NW_003037975.1	-	186803	186754
Sma-1-22238	NS_000200.1	-	205769027	205768978
Sma-1-22239	NW_003037987.1	-	36935	36887
Sma-1-22240	NS_000200.1	-	215789556	215789508
Sma-1-22241	NW_003037991.1	-	391238	391190
Sma-1-22242	NS_000200.1	-	217540806	217540758
Sma-1-22243	NW_003037964.1	-	491571	491523
Sma-1-22244	NW_003038147.1	-	480166	480118
Sma-1-22245	NS_000200.1	-	290007909	290007861
Sma-1-22246	NS_000200.1	-	198093093	198093045
Sma-1-22247	NW_003037829.1	+	824088	824136
Sma-1-22248	NS_000200.1	+	66724626	66724674
Sma-1-22249	NW_003037894.1	+	462822	462870
Sma-1-22250	NS_000200.1	+	142359395	142359443
Sma-1-22251	NW_003037881.1	+	851623	851671
Sma-1-22252	NW_003037922.1	-	253087	253039
Sma-1-22253	NS_000200.1	+	125283414	125283462
Sma-1-22254	NS_000200.1	-	170177084	170177036
Sma-1-22255	NW_003039130.1	+	113655	113703
Sma-1-22256	NS_000200.1	+	317788266	317788314
Sma-1-22257	NW_003037888.1	-	1474582	1474534
Sma-1-22258	NS_000200.1	-	133683074	133683026
Sma-1-22259	NW_003037769.1	-	2736	2688
Sma-1-22260	NS_000200.1	-	378147688	378147640
Sma-1-22261	NW_003022453.1	+	729	777
Sma-1-22262	NS_000200.1	+	364970494	364970542
Sma-1-22263	NW_003037979.1	-	24052	24004
Sma-1-22264	NS_000200.1	-	210085072	210085024

Sma-1-22265	NW_003038019.1	-	521635	521587
Sma-1-22266	NS_000200.1	-	233838453	233838405
Sma-1-22267	NW_003038514.1	-	306813	306765
Sma-1-22268	NW_003038077.1	-	128903	128855
Sma-1-22269	NS_000200.1	-	339931352	339931304
Sma-1-22270	NS_000200.1	-	266900543	266900495
Sma-1-22271	NW_003037854.1	+	669102	669150
Sma-1-22272	NS_000200.1	+	97573523	97573571
Sma-1-22273	NW_003037854.1	-	675743	675695
Sma-1-22274	NS_000200.1	-	97580164	97580116
Sma-1-22275	NW_003038008.1	+	505729	505777
Sma-1-22276	NS_000200.1	+	226178825	226178873
Sma-1-22277	NW_003026472.1	+	92550	92598
Sma-1-22278	NS_000200.1	+	236504542	236504590
Sma-1-22279	NW_003037978.1	-	529614	529566
Sma-1-22280	NS_000200.1	-	209797624	209797576
Sma-1-22281	NW_003037854.1	+	3193	3241
Sma-1-22282	NW_003038158.1	-	316894	316846
Sma-1-22283	NS_000200.1	+	96907614	96907662
Sma-1-22284	NS_000200.1	-	296200062	296200014
Sma-1-22285	NW_003038218.1	-	230157	230109
Sma-1-22286	NS_000200.1	-	307670455	307670407
Sma-1-22287	NW_003038034.1	-	253850	253802
Sma-1-22288	NS_000200.1	-	247195606	247195558
Sma-1-22289	NW_003037804.1	-	400649	400601
Sma-1-22290	NW_003038072.1	+	324939	324987
Sma-1-22291	NW_003038056.1	-	1968	1920
Sma-1-22292	NW_003038124.1	-	44476	44428
Sma-1-22293	NS_000200.1	+	265487167	265487215
Sma-1-22294	NS_000200.1	-	279037120	279037072
Sma-1-22295	NS_000200.1	-	258979619	258979571
Sma-1-22296	NS_000200.1	-	25540669	25540621
Sma-1-22297	NW_003037813.1	+	1331823	1331871
Sma-1-22298	NS_000200.1	+	42318686	42318734
Sma-1-22299	NW_003037580.1	-	26220	26172
Sma-1-22300	NS_000200.1	-	372329434	372329386
Sma-1-22301	NW_003037826.1	+	1284241	1284289
Sma-1-22302	NS_000200.1	+	62283757	62283805
Sma-1-22303	NW_003038159.1	+	61962	62010
Sma-1-22304	NS_000200.1	+	296449983	296450031
Sma-1-22305	NW_003026456.1	+	508072	508120
Sma-1-22306	NW_003037877.1	+	290967	291015
Sma-1-22307	NS_000200.1	+	58431208	58431256
Sma-1-22308	NS_000200.1	+	121329529	121329577
Sma-1-22309	NW_003038151.1	-	209091	209043
Sma-1-22310	NS_000200.1	-	292116334	292116286
Sma-1-22311	NW_003037909.1	+	538922	538970
Sma-1-22312	NW_003038518.1	+	302142	302190
Sma-1-22313	NS_000200.1	+	154080571	154080619
Sma-1-22314	NS_000200.1	+	341938975	341939023
Sma-1-22315	NW_003038112.1	-	101434	101386
Sma-1-22316	NS_000200.1	-	276556612	276556564

Sma-1-22317	NW_003038276.1	+	9767	9815
Sma-1-22318	NS_000200.1	+	325170122	325170170
Sma-1-22319	NW_003038029.1	-	801492	801444
Sma-1-22320	NS_000200.1	-	243002393	243002345
Sma-1-22321	NW_003037942.1	-	389540	389492
Sma-1-22322	NS_000200.1	-	186149448	186149400
Sma-1-22323	NW_003038137.1	-	186507	186459
Sma-1-22324	NS_000200.1	-	284975403	284975355
Sma-1-22325	NW_003037879.1	-	473021	472973
Sma-1-22326	NS_000200.1	-	123475277	123475229
Sma-1-22327	NW_003033803.1	-	1291	1243
Sma-1-22328	NW_003026617.1	-	56645	56597
Sma-1-22329	NW_003037793.1	-	1364	1316
Sma-1-22330	NW_003037800.1	+	2200467	2200515
Sma-1-22331	NW_003037803.1	-	1140513	1140465
Sma-1-22332	NW_003037822.1	-	1196763	1196715
Sma-1-22333	NW_003037877.1	-	298181	298133
Sma-1-22334	NW_003037953.1	+	62507	62555
Sma-1-22335	NW_003038008.1	+	282343	282391
Sma-1-22336	NW_003038008.1	+	308484	308532
Sma-1-22337	NW_003038008.1	+	571313	571361
Sma-1-22338	NS_000200.1	+	20829827	20829875
Sma-1-22339	NS_000200.1	+	192975558	192975606
Sma-1-22340	NS_000200.1	+	225955439	225955487
Sma-1-22341	NS_000200.1	+	225981580	225981628
Sma-1-22342	NS_000200.1	+	226244409	226244457
Sma-1-22343	NS_000200.1	-	359035258	359035210
Sma-1-22344	NS_000200.1	-	319627504	319627456
Sma-1-22345	NS_000200.1	-	121336743	121336695
Sma-1-22346	NS_000200.1	-	55504769	55504721
Sma-1-22347	NS_000200.1	-	24641050	24641002
Sma-1-22348	NS_000200.1	-	1364	1316
Sma-1-22349	NW_003038033.1	+	796420	796468
Sma-1-22350	NS_000200.1	+	246776422	246776470
Sma-1-22351	NW_003037795.1	-	1599883	1599835
Sma-1-22352	NS_000200.1	-	7990766	7990718
Sma-1-22353	NW_003037797.1	-	755365	755317
Sma-1-22354	NS_000200.1	-	11183225	11183177
Sma-1-22355	NW_003037800.1	+	2422821	2422869
Sma-1-22356	NS_000200.1	+	21052181	21052229
Sma-1-22357	NW_003037800.1	+	579419	579468
Sma-1-22358	NS_000200.1	+	19208779	19208828
Sma-1-22359	NW_003037807.1	-	1297708	1297660
Sma-1-22360	NS_000200.1	-	31559224	31559176
Sma-1-22361	NW_003037804.1	+	887836	887884
Sma-1-22362	NW_003037804.1	+	888369	888417
Sma-1-22363	NS_000200.1	+	26027856	26027904
Sma-1-22364	NS_000200.1	+	26028389	26028437
Sma-1-22365	NW_003037845.1	-	500939	500891
Sma-1-22366	NS_000200.1	-	87502842	87502794
Sma-1-22367	NW_003038022.1	-	562968	562920
Sma-1-22368	NS_000200.1	-	236243810	236243762

Sma-1-22369	NW_003032210.1	+	921	969
Sma-1-22370	NW_003038002.1	+	62592	62640
Sma-1-22371	NW_003037818.1	+	860530	860578
Sma-1-22372	NW_003037914.1	+	821291	821339
Sma-1-22373	NW_003037998.1	-	19185	19137
Sma-1-22374	NW_003038083.1	+	172916	172964
Sma-1-22375	NW_003038085.1	-	375375	375327
Sma-1-22376	NW_003038126.1	+	243757	243805
Sma-1-22377	NW_003038161.1	-	509556	509508
Sma-1-22378	NS_000200.1	+	49575937	49575985
Sma-1-22379	NS_000200.1	+	160965541	160965589
Sma-1-22380	NS_000200.1	+	223182801	223182849
Sma-1-22381	NS_000200.1	+	270416253	270416301
Sma-1-22382	NS_000200.1	+	280254169	280254217
Sma-1-22383	NS_000200.1	+	357915750	357915798
Sma-1-22384	NS_000200.1	-	297879407	297879359
Sma-1-22385	NS_000200.1	-	272065468	272065420
Sma-1-22386	NS_000200.1	-	221305781	221305733
Sma-1-22387	NW_003037918.1	-	528445	528397
Sma-1-22388	NS_000200.1	-	167095435	167095387
Sma-1-22389	NW_003037925.1	+	216126	216174
Sma-1-22390	NS_000200.1	+	172618129	172618177
Sma-1-22391	NW_003038137.1	+	185833	185881
Sma-1-22392	NS_000200.1	+	284974729	284974777
Sma-1-22393	NW_003038261.1	-	825630	825582
Sma-1-22394	NS_000200.1	-	324439281	324439233
Sma-1-22395	NW_003038507.1	-	115633	115585
Sma-1-22396	NS_000200.1	-	336784527	336784479
Sma-1-22397	NW_003038249.1	-	168436	168388
Sma-1-22398	NW_003037797.1	+	1266553	1266601
Sma-1-22399	NW_003037906.1	+	90724	90772
Sma-1-22400	NW_003038021.1	-	127193	127145
Sma-1-22401	NW_003038030.1	+	883615	883663
Sma-1-22402	NW_003038038.1	+	381901	381949
Sma-1-22403	NS_000200.1	+	11694413	11694461
Sma-1-22404	NS_000200.1	+	150398503	150398551
Sma-1-22405	NS_000200.1	+	244019482	244019530
Sma-1-22406	NS_000200.1	+	252285294	252285342
Sma-1-22407	NS_000200.1	-	316850689	316850641
Sma-1-22408	NS_000200.1	-	234955768	234955720
Sma-1-22409	NW_003037803.1	-	523614	523566
Sma-1-22410	NS_000200.1	-	24024151	24024103
Sma-1-22411	NW_003037921.1	+	40729	40777
Sma-1-22412	NS_000200.1	+	169570864	169570912
Sma-1-22413	NW_003035877.1	-	50709	50661
Sma-1-22414	NS_000200.1	-	337031243	337031195
Sma-1-22415	NW_003038163.1	+	314520	314568
Sma-1-22416	NS_000200.1	+	298714800	298714848
Sma-1-22417	NW_003037858.1	+	257157	257205
Sma-1-22418	NS_000200.1	+	100921433	100921481
Sma-1-22419	NW_003037874.1	+	206652	206700
Sma-1-22420	NW_003037793.1	-	3228728	3228680

Sma-1-22421	NW_003037824.1	-	907319	907271
Sma-1-22422	NW_003037878.1	-	189316	189268
Sma-1-22423	NS_000200.1	+	119631602	119631650
Sma-1-22424	NS_000200.1	-	122242794	122242746
Sma-1-22425	NS_000200.1	-	57838259	57838211
Sma-1-22426	NS_000200.1	-	3228728	3228680
Sma-1-22427	NW_003038155.1	-	390338	390290
Sma-1-22428	NS_000200.1	-	294513456	294513408
Sma-1-22429	NW_003037579.1	+	11526	11574
Sma-1-22430	NS_000200.1	+	372296468	372296516
Sma-1-22431	NW_003039127.1	-	299313	299265
Sma-1-22432	NS_000200.1	-	113376812	113376764
Sma-1-22433	NW_003037976.1	+	369975	370023
Sma-1-22434	NS_000200.1	+	206984290	206984338
Sma-1-22435	NW_003037916.1	-	94037	93989
Sma-1-22436	NW_003038169.1	-	45570	45522
Sma-1-22437	NS_000200.1	-	300528738	300528690
Sma-1-22438	NS_000200.1	-	163287448	163287400
Sma-1-22439	NW_003037925.1	+	12911	12959
Sma-1-22440	NS_000200.1	+	172414914	172414962
Sma-1-22441	NW_003038018.1	+	345318	345366
Sma-1-22442	NS_000200.1	+	232746168	232746216
Sma-1-22443	NW_003036431.1	+	28	76
Sma-1-22444	NS_000200.1	+	359973889	359973937
Sma-1-22445	NW_003038216.1	-	49772	49724
Sma-1-22446	NS_000200.1	-	306835404	306835356
Sma-1-22447	NW_003026471.1	+	9966	10014
Sma-1-22448	NS_000200.1	+	222122199	222122247
Sma-1-22449	NW_003026465.1	-	207715	207667
Sma-1-22450	NS_000200.1	-	207600309	207600261
Sma-1-22451	NW_003038042.1	+	167356	167404
Sma-1-22452	NS_000200.1	+	254112955	254113003
Sma-1-22453	NW_003037952.1	+	66505	66553
Sma-1-22454	NS_000200.1	+	192886638	192886686
Sma-1-22455	NW_003035602.1	+	7470	7518
Sma-1-22456	NS_000200.1	+	329346664	329346712
Sma-1-22457	NW_003037916.1	+	844200	844248
Sma-1-22458	NS_000200.1	+	164037611	164037659
Sma-1-22459	NW_003037797.1	-	3524701	3524653
Sma-1-22460	NS_000200.1	-	13952561	13952513
Sma-1-22461	NW_003037812.1	-	1062916	1062868
Sma-1-22462	NS_000200.1	-	40545655	40545607
Sma-1-22463	NW_003037863.1	-	233469	233421
Sma-1-22464	NS_000200.1	-	106991836	106991788
Sma-1-22465	NW_003037878.1	-	521369	521321
Sma-1-22466	NS_000200.1	-	122574847	122574799
Sma-1-22467	NW_003038161.1	+	337517	337565
Sma-1-22468	NS_000200.1	+	297707368	297707416
Sma-1-22469	NW_003037809.1	+	2185687	2185735
Sma-1-22470	NW_003037866.1	+	1319546	1319594
Sma-1-22471	NS_000200.1	+	35850151	35850199
Sma-1-22472	NS_000200.1	+	112807345	112807393

Sma-1-22473	NW_003038028.1	-	604885	604837
Sma-1-22474	NS_000200.1	-	241912459	241912411
Sma-1-22475	NW_003037812.1	-	1101050	1101002
Sma-1-22476	NS_000200.1	-	40583789	40583741
Sma-1-22477	NW_003037794.1	+	1815391	1815439
Sma-1-22478	NS_000200.1	+	5994986	5995034
Sma-1-22479	NW_003034696.1	-	688	640
Sma-1-22480	NW_003037817.1	-	884665	884617
Sma-1-22481	NS_000200.1	-	328209177	328209129
Sma-1-22482	NS_000200.1	-	48267017	48266969
Sma-1-22483	NW_003037814.1	-	1861395	1861347
Sma-1-22484	NW_003037976.1	+	541744	541792
Sma-1-22485	NS_000200.1	+	207156059	207156107
Sma-1-22486	NS_000200.1	-	44845255	44845207
Sma-1-22487	NW_003030440.1	+	36	84
Sma-1-22488	NW_003037827.1	+	2300732	2300780
Sma-1-22489	NS_000200.1	+	64922873	64922921
Sma-1-22490	NS_000200.1	+	362904643	362904691
Sma-1-22491	NW_003037916.1	-	299176	299128
Sma-1-22492	NW_003038015.1	-	781686	781638
Sma-1-22493	NS_000200.1	-	231105176	231105128
Sma-1-22494	NS_000200.1	-	163492587	163492539
Sma-1-22495	NW_003038200.1	+	5954	6002
Sma-1-22496	NS_000200.1	+	303605248	303605296
Sma-1-22497	NW_003037831.1	+	1198542	1198590
Sma-1-22498	NS_000200.1	+	70647844	70647892
Sma-1-22499	NW_003026512.1	-	104365	104317
Sma-1-22500	NS_000200.1	-	285198405	285198357
Sma-1-22501	NW_003037911.1	+	126091	126139
Sma-1-22502	NS_000200.1	+	155913444	155913492
Sma-1-22503	NW_003037852.1	-	182834	182786
Sma-1-22504	NS_000200.1	-	94574729	94574681
Sma-1-22505	NW_003038228.1	-	124542	124492
Sma-1-22506	NS_000200.1	-	310997822	310997772
Sma-1-22507	NW_003038261.1	+	207139	207187
Sma-1-22508	NS_000200.1	+	323820790	323820838
Sma-1-22509	NW_003038188.1	-	72965	72917
Sma-1-22510	NS_000200.1	-	302166708	302166660
Sma-1-22511	NW_003026517.1	-	670095	670047
Sma-1-22512	NS_000200.1	-	300276104	300276056
Sma-1-22513	NW_003036415.1	+	58	106
Sma-1-22514	NW_003037797.1	+	2660178	2660226
Sma-1-22515	NS_000200.1	+	13088038	13088086
Sma-1-22516	NS_000200.1	+	359923883	359923931
Sma-1-22517	NW_003037814.1	-	774978	774930
Sma-1-22518	NW_003037850.1	+	425920	425968
Sma-1-22519	NW_003037902.1	-	325427	325379
Sma-1-22520	NS_000200.1	+	92411950	92411998
Sma-1-22521	NS_000200.1	-	147080661	147080613
Sma-1-22522	NS_000200.1	-	43758838	43758790
Sma-1-22523	NW_003038261.1	-	191268	191220
Sma-1-22524	NS_000200.1	-	323804919	323804871

Sma-1-22525	NW_003037905.1	+	157697	157745
Sma-1-22526	NS_000200.1	+	149426709	149426757
Sma-1-22527	NW_003038078.1	+	37156	37204
Sma-1-22528	NS_000200.1	+	267243359	267243407
Sma-1-22529	NW_003038027.1	-	865943	865895
Sma-1-22530	NS_000200.1	-	241236657	241236609
Sma-1-22531	NW_003037791.1	-	479517	479469
Sma-1-22532	NS_000200.1	-	382184945	382184897
Sma-1-22533	NW_003037771.1	-	107918	107870
Sma-1-22534	NS_000200.1	-	378408824	378408776
Sma-1-22535	NW_003038145.1	-	134283	134235
Sma-1-22536	NS_000200.1	-	288745706	288745658
Sma-1-22537	NW_003026515.1	-	115595	115547
Sma-1-22538	NS_000200.1	-	290760006	290759958
Sma-1-22539	NW_003037830.1	+	1456202	1456250
Sma-1-22540	NS_000200.1	+	69346573	69346621
Sma-1-22541	NW_003034429.1	-	207	159
Sma-1-22542	NS_000200.1	-	346706778	346706730
Sma-1-22543	NW_003037831.1	-	1381245	1381197
Sma-1-22544	NS_000200.1	-	70830547	70830499
Sma-1-22545	NW_003037998.1	-	44765	44717
Sma-1-22546	NS_000200.1	-	221331361	221331313
Sma-1-22547	NW_003037847.1	+	989470	989518
Sma-1-22548	NS_000200.1	+	89641200	89641248
Sma-1-22549	NW_003029502.1	-	616	568
Sma-1-22550	NW_003038053.1	-	497788	497740
Sma-1-22551	NS_000200.1	-	362177382	362177334
Sma-1-22552	NS_000200.1	-	257857883	257857835
Sma-1-22553	NW_003037885.1	+	434704	434752
Sma-1-22554	NS_000200.1	+	128532651	128532699
Sma-1-22555	NW_003037878.1	-	411409	411361
Sma-1-22556	NW_003037900.1	+	126415	126463
Sma-1-22557	NS_000200.1	+	145255281	145255329
Sma-1-22558	NS_000200.1	-	122464887	122464839
Sma-1-22559	NW_003038503.1	-	53612	53564
Sma-1-22560	NS_000200.1	-	335579662	335579614
Sma-1-22561	NW_003035873.1	+	32637	32685
Sma-1-22562	NW_003037874.1	+	330350	330398
Sma-1-22563	NW_003037793.1	+	100213	100261
Sma-1-22564	NW_003037793.1	+	489546	489594
Sma-1-22565	NW_003037808.1	-	1621092	1621044
Sma-1-22566	NW_003037809.1	+	1402665	1402713
Sma-1-22567	NW_003037826.1	-	427793	427745
Sma-1-22568	NW_003037869.1	-	117549	117501
Sma-1-22569	NW_003037916.1	-	1023718	1023670
Sma-1-22570	NW_003037918.1	+	459149	459197
Sma-1-22571	NW_003037944.1	+	772467	772515
Sma-1-22572	NW_003037954.1	+	319	367
Sma-1-22573	NW_003038017.1	+	36631	36679
Sma-1-22574	NW_003038032.1	+	157883	157931
Sma-1-22575	NW_003038132.1	-	14061	14013
Sma-1-22576	NW_003038146.1	-	359176	359128

Sma-1-22577	NS_000200.1	+	100213	100261
Sma-1-22578	NS_000200.1	+	489546	489594
Sma-1-22579	NS_000200.1	+	35067129	35067177
Sma-1-22580	NS_000200.1	+	119755300	119755348
Sma-1-22581	NS_000200.1	+	167026139	167026187
Sma-1-22582	NS_000200.1	+	188993820	188993868
Sma-1-22583	NS_000200.1	+	193229941	193229989
Sma-1-22584	NS_000200.1	+	231802662	231802710
Sma-1-22585	NS_000200.1	+	245317043	245317091
Sma-1-22586	NS_000200.1	+	335793639	335793687
Sma-1-22587	NS_000200.1	-	289339846	289339798
Sma-1-22588	NS_000200.1	-	282434531	282434483
Sma-1-22589	NS_000200.1	-	164217129	164217081
Sma-1-22590	NS_000200.1	-	116589894	116589846
Sma-1-22591	NS_000200.1	-	61427309	61427261
Sma-1-22592	NS_000200.1	-	33407729	33407681
Sma-1-22593	NW_003038128.1	+	297371	297419
Sma-1-22594	NS_000200.1	+	281015659	281015707
Sma-1-22595	NW_003038083.1	-	234219	234171
Sma-1-22596	NS_000200.1	-	270477556	270477508
Sma-1-22597	NW_003034049.1	-	907	859
Sma-1-22598	NW_003038157.1	+	409357	409405
Sma-1-22599	NS_000200.1	+	295660277	295660325
Sma-1-22600	NS_000200.1	-	363608841	363608793
Sma-1-22601	NW_003037952.1	-	74284	74236
Sma-1-22602	NS_000200.1	-	192894417	192894369
Sma-1-22603	NW_003038518.1	-	69042	68994
Sma-1-22604	NS_000200.1	-	341705875	341705827
Sma-1-22605	NW_003039127.1	+	265664	265712
Sma-1-22606	NS_000200.1	+	113343163	113343211
Sma-1-22607	NW_003026516.1	+	313158	313206
Sma-1-22608	NS_000200.1	+	299421455	299421503
Sma-1-22609	NW_003038117.1	+	15631	15679
Sma-1-22610	NS_000200.1	+	277333456	277333504
Sma-1-22611	NW_003037943.1	-	819371	819323
Sma-1-22612	NS_000200.1	-	187840904	187840856
Sma-1-22613	NW_003037824.1	+	80116	80164
Sma-1-22614	NS_000200.1	+	57011056	57011104
Sma-1-22615	NW_003037906.1	+	670349	670397
Sma-1-22616	NS_000200.1	+	150978128	150978176
Sma-1-22617	NW_003038038.1	+	1228128	1228176
Sma-1-22618	NS_000200.1	+	253131521	253131569
Sma-1-22619	NW_003038246.1	-	130321	130273
Sma-1-22620	NS_000200.1	-	315771449	315771401
Sma-1-22621	NW_003037965.1	-	194748	194700
Sma-1-22622	NS_000200.1	-	198483833	198483785
Sma-1-22623	NW_003037908.1	-	58921	58873
Sma-1-22624	NS_000200.1	-	152532052	152532004
Sma-1-22625	NW_003038085.1	+	216668	216716
Sma-1-22626	NS_000200.1	+	271906761	271906809
Sma-1-22627	NW_003037792.1	-	12159	12111
Sma-1-22628	NS_000200.1	-	382449503	382449455

Sma-1-22629	NW_003037833.1	-	611715	611667
Sma-1-22630	NS_000200.1	-	72802811	72802763
Sma-1-22631	NW_003037836.1	-	752206	752158
Sma-1-22632	NS_000200.1	-	76309305	76309257
Sma-1-22633	NW_003039127.1	-	971674	971626
Sma-1-22634	NS_000200.1	-	114049173	114049125
Sma-1-22635	NW_003038037.1	+	254683	254731
Sma-1-22636	NS_000200.1	+	250410092	250410140
Sma-1-22637	NW_003038156.1	+	257877	257925
Sma-1-22638	NS_000200.1	+	294989990	294990038
Sma-1-22639	NW_003037829.1	-	1007315	1007267
Sma-1-22640	NS_000200.1	-	66907853	66907805
Sma-1-22641	NW_003038512.1	-	315235	315187
Sma-1-22642	NS_000200.1	-	338871932	338871884
Sma-1-22643	NW_003037852.1	-	121128	121080
Sma-1-22644	NS_000200.1	-	94513023	94512975
Sma-1-22645	NW_003037804.1	-	488188	488140
Sma-1-22646	NS_000200.1	-	25628208	25628160
Sma-1-22647	NW_003038026.1	-	651649	651601
Sma-1-22648	NS_000200.1	-	240159410	240159362
Sma-1-22649	NW_003038008.1	-	176784	176736
Sma-1-22650	NS_000200.1	-	225849880	225849832
Sma-1-22651	NW_003037868.1	+	208687	208735
Sma-1-22652	NS_000200.1	+	116180399	116180447
Sma-1-22653	NW_003037964.1	+	191242	191290
Sma-1-22654	NW_003037964.1	+	588381	588429
Sma-1-22655	NS_000200.1	+	197792764	197792812
Sma-1-22656	NS_000200.1	+	198189903	198189951
Sma-1-22657	NW_003037890.1	-	1166318	1166270
Sma-1-22658	NS_000200.1	-	136849679	136849631
Sma-1-22659	NW_003038041.1	-	127229	127181
Sma-1-22660	NS_000200.1	-	253794511	253794463
Sma-1-22661	NW_003037893.1	+	141799	141847
Sma-1-22662	NS_000200.1	+	141256109	141256157
Sma-1-22663	NW_003038161.1	-	47731	47683
Sma-1-22664	NS_000200.1	-	297417582	297417534
Sma-1-22665	NW_003037994.1	-	245487	245439
Sma-1-22666	NS_000200.1	-	218777301	218777253
Sma-1-22667	NW_003037888.1	+	488423	488471
Sma-1-22668	NS_000200.1	+	132696915	132696963
Sma-1-22669	NW_003038015.1	-	766991	766943
Sma-1-22670	NW_003038124.1	-	136751	136703
Sma-1-22671	NS_000200.1	-	279129395	279129347
Sma-1-22672	NS_000200.1	-	231090481	231090433
Sma-1-22673	NW_003038023.1	-	65033	64985
Sma-1-22674	NS_000200.1	-	237225324	237225276
Sma-1-22675	NW_003037896.1	+	163891	163939
Sma-1-22676	NS_000200.1	+	143084314	143084362
Sma-1-22677	NW_003038026.1	+	102209	102257
Sma-1-22678	NS_000200.1	+	239609970	239610018
Sma-1-22679	NW_003021195.1	-	1802	1754
Sma-1-22680	NW_003028402.1	-	443	395

Sma-1-22681	NW_003030241.1	+	653	701
Sma-1-22682	NW_003037793.1	-	2135442	2135394
Sma-1-22683	NW_003037813.1	-	299057	299009
Sma-1-22684	NW_003037821.1	-	265365	265317
Sma-1-22685	NW_003037840.1	+	688259	688307
Sma-1-22686	NW_003037849.1	+	259103	259151
Sma-1-22687	NW_003037849.1	-	247426	247378
Sma-1-22688	NW_003037973.1	-	380681	380633
Sma-1-22689	NW_003037989.1	-	18475	18427
Sma-1-22690	NW_003026574.1	+	116006	116054
Sma-1-22691	NW_003038125.1	-	61518	61470
Sma-1-22692	NW_003038147.1	-	250496	250448
Sma-1-22693	NS_000200.1	+	81311583	81311631
Sma-1-22694	NS_000200.1	+	91235376	91235424
Sma-1-22695	NS_000200.1	+	301923339	301923387
Sma-1-22696	NS_000200.1	+	362455255	362455303
Sma-1-22697	NS_000200.1	-	371556844	371556796
Sma-1-22698	NS_000200.1	-	369529708	369529660
Sma-1-22699	NS_000200.1	-	289778239	289778191
Sma-1-22700	NS_000200.1	-	279498474	279498426
Sma-1-22701	NS_000200.1	-	216629034	216628986
Sma-1-22702	NS_000200.1	-	204362408	204362360
Sma-1-22703	NS_000200.1	-	91223699	91223651
Sma-1-22704	NS_000200.1	-	53409849	53409801
Sma-1-22705	NS_000200.1	-	41285920	41285872
Sma-1-22706	NS_000200.1	-	2135442	2135394
Sma-1-22707	NW_003037995.1	+	248028	248076
Sma-1-22708	NW_003026490.1	-	477206	477158
Sma-1-22709	NS_000200.1	+	219937796	219937844
Sma-1-22710	NS_000200.1	-	268108922	268108874
Sma-1-22711	NW_003037837.1	-	1120478	1120430
Sma-1-22712	NS_000200.1	-	77926336	77926288
Sma-1-22713	NW_003038131.1	+	155608	155656
Sma-1-22714	NS_000200.1	+	282117452	282117500
Sma-1-22715	NW_003037793.1	+	625520	625568
Sma-1-22716	NS_000200.1	+	625520	625568
Sma-1-22717	NW_003037864.1	+	1272626	1272674
Sma-1-22718	NS_000200.1	+	109765200	109765248
Sma-1-22719	NW_003038251.1	+	474806	474854
Sma-1-22720	NS_000200.1	+	318806578	318806626
Sma-1-22721	NW_003037864.1	-	111675	111627
Sma-1-22722	NS_000200.1	-	108604249	108604201
Sma-1-22723	NW_003037866.1	-	1399171	1399123
Sma-1-22724	NS_000200.1	-	112886970	112886922
Sma-1-22725	NW_003038261.1	-	172893	172845
Sma-1-22726	NS_000200.1	-	323786544	323786496
Sma-1-22727	NW_003037792.1	+	556009	556057
Sma-1-22728	NW_003037792.1	-	496663	496615
Sma-1-22729	NW_003037863.1	+	191186	191234
Sma-1-22730	NW_003037865.1	+	651089	651137
Sma-1-22731	NW_003037902.1	-	431743	431695
Sma-1-22732	NW_003037906.1	-	50357	50309

Sma-1-22733	NW_003037992.1	+	328636	328684
Sma-1-22734	NS_000200.1	+	106949553	106949601
Sma-1-22735	NS_000200.1	+	111073008	111073056
Sma-1-22736	NS_000200.1	+	218170117	218170165
Sma-1-22737	NS_000200.1	+	382993353	382993401
Sma-1-22738	NS_000200.1	-	382934007	382933959
Sma-1-22739	NS_000200.1	-	150358136	150358088
Sma-1-22740	NS_000200.1	-	147186977	147186929
Sma-1-22741	NW_003037849.1	-	446097	446049
Sma-1-22742	NW_003037888.1	+	948006	948054
Sma-1-22743	NS_000200.1	+	133156498	133156546
Sma-1-22744	NS_000200.1	-	91422370	91422322
Sma-1-22745	NW_003037891.1	+	1403204	1403252
Sma-1-22746	NS_000200.1	+	139284482	139284530
Sma-1-22747	NW_003037851.1	-	608644	608596
Sma-1-22748	NS_000200.1	-	94011954	94011906
Sma-1-22749	NW_003037886.1	+	977512	977560
Sma-1-22750	NS_000200.1	+	130233079	130233127
env-91	AB231864.1	+	69209	69257
Sma-1-22751	NW_003029812.1	-	2552	2504
Sma-1-22752	NS_000200.1	-	376057522	376057474
Sma-1-22753	NW_003037993.1	-	52818	52770
Sma-1-22754	NS_000200.1	-	218239521	218239473
Sma-1-22755	NW_003035866.1	+	61148	61196
Sma-1-22756	NW_003038149.1	-	46809	46761
Sma-1-22757	NS_000200.1	+	334154958	334155006
Sma-1-22758	NS_000200.1	-	291075422	291075374
Sma-1-22759	NW_003037866.1	-	947943	947895
Sma-1-22760	NS_000200.1	-	112435742	112435694
Sma-1-22761	NW_003039127.1	-	284162	284114
Sma-1-22762	NS_000200.1	-	113361661	113361613
Sma-1-22763	NW_003037870.1	+	508846	508894
Sma-1-22764	NS_000200.1	+	117382511	117382559
env-92	AB231864.1	+	9996	10044
Sma-1-22765	NW_003026618.1	+	376299	376347
Sma-1-22766	NW_003037792.1	+	91314	91362
Sma-1-22767	NW_003037801.1	-	737938	737890
Sma-1-22768	NW_003037819.1	-	296058	296010
Sma-1-22769	NW_003037913.1	+	477565	477613
Sma-1-22770	NW_003037913.1	+	1465479	1465527
Sma-1-22771	NW_003037917.1	+	459808	459856
Sma-1-22772	NW_003037980.1	+	159432	159480
Sma-1-22773	NW_003038007.1	-	289826	289778
Sma-1-22774	NW_003038017.1	+	623177	623225
Sma-1-22775	NW_003038018.1	-	164425	164377
Sma-1-22776	NW_003038150.1	+	292851	292899
Sma-1-22777	NS_000200.1	+	159041316	159041364
Sma-1-22778	NS_000200.1	+	160029230	160029278
Sma-1-22779	NS_000200.1	+	165496612	165496660
Sma-1-22780	NS_000200.1	+	211120294	211120342
Sma-1-22781	NS_000200.1	+	232389208	232389256
Sma-1-22782	NS_000200.1	+	291771589	291771637

Sma-1-22783	NS_000200.1	+	322131301	322131349
Sma-1-22784	NS_000200.1	+	382528658	382528706
Sma-1-22785	NS_000200.1	-	232565275	232565227
Sma-1-22786	NS_000200.1	-	225538381	225538333
Sma-1-22787	NS_000200.1	-	51384323	51384275
Sma-1-22788	NS_000200.1	-	21827418	21827370
Sma-1-22789	NW_003038039.1	-	42478	42430
Sma-1-22790	NS_000200.1	-	253480324	253480276
Sma-1-22791	NW_003038161.1	-	410985	410937
Sma-1-22792	NS_000200.1	-	297780836	297780788
Sma-1-22793	NW_003038026.1	+	24378	24426
Sma-1-22794	NS_000200.1	+	239532139	239532187
Sma-1-22795	NW_003023005.1	+	2224	2272
Sma-1-22796	NW_003037888.1	-	877656	877608
Sma-1-22797	NS_000200.1	+	372037887	372037935
Sma-1-22798	NS_000200.1	-	133086148	133086100
Sma-1-22799	NW_003026517.1	-	596551	596503
Sma-1-22800	NS_000200.1	-	300202560	300202512
Sma-1-22801	NW_003031097.1	+	1556	1604
Sma-1-22802	NS_000200.1	+	325689280	325689328
Sma-1-22803	NW_003037766.1	-	27537	27489
Sma-1-22804	NS_000200.1	-	377998935	377998887
Sma-1-22805	NW_003037809.1	+	2231619	2231667
Sma-1-22806	NS_000200.1	+	35896083	35896131
Sma-1-22807	NW_003037793.1	-	3588785	3588737
Sma-1-22808	NS_000200.1	-	3588785	3588737
Sma-1-22809	NW_003037351.1	-	1741	1693
Sma-1-22810	NS_000200.1	-	366838221	366838173
Sma-1-22811	NW_003037837.1	+	409813	409861
Sma-1-22812	NS_000200.1	+	77215671	77215719
Sma-1-22813	NW_003038020.1	-	421662	421614
Sma-1-22814	NS_000200.1	-	234389006	234388958
Sma-1-22815	NW_003038153.1	+	474101	474149
Sma-1-22816	NS_000200.1	+	293536150	293536198
Sma-1-22817	NW_003038537.1	-	1709	1661
Sma-1-22818	NW_003039130.1	-	128793	128745
Sma-1-22819	NW_003037798.1	-	2484975	2484927
Sma-1-22820	NW_003037803.1	+	853212	853260
Sma-1-22821	NW_003038067.1	+	98354	98402
Sma-1-22822	NS_000200.1	+	24353749	24353797
Sma-1-22823	NS_000200.1	+	262952407	262952455
Sma-1-22824	NS_000200.1	-	343335721	343335673
Sma-1-22825	NS_000200.1	-	317803404	317803356
Sma-1-22826	NS_000200.1	-	16744133	16744085
Sma-1-22827	NW_003038261.1	+	576554	576602
Sma-1-22828	NW_003038261.1	+	577436	577484
Sma-1-22829	NS_000200.1	+	324190205	324190253
Sma-1-22830	NS_000200.1	+	324191087	324191135
Sma-1-22831	NW_003038033.1	-	102966	102918
Sma-1-22832	NS_000200.1	-	246082968	246082920
Sma-1-22833	NW_003026510.1	+	343553	343601
Sma-1-22834	NS_000200.1	+	283783734	283783782

Sma-1-22835	NW_003037795.1	-	1458110	1458062
Sma-1-22836	NS_000200.1	-	7848993	7848945
Sma-1-22837	NW_003037813.1	+	626435	626483
Sma-1-22838	NS_000200.1	+	41613298	41613346
Sma-1-22839	NW_003037929.1	-	36668	36620
Sma-1-22840	NW_003037804.1	-	1279303	1279255
Sma-1-22841	NW_003037826.1	+	71958	72006
Sma-1-22842	NW_003038141.1	-	228310	228262
Sma-1-22843	NS_000200.1	+	61071474	61071522
Sma-1-22844	NS_000200.1	-	287118289	287118241
Sma-1-22845	NS_000200.1	-	175277446	175277398
Sma-1-22846	NS_000200.1	-	26419323	26419275
Sma-1-22847	NW_003038228.1	-	101434	101386
Sma-1-22848	NS_000200.1	-	310974714	310974666
Sma-1-22849	NW_003037939.1	+	431030	431078
Sma-1-22850	NW_003038033.1	-	378510	378462
Sma-1-22851	NS_000200.1	+	183300784	183300832
Sma-1-22852	NS_000200.1	-	246358512	246358464
Sma-1-22853	NW_003037799.1	+	392791	392839
Sma-1-22854	NS_000200.1	+	17383145	17383193
Sma-1-22855	NW_003037840.1	-	937992	937944
Sma-1-22856	NS_000200.1	-	81561316	81561268
Sma-1-22857	NW_003038502.1	-	179893	179845
Sma-1-22858	NS_000200.1	-	335178938	335178890
Sma-1-22859	NW_003037863.1	-	981886	981838
Sma-1-22860	NS_000200.1	-	107740253	107740205
Sma-1-22861	NW_003038035.1	+	400322	400370
Sma-1-22862	NS_000200.1	+	248261814	248261862
Sma-1-22863	NW_003037890.1	+	1953714	1953762
Sma-1-22864	NS_000200.1	+	137637075	137637123
Sma-1-22865	NW_003037796.1	-	2179592	2179544
Sma-1-22866	NS_000200.1	-	10389236	10389188
Sma-1-22867	NW_003038240.1	+	299236	299284
Sma-1-22868	NS_000200.1	+	314438167	314438215
Sma-1-22869	NW_003038228.1	+	118623	118671
Sma-1-22870	NS_000200.1	+	310991903	310991951
Sma-1-22871	NW_003037918.1	+	1985815	1985863
Sma-1-22872	NS_000200.1	+	168552805	168552853
Sma-1-22873	NW_003037806.1	-	1810352	1810304
Sma-1-22874	NS_000200.1	-	30005566	30005518
Sma-1-22875	NW_003037918.1	-	1345837	1345789
Sma-1-22876	NS_000200.1	-	167912827	167912779
Sma-1-22877	NW_003037807.1	-	1167700	1167652
Sma-1-22878	NS_000200.1	-	31429216	31429168
Sma-1-22879	NW_003037805.1	-	1534118	1534070
Sma-1-22880	NS_000200.1	-	28091962	28091914
Sma-1-22881	NW_003037806.1	-	306291	306243
Sma-1-22882	NS_000200.1	-	28501505	28501457
Sma-1-22883	NW_003037806.1	+	345541	345589
Sma-1-22884	NS_000200.1	+	28540755	28540803
Sma-1-22885	NW_003037918.1	-	1046439	1046391
Sma-1-22886	NS_000200.1	-	167613429	167613381

Sma-1-22887	NW_003037917.1	+	825666	825714
Sma-1-22888	NS_000200.1	+	165862470	165862518
Sma-1-22889	NW_003037805.1	-	134710	134662
Sma-1-22890	NS_000200.1	-	26692554	26692506
Sma-1-22891	NW_003037809.1	+	795020	795068
Sma-1-22892	NS_000200.1	+	34459484	34459532
Sma-1-22893	NW_003037809.1	-	787227	787179
Sma-1-22894	NS_000200.1	-	34451691	34451643
Sma-1-22895	NW_003037801.1	-	869914	869866
Sma-1-22896	NS_000200.1	-	21959394	21959346
Sma-1-22897	NW_003037932.1	-	161335	161287
Sma-1-22898	NS_000200.1	-	177694531	177694483
Sma-1-22899	NW_003037810.1	-	1251332	1251284
Sma-1-22900	NW_003037894.1	-	364007	363959
Sma-1-22901	NS_000200.1	-	142260580	142260532
Sma-1-22902	NS_000200.1	-	37855854	37855806
Sma-1-22903	NW_003037800.1	-	1564469	1564421
Sma-1-22904	NW_003038028.1	-	305798	305750
Sma-1-22905	NW_003038037.1	-	1675572	1675524
Sma-1-22906	NW_003038153.1	-	15869	15821
Sma-1-22907	NS_000200.1	-	293077918	293077870
Sma-1-22908	NS_000200.1	-	251830981	251830933
Sma-1-22909	NS_000200.1	-	241613372	241613324
Sma-1-22910	NS_000200.1	-	20193829	20193781
Sma-1-22911	NW_003037807.1	+	384950	384998
Sma-1-22912	NS_000200.1	+	30646466	30646514
Sma-1-22913	NW_003037810.1	-	680085	680037
Sma-1-22914	NS_000200.1	-	37284607	37284559
Sma-1-22915	NW_003037888.1	+	1464171	1464219
Sma-1-22916	NW_003037888.1	+	1466858	1466906
Sma-1-22917	NS_000200.1	+	133672663	133672711
Sma-1-22918	NS_000200.1	+	133675350	133675398
Sma-1-22919	NW_003038218.1	+	148168	148216
Sma-1-22920	NS_000200.1	+	307588466	307588514
Sma-1-22921	NW_003037823.1	-	133957	133909
Sma-1-22922	NS_000200.1	-	56050576	56050528
Sma-1-22923	NW_003037827.1	+	2290154	2290202
Sma-1-22924	NS_000200.1	+	64912295	64912343
Sma-1-22925	NW_003037946.1	-	860791	860743
Sma-1-22926	NW_003038501.1	+	679	727
Sma-1-22927	NS_000200.1	+	334794731	334794779
Sma-1-22928	NS_000200.1	-	191554651	191554603
Sma-1-22929	NW_003037804.1	-	1361460	1361412
Sma-1-22930	NS_000200.1	-	26501480	26501432
Sma-1-22931	NW_003038026.1	-	773921	773873
Sma-1-22932	NS_000200.1	-	240281682	240281634
Sma-1-22933	NW_003026517.1	+	593694	593742
Sma-1-22934	NS_000200.1	+	300199703	300199751
Sma-1-22935	NW_003037917.1	-	264767	264719
Sma-1-22936	NS_000200.1	-	165301571	165301523
Sma-1-22937	NW_003037801.1	+	882099	882147
Sma-1-22938	NS_000200.1	+	21971579	21971627

Sma-1-22939	NW_003037925.1	+	331366	331414
Sma-1-22940	NS_000200.1	+	172733369	172733417
Sma-1-22941	NW_003037925.1	-	327615	327567
Sma-1-22942	NW_003037925.1	-	324143	324095
Sma-1-22943	NS_000200.1	-	172729618	172729570
Sma-1-22944	NS_000200.1	-	172726146	172726098
Sma-1-22945	NW_003038117.1	+	129716	129764
Sma-1-22946	NS_000200.1	+	277447541	277447589
Sma-1-22947	NW_003037953.1	+	155498	155546
Sma-1-22948	NS_000200.1	+	193068549	193068597
Sma-1-22949	NW_003038288.1	+	3073	3121
Sma-1-22950	NS_000200.1	+	325370375	325370423
Sma-1-22951	NW_003038033.1	+	892025	892073
Sma-1-22952	NS_000200.1	+	246872027	246872075
Sma-1-22953	NW_003037933.1	-	558928	558880
Sma-1-22954	NS_000200.1	-	178972100	178972052
Sma-1-22955	NW_003037838.1	+	274514	274562
Sma-1-22956	NS_000200.1	+	78273077	78273125
Sma-1-22957	NW_003038024.1	-	950569	950521
Sma-1-22958	NS_000200.1	-	238847598	238847550
Sma-1-22959	NW_003037807.1	+	983875	983920
Sma-1-22960	NS_000200.1	+	31245391	31245436
Sma-1-22961	NW_003037834.1	+	776446	776494
Sma-1-22962	NS_000200.1	+	73936932	73936980
Sma-1-22963	NW_003026456.1	-	572576	572528
Sma-1-22964	NS_000200.1	-	58495712	58495664
Sma-1-22965	NW_003039127.1	-	8335	8287
Sma-1-22966	NS_000200.1	-	113085834	113085786
Sma-1-22967	NW_003038255.1	+	273782	273830
Sma-1-22968	NS_000200.1	+	320662927	320662975
Sma-1-22969	NW_003037874.1	-	303112	303064
Sma-1-22970	NS_000200.1	-	119728062	119728014
Sma-1-22971	NW_003035241.1	-	224	176
Sma-1-22972	NS_000200.1	-	370865140	370865092
Sma-1-22973	NW_003037797.1	-	1906212	1906164
Sma-1-22974	NS_000200.1	-	12334072	12334024
Sma-1-22975	NW_003037796.1	+	153435	153483
Sma-1-22976	NS_000200.1	+	8363079	8363127
Sma-1-22977	NW_003037819.1	-	615824	615776
Sma-1-22978	NS_000200.1	-	51704089	51704041
Sma-1-22979	NW_003037821.1	+	464359	464407
Sma-1-22980	NS_000200.1	+	53608843	53608891
Sma-1-22981	NW_003037805.1	+	669095	669143
Sma-1-22982	NW_003037805.1	+	675311	675359
Sma-1-22983	NS_000200.1	+	27226939	27226987
Sma-1-22984	NS_000200.1	+	27233155	27233203
Sma-1-22985	NW_003037951.1	+	86655	86703
Sma-1-22986	NW_003037918.1	+	157287	157335
Sma-1-22987	NS_000200.1	+	166724277	166724325
Sma-1-22988	NS_000200.1	+	192764380	192764428
Sma-1-22989	NW_003026490.1	-	72396	72348
Sma-1-22990	NS_000200.1	-	267704112	267704064

Sma-1-22991	NW_003037838.1	+	672157	672205
Sma-1-22992	NS_000200.1	+	78670720	78670768
Sma-1-22993	NW_003038005.1	-	234425	234377
Sma-1-22994	NS_000200.1	-	224640379	224640331
Sma-1-22995	NW_003038014.1	+	292827	292875
Sma-1-22996	NS_000200.1	+	230033516	230033564
Sma-1-22997	NW_003022764.1	+	367	415
Sma-1-22998	NW_003037853.1	-	414647	414599
Sma-1-22999	NS_000200.1	+	368054518	368054566
Sma-1-23000	NS_000200.1	-	95906698	95906650
Sma-1-23001	NW_003037826.1	+	70433	70481
Sma-1-23002	NS_000200.1	+	61069949	61069997
Sma-1-23003	NW_003026611.1	+	161475	161523
Sma-1-23004	NS_000200.1	+	310731259	310731307
Sma-1-23005	NW_003037977.1	+	615454	615502
Sma-1-23006	NS_000200.1	+	208901474	208901522
Sma-1-23007	NW_003037834.1	-	405188	405140
Sma-1-23008	NW_003038086.1	-	254459	254411
Sma-1-23009	NS_000200.1	-	272503052	272503004
Sma-1-23010	NS_000200.1	-	73565674	73565626
Sma-1-23011	NW_003037891.1	+	1606362	1606410
Sma-1-23012	NS_000200.1	+	139487640	139487688
Sma-1-23013	NW_003037810.1	-	1479356	1479308
Sma-1-23014	NS_000200.1	-	38083878	38083830
Sma-1-23015	NW_003037861.1	-	66431	66383
Sma-1-23016	NW_003038041.1	+	54781	54829
Sma-1-23017	NS_000200.1	+	253722063	253722111
Sma-1-23018	NS_000200.1	-	104842885	104842837
Sma-1-23019	NW_003023171.1	-	1064	1016
Sma-1-23020	NW_003038043.1	+	48890	48938
Sma-1-23021	NS_000200.1	+	254358932	254358980
Sma-1-23022	NS_000200.1	-	368418992	368418944
Sma-1-23023	NW_003026466.1	+	107620	107668
Sma-1-23024	NS_000200.1	+	215374727	215374775
Sma-1-23025	NW_003037803.1	+	173463	173511
Sma-1-23026	NS_000200.1	+	23674000	23674048
Sma-1-23027	NW_003038257.1	-	434082	434034
Sma-1-23028	NW_003026486.1	+	203688	203736
Sma-1-23029	NS_000200.1	+	259824807	259824855
Sma-1-23030	NS_000200.1	-	321608410	321608362
Sma-1-23031	NW_003038011.1	+	26481	26529
Sma-1-23032	NW_003038029.1	-	88257	88209
Sma-1-23033	NS_000200.1	+	227554131	227554179
Sma-1-23034	NS_000200.1	-	242289158	242289110
Sma-1-23035	NW_003037801.1	+	619914	619962
Sma-1-23036	NS_000200.1	+	21709394	21709442
Sma-1-23037	NW_003037923.1	-	72618	72570
Sma-1-23038	NS_000200.1	-	171128831	171128783
Sma-1-23039	NW_003037800.1	+	1703903	1703951
Sma-1-23040	NS_000200.1	+	20333263	20333311
Sma-1-23041	NW_003037924.1	-	541738	541690
Sma-1-23042	NS_000200.1	-	172248696	172248648

Sma-1-23043	NW_003037809.1	+	2041852	2041900
Sma-1-23044	NW_003037812.1	+	510693	510741
Sma-1-23045	NS_000200.1	+	35706316	35706364
Sma-1-23046	NS_000200.1	+	39993432	39993480
Sma-1-23047	NW_003037935.1	-	277420	277372
Sma-1-23048	NW_003037976.1	-	127113	127065
Sma-1-23049	NS_000200.1	-	206741428	206741380
Sma-1-23050	NS_000200.1	-	179693955	179693907
Sma-1-23051	NW_003037945.1	+	552722	552770
Sma-1-23052	NS_000200.1	+	189654686	189654734
Sma-1-23053	NW_003037805.1	-	195623	195575
Sma-1-23054	NW_003038106.1	-	47696	47648
Sma-1-23055	NS_000200.1	-	275236461	275236413
Sma-1-23056	NS_000200.1	-	26753467	26753419
Sma-1-23057	NW_003037799.1	+	702255	702303
Sma-1-23058	NS_000200.1	+	17692609	17692657
Sma-1-23059	NW_003037867.1	+	111942	111990
Sma-1-23060	NS_000200.1	+	115712761	115712809
Sma-1-23061	NW_003037932.1	+	9471	9519
Sma-1-23062	NW_003038037.1	+	1701661	1701709
Sma-1-23063	NS_000200.1	+	177542667	177542715
Sma-1-23064	NS_000200.1	+	251857070	251857118
Sma-1-23065	NW_003037797.1	+	405350	405398
Sma-1-23066	NS_000200.1	+	10833210	10833258
Sma-1-23067	NW_003035880.1	-	53355	53307
Sma-1-23068	NS_000200.1	-	339294419	339294371
Sma-1-23069	NW_003037971.1	-	241594	241546
Sma-1-23070	NS_000200.1	-	201863644	201863596
Sma-1-23071	NW_003037862.1	-	55870	55822
Sma-1-23072	NW_003037909.1	-	19919	19871
Sma-1-23073	NW_003037981.1	+	573081	573129
Sma-1-23074	NS_000200.1	+	212970949	212970997
Sma-1-23075	NS_000200.1	-	153561568	153561520
Sma-1-23076	NS_000200.1	-	105707531	105707483
Sma-1-23077	NW_003038014.1	+	396522	396570
Sma-1-23078	NS_000200.1	+	230137211	230137259
Sma-1-23079	NW_003037797.1	-	304049	304001
Sma-1-23080	NS_000200.1	-	10731909	10731861
Sma-1-23081	NW_003037932.1	+	33218	33266
Sma-1-23082	NS_000200.1	+	177566414	177566462
Sma-1-23083	NW_003037797.1	-	281983	281935
Sma-1-23084	NS_000200.1	-	10709843	10709795
Sma-1-23085	NW_003037827.1	-	239854	239806
Sma-1-23086	NS_000200.1	-	62861995	62861947
Sma-1-23087	NW_003026510.1	-	33670	33622
Sma-1-23088	NS_000200.1	-	283473851	283473803
Sma-1-23089	NW_003037856.1	+	359174	359222
Sma-1-23090	NW_003037909.1	+	218412	218460
Sma-1-23091	NW_003037916.1	+	1794055	1794103
Sma-1-23092	NW_003037944.1	+	793980	794028
Sma-1-23093	NS_000200.1	+	99717130	99717178
Sma-1-23094	NS_000200.1	+	153760061	153760109

Sma-1-23095	NS_000200.1	+	164987466	164987514
Sma-1-23096	NS_000200.1	+	189015333	189015381
Sma-1-23097	NW_003038063.1	-	207178	207130
Sma-1-23098	NS_000200.1	-	261703456	261703408
Sma-1-23099	NW_003026472.1	+	207396	207444
Sma-1-23100	NS_000200.1	+	236619388	236619436
Sma-1-23101	NW_003037798.1	+	1231517	1231565
Sma-1-23102	NW_003037798.1	+	1240591	1240639
Sma-1-23103	NS_000200.1	+	15490675	15490723
Sma-1-23104	NS_000200.1	+	15499749	15499797
Sma-1-23105	NW_003037796.1	-	474957	474909
Sma-1-23106	NS_000200.1	-	8684601	8684553
Sma-1-23107	NW_003037811.1	-	1295659	1295611
Sma-1-23108	NW_003038025.1	-	1455	1407
Sma-1-23109	NS_000200.1	-	238859700	238859652
Sma-1-23110	NS_000200.1	-	39465901	39465853
Sma-1-23111	NW_003037817.1	-	450895	450847
Sma-1-23112	NW_003038025.1	+	167099	167147
Sma-1-23113	NS_000200.1	+	239025344	239025392
Sma-1-23114	NS_000200.1	-	47833247	47833199
Sma-1-23115	NW_003038163.1	-	93375	93327
Sma-1-23116	NS_000200.1	-	298493655	298493607
Sma-1-23117	NW_003037791.1	+	152108	152156
Sma-1-23118	NW_003037797.1	-	1984083	1984035
Sma-1-23119	NS_000200.1	+	381857536	381857584
Sma-1-23120	NS_000200.1	-	12411943	12411895
Sma-1-23121	NW_003037873.1	-	516623	516575
Sma-1-23122	NS_000200.1	-	119255751	119255703
Sma-1-23123	NW_003026611.1	-	112830	112782
Sma-1-23124	NS_000200.1	-	310682614	310682566
Sma-1-23125	NW_003026463.1	-	20899	20851
Sma-1-23126	NS_000200.1	-	198636269	198636221
Sma-1-23127	NW_003037941.1	+	162954	163002
Sma-1-23128	NS_000200.1	+	184897428	184897476
Sma-1-23129	NW_003038037.1	-	1650776	1650728
Sma-1-23130	NS_000200.1	-	251806185	251806137
Sma-1-23131	NW_003037792.1	-	205475	205427
Sma-1-23132	NS_000200.1	-	382642819	382642771
Sma-1-23133	NW_003026602.1	-	26169	26121
Sma-1-23134	NS_000200.1	-	306946152	306946104
Sma-1-23135	NW_003037962.1	-	358406	358358
Sma-1-23136	NW_003037794.1	+	1384057	1384105
Sma-1-23137	NS_000200.1	+	5563652	5563700
Sma-1-23138	NS_000200.1	-	196435249	196435201
Sma-1-23139	NW_003037829.1	+	1136118	1136166
Sma-1-23140	NS_000200.1	+	67036656	67036704
Sma-1-23141	NW_003037944.1	+	710703	710751
Sma-1-23142	NS_000200.1	+	188932056	188932104
Sma-1-23143	NW_003037941.1	-	897921	897873
Sma-1-23144	NS_000200.1	-	185632395	185632347
Sma-1-23145	NW_003037233.1	-	2031	1983
Sma-1-23146	NW_003037917.1	+	824598	824646

Sma-1-23147	NS_000200.1	+	165861402	165861450
Sma-1-23148	NS_000200.1	-	363944376	363944328
Sma-1-23149	NW_003037824.1	+	946140	946188
Sma-1-23150	NS_000200.1	+	57877080	57877128
Sma-1-23151	NW_003038086.1	-	678435	678387
Sma-1-23152	NS_000200.1	-	272927028	272926980
Sma-1-23153	NW_003038137.1	-	274477	274429
Sma-1-23154	NS_000200.1	-	285063373	285063325
Sma-1-23155	NW_003037953.1	-	177297	177249
Sma-1-23156	NS_000200.1	-	193090348	193090300
Sma-1-23157	NW_003037904.1	-	294814	294766
Sma-1-23158	NS_000200.1	-	149009313	149009265
Sma-1-23159	NW_003037848.1	-	349209	349161
Sma-1-23160	NS_000200.1	-	90042280	90042232
Sma-1-23161	NW_003038021.1	+	314017	314065
Sma-1-23162	NS_000200.1	+	235142592	235142640
Sma-1-23163	NW_003037825.1	+	581207	581255
Sma-1-23164	NW_003037852.1	+	765125	765173
Sma-1-23165	NW_003037970.1	+	339057	339105
Sma-1-23166	NS_000200.1	+	59710989	59711037
Sma-1-23167	NS_000200.1	+	95157020	95157068
Sma-1-23168	NS_000200.1	+	201395247	201395295
Sma-1-23169	NW_003037890.1	+	837272	837320
Sma-1-23170	NS_000200.1	+	136520633	136520681
Sma-1-23171	NW_003038399.1	+	3512	3560
Sma-1-23172	NS_000200.1	+	328168233	328168281
Sma-1-23173	NW_003037879.1	-	274735	274687
Sma-1-23174	NS_000200.1	-	123276991	123276943
Sma-1-23175	NW_003037925.1	+	364728	364776
Sma-1-23176	NS_000200.1	+	172766731	172766779
Sma-1-23177	NW_003037925.1	-	373584	373536
Sma-1-23178	NS_000200.1	-	172775587	172775539
Sma-1-23179	NW_003037982.1	+	231697	231745
Sma-1-23180	NS_000200.1	+	213890892	213890940
Sma-1-23181	NW_003038232.1	-	304494	304446
Sma-1-23182	NS_000200.1	-	312268285	312268237
Sma-1-23183	NW_003020742.1	-	2293	2245
Sma-1-23184	NS_000200.1	-	364364880	364364832
Sma-1-23185	NW_003038071.1	+	343086	343134
Sma-1-23186	NS_000200.1	+	265146129	265146177
Sma-1-23187	NW_003020747.1	-	2401	2353
Sma-1-23188	NW_003031243.1	+	598	646
Sma-1-23189	NW_003026465.1	+	780898	780946
Sma-1-23190	NW_003038487.1	-	41836	41788
Sma-1-23191	NW_003037793.1	-	1679539	1679491
Sma-1-23192	NW_003037826.1	+	642742	642790
Sma-1-23193	NW_003037828.1	+	255084	255132
Sma-1-23194	NW_003037840.1	+	253132	253180
Sma-1-23195	NW_003037854.1	+	1783080	1783128
Sma-1-23196	NW_003037942.1	+	9332	9380
Sma-1-23197	NW_003037942.1	-	5245	5197
Sma-1-23198	NW_003037976.1	+	541411	541459

Sma-1-23199	NW_003038003.1	+	68807	68855
Sma-1-23200	NW_003039127.1	-	1412020	1411972
Sma-1-23201	NW_003038059.1	-	256326	256278
Sma-1-23202	NW_003038066.1	+	114577	114625
Sma-1-23203	NS_000200.1	+	61642258	61642306
Sma-1-23204	NS_000200.1	+	65344569	65344617
Sma-1-23205	NS_000200.1	+	80876456	80876504
Sma-1-23206	NS_000200.1	+	98687501	98687549
Sma-1-23207	NS_000200.1	+	185769240	185769288
Sma-1-23208	NS_000200.1	+	207155726	207155774
Sma-1-23209	NS_000200.1	+	208173492	208173540
Sma-1-23210	NS_000200.1	+	223568089	223568137
Sma-1-23211	NS_000200.1	+	262583521	262583569
Sma-1-23212	NS_000200.1	+	345595862	345595910
Sma-1-23213	NS_000200.1	-	364373568	364373520
Sma-1-23214	NS_000200.1	-	333497623	333497575
Sma-1-23215	NS_000200.1	-	260498398	260498350
Sma-1-23216	NS_000200.1	-	185765153	185765105
Sma-1-23217	NS_000200.1	-	114489519	114489471
Sma-1-23218	NS_000200.1	-	1679539	1679491
Sma-1-23219	NW_003037933.1	-	68869	68821
Sma-1-23220	NS_000200.1	-	178482041	178481993
Sma-1-23221	NW_003037796.1	-	1762508	1762460
Sma-1-23222	NS_000200.1	-	9972152	9972104
Sma-1-23223	NW_003037912.1	-	1485070	1485022
Sma-1-23224	NS_000200.1	-	158296645	158296597
Sma-1-23225	NW_003037864.1	+	1128736	1128784
Sma-1-23226	NS_000200.1	+	109621310	109621358
Sma-1-23227	NW_003037512.1	+	977	1025
Sma-1-23228	NS_000200.1	+	370745091	370745139
Sma-1-23229	NW_003038003.1	+	71277	71325
Sma-1-23230	NS_000200.1	+	223570559	223570607
Sma-1-23231	NW_003038261.1	-	1054691	1054643
Sma-1-23232	NS_000200.1	-	324668342	324668294
Sma-1-23233	NW_003037886.1	+	1019687	1019735
Sma-1-23234	NS_000200.1	+	130275254	130275302
Sma-1-23235	NW_003037969.1	+	130684	130732
Sma-1-23236	NS_000200.1	+	200736811	200736859
Sma-1-23237	NW_003038079.1	-	305965	305917
Sma-1-23238	NS_000200.1	-	268435953	268435905
Sma-1-23239	NW_003037836.1	+	885105	885153
Sma-1-23240	NS_000200.1	+	76442204	76442252
Sma-1-23241	NW_003038030.1	-	103693	103645
Sma-1-23242	NS_000200.1	-	243239560	243239512
Sma-1-23243	NW_003037798.1	+	2198035	2198083
Sma-1-23244	NW_003037914.1	+	1732100	1732148
Sma-1-23245	NS_000200.1	+	16457193	16457241
Sma-1-23246	NS_000200.1	+	161876350	161876398
Sma-1-23247	NW_003037915.1	-	1283989	1283941
Sma-1-23248	NS_000200.1	-	163183975	163183927
Sma-1-23249	NW_003037937.1	-	113351	113303
Sma-1-23250	NS_000200.1	-	181313260	181313212

Sma-1-23251	NW_003038154.1	+	73238	73286
Sma-1-23252	NS_000200.1	+	293870947	293870995
Sma-1-23253	NW_003026502.1	-	73002	72954
Sma-1-23254	NW_003039127.1	-	829871	829823
Sma-1-23255	NS_000200.1	-	276109438	276109390
Sma-1-23256	NS_000200.1	-	113907370	113907322
Sma-1-23257	NW_003037937.1	-	238958	238910
Sma-1-23258	NS_000200.1	-	181438867	181438819
Sma-1-23259	NW_003037793.1	+	3318175	3318223
Sma-1-23260	NS_000200.1	+	3318175	3318223
Sma-1-23261	NW_003037827.1	-	779879	779831
Sma-1-23262	NS_000200.1	-	63402020	63401972
Sma-1-23263	NW_003037767.1	-	6652	6604
Sma-1-23264	NW_003037846.1	+	227395	227443
Sma-1-23265	NW_003037926.1	-	492617	492569
Sma-1-23266	NS_000200.1	+	87861965	87862013
Sma-1-23267	NS_000200.1	-	378054532	378054484
Sma-1-23268	NS_000200.1	-	173660887	173660839
Sma-1-23269	NW_003037814.1	+	1468219	1468267
Sma-1-23270	NS_000200.1	+	44452079	44452127
Sma-1-23271	NW_003037910.1	-	101978	101930
Sma-1-23272	NS_000200.1	-	154613953	154613905
Sma-1-23273	NW_003037793.1	-	1465713	1465665
Sma-1-23274	NS_000200.1	-	1465713	1465665
Sma-1-23275	NW_003037814.1	-	1799217	1799169
Sma-1-23276	NS_000200.1	-	44783077	44783029
Sma-1-23277	NW_003026472.1	+	280019	280067
Sma-1-23278	NS_000200.1	+	236692011	236692059
Sma-1-23279	NW_003037884.1	+	1298266	1298314
Sma-1-23280	NS_000200.1	+	128077931	128077979
Sma-1-23281	NW_003037798.1	+	2469685	2469733
Sma-1-23282	NS_000200.1	+	16728843	16728891
Sma-1-23283	NW_003037994.1	+	480077	480125
Sma-1-23284	NS_000200.1	+	219011891	219011939
Sma-1-23285	NW_003038006.1	-	59801	59753
Sma-1-23286	NS_000200.1	-	224944285	224944237
Sma-1-23287	NW_003037913.1	-	56108	56060
Sma-1-23288	NS_000200.1	-	158619859	158619811
Sma-1-23289	NW_003037854.1	-	665930	665882
Sma-1-23290	NS_000200.1	-	97570351	97570303
Sma-1-23291	NW_003038034.1	+	503062	503110
Sma-1-23292	NS_000200.1	+	247444818	247444866
Sma-1-23293	NW_003037784.1	-	21410	21362
Sma-1-23294	NW_003038008.1	-	174957	174909
Sma-1-23295	NS_000200.1	-	379850277	379850229
Sma-1-23296	NS_000200.1	-	225848053	225848005
Sma-1-23297	NW_003039127.1	+	1905346	1905394
Sma-1-23298	NS_000200.1	+	114982845	114982893
Sma-1-23299	NW_003038482.1	+	43640	43688
Sma-1-23300	NS_000200.1	+	333021091	333021139
Sma-1-23301	NW_003038516.1	+	197442	197490
Sma-1-23302	NS_000200.1	+	340685847	340685895

Sma-1-23303	NW_003037792.1	-	438280	438232
Sma-1-23304	NS_000200.1	-	382875624	382875576
Sma-1-23305	NW_003037816.1	-	39575	39527
Sma-1-23306	NS_000200.1	-	46248447	46248399
Sma-1-23307	NW_003026458.1	-	424546	424498
Sma-1-23308	NS_000200.1	-	102292776	102292728
Sma-1-23309	NW_003026485.1	-	180169	180121
Sma-1-23310	NS_000200.1	-	258535074	258535026
Sma-1-23311	NW_003037816.1	+	639677	639725
Sma-1-23312	NW_003037816.1	+	640749	640797
Sma-1-23313	NS_000200.1	+	46848549	46848597
Sma-1-23314	NS_000200.1	+	46849621	46849669
Sma-1-23315	NW_003037816.1	-	830102	830054
Sma-1-23316	NS_000200.1	-	47038974	47038926
Sma-1-23317	NW_003037791.1	+	52241	52289
Sma-1-23318	NW_003038064.1	-	174264	174216
Sma-1-23319	NS_000200.1	+	381757669	381757717
Sma-1-23320	NS_000200.1	-	262010841	262010793
Sma-1-23321	NW_003037791.1	-	324027	323979
Sma-1-23322	NS_000200.1	-	382029455	382029407
Sma-1-23323	NW_003037791.1	+	270702	270750
Sma-1-23324	NS_000200.1	+	381976130	381976178
Sma-1-23325	NW_003037817.1	-	390635	390587
Sma-1-23326	NW_003037964.1	+	514576	514624
Sma-1-23327	NW_003026508.1	+	161424	161472
Sma-1-23328	NW_003038124.1	-	251106	251058
Sma-1-23329	NS_000200.1	+	198116098	198116146
Sma-1-23330	NS_000200.1	+	281206038	281206086
Sma-1-23331	NS_000200.1	-	279243750	279243702
Sma-1-23332	NS_000200.1	-	47772987	47772939
Sma-1-23333	NW_003035866.1	-	20287	20239
Sma-1-23334	NS_000200.1	-	334114097	334114049
Sma-1-23335	NW_003037796.1	-	1723749	1723701
Sma-1-23336	NS_000200.1	-	9933393	9933345
Sma-1-23337	NW_003037940.1	-	129493	129445
Sma-1-23338	NS_000200.1	-	183871623	183871575
Sma-1-23339	NW_003037940.1	-	470963	470915
Sma-1-23340	NS_000200.1	-	184213093	184213045
Sma-1-23341	NW_003037771.1	+	92828	92876
Sma-1-23342	NS_000200.1	+	378393734	378393782
Sma-1-23343	NW_003037818.1	-	1767567	1767519
Sma-1-23344	NS_000200.1	-	50482974	50482926
Sma-1-23345	NW_003037940.1	-	848513	848465
Sma-1-23346	NS_000200.1	-	184590643	184590595
Sma-1-23347	NW_003037818.1	+	2069814	2069862
Sma-1-23348	NS_000200.1	+	50785221	50785269
Sma-1-23349	NW_003037838.1	-	663772	663724
Sma-1-23350	NS_000200.1	-	78662335	78662287
Sma-1-23351	NW_003031752.1	+	606	654
Sma-1-23352	NW_003031752.1	+	960	1008
Sma-1-23353	NS_000200.1	+	366945569	366945617
Sma-1-23354	NS_000200.1	+	366945923	366945971

Sma-1-23355	NW_003035880.1	-	282339	282291
Sma-1-23356	NS_000200.1	-	339523403	339523355
Sma-1-23357	NW_003038063.1	-	106883	106835
Sma-1-23358	NS_000200.1	-	261603161	261603113
Sma-1-23359	NW_003037916.1	-	1833814	1833766
Sma-1-23360	NS_000200.1	-	165027225	165027177
Sma-1-23361	NW_003037976.1	-	385317	385269
Sma-1-23362	NS_000200.1	-	206999632	206999584
Sma-1-23363	NW_003037718.1	-	2600	2552
Sma-1-23364	NS_000200.1	-	376195208	376195160
Sma-1-23365	NW_003037942.1	-	357922	357874
Sma-1-23366	NS_000200.1	-	186117830	186117782
Sma-1-23367	NW_003037942.1	+	454007	454055
Sma-1-23368	NS_000200.1	+	186213915	186213963
Sma-1-23369	NW_003038512.1	-	327924	327876
Sma-1-23370	NS_000200.1	-	338884621	338884573
Sma-1-23371	NW_003037967.1	+	543384	543432
Sma-1-23372	NS_000200.1	+	199783355	199783403
Sma-1-23373	NW_003037962.1	-	181608	181560
Sma-1-23374	NW_003037883.1	+	306779	306827
Sma-1-23375	NS_000200.1	+	126370502	126370550
Sma-1-23376	NS_000200.1	-	196258451	196258403
Sma-1-23377	NW_003037796.1	+	1781190	1781238
Sma-1-23378	NS_000200.1	+	9990834	9990882
Sma-1-23379	NW_003035876.1	+	54407	54455
Sma-1-23380	NW_003037885.1	-	198773	198725
Sma-1-23381	NW_003037891.1	+	2572164	2572212
Sma-1-23382	NW_003037891.1	-	1479813	1479765
Sma-1-23383	NW_003037964.1	-	85976	85928
Sma-1-23384	NS_000200.1	+	140453442	140453490
Sma-1-23385	NS_000200.1	+	336885907	336885955
Sma-1-23386	NS_000200.1	-	197687498	197687450
Sma-1-23387	NS_000200.1	-	139361091	139361043
Sma-1-23388	NS_000200.1	-	128296720	128296672
Sma-1-23389	NW_003037819.1	-	701980	701932
Sma-1-23390	NW_003037922.1	+	133288	133336
Sma-1-23391	NS_000200.1	+	170057285	170057333
Sma-1-23392	NS_000200.1	-	51790245	51790197
Sma-1-23393	NW_003037819.1	-	980304	980256
Sma-1-23394	NS_000200.1	-	52068569	52068521
Sma-1-23395	NW_003037820.1	+	182808	182856
Sma-1-23396	NS_000200.1	+	52455790	52455838
Sma-1-23397	NW_003035154.1	-	1518	1470
Sma-1-23398	NW_003037995.1	+	303010	303058
Sma-1-23399	NS_000200.1	+	219992778	219992826
Sma-1-23400	NS_000200.1	-	370698688	370698640
Sma-1-23401	NW_003037948.1	-	98256	98208
Sma-1-23402	NW_003038063.1	-	241627	241579
Sma-1-23403	NS_000200.1	-	261737905	261737857
Sma-1-23404	NS_000200.1	-	192118200	192118152
Sma-1-23405	NW_003026474.1	+	90768	90816
Sma-1-23406	NS_000200.1	+	253289343	253289391

Sma-1-23407	NW_003026611.1	+	267644	267692
Sma-1-23408	NS_000200.1	+	310837428	310837476
Sma-1-23409	NW_003038518.1	+	559987	560035
Sma-1-23410	NS_000200.1	+	342196820	342196868
Sma-1-23411	NW_003037822.1	+	1031676	1031724
Sma-1-23412	NS_000200.1	+	55339682	55339730
Sma-1-23413	NW_003037824.1	-	856764	856716
Sma-1-23414	NS_000200.1	-	57787704	57787656
Sma-1-23415	NW_003037890.1	+	283007	283055
Sma-1-23416	NS_000200.1	+	135966368	135966416
Sma-1-23417	NW_003026722.1	-	16223	16175
Sma-1-23418	NS_000200.1	-	325427486	325427438
Sma-1-23419	NW_003038038.1	-	524142	524094
Sma-1-23420	NS_000200.1	-	252427535	252427487
Sma-1-23421	NW_003039127.1	-	369906	369858
Sma-1-23422	NS_000200.1	-	113447405	113447357
Sma-1-23423	NW_003032266.1	+	51362	51410
Sma-1-23424	NW_003037827.1	+	2328678	2328726
Sma-1-23425	NW_003037923.1	-	372889	372841
Sma-1-23426	NS_000200.1	+	64950819	64950867
Sma-1-23427	NS_000200.1	+	358242109	358242157
Sma-1-23428	NS_000200.1	-	171429102	171429054
Sma-1-23429	NW_003037822.1	-	374647	374599
Sma-1-23430	NS_000200.1	-	54682653	54682605
Sma-1-23431	NW_003037964.1	+	412008	412056
Sma-1-23432	NS_000200.1	+	198013530	198013578
Sma-1-23433	NW_003028808.1	+	1977	2025
Sma-1-23434	NS_000200.1	+	369977789	369977837
Sma-1-23435	NW_003037825.1	+	48129	48177
Sma-1-23436	NS_000200.1	+	59177911	59177959
Sma-1-23437	NW_003037798.1	+	1567789	1567837
Sma-1-23438	NW_003037909.1	-	427476	427428
Sma-1-23439	NW_003038149.1	-	80316	80268
Sma-1-23440	NS_000200.1	+	15826947	15826995
Sma-1-23441	NS_000200.1	-	291108929	291108881
Sma-1-23442	NS_000200.1	-	153969125	153969077
Sma-1-23443	NW_003037967.1	+	528560	528608
Sma-1-23444	NS_000200.1	+	199768531	199768579
Sma-1-23445	NW_003028269.1	-	307	259
Sma-1-23446	NS_000200.1	-	374433989	374433941
Sma-1-23447	NW_003022291.1	-	1478	1430
Sma-1-23448	NS_000200.1	-	361111834	361111786
Sma-1-23449	NW_003029166.1	+	850	898
Sma-1-23450	NW_003037819.1	+	1057639	1057687
Sma-1-23451	NW_003037997.1	-	456291	456243
Sma-1-23452	NS_000200.1	+	52145904	52145952
Sma-1-23453	NS_000200.1	+	357014642	357014690
Sma-1-23454	NS_000200.1	-	221267733	221267685
Sma-1-23455	NW_003037848.1	-	156027	155979
Sma-1-23456	NS_000200.1	-	89849098	89849050
Sma-1-23457	NW_003038025.1	-	403161	403113
Sma-1-23458	NS_000200.1	-	239261406	239261358

Sma-1-23459	NW_003038514.1	-	302712	302664
Sma-1-23460	NS_000200.1	-	339927251	339927203
Sma-1-23461	NW_003038510.1	-	19570	19522
Sma-1-23462	NS_000200.1	-	338301196	338301148
Sma-1-23463	NW_003023368.1	-	1919	1871
Sma-1-23464	NW_003037905.1	+	1009775	1009823
Sma-1-23465	NS_000200.1	+	150278787	150278835
Sma-1-23466	NS_000200.1	-	373150207	373150159
Sma-1-23467	NW_003038067.1	+	103592	103640
Sma-1-23468	NW_003038067.1	-	103251	103203
Sma-1-23469	NS_000200.1	+	262957645	262957693
Sma-1-23470	NS_000200.1	-	262957304	262957256
Sma-1-23471	NW_003037979.1	+	354214	354262
Sma-1-23472	NS_000200.1	+	210415234	210415282
Sma-1-23473	NW_003037937.1	-	756378	756330
Sma-1-23474	NS_000200.1	-	181956287	181956239
Sma-1-23475	NW_003026458.1	-	677854	677806
Sma-1-23476	NS_000200.1	-	102546084	102546036
Sma-1-23477	NW_003037980.1	+	924651	924699
Sma-1-23478	NS_000200.1	+	211885513	211885561
Sma-1-23479	NW_003037865.1	+	738073	738121
Sma-1-23480	NS_000200.1	+	111159992	111160040
Sma-1-23481	NW_003037808.1	+	682517	682565
Sma-1-23482	NS_000200.1	+	32469154	32469202
Sma-1-23483	NW_003037836.1	+	251976	252024
Sma-1-23484	NW_003037914.1	-	479005	478957
Sma-1-23485	NW_003038515.1	+	182022	182070
Sma-1-23486	NS_000200.1	+	75809075	75809123
Sma-1-23487	NS_000200.1	+	340266087	340266135
Sma-1-23488	NS_000200.1	-	160623255	160623207
Sma-1-23489	NW_003038073.1	-	339791	339743
Sma-1-23490	NW_003037890.1	+	1353189	1353237
Sma-1-23491	NS_000200.1	+	137036550	137036598
Sma-1-23492	NS_000200.1	-	265862224	265862176
Sma-1-23493	NW_003037867.1	+	366300	366348
Sma-1-23494	NW_003037913.1	-	1372147	1372099
Sma-1-23495	NS_000200.1	+	115967119	115967167
Sma-1-23496	NS_000200.1	-	159935898	159935850
Sma-1-23497	NW_003037882.1	-	611812	611764
Sma-1-23498	NS_000200.1	-	125903486	125903438
Sma-1-23499	NW_003037808.1	-	1550641	1550593
Sma-1-23500	NS_000200.1	-	33337278	33337230
Sma-1-23501	NW_003038150.1	-	291534	291486
Sma-1-23502	NS_000200.1	-	291770272	291770224
Sma-1-23503	NW_003038010.1	+	657237	657285
Sma-1-23504	NW_003038065.1	+	31360	31408
Sma-1-23505	NS_000200.1	+	227475460	227475508
Sma-1-23506	NS_000200.1	+	262182977	262183025
Sma-1-23507	NW_003037997.1	+	89219	89267
Sma-1-23508	NS_000200.1	+	220900661	220900709
Sma-1-23509	NW_003037841.1	+	460017	460065
Sma-1-23510	NW_003037864.1	-	695880	695832

Sma-1-23511	NS_000200.1	+	83071198	83071246
Sma-1-23512	NS_000200.1	-	109188454	109188406
Sma-1-23513	NW_003037847.1	+	1011363	1011411
Sma-1-23514	NS_000200.1	+	89663093	89663141
Sma-1-23515	NW_003037890.1	+	1603837	1603885
Sma-1-23516	NS_000200.1	+	137287198	137287246
Sma-1-23517	NW_003037909.1	+	903456	903504
Sma-1-23518	NS_000200.1	+	154445105	154445153
Sma-1-23519	NW_003038132.1	-	132753	132705
Sma-1-23520	NS_000200.1	-	282553223	282553175
Sma-1-23521	NW_003038034.1	-	388008	387960
Sma-1-23522	NS_000200.1	-	247329764	247329716
Sma-1-23523	NW_003030134.1	-	223	175
Sma-1-23524	NW_003038250.1	-	228356	228308
Sma-1-23525	NW_003026456.1	+	831657	831705
Sma-1-23526	NW_003037792.1	-	220096	220048
Sma-1-23527	NW_003037792.1	-	214048	214000
Sma-1-23528	NW_003037852.1	+	128721	128769
Sma-1-23529	NW_003037865.1	+	229118	229166
Sma-1-23530	NW_003037885.1	-	806291	806243
Sma-1-23531	NW_003037895.1	-	258952	258904
Sma-1-23532	NW_003037901.1	+	457595	457643
Sma-1-23533	NW_003037994.1	+	451059	451107
Sma-1-23534	NW_003038515.1	+	197358	197406
Sma-1-23535	NS_000200.1	+	58754793	58754841
Sma-1-23536	NS_000200.1	+	94520616	94520664
Sma-1-23537	NS_000200.1	+	110651037	110651085
Sma-1-23538	NS_000200.1	+	146306056	146306104
Sma-1-23539	NS_000200.1	+	218982873	218982921
Sma-1-23540	NS_000200.1	+	340281423	340281471
Sma-1-23541	NS_000200.1	-	382657440	382657392
Sma-1-23542	NS_000200.1	-	382651392	382651344
Sma-1-23543	NS_000200.1	-	357658742	357658694
Sma-1-23544	NS_000200.1	-	318281033	318280985
Sma-1-23545	NS_000200.1	-	142705154	142705106
Sma-1-23546	NS_000200.1	-	128904238	128904190
Sma-1-23547	NW_003037853.1	-	756583	756535
Sma-1-23548	NS_000200.1	-	96248634	96248586
Sma-1-23549	NW_003037855.1	+	343435	343483
Sma-1-23550	NW_003038495.1	-	15236	15188
Sma-1-23551	NS_000200.1	+	99208185	99208233
Sma-1-23552	NS_000200.1	-	334268456	334268408
Sma-1-23553	NW_003037795.1	-	1700508	1700460
Sma-1-23554	NW_003037803.1	+	1405568	1405616
Sma-1-23555	NW_003037848.1	+	764965	765013
Sma-1-23556	NW_003037864.1	-	46277	46229
Sma-1-23557	NW_003037918.1	+	934677	934725
Sma-1-23558	NW_003037918.1	-	934461	934413
Sma-1-23559	NW_003037979.1	+	879738	879786
Sma-1-23560	NW_003037979.1	+	880071	880119
Sma-1-23561	NW_003037979.1	-	879626	879578
Sma-1-23562	NW_003038030.1	+	133370	133418

Sma-1-23563	NW_003038241.1	-	160020	159972
Sma-1-23564	NW_003038259.1	+	72882	72930
Sma-1-23565	NW_003026517.1	+	247443	247491
Sma-1-23566	NW_003038193.1	+	13112	13160
Sma-1-23567	NS_000200.1	+	24906105	24906153
Sma-1-23568	NS_000200.1	+	90458036	90458084
Sma-1-23569	NS_000200.1	+	167501667	167501715
Sma-1-23570	NS_000200.1	+	210940758	210940806
Sma-1-23571	NS_000200.1	+	210941091	210941139
Sma-1-23572	NS_000200.1	+	243269237	243269285
Sma-1-23573	NS_000200.1	+	299853452	299853500
Sma-1-23574	NS_000200.1	+	302892992	302893040
Sma-1-23575	NS_000200.1	+	322933481	322933529
Sma-1-23576	NS_000200.1	-	314622725	314622677
Sma-1-23577	NS_000200.1	-	210940646	210940598
Sma-1-23578	NS_000200.1	-	167501451	167501403
Sma-1-23579	NS_000200.1	-	108538851	108538803
Sma-1-23580	NS_000200.1	-	8091391	8091343
Sma-1-23581	NW_003038012.1	-	366150	366102
Sma-1-23582	NW_003026508.1	+	53355	53403
Sma-1-23583	NS_000200.1	+	281097969	281098017
Sma-1-23584	NS_000200.1	-	228690089	228690041
Sma-1-23585	NW_003037975.1	+	66173	66221
Sma-1-23586	NW_003037975.1	+	66798	66846
Sma-1-23587	NS_000200.1	+	205648397	205648445
Sma-1-23588	NS_000200.1	+	205649022	205649070
Sma-1-23589	NW_003037829.1	+	127926	127974
Sma-1-23590	NW_003038012.1	-	536256	536208
Sma-1-23591	NS_000200.1	+	66028464	66028512
Sma-1-23592	NS_000200.1	-	228860195	228860147
Sma-1-23593	NW_003037906.1	+	517753	517801
Sma-1-23594	NS_000200.1	+	150825532	150825580
Sma-1-23595	NW_003037830.1	-	1549636	1549588
Sma-1-23596	NW_003037830.1	-	1549303	1549255
Sma-1-23597	NS_000200.1	-	69440007	69439959
Sma-1-23598	NS_000200.1	-	69439674	69439626
Sma-1-23599	NW_003026471.1	-	93312	93264
Sma-1-23600	NS_000200.1	-	222205545	222205497
Sma-1-23601	NW_003026606.1	-	161435	161387
Sma-1-23602	NS_000200.1	-	308017128	308017080
Sma-1-23603	NW_003037979.1	-	641250	641202
Sma-1-23604	NS_000200.1	-	210702270	210702222
Sma-1-23605	NW_003037981.1	+	1254106	1254154
Sma-1-23606	NS_000200.1	+	213651974	213652022
Sma-1-23607	NW_003038260.1	+	382583	382631
Sma-1-23608	NS_000200.1	+	323554358	323554406
Sma-1-23609	NW_003037803.1	-	1429839	1429791
Sma-1-23610	NS_000200.1	-	24930376	24930328
Sma-1-23611	NW_003025078.1	+	606	654
Sma-1-23612	NS_000200.1	+	356010410	356010458
Sma-1-23613	NW_003035876.1	+	111657	111705
Sma-1-23614	NS_000200.1	+	336943157	336943205

Sma-1-23615	NW_003037982.1	-	1059604	1059556
Sma-1-23616	NS_000200.1	-	214718799	214718751
Sma-1-23617	NW_003037841.1	+	1875678	1875726
Sma-1-23618	NS_000200.1	+	84486859	84486907
Sma-1-23619	NW_003037891.1	-	813340	813292
Sma-1-23620	NS_000200.1	-	138694618	138694570
Sma-1-23621	NW_003038150.1	-	164094	164046
Sma-1-23622	NS_000200.1	-	291642832	291642784
Sma-1-23623	NW_003037846.1	-	790807	790759
Sma-1-23624	NS_000200.1	-	88425377	88425329
Sma-1-23625	NW_003038013.1	-	55572	55524
Sma-1-23626	NS_000200.1	-	229445198	229445150
Sma-1-23627	NW_003038150.1	-	165825	165777
Sma-1-23628	NS_000200.1	-	291644563	291644515
Sma-1-23629	NW_003038102.1	+	28287	28335
Sma-1-23630	NS_000200.1	+	274707233	274707281
Sma-1-23631	NW_003038128.1	+	114491	114539
Sma-1-23632	NS_000200.1	+	280832779	280832827
Sma-1-23633	NW_003029647.1	-	3973	3925
Sma-1-23634	NW_003038002.1	-	331556	331508
Sma-1-23635	NW_003037810.1	+	145553	145601
Sma-1-23636	NW_003037857.1	-	601568	601520
Sma-1-23637	NW_003037899.1	+	219400	219448
Sma-1-23638	NW_003037899.1	-	503018	502970
Sma-1-23639	NW_003037914.1	+	723040	723088
Sma-1-23640	NW_003037926.1	-	206119	206071
Sma-1-23641	NW_003038003.1	+	195670	195718
Sma-1-23642	NW_003038501.1	+	144344	144392
Sma-1-23643	NW_003038154.1	+	283607	283655
Sma-1-23644	NS_000200.1	+	36750075	36750123
Sma-1-23645	NS_000200.1	+	144738511	144738559
Sma-1-23646	NS_000200.1	+	160867290	160867338
Sma-1-23647	NS_000200.1	+	223694952	223695000
Sma-1-23648	NS_000200.1	+	294081316	294081364
Sma-1-23649	NS_000200.1	+	334938396	334938444
Sma-1-23650	NS_000200.1	-	366345707	366345659
Sma-1-23651	NS_000200.1	-	223451765	223451717
Sma-1-23652	NS_000200.1	-	173374389	173374341
Sma-1-23653	NS_000200.1	-	145022129	145022081
Sma-1-23654	NS_000200.1	-	100621925	100621877
Sma-1-23655	NW_003037903.1	+	626047	626095
Sma-1-23656	NS_000200.1	+	148180128	148180176
Sma-1-23657	NW_003037853.1	-	81610	81562
Sma-1-23658	NS_000200.1	-	95573661	95573613
Sma-1-23659	NW_003038002.1	+	310974	311022
Sma-1-23660	NS_000200.1	+	223431183	223431231
Sma-1-23661	NW_003020614.1	-	3456	3408
Sma-1-23662	NW_003037889.1	+	920036	920084
Sma-1-23663	NS_000200.1	+	134696526	134696574
Sma-1-23664	NS_000200.1	-	360436161	360436113
Sma-1-23665	NW_003037891.1	-	1567417	1567369
Sma-1-23666	NS_000200.1	-	139448695	139448647

Sma-1-23667	NW_003026471.1	-	191649	191601
Sma-1-23668	NS_000200.1	-	222303882	222303834
Sma-1-23669	NW_003038081.1	-	345961	345913
Sma-1-23670	NS_000200.1	-	269508004	269507956
Sma-1-23671	NW_003026432.1	-	1351	1303
Sma-1-23672	NW_003035880.1	+	193289	193337
Ehi-1-2	NW_001916244.1	-	949	901
Ehi-1-3	NW_001916244.1	-	283	235
Sma-1-23673	NW_003026471.1	-	353755	353707
Sma-1-23674	NW_003037818.1	+	1438740	1438788
Sma-1-23675	NW_003037821.1	-	645951	645903
Sma-1-23676	NW_003037837.1	-	719002	718954
Sma-1-23677	NW_003037853.1	+	436959	437007
Sma-1-23678	NW_003037865.1	-	497851	497803
Sma-1-23679	NW_003037902.1	-	124151	124103
Sma-1-23680	NW_003037912.1	+	1353938	1353986
Sma-1-23681	NW_003037916.1	+	1120285	1120333
Sma-1-23682	NW_003037939.1	+	140421	140469
Sma-1-23683	NW_003037978.1	-	297238	297190
Sma-1-23684	NW_003037980.1	-	201535	201487
Sma-1-23685	NW_003037981.1	+	139328	139376
Sma-1-23686	NW_003038016.1	-	160261	160213
Sma-1-23687	NW_003026515.1	+	144961	145009
Sma-1-23688	NW_003038048.1	+	67787	67835
Sma-1-23689	NW_003038156.1	-	170587	170539
Sma-1-23690	NS_000200.1	+	50154147	50154195
Sma-1-23691	NS_000200.1	+	95929010	95929058
Sma-1-23692	NS_000200.1	+	158165513	158165561
Sma-1-23693	NS_000200.1	+	164313696	164313744
Sma-1-23694	NS_000200.1	+	183010175	183010223
Sma-1-23695	NS_000200.1	+	212537196	212537244
Sma-1-23696	NS_000200.1	+	255645618	255645666
Sma-1-23697	NS_000200.1	+	290789372	290789420
Sma-1-23698	NS_000200.1	+	339434353	339434401
Sma-1-23699	NS_000200.1	-	345004182	345004134
Sma-1-23700	NS_000200.1	-	294902700	294902652
Sma-1-23701	NS_000200.1	-	231276798	231276750
Sma-1-23702	NS_000200.1	-	222465988	222465940
Sma-1-23703	NS_000200.1	-	211162397	211162349
Sma-1-23704	NS_000200.1	-	209565248	209565200
Sma-1-23705	NS_000200.1	-	146879385	146879337
Sma-1-23706	NS_000200.1	-	110919770	110919722
Sma-1-23707	NS_000200.1	-	77524860	77524812
Sma-1-23708	NS_000200.1	-	53790435	53790387
Sma-1-23709	NW_003038052.1	+	13239	13287
Sma-1-23710	NS_000200.1	+	257060715	257060763
Sma-1-23711	NW_003037874.1	-	218935	218887
Sma-1-23712	NS_000200.1	-	119643885	119643837
Sma-1-23713	NW_003028088.1	-	1633	1585
Sma-1-23714	NS_000200.1	-	369314959	369314911
Sma-1-23715	NW_003038206.1	-	40229	40181
Sma-1-23716	NW_003026456.1	-	190793	190745

Sma-1-23717	NW_003037816.1	+	846805	846853
Sma-1-23718	NW_003037831.1	+	1312144	1312192
Sma-1-23719	NW_003038246.1	-	170829	170781
Sma-1-23720	NW_003038048.1	+	68121	68169
Sma-1-23721	NS_000200.1	+	47055677	47055725
Sma-1-23722	NS_000200.1	+	70761446	70761494
Sma-1-23723	NS_000200.1	+	255645952	255646000
Sma-1-23724	NS_000200.1	-	315811957	315811909
Sma-1-23725	NS_000200.1	-	304866993	304866945
Sma-1-23726	NS_000200.1	-	58113929	58113881
Sma-1-23727	NW_003038008.1	-	189379	189331
Sma-1-23728	NS_000200.1	-	225862475	225862427
Sma-1-23729	NW_003022642.1	-	664	616
Sma-1-23730	NS_000200.1	-	365335190	365335142
Sma-1-23731	NW_003037889.1	-	777274	777226
Sma-1-23732	NS_000200.1	-	134553764	134553716
Sma-1-23733	NW_003038516.1	+	139135	139183
Sma-1-23734	NW_003035881.1	+	138399	138447
Sma-1-23735	NW_003037800.1	+	1262582	1262630
Sma-1-23736	NW_003037800.1	-	1294421	1294373
Sma-1-23737	NW_003037853.1	-	962610	962562
Sma-1-23738	NW_003037942.1	+	478736	478784
Sma-1-23739	NW_003037942.1	-	463447	463399
Sma-1-23740	NW_003038032.1	+	664596	664644
Sma-1-23741	NS_000200.1	+	19891942	19891990
Sma-1-23742	NS_000200.1	+	186238644	186238692
Sma-1-23743	NS_000200.1	+	245823756	245823804
Sma-1-23744	NS_000200.1	+	340627540	340627588
Sma-1-23745	NS_000200.1	+	341202604	341202652
Sma-1-23746	NS_000200.1	-	186223355	186223307
Sma-1-23747	NS_000200.1	-	96454661	96454613
Sma-1-23748	NS_000200.1	-	19923781	19923733
Sma-1-23749	NW_003037979.1	+	777393	777441
Sma-1-23750	NS_000200.1	+	210838413	210838461
Sma-1-23751	NW_003037993.1	+	180427	180475
Sma-1-23752	NS_000200.1	+	218367130	218367178
Sma-1-23753	NW_003037861.1	-	440117	440069
Sma-1-23754	NS_000200.1	-	105216571	105216523
Sma-1-23755	NW_003037964.1	+	38113	38161
Sma-1-23756	NS_000200.1	+	197639635	197639683
Sma-1-23757	NW_003037864.1	+	1272959	1273007
Sma-1-23758	NS_000200.1	+	109765533	109765581
Sma-1-23759	NW_003035881.1	+	25808	25856
Sma-1-23760	NS_000200.1	+	341090013	341090061
Sma-1-23761	NW_003037591.1	+	4329	4377
Sma-1-23762	NW_003035662.1	+	18917	18965
Sma-1-23763	NW_003037798.1	+	1917228	1917276
Sma-1-23764	NW_003037798.1	+	2375775	2375823
Sma-1-23765	NW_003037808.1	+	1307796	1307844
Sma-1-23766	NW_003037811.1	+	878314	878362
Sma-1-23767	NW_003037813.1	+	87118	87166
Sma-1-23768	NW_003037819.1	-	883041	882993

Sma-1-23769	NW_003037826.1	+	1527793	1527841
Sma-1-23770	NW_003037871.1	-	315466	315418
Sma-1-23771	NW_003037910.1	-	772734	772686
Sma-1-23772	NW_003037915.1	+	697929	697977
Sma-1-23773	NW_003037938.1	-	825841	825793
Sma-1-23774	NW_003037941.1	+	375516	375564
Sma-1-23775	NW_003037977.1	+	318388	318436
Sma-1-23776	NW_003038410.1	+	27267	27315
Sma-1-23777	NW_003038410.1	+	35755	35803
Sma-1-23778	NW_003038474.1	-	41515	41467
Sma-1-23779	NW_003038083.1	-	442048	442000
Sma-1-23780	NW_003038187.1	-	18741	18693
Sma-1-23781	NS_000200.1	+	16176386	16176434
Sma-1-23782	NS_000200.1	+	16634933	16634981
Sma-1-23783	NS_000200.1	+	33094433	33094481
Sma-1-23784	NS_000200.1	+	39048556	39048604
Sma-1-23785	NS_000200.1	+	41073981	41074029
Sma-1-23786	NS_000200.1	+	62527309	62527357
Sma-1-23787	NS_000200.1	+	162597915	162597963
Sma-1-23788	NS_000200.1	+	185109990	185110038
Sma-1-23789	NS_000200.1	+	208604408	208604456
Sma-1-23790	NS_000200.1	+	328493845	328493893
Sma-1-23791	NS_000200.1	+	328502333	328502381
Sma-1-23792	NS_000200.1	+	331031223	331031271
Sma-1-23793	NS_000200.1	+	372705128	372705176
Sma-1-23794	NS_000200.1	-	332272729	332272681
Sma-1-23795	NS_000200.1	-	301970599	301970551
Sma-1-23796	NS_000200.1	-	270685385	270685337
Sma-1-23797	NS_000200.1	-	182862954	182862906
Sma-1-23798	NS_000200.1	-	155284709	155284661
Sma-1-23799	NS_000200.1	-	117763204	117763156
Sma-1-23800	NS_000200.1	-	51971306	51971258
Sma-1-23801	NW_003038240.1	+	90481	90529
Sma-1-23802	NS_000200.1	+	314229412	314229460
Sma-1-23803	NW_003038023.1	-	333385	333337
Sma-1-23804	NS_000200.1	-	237493676	237493628
Sma-1-23805	NW_003026464.1	-	473621	473573
Sma-1-23806	NW_003037976.1	+	66342	66390
Sma-1-23807	NS_000200.1	+	206680657	206680705
Sma-1-23808	NS_000200.1	-	203773930	203773882
Sma-1-23809	NW_003038008.1	+	700422	700470
Sma-1-23810	NS_000200.1	+	226373518	226373566
Sma-1-23811	NW_003026465.1	+	343139	343187
Sma-1-23812	NS_000200.1	+	207735733	207735781
Sma-1-23813	NW_003038156.1	-	11694	11646
Sma-1-23814	NS_000200.1	-	294743807	294743759
Sma-1-23815	NW_003038159.1	+	60563	60611
Sma-1-23816	NS_000200.1	+	296448584	296448632
Sma-1-23817	NW_003031098.1	-	1123	1075
Sma-1-23818	NW_003038014.1	+	300971	301019
Sma-1-23819	NW_003038014.1	+	312592	312640
Sma-1-23820	NS_000200.1	+	230041660	230041708

Sma-1-23821	NS_000200.1	+	230053281	230053329
Sma-1-23822	NS_000200.1	-	325692376	325692328
Sma-1-23823	NW_003037884.1	-	890458	890410
Sma-1-23824	NS_000200.1	-	127670123	127670075
Sma-1-23825	NW_003025092.1	+	165	213
Sma-1-23826	NW_003029795.1	+	829	877
Sma-1-23827	NW_003038420.1	-	11787	11739
Sma-1-23828	NW_003037822.1	+	78801	78849
Sma-1-23829	NW_003037833.1	-	603297	603249
Sma-1-23830	NW_003037886.1	-	1005334	1005286
Sma-1-23831	NW_003037915.1	-	593648	593600
Sma-1-23832	NW_003037973.1	+	86707	86755
Sma-1-23833	NW_003038036.1	-	1125280	1125232
Sma-1-23834	NW_003026488.1	+	66099	66147
Sma-1-23835	NW_003038129.1	-	207961	207913
Sma-1-23836	NS_000200.1	+	54386807	54386855
Sma-1-23837	NS_000200.1	+	204068434	204068482
Sma-1-23838	NS_000200.1	+	263572593	263572641
Sma-1-23839	NS_000200.1	+	361160723	361160771
Sma-1-23840	NS_000200.1	+	375627531	375627579
Sma-1-23841	NS_000200.1	-	328933818	328933770
Sma-1-23842	NS_000200.1	-	281461308	281461260
Sma-1-23843	NS_000200.1	-	250059132	250059084
Sma-1-23844	NS_000200.1	-	162493634	162493586
Sma-1-23845	NS_000200.1	-	130260901	130260853
Sma-1-23846	NS_000200.1	-	72794393	72794345
Sma-1-23847	NW_003038216.1	-	84506	84458
Sma-1-23848	NS_000200.1	-	306870138	306870090
Sma-1-23849	NW_003037853.1	+	709130	709178
Sma-1-23850	NS_000200.1	+	96201181	96201229
Sma-1-23851	NW_003032295.1	+	3171	3219
Sma-1-23852	NW_003037965.1	+	318828	318876
Sma-1-23853	NW_003037993.1	-	162791	162743
Sma-1-23854	NS_000200.1	+	198607913	198607961
Sma-1-23855	NS_000200.1	+	358631766	358631814
Sma-1-23856	NS_000200.1	-	218349494	218349446
Sma-1-23857	NW_003037996.1	+	313652	313700
Sma-1-23858	NS_000200.1	+	220558528	220558576
Sma-1-23859	NW_003037898.1	+	447803	447851
Sma-1-23860	NW_003037826.1	+	678080	678128
Sma-1-23861	NS_000200.1	+	61677596	61677644
Sma-1-23862	NS_000200.1	+	144431625	144431673
Sma-1-23863	NW_003037871.1	+	257852	257900
Sma-1-23864	NS_000200.1	+	117705590	117705638
Sma-1-23865	NW_003037881.1	-	475075	475027
Sma-1-23866	NS_000200.1	-	124906866	124906818
Sma-1-23867	NW_003037972.1	+	258436	258484
Sma-1-23868	NS_000200.1	+	202734140	202734188
Sma-1-23869	NW_003038033.1	-	816869	816821
Sma-1-23870	NS_000200.1	-	246796871	246796823
Sma-1-23871	NW_003038032.1	-	442759	442711
Sma-1-23872	NS_000200.1	-	245601919	245601871

Sma-1-23873	NW_003039129.1	-	401254	401206
Sma-1-23874	NW_003037887.1	-	424401	424353
Sma-1-23875	NW_003037903.1	+	641504	641552
Sma-1-23876	NS_000200.1	+	148195585	148195633
Sma-1-23877	NS_000200.1	-	229356062	229356014
Sma-1-23878	NS_000200.1	-	130888379	130888331
Sma-1-23879	NW_003038038.1	-	757444	757396
Sma-1-23880	NS_000200.1	-	252660837	252660789
Sma-1-23881	NW_003020794.1	-	1990	1942
Sma-1-23882	NW_003037874.1	-	117066	117018
Sma-1-23883	NW_003037774.1	-	4044	3996
Sma-1-23884	NW_003038006.1	-	37380	37332
Sma-1-23885	NW_003037929.1	-	330609	330561
Sma-1-23886	NW_003037793.1	-	2362213	2362165
Sma-1-23887	NW_003037800.1	-	1418390	1418342
Sma-1-23888	NW_003037809.1	+	753309	753357
Sma-1-23889	NW_003037822.1	+	456737	456785
Sma-1-23890	NW_003037839.1	+	977238	977286
Sma-1-23891	NW_003037845.1	-	500339	500291
Sma-1-23892	NW_003037884.1	-	1084145	1084097
Sma-1-23893	NW_003037901.1	-	518937	518889
Sma-1-23894	NW_003037910.1	-	203604	203556
Sma-1-23895	NW_003037915.1	+	1112921	1112969
Sma-1-23896	NW_003037971.1	-	276133	276085
Sma-1-23897	NW_003037976.1	-	59347	59299
Sma-1-23898	NW_003037979.1	+	110606	110654
Sma-1-23899	NW_003038059.1	-	271221	271173
Sma-1-23900	NW_003038120.1	+	193503	193551
Sma-1-23901	NW_003038172.1	-	42493	42445
Sma-1-23902	NS_000200.1	+	34417773	34417821
Sma-1-23903	NS_000200.1	+	54764743	54764791
Sma-1-23904	NS_000200.1	+	79935388	79935436
Sma-1-23905	NS_000200.1	+	163012907	163012955
Sma-1-23906	NS_000200.1	+	210171626	210171674
Sma-1-23907	NS_000200.1	+	278378726	278378774
Sma-1-23908	NS_000200.1	-	378589421	378589373
Sma-1-23909	NS_000200.1	-	364471167	364471119
Sma-1-23910	NS_000200.1	-	300716008	300715960
Sma-1-23911	NS_000200.1	-	260513293	260513245
Sma-1-23912	NS_000200.1	-	224921864	224921816
Sma-1-23913	NS_000200.1	-	206673662	206673614
Sma-1-23914	NS_000200.1	-	201898183	201898135
Sma-1-23915	NS_000200.1	-	175571387	175571339
Sma-1-23916	NS_000200.1	-	154715579	154715531
Sma-1-23917	NS_000200.1	-	146367398	146367350
Sma-1-23918	NS_000200.1	-	127863810	127863762
Sma-1-23919	NS_000200.1	-	119542016	119541968
Sma-1-23920	NS_000200.1	-	87502242	87502194
Sma-1-23921	NS_000200.1	-	20047750	20047702
Sma-1-23922	NS_000200.1	-	2362213	2362165
Sma-1-23923	NW_003037861.1	+	269592	269640
Sma-1-23924	NS_000200.1	+	105046046	105046094

Sma-1-23925	NW_003038242.1	+	224205	224253
Sma-1-23926	NS_000200.1	+	314923351	314923399
Sma-1-23927	NW_003037831.1	-	1649280	1649232
Sma-1-23928	NS_000200.1	-	71098582	71098534
Sma-1-23929	NW_003026458.1	+	436075	436123
Sma-1-23930	NS_000200.1	+	102304305	102304353
Sma-1-23931	NW_003038516.1	-	197160	197112
Sma-1-23932	NW_003037904.1	+	327615	327663
Sma-1-23933	NS_000200.1	+	149042114	149042162
Sma-1-23934	NS_000200.1	-	340685565	340685517
Sma-1-23935	NW_003025959.1	-	860	812
Sma-1-23936	NS_000200.1	-	352937212	352937164
Sma-1-23937	NW_003038038.1	+	809411	809459
Sma-1-23938	NS_000200.1	+	252712804	252712852
Sma-1-23939	NW_003037798.1	+	2098887	2098935
Sma-1-23940	NW_003037809.1	-	2531852	2531804
Sma-1-23941	NW_003037903.1	+	657307	657355
Sma-1-23942	NS_000200.1	+	16358045	16358093
Sma-1-23943	NS_000200.1	+	148211388	148211436
Sma-1-23944	NS_000200.1	-	36196316	36196268
Sma-1-23945	NW_003038036.1	-	722407	722359
Sma-1-23946	NS_000200.1	-	249656259	249656211
Sma-1-23947	NW_003035715.1	-	260	212
Sma-1-23948	NW_003037934.1	+	33989	34037
Sma-1-23949	NW_003037795.1	-	440527	440479
Sma-1-23950	NW_003037797.1	+	1032821	1032869
Sma-1-23951	NW_003037814.1	+	1605112	1605160
Sma-1-23952	NW_003037827.1	+	1111726	1111774
Sma-1-23953	NW_003037829.1	+	1808792	1808840
Sma-1-23954	NW_003037830.1	-	1078098	1078050
Sma-1-23955	NW_003037841.1	+	2327944	2327992
Sma-1-23956	NW_003037841.1	-	2327765	2327717
Sma-1-23957	NW_003037860.1	-	865758	865710
Sma-1-23958	NW_003037868.1	+	219831	219879
Sma-1-23959	NW_003037878.1	-	773174	773126
Sma-1-23960	NW_003037902.1	-	130110	130062
Sma-1-23961	NW_003037916.1	-	1615707	1615659
Sma-1-23962	NW_003037941.1	+	989084	989132
Sma-1-23963	NW_003037964.1	-	120173	120125
Sma-1-23964	NW_003037980.1	+	1361651	1361699
Sma-1-23965	NW_003038232.1	+	287560	287608
Sma-1-23966	NW_003038086.1	-	58168	58120
Sma-1-23967	NW_003038098.1	+	31460	31508
Sma-1-23968	NS_000200.1	+	11460681	11460729
Sma-1-23969	NS_000200.1	+	44588972	44589020
Sma-1-23970	NS_000200.1	+	63733867	63733915
Sma-1-23971	NS_000200.1	+	67709330	67709378
Sma-1-23972	NS_000200.1	+	84939125	84939173
Sma-1-23973	NS_000200.1	+	116191543	116191591
Sma-1-23974	NS_000200.1	+	179080736	179080784
Sma-1-23975	NS_000200.1	+	185723558	185723606
Sma-1-23976	NS_000200.1	+	212322513	212322561

Sma-1-23977	NS_000200.1	+	274416258	274416306
Sma-1-23978	NS_000200.1	+	312251351	312251399
Sma-1-23979	NS_000200.1	-	363932808	363932760
Sma-1-23980	NS_000200.1	-	272306761	272306713
Sma-1-23981	NS_000200.1	-	197721695	197721647
Sma-1-23982	NS_000200.1	-	164809118	164809070
Sma-1-23983	NS_000200.1	-	146885344	146885296
Sma-1-23984	NS_000200.1	-	122826652	122826604
Sma-1-23985	NS_000200.1	-	104608786	104608738
Sma-1-23986	NS_000200.1	-	84938946	84938898
Sma-1-23987	NS_000200.1	-	68968469	68968421
Sma-1-23988	NS_000200.1	-	6831410	6831362
Sma-1-23989	NW_003038033.1	+	401628	401676
Sma-1-23990	NS_000200.1	+	246381630	246381678
Sma-1-23991	NW_003026490.1	+	168479	168527
Sma-1-23992	NS_000200.1	+	267800195	267800243
Sma-1-23993	NW_003027359.1	+	114	162
Sma-1-23994	NW_003037818.1	-	1505247	1505199
Sma-1-23995	NS_000200.1	+	356726689	356726737
Sma-1-23996	NS_000200.1	-	50220654	50220606
Sma-1-23997	NW_003026609.1	-	40587	40539
Sma-1-23998	NS_000200.1	-	309695579	309695531
Sma-1-23999	NW_003037882.1	-	240673	240625
Sma-1-24000	NS_000200.1	-	125532347	125532299
Sma-1-24001	NW_003023776.1	-	968	920
Sma-1-24002	NS_000200.1	-	368789031	368788983
Sma-1-24003	NW_003037830.1	-	1491575	1491527
Sma-1-24004	NS_000200.1	-	69381946	69381898
Sma-1-24005	NW_003037972.1	-	355921	355873
Sma-1-24006	NS_000200.1	-	202831625	202831577
Sma-1-24007	NW_003037908.1	+	329451	329499
Sma-1-24008	NS_000200.1	+	152802582	152802630
Sma-1-24009	NW_003026600.1	+	20282	20330
Sma-1-24010	NS_000200.1	+	306291783	306291831
Sma-1-24011	NW_003037829.1	-	1641169	1641121
Sma-1-24012	NS_000200.1	-	67541707	67541659
Sma-1-24013	NW_003037904.1	-	287440	287392
Sma-1-24014	NS_000200.1	-	149001939	149001891
Sma-1-24015	NW_003037977.1	-	865002	864954
Sma-1-24016	NS_000200.1	-	209151022	209150974
Sma-1-24017	NW_003037834.1	+	970724	970772
Sma-1-24018	NS_000200.1	+	74131210	74131258
Sma-1-24019	NW_003037861.1	+	151888	151936
Sma-1-24020	NS_000200.1	+	104928342	104928390
Sma-1-24021	NW_003037804.1	-	1198365	1198314
Sma-1-24022	NS_000200.1	-	26338385	26338334
Sma-1-24023	NW_003037901.1	-	374912	374864
Sma-1-24024	NW_003038471.1	-	46603	46555
Sma-1-24025	NS_000200.1	-	331887517	331887469
Sma-1-24026	NS_000200.1	-	146223373	146223325
Sma-1-24027	NW_003038034.1	+	211830	211878
Sma-1-24028	NW_003038071.1	+	78257	78305

Sma-1-24029	NS_000200.1	+	247153586	247153634
Sma-1-24030	NS_000200.1	+	264881300	264881348
Sma-1-24031	NW_003037977.1	+	396045	396093
Sma-1-24032	NS_000200.1	+	208682065	208682113
Sma-1-24033	NW_003026597.1	+	109019	109067
Sma-1-24034	NS_000200.1	+	305730205	305730253
Sma-1-24035	NW_003037838.1	+	354932	354980
Sma-1-24036	NS_000200.1	+	78353495	78353543
Sma-1-24037	NW_003037840.1	-	1891060	1891012
Sma-1-24038	NW_003037840.1	-	1885014	1884966
Sma-1-24039	NS_000200.1	-	82514384	82514336
Sma-1-24040	NS_000200.1	-	82508338	82508290
Sma-1-24041	NW_003037847.1	+	849287	849335
Sma-1-24042	NS_000200.1	+	89501017	89501065
Sma-1-24043	NW_003037914.1	+	241794	241842
Sma-1-24044	NW_003037969.1	-	334437	334389
Sma-1-24045	NW_003038031.1	+	19901	19949
Sma-1-24046	NS_000200.1	+	160386044	160386092
Sma-1-24047	NS_000200.1	+	244297659	244297707
Sma-1-24048	NS_000200.1	-	200940564	200940516
Sma-1-24049	NW_003037829.1	+	517013	517061
Sma-1-24050	NS_000200.1	+	66417551	66417599
Sma-1-24051	NW_003037830.1	+	756445	756493
Sma-1-24052	NS_000200.1	+	68646816	68646864
Sma-1-24053	NW_003020771.1	+	233	281
Sma-1-24054	NW_003026472.1	+	21884	21932
Sma-1-24055	NS_000200.1	+	236433876	236433924
Sma-1-24056	NS_000200.1	+	364423463	364423511
Sma-1-24057	NW_003026458.1	-	402527	402479
Sma-1-24058	NW_003038033.1	-	306723	306675
Sma-1-24059	NS_000200.1	-	246286725	246286677
Sma-1-24060	NS_000200.1	-	102270757	102270709
Sma-1-24061	NW_003037982.1	+	10519	10567
Sma-1-24062	NS_000200.1	+	213669714	213669762
Sma-1-24063	NW_003026608.1	+	57177	57225
Sma-1-24064	NW_003037825.1	+	1205489	1205537
Sma-1-24065	NS_000200.1	+	60335271	60335319
Sma-1-24066	NS_000200.1	+	309335297	309335345
Sma-1-24067	NW_003037903.1	-	964540	964492
Sma-1-24068	NS_000200.1	-	148518621	148518573
Sma-1-24069	NW_003037841.1	+	582404	582452
Sma-1-24070	NW_003037884.1	-	248645	248597
Sma-1-24071	NW_003038145.1	-	241502	241454
Sma-1-24072	NS_000200.1	+	83193585	83193633
Sma-1-24073	NS_000200.1	-	288852925	288852877
Sma-1-24074	NS_000200.1	-	127028310	127028262
Sma-1-24075	NW_003038009.1	+	33530	33578
Sma-1-24076	NS_000200.1	+	226407593	226407641
Sma-1-24077	NW_003037926.1	+	296102	296150
Sma-1-24078	NS_000200.1	+	173464372	173464420
Sma-1-24079	NW_003037845.1	-	347853	347805
Sma-1-24080	NS_000200.1	-	87349756	87349708

Sma-1-24081	NW_003037804.1	-	212958	212910
Sma-1-24082	NW_003038261.1	+	449549	449597
Sma-1-24083	NS_000200.1	+	324063200	324063248
Sma-1-24084	NS_000200.1	-	25352978	25352930
Sma-1-24085	NW_003039127.1	+	1465736	1465784
Sma-1-24086	NW_003039127.1	-	1465307	1465259
Sma-1-24087	NS_000200.1	+	114543235	114543283
Sma-1-24088	NS_000200.1	-	114542806	114542758
Sma-1-24089	NW_003037873.1	-	517463	517415
Sma-1-24090	NS_000200.1	-	119256591	119256543
Sma-1-24091	NW_003038035.1	+	695782	695830
Sma-1-24092	NS_000200.1	+	248557274	248557322
Sma-1-24093	NW_003038157.1	+	302300	302348
Sma-1-24094	NS_000200.1	+	295553220	295553268
Sma-1-24095	NW_003021339.1	+	1110	1158
Sma-1-24096	NW_003029697.1	-	1297	1249
Sma-1-24097	NW_003038215.1	+	84620	84668
Sma-1-24098	NW_003038006.1	-	51217	51169
Sma-1-24099	NW_003038006.1	-	50010	49962
Sma-1-24100	NW_003026619.1	-	107581	107533
Sma-1-24101	NW_003037810.1	-	1372968	1372920
Sma-1-24102	NW_003037818.1	+	494603	494651
Sma-1-24103	NW_003037829.1	+	672346	672394
Sma-1-24104	NW_003037839.1	+	945325	945373
Sma-1-24105	NW_003037865.1	-	199320	199272
Sma-1-24106	NW_003037866.1	-	328046	327998
Sma-1-24107	NW_003037889.1	+	1506698	1506746
Sma-1-24108	NW_003037891.1	+	1629992	1630040
Sma-1-24109	NW_003037918.1	-	1608331	1608283
Sma-1-24110	NW_003038033.1	+	539002	539050
Sma-1-24111	NW_003038227.1	+	147151	147199
Sma-1-24112	NW_003038066.1	-	229778	229730
Sma-1-24113	NW_003038083.1	+	217343	217391
Sma-1-24114	NS_000200.1	+	49210010	49210058
Sma-1-24115	NS_000200.1	+	66572884	66572932
Sma-1-24116	NS_000200.1	+	79903475	79903523
Sma-1-24117	NS_000200.1	+	135283188	135283236
Sma-1-24118	NS_000200.1	+	139511270	139511318
Sma-1-24119	NS_000200.1	+	246519004	246519052
Sma-1-24120	NS_000200.1	+	270460680	270460728
Sma-1-24121	NS_000200.1	+	306697656	306697704
Sma-1-24122	NS_000200.1	+	310428505	310428553
Sma-1-24123	NS_000200.1	+	343892850	343892898
Sma-1-24124	NS_000200.1	-	366441001	366440953
Sma-1-24125	NS_000200.1	-	322298325	322298277
Sma-1-24126	NS_000200.1	-	262698722	262698674
Sma-1-24127	NS_000200.1	-	224935701	224935653
Sma-1-24128	NS_000200.1	-	224934494	224934446
Sma-1-24129	NS_000200.1	-	168175321	168175273
Sma-1-24130	NS_000200.1	-	111815845	111815797
Sma-1-24131	NS_000200.1	-	110621239	110621191
Sma-1-24132	NS_000200.1	-	37977490	37977442

Sma-1-24133	NW_003037853.1	+	432201	432249
Sma-1-24134	NS_000200.1	+	95924252	95924300
Sma-1-24135	NW_003037793.1	+	908099	908147
Sma-1-24136	NS_000200.1	+	908099	908147
Sma-1-24137	NW_003037975.1	+	375260	375308
Sma-1-24138	NS_000200.1	+	205957484	205957532
Sma-1-24139	NW_003026487.1	+	88966	89014
Sma-1-24140	NS_000200.1	+	260999660	260999708
Sma-1-24141	NW_003026474.1	-	26934	26886
Sma-1-24142	NS_000200.1	-	253225509	253225461
Sma-1-24143	NW_003037834.1	+	941441	941489
Sma-1-24144	NW_003037852.1	+	96122	96170
Sma-1-24145	NS_000200.1	+	74101927	74101975
Sma-1-24146	NS_000200.1	+	94488017	94488065
Sma-1-24147	NW_003037837.1	-	965981	965933
Sma-1-24148	NS_000200.1	-	77771839	77771791
Sma-1-24149	NW_003023394.1	+	136	184
Sma-1-24150	NS_000200.1	+	373264643	373264691
Sma-1-24151	NW_003037888.1	-	66203	66155
Sma-1-24152	NS_000200.1	-	132274695	132274647
Sma-1-24153	NW_003037909.1	-	287487	287439
Sma-1-24154	NS_000200.1	-	153829136	153829088
Sma-1-24155	NW_003020571.1	+	153	201
Sma-1-24156	NS_000200.1	+	360236053	360236101
Sma-1-24157	NW_003037848.1	-	1056667	1056619
Sma-1-24158	NS_000200.1	-	90749738	90749690
Sma-1-24159	NW_003038018.1	-	427602	427554
Sma-1-24160	NS_000200.1	-	232828452	232828404
Sma-1-24161	NW_003039127.1	-	368083	368035
Sma-1-24162	NS_000200.1	-	113445582	113445534
Sma-1-24163	NW_003038083.1	+	235226	235274
Sma-1-24164	NS_000200.1	+	270478563	270478611
Sma-1-24165	NW_003038087.1	-	116796	116748
Sma-1-24166	NS_000200.1	-	273145197	273145149
Sma-1-24167	NW_003038494.1	-	8015	7967
Sma-1-24168	NS_000200.1	-	334195716	334195668
Sma-1-24169	NW_003023437.1	-	103	55
Sma-1-24170	NW_003037874.1	+	576109	576157
Sma-1-24171	NW_003026472.1	-	102839	102791
Sma-1-24172	NW_003037920.1	-	128574	128526
Sma-1-24173	NW_003026465.1	-	632461	632413
Sma-1-24174	NW_003026619.1	-	270539	270491
Sma-1-24175	NW_003037797.1	-	1516666	1516618
Sma-1-24176	NW_003037813.1	+	1969684	1969732
Sma-1-24177	NW_003037816.1	+	176532	176580
Sma-1-24178	NW_003037845.1	+	499684	499732
Sma-1-24179	NW_003037863.1	+	759909	759957
Sma-1-24180	NW_003037903.1	+	288975	289023
Sma-1-24181	NW_003037906.1	-	402790	402742
Sma-1-24182	NW_003037931.1	+	172036	172084
Sma-1-24183	NW_003037933.1	-	330379	330331
Sma-1-24184	NW_003037964.1	-	372474	372426

Sma-1-24185	NW_003037976.1	+	327713	327761
Sma-1-24186	NW_003038042.1	-	84527	84479
Sma-1-24187	NS_000200.1	+	42956547	42956595
Sma-1-24188	NS_000200.1	+	46385404	46385452
Sma-1-24189	NS_000200.1	+	87501587	87501635
Sma-1-24190	NS_000200.1	+	107518276	107518324
Sma-1-24191	NS_000200.1	+	120001059	120001107
Sma-1-24192	NS_000200.1	+	147843056	147843104
Sma-1-24193	NS_000200.1	+	177004703	177004751
Sma-1-24194	NS_000200.1	+	206942028	206942076
Sma-1-24195	NS_000200.1	-	373379266	373379218
Sma-1-24196	NS_000200.1	-	322461283	322461235
Sma-1-24197	NS_000200.1	-	254030126	254030078
Sma-1-24198	NS_000200.1	-	236514831	236514783
Sma-1-24199	NS_000200.1	-	208025055	208025007
Sma-1-24200	NS_000200.1	-	197973996	197973948
Sma-1-24201	NS_000200.1	-	178743551	178743503
Sma-1-24202	NS_000200.1	-	169309381	169309333
Sma-1-24203	NS_000200.1	-	150710569	150710521
Sma-1-24204	NS_000200.1	-	11944526	11944478
Sma-1-24205	NW_003037865.1	+	526015	526063
Sma-1-24206	NS_000200.1	+	110947934	110947982
Sma-1-24207	NW_003029842.1	+	10028	10076
Sma-1-24208	NS_000200.1	+	376360547	376360595
Sma-1-24209	NW_003038197.1	+	48391	48439
Sma-1-24210	NS_000200.1	+	303284718	303284766
Sma-1-24211	NW_003037906.1	+	763724	763772
Sma-1-24212	NS_000200.1	+	151071503	151071551
Sma-1-24213	NW_003037859.1	-	689061	689013
Sma-1-24214	NS_000200.1	-	103367902	103367854
Sma-1-24215	NW_003038035.1	+	363681	363729
Sma-1-24216	NS_000200.1	+	248225173	248225221
Sma-1-24217	NW_003035319.1	-	338	290
Sma-1-24218	NW_003038457.1	-	22678	22630
Sma-1-24219	NW_003037813.1	+	1994159	1994207
Sma-1-24220	NW_003037887.1	-	1629637	1629589
Sma-1-24221	NW_003037900.1	+	38326	38374
Sma-1-24222	NW_003037916.1	-	174182	174134
Sma-1-24223	NW_003037918.1	-	1835069	1835021
Sma-1-24224	NS_000200.1	+	42981022	42981070
Sma-1-24225	NS_000200.1	+	145167192	145167240
Sma-1-24226	NS_000200.1	-	371004181	371004133
Sma-1-24227	NS_000200.1	-	331104542	331104494
Sma-1-24228	NS_000200.1	-	168402059	168402011
Sma-1-24229	NS_000200.1	-	163367593	163367545
Sma-1-24230	NS_000200.1	-	132093615	132093567
Sma-1-24231	NW_003037914.1	+	477885	477933
Sma-1-24232	NS_000200.1	+	160622135	160622183
Sma-1-24233	NW_003037793.1	+	2084803	2084851
Sma-1-24234	NW_003037846.1	-	658461	658413
Sma-1-24235	NW_003037864.1	-	976016	975968
Sma-1-24236	NW_003037878.1	+	768223	768271

Sma-1-24237	NW_003037900.1	-	63275	63227
Sma-1-24238	NW_003038241.1	+	69886	69934
Sma-1-24239	NS_000200.1	+	2084803	2084851
Sma-1-24240	NS_000200.1	+	122821701	122821749
Sma-1-24241	NS_000200.1	+	314532591	314532639
Sma-1-24242	NS_000200.1	-	145192141	145192093
Sma-1-24243	NS_000200.1	-	109468590	109468542
Sma-1-24244	NS_000200.1	-	88293031	88292983
Sma-1-24245	NW_003037873.1	-	69858	69810
Sma-1-24246	NS_000200.1	-	118808986	118808938
Sma-1-24247	NW_003037793.1	-	1950236	1950188
Sma-1-24248	NS_000200.1	-	1950236	1950188
Sma-1-24249	NW_003037793.1	+	2606627	2606675
Sma-1-24250	NS_000200.1	+	2606627	2606675
Sma-1-24251	NW_003037823.1	-	794226	794178
Sma-1-24252	NS_000200.1	-	56710845	56710797
Sma-1-24253	NW_003026488.1	-	267453	267404
Sma-1-24254	NS_000200.1	-	263773947	263773898
Sma-1-24255	NW_003037814.1	+	169823	169871
Sma-1-24256	NW_003037829.1	-	1479728	1479680
Sma-1-24257	NW_003037943.1	+	1171095	1171143
Sma-1-24258	NS_000200.1	+	43153683	43153731
Sma-1-24259	NS_000200.1	+	188192628	188192676
Sma-1-24260	NS_000200.1	-	67380266	67380218
Sma-1-24261	NW_003035551.1	-	4836	4788
Sma-1-24262	NS_000200.1	-	328405009	328404961
Sma-1-24263	NW_003037796.1	-	1042298	1042250
Sma-1-24264	NS_000200.1	-	9251942	9251894
Sma-1-24265	NW_003037932.1	+	128507	128555
Sma-1-24266	NS_000200.1	+	177661703	177661751
Sma-1-24267	NW_003037853.1	-	318041	317993
Sma-1-24268	NS_000200.1	-	95810092	95810044
Sma-1-24269	NW_003038517.1	+	71867	71915
Sma-1-24270	NS_000200.1	+	340836355	340836403
Sma-1-24271	NW_003037924.1	-	631961	631913
Sma-1-24272	NS_000200.1	-	172338919	172338871
Sma-1-24273	NW_003037805.1	+	1263142	1263190
Sma-1-24274	NS_000200.1	+	27820986	27821034
Sma-1-24275	NW_003037918.1	+	2042011	2042059
Sma-1-24276	NS_000200.1	+	168609001	168609049
Sma-1-24277	NW_003037917.1	-	550200	550152
Sma-1-24278	NS_000200.1	-	165587004	165586956
Sma-1-24279	NW_003037807.1	+	1479859	1479907
Sma-1-24280	NS_000200.1	+	31741375	31741423
Sma-1-24281	NW_003037792.1	-	445377	445329
Sma-1-24282	NS_000200.1	-	382882721	382882673
Sma-1-24283	NW_003037792.1	-	456949	456901
Sma-1-24284	NS_000200.1	-	382894293	382894245
Sma-1-24285	NW_003030424.1	+	1076	1124
Sma-1-24286	NS_000200.1	+	362871421	362871469
Sma-1-24287	NW_003026615.1	+	84724	84772
Sma-1-24288	NS_000200.1	+	317079165	317079213

Sma-1-24289	NW_003039127.1	+	579302	579350
Sma-1-24290	NS_000200.1	+	113656801	113656849
Sma-1-24291	NW_003038241.1	+	56932	56980
Sma-1-24292	NS_000200.1	+	314519637	314519685
Sma-1-24293	NW_003037910.1	-	1045099	1045051
Sma-1-24294	NS_000200.1	-	155557074	155557026
Sma-1-24295	NW_003037890.1	+	2091760	2091808
Sma-1-24296	NS_000200.1	+	137775121	137775169
Sma-1-24297	NW_003037904.1	-	110134	110086
Sma-1-24298	NS_000200.1	-	148824633	148824585
Sma-1-24299	NW_003037917.1	-	1275368	1275320
Sma-1-24300	NS_000200.1	-	166312172	166312124
Sma-1-24301	NW_003038102.1	-	35323	35275
Sma-1-24302	NS_000200.1	-	274714269	274714221
Sma-1-24303	NW_003037939.1	-	389571	389523
Sma-1-24304	NS_000200.1	-	183259325	183259277
Sma-1-24305	NW_003037808.1	+	940343	940391
Sma-1-24306	NS_000200.1	+	32726980	32727028
Sma-1-24307	NW_003037928.1	-	114822	114774
Sma-1-24308	NS_000200.1	-	174438775	174438727
Sma-1-24309	NW_003038261.1	+	298618	298666
Sma-1-24310	NS_000200.1	+	323912269	323912317
Sma-1-24311	NW_003020280.1	+	451	499
Sma-1-24312	NW_003038079.1	-	124775	124727
Sma-1-24313	NS_000200.1	+	371335409	371335457
Sma-1-24314	NS_000200.1	-	268254763	268254715
Sma-1-24315	NW_003026465.1	-	587236	587188
Sma-1-24316	NS_000200.1	-	207979830	207979782
Sma-1-24317	NW_003038518.1	-	932279	932231
Sma-1-24318	NS_000200.1	-	342569112	342569064
Sma-1-24319	NW_003037800.1	+	1892725	1892773
Sma-1-24320	NS_000200.1	+	20522085	20522133
Sma-1-24321	NW_003037925.1	+	335597	335645
Sma-1-24322	NS_000200.1	+	172737600	172737648
Sma-1-24323	NW_003037799.1	-	816903	816855
Sma-1-24324	NS_000200.1	-	17807257	17807209
Sma-1-24325	NW_003037911.1	-	217841	217793
Sma-1-24326	NS_000200.1	-	156005194	156005146
Sma-1-24327	NW_003038084.1	+	366363	366411
Sma-1-24328	NS_000200.1	+	271381607	271381655
Sma-1-24329	NW_003038029.1	-	237664	237616
Sma-1-24330	NS_000200.1	-	242438565	242438517
Sma-1-24331	NW_003029627.1	-	2800	2752
Sma-1-24332	NS_000200.1	-	366289629	366289581
Sma-1-24333	NW_003032336.1	+	150	198
Sma-1-24334	NW_003037802.1	-	532630	532582
Sma-1-24335	NS_000200.1	+	345912717	345912765
Sma-1-24336	NS_000200.1	-	22535424	22535376
Sma-1-24337	NW_003037809.1	-	2420758	2420710
Sma-1-24338	NW_003038145.1	-	343988	343940
Sma-1-24339	NS_000200.1	-	288955411	288955363
Sma-1-24340	NS_000200.1	-	36085222	36085174

Sma-1-24341	NW_003037916.1	-	1695060	1695012
Sma-1-24342	NS_000200.1	-	164888471	164888423
Sma-1-24343	NW_003037914.1	+	252538	252586
Sma-1-24344	NS_000200.1	+	160396788	160396836
Sma-1-24345	NW_003037815.1	-	302688	302640
Sma-1-24346	NS_000200.1	-	45565679	45565631
Sma-1-24347	NW_003037816.1	-	692719	692671
Sma-1-24348	NS_000200.1	-	46901591	46901543
Sma-1-24349	NW_003037914.1	+	1551824	1551872
Sma-1-24350	NS_000200.1	+	161696074	161696122
Sma-1-24351	NW_003037816.1	-	951454	951406
Sma-1-24352	NS_000200.1	-	47160326	47160278
Sma-1-24353	NW_003037824.1	+	703773	703821
Sma-1-24354	NS_000200.1	+	57634713	57634761
Sma-1-24355	NW_003037791.1	+	233356	233404
Sma-1-24356	NS_000200.1	+	381938784	381938832
Sma-1-24357	NW_003035424.1	+	389	437
Sma-1-24358	NS_000200.1	+	367443284	367443332
Sma-1-24359	NW_003032930.1	+	800	848
Sma-1-24360	NS_000200.1	+	326007774	326007822
Sma-1-24361	NW_003020608.1	-	1383	1335
Sma-1-24362	NW_003038137.1	-	138005	137957
Sma-1-24363	NS_000200.1	-	360418837	360418789
Sma-1-24364	NS_000200.1	-	284926901	284926853
Sma-1-24365	NW_003037861.1	-	623679	623631
Sma-1-24366	NS_000200.1	-	105400133	105400085
Sma-1-24367	NW_003038259.1	+	229510	229558
Sma-1-24368	NS_000200.1	+	323090109	323090157
Sma-1-24369	NW_003038037.1	+	1693902	1693950
Sma-1-24370	NS_000200.1	+	251849311	251849359
Sma-1-24371	NW_003026465.1	+	163268	163316
Sma-1-24372	NW_003037798.1	-	2673282	2673234
Sma-1-24373	NW_003037854.1	+	125992	126040
Sma-1-24374	NW_003037878.1	+	179621	179669
Sma-1-24375	NW_003038023.1	-	620393	620345
Sma-1-24376	NW_003038027.1	+	92226	92274
Sma-1-24377	NS_000200.1	+	97030413	97030461
Sma-1-24378	NS_000200.1	+	122233099	122233147
Sma-1-24379	NS_000200.1	+	207555862	207555910
Sma-1-24380	NS_000200.1	+	240462940	240462988
Sma-1-24381	NS_000200.1	-	237780684	237780636
Sma-1-24382	NS_000200.1	-	16932440	16932392
Sma-1-24383	NW_003037912.1	-	1712800	1712752
Sma-1-24384	NS_000200.1	-	158524375	158524327
Sma-1-24385	NW_003037841.1	-	2014354	2014306
Sma-1-24386	NS_000200.1	-	84625535	84625487
Sma-1-24387	NW_003037886.1	-	866799	866751
Sma-1-24388	NW_003037896.1	-	504233	504185
Sma-1-24389	NS_000200.1	-	143424656	143424608
Sma-1-24390	NS_000200.1	-	130122366	130122318
Sma-1-24391	NW_003037974.1	+	259986	260034
Sma-1-24392	NS_000200.1	+	204935292	204935340

Sma-1-24393	NW_003026512.1	+	181671	181719
Sma-1-24394	NW_003038081.1	+	89326	89374
Sma-1-24395	NS_000200.1	+	269251369	269251417
Sma-1-24396	NS_000200.1	+	285275711	285275759
Sma-1-24397	NW_003037777.1	-	80206	80158
Sma-1-24398	NW_003037799.1	+	983797	983845
Sma-1-24399	NW_003037903.1	-	431729	431681
Sma-1-24400	NW_003037905.1	-	386166	386118
Sma-1-24401	NS_000200.1	+	17974151	17974199
Sma-1-24402	NS_000200.1	-	378897992	378897944
Sma-1-24403	NS_000200.1	-	149655178	149655130
Sma-1-24404	NS_000200.1	-	147985810	147985762
Sma-1-24405	NW_003038514.1	-	179573	179525
Sma-1-24406	NW_003038009.1	-	184683	184635
Sma-1-24407	NW_003026456.1	-	667353	667305
Sma-1-24408	NW_003037810.1	-	545014	544966
Sma-1-24409	NW_003037813.1	-	1491896	1491848
Sma-1-24410	NW_003037816.1	-	755004	754956
Sma-1-24411	NW_003037839.1	-	995564	995516
Sma-1-24412	NW_003037882.1	+	218739	218787
Sma-1-24413	NW_003037911.1	-	980338	980290
Sma-1-24414	NW_003037935.1	-	902502	902454
Sma-1-24415	NW_003037945.1	-	1449876	1449828
Sma-1-24416	NW_003038034.1	-	588223	588175
Sma-1-24417	NW_003026517.1	+	82238	82286
Sma-1-24418	NW_003038145.1	-	2911	2863
Sma-1-24419	NS_000200.1	+	125510413	125510461
Sma-1-24420	NS_000200.1	+	299688247	299688295
Sma-1-24421	NS_000200.1	-	339804112	339804064
Sma-1-24422	NS_000200.1	-	288614334	288614286
Sma-1-24423	NS_000200.1	-	247529979	247529931
Sma-1-24424	NS_000200.1	-	226558746	226558698
Sma-1-24425	NS_000200.1	-	190551840	190551792
Sma-1-24426	NS_000200.1	-	180319037	180318989
Sma-1-24427	NS_000200.1	-	156767691	156767643
Sma-1-24428	NS_000200.1	-	79953714	79953666
Sma-1-24429	NS_000200.1	-	58590489	58590441
Sma-1-24430	NS_000200.1	-	46963876	46963828
Sma-1-24431	NS_000200.1	-	42478759	42478711
Sma-1-24432	NS_000200.1	-	37149536	37149488
Sma-1-24433	NW_003026465.1	+	725275	725323
Sma-1-24434	NS_000200.1	+	208117869	208117917
Sma-1-24435	NW_003039127.1	-	1476422	1476374
Sma-1-24436	NS_000200.1	-	114553921	114553873
Sma-1-24437	NW_003037797.1	-	3449967	3449919
Sma-1-24438	NS_000200.1	-	13877827	13877779
Sma-1-24439	NW_003038001.1	+	224909	224957
Sma-1-24440	NS_000200.1	+	222934470	222934518
Sma-1-24441	NW_003038252.1	-	345285	345237
Sma-1-24442	NS_000200.1	-	319200873	319200825
Sma-1-24443	NW_003030936.1	-	790	742
Sma-1-24444	NW_003037802.1	-	290064	290016

Sma-1-24445	NW_003037813.1	+	1302144	1302192
Sma-1-24446	NW_003037858.1	+	842068	842116
Sma-1-24447	NW_003037884.1	-	897452	897404
Sma-1-24448	NW_003037887.1	+	1660732	1660780
Sma-1-24449	NS_000200.1	+	42289007	42289055
Sma-1-24450	NS_000200.1	+	101506344	101506392
Sma-1-24451	NS_000200.1	+	132124710	132124758
Sma-1-24452	NS_000200.1	-	370555173	370555125
Sma-1-24453	NS_000200.1	-	127677117	127677069
Sma-1-24454	NS_000200.1	-	22292858	22292810
Sma-1-24455	NW_003037911.1	+	546968	547016
Sma-1-24456	NS_000200.1	+	156334321	156334369
Sma-1-24457	NW_003037889.1	-	1296344	1296296
Sma-1-24458	NS_000200.1	-	135072834	135072786
Sma-1-24459	NW_003037923.1	+	379208	379256
Sma-1-24460	NW_003026507.1	-	135084	135036
Sma-1-24461	NW_003038042.1	-	186700	186652
Sma-1-24462	NS_000200.1	+	171435421	171435469
Sma-1-24463	NS_000200.1	-	279795235	279795187
Sma-1-24464	NS_000200.1	-	254132299	254132251
Sma-1-24465	NW_003037910.1	-	75532	75484
Sma-1-24466	NS_000200.1	-	154587507	154587459
Sma-1-24467	NW_003037829.1	+	609368	609416
Sma-1-24468	NW_003037914.1	+	161023	161071
Sma-1-24469	NS_000200.1	+	66509906	66509954
Sma-1-24470	NS_000200.1	+	160305273	160305321
Sma-1-24471	NW_003028283.1	-	961	913
Sma-1-24472	NS_000200.1	-	374470433	374470385
Sma-1-24473	NW_003037974.1	-	217008	216960
Sma-1-24474	NS_000200.1	-	204892314	204892266
Sma-1-24475	NW_003037837.1	+	322691	322739
Sma-1-24476	NS_000200.1	+	77128549	77128597
Sma-1-24477	NW_003038033.1	-	730232	730184
Sma-1-24478	NS_000200.1	-	246710234	246710186
Sma-1-24479	NW_003037799.1	+	1519035	1519083
Sma-1-24480	NS_000200.1	+	18509389	18509437
Sma-1-24481	NW_003037791.1	+	527982	528030
Sma-1-24482	NS_000200.1	+	382233410	382233458
Sma-1-24483	NW_003037962.1	+	32446	32494
Sma-1-24484	NS_000200.1	+	196109289	196109337
Sma-1-24485	NW_003037825.1	+	581649	581697
Sma-1-24486	NW_003037916.1	-	115300	115252
Sma-1-24487	NS_000200.1	+	59711431	59711479
Sma-1-24488	NS_000200.1	-	163308711	163308663
Sma-1-24489	NW_003038083.1	+	689042	689090
Sma-1-24490	NS_000200.1	+	270932379	270932427
Sma-1-24491	NW_003037999.1	-	104575	104527
Sma-1-24492	NS_000200.1	-	221799129	221799081
Sma-1-24493	NW_003035519.1	+	860	908
Sma-1-24494	NW_003037793.1	+	2061343	2061391
Sma-1-24495	NW_003037798.1	-	1866891	1866843
Sma-1-24496	NW_003038059.1	+	85531	85579

Sma-1-24497	NS_000200.1	+	2061343	2061391
Sma-1-24498	NS_000200.1	+	260327603	260327651
Sma-1-24499	NS_000200.1	+	328331815	328331863
Sma-1-24500	NS_000200.1	-	16126049	16126001
Sma-1-24501	NW_003026458.1	-	688234	688186
Sma-1-24502	NS_000200.1	-	102556464	102556416
Sma-1-24503	NW_003038030.1	+	935365	935413
Sma-1-24504	NS_000200.1	+	244071232	244071280
Sma-1-24505	NW_003037851.1	-	405273	405225
Sma-1-24506	NS_000200.1	-	93808583	93808535
Sma-1-24507	NW_003037977.1	-	89067	89019
Sma-1-24508	NS_000200.1	-	208375087	208375039
Sma-1-24509	NW_003037904.1	-	124903	124855
Sma-1-24510	NS_000200.1	-	148839402	148839354
Sma-1-24511	NW_003038136.1	-	55019	54971
Sma-1-24512	NS_000200.1	-	284177088	284177040
Sma-1-24513	NW_003037865.1	-	294608	294560
Sma-1-24514	NW_003038227.1	-	90333	90285
Sma-1-24515	NS_000200.1	-	310371687	310371639
Sma-1-24516	NS_000200.1	-	110716527	110716479
Sma-1-24517	NW_003037872.1	+	520913	520961
Sma-1-24518	NS_000200.1	+	118477919	118477967
Sma-1-24519	NW_003037805.1	-	3280	3232
Sma-1-24520	NW_003038149.1	+	317658	317706
Sma-1-24521	NS_000200.1	+	291346271	291346319
Sma-1-24522	NS_000200.1	-	26561124	26561076
Sma-1-24523	NW_003038131.1	-	234974	234926
Sma-1-24524	NS_000200.1	-	282196818	282196770
Sma-1-24525	NW_003028775.1	+	164	212
Sma-1-24526	NS_000200.1	+	369914603	369914651
Sma-1-24527	NW_003037878.1	-	198943	198895
Sma-1-24528	NS_000200.1	-	122252421	122252373
Sma-1-24529	NW_003038517.1	+	106097	106145
Sma-1-24530	NS_000200.1	+	340870585	340870633
Sma-1-24531	NW_003028877.1	+	1592	1640
Sma-1-24532	NW_003037822.1	+	508470	508518
Sma-1-24533	NW_003037943.1	-	1192948	1192900
Sma-1-24534	NW_003037946.1	-	1132140	1132092
Sma-1-24535	NW_003038247.1	-	43559	43511
Sma-1-24536	NS_000200.1	+	54816476	54816524
Sma-1-24537	NS_000200.1	+	370135953	370136001
Sma-1-24538	NS_000200.1	-	315992287	315992239
Sma-1-24539	NS_000200.1	-	191826000	191825952
Sma-1-24540	NS_000200.1	-	188214481	188214433
Sma-1-24541	NW_003026611.1	-	253598	253550
Sma-1-24542	NS_000200.1	-	310823382	310823334
Sma-1-24543	NW_003037825.1	+	1022853	1022901
Sma-1-24544	NW_003037953.1	+	163171	163219
Sma-1-24545	NW_003037976.1	+	398800	398848
Sma-1-24546	NW_003038027.1	+	33822	33870
Sma-1-24547	NS_000200.1	+	60152635	60152683
Sma-1-24548	NS_000200.1	+	193076222	193076270

Sma-1-24549	NS_000200.1	+	207013115	207013163
Sma-1-24550	NS_000200.1	+	240404536	240404584
Sma-1-24551	NW_003038480.1	+	13501	13549
Sma-1-24552	NW_003037846.1	-	299968	299920
Sma-1-24553	NW_003037886.1	-	967268	967220
Sma-1-24554	NW_003037971.1	+	88223	88271
Sma-1-24555	NW_003038031.1	+	377653	377701
Sma-1-24556	NS_000200.1	+	201710273	201710321
Sma-1-24557	NS_000200.1	+	244655411	244655459
Sma-1-24558	NS_000200.1	+	332750064	332750112
Sma-1-24559	NS_000200.1	-	130222835	130222787
Sma-1-24560	NS_000200.1	-	87934538	87934490
Sma-1-24561	NW_003038161.1	+	416127	416175
Sma-1-24562	NS_000200.1	+	297785978	297786026
Sma-1-24563	NW_003038223.1	+	129986	130034
Sma-1-24564	NS_000200.1	+	309198420	309198468
Sma-1-24565	NW_003037939.1	-	634525	634477
Sma-1-24566	NS_000200.1	-	183504279	183504231
Sma-1-24567	NW_003037842.1	+	121766	121814
Sma-1-24568	NW_003037914.1	-	1475272	1475224
Sma-1-24569	NS_000200.1	+	85220956	85221004
Sma-1-24570	NS_000200.1	-	161619522	161619474
Sma-1-24571	NW_003037909.1	+	223376	223427
Sma-1-24572	NS_000200.1	+	153765025	153765076
Sma-1-24573	NW_003037825.1	-	345896	345848
Sma-1-24574	NS_000200.1	-	59475678	59475630
Sma-1-24575	NW_003038249.1	-	302403	302355
Sma-1-24576	NS_000200.1	-	316984656	316984608
Sma-1-24577	NW_003037863.1	-	1299309	1299261
Sma-1-24578	NW_003038251.1	-	106208	106160
Sma-1-24579	NS_000200.1	-	318437980	318437932
Sma-1-24580	NS_000200.1	-	108057676	108057628
Sma-1-24581	NW_003038511.1	-	168148	168100
Sma-1-24582	NW_003038067.1	-	150977	150929
Sma-1-24583	NS_000200.1	-	338513360	338513312
Sma-1-24584	NS_000200.1	-	263005030	263004982
Sma-1-24585	NW_003038037.1	+	590988	591036
Sma-1-24586	NS_000200.1	+	250746397	250746445
Sma-1-24587	NW_003038097.1	-	25093	25045
Sma-1-24588	NS_000200.1	-	274275395	274275347
Sma-1-24589	NW_003024383.1	-	1935	1887
Sma-1-24590	NW_003037591.1	+	4663	4711
Sma-1-24591	NW_003038231.1	+	180619	180667
Sma-1-24592	NW_003037934.1	-	299227	299179
Sma-1-24593	NW_003026458.1	-	166312	166264
Sma-1-24594	NW_003026619.1	+	340723	340771
Sma-1-24595	NW_003037799.1	-	392195	392147
Sma-1-24596	NW_003037802.1	-	313914	313866
Sma-1-24597	NW_003037807.1	+	1154875	1154923
Sma-1-24598	NW_003037827.1	-	1519597	1519549
Sma-1-24599	NW_003037829.1	+	676626	676674
Sma-1-24600	NW_003037831.1	+	251911	251959

Sma-1-24601	NW_003037836.1	+	825669	825717
Sma-1-24602	NW_003037838.1	+	553856	553904
Sma-1-24603	NW_003037847.1	+	185753	185801
Sma-1-24604	NW_003037848.1	-	537687	537639
Sma-1-24605	NW_003037853.1	+	518312	518360
Sma-1-24606	NW_003037872.1	-	470789	470741
Sma-1-24607	NW_003037891.1	-	912903	912855
Sma-1-24608	NW_003037911.1	+	951990	952038
Sma-1-24609	NW_003037916.1	-	908924	908876
Sma-1-24610	NW_003037958.1	-	321371	321323
Sma-1-24611	NW_003038033.1	-	919140	919092
Sma-1-24612	NW_003026507.1	-	103777	103729
Sma-1-24613	NW_003026509.1	-	49687	49639
Sma-1-24614	NW_003038053.1	+	578925	578973
Sma-1-24615	NW_003038053.1	-	578641	578593
Sma-1-24616	NW_003038083.1	-	80730	80682
Sma-1-24617	NW_003038126.1	+	285888	285936
Sma-1-24618	NW_003038129.1	-	246145	246097
Sma-1-24619	NW_003038148.1	+	510997	511045
Sma-1-24620	NW_003038148.1	+	513687	513735
Sma-1-24621	NW_003038161.1	+	554866	554914
Sma-1-24622	NW_003038192.1	+	13697	13745
Sma-1-24623	NS_000200.1	+	31416391	31416439
Sma-1-24624	NS_000200.1	+	66577164	66577212
Sma-1-24625	NS_000200.1	+	69701213	69701261
Sma-1-24626	NS_000200.1	+	76382768	76382816
Sma-1-24627	NS_000200.1	+	78552419	78552467
Sma-1-24628	NS_000200.1	+	88837483	88837531
Sma-1-24629	NS_000200.1	+	96010363	96010411
Sma-1-24630	NS_000200.1	+	156739343	156739391
Sma-1-24631	NS_000200.1	+	257939020	257939068
Sma-1-24632	NS_000200.1	+	280296300	280296348
Sma-1-24633	NS_000200.1	+	290584981	290585029
Sma-1-24634	NS_000200.1	+	290587671	290587719
Sma-1-24635	NS_000200.1	+	297924717	297924765
Sma-1-24636	NS_000200.1	+	302575604	302575652
Sma-1-24637	NS_000200.1	+	311788772	311788820
Sma-1-24638	NS_000200.1	+	322531467	322531515
Sma-1-24639	NS_000200.1	+	372705462	372705510
Sma-1-24640	NS_000200.1	-	365622921	365622873
Sma-1-24641	NS_000200.1	-	281549796	281549748
Sma-1-24642	NS_000200.1	-	281499492	281499444
Sma-1-24643	NS_000200.1	-	279763928	279763880
Sma-1-24644	NS_000200.1	-	270324067	270324019
Sma-1-24645	NS_000200.1	-	257938736	257938688
Sma-1-24646	NS_000200.1	-	246899142	246899094
Sma-1-24647	NS_000200.1	-	194985555	194985507
Sma-1-24648	NS_000200.1	-	179345974	179345926
Sma-1-24649	NS_000200.1	-	164102335	164102287
Sma-1-24650	NS_000200.1	-	138794181	138794133
Sma-1-24651	NS_000200.1	-	118427795	118427747
Sma-1-24652	NS_000200.1	-	102034542	102034494

Sma-1-24653	NS_000200.1	-	90230758	90230710
Sma-1-24654	NS_000200.1	-	64141738	64141690
Sma-1-24655	NS_000200.1	-	22316708	22316660
Sma-1-24656	NS_000200.1	-	17382549	17382501
Sma-1-24657	NW_003038011.1	-	289757	289709
Sma-1-24658	NS_000200.1	-	227817407	227817359
Sma-1-24659	NW_003037839.1	-	807896	807848
Sma-1-24660	NW_003037877.1	+	604857	604905
Sma-1-24661	NW_003026507.1	-	43440	43392
Sma-1-24662	NS_000200.1	+	121643419	121643467
Sma-1-24663	NS_000200.1	-	279703591	279703543
Sma-1-24664	NS_000200.1	-	79766046	79765998
Sma-1-24665	NW_003026616.1	+	315118	315166
Sma-1-24666	NS_000200.1	+	317671717	317671765
Sma-1-24667	NW_003034794.1	-	583	535
Sma-1-24668	NW_003026466.1	-	132955	132907
Sma-1-24669	NW_003038368.1	+	48124	48172
Sma-1-24670	NS_000200.1	+	327066066	327066114
Sma-1-24671	NS_000200.1	-	347003259	347003211
Sma-1-24672	NS_000200.1	-	215400062	215400014
Sma-1-24673	NW_003035682.1	+	33182	33230
Sma-1-24674	NS_000200.1	+	332655235	332655283
Sma-1-24675	NW_003037798.1	+	1249131	1249179
Sma-1-24676	NS_000200.1	+	15508289	15508337
Sma-1-24677	NW_003037916.1	+	1093692	1093740
Sma-1-24678	NS_000200.1	+	164287103	164287151
Sma-1-24679	NW_003038471.1	+	65966	66014
Sma-1-24680	NS_000200.1	+	331906880	331906928
Sma-1-24681	NW_003037854.1	+	1771812	1771860
Sma-1-24682	NS_000200.1	+	98676233	98676281
Sma-1-24683	NW_003037810.1	-	1275461	1275413
Sma-1-24684	NW_003037856.1	-	629240	629192
Sma-1-24685	NS_000200.1	-	99987196	99987148
Sma-1-24686	NS_000200.1	-	37879983	37879935
Sma-1-24687	NW_003037796.1	+	573003	573051
Sma-1-24688	NS_000200.1	+	8782647	8782695
Sma-1-24689	NW_003038152.1	+	336918	336966
Sma-1-24690	NS_000200.1	+	292942642	292942690
Sma-1-24691	NW_003038070.1	-	262982	262934
Sma-1-24692	NW_003037793.1	-	2076154	2076106
Sma-1-24693	NW_003037812.1	+	1005884	1005932
Sma-1-24694	NW_003038053.1	-	427527	427479
Sma-1-24695	NS_000200.1	+	40488623	40488671
Sma-1-24696	NS_000200.1	-	264600223	264600175
Sma-1-24697	NS_000200.1	-	257787622	257787574
Sma-1-24698	NS_000200.1	-	2076154	2076106
Sma-1-24699	NW_003038420.1	-	28819	28771
Sma-1-24700	NS_000200.1	-	328950850	328950802
Sma-1-24701	NW_003032868.1	-	242	194
Sma-1-24702	NW_003038044.1	+	24606	24654
Sma-1-24703	NS_000200.1	+	254649388	254649436
Sma-1-24704	NS_000200.1	-	354185712	354185664

Sma-1-24705	NW_003038221.1	+	69055	69103
Sma-1-24706	NS_000200.1	+	308710319	308710367
Sma-1-24707	NW_003037968.1	+	455197	455245
Sma-1-24708	NS_000200.1	+	200372367	200372415
Sma-1-24709	NW_003037979.1	+	460526	460574
Sma-1-24710	NS_000200.1	+	210521546	210521594
Sma-1-24711	NW_003037913.1	+	407240	407288
Sma-1-24712	NS_000200.1	+	158970991	158971039
Sma-1-24713	NW_003037913.1	+	283753	283801
Sma-1-24714	NS_000200.1	+	158847504	158847552
Sma-1-24715	NW_003037818.1	-	2160447	2160399
Sma-1-24716	NW_003037854.1	+	927221	927269
Sma-1-24717	NS_000200.1	+	97831642	97831690
Sma-1-24718	NS_000200.1	-	50875854	50875806
Sma-1-24719	NW_003038504.1	+	95585	95633
Sma-1-24720	NS_000200.1	+	335944117	335944165
Sma-1-24721	NW_003026508.1	-	30702	30654
Sma-1-24722	NW_003038045.1	+	34382	34430
Sma-1-24723	NS_000200.1	+	254811993	254812041
Sma-1-24724	NS_000200.1	-	281075316	281075268
Sma-1-24725	NW_003037939.1	+	374962	375010
Sma-1-24726	NW_003038078.1	+	97002	97050
Sma-1-24727	NS_000200.1	+	183244716	183244764
Sma-1-24728	NS_000200.1	+	267303205	267303253
Sma-1-24729	NW_003037830.1	-	419523	419475
Sma-1-24730	NS_000200.1	-	68309894	68309846
Sma-1-24731	NW_003037976.1	-	650697	650649
Sma-1-24732	NS_000200.1	-	207265012	207264964
Sma-1-24733	NW_003037878.1	-	743685	743636
Sma-1-24734	NS_000200.1	-	122797163	122797114
Sma-1-24735	NW_003036888.1	+	248	296
Sma-1-24736	NS_000200.1	+	347909310	347909358
Sma-1-24737	NW_003026472.1	-	161721	161673
Sma-1-24738	NS_000200.1	-	236573713	236573665
Sma-1-24739	NW_003037816.1	-	844234	844186
Sma-1-24740	NS_000200.1	-	47053106	47053058
Sma-1-24741	NW_003038030.1	+	354981	355029
Sma-1-24742	NS_000200.1	+	243490848	243490896
Sma-1-24743	NW_003023461.1	-	1631	1583
Sma-1-24744	NS_000200.1	-	373443669	373443621
Sma-1-24745	NW_003037841.1	+	184404	184452
Sma-1-24746	NS_000200.1	+	82795585	82795633
Sma-1-24747	NW_003037891.1	+	197630	197678
Sma-1-24748	NW_003037912.1	+	509457	509505
Sma-1-24749	NW_003038029.1	-	715749	715701
Sma-1-24750	NS_000200.1	+	138078908	138078956
Sma-1-24751	NS_000200.1	+	157321032	157321080
Sma-1-24752	NS_000200.1	-	242916650	242916602
Sma-1-24753	NW_003037851.1	-	563187	563139
Sma-1-24754	NS_000200.1	-	93966497	93966449
Sma-1-24755	NW_003038457.1	-	18182	18134
Sma-1-24756	NS_000200.1	-	331100046	331099998

Sma-1-24757	NW_003038119.1	-	139072	139024
Sma-1-24758	NS_000200.1	-	278046628	278046580
Sma-1-24759	NW_003037856.1	+	618048	618096
Sma-1-24760	NW_003037943.1	-	492021	491973
Sma-1-24761	NS_000200.1	+	99976004	99976052
Sma-1-24762	NS_000200.1	-	187513554	187513506
Sma-1-24763	NW_003037798.1	+	1351792	1351840
Sma-1-24764	NW_003037859.1	+	850064	850112
Sma-1-24765	NS_000200.1	+	15610950	15610998
Sma-1-24766	NS_000200.1	+	103528905	103528953
Sma-1-24767	NW_003037975.1	+	792656	792704
Sma-1-24768	NS_000200.1	+	206374880	206374928
Sma-1-24769	NW_003037810.1	+	162808	162856
Sma-1-24770	NS_000200.1	+	36767330	36767378
Sma-1-24771	NW_003037903.1	+	464086	464134
Sma-1-24772	NS_000200.1	+	148018167	148018215
Sma-1-24773	NW_003037797.1	+	3432953	3433001
Sma-1-24774	NS_000200.1	+	13860813	13860861
Sma-1-24775	NW_003029997.1	-	219	171
Sma-1-24776	NW_003038084.1	-	523503	523455
Sma-1-24777	NS_000200.1	-	357401459	357401411
Sma-1-24778	NS_000200.1	-	271538747	271538699
Sma-1-24779	NW_003037787.1	-	62898	62850
Sma-1-24780	NS_000200.1	-	380311067	380311019
Sma-1-24781	NW_003037847.1	-	996410	996362
Sma-1-24782	NS_000200.1	-	89648140	89648092
Sma-1-24783	NW_003037908.1	+	182358	182406
Sma-1-24784	NS_000200.1	+	152655489	152655537
Sma-1-24785	NW_003028987.1	+	66	114
Sma-1-24786	NW_003038474.1	-	82040	81992
Sma-1-24787	NS_000200.1	+	375075180	375075228
Sma-1-24788	NS_000200.1	-	332313254	332313206
Sma-1-24789	NW_003027666.1	-	923	875
Sma-1-24790	NW_003038037.1	-	46536	46488
Sma-1-24791	NS_000200.1	-	361828127	361828079
Sma-1-24792	NS_000200.1	-	250201945	250201897
Sma-1-24793	NW_003037854.1	-	510231	510183
Sma-1-24794	NS_000200.1	-	97414652	97414604
Sma-1-24795	NW_003037839.1	-	1225714	1225666
Sma-1-24796	NS_000200.1	-	80183864	80183816
Sma-1-24797	NW_003037827.1	-	582758	582710
Sma-1-24798	NS_000200.1	-	63204899	63204851
Sma-1-24799	NW_003038070.1	+	8786	8834
Sma-1-24800	NS_000200.1	+	264346027	264346075
Sma-1-24801	NW_003038148.1	-	158116	158068
Sma-1-24802	NS_000200.1	-	290232100	290232052
Sma-1-24803	NW_003037885.1	-	577299	577251
Sma-1-24804	NS_000200.1	-	128675246	128675198
Sma-1-24805	NW_003037790.1	+	72787	72835
Sma-1-24806	NS_000200.1	+	381416959	381417007
Sma-1-24807	NW_003037834.1	+	1063376	1063424
Sma-1-24808	NS_000200.1	+	74223862	74223910

Sma-1-24809	NW_003037859.1	+	734172	734220
Sma-1-24810	NS_000200.1	+	103413013	103413061
Sma-1-24811	NW_003038839.1	-	685	637
Sma-1-24812	NW_003038364.1	-	52	4
Sma-1-24813	NW_003037907.1	+	460509	460557
Sma-1-24814	NW_003037967.1	-	108356	108308
Sma-1-24815	NS_000200.1	+	151864998	151865046
Sma-1-24816	NS_000200.1	-	352560000	352559952
Sma-1-24817	NS_000200.1	-	326886349	326886301
Sma-1-24818	NS_000200.1	-	199348327	199348279
Sma-1-24819	NW_003038025.1	+	225539	225589
Sma-1-24820	NS_000200.1	+	239083784	239083834
Sma-1-24821	NW_003037886.1	-	383597	383549
Sma-1-24822	NS_000200.1	-	129639164	129639116
Sma-1-24823	NW_003037902.1	+	466906	466954
Sma-1-24824	NW_003037916.1	-	1624090	1624042
Sma-1-24825	NS_000200.1	+	147222140	147222188
Sma-1-24826	NS_000200.1	-	164817501	164817453
Sma-1-24827	NW_003038053.1	-	411280	411232
Sma-1-24828	NS_000200.1	-	257771375	257771327
Sma-1-24829	NW_003037885.1	-	509301	509253
Sma-1-24830	NS_000200.1	-	128607248	128607200
Sma-1-24831	NW_003037945.1	+	750612	750660
Sma-1-24832	NS_000200.1	+	189852576	189852624
Sma-1-24833	NW_003026342.1	-	701	653
Sma-1-24834	NW_003026805.1	-	102	54
Sma-1-24835	NW_003027824.1	-	945	897
Sma-1-24836	NW_003028092.1	-	518	470
Sma-1-24837	NW_003030968.1	+	633	681
Sma-1-24838	NW_003032158.1	-	24934	24886
Sma-1-24839	NW_003032158.1	-	15248	15200
Sma-1-24840	NW_003033658.1	+	41	89
Sma-1-24841	NW_003036382.1	-	3625	3577
Sma-1-24842	NW_003035680.1	+	32338	32386
Sma-1-24843	NW_003037794.1	-	1789088	1789040
Sma-1-24844	NW_003037794.1	-	1788422	1788374
Sma-1-24845	NW_003037798.1	+	2459146	2459194
Sma-1-24846	NW_003037806.1	-	1898113	1898065
Sma-1-24847	NW_003037818.1	+	2252170	2252218
Sma-1-24848	NW_003037827.1	-	1848719	1848671
Sma-1-24849	NW_003037829.1	-	1955931	1955883
Sma-1-24850	NW_003037831.1	-	1543080	1543032
Sma-1-24851	NW_003037848.1	+	658033	658081
Sma-1-24852	NW_003037854.1	-	443612	443564
Sma-1-24853	NW_003037870.1	+	230358	230406
Sma-1-24854	NW_003037880.1	-	465802	465754
Sma-1-24855	NW_003037885.1	-	805727	805679
Sma-1-24856	NW_003037913.1	+	233514	233562
Sma-1-24857	NW_003037961.1	-	159400	159352
Sma-1-24858	NW_003037968.1	+	201974	202022
Sma-1-24859	NW_003037972.1	+	413901	413949
Sma-1-24860	NW_003038015.1	-	619475	619427

Sma-1-24861	NW_003038033.1	-	284718	284670
Sma-1-24862	NW_003038515.1	+	390623	390671
Sma-1-24863	NW_003038518.1	+	649058	649106
Sma-1-24864	NW_003026485.1	-	145858	145810
Sma-1-24865	NW_003026490.1	-	143464	143416
Sma-1-24866	NW_003026511.1	+	339205	339253
Sma-1-24867	NW_003026514.1	-	115215	115167
Sma-1-24868	NW_003038059.1	+	53467	53515
Sma-1-24869	NW_003038152.1	+	101794	101842
Sma-1-24870	NW_003038153.1	+	657238	657286
Sma-1-24871	NW_003038153.1	+	691870	691918
Sma-1-24872	NS_000200.1	+	16718304	16718352
Sma-1-24873	NS_000200.1	+	50967577	50967625
Sma-1-24874	NS_000200.1	+	90351104	90351152
Sma-1-24875	NS_000200.1	+	117104023	117104071
Sma-1-24876	NS_000200.1	+	158797265	158797313
Sma-1-24877	NS_000200.1	+	200119144	200119192
Sma-1-24878	NS_000200.1	+	202889605	202889653
Sma-1-24879	NS_000200.1	+	260295539	260295587
Sma-1-24880	NS_000200.1	+	284722257	284722305
Sma-1-24881	NS_000200.1	+	292707518	292707566
Sma-1-24882	NS_000200.1	+	293719287	293719335
Sma-1-24883	NS_000200.1	+	293753919	293753967
Sma-1-24884	NS_000200.1	+	331975777	331975825
Sma-1-24885	NS_000200.1	+	340474688	340474736
Sma-1-24886	NS_000200.1	+	342285891	342285939
Sma-1-24887	NS_000200.1	+	354426439	354426487
Sma-1-24888	NS_000200.1	+	370628330	370628378
Sma-1-24889	NS_000200.1	-	380757213	380757165
Sma-1-24890	NS_000200.1	-	380747527	380747479
Sma-1-24891	NS_000200.1	-	369321348	369321300
Sma-1-24892	NS_000200.1	-	365989334	365989286
Sma-1-24893	NS_000200.1	-	359772370	359772322
Sma-1-24894	NS_000200.1	-	345234134	345234086
Sma-1-24895	NS_000200.1	-	344773068	344773020
Sma-1-24896	NS_000200.1	-	288573902	288573854
Sma-1-24897	NS_000200.1	-	267775180	267775132
Sma-1-24898	NS_000200.1	-	258500763	258500715
Sma-1-24899	NS_000200.1	-	246264720	246264672
Sma-1-24900	NS_000200.1	-	230942965	230942917
Sma-1-24901	NS_000200.1	-	195783642	195783594
Sma-1-24902	NS_000200.1	-	128903674	128903626
Sma-1-24903	NS_000200.1	-	124149360	124149312
Sma-1-24904	NS_000200.1	-	97348033	97347985
Sma-1-24905	NS_000200.1	-	70992382	70992334
Sma-1-24906	NS_000200.1	-	67856469	67856421
Sma-1-24907	NS_000200.1	-	64470860	64470812
Sma-1-24908	NS_000200.1	-	30093327	30093279
Sma-1-24909	NS_000200.1	-	5968683	5968635
Sma-1-24910	NS_000200.1	-	5968017	5967969
Sma-1-24911	NW_003028166.1	-	2394	2346
Sma-1-24912	NW_003037914.1	+	1036084	1036132

Sma-1-24913	NW_003038057.1	+	175604	175652
Sma-1-24914	NW_003038080.1	+	238116	238164
Sma-1-24915	NS_000200.1	+	161180334	161180382
Sma-1-24916	NS_000200.1	+	259381420	259381468
Sma-1-24917	NS_000200.1	+	268690726	268690774
Sma-1-24918	NS_000200.1	-	374191140	374191092
Sma-1-24919	NW_003037972.1	+	243879	243927
Sma-1-24920	NS_000200.1	+	202719583	202719631
Sma-1-24921	NW_003037854.1	-	13158	13110
Sma-1-24922	NS_000200.1	-	96917579	96917531
Sma-1-24923	NW_003037925.1	-	468086	468038
Sma-1-24924	NS_000200.1	-	172870089	172870041
Sma-1-24925	NW_003026599.1	+	128348	128396
Sma-1-24926	NS_000200.1	+	306006677	306006725
Sma-1-24927	NW_003037830.1	+	1137142	1137190
Sma-1-24928	NS_000200.1	+	69027513	69027561
Sma-1-24929	NW_003039127.1	-	1643105	1643057
Sma-1-24930	NS_000200.1	-	114720604	114720556
Sma-1-24931	NW_003025687.1	-	1485	1437
Sma-1-24932	NW_003037852.1	+	1050973	1051021
Sma-1-24933	NS_000200.1	+	95442868	95442916
Sma-1-24934	NS_000200.1	-	356361787	356361739
Sma-1-24935	NW_003037894.1	-	202175	202127
Sma-1-24936	NS_000200.1	-	142098748	142098700
Sma-1-24937	NW_003037891.1	-	2311652	2311604
Sma-1-24938	NS_000200.1	-	140192930	140192882
Sma-1-24939	NW_003037889.1	-	1716500	1716452
Sma-1-24940	NS_000200.1	-	135492990	135492942
Sma-1-24941	NW_003037793.1	-	826491	826443
Sma-1-24942	NW_003038066.1	-	382865	382817
Sma-1-24943	NS_000200.1	-	262851809	262851761
Sma-1-24944	NS_000200.1	-	826491	826443
Sma-1-24945	NW_003038032.1	-	526607	526559
Sma-1-24946	NS_000200.1	-	245685767	245685719
Sma-1-24947	NW_003037873.1	-	52201	52153
Sma-1-24948	NS_000200.1	-	118791329	118791281
env-93	AB231864.1	-	54792	54744
Sma-1-24949	NW_003020574.1	+	1440	1488
Sma-1-24950	NS_000200.1	+	360261886	360261934
Sma-1-24951	NW_003020796.1	+	455	503
Sma-1-24952	NW_003037793.1	+	426281	426329
Sma-1-24953	NW_003037804.1	+	947299	947347
Sma-1-24954	NW_003037810.1	+	774926	774974
Sma-1-24955	NW_003037810.1	-	770819	770771
Sma-1-24956	NW_003037825.1	+	74563	74611
Sma-1-24957	NW_003037838.1	-	289723	289675
Sma-1-24958	NW_003037852.1	-	764916	764868
Sma-1-24959	NW_003037891.1	+	2777015	2777063
Sma-1-24960	NW_003037906.1	-	494833	494785
Sma-1-24961	NW_003037971.1	-	253153	253105
Sma-1-24962	NW_003038014.1	-	384773	384725
Sma-1-24963	NW_003038026.1	-	271025	270977

Sma-1-24964	NW_003038033.1	-	817694	817646
Sma-1-24965	NW_003038518.1	+	872485	872533
Sma-1-24966	NW_003039127.1	+	2355479	2355527
Sma-1-24967	NW_003038056.1	-	108397	108349
Sma-1-24968	NW_003038057.1	-	197647	197599
Sma-1-24969	NW_003038083.1	-	170476	170428
Sma-1-24970	NW_003038155.1	+	360391	360439
Sma-1-24971	NS_000200.1	+	426281	426329
Sma-1-24972	NS_000200.1	+	26087319	26087367
Sma-1-24973	NS_000200.1	+	37379448	37379496
Sma-1-24974	NS_000200.1	+	59204345	59204393
Sma-1-24975	NS_000200.1	+	115432978	115433026
Sma-1-24976	NS_000200.1	+	140658293	140658341
Sma-1-24977	NS_000200.1	+	294483509	294483557
Sma-1-24978	NS_000200.1	+	342509318	342509366
Sma-1-24979	NS_000200.1	+	364475447	364475495
Sma-1-24980	NS_000200.1	-	270413813	270413765
Sma-1-24981	NS_000200.1	-	259403463	259403415
Sma-1-24982	NS_000200.1	-	259086048	259086000
Sma-1-24983	NS_000200.1	-	246797696	246797648
Sma-1-24984	NS_000200.1	-	239778786	239778738
Sma-1-24985	NS_000200.1	-	230125462	230125414
Sma-1-24986	NS_000200.1	-	201875203	201875155
Sma-1-24987	NS_000200.1	-	150802612	150802564
Sma-1-24988	NS_000200.1	-	95156811	95156763
Sma-1-24989	NS_000200.1	-	78288286	78288238
Sma-1-24990	NS_000200.1	-	37375341	37375293
Sma-1-24991	NW_003037875.1	+	480906	480954
Sma-1-24992	NS_000200.1	+	120509575	120509623
Sma-1-24993	NW_003037876.1	-	256515	256467
Sma-1-24994	NS_000200.1	-	120802151	120802103
Sma-1-24995	NW_003021162.1	+	187	235
Sma-1-24996	NS_000200.1	+	371493950	371493998
Sma-1-24997	NW_003023518.1	+	1982	2030
Sma-1-24998	NW_003037853.1	-	404534	404486
Sma-1-24999	NS_000200.1	+	373554700	373554748
Sma-1-25000	NS_000200.1	-	95896585	95896537
Sma-1-25001	NW_003037888.1	+	692072	692120
Sma-1-25002	NS_000200.1	+	132900564	132900612
Sma-1-25003	NW_003038083.1	+	641509	641557
Sma-1-25004	NW_003038158.1	+	454466	454514
Sma-1-25005	NS_000200.1	+	270884846	270884894
Sma-1-25006	NS_000200.1	+	296337634	296337682
Sma-1-25007	NW_003026488.1	+	116205	116253
Sma-1-25008	NS_000200.1	+	263622699	263622747
Sma-1-25009	NW_003037827.1	-	775046	774998
Sma-1-25010	NS_000200.1	-	63397187	63397139
Sma-1-25011	NW_003037794.1	-	1717108	1717060
Sma-1-25012	NS_000200.1	-	5896703	5896655
Sma-1-25013	NW_003037933.1	-	193328	193280
Sma-1-25014	NS_000200.1	-	178606500	178606452
Sma-1-25015	NW_003037915.1	-	421380	421332

Sma-1-25016	NS_000200.1	-	162321366	162321318
Sma-1-25017	NW_003037810.1	-	768351	768303
Sma-1-25018	NS_000200.1	-	37372873	37372825
Sma-1-25019	NW_003037994.1	+	293401	293449
Sma-1-25020	NS_000200.1	+	218825215	218825263
Sma-1-25021	NW_003037915.1	-	368033	367985
Sma-1-25022	NS_000200.1	-	162268019	162267971
Sma-1-25023	NW_003037793.1	+	918640	918688
Sma-1-25024	NW_003037812.1	-	1404215	1404167
Sma-1-25025	NS_000200.1	+	918640	918688
Sma-1-25026	NS_000200.1	-	40886954	40886906
Sma-1-25027	NW_003026465.1	+	786254	786305
Sma-1-25028	NS_000200.1	+	208178848	208178899
Sma-1-25029	NW_003038251.1	-	351705	351657
Sma-1-25030	NS_000200.1	-	318683477	318683429
Sma-1-25031	NW_003038086.1	-	412813	412765
Sma-1-25032	NS_000200.1	-	272661406	272661358
Sma-1-25033	NW_003037939.1	-	84708	84660
Sma-1-25034	NS_000200.1	-	182954462	182954414
Sma-1-25035	NW_003037975.1	-	944818	944770
Sma-1-25036	NS_000200.1	-	206527042	206526994
Sma-1-25037	NW_003037935.1	-	679931	679883
Sma-1-25038	NS_000200.1	-	180096466	180096418
Sma-1-25039	NW_003037791.1	-	142679	142631
Sma-1-25040	NS_000200.1	-	381848107	381848059
Sma-1-25041	NW_003037962.1	-	13025	12977
Sma-1-25042	NS_000200.1	-	196089868	196089820
Sma-1-25043	NW_003031319.1	-	1035	987
Sma-1-25044	NS_000200.1	-	345744682	345744634
Sma-1-25045	NW_003037844.1	+	580417	580465
Sma-1-25046	NW_003038032.1	+	736461	736509
Sma-1-25047	NW_003038037.1	+	761456	761504
Sma-1-25048	NS_000200.1	+	86763863	86763911
Sma-1-25049	NS_000200.1	+	245895621	245895669
Sma-1-25050	NS_000200.1	+	250916865	250916913
Sma-1-25051	NW_003037891.1	-	1349893	1349845
Sma-1-25052	NS_000200.1	-	139231171	139231123
Sma-1-25053	NW_003037852.1	-	1070887	1070839
Sma-1-25054	NS_000200.1	-	95462782	95462734
Sma-1-25055	NW_003038135.1	+	28543	28591
Sma-1-25056	NS_000200.1	+	283900882	283900930
Sma-1-25057	NW_003037908.1	-	1000661	1000613
Sma-1-25058	NS_000200.1	-	153473792	153473744
Sma-1-25059	NW_003037888.1	+	622483	622531
Sma-1-25060	NS_000200.1	+	132830975	132831023
Sma-1-25061	NW_003037863.1	+	1322944	1322992
Sma-1-25062	NS_000200.1	+	108081311	108081359
Sma-1-25063	NW_003038227.1	-	24507	24459
Sma-1-25064	NS_000200.1	-	310305861	310305813
Sma-1-25065	NW_003038158.1	+	56755	56803
Sma-1-25066	NS_000200.1	+	295939923	295939971
Sma-1-25067	NW_003026469.1	-	23024	22976

Sma-1-25068	NW_003037800.1	+	42214	42262
Sma-1-25069	NW_003037800.1	+	2061108	2061156
Sma-1-25070	NS_000200.1	+	18671574	18671622
Sma-1-25071	NS_000200.1	+	20690468	20690516
Sma-1-25072	NS_000200.1	-	217644683	217644635
Sma-1-25073	NW_003037853.1	-	625901	625853
Sma-1-25074	NS_000200.1	-	96117952	96117904
Sma-1-25075	NW_003037992.1	-	197976	197928
Sma-1-25076	NS_000200.1	-	218039457	218039409
Sma-1-25077	NW_003031752.1	+	252	300
Sma-1-25078	NW_003037838.1	-	664126	664078
Sma-1-25079	NS_000200.1	+	366945215	366945263
Sma-1-25080	NS_000200.1	-	78662689	78662641
Sma-1-25081	NW_003037838.1	+	642209	642257
Sma-1-25082	NS_000200.1	+	78640772	78640820
Sma-1-25083	NW_003037896.1	-	153469	153421
Sma-1-25084	NS_000200.1	-	143073892	143073844
Sma-1-25085	NW_003020356.1	+	312	360
Sma-1-25086	NW_003020356.1	+	1489	1537
Sma-1-25087	NW_003037039.1	-	714	666
Sma-1-25088	NW_003037944.1	-	56430	56382
Sma-1-25089	NS_000200.1	+	354962711	354962759
Sma-1-25090	NS_000200.1	+	354963888	354963936
Sma-1-25091	NS_000200.1	-	343714855	343714807
Sma-1-25092	NS_000200.1	-	188277783	188277735
Sma-1-25093	NW_003026616.1	+	167739	167787
Sma-1-25094	NS_000200.1	+	317524338	317524386
Sma-1-25095	NW_003037771.1	-	128660	128612
Sma-1-25096	NS_000200.1	-	378429566	378429518
Sma-1-25097	NW_003026611.1	-	149248	149200
Sma-1-25098	NS_000200.1	-	310719032	310718984
Sma-1-25099	NW_003037827.1	+	1139189	1139237
Sma-1-25100	NS_000200.1	+	63761330	63761378
Sma-1-25101	NW_003037886.1	+	777320	777368
Sma-1-25102	NS_000200.1	+	130032887	130032935
Sma-1-25103	NW_003026472.1	+	79236	79284
Sma-1-25104	NW_003037902.1	+	28946	28994
Sma-1-25105	NS_000200.1	+	146784180	146784228
Sma-1-25106	NS_000200.1	+	236491228	236491276
Sma-1-25107	NW_003026516.1	-	140601	140553
Sma-1-25108	NS_000200.1	-	299248898	299248850
Sma-1-25109	NW_003038161.1	-	96664	96616
Sma-1-25110	NS_000200.1	-	297466515	297466467
Sma-1-25111	NW_003038455.1	+	10706	10754
Sma-1-25112	NS_000200.1	+	330792757	330792805
Sma-1-25113	NW_003030280.1	+	153	201
Sma-1-25114	NW_003038511.1	-	102300	102252
Sma-1-25115	NW_003026471.1	-	117077	117029
Sma-1-25116	NW_003038000.1	+	70568	70616
Sma-1-25117	NW_003026465.1	+	656763	656811
Sma-1-25118	NW_003035688.1	+	25996	26044
Sma-1-25119	NW_003037799.1	-	1524131	1524083

Sma-1-25120	NW_003037799.1	-	636011	635963
Sma-1-25121	NW_003037814.1	-	1877075	1877027
Sma-1-25122	NW_003037823.1	+	993668	993716
Sma-1-25123	NW_003037838.1	-	547093	547045
Sma-1-25124	NW_003037839.1	-	708704	708656
Sma-1-25125	NW_003037841.1	+	298795	298843
Sma-1-25126	NW_003037857.1	-	140149	140101
Sma-1-25127	NW_003037859.1	+	840640	840688
Sma-1-25128	NW_003037881.1	-	467439	467391
Sma-1-25129	NW_003037942.1	-	294928	294880
Sma-1-25130	NW_003038018.1	+	334743	334791
Sma-1-25131	NW_003038027.1	+	142348	142396
Sma-1-25132	NW_003038518.1	+	62879	62927
Sma-1-25133	NW_003038518.1	+	136853	136901
Sma-1-25134	NW_003026517.1	-	620724	620676
Sma-1-25135	NW_003038083.1	+	206776	206824
Sma-1-25136	NW_003038084.1	+	175822	175870
Sma-1-25137	NW_003038084.1	+	584174	584222
Sma-1-25138	NW_003038091.1	+	4138	4186
Sma-1-25139	NW_003038128.1	+	73664	73712
Sma-1-25140	NW_003038161.1	-	356415	356367
Sma-1-25141	NS_000200.1	+	56910287	56910335
Sma-1-25142	NS_000200.1	+	82909976	82910024
Sma-1-25143	NS_000200.1	+	103519481	103519529
Sma-1-25144	NS_000200.1	+	208049357	208049405
Sma-1-25145	NS_000200.1	+	222541346	222541394
Sma-1-25146	NS_000200.1	+	232735593	232735641
Sma-1-25147	NS_000200.1	+	240513062	240513110
Sma-1-25148	NS_000200.1	+	270450113	270450161
Sma-1-25149	NS_000200.1	+	271191066	271191114
Sma-1-25150	NS_000200.1	+	271599418	271599466
Sma-1-25151	NS_000200.1	+	273943697	273943745
Sma-1-25152	NS_000200.1	+	280791952	280792000
Sma-1-25153	NS_000200.1	+	332928474	332928522
Sma-1-25154	NS_000200.1	+	341699712	341699760
Sma-1-25155	NS_000200.1	+	341773686	341773734
Sma-1-25156	NS_000200.1	+	362566846	362566894
Sma-1-25157	NS_000200.1	-	338447512	338447464
Sma-1-25158	NS_000200.1	-	300226733	300226685
Sma-1-25159	NS_000200.1	-	297726266	297726218
Sma-1-25160	NS_000200.1	-	222229310	222229262
Sma-1-25161	NS_000200.1	-	186054836	186054788
Sma-1-25162	NS_000200.1	-	124899230	124899182
Sma-1-25163	NS_000200.1	-	100160506	100160458
Sma-1-25164	NS_000200.1	-	79666854	79666806
Sma-1-25165	NS_000200.1	-	78545656	78545608
Sma-1-25166	NS_000200.1	-	44860935	44860887
Sma-1-25167	NS_000200.1	-	18514485	18514437
Sma-1-25168	NS_000200.1	-	17626365	17626317
Sma-1-25169	NW_003027990.1	-	788	740
Sma-1-25170	NW_003037830.1	-	245175	245127
Sma-1-25171	NS_000200.1	-	369125906	369125858

Sma-1-25172	NS_000200.1	-	68135546	68135498
Sma-1-25173	NW_003037962.1	-	536946	536898
Sma-1-25174	NS_000200.1	-	196613789	196613741
Sma-1-25175	NW_003037937.1	-	315184	315136
Sma-1-25176	NS_000200.1	-	181515093	181515045
Sma-1-25177	NW_003037794.1	+	685104	685152
Sma-1-25178	NS_000200.1	+	4864699	4864747
Sma-1-25179	NW_003037779.1	-	45088	45040
Sma-1-25180	NW_003037854.1	-	77986	77938
Sma-1-25181	NW_003037878.1	-	157171	157123
Sma-1-25182	NS_000200.1	-	378996250	378996202
Sma-1-25183	NS_000200.1	-	122210649	122210601
Sma-1-25184	NS_000200.1	-	96982407	96982359
Sma-1-25185	NW_003026466.1	+	59418	59466
Sma-1-25186	NS_000200.1	+	215326525	215326573
Sma-1-25187	NW_003037890.1	+	1966631	1966679
Sma-1-25188	NS_000200.1	+	137649992	137650040
Sma-1-25189	NW_003037858.1	-	487908	487860
Sma-1-25190	NS_000200.1	-	101152184	101152136
Sma-1-25191	NW_003030461.1	+	1493	1541
Sma-1-25192	NS_000200.1	+	362959102	362959150
Sma-1-25193	NW_003037797.1	-	2649410	2649362
Sma-1-25194	NS_000200.1	-	13077270	13077222
Sma-1-25195	NW_003036241.1	+	1741	1789
Sma-1-25196	NS_000200.1	+	347591435	347591483
Sma-1-25197	NW_003037943.1	+	748164	748212
Sma-1-25198	NS_000200.1	+	187769697	187769745
Sma-1-25199	NW_003037823.1	-	678211	678163
Sma-1-25200	NS_000200.1	-	56594830	56594782
Sma-1-25201	NW_003026458.1	+	379222	379270
Sma-1-25202	NS_000200.1	+	102247452	102247500
Sma-1-25203	NW_003037894.1	+	382565	382613
Sma-1-25204	NS_000200.1	+	142279138	142279186
Sma-1-25205	NW_003037891.1	+	642918	642966
Sma-1-25206	NS_000200.1	+	138524196	138524244
Sma-1-25207	NW_003037891.1	-	2801088	2801040
Sma-1-25208	NS_000200.1	-	140682366	140682318
Sma-1-25209	NW_003032675.1	-	857	809
Sma-1-25210	NW_003037792.1	+	455340	455388
Sma-1-25211	NW_003037792.1	-	20134	20086
Sma-1-25212	NW_003037822.1	-	1366759	1366711
Sma-1-25213	NW_003037825.1	+	1375635	1375683
Sma-1-25214	NW_003037840.1	-	629318	629270
Sma-1-25215	NW_003038086.1	-	696251	696203
Sma-1-25216	NS_000200.1	+	60505417	60505465
Sma-1-25217	NS_000200.1	+	382892684	382892732
Sma-1-25218	NS_000200.1	-	382457478	382457430
Sma-1-25219	NS_000200.1	-	350599895	350599847
Sma-1-25220	NS_000200.1	-	272944844	272944796
Sma-1-25221	NS_000200.1	-	81252642	81252594
Sma-1-25222	NS_000200.1	-	55674765	55674717
Sma-1-25223	NW_003031783.1	-	74	26

Sma-1-25224	NS_000200.1	-	367005282	367005234
Sma-1-25225	NW_003037890.1	+	2187189	2187237
Sma-1-25226	NS_000200.1	+	137870550	137870598
Sma-1-25227	NW_003026512.1	+	246790	246838
Sma-1-25228	NS_000200.1	+	285340830	285340878
Sma-1-25229	NW_003035818.1	+	2001	2049
Sma-1-25230	NW_003037884.1	-	699040	698992
Sma-1-25231	NS_000200.1	+	364157428	364157476
Sma-1-25232	NS_000200.1	-	127478705	127478657
Sma-1-25233	NW_003037918.1	-	2034692	2034644
Sma-1-25234	NS_000200.1	-	168601682	168601634
Sma-1-25235	NW_003035475.1	-	486	438
Sma-1-25236	NW_003037919.1	+	225885	225933
Sma-1-25237	NS_000200.1	+	169083814	169083862
Sma-1-25238	NS_000200.1	-	367529107	367529059
Sma-1-25239	NW_003037829.1	+	249581	249629
Sma-1-25240	NS_000200.1	+	66150119	66150167
Sma-1-25241	NW_003038137.1	+	282204	282252
Sma-1-25242	NS_000200.1	+	285071100	285071148
Sma-1-25243	NW_003026609.1	+	164945	164993
Sma-1-25244	NS_000200.1	+	309819937	309819985
Sma-1-25245	NW_003038024.1	+	206146	206194
Sma-1-25246	NS_000200.1	+	238103175	238103223
Sma-1-25247	NW_003037980.1	+	1152861	1152909
Sma-1-25248	NS_000200.1	+	212113723	212113771
Sma-1-25249	NW_003037911.1	+	723790	723838
Sma-1-25250	NS_000200.1	+	156511143	156511191
Sma-1-25251	NW_003034807.1	+	471	519
Sma-1-25252	NW_003037831.1	-	360840	360792
Sma-1-25253	NW_003037880.1	+	343220	343268
Sma-1-25254	NS_000200.1	+	124026778	124026826
Sma-1-25255	NS_000200.1	+	347019908	347019956
Sma-1-25256	NS_000200.1	-	69810142	69810094
Sma-1-25257	NW_003037806.1	+	1257345	1257393
Sma-1-25258	NW_003038515.1	+	403155	403203
Sma-1-25259	NS_000200.1	+	29452559	29452607
Sma-1-25260	NS_000200.1	+	340487220	340487268
Sma-1-25261	NW_003037921.1	+	71754	71802
Sma-1-25262	NS_000200.1	+	169601889	169601937
Sma-1-25263	NW_003038014.1	+	84104	84152
Sma-1-25264	NW_003039127.1	+	1542514	1542562
Sma-1-25265	NS_000200.1	+	114620013	114620061
Sma-1-25266	NS_000200.1	+	229824793	229824841
Sma-1-25267	NW_003037973.1	+	563305	563353
Sma-1-25268	NS_000200.1	+	204545032	204545080
Sma-1-25269	NW_003037887.1	-	267745	267698
Sma-1-25270	NS_000200.1	-	130731723	130731676
Sma-1-25271	NW_003038242.1	-	191260	191213
Sma-1-25272	NS_000200.1	-	314890406	314890359
Sma-1-25273	NW_003025230.1	+	1042	1089
Sma-1-25274	NS_000200.1	+	361440300	361440347
Sma-1-25275	NW_003038237.1	+	163075	163123

Sma-1-25276	NS_000200.1	+	313602994	313603042
Sma-1-25277	NW_003039127.1	+	2112991	2113039
Sma-1-25278	NW_003039127.1	-	2116540	2116492
Sma-1-25279	NS_000200.1	+	115190490	115190538
Sma-1-25280	NS_000200.1	-	115194039	115193991
env-94	AF036402.1	+	123	167
env-95	AF036399.1	+	123	167
env-96	AF036404.1	+	122	166
env-97	AF036400.1	+	123	167
Sma-1-25281	NW_003037809.1	-	2492623	2492572
Sma-1-25282	NS_000200.1	-	36157087	36157036
Sma-1-25283	NW_003038010.1	-	165664	165613
Sma-1-25284	NS_000200.1	-	226983887	226983836
Sma-1-25285	NW_003037881.1	-	437681	437630
Sma-1-25286	NS_000200.1	-	124869472	124869421
Sma-1-25287	NW_003037841.1	+	1315978	1316029
Sma-1-25288	NS_000200.1	+	83927159	83927210
Sma-1-25289	NW_003037940.1	-	57779	57728
Sma-1-25290	NS_000200.1	-	183799909	183799858
Sma-1-25291	NW_003037783.1	+	30556	30607
Sma-1-25292	NS_000200.1	+	379764160	379764211
Sma-1-25293	NW_003037829.1	+	1117717	1117766
Sma-1-25294	NS_000200.1	+	67018255	67018304
Sma-1-25295	NW_003037791.1	+	147585	147634
Sma-1-25296	NS_000200.1	+	381853013	381853062
Sma-1-25297	NW_003038133.1	+	36308	36357
Sma-1-25298	NS_000200.1	+	282908490	282908539
Sma-1-25299	NW_003037827.1	+	1423833	1423881
Sma-1-25300	NS_000200.1	+	64045974	64046022
Sma-1-25301	NW_003037797.1	+	42429	42477
Sma-1-25302	NS_000200.1	+	10470289	10470337
Sma-1-25303	NW_003038026.1	-	554048	554001
Sma-1-25304	NS_000200.1	-	240061809	240061762
Sma-1-25305	NW_003037826.1	+	1159507	1159554
Sma-1-25306	NS_000200.1	+	62159023	62159070
Sma-1-25307	NW_003037852.1	-	626159	626112
Sma-1-25308	NS_000200.1	-	95018054	95018007
Sma-1-25309	NW_003037884.1	+	861586	861632
Sma-1-25310	NS_000200.1	+	127641251	127641297
Sma-1-25311	NW_003038002.1	+	10203	10249
Sma-1-25312	NS_000200.1	+	223130412	223130458
Sma-1-25313	NW_003038251.1	-	92092	92041
Sma-1-25314	NS_000200.1	-	318423864	318423813
Sma-1-25315	NW_003038149.1	-	422787	422736
Sma-1-25316	NS_000200.1	-	291451400	291451349
Sma-1-25317	NW_003037796.1	-	2006571	2006521
Sma-1-25318	NS_000200.1	-	10216215	10216165
Sma-1-25319	NW_003037906.1	-	470462	470413
Sma-1-25320	NS_000200.1	-	150778241	150778192
Sma-1-25321	NW_003038032.1	+	682795	682844
Sma-1-25322	NS_000200.1	+	245841955	245842004
Sma-1-25323	NW_003037918.1	-	140067	140018

Sma-1-25324	NS_000200.1	-	166707057	166707008
Sma-1-25325	NW_003037918.1	+	131685	131734
Sma-1-25326	NS_000200.1	+	166698675	166698724
Sma-1-25327	NW_003037839.1	-	1503313	1503264
Sma-1-25328	NS_000200.1	-	80461463	80461414
Sma-1-25329	NW_003038075.1	+	82	131
Sma-1-25330	NS_000200.1	+	266118293	266118342
Sma-1-25331	NW_003037791.1	-	432898	432847
Sma-1-25332	NS_000200.1	-	382138326	382138275
Sma-1-25333	NW_003037873.1	+	14522	14569
Sma-1-25334	NS_000200.1	+	118753650	118753697
Sma-1-25335	NW_003038148.1	-	519979	519931
Sma-1-25336	NS_000200.1	-	290593963	290593915
Sma-1-25337	NW_003037888.1	-	41794	41745
Sma-1-25338	NS_000200.1	-	132250286	132250237
Sma-1-25339	NW_003037930.1	-	780769	780721
Sma-1-25340	NS_000200.1	-	176638733	176638685
Sma-1-25341	NW_003037807.1	-	417071	417025
Sma-1-25342	NS_000200.1	-	30678587	30678541
env-98	AF036398.1	+	58	102
Sma-1-25343	NW_003038124.1	+	231681	231729
Sma-1-25344	NS_000200.1	+	279224325	279224373
Sma-1-25345	NW_003037838.1	-	334342	334279
Sma-1-25346	NS_000200.1	-	78332905	78332842
Sma-1-25347	NW_003038238.1	+	96992	97069
Sma-1-25348	NS_000200.1	+	313703773	313703850
Nve-1-472	NW_001834397.1	+	1045121	1045175
env-99	JCVI_SCAF_1096627289312	+	327	451
env-100	JCVI_SCAF_1096627755477	-	1322	1276
env-101	JCVI_SCAF_1096628364820	-	116	70
env-102	NCBI_BBAY_READ_1106105143237	-	939	884
env-103	JCVI_SCAF_1096627869476	-	1185	1130
env-104	JCVI_SCAF_1101668008592	-	195	142
env-105	JCVI_SCAF_1101668008592	-	1148	1095
env-106	JCVI_SCAF_1101668008592	-	781	728
env-107	ALVINELLA_READ_1878650945	+	36	82
env-108	ALVINELLA_READ_1878688557	+	601	646
env-109	ALVINELLA_READ_1878673005	-	379	332
env-110	ALVINELLA_READ_1878573841	+	389	435
env-111	ALVINELLA_READ_1878554883	+	46	92
env-112	ALVINELLA_READ_1878617291	+	441	487
env-113	ALVINELLA_READ_1878651869	+	488	534
env-114	ALVINELLA_READ_1878678796	+	496	542
env-115	ALVINELLA_READ_1878518601	+	234	280
env-116	ALVINELLA_READ_1878691276	+	217	263
env-117	ALVINELLA_READ_1878443912	+	86	132
env-118	ALVINELLA_READ_1878701031	+	336	382
env-119	ALVINELLA_READ_1878600291	+	38	84
env-120	ALVINELLA_READ_1878665302	+	672	720
env-121	NCBI_BBAY_READ_1106850418211	-	745	697
Tca-1-14	NW_001094318.1	+	62683	62728
env-122	AAMC01022016.1	-	20797	20756

env-123	AAMC01032538.1	+	14077	14119
env-124	AAMC01119286.1	-	24154	24113
env-125	AAMC01105789.1	-	1966	1925
env-126	AAMC01064751.1	+	7282	7323
env-127	AAMC01150818.1	-	1193	1151
env-128	AAMC01112992.1	-	5427	5385
env-129	AAMC01106794.1	-	13377	13335
env-130	AAMC01060971.1	+	4170	4212
env-131	AAMC01045075.1	+	21328	21370
env-132	AAMC01044369.1	-	25956	25914
env-133	AAMC01038390.1	+	287	329
env-134	AAMC01038390.1	+	553	595
env-135	AAMC01024578.1	-	11624	11582
env-136	AAMC01013308.1	-	3326	3284
env-137	AAMC01013199.1	-	3768	3726
env-138	AAMC01008676.1	+	20113	20155
env-139	AAMC01006953.1	+	42502	42544
env-140	AAMC01044441.1	+	13006	13048
env-141	AAMC01010361.1	-	3439	3397
env-142	AAMC01097028.1	-	13512	13472
env-143	AAMC01039721.1	-	485	445
env-144	AAMC01091277.1	-	7121	7081
env-145	AAMC01145184.1	-	1094	1053
env-146	AAMC01134896.1	+	6769	6810
env-147	AAMC01022403.1	+	21798	21839
env-148	AAMC01131716.1	+	1789	1831
env-149	AAMC01128868.1	+	3353	3394
env-150	AAMC01118869.1	+	387	428
env-151	AAMC01113202.1	+	61	102
env-152	AAMC01105789.1	-	2539	2498
env-153	AAMC01102498.1	-	10532	10491
env-154	AAMC01030396.1	+	3010	3051
env-155	AAMC01085569.1	-	10976	10935
env-156	AAMC01115073.1	-	4234	4193
env-157	AAMC01097034.1	+	26267	26308
env-158	AAMC01097034.1	-	26101	26060
env-159	AAMC01099511.1	-	12719	12678
env-160	AAMC01097952.1	-	17828	17787
env-161	AAMC01027799.1	-	13352	13311
env-162	AAMC01099526.1	-	28471	28430
env-163	AAMC01092595.1	+	6709	6750
env-164	AAMC01084679.1	+	25725	25766
env-165	AAMC01000412.1	-	66937	66895
env-166	AAMC01000504.1	+	688	730
env-167	AAMC01011783.1	-	3908	3867
env-168	AAMC01050768.1	-	8078	8037
env-169	AAMC01080264.1	+	37272	37313
env-170	AAMC01065649.1	+	58	99
env-171	AAMC01065648.1	-	4859	4818
env-172	AAMC01064830.1	-	6994	6953
env-173	AAMC01008353.1	+	27216	27258
env-174	AAMC01084329.1	-	8995	8954

env-175	AAMC01048972.1	+	5091	5132
env-176	AAMC01039278.1	+	10815	10856
env-177	AAMC01033430.1	-	326	285
env-178	AAMC01026504.1	+	1352	1393
env-179	AAMC01025780.1	-	14715	14673
env-180	AAMC01018534.1	-	4889	4848
env-181	AAMC01056829.1	-	6375	6334
env-182	AAMC01015602.1	-	7055	7014
env-183	AAMC01156781.1	+	3386	3427
env-184	AAMC01004340.1	+	11370	11411
env-185	AAMC01003204.1	-	23629	23588
env-186	AAMC01115196.1	+	2142	2184
env-187	AAMC01140161.1	-	848	806
env-188	AAMC01167915.1	-	3977	3935
env-189	AAMC01190753.1	-	1009	967
env-190	AAMC01141856.1	-	2126	2084
env-191	AAMC01140629.1	-	1209	1167
env-192	AAMC01140629.1	-	668	626
env-193	AAMC01140629.1	-	127	85
env-194	AAMC01104645.1	-	10624	10582
env-195	AAMC01098835.1	+	33279	33321
env-196	AAMC01098838.1	+	149	191
env-197	AAMC01080061.1	+	6242	6284
env-198	AAMC01077408.1	-	2995	2953
env-199	AAMC01060953.1	-	351	309
env-200	AAMC01010885.1	-	9121	9079
env-201	AAMC01054043.1	+	846	888
env-202	AAMC01017092.1	-	18685	18643
env-203	AAMC01035689.1	+	8913	8955
env-204	AAMC01104142.1	-	2836	2794
env-205	AAMC01017643.1	-	1330	1288
env-206	AAMC01008982.1	+	1966	2008
env-207	AAMC01001723.1	+	18051	18093
env-208	AAMC01083573.1	-	12551	12509
env-209	AAMC01095194.1	+	27605	27647
env-210	AAMC01000594.1	-	2276	2234
env-211	AAMC01004780.1	-	18146	18104
env-212	AAMC01011179.1	-	13402	13360
env-213	AAMC01011179.1	-	13139	13097
env-214	AAMC01014574.1	-	2096	2054
env-215	AAMC01020983.1	-	2560	2518
env-216	AAMC01036569.1	+	11274	11316
env-217	AAMC01041125.1	-	27002	26960
env-218	AAMC01042344.1	-	2314	2272
env-219	AAMC01046861.1	+	6048	6090
env-220	AAMC01048514.1	-	7078	7036
env-221	AAMC01072515.1	+	159	201
env-222	AAMC01082747.1	+	18201	18243
env-223	AAMC01083624.1	-	7644	7602
env-224	AAMC01090569.1	+	5102	5144
env-225	AAMC01094959.1	-	9447	9405
env-226	AAMC01109753.1	+	5763	5805

env-227	AAMC01111876.1	+	5589	5631
env-228	AAMC01112903.1	-	3577	3535
env-229	AAMC01000808.1	+	37844	37886
env-230	AAMC01059828.1	+	7311	7353
env-231	AAMC01165124.1	-	1147	1105
env-232	AAMC01038925.1	+	253	295
env-233	AAMC01038984.1	-	5485	5443
env-234	AAMC01065800.1	+	2636	2678
env-235	AAMC01065803.1	+	1158	1200
env-236	AAMC01069250.1	-	37067	37025
env-237	AAMC01042570.1	-	4852	4810
env-238	AAMC01071358.1	-	847	805
env-239	AAMC01074735.1	-	10911	10869
env-240	AAMC01074735.1	-	7666	7624
env-241	AAMC01093138.1	+	20986	21028
env-242	AAMC01047568.1	+	7808	7850
env-243	AAMC01062343.1	-	7185	7143
env-244	AAMC01056398.1	+	54078	54120
env-245	AAMC01111912.1	+	8175	8217
env-246	AAMC01114550.1	-	7323	7281
env-247	AAMC01114551.1	-	1043	1001
env-248	AAMC01038953.1	-	5300	5258
env-249	AAMC01049610.1	+	557	599
env-250	AAMC01129716.1	-	6805	6763
env-251	AAMC01127315.1	-	2424	2382
env-252	AAMC01120168.1	-	19273	19231
env-253	AAMC01116604.1	-	2871	2829
env-254	AAMC01113646.1	+	5505	5547
env-255	AAMC01112160.1	+	15845	15887
env-256	AAMC01110099.1	+	1075	1117
env-257	AAMC01108600.1	-	28082	28040
env-258	AAMC01107866.1	+	5532	5574
env-259	AAMC01100204.1	+	263	305
env-260	AAMC01093665.1	-	267	225
env-261	AAMC01150818.1	-	1625	1583
env-262	AAMC01092129.1	-	2609	2567
env-263	AAMC01101406.1	-	11373	11331
env-264	AAMC01089558.1	+	11620	11662
env-265	AAMC01083081.1	+	2444	2486
env-266	AAMC01172710.1	-	277	235
env-267	AAMC01068245.1	-	11877	11835
env-268	AAMC01001723.1	-	17795	17753
env-269	AAMC01002429.1	-	4538	4496
env-270	AAMC01005414.1	+	41337	41379
env-271	AAMC01017303.1	+	10765	10807
env-272	AAMC01017644.1	+	58	100
env-273	AAMC01022881.1	+	22265	22307
env-274	AAMC01035455.1	-	8028	7986
env-275	AAMC01043548.1	-	3884	3842
env-276	AAMC01047488.1	-	3550	3508
env-277	AAMC01048837.1	-	4681	4639
env-278	AAMC01048948.1	+	13858	13900

env-279	AAMC01084388.1	+	6133	6175
env-280	AAMC01102278.1	+	773	815
env-281	AAMC01107257.1	+	29850	29892
env-282	AAMC01108544.1	+	5844	5886
env-283	AAMC01116727.1	-	790	748
env-284	AAMC01120548.1	-	9268	9226
env-285	AAMC01067352.1	-	2344	2302
env-286	AAMC01065209.1	+	11868	11910
env-287	AAMC01063731.1	-	1613	1571
env-288	AAMC01140410.1	-	3550	3508
env-289	AAMC01020468.1	-	14170	14128
env-290	AAMC01031700.1	+	11821	11863
env-291	AAMC01038390.1	+	21	63
env-292	AAMC01002174.1	-	34780	34738
env-293	AAMC01070560.1	+	2283	2325
env-294	AAMC01094973.1	+	8400	8442
env-295	AAMC01001705.1	+	2052	2094
env-296	AAMC01004122.1	-	35972	35931
env-297	AAMC01004122.1	-	35707	35666
env-298	AAMC01004122.1	-	35440	35399
env-299	AAMC01006134.1	-	7480	7439
env-300	AAMC01009496.1	-	14913	14872
env-301	AAMC01011506.1	+	124	165
env-302	AAMC01014096.1	+	4814	4855
env-303	AAMC01014243.1	+	5799	5840
env-304	AAMC01014385.1	-	7251	7210
env-305	AAMC01015271.1	+	25034	25075
env-306	AAMC01019616.1	-	13331	13290
env-307	AAMC01025241.1	+	5246	5287
env-308	AAMC01028790.1	-	6481	6440
env-309	AAMC01028790.1	-	6214	6173
env-310	AAMC01030759.1	+	57253	57294
env-311	AAMC01031700.1	+	11553	11594
env-312	AAMC01032105.1	-	16576	16535
env-313	AAMC01038387.1	+	13099	13140
env-314	AAMC01041655.1	-	37586	37545
env-315	AAMC01048024.1	+	1637	1678
env-316	AAMC01053673.1	+	26607	26648
env-317	AAMC01062835.1	-	875	834
env-318	AAMC01064490.1	-	1452	1411
env-319	AAMC01064491.1	-	797	756
env-320	AAMC01069175.1	+	3066	3107
env-321	AAMC01070487.1	+	19414	19455
env-322	AAMC01072731.1	+	9582	9623
env-323	AAMC01084365.1	+	1426	1467
env-324	AAMC01088337.1	+	12491	12532
env-325	AAMC01089502.1	-	23068	23027
env-326	AAMC01090536.1	-	979	938
env-327	AAMC01096429.1	+	11474	11515
env-328	AAMC01100125.1	+	9860	9901
env-329	AAMC01105184.1	-	2336	2295
env-330	AAMC01107440.1	+	8057	8098

env-331	AAMC01112245.1	+	8340	8381
env-332	AAMC01117070.1	+	550	591
env-333	AAMC01122825.1	+	17578	17619
env-334	AAMC01123017.1	-	30135	30094
env-335	AAMC01004122.1	-	35973	35931
env-336	AAMC01004122.1	-	35708	35666
env-337	AAMC01004122.1	-	35441	35399
env-338	AAMC01006134.1	-	7481	7439
env-339	AAMC01009496.1	-	14914	14872
env-340	AAMC01011506.1	+	123	165
env-341	AAMC01014096.1	+	4813	4855
env-342	AAMC01014243.1	+	5798	5840
env-343	AAMC01014385.1	-	7252	7210
env-344	AAMC01015271.1	+	25033	25075
env-345	AAMC01025241.1	+	5245	5287
env-346	AAMC01028790.1	-	6482	6440
env-347	AAMC01028790.1	-	6215	6173
env-348	AAMC01030759.1	+	57252	57294
env-349	AAMC01031700.1	+	11552	11594
env-350	AAMC01032105.1	-	16577	16535
env-351	AAMC01038387.1	+	13098	13140
env-352	AAMC01041655.1	-	37587	37545
env-353	AAMC01048024.1	+	1636	1678
env-354	AAMC01053673.1	+	26606	26648
env-355	AAMC01062835.1	-	876	834
env-356	AAMC01064490.1	-	1453	1411
env-357	AAMC01064491.1	-	798	756
env-358	AAMC01069175.1	+	3065	3107
env-359	AAMC01070487.1	+	19413	19455
env-360	AAMC01072731.1	+	9581	9623
env-361	AAMC01084365.1	+	1425	1467
env-362	AAMC01088337.1	+	12490	12532
env-363	AAMC01089502.1	-	23069	23027
env-364	AAMC01090536.1	-	980	938
env-365	AAMC01096429.1	+	11473	11515
env-366	AAMC01100125.1	+	9859	9901
env-367	AAMC01105184.1	-	2337	2295
env-368	AAMC01107440.1	+	8056	8098
env-369	AAMC01117070.1	+	549	591
env-370	AAMC01122825.1	+	17577	17619
env-371	AAMC01123017.1	-	30136	30094
env-372	AAMC01025566.1	-	33924	33882
env-373	AAMC01047488.1	-	3819	3777
env-374	AAMC01007693.1	-	6485	6443
env-375	AAMC01010803.1	-	14757	14715
env-376	AAMC01013474.1	-	1331	1289
env-377	AAMC01075491.1	+	14487	14529
env-378	AAMC01030270.1	-	2526	2484
env-379	AAMC01014232.1	+	4453	4495
env-380	AAMC01038925.1	-	86	44
env-381	AAMC01045827.1	+	1503	1545
env-382	AAMC01077541.1	+	22241	22283

env-383	AAMC01084427.1	-	21723	21681
env-384	AAMC01094742.1	-	1612	1570
env-385	AAMC01098501.1	+	3037	3079
env-386	AAMC01098668.1	-	7745	7703
env-387	AAMC01120705.1	+	8977	9019
env-388	AAMC01156494.1	-	2510	2468
env-389	AAMC01032066.1	+	6021	6063
env-390	AAMC01081949.1	-	4494	4452
env-391	AAMC01138076.1	-	3112	3070
env-392	AAMC01021197.1	-	1032	990
env-393	AAMC01012341.1	+	34989	35031
env-394	AAMC01008887.1	+	3975	4017
env-395	AAMC01002923.1	+	43332	43374
env-396	AAMC01009668.1	-	34299	34257
env-397	AAMC01019920.1	+	46	88
env-398	AAMC01020468.1	-	13904	13862
env-399	AAMC01037305.1	+	14576	14618
env-400	AAMC01045899.1	+	22322	22364
env-401	AAMC01070560.1	+	895	937
env-402	AAMC01090418.1	+	16893	16935
env-403	AAMC01092742.1	-	3051	3009
env-404	AAMC01103892.1	-	18941	18899
env-405	AAMC01103988.1	-	6105	6063
env-406	AAMC01105995.1	-	8560	8518
env-407	AAMC01112864.1	-	17128	17086
env-408	AAMC01125562.1	+	20574	20616
env-409	AAMC01014207.1	-	31264	31222
env-410	AAMC01004321.1	-	419	377
env-411	AAMC01030853.1	-	3174	3132
env-412	AAMC01048300.1	+	10208	10250
env-413	AAMC01022016.1	-	20552	20510
env-414	AAMC01024065.1	-	932	890
env-415	AAMC01031366.1	-	2701	2659
env-416	AAMC01031605.1	+	3331	3373
env-417	AAMC01090887.1	-	47254	47212
env-418	AAMC01010244.1	-	5538	5496
env-419	AAMC01020620.1	-	4077	4035
env-420	AAMC01046090.1	+	11079	11121
env-421	AAMC01033666.1	+	7693	7735
env-422	AAMC01033655.1	+	327	369
env-423	AAMC01039212.1	-	6189	6147
env-424	AAMC01028322.1	+	6318	6360
env-425	AAMC01021516.1	+	937	979
env-426	AAMC01020984.1	+	11285	11327
env-427	AAMC01041857.1	-	4950	4908
env-428	AAMC01080566.1	-	9927	9885
env-429	AAMC01004621.1	+	10600	10642
env-430	AAMC01001312.1	-	6615	6573
env-431	AAMC01025231.1	-	9588	9546
env-432	AAMC01072190.1	+	11449	11491
env-433	AAMC01102330.1	+	3208	3250
env-434	AAMC01102330.1	+	3480	3522

env-435	AAMC01105148.1	-	5178	5136
env-436	AAMC01123224.1	+	1084	1126
env-437	AAMC01173769.1	+	725	767
env-438	AAMC01002415.1	-	212	170
env-439	AAMC01003006.1	+	2352	2393
env-440	AAMC01004276.1	-	30588	30547
env-441	AAMC01006594.1	+	836	877
env-442	AAMC01006953.1	+	42783	42824
env-443	AAMC01009537.1	+	3664	3705
env-444	AAMC01010558.1	+	12903	12944
env-445	AAMC01011142.1	+	9517	9558
env-446	AAMC01014607.1	-	1553	1512
env-447	AAMC01017133.1	+	42194	42235
env-448	AAMC01018312.1	+	7927	7968
env-449	AAMC01025633.1	-	600	559
env-450	AAMC01031374.1	-	4171	4130
env-451	AAMC01033439.1	+	13474	13515
env-452	AAMC01033658.1	+	6393	6434
env-453	AAMC01034744.1	-	64	23
env-454	AAMC01038984.1	-	5928	5887
env-455	AAMC01044418.1	+	8101	8142
env-456	AAMC01047486.1	-	2710	2669
env-457	AAMC01048213.1	-	25295	25254
env-458	AAMC01048899.1	+	20547	20588
env-459	AAMC01050139.1	-	12424	12383
env-460	AAMC01050247.1	+	41556	41597
env-461	AAMC01054043.1	+	1120	1161
env-462	AAMC01058802.1	-	14953	14912
env-463	AAMC01061152.1	+	7010	7051
env-464	AAMC01061176.1	-	4608	4567
env-465	AAMC01063102.1	+	10900	10941
env-466	AAMC01063146.1	+	9594	9635
env-467	AAMC01063290.1	-	1317	1276
env-468	AAMC01063742.1	-	5634	5593
env-469	AAMC01067101.1	+	21330	21371
env-470	AAMC01069551.1	+	1935	1976
env-471	AAMC01069551.1	+	2480	2521
env-472	AAMC01069631.1	+	4470	4511
env-473	AAMC01072219.1	+	133	174
env-474	AAMC01073868.1	-	13845	13804
env-475	AAMC01077245.1	+	123	164
env-476	AAMC01077753.1	+	3079	3120
env-477	AAMC01078778.1	-	490	449
env-478	AAMC01080620.1	-	1170	1129
env-479	AAMC01081204.1	+	31242	31283
env-480	AAMC01086674.1	+	3188	3229
env-481	AAMC01087105.1	+	9489	9530
env-482	AAMC01094616.1	-	967	926
env-483	AAMC01094983.1	-	73580	73539
env-484	AAMC01096177.1	-	1885	1844
env-485	AAMC01097952.1	-	17609	17568
env-486	AAMC01099973.1	-	21306	21265

env-487	AAMC01101467.1	+	323	364
env-488	AAMC01103205.1	+	6147	6188
env-489	AAMC01105782.1	-	2567	2526
env-490	AAMC01106794.1	-	14190	14149
env-491	AAMC01108296.1	-	9524	9483
env-492	AAMC01108600.1	-	27809	27768
env-493	AAMC01112304.1	-	7880	7839
env-494	AAMC01112304.1	-	7609	7568
env-495	AAMC01112403.1	+	4595	4636
env-496	AAMC01113153.1	-	15468	15427
env-497	AAMC01114550.1	-	5918	5877
env-498	AAMC01123480.1	+	15962	16003
env-499	AAMC01140008.1	+	884	925
env-500	AAMC01140629.1	-	938	897
env-501	AAMC01140629.1	-	397	356
env-502	AAMC01151225.1	-	1866	1825
env-503	AAMC01156494.1	-	2238	2197
env-504	AAMC01156606.1	-	1076	1035
env-505	AAMC01162244.1	+	789	830
env-506	AAMC01004276.1	-	30589	30547
env-507	AAMC01006594.1	+	835	877
env-508	AAMC01006953.1	+	42782	42824
env-509	AAMC01009537.1	+	3663	3705
env-510	AAMC01010558.1	+	12902	12944
env-511	AAMC01011142.1	+	9516	9558
env-512	AAMC01014607.1	-	1554	1512
env-513	AAMC01017133.1	+	42193	42235
env-514	AAMC01025633.1	-	601	559
env-515	AAMC01031374.1	-	4172	4130
env-516	AAMC01033439.1	+	13473	13515
env-517	AAMC01033658.1	+	6392	6434
env-518	AAMC01034744.1	-	65	23
env-519	AAMC01044418.1	+	8100	8142
env-520	AAMC01048213.1	-	25296	25254
env-521	AAMC01048899.1	+	20546	20588
env-522	AAMC01050247.1	+	41555	41597
env-523	AAMC01054043.1	+	1119	1161
env-524	AAMC01058802.1	-	14954	14912
env-525	AAMC01061152.1	+	7009	7051
env-526	AAMC01061176.1	-	4609	4567
env-527	AAMC01063102.1	+	10899	10941
env-528	AAMC01063146.1	+	9593	9635
env-529	AAMC01063290.1	-	1318	1276
env-530	AAMC01063742.1	-	5635	5593
env-531	AAMC01067101.1	+	21329	21371
env-532	AAMC01069551.1	+	1934	1976
env-533	AAMC01069551.1	+	2479	2521
env-534	AAMC01069631.1	+	4469	4511
env-535	AAMC01073868.1	-	13846	13804
env-536	AAMC01077245.1	+	122	164
env-537	AAMC01077753.1	+	3078	3120
env-538	AAMC01078778.1	-	491	449

env-539	AAMC01080620.1	-	1171	1129
env-540	AAMC01081204.1	+	31241	31283
env-541	AAMC01086674.1	+	3187	3229
env-542	AAMC01087105.1	+	9488	9530
env-543	AAMC01094616.1	-	968	926
env-544	AAMC01094983.1	-	73581	73539
env-545	AAMC01097952.1	-	17610	17568
env-546	AAMC01099973.1	-	21307	21265
env-547	AAMC01101467.1	+	322	364
env-548	AAMC01103205.1	+	6146	6188
env-549	AAMC01105782.1	-	2568	2526
env-550	AAMC01106794.1	-	14191	14149
env-551	AAMC01108600.1	-	27810	27768
env-552	AAMC01112304.1	-	7881	7839
env-553	AAMC01112304.1	-	7610	7568
env-554	AAMC01112403.1	+	4594	4636
env-555	AAMC01113153.1	-	15469	15427
env-556	AAMC01114550.1	-	5919	5877
env-557	AAMC01123480.1	+	15961	16003
env-558	AAMC01140008.1	+	883	925
env-559	AAMC01140629.1	-	939	897
env-560	AAMC01140629.1	-	398	356
env-561	AAMC01151225.1	-	1867	1825
env-562	AAMC01156494.1	-	2239	2197
env-563	AAMC01156606.1	-	1077	1035
env-564	AAMC01162244.1	+	788	830
env-565	AAMC01011176.1	-	1900	1858
env-566	AAMC01033554.1	-	1395	1353
env-567	AAMC01048213.1	-	25568	25526
env-568	AAMC01020983.1	-	2832	2790
env-569	AAMC01026511.1	+	27	69
env-570	AAMC01077245.1	+	396	438
env-571	AAMC01106794.1	-	13919	13877
env-572	AAMC01106794.1	-	13648	13606
env-573	AAMC01104154.1	-	1733	1691
env-574	AAMC01089528.1	+	4599	4641
env-575	AAMC01084520.1	-	7797	7755
env-576	AAMC01121068.1	-	3032	2990
env-577	AAMC01006818.1	-	6147	6105
env-578	AAMC01006850.1	-	7375	7333
env-579	AAMC01071329.1	+	10214	10256
env-580	AAMC01117341.1	+	8662	8704
env-581	AAMC01041699.1	-	5236	5194
env-582	AAMC01071329.1	+	9948	9990
env-583	AAMC01084359.1	+	8416	8458
env-584	AAMC01074853.1	+	8426	8468
env-585	AAMC01129964.1	+	853	895
env-586	AAMC01148345.1	+	19	61
env-587	AAMC01154488.1	-	181	139
env-588	AAMC01082585.1	-	22638	22596
env-589	AAMC01072578.1	-	368	326
env-590	AAMC01107440.1	-	5666	5624

env-591	AAMC01040219.1	+	5012	5054
env-592	AAMC01046892.1	-	4761	4719
env-593	AAMC01051430.1	-	12234	12192
env-594	AAMC01046318.1	+	3221	3263
env-595	AAMC01120548.1	-	8989	8947
env-596	AAMC01048956.1	-	4241	4199
env-597	AAMC01051203.1	-	469	427
env-598	AAMC01047095.1	-	53247	53205
env-599	AAMC01039510.1	+	1876	1918
env-600	AAMC01052866.1	+	7448	7490
env-601	AAMC01127475.1	+	177	219
env-602	AAMC01041938.1	-	1256	1214
env-603	AAMC01073187.1	+	145	187
env-604	AAMC01075589.1	-	19233	19191
env-605	AAMC01005245.1	+	11703	11744
env-606	AAMC01048956.1	-	4547	4505
env-607	AAMC01012830.1	+	34775	34817
env-608	AAMC01046682.1	-	71259	71217
env-609	AAMC01032896.1	-	54572	54530
env-610	AAMC01011366.1	-	72	30
env-611	AAMC01011373.1	-	856	814
env-612	AAMC01011373.1	-	564	522
env-613	AAMC01081668.1	-	15703	15661
env-614	AAMC01057290.1	-	2414	2372
env-615	AAMC01010632.1	+	59	101
env-616	AAMC01005766.1	-	14223	14181
env-617	AAMC01003482.1	-	13241	13199
env-618	AAMC01000996.1	-	41725	41683
env-619	AAMC01053630.1	-	2998	2956
env-620	AAMC01129733.1	+	3804	3846
env-621	AAMC01046152.1	+	5515	5557
env-622	AAMC01000603.1	+	10426	10468
env-623	AAMC01000745.1	+	12483	12525
env-624	AAMC01000932.1	-	13399	13357
env-625	AAMC01001216.1	-	22840	22798
env-626	AAMC01003030.1	-	3741	3699
env-627	AAMC01003125.1	-	37532	37490
env-628	AAMC01003711.1	+	3667	3709
env-629	AAMC01004150.1	+	530	572
env-630	AAMC01004151.1	+	4065	4107
env-631	AAMC01006071.1	-	15800	15758
env-632	AAMC01006345.1	+	23845	23887
env-633	AAMC01006905.1	-	1007	965
env-634	AAMC01006905.1	-	830	788
env-635	AAMC01006925.1	-	14040	13998
env-636	AAMC01006953.1	+	31901	31943
env-637	AAMC01006976.1	+	3458	3500
env-638	AAMC01006986.1	-	46534	46492
env-639	AAMC01008473.1	-	12542	12500
env-640	AAMC01009030.1	+	15904	15946
env-641	AAMC01009471.1	-	2433	2391
env-642	AAMC01009537.1	-	3444	3402

env-643	AAMC01014236.1	+	854	896
env-644	AAMC01014244.1	+	8233	8275
env-645	AAMC01017484.1	+	16564	16606
env-646	AAMC01019058.1	+	16815	16857
env-647	AAMC01023669.1	-	33354	33312
env-648	AAMC01024288.1	+	13732	13774
env-649	AAMC01026549.1	+	16786	16828
env-650	AAMC01029029.1	-	578	536
env-651	AAMC01029382.1	+	14998	15040
env-652	AAMC01030585.1	+	110	152
env-653	AAMC01031520.1	+	4322	4364
env-654	AAMC01033430.1	-	211	169
env-655	AAMC01034136.1	-	16793	16751
env-656	AAMC01035993.1	-	16604	16562
env-657	AAMC01036080.1	+	7427	7469
env-658	AAMC01039721.1	-	225	183
env-659	AAMC01040391.1	-	2915	2873
env-660	AAMC01041126.1	-	22016	21974
env-661	AAMC01041132.1	-	4784	4742
env-662	AAMC01042344.1	-	2580	2538
env-663	AAMC01042648.1	+	8805	8847
env-664	AAMC01042882.1	-	8795	8753
env-665	AAMC01043548.1	+	3995	4037
env-666	AAMC01044407.1	-	12412	12370
env-667	AAMC01044586.1	+	77	119
env-668	AAMC01044598.1	-	15993	15951
env-669	AAMC01045082.1	-	41847	41805
env-670	AAMC01045823.1	+	3108	3150
env-671	AAMC01046861.1	+	6320	6362
env-672	AAMC01046861.1	+	6592	6634
env-673	AAMC01046861.1	+	6864	6906
env-674	AAMC01048947.1	-	23509	23467
env-675	AAMC01049999.1	+	14262	14304
env-676	AAMC01050517.1	+	19183	19225
env-677	AAMC01050917.1	+	21416	21458
env-678	AAMC01052061.1	+	42881	42923
env-679	AAMC01053358.1	+	107	149
env-680	AAMC01053947.1	-	15883	15841
env-681	AAMC01055991.1	-	1443	1401
env-682	AAMC01056080.1	-	3174	3132
env-683	AAMC01056761.1	+	4227	4269
env-684	AAMC01060021.1	+	2181	2223
env-685	AAMC01060021.1	-	10311	10269
env-686	AAMC01064828.1	+	11218	11260
env-687	AAMC01064830.1	-	6723	6681
env-688	AAMC01065876.1	-	5582	5540
env-689	AAMC01066582.1	+	77	119
env-690	AAMC01069790.1	-	45396	45354
env-691	AAMC01070594.1	-	14584	14542
env-692	AAMC01071081.1	-	25715	25673
env-693	AAMC01071134.1	-	24599	24557
env-694	AAMC01071170.1	-	17411	17369

env-695	AAMC01071356.1	-	52780	52738
env-696	AAMC01073143.1	+	58986	59028
env-697	AAMC01073851.1	+	16445	16487
env-698	AAMC01074079.1	+	28305	28347
env-699	AAMC01074163.1	+	7179	7221
env-700	AAMC01075174.1	-	3170	3128
env-701	AAMC01076291.1	+	7895	7937
env-702	AAMC01076565.1	+	963	1005
env-703	AAMC01080955.1	-	1604	1562
env-704	AAMC01082242.1	+	6215	6257
env-705	AAMC01084388.1	+	6405	6447
env-706	AAMC01084520.1	+	8504	8546
env-707	AAMC01085087.1	+	10250	10292
env-708	AAMC01086741.1	-	6364	6322
env-709	AAMC01087379.1	-	896	854
env-710	AAMC01087627.1	-	6216	6174
env-711	AAMC01089516.1	-	2589	2547
env-712	AAMC01089696.1	-	39534	39492
env-713	AAMC01089706.1	-	192	150
env-714	AAMC01090128.1	+	10484	10526
env-715	AAMC01090636.1	-	662	620
env-716	AAMC01091331.1	-	4493	4451
env-717	AAMC01097813.1	-	19091	19049
env-718	AAMC01100467.1	+	5023	5065
env-719	AAMC01101417.1	+	919	961
env-720	AAMC01102238.1	+	1736	1778
env-721	AAMC01102384.1	-	4806	4764
env-722	AAMC01103604.1	+	11810	11852
env-723	AAMC01103773.1	-	950	908
env-724	AAMC01105762.1	-	43194	43152
env-725	AAMC01109241.1	+	9249	9291
env-726	AAMC01110916.1	-	2200	2158
env-727	AAMC01112256.1	-	13832	13790
env-728	AAMC01117266.1	+	12847	12889
env-729	AAMC01123502.1	+	8809	8851
env-730	AAMC01129400.1	+	7256	7298
env-731	AAMC01145236.1	+	103	145
env-732	AAMC01145696.1	+	418	460
env-733	AAMC01146018.1	+	554	596
env-734	AAMC01150666.1	+	682	724
env-735	AAMC01150991.1	+	3842	3884
env-736	AAMC01156592.1	-	2261	2219
env-737	AAMC01156969.1	+	1219	1261
env-738	AAMC01164257.1	-	746	704
env-739	AAMC01172369.1	+	672	714
env-740	AAMC01176648.1	-	234	192
env-741	AAMC01184383.1	-	378	336
env-742	AAMC01026906.1	-	33194	33153
env-743	AAMC01004150.1	+	639	681
env-744	AAMC01066542.1	-	706	664
env-745	AAMC01127300.1	+	76	118
env-746	AAMC01046932.1	+	3383	3422

env-747	AAMC01012412.1	+	15081	15123
env-748	AAMC01018426.1	-	13757	13715
env-749	AAMC01022708.1	-	14475	14433
env-750	AAMC01028208.1	+	7208	7250
env-751	AAMC01037788.1	+	4602	4644
env-752	AAMC01057456.1	+	1047	1089
env-753	AAMC01084911.1	+	27241	27283
env-754	AAMC01099287.1	+	37	79
env-755	AAMC01100540.1	+	1158	1200
env-756	AAMC01103103.1	+	4328	4370
env-757	AAMC01106477.1	+	56	98
env-758	AAMC01138065.1	+	23466	23508
env-759	AAMC01177187.1	+	943	985
env-760	AAMC01182636.1	-	560	518
env-761	AAMC01039176.1	+	9663	9704
env-762	AAMC01000242.1	+	22809	22851
env-763	AAMC01082008.1	+	102	144
env-764	AAMC01000319.1	-	1274	1232
env-765	AAMC01002253.1	-	956	914
env-766	AAMC01036698.1	+	518	560
env-767	AAMC01040134.1	-	6770	6728
env-768	AAMC01041699.1	+	5443	5485
env-769	AAMC01044449.1	+	1896	1938
env-770	AAMC01057482.1	+	21124	21166
env-771	AAMC01061979.1	-	4276	4234
env-772	AAMC01077532.1	-	5961	5919
env-773	AAMC01084359.1	+	8150	8192
env-774	AAMC01094935.1	+	23050	23092
env-775	AAMC01096810.1	+	6335	6377
env-776	AAMC01096810.1	+	6602	6644
env-777	AAMC01103780.1	-	8382	8340
env-778	AAMC01112804.1	-	24411	24369
env-779	AAMC01123426.1	+	748	790
env-780	AAMC01152085.1	-	1531	1489
env-781	AAMC01007333.1	-	1623	1581
env-782	AAMC01007353.1	-	740	698
env-783	AAMC01045280.1	+	1785	1827
env-784	AAMC01052699.1	-	1018	976
env-785	AAMC01052700.1	-	5489	5447
env-786	AAMC01097875.1	+	12675	12717
env-787	AAMC01187267.1	+	284	326
env-788	AAMC01009351.1	-	6944	6902
env-789	AAMC01079003.1	-	19909	19867
env-790	AAMC01004883.1	-	2691	2649
env-791	AAMC01003496.1	-	2222	2180
env-792	AAMC01048513.1	-	49433	49391
env-793	AAMC01080860.1	+	8546	8588
env-794	AAMC01153362.1	+	1359	1401
env-795	AAMC01012190.1	-	3532	3490
env-796	AAMC01015913.1	-	4548	4506
env-797	AAMC01017834.1	-	9974	9932
env-798	AAMC01048612.1	-	3933	3891

env-799	AAMC01064596.1	-	5489	5447
env-800	AAMC01071319.1	+	9451	9493
env-801	AAMC01171713.1	+	399	441
env-802	AAMC01051899.1	-	10617	10575
env-803	AAMC01011179.1	-	13665	13623
env-804	AAMC01031773.1	-	34407	34365
env-805	AAMC01007197.1	-	21238	21196
env-806	AAMC01004450.1	-	17747	17705
env-807	AAMC01001575.1	-	30181	30140
env-808	AAMC01026209.1	-	10806	10765
env-809	AAMC01061592.1	-	21840	21799
env-810	AAMC01105695.1	-	12259	12218
env-811	AAMC01121475.1	-	30323	30282
env-812	AAMC01118697.1	+	8575	8617
env-813	AAMC01000539.1	-	17955	17913
env-814	AAMC01003674.1	+	1229	1271
env-815	AAMC01040193.1	+	167	209
env-816	AAMC01041132.1	+	4911	4953
env-817	AAMC01099631.1	+	6626	6668
env-818	AAMC01109278.1	+	4092	4134
env-819	AAMC01003675.1	+	500	542
env-820	AAMC01075689.1	-	10756	10714
env-821	AAMC01181311.1	-	300	258
env-822	AAMC01003030.1	+	3869	3911
env-823	AAMC01017159.1	+	19941	19983
env-824	AAMC01018408.1	+	1022	1064
env-825	AAMC01035438.1	+	8	50
env-826	AAMC01048300.1	-	9980	9938
env-827	AAMC01058697.1	+	22132	22174
env-828	AAMC01089696.1	+	39720	39762
env-829	AAMC01102331.1	+	12974	13016
env-830	AAMC01102498.1	-	10260	10218
env-831	AAMC01000995.1	-	2165	2123
env-832	AAMC01014704.1	+	9566	9608
env-833	AAMC01165257.1	-	361	319
env-834	AAMC01014629.1	-	8110	8068
env-835	AAMC01168955.1	+	1777	1819
env-836	AAMC01063742.1	-	20708	20666
env-837	AAMC01102193.1	+	8486	8528
env-838	AAMC01104100.1	-	23203	23161
env-839	JCVI_SCAF_1096627096558	+	3354	3410
env-840	JCVI_SCAF_1096627928087	-	1276	1223
env-841	NCBI_BBAY_READ_1106105089367	+	998	1055
env-842	JCVI_SCAF_1101668011206	+	75	128
env-843	JCVI_SCAF_1096627709283	+	1294	1347
env-844	JCVI_SCAF_1096628035178	+	1112	1165
env-845	JCVI_SCAF_1101668029935	-	1075	1022
env-846	JCVI_SCAF_1101668395648	-	202	149
env-847	JCVI_SCAF_1101668047413	-	1345	1288
env-848	JCVI_SCAF_1101668047413	-	567	514
env-849	JCVI_SCAF_1096627431775	-	186	131
env-850	JCVI_SCAF_1096627033904	-	268	209

env-851	JCVI_SCAF_1101668067376	+	1219	1278
env-852	NCBI_BBAY_READ_1106073101911	+	400	459
env-853	ABLX01261490	+	46	99
env-854	NCBI_BBAY_READ_1106073039913	-	459	406
env-855	JCVI_SCAF_1101668030970	-	643	588
env-856	JCVI_SCAF_1096627166296	-	797	740
env-857	JCVI_SCAF_1096627423712	+	563	635
env-858	JCVI_SCAF_1101668059266	-	770	719
env-859	JCVI_SCAF_1101668082200	+	290	342
Aae-1-4346	NW_001810912.1	+	2388932	2388984
env-860	JCVI_SCAF_1096626414779	+	849	949
Csy-1-2	NS_000189.1	+	686187	686236

Duplicate sequences: the following putative homologs are not shown in the alignment because their sequences are identical to a homolog already shown: , , , , , , , , , , , ,
, ,
, ,
, ,
, ,
, ,
, ,
, ,
, ,
, ,
, ,
, ,
, ,
, ,
, ,
, ,
, ,
, ,
, ,
, ,
, ,
, ,
, ,
, ,
, ,
, ,
, ,
, ,
, ,
, ,
, ,
, ,
, ,
, ,
, ,

, ,
, ,
, ,
, ,
, ,
, ,
, ,
, ,
, ,
, ,
, ,
, ,
, ,
, ,
, ,
, ,
, ,
, ,
, ,
, ,
, ,
, ,
, ,
, ,
, ,
, ,
, ,
, ,
, ,
, ,
, ,
, ,
, ,
, ,
, ,
, ,
, ,
, ,
, ,
, ,
, ,
, ,
, ,
, ,
, ,

, ,
, ,
, ,
, ,
, ,
, ,
, ,
, ,
, ,
, ,
, ,
, Aae-1-10, Aae-1-1000, Aae-1-1001, Aae-1-1002, Aae-1-1003, Aae-1-1004, Aae-1-1005, Aae-1-1006, Aae-1-1007, Aae-1-1008, Aae-1-1009, Aae-1-1010, Aae-1-1011, Aae-1-1012, Aae-1-1013, Aae-1-1014, Aae-1-1015, Aae-1-1016, Aae-1-1017, Aae-1-1018, Aae-1-1019, Aae-1-1020, Aae-1-1021, Aae-1-1022, Aae-1-1023, Aae-1-1024, Aae-1-1025, Aae-1-1026, Aae-1-1027, Aae-1-1028, Aae-1-1029, Aae-1-1030, Aae-1-1031, Aae-1-1032, Aae-1-1033, Aae-1-1034, Aae-1-1035, Aae-1-1036, Aae-1-1037, Aae-1-1038, Aae-1-1039, Aae-1-1040, Aae-1-1041, Aae-1-1042, Aae-1-1043, Aae-1-1044, Aae-1-1045, Aae-1-1046, Aae-1-1047, Aae-1-1048, Aae-1-1049, Aae-1-1050, Aae-1-1051, Aae-1-1052, Aae-1-1053, Aae-1-1054, Aae-1-1055, Aae-1-1056, Aae-1-1057, Aae-1-1058, Aae-1-1059, Aae-1-1060, Aae-1-1061, Aae-1-1062, Aae-1-1063, Aae-1-1064, Aae-1-1065, Aae-1-1066, Aae-1-1067, Aae-1-1068, Aae-1-1069, Aae-1-1070, Aae-1-1071, Aae-1-1072, Aae-1-1073, Aae-1-1074, Aae-1-1075, Aae-1-1076, Aae-1-1077, Aae-1-1078, Aae-1-1079, Aae-1-1080, Aae-1-1081, Aae-1-1082, Aae-1-1083, Aae-1-1084, Aae-1-1085, Aae-1-1086, Aae-1-1087, Aae-1-1088, Aae-1-1089, Aae-1-1090, Aae-1-1091, Aae-1-1092, Aae-1-1093, Aae-1-1094, Aae-1-1095, Aae-1-1096, Aae-1-1097, Aae-1-1098, Aae-1-1099, Aae-1-11, Aae-1-1100, Aae-1-1101, Aae-1-1102, Aae-1-1103, Aae-1-1104, Aae-1-1105, Aae-1-1106, Aae-1-1107, Aae-1-1108, Aae-1-1109, Aae-1-1110, Aae-1-1111, Aae-1-1112, Aae-1-1113, Aae-1-1114, Aae-1-1115, Aae-1-1116, Aae-1-1117, Aae-1-1118, Aae-1-1119, Aae-1-1120, Aae-1-1121, Aae-1-1122, Aae-1-1123, Aae-1-1124, Aae-1-1125, Aae-1-1126, Aae-1-1127, Aae-1-1128, Aae-1-1129, Aae-1-1130, Aae-1-1131, Aae-1-1132, Aae-1-1133, Aae-1-1134, Aae-1-1135, Aae-1-1136, Aae-1-1137, Aae-1-1138, Aae-1-1139, Aae-1-1140, Aae-1-1141, Aae-1-1142, Aae-1-1143, Aae-1-1144, Aae-1-1145, Aae-1-1146, Aae-1-1147, Aae-1-1148, Aae-1-1149, Aae-1-1150, Aae-1-1151, Aae-1-1152, Aae-1-1153, Aae-1-1154, Aae-1-1155, Aae-1-1156, Aae-1-1157, Aae-1-1158, Aae-1-1159, Aae-1-1160, Aae-1-1161, Aae-1-1162, Aae-1-1163, Aae-1-1164, Aae-1-1165, Aae-1-1166, Aae-1-1167, Aae-1-1168, Aae-1-1169, Aae-1-1170, Aae-1-1171, Aae-1-1172, Aae-1-1173, Aae-1-1174, Aae-1-1175, Aae-1-1176, Aae-1-1177, Aae-1-1178, Aae-1-1179, Aae-1-1180, Aae-1-1181, Aae-1-1182, Aae-1-1183, Aae-1-1184, Aae-1-1185, Aae-1-1186, Aae-1-1187, Aae-1-1188, Aae-1-1189, Aae-1-1190, Aae-1-1191, Aae-1-1192, Aae-1-1193, Aae-1-1194, Aae-1-1195, Aae-1-1196, Aae-1-1197, Aae-1-1198, Aae-1-1199, Aae-1-12, Aae-1-1200, Aae-1-1201, Aae-1-1202, Aae-1-1203, Aae-1-1204, Aae-1-1205, Aae-1-1206, Aae-1-1207, Aae-1-1208, Aae-1-1209, Aae-1-1210, Aae-1-1211, Aae-1-1212, Aae-1-1213, Aae-1-1214, Aae-1-1215, Aae-1-1216, Aae-1-1217, Aae-1-1218, Aae-1-1219, Aae-1-1220, Aae-1-1221, Aae-1-1222, Aae-1-1223, Aae-1-1224, Aae-1-1225, Aae-1-1226, Aae-1-1227, Aae-1-1228, Aae-1-1229, Aae-1-1230, Aae-1-1231, Aae-1-1232, Aae-1-1233, Aae-1-1234, Aae-1-1235, Aae-1-1236, Aae-1-1237, Aae-1-1238, Aae-1-1239, Aae-1-124, Aae-1-1240, Aae-1-1241, Aae-1-1242, Aae-1-1243, Aae-1-1244, Aae-1-1245, Aae-1-1246, Aae-1-1247, Aae-1-1248, Aae-1-1249, Aae-1-1250, Aae-1-1251, Aae-1-1252, Aae-1-1253, Aae-1-1254, Aae-1-1255, Aae-1-1256, Aae-1-1257, Aae-1-1258, Aae-1-1259, Aae-1-1260, Aae-1-1261, Aae-1-1262, Aae-1-1263, Aae-1-1264, Aae-1-1265, Aae-1-1266, Aae-1-1267, Aae-1-1268, Aae-1-1269, Aae-1-1270, Aae-1-1271, Aae-1-1272, Aae-1-1273, Aae-1-1274, Aae-1-1275, Aae-1-1276, Aae-1-1277, Aae-1-1278, Aae-1-1279, Aae-1-1280, Aae-1-1281, Aae-1-1282, Aae-1-1283, Aae-1-1284, Aae-1-1285, Aae-1-1286, Aae-1-1287, Aae-1-1288, Aae-1-1289, Aae-1-1290, Aae-1-1291, Aae-1-1292, Aae-1-1293, Aae-1-1294, Aae-1-1295, Aae-1-1296, Aae-1-1297, Aae-1-1298, Aae-1-1299, Aae-1-13, Aae-1-1300, Aae-1-1301, Aae-1-1302, Aae-1-1303, Aae-1-1304, Aae-1-1305, Aae-1-1306, Aae-1-1307, Aae-1-1308, Aae-1-1309, Aae-1-1310, Aae-1-1311, Aae-1-1312, Aae-1-1313, Aae-1-1314, Aae-1-1315, Aae-1-1316, Aae-1-1317, Aae-1-1318, Aae-1-1319, Aae-1-1320, Aae-1-1321, Aae-1-1322, Aae-1-1323, Aae-1-1324, Aae-1-1325, Aae-1-1326, Aae-1-1327, Aae-1-1328, Aae-1-1329, Aae-1-1330, Aae-1-1331, Aae-1-1332, Aae-1-1333, Aae-1-1334, Aae-1-1335, Aae-1-1336, Aae-1-1337, Aae-1-1338, Aae-1-1339, Aae-1-1340, Aae-1-1341, Aae-1-1342, Aae-1-1343, Aae-1-1344, Aae-1-1345, Aae-1-1346, Aae-1-1347, Aae-1-1348, Aae-1-1349, Aae-1-1350, Aae-1-1351, Aae-1-1352, Aae-1-1353, Aae-1-1354, Aae-1-1355, Aae-1-1356, Aae-1-1357, Aae-1-1358, Aae-1-1359, Aae-1-1360, Aae-1-1361, Aae-1-1362, Aae-1-1363, Aae-1-1364, Aae-1-1365, Aae-1-1366, Aae-1-1367, Aae-1-1368, Aae-1-1369, Aae-1-1370, Aae-1-1371, Aae-1-1372, Aae-1-1373, Aae-1-1374, Aae-1-1375, Aae-1-1376, Aae-1-1377, Aae-1-1378, Aae-1-1379, Aae-1-1380, Aae-1-1381, Aae-1-1382, Aae-1-1383, Aae-1-1384, Aae-1-1385, Aae-1-1386, Aae-1-1387, Aae-1-1388, Aae-1-1389, Aae-1-1390, Aae-1-1391, Aae-1-1392, Aae-1-1393, Aae-1-1394, Aae-1-1395, Aae-1-1396, Aae-1-1397, Aae-1-1398, Aae-1-1399, Aae-1-14, Aae-1-1400, Aae-1-1401, Aae-1-1402, Aae-1-1403, Aae-1-1404, Aae-1-1405, Aae-1-1406, Aae-1-1407, Aae-1-1408, Aae-1-1409, Aae-1-1410, Aae-1-1411, Aae-1-1412, Aae-1-1413, Aae-1-1414, Aae-1-1415, Aae-1-1416, Aae-1-1417, Aae-1-1418, Aae-1-1419, Aae-1-1420, Aae-1-1421, Aae-1-1422, Aae-1-1423, Aae-1-1424, Aae-1-1425, Aae-1-1426, Aae-1-1427, Aae-1-1428, Aae-1-1429, Aae-1-1430, Aae-1-1431, Aae-1-1432,

Aae-1-1433, Aae-1-1434, Aae-1-1435, Aae-1-1436, Aae-1-1437, Aae-1-1438, Aae-1-1439, Aae-1-1440, Aae-1-1441, Aae-1-1442, Aae-1-1443, Aae-1-1444, Aae-1-1445, Aae-1-1446, Aae-1-1447, Aae-1-1448, Aae-1-1449, Aae-1-1450, Aae-1-1451, Aae-1-1452, Aae-1-1453, Aae-1-1454, Aae-1-1455, Aae-1-1456, Aae-1-1457, Aae-1-1458, Aae-1-1459, Aae-1-1460, Aae-1-1461, Aae-1-1462, Aae-1-1463, Aae-1-1464, Aae-1-1465, Aae-1-1466, Aae-1-1467, Aae-1-1468, Aae-1-1469, Aae-1-1470, Aae-1-1471, Aae-1-1472, Aae-1-1473, Aae-1-1474, Aae-1-1475, Aae-1-1476, Aae-1-1477, Aae-1-1478, Aae-1-1479, Aae-1-1480, Aae-1-1481, Aae-1-1482, Aae-1-1483, Aae-1-1484, Aae-1-1485, Aae-1-1486, Aae-1-1487, Aae-1-1488, Aae-1-1489, Aae-1-1490, Aae-1-1491, Aae-1-1492, Aae-1-1493, Aae-1-1494, Aae-1-1495, Aae-1-1496, Aae-1-1497, Aae-1-1498, Aae-1-1499, Aae-1-15, Aae-1-1500, Aae-1-1501, Aae-1-1502, Aae-1-1503, Aae-1-1504, Aae-1-1505, Aae-1-1506, Aae-1-1507, Aae-1-1508, Aae-1-1509, Aae-1-1510, Aae-1-1511, Aae-1-1512, Aae-1-1513, Aae-1-1514, Aae-1-1515, Aae-1-1516, Aae-1-1517, Aae-1-1518, Aae-1-1519, Aae-1-1520, Aae-1-1521, Aae-1-1522, Aae-1-1523, Aae-1-1524, Aae-1-1525, Aae-1-1526, Aae-1-1527, Aae-1-1528, Aae-1-1529, Aae-1-1530, Aae-1-1531, Aae-1-1532, Aae-1-1533, Aae-1-1534, Aae-1-1535, Aae-1-1536, Aae-1-1537, Aae-1-1538, Aae-1-1539, Aae-1-1540, Aae-1-1541, Aae-1-1542, Aae-1-1543, Aae-1-1544, Aae-1-1545, Aae-1-1546, Aae-1-1547, Aae-1-1548, Aae-1-1549, Aae-1-1550, Aae-1-1551, Aae-1-1552, Aae-1-1553, Aae-1-1554, Aae-1-1555, Aae-1-1556, Aae-1-1557, Aae-1-1558, Aae-1-1559, Aae-1-1560, Aae-1-1561, Aae-1-1562, Aae-1-1563, Aae-1-1564, Aae-1-1565, Aae-1-1566, Aae-1-1567, Aae-1-1568, Aae-1-1569, Aae-1-1570, Aae-1-1571, Aae-1-1572, Aae-1-1573, Aae-1-1574, Aae-1-1575, Aae-1-1576, Aae-1-1577, Aae-1-1578, Aae-1-1579, Aae-1-1580, Aae-1-1581, Aae-1-1582, Aae-1-1583, Aae-1-1584, Aae-1-1585, Aae-1-1586, Aae-1-1587, Aae-1-1588, Aae-1-1589, Aae-1-1590, Aae-1-1591, Aae-1-1592, Aae-1-1593, Aae-1-1594, Aae-1-1595, Aae-1-1596, Aae-1-1597, Aae-1-1598, Aae-1-1599, Aae-1-16, Aae-1-160, Aae-1-1600, Aae-1-1601, Aae-1-1602, Aae-1-1603, Aae-1-1604, Aae-1-1605, Aae-1-1606, Aae-1-1607, Aae-1-1608, Aae-1-1609, Aae-1-1610, Aae-1-1611, Aae-1-1612, Aae-1-1613, Aae-1-1614, Aae-1-1615, Aae-1-1616, Aae-1-1617, Aae-1-1618, Aae-1-1619, Aae-1-1620, Aae-1-1621, Aae-1-1622, Aae-1-1623, Aae-1-1624, Aae-1-1625, Aae-1-1626, Aae-1-1627, Aae-1-1628, Aae-1-1629, Aae-1-1630, Aae-1-1631, Aae-1-1632, Aae-1-1633, Aae-1-1634, Aae-1-1635, Aae-1-1636, Aae-1-1637, Aae-1-1638, Aae-1-1639, Aae-1-1640, Aae-1-1641, Aae-1-1642, Aae-1-1643, Aae-1-1644, Aae-1-1645, Aae-1-1646, Aae-1-1647, Aae-1-1648, Aae-1-1649, Aae-1-1650, Aae-1-1651, Aae-1-1652, Aae-1-1653, Aae-1-1654, Aae-1-1655, Aae-1-1656, Aae-1-1657, Aae-1-1658, Aae-1-1659, Aae-1-1660, Aae-1-1661, Aae-1-1662, Aae-1-1663, Aae-1-1664, Aae-1-1665, Aae-1-1666, Aae-1-1667, Aae-1-1668, Aae-1-1669, Aae-1-1670, Aae-1-1671, Aae-1-1672, Aae-1-1673, Aae-1-1674, Aae-1-1675, Aae-1-1676, Aae-1-1677, Aae-1-1678, Aae-1-1679, Aae-1-1680, Aae-1-1681, Aae-1-1682, Aae-1-1683, Aae-1-1684, Aae-1-1685, Aae-1-1686, Aae-1-1687, Aae-1-1688, Aae-1-1689, Aae-1-1690, Aae-1-1691, Aae-1-1692, Aae-1-1693, Aae-1-1694, Aae-1-1695, Aae-1-1696, Aae-1-1697, Aae-1-1698, Aae-1-1699, Aae-1-17, Aae-1-1700, Aae-1-1701, Aae-1-1702, Aae-1-1703, Aae-1-1704, Aae-1-1705, Aae-1-1706, Aae-1-1707, Aae-1-1708, Aae-1-1709, Aae-1-1710, Aae-1-1711, Aae-1-1712, Aae-1-1713, Aae-1-1714, Aae-1-1715, Aae-1-1716, Aae-1-1717, Aae-1-1718, Aae-1-1719, Aae-1-1720, Aae-1-1721, Aae-1-1722, Aae-1-1723, Aae-1-1724, Aae-1-1725, Aae-1-1726, Aae-1-1727, Aae-1-1728, Aae-1-1729, Aae-1-1730, Aae-1-1731, Aae-1-1732, Aae-1-1733, Aae-1-1734, Aae-1-1735, Aae-1-1736, Aae-1-1737, Aae-1-1738, Aae-1-1739, Aae-1-1740, Aae-1-1741, Aae-1-1742, Aae-1-1744, Aae-1-1748, Aae-1-1749, Aae-1-1752, Aae-1-1753, Aae-1-1755, Aae-1-1756, Aae-1-1762, Aae-1-1765, Aae-1-1766, Aae-1-1767, Aae-1-1768, Aae-1-1769, Aae-1-1770, Aae-1-1771, Aae-1-1772, Aae-1-1773, Aae-1-1774, Aae-1-1775, Aae-1-1776, Aae-1-1777, Aae-1-1778, Aae-1-1779, Aae-1-1780, Aae-1-1781, Aae-1-1782, Aae-1-1783, Aae-1-1784, Aae-1-1785, Aae-1-1786, Aae-1-1787, Aae-1-1788, Aae-1-1789, Aae-1-1790, Aae-1-1791, Aae-1-1792, Aae-1-1796, Aae-1-1797, Aae-1-1798, Aae-1-1799, Aae-1-18, Aae-1-1800, Aae-1-1801, Aae-1-1802, Aae-1-1803, Aae-1-1818, Aae-1-1819, Aae-1-1832, Aae-1-1833, Aae-1-1834, Aae-1-1835, Aae-1-1838, Aae-1-1839, Aae-1-1840, Aae-1-1841, Aae-1-1842, Aae-1-1843, Aae-1-1844, Aae-1-1846, Aae-1-1847, Aae-1-1848, Aae-1-1849, Aae-1-1850, Aae-1-1851, Aae-1-1852, Aae-1-1853, Aae-1-1854, Aae-1-1855, Aae-1-1856, Aae-1-1862, Aae-1-1864, Aae-1-1865, Aae-1-1867, Aae-1-1868, Aae-1-1869, Aae-1-1870, Aae-1-1871, Aae-1-1872, Aae-1-1873, Aae-1-1874, Aae-1-1875, Aae-1-1876, Aae-1-1877, Aae-1-1878, Aae-1-1879, Aae-1-1880, Aae-1-1881, Aae-1-1882, Aae-1-1883, Aae-1-1884, Aae-1-1885, Aae-1-1886, Aae-1-1887, Aae-1-1888, Aae-1-1889, Aae-1-1890, Aae-1-1891, Aae-1-1892, Aae-1-1897, Aae-1-1898, Aae-1-19, Aae-1-1903, Aae-1-1904, Aae-1-1905, Aae-1-1906, Aae-1-1907, Aae-1-1908, Aae-1-1909, Aae-1-1910, Aae-1-1917, Aae-1-1918, Aae-1-1919, Aae-1-1920, Aae-1-1921, Aae-1-1922, Aae-1-1923, Aae-1-1924, Aae-1-1925, Aae-1-1926, Aae-1-1927, Aae-1-1928, Aae-1-1930, Aae-1-1932, Aae-1-1933, Aae-1-1934, Aae-1-1935, Aae-1-1936, Aae-1-1938, Aae-1-1942, Aae-1-1943, Aae-1-1944, Aae-1-1945, Aae-1-1946, Aae-1-1958, Aae-1-1960, Aae-1-1977, Aae-1-1978, Aae-1-1979, Aae-1-1984, Aae-1-1985, Aae-1-1988, Aae-1-1989, Aae-1-1990, Aae-1-1991, Aae-1-1992, Aae-1-1998, Aae-1-20, Aae-1-2003, Aae-1-2004, Aae-1-2005, Aae-1-2006, Aae-1-2007, Aae-1-2008, Aae-1-2009, Aae-1-2014, Aae-1-2016, Aae-1-2018, Aae-1-2019, Aae-1-2020, Aae-1-2021, Aae-1-2022, Aae-1-2023, Aae-1-2024, Aae-1-2025, Aae-1-2026, Aae-1-2027, Aae-1-2047, Aae-1-2048, Aae-1-2049, Aae-1-2050, Aae-1-2051, Aae-1-2052, Aae-1-2053, Aae-1-2057, Aae-1-2058, Aae-1-2060, Aae-1-2061, Aae-1-2062, Aae-1-2063, Aae-1-2064, Aae-1-2065, Aae-1-2066, Aae-1-2069, Aae-1-2070, Aae-1-2071, Aae-1-2072, Aae-1-2073, Aae-1-2074, Aae-1-2085, Aae-1-2086, Aae-1-2087, Aae-1-2088, Aae-1-2089, Aae-1-2090, Aae-1-2091, Aae-1-2092, Aae-1-2093, Aae-1-2094, Aae-1-2095, Aae-1-2096, Aae-1-2097, Aae-1-2098, Aae-1-2101, Aae-1-2102, Aae-1-2104, Aae-1-2105, Aae-1-2106, Aae-1-2107, Aae-1-2108, Aae-1-2109, Aae-1-2110, Aae-1-2111, Aae-1-2112, Aae-1-2113, Aae-1-2114, Aae-1-2115, Aae-1-2116, Aae-1-2117, Aae-1-2118, Aae-1-2119, Aae-1-2120, Aae-1-2121, Aae-1-2122, Aae-1-2123, Aae-1-2124, Aae-1-2125, Aae-1-2126, Aae-1-2127, Aae-1-2129, Aae-1-2130, Aae-1-2131, Aae-1-2132, Aae-1-2133, Aae-1-2134, Aae-1-2135, Aae-1-2136, Aae-1-2140, Aae-1-2142, Aae-1-2144, Aae-1-2147, Aae-1-2149, Aae-1-2150, Aae-1-2151, Aae-1-2154, Aae-1-2155, Aae-1-2156, Aae-1-2157, Aae-1-2159, Aae-1-2160, Aae-1-2161, Aae-1-2162, Aae-1-2165, Aae-1-2166, Aae-1-2167, Aae-1-2168, Aae-1-2169, Aae-1-2172, Aae-1-2174, Aae-1-2175, Aae-1-2176, Aae-1-2177, Aae-1-2178, Aae-1-2179, Aae-1-2181, Aae-1-2182, Aae-1-2190, Aae-1-2198, Aae-1-2199, Aae-1-22, Aae-1-2200, Aae-1-2201, Aae-1-2202, Aae-1-2208, Aae-1-2212, Aae-1-2213, Aae-1-2216, Aae-1-2217, Aae-1-2219, Aae-1-2220, Aae-1-2221, Aae-1-2229, Aae-1-2245, Aae-

1-2246, Aae-1-2247, Aae-1-2248, Aae-1-2249, Aae-1-2250, Aae-1-2251, Aae-1-2253, Aae-1-2254, Aae-1-2255, Aae-1-2256, Aae-1-2257, Aae-1-2258, Aae-1-2259, Aae-1-226, Aae-1-2260, Aae-1-2261, Aae-1-2262, Aae-1-2263, Aae-1-2264, Aae-1-2265, Aae-1-2266, Aae-1-2267, Aae-1-2268, Aae-1-2269, Aae-1-2271, Aae-1-2283, Aae-1-2286, Aae-1-2289, Aae-1-2290, Aae-1-2291, Aae-1-2296, Aae-1-2297, Aae-1-2298, Aae-1-2299, Aae-1-2302, Aae-1-2303, Aae-1-2304, Aae-1-2307, Aae-1-2308, Aae-1-2310, Aae-1-2311, Aae-1-2313, Aae-1-2314, Aae-1-2315, Aae-1-2317, Aae-1-2318, Aae-1-2319, Aae-1-2320, Aae-1-2321, Aae-1-2322, Aae-1-2323, Aae-1-2324, Aae-1-2325, Aae-1-2326, Aae-1-2327, Aae-1-2328, Aae-1-2329, Aae-1-2330, Aae-1-2331, Aae-1-2332, Aae-1-2333, Aae-1-2334, Aae-1-2335, Aae-1-2336, Aae-1-2337, Aae-1-2338, Aae-1-2339, Aae-1-2340, Aae-1-2341, Aae-1-2342, Aae-1-2343, Aae-1-2344, Aae-1-2345, Aae-1-2346, Aae-1-2347, Aae-1-2348, Aae-1-2349, Aae-1-2350, Aae-1-2351, Aae-1-2352, Aae-1-2353, Aae-1-2354, Aae-1-2355, Aae-1-2356, Aae-1-2357, Aae-1-2358, Aae-1-2359, Aae-1-2360, Aae-1-2361, Aae-1-2362, Aae-1-2363, Aae-1-2364, Aae-1-2365, Aae-1-2366, Aae-1-2367, Aae-1-2368, Aae-1-2369, Aae-1-2370, Aae-1-2371, Aae-1-2372, Aae-1-2373, Aae-1-2374, Aae-1-2375, Aae-1-2376, Aae-1-2377, Aae-1-2378, Aae-1-2379, Aae-1-238, Aae-1-2380, Aae-1-2381, Aae-1-2382, Aae-1-2383, Aae-1-2384, Aae-1-2385, Aae-1-2386, Aae-1-2387, Aae-1-2388, Aae-1-2389, Aae-1-239, Aae-1-2390, Aae-1-2391, Aae-1-2392, Aae-1-2393, Aae-1-2394, Aae-1-2395, Aae-1-2397, Aae-1-2399, Aae-1-240, Aae-1-241, Aae-1-2414, Aae-1-242, Aae-1-2424, Aae-1-2426, Aae-1-243, Aae-1-2432, Aae-1-2433, Aae-1-244, Aae-1-2444, Aae-1-2446, Aae-1-2447, Aae-1-245, Aae-1-2454, Aae-1-2458, Aae-1-2459, Aae-1-2460, Aae-1-2463, Aae-1-2464, Aae-1-2465, Aae-1-2466, Aae-1-2467, Aae-1-2468, Aae-1-2469, Aae-1-247, Aae-1-2470, Aae-1-2471, Aae-1-2472, Aae-1-2473, Aae-1-2474, Aae-1-2475, Aae-1-2476, Aae-1-2480, Aae-1-2482, Aae-1-2483, Aae-1-2484, Aae-1-2485, Aae-1-2486, Aae-1-2487, Aae-1-2488, Aae-1-2489, Aae-1-2490, Aae-1-2491, Aae-1-2492, Aae-1-2493, Aae-1-2494, Aae-1-2495, Aae-1-2501, Aae-1-2505, Aae-1-251, Aae-1-2510, Aae-1-2512, Aae-1-2513, Aae-1-2514, Aae-1-2515, Aae-1-2516, Aae-1-2519, Aae-1-252, Aae-1-2521, Aae-1-2522, Aae-1-2524, Aae-1-2526, Aae-1-253, Aae-1-2531, Aae-1-2532, Aae-1-2533, Aae-1-2534, Aae-1-2535, Aae-1-2536, Aae-1-2537, Aae-1-2538, Aae-1-2539, Aae-1-2540, Aae-1-2541, Aae-1-2542, Aae-1-2543, Aae-1-2544, Aae-1-2545, Aae-1-2546, Aae-1-2547, Aae-1-2548, Aae-1-2549, Aae-1-2550, Aae-1-2551, Aae-1-2557, Aae-1-2558, Aae-1-2559, Aae-1-2560, Aae-1-2561, Aae-1-2562, Aae-1-2563, Aae-1-2564, Aae-1-2565, Aae-1-2566, Aae-1-2567, Aae-1-2568, Aae-1-2569, Aae-1-2570, Aae-1-2571, Aae-1-2572, Aae-1-2573, Aae-1-2574, Aae-1-2575, Aae-1-2576, Aae-1-2578, Aae-1-2579, Aae-1-2580, Aae-1-2589, Aae-1-2592, Aae-1-2595, Aae-1-2596, Aae-1-2597, Aae-1-2598, Aae-1-2599, Aae-1-2600, Aae-1-2601, Aae-1-2602, Aae-1-2603, Aae-1-2604, Aae-1-2605, Aae-1-2606, Aae-1-2607, Aae-1-2608, Aae-1-2609, Aae-1-2610, Aae-1-2622, Aae-1-2631, Aae-1-2633, Aae-1-2636, Aae-1-2638, Aae-1-2639, Aae-1-2640, Aae-1-2641, Aae-1-2642, Aae-1-2643, Aae-1-2645, Aae-1-2646, Aae-1-2647, Aae-1-2648, Aae-1-2650, Aae-1-2651, Aae-1-2652, Aae-1-2653, Aae-1-2654, Aae-1-2655, Aae-1-2656, Aae-1-2657, Aae-1-2658, Aae-1-2659, Aae-1-2660, Aae-1-2661, Aae-1-2662, Aae-1-2663, Aae-1-2664, Aae-1-2665, Aae-1-2666, Aae-1-2670, Aae-1-2673, Aae-1-2675, Aae-1-2676, Aae-1-2677, Aae-1-2678, Aae-1-2679, Aae-1-2680, Aae-1-2681, Aae-1-2684, Aae-1-2685, Aae-1-2686, Aae-1-2687, Aae-1-2689, Aae-1-2694, Aae-1-2695, Aae-1-2696, Aae-1-2697, Aae-1-2698, Aae-1-2699, Aae-1-2700, Aae-1-2701, Aae-1-2702, Aae-1-2703, Aae-1-2704, Aae-1-2705, Aae-1-2706, Aae-1-2707, Aae-1-2712, Aae-1-2714, Aae-1-2724, Aae-1-2725, Aae-1-2726, Aae-1-2727, Aae-1-2730, Aae-1-2731, Aae-1-2732, Aae-1-2733, Aae-1-2734, Aae-1-2735, Aae-1-2736, Aae-1-2737, Aae-1-2738, Aae-1-2739, Aae-1-2746, Aae-1-2751, Aae-1-2752, Aae-1-2756, Aae-1-2757, Aae-1-2758, Aae-1-2759, Aae-1-2760, Aae-1-2761, Aae-1-2762, Aae-1-2763, Aae-1-2764, Aae-1-2765, Aae-1-2766, Aae-1-2767, Aae-1-2768, Aae-1-2769, Aae-1-2770, Aae-1-2771, Aae-1-2772, Aae-1-2773, Aae-1-2774, Aae-1-2778, Aae-1-2779, Aae-1-2780, Aae-1-2781, Aae-1-2782, Aae-1-2785, Aae-1-2786, Aae-1-2787, Aae-1-2788, Aae-1-2789, Aae-1-2790, Aae-1-2791, Aae-1-2792, Aae-1-2793, Aae-1-2794, Aae-1-2795, Aae-1-2796, Aae-1-2797, Aae-1-2798, Aae-1-2799, Aae-1-2800, Aae-1-2801, Aae-1-2802, Aae-1-2803, Aae-1-2804, Aae-1-2805, Aae-1-2806, Aae-1-2807, Aae-1-2808, Aae-1-2809, Aae-1-2810, Aae-1-2811, Aae-1-2812, Aae-1-2813, Aae-1-2814, Aae-1-2815, Aae-1-2816, Aae-1-2817, Aae-1-2818, Aae-1-2819, Aae-1-2820, Aae-1-2821, Aae-1-2823, Aae-1-2824, Aae-1-2825, Aae-1-2826, Aae-1-2830, Aae-1-2831, Aae-1-2838, Aae-1-2848, Aae-1-2850, Aae-1-2853, Aae-1-2854, Aae-1-2855, Aae-1-2856, Aae-1-2857, Aae-1-2858, Aae-1-2859, Aae-1-2860, Aae-1-2861, Aae-1-2862, Aae-1-2863, Aae-1-2864, Aae-1-2865, Aae-1-2866, Aae-1-2867, Aae-1-2869, Aae-1-2870, Aae-1-2871, Aae-1-2903, Aae-1-2905, Aae-1-2906, Aae-1-2908, Aae-1-2915, Aae-1-2916, Aae-1-2917, Aae-1-2919, Aae-1-2924, Aae-1-3005, Aae-1-3007, Aae-1-3051, Aae-1-3052, Aae-1-3053, Aae-1-3057, Aae-1-3058, Aae-1-3079, Aae-1-3091, Aae-1-3092, Aae-1-3093, Aae-1-3094, Aae-1-3095, Aae-1-3119, Aae-1-314, Aae-1-3169, Aae-1-3170, Aae-1-3171, Aae-1-3178, Aae-1-3179, Aae-1-3180, Aae-1-3181, Aae-1-3195, Aae-1-3196, Aae-1-3207, Aae-1-3208, Aae-1-3214, Aae-1-3219, Aae-1-3222, Aae-1-3223, Aae-1-3224, Aae-1-3226, Aae-1-3227, Aae-1-3228, Aae-1-3229, Aae-1-3230, Aae-1-3231, Aae-1-3232, Aae-1-3233, Aae-1-3234, Aae-1-3238, Aae-1-324, Aae-1-3240, Aae-1-3252, Aae-1-3274, Aae-1-3275, Aae-1-3276, Aae-1-3278, Aae-1-3279, Aae-1-3280, Aae-1-3281, Aae-1-3282, Aae-1-3286, Aae-1-3298, Aae-1-3299, Aae-1-33, Aae-1-3300, Aae-1-3305, Aae-1-3307, Aae-1-3308, Aae-1-3309, Aae-1-3310, Aae-1-3311, Aae-1-3312, Aae-1-3314, Aae-1-3315, Aae-1-3316, Aae-1-3317, Aae-1-3318, Aae-1-3319, Aae-1-332, Aae-1-3326, Aae-1-3327, Aae-1-3328, Aae-1-3341, Aae-1-3347, Aae-1-3348, Aae-1-3349, Aae-1-3350, Aae-1-3351, Aae-1-3363, Aae-1-3364, Aae-1-3369, Aae-1-3373, Aae-1-3374, Aae-1-3375, Aae-1-3376, Aae-1-3377, Aae-1-3378, Aae-1-3382, Aae-1-3387, Aae-1-3390, Aae-1-3391, Aae-1-3398, Aae-1-34, Aae-1-3403, Aae-1-3404, Aae-1-3405, Aae-1-3406, Aae-1-3412, Aae-1-3422, Aae-1-3423, Aae-1-3431, Aae-1-3432, Aae-1-3433, Aae-1-3434, Aae-1-3435, Aae-1-3436, Aae-1-3439, Aae-1-3448, Aae-1-3449, Aae-1-3451, Aae-1-3469, Aae-1-3471, Aae-1-3473, Aae-1-3477, Aae-1-3481, Aae-1-3483, Aae-1-349, Aae-1-3490, Aae-1-3492, Aae-1-3493, Aae-1-3496, Aae-1-35, Aae-1-3503, Aae-1-3506, Aae-1-3507, Aae-1-3514, Aae-1-3517, Aae-1-3518, Aae-1-3519, Aae-1-3520, Aae-1-3521, Aae-1-3522, Aae-1-3523, Aae-1-3525, Aae-1-3529, Aae-1-3530, Aae-1-3531, Aae-1-3540, Aae-1-3541, Aae-1-3542, Aae-1-3543, Aae-1-3547, Aae-1-3554, Aae-1-3556, Aae-1-3557, Aae-1-3558, Aae-1-3559, Aae-1-3560, Aae-1-3561, Aae-1-3562, Aae-1-3567, Aae-1-3570, Aae-1-3572, Aae-1-3573, Aae-1-3575, Aae-1-3578, Aae-1-3579, Aae-1-3580, Aae-1-3581, Aae-1-3582, Aae-1-3585, Aae-1-3586, Aae-1-3587, Aae-1-3588, Aae-1-36, Aae-1-3628, Aae-1-3659, Aae-1-3663, Aae-1-3665,

Aae-1-3675, Aae-1-37, Aae-1-3710, Aae-1-3722, Aae-1-3737, Aae-1-3752, Aae-1-3755, Aae-1-3756, Aae-1-3757, Aae-1-3758, Aae-1-3765, Aae-1-3771, Aae-1-3772, Aae-1-3773, Aae-1-3774, Aae-1-3781, Aae-1-3782, Aae-1-3783, Aae-1-3784, Aae-1-3786, Aae-1-3788, Aae-1-3791, Aae-1-38, Aae-1-3806, Aae-1-3807, Aae-1-3808, Aae-1-3809, Aae-1-3811, Aae-1-3814, Aae-1-3816, Aae-1-3823, Aae-1-3824, Aae-1-3838, Aae-1-3852, Aae-1-3854, Aae-1-3858, Aae-1-386, Aae-1-3861, Aae-1-3867, Aae-1-3869, Aae-1-3873, Aae-1-3874, Aae-1-3875, Aae-1-3876, Aae-1-3877, Aae-1-3879, Aae-1-3880, Aae-1-3882, Aae-1-3884, Aae-1-3885, Aae-1-3888, Aae-1-3889, Aae-1-3895, Aae-1-3896, Aae-1-3897, Aae-1-3908, Aae-1-3909, Aae-1-3910, Aae-1-3911, Aae-1-3912, Aae-1-3913, Aae-1-3914, Aae-1-3915, Aae-1-3916, Aae-1-3917, Aae-1-3918, Aae-1-3919, Aae-1-3920, Aae-1-3921, Aae-1-3922, Aae-1-3923, Aae-1-3924, Aae-1-3925, Aae-1-3926, Aae-1-3927, Aae-1-3928, Aae-1-3929, Aae-1-3930, Aae-1-3931, Aae-1-3932, Aae-1-3933, Aae-1-3934, Aae-1-3935, Aae-1-3936, Aae-1-3937, Aae-1-3938, Aae-1-3939, Aae-1-3940, Aae-1-3941, Aae-1-3942, Aae-1-3943, Aae-1-3944, Aae-1-3945, Aae-1-3946, Aae-1-3947, Aae-1-3948, Aae-1-3949, Aae-1-3950, Aae-1-3951, Aae-1-3952, Aae-1-3953, Aae-1-3954, Aae-1-3955, Aae-1-3956, Aae-1-3957, Aae-1-3958, Aae-1-3959, Aae-1-3960, Aae-1-3961, Aae-1-3962, Aae-1-3963, Aae-1-3964, Aae-1-3965, Aae-1-3966, Aae-1-3967, Aae-1-3968, Aae-1-3969, Aae-1-3970, Aae-1-3971, Aae-1-3972, Aae-1-3973, Aae-1-3974, Aae-1-3975, Aae-1-3976, Aae-1-3977, Aae-1-3978, Aae-1-3979, Aae-1-3980, Aae-1-3981, Aae-1-3982, Aae-1-3983, Aae-1-3984, Aae-1-3985, Aae-1-3986, Aae-1-3987, Aae-1-3988, Aae-1-3989, Aae-1-3990, Aae-1-3991, Aae-1-3992, Aae-1-3993, Aae-1-3994, Aae-1-3995, Aae-1-3996, Aae-1-3997, Aae-1-3998, Aae-1-3999, Aae-1-40, Aae-1-4000, Aae-1-4001, Aae-1-4002, Aae-1-4003, Aae-1-4004, Aae-1-4005, Aae-1-4006, Aae-1-4007, Aae-1-4008, Aae-1-4009, Aae-1-4010, Aae-1-4011, Aae-1-4012, Aae-1-4013, Aae-1-4014, Aae-1-4015, Aae-1-4016, Aae-1-4017, Aae-1-4018, Aae-1-4019, Aae-1-4020, Aae-1-4021, Aae-1-4022, Aae-1-4023, Aae-1-4024, Aae-1-4025, Aae-1-4026, Aae-1-4027, Aae-1-4028, Aae-1-4029, Aae-1-4030, Aae-1-4031, Aae-1-4032, Aae-1-4033, Aae-1-4034, Aae-1-4035, Aae-1-4036, Aae-1-4037, Aae-1-4038, Aae-1-4039, Aae-1-4040, Aae-1-4041, Aae-1-4042, Aae-1-4043, Aae-1-4044, Aae-1-4045, Aae-1-4046, Aae-1-4047, Aae-1-4048, Aae-1-4049, Aae-1-4050, Aae-1-4051, Aae-1-4052, Aae-1-4053, Aae-1-4054, Aae-1-4055, Aae-1-4056, Aae-1-4057, Aae-1-4058, Aae-1-4059, Aae-1-4060, Aae-1-4061, Aae-1-4062, Aae-1-4063, Aae-1-4064, Aae-1-4065, Aae-1-4066, Aae-1-4067, Aae-1-4068, Aae-1-4069, Aae-1-4070, Aae-1-4071, Aae-1-4072, Aae-1-4073, Aae-1-4074, Aae-1-4075, Aae-1-4076, Aae-1-4077, Aae-1-4078, Aae-1-4079, Aae-1-4080, Aae-1-4081, Aae-1-4082, Aae-1-4083, Aae-1-4084, Aae-1-4085, Aae-1-4086, Aae-1-4087, Aae-1-4088, Aae-1-4089, Aae-1-4090, Aae-1-4091, Aae-1-4092, Aae-1-4093, Aae-1-4094, Aae-1-4095, Aae-1-4096, Aae-1-4097, Aae-1-4098, Aae-1-4099, Aae-1-41, Aae-1-4100, Aae-1-4101, Aae-1-4102, Aae-1-4103, Aae-1-4104, Aae-1-4105, Aae-1-4106, Aae-1-4107, Aae-1-4108, Aae-1-4109, Aae-1-4110, Aae-1-4111, Aae-1-4112, Aae-1-4113, Aae-1-4114, Aae-1-4115, Aae-1-4116, Aae-1-4117, Aae-1-4118, Aae-1-4119, Aae-1-4120, Aae-1-4121, Aae-1-4122, Aae-1-4123, Aae-1-4124, Aae-1-4125, Aae-1-4126, Aae-1-4127, Aae-1-4128, Aae-1-4129, Aae-1-4130, Aae-1-4131, Aae-1-4132, Aae-1-4133, Aae-1-4134, Aae-1-4135, Aae-1-4136, Aae-1-4137, Aae-1-4138, Aae-1-4139, Aae-1-4140, Aae-1-4141, Aae-1-4142, Aae-1-4143, Aae-1-4144, Aae-1-4145, Aae-1-4146, Aae-1-4147, Aae-1-4148, Aae-1-4149, Aae-1-4150, Aae-1-4151, Aae-1-4152, Aae-1-4153, Aae-1-4154, Aae-1-4155, Aae-1-4156, Aae-1-4157, Aae-1-4158, Aae-1-4159, Aae-1-4160, Aae-1-4161, Aae-1-4162, Aae-1-4163, Aae-1-4164, Aae-1-4165, Aae-1-4166, Aae-1-4167, Aae-1-4168, Aae-1-4169, Aae-1-4170, Aae-1-4171, Aae-1-4172, Aae-1-4173, Aae-1-4174, Aae-1-4175, Aae-1-4176, Aae-1-4177, Aae-1-4178, Aae-1-4179, Aae-1-4180, Aae-1-4181, Aae-1-4187, Aae-1-4191, Aae-1-4193, Aae-1-4194, Aae-1-4196, Aae-1-4197, Aae-1-4199, Aae-1-42, Aae-1-420, Aae-1-4201, Aae-1-4203, Aae-1-4204, Aae-1-4205, Aae-1-4207, Aae-1-421, Aae-1-4214, Aae-1-4215, Aae-1-4216, Aae-1-4232, Aae-1-4234, Aae-1-4235, Aae-1-4243, Aae-1-4244, Aae-1-4253, Aae-1-4255, Aae-1-4256, Aae-1-4264, Aae-1-4265, Aae-1-4266, Aae-1-4267, Aae-1-4268, Aae-1-4269, Aae-1-4270, Aae-1-4271, Aae-1-4272, Aae-1-4273, Aae-1-4274, Aae-1-4275, Aae-1-4276, Aae-1-4277, Aae-1-4278, Aae-1-4279, Aae-1-4280, Aae-1-4281, Aae-1-4282, Aae-1-4283, Aae-1-4284, Aae-1-4285, Aae-1-4286, Aae-1-4287, Aae-1-4288, Aae-1-4289, Aae-1-4290, Aae-1-4291, Aae-1-4292, Aae-1-4293, Aae-1-4294, Aae-1-4295, Aae-1-4296, Aae-1-4297, Aae-1-4298, Aae-1-4299, Aae-1-43, Aae-1-4300, Aae-1-4301, Aae-1-4302, Aae-1-4303, Aae-1-4304, Aae-1-4305, Aae-1-4306, Aae-1-4307, Aae-1-4308, Aae-1-4309, Aae-1-4310, Aae-1-4311, Aae-1-4312, Aae-1-4313, Aae-1-4323, Aae-1-438, Aae-1-44, Aae-1-441, Aae-1-45, Aae-1-46, Aae-1-468, Aae-1-47, Aae-1-48, Aae-1-482, Aae-1-485, Aae-1-486, Aae-1-488, Aae-1-49, Aae-1-492, Aae-1-493, Aae-1-494, Aae-1-495, Aae-1-496, Aae-1-497, Aae-1-498, Aae-1-499, Aae-1-50, Aae-1-500, Aae-1-501, Aae-1-502, Aae-1-503, Aae-1-504, Aae-1-505, Aae-1-506, Aae-1-507, Aae-1-508, Aae-1-509, Aae-1-51, Aae-1-510, Aae-1-511, Aae-1-512, Aae-1-513, Aae-1-514, Aae-1-515, Aae-1-516, Aae-1-517, Aae-1-518, Aae-1-519, Aae-1-52, Aae-1-520, Aae-1-521, Aae-1-522, Aae-1-523, Aae-1-524, Aae-1-525, Aae-1-526, Aae-1-527, Aae-1-528, Aae-1-529, Aae-1-53, Aae-1-530, Aae-1-531, Aae-1-532, Aae-1-533, Aae-1-534, Aae-1-535, Aae-1-536, Aae-1-537, Aae-1-538, Aae-1-539, Aae-1-54, Aae-1-540, Aae-1-541, Aae-1-542, Aae-1-543, Aae-1-544, Aae-1-545, Aae-1-546, Aae-1-547, Aae-1-548, Aae-1-549, Aae-1-550, Aae-1-551, Aae-1-552, Aae-1-553, Aae-1-554, Aae-1-555, Aae-1-556, Aae-1-557, Aae-1-558, Aae-1-559, Aae-1-56, Aae-1-560, Aae-1-561, Aae-1-562, Aae-1-563, Aae-1-564, Aae-1-565, Aae-1-566, Aae-1-567, Aae-1-568, Aae-1-569, Aae-1-57, Aae-1-570, Aae-1-571, Aae-1-572, Aae-1-573, Aae-1-574, Aae-1-575, Aae-1-576, Aae-1-577, Aae-1-578, Aae-1-579, Aae-1-58, Aae-1-580, Aae-1-581, Aae-1-582, Aae-1-583, Aae-1-584, Aae-1-585, Aae-1-586, Aae-1-587, Aae-1-588, Aae-1-589, Aae-1-59, Aae-1-590, Aae-1-591, Aae-1-592, Aae-1-593, Aae-1-594, Aae-1-595, Aae-1-596, Aae-1-597, Aae-1-598, Aae-1-599, Aae-1-60, Aae-1-600, Aae-1-601, Aae-1-602, Aae-1-603, Aae-1-604, Aae-1-605, Aae-1-606, Aae-1-607, Aae-1-608, Aae-1-609, Aae-1-61, Aae-1-610, Aae-1-611, Aae-1-612, Aae-1-613, Aae-1-614, Aae-1-615, Aae-1-616, Aae-1-617, Aae-1-618, Aae-1-619, Aae-1-62, Aae-1-620, Aae-1-621, Aae-1-622, Aae-1-623, Aae-1-624, Aae-1-625, Aae-1-626, Aae-1-627, Aae-1-628, Aae-1-629, Aae-1-630, Aae-1-631, Aae-1-632, Aae-1-633, Aae-1-634, Aae-1-635, Aae-1-636, Aae-1-637, Aae-1-638, Aae-1-639, Aae-1-640, Aae-1-641, Aae-1-642, Aae-1-643, Aae-1-644, Aae-1-645, Aae-1-646, Aae-1-647, Aae-1-648, Aae-1-649, Aae-1-65, Aae-1-650, Aae-1-651, Aae-1-652, Aae-1-653, Aae-1-654, Aae-1-655, Aae-1-656, Aae-1-657, Aae-1-658, Aae-1-659, Aae-1-66, Aae-1-660, Aae-1-661, Aae-1-662, Aae-1-663, Aae-1-664, Aae-1-665, Aae-1-666, Aae-1-667, Aae-1-668, Aae-1-669, Aae-1-67, Aae-1-670, Aae-1-671, Aae-1-672,

Aae-1-673, Aae-1-674, Aae-1-675, Aae-1-676, Aae-1-677, Aae-1-678, Aae-1-679, Aae-1-680, Aae-1-681, Aae-1-682, Aae-1-683, Aae-1-684, Aae-1-685, Aae-1-686, Aae-1-687, Aae-1-688, Aae-1-689, Aae-1-690, Aae-1-691, Aae-1-692, Aae-1-693, Aae-1-694, Aae-1-695, Aae-1-696, Aae-1-697, Aae-1-698, Aae-1-699, Aae-1-700, Aae-1-701, Aae-1-702, Aae-1-703, Aae-1-704, Aae-1-705, Aae-1-706, Aae-1-707, Aae-1-708, Aae-1-709, Aae-1-710, Aae-1-711, Aae-1-712, Aae-1-713, Aae-1-714, Aae-1-715, Aae-1-716, Aae-1-717, Aae-1-718, Aae-1-719, Aae-1-720, Aae-1-721, Aae-1-722, Aae-1-723, Aae-1-724, Aae-1-725, Aae-1-726, Aae-1-727, Aae-1-728, Aae-1-729, Aae-1-730, Aae-1-731, Aae-1-732, Aae-1-733, Aae-1-734, Aae-1-735, Aae-1-736, Aae-1-737, Aae-1-738, Aae-1-739, Aae-1-740, Aae-1-741, Aae-1-742, Aae-1-743, Aae-1-744, Aae-1-745, Aae-1-746, Aae-1-747, Aae-1-748, Aae-1-749, Aae-1-750, Aae-1-751, Aae-1-752, Aae-1-753, Aae-1-754, Aae-1-755, Aae-1-756, Aae-1-757, Aae-1-758, Aae-1-759, Aae-1-760, Aae-1-761, Aae-1-762, Aae-1-763, Aae-1-764, Aae-1-765, Aae-1-766, Aae-1-767, Aae-1-768, Aae-1-769, Aae-1-770, Aae-1-771, Aae-1-772, Aae-1-773, Aae-1-774, Aae-1-775, Aae-1-776, Aae-1-777, Aae-1-778, Aae-1-779, Aae-1-780, Aae-1-781, Aae-1-782, Aae-1-783, Aae-1-784, Aae-1-785, Aae-1-786, Aae-1-787, Aae-1-788, Aae-1-789, Aae-1-790, Aae-1-791, Aae-1-792, Aae-1-793, Aae-1-794, Aae-1-795, Aae-1-796, Aae-1-797, Aae-1-798, Aae-1-799, Aae-1-8, Aae-1-800, Aae-1-801, Aae-1-802, Aae-1-803, Aae-1-804, Aae-1-805, Aae-1-806, Aae-1-807, Aae-1-808, Aae-1-809, Aae-1-810, Aae-1-811, Aae-1-812, Aae-1-813, Aae-1-814, Aae-1-815, Aae-1-816, Aae-1-817, Aae-1-818, Aae-1-819, Aae-1-820, Aae-1-821, Aae-1-822, Aae-1-823, Aae-1-824, Aae-1-825, Aae-1-826, Aae-1-827, Aae-1-828, Aae-1-829, Aae-1-830, Aae-1-831, Aae-1-832, Aae-1-833, Aae-1-834, Aae-1-835, Aae-1-836, Aae-1-837, Aae-1-838, Aae-1-839, Aae-1-840, Aae-1-841, Aae-1-842, Aae-1-843, Aae-1-844, Aae-1-845, Aae-1-846, Aae-1-847, Aae-1-848, Aae-1-849, Aae-1-850, Aae-1-851, Aae-1-852, Aae-1-853, Aae-1-854, Aae-1-855, Aae-1-856, Aae-1-857, Aae-1-858, Aae-1-859, Aae-1-860, Aae-1-861, Aae-1-862, Aae-1-863, Aae-1-864, Aae-1-865, Aae-1-866, Aae-1-867, Aae-1-868, Aae-1-869, Aae-1-870, Aae-1-871, Aae-1-872, Aae-1-873, Aae-1-874, Aae-1-875, Aae-1-876, Aae-1-877, Aae-1-878, Aae-1-879, Aae-1-880, Aae-1-881, Aae-1-882, Aae-1-883, Aae-1-884, Aae-1-885, Aae-1-886, Aae-1-887, Aae-1-888, Aae-1-889, Aae-1-890, Aae-1-891, Aae-1-892, Aae-1-893, Aae-1-894, Aae-1-895, Aae-1-896, Aae-1-897, Aae-1-898, Aae-1-899, Aae-1-9, Aae-1-900, Aae-1-901, Aae-1-902, Aae-1-903, Aae-1-904, Aae-1-905, Aae-1-906, Aae-1-907, Aae-1-908, Aae-1-909, Aae-1-910, Aae-1-911, Aae-1-912, Aae-1-913, Aae-1-914, Aae-1-915, Aae-1-916, Aae-1-917, Aae-1-918, Aae-1-919, Aae-1-920, Aae-1-921, Aae-1-922, Aae-1-923, Aae-1-924, Aae-1-925, Aae-1-926, Aae-1-927, Aae-1-928, Aae-1-929, Aae-1-930, Aae-1-931, Aae-1-932, Aae-1-933, Aae-1-934, Aae-1-935, Aae-1-936, Aae-1-937, Aae-1-938, Aae-1-939, Aae-1-940, Aae-1-941, Aae-1-942, Aae-1-943, Aae-1-944, Aae-1-945, Aae-1-946, Aae-1-947, Aae-1-948, Aae-1-949, Aae-1-950, Aae-1-951, Aae-1-952, Aae-1-953, Aae-1-954, Aae-1-955, Aae-1-956, Aae-1-957, Aae-1-958, Aae-1-959, Aae-1-960, Aae-1-961, Aae-1-962, Aae-1-963, Aae-1-964, Aae-1-965, Aae-1-966, Aae-1-967, Aae-1-968, Aae-1-969, Aae-1-970, Aae-1-971, Aae-1-972, Aae-1-973, Aae-1-974, Aae-1-975, Aae-1-976, Aae-1-977, Aae-1-978, Aae-1-979, Aae-1-980, Aae-1-981, Aae-1-982, Aae-1-983, Aae-1-984, Aae-1-985, Aae-1-986, Aae-1-987, Aae-1-988, Aae-1-989, Aae-1-990, Aae-1-991, Aae-1-992, Aae-1-993, Aae-1-994, Aae-1-995, Aae-1-996, Aae-1-997, Aae-1-998, Aae-1-999, Ama-1-2, Bfl-1-10, Bfl-1-100, Bfl-1-101, Bfl-1-102, Bfl-1-103, Bfl-1-104, Bfl-1-105, Bfl-1-106, Bfl-1-107, Bfl-1-108, Bfl-1-109, Bfl-1-11, Bfl-1-110, Bfl-1-111, Bfl-1-112, Bfl-1-113, Bfl-1-114, Bfl-1-115, Bfl-1-116, Bfl-1-117, Bfl-1-118, Bfl-1-119, Bfl-1-120, Bfl-1-121, Bfl-1-122, Bfl-1-123, Bfl-1-124, Bfl-1-125, Bfl-1-126, Bfl-1-127, Bfl-1-128, Bfl-1-129, Bfl-1-130, Bfl-1-131, Bfl-1-132, Bfl-1-133, Bfl-1-134, Bfl-1-135, Bfl-1-136, Bfl-1-17, Bfl-1-18, Bfl-1-19, Bfl-1-20, Bfl-1-21, Bfl-1-22, Bfl-1-23, Bfl-1-24, Bfl-1-25, Bfl-1-26, Bfl-1-27, Bfl-1-28, Bfl-1-29, Bfl-1-30, Bfl-1-31, Bfl-1-32, Bfl-1-33, Bfl-1-34, Bfl-1-35, Bfl-1-36, Bfl-1-37, Bfl-1-38, Bfl-1-39, Bfl-1-40, Bfl-1-41, Bfl-1-42, Bfl-1-43, Bfl-1-44, Bfl-1-45, Bfl-1-46, Bfl-1-47, Bfl-1-48, Bfl-1-49, Bfl-1-50, Bfl-1-51, Bfl-1-52, Bfl-1-53, Bfl-1-54, Bfl-1-55, Bfl-1-56, Bfl-1-57, Bfl-1-58, Bfl-1-59, Bfl-1-60, Bfl-1-62, Bfl-1-63, Bfl-1-64, Bfl-1-65, Bfl-1-66, Bfl-1-67, Bfl-1-68, Bfl-1-69, Bfl-1-70, Bfl-1-71, Bfl-1-72, Bfl-1-76, Bfl-1-79, Bfl-1-80, Bfl-1-81, Bfl-1-82, Bfl-1-83, Bfl-1-84, Bfl-1-85, Bfl-1-86, Bfl-1-87, Bfl-1-88, Bfl-1-89, Bfl-1-90, Bfl-1-91, Bfl-1-92, Bfl-1-93, Bfl-1-94, Bfl-1-95, Bfl-1-96, Bfl-1-97, Bfl-1-98, Bfl-1-99, Bta-1-2, Bta-1-4, Bta-1-5, Bta-1-6, Clu-1-2, Clu-1-3, Clu-1-4, Cpe-1-1, Cpe-2-1, Cpe-3-1, Cpe-4-1, Cpe-5-1, Cqu-1-13, Cqu-1-14, Cqu-1-5, Cqu-1-6, Cqu-1-7, Csp-1-2, Dre-1-2, Eca-1-10, Eca-1-12, Eca-1-14, Eca-1-16, Eca-1-18, Eca-1-2, Eca-1-4, Eca-1-6, Eca-1-8, Ehi-1-1, Ehi-1-2, Ehi-1-3, Fpr-1-2, Gga-1-2, Gga-1-4, Hma-1-4, Hma-1-5, Hma-1-6, Hma-1-8, Hsa-1-1, Hsa-1-10, Hsa-1-11, Hsa-1-12, Hsa-1-13, Hsa-1-14, Hsa-1-15, Hsa-1-16, Hsa-1-17, Hsa-1-18, Hsa-1-19, Hsa-1-2, Hsa-1-21, Hsa-1-22, Hsa-1-23, Hsa-1-24, Hsa-1-25, Hsa-1-26, Hsa-1-27, Hsa-1-28, Hsa-1-29, Hsa-1-3, Hsa-1-30, Hsa-1-31, Hsa-1-4, Hsa-1-5, Hsa-1-6, Hsa-1-8, Hsa-1-9, Isc-1-13, Isc-1-16, Isc-1-18, Isc-1-19, Isc-1-21, Isc-1-22, Isc-1-28, Isc-1-29, Isc-1-30, Isc-1-32, Isc-1-33, Isc-1-35, Isc-1-36, Isc-1-37, Isc-1-38, Isc-1-39, Isc-1-40, Isc-1-41, Isc-1-42, Isc-1-43, Isc-1-44, Isc-1-45, Isc-1-46, Lbi-1-3, Ldr-1-10, Ldr-1-12, Ldr-1-14, Ldr-1-16, Ldr-1-18, Ldr-1-2, Ldr-1-3, Ldr-1-4, Ldr-1-6, Ldr-1-8, Lpn-1-1, Lpn-3-1, Lva-1-2, Mdo-1-10, Mdo-1-12, Mdo-1-14, Mdo-1-16, Mdo-1-18, Mdo-1-2, Mdo-1-20, Mdo-1-22, Mdo-1-24, Mdo-1-26, Mdo-1-4, Mdo-1-6, Mdo-1-8, Mmu-1-10, Mmu-1-11, Mmu-1-12, Mmu-1-14, Mmu-1-16, Mmu-1-17, Mmu-1-18, Mmu-1-2, Mmu-1-20, Mmu-1-21, Mmu-1-22, Mmu-1-24, Mmu-1-25, Mmu-1-26, Mmu-1-28, Mmu-1-29, Mmu-1-3, Mmu-1-30, Mmu-1-32, Mmu-1-33, Mmu-1-34, Mmu-1-4, Mmu-1-6, Mmu-1-7, Mmu-1-8, Mmu-2-10, Mmu-2-13, Mmu-2-2, Mmu-2-4, Mmu-2-6, Mmu-2-8, Nve-1-100, Nve-1-101, Nve-1-102, Nve-1-103, Nve-1-104, Nve-1-108, Nve-1-126, Nve-1-128, Nve-1-137, Nve-1-139, Nve-1-14, Nve-1-140, Nve-1-141, Nve-1-142, Nve-1-143, Nve-1-144, Nve-1-145, Nve-1-146, Nve-1-147, Nve-1-148, Nve-1-149, Nve-1-151, Nve-1-152, Nve-1-154, Nve-1-156, Nve-1-157, Nve-1-158, Nve-1-159, Nve-1-16, Nve-1-160, Nve-1-161, Nve-1-162, Nve-1-163, Nve-1-164, Nve-1-165, Nve-1-166, Nve-1-167, Nve-1-17, Nve-1-176, Nve-1-177, Nve-1-181, Nve-1-185, Nve-1-190, Nve-1-200, Nve-1-204, Nve-1-211, Nve-1-217, Nve-1-218, Nve-1-220, Nve-1-223, Nve-1-224, Nve-1-225, Nve-1-226, Nve-1-227, Nve-1-228, Nve-1-229, Nve-1-230, Nve-1-231, Nve-1-232, Nve-1-233, Nve-1-234, Nve-1-235, Nve-1-236, Nve-1-237, Nve-1-238, Nve-1-239, Nve-1-240, Nve-1-241, Nve-1-242, Nve-1-243, Nve-1-244, Nve-1-245, Nve-1-246, Nve-1-247, Nve-1-248, Nve-1-249, Nve-1-250, Nve-1-251, Nve-1-252, Nve-1-253, Nve-1-254, Nve-1-255, Nve-1-256, Nve-1-257, Nve-1-258, Nve-1-259, Nve-1-26, Nve-1-261, Nve-1-262, Nve-1-263, Nve-1-264, Nve-1-265, Nve-1-266, Nve-1-270, Nve-1-272, Nve-1-274, Nve-1-277, Nve-1-278, Nve-1-28, Nve-1-283, Nve-1-284, Nve-1-285, Nve-1-288, Nve-1-290,

Nve-1-296, Nve-1-297, Nve-1-30, Nve-1-300, Nve-1-302, Nve-1-303, Nve-1-307, Nve-1-308, Nve-1-313, Nve-1-314, Nve-1-317, Nve-1-319, Nve-1-32, Nve-1-320, Nve-1-321, Nve-1-327, Nve-1-329, Nve-1-333, Nve-1-334, Nve-1-336, Nve-1-339, Nve-1-34, Nve-1-341, Nve-1-343, Nve-1-344, Nve-1-348, Nve-1-35, Nve-1-352, Nve-1-36, Nve-1-360, Nve-1-361, Nve-1-362, Nve-1-363, Nve-1-364, Nve-1-365, Nve-1-366, Nve-1-367, Nve-1-368, Nve-1-369, Nve-1-370, Nve-1-371, Nve-1-375, Nve-1-376, Nve-1-377, Nve-1-379, Nve-1-38, Nve-1-380, Nve-1-381, Nve-1-388, Nve-1-389, Nve-1-39, Nve-1-390, Nve-1-391, Nve-1-392, Nve-1-393, Nve-1-394, Nve-1-395, Nve-1-397, Nve-1-398, Nve-1-400, Nve-1-401, Nve-1-405, Nve-1-407, Nve-1-408, Nve-1-409, Nve-1-41, Nve-1-410, Nve-1-411, Nve-1-415, Nve-1-42, Nve-1-420, Nve-1-424, Nve-1-426, Nve-1-427, Nve-1-428, Nve-1-43, Nve-1-430, Nve-1-431, Nve-1-432, Nve-1-434, Nve-1-437, Nve-1-438, Nve-1-439, Nve-1-44, Nve-1-440, Nve-1-441, Nve-1-442, Nve-1-444, Nve-1-447, Nve-1-449, Nve-1-45, Nve-1-451, Nve-1-454, Nve-1-455, Nve-1-456, Nve-1-459, Nve-1-46, Nve-1-460, Nve-1-465, Nve-1-47, Nve-1-48, Nve-1-49, Nve-1-50, Nve-1-51, Nve-1-52, Nve-1-53, Nve-1-54, Nve-1-55, Nve-1-56, Nve-1-57, Nve-1-58, Nve-1-59, Nve-1-60, Nve-1-61, Nve-1-62, Nve-1-63, Nve-1-64, Nve-1-65, Nve-1-66, Nve-1-67, Nve-1-68, Nve-1-69, Nve-1-70, Nve-1-71, Nve-1-72, Nve-1-73, Nve-1-74, Nve-1-75, Nve-1-76, Nve-1-77, Nve-1-78, Nve-1-79, Nve-1-80, Nve-1-81, Nve-1-82, Nve-1-83, Nve-1-84, Nve-1-85, Nve-1-86, Nve-1-87, Nve-1-88, Nve-1-89, Nve-1-90, Nve-1-91, Nve-1-92, Nve-1-93, Nve-1-94, Nve-1-95, Nve-1-96, Nve-1-97, Nve-1-98, Nve-1-99, Ppl-1-10, Ppl-1-11, Ppl-1-12, Ppl-1-14, Ppl-1-15, Ppl-1-16, Ppl-1-17, Ppl-1-18, Ppl-1-19, Ppl-1-2, Ppl-1-20, Ppl-1-22, Ppl-1-23, Ppl-1-24, Ppl-1-25, Ppl-1-29, Ppl-1-3, Ppl-1-30, Ppl-1-31, Ppl-1-32, Ppl-1-33, Ppl-1-34, Ppl-1-35, Ppl-1-37, Ppl-1-38, Ppl-1-39, Ppl-1-8, Ppl-1-9, Ptr-1-10, Ptr-1-12, Ptr-1-13, Ptr-1-14, Ptr-1-2, Ptr-1-4, Ptr-1-6, Ptr-1-8, Rno-1-10, Rno-1-11, Rno-1-12, Rno-1-14, Rno-1-15, Rno-1-16, Rno-1-18, Rno-1-19, Rno-1-2, Rno-1-20, Rno-1-22, Rno-1-23, Rno-1-24, Rno-1-3, Rno-1-4, Rno-1-6, Rno-1-7, Rno-1-8, Sma-1-10, Sma-1-100, Sma-1-1000, Sma-1-10000, Sma-1-10001, Sma-1-10002, Sma-1-10003, Sma-1-10004, Sma-1-10005, Sma-1-10006, Sma-1-10007, Sma-1-10008, Sma-1-10009, Sma-1-10010, Sma-1-10011, Sma-1-10012, Sma-1-10013, Sma-1-10014, Sma-1-10015, Sma-1-10016, Sma-1-10017, Sma-1-10018, Sma-1-10019, Sma-1-1002, Sma-1-10020, Sma-1-10021, Sma-1-10022, Sma-1-10023, Sma-1-10024, Sma-1-10025, Sma-1-10026, Sma-1-10027, Sma-1-10028, Sma-1-10029, Sma-1-10030, Sma-1-10031, Sma-1-10032, Sma-1-10033, Sma-1-10034, Sma-1-10035, Sma-1-10036, Sma-1-10037, Sma-1-10038, Sma-1-10039, Sma-1-1004, Sma-1-10040, Sma-1-10041, Sma-1-10042, Sma-1-10043, Sma-1-10044, Sma-1-10045, Sma-1-10046, Sma-1-10047, Sma-1-10048, Sma-1-10049, Sma-1-10050, Sma-1-10051, Sma-1-10052, Sma-1-10053, Sma-1-10054, Sma-1-10055, Sma-1-10056, Sma-1-10057, Sma-1-10058, Sma-1-10059, Sma-1-1006, Sma-1-10060, Sma-1-10061, Sma-1-10062, Sma-1-10063, Sma-1-10064, Sma-1-10065, Sma-1-10066, Sma-1-10067, Sma-1-10068, Sma-1-10069, Sma-1-10070, Sma-1-10071, Sma-1-10072, Sma-1-10073, Sma-1-10074, Sma-1-10075, Sma-1-10076, Sma-1-10077, Sma-1-10078, Sma-1-10079, Sma-1-1008, Sma-1-10080, Sma-1-10081, Sma-1-10082, Sma-1-10083, Sma-1-10084, Sma-1-10085, Sma-1-10086, Sma-1-10087, Sma-1-10088, Sma-1-10089, Sma-1-10090, Sma-1-10091, Sma-1-10092, Sma-1-10093, Sma-1-10094, Sma-1-10095, Sma-1-10096, Sma-1-10097, Sma-1-10098, Sma-1-10099, Sma-1-1010, Sma-1-10100, Sma-1-10101, Sma-1-10102, Sma-1-10103, Sma-1-10104, Sma-1-10105, Sma-1-10106, Sma-1-10107, Sma-1-10108, Sma-1-10109, Sma-1-10110, Sma-1-10111, Sma-1-10112, Sma-1-10113, Sma-1-10114, Sma-1-10115, Sma-1-10116, Sma-1-10117, Sma-1-10118, Sma-1-10119, Sma-1-1012, Sma-1-10120, Sma-1-10121, Sma-1-10122, Sma-1-10123, Sma-1-10124, Sma-1-10125, Sma-1-10126, Sma-1-10127, Sma-1-10128, Sma-1-10129, Sma-1-10130, Sma-1-10131, Sma-1-10132, Sma-1-10133, Sma-1-10134, Sma-1-10135, Sma-1-10136, Sma-1-10137, Sma-1-10138, Sma-1-10139, Sma-1-1014, Sma-1-10140, Sma-1-10141, Sma-1-10142, Sma-1-10143, Sma-1-10144, Sma-1-10145, Sma-1-10146, Sma-1-10147, Sma-1-10148, Sma-1-10149, Sma-1-10150, Sma-1-10151, Sma-1-10152, Sma-1-10153, Sma-1-10154, Sma-1-10155, Sma-1-10156, Sma-1-10157, Sma-1-10158, Sma-1-10159, Sma-1-1016, Sma-1-10160, Sma-1-10161, Sma-1-10162, Sma-1-10163, Sma-1-10164, Sma-1-10165, Sma-1-10166, Sma-1-10167, Sma-1-10168, Sma-1-10169, Sma-1-10170, Sma-1-10171, Sma-1-10172, Sma-1-10173, Sma-1-10174, Sma-1-10175, Sma-1-10176, Sma-1-10177, Sma-1-10178, Sma-1-10179, Sma-1-1018, Sma-1-10180, Sma-1-10181, Sma-1-10182, Sma-1-10183, Sma-1-10184, Sma-1-10185, Sma-1-10186, Sma-1-10187, Sma-1-10188, Sma-1-10189, Sma-1-10190, Sma-1-10191, Sma-1-10192, Sma-1-10193, Sma-1-10194, Sma-1-10195, Sma-1-10196, Sma-1-10197, Sma-1-10198, Sma-1-10199, Sma-1-102, Sma-1-1020, Sma-1-10200, Sma-1-10201, Sma-1-10202, Sma-1-10203, Sma-1-10204, Sma-1-10205, Sma-1-10206, Sma-1-10207, Sma-1-10208, Sma-1-10209, Sma-1-10210, Sma-1-10211, Sma-1-10212, Sma-1-10213, Sma-1-10214, Sma-1-10215, Sma-1-10216, Sma-1-10217, Sma-1-10218, Sma-1-10219, Sma-1-1022, Sma-1-10220, Sma-1-10221, Sma-1-10222, Sma-1-10223, Sma-1-10224, Sma-1-10225, Sma-1-10226, Sma-1-10227, Sma-1-10228, Sma-1-10229, Sma-1-10230, Sma-1-10231, Sma-1-10232, Sma-1-10233, Sma-1-10234, Sma-1-10235, Sma-1-10236, Sma-1-10237, Sma-1-10238, Sma-1-10239, Sma-1-1024, Sma-1-10240, Sma-1-10241, Sma-1-10242, Sma-1-10243, Sma-1-10244, Sma-1-10245, Sma-1-10246, Sma-1-10247, Sma-1-10248, Sma-1-10249, Sma-1-10250, Sma-1-10251, Sma-1-10252, Sma-1-10253, Sma-1-10254, Sma-1-10255, Sma-1-10256, Sma-1-10257, Sma-1-10258, Sma-1-10259, Sma-1-1026, Sma-1-10260, Sma-1-10261, Sma-1-10262, Sma-1-10263, Sma-1-10264, Sma-1-10265, Sma-1-10266, Sma-1-10267, Sma-1-10268, Sma-1-10269, Sma-1-10270, Sma-1-10271, Sma-1-10272, Sma-1-10273, Sma-1-10274, Sma-1-10275, Sma-1-10276, Sma-1-10277, Sma-1-10278, Sma-1-10279, Sma-1-1028, Sma-1-10280, Sma-1-10281, Sma-1-10282, Sma-1-10283, Sma-1-10284, Sma-1-10285, Sma-1-10286, Sma-1-10287, Sma-1-10288, Sma-1-10289, Sma-1-10290, Sma-1-10291, Sma-1-10292, Sma-1-10293, Sma-1-10294, Sma-1-10295, Sma-1-10296, Sma-1-10297, Sma-1-10298, Sma-1-10299, Sma-1-1030, Sma-1-10300, Sma-1-10301, Sma-1-10302, Sma-1-10303, Sma-1-10304, Sma-1-10305, Sma-1-10306, Sma-1-10307, Sma-1-10308, Sma-1-10309, Sma-1-10310, Sma-1-10311, Sma-1-10312, Sma-1-10313, Sma-1-10314, Sma-1-10315, Sma-1-10316, Sma-1-10317, Sma-1-10318, Sma-1-10319, Sma-1-1032, Sma-1-10320, Sma-1-10321, Sma-1-10322, Sma-1-10323, Sma-1-10324, Sma-1-10325, Sma-1-10326, Sma-1-10327, Sma-1-10328, Sma-1-10329, Sma-1-10330, Sma-1-10331, Sma-1-10332, Sma-1-10333, Sma-1-10334, Sma-1-10335, Sma-1-10336, Sma-1-10337, Sma-1-10338, Sma-1-10339, Sma-1-1034, Sma-1-10340, Sma-1-10341, Sma-1-10342, Sma-1-10343, Sma-1-10344, Sma-1-10345, Sma-1-10346, Sma-1-10347, Sma-1-10348, Sma-1-10349, Sma-1-10350, Sma-1-10351, Sma-1-10352, Sma-1-10353, Sma-1-10354, Sma-1-10355,

Sma-1-10356, Sma-1-10357, Sma-1-10358, Sma-1-10359, Sma-1-1036, Sma-1-10360, Sma-1-10361, Sma-1-10362, Sma-1-10363, Sma-1-10364, Sma-1-10365, Sma-1-10366,
Sma-1-10367, Sma-1-10368, Sma-1-10369, Sma-1-10370, Sma-1-10371, Sma-1-10372, Sma-1-10373, Sma-1-10374, Sma-1-10375, Sma-1-10376, Sma-1-10377, Sma-1-10378,
Sma-1-10379, Sma-1-1038, Sma-1-10380, Sma-1-10381, Sma-1-10382, Sma-1-10383, Sma-1-10384, Sma-1-10385, Sma-1-10386, Sma-1-10387, Sma-1-10388, Sma-1-10389, Sma-
1-10390, Sma-1-10391, Sma-1-10392, Sma-1-10393, Sma-1-10394, Sma-1-10395, Sma-1-10396, Sma-1-10397, Sma-1-10398, Sma-1-10399, Sma-1-104, Sma-1-1040, Sma-1-10400,
Sma-1-10401, Sma-1-10402, Sma-1-10403, Sma-1-10404, Sma-1-10405, Sma-1-10406, Sma-1-10407, Sma-1-10408, Sma-1-10409, Sma-1-10410, Sma-1-10411, Sma-1-10412,
Sma-1-10413, Sma-1-10414, Sma-1-10415, Sma-1-10416, Sma-1-10417, Sma-1-10418, Sma-1-10419, Sma-1-1042, Sma-1-10420, Sma-1-10421, Sma-1-10422, Sma-1-10423,
Sma-1-10424, Sma-1-10425, Sma-1-10426, Sma-1-10427, Sma-1-10428, Sma-1-10429, Sma-1-10430, Sma-1-10431, Sma-1-10432, Sma-1-10433, Sma-1-10434, Sma-1-10435,
Sma-1-10436, Sma-1-10437, Sma-1-10438, Sma-1-10439, Sma-1-1044, Sma-1-10440, Sma-1-10441, Sma-1-10442, Sma-1-10443, Sma-1-10444, Sma-1-10445, Sma-1-10446,
Sma-1-10447, Sma-1-10448, Sma-1-10449, Sma-1-10450, Sma-1-10451, Sma-1-10452, Sma-1-10453, Sma-1-10454, Sma-1-10455, Sma-1-10456, Sma-1-10457, Sma-1-10458,
Sma-1-10459, Sma-1-1046, Sma-1-10460, Sma-1-10461, Sma-1-10462, Sma-1-10463, Sma-1-10464, Sma-1-10465, Sma-1-10466, Sma-1-10467, Sma-1-10468, Sma-1-10469,
Sma-1-10470, Sma-1-10471, Sma-1-10472, Sma-1-10473, Sma-1-10474, Sma-1-10475, Sma-1-10476, Sma-1-10477, Sma-1-10478, Sma-1-10479, Sma-1-1048, Sma-1-10480,
Sma-1-10481, Sma-1-10482, Sma-1-10483, Sma-1-10484, Sma-1-10485, Sma-1-10486, Sma-1-10487, Sma-1-10488, Sma-1-10489, Sma-1-10490, Sma-1-10491, Sma-1-10492,
Sma-1-10493, Sma-1-10494, Sma-1-10495, Sma-1-10496, Sma-1-10497, Sma-1-10498, Sma-1-10499, Sma-1-1050, Sma-1-10500, Sma-1-10501, Sma-1-10502, Sma-1-10503,
Sma-1-10504, Sma-1-10505, Sma-1-10506, Sma-1-10507, Sma-1-10508, Sma-1-10509, Sma-1-10510, Sma-1-10511, Sma-1-10512, Sma-1-10513, Sma-1-10514, Sma-1-10515,
Sma-1-10516, Sma-1-10517, Sma-1-10518, Sma-1-10519, Sma-1-1052, Sma-1-10520, Sma-1-10521, Sma-1-10522, Sma-1-10523, Sma-1-10524, Sma-1-10525, Sma-1-10526,
Sma-1-10527, Sma-1-10528, Sma-1-10529, Sma-1-10530, Sma-1-10531, Sma-1-10532, Sma-1-10533, Sma-1-10534, Sma-1-10535, Sma-1-10536, Sma-1-10537, Sma-1-10538,
Sma-1-10539, Sma-1-1054, Sma-1-10540, Sma-1-10541, Sma-1-10542, Sma-1-10543, Sma-1-10544, Sma-1-10545, Sma-1-10546, Sma-1-10547, Sma-1-10548, Sma-1-10549,
Sma-1-10550, Sma-1-10551, Sma-1-10552, Sma-1-10553, Sma-1-10554, Sma-1-10555, Sma-1-10556, Sma-1-10557, Sma-1-10558, Sma-1-10559, Sma-1-1056, Sma-1-10560,
Sma-1-10561, Sma-1-10562, Sma-1-10563, Sma-1-10564, Sma-1-10565, Sma-1-10566, Sma-1-10567, Sma-1-10568, Sma-1-10569, Sma-1-10570, Sma-1-10571, Sma-1-10572,
Sma-1-10573, Sma-1-10574, Sma-1-10575, Sma-1-10576, Sma-1-10577, Sma-1-10578, Sma-1-10579, Sma-1-1058, Sma-1-10580, Sma-1-10581, Sma-1-10582, Sma-1-10583, Sma-
1-10584, Sma-1-10585, Sma-1-10586, Sma-1-10587, Sma-1-10588, Sma-1-10589, Sma-1-10590, Sma-1-10591, Sma-1-10592, Sma-1-10593, Sma-1-10594, Sma-1-10595, Sma-1-
10596, Sma-1-10597, Sma-1-10598, Sma-1-10599, Sma-1-106, Sma-1-1060, Sma-1-10600, Sma-1-10601, Sma-1-10602, Sma-1-10603, Sma-1-10604, Sma-1-10605, Sma-1-10606,
Sma-1-10607, Sma-1-10608, Sma-1-10609, Sma-1-10610, Sma-1-10611, Sma-1-10612, Sma-1-10613, Sma-1-10614, Sma-1-10615, Sma-1-10616, Sma-1-10617, Sma-1-10618,
Sma-1-10619, Sma-1-1062, Sma-1-10620, Sma-1-10621, Sma-1-10622, Sma-1-10623, Sma-1-10624, Sma-1-10625, Sma-1-10626, Sma-1-10627, Sma-1-10628, Sma-1-10629,
Sma-1-10630, Sma-1-10631, Sma-1-10632, Sma-1-10633, Sma-1-10634, Sma-1-10635, Sma-1-10636, Sma-1-10637, Sma-1-10638, Sma-1-10639, Sma-1-1064, Sma-1-10640,
Sma-1-10641, Sma-1-10642, Sma-1-10643, Sma-1-10644, Sma-1-10645, Sma-1-10646, Sma-1-10647, Sma-1-10648, Sma-1-10649, Sma-1-10650, Sma-1-10651, Sma-1-10652,
Sma-1-10653, Sma-1-10654, Sma-1-10655, Sma-1-10656, Sma-1-10657, Sma-1-10658, Sma-1-10659, Sma-1-1066, Sma-1-10660, Sma-1-10661, Sma-1-10662, Sma-1-10663,
Sma-1-10664, Sma-1-10665, Sma-1-10666, Sma-1-10667, Sma-1-10668, Sma-1-10669, Sma-1-10670, Sma-1-10671, Sma-1-10672, Sma-1-10673, Sma-1-10675, Sma-1-10677,
Sma-1-10678, Sma-1-10679, Sma-1-1068, Sma-1-10680, Sma-1-10681, Sma-1-10683, Sma-1-10685, Sma-1-10687, Sma-1-10689, Sma-1-10690, Sma-1-10691, Sma-1-10693,
Sma-1-10695, Sma-1-10696, Sma-1-10697, Sma-1-10699, Sma-1-1070, Sma-1-10701, Sma-1-10702, Sma-1-10703, Sma-1-10704, Sma-1-10705, Sma-1-10706, Sma-1-10707,
Sma-1-10709, Sma-1-10710, Sma-1-10711, Sma-1-10713, Sma-1-10715, Sma-1-10717, Sma-1-10719, Sma-1-1072, Sma-1-10721, Sma-1-10723, Sma-1-10725, Sma-1-10727,
Sma-1-10729, Sma-1-1073, Sma-1-10731, Sma-1-10733, Sma-1-10735, Sma-1-10736, Sma-1-10737, Sma-1-10739, Sma-1-1074, Sma-1-10741, Sma-1-10742, Sma-1-10743, Sma-
1-10744, Sma-1-10745, Sma-1-10746, Sma-1-10747, Sma-1-10749, Sma-1-10750, Sma-1-10751, Sma-1-10753, Sma-1-10755, Sma-1-10757, Sma-1-10759, Sma-1-1076, Sma-
1-10760, Sma-1-10761, Sma-1-10763, Sma-1-10765, Sma-1-10767, Sma-1-10769, Sma-1-10770, Sma-1-10771, Sma-1-10772, Sma-1-10773, Sma-1-10775, Sma-1-10776, Sma-
1-10777, Sma-1-10778, Sma-1-10779, Sma-1-1078, Sma-1-10780, Sma-1-10781, Sma-1-10782, Sma-1-10783, Sma-1-10784, Sma-1-10785, Sma-1-10786, Sma-1-10787, Sma-1-
10788, Sma-1-10789, Sma-1-10791, Sma-1-10792, Sma-1-10793, Sma-1-10795, Sma-1-10797, Sma-1-10798, Sma-1-10799, Sma-1-108, Sma-1-1080, Sma-1-10800, Sma-1-10801,
Sma-1-10802, Sma-1-10803, Sma-1-10804, Sma-1-10805, Sma-1-10807, Sma-1-10809, Sma-1-10810, Sma-1-10811, Sma-1-10812, Sma-1-10813, Sma-1-10815, Sma-1-10817,
Sma-1-10819, Sma-1-1082, Sma-1-10820, Sma-1-10821, Sma-1-10823, Sma-1-10825, Sma-1-10827, Sma-1-10828, Sma-1-10829, Sma-1-10831, Sma-1-10833, Sma-1-10835,
Sma-1-10837, Sma-1-10839, Sma-1-1084, Sma-1-10841, Sma-1-10842, Sma-1-10843, Sma-1-10845, Sma-1-10846, Sma-1-10847, Sma-1-10849, Sma-1-10851, Sma-1-10852,
Sma-1-10853, Sma-1-10855, Sma-1-10857, Sma-1-10858, Sma-1-10859, Sma-1-1086, Sma-1-10861, Sma-1-10863, Sma-1-10865, Sma-1-10867, Sma-1-10869, Sma-1-10871, Sma-
1-10873, Sma-1-10874, Sma-1-10875, Sma-1-10876, Sma-1-10877, Sma-1-10879, Sma-1-1088, Sma-1-10881, Sma-1-10883, Sma-1-10885, Sma-1-10886, Sma-1-10887, Sma-1-
10888, Sma-1-10889, Sma-1-10891, Sma-1-10893, Sma-1-10895, Sma-1-10897, Sma-1-10899, Sma-1-109, Sma-1-1090, Sma-1-10901, Sma-1-10903, Sma-1-10905, Sma-1-10906,
Sma-1-10907, Sma-1-10909, Sma-1-10911, Sma-1-10912, Sma-1-10913, Sma-1-10914, Sma-1-10915, Sma-1-10916, Sma-1-10917, Sma-1-10919, Sma-1-1092, Sma-1-10921,
Sma-1-10922, Sma-1-10923, Sma-1-10925, Sma-1-10927, Sma-1-10929, Sma-1-10931, Sma-1-10933, Sma-1-10934, Sma-1-10935, Sma-1-10936, Sma-1-10937, Sma-1-10939,

Sma-1-1094, Sma-1-10941, Sma-1-10943, Sma-1-10945, Sma-1-10946, Sma-1-10947, Sma-1-10949, Sma-1-10951, Sma-1-10953, Sma-1-10954, Sma-1-10955, Sma-1-10956, Sma-1-10957, Sma-1-10958, Sma-1-10959, Sma-1-1096, Sma-1-10960, Sma-1-10961, Sma-1-10962, Sma-1-10963, Sma-1-10964, Sma-1-10965, Sma-1-10966, Sma-1-10967, Sma-1-10968, Sma-1-10969, Sma-1-10970, Sma-1-10971, Sma-1-10972, Sma-1-10973, Sma-1-10974, Sma-1-10975, Sma-1-10976, Sma-1-10977, Sma-1-10978, Sma-1-10979, Sma-1-1098, Sma-1-10980, Sma-1-10981, Sma-1-10982, Sma-1-10983, Sma-1-10985, Sma-1-10987, Sma-1-10989, Sma-1-10991, Sma-1-10992, Sma-1-10993, Sma-1-10994, Sma-1-10995, Sma-1-10996, Sma-1-10997, Sma-1-10998, Sma-1-10999, Sma-1-110, Sma-1-1100, Sma-1-11000, Sma-1-11001, Sma-1-11003, Sma-1-11004, Sma-1-11005, Sma-1-11006, Sma-1-11007, Sma-1-11008, Sma-1-11009, Sma-1-11010, Sma-1-11011, Sma-1-11012, Sma-1-11013, Sma-1-11014, Sma-1-11015, Sma-1-11016, Sma-1-11017, Sma-1-11018, Sma-1-11019, Sma-1-1102, Sma-1-11020, Sma-1-11021, Sma-1-11022, Sma-1-11023, Sma-1-11024, Sma-1-11025, Sma-1-11026, Sma-1-11027, Sma-1-11028, Sma-1-11029, Sma-1-11031, Sma-1-11033, Sma-1-11034, Sma-1-11035, Sma-1-11036, Sma-1-11037, Sma-1-11038, Sma-1-11039, Sma-1-1104, Sma-1-11041, Sma-1-11042, Sma-1-11043, Sma-1-11044, Sma-1-11045, Sma-1-11046, Sma-1-11047, Sma-1-11048, Sma-1-11049, Sma-1-11051, Sma-1-11053, Sma-1-11054, Sma-1-11055, Sma-1-11056, Sma-1-11057, Sma-1-11058, Sma-1-11059, Sma-1-1106, Sma-1-11060, Sma-1-11061, Sma-1-11062, Sma-1-11063, Sma-1-11064, Sma-1-11065, Sma-1-11066, Sma-1-11067, Sma-1-11068, Sma-1-11069, Sma-1-1107, Sma-1-11070, Sma-1-11071, Sma-1-11072, Sma-1-11073, Sma-1-11074, Sma-1-11075, Sma-1-11076, Sma-1-11077, Sma-1-11078, Sma-1-11079, Sma-1-1108, Sma-1-11080, Sma-1-11081, Sma-1-11082, Sma-1-11083, Sma-1-11084, Sma-1-11085, Sma-1-11086, Sma-1-11087, Sma-1-11088, Sma-1-11089, Sma-1-11090, Sma-1-11091, Sma-1-11092, Sma-1-11093, Sma-1-11094, Sma-1-11095, Sma-1-11096, Sma-1-11097, Sma-1-11098, Sma-1-11099, Sma-1-1110, Sma-1-11100, Sma-1-11101, Sma-1-11102, Sma-1-11103, Sma-1-11104, Sma-1-11105, Sma-1-11106, Sma-1-11107, Sma-1-11108, Sma-1-11109, Sma-1-11110, Sma-1-11111, Sma-1-11112, Sma-1-11113, Sma-1-11114, Sma-1-11115, Sma-1-11116, Sma-1-11117, Sma-1-11118, Sma-1-11119, Sma-1-1112, Sma-1-11120, Sma-1-11121, Sma-1-11122, Sma-1-11123, Sma-1-11124, Sma-1-11125, Sma-1-11126, Sma-1-11127, Sma-1-11128, Sma-1-11129, Sma-1-1113, Sma-1-11130, Sma-1-11131, Sma-1-11132, Sma-1-11133, Sma-1-11134, Sma-1-11135, Sma-1-11136, Sma-1-11137, Sma-1-11138, Sma-1-11139, Sma-1-1114, Sma-1-11140, Sma-1-11141, Sma-1-11142, Sma-1-11143, Sma-1-11144, Sma-1-11145, Sma-1-11146, Sma-1-11147, Sma-1-11148, Sma-1-11149, Sma-1-11150, Sma-1-11151, Sma-1-11152, Sma-1-11153, Sma-1-11154, Sma-1-11155, Sma-1-11156, Sma-1-11157, Sma-1-11158, Sma-1-11159, Sma-1-1116, Sma-1-11160, Sma-1-11161, Sma-1-11162, Sma-1-11163, Sma-1-11164, Sma-1-11165, Sma-1-11166, Sma-1-11167, Sma-1-11168, Sma-1-11169, Sma-1-11170, Sma-1-11171, Sma-1-11172, Sma-1-11173, Sma-1-11174, Sma-1-11175, Sma-1-11176, Sma-1-11177, Sma-1-11178, Sma-1-11179, Sma-1-1118, Sma-1-11180, Sma-1-11181, Sma-1-11182, Sma-1-11183, Sma-1-11184, Sma-1-11185, Sma-1-11186, Sma-1-11187, Sma-1-11188, Sma-1-11189, Sma-1-11190, Sma-1-11191, Sma-1-11192, Sma-1-11193, Sma-1-11194, Sma-1-11195, Sma-1-11196, Sma-1-11197, Sma-1-11198, Sma-1-11199, Sma-1-112, Sma-1-1120, Sma-1-11200, Sma-1-11201, Sma-1-11202, Sma-1-11203, Sma-1-11204, Sma-1-11205, Sma-1-11206, Sma-1-11207, Sma-1-11208, Sma-1-11209, Sma-1-11210, Sma-1-11211, Sma-1-11212, Sma-1-11213, Sma-1-11214, Sma-1-11215, Sma-1-11216, Sma-1-11217, Sma-1-11218, Sma-1-11219, Sma-1-1122, Sma-1-11220, Sma-1-11221, Sma-1-11222, Sma-1-11223, Sma-1-11224, Sma-1-11225, Sma-1-11226, Sma-1-11227, Sma-1-11228, Sma-1-11229, Sma-1-11230, Sma-1-11231, Sma-1-11232, Sma-1-11233, Sma-1-11234, Sma-1-11235, Sma-1-11236, Sma-1-11237, Sma-1-11238, Sma-1-11239, Sma-1-1124, Sma-1-11240, Sma-1-11241, Sma-1-11242, Sma-1-11243, Sma-1-11244, Sma-1-11245, Sma-1-11246, Sma-1-11247, Sma-1-11248, Sma-1-11249, Sma-1-11250, Sma-1-11251, Sma-1-11252, Sma-1-11253, Sma-1-11254, Sma-1-11255, Sma-1-11256, Sma-1-11257, Sma-1-11258, Sma-1-11259, Sma-1-1126, Sma-1-11260, Sma-1-11261, Sma-1-11262, Sma-1-11263, Sma-1-11264, Sma-1-11265, Sma-1-11266, Sma-1-11267, Sma-1-11268, Sma-1-11269, Sma-1-11270, Sma-1-11271, Sma-1-11272, Sma-1-11273, Sma-1-11274, Sma-1-11275, Sma-1-11276, Sma-1-11277, Sma-1-11278, Sma-1-11279, Sma-1-1128, Sma-1-11280, Sma-1-11281, Sma-1-11282, Sma-1-11283, Sma-1-11284, Sma-1-11285, Sma-1-11286, Sma-1-11287, Sma-1-11288, Sma-1-11289, Sma-1-11290, Sma-1-11291, Sma-1-11292, Sma-1-11293, Sma-1-11294, Sma-1-11295, Sma-1-11296, Sma-1-11297, Sma-1-11298, Sma-1-11299, Sma-1-1130, Sma-1-11300, Sma-1-11301, Sma-1-11302, Sma-1-11303, Sma-1-11304, Sma-1-11305, Sma-1-11306, Sma-1-11307, Sma-1-11308, Sma-1-11309, Sma-1-11310, Sma-1-11311, Sma-1-11312, Sma-1-11313, Sma-1-11314, Sma-1-11315, Sma-1-11316, Sma-1-11317, Sma-1-11318, Sma-1-11319, Sma-1-1132, Sma-1-11320, Sma-1-11321, Sma-1-11322, Sma-1-11323, Sma-1-11324, Sma-1-11325, Sma-1-11326, Sma-1-11327, Sma-1-11328, Sma-1-11329, Sma-1-11330, Sma-1-11331, Sma-1-11332, Sma-1-11333, Sma-1-11334, Sma-1-11335, Sma-1-11336, Sma-1-11337, Sma-1-11338, Sma-1-11339, Sma-1-1134, Sma-1-11340, Sma-1-11341, Sma-1-11342, Sma-1-11343, Sma-1-11344, Sma-1-11345, Sma-1-11346, Sma-1-11347, Sma-1-11348, Sma-1-11349, Sma-1-11350, Sma-1-11351, Sma-1-11352, Sma-1-11353, Sma-1-11354, Sma-1-11355, Sma-1-11356, Sma-1-11357, Sma-1-11358, Sma-1-11359, Sma-1-1136, Sma-1-11360, Sma-1-11361, Sma-1-11362, Sma-1-11363, Sma-1-11364, Sma-1-11365, Sma-1-11366, Sma-1-11367, Sma-1-11368, Sma-1-11369, Sma-1-11370, Sma-1-11371, Sma-1-11372, Sma-1-11373, Sma-1-11374, Sma-1-11375, Sma-1-11376, Sma-1-11377, Sma-1-11378, Sma-1-11379, Sma-1-1138, Sma-1-11380, Sma-1-11381, Sma-1-11382, Sma-1-11383, Sma-1-11384, Sma-1-11385, Sma-1-11386, Sma-1-11387, Sma-1-11388, Sma-1-11389, Sma-1-11390, Sma-1-11391, Sma-1-11392, Sma-1-11393, Sma-1-11394, Sma-1-11395, Sma-1-11396, Sma-1-11397, Sma-1-11398, Sma-1-11399, Sma-1-114, Sma-1-1140, Sma-1-11400, Sma-1-11401, Sma-1-11402, Sma-1-11403, Sma-1-11404, Sma-1-11405, Sma-1-11406, Sma-1-11407, Sma-1-11408, Sma-1-11409, Sma-1-1141, Sma-1-11410, Sma-1-11411, Sma-1-11412, Sma-1-11413, Sma-1-11414, Sma-1-11415, Sma-1-11416, Sma-1-11417, Sma-1-11418, Sma-1-11419, Sma-1-1142, Sma-1-11420, Sma-1-11421, Sma-1-11422, Sma-1-11423, Sma-1-11424, Sma-1-11425, Sma-1-11426, Sma-1-11427, Sma-1-11428, Sma-1-11429, Sma-1-11430, Sma-1-11431, Sma-1-11432, Sma-1-11433, Sma-1-11434, Sma-1-11435, Sma-1-11436, Sma-1-11437, Sma-1-11438, Sma-1-11439, Sma-1-1144, Sma-1-11440, Sma-1-11441, Sma-1-11442, Sma-1-11443, Sma-1-11444, Sma-1-11445, Sma-1-11446, Sma-1-11447, Sma-1-11448, Sma-1-11449, Sma-1-1145, Sma-1-11450, Sma-1-11451, Sma-1-11452, Sma-1-11453,

Sma-1-11454, Sma-1-11455, Sma-1-11456, Sma-1-11457, Sma-1-11458, Sma-1-11459, Sma-1-1146, Sma-1-11460, Sma-1-11461, Sma-1-11462, Sma-1-11463, Sma-1-11464, Sma-1-11465, Sma-1-11466, Sma-1-11467, Sma-1-11468, Sma-1-11469, Sma-1-11470, Sma-1-11471, Sma-1-11472, Sma-1-11473, Sma-1-11474, Sma-1-11475, Sma-1-11476, Sma-1-11477, Sma-1-11478, Sma-1-11479, Sma-1-1148, Sma-1-11480, Sma-1-11481, Sma-1-11482, Sma-1-11483, Sma-1-11484, Sma-1-11485, Sma-1-11486, Sma-1-11487, Sma-1-11488, Sma-1-11489, Sma-1-11490, Sma-1-11491, Sma-1-11492, Sma-1-11493, Sma-1-11494, Sma-1-11495, Sma-1-11496, Sma-1-11497, Sma-1-11498, Sma-1-11499, Sma-1-1150, Sma-1-11500, Sma-1-11501, Sma-1-11502, Sma-1-11503, Sma-1-11504, Sma-1-11505, Sma-1-11506, Sma-1-11507, Sma-1-11508, Sma-1-11509, Sma-1-11510, Sma-1-11511, Sma-1-11512, Sma-1-11513, Sma-1-11514, Sma-1-11515, Sma-1-11516, Sma-1-11517, Sma-1-11518, Sma-1-11519, Sma-1-1152, Sma-1-11520, Sma-1-11521, Sma-1-11522, Sma-1-11523, Sma-1-11524, Sma-1-11525, Sma-1-11527, Sma-1-11529, Sma-1-11531, Sma-1-11533, Sma-1-11535, Sma-1-11537, Sma-1-11539, Sma-1-1154, Sma-1-11541, Sma-1-11543, Sma-1-11545, Sma-1-11547, Sma-1-11549, Sma-1-11551, Sma-1-11553, Sma-1-11555, Sma-1-11557, Sma-1-11559, Sma-1-1156, Sma-1-11560, Sma-1-11561, Sma-1-11562, Sma-1-11563, Sma-1-11564, Sma-1-11565, Sma-1-11567, Sma-1-11569, Sma-1-11571, Sma-1-11573, Sma-1-11575, Sma-1-11576, Sma-1-11577, Sma-1-11579, Sma-1-1158, Sma-1-11581, Sma-1-11582, Sma-1-11583, Sma-1-11584, Sma-1-11585, Sma-1-11587, Sma-1-11589, Sma-1-11591, Sma-1-11592, Sma-1-11593, Sma-1-11594, Sma-1-11595, Sma-1-11597, Sma-1-11598, Sma-1-11599, Sma-1-116, Sma-1-1160, Sma-1-11601, Sma-1-11603, Sma-1-11604, Sma-1-11605, Sma-1-11606, Sma-1-11607, Sma-1-11608, Sma-1-11609, Sma-1-11610, Sma-1-11611, Sma-1-11613, Sma-1-11614, Sma-1-11615, Sma-1-11616, Sma-1-11617, Sma-1-11618, Sma-1-11619, Sma-1-1162, Sma-1-11620, Sma-1-11621, Sma-1-11622, Sma-1-11623, Sma-1-11624, Sma-1-11625, Sma-1-11626, Sma-1-11627, Sma-1-11628, Sma-1-11629, Sma-1-11631, Sma-1-11633, Sma-1-11635, Sma-1-11637, Sma-1-11639, Sma-1-1164, Sma-1-11640, Sma-1-11641, Sma-1-11642, Sma-1-11643, Sma-1-11645, Sma-1-11647, Sma-1-11649, Sma-1-11651, Sma-1-11653, Sma-1-11655, Sma-1-11657, Sma-1-11659, Sma-1-1166, Sma-1-11661, Sma-1-11662, Sma-1-11663, Sma-1-11665, Sma-1-11667, Sma-1-11668, Sma-1-11669, Sma-1-11671, Sma-1-11673, Sma-1-11675, Sma-1-11677, Sma-1-11679, Sma-1-1168, Sma-1-11681, Sma-1-11682, Sma-1-11683, Sma-1-11685, Sma-1-11687, Sma-1-11689, Sma-1-11691, Sma-1-11692, Sma-1-11693, Sma-1-11694, Sma-1-11695, Sma-1-11696, Sma-1-11697, Sma-1-11698, Sma-1-11699, Sma-1-1170, Sma-1-11700, Sma-1-11701, Sma-1-11702, Sma-1-11703, Sma-1-11704, Sma-1-11705, Sma-1-11707, Sma-1-11709, Sma-1-11711, Sma-1-11713, Sma-1-11714, Sma-1-11715, Sma-1-11716, Sma-1-11717, Sma-1-11718, Sma-1-11719, Sma-1-1172, Sma-1-11720, Sma-1-11721, Sma-1-11722, Sma-1-11723, Sma-1-11724, Sma-1-11725, Sma-1-11726, Sma-1-11727, Sma-1-11729, Sma-1-11730, Sma-1-11731, Sma-1-11733, Sma-1-11735, Sma-1-11736, Sma-1-11737, Sma-1-11739, Sma-1-1174, Sma-1-11741, Sma-1-11743, Sma-1-11745, Sma-1-11747, Sma-1-11748, Sma-1-11749, Sma-1-11750, Sma-1-11751, Sma-1-11753, Sma-1-11755, Sma-1-11757, Sma-1-11759, Sma-1-1176, Sma-1-11761, Sma-1-11762, Sma-1-11763, Sma-1-11765, Sma-1-11766, Sma-1-11767, Sma-1-11769, Sma-1-11770, Sma-1-11771, Sma-1-11773, Sma-1-11775, Sma-1-11777, Sma-1-11779, Sma-1-1178, Sma-1-11780, Sma-1-11781, Sma-1-11783, Sma-1-11785, Sma-1-11787, Sma-1-11789, Sma-1-11791, Sma-1-11793, Sma-1-11795, Sma-1-11797, Sma-1-11799, Sma-1-118, Sma-1-1180, Sma-1-11801, Sma-1-11803, Sma-1-11805, Sma-1-11807, Sma-1-11809, Sma-1-11811, Sma-1-11813, Sma-1-11814, Sma-1-11815, Sma-1-11817, Sma-1-11819, Sma-1-1182, Sma-1-11821, Sma-1-11823, Sma-1-11825, Sma-1-11827, Sma-1-11829, Sma-1-11831, Sma-1-11833, Sma-1-11835, Sma-1-11837, Sma-1-11839, Sma-1-1184, Sma-1-11841, Sma-1-11843, Sma-1-11845, Sma-1-11847, Sma-1-11848, Sma-1-11849, Sma-1-11851, Sma-1-11853, Sma-1-11855, Sma-1-11857, Sma-1-11858, Sma-1-11859, Sma-1-1186, Sma-1-11861, Sma-1-11862, Sma-1-11863, Sma-1-11865, Sma-1-11867, Sma-1-11869, Sma-1-11871, Sma-1-11873, Sma-1-11874, Sma-1-11875, Sma-1-11876, Sma-1-11877, Sma-1-11879, Sma-1-1188, Sma-1-11881, Sma-1-11882, Sma-1-11883, Sma-1-11884, Sma-1-11885, Sma-1-11886, Sma-1-11887, Sma-1-11888, Sma-1-11889, Sma-1-11890, Sma-1-11891, Sma-1-11893, Sma-1-11895, Sma-1-11897, Sma-1-11899, Sma-1-1190, Sma-1-11901, Sma-1-11903, Sma-1-11905, Sma-1-11906, Sma-1-11907, Sma-1-11908, Sma-1-11909, Sma-1-11910, Sma-1-11911, Sma-1-11913, Sma-1-11914, Sma-1-11915, Sma-1-11917, Sma-1-11919, Sma-1-1192, Sma-1-11921, Sma-1-11923, Sma-1-11925, Sma-1-11927, Sma-1-11929, Sma-1-11930, Sma-1-11931, Sma-1-11933, Sma-1-11935, Sma-1-11936, Sma-1-11937, Sma-1-11939, Sma-1-1194, Sma-1-11941, Sma-1-11943, Sma-1-11945, Sma-1-11947, Sma-1-11949, Sma-1-11951, Sma-1-11952, Sma-1-11953, Sma-1-11954, Sma-1-11955, Sma-1-11956, Sma-1-11957, Sma-1-11958, Sma-1-11959, Sma-1-1196, Sma-1-11960, Sma-1-11961, Sma-1-11962, Sma-1-11963, Sma-1-11964, Sma-1-11965, Sma-1-11966, Sma-1-11967, Sma-1-11969, Sma-1-11970, Sma-1-11971, Sma-1-11972, Sma-1-11973, Sma-1-11975, Sma-1-11977, Sma-1-11979, Sma-1-1198, Sma-1-11981, Sma-1-11982, Sma-1-11983, Sma-1-11985, Sma-1-11986, Sma-1-11987, Sma-1-11988, Sma-1-11989, Sma-1-11990, Sma-1-11991, Sma-1-11992, Sma-1-11993, Sma-1-11994, Sma-1-11995, Sma-1-11996, Sma-1-11997, Sma-1-11999, Sma-1-12, Sma-1-120, Sma-1-1200, Sma-1-12001, Sma-1-12003, Sma-1-12005, Sma-1-12007, Sma-1-12008, Sma-1-12009, Sma-1-12010, Sma-1-12011, Sma-1-12013, Sma-1-12015, Sma-1-12016, Sma-1-12017, Sma-1-12018, Sma-1-12019, Sma-1-1202, Sma-1-12020, Sma-1-12021, Sma-1-12022, Sma-1-12023, Sma-1-12024, Sma-1-12025, Sma-1-12026, Sma-1-12027, Sma-1-12028, Sma-1-12029, Sma-1-12030, Sma-1-12031, Sma-1-12032, Sma-1-12033, Sma-1-12034, Sma-1-12035, Sma-1-12036, Sma-1-12037, Sma-1-12038, Sma-1-12039, Sma-1-1204, Sma-1-12040, Sma-1-12041, Sma-1-12042, Sma-1-12043, Sma-1-12044, Sma-1-12045, Sma-1-12046, Sma-1-12047, Sma-1-12048, Sma-1-12049, Sma-1-12050, Sma-1-12051, Sma-1-12052, Sma-1-12053, Sma-1-12054, Sma-1-12055, Sma-1-12056, Sma-1-12057, Sma-1-12058, Sma-1-12059, Sma-1-1206, Sma-1-12060, Sma-1-12061, Sma-1-12062, Sma-1-12063, Sma-1-12065, Sma-1-12067, Sma-1-12069, Sma-1-12071, Sma-1-12073, Sma-1-12074, Sma-1-12075, Sma-1-12076, Sma-1-12077, Sma-1-12079, Sma-1-1208, Sma-1-12080, Sma-1-12081, Sma-1-12083, Sma-1-12085, Sma-1-12086, Sma-1-12087, Sma-1-12089, Sma-1-12091, Sma-1-12093, Sma-1-12095, Sma-1-12096, Sma-1-12097, Sma-1-12099, Sma-1-1210, Sma-1-12101, Sma-1-12102, Sma-1-12103, Sma-1-12105, Sma-1-12107, Sma-1-12109, Sma-1-12110, Sma-1-12111, Sma-1-12112, Sma-1-12113, Sma-1-12115, Sma-1-12117, Sma-1-12119, Sma-1-1212, Sma-1-12120, Sma-1-12121, Sma-1-12122, Sma-1-12123, Sma-1-12124, Sma-1-12125, Sma-1-12127, Sma-1-12129,

Sma-1-12131, Sma-1-12133, Sma-1-12135, Sma-1-12137, Sma-1-12138, Sma-1-12139, Sma-1-1214, Sma-1-12141, Sma-1-12143, Sma-1-12144, Sma-1-12145, Sma-1-12146, Sma-1-12147, Sma-1-12149, Sma-1-12150, Sma-1-12151, Sma-1-12152, Sma-1-12153, Sma-1-12155, Sma-1-12157, Sma-1-12159, Sma-1-1216, Sma-1-12161, Sma-1-12163, Sma-1-12165, Sma-1-12167, Sma-1-12169, Sma-1-1217, Sma-1-12171, Sma-1-12172, Sma-1-12173, Sma-1-12175, Sma-1-12177, Sma-1-12179, Sma-1-1218, Sma-1-12180, Sma-1-12181, Sma-1-12182, Sma-1-12183, Sma-1-12184, Sma-1-12185, Sma-1-12186, Sma-1-12187, Sma-1-12188, Sma-1-12189, Sma-1-12190, Sma-1-12191, Sma-1-12192, Sma-1-12193, Sma-1-12194, Sma-1-12195, Sma-1-12196, Sma-1-12197, Sma-1-12198, Sma-1-12199, Sma-1-122, Sma-1-1220, Sma-1-12200, Sma-1-12201, Sma-1-12202, Sma-1-12203, Sma-1-12205, Sma-1-12207, Sma-1-12209, Sma-1-12210, Sma-1-12211, Sma-1-12212, Sma-1-12213, Sma-1-12214, Sma-1-12215, Sma-1-12216, Sma-1-12217, Sma-1-12218, Sma-1-12219, Sma-1-1222, Sma-1-12220, Sma-1-12221, Sma-1-12222, Sma-1-12223, Sma-1-12224, Sma-1-12225, Sma-1-12226, Sma-1-12227, Sma-1-12229, Sma-1-12231, Sma-1-12233, Sma-1-12235, Sma-1-12237, Sma-1-12239, Sma-1-1224, Sma-1-12241, Sma-1-12243, Sma-1-12245, Sma-1-12247, Sma-1-12248, Sma-1-12249, Sma-1-12250, Sma-1-12251, Sma-1-12252, Sma-1-12253, Sma-1-12254, Sma-1-12255, Sma-1-12256, Sma-1-12257, Sma-1-12258, Sma-1-12259, Sma-1-1226, Sma-1-12260, Sma-1-12261, Sma-1-12262, Sma-1-12263, Sma-1-12264, Sma-1-12265, Sma-1-12267, Sma-1-12269, Sma-1-1227, Sma-1-12271, Sma-1-12272, Sma-1-12273, Sma-1-12275, Sma-1-12277, Sma-1-12279, Sma-1-1228, Sma-1-12281, Sma-1-12283, Sma-1-12285, Sma-1-12287, Sma-1-12288, Sma-1-12289, Sma-1-12291, Sma-1-12293, Sma-1-12295, Sma-1-12297, Sma-1-12299, Sma-1-1230, Sma-1-12301, Sma-1-12302, Sma-1-12303, Sma-1-12305, Sma-1-12307, Sma-1-12308, Sma-1-12309, Sma-1-12311, Sma-1-12313, Sma-1-12315, Sma-1-12317, Sma-1-12319, Sma-1-1232, Sma-1-12321, Sma-1-12323, Sma-1-12325, Sma-1-12327, Sma-1-12329, Sma-1-12331, Sma-1-12333, Sma-1-12335, Sma-1-12337, Sma-1-12339, Sma-1-1234, Sma-1-12340, Sma-1-12341, Sma-1-12342, Sma-1-12343, Sma-1-12344, Sma-1-12345, Sma-1-12346, Sma-1-12347, Sma-1-12348, Sma-1-12349, Sma-1-12350, Sma-1-12351, Sma-1-12352, Sma-1-12353, Sma-1-12354, Sma-1-12355, Sma-1-12356, Sma-1-12357, Sma-1-12358, Sma-1-12359, Sma-1-1236, Sma-1-12360, Sma-1-12361, Sma-1-12362, Sma-1-12363, Sma-1-12364, Sma-1-12365, Sma-1-12367, Sma-1-12369, Sma-1-12371, Sma-1-12373, Sma-1-12374, Sma-1-12375, Sma-1-12376, Sma-1-12377, Sma-1-12379, Sma-1-1238, Sma-1-12381, Sma-1-12383, Sma-1-12384, Sma-1-12385, Sma-1-12387, Sma-1-12389, Sma-1-12391, Sma-1-12393, Sma-1-12395, Sma-1-12397, Sma-1-12399, Sma-1-124, Sma-1-1240, Sma-1-12401, Sma-1-12403, Sma-1-12405, Sma-1-12407, Sma-1-12409, Sma-1-12411, Sma-1-12413, Sma-1-12414, Sma-1-12415, Sma-1-12416, Sma-1-12417, Sma-1-12419, Sma-1-1242, Sma-1-12421, Sma-1-12423, Sma-1-12425, Sma-1-12427, Sma-1-12428, Sma-1-12429, Sma-1-12431, Sma-1-12433, Sma-1-12435, Sma-1-12437, Sma-1-12439, Sma-1-1244, Sma-1-12441, Sma-1-12443, Sma-1-12445, Sma-1-12447, Sma-1-12449, Sma-1-12451, Sma-1-12453, Sma-1-12455, Sma-1-12457, Sma-1-12458, Sma-1-12459, Sma-1-1246, Sma-1-12461, Sma-1-12463, Sma-1-12465, Sma-1-12466, Sma-1-12467, Sma-1-12468, Sma-1-12469, Sma-1-12470, Sma-1-12471, Sma-1-12472, Sma-1-12473, Sma-1-12474, Sma-1-12475, Sma-1-12476, Sma-1-12477, Sma-1-12478, Sma-1-12479, Sma-1-1248, Sma-1-12480, Sma-1-12481, Sma-1-12482, Sma-1-12483, Sma-1-12484, Sma-1-12485, Sma-1-12486, Sma-1-12487, Sma-1-12488, Sma-1-12489, Sma-1-12490, Sma-1-12491, Sma-1-12492, Sma-1-12493, Sma-1-12494, Sma-1-12495, Sma-1-12496, Sma-1-12497, Sma-1-12498, Sma-1-12499, Sma-1-1250, Sma-1-12500, Sma-1-12501, Sma-1-12502, Sma-1-12503, Sma-1-12504, Sma-1-12505, Sma-1-12506, Sma-1-12507, Sma-1-12508, Sma-1-12509, Sma-1-12510, Sma-1-12511, Sma-1-12512, Sma-1-12513, Sma-1-12514, Sma-1-12515, Sma-1-12516, Sma-1-12517, Sma-1-12518, Sma-1-12519, Sma-1-1252, Sma-1-12520, Sma-1-12521, Sma-1-12522, Sma-1-12523, Sma-1-12525, Sma-1-12527, Sma-1-12528, Sma-1-12529, Sma-1-12530, Sma-1-12531, Sma-1-12532, Sma-1-12533, Sma-1-12535, Sma-1-12537, Sma-1-12539, Sma-1-1254, Sma-1-12540, Sma-1-12541, Sma-1-12542, Sma-1-12543, Sma-1-12545, Sma-1-12547, Sma-1-12548, Sma-1-12549, Sma-1-12550, Sma-1-12551, Sma-1-12552, Sma-1-12553, Sma-1-12554, Sma-1-12555, Sma-1-12556, Sma-1-12557, Sma-1-12559, Sma-1-1256, Sma-1-12561, Sma-1-12563, Sma-1-12564, Sma-1-12565, Sma-1-12566, Sma-1-12567, Sma-1-12568, Sma-1-12569, Sma-1-12570, Sma-1-12571, Sma-1-12572, Sma-1-12573, Sma-1-12575, Sma-1-12576, Sma-1-12577, Sma-1-12579, Sma-1-1258, Sma-1-12581, Sma-1-12582, Sma-1-12583, Sma-1-12585, Sma-1-12586, Sma-1-12587, Sma-1-12588, Sma-1-12589, Sma-1-12591, Sma-1-12593, Sma-1-12595, Sma-1-12597, Sma-1-12599, Sma-1-126, Sma-1-1260, Sma-1-12600, Sma-1-12601, Sma-1-12602, Sma-1-12603, Sma-1-12605, Sma-1-12607, Sma-1-12609, Sma-1-12611, Sma-1-12613, Sma-1-12615, Sma-1-12616, Sma-1-12617, Sma-1-12619, Sma-1-1262, Sma-1-12621, Sma-1-12623, Sma-1-12625, Sma-1-12626, Sma-1-12627, Sma-1-12628, Sma-1-12629, Sma-1-12631, Sma-1-12633, Sma-1-12634, Sma-1-12635, Sma-1-12637, Sma-1-12639, Sma-1-1264, Sma-1-12640, Sma-1-12641, Sma-1-12643, Sma-1-12645, Sma-1-12647, Sma-1-12648, Sma-1-12649, Sma-1-12650, Sma-1-12651, Sma-1-12652, Sma-1-12653, Sma-1-12655, Sma-1-12657, Sma-1-12659, Sma-1-1266, Sma-1-12661, Sma-1-12662, Sma-1-12663, Sma-1-12664, Sma-1-12665, Sma-1-12666, Sma-1-12667, Sma-1-12668, Sma-1-12669, Sma-1-12670, Sma-1-12671, Sma-1-12672, Sma-1-12673, Sma-1-12674, Sma-1-12675, Sma-1-12676, Sma-1-12677, Sma-1-12678, Sma-1-12679, Sma-1-1268, Sma-1-12680, Sma-1-12681, Sma-1-12682, Sma-1-12683, Sma-1-12684, Sma-1-12685, Sma-1-12686, Sma-1-12687, Sma-1-12688, Sma-1-12689, Sma-1-1269, Sma-1-12690, Sma-1-12691, Sma-1-12692, Sma-1-12693, Sma-1-12694, Sma-1-12695, Sma-1-12696, Sma-1-12697, Sma-1-12698, Sma-1-12699, Sma-1-1270, Sma-1-12700, Sma-1-12701, Sma-1-12702, Sma-1-12703, Sma-1-12704, Sma-1-12705, Sma-1-12706, Sma-1-12707, Sma-1-12708, Sma-1-12709, Sma-1-12710, Sma-1-12711, Sma-1-12712, Sma-1-12713, Sma-1-12714, Sma-1-12715, Sma-1-12716, Sma-1-12717, Sma-1-12718, Sma-1-12719, Sma-1-1272, Sma-1-12720, Sma-1-12721, Sma-1-12722, Sma-1-12723, Sma-1-12724, Sma-1-12725, Sma-1-12726, Sma-1-12727, Sma-1-12728, Sma-1-12729, Sma-1-12731, Sma-1-12733, Sma-1-12734, Sma-1-12735, Sma-1-12736, Sma-1-12737, Sma-1-12739, Sma-1-1274, Sma-1-12741, Sma-1-12742, Sma-1-12743, Sma-1-12745, Sma-1-12747, Sma-1-12749, Sma-1-12751, Sma-1-12753, Sma-1-12755, Sma-1-12756, Sma-1-12757, Sma-1-12758, Sma-1-12759, Sma-1-1276, Sma-1-12761, Sma-1-12762, Sma-1-12763, Sma-1-12764, Sma-1-12765, Sma-1-12766, Sma-1-12767, Sma-1-12768, Sma-1-12769, Sma-1-12770, Sma-1-12771, Sma-1-12772, Sma-1-12773, Sma-1-12774, Sma-1-12775, Sma-1-12776, Sma-1-12777, Sma-1-12778, Sma-1-12779, Sma-1-1278, Sma-1-12780, Sma-

1-12781, Sma-1-12782, Sma-1-12783, Sma-1-12784, Sma-1-12785, Sma-1-12786, Sma-1-12787, Sma-1-12788, Sma-1-12789, Sma-1-12790, Sma-1-12791, Sma-1-12792, Sma-1-12793, Sma-1-12794, Sma-1-12795, Sma-1-12796, Sma-1-12797, Sma-1-12798, Sma-1-12799, Sma-1-128, Sma-1-1280, Sma-1-12801, Sma-1-12802, Sma-1-12803, Sma-1-12804, Sma-1-12805, Sma-1-12807, Sma-1-12808, Sma-1-12809, Sma-1-12810, Sma-1-12811, Sma-1-12813, Sma-1-12815, Sma-1-12817, Sma-1-12819, Sma-1-1282, Sma-1-12821, Sma-1-12823, Sma-1-12825, Sma-1-12827, Sma-1-12829, Sma-1-12831, Sma-1-12833, Sma-1-12835, Sma-1-12836, Sma-1-12837, Sma-1-12838, Sma-1-12839, Sma-1-1284, Sma-1-12841, Sma-1-12843, Sma-1-12845, Sma-1-12847, Sma-1-12849, Sma-1-12850, Sma-1-12851, Sma-1-12853, Sma-1-12855, Sma-1-12857, Sma-1-12858, Sma-1-12859, Sma-1-1286, Sma-1-12861, Sma-1-12863, Sma-1-12865, Sma-1-12866, Sma-1-12867, Sma-1-12869, Sma-1-12871, Sma-1-12873, Sma-1-12875, Sma-1-12876, Sma-1-12877, Sma-1-12879, Sma-1-1288, Sma-1-12881, Sma-1-12882, Sma-1-12883, Sma-1-12884, Sma-1-12885, Sma-1-12886, Sma-1-12887, Sma-1-12888, Sma-1-12889, Sma-1-12890, Sma-1-12891, Sma-1-12893, Sma-1-12895, Sma-1-12897, Sma-1-12899, Sma-1-1290, Sma-1-12901, Sma-1-12903, Sma-1-12905, Sma-1-12907, Sma-1-12909, Sma-1-12910, Sma-1-12911, Sma-1-12912, Sma-1-12913, Sma-1-12914, Sma-1-12915, Sma-1-12917, Sma-1-12919, Sma-1-1292, Sma-1-12920, Sma-1-12921, Sma-1-12922, Sma-1-12923, Sma-1-12925, Sma-1-12926, Sma-1-12927, Sma-1-12929, Sma-1-1293, Sma-1-12930, Sma-1-12931, Sma-1-12932, Sma-1-12933, Sma-1-12935, Sma-1-12937, Sma-1-12939, Sma-1-1294, Sma-1-12941, Sma-1-12943, Sma-1-12944, Sma-1-12945, Sma-1-12946, Sma-1-12947, Sma-1-12949, Sma-1-12951, Sma-1-12953, Sma-1-12955, Sma-1-12956, Sma-1-12957, Sma-1-12958, Sma-1-12959, Sma-1-1296, Sma-1-12961, Sma-1-12963, Sma-1-12965, Sma-1-12967, Sma-1-12968, Sma-1-12969, Sma-1-12970, Sma-1-12971, Sma-1-12972, Sma-1-12973, Sma-1-12974, Sma-1-12975, Sma-1-12976, Sma-1-12977, Sma-1-12978, Sma-1-12979, Sma-1-1298, Sma-1-12980, Sma-1-12981, Sma-1-12983, Sma-1-12984, Sma-1-12985, Sma-1-12987, Sma-1-12988, Sma-1-12989, Sma-1-12990, Sma-1-12991, Sma-1-12992, Sma-1-12993, Sma-1-12995, Sma-1-12997, Sma-1-12999, Sma-1-130, Sma-1-1300, Sma-1-13001, Sma-1-13003, Sma-1-13005, Sma-1-13007, Sma-1-13008, Sma-1-13009, Sma-1-13010, Sma-1-13011, Sma-1-13012, Sma-1-13013, Sma-1-13014, Sma-1-13015, Sma-1-13016, Sma-1-13017, Sma-1-13018, Sma-1-13019, Sma-1-1302, Sma-1-13020, Sma-1-13021, Sma-1-13022, Sma-1-13023, Sma-1-13024, Sma-1-13025, Sma-1-13026, Sma-1-13027, Sma-1-13028, Sma-1-13029, Sma-1-1303, Sma-1-13030, Sma-1-13031, Sma-1-13032, Sma-1-13033, Sma-1-13034, Sma-1-13035, Sma-1-13036, Sma-1-13037, Sma-1-13038, Sma-1-13039, Sma-1-1304, Sma-1-13040, Sma-1-13041, Sma-1-13042, Sma-1-13043, Sma-1-13045, Sma-1-13047, Sma-1-13048, Sma-1-13049, Sma-1-13050, Sma-1-13051, Sma-1-13052, Sma-1-13053, Sma-1-13054, Sma-1-13055, Sma-1-13057, Sma-1-13058, Sma-1-13059, Sma-1-1306, Sma-1-13061, Sma-1-13063, Sma-1-13065, Sma-1-13067, Sma-1-13069, Sma-1-13071, Sma-1-13072, Sma-1-13073, Sma-1-13075, Sma-1-13076, Sma-1-13077, Sma-1-13079, Sma-1-1308, Sma-1-13080, Sma-1-13081, Sma-1-13082, Sma-1-13083, Sma-1-13085, Sma-1-13087, Sma-1-13089, Sma-1-13091, Sma-1-13093, Sma-1-13095, Sma-1-13097, Sma-1-13098, Sma-1-13099, Sma-1-1310, Sma-1-13100, Sma-1-13101, Sma-1-13102, Sma-1-13103, Sma-1-13104, Sma-1-13105, Sma-1-13106, Sma-1-13107, Sma-1-13108, Sma-1-13109, Sma-1-13110, Sma-1-13111, Sma-1-13112, Sma-1-13113, Sma-1-13115, Sma-1-13117, Sma-1-13119, Sma-1-1312, Sma-1-13121, Sma-1-13123, Sma-1-13125, Sma-1-13126, Sma-1-13127, Sma-1-13128, Sma-1-13129, Sma-1-13131, Sma-1-13133, Sma-1-13135, Sma-1-13137, Sma-1-13139, Sma-1-1314, Sma-1-13141, Sma-1-13142, Sma-1-13143, Sma-1-13144, Sma-1-13145, Sma-1-13147, Sma-1-13148, Sma-1-13149, Sma-1-13150, Sma-1-13151, Sma-1-13153, Sma-1-13154, Sma-1-13155, Sma-1-13157, Sma-1-13159, Sma-1-1316, Sma-1-13161, Sma-1-13163, Sma-1-13165, Sma-1-13166, Sma-1-13167, Sma-1-13169, Sma-1-13171, Sma-1-13173, Sma-1-13174, Sma-1-13175, Sma-1-13177, Sma-1-13179, Sma-1-1318, Sma-1-13181, Sma-1-13182, Sma-1-13183, Sma-1-13185, Sma-1-13186, Sma-1-13187, Sma-1-13189, Sma-1-1319, Sma-1-13191, Sma-1-13193, Sma-1-13195, Sma-1-13197, Sma-1-13199, Sma-1-132, Sma-1-1320, Sma-1-13201, Sma-1-13202, Sma-1-13203, Sma-1-13205, Sma-1-13207, Sma-1-13209, Sma-1-13211, Sma-1-13212, Sma-1-13213, Sma-1-13214, Sma-1-13215, Sma-1-13216, Sma-1-13217, Sma-1-13218, Sma-1-13219, Sma-1-1322, Sma-1-13220, Sma-1-13221, Sma-1-13222, Sma-1-13223, Sma-1-13224, Sma-1-13225, Sma-1-13226, Sma-1-13227, Sma-1-13228, Sma-1-13229, Sma-1-13230, Sma-1-13231, Sma-1-13232, Sma-1-13233, Sma-1-13234, Sma-1-13235, Sma-1-13236, Sma-1-13237, Sma-1-13239, Sma-1-1324, Sma-1-13240, Sma-1-13241, Sma-1-13242, Sma-1-13243, Sma-1-13245, Sma-1-13246, Sma-1-13247, Sma-1-13248, Sma-1-13249, Sma-1-13250, Sma-1-13251, Sma-1-13252, Sma-1-13253, Sma-1-13254, Sma-1-13255, Sma-1-13256, Sma-1-13257, Sma-1-13259, Sma-1-1326, Sma-1-13261, Sma-1-13263, Sma-1-13265, Sma-1-13266, Sma-1-13267, Sma-1-13268, Sma-1-13269, Sma-1-1327, Sma-1-13271, Sma-1-13273, Sma-1-13275, Sma-1-13277, Sma-1-13279, Sma-1-1328, Sma-1-13281, Sma-1-13283, Sma-1-13285, Sma-1-13287, Sma-1-13288, Sma-1-13289, Sma-1-1329, Sma-1-13290, Sma-1-13291, Sma-1-13293, Sma-1-13295, Sma-1-13297, Sma-1-13298, Sma-1-13299, Sma-1-1330, Sma-1-13301, Sma-1-13303, Sma-1-13305, Sma-1-13307, Sma-1-13308, Sma-1-13309, Sma-1-13311, Sma-1-13313, Sma-1-13315, Sma-1-13317, Sma-1-13318, Sma-1-13319, Sma-1-1332, Sma-1-13321, Sma-1-13323, Sma-1-13325, Sma-1-13327, Sma-1-13329, Sma-1-13331, Sma-1-13333, Sma-1-13334, Sma-1-13335, Sma-1-13337, Sma-1-13338, Sma-1-13339, Sma-1-1334, Sma-1-13341, Sma-1-13343, Sma-1-13345, Sma-1-13347, Sma-1-13349, Sma-1-13351, Sma-1-13352, Sma-1-13353, Sma-1-13354, Sma-1-13355, Sma-1-13356, Sma-1-13357, Sma-1-13359, Sma-1-1336, Sma-1-13361, Sma-1-13363, Sma-1-13365, Sma-1-13367, Sma-1-13369, Sma-1-13371, Sma-1-13373, Sma-1-13375, Sma-1-13376, Sma-1-13377, Sma-1-13379, Sma-1-1338, Sma-1-13381, Sma-1-13382, Sma-1-13383, Sma-1-13385, Sma-1-13386, Sma-1-13387, Sma-1-13389, Sma-1-13390, Sma-1-13391, Sma-1-13393, Sma-1-13395, Sma-1-13397, Sma-1-13399, Sma-1-134, Sma-1-1340, Sma-1-13401, Sma-1-13402, Sma-1-13403, Sma-1-13404, Sma-1-13405, Sma-1-13406, Sma-1-13407, Sma-1-13409, Sma-1-13411, Sma-1-13413, Sma-1-13415, Sma-1-13417, Sma-1-13419, Sma-1-1342, Sma-1-13421, Sma-1-13423, Sma-1-13425, Sma-1-13427, Sma-1-13429, Sma-1-13431, Sma-1-13433, Sma-1-13434, Sma-1-13435, Sma-1-13437, Sma-1-13438, Sma-1-13439, Sma-1-1344, Sma-1-13440, Sma-1-13441, Sma-1-13442, Sma-1-13443, Sma-1-13445, Sma-1-13447, Sma-1-13449, Sma-1-13451, Sma-1-13452, Sma-1-13453, Sma-1-13455, Sma-1-13456, Sma-1-13457, Sma-1-13459, Sma-1-1346, Sma-1-13461, Sma-1-13463, Sma-1-13465, Sma-1-13467, Sma-1-13468, Sma-1-13469, Sma-1-13471, Sma-1-13473, Sma-1-13474, Sma-1-13475,

Sma-1-13476, Sma-1-13477, Sma-1-13479, Sma-1-1348, Sma-1-13481, Sma-1-13482, Sma-1-13483, Sma-1-13485, Sma-1-13487, Sma-1-13489, Sma-1-13491, Sma-1-13492, Sma-1-13493, Sma-1-13494, Sma-1-13495, Sma-1-13497, Sma-1-13499, Sma-1-1350, Sma-1-13500, Sma-1-13501, Sma-1-13503, Sma-1-13504, Sma-1-13505, Sma-1-13507, Sma-1-13508, Sma-1-13509, Sma-1-13510, Sma-1-13511, Sma-1-13512, Sma-1-13513, Sma-1-13514, Sma-1-13515, Sma-1-13516, Sma-1-13517, Sma-1-13518, Sma-1-13519, Sma-1-1352, Sma-1-13520, Sma-1-13521, Sma-1-13522, Sma-1-13523, Sma-1-13524, Sma-1-13525, Sma-1-13526, Sma-1-13527, Sma-1-13528, Sma-1-13529, Sma-1-13530, Sma-1-13531, Sma-1-13532, Sma-1-13533, Sma-1-13534, Sma-1-13535, Sma-1-13536, Sma-1-13537, Sma-1-13538, Sma-1-13539, Sma-1-1354, Sma-1-13541, Sma-1-13543, Sma-1-13545, Sma-1-13547, Sma-1-13549, Sma-1-13551, Sma-1-13553, Sma-1-13555, Sma-1-13557, Sma-1-13559, Sma-1-1356, Sma-1-13560, Sma-1-13561, Sma-1-13563, Sma-1-13565, Sma-1-13567, Sma-1-13569, Sma-1-1357, Sma-1-13570, Sma-1-13571, Sma-1-13573, Sma-1-13575, Sma-1-13577, Sma-1-13579, Sma-1-1358, Sma-1-13580, Sma-1-13581, Sma-1-13583, Sma-1-13585, Sma-1-13587, Sma-1-13589, Sma-1-1359, Sma-1-13591, Sma-1-13592, Sma-1-13593, Sma-1-13595, Sma-1-13597, Sma-1-13599, Sma-1-136, Sma-1-1360, Sma-1-13601, Sma-1-13603, Sma-1-13605, Sma-1-13607, Sma-1-13608, Sma-1-13609, Sma-1-1361, Sma-1-13611, Sma-1-13613, Sma-1-13615, Sma-1-13617, Sma-1-13619, Sma-1-1362, Sma-1-13621, Sma-1-13623, Sma-1-13625, Sma-1-13626, Sma-1-13627, Sma-1-13629, Sma-1-1363, Sma-1-13631, Sma-1-13633, Sma-1-13635, Sma-1-13637, Sma-1-13639, Sma-1-1364, Sma-1-13641, Sma-1-13643, Sma-1-13645, Sma-1-13647, Sma-1-13649, Sma-1-1365, Sma-1-13651, Sma-1-13653, Sma-1-13654, Sma-1-13655, Sma-1-13657, Sma-1-13659, Sma-1-1366, Sma-1-13661, Sma-1-13662, Sma-1-13663, Sma-1-13664, Sma-1-13665, Sma-1-13666, Sma-1-13667, Sma-1-13669, Sma-1-1367, Sma-1-13671, Sma-1-13673, Sma-1-13675, Sma-1-13676, Sma-1-13677, Sma-1-13679, Sma-1-1368, Sma-1-13681, Sma-1-13683, Sma-1-13685, Sma-1-13686, Sma-1-13687, Sma-1-13689, Sma-1-1369, Sma-1-13691, Sma-1-13693, Sma-1-13694, Sma-1-13695, Sma-1-13697, Sma-1-13699, Sma-1-1370, Sma-1-13701, Sma-1-13703, Sma-1-13705, Sma-1-13706, Sma-1-13707, Sma-1-13708, Sma-1-13709, Sma-1-1371, Sma-1-13710, Sma-1-13711, Sma-1-13712, Sma-1-13713, Sma-1-13714, Sma-1-13715, Sma-1-13716, Sma-1-13717, Sma-1-13718, Sma-1-13719, Sma-1-1372, Sma-1-13720, Sma-1-13721, Sma-1-13723, Sma-1-13725, Sma-1-13727, Sma-1-13729, Sma-1-1373, Sma-1-13730, Sma-1-13731, Sma-1-13732, Sma-1-13733, Sma-1-13735, Sma-1-13737, Sma-1-13738, Sma-1-13739, Sma-1-1374, Sma-1-13740, Sma-1-13741, Sma-1-13742, Sma-1-13743, Sma-1-13744, Sma-1-13745, Sma-1-13746, Sma-1-13747, Sma-1-13748, Sma-1-13749, Sma-1-1375, Sma-1-13750, Sma-1-13751, Sma-1-13752, Sma-1-13753, Sma-1-13754, Sma-1-13755, Sma-1-13756, Sma-1-13757, Sma-1-13758, Sma-1-13759, Sma-1-1376, Sma-1-13761, Sma-1-13763, Sma-1-13765, Sma-1-13767, Sma-1-13769, Sma-1-1377, Sma-1-13771, Sma-1-13773, Sma-1-13774, Sma-1-13775, Sma-1-13777, Sma-1-13779, Sma-1-1378, Sma-1-13781, Sma-1-13783, Sma-1-13785, Sma-1-13787, Sma-1-13789, Sma-1-1379, Sma-1-13791, Sma-1-13792, Sma-1-13793, Sma-1-13795, Sma-1-13797, Sma-1-13799, Sma-1-138, Sma-1-1380, Sma-1-13801, Sma-1-13803, Sma-1-13805, Sma-1-13806, Sma-1-13807, Sma-1-13809, Sma-1-1381, Sma-1-13811, Sma-1-13813, Sma-1-13815, Sma-1-13817, Sma-1-13819, Sma-1-1382, Sma-1-13821, Sma-1-13823, Sma-1-13824, Sma-1-13825, Sma-1-13826, Sma-1-13827, Sma-1-13828, Sma-1-13829, Sma-1-1383, Sma-1-13830, Sma-1-13831, Sma-1-13832, Sma-1-13833, Sma-1-13834, Sma-1-13835, Sma-1-13836, Sma-1-13837, Sma-1-13838, Sma-1-13839, Sma-1-1384, Sma-1-13840, Sma-1-13841, Sma-1-13843, Sma-1-13845, Sma-1-13847, Sma-1-13849, Sma-1-1385, Sma-1-13850, Sma-1-13851, Sma-1-13852, Sma-1-13853, Sma-1-13854, Sma-1-13855, Sma-1-13856, Sma-1-13857, Sma-1-13858, Sma-1-13859, Sma-1-1386, Sma-1-13860, Sma-1-13861, Sma-1-13862, Sma-1-13863, Sma-1-13864, Sma-1-13865, Sma-1-13866, Sma-1-13867, Sma-1-13868, Sma-1-13869, Sma-1-1387, Sma-1-13870, Sma-1-13871, Sma-1-13872, Sma-1-13873, Sma-1-13874, Sma-1-13875, Sma-1-13877, Sma-1-13879, Sma-1-1388, Sma-1-13881, Sma-1-13883, Sma-1-13885, Sma-1-13887, Sma-1-13889, Sma-1-1389, Sma-1-13890, Sma-1-13891, Sma-1-13893, Sma-1-13895, Sma-1-13897, Sma-1-13899, Sma-1-1390, Sma-1-13901, Sma-1-13903, Sma-1-13905, Sma-1-13906, Sma-1-13907, Sma-1-13909, Sma-1-13911, Sma-1-13912, Sma-1-13913, Sma-1-13915, Sma-1-13917, Sma-1-13919, Sma-1-1392, Sma-1-13921, Sma-1-13923, Sma-1-13925, Sma-1-13926, Sma-1-13927, Sma-1-13928, Sma-1-13929, Sma-1-13930, Sma-1-13931, Sma-1-13932, Sma-1-13933, Sma-1-13934, Sma-1-13935, Sma-1-13936, Sma-1-13937, Sma-1-13938, Sma-1-13939, Sma-1-1394, Sma-1-13940, Sma-1-13941, Sma-1-13942, Sma-1-13943, Sma-1-13944, Sma-1-13945, Sma-1-13946, Sma-1-13947, Sma-1-13948, Sma-1-13949, Sma-1-13950, Sma-1-13951, Sma-1-13952, Sma-1-13953, Sma-1-13954, Sma-1-13955, Sma-1-13957, Sma-1-13959, Sma-1-1396, Sma-1-13961, Sma-1-13963, Sma-1-13965, Sma-1-13967, Sma-1-13969, Sma-1-1397, Sma-1-13971, Sma-1-13973, Sma-1-13975, Sma-1-13977, Sma-1-13979, Sma-1-1398, Sma-1-13981, Sma-1-13982, Sma-1-13983, Sma-1-13984, Sma-1-13985, Sma-1-13987, Sma-1-13988, Sma-1-13989, Sma-1-13990, Sma-1-13991, Sma-1-13992, Sma-1-13993, Sma-1-13994, Sma-1-13995, Sma-1-13996, Sma-1-13997, Sma-1-13998, Sma-1-13999, Sma-1-14, Sma-1-140, Sma-1-1400, Sma-1-14000, Sma-1-14001, Sma-1-14002, Sma-1-14003, Sma-1-14004, Sma-1-14005, Sma-1-14006, Sma-1-14007, Sma-1-14008, Sma-1-14009, Sma-1-14010, Sma-1-14011, Sma-1-14013, Sma-1-14015, Sma-1-14017, Sma-1-14019, Sma-1-1402, Sma-1-14020, Sma-1-14021, Sma-1-14023, Sma-1-14025, Sma-1-14027, Sma-1-14029, Sma-1-14031, Sma-1-14033, Sma-1-14035, Sma-1-14037, Sma-1-14039, Sma-1-1404, Sma-1-14040, Sma-1-14041, Sma-1-14043, Sma-1-14045, Sma-1-14047, Sma-1-14049, Sma-1-14050, Sma-1-14051, Sma-1-14053, Sma-1-14055, Sma-1-14057, Sma-1-14058, Sma-1-14059, Sma-1-1406, Sma-1-14060, Sma-1-14061, Sma-1-14063, Sma-1-14065, Sma-1-14067, Sma-1-14069, Sma-1-14070, Sma-1-14071, Sma-1-14073, Sma-1-14075, Sma-1-14077, Sma-1-14079, Sma-1-1408, Sma-1-14080, Sma-1-14081, Sma-1-14082, Sma-1-14083, Sma-1-14084, Sma-1-14085, Sma-1-14086, Sma-1-14087, Sma-1-14088, Sma-1-14089, Sma-1-14090, Sma-1-14091, Sma-1-14092, Sma-1-14093, Sma-1-14095, Sma-1-14096, Sma-1-14097, Sma-1-14098, Sma-1-14099, Sma-1-1410, Sma-1-14100, Sma-1-14101, Sma-1-14103, Sma-1-14104, Sma-1-14105, Sma-1-14107, Sma-1-14109, Sma-1-1411, Sma-1-14111, Sma-1-14113, Sma-1-14114, Sma-1-14115, Sma-1-14116, Sma-1-14117, Sma-1-14118, Sma-1-14119, Sma-1-1412, Sma-1-14120, Sma-1-14121, Sma-1-14123, Sma-1-14125, Sma-1-14127, Sma-1-14129, Sma-1-14131, Sma-1-14133, Sma-1-14135, Sma-1-14137, Sma-1-14138, Sma-1-14139, Sma-1-1414, Sma-1-14140, Sma-1-14141, Sma-1-14143, Sma-1-14145, Sma-1-14147, Sma-1-14149, Sma-1-14151, Sma-1-14152, Sma-1-14153, Sma-1-14154, Sma-1-14155, Sma-1-14156, Sma-1-14157, Sma-

1-14158, Sma-1-14159, Sma-1-1416, Sma-1-14160, Sma-1-14161, Sma-1-14162, Sma-1-14163, Sma-1-14164, Sma-1-14165, Sma-1-14166, Sma-1-14167, Sma-1-14168, Sma-1-14169, Sma-1-1417, Sma-1-14170, Sma-1-14171, Sma-1-14172, Sma-1-14173, Sma-1-14174, Sma-1-14175, Sma-1-14176, Sma-1-14177, Sma-1-14178, Sma-1-14179, Sma-1-1418, Sma-1-14180, Sma-1-14181, Sma-1-14182, Sma-1-14183, Sma-1-14184, Sma-1-14185, Sma-1-14186, Sma-1-14187, Sma-1-14188, Sma-1-14189, Sma-1-14190, Sma-1-14191, Sma-1-14192, Sma-1-14193, Sma-1-14194, Sma-1-14195, Sma-1-14197, Sma-1-14199, Sma-1-142, Sma-1-1420, Sma-1-14201, Sma-1-14203, Sma-1-14204, Sma-1-14205, Sma-1-14207, Sma-1-14208, Sma-1-14209, Sma-1-14211, Sma-1-14213, Sma-1-14215, Sma-1-14217, Sma-1-14219, Sma-1-1422, Sma-1-14221, Sma-1-14223, Sma-1-14225, Sma-1-14227, Sma-1-14229, Sma-1-14231, Sma-1-14233, Sma-1-14234, Sma-1-14235, Sma-1-14237, Sma-1-14239, Sma-1-1424, Sma-1-14241, Sma-1-14243, Sma-1-14245, Sma-1-14247, Sma-1-14249, Sma-1-1425, Sma-1-14251, Sma-1-14253, Sma-1-14254, Sma-1-14255, Sma-1-14256, Sma-1-14257, Sma-1-14259, Sma-1-1426, Sma-1-14261, Sma-1-14262, Sma-1-14263, Sma-1-14264, Sma-1-14265, Sma-1-14266, Sma-1-14267, Sma-1-14269, Sma-1-14270, Sma-1-14271, Sma-1-14273, Sma-1-14275, Sma-1-14277, Sma-1-14278, Sma-1-14279, Sma-1-1428, Sma-1-14280, Sma-1-14281, Sma-1-14283, Sma-1-14285, Sma-1-14287, Sma-1-14289, Sma-1-14290, Sma-1-14291, Sma-1-14292, Sma-1-14293, Sma-1-14295, Sma-1-14297, Sma-1-14299, Sma-1-1430, Sma-1-14301, Sma-1-14302, Sma-1-14303, Sma-1-14304, Sma-1-14305, Sma-1-14306, Sma-1-14307, Sma-1-14309, Sma-1-14311, Sma-1-14313, Sma-1-14314, Sma-1-14315, Sma-1-14316, Sma-1-14317, Sma-1-14318, Sma-1-14319, Sma-1-1432, Sma-1-14320, Sma-1-14321, Sma-1-14322, Sma-1-14323, Sma-1-14324, Sma-1-14325, Sma-1-14326, Sma-1-14327, Sma-1-14328, Sma-1-14329, Sma-1-1433, Sma-1-14330, Sma-1-14331, Sma-1-14332, Sma-1-14333, Sma-1-14334, Sma-1-14335, Sma-1-14336, Sma-1-14337, Sma-1-14338, Sma-1-14339, Sma-1-1434, Sma-1-14340, Sma-1-14341, Sma-1-14342, Sma-1-14343, Sma-1-14344, Sma-1-14345, Sma-1-14346, Sma-1-14347, Sma-1-14348, Sma-1-14349, Sma-1-14350, Sma-1-14351, Sma-1-14352, Sma-1-14353, Sma-1-14354, Sma-1-14355, Sma-1-14356, Sma-1-14357, Sma-1-14358, Sma-1-14359, Sma-1-1436, Sma-1-14360, Sma-1-14361, Sma-1-14362, Sma-1-14363, Sma-1-14364, Sma-1-14365, Sma-1-14366, Sma-1-14367, Sma-1-14368, Sma-1-14369, Sma-1-14370, Sma-1-14371, Sma-1-14372, Sma-1-14373, Sma-1-14374, Sma-1-14375, Sma-1-14376, Sma-1-14377, Sma-1-14378, Sma-1-14379, Sma-1-1438, Sma-1-14380, Sma-1-14381, Sma-1-14382, Sma-1-14383, Sma-1-14385, Sma-1-14387, Sma-1-14388, Sma-1-14389, Sma-1-1439, Sma-1-14391, Sma-1-14393, Sma-1-14395, Sma-1-14397, Sma-1-14399, Sma-1-144, Sma-1-1440, Sma-1-14400, Sma-1-14401, Sma-1-14402, Sma-1-14403, Sma-1-14405, Sma-1-14407, Sma-1-14409, Sma-1-14411, Sma-1-14413, Sma-1-14415, Sma-1-14416, Sma-1-14417, Sma-1-14419, Sma-1-1442, Sma-1-14421, Sma-1-14423, Sma-1-14425, Sma-1-14427, Sma-1-14429, Sma-1-14430, Sma-1-14431, Sma-1-14433, Sma-1-14435, Sma-1-14437, Sma-1-14439, Sma-1-1444, Sma-1-14441, Sma-1-14443, Sma-1-14445, Sma-1-14446, Sma-1-14447, Sma-1-14449, Sma-1-14451, Sma-1-14453, Sma-1-14455, Sma-1-14457, Sma-1-14459, Sma-1-1446, Sma-1-14461, Sma-1-14463, Sma-1-14464, Sma-1-14465, Sma-1-14467, Sma-1-14469, Sma-1-14471, Sma-1-14473, Sma-1-14475, Sma-1-14477, Sma-1-14478, Sma-1-14479, Sma-1-1448, Sma-1-14480, Sma-1-14481, Sma-1-14482, Sma-1-14483, Sma-1-14484, Sma-1-14485, Sma-1-14486, Sma-1-14487, Sma-1-14488, Sma-1-14489, Sma-1-14490, Sma-1-14491, Sma-1-14492, Sma-1-14493, Sma-1-14494, Sma-1-14495, Sma-1-14496, Sma-1-14497, Sma-1-14498, Sma-1-14499, Sma-1-1450, Sma-1-14500, Sma-1-14501, Sma-1-14502, Sma-1-14503, Sma-1-14504, Sma-1-14505, Sma-1-14506, Sma-1-14507, Sma-1-14509, Sma-1-14511, Sma-1-14513, Sma-1-14515, Sma-1-14517, Sma-1-14519, Sma-1-1452, Sma-1-14521, Sma-1-14523, Sma-1-14524, Sma-1-14525, Sma-1-14527, Sma-1-14529, Sma-1-14531, Sma-1-14533, Sma-1-14535, Sma-1-14537, Sma-1-14539, Sma-1-1454, Sma-1-14540, Sma-1-14541, Sma-1-14543, Sma-1-14545, Sma-1-14547, Sma-1-14549, Sma-1-14551, Sma-1-14553, Sma-1-14555, Sma-1-14556, Sma-1-14557, Sma-1-14559, Sma-1-1456, Sma-1-14561, Sma-1-14563, Sma-1-14565, Sma-1-14567, Sma-1-14568, Sma-1-14569, Sma-1-14571, Sma-1-14573, Sma-1-14575, Sma-1-14577, Sma-1-14579, Sma-1-1458, Sma-1-14580, Sma-1-14581, Sma-1-14582, Sma-1-14583, Sma-1-14585, Sma-1-14587, Sma-1-14589, Sma-1-14590, Sma-1-14591, Sma-1-14592, Sma-1-14593, Sma-1-14595, Sma-1-14597, Sma-1-14599, Sma-1-146, Sma-1-1460, Sma-1-14601, Sma-1-14602, Sma-1-14603, Sma-1-14605, Sma-1-14606, Sma-1-14607, Sma-1-14608, Sma-1-14609, Sma-1-14610, Sma-1-14611, Sma-1-14612, Sma-1-14613, Sma-1-14614, Sma-1-14615, Sma-1-14617, Sma-1-14619, Sma-1-1462, Sma-1-14621, Sma-1-14623, Sma-1-14624, Sma-1-14625, Sma-1-14626, Sma-1-14627, Sma-1-14629, Sma-1-1463, Sma-1-14631, Sma-1-14633, Sma-1-14634, Sma-1-14635, Sma-1-14636, Sma-1-14637, Sma-1-14638, Sma-1-14639, Sma-1-1464, Sma-1-14640, Sma-1-14641, Sma-1-14642, Sma-1-14643, Sma-1-14645, Sma-1-14647, Sma-1-14648, Sma-1-14649, Sma-1-1465, Sma-1-14650, Sma-1-14651, Sma-1-14652, Sma-1-14653, Sma-1-14654, Sma-1-14655, Sma-1-14657, Sma-1-14659, Sma-1-1466, Sma-1-14661, Sma-1-14662, Sma-1-14663, Sma-1-14664, Sma-1-14665, Sma-1-14666, Sma-1-14667, Sma-1-14668, Sma-1-14669, Sma-1-14670, Sma-1-14671, Sma-1-14672, Sma-1-14673, Sma-1-14674, Sma-1-14675, Sma-1-14676, Sma-1-14677, Sma-1-14678, Sma-1-14679, Sma-1-1468, Sma-1-14680, Sma-1-14681, Sma-1-14682, Sma-1-14683, Sma-1-14684, Sma-1-14685, Sma-1-14686, Sma-1-14687, Sma-1-14688, Sma-1-14689, Sma-1-14690, Sma-1-14691, Sma-1-14692, Sma-1-14693, Sma-1-14694, Sma-1-14695, Sma-1-14696, Sma-1-14697, Sma-1-14698, Sma-1-14699, Sma-1-1470, Sma-1-14700, Sma-1-14701, Sma-1-14702, Sma-1-14703, Sma-1-14704, Sma-1-14705, Sma-1-14706, Sma-1-14707, Sma-1-14708, Sma-1-14709, Sma-1-14710, Sma-1-14711, Sma-1-14712, Sma-1-14713, Sma-1-14714, Sma-1-14715, Sma-1-14716, Sma-1-14717, Sma-1-14718, Sma-1-14719, Sma-1-1472, Sma-1-14720, Sma-1-14721, Sma-1-14722, Sma-1-14723, Sma-1-14724, Sma-1-14725, Sma-1-14726, Sma-1-14727, Sma-1-14728, Sma-1-14729, Sma-1-14730, Sma-1-14731, Sma-1-14732, Sma-1-14733, Sma-1-14734, Sma-1-14735, Sma-1-14736, Sma-1-14737, Sma-1-14739, Sma-1-1474, Sma-1-14740, Sma-1-14741, Sma-1-14742, Sma-1-14743, Sma-1-14744, Sma-1-14745, Sma-1-14746, Sma-1-14747, Sma-1-14748, Sma-1-14749, Sma-1-14750, Sma-1-14751, Sma-1-14752, Sma-1-14753, Sma-1-14754, Sma-1-14755, Sma-1-14756, Sma-1-14757, Sma-1-14758, Sma-1-14759, Sma-1-1476, Sma-1-14760, Sma-1-14761, Sma-1-14762, Sma-1-14763, Sma-1-14764, Sma-1-14765, Sma-1-14766, Sma-1-14767, Sma-1-14768, Sma-1-14769, Sma-1-14770, Sma-1-14771, Sma-1-14772, Sma-1-14773, Sma-1-14774, Sma-1-14775, Sma-1-14776, Sma-1-14777, Sma-1-14778, Sma-1-14779, Sma-1-1478, Sma-1-14780, Sma-1-14781, Sma-1-14782, Sma-1-14783, Sma-1-14784, Sma-

1-14785, Sma-1-14786, Sma-1-14787, Sma-1-14788, Sma-1-14789, Sma-1-14790, Sma-1-14791, Sma-1-14792, Sma-1-14793, Sma-1-14794, Sma-1-14795, Sma-1-14796, Sma-1-14797, Sma-1-14798, Sma-1-14799, Sma-1-148, Sma-1-1480, Sma-1-14800, Sma-1-14801, Sma-1-14802, Sma-1-14803, Sma-1-14804, Sma-1-14805, Sma-1-14807, Sma-1-14809, Sma-1-14811, Sma-1-14813, Sma-1-14814, Sma-1-14815, Sma-1-14817, Sma-1-14819, Sma-1-1482, Sma-1-14821, Sma-1-14823, Sma-1-14824, Sma-1-14825, Sma-1-14827, Sma-1-14829, Sma-1-14830, Sma-1-14831, Sma-1-14832, Sma-1-14833, Sma-1-14834, Sma-1-14835, Sma-1-14837, Sma-1-14839, Sma-1-1484, Sma-1-14841, Sma-1-14843, Sma-1-14844, Sma-1-14845, Sma-1-14846, Sma-1-14847, Sma-1-14849, Sma-1-14850, Sma-1-14851, Sma-1-14852, Sma-1-14853, Sma-1-14855, Sma-1-14857, Sma-1-14859, Sma-1-1486, Sma-1-14861, Sma-1-14863, Sma-1-14865, Sma-1-14867, Sma-1-14868, Sma-1-14869, Sma-1-14871, Sma-1-14872, Sma-1-14873, Sma-1-14875, Sma-1-14877, Sma-1-14879, Sma-1-1488, Sma-1-14880, Sma-1-14881, Sma-1-14882, Sma-1-14883, Sma-1-14885, Sma-1-14887, Sma-1-14889, Sma-1-14890, Sma-1-14891, Sma-1-14893, Sma-1-14895, Sma-1-14897, Sma-1-14898, Sma-1-14899, Sma-1-1490, Sma-1-14900, Sma-1-14901, Sma-1-14902, Sma-1-14903, Sma-1-14904, Sma-1-14905, Sma-1-14906, Sma-1-14907, Sma-1-14908, Sma-1-14909, Sma-1-14910, Sma-1-14911, Sma-1-14912, Sma-1-14913, Sma-1-14914, Sma-1-14915, Sma-1-14916, Sma-1-14917, Sma-1-14918, Sma-1-14919, Sma-1-1492, Sma-1-14920, Sma-1-14921, Sma-1-14922, Sma-1-14923, Sma-1-14924, Sma-1-14925, Sma-1-14926, Sma-1-14927, Sma-1-14928, Sma-1-14929, Sma-1-14930, Sma-1-14931, Sma-1-14932, Sma-1-14933, Sma-1-14934, Sma-1-14935, Sma-1-14936, Sma-1-14937, Sma-1-14938, Sma-1-14939, Sma-1-1494, Sma-1-14940, Sma-1-14941, Sma-1-14942, Sma-1-14943, Sma-1-14944, Sma-1-14945, Sma-1-14946, Sma-1-14947, Sma-1-14948, Sma-1-14949, Sma-1-14950, Sma-1-14951, Sma-1-14952, Sma-1-14953, Sma-1-14954, Sma-1-14955, Sma-1-14956, Sma-1-14957, Sma-1-14958, Sma-1-14959, Sma-1-1496, Sma-1-14960, Sma-1-14961, Sma-1-14962, Sma-1-14963, Sma-1-14965, Sma-1-14967, Sma-1-14969, Sma-1-14971, Sma-1-14973, Sma-1-14975, Sma-1-14976, Sma-1-14977, Sma-1-14979, Sma-1-1498, Sma-1-14981, Sma-1-14982, Sma-1-14983, Sma-1-14985, Sma-1-14987, Sma-1-14989, Sma-1-14990, Sma-1-14991, Sma-1-14992, Sma-1-14993, Sma-1-14994, Sma-1-14995, Sma-1-14997, Sma-1-14999, Sma-1-150, Sma-1-1500, Sma-1-15001, Sma-1-15003, Sma-1-15005, Sma-1-15007, Sma-1-15009, Sma-1-15011, Sma-1-15013, Sma-1-15015, Sma-1-15017, Sma-1-15019, Sma-1-1502, Sma-1-15021, Sma-1-15022, Sma-1-15023, Sma-1-15025, Sma-1-15027, Sma-1-15029, Sma-1-15030, Sma-1-15031, Sma-1-15033, Sma-1-15035, Sma-1-15037, Sma-1-15039, Sma-1-1504, Sma-1-15041, Sma-1-15043, Sma-1-15045, Sma-1-15047, Sma-1-15049, Sma-1-15051, Sma-1-15053, Sma-1-15055, Sma-1-15057, Sma-1-15059, Sma-1-1506, Sma-1-15061, Sma-1-15063, Sma-1-15065, Sma-1-15067, Sma-1-15069, Sma-1-15071, Sma-1-15073, Sma-1-15075, Sma-1-15077, Sma-1-15079, Sma-1-1508, Sma-1-15081, Sma-1-15083, Sma-1-15085, Sma-1-15087, Sma-1-15089, Sma-1-15091, Sma-1-15093, Sma-1-15095, Sma-1-15097, Sma-1-15099, Sma-1-151, Sma-1-1510, Sma-1-15101, Sma-1-15103, Sma-1-15105, Sma-1-15107, Sma-1-15109, Sma-1-15111, Sma-1-15113, Sma-1-15115, Sma-1-15117, Sma-1-15119, Sma-1-1512, Sma-1-15121, Sma-1-15123, Sma-1-15125, Sma-1-15127, Sma-1-15129, Sma-1-15131, Sma-1-15133, Sma-1-15134, Sma-1-15135, Sma-1-15137, Sma-1-15139, Sma-1-1514, Sma-1-15141, Sma-1-15143, Sma-1-15145, Sma-1-15147, Sma-1-15149, Sma-1-1515, Sma-1-15151, Sma-1-15153, Sma-1-15155, Sma-1-15157, Sma-1-15159, Sma-1-1516, Sma-1-15161, Sma-1-15163, Sma-1-15165, Sma-1-15167, Sma-1-15169, Sma-1-15171, Sma-1-15173, Sma-1-15175, Sma-1-15177, Sma-1-15179, Sma-1-1518, Sma-1-15181, Sma-1-15183, Sma-1-15185, Sma-1-15187, Sma-1-15189, Sma-1-15191, Sma-1-15193, Sma-1-15195, Sma-1-15197, Sma-1-15198, Sma-1-15199, Sma-1-152, Sma-1-1520, Sma-1-15201, Sma-1-15203, Sma-1-15205, Sma-1-15207, Sma-1-15209, Sma-1-15211, Sma-1-15213, Sma-1-15215, Sma-1-15217, Sma-1-15219, Sma-1-1522, Sma-1-15221, Sma-1-15223, Sma-1-15225, Sma-1-15227, Sma-1-15229, Sma-1-15231, Sma-1-15233, Sma-1-15235, Sma-1-15237, Sma-1-15239, Sma-1-1524, Sma-1-15241, Sma-1-15243, Sma-1-15245, Sma-1-15247, Sma-1-15249, Sma-1-1525, Sma-1-15251, Sma-1-15253, Sma-1-15255, Sma-1-15257, Sma-1-15259, Sma-1-1526, Sma-1-15261, Sma-1-15263, Sma-1-15265, Sma-1-15267, Sma-1-15269, Sma-1-15271, Sma-1-15273, Sma-1-15275, Sma-1-15277, Sma-1-15279, Sma-1-1528, Sma-1-15281, Sma-1-15283, Sma-1-15285, Sma-1-15287, Sma-1-15289, Sma-1-1529, Sma-1-15291, Sma-1-15293, Sma-1-15295, Sma-1-15297, Sma-1-15299, Sma-1-1530, Sma-1-15301, Sma-1-15303, Sma-1-15305, Sma-1-15307, Sma-1-15309, Sma-1-1531, Sma-1-15311, Sma-1-15313, Sma-1-15315, Sma-1-15317, Sma-1-15319, Sma-1-1532, Sma-1-15321, Sma-1-15323, Sma-1-15325, Sma-1-15327, Sma-1-15328, Sma-1-15329, Sma-1-1533, Sma-1-15330, Sma-1-15331, Sma-1-15333, Sma-1-15335, Sma-1-15337, Sma-1-15339, Sma-1-1534, Sma-1-15341, Sma-1-15343, Sma-1-15345, Sma-1-15347, Sma-1-15349, Sma-1-15351, Sma-1-15353, Sma-1-15355, Sma-1-15357, Sma-1-15359, Sma-1-1536, Sma-1-15361, Sma-1-15363, Sma-1-15365, Sma-1-15367, Sma-1-15369, Sma-1-15371, Sma-1-15373, Sma-1-15375, Sma-1-15377, Sma-1-15379, Sma-1-1538, Sma-1-15381, Sma-1-15383, Sma-1-15384, Sma-1-15385, Sma-1-15386, Sma-1-15387, Sma-1-15389, Sma-1-15391, Sma-1-15393, Sma-1-15395, Sma-1-15397, Sma-1-15399, Sma-1-154, Sma-1-1540, Sma-1-15401, Sma-1-15403, Sma-1-15405, Sma-1-15407, Sma-1-15408, Sma-1-15409, Sma-1-15411, Sma-1-15413, Sma-1-15415, Sma-1-15417, Sma-1-15419, Sma-1-1542, Sma-1-15421, Sma-1-15423, Sma-1-15425, Sma-1-15427, Sma-1-15428, Sma-1-15429, Sma-1-15431, Sma-1-15433, Sma-1-15435, Sma-1-15437, Sma-1-15439, Sma-1-1544, Sma-1-15441, Sma-1-15443, Sma-1-15445, Sma-1-15447, Sma-1-15449, Sma-1-1545, Sma-1-15451, Sma-1-15453, Sma-1-15455, Sma-1-15457, Sma-1-15459, Sma-1-1546, Sma-1-15461, Sma-1-15462, Sma-1-15463, Sma-1-15464, Sma-1-15465, Sma-1-15466, Sma-1-15467, Sma-1-15468, Sma-1-15469, Sma-1-1547, Sma-1-15470, Sma-1-15471, Sma-1-15472, Sma-1-15473, Sma-1-15474, Sma-1-15475, Sma-1-15477, Sma-1-15479, Sma-1-1548, Sma-1-15480, Sma-1-15481, Sma-1-15483, Sma-1-15485, Sma-1-15487, Sma-1-15489, Sma-1-1549, Sma-1-15491, Sma-1-15493, Sma-1-15495, Sma-1-15497, Sma-1-15499, Sma-1-1550, Sma-1-15501, Sma-1-15502, Sma-1-15503, Sma-1-15505, Sma-1-15507, Sma-1-15508, Sma-1-15509, Sma-1-1551, Sma-1-15510, Sma-1-15511, Sma-1-15512, Sma-1-15513, Sma-1-15514, Sma-1-15515, Sma-1-15517, Sma-1-15519, Sma-1-1552, Sma-1-15520, Sma-1-15521, Sma-1-15523, Sma-1-15525, Sma-1-15527, Sma-1-15529, Sma-1-1553, Sma-1-15530, Sma-1-15531, Sma-1-15533, Sma-1-15534, Sma-1-15535, Sma-1-15536, Sma-1-15537, Sma-1-15539, Sma-1-1554, Sma-1-15541, Sma-1-15543, Sma-1-15545, Sma-1-15547, Sma-1-15548, Sma-1-15549, Sma-1-1555, Sma-1-15551, Sma-1-15553, Sma-1-15555, Sma-1-15557, Sma-1-15558, Sma-1-15559, Sma-1-1556, Sma-1-15561, Sma-1-15563, Sma-1-15564, Sma-1-15565, Sma-

1-15566, Sma-1-15567, Sma-1-15568, Sma-1-15569, Sma-1-1557, Sma-1-15570, Sma-1-15571, Sma-1-15572, Sma-1-15573, Sma-1-15574, Sma-1-15575, Sma-1-15576, Sma-1-15577, Sma-1-15578, Sma-1-15579, Sma-1-1558, Sma-1-15580, Sma-1-15581, Sma-1-15582, Sma-1-15583, Sma-1-15585, Sma-1-15587, Sma-1-15588, Sma-1-15589, Sma-1-1559, Sma-1-15591, Sma-1-15593, Sma-1-15595, Sma-1-15597, Sma-1-15599, Sma-1-156, Sma-1-1560, Sma-1-15600, Sma-1-15601, Sma-1-15603, Sma-1-15605, Sma-1-15607, Sma-1-15608, Sma-1-15609, Sma-1-1561, Sma-1-15611, Sma-1-15613, Sma-1-15614, Sma-1-15615, Sma-1-15616, Sma-1-15617, Sma-1-15618, Sma-1-15619, Sma-1-1562, Sma-1-15620, Sma-1-15621, Sma-1-15622, Sma-1-15623, Sma-1-15624, Sma-1-15625, Sma-1-15626, Sma-1-15627, Sma-1-15629, Sma-1-1563, Sma-1-15630, Sma-1-15631, Sma-1-15633, Sma-1-15634, Sma-1-15635, Sma-1-15637, Sma-1-15639, Sma-1-1564, Sma-1-15641, Sma-1-15642, Sma-1-15643, Sma-1-15644, Sma-1-15645, Sma-1-15647, Sma-1-15648, Sma-1-15649, Sma-1-1565, Sma-1-15651, Sma-1-15653, Sma-1-15655, Sma-1-15657, Sma-1-15659, Sma-1-1566, Sma-1-15661, Sma-1-15663, Sma-1-15664, Sma-1-15665, Sma-1-15666, Sma-1-15667, Sma-1-15669, Sma-1-15671, Sma-1-15673, Sma-1-15675, Sma-1-15676, Sma-1-15677, Sma-1-15678, Sma-1-15679, Sma-1-1568, Sma-1-15680, Sma-1-15681, Sma-1-15682, Sma-1-15683, Sma-1-15684, Sma-1-15685, Sma-1-15686, Sma-1-15687, Sma-1-15689, Sma-1-15691, Sma-1-15692, Sma-1-15693, Sma-1-15695, Sma-1-15696, Sma-1-15697, Sma-1-15698, Sma-1-15699, Sma-1-1570, Sma-1-15701, Sma-1-15703, Sma-1-15705, Sma-1-15707, Sma-1-15708, Sma-1-15709, Sma-1-15711, Sma-1-15713, Sma-1-15715, Sma-1-15717, Sma-1-15718, Sma-1-15719, Sma-1-1572, Sma-1-15721, Sma-1-15723, Sma-1-15725, Sma-1-15726, Sma-1-15727, Sma-1-15729, Sma-1-15731, Sma-1-15733, Sma-1-15735, Sma-1-15736, Sma-1-15737, Sma-1-15738, Sma-1-15739, Sma-1-1574, Sma-1-15741, Sma-1-15743, Sma-1-15744, Sma-1-15745, Sma-1-15747, Sma-1-15748, Sma-1-15749, Sma-1-15750, Sma-1-15751, Sma-1-15753, Sma-1-15755, Sma-1-15756, Sma-1-15757, Sma-1-15758, Sma-1-15759, Sma-1-1576, Sma-1-15760, Sma-1-15761, Sma-1-15762, Sma-1-15763, Sma-1-15764, Sma-1-15765, Sma-1-15766, Sma-1-15767, Sma-1-15768, Sma-1-15769, Sma-1-15770, Sma-1-15771, Sma-1-15772, Sma-1-15773, Sma-1-15774, Sma-1-15775, Sma-1-15776, Sma-1-15777, Sma-1-15778, Sma-1-15779, Sma-1-1578, Sma-1-15780, Sma-1-15781, Sma-1-15782, Sma-1-15783, Sma-1-15785, Sma-1-15787, Sma-1-15788, Sma-1-15789, Sma-1-15791, Sma-1-15793, Sma-1-15795, Sma-1-15796, Sma-1-15797, Sma-1-15798, Sma-1-15799, Sma-1-158, Sma-1-1580, Sma-1-15801, Sma-1-15803, Sma-1-15805, Sma-1-15807, Sma-1-15809, Sma-1-15811, Sma-1-15812, Sma-1-15813, Sma-1-15814, Sma-1-15815, Sma-1-15817, Sma-1-15819, Sma-1-1582, Sma-1-15821, Sma-1-15823, Sma-1-15825, Sma-1-15827, Sma-1-15829, Sma-1-1583, Sma-1-15831, Sma-1-15833, Sma-1-15834, Sma-1-15835, Sma-1-15836, Sma-1-15837, Sma-1-15838, Sma-1-15839, Sma-1-1584, Sma-1-15840, Sma-1-15841, Sma-1-15842, Sma-1-15843, Sma-1-15844, Sma-1-15845, Sma-1-15846, Sma-1-15847, Sma-1-15848, Sma-1-15849, Sma-1-15850, Sma-1-15851, Sma-1-15853, Sma-1-15855, Sma-1-15857, Sma-1-15859, Sma-1-1586, Sma-1-15861, Sma-1-15862, Sma-1-15863, Sma-1-15864, Sma-1-15865, Sma-1-15867, Sma-1-15869, Sma-1-1587, Sma-1-15871, Sma-1-15873, Sma-1-15875, Sma-1-15877, Sma-1-15879, Sma-1-1588, Sma-1-15881, Sma-1-15883, Sma-1-15885, Sma-1-15887, Sma-1-15889, Sma-1-15891, Sma-1-15893, Sma-1-15894, Sma-1-15895, Sma-1-15897, Sma-1-15898, Sma-1-15899, Sma-1-1590, Sma-1-15901, Sma-1-15903, Sma-1-15904, Sma-1-15905, Sma-1-15907, Sma-1-15909, Sma-1-15911, Sma-1-15913, Sma-1-15914, Sma-1-15915, Sma-1-15917, Sma-1-15919, Sma-1-1592, Sma-1-15921, Sma-1-15923, Sma-1-15924, Sma-1-15925, Sma-1-15926, Sma-1-15927, Sma-1-15928, Sma-1-15929, Sma-1-15930, Sma-1-15931, Sma-1-15932, Sma-1-15933, Sma-1-15935, Sma-1-15937, Sma-1-15938, Sma-1-15939, Sma-1-1594, Sma-1-15941, Sma-1-15943, Sma-1-15945, Sma-1-15946, Sma-1-15947, Sma-1-15949, Sma-1-1595, Sma-1-15951, Sma-1-15953, Sma-1-15955, Sma-1-15957, Sma-1-15959, Sma-1-1596, Sma-1-15961, Sma-1-15963, Sma-1-15964, Sma-1-15965, Sma-1-15966, Sma-1-15967, Sma-1-15968, Sma-1-15969, Sma-1-15970, Sma-1-15971, Sma-1-15972, Sma-1-15973, Sma-1-15974, Sma-1-15975, Sma-1-15976, Sma-1-15977, Sma-1-15978, Sma-1-15979, Sma-1-1598, Sma-1-15980, Sma-1-15981, Sma-1-15982, Sma-1-15983, Sma-1-15984, Sma-1-15985, Sma-1-15986, Sma-1-15987, Sma-1-15988, Sma-1-15989, Sma-1-15990, Sma-1-15991, Sma-1-15992, Sma-1-15993, Sma-1-15994, Sma-1-15995, Sma-1-15996, Sma-1-15997, Sma-1-15998, Sma-1-15999, Sma-1-16, Sma-1-160, Sma-1-1600, Sma-1-16000, Sma-1-16001, Sma-1-16002, Sma-1-16003, Sma-1-16004, Sma-1-16005, Sma-1-16006, Sma-1-16007, Sma-1-16008, Sma-1-16009, Sma-1-16010, Sma-1-16011, Sma-1-16012, Sma-1-16013, Sma-1-16014, Sma-1-16015, Sma-1-16016, Sma-1-16017, Sma-1-16018, Sma-1-16019, Sma-1-1602, Sma-1-16020, Sma-1-16021, Sma-1-16022, Sma-1-16023, Sma-1-16025, Sma-1-16027, Sma-1-16028, Sma-1-16029, Sma-1-16031, Sma-1-16033, Sma-1-16035, Sma-1-16037, Sma-1-16039, Sma-1-1604, Sma-1-16041, Sma-1-16043, Sma-1-16044, Sma-1-16045, Sma-1-16047, Sma-1-16049, Sma-1-16050, Sma-1-16051, Sma-1-16053, Sma-1-16055, Sma-1-16057, Sma-1-16059, Sma-1-1606, Sma-1-16061, Sma-1-16063, Sma-1-16065, Sma-1-16067, Sma-1-16069, Sma-1-16071, Sma-1-16073, Sma-1-16075, Sma-1-16077, Sma-1-16079, Sma-1-1608, Sma-1-16081, Sma-1-16083, Sma-1-16085, Sma-1-16086, Sma-1-16087, Sma-1-16089, Sma-1-16091, Sma-1-16093, Sma-1-16095, Sma-1-16097, Sma-1-16098, Sma-1-16099, Sma-1-1610, Sma-1-16101, Sma-1-16102, Sma-1-16103, Sma-1-16105, Sma-1-16107, Sma-1-16108, Sma-1-16109, Sma-1-16110, Sma-1-16111, Sma-1-16112, Sma-1-16113, Sma-1-16114, Sma-1-16115, Sma-1-16117, Sma-1-16119, Sma-1-1612, Sma-1-16120, Sma-1-16121, Sma-1-16122, Sma-1-16123, Sma-1-16125, Sma-1-16126, Sma-1-16127, Sma-1-16128, Sma-1-16129, Sma-1-16130, Sma-1-16131, Sma-1-16132, Sma-1-16133, Sma-1-16135, Sma-1-16136, Sma-1-16137, Sma-1-16138, Sma-1-16139, Sma-1-1614, Sma-1-16140, Sma-1-16141, Sma-1-16143, Sma-1-16145, Sma-1-16146, Sma-1-16147, Sma-1-16149, Sma-1-16151, Sma-1-16152, Sma-1-16153, Sma-1-16155, Sma-1-16157, Sma-1-16159, Sma-1-1616, Sma-1-16161, Sma-1-16162, Sma-1-16163, Sma-1-16164, Sma-1-16165, Sma-1-16166, Sma-1-16167, Sma-1-16168, Sma-1-16169, Sma-1-16170, Sma-1-16171, Sma-1-16172, Sma-1-16173, Sma-1-16174, Sma-1-16175, Sma-1-16176, Sma-1-16177, Sma-1-16179, Sma-1-1618, Sma-1-16181, Sma-1-16182, Sma-1-16183, Sma-1-16185, Sma-1-16187, Sma-1-16189, Sma-1-16190, Sma-1-16191, Sma-1-16192, Sma-1-16193, Sma-1-16194, Sma-1-16195, Sma-1-16196, Sma-1-16197, Sma-1-16198, Sma-1-16199, Sma-1-162, Sma-1-1620, Sma-1-16200, Sma-1-16201, Sma-1-16202, Sma-1-16203, Sma-1-16205, Sma-1-16207, Sma-1-16209, Sma-1-16210, Sma-1-16211, Sma-1-16213, Sma-1-16215, Sma-1-16217, Sma-1-16219, Sma-1-1622, Sma-1-16220, Sma-1-16221, Sma-1-16223, Sma-1-16224, Sma-1-16225, Sma-1-16227, Sma-1-16229, Sma-1-16231, Sma-1-16232, Sma-1-16233, Sma-1-

16235, Sma-1-16237, Sma-1-16239, Sma-1-1624, Sma-1-16241, Sma-1-16242, Sma-1-16243, Sma-1-16244, Sma-1-16245, Sma-1-16247, Sma-1-16249, Sma-1-1625, Sma-1-16251, Sma-1-16253, Sma-1-16254, Sma-1-16255, Sma-1-16256, Sma-1-16257, Sma-1-16258, Sma-1-16259, Sma-1-1626, Sma-1-16261, Sma-1-16263, Sma-1-16264, Sma-1-16265, Sma-1-16267, Sma-1-16269, Sma-1-16271, Sma-1-16273, Sma-1-16275, Sma-1-16277, Sma-1-16278, Sma-1-16279, Sma-1-1628, Sma-1-16280, Sma-1-16281, Sma-1-16282, Sma-1-16283, Sma-1-16285, Sma-1-16287, Sma-1-16289, Sma-1-1629, Sma-1-16291, Sma-1-16292, Sma-1-16293, Sma-1-16294, Sma-1-16295, Sma-1-16297, Sma-1-16299, Sma-1-1630, Sma-1-16301, Sma-1-16302, Sma-1-16303, Sma-1-16305, Sma-1-16306, Sma-1-16307, Sma-1-16308, Sma-1-16309, Sma-1-16310, Sma-1-16311, Sma-1-16312, Sma-1-16313, Sma-1-16314, Sma-1-16315, Sma-1-16316, Sma-1-16317, Sma-1-16318, Sma-1-16319, Sma-1-1632, Sma-1-16320, Sma-1-16321, Sma-1-16322, Sma-1-16323, Sma-1-16324, Sma-1-16325, Sma-1-16326, Sma-1-16327, Sma-1-16329, Sma-1-16331, Sma-1-16332, Sma-1-16333, Sma-1-16335, Sma-1-16337, Sma-1-16338, Sma-1-16339, Sma-1-1634, Sma-1-16341, Sma-1-16342, Sma-1-16343, Sma-1-16345, Sma-1-16347, Sma-1-16349, Sma-1-16350, Sma-1-16351, Sma-1-16353, Sma-1-16355, Sma-1-16357, Sma-1-16359, Sma-1-1636, Sma-1-16361, Sma-1-16362, Sma-1-16363, Sma-1-16364, Sma-1-16365, Sma-1-16367, Sma-1-16369, Sma-1-1637, Sma-1-16370, Sma-1-16371, Sma-1-16372, Sma-1-16373, Sma-1-16374, Sma-1-16375, Sma-1-16376, Sma-1-16377, Sma-1-16378, Sma-1-16379, Sma-1-1638, Sma-1-16380, Sma-1-16381, Sma-1-16382, Sma-1-16383, Sma-1-16384, Sma-1-16385, Sma-1-16386, Sma-1-16387, Sma-1-16388, Sma-1-16389, Sma-1-16390, Sma-1-16391, Sma-1-16392, Sma-1-16393, Sma-1-16394, Sma-1-16395, Sma-1-16396, Sma-1-16397, Sma-1-16398, Sma-1-16399, Sma-1-164, Sma-1-1640, Sma-1-16400, Sma-1-16401, Sma-1-16402, Sma-1-16403, Sma-1-16404, Sma-1-16405, Sma-1-16406, Sma-1-16407, Sma-1-16408, Sma-1-16409, Sma-1-1641, Sma-1-16410, Sma-1-16411, Sma-1-16412, Sma-1-16413, Sma-1-16414, Sma-1-16415, Sma-1-16416, Sma-1-16417, Sma-1-16418, Sma-1-16419, Sma-1-1642, Sma-1-16420, Sma-1-16421, Sma-1-16422, Sma-1-16423, Sma-1-16424, Sma-1-16425, Sma-1-16426, Sma-1-16427, Sma-1-16428, Sma-1-16429, Sma-1-1643, Sma-1-16430, Sma-1-16431, Sma-1-16432, Sma-1-16433, Sma-1-16434, Sma-1-16435, Sma-1-16436, Sma-1-16437, Sma-1-16438, Sma-1-16439, Sma-1-1644, Sma-1-16440, Sma-1-16441, Sma-1-16442, Sma-1-16443, Sma-1-16444, Sma-1-16445, Sma-1-16446, Sma-1-16447, Sma-1-16448, Sma-1-16449, Sma-1-16450, Sma-1-16451, Sma-1-16452, Sma-1-16453, Sma-1-16454, Sma-1-16455, Sma-1-16456, Sma-1-16457, Sma-1-16458, Sma-1-16459, Sma-1-1646, Sma-1-16460, Sma-1-16461, Sma-1-16462, Sma-1-16463, Sma-1-16464, Sma-1-16465, Sma-1-16466, Sma-1-16467, Sma-1-16468, Sma-1-16469, Sma-1-16470, Sma-1-16471, Sma-1-16472, Sma-1-16473, Sma-1-16474, Sma-1-16475, Sma-1-16476, Sma-1-16477, Sma-1-16478, Sma-1-16479, Sma-1-1648, Sma-1-16480, Sma-1-16481, Sma-1-16482, Sma-1-16483, Sma-1-16484, Sma-1-16485, Sma-1-16486, Sma-1-16487, Sma-1-16488, Sma-1-16489, Sma-1-16490, Sma-1-16491, Sma-1-16492, Sma-1-16493, Sma-1-16494, Sma-1-16495, Sma-1-16496, Sma-1-16497, Sma-1-16498, Sma-1-16499, Sma-1-1650, Sma-1-16500, Sma-1-16501, Sma-1-16502, Sma-1-16503, Sma-1-16504, Sma-1-16505, Sma-1-16506, Sma-1-16507, Sma-1-16508, Sma-1-16509, Sma-1-16510, Sma-1-16511, Sma-1-16512, Sma-1-16513, Sma-1-16514, Sma-1-16515, Sma-1-16516, Sma-1-16517, Sma-1-16518, Sma-1-16519, Sma-1-1652, Sma-1-16520, Sma-1-16521, Sma-1-16522, Sma-1-16523, Sma-1-16524, Sma-1-16525, Sma-1-16526, Sma-1-16527, Sma-1-16528, Sma-1-16529, Sma-1-16530, Sma-1-16531, Sma-1-16532, Sma-1-16533, Sma-1-16534, Sma-1-16535, Sma-1-16536, Sma-1-16537, Sma-1-16538, Sma-1-16539, Sma-1-1654, Sma-1-16540, Sma-1-16541, Sma-1-16542, Sma-1-16543, Sma-1-16544, Sma-1-16545, Sma-1-16546, Sma-1-16547, Sma-1-16548, Sma-1-16549, Sma-1-16550, Sma-1-16551, Sma-1-16552, Sma-1-16553, Sma-1-16554, Sma-1-16555, Sma-1-16556, Sma-1-16557, Sma-1-16558, Sma-1-16559, Sma-1-1656, Sma-1-16560, Sma-1-16561, Sma-1-16562, Sma-1-16563, Sma-1-16564, Sma-1-16565, Sma-1-16566, Sma-1-16567, Sma-1-16568, Sma-1-16569, Sma-1-16570, Sma-1-16571, Sma-1-16572, Sma-1-16573, Sma-1-16574, Sma-1-16575, Sma-1-16576, Sma-1-16577, Sma-1-16578, Sma-1-16579, Sma-1-1658, Sma-1-16580, Sma-1-16581, Sma-1-16582, Sma-1-16583, Sma-1-16584, Sma-1-16585, Sma-1-16586, Sma-1-16587, Sma-1-16588, Sma-1-16589, Sma-1-16590, Sma-1-16591, Sma-1-16592, Sma-1-16593, Sma-1-16594, Sma-1-16595, Sma-1-16596, Sma-1-16597, Sma-1-16598, Sma-1-16599, Sma-1-166, Sma-1-16600, Sma-1-16601, Sma-1-16602, Sma-1-16603, Sma-1-16604, Sma-1-16605, Sma-1-16606, Sma-1-16607, Sma-1-16608, Sma-1-16609, Sma-1-1661, Sma-1-16610, Sma-1-16611, Sma-1-16612, Sma-1-16613, Sma-1-16614, Sma-1-16615, Sma-1-16616, Sma-1-16617, Sma-1-16618, Sma-1-16619, Sma-1-16620, Sma-1-16621, Sma-1-16622, Sma-1-16623, Sma-1-16624, Sma-1-16625, Sma-1-16626, Sma-1-16627, Sma-1-16628, Sma-1-16629, Sma-1-1663, Sma-1-16630, Sma-1-16631, Sma-1-16632, Sma-1-16633, Sma-1-16634, Sma-1-16635, Sma-1-16636, Sma-1-16637, Sma-1-16638, Sma-1-16639, Sma-1-16640, Sma-1-16641, Sma-1-16642, Sma-1-16643, Sma-1-16644, Sma-1-16645, Sma-1-16646, Sma-1-16647, Sma-1-16648, Sma-1-16649, Sma-1-1665, Sma-1-16650, Sma-1-16651, Sma-1-16652, Sma-1-16653, Sma-1-16654, Sma-1-16655, Sma-1-16656, Sma-1-16657, Sma-1-16658, Sma-1-16659, Sma-1-16660, Sma-1-16661, Sma-1-16662, Sma-1-16663, Sma-1-16664, Sma-1-16665, Sma-1-16666, Sma-1-16667, Sma-1-16668, Sma-1-16669, Sma-1-1667, Sma-1-16670, Sma-1-16671, Sma-1-16672, Sma-1-16673, Sma-1-16674, Sma-1-16675, Sma-1-16676, Sma-1-16677, Sma-1-16678, Sma-1-16679, Sma-1-16680, Sma-1-16681, Sma-1-16682, Sma-1-16683, Sma-1-16684, Sma-1-16685, Sma-1-16686, Sma-1-16687, Sma-1-16688, Sma-1-16689, Sma-1-1669, Sma-1-16690, Sma-1-16691, Sma-1-16692, Sma-1-16693, Sma-1-16694, Sma-1-16695, Sma-1-16696, Sma-1-16697, Sma-1-16698, Sma-1-16699, Sma-1-16700, Sma-1-16701, Sma-1-16702, Sma-1-16703, Sma-1-16704, Sma-1-16705, Sma-1-16706, Sma-1-16707, Sma-1-16708, Sma-1-16709, Sma-1-1671, Sma-1-16710, Sma-1-16711, Sma-1-16712, Sma-1-16713, Sma-1-16714, Sma-1-16715, Sma-1-16716, Sma-1-16717, Sma-1-16718, Sma-1-16719, Sma-1-16720, Sma-1-16721, Sma-1-16722, Sma-1-16723, Sma-1-16724, Sma-1-16725, Sma-1-16727, Sma-1-16729, Sma-1-1673, Sma-1-16730, Sma-1-16731, Sma-1-16733, Sma-1-16734, Sma-1-16735, Sma-1-16737, Sma-1-16739, Sma-1-16741, Sma-1-16743, Sma-1-16744, Sma-1-16745, Sma-1-16746, Sma-1-16747, Sma-1-16748, Sma-1-16749, Sma-1-1675, Sma-1-16750, Sma-1-16751, Sma-1-16753, Sma-1-16754, Sma-1-16755, Sma-1-16757, Sma-1-16759, Sma-1-16760, Sma-1-16761, Sma-1-16762, Sma-1-16763, Sma-1-16764, Sma-1-16765, Sma-1-16766, Sma-1-16767, Sma-1-16768, Sma-1-16769, Sma-1-1677, Sma-1-16770, Sma-1-16771, Sma-1-16772, Sma-1-16773, Sma-1-16774, Sma-1-16775, Sma-1-16776, Sma-1-16777, Sma-1-16778, Sma-1-16779, Sma-

1-16780, Sma-1-16781, Sma-1-16783, Sma-1-16785, Sma-1-16787, Sma-1-16789, Sma-1-1679, Sma-1-16791, Sma-1-16793, Sma-1-16795, Sma-1-16797, Sma-1-16798, Sma-1-16799, Sma-1-168, Sma-1-16801, Sma-1-16803, Sma-1-16804, Sma-1-16805, Sma-1-16807, Sma-1-16808, Sma-1-16809, Sma-1-1681, Sma-1-16810, Sma-1-16811, Sma-1-16812, Sma-1-16813, Sma-1-16814, Sma-1-16815, Sma-1-16817, Sma-1-16819, Sma-1-16821, Sma-1-16822, Sma-1-16823, Sma-1-16825, Sma-1-16826, Sma-1-16827, Sma-1-16829, Sma-1-1683, Sma-1-16831, Sma-1-16833, Sma-1-16835, Sma-1-16837, Sma-1-16839, Sma-1-16841, Sma-1-16843, Sma-1-16845, Sma-1-16846, Sma-1-16847, Sma-1-16848, Sma-1-16849, Sma-1-1685, Sma-1-16850, Sma-1-16851, Sma-1-16852, Sma-1-16853, Sma-1-16854, Sma-1-16855, Sma-1-16856, Sma-1-16857, Sma-1-16858, Sma-1-16859, Sma-1-16861, Sma-1-16863, Sma-1-16864, Sma-1-16865, Sma-1-16867, Sma-1-16869, Sma-1-1687, Sma-1-16870, Sma-1-16871, Sma-1-16873, Sma-1-16875, Sma-1-16876, Sma-1-16877, Sma-1-16879, Sma-1-16881, Sma-1-16883, Sma-1-16885, Sma-1-16886, Sma-1-16887, Sma-1-16889, Sma-1-1689, Sma-1-16891, Sma-1-16893, Sma-1-16895, Sma-1-16896, Sma-1-16897, Sma-1-16898, Sma-1-16899, Sma-1-16901, Sma-1-16903, Sma-1-16904, Sma-1-16905, Sma-1-16906, Sma-1-16907, Sma-1-16909, Sma-1-1691, Sma-1-16911, Sma-1-16913, Sma-1-16915, Sma-1-16917, Sma-1-16919, Sma-1-16921, Sma-1-16923, Sma-1-16925, Sma-1-16927, Sma-1-16929, Sma-1-1693, Sma-1-16931, Sma-1-16933, Sma-1-16935, Sma-1-16937, Sma-1-16939, Sma-1-16941, Sma-1-16943, Sma-1-16945, Sma-1-16947, Sma-1-16949, Sma-1-1695, Sma-1-16951, Sma-1-16953, Sma-1-16955, Sma-1-16957, Sma-1-16959, Sma-1-16961, Sma-1-16963, Sma-1-16965, Sma-1-16967, Sma-1-16969, Sma-1-1697, Sma-1-16971, Sma-1-16973, Sma-1-16975, Sma-1-16977, Sma-1-16979, Sma-1-16981, Sma-1-16983, Sma-1-16985, Sma-1-16987, Sma-1-16989, Sma-1-1699, Sma-1-16991, Sma-1-16993, Sma-1-16995, Sma-1-16997, Sma-1-16999, Sma-1-170, Sma-1-17001, Sma-1-17003, Sma-1-17005, Sma-1-17007, Sma-1-17009, Sma-1-1701, Sma-1-17011, Sma-1-17013, Sma-1-17015, Sma-1-17017, Sma-1-17019, Sma-1-17021, Sma-1-17023, Sma-1-17025, Sma-1-17027, Sma-1-17029, Sma-1-1703, Sma-1-17031, Sma-1-17033, Sma-1-17035, Sma-1-17037, Sma-1-17039, Sma-1-17041, Sma-1-17043, Sma-1-17045, Sma-1-17047, Sma-1-17049, Sma-1-1705, Sma-1-17051, Sma-1-17053, Sma-1-17055, Sma-1-17057, Sma-1-17059, Sma-1-17061, Sma-1-17063, Sma-1-17065, Sma-1-17067, Sma-1-17069, Sma-1-1707, Sma-1-17071, Sma-1-17073, Sma-1-17075, Sma-1-17077, Sma-1-17079, Sma-1-17080, Sma-1-17081, Sma-1-17083, Sma-1-17085, Sma-1-17087, Sma-1-17089, Sma-1-1709, Sma-1-17091, Sma-1-17093, Sma-1-17095, Sma-1-17097, Sma-1-17099, Sma-1-17101, Sma-1-17103, Sma-1-17105, Sma-1-17107, Sma-1-17109, Sma-1-1711, Sma-1-17111, Sma-1-17113, Sma-1-17115, Sma-1-17117, Sma-1-17119, Sma-1-17121, Sma-1-17123, Sma-1-17125, Sma-1-17127, Sma-1-17129, Sma-1-1713, Sma-1-17131, Sma-1-17133, Sma-1-17135, Sma-1-17137, Sma-1-17139, Sma-1-17141, Sma-1-17143, Sma-1-17145, Sma-1-17147, Sma-1-17149, Sma-1-1715, Sma-1-17151, Sma-1-17153, Sma-1-17155, Sma-1-17157, Sma-1-17159, Sma-1-17161, Sma-1-17163, Sma-1-17165, Sma-1-17167, Sma-1-17169, Sma-1-1717, Sma-1-17171, Sma-1-17173, Sma-1-17175, Sma-1-17177, Sma-1-17179, Sma-1-17181, Sma-1-17183, Sma-1-17185, Sma-1-17187, Sma-1-17189, Sma-1-1719, Sma-1-17191, Sma-1-17193, Sma-1-17195, Sma-1-17197, Sma-1-17199, Sma-1-172, Sma-1-17201, Sma-1-17203, Sma-1-17205, Sma-1-17207, Sma-1-17209, Sma-1-1721, Sma-1-17211, Sma-1-17213, Sma-1-17215, Sma-1-17217, Sma-1-17219, Sma-1-17221, Sma-1-17223, Sma-1-17225, Sma-1-17227, Sma-1-17229, Sma-1-1723, Sma-1-17231, Sma-1-17233, Sma-1-17235, Sma-1-17237, Sma-1-17239, Sma-1-17241, Sma-1-17243, Sma-1-17245, Sma-1-17247, Sma-1-17249, Sma-1-1725, Sma-1-17251, Sma-1-17253, Sma-1-17255, Sma-1-17257, Sma-1-17259, Sma-1-17261, Sma-1-17263, Sma-1-17265, Sma-1-17267, Sma-1-17269, Sma-1-1727, Sma-1-17271, Sma-1-17273, Sma-1-17275, Sma-1-17277, Sma-1-17279, Sma-1-17281, Sma-1-17283, Sma-1-17285, Sma-1-17287, Sma-1-17289, Sma-1-1729, Sma-1-17291, Sma-1-17292, Sma-1-17293, Sma-1-17295, Sma-1-17297, Sma-1-17299, Sma-1-17301, Sma-1-17303, Sma-1-17305, Sma-1-17307, Sma-1-17309, Sma-1-1731, Sma-1-17311, Sma-1-17313, Sma-1-17315, Sma-1-17317, Sma-1-17319, Sma-1-17321, Sma-1-17323, Sma-1-17325, Sma-1-17327, Sma-1-17329, Sma-1-1733, Sma-1-17331, Sma-1-17333, Sma-1-17335, Sma-1-17337, Sma-1-17339, Sma-1-17341, Sma-1-17343, Sma-1-17345, Sma-1-17347, Sma-1-17349, Sma-1-1735, Sma-1-17351, Sma-1-17353, Sma-1-17355, Sma-1-17357, Sma-1-17359, Sma-1-17361, Sma-1-17363, Sma-1-17365, Sma-1-17367, Sma-1-17369, Sma-1-1737, Sma-1-17371, Sma-1-17373, Sma-1-17375, Sma-1-17377, Sma-1-17379, Sma-1-1738, Sma-1-17381, Sma-1-17383, Sma-1-17385, Sma-1-17387, Sma-1-17389, Sma-1-1739, Sma-1-17391, Sma-1-17393, Sma-1-17395, Sma-1-17397, Sma-1-17399, Sma-1-174, Sma-1-1740, Sma-1-17401, Sma-1-17403, Sma-1-17405, Sma-1-17407, Sma-1-17409, Sma-1-1741, Sma-1-17411, Sma-1-17413, Sma-1-17415, Sma-1-17416, Sma-1-17417, Sma-1-17419, Sma-1-1742, Sma-1-17421, Sma-1-17423, Sma-1-17425, Sma-1-17427, Sma-1-17429, Sma-1-1743, Sma-1-17431, Sma-1-17433, Sma-1-17435, Sma-1-17437, Sma-1-17439, Sma-1-1744, Sma-1-17441, Sma-1-17443, Sma-1-17445, Sma-1-17447, Sma-1-17449, Sma-1-1745, Sma-1-17451, Sma-1-17453, Sma-1-17455, Sma-1-17457, Sma-1-17459, Sma-1-17461, Sma-1-17463, Sma-1-17465, Sma-1-17467, Sma-1-17469, Sma-1-1747, Sma-1-17471, Sma-1-17473, Sma-1-17475, Sma-1-17477, Sma-1-17479, Sma-1-17481, Sma-1-17483, Sma-1-17485, Sma-1-17487, Sma-1-17489, Sma-1-1749, Sma-1-17491, Sma-1-17493, Sma-1-17495, Sma-1-17497, Sma-1-17499, Sma-1-17501, Sma-1-17503, Sma-1-17505, Sma-1-17507, Sma-1-17509, Sma-1-1751, Sma-1-17511, Sma-1-17513, Sma-1-17515, Sma-1-17517, Sma-1-17519, Sma-1-17520, Sma-1-17521, Sma-1-17522, Sma-1-17523, Sma-1-17525, Sma-1-17527, Sma-1-17529, Sma-1-1753, Sma-1-17531, Sma-1-17533, Sma-1-17535, Sma-1-17537, Sma-1-17539, Sma-1-17541, Sma-1-17543, Sma-1-17545, Sma-1-17547, Sma-1-17549, Sma-1-1755, Sma-1-17551, Sma-1-17553, Sma-1-17555, Sma-1-17557, Sma-1-17559, Sma-1-17561, Sma-1-17563, Sma-1-17565, Sma-1-17567, Sma-1-17569, Sma-1-1757, Sma-1-17571, Sma-1-17573, Sma-1-17575, Sma-1-17577, Sma-1-17579, Sma-1-17581, Sma-1-17583, Sma-1-17585, Sma-1-17587, Sma-1-17589, Sma-1-1759, Sma-1-17591, Sma-1-17593, Sma-1-17595, Sma-1-17597, Sma-1-17599, Sma-1-176, Sma-1-17601, Sma-1-17603, Sma-1-17605, Sma-1-17607, Sma-1-17609, Sma-1-1761, Sma-1-17611, Sma-1-17613, Sma-1-17615, Sma-1-17617, Sma-1-17619, Sma-1-17621, Sma-1-17623, Sma-1-17625, Sma-1-17627, Sma-1-17629, Sma-1-1763, Sma-1-17631, Sma-1-17633, Sma-1-17635, Sma-1-17637, Sma-1-17639, Sma-1-17641, Sma-1-17643, Sma-1-17645, Sma-1-17647, Sma-1-17649, Sma-1-1765, Sma-1-17651, Sma-1-17653, Sma-1-17655, Sma-1-17657, Sma-1-17659, Sma-1-1766, Sma-1-17661, Sma-1-17663, Sma-1-17665, Sma-1-17667, Sma-1-17669, Sma-1-1767, Sma-1-17671, Sma-1-17673, Sma-1-17675, Sma-1-17677, Sma-1-17679, Sma-1-17681, Sma-1-

17683, Sma-1-17685, Sma-1-17687, Sma-1-17689, Sma-1-1769, Sma-1-17691, Sma-1-17693, Sma-1-17695, Sma-1-17697, Sma-1-17699, Sma-1-1770, Sma-1-17701, Sma-1-17703, Sma-1-17705, Sma-1-17707, Sma-1-17709, Sma-1-1771, Sma-1-17711, Sma-1-17713, Sma-1-17715, Sma-1-17717, Sma-1-17719, Sma-1-17721, Sma-1-17723, Sma-1-17725, Sma-1-17727, Sma-1-17729, Sma-1-1773, Sma-1-17731, Sma-1-17733, Sma-1-17735, Sma-1-17737, Sma-1-17739, Sma-1-17741, Sma-1-17743, Sma-1-17745, Sma-1-17747, Sma-1-17749, Sma-1-1775, Sma-1-17751, Sma-1-17753, Sma-1-17755, Sma-1-17757, Sma-1-17759, Sma-1-1776, Sma-1-17761, Sma-1-17763, Sma-1-17765, Sma-1-17767, Sma-1-17769, Sma-1-1777, Sma-1-17771, Sma-1-17773, Sma-1-17775, Sma-1-17777, Sma-1-17779, Sma-1-17781, Sma-1-17783, Sma-1-17785, Sma-1-17787, Sma-1-17789, Sma-1-1779, Sma-1-17791, Sma-1-17793, Sma-1-17795, Sma-1-17797, Sma-1-17799, Sma-1-178, Sma-1-17801, Sma-1-17803, Sma-1-17805, Sma-1-17807, Sma-1-17809, Sma-1-1781, Sma-1-17811, Sma-1-17813, Sma-1-17815, Sma-1-17817, Sma-1-17819, Sma-1-17821, Sma-1-17822, Sma-1-17823, Sma-1-17825, Sma-1-17827, Sma-1-17829, Sma-1-1783, Sma-1-17831, Sma-1-17833, Sma-1-17835, Sma-1-17837, Sma-1-17839, Sma-1-17841, Sma-1-17843, Sma-1-17845, Sma-1-17847, Sma-1-17849, Sma-1-1785, Sma-1-17851, Sma-1-17853, Sma-1-17855, Sma-1-17857, Sma-1-17859, Sma-1-1786, Sma-1-17861, Sma-1-17863, Sma-1-17865, Sma-1-17867, Sma-1-17869, Sma-1-1787, Sma-1-17871, Sma-1-17873, Sma-1-17875, Sma-1-17877, Sma-1-17879, Sma-1-17881, Sma-1-17883, Sma-1-17885, Sma-1-17887, Sma-1-17889, Sma-1-1789, Sma-1-17891, Sma-1-17893, Sma-1-17895, Sma-1-17897, Sma-1-17899, Sma-1-17900, Sma-1-17901, Sma-1-17903, Sma-1-17905, Sma-1-17907, Sma-1-17909, Sma-1-1791, Sma-1-17911, Sma-1-17913, Sma-1-17915, Sma-1-17917, Sma-1-17919, Sma-1-17921, Sma-1-17923, Sma-1-17925, Sma-1-17927, Sma-1-17929, Sma-1-1793, Sma-1-17931, Sma-1-17933, Sma-1-17935, Sma-1-17937, Sma-1-17939, Sma-1-1794, Sma-1-17941, Sma-1-17943, Sma-1-17945, Sma-1-17947, Sma-1-17949, Sma-1-1795, Sma-1-17950, Sma-1-17951, Sma-1-17953, Sma-1-17955, Sma-1-17957, Sma-1-17959, Sma-1-17961, Sma-1-17963, Sma-1-17965, Sma-1-17967, Sma-1-17969, Sma-1-1797, Sma-1-17971, Sma-1-17973, Sma-1-17975, Sma-1-17977, Sma-1-17979, Sma-1-17981, Sma-1-17983, Sma-1-17985, Sma-1-17987, Sma-1-17989, Sma-1-1799, Sma-1-17991, Sma-1-17993, Sma-1-17995, Sma-1-17997, Sma-1-17999, Sma-1-18, Sma-1-180, Sma-1-18001, Sma-1-18003, Sma-1-18005, Sma-1-18007, Sma-1-18009, Sma-1-1801, Sma-1-18011, Sma-1-18013, Sma-1-18015, Sma-1-18017, Sma-1-18019, Sma-1-1802, Sma-1-18020, Sma-1-18021, Sma-1-18023, Sma-1-18025, Sma-1-18027, Sma-1-18029, Sma-1-1803, Sma-1-18031, Sma-1-18033, Sma-1-18035, Sma-1-18037, Sma-1-18039, Sma-1-18040, Sma-1-18041, Sma-1-18043, Sma-1-18044, Sma-1-18045, Sma-1-18047, Sma-1-18049, Sma-1-1805, Sma-1-18051, Sma-1-18053, Sma-1-18055, Sma-1-18057, Sma-1-18059, Sma-1-1806, Sma-1-18061, Sma-1-18063, Sma-1-18065, Sma-1-18067, Sma-1-18069, Sma-1-1807, Sma-1-18071, Sma-1-18072, Sma-1-18073, Sma-1-18075, Sma-1-18077, Sma-1-18079, Sma-1-18081, Sma-1-18083, Sma-1-18085, Sma-1-18087, Sma-1-18089, Sma-1-1809, Sma-1-18091, Sma-1-18093, Sma-1-18095, Sma-1-18097, Sma-1-18099, Sma-1-18100, Sma-1-18101, Sma-1-18103, Sma-1-18105, Sma-1-18107, Sma-1-18109, Sma-1-1811, Sma-1-18111, Sma-1-18113, Sma-1-18115, Sma-1-18117, Sma-1-18119, Sma-1-18121, Sma-1-18122, Sma-1-18123, Sma-1-18125, Sma-1-18127, Sma-1-18129, Sma-1-1813, Sma-1-18131, Sma-1-18133, Sma-1-18135, Sma-1-18137, Sma-1-18139, Sma-1-18141, Sma-1-18143, Sma-1-18145, Sma-1-18147, Sma-1-18149, Sma-1-1815, Sma-1-18151, Sma-1-18153, Sma-1-18155, Sma-1-18157, Sma-1-18159, Sma-1-18161, Sma-1-18163, Sma-1-18165, Sma-1-18167, Sma-1-18169, Sma-1-1817, Sma-1-18171, Sma-1-18173, Sma-1-18175, Sma-1-18176, Sma-1-18177, Sma-1-18178, Sma-1-18179, Sma-1-18180, Sma-1-18181, Sma-1-18183, Sma-1-18184, Sma-1-18185, Sma-1-18186, Sma-1-18187, Sma-1-18189, Sma-1-1819, Sma-1-18191, Sma-1-18193, Sma-1-18195, Sma-1-18197, Sma-1-18199, Sma-1-182, Sma-1-1820, Sma-1-18201, Sma-1-18203, Sma-1-18205, Sma-1-18207, Sma-1-18209, Sma-1-1821, Sma-1-18211, Sma-1-18213, Sma-1-18215, Sma-1-18216, Sma-1-18217, Sma-1-18219, Sma-1-18221, Sma-1-18222, Sma-1-18223, Sma-1-18224, Sma-1-18225, Sma-1-18226, Sma-1-18227, Sma-1-18229, Sma-1-1823, Sma-1-18230, Sma-1-18231, Sma-1-18232, Sma-1-18233, Sma-1-18234, Sma-1-18235, Sma-1-18236, Sma-1-18237, Sma-1-18239, Sma-1-18240, Sma-1-18241, Sma-1-18243, Sma-1-18245, Sma-1-18247, Sma-1-18248, Sma-1-18249, Sma-1-1825, Sma-1-18250, Sma-1-18251, Sma-1-18252, Sma-1-18253, Sma-1-18254, Sma-1-18255, Sma-1-18256, Sma-1-18257, Sma-1-18258, Sma-1-18259, Sma-1-18260, Sma-1-18261, Sma-1-18262, Sma-1-18263, Sma-1-18264, Sma-1-18265, Sma-1-18266, Sma-1-18267, Sma-1-18268, Sma-1-18269, Sma-1-1827, Sma-1-18270, Sma-1-18271, Sma-1-18272, Sma-1-18273, Sma-1-18274, Sma-1-18275, Sma-1-18276, Sma-1-18277, Sma-1-18278, Sma-1-18279, Sma-1-1828, Sma-1-18280, Sma-1-18281, Sma-1-18282, Sma-1-18283, Sma-1-18284, Sma-1-18285, Sma-1-18286, Sma-1-18287, Sma-1-18288, Sma-1-18289, Sma-1-1829, Sma-1-18290, Sma-1-18291, Sma-1-18292, Sma-1-18293, Sma-1-18294, Sma-1-18295, Sma-1-18296, Sma-1-18297, Sma-1-18298, Sma-1-18299, Sma-1-18300, Sma-1-18301, Sma-1-18302, Sma-1-18303, Sma-1-18304, Sma-1-18305, Sma-1-18306, Sma-1-18307, Sma-1-18308, Sma-1-18309, Sma-1-1831, Sma-1-18310, Sma-1-18311, Sma-1-18312, Sma-1-18313, Sma-1-18314, Sma-1-18315, Sma-1-18316, Sma-1-18317, Sma-1-18318, Sma-1-18319, Sma-1-18320, Sma-1-18321, Sma-1-18322, Sma-1-18323, Sma-1-18324, Sma-1-18325, Sma-1-18326, Sma-1-18327, Sma-1-18328, Sma-1-18329, Sma-1-1833, Sma-1-18330, Sma-1-18331, Sma-1-18332, Sma-1-18333, Sma-1-18334, Sma-1-18335, Sma-1-18336, Sma-1-18337, Sma-1-18338, Sma-1-18339, Sma-1-1834, Sma-1-18340, Sma-1-18341, Sma-1-18342, Sma-1-18343, Sma-1-18345, Sma-1-18347, Sma-1-18348, Sma-1-18349, Sma-1-1835, Sma-1-18351, Sma-1-18353, Sma-1-18355, Sma-1-18357, Sma-1-18359, Sma-1-18361, Sma-1-18363, Sma-1-18364, Sma-1-18365, Sma-1-18367, Sma-1-18369, Sma-1-1837, Sma-1-18371, Sma-1-18373, Sma-1-18375, Sma-1-18377, Sma-1-18379, Sma-1-18380, Sma-1-18381, Sma-1-18383, Sma-1-18385, Sma-1-18387, Sma-1-18389, Sma-1-1839, Sma-1-18390, Sma-1-18391, Sma-1-18392, Sma-1-18393, Sma-1-18394, Sma-1-18395, Sma-1-18397, Sma-1-18399, Sma-1-184, Sma-1-18401, Sma-1-18403, Sma-1-18405, Sma-1-18407, Sma-1-18408, Sma-1-18409, Sma-1-1841, Sma-1-18411, Sma-1-18413, Sma-1-18415, Sma-1-18417, Sma-1-18419, Sma-1-18421, Sma-1-18423, Sma-1-18424, Sma-1-18425, Sma-1-18427, Sma-1-18429, Sma-1-1843, Sma-1-18431, Sma-1-18432, Sma-1-18433, Sma-1-18435, Sma-1-18437, Sma-1-18439, Sma-1-18441, Sma-1-18442, Sma-1-18443, Sma-1-18445, Sma-1-18447, Sma-1-18449, Sma-1-1845, Sma-1-18451, Sma-1-18453, Sma-1-18454, Sma-1-18455, Sma-1-18456, Sma-1-18457, Sma-1-18459, Sma-1-18461, Sma-1-18463, Sma-1-18465, Sma-1-18466, Sma-1-18467, Sma-1-18469, Sma-1-1847, Sma-1-18471, Sma-1-18473, Sma-1-18475, Sma-1-18477, Sma-1-18479, Sma-1-18480,

Sma-1-18481, Sma-1-18482, Sma-1-18483, Sma-1-18485, Sma-1-18486, Sma-1-18487, Sma-1-18489, Sma-1-1849, Sma-1-18491, Sma-1-18493, Sma-1-18495, Sma-1-18497, Sma-1-18499, Sma-1-18500, Sma-1-18501, Sma-1-18502, Sma-1-18503, Sma-1-18505, Sma-1-18507, Sma-1-18509, Sma-1-1851, Sma-1-18510, Sma-1-18511, Sma-1-18512, Sma-1-18513, Sma-1-18514, Sma-1-18515, Sma-1-18516, Sma-1-18517, Sma-1-18518, Sma-1-18519, Sma-1-18520, Sma-1-18521, Sma-1-18522, Sma-1-18523, Sma-1-18524, Sma-1-18525, Sma-1-18526, Sma-1-18527, Sma-1-18528, Sma-1-18529, Sma-1-1853, Sma-1-18530, Sma-1-18531, Sma-1-18532, Sma-1-18533, Sma-1-18534, Sma-1-18535, Sma-1-18536, Sma-1-18537, Sma-1-18538, Sma-1-18539, Sma-1-18540, Sma-1-18541, Sma-1-18542, Sma-1-18543, Sma-1-18544, Sma-1-18545, Sma-1-18546, Sma-1-18547, Sma-1-18548, Sma-1-18549, Sma-1-1855, Sma-1-18550, Sma-1-18551, Sma-1-18552, Sma-1-18553, Sma-1-18555, Sma-1-18557, Sma-1-18559, Sma-1-18561, Sma-1-18563, Sma-1-18565, Sma-1-18567, Sma-1-18569, Sma-1-1857, Sma-1-18570, Sma-1-18571, Sma-1-18572, Sma-1-18573, Sma-1-18574, Sma-1-18575, Sma-1-18577, Sma-1-18579, Sma-1-1858, Sma-1-18581, Sma-1-18583, Sma-1-18585, Sma-1-18587, Sma-1-18589, Sma-1-1859, Sma-1-18591, Sma-1-18593, Sma-1-18595, Sma-1-18597, Sma-1-18599, Sma-1-186, Sma-1-18601, Sma-1-18602, Sma-1-18603, Sma-1-18605, Sma-1-18607, Sma-1-18609, Sma-1-1861, Sma-1-18611, Sma-1-18612, Sma-1-18613, Sma-1-18615, Sma-1-18617, Sma-1-18619, Sma-1-18620, Sma-1-18621, Sma-1-18623, Sma-1-18625, Sma-1-18627, Sma-1-18629, Sma-1-1863, Sma-1-18631, Sma-1-18633, Sma-1-18635, Sma-1-18637, Sma-1-18639, Sma-1-18641, Sma-1-18643, Sma-1-18645, Sma-1-18647, Sma-1-18649, Sma-1-1865, Sma-1-18651, Sma-1-18653, Sma-1-18655, Sma-1-18657, Sma-1-18659, Sma-1-1866, Sma-1-18661, Sma-1-18663, Sma-1-18665, Sma-1-18667, Sma-1-18669, Sma-1-1867, Sma-1-18671, Sma-1-18673, Sma-1-18675, Sma-1-18677, Sma-1-18679, Sma-1-1868, Sma-1-18681, Sma-1-18683, Sma-1-18685, Sma-1-18687, Sma-1-18689, Sma-1-1869, Sma-1-18691, Sma-1-18693, Sma-1-18695, Sma-1-18697, Sma-1-18699, Sma-1-18701, Sma-1-18703, Sma-1-18704, Sma-1-18705, Sma-1-18707, Sma-1-18709, Sma-1-1871, Sma-1-18711, Sma-1-18713, Sma-1-18715, Sma-1-18717, Sma-1-18719, Sma-1-18721, Sma-1-18723, Sma-1-18725, Sma-1-18727, Sma-1-18729, Sma-1-1873, Sma-1-18731, Sma-1-18733, Sma-1-18735, Sma-1-18736, Sma-1-18737, Sma-1-18739, Sma-1-18741, Sma-1-18743, Sma-1-18745, Sma-1-18747, Sma-1-18749, Sma-1-1875, Sma-1-18751, Sma-1-18753, Sma-1-18755, Sma-1-18757, Sma-1-18759, Sma-1-18761, Sma-1-18763, Sma-1-18765, Sma-1-18767, Sma-1-18769, Sma-1-1877, Sma-1-18771, Sma-1-18773, Sma-1-18775, Sma-1-18777, Sma-1-18779, Sma-1-18781, Sma-1-18783, Sma-1-18785, Sma-1-18786, Sma-1-18787, Sma-1-18789, Sma-1-1879, Sma-1-18791, Sma-1-18793, Sma-1-18795, Sma-1-18797, Sma-1-18799, Sma-1-188, Sma-1-18801, Sma-1-18802, Sma-1-18803, Sma-1-18805, Sma-1-18807, Sma-1-18809, Sma-1-1881, Sma-1-18811, Sma-1-18813, Sma-1-18815, Sma-1-18817, Sma-1-18819, Sma-1-1882, Sma-1-18821, Sma-1-18823, Sma-1-18825, Sma-1-18827, Sma-1-18829, Sma-1-1883, Sma-1-18831, Sma-1-18833, Sma-1-18835, Sma-1-18837, Sma-1-18839, Sma-1-18841, Sma-1-18842, Sma-1-18843, Sma-1-18845, Sma-1-18847, Sma-1-18849, Sma-1-1885, Sma-1-18851, Sma-1-18853, Sma-1-18855, Sma-1-18857, Sma-1-18859, Sma-1-1886, Sma-1-18861, Sma-1-18863, Sma-1-18864, Sma-1-18865, Sma-1-18867, Sma-1-18868, Sma-1-18869, Sma-1-1887, Sma-1-18871, Sma-1-18873, Sma-1-18875, Sma-1-18877, Sma-1-18879, Sma-1-18881, Sma-1-18883, Sma-1-18885, Sma-1-18887, Sma-1-18888, Sma-1-18889, Sma-1-1889, Sma-1-18890, Sma-1-18891, Sma-1-18892, Sma-1-18893, Sma-1-18895, Sma-1-18896, Sma-1-18897, Sma-1-18899, Sma-1-18901, Sma-1-18903, Sma-1-18905, Sma-1-18907, Sma-1-18909, Sma-1-1891, Sma-1-18911, Sma-1-18913, Sma-1-18915, Sma-1-18917, Sma-1-18919, Sma-1-18921, Sma-1-18923, Sma-1-18925, Sma-1-18927, Sma-1-18929, Sma-1-1893, Sma-1-18931, Sma-1-18933, Sma-1-18935, Sma-1-18937, Sma-1-18939, Sma-1-18941, Sma-1-18943, Sma-1-18945, Sma-1-18947, Sma-1-18949, Sma-1-1895, Sma-1-18951, Sma-1-18953, Sma-1-18955, Sma-1-18956, Sma-1-18957, Sma-1-18959, Sma-1-1896, Sma-1-18961, Sma-1-18963, Sma-1-18965, Sma-1-18967, Sma-1-18969, Sma-1-1897, Sma-1-18971, Sma-1-18973, Sma-1-18975, Sma-1-18977, Sma-1-18979, Sma-1-18981, Sma-1-18983, Sma-1-18985, Sma-1-18987, Sma-1-18989, Sma-1-1899, Sma-1-18991, Sma-1-18993, Sma-1-18995, Sma-1-18997, Sma-1-18999, Sma-1-19, Sma-1-190, Sma-1-1900, Sma-1-19001, Sma-1-19003, Sma-1-19005, Sma-1-19007, Sma-1-19009, Sma-1-1901, Sma-1-19011, Sma-1-19013, Sma-1-19015, Sma-1-19017, Sma-1-19019, Sma-1-1902, Sma-1-19021, Sma-1-19023, Sma-1-19025, Sma-1-19027, Sma-1-19029, Sma-1-1903, Sma-1-19031, Sma-1-19033, Sma-1-19035, Sma-1-19037, Sma-1-19039, Sma-1-1904, Sma-1-19041, Sma-1-19043, Sma-1-19045, Sma-1-19047, Sma-1-19049, Sma-1-1905, Sma-1-19051, Sma-1-19053, Sma-1-19055, Sma-1-19057, Sma-1-19059, Sma-1-1906, Sma-1-19061, Sma-1-19063, Sma-1-19065, Sma-1-19067, Sma-1-19069, Sma-1-1907, Sma-1-19071, Sma-1-19073, Sma-1-19075, Sma-1-19077, Sma-1-19079, Sma-1-1908, Sma-1-19080, Sma-1-19081, Sma-1-19082, Sma-1-19083, Sma-1-19085, Sma-1-19087, Sma-1-19089, Sma-1-1909, Sma-1-19091, Sma-1-19093, Sma-1-19095, Sma-1-19097, Sma-1-19099, Sma-1-1910, Sma-1-19101, Sma-1-19103, Sma-1-19105, Sma-1-19107, Sma-1-19109, Sma-1-1911, Sma-1-19111, Sma-1-19113, Sma-1-19115, Sma-1-19117, Sma-1-19119, Sma-1-1912, Sma-1-19121, Sma-1-19123, Sma-1-19125, Sma-1-19127, Sma-1-19129, Sma-1-1913, Sma-1-19131, Sma-1-19133, Sma-1-19135, Sma-1-19137, Sma-1-19139, Sma-1-1914, Sma-1-19141, Sma-1-19143, Sma-1-19145, Sma-1-19147, Sma-1-19149, Sma-1-1915, Sma-1-19151, Sma-1-19153, Sma-1-19155, Sma-1-19157, Sma-1-19159, Sma-1-1916, Sma-1-19161, Sma-1-19163, Sma-1-19165, Sma-1-19167, Sma-1-19169, Sma-1-1917, Sma-1-19171, Sma-1-19173, Sma-1-19175, Sma-1-19177, Sma-1-19179, Sma-1-1918, Sma-1-19181, Sma-1-19183, Sma-1-19185, Sma-1-19187, Sma-1-19189, Sma-1-1919, Sma-1-19191, Sma-1-19193, Sma-1-19195, Sma-1-19197, Sma-1-19199, Sma-1-192, Sma-1-1920, Sma-1-19201, Sma-1-19203, Sma-1-19205, Sma-1-19207, Sma-1-19209, Sma-1-1921, Sma-1-19211, Sma-1-19213, Sma-1-19215, Sma-1-19217, Sma-1-19219, Sma-1-1922, Sma-1-19221, Sma-1-19223, Sma-1-19225, Sma-1-19226, Sma-1-19227, Sma-1-19229, Sma-1-1923, Sma-1-19231, Sma-1-19233, Sma-1-19235, Sma-1-19237, Sma-1-19239, Sma-1-1924, Sma-1-19241, Sma-1-19243, Sma-1-19245, Sma-1-19247, Sma-1-19249, Sma-1-1925, Sma-1-19251, Sma-1-19253, Sma-1-19255, Sma-1-19257, Sma-1-19259, Sma-1-1926, Sma-1-19261, Sma-1-19262, Sma-1-19263, Sma-1-19265, Sma-1-19267, Sma-1-19269, Sma-1-1927, Sma-1-19271, Sma-1-19273, Sma-1-19275, Sma-1-19277, Sma-1-19279, Sma-1-1928, Sma-1-19281, Sma-1-19283, Sma-1-19285, Sma-1-19287, Sma-1-19289, Sma-1-1929, Sma-1-19291, Sma-1-19293, Sma-1-19295, Sma-1-19297, Sma-1-19299, Sma-1-1930, Sma-1-19301, Sma-1-19303, Sma-1-19305, Sma-1-19307, Sma-1-19309, Sma-1-1931, Sma-1-19311, Sma-1-19313, Sma-1-19315, Sma-1-19317, Sma-1-19319, Sma-1-19321,

Sma-1-19323, Sma-1-19325, Sma-1-19327, Sma-1-19329, Sma-1-1933, Sma-1-19331, Sma-1-19332, Sma-1-19333, Sma-1-19335, Sma-1-19337, Sma-1-19339, Sma-1-19341, Sma-1-19343, Sma-1-19345, Sma-1-19347, Sma-1-19349, Sma-1-1935, Sma-1-19351, Sma-1-19353, Sma-1-19355, Sma-1-19357, Sma-1-19359, Sma-1-19361, Sma-1-19362, Sma-1-19363, Sma-1-19365, Sma-1-19367, Sma-1-19369, Sma-1-1937, Sma-1-19371, Sma-1-19373, Sma-1-19375, Sma-1-19377, Sma-1-19378, Sma-1-19379, Sma-1-19381, Sma-1-19383, Sma-1-19385, Sma-1-19387, Sma-1-19389, Sma-1-1939, Sma-1-19391, Sma-1-19393, Sma-1-19394, Sma-1-19395, Sma-1-19397, Sma-1-19399, Sma-1-194, Sma-1-19401, Sma-1-19403, Sma-1-19405, Sma-1-19407, Sma-1-19409, Sma-1-1941, Sma-1-19411, Sma-1-19412, Sma-1-19413, Sma-1-19415, Sma-1-19417, Sma-1-19419, Sma-1-19421, Sma-1-19423, Sma-1-19425, Sma-1-19427, Sma-1-19429, Sma-1-1943, Sma-1-19431, Sma-1-19433, Sma-1-19435, Sma-1-19437, Sma-1-19439, Sma-1-19441, Sma-1-19443, Sma-1-19445, Sma-1-19447, Sma-1-19449, Sma-1-1945, Sma-1-19451, Sma-1-19453, Sma-1-19455, Sma-1-19457, Sma-1-19459, Sma-1-19461, Sma-1-19463, Sma-1-19465, Sma-1-19467, Sma-1-19469, Sma-1-1947, Sma-1-19471, Sma-1-19473, Sma-1-19475, Sma-1-19476, Sma-1-19477, Sma-1-19479, Sma-1-19481, Sma-1-19483, Sma-1-19485, Sma-1-19487, Sma-1-19488, Sma-1-19489, Sma-1-1949, Sma-1-19490, Sma-1-19491, Sma-1-19493, Sma-1-19495, Sma-1-19496, Sma-1-19497, Sma-1-19499, Sma-1-19501, Sma-1-19503, Sma-1-19505, Sma-1-19507, Sma-1-19509, Sma-1-1951, Sma-1-19511, Sma-1-19513, Sma-1-19515, Sma-1-19517, Sma-1-19519, Sma-1-19521, Sma-1-19523, Sma-1-19525, Sma-1-19527, Sma-1-19529, Sma-1-1953, Sma-1-19531, Sma-1-19532, Sma-1-19533, Sma-1-19535, Sma-1-19537, Sma-1-19539, Sma-1-19541, Sma-1-19543, Sma-1-19545, Sma-1-19547, Sma-1-19549, Sma-1-1955, Sma-1-19550, Sma-1-19551, Sma-1-19553, Sma-1-19555, Sma-1-19557, Sma-1-19559, Sma-1-19561, Sma-1-19563, Sma-1-19565, Sma-1-19566, Sma-1-19567, Sma-1-19569, Sma-1-1957, Sma-1-19571, Sma-1-19573, Sma-1-19575, Sma-1-19577, Sma-1-19579, Sma-1-1958, Sma-1-19581, Sma-1-19583, Sma-1-19585, Sma-1-19587, Sma-1-19589, Sma-1-1959, Sma-1-19591, Sma-1-19593, Sma-1-19595, Sma-1-19597, Sma-1-19599, Sma-1-196, Sma-1-19601, Sma-1-19603, Sma-1-19605, Sma-1-19607, Sma-1-19609, Sma-1-1961, Sma-1-19611, Sma-1-19613, Sma-1-19615, Sma-1-19616, Sma-1-19617, Sma-1-19619, Sma-1-19621, Sma-1-19623, Sma-1-19625, Sma-1-19627, Sma-1-19629, Sma-1-1963, Sma-1-19631, Sma-1-19633, Sma-1-19635, Sma-1-19637, Sma-1-19639, Sma-1-19641, Sma-1-19643, Sma-1-19645, Sma-1-19647, Sma-1-19649, Sma-1-1965, Sma-1-19651, Sma-1-19653, Sma-1-19655, Sma-1-19657, Sma-1-19659, Sma-1-1966, Sma-1-19661, Sma-1-19663, Sma-1-19665, Sma-1-19667, Sma-1-19669, Sma-1-1967, Sma-1-19671, Sma-1-19673, Sma-1-19675, Sma-1-19677, Sma-1-19679, Sma-1-1968, Sma-1-19681, Sma-1-19683, Sma-1-19685, Sma-1-19687, Sma-1-19689, Sma-1-1969, Sma-1-19691, Sma-1-19693, Sma-1-19695, Sma-1-19697, Sma-1-19698, Sma-1-19699, Sma-1-1970, Sma-1-19702, Sma-1-19704, Sma-1-19706, Sma-1-19708, Sma-1-19709, Sma-1-1971, Sma-1-19710, Sma-1-19712, Sma-1-19714, Sma-1-19716, Sma-1-19718, Sma-1-1972, Sma-1-19720, Sma-1-19722, Sma-1-19724, Sma-1-19726, Sma-1-19728, Sma-1-1973, Sma-1-19730, Sma-1-19732, Sma-1-19734, Sma-1-19736, Sma-1-19738, Sma-1-1974, Sma-1-19740, Sma-1-19742, Sma-1-19744, Sma-1-19746, Sma-1-19748, Sma-1-1975, Sma-1-19750, Sma-1-19752, Sma-1-19754, Sma-1-19756, Sma-1-19758, Sma-1-1976, Sma-1-19760, Sma-1-19762, Sma-1-19764, Sma-1-19766, Sma-1-19768, Sma-1-1977, Sma-1-19770, Sma-1-19772, Sma-1-19774, Sma-1-19776, Sma-1-19778, Sma-1-1978, Sma-1-19780, Sma-1-19782, Sma-1-19784, Sma-1-19786, Sma-1-19788, Sma-1-1979, Sma-1-19790, Sma-1-19792, Sma-1-19794, Sma-1-19796, Sma-1-19798, Sma-1-198, Sma-1-1980, Sma-1-19800, Sma-1-19802, Sma-1-19804, Sma-1-19806, Sma-1-19808, Sma-1-1981, Sma-1-19810, Sma-1-19812, Sma-1-19814, Sma-1-19816, Sma-1-19818, Sma-1-1982, Sma-1-19820, Sma-1-19822, Sma-1-19824, Sma-1-19825, Sma-1-19826, Sma-1-19828, Sma-1-1983, Sma-1-19830, Sma-1-19832, Sma-1-19834, Sma-1-19836, Sma-1-19838, Sma-1-1984, Sma-1-19840, Sma-1-19842, Sma-1-19844, Sma-1-19846, Sma-1-19848, Sma-1-1985, Sma-1-19850, Sma-1-19852, Sma-1-19854, Sma-1-19856, Sma-1-19857, Sma-1-19858, Sma-1-1986, Sma-1-19860, Sma-1-19862, Sma-1-19864, Sma-1-19866, Sma-1-19868, Sma-1-1987, Sma-1-19870, Sma-1-19872, Sma-1-19874, Sma-1-19876, Sma-1-19878, Sma-1-1988, Sma-1-19880, Sma-1-19882, Sma-1-19884, Sma-1-19886, Sma-1-19888, Sma-1-1989, Sma-1-19890, Sma-1-19892, Sma-1-19894, Sma-1-19896, Sma-1-19898, Sma-1-19899, Sma-1-1990, Sma-1-19900, Sma-1-19902, Sma-1-19904, Sma-1-19906, Sma-1-19908, Sma-1-1991, Sma-1-19910, Sma-1-19912, Sma-1-19914, Sma-1-19915, Sma-1-19916, Sma-1-19918, Sma-1-1992, Sma-1-19920, Sma-1-19921, Sma-1-19922, Sma-1-19924, Sma-1-19926, Sma-1-19928, Sma-1-1993, Sma-1-19930, Sma-1-19932, Sma-1-19934, Sma-1-19936, Sma-1-19937, Sma-1-19938, Sma-1-1994, Sma-1-19940, Sma-1-19942, Sma-1-19943, Sma-1-19944, Sma-1-19945, Sma-1-19946, Sma-1-19948, Sma-1-1995, Sma-1-19950, Sma-1-19952, Sma-1-19954, Sma-1-19956, Sma-1-19958, Sma-1-1996, Sma-1-19960, Sma-1-19962, Sma-1-19964, Sma-1-19966, Sma-1-19968, Sma-1-1997, Sma-1-19970, Sma-1-19972, Sma-1-19974, Sma-1-19976, Sma-1-19978, Sma-1-1998, Sma-1-19980, Sma-1-19981, Sma-1-19982, Sma-1-19984, Sma-1-19986, Sma-1-19988, Sma-1-1999, Sma-1-19990, Sma-1-19992, Sma-1-19993, Sma-1-19994, Sma-1-19996, Sma-1-19998, Sma-1-2, Sma-1-20, Sma-1-200, Sma-1-2000, Sma-1-20000, Sma-1-20002, Sma-1-20003, Sma-1-20004, Sma-1-20005, Sma-1-20006, Sma-1-20008, Sma-1-2001, Sma-1-20010, Sma-1-20012, Sma-1-20014, Sma-1-20016, Sma-1-20018, Sma-1-2002, Sma-1-20020, Sma-1-20022, Sma-1-20024, Sma-1-20026, Sma-1-20028, Sma-1-2003, Sma-1-20030, Sma-1-20032, Sma-1-20034, Sma-1-20035, Sma-1-20036, Sma-1-20038, Sma-1-20039, Sma-1-2004, Sma-1-20040, Sma-1-20041, Sma-1-20042, Sma-1-20043, Sma-1-20044, Sma-1-20046, Sma-1-20048, Sma-1-2005, Sma-1-20050, Sma-1-20051, Sma-1-20052, Sma-1-20054, Sma-1-20056, Sma-1-20057, Sma-1-20058, Sma-1-20059, Sma-1-2006, Sma-1-20060, Sma-1-20061, Sma-1-20062, Sma-1-20063, Sma-1-20064, Sma-1-20066, Sma-1-20068, Sma-1-2007, Sma-1-20070, Sma-1-20072, Sma-1-20073, Sma-1-20074, Sma-1-20076, Sma-1-20078, Sma-1-2008, Sma-1-20080, Sma-1-20082, Sma-1-20083, Sma-1-20084, Sma-1-20086, Sma-1-20088, Sma-1-2009, Sma-1-20090, Sma-1-20092, Sma-1-20094, Sma-1-20096, Sma-1-20098, Sma-1-201, Sma-1-2010, Sma-1-20100, Sma-1-20102, Sma-1-20104, Sma-1-20106, Sma-1-20108, Sma-1-2011, Sma-1-20110, Sma-1-20112, Sma-1-20114, Sma-1-20116, Sma-1-20118, Sma-1-20119, Sma-1-2012, Sma-1-20120, Sma-1-20122, Sma-1-20123, Sma-1-20124, Sma-1-20125, Sma-1-20126, Sma-1-20128, Sma-1-2013, Sma-1-20130, Sma-1-20132, Sma-1-20134, Sma-1-20136, Sma-1-20138, Sma-1-20139, Sma-1-2014, Sma-1-20140, Sma-1-20141, Sma-1-20142, Sma-1-20143, Sma-1-20144, Sma-1-20145, Sma-1-20146, Sma-1-20148, Sma-1-2015, Sma-1-20150, Sma-1-20152, Sma-1-20154, Sma-1-20155, Sma-1-20156, Sma-1-20158, Sma-1-2016, Sma-1-20160, Sma-1-20161, Sma-1-20162,

Sma-1-20164, Sma-1-20166, Sma-1-20168, Sma-1-20169, Sma-1-2017, Sma-1-20170, Sma-1-20172, Sma-1-20174, Sma-1-20176, Sma-1-20178, Sma-1-20180, Sma-1-20182, Sma-1-20184, Sma-1-20186, Sma-1-20188, Sma-1-2019, Sma-1-20190, Sma-1-20192, Sma-1-20194, Sma-1-20196, Sma-1-20197, Sma-1-20198, Sma-1-202, Sma-1-20200, Sma-1-20202, Sma-1-20204, Sma-1-20206, Sma-1-20207, Sma-1-20208, Sma-1-2021, Sma-1-20210, Sma-1-20212, Sma-1-20214, Sma-1-20216, Sma-1-20218, Sma-1-20220, Sma-1-20221, Sma-1-20222, Sma-1-20224, Sma-1-20226, Sma-1-20228, Sma-1-2023, Sma-1-20230, Sma-1-20232, Sma-1-20233, Sma-1-20234, Sma-1-20235, Sma-1-20236, Sma-1-20237, Sma-1-20238, Sma-1-20239, Sma-1-20240, Sma-1-20241, Sma-1-20242, Sma-1-20243, Sma-1-20244, Sma-1-20245, Sma-1-20246, Sma-1-20247, Sma-1-20248, Sma-1-20249, Sma-1-2025, Sma-1-20250, Sma-1-20251, Sma-1-20252, Sma-1-20253, Sma-1-20254, Sma-1-20255, Sma-1-20256, Sma-1-20257, Sma-1-20258, Sma-1-20259, Sma-1-2026, Sma-1-20260, Sma-1-20261, Sma-1-20262, Sma-1-20263, Sma-1-20264, Sma-1-20265, Sma-1-20266, Sma-1-20267, Sma-1-20268, Sma-1-20269, Sma-1-2027, Sma-1-20270, Sma-1-20271, Sma-1-20272, Sma-1-20273, Sma-1-20274, Sma-1-20275, Sma-1-20276, Sma-1-20278, Sma-1-20279, Sma-1-20280, Sma-1-20281, Sma-1-20282, Sma-1-20283, Sma-1-20284, Sma-1-20286, Sma-1-20287, Sma-1-20288, Sma-1-2029, Sma-1-20290, Sma-1-20291, Sma-1-20292, Sma-1-20294, Sma-1-20296, Sma-1-20298, Sma-1-2030, Sma-1-20300, Sma-1-20302, Sma-1-20304, Sma-1-20306, Sma-1-20308, Sma-1-2031, Sma-1-20310, Sma-1-20312, Sma-1-20314, Sma-1-20316, Sma-1-20317, Sma-1-20318, Sma-1-20320, Sma-1-20322, Sma-1-20324, Sma-1-20326, Sma-1-20328, Sma-1-2033, Sma-1-20330, Sma-1-20332, Sma-1-20334, Sma-1-20336, Sma-1-20338, Sma-1-20340, Sma-1-20342, Sma-1-20344, Sma-1-20346, Sma-1-20348, Sma-1-20349, Sma-1-2035, Sma-1-20350, Sma-1-20351, Sma-1-20352, Sma-1-20354, Sma-1-20355, Sma-1-20356, Sma-1-20358, Sma-1-20360, Sma-1-20362, Sma-1-20363, Sma-1-20364, Sma-1-20366, Sma-1-20368, Sma-1-2037, Sma-1-20370, Sma-1-20372, Sma-1-20374, Sma-1-20376, Sma-1-20378, Sma-1-2038, Sma-1-20380, Sma-1-20382, Sma-1-20384, Sma-1-20386, Sma-1-20388, Sma-1-2039, Sma-1-20390, Sma-1-20391, Sma-1-20392, Sma-1-20394, Sma-1-20396, Sma-1-20397, Sma-1-20398, Sma-1-20399, Sma-1-204, Sma-1-20400, Sma-1-20401, Sma-1-20402, Sma-1-20403, Sma-1-20404, Sma-1-20405, Sma-1-20406, Sma-1-20407, Sma-1-20408, Sma-1-2041, Sma-1-20410, Sma-1-20412, Sma-1-20414, Sma-1-20416, Sma-1-20417, Sma-1-20418, Sma-1-20419, Sma-1-2042, Sma-1-20420, Sma-1-20422, Sma-1-20424, Sma-1-20426, Sma-1-20427, Sma-1-20428, Sma-1-2043, Sma-1-20430, Sma-1-20432, Sma-1-20433, Sma-1-20434, Sma-1-20435, Sma-1-20436, Sma-1-20437, Sma-1-20438, Sma-1-20439, Sma-1-20440, Sma-1-20441, Sma-1-20442, Sma-1-20443, Sma-1-20444, Sma-1-20445, Sma-1-20446, Sma-1-20447, Sma-1-20448, Sma-1-20449, Sma-1-2045, Sma-1-20450, Sma-1-20451, Sma-1-20452, Sma-1-20453, Sma-1-20454, Sma-1-20455, Sma-1-20456, Sma-1-20457, Sma-1-20458, Sma-1-20459, Sma-1-2046, Sma-1-20460, Sma-1-20461, Sma-1-20462, Sma-1-20463, Sma-1-20464, Sma-1-20465, Sma-1-20466, Sma-1-20467, Sma-1-20468, Sma-1-20469, Sma-1-2047, Sma-1-20470, Sma-1-20471, Sma-1-20472, Sma-1-20473, Sma-1-20474, Sma-1-20475, Sma-1-20476, Sma-1-20477, Sma-1-20478, Sma-1-20479, Sma-1-2048, Sma-1-20480, Sma-1-20481, Sma-1-20482, Sma-1-20483, Sma-1-20484, Sma-1-20485, Sma-1-20486, Sma-1-20487, Sma-1-20488, Sma-1-20489, Sma-1-2049, Sma-1-20490, Sma-1-20491, Sma-1-20492, Sma-1-20493, Sma-1-20494, Sma-1-20495, Sma-1-20496, Sma-1-20497, Sma-1-20498, Sma-1-20499, Sma-1-20500, Sma-1-20501, Sma-1-20502, Sma-1-20503, Sma-1-20504, Sma-1-20506, Sma-1-20507, Sma-1-20508, Sma-1-2051, Sma-1-20510, Sma-1-20512, Sma-1-20514, Sma-1-20516, Sma-1-20517, Sma-1-20518, Sma-1-20520, Sma-1-20522, Sma-1-20523, Sma-1-20524, Sma-1-20526, Sma-1-20528, Sma-1-20529, Sma-1-2053, Sma-1-20530, Sma-1-20532, Sma-1-20533, Sma-1-20534, Sma-1-20535, Sma-1-20536, Sma-1-20537, Sma-1-20538, Sma-1-20539, Sma-1-20540, Sma-1-20541, Sma-1-20542, Sma-1-20543, Sma-1-20544, Sma-1-20545, Sma-1-20546, Sma-1-20547, Sma-1-20548, Sma-1-2055, Sma-1-20550, Sma-1-20552, Sma-1-20554, Sma-1-20556, Sma-1-20557, Sma-1-20558, Sma-1-20559, Sma-1-20560, Sma-1-20561, Sma-1-20562, Sma-1-20563, Sma-1-20564, Sma-1-20566, Sma-1-20568, Sma-1-20569, Sma-1-2057, Sma-1-20570, Sma-1-20571, Sma-1-20572, Sma-1-20574, Sma-1-20576, Sma-1-20578, Sma-1-2058, Sma-1-20580, Sma-1-20581, Sma-1-20582, Sma-1-20584, Sma-1-20586, Sma-1-20588, Sma-1-2059, Sma-1-20590, Sma-1-20592, Sma-1-20594, Sma-1-20596, Sma-1-20597, Sma-1-20598, Sma-1-206, Sma-1-20600, Sma-1-20602, Sma-1-20603, Sma-1-20604, Sma-1-20606, Sma-1-20608, Sma-1-20609, Sma-1-2061, Sma-1-20610, Sma-1-20611, Sma-1-20612, Sma-1-20613, Sma-1-20614, Sma-1-20615, Sma-1-20616, Sma-1-20617, Sma-1-20618, Sma-1-20619, Sma-1-20620, Sma-1-20621, Sma-1-20622, Sma-1-20623, Sma-1-20624, Sma-1-20625, Sma-1-20626, Sma-1-20627, Sma-1-20628, Sma-1-20629, Sma-1-2063, Sma-1-20630, Sma-1-20631, Sma-1-20632, Sma-1-20633, Sma-1-20634, Sma-1-20635, Sma-1-20636, Sma-1-20637, Sma-1-20638, Sma-1-20639, Sma-1-20640, Sma-1-20641, Sma-1-20642, Sma-1-20644, Sma-1-20645, Sma-1-20646, Sma-1-20648, Sma-1-20649, Sma-1-2065, Sma-1-20650, Sma-1-20651, Sma-1-20652, Sma-1-20653, Sma-1-20654, Sma-1-20656, Sma-1-20657, Sma-1-20658, Sma-1-20660, Sma-1-20662, Sma-1-20664, Sma-1-20666, Sma-1-20667, Sma-1-20668, Sma-1-20669, Sma-1-2067, Sma-1-20670, Sma-1-20671, Sma-1-20672, Sma-1-20673, Sma-1-20674, Sma-1-20675, Sma-1-20676, Sma-1-20677, Sma-1-20678, Sma-1-20679, Sma-1-20680, Sma-1-20682, Sma-1-20684, Sma-1-20686, Sma-1-20688, Sma-1-2069, Sma-1-20690, Sma-1-20692, Sma-1-20694, Sma-1-20696, Sma-1-20698, Sma-1-20700, Sma-1-20701, Sma-1-20702, Sma-1-20703, Sma-1-20704, Sma-1-20705, Sma-1-20706, Sma-1-20707, Sma-1-20708, Sma-1-20709, Sma-1-2071, Sma-1-20710, Sma-1-20711, Sma-1-20712, Sma-1-20713, Sma-1-20714, Sma-1-20715, Sma-1-20716, Sma-1-20717, Sma-1-20718, Sma-1-20719, Sma-1-20720, Sma-1-20721, Sma-1-20722, Sma-1-20723, Sma-1-20724, Sma-1-20725, Sma-1-20726, Sma-1-20727, Sma-1-20728, Sma-1-20729, Sma-1-2073, Sma-1-20730, Sma-1-20731, Sma-1-20732, Sma-1-20733, Sma-1-20734, Sma-1-20735, Sma-1-20736, Sma-1-20737, Sma-1-20738, Sma-1-20739, Sma-1-20740, Sma-1-20741, Sma-1-20742, Sma-1-20743, Sma-1-20744, Sma-1-20746, Sma-1-20748, Sma-1-20749, Sma-1-2075, Sma-1-20750, Sma-1-20752, Sma-1-20754, Sma-1-20756, Sma-1-20758, Sma-1-20760, Sma-1-20762, Sma-1-20764, Sma-1-20766, Sma-1-20767, Sma-1-20768, Sma-1-20769, Sma-1-2077, Sma-1-20770, Sma-1-20772, Sma-1-20773, Sma-1-20774, Sma-1-20776, Sma-1-20778, Sma-1-20779, Sma-1-2078, Sma-1-20780, Sma-1-20782, Sma-1-20784, Sma-1-20785, Sma-1-20786, Sma-1-20787, Sma-1-20788, Sma-1-2079, Sma-1-20790, Sma-1-20792, Sma-1-20793, Sma-1-20794, Sma-1-20796, Sma-1-20798, Sma-1-208, Sma-1-20800, Sma-1-20802, Sma-1-20804, Sma-1-20806, Sma-1-20808, Sma-1-2081, Sma-1-20810, Sma-1-20812, Sma-1-20814, Sma-1-20815,

Sma-1-20816, Sma-1-20818, Sma-1-20820, Sma-1-20822, Sma-1-20824, Sma-1-20826, Sma-1-20827, Sma-1-20828, Sma-1-2083, Sma-1-20830, Sma-1-20832, Sma-1-20834, Sma-1-20836, Sma-1-20838, Sma-1-20840, Sma-1-20841, Sma-1-20842, Sma-1-20843, Sma-1-20844, Sma-1-20846, Sma-1-20848, Sma-1-2085, Sma-1-20850, Sma-1-20852, Sma-1-20853, Sma-1-20854, Sma-1-20856, Sma-1-20858, Sma-1-20860, Sma-1-20862, Sma-1-20864, Sma-1-20865, Sma-1-20866, Sma-1-20868, Sma-1-2087, Sma-1-20870, Sma-1-20872, Sma-1-20874, Sma-1-20876, Sma-1-20877, Sma-1-20878, Sma-1-20879, Sma-1-2088, Sma-1-20880, Sma-1-20881, Sma-1-20882, Sma-1-20883, Sma-1-20884, Sma-1-20885, Sma-1-20886, Sma-1-20887, Sma-1-20888, Sma-1-2089, Sma-1-20890, Sma-1-20892, Sma-1-20893, Sma-1-20894, Sma-1-20895, Sma-1-20896, Sma-1-20897, Sma-1-20898, Sma-1-20899, Sma-1-20900, Sma-1-20901, Sma-1-20902, Sma-1-20903, Sma-1-20904, Sma-1-20905, Sma-1-20906, Sma-1-20907, Sma-1-20908, Sma-1-20909, Sma-1-2091, Sma-1-20910, Sma-1-20911, Sma-1-20912, Sma-1-20913, Sma-1-20914, Sma-1-20916, Sma-1-20918, Sma-1-20919, Sma-1-20920, Sma-1-20921, Sma-1-20922, Sma-1-20923, Sma-1-20924, Sma-1-20925, Sma-1-20926, Sma-1-20927, Sma-1-20928, Sma-1-20929, Sma-1-2093, Sma-1-20930, Sma-1-20931, Sma-1-20932, Sma-1-20933, Sma-1-20934, Sma-1-20936, Sma-1-20937, Sma-1-20938, Sma-1-20940, Sma-1-20941, Sma-1-20942, Sma-1-20943, Sma-1-20944, Sma-1-20945, Sma-1-20946, Sma-1-20948, Sma-1-2095, Sma-1-20950, Sma-1-20952, Sma-1-20953, Sma-1-20954, Sma-1-20955, Sma-1-20956, Sma-1-20957, Sma-1-20958, Sma-1-20959, Sma-1-2096, Sma-1-20960, Sma-1-20961, Sma-1-20962, Sma-1-20963, Sma-1-20964, Sma-1-20965, Sma-1-20966, Sma-1-20967, Sma-1-20968, Sma-1-20969, Sma-1-2097, Sma-1-20970, Sma-1-20971, Sma-1-20972, Sma-1-20973, Sma-1-20974, Sma-1-20975, Sma-1-20976, Sma-1-20977, Sma-1-20978, Sma-1-20979, Sma-1-2098, Sma-1-20980, Sma-1-20981, Sma-1-20982, Sma-1-20983, Sma-1-20984, Sma-1-20985, Sma-1-20986, Sma-1-20987, Sma-1-20988, Sma-1-20989, Sma-1-2099, Sma-1-20990, Sma-1-20991, Sma-1-20992, Sma-1-20993, Sma-1-20994, Sma-1-20995, Sma-1-20996, Sma-1-20997, Sma-1-20998, Sma-1-20999, Sma-1-210, Sma-1-2100, Sma-1-21000, Sma-1-21001, Sma-1-21002, Sma-1-21003, Sma-1-21004, Sma-1-21005, Sma-1-21006, Sma-1-21007, Sma-1-21008, Sma-1-21009, Sma-1-2101, Sma-1-21010, Sma-1-21011, Sma-1-21012, Sma-1-21013, Sma-1-21014, Sma-1-21015, Sma-1-21016, Sma-1-21017, Sma-1-21018, Sma-1-21019, Sma-1-2102, Sma-1-21020, Sma-1-21021, Sma-1-21022, Sma-1-21023, Sma-1-21024, Sma-1-21025, Sma-1-21026, Sma-1-21027, Sma-1-21028, Sma-1-21029, Sma-1-2103, Sma-1-21030, Sma-1-21031, Sma-1-21032, Sma-1-21033, Sma-1-21034, Sma-1-21035, Sma-1-21036, Sma-1-21037, Sma-1-21038, Sma-1-21039, Sma-1-21040, Sma-1-21041, Sma-1-21042, Sma-1-21043, Sma-1-21044, Sma-1-21045, Sma-1-21046, Sma-1-21047, Sma-1-21048, Sma-1-21049, Sma-1-2105, Sma-1-21050, Sma-1-21051, Sma-1-21052, Sma-1-21053, Sma-1-21054, Sma-1-21055, Sma-1-21056, Sma-1-21057, Sma-1-21058, Sma-1-21059, Sma-1-21060, Sma-1-21061, Sma-1-21062, Sma-1-21063, Sma-1-21064, Sma-1-21065, Sma-1-21066, Sma-1-21067, Sma-1-21068, Sma-1-21069, Sma-1-2107, Sma-1-21070, Sma-1-21071, Sma-1-21072, Sma-1-21073, Sma-1-21074, Sma-1-21075, Sma-1-21076, Sma-1-21077, Sma-1-21078, Sma-1-21079, Sma-1-21080, Sma-1-21081, Sma-1-21082, Sma-1-21083, Sma-1-21084, Sma-1-21085, Sma-1-21086, Sma-1-21087, Sma-1-21088, Sma-1-21089, Sma-1-2109, Sma-1-21090, Sma-1-21091, Sma-1-21092, Sma-1-21093, Sma-1-21094, Sma-1-21095, Sma-1-21096, Sma-1-21097, Sma-1-21098, Sma-1-21099, Sma-1-21100, Sma-1-21101, Sma-1-21102, Sma-1-21103, Sma-1-21104, Sma-1-21105, Sma-1-21106, Sma-1-21107, Sma-1-21108, Sma-1-21109, Sma-1-2111, Sma-1-21110, Sma-1-21111, Sma-1-21112, Sma-1-21113, Sma-1-21114, Sma-1-21115, Sma-1-21116, Sma-1-21117, Sma-1-21118, Sma-1-21119, Sma-1-21120, Sma-1-21121, Sma-1-21122, Sma-1-21123, Sma-1-21124, Sma-1-21125, Sma-1-21126, Sma-1-21127, Sma-1-21128, Sma-1-21129, Sma-1-2113, Sma-1-21130, Sma-1-21131, Sma-1-21132, Sma-1-21133, Sma-1-21134, Sma-1-21135, Sma-1-21136, Sma-1-21137, Sma-1-21138, Sma-1-21139, Sma-1-21140, Sma-1-21141, Sma-1-21142, Sma-1-21143, Sma-1-21144, Sma-1-21145, Sma-1-21146, Sma-1-21147, Sma-1-21148, Sma-1-21149, Sma-1-2115, Sma-1-21150, Sma-1-21151, Sma-1-21152, Sma-1-21153, Sma-1-21154, Sma-1-21155, Sma-1-21156, Sma-1-21157, Sma-1-21158, Sma-1-21159, Sma-1-21160, Sma-1-21161, Sma-1-21162, Sma-1-21163, Sma-1-21164, Sma-1-21165, Sma-1-21166, Sma-1-21167, Sma-1-21168, Sma-1-21169, Sma-1-2117, Sma-1-21170, Sma-1-21171, Sma-1-21172, Sma-1-21173, Sma-1-21174, Sma-1-21175, Sma-1-21176, Sma-1-21177, Sma-1-21178, Sma-1-21179, Sma-1-2118, Sma-1-21180, Sma-1-21181, Sma-1-21182, Sma-1-21183, Sma-1-21184, Sma-1-21185, Sma-1-21186, Sma-1-21187, Sma-1-21188, Sma-1-21189, Sma-1-2119, Sma-1-21190, Sma-1-21191, Sma-1-21192, Sma-1-21193, Sma-1-21194, Sma-1-21195, Sma-1-21196, Sma-1-21197, Sma-1-21198, Sma-1-21199, Sma-1-212, Sma-1-21200, Sma-1-21201, Sma-1-21202, Sma-1-21203, Sma-1-21204, Sma-1-21205, Sma-1-21206, Sma-1-21207, Sma-1-21208, Sma-1-21209, Sma-1-2121, Sma-1-21210, Sma-1-21211, Sma-1-21212, Sma-1-21213, Sma-1-21214, Sma-1-21215, Sma-1-21216, Sma-1-21217, Sma-1-21218, Sma-1-21219, Sma-1-2122, Sma-1-21220, Sma-1-21221, Sma-1-21222, Sma-1-21223, Sma-1-21224, Sma-1-21225, Sma-1-21226, Sma-1-21227, Sma-1-21228, Sma-1-21229, Sma-1-2123, Sma-1-21230, Sma-1-21231, Sma-1-21232, Sma-1-21233, Sma-1-21234, Sma-1-21235, Sma-1-21236, Sma-1-21237, Sma-1-21238, Sma-1-21239, Sma-1-2124, Sma-1-21240, Sma-1-21241, Sma-1-21242, Sma-1-21243, Sma-1-21244, Sma-1-21245, Sma-1-21246, Sma-1-21247, Sma-1-21248, Sma-1-21249, Sma-1-2125, Sma-1-21250, Sma-1-21251, Sma-1-21252, Sma-1-21253, Sma-1-21254, Sma-1-21255, Sma-1-21256, Sma-1-21257, Sma-1-21258, Sma-1-21259, Sma-1-21260, Sma-1-21261, Sma-1-21262, Sma-1-21263, Sma-1-21264, Sma-1-21265, Sma-1-21266, Sma-1-21267, Sma-1-21268, Sma-1-21269, Sma-1-2127, Sma-1-21270, Sma-1-21271, Sma-1-21272, Sma-1-21273, Sma-1-21274, Sma-1-21275, Sma-1-21276, Sma-1-21277, Sma-1-21278, Sma-1-21279, Sma-1-21280, Sma-1-21281, Sma-1-21282, Sma-1-21283, Sma-1-21284, Sma-1-21285, Sma-1-21286, Sma-1-21287, Sma-1-21288, Sma-1-21289, Sma-1-2129, Sma-1-21290, Sma-1-21291, Sma-1-21292, Sma-1-21293, Sma-1-21294, Sma-1-21295, Sma-1-21296, Sma-1-21297, Sma-1-21298, Sma-1-21299, Sma-1-21300, Sma-1-21301, Sma-1-21302, Sma-1-21303, Sma-1-21304, Sma-1-21305, Sma-1-21306, Sma-1-21307, Sma-1-21308, Sma-1-21309, Sma-1-2131, Sma-1-21310, Sma-1-21311, Sma-1-21312, Sma-1-21313, Sma-1-21314, Sma-1-21315, Sma-1-21316, Sma-1-21317, Sma-1-21318, Sma-1-21319, Sma-1-21320, Sma-1-21321, Sma-1-21322, Sma-1-21323, Sma-1-21324, Sma-1-21325, Sma-1-21326, Sma-1-21327, Sma-1-21328, Sma-1-21329, Sma-1-2133, Sma-1-21330, Sma-1-21331, Sma-1-21332, Sma-1-21333, Sma-1-21334, Sma-1-21335, Sma-1-21336, Sma-1-21337, Sma-1-21338, Sma-1-21339, Sma-1-21340, Sma-1-21341, Sma-1-21342, Sma-1-21343,

Sma-1-21344, Sma-1-21345, Sma-1-21346, Sma-1-21347, Sma-1-21348, Sma-1-21349, Sma-1-2135, Sma-1-21350, Sma-1-21351, Sma-1-21352, Sma-1-21353, Sma-1-21354, Sma-1-21355, Sma-1-21356, Sma-1-21357, Sma-1-21358, Sma-1-21359, Sma-1-21360, Sma-1-21361, Sma-1-21362, Sma-1-21363, Sma-1-21364, Sma-1-21365, Sma-1-21366, Sma-1-21367, Sma-1-21368, Sma-1-21369, Sma-1-2137, Sma-1-21370, Sma-1-21371, Sma-1-21372, Sma-1-21373, Sma-1-21374, Sma-1-21375, Sma-1-21376, Sma-1-21377, Sma-1-21378, Sma-1-21379, Sma-1-21380, Sma-1-21381, Sma-1-21382, Sma-1-21383, Sma-1-21384, Sma-1-21385, Sma-1-21386, Sma-1-21387, Sma-1-21388, Sma-1-21389, Sma-1-2139, Sma-1-21390, Sma-1-21391, Sma-1-21392, Sma-1-21393, Sma-1-21394, Sma-1-21395, Sma-1-21396, Sma-1-21397, Sma-1-21398, Sma-1-21399, Sma-1-214, Sma-1-21400, Sma-1-21401, Sma-1-21402, Sma-1-21403, Sma-1-21404, Sma-1-21405, Sma-1-21406, Sma-1-21407, Sma-1-21408, Sma-1-21409, Sma-1-2141, Sma-1-21410, Sma-1-21411, Sma-1-21412, Sma-1-21413, Sma-1-21414, Sma-1-21415, Sma-1-21416, Sma-1-21417, Sma-1-21418, Sma-1-21419, Sma-1-21420, Sma-1-21421, Sma-1-21422, Sma-1-21423, Sma-1-21424, Sma-1-21425, Sma-1-21426, Sma-1-21427, Sma-1-21428, Sma-1-21429, Sma-1-2143, Sma-1-21430, Sma-1-21431, Sma-1-21432, Sma-1-21433, Sma-1-21434, Sma-1-21435, Sma-1-21436, Sma-1-21437, Sma-1-21438, Sma-1-21439, Sma-1-2144, Sma-1-21440, Sma-1-21441, Sma-1-21442, Sma-1-21443, Sma-1-21444, Sma-1-21445, Sma-1-21446, Sma-1-21447, Sma-1-21448, Sma-1-21449, Sma-1-2145, Sma-1-21450, Sma-1-21451, Sma-1-21452, Sma-1-21453, Sma-1-21454, Sma-1-21455, Sma-1-21456, Sma-1-21457, Sma-1-21458, Sma-1-21459, Sma-1-21460, Sma-1-21461, Sma-1-21462, Sma-1-21463, Sma-1-21464, Sma-1-21465, Sma-1-21466, Sma-1-21467, Sma-1-21468, Sma-1-21469, Sma-1-2147, Sma-1-21470, Sma-1-21471, Sma-1-21472, Sma-1-21473, Sma-1-21474, Sma-1-21475, Sma-1-21476, Sma-1-21477, Sma-1-21478, Sma-1-21479, Sma-1-21480, Sma-1-21481, Sma-1-21482, Sma-1-21483, Sma-1-21484, Sma-1-21485, Sma-1-21486, Sma-1-21487, Sma-1-21488, Sma-1-21489, Sma-1-2149, Sma-1-21490, Sma-1-21491, Sma-1-21492, Sma-1-21493, Sma-1-21494, Sma-1-21495, Sma-1-21496, Sma-1-21497, Sma-1-21498, Sma-1-21499, Sma-1-2150, Sma-1-21500, Sma-1-21501, Sma-1-21502, Sma-1-21503, Sma-1-21504, Sma-1-21505, Sma-1-21506, Sma-1-21507, Sma-1-21508, Sma-1-21509, Sma-1-2151, Sma-1-21510, Sma-1-21511, Sma-1-21512, Sma-1-21513, Sma-1-21514, Sma-1-21515, Sma-1-21516, Sma-1-21517, Sma-1-21518, Sma-1-21519, Sma-1-21520, Sma-1-21521, Sma-1-21522, Sma-1-21523, Sma-1-21524, Sma-1-21525, Sma-1-21526, Sma-1-21527, Sma-1-21528, Sma-1-21529, Sma-1-2153, Sma-1-21530, Sma-1-21531, Sma-1-21532, Sma-1-21533, Sma-1-21534, Sma-1-21535, Sma-1-21536, Sma-1-21537, Sma-1-21538, Sma-1-21539, Sma-1-21540, Sma-1-21541, Sma-1-21542, Sma-1-21543, Sma-1-21544, Sma-1-21545, Sma-1-21546, Sma-1-21547, Sma-1-21548, Sma-1-21549, Sma-1-2155, Sma-1-21550, Sma-1-21551, Sma-1-21552, Sma-1-21553, Sma-1-21554, Sma-1-21555, Sma-1-21556, Sma-1-21557, Sma-1-21558, Sma-1-21559, Sma-1-21560, Sma-1-21561, Sma-1-21562, Sma-1-21563, Sma-1-21564, Sma-1-21565, Sma-1-21566, Sma-1-21567, Sma-1-21568, Sma-1-21569, Sma-1-2157, Sma-1-21570, Sma-1-21571, Sma-1-21572, Sma-1-21573, Sma-1-21574, Sma-1-21575, Sma-1-21576, Sma-1-21577, Sma-1-21578, Sma-1-21579, Sma-1-21580, Sma-1-21581, Sma-1-21582, Sma-1-21583, Sma-1-21584, Sma-1-21585, Sma-1-21586, Sma-1-21587, Sma-1-21588, Sma-1-21589, Sma-1-2159, Sma-1-21590, Sma-1-21591, Sma-1-21592, Sma-1-21593, Sma-1-21594, Sma-1-21595, Sma-1-21596, Sma-1-21597, Sma-1-21598, Sma-1-21599, Sma-1-216, Sma-1-21600, Sma-1-21601, Sma-1-21602, Sma-1-21603, Sma-1-21604, Sma-1-21605, Sma-1-21606, Sma-1-21607, Sma-1-21608, Sma-1-21609, Sma-1-2161, Sma-1-21610, Sma-1-21611, Sma-1-21612, Sma-1-21613, Sma-1-21614, Sma-1-21615, Sma-1-21616, Sma-1-21617, Sma-1-21618, Sma-1-21619, Sma-1-21620, Sma-1-21621, Sma-1-21622, Sma-1-21623, Sma-1-21624, Sma-1-21625, Sma-1-21626, Sma-1-21627, Sma-1-21628, Sma-1-21629, Sma-1-2163, Sma-1-21630, Sma-1-21631, Sma-1-21632, Sma-1-21633, Sma-1-21634, Sma-1-21635, Sma-1-21636, Sma-1-21637, Sma-1-21638, Sma-1-21639, Sma-1-2164, Sma-1-21640, Sma-1-21641, Sma-1-21642, Sma-1-21643, Sma-1-21644, Sma-1-21645, Sma-1-21646, Sma-1-21647, Sma-1-21648, Sma-1-21649, Sma-1-2165, Sma-1-21650, Sma-1-21651, Sma-1-21652, Sma-1-21653, Sma-1-21654, Sma-1-21655, Sma-1-21656, Sma-1-21657, Sma-1-21658, Sma-1-21659, Sma-1-2166, Sma-1-21660, Sma-1-21661, Sma-1-21662, Sma-1-21663, Sma-1-21664, Sma-1-21665, Sma-1-21666, Sma-1-21667, Sma-1-21668, Sma-1-21669, Sma-1-2167, Sma-1-21670, Sma-1-21671, Sma-1-21672, Sma-1-21673, Sma-1-21674, Sma-1-21675, Sma-1-21676, Sma-1-21677, Sma-1-21678, Sma-1-21679, Sma-1-21680, Sma-1-21681, Sma-1-21682, Sma-1-21683, Sma-1-21684, Sma-1-21685, Sma-1-21686, Sma-1-21687, Sma-1-21688, Sma-1-21689, Sma-1-2169, Sma-1-21690, Sma-1-21691, Sma-1-21692, Sma-1-21693, Sma-1-21694, Sma-1-21695, Sma-1-21696, Sma-1-21697, Sma-1-21698, Sma-1-21699, Sma-1-2170, Sma-1-21700, Sma-1-21701, Sma-1-21702, Sma-1-21703, Sma-1-21704, Sma-1-21705, Sma-1-21706, Sma-1-21707, Sma-1-21708, Sma-1-21709, Sma-1-2171, Sma-1-21710, Sma-1-21711, Sma-1-21712, Sma-1-21713, Sma-1-21714, Sma-1-21715, Sma-1-21716, Sma-1-21717, Sma-1-21718, Sma-1-21719, Sma-1-2172, Sma-1-21720, Sma-1-21721, Sma-1-21722, Sma-1-21723, Sma-1-21724, Sma-1-21725, Sma-1-21726, Sma-1-21727, Sma-1-21728, Sma-1-21729, Sma-1-2173, Sma-1-21730, Sma-1-21731, Sma-1-21732, Sma-1-21733, Sma-1-21734, Sma-1-21735, Sma-1-21736, Sma-1-21737, Sma-1-21738, Sma-1-21739, Sma-1-21740, Sma-1-21741, Sma-1-21742, Sma-1-21743, Sma-1-21744, Sma-1-21745, Sma-1-21746, Sma-1-21747, Sma-1-21748, Sma-1-21749, Sma-1-2175, Sma-1-21750, Sma-1-21751, Sma-1-21752, Sma-1-21753, Sma-1-21754, Sma-1-21755, Sma-1-21756, Sma-1-21757, Sma-1-21758, Sma-1-21759, Sma-1-21760, Sma-1-21761, Sma-1-21762, Sma-1-21763, Sma-1-21764, Sma-1-21765, Sma-1-21766, Sma-1-21767, Sma-1-21768, Sma-1-21769, Sma-1-2177, Sma-1-21770, Sma-1-21771, Sma-1-21772, Sma-1-21773, Sma-1-21774, Sma-1-21775, Sma-1-21776, Sma-1-21777, Sma-1-21778, Sma-1-21779, Sma-1-21780, Sma-1-21781, Sma-1-21782, Sma-1-21783, Sma-1-21784, Sma-1-21785, Sma-1-21786, Sma-1-21787, Sma-1-21788, Sma-1-21789, Sma-1-2179, Sma-1-21790, Sma-1-21791, Sma-1-21792, Sma-1-21793, Sma-1-21794, Sma-1-21795, Sma-1-21796, Sma-1-21797, Sma-1-21798, Sma-1-21799, Sma-1-218, Sma-1-21800, Sma-1-21801, Sma-1-21802, Sma-1-21803, Sma-1-21804, Sma-1-21805, Sma-1-21806, Sma-1-21807, Sma-1-21808, Sma-1-21809, Sma-1-2181, Sma-1-21810, Sma-1-21811, Sma-1-21812, Sma-1-21813, Sma-1-21814, Sma-1-21815, Sma-1-21816, Sma-1-21817, Sma-1-21818, Sma-1-21819, Sma-1-21820, Sma-1-21821, Sma-1-21822, Sma-1-21823, Sma-1-21824, Sma-1-21825, Sma-1-21826, Sma-1-21827, Sma-1-21828, Sma-1-21829, Sma-1-2183, Sma-1-21830, Sma-1-21831, Sma-1-21832, Sma-1-21833, Sma-1-21834, Sma-1-21835, Sma-1-21836, Sma-1-21837, Sma-1-21838, Sma-1-21839, Sma-1-21840,

Sma-1-21841, Sma-1-21842, Sma-1-21843, Sma-1-21844, Sma-1-21845, Sma-1-21846, Sma-1-21847, Sma-1-21848, Sma-1-21849, Sma-1-2185, Sma-1-21850, Sma-1-21851, Sma-1-21852, Sma-1-21853, Sma-1-21854, Sma-1-21855, Sma-1-21856, Sma-1-21857, Sma-1-21858, Sma-1-21859, Sma-1-21860, Sma-1-21861, Sma-1-21862, Sma-1-21863, Sma-1-21864, Sma-1-21865, Sma-1-21866, Sma-1-21867, Sma-1-21868, Sma-1-21869, Sma-1-2187, Sma-1-21870, Sma-1-21871, Sma-1-21872, Sma-1-21873, Sma-1-21874, Sma-1-21875, Sma-1-21876, Sma-1-21877, Sma-1-21878, Sma-1-21879, Sma-1-2188, Sma-1-21880, Sma-1-21881, Sma-1-21882, Sma-1-21883, Sma-1-21884, Sma-1-21885, Sma-1-21886, Sma-1-21887, Sma-1-21888, Sma-1-21889, Sma-1-2189, Sma-1-21890, Sma-1-21891, Sma-1-21892, Sma-1-21893, Sma-1-21894, Sma-1-21895, Sma-1-21896, Sma-1-21897, Sma-1-21898, Sma-1-21899, Sma-1-21900, Sma-1-21901, Sma-1-21902, Sma-1-21903, Sma-1-21904, Sma-1-21905, Sma-1-21906, Sma-1-21907, Sma-1-21908, Sma-1-21909, Sma-1-2191, Sma-1-21910, Sma-1-21911, Sma-1-21912, Sma-1-21913, Sma-1-21914, Sma-1-21915, Sma-1-21916, Sma-1-21917, Sma-1-21918, Sma-1-21919, Sma-1-21920, Sma-1-21921, Sma-1-21922, Sma-1-21923, Sma-1-21924, Sma-1-21925, Sma-1-21926, Sma-1-21927, Sma-1-21928, Sma-1-21929, Sma-1-2193, Sma-1-21930, Sma-1-21931, Sma-1-21932, Sma-1-21933, Sma-1-21934, Sma-1-21935, Sma-1-21936, Sma-1-21937, Sma-1-21938, Sma-1-21939, Sma-1-21940, Sma-1-21941, Sma-1-21942, Sma-1-21943, Sma-1-21944, Sma-1-21945, Sma-1-21946, Sma-1-21947, Sma-1-21948, Sma-1-21949, Sma-1-2195, Sma-1-21950, Sma-1-21951, Sma-1-21952, Sma-1-21953, Sma-1-21954, Sma-1-21955, Sma-1-21956, Sma-1-21957, Sma-1-21958, Sma-1-21959, Sma-1-21960, Sma-1-21961, Sma-1-21962, Sma-1-21963, Sma-1-21964, Sma-1-21965, Sma-1-21966, Sma-1-21967, Sma-1-21968, Sma-1-21969, Sma-1-2197, Sma-1-21970, Sma-1-21971, Sma-1-21972, Sma-1-21973, Sma-1-21974, Sma-1-21975, Sma-1-21976, Sma-1-21977, Sma-1-21978, Sma-1-21979, Sma-1-21980, Sma-1-21981, Sma-1-21982, Sma-1-21983, Sma-1-21984, Sma-1-21985, Sma-1-21986, Sma-1-21987, Sma-1-21988, Sma-1-21989, Sma-1-2199, Sma-1-21990, Sma-1-21992, Sma-1-21993, Sma-1-21994, Sma-1-21995, Sma-1-21996, Sma-1-21997, Sma-1-21998, Sma-1-21999, Sma-1-22, Sma-1-220, Sma-1-22000, Sma-1-22001, Sma-1-22002, Sma-1-22003, Sma-1-22004, Sma-1-22005, Sma-1-22006, Sma-1-22007, Sma-1-22008, Sma-1-22009, Sma-1-2201, Sma-1-22010, Sma-1-22011, Sma-1-22012, Sma-1-22013, Sma-1-22014, Sma-1-22015, Sma-1-22016, Sma-1-22017, Sma-1-22018, Sma-1-22019, Sma-1-22020, Sma-1-22021, Sma-1-22022, Sma-1-22023, Sma-1-22024, Sma-1-22025, Sma-1-22026, Sma-1-22027, Sma-1-22028, Sma-1-22029, Sma-1-2203, Sma-1-22030, Sma-1-22031, Sma-1-22032, Sma-1-22034, Sma-1-22035, Sma-1-22036, Sma-1-22037, Sma-1-22038, Sma-1-22039, Sma-1-2204, Sma-1-22040, Sma-1-22041, Sma-1-22042, Sma-1-22043, Sma-1-22044, Sma-1-22045, Sma-1-22046, Sma-1-22047, Sma-1-22048, Sma-1-2205, Sma-1-22050, Sma-1-22051, Sma-1-22052, Sma-1-22053, Sma-1-22054, Sma-1-22055, Sma-1-22056, Sma-1-22057, Sma-1-22058, Sma-1-22059, Sma-1-22060, Sma-1-22061, Sma-1-22062, Sma-1-22063, Sma-1-22064, Sma-1-22065, Sma-1-22066, Sma-1-22067, Sma-1-22068, Sma-1-22069, Sma-1-2207, Sma-1-22070, Sma-1-22071, Sma-1-22072, Sma-1-22073, Sma-1-22074, Sma-1-22075, Sma-1-22076, Sma-1-22078, Sma-1-22079, Sma-1-22080, Sma-1-22081, Sma-1-22082, Sma-1-22083, Sma-1-22084, Sma-1-22085, Sma-1-22086, Sma-1-22088, Sma-1-2209, Sma-1-22090, Sma-1-22092, Sma-1-22093, Sma-1-22094, Sma-1-22095, Sma-1-22096, Sma-1-22097, Sma-1-22098, Sma-1-22100, Sma-1-22102, Sma-1-22104, Sma-1-22105, Sma-1-22106, Sma-1-22108, Sma-1-2211, Sma-1-22110, Sma-1-22112, Sma-1-22114, Sma-1-22116, Sma-1-22117, Sma-1-22118, Sma-1-22119, Sma-1-22120, Sma-1-22121, Sma-1-22122, Sma-1-22124, Sma-1-22125, Sma-1-22126, Sma-1-22127, Sma-1-22128, Sma-1-22129, Sma-1-2213, Sma-1-22130, Sma-1-22132, Sma-1-22133, Sma-1-22134, Sma-1-22135, Sma-1-22136, Sma-1-22138, Sma-1-22140, Sma-1-22141, Sma-1-22142, Sma-1-22143, Sma-1-22144, Sma-1-22145, Sma-1-22146, Sma-1-22147, Sma-1-22148, Sma-1-22149, Sma-1-2215, Sma-1-22150, Sma-1-22151, Sma-1-22152, Sma-1-22153, Sma-1-22154, Sma-1-22155, Sma-1-22156, Sma-1-22157, Sma-1-22158, Sma-1-22159, Sma-1-22160, Sma-1-22161, Sma-1-22162, Sma-1-22163, Sma-1-22164, Sma-1-22165, Sma-1-22166, Sma-1-22167, Sma-1-22168, Sma-1-22169, Sma-1-2217, Sma-1-22170, Sma-1-22171, Sma-1-22172, Sma-1-22173, Sma-1-22174, Sma-1-22176, Sma-1-22178, Sma-1-22179, Sma-1-22180, Sma-1-22182, Sma-1-22184, Sma-1-22186, Sma-1-22188, Sma-1-2219, Sma-1-22190, Sma-1-22191, Sma-1-22192, Sma-1-22193, Sma-1-22194, Sma-1-22196, Sma-1-22198, Sma-1-222, Sma-1-22200, Sma-1-22202, Sma-1-22204, Sma-1-22206, Sma-1-22208, Sma-1-2221, Sma-1-22210, Sma-1-22212, Sma-1-22214, Sma-1-22216, Sma-1-22218, Sma-1-22220, Sma-1-22221, Sma-1-22222, Sma-1-22224, Sma-1-22226, Sma-1-22228, Sma-1-2223, Sma-1-22230, Sma-1-22231, Sma-1-22232, Sma-1-22234, Sma-1-22236, Sma-1-22238, Sma-1-22240, Sma-1-22242, Sma-1-22244, Sma-1-22245, Sma-1-22246, Sma-1-22248, Sma-1-2225, Sma-1-22250, Sma-1-22252, Sma-1-22253, Sma-1-22254, Sma-1-22256, Sma-1-22258, Sma-1-22260, Sma-1-22262, Sma-1-22264, Sma-1-22266, Sma-1-22268, Sma-1-22269, Sma-1-2227, Sma-1-22270, Sma-1-22272, Sma-1-22274, Sma-1-22276, Sma-1-22278, Sma-1-22280, Sma-1-22282, Sma-1-22283, Sma-1-22284, Sma-1-22286, Sma-1-22288, Sma-1-2229, Sma-1-22290, Sma-1-22291, Sma-1-22292, Sma-1-22293, Sma-1-22294, Sma-1-22295, Sma-1-22296, Sma-1-22298, Sma-1-22300, Sma-1-22302, Sma-1-22304, Sma-1-22306, Sma-1-22307, Sma-1-22308, Sma-1-2231, Sma-1-22310, Sma-1-22312, Sma-1-22313, Sma-1-22314, Sma-1-22316, Sma-1-22318, Sma-1-22320, Sma-1-22322, Sma-1-22324, Sma-1-22326, Sma-1-22328, Sma-1-22329, Sma-1-2233, Sma-1-22330, Sma-1-22331, Sma-1-22332, Sma-1-22333, Sma-1-22334, Sma-1-22335, Sma-1-22336, Sma-1-22337, Sma-1-22338, Sma-1-22339, Sma-1-22340, Sma-1-22341, Sma-1-22342, Sma-1-22343, Sma-1-22344, Sma-1-22345, Sma-1-22346, Sma-1-22347, Sma-1-22348, Sma-1-2235, Sma-1-22350, Sma-1-22352, Sma-1-22354, Sma-1-22356, Sma-1-22358, Sma-1-22360, Sma-1-22362, Sma-1-22363, Sma-1-22364, Sma-1-22366, Sma-1-22368, Sma-1-2237, Sma-1-22370, Sma-1-22371, Sma-1-22372, Sma-1-22373, Sma-1-22374, Sma-1-22375, Sma-1-22376, Sma-1-22377, Sma-1-22378, Sma-1-22379, Sma-1-22380, Sma-1-22381, Sma-1-22382, Sma-1-22383, Sma-1-22384, Sma-1-22385, Sma-1-22386, Sma-1-22388, Sma-1-2239, Sma-1-22390, Sma-1-22392, Sma-1-22394, Sma-1-22396, Sma-1-22398, Sma-1-22399, Sma-1-224, Sma-1-22400, Sma-1-22401, Sma-1-22402, Sma-1-22403, Sma-1-22404, Sma-1-22405, Sma-1-22406, Sma-1-22407, Sma-1-22408, Sma-1-2241, Sma-1-22410, Sma-1-22412, Sma-1-22414, Sma-1-22416, Sma-1-22418, Sma-1-22420, Sma-1-22421, Sma-1-22422, Sma-1-22423, Sma-1-22424, Sma-1-22425, Sma-1-22426, Sma-1-22428, Sma-1-2243, Sma-1-22430, Sma-1-22432, Sma-1-22434, Sma-1-22436, Sma-1-22437, Sma-1-22438, Sma-1-22440, Sma-1-22442, Sma-1-22444, Sma-1-22446, Sma-1-22448, Sma-1-2245,

Sma-1-22450, Sma-1-22452, Sma-1-22454, Sma-1-22456, Sma-1-22458, Sma-1-22460, Sma-1-22462, Sma-1-22464, Sma-1-22466, Sma-1-22468, Sma-1-2247, Sma-1-22470, Sma-1-22471, Sma-1-22472, Sma-1-22474, Sma-1-22476, Sma-1-22478, Sma-1-2248, Sma-1-22480, Sma-1-22481, Sma-1-22482, Sma-1-22484, Sma-1-22485, Sma-1-22486, Sma-1-22488, Sma-1-22489, Sma-1-2249, Sma-1-22490, Sma-1-22492, Sma-1-22493, Sma-1-22494, Sma-1-22496, Sma-1-22498, Sma-1-22500, Sma-1-22502, Sma-1-22504, Sma-1-22506, Sma-1-22508, Sma-1-2251, Sma-1-22510, Sma-1-22512, Sma-1-22514, Sma-1-22515, Sma-1-22516, Sma-1-22518, Sma-1-22519, Sma-1-22520, Sma-1-22521, Sma-1-22522, Sma-1-22524, Sma-1-22526, Sma-1-22528, Sma-1-2253, Sma-1-22530, Sma-1-22532, Sma-1-22534, Sma-1-22536, Sma-1-22538, Sma-1-22540, Sma-1-22542, Sma-1-22544, Sma-1-22546, Sma-1-22548, Sma-1-2255, Sma-1-22550, Sma-1-22551, Sma-1-22552, Sma-1-22554, Sma-1-22556, Sma-1-22557, Sma-1-22558, Sma-1-22560, Sma-1-22562, Sma-1-22563, Sma-1-22564, Sma-1-22565, Sma-1-22566, Sma-1-22567, Sma-1-22568, Sma-1-22569, Sma-1-2257, Sma-1-22570, Sma-1-22571, Sma-1-22572, Sma-1-22573, Sma-1-22574, Sma-1-22575, Sma-1-22576, Sma-1-22577, Sma-1-22578, Sma-1-22579, Sma-1-2258, Sma-1-22580, Sma-1-22581, Sma-1-22582, Sma-1-22583, Sma-1-22584, Sma-1-22585, Sma-1-22586, Sma-1-22587, Sma-1-22588, Sma-1-22589, Sma-1-2259, Sma-1-22590, Sma-1-22591, Sma-1-22592, Sma-1-22594, Sma-1-22596, Sma-1-22598, Sma-1-22599, Sma-1-226, Sma-1-22600, Sma-1-22602, Sma-1-22604, Sma-1-22606, Sma-1-22608, Sma-1-2261, Sma-1-22610, Sma-1-22612, Sma-1-22614, Sma-1-22616, Sma-1-22618, Sma-1-22620, Sma-1-22622, Sma-1-22624, Sma-1-22626, Sma-1-22628, Sma-1-2263, Sma-1-22630, Sma-1-22632, Sma-1-22634, Sma-1-22636, Sma-1-22638, Sma-1-22640, Sma-1-22642, Sma-1-22644, Sma-1-22646, Sma-1-22648, Sma-1-2265, Sma-1-22650, Sma-1-22652, Sma-1-22654, Sma-1-22655, Sma-1-22656, Sma-1-22658, Sma-1-22660, Sma-1-22662, Sma-1-22664, Sma-1-22666, Sma-1-22668, Sma-1-2267, Sma-1-22670, Sma-1-22671, Sma-1-22672, Sma-1-22674, Sma-1-22676, Sma-1-22678, Sma-1-22680, Sma-1-22681, Sma-1-22682, Sma-1-22683, Sma-1-22684, Sma-1-22685, Sma-1-22686, Sma-1-22687, Sma-1-22688, Sma-1-22689, Sma-1-2269, Sma-1-22690, Sma-1-22691, Sma-1-22692, Sma-1-22693, Sma-1-22694, Sma-1-22695, Sma-1-22696, Sma-1-22697, Sma-1-22698, Sma-1-22699, Sma-1-22700, Sma-1-22701, Sma-1-22702, Sma-1-22703, Sma-1-22704, Sma-1-22705, Sma-1-22706, Sma-1-22708, Sma-1-22709, Sma-1-2271, Sma-1-22710, Sma-1-22712, Sma-1-22714, Sma-1-22716, Sma-1-22718, Sma-1-22720, Sma-1-22722, Sma-1-22724, Sma-1-22726, Sma-1-22728, Sma-1-22729, Sma-1-2273, Sma-1-22730, Sma-1-22731, Sma-1-22732, Sma-1-22733, Sma-1-22734, Sma-1-22735, Sma-1-22736, Sma-1-22737, Sma-1-22738, Sma-1-22739, Sma-1-22740, Sma-1-22742, Sma-1-22743, Sma-1-22744, Sma-1-22746, Sma-1-22748, Sma-1-2275, Sma-1-22750, Sma-1-22751, Sma-1-22752, Sma-1-22754, Sma-1-22756, Sma-1-22757, Sma-1-22758, Sma-1-22760, Sma-1-22762, Sma-1-22764, Sma-1-22765, Sma-1-22766, Sma-1-22767, Sma-1-22768, Sma-1-22769, Sma-1-2277, Sma-1-22770, Sma-1-22771, Sma-1-22772, Sma-1-22773, Sma-1-22774, Sma-1-22775, Sma-1-22776, Sma-1-22777, Sma-1-22778, Sma-1-22779, Sma-1-22780, Sma-1-22781, Sma-1-22782, Sma-1-22783, Sma-1-22784, Sma-1-22785, Sma-1-22786, Sma-1-22787, Sma-1-22788, Sma-1-2279, Sma-1-22790, Sma-1-22792, Sma-1-22794, Sma-1-22796, Sma-1-22797, Sma-1-22798, Sma-1-228, Sma-1-22800, Sma-1-22802, Sma-1-22804, Sma-1-22806, Sma-1-22808, Sma-1-2281, Sma-1-22810, Sma-1-22812, Sma-1-22814, Sma-1-22816, Sma-1-22818, Sma-1-22819, Sma-1-22820, Sma-1-22821, Sma-1-22822, Sma-1-22823, Sma-1-22824, Sma-1-22825, Sma-1-22826, Sma-1-22828, Sma-1-22829, Sma-1-2283, Sma-1-22830, Sma-1-22832, Sma-1-22834, Sma-1-22836, Sma-1-22838, Sma-1-22840, Sma-1-22841, Sma-1-22842, Sma-1-22843, Sma-1-22844, Sma-1-22845, Sma-1-22846, Sma-1-22848, Sma-1-2285, Sma-1-22850, Sma-1-22851, Sma-1-22852, Sma-1-22854, Sma-1-22856, Sma-1-22858, Sma-1-22860, Sma-1-22862, Sma-1-22864, Sma-1-22866, Sma-1-22868, Sma-1-2287, Sma-1-22870, Sma-1-22872, Sma-1-22874, Sma-1-22876, Sma-1-22878, Sma-1-22880, Sma-1-22882, Sma-1-22884, Sma-1-22886, Sma-1-22888, Sma-1-2289, Sma-1-22890, Sma-1-22892, Sma-1-22894, Sma-1-22896, Sma-1-22898, Sma-1-22900, Sma-1-22901, Sma-1-22902, Sma-1-22904, Sma-1-22905, Sma-1-22906, Sma-1-22907, Sma-1-22908, Sma-1-22909, Sma-1-2291, Sma-1-22910, Sma-1-22912, Sma-1-22914, Sma-1-22916, Sma-1-22917, Sma-1-22918, Sma-1-22920, Sma-1-22922, Sma-1-22924, Sma-1-22926, Sma-1-22927, Sma-1-22928, Sma-1-2293, Sma-1-22930, Sma-1-22932, Sma-1-22934, Sma-1-22936, Sma-1-22938, Sma-1-22940, Sma-1-22942, Sma-1-22943, Sma-1-22944, Sma-1-22946, Sma-1-22948, Sma-1-2295, Sma-1-22950, Sma-1-22952, Sma-1-22954, Sma-1-22956, Sma-1-22958, Sma-1-22960, Sma-1-22962, Sma-1-22964, Sma-1-22966, Sma-1-22968, Sma-1-2297, Sma-1-22970, Sma-1-22972, Sma-1-22974, Sma-1-22976, Sma-1-22978, Sma-1-22980, Sma-1-22982, Sma-1-22983, Sma-1-22984, Sma-1-22986, Sma-1-22987, Sma-1-22988, Sma-1-2299, Sma-1-22990, Sma-1-22992, Sma-1-22994, Sma-1-22996, Sma-1-22998, Sma-1-22999, Sma-1-230, Sma-1-23000, Sma-1-23002, Sma-1-23004, Sma-1-23006, Sma-1-23008, Sma-1-23009, Sma-1-2301, Sma-1-23010, Sma-1-23012, Sma-1-23014, Sma-1-23016, Sma-1-23017, Sma-1-23018, Sma-1-2302, Sma-1-23020, Sma-1-23021, Sma-1-23022, Sma-1-23024, Sma-1-23026, Sma-1-23028, Sma-1-23029, Sma-1-2303, Sma-1-23030, Sma-1-23032, Sma-1-23033, Sma-1-23034, Sma-1-23036, Sma-1-23038, Sma-1-23040, Sma-1-23042, Sma-1-23044, Sma-1-23045, Sma-1-23046, Sma-1-23048, Sma-1-23049, Sma-1-2305, Sma-1-23050, Sma-1-23052, Sma-1-23054, Sma-1-23055, Sma-1-23056, Sma-1-23058, Sma-1-23060, Sma-1-23062, Sma-1-23063, Sma-1-23064, Sma-1-23066, Sma-1-23068, Sma-1-2307, Sma-1-23070, Sma-1-23072, Sma-1-23073, Sma-1-23074, Sma-1-23075, Sma-1-23076, Sma-1-23078, Sma-1-23080, Sma-1-23082, Sma-1-23084, Sma-1-23086, Sma-1-23088, Sma-1-2309, Sma-1-23090, Sma-1-23091, Sma-1-23092, Sma-1-23093, Sma-1-23094, Sma-1-23095, Sma-1-23096, Sma-1-23098, Sma-1-23100, Sma-1-23102, Sma-1-23103, Sma-1-23104, Sma-1-23106, Sma-1-23108, Sma-1-23109, Sma-1-2311, Sma-1-23110, Sma-1-23112, Sma-1-23113, Sma-1-23114, Sma-1-23116, Sma-1-23118, Sma-1-23119, Sma-1-23120, Sma-1-23122, Sma-1-23124, Sma-1-23126, Sma-1-23128, Sma-1-2313, Sma-1-23130, Sma-1-23132, Sma-1-23134, Sma-1-23136, Sma-1-23137, Sma-1-23138, Sma-1-23140, Sma-1-23142, Sma-1-23144, Sma-1-23146, Sma-1-23147, Sma-1-23148, Sma-1-2315, Sma-1-23150, Sma-1-23152, Sma-1-23154, Sma-1-23156, Sma-1-23158, Sma-1-23160, Sma-1-23162, Sma-1-23164, Sma-1-23165, Sma-1-23166, Sma-1-23167, Sma-1-23168, Sma-1-2317, Sma-1-23170, Sma-1-23172, Sma-1-23174, Sma-1-23176, Sma-1-23178, Sma-1-23180, Sma-1-23182, Sma-1-23184, Sma-1-23186, Sma-1-23188, Sma-1-23189, Sma-1-2319, Sma-1-23190, Sma-1-23191, Sma-1-23192, Sma-1-23193, Sma-1-23194, Sma-1-23195, Sma-1-23196, Sma-1-23197, Sma-1-23198, Sma-1-23199, Sma-1-232, Sma-1-23200, Sma-

1-23201, Sma-1-23202, Sma-1-23203, Sma-1-23204, Sma-1-23205, Sma-1-23206, Sma-1-23207, Sma-1-23208, Sma-1-23209, Sma-1-2321, Sma-1-23210, Sma-1-23211, Sma-1-23212, Sma-1-23213, Sma-1-23214, Sma-1-23215, Sma-1-23216, Sma-1-23217, Sma-1-23218, Sma-1-23220, Sma-1-23222, Sma-1-23224, Sma-1-23226, Sma-1-23228, Sma-1-2323, Sma-1-23230, Sma-1-23232, Sma-1-23234, Sma-1-23236, Sma-1-23238, Sma-1-23240, Sma-1-23242, Sma-1-23244, Sma-1-23245, Sma-1-23246, Sma-1-23248, Sma-1-2325, Sma-1-23250, Sma-1-23252, Sma-1-23254, Sma-1-23255, Sma-1-23256, Sma-1-23258, Sma-1-23260, Sma-1-23262, Sma-1-23264, Sma-1-23265, Sma-1-23266, Sma-1-23267, Sma-1-23268, Sma-1-2327, Sma-1-23270, Sma-1-23272, Sma-1-23274, Sma-1-23276, Sma-1-23278, Sma-1-23280, Sma-1-23282, Sma-1-23284, Sma-1-23286, Sma-1-23288, Sma-1-2329, Sma-1-23290, Sma-1-23292, Sma-1-23294, Sma-1-23295, Sma-1-23296, Sma-1-23298, Sma-1-23300, Sma-1-23302, Sma-1-23304, Sma-1-23306, Sma-1-23308, Sma-1-2331, Sma-1-23310, Sma-1-23312, Sma-1-23313, Sma-1-23314, Sma-1-23316, Sma-1-23318, Sma-1-23319, Sma-1-23320, Sma-1-23322, Sma-1-23324, Sma-1-23326, Sma-1-23327, Sma-1-23328, Sma-1-23329, Sma-1-2333, Sma-1-23330, Sma-1-23331, Sma-1-23332, Sma-1-23334, Sma-1-23336, Sma-1-23338, Sma-1-23340, Sma-1-23342, Sma-1-23344, Sma-1-23346, Sma-1-23348, Sma-1-2335, Sma-1-23350, Sma-1-23352, Sma-1-23353, Sma-1-23354, Sma-1-23356, Sma-1-23358, Sma-1-23360, Sma-1-23362, Sma-1-23364, Sma-1-23366, Sma-1-23368, Sma-1-2337, Sma-1-23370, Sma-1-23372, Sma-1-23374, Sma-1-23375, Sma-1-23376, Sma-1-23378, Sma-1-23380, Sma-1-23381, Sma-1-23382, Sma-1-23383, Sma-1-23384, Sma-1-23385, Sma-1-23386, Sma-1-23387, Sma-1-23388, Sma-1-2339, Sma-1-23390, Sma-1-23391, Sma-1-23392, Sma-1-23394, Sma-1-23396, Sma-1-23398, Sma-1-23399, Sma-1-234, Sma-1-23400, Sma-1-23402, Sma-1-23403, Sma-1-23404, Sma-1-23406, Sma-1-23408, Sma-1-2341, Sma-1-23410, Sma-1-23412, Sma-1-23414, Sma-1-23416, Sma-1-23418, Sma-1-23420, Sma-1-23422, Sma-1-23424, Sma-1-23425, Sma-1-23426, Sma-1-23427, Sma-1-23428, Sma-1-2343, Sma-1-23430, Sma-1-23432, Sma-1-23434, Sma-1-23436, Sma-1-23438, Sma-1-23439, Sma-1-23440, Sma-1-23441, Sma-1-23442, Sma-1-23444, Sma-1-23446, Sma-1-23448, Sma-1-2345, Sma-1-23450, Sma-1-23451, Sma-1-23452, Sma-1-23453, Sma-1-23454, Sma-1-23456, Sma-1-23458, Sma-1-2346, Sma-1-23460, Sma-1-23462, Sma-1-23464, Sma-1-23465, Sma-1-23466, Sma-1-23468, Sma-1-23469, Sma-1-2347, Sma-1-23470, Sma-1-23472, Sma-1-23474, Sma-1-23476, Sma-1-23478, Sma-1-23480, Sma-1-23482, Sma-1-23484, Sma-1-23485, Sma-1-23486, Sma-1-23487, Sma-1-23488, Sma-1-2349, Sma-1-23490, Sma-1-23491, Sma-1-23492, Sma-1-23494, Sma-1-23495, Sma-1-23496, Sma-1-23498, Sma-1-23500, Sma-1-23502, Sma-1-23504, Sma-1-23505, Sma-1-23506, Sma-1-23508, Sma-1-2351, Sma-1-23510, Sma-1-23511, Sma-1-23512, Sma-1-23514, Sma-1-23516, Sma-1-23518, Sma-1-23520, Sma-1-23522, Sma-1-23524, Sma-1-23525, Sma-1-23526, Sma-1-23527, Sma-1-23528, Sma-1-23529, Sma-1-2353, Sma-1-23530, Sma-1-23531, Sma-1-23532, Sma-1-23533, Sma-1-23534, Sma-1-23535, Sma-1-23536, Sma-1-23537, Sma-1-23538, Sma-1-23539, Sma-1-23540, Sma-1-23541, Sma-1-23542, Sma-1-23543, Sma-1-23544, Sma-1-23545, Sma-1-23546, Sma-1-23548, Sma-1-2355, Sma-1-23550, Sma-1-23551, Sma-1-23552, Sma-1-23554, Sma-1-23555, Sma-1-23556, Sma-1-23557, Sma-1-23558, Sma-1-23559, Sma-1-23560, Sma-1-23561, Sma-1-23562, Sma-1-23563, Sma-1-23564, Sma-1-23565, Sma-1-23566, Sma-1-23567, Sma-1-23568, Sma-1-23569, Sma-1-2357, Sma-1-23570, Sma-1-23571, Sma-1-23572, Sma-1-23573, Sma-1-23574, Sma-1-23575, Sma-1-23576, Sma-1-23577, Sma-1-23578, Sma-1-23579, Sma-1-23580, Sma-1-23582, Sma-1-23583, Sma-1-23584, Sma-1-23586, Sma-1-23587, Sma-1-23588, Sma-1-2359, Sma-1-23590, Sma-1-23591, Sma-1-23592, Sma-1-23594, Sma-1-23596, Sma-1-23597, Sma-1-23598, Sma-1-236, Sma-1-23600, Sma-1-23602, Sma-1-23604, Sma-1-23606, Sma-1-23608, Sma-1-2361, Sma-1-23610, Sma-1-23612, Sma-1-23614, Sma-1-23616, Sma-1-23618, Sma-1-23620, Sma-1-23622, Sma-1-23624, Sma-1-23626, Sma-1-23628, Sma-1-2363, Sma-1-23630, Sma-1-23632, Sma-1-23634, Sma-1-23635, Sma-1-23636, Sma-1-23637, Sma-1-23638, Sma-1-23639, Sma-1-23640, Sma-1-23641, Sma-1-23642, Sma-1-23643, Sma-1-23644, Sma-1-23645, Sma-1-23646, Sma-1-23647, Sma-1-23648, Sma-1-23649, Sma-1-2365, Sma-1-23650, Sma-1-23651, Sma-1-23652, Sma-1-23653, Sma-1-23654, Sma-1-23656, Sma-1-23658, Sma-1-23660, Sma-1-23662, Sma-1-23663, Sma-1-23664, Sma-1-23666, Sma-1-23668, Sma-1-2367, Sma-1-23670, Sma-1-23672, Sma-1-23673, Sma-1-23674, Sma-1-23675, Sma-1-23676, Sma-1-23677, Sma-1-23678, Sma-1-23679, Sma-1-23680, Sma-1-23681, Sma-1-23682, Sma-1-23683, Sma-1-23684, Sma-1-23685, Sma-1-23686, Sma-1-23687, Sma-1-23688, Sma-1-23689, Sma-1-2369, Sma-1-23690, Sma-1-23691, Sma-1-23692, Sma-1-23693, Sma-1-23694, Sma-1-23695, Sma-1-23696, Sma-1-23697, Sma-1-23698, Sma-1-23699, Sma-1-23700, Sma-1-23701, Sma-1-23702, Sma-1-23703, Sma-1-23704, Sma-1-23705, Sma-1-23706, Sma-1-23707, Sma-1-23708, Sma-1-2371, Sma-1-23710, Sma-1-23712, Sma-1-23714, Sma-1-23716, Sma-1-23717, Sma-1-23718, Sma-1-23719, Sma-1-23720, Sma-1-23721, Sma-1-23722, Sma-1-23723, Sma-1-23724, Sma-1-23725, Sma-1-23726, Sma-1-23728, Sma-1-2373, Sma-1-23730, Sma-1-23732, Sma-1-23734, Sma-1-23735, Sma-1-23736, Sma-1-23737, Sma-1-23738, Sma-1-23739, Sma-1-23740, Sma-1-23741, Sma-1-23742, Sma-1-23743, Sma-1-23744, Sma-1-23745, Sma-1-23746, Sma-1-23747, Sma-1-23748, Sma-1-2375, Sma-1-23750, Sma-1-23752, Sma-1-23754, Sma-1-23756, Sma-1-23758, Sma-1-23760, Sma-1-23762, Sma-1-23763, Sma-1-23764, Sma-1-23765, Sma-1-23766, Sma-1-23767, Sma-1-23768, Sma-1-23769, Sma-1-2377, Sma-1-23770, Sma-1-23771, Sma-1-23772, Sma-1-23773, Sma-1-23774, Sma-1-23775, Sma-1-23776, Sma-1-23777, Sma-1-23778, Sma-1-23779, Sma-1-23780, Sma-1-23781, Sma-1-23782, Sma-1-23783, Sma-1-23784, Sma-1-23785, Sma-1-23786, Sma-1-23787, Sma-1-23788, Sma-1-23789, Sma-1-2379, Sma-1-23790, Sma-1-23791, Sma-1-23792, Sma-1-23793, Sma-1-23794, Sma-1-23795, Sma-1-23796, Sma-1-23797, Sma-1-23798, Sma-1-23799, Sma-1-238, Sma-1-23800, Sma-1-23802, Sma-1-23804, Sma-1-23806, Sma-1-23807, Sma-1-23808, Sma-1-2381, Sma-1-23810, Sma-1-23812, Sma-1-23814, Sma-1-23816, Sma-1-23818, Sma-1-23819, Sma-1-23820, Sma-1-23821, Sma-1-23822, Sma-1-23824, Sma-1-23826, Sma-1-23827, Sma-1-23828, Sma-1-23829, Sma-1-2383, Sma-1-23830, Sma-1-23831, Sma-1-23832, Sma-1-23833, Sma-1-23834, Sma-1-23835, Sma-1-23836, Sma-1-23837, Sma-1-23838, Sma-1-23839, Sma-1-23840, Sma-1-23841, Sma-1-23842, Sma-1-23843, Sma-1-23844, Sma-1-23845, Sma-1-23846, Sma-1-23848, Sma-1-2385, Sma-1-23850, Sma-1-23852, Sma-1-23853, Sma-1-23854, Sma-1-23855, Sma-1-23856, Sma-1-23858, Sma-1-23860, Sma-1-23861, Sma-1-23862, Sma-1-23864, Sma-1-23866, Sma-1-23868, Sma-1-2387, Sma-1-23870, Sma-1-23872, Sma-1-23874, Sma-1-23875, Sma-1-23876, Sma-1-23877, Sma-1-23878, Sma-1-23880, Sma-1-23882, Sma-1-23883, Sma-1-23884, Sma-1-23885, Sma-1-23886,

Sma-1-23887, Sma-1-23888, Sma-1-23889, Sma-1-2389, Sma-1-23890, Sma-1-23891, Sma-1-23892, Sma-1-23893, Sma-1-23894, Sma-1-23895, Sma-1-23896, Sma-1-23897, Sma-1-23898, Sma-1-23899, Sma-1-23900, Sma-1-23901, Sma-1-23902, Sma-1-23903, Sma-1-23904, Sma-1-23905, Sma-1-23906, Sma-1-23907, Sma-1-23908, Sma-1-23909, Sma-1-2391, Sma-1-23910, Sma-1-23911, Sma-1-23912, Sma-1-23913, Sma-1-23914, Sma-1-23915, Sma-1-23916, Sma-1-23917, Sma-1-23918, Sma-1-23919, Sma-1-23920, Sma-1-23921, Sma-1-23922, Sma-1-23924, Sma-1-23926, Sma-1-23928, Sma-1-2393, Sma-1-23930, Sma-1-23932, Sma-1-23933, Sma-1-23934, Sma-1-23936, Sma-1-23938, Sma-1-23940, Sma-1-23941, Sma-1-23942, Sma-1-23943, Sma-1-23944, Sma-1-23946, Sma-1-23948, Sma-1-23949, Sma-1-2395, Sma-1-23950, Sma-1-23951, Sma-1-23952, Sma-1-23953, Sma-1-23954, Sma-1-23955, Sma-1-23956, Sma-1-23957, Sma-1-23958, Sma-1-23959, Sma-1-23960, Sma-1-23961, Sma-1-23962, Sma-1-23963, Sma-1-23964, Sma-1-23965, Sma-1-23966, Sma-1-23967, Sma-1-23968, Sma-1-23969, Sma-1-2397, Sma-1-23970, Sma-1-23971, Sma-1-23972, Sma-1-23973, Sma-1-23974, Sma-1-23975, Sma-1-23976, Sma-1-23977, Sma-1-23978, Sma-1-23979, Sma-1-23980, Sma-1-23981, Sma-1-23982, Sma-1-23983, Sma-1-23984, Sma-1-23985, Sma-1-23986, Sma-1-23987, Sma-1-23988, Sma-1-2399, Sma-1-23990, Sma-1-23992, Sma-1-23994, Sma-1-23995, Sma-1-23996, Sma-1-23998, Sma-1-24, Sma-1-240, Sma-1-24000, Sma-1-24002, Sma-1-24004, Sma-1-24006, Sma-1-24008, Sma-1-2401, Sma-1-24010, Sma-1-24012, Sma-1-24014, Sma-1-24016, Sma-1-24018, Sma-1-24020, Sma-1-24022, Sma-1-24024, Sma-1-24025, Sma-1-24026, Sma-1-24028, Sma-1-24029, Sma-1-2403, Sma-1-24030, Sma-1-24032, Sma-1-24034, Sma-1-24036, Sma-1-24038, Sma-1-24039, Sma-1-24040, Sma-1-24042, Sma-1-24044, Sma-1-24045, Sma-1-24046, Sma-1-24047, Sma-1-24048, Sma-1-2405, Sma-1-24050, Sma-1-24052, Sma-1-24054, Sma-1-24055, Sma-1-24056, Sma-1-24058, Sma-1-24059, Sma-1-24060, Sma-1-24062, Sma-1-24064, Sma-1-24065, Sma-1-24066, Sma-1-24068, Sma-1-2407, Sma-1-24070, Sma-1-24071, Sma-1-24072, Sma-1-24073, Sma-1-24074, Sma-1-24076, Sma-1-24078, Sma-1-24080, Sma-1-24082, Sma-1-24083, Sma-1-24084, Sma-1-24086, Sma-1-24087, Sma-1-24088, Sma-1-2409, Sma-1-24090, Sma-1-24092, Sma-1-24094, Sma-1-24096, Sma-1-24097, Sma-1-24098, Sma-1-24099, Sma-1-24100, Sma-1-24101, Sma-1-24102, Sma-1-24103, Sma-1-24104, Sma-1-24105, Sma-1-24106, Sma-1-24107, Sma-1-24108, Sma-1-24109, Sma-1-2411, Sma-1-24110, Sma-1-24111, Sma-1-24112, Sma-1-24113, Sma-1-24114, Sma-1-24115, Sma-1-24116, Sma-1-24117, Sma-1-24118, Sma-1-24119, Sma-1-24120, Sma-1-24121, Sma-1-24122, Sma-1-24123, Sma-1-24124, Sma-1-24125, Sma-1-24126, Sma-1-24127, Sma-1-24128, Sma-1-24129, Sma-1-2413, Sma-1-24130, Sma-1-24131, Sma-1-24132, Sma-1-24134, Sma-1-24136, Sma-1-24138, Sma-1-24140, Sma-1-24142, Sma-1-24144, Sma-1-24145, Sma-1-24146, Sma-1-24148, Sma-1-2415, Sma-1-24150, Sma-1-24152, Sma-1-24154, Sma-1-24156, Sma-1-24158, Sma-1-24160, Sma-1-24162, Sma-1-24164, Sma-1-24166, Sma-1-24168, Sma-1-2417, Sma-1-24170, Sma-1-24171, Sma-1-24172, Sma-1-24173, Sma-1-24174, Sma-1-24175, Sma-1-24176, Sma-1-24177, Sma-1-24178, Sma-1-24179, Sma-1-24180, Sma-1-24181, Sma-1-24182, Sma-1-24183, Sma-1-24184, Sma-1-24185, Sma-1-24186, Sma-1-24187, Sma-1-24188, Sma-1-24189, Sma-1-2419, Sma-1-24190, Sma-1-24191, Sma-1-24192, Sma-1-24193, Sma-1-24194, Sma-1-24195, Sma-1-24196, Sma-1-24197, Sma-1-24198, Sma-1-24199, Sma-1-242, Sma-1-24200, Sma-1-24201, Sma-1-24202, Sma-1-24203, Sma-1-24204, Sma-1-24206, Sma-1-24208, Sma-1-2421, Sma-1-24210, Sma-1-24212, Sma-1-24214, Sma-1-24216, Sma-1-24218, Sma-1-24219, Sma-1-24220, Sma-1-24221, Sma-1-24222, Sma-1-24223, Sma-1-24224, Sma-1-24225, Sma-1-24226, Sma-1-24227, Sma-1-24228, Sma-1-24229, Sma-1-2423, Sma-1-24230, Sma-1-24232, Sma-1-24234, Sma-1-24235, Sma-1-24236, Sma-1-24237, Sma-1-24238, Sma-1-24239, Sma-1-24240, Sma-1-24241, Sma-1-24242, Sma-1-24243, Sma-1-24244, Sma-1-24246, Sma-1-24248, Sma-1-2425, Sma-1-24250, Sma-1-24252, Sma-1-24254, Sma-1-24256, Sma-1-24257, Sma-1-24258, Sma-1-24259, Sma-1-24260, Sma-1-24262, Sma-1-24264, Sma-1-24266, Sma-1-24268, Sma-1-2427, Sma-1-24270, Sma-1-24272, Sma-1-24274, Sma-1-24276, Sma-1-24278, Sma-1-24280, Sma-1-24282, Sma-1-24284, Sma-1-24286, Sma-1-24288, Sma-1-2429, Sma-1-24290, Sma-1-24292, Sma-1-24294, Sma-1-24296, Sma-1-24298, Sma-1-24300, Sma-1-24302, Sma-1-24304, Sma-1-24306, Sma-1-24308, Sma-1-2431, Sma-1-24310, Sma-1-24312, Sma-1-24313, Sma-1-24314, Sma-1-24316, Sma-1-24318, Sma-1-24320, Sma-1-24322, Sma-1-24324, Sma-1-24326, Sma-1-24328, Sma-1-2433, Sma-1-24330, Sma-1-24332, Sma-1-24334, Sma-1-24335, Sma-1-24336, Sma-1-24338, Sma-1-24339, Sma-1-24340, Sma-1-24342, Sma-1-24344, Sma-1-24346, Sma-1-24348, Sma-1-2435, Sma-1-24350, Sma-1-24352, Sma-1-24354, Sma-1-24356, Sma-1-24358, Sma-1-24360, Sma-1-24362, Sma-1-24363, Sma-1-24364, Sma-1-24366, Sma-1-24368, Sma-1-2437, Sma-1-24370, Sma-1-24372, Sma-1-24373, Sma-1-24374, Sma-1-24375, Sma-1-24376, Sma-1-24377, Sma-1-24378, Sma-1-24379, Sma-1-24380, Sma-1-24381, Sma-1-24382, Sma-1-24384, Sma-1-24386, Sma-1-24388, Sma-1-24389, Sma-1-2439, Sma-1-24390, Sma-1-24392, Sma-1-24394, Sma-1-24395, Sma-1-24396, Sma-1-24398, Sma-1-24399, Sma-1-244, Sma-1-24400, Sma-1-24401, Sma-1-24402, Sma-1-24403, Sma-1-24404, Sma-1-24406, Sma-1-24407, Sma-1-24408, Sma-1-24409, Sma-1-2441, Sma-1-24410, Sma-1-24411, Sma-1-24412, Sma-1-24413, Sma-1-24414, Sma-1-24415, Sma-1-24416, Sma-1-24417, Sma-1-24418, Sma-1-24419, Sma-1-2442, Sma-1-24420, Sma-1-24421, Sma-1-24422, Sma-1-24423, Sma-1-24424, Sma-1-24425, Sma-1-24426, Sma-1-24427, Sma-1-24428, Sma-1-24429, Sma-1-2443, Sma-1-24430, Sma-1-24431, Sma-1-24432, Sma-1-24434, Sma-1-24436, Sma-1-24438, Sma-1-24440, Sma-1-24442, Sma-1-24444, Sma-1-24445, Sma-1-24446, Sma-1-24447, Sma-1-24448, Sma-1-24449, Sma-1-2445, Sma-1-24450, Sma-1-24451, Sma-1-24452, Sma-1-24453, Sma-1-24454, Sma-1-24456, Sma-1-24458, Sma-1-24460, Sma-1-24461, Sma-1-24462, Sma-1-24463, Sma-1-24464, Sma-1-24466, Sma-1-24468, Sma-1-24469, Sma-1-2447, Sma-1-24470, Sma-1-24472, Sma-1-24474, Sma-1-24476, Sma-1-24478, Sma-1-24480, Sma-1-24482, Sma-1-24484, Sma-1-24486, Sma-1-24487, Sma-1-24488, Sma-1-2449, Sma-1-24490, Sma-1-24492, Sma-1-24494, Sma-1-24495, Sma-1-24496, Sma-1-24497, Sma-1-24498, Sma-1-24499, Sma-1-24500, Sma-1-24502, Sma-1-24504, Sma-1-24506, Sma-1-24508, Sma-1-2451, Sma-1-24510, Sma-1-24512, Sma-1-24514, Sma-1-24515, Sma-1-24516, Sma-1-24518, Sma-1-24520, Sma-1-24521, Sma-1-24522, Sma-1-24524, Sma-1-24526, Sma-1-24528, Sma-1-2453, Sma-1-24530, Sma-1-24532, Sma-1-24533, Sma-1-24534, Sma-1-24535, Sma-1-24536, Sma-1-24537, Sma-1-24538, Sma-1-24539, Sma-1-24540, Sma-1-24542, Sma-1-24544, Sma-1-24545, Sma-1-24546, Sma-1-24547, Sma-1-24548, Sma-1-24549, Sma-1-2455, Sma-1-24550, Sma-1-24552, Sma-1-24553, Sma-1-24554, Sma-1-24555, Sma-1-24556, Sma-1-24557, Sma-1-24558, Sma-1-24559, Sma-1-24560,

Sma-1-24562, Sma-1-24564, Sma-1-24566, Sma-1-24568, Sma-1-24569, Sma-1-2457, Sma-1-24570, Sma-1-24572, Sma-1-24574, Sma-1-24576, Sma-1-24578, Sma-1-24579, Sma-1-24580, Sma-1-24582, Sma-1-24583, Sma-1-24584, Sma-1-24586, Sma-1-24588, Sma-1-2459, Sma-1-24590, Sma-1-24591, Sma-1-24592, Sma-1-24593, Sma-1-24594, Sma-1-24595, Sma-1-24596, Sma-1-24597, Sma-1-24598, Sma-1-24599, Sma-1-246, Sma-1-24600, Sma-1-24601, Sma-1-24602, Sma-1-24603, Sma-1-24604, Sma-1-24605, Sma-1-24606, Sma-1-24607, Sma-1-24608, Sma-1-24609, Sma-1-2461, Sma-1-24610, Sma-1-24611, Sma-1-24612, Sma-1-24613, Sma-1-24614, Sma-1-24615, Sma-1-24616, Sma-1-24617, Sma-1-24618, Sma-1-24619, Sma-1-24620, Sma-1-24621, Sma-1-24622, Sma-1-24623, Sma-1-24624, Sma-1-24625, Sma-1-24626, Sma-1-24627, Sma-1-24628, Sma-1-24629, Sma-1-2463, Sma-1-24630, Sma-1-24631, Sma-1-24632, Sma-1-24633, Sma-1-24634, Sma-1-24635, Sma-1-24636, Sma-1-24637, Sma-1-24638, Sma-1-24639, Sma-1-24640, Sma-1-24641, Sma-1-24642, Sma-1-24643, Sma-1-24644, Sma-1-24645, Sma-1-24646, Sma-1-24647, Sma-1-24648, Sma-1-24649, Sma-1-2465, Sma-1-24650, Sma-1-24651, Sma-1-24652, Sma-1-24653, Sma-1-24654, Sma-1-24655, Sma-1-24656, Sma-1-24658, Sma-1-24660, Sma-1-24661, Sma-1-24662, Sma-1-24663, Sma-1-24664, Sma-1-24666, Sma-1-24668, Sma-1-24669, Sma-1-2467, Sma-1-24670, Sma-1-24671, Sma-1-24672, Sma-1-24674, Sma-1-24676, Sma-1-24678, Sma-1-2468, Sma-1-24680, Sma-1-24682, Sma-1-24684, Sma-1-24685, Sma-1-24686, Sma-1-24688, Sma-1-2469, Sma-1-24690, Sma-1-24692, Sma-1-24693, Sma-1-24694, Sma-1-24695, Sma-1-24696, Sma-1-24697, Sma-1-24698, Sma-1-24700, Sma-1-24702, Sma-1-24703, Sma-1-24704, Sma-1-24706, Sma-1-24708, Sma-1-2471, Sma-1-24710, Sma-1-24712, Sma-1-24714, Sma-1-24716, Sma-1-24717, Sma-1-24718, Sma-1-24720, Sma-1-24722, Sma-1-24723, Sma-1-24724, Sma-1-24726, Sma-1-24727, Sma-1-24728, Sma-1-2473, Sma-1-24730, Sma-1-24732, Sma-1-24734, Sma-1-24736, Sma-1-24738, Sma-1-24740, Sma-1-24742, Sma-1-24744, Sma-1-24746, Sma-1-24748, Sma-1-24749, Sma-1-2475, Sma-1-24750, Sma-1-24751, Sma-1-24752, Sma-1-24754, Sma-1-24756, Sma-1-24758, Sma-1-24760, Sma-1-24761, Sma-1-24762, Sma-1-24764, Sma-1-24765, Sma-1-24766, Sma-1-24768, Sma-1-2477, Sma-1-24770, Sma-1-24772, Sma-1-24774, Sma-1-24776, Sma-1-24777, Sma-1-24778, Sma-1-24780, Sma-1-24782, Sma-1-24784, Sma-1-24786, Sma-1-24787, Sma-1-24788, Sma-1-2479, Sma-1-24790, Sma-1-24791, Sma-1-24792, Sma-1-24794, Sma-1-24796, Sma-1-24798, Sma-1-248, Sma-1-24800, Sma-1-24802, Sma-1-24804, Sma-1-24806, Sma-1-24808, Sma-1-2481, Sma-1-24810, Sma-1-24812, Sma-1-24813, Sma-1-24814, Sma-1-24815, Sma-1-24816, Sma-1-24817, Sma-1-24818, Sma-1-24820, Sma-1-24822, Sma-1-24824, Sma-1-24825, Sma-1-24826, Sma-1-24828, Sma-1-2483, Sma-1-24830, Sma-1-24832, Sma-1-24834, Sma-1-24835, Sma-1-24836, Sma-1-24837, Sma-1-24838, Sma-1-24839, Sma-1-24840, Sma-1-24841, Sma-1-24842, Sma-1-24843, Sma-1-24844, Sma-1-24845, Sma-1-24846, Sma-1-24847, Sma-1-24848, Sma-1-24849, Sma-1-2485, Sma-1-24850, Sma-1-24851, Sma-1-24852, Sma-1-24853, Sma-1-24854, Sma-1-24855, Sma-1-24856, Sma-1-24857, Sma-1-24858, Sma-1-24859, Sma-1-24860, Sma-1-24861, Sma-1-24862, Sma-1-24863, Sma-1-24864, Sma-1-24865, Sma-1-24866, Sma-1-24867, Sma-1-24868, Sma-1-24869, Sma-1-2487, Sma-1-24870, Sma-1-24871, Sma-1-24872, Sma-1-24873, Sma-1-24874, Sma-1-24875, Sma-1-24876, Sma-1-24877, Sma-1-24878, Sma-1-24879, Sma-1-24880, Sma-1-24881, Sma-1-24882, Sma-1-24883, Sma-1-24884, Sma-1-24885, Sma-1-24886, Sma-1-24887, Sma-1-24888, Sma-1-24889, Sma-1-2489, Sma-1-24890, Sma-1-24891, Sma-1-24892, Sma-1-24893, Sma-1-24894, Sma-1-24895, Sma-1-24896, Sma-1-24897, Sma-1-24898, Sma-1-24899, Sma-1-24900, Sma-1-24901, Sma-1-24902, Sma-1-24903, Sma-1-24904, Sma-1-24905, Sma-1-24906, Sma-1-24907, Sma-1-24908, Sma-1-24909, Sma-1-2491, Sma-1-24910, Sma-1-24912, Sma-1-24913, Sma-1-24914, Sma-1-24915, Sma-1-24916, Sma-1-24917, Sma-1-24918, Sma-1-24920, Sma-1-24922, Sma-1-24924, Sma-1-24926, Sma-1-24928, Sma-1-2493, Sma-1-24930, Sma-1-24932, Sma-1-24933, Sma-1-24934, Sma-1-24936, Sma-1-24938, Sma-1-24940, Sma-1-24942, Sma-1-24943, Sma-1-24944, Sma-1-24946, Sma-1-24948, Sma-1-24949, Sma-1-2495, Sma-1-24950, Sma-1-24952, Sma-1-24953, Sma-1-24954, Sma-1-24955, Sma-1-24956, Sma-1-24957, Sma-1-24958, Sma-1-24959, Sma-1-24960, Sma-1-24961, Sma-1-24962, Sma-1-24963, Sma-1-24964, Sma-1-24965, Sma-1-24966, Sma-1-24967, Sma-1-24968, Sma-1-24969, Sma-1-2497, Sma-1-24970, Sma-1-24971, Sma-1-24972, Sma-1-24973, Sma-1-24974, Sma-1-24975, Sma-1-24976, Sma-1-24977, Sma-1-24978, Sma-1-24979, Sma-1-24980, Sma-1-24981, Sma-1-24982, Sma-1-24983, Sma-1-24984, Sma-1-24985, Sma-1-24986, Sma-1-24987, Sma-1-24988, Sma-1-24989, Sma-1-2499, Sma-1-24990, Sma-1-24992, Sma-1-24994, Sma-1-24996, Sma-1-24998, Sma-1-24999, Sma-1-250, Sma-1-25000, Sma-1-25002, Sma-1-25004, Sma-1-25005, Sma-1-25006, Sma-1-25008, Sma-1-2501, Sma-1-25010, Sma-1-25012, Sma-1-25014, Sma-1-25016, Sma-1-25018, Sma-1-25020, Sma-1-25022, Sma-1-25024, Sma-1-25025, Sma-1-25026, Sma-1-25028, Sma-1-2503, Sma-1-25030, Sma-1-25032, Sma-1-25034, Sma-1-25036, Sma-1-25038, Sma-1-2504, Sma-1-25040, Sma-1-25042, Sma-1-25044, Sma-1-25046, Sma-1-25047, Sma-1-25048, Sma-1-25049, Sma-1-2505, Sma-1-25050, Sma-1-25052, Sma-1-25054, Sma-1-25056, Sma-1-25058, Sma-1-2506, Sma-1-25060, Sma-1-25062, Sma-1-25064, Sma-1-25066, Sma-1-25068, Sma-1-25069, Sma-1-2507, Sma-1-25070, Sma-1-25071, Sma-1-25072, Sma-1-25074, Sma-1-25076, Sma-1-25078, Sma-1-25079, Sma-1-25080, Sma-1-25082, Sma-1-25084, Sma-1-25086, Sma-1-25087, Sma-1-25088, Sma-1-25089, Sma-1-2509, Sma-1-25090, Sma-1-25091, Sma-1-25092, Sma-1-25094, Sma-1-25096, Sma-1-25098, Sma-1-25100, Sma-1-25102, Sma-1-25104, Sma-1-25105, Sma-1-25106, Sma-1-25108, Sma-1-2511, Sma-1-25110, Sma-1-25112, Sma-1-25114, Sma-1-25115, Sma-1-25116, Sma-1-25117, Sma-1-25118, Sma-1-25119, Sma-1-2512, Sma-1-25120, Sma-1-25121, Sma-1-25122, Sma-1-25123, Sma-1-25124, Sma-1-25125, Sma-1-25126, Sma-1-25127, Sma-1-25128, Sma-1-25129, Sma-1-2513, Sma-1-25130, Sma-1-25131, Sma-1-25132, Sma-1-25133, Sma-1-25134, Sma-1-25135, Sma-1-25136, Sma-1-25137, Sma-1-25138, Sma-1-25139, Sma-1-25140, Sma-1-25141, Sma-1-25142, Sma-1-25143, Sma-1-25144, Sma-1-25145, Sma-1-25146, Sma-1-25147, Sma-1-25148, Sma-1-25149, Sma-1-2515, Sma-1-25150, Sma-1-25151, Sma-1-25152, Sma-1-25153, Sma-1-25154, Sma-1-25155, Sma-1-25156, Sma-1-25157, Sma-1-25158, Sma-1-25159, Sma-1-25160, Sma-1-25161, Sma-1-25162, Sma-1-25163, Sma-1-25164, Sma-1-25165, Sma-1-25166, Sma-1-25167, Sma-1-25168, Sma-1-2517, Sma-1-25170, Sma-1-25171, Sma-1-25172, Sma-1-25174, Sma-1-25176, Sma-1-25178, Sma-1-25180, Sma-1-25181, Sma-1-25182, Sma-1-25183, Sma-1-25184, Sma-1-25186, Sma-1-25188, Sma-1-2519, Sma-1-25190, Sma-1-25192, Sma-1-25194, Sma-1-25196, Sma-1-25198, Sma-1-252, Sma-1-25200, Sma-1-25202, Sma-1-25204, Sma-1-25206, Sma-1-25208, Sma-1-2521, Sma-1-25210, Sma-1-25211, Sma-1-25212, Sma-1-25213, Sma-1-25214,

Sma-1-25215, Sma-1-25216, Sma-1-25217, Sma-1-25218, Sma-1-25219, Sma-1-25220, Sma-1-25221, Sma-1-25222, Sma-1-25224, Sma-1-25226, Sma-1-25228, Sma-1-2523, Sma-1-25230, Sma-1-25231, Sma-1-25232, Sma-1-25234, Sma-1-25236, Sma-1-25237, Sma-1-25238, Sma-1-25240, Sma-1-25242, Sma-1-25244, Sma-1-25246, Sma-1-25248, Sma-1-2525, Sma-1-25250, Sma-1-25252, Sma-1-25253, Sma-1-25254, Sma-1-25255, Sma-1-25256, Sma-1-25258, Sma-1-25259, Sma-1-25260, Sma-1-25262, Sma-1-25264, Sma-1-25265, Sma-1-25266, Sma-1-25268, Sma-1-2527, Sma-1-25270, Sma-1-25272, Sma-1-25274, Sma-1-25276, Sma-1-25278, Sma-1-25279, Sma-1-25280, Sma-1-25282, Sma-1-25284, Sma-1-25286, Sma-1-25288, Sma-1-2529, Sma-1-25290, Sma-1-25292, Sma-1-25294, Sma-1-25296, Sma-1-25298, Sma-1-25300, Sma-1-25302, Sma-1-25304, Sma-1-25306, Sma-1-25308, Sma-1-2531, Sma-1-25310, Sma-1-25312, Sma-1-25314, Sma-1-25316, Sma-1-25318, Sma-1-25320, Sma-1-25322, Sma-1-25324, Sma-1-25326, Sma-1-25328, Sma-1-2533, Sma-1-25330, Sma-1-25332, Sma-1-25334, Sma-1-25336, Sma-1-25338, Sma-1-25340, Sma-1-25342, Sma-1-25344, Sma-1-25346, Sma-1-25348, Sma-1-2535, Sma-1-2537, Sma-1-2539, Sma-1-254, Sma-1-2541, Sma-1-2543, Sma-1-2545, Sma-1-2547, Sma-1-2549, Sma-1-2551, Sma-1-2553, Sma-1-2555, Sma-1-2557, Sma-1-2559, Sma-1-256, Sma-1-2561, Sma-1-2563, Sma-1-2565, Sma-1-2567, Sma-1-2569, Sma-1-2571, Sma-1-2573, Sma-1-2575, Sma-1-2577, Sma-1-2579, Sma-1-258, Sma-1-2581, Sma-1-2583, Sma-1-2585, Sma-1-2587, Sma-1-2589, Sma-1-2591, Sma-1-2593, Sma-1-2595, Sma-1-2597, Sma-1-2599, Sma-1-26, Sma-1-260, Sma-1-2601, Sma-1-2603, Sma-1-2605, Sma-1-2607, Sma-1-2609, Sma-1-2611, Sma-1-2613, Sma-1-2615, Sma-1-2617, Sma-1-2619, Sma-1-262, Sma-1-2621, Sma-1-2623, Sma-1-2625, Sma-1-2626, Sma-1-2627, Sma-1-2629, Sma-1-2631, Sma-1-2633, Sma-1-2635, Sma-1-2637, Sma-1-2639, Sma-1-264, Sma-1-2641, Sma-1-2643, Sma-1-2645, Sma-1-2647, Sma-1-2649, Sma-1-2651, Sma-1-2653, Sma-1-2655, Sma-1-2657, Sma-1-2659, Sma-1-266, Sma-1-2661, Sma-1-2663, Sma-1-2665, Sma-1-2667, Sma-1-2669, Sma-1-2671, Sma-1-2672, Sma-1-2673, Sma-1-2675, Sma-1-2677, Sma-1-2679, Sma-1-268, Sma-1-2681, Sma-1-2683, Sma-1-2685, Sma-1-2687, Sma-1-2689, Sma-1-2691, Sma-1-2693, Sma-1-2695, Sma-1-2697, Sma-1-2699, Sma-1-27, Sma-1-270, Sma-1-2701, Sma-1-2703, Sma-1-2705, Sma-1-2707, Sma-1-2709, Sma-1-2711, Sma-1-2713, Sma-1-2715, Sma-1-2716, Sma-1-2717, Sma-1-2719, Sma-1-272, Sma-1-2721, Sma-1-2723, Sma-1-2725, Sma-1-2727, Sma-1-2729, Sma-1-2731, Sma-1-2733, Sma-1-2735, Sma-1-2737, Sma-1-2739, Sma-1-274, Sma-1-2741, Sma-1-2743, Sma-1-2745, Sma-1-2747, Sma-1-2749, Sma-1-2751, Sma-1-2753, Sma-1-2755, Sma-1-2757, Sma-1-2759, Sma-1-276, Sma-1-2761, Sma-1-2763, Sma-1-2765, Sma-1-2767, Sma-1-2769, Sma-1-2771, Sma-1-2773, Sma-1-2775, Sma-1-2777, Sma-1-278, Sma-1-2780, Sma-1-2782, Sma-1-2784, Sma-1-2785, Sma-1-2786, Sma-1-2788, Sma-1-2790, Sma-1-2792, Sma-1-2794, Sma-1-2796, Sma-1-2798, Sma-1-28, Sma-1-280, Sma-1-2800, Sma-1-2802, Sma-1-2804, Sma-1-2806, Sma-1-2808, Sma-1-2810, Sma-1-2812, Sma-1-2814, Sma-1-2816, Sma-1-2818, Sma-1-282, Sma-1-2820, Sma-1-2822, Sma-1-2824, Sma-1-2826, Sma-1-2828, Sma-1-2830, Sma-1-2832, Sma-1-2834, Sma-1-2836, Sma-1-2838, Sma-1-284, Sma-1-2840, Sma-1-2841, Sma-1-2842, Sma-1-2844, Sma-1-2846, Sma-1-2848, Sma-1-2850, Sma-1-2852, Sma-1-2854, Sma-1-2856, Sma-1-2858, Sma-1-286, Sma-1-2860, Sma-1-2862, Sma-1-2864, Sma-1-2866, Sma-1-2867, Sma-1-2868, Sma-1-2870, Sma-1-2872, Sma-1-2874, Sma-1-2876, Sma-1-2878, Sma-1-288, Sma-1-2880, Sma-1-2882, Sma-1-2884, Sma-1-2886, Sma-1-2888, Sma-1-2890, Sma-1-2892, Sma-1-2894, Sma-1-2896, Sma-1-2898, Sma-1-290, Sma-1-2900, Sma-1-2902, Sma-1-2904, Sma-1-2906, Sma-1-2908, Sma-1-2910, Sma-1-2912, Sma-1-2914, Sma-1-2916, Sma-1-2918, Sma-1-292, Sma-1-2920, Sma-1-2922, Sma-1-2924, Sma-1-2926, Sma-1-2928, Sma-1-2930, Sma-1-2932, Sma-1-2934, Sma-1-2936, Sma-1-2938, Sma-1-294, Sma-1-2940, Sma-1-2942, Sma-1-2944, Sma-1-2946, Sma-1-2948, Sma-1-2949, Sma-1-2950, Sma-1-2951, Sma-1-2953, Sma-1-2955, Sma-1-2957, Sma-1-2959, Sma-1-296, Sma-1-2961, Sma-1-2962, Sma-1-2963, Sma-1-2965, Sma-1-2967, Sma-1-2969, Sma-1-2970, Sma-1-2971, Sma-1-2972, Sma-1-2973, Sma-1-2975, Sma-1-2977, Sma-1-2979, Sma-1-298, Sma-1-2981, Sma-1-2983, Sma-1-2985, Sma-1-2987, Sma-1-2989, Sma-1-2991, Sma-1-2993, Sma-1-2995, Sma-1-2997, Sma-1-2999, Sma-1-30, Sma-1-300, Sma-1-3001, Sma-1-3002, Sma-1-3003, Sma-1-3005, Sma-1-3007, Sma-1-3009, Sma-1-3011, Sma-1-3013, Sma-1-3015, Sma-1-3017, Sma-1-3019, Sma-1-302, Sma-1-3021, Sma-1-3023, Sma-1-3025, Sma-1-3027, Sma-1-3029, Sma-1-3031, Sma-1-3033, Sma-1-3035, Sma-1-3037, Sma-1-3039, Sma-1-304, Sma-1-3041, Sma-1-3043, Sma-1-3045, Sma-1-3047, Sma-1-3049, Sma-1-3051, Sma-1-3053, Sma-1-3055, Sma-1-3057, Sma-1-3059, Sma-1-306, Sma-1-3061, Sma-1-3063, Sma-1-3065, Sma-1-3067, Sma-1-3069, Sma-1-3071, Sma-1-3073, Sma-1-3075, Sma-1-3077, Sma-1-3079, Sma-1-308, Sma-1-3081, Sma-1-3083, Sma-1-3084, Sma-1-3085, Sma-1-3087, Sma-1-3089, Sma-1-3091, Sma-1-3093, Sma-1-3095, Sma-1-3097, Sma-1-3099, Sma-1-310, Sma-1-3101, Sma-1-3103, Sma-1-3105, Sma-1-3106, Sma-1-3107, Sma-1-3109, Sma-1-3111, Sma-1-3112, Sma-1-3113, Sma-1-3115, Sma-1-3117, Sma-1-3119, Sma-1-312, Sma-1-3121, Sma-1-3123, Sma-1-3125, Sma-1-3127, Sma-1-3129, Sma-1-3131, Sma-1-3133, Sma-1-3135, Sma-1-3137, Sma-1-3139, Sma-1-314, Sma-1-3141, Sma-1-3143, Sma-1-3145, Sma-1-3147, Sma-1-3149, Sma-1-3151, Sma-1-3153, Sma-1-3155, Sma-1-3157, Sma-1-3158, Sma-1-3159, Sma-1-316, Sma-1-3161, Sma-1-3163, Sma-1-3165, Sma-1-3167, Sma-1-3169, Sma-1-3171, Sma-1-3173, Sma-1-3175, Sma-1-3177, Sma-1-3179, Sma-1-318, Sma-1-3181, Sma-1-3183, Sma-1-3185, Sma-1-3186, Sma-1-3187, Sma-1-3189, Sma-1-319, Sma-1-3191, Sma-1-3193, Sma-1-3195, Sma-1-3197, Sma-1-3199, Sma-1-32, Sma-1-320, Sma-1-3201, Sma-1-3203, Sma-1-3205, Sma-1-3207, Sma-1-3209, Sma-1-3211, Sma-1-3213, Sma-1-3215, Sma-1-3217, Sma-1-3219, Sma-1-322, Sma-1-3221, Sma-1-3223, Sma-1-3225, Sma-1-3227, Sma-1-3229, Sma-1-3231, Sma-1-3233, Sma-1-3235, Sma-1-3237, Sma-1-3238, Sma-1-3239, Sma-1-324, Sma-1-3241, Sma-1-3243, Sma-1-3245, Sma-1-3247, Sma-1-3249, Sma-1-3251, Sma-1-3253, Sma-1-3255, Sma-1-3257, Sma-1-3259, Sma-1-326, Sma-1-3261, Sma-1-3263, Sma-1-3265, Sma-1-3267, Sma-1-3269, Sma-1-3271, Sma-1-3273, Sma-1-3274, Sma-1-3275, Sma-1-3277, Sma-1-3279, Sma-1-328, Sma-1-3281, Sma-1-3283, Sma-1-3285, Sma-1-3287, Sma-1-3289, Sma-1-3291, Sma-1-3293, Sma-1-3295, Sma-1-3297, Sma-1-3299, Sma-1-330, Sma-1-3301, Sma-1-3303, Sma-1-3305, Sma-1-3307, Sma-1-3309, Sma-1-3311, Sma-1-3313, Sma-1-3315, Sma-1-3317, Sma-1-3319, Sma-1-332, Sma-1-3321, Sma-1-3323, Sma-1-3325, Sma-1-3327, Sma-1-3329, Sma-1-3331, Sma-1-3333, Sma-1-3335, Sma-1-3337, Sma-1-3338, Sma-1-3339, Sma-1-334, Sma-1-3340, Sma-1-3341, Sma-1-3343, Sma-1-3345, Sma-1-3347, Sma-1-3349, Sma-1-3351, Sma-1-3353, Sma-1-3355, Sma-1-3357, Sma-1-3359, Sma-1-336, Sma-1-3361, Sma-1-3363, Sma-1-3365, Sma-1-3367, Sma-1-3369, Sma-1-3371, Sma-1-3373, Sma-1-3375, Sma-1-3377, Sma-1-3379, Sma-1-338, Sma-1-3381, Sma-1-3383, Sma-1-3385, Sma-1-3387, Sma-1-3388, Sma-

1-3389, Sma-1-3391, Sma-1-3393, Sma-1-3395, Sma-1-3397, Sma-1-3399, Sma-1-34, Sma-1-340, Sma-1-3401, Sma-1-3403, Sma-1-3405, Sma-1-3407, Sma-1-3408, Sma-1-3409, Sma-1-3411, Sma-1-3413, Sma-1-3415, Sma-1-3416, Sma-1-3417, Sma-1-3419, Sma-1-342, Sma-1-3421, Sma-1-3423, Sma-1-3425, Sma-1-3427, Sma-1-3429, Sma-1-3431, Sma-1-3433, Sma-1-3435, Sma-1-3437, Sma-1-3439, Sma-1-344, Sma-1-3441, Sma-1-3443, Sma-1-3445, Sma-1-3447, Sma-1-3449, Sma-1-3451, Sma-1-3453, Sma-1-3455, Sma-1-3457, Sma-1-3459, Sma-1-346, Sma-1-3461, Sma-1-3463, Sma-1-3465, Sma-1-3467, Sma-1-3469, Sma-1-3471, Sma-1-3473, Sma-1-3475, Sma-1-3477, Sma-1-3479, Sma-1-348, Sma-1-3481, Sma-1-3483, Sma-1-3485, Sma-1-3487, Sma-1-3489, Sma-1-3490, Sma-1-3491, Sma-1-3493, Sma-1-3495, Sma-1-3497, Sma-1-3499, Sma-1-350, Sma-1-3501, Sma-1-3503, Sma-1-3505, Sma-1-3507, Sma-1-3509, Sma-1-351, Sma-1-3511, Sma-1-3513, Sma-1-3515, Sma-1-3517, Sma-1-3519, Sma-1-352, Sma-1-3521, Sma-1-3523, Sma-1-3525, Sma-1-3527, Sma-1-3529, Sma-1-3531, Sma-1-3533, Sma-1-3535, Sma-1-3537, Sma-1-3539, Sma-1-354, Sma-1-3541, Sma-1-3543, Sma-1-3545, Sma-1-3547, Sma-1-3549, Sma-1-3551, Sma-1-3553, Sma-1-3555, Sma-1-3557, Sma-1-3559, Sma-1-356, Sma-1-3561, Sma-1-3563, Sma-1-3565, Sma-1-3567, Sma-1-3569, Sma-1-3571, Sma-1-3573, Sma-1-3575, Sma-1-3577, Sma-1-3579, Sma-1-358, Sma-1-3581, Sma-1-3583, Sma-1-3585, Sma-1-3587, Sma-1-3589, Sma-1-3591, Sma-1-3593, Sma-1-3595, Sma-1-3597, Sma-1-3599, Sma-1-36, Sma-1-360, Sma-1-3601, Sma-1-3603, Sma-1-3605, Sma-1-3607, Sma-1-3609, Sma-1-3611, Sma-1-3613, Sma-1-3615, Sma-1-3617, Sma-1-3619, Sma-1-362, Sma-1-3621, Sma-1-3622, Sma-1-3623, Sma-1-3624, Sma-1-3625, Sma-1-3627, Sma-1-3629, Sma-1-3631, Sma-1-3633, Sma-1-3635, Sma-1-3637, Sma-1-3639, Sma-1-364, Sma-1-3641, Sma-1-3643, Sma-1-3644, Sma-1-3645, Sma-1-3647, Sma-1-3649, Sma-1-3651, Sma-1-3653, Sma-1-3655, Sma-1-3657, Sma-1-3659, Sma-1-366, Sma-1-3661, Sma-1-3663, Sma-1-3665, Sma-1-3667, Sma-1-3669, Sma-1-3671, Sma-1-3673, Sma-1-3675, Sma-1-3677, Sma-1-3679, Sma-1-368, Sma-1-3681, Sma-1-3683, Sma-1-3685, Sma-1-3687, Sma-1-3689, Sma-1-3691, Sma-1-3693, Sma-1-3695, Sma-1-3697, Sma-1-3699, Sma-1-370, Sma-1-3701, Sma-1-3703, Sma-1-3705, Sma-1-3707, Sma-1-3709, Sma-1-3711, Sma-1-3713, Sma-1-3715, Sma-1-3717, Sma-1-3719, Sma-1-372, Sma-1-3721, Sma-1-3723, Sma-1-3725, Sma-1-3727, Sma-1-3728, Sma-1-3729, Sma-1-3731, Sma-1-3733, Sma-1-3735, Sma-1-3737, Sma-1-3739, Sma-1-374, Sma-1-3741, Sma-1-3743, Sma-1-3745, Sma-1-3747, Sma-1-3749, Sma-1-3751, Sma-1-3753, Sma-1-3755, Sma-1-3757, Sma-1-3759, Sma-1-376, Sma-1-3761, Sma-1-3763, Sma-1-3765, Sma-1-3766, Sma-1-3767, Sma-1-3769, Sma-1-3771, Sma-1-3773, Sma-1-3775, Sma-1-3777, Sma-1-3779, Sma-1-378, Sma-1-3780, Sma-1-3781, Sma-1-3783, Sma-1-3785, Sma-1-3787, Sma-1-3789, Sma-1-3791, Sma-1-3793, Sma-1-3795, Sma-1-3797, Sma-1-3799, Sma-1-38, Sma-1-380, Sma-1-3801, Sma-1-3803, Sma-1-3805, Sma-1-3807, Sma-1-3809, Sma-1-3811, Sma-1-3813, Sma-1-3815, Sma-1-3817, Sma-1-3819, Sma-1-382, Sma-1-3821, Sma-1-3823, Sma-1-3825, Sma-1-3827, Sma-1-3829, Sma-1-3831, Sma-1-3833, Sma-1-3835, Sma-1-3837, Sma-1-3838, Sma-1-3839, Sma-1-384, Sma-1-3841, Sma-1-3843, Sma-1-3845, Sma-1-3847, Sma-1-3849, Sma-1-3851, Sma-1-3852, Sma-1-3853, Sma-1-3855, Sma-1-3857, Sma-1-3859, Sma-1-386, Sma-1-3861, Sma-1-3863, Sma-1-3865, Sma-1-3867, Sma-1-3869, Sma-1-3871, Sma-1-3873, Sma-1-3875, Sma-1-3877, Sma-1-3879, Sma-1-388, Sma-1-3881, Sma-1-3883, Sma-1-3885, Sma-1-3887, Sma-1-3889, Sma-1-3891, Sma-1-3893, Sma-1-3895, Sma-1-3897, Sma-1-3899, Sma-1-390, Sma-1-3901, Sma-1-3903, Sma-1-3905, Sma-1-3907, Sma-1-3909, Sma-1-3911, Sma-1-3913, Sma-1-3915, Sma-1-3917, Sma-1-3919, Sma-1-392, Sma-1-3921, Sma-1-3923, Sma-1-3925, Sma-1-3927, Sma-1-3929, Sma-1-3931, Sma-1-3933, Sma-1-3935, Sma-1-3937, Sma-1-3939, Sma-1-394, Sma-1-3941, Sma-1-3943, Sma-1-3945, Sma-1-3947, Sma-1-3949, Sma-1-3951, Sma-1-3953, Sma-1-3955, Sma-1-3957, Sma-1-3959, Sma-1-396, Sma-1-3961, Sma-1-3963, Sma-1-3965, Sma-1-3967, Sma-1-3969, Sma-1-3971, Sma-1-3973, Sma-1-3975, Sma-1-3977, Sma-1-3979, Sma-1-398, Sma-1-3981, Sma-1-3983, Sma-1-3985, Sma-1-3986, Sma-1-3987, Sma-1-3989, Sma-1-3991, Sma-1-3992, Sma-1-3993, Sma-1-3995, Sma-1-3997, Sma-1-3999, Sma-1-4, Sma-1-40, Sma-1-400, Sma-1-4001, Sma-1-4003, Sma-1-4005, Sma-1-4007, Sma-1-4009, Sma-1-4011, Sma-1-4013, Sma-1-4015, Sma-1-4017, Sma-1-4019, Sma-1-402, Sma-1-4021, Sma-1-4023, Sma-1-4025, Sma-1-4027, Sma-1-4029, Sma-1-4031, Sma-1-4032, Sma-1-4033, Sma-1-4035, Sma-1-4037, Sma-1-4038, Sma-1-4039, Sma-1-404, Sma-1-4041, Sma-1-4043, Sma-1-4044, Sma-1-4045, Sma-1-4047, Sma-1-4048, Sma-1-4049, Sma-1-4051, Sma-1-4053, Sma-1-4055, Sma-1-4057, Sma-1-4059, Sma-1-406, Sma-1-4061, Sma-1-4063, Sma-1-4065, Sma-1-4067, Sma-1-4069, Sma-1-4071, Sma-1-4073, Sma-1-4074, Sma-1-4075, Sma-1-4077, Sma-1-4079, Sma-1-408, Sma-1-4081, Sma-1-4083, Sma-1-4085, Sma-1-4087, Sma-1-4089, Sma-1-4091, Sma-1-4093, Sma-1-4095, Sma-1-4097, Sma-1-4099, Sma-1-410, Sma-1-4101, Sma-1-4103, Sma-1-4105, Sma-1-4107, Sma-1-4109, Sma-1-4111, Sma-1-4113, Sma-1-4115, Sma-1-4117, Sma-1-4119, Sma-1-412, Sma-1-4121, Sma-1-4123, Sma-1-4125, Sma-1-4127, Sma-1-4129, Sma-1-4131, Sma-1-4133, Sma-1-4135, Sma-1-4137, Sma-1-4139, Sma-1-414, Sma-1-4141, Sma-1-4143, Sma-1-4145, Sma-1-4147, Sma-1-4149, Sma-1-4151, Sma-1-4153, Sma-1-4155, Sma-1-4157, Sma-1-4159, Sma-1-416, Sma-1-4161, Sma-1-4163, Sma-1-4165, Sma-1-4167, Sma-1-4169, Sma-1-4171, Sma-1-4173, Sma-1-4175, Sma-1-4177, Sma-1-4179, Sma-1-418, Sma-1-4181, Sma-1-4183, Sma-1-4185, Sma-1-4187, Sma-1-4189, Sma-1-4191, Sma-1-4193, Sma-1-4195, Sma-1-4197, Sma-1-4199, Sma-1-42, Sma-1-420, Sma-1-4201, Sma-1-4203, Sma-1-4205, Sma-1-4207, Sma-1-4209, Sma-1-4211, Sma-1-4212, Sma-1-4213, Sma-1-4215, Sma-1-4217, Sma-1-4219, Sma-1-422, Sma-1-4221, Sma-1-4223, Sma-1-4225, Sma-1-4227, Sma-1-4229, Sma-1-4231, Sma-1-4233, Sma-1-4235, Sma-1-4237, Sma-1-4239, Sma-1-424, Sma-1-4241, Sma-1-4243, Sma-1-4245, Sma-1-4247, Sma-1-4248, Sma-1-4249, Sma-1-4251, Sma-1-4253, Sma-1-4255, Sma-1-4257, Sma-1-4259, Sma-1-426, Sma-1-4261, Sma-1-4263, Sma-1-4265, Sma-1-4267, Sma-1-4269, Sma-1-4271, Sma-1-4273, Sma-1-4275, Sma-1-4277, Sma-1-4279, Sma-1-428, Sma-1-4281, Sma-1-4283, Sma-1-4285, Sma-1-4287, Sma-1-4289, Sma-1-4291, Sma-1-4293, Sma-1-4295, Sma-1-4297, Sma-1-4299, Sma-1-430, Sma-1-4301, Sma-1-4303, Sma-1-4305, Sma-1-4307, Sma-1-4309, Sma-1-4311, Sma-1-4313, Sma-1-4315, Sma-1-4317, Sma-1-4319, Sma-1-432, Sma-1-4321, Sma-1-4322, Sma-1-4323, Sma-1-4325, Sma-1-4327, Sma-1-4329, Sma-1-4331, Sma-1-4333, Sma-1-4335, Sma-1-4337, Sma-1-4339, Sma-1-434, Sma-1-4341, Sma-1-4343, Sma-1-4345, Sma-1-4347, Sma-1-4348, Sma-1-4349, Sma-1-4351, Sma-1-4353, Sma-1-4355, Sma-1-4357, Sma-1-4359, Sma-1-436, Sma-1-4361, Sma-1-4363, Sma-1-4365, Sma-1-4367, Sma-1-4369, Sma-1-4371, Sma-1-4373, Sma-1-4375, Sma-1-4377, Sma-1-4379, Sma-1-438, Sma-1-4381, Sma-1-4382, Sma-1-4383, Sma-1-4385, Sma-1-4387, Sma-1-4389, Sma-1-4391, Sma-1-4392, Sma-1-4393, Sma-1-4395, Sma-1-4397, Sma-1-4399, Sma-1-44, Sma-1-440, Sma-1-4401, Sma-1-4403, Sma-1-4405, Sma-1-
[truncated: 1,281,307 more chars]
